# A Clinicopathological Approach to Odontogenic Cysts: the Role of Cytokeratin 17 and bcl2 Immunohistochemistry in Identifying Odontogenic Keratocysts

**DOI:** 10.1007/s12253-020-00866-4

**Published:** 2020-07-06

**Authors:** Dorottya Cserni, Tamás Zombori, András Vörös, Anette Stájer, Annamária Rimovszki, Krisztián Daru, Zoltán Baráth, Gábor Cserni

**Affiliations:** 1 Department of Prosthodontics, Faculty of Dentistry, University of Szeged, Tisza Lajos krt 64-66, Szeged, H-6720 Hungary; 2 Department of Pathology, University of Szeged, Állomás u. 1, Szeged, H-6725 Hungary; 3 Department of Pathology, Bács-Kiskun County Teaching Hospital, Nyíri út 38, Kecskemét, H-6000 Hungary

**Keywords:** Odontogenic keratocyst, Cytokeratin 17, B cell lymphoma – 2 (bcl2), Dentigerous cyst, Radicular cyst

## Abstract

Odontogenic keratocysts (OKCs) are developmental cysts of the jaws that require proper diagnosis due to their potential for local aggressive growth and recurrences. OKCs have a typical parakeratotic epithelium demonstrating transepithelial cytokeratin 17 (CK17) and basal bcl2 staining on immunohistochemistry (IHC), which distinguishes them from other common jaw cysts. Secondary to inflammation, the epithelial lining may be altered and loses the typical IHC phenotype. The aim of the present study was to analyse a series of consecutive jaw cysts for their expression of CK17 and bcl2 and assess how these IHC stains may help in their diagnosis. All cysts were retrospectively assessed for available clinical, radiological and pathological findings and diagnoses were revised whenever needed. 85 cysts from 72 patients were collected from two departments. The series had 21 OKCs, the remaining non-OKCs included radicular/residual, dentigerous, paradental, lateral periodontal, botryoid odontogenic cysts. OKCs with typical epithelium showed the typical IHC phenotype, which was generally lost in inflammation-associated altered epithelium. Contrarily to earlier descriptions, a wide variety of CK17 positivity was seen in the majority of non-OKCs, including focal transepithelial staining. Basal bcl2 staining was also seen in 16 non-OKCs. These stainings were never as strong in intensity as seen in OKCs. One case was histopathologically identified as OKC due to focally maintained IHC profile. CK17 and bcl2 IHC may help in the diagnosis of OKCs, but must be interpreted with caution and is not a yes or no tool in the diagnostic puzzle.

**Electronic supplementary material:**

The online version of this article (10.1007/s12253-020-00866-4) contains supplementary material, which is available to authorized users.

## Introduction

Cystic or cyst-like lesions of odontogenic origin include inflammatory, developmental and neoplastic lesions [1]. They may lead to the loosening of the teeth, predisposition to fracture of the involved bone, inflammatory consequences, pain, swelling or other symptoms. The lesions are sometimes very difficult to differentiate from each other. For example, an apical granuloma and a radicular cyst cannot be distinguished on clinical grounds alone unless the dynamism of development is available; the verification of epithelial lining requires a histological sampling, most often the removal and histological examination of the lesion [2]. Some of the cysts are less noxious than others. Most have characteristic clinical / radiological findings, but these are often overlapping. Ameloblastoma, the most common odontogenic tumour is also characteristically cystic, and unicystic ameloblastoma shows an overlap in localisation, age group and radiological findings with odontogenic keratocysts (OKC) [1].

OKCs are presently classified as tumour-like cystic lesions, but in the previous edition of the WHO classification, they were among the tumours, as keratocystic odontogenic tumours, on the basis of the common mutation identified in the Patched-1 gene of the sonic hedgehog pathway, a key event in the development of the nevoid basal cell carcinoma syndrome, the Gorlin-Goltz syndrome [3, 4]. They are progressively growing, locally aggressive lesions prone to recurrence unless completely excised. Recurrences may occur from daughter cysts, invaginations or nests of odontogenic epithelium. These clinical features make the recognition, proper diagnosis and treatment of OKCs important.

OKCs have a predilection site at the posterior mandible, in the angle and ramus of the bone and an association with third molars, but may occur anywhere in the jaws. They are often discovered incidentally as radiolucencies of regular, often scalloped outlines with a common corticated edge; often the size is considerable at the time of diagnosis [1, 5]. The age distribution peaks in the 2nd and 3rd decade, with a smaller peak around the 6th decade, but the lesion may be seen in a wide age range. Males are about two times more commonly affected than females. These features may lead to the clinical diagnosis or suspicion of OKC, but are not distinctive enough.

**Fig. 1:**
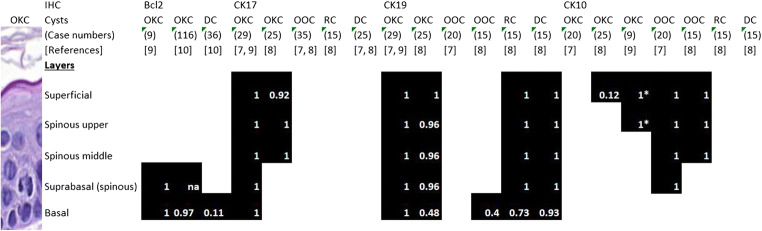
Differential immunoprofile of common odontogenic cysts based on profiling studies and personal experience [7–10]. (CK: cytokeratin, OKC: odontogenic keratocyst, OOC: orthokeratotic odontogenic cyst, RC: radicular cyst, DC: dentigerous (follicular) cyst; na: not available). White numbers in the figure refer to the proportion of cases stained. Data adopted and derived from results and figures of the referenced publications. Differences in staining intensity and diffuse versus patchy staining could not be considered in the figure, except for *: all cases showed focal superficial and upper spinous CK10 staining, with areas of epithelium fully devoid of staining, and this could be interpreted as negative or positive, depending on the vantage point

OKCs have a characteristic and therefore diagnostic epithelial lining consisting of a relatively thin (a few, 5–8 cells thick) parakeratotic squamous epithelium typically showing at least partial waviness of the surface. The basal cells are arranged in a palisading pattern, may show a reverted polarity with the nucleus located in the upper half of the cell rather than basally, and the boundary of the supportive connective tissue and the epithelium is generally smooth and even. This histology permits the diagnosis of OKCs, and helps to distinguish them from orthokeratotic odontogenic cysts (which were separated from OKCs in the latest edition of the WHO classification of head and neck tumours [1, 6]) and other odontogenic cysts [1]. The epithelial lining of OKCs has also a characteristic staining profile on immunohistochemistry (IHC), which includes transepithelial staining with cytokeratin 17 (CK17) and to a lesser degree CK19, superficial staining with CK10 and basal/suprabasal staining with bcl2 [7–10]. This also distinguishes them from other common cystic lesions of the jaws (Fig. 1) [7, 8].

It has been recognized earlier, that this staining pattern may be disturbed at sites of inflammation [7], and inflamed cysts show a non-specific epithelial lining. Indeed, epithelial changes seen in connection with secondary inflammation due to fistular connections, previous biopsy or cyst reduction procedures have been associated with complete or partial loss of the diagnostic epithelium and its immunostaining profile [9]. This partial or potentially complete loss of the typical epithelium, just as the lack of adequate and relevant clinico-radiological features may lead to misdiagnosis of OKCs as inflammatory cysts. Our previous work has suggested that with the epithelial changes in place, some areas may still show the IHC profile of OKCs, and this phenomenon could support the diagnosis or be consistent with the diagnosis in some difficult cases, especially on biopsy [9].

In the present work we sought at analysing consecutive jaw cysts with residual epithelium to look for the potential value of CK17 and bcl2 IHC (the two most consistent and distinguishing IHC reactions available, Fig. 1) in the proper diagnosis of these cysts and see how these stains (along with clinical and radiological data) are helpful in the differential diagnosis, especially the diagnosis of OKCs.

## Materials and Methods

Materials from patients consecutively operated on for jaw cysts and received at the Departments of Pathology of either the Bács-Kiskun County Teaching Hospital of the University of Szeged (Site A) or the Albert Szent-Györgyi Medical Centre of the University of Szeged (Site B) were used for analysis. Clinical and radiological data were retrieved from the patients’ digital medical charts.

IHC was performed on 3–4-μm-thick sections from formalin fixed and paraffin embedded blocks with the following antibodies: (Site A:) bcl2: Novocastra/Leica (Newcastle, UK), clone E3.1 (dilution 1:80); CK17 Novocastra/Leica (Newcastle, UK), clone E3 (ready-to-use, dilution 1:2); occasionally CK10 MasterDiagnostica (Granada, Spain), clone SP99 (ready-to-use); and CK19 CellMarque (Rocklin, California, USA), clone A53B/A2.26 (dilution 1:500); (Site B:) bcl2: Labvision/NeoMarkers (Fremont, CA, USA), clone 124 (dilution 1:300); CK17 Labvision/NeoMarkers (Fremont, CA, USA), clone E3 (dilution 1:40). At site A, a 45-min-water bath (98 °C, pH 6, citrate buffer; for bcl2 and CK19) or a high-pressure (pH 9, TRIS-EGTA buffer; for CK10) heat induced epitope retrieval was used prior to the 60-min-incubation at room temperature, and the reactions were developed with Vector ImmPRESS (Vector Laboratories, Burlingame, CA, USA; 30 min, room temperature) using Novared as chromogen; The CK17 reaction used epitope retrieval with ER1 solution (20 min), incubation time on the Bond Max autostainer (Leica Biosystems, Wetzlar, Germany) was 15 min. At site B, bcl2 and CK17 IHC were run on a Bond Max autostainer (Leica Biosystems, Wetzlar, Germany) with epitope retrieval with ER2 (pH 9) solution for 20 min and incubation time of 20 min. (CK10 and CK19 were only occasionally obtained, and results are presented only in the supplementary material.) While interpreting the bcl2 IHC reactions, obvious, strong staining patterns were appreciated, whereas diffuse weak background-like staining was ignored, and intralesional lymphocytes served as a control for the staining, both for its presence and its intensity (see supplementary material).

As the clinical diagnoses were sometimes non-specific (e.g. maxillary cyst or odontogenic cyst?), relevant clinical data were occasionally missing from the pathology request forms, and the pathology reports commonly neglected the (missing) clinical data, the clinical, radiological and histopathological findings were revised in all cases, as much as possible, and a final, clinicopathological diagnosis was established. The clinical data, imaging (where available), histology and diagnoses are shown on a case by case basis in the supplementary material.

The study was approved by the Ethical Committee of the University of Szeged.

## Results

Altogether 50 cysts were collected from 45 patients at site A, and 35 cysts from 27 patients at site B. The 85 cysts analysed were removed from 45 male and 27 female patients. Their median age was 44 years (range: 11–76).

The histology request forms at site A were often more informative with helpful clinical information communicated and diagnoses suggested, whereas those of site B were generally less helpful, and often included the questioned diagnosis of OKC even if this was not well founded clinically (radiologically). On the basis of the clinical diagnoses suggested on the histopathology request forms at site A, there were 20 cysts not otherwise specified (NOS) or with at most the bone and/or the teeth places specified; 19 radicular, residual or periapical cysts; 4 dentigerous (follicular) cysts; 2 OKCs; 1 “globulomaxillary” cyst; and 3 cysts with a differential diagnosis of two entities (2 OKC vs follicular cyst and 1 radicular vs “globulomaxillary” cyst). An OKC was sent in as inflamed maxillary sinus mucosa (case K1), although the diagnosis of a dentigerous cyst was mentioned in previous medical records not cited on the request form. The request forms at site B suggested 4 cysts NOS (with or without a communicated location; 1 with the possibility of ameloblastoma questioned), 3 radicular cysts, and the remaining 28 forms all raised the question whether the cyst represented an OKC or not.

**Fig. 2:**
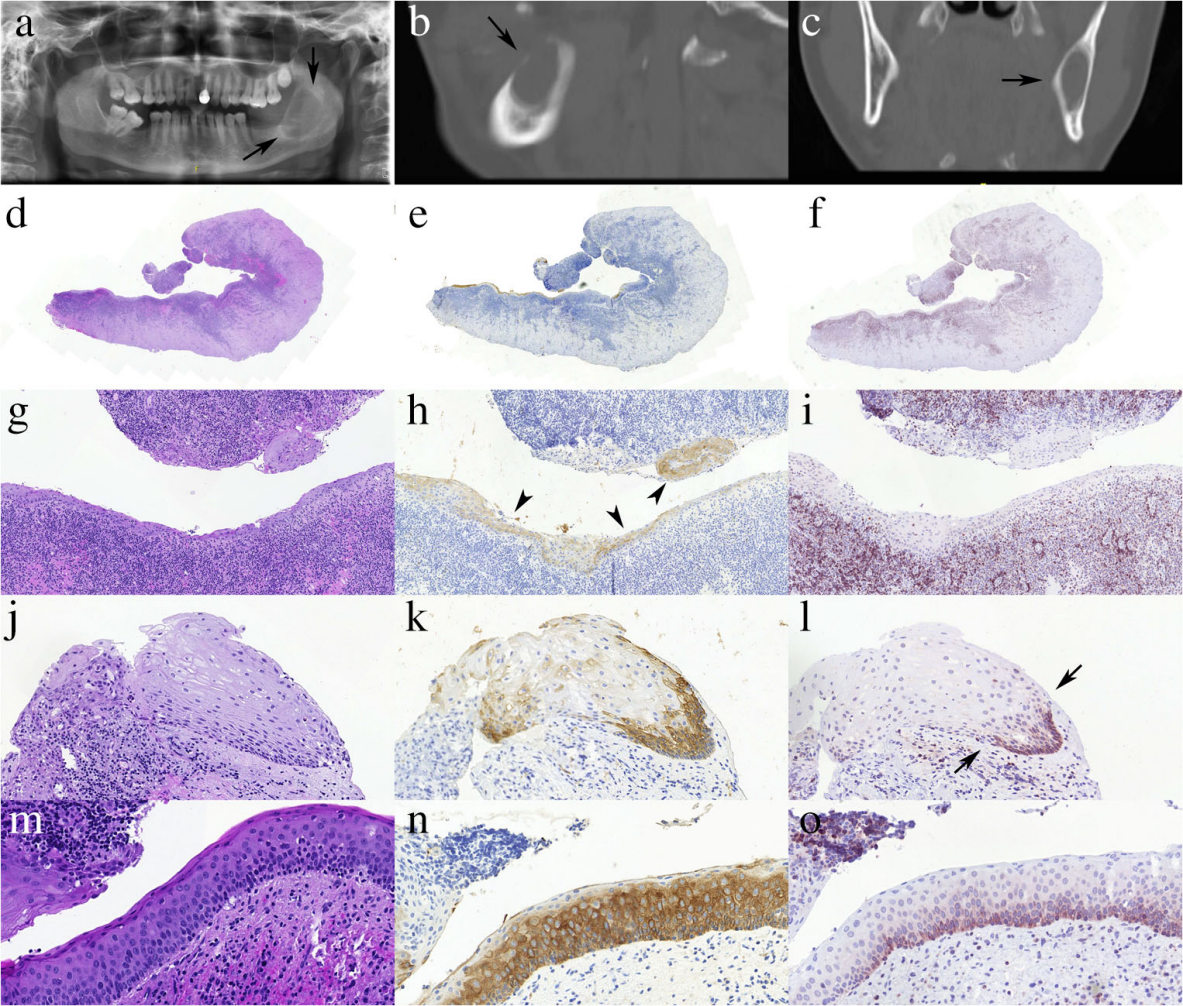
Inflamed OKC with minimal residual typical epithelium. A-C: orthopantomogram, and details of a sagittal and coronal plane image from the computed tomography scan of the cyst highlighted by arrows (case K2); note the large corticated radiolucent area in the angle and ramus of the mandible growing “within the bone” without conspicuous lingual / buccal extension. D-F: low power views of one section highlighting the heavy inflammatory infiltrate; H-I: medium power views of the non-specific squamous epithelium demonstrating focally transepithelial (arrowheads) CK17 staining (H) without bcl2 labelling of the epithelium (I); J-L: high power view of the tiny non-typical (probably transitional) epithelial fragment pointing to the possible OKC origin of the inflamed cyst with strong basal bcl2 labelling (arrows) (L) and non-typical patchy basal-spinous-superficial, i.e. “transepithelial” CK17 staining (K); M-O: the 1.1-mm-long typical epithelial fragment identified on revision and recut also demonstrating the typical strong transepithelial CK17 (N) and basal bcl2 (O) stainings. (D,E,F: ×1; G, H, I: ×12; J, K, L: ×25; M, N, O: ×35; D, G, J, M: haematoxylin and eosin; E, H, K, N: CK17; F, I, L, O: bcl2)

**Fig. 3:**
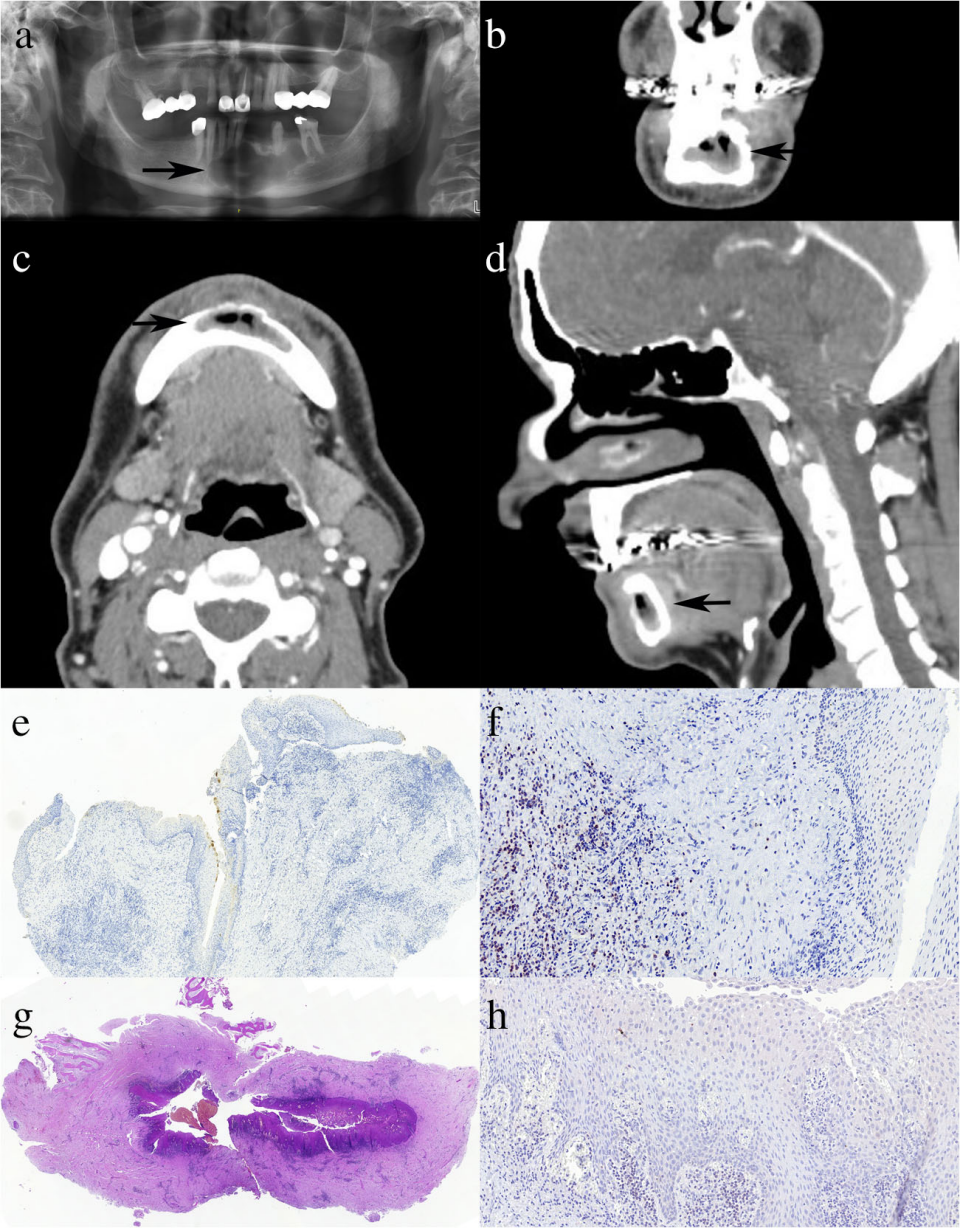
Inflamed OKC without residual typical epithelium. A-D: orthopantomogram, and details of a coronal, axial and sagittal plane images from the computed tomography scan of the cyst highlighted by arrows (case K19); note the large corticated radiolucent area with scalloped borders growing “within the bone” without conspicuous lingual or labial/buccal extension. E: Non-specific focal superficial CK17 labelling of the lining epithelium (CK17, ×5); F: The epithelium is bcl2-negative in the previous biopsy sample with strong lymphocyte staining as internal control (bcl2, ×20); which is much weaker in the decalcified resection specimen (H, bcl2, ×18); G: Overview of the inflamed cyst wall with fragments of bone on the top part of the image (haematoxylin and eosin, ×1.4)

Altogether, the final revised clinicopathological diagnosis was (or was consistent with) OKC in 21 cysts from 13 patients (24.7%;) of which 16 cysts had this diagnosis mentioned on the histology request form. These cases included two recurrent OKCs (with only one mentioning the previous operation on the request form) and 12 cysts from 4 patients associated with the Gorlin-Goltz syndrome. Further 3, 1 and 1 cases were submitted as residual, dentigerous or NOS cysts, respectively, the latter also raising the possibility of an ameloblastoma. Nineteen of these OKCs had typical histological findings including a thin parakeratotic epithelium with wavy surface and basal palisading of nuclei, commonly with an even epithelial stromal interface. They also showed a diffuse and strong transepithelial CK17 and basal/suprabasal bcl2 staining. One syndromic case also demonstrated areas with superficial layer negativity, but had other areas of well-developed transepithelial staining. A further case (K2) showed thickened non-keratinizing squamous epithelium, with a tiny focus of desquamated epithelium with basal palisading and marked basal bcl2 staining and patchy (non-OKC-like) transepithelial CK17 staining. In addition, CK10 staining was also available and demonstrated a superficial focal staining. Other areas of the non-OKC-like epithelium demonstrated transepithelial CK19 positivity. These IHC results were at least partially in keeping with the typical patterns seen in OKCs, and the radiology and clinical features were also consistent with this diagnosis, despite the original clinical diagnosis of a residual cyst (Figure 2). On revision of the case and recut slides, a tiny part of more typical OKC epithelium was also discovered, this demonstrated the typical transepithelial CK17 and basal bcl2 staining (Fig. 2 M, N, O). One further cyst (K19) demonstrated a non-specific thickened squamous epithelial lining in a background of heavy inflammatory infiltrate. None of the 6 blocks taken from this cyst of 43 mm in greatest dimension demonstrated the histological or IHC features of an OKC. However, on clinical and radiological grounds (dimensions, mesiodistal growth without buccolingual extension), the cyst was interpreted as best fitting the diagnosis of and inflamed OKC with modified epithelium, in keeping with the original clinical diagnosis (Figure 3).

From the clinical side, 31 cases had the diagnosis of OKC mentioned on the histology request form either as a clinical diagnosis or as part of clinical differential diagnoses or reflecting the surgeon’s fear of missing an OKC and asking on OKC just as on possible signs of malignancy. Sixteen of these proved to be OKCs, whereas 5 were finally diagnosed as dentigerous cysts or were consistent with this diagnosis (K3, KR10, S3, S17, S21), 7 were classified as radicular/residual cysts (S7, S18, S19, S32, S34, S37, S39), and finally 4 cases (S6, S8, S27, S41) had a diagnosis of odontogenic cyst NOS. None of the latter demonstrated the HE features of OKCs, or a strong transepithelial CK17 and basal/suprabasal bcl2 positivity. However, weak focal superficial to marked superficial to focal transepithelial CK17 staining was identified in their majority (see below).

**Fig. 4:**
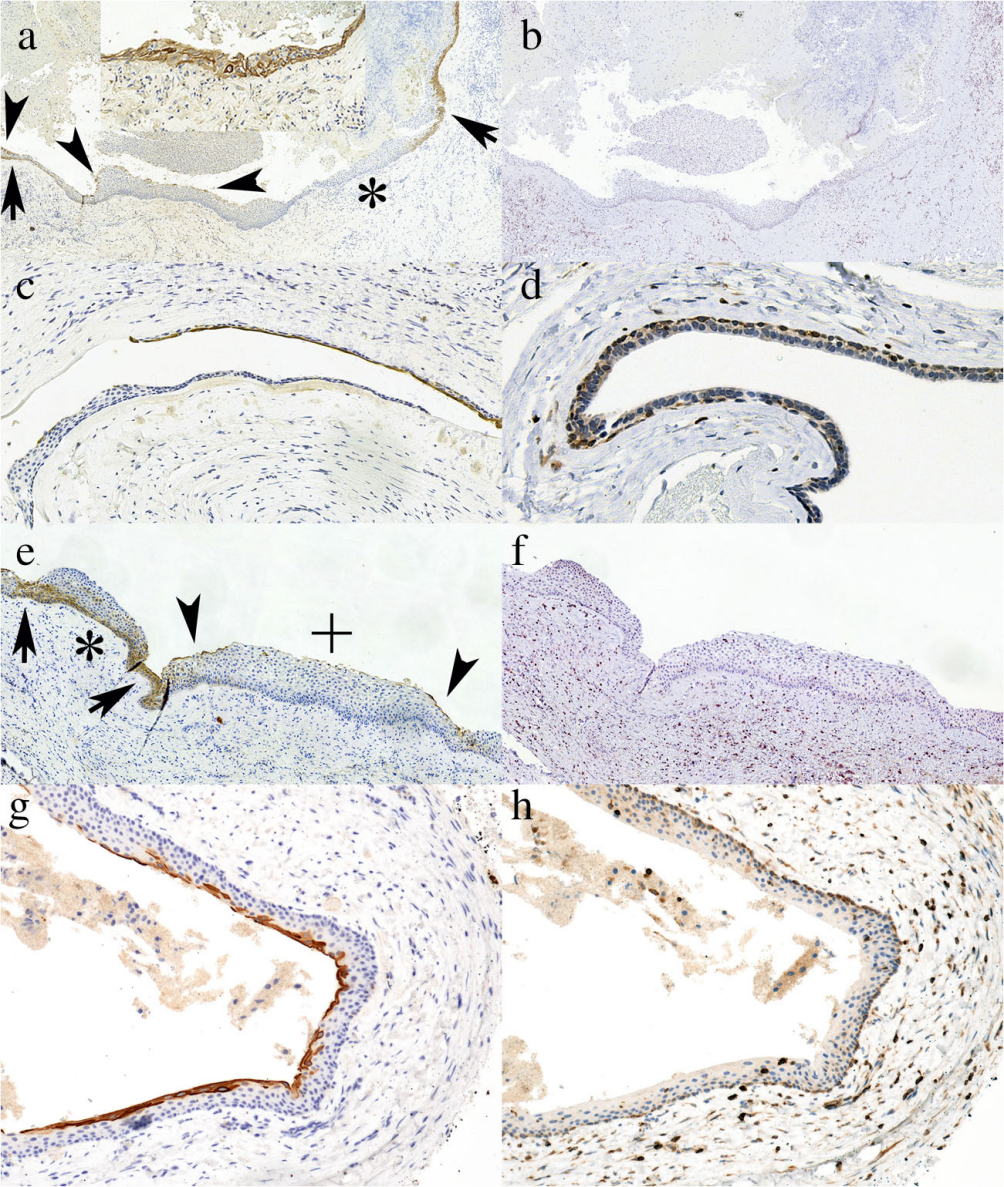
Examples of non-OKCs with CK17 and / or bcl2 positivity. A-B: Mature radicular cyst (Case K5) with various patterns of CK17 positivity (A) including basal (arrows), superficial (arrowheads), transepithelial (inset) staining and negativity (*) and no bcl2 staining (B). C-D: Botryoid odontogenic cyst (Case K9) with partial superficial CK17 positivity (C) and basal bcl2 positivity (D). E-F: Dentigerous cyst (Case K15) with various patterns of CK17 positivity (E) including superficial (arrowheads), basal (*), transepithelial (arrows) staining and negativity (+), associated with bcl2 negativity (F). Bcl2 was basally positive elsewhere. G-H: dentigerous cyst (Case S21) with superficial CK17 (G) and basal bcl2 (H) stainings (A-B: ×5, C: ×20, D: ×40, E-F: ×10, G-H: ×20; A, C, E, G: CK17; B, D, F, H: bcl2)

When taking into consideration the 64 cysts with a non-OKC final clinicopathological diagnosis, a generally focal superficial CK17 staining with a few cells of the luminal layer nearly unnoticeably labelled to definite superficial cell positivity was very commonly seen (*n* = 16), and this was either associated with focal transepithelial staining or focal transepithelial staining occurred without such superficial staining in many (*n* = 24) further cases. Additionally, some cases (*n* = 10) showed basal or upper to middle spinous layer cells labelled focally (Figure 4). Only 14 cases showed no CK17 positivity at all in the investigated tissue block. Bcl2 was generally interpreted as negative in these cysts, but the basal cell layer was stained either focally or diffusely in 16 cases, and these included 8 dentigerous cysts (K15, KR3, KR7, KR11, S3, S17, S21, S27), 1 botryoid odontogenic cyst (K9) 1 nasopalatine duct cyst (S38), 1 inflamed lateral periodontal cyst (K7), 4 residual/radicular cysts (K6, S20, S34, S37) and 1 odontogenic cyst NOS (S8) (Figure 4). Focal weak/moderate intensity spinous cell or transepithelial labelling was seen in 5 cases with non-OKC histology.

Overall, 7 non-OKC cases (K7, K9, K15, KR7, KR11, S27, S37) had both a focal transepithelial CK17 and a basal bcl2 staining. Of these, a botryoid odontogenic cyst (K9) had distinctive histology (Fig. 4 C, D); the remaining included 4 dentigerous cysts, a mature radicular cyst (S37), and an inflamed lateral periodontal cyst (K7). None of the IHC stainings overlapping with the transepithelial CK17 and basal/(suprabasal) bcl2 positivity of the OKC had patterns (stainings occurring at the same site) and intensity to be mixed up with that seen in OKCs.

In contrast, 12 OKCs with inflammation and areas of modified epithelium associated with it, showed a staining typical of non-OKCs in these areas, in keeping with the altered epithelial lining of the cysts. Lack of the marked basal bcl2 staining and negativity or variable superficial or focal, weaker, patchy transepithelial CK17 staining were the characteristic patterns seen in these areas (Fig. 3) with no combination of strong transepithelial CK17 and basal bcl2 positivity. To note, bcl2 staining of control lymphocytes was weak after decalcification of case K19 compared to the biopsy sample of the same cyst and samples of other cysts (Fig. 3, Supplementary material).

## Discussion

Of the odontogenic cysts, OKC is one of the most challenging ones. It is sufficiently common, has a relatively high rate of recurrence if not completely removed, and can have outgrowths, daughter cysts making it difficult to be removed in toto. Although OKCs have clinical and radiological features allowing for a clinical suspicion or diagnosis, there are other lesions with overlapping qualities, therefore a histological confirmation of the diagnosis is always required. On the other hand, although OKCs have a typical histological appearance (Fig. 2 M, N, O), these characteristics may be completely or largely lost in association with secondary inflammation. This is why combination of clinical, radiological and pathological findings are optimally needed for the proper diagnosis of the lesions. This combination of data was missing in a number of cases for the original reports, and was compensated for by the clinical, radiological and pathological review of all available data from the patients’ charts. This review of each individual case is a special strength of this study.

The distinctive CK17 and bcl2 IHC of OKCs consists of a strong transepithelial CK17 positivity (with or without focal basal or superficial sparing) and a strong basal (with or without weaker suprabasal) bcl2 positivity (Fig. 1, Fig. 2 M, N, O) [7–9]. Meara et al. have suggested that a strong CK17 positivity in all layers may point to syndromic OKCs, although they noted similar staining in 2/7 of their sporadic OKC cases as well; they also reported focal or only basal or only superficial CK17 labelling in 4/7 non-syndromic OKCs [11]. In keeping with these results, syndromic OKCs generally had strong CK17 staining, but sporadic cases also displayed similar intensities; in contrast, some areas displayed superficial sparing with CK17 in two OKCs from Gorlin-Goltz syndrome patients. Focal basal sparing was also seen in a few OKCs of this series, but all 20 cases studied with typical epithelium had marked transepithelially stained areas.

Secondary to inflammation, an altered epithelium may replace the typical lining of OKCs, and this is characterized by the loss of the typical IHC staining profile as well, although focal (transitional or typical) areas with typical IHC staining may remain and can be helpful in the diagnosis [9]. Twenty of the 21 OKCs of this consecutive series had the typical epithelium and IHC profile at least focally, whereas in one case, the epithelium was completely replaced by a non-specific squamous one lacking the typical IHC staining of OKCs; its diagnosis was established as being most consistent with an inflamed OKC on the basis of clinical and radiological data (Fig. 3). Marsupialization also leads to secondary inflammation, and loss of the typical basal bcl2 staining was interpreted as a sign of treatment effect in OKCs [12].

In contrast to the results of the detailed cytokeratin profiling studies by Aragaki [7] and Tsuji [8] comparing other common odontogenic cysts with OKCs (Fig. 1), many, though not all radicular and dentigerous cysts were not completely negative for CK17, but demonstrated some kind of staining (superficial layer, spinous layer, basal or even focal transepithelial labelling). This was never very strong, but small biopsies obtained during cystostomies may yield a confounding piece of epithelium with OKC-mimicking transepithelial CK17 staining. Others have also reported at least focal CK17 labelling in (some) dentigerous cysts, including the superficial layer staining often observed in the present study [11, 13–15]. Although a study by Zivkovic and colleagues looking at 30 dentigerous cysts reported transepithelial staining in these, using the same E3 clone CK17 antibody as in the present study (although from another manufacturer) [16], their results are in contradiction with our and others’ findings [7, 8, 11, 13–15]. In contrast to previous reports on the lack of expression of CK17 in radicular cysts [8, 16], the present study also found at least focal CK17 positivity in 25/34 radicular cysts. In concordance with the present results, non-transepithelial CK17 staining of the epithelium of some radicular cysts weaker than in OKCs was reported by Stoll et al. [13].

Basal bcl2 labelling was not common in non-OKCs, but was also seen in some other cyst types, most commonly in dentigerous cysts. Several studies have examined the bcl2 staining of OKCs versus other cysts, with the aim of substantiating the neoplastic nature of the former. For example, Piattelli et al. found bcl2 positivity (defined as >10% staining) in only 1/20 dentigerous cysts, and their 20 radicular cysts were all negative, in contrast to the 20 OKCs studied and unanimously showing basal bcl2 labelling. The authors not only proposed that bcl2 could play a role in the aggressive growth of OKCs, but believed that this was also useful as a diagnostic tool [17]. Similar results were reported by others, and finally a recent systematic review concluded that bcl2 positivity was so much greater in OKCs than dentigerous cysts, that it should be partly responsible for the active growth of OKCs through the inhibition of apoptosis, and therefore be a phenomenon in support of the neoplastic nature of OKCs [10]. However, the systematic review included only 36 dentigerous cysts and 4 of these (11%) showed bcl2 positivity [10]. Our study concentrated more on diagnostic uses of staining patterns rather than quantifying the cells stained. As a consequence, we were interested in any staining pattern that could mimic the typical basal positivity of OKCs, even if this was focal. In fact, bcl2 positivity was never as strong and diffuse in other cyst types, but there were some cysts that clearly exhibited continuous basal layer bcl2 labelling of weak to moderate intensity; these were predominantly developmental cysts, mostly dentigerous cysts (8/16 non-OKCs with basal bcl2 labelling), but radicular cysts (especially mature ones with atrophic epithelium) were also identified as having this staining pattern. Our findings are reinforced by the findings of Martins et al., who also found some basal bcl2 expression in non-inflamed radicular cysts although this was less common than that seen in similarly atrophic dentigerous cysts [18]. These authors also proposed that inflammation may inhibit bcl2 expression and therefore contribute to similarities of inflamed cysts of radicular and dentigerous origin. This suggestion may probably be generalized to most inflamed cysts with non-specific squamous epithelium.

The association of a strong basal bcl2 and significant transepithelial CK17 stainings in the same areas of non-OKC was not encountered in this series, but cannot be completely excluded on the basis of the number of cases studied.

The consecutive series (on the basis of the clinicopathological diagnoses), as could be expected, contained mainly the commonest odontogenic cysts, i.e. radicular cysts, dentigerous cysts and OKCs. The latter were slightly overrepresented because of surgeries on 4 patients with the Gorlin-Goltz syndrome and up to 5 OKCs removed from these patients. However, the series also included a few rarer cysts like a paradental cyst (inflammatory collateral cyst) (K17), one botryoid odontogenic cyst (K9), one nasopalatine duct cyst (S38) and lateral periodontal cysts (K7, K16, K22). Of these, the developmental cysts without inflammation demonstrated some (faint to moderate) basal bcl2 staining, without suprabasal extension, and all of the rarer cyst types also displayed various CK17 expression, including focal transepithelial staining (Fig. 4, Supplementary material). None of these created differential diagnostic problems regarding OKC. In fact, the basal bcl2 seen in some dentigerous and botryoid cysts suggests that this pattern may be more common in developmental cysts. A strong bcl2 expression was also described in a botryoid odontogenic cyst reported recently, being the only case studied in this respect we could identify [19].

To summarize, the study strengthens that the strong diffuse transepithelial CK17 and basal bcl2 are typical of OKCs, but also highlights that these patterns of staining, although to a lesser intensity and extent, can be seen in other odontogenic cysts, too. This is in contradiction with some previous publications referring to the CK17 negativity of radicular and dentigerous cysts [7, 8]. Although the characteristic CK17 and bcl2 IHCs are rare in combination in non-OKCs, inflamed OKCs also loose these stains at the sites of altered epithelium, which may form the majority or the whole of the cyst lining. Therefore, these IHC patterns cannot serve as a yes or no alternative of the clinicopathological diagnostic steps of OKCs, but are rather adjuncts that can serve in some difficult cases. The present experience suggests that they have limited value in the differential diagnosis. The only cyst (K2) where the IHC helped to make the diagnosis of OKC was an inflamed OKC with a small bit of transitional epithelium with basal bcl2 and patchy transepithelial (non-typical) CK17 staining and minimal typical residual epithelium identified only on revision and recut.

## Electronic supplementary material

ESM 1 (PDF 36.3 mb)

## Data Availability

The datasets generated during and/or analysed during the current study are available from the corresponding author on reasonable request. The supplementary material contains all cases illustrated.
