# Supplementary material for: A Clinicopathological Approach to Odontogenic Cysts: the Role of Cytokeratin 17 and bcl2 Immunohistochemistry in Identifying Odontogenic Keratocysts
Source: Pathol Oncol Res. 2020 Jul 6;26(4):2613–20. doi: 10.1007/s12253-020-00866-4 (PMC7471163; doi:10.1007/s12253-020-00866-4)
Supplement: Supplementary file 1 — (PDF 36.3 mb) [file 12253_2020_866_MOESM1_ESM.pdf]

Supplementary material (illustration to all cases) of

A clinico-pathological approach to odontogenic cysts: the role of cytokeratin 17 and bcl2 immunohistochemistry in identifying odontogenic keratocysts

by

Dorottya CSERNI, Tamás ZOMBORI, András VÖRÖS, Anette STÁJER, Annamária RIMOVSZKI, Krisztián Daru, Zoltán BARÁTH, Gábor CSERNI

Cases K1-32 and KR1-19

Abbreviations

- CK17: cytokeratin 17
- CT: computed tomography, CBCT: cone beam CT
- IHC: immunohistochemistry
- N.O.S.: not otherwise specified
- OKC: odontogenic keratocyst
- OPT: orthopantomogram
- Rtg: radiograph

- TECHNICAL NOTE: Cases K and KR have CK17 developed with Novared as chromogen (lighter brown) whereas the rest of the IHC is developed with diaminobenzidine (DAB) as chromogen resulting in dark brown colour.

Case K1

- 16-year-old male
- Dental hygiene/status: conserved; treated teeth; impaction of all 3rd molars; ectopic tooth 17 in the maxillary sinus, in horizontal position
- Radiology: „Fluid" filled demarcated cyst around the ectopic tooth 17
- Operation: Caldwell-Luc surgery to remove impacted tooth 18 and ectopic horizontal tooth 17 along with cyst removal from the maxillary sinus
- Clinical diagnosis: follicular cyst mentioned in previous medical records, but histology sent with „inflamed maxillary sinus mucosa" labelling

K1 - OPT

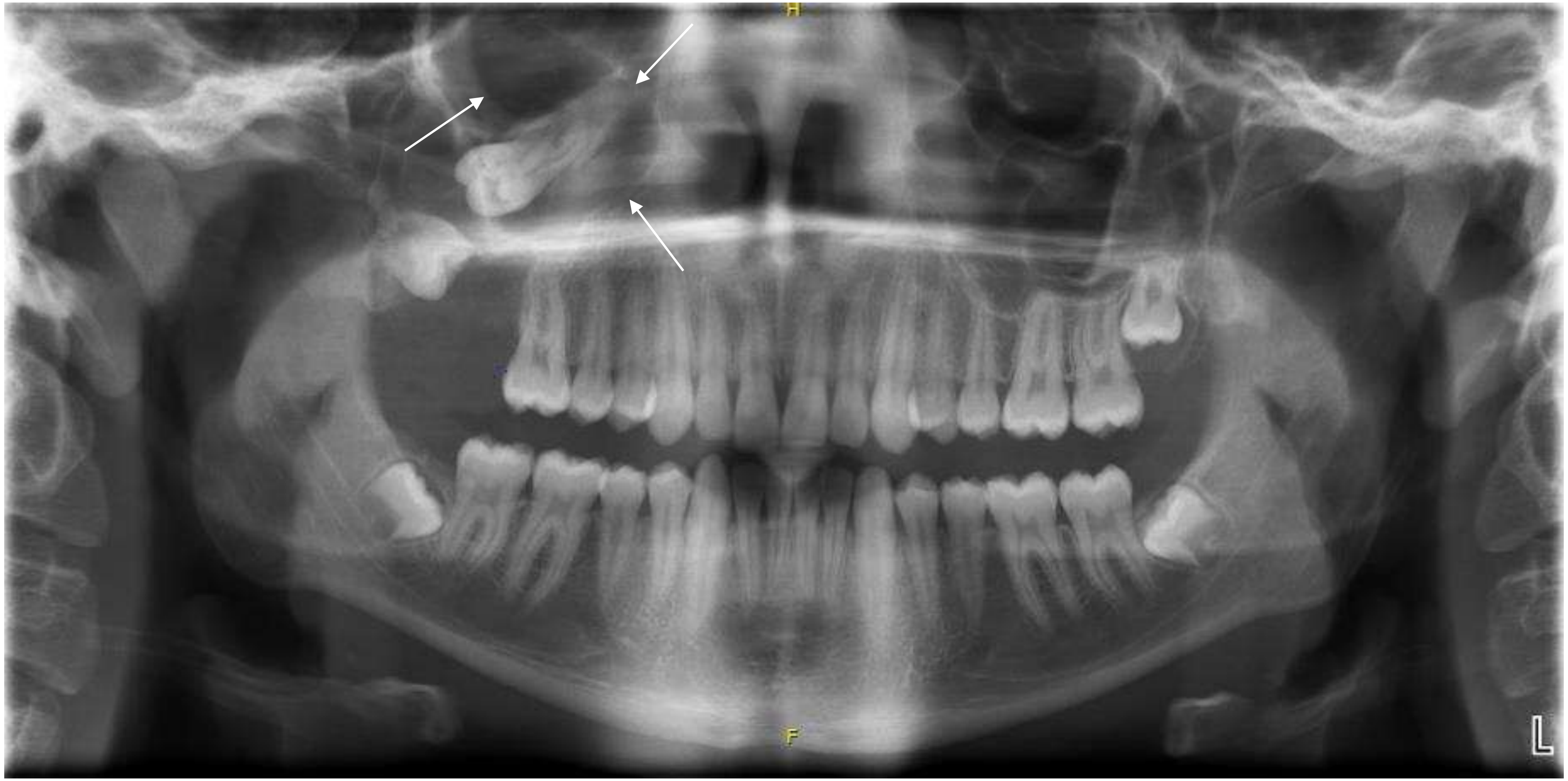

K1 - CT coronal

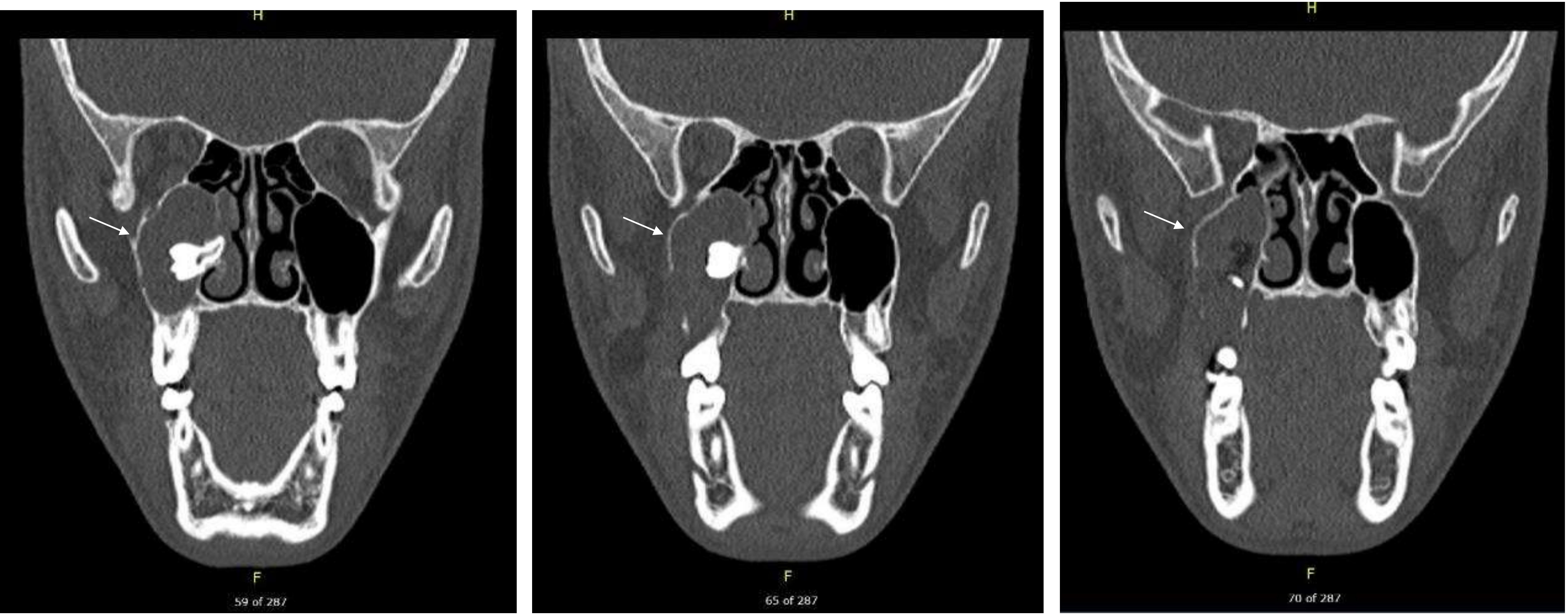

K1 - CT sagittal

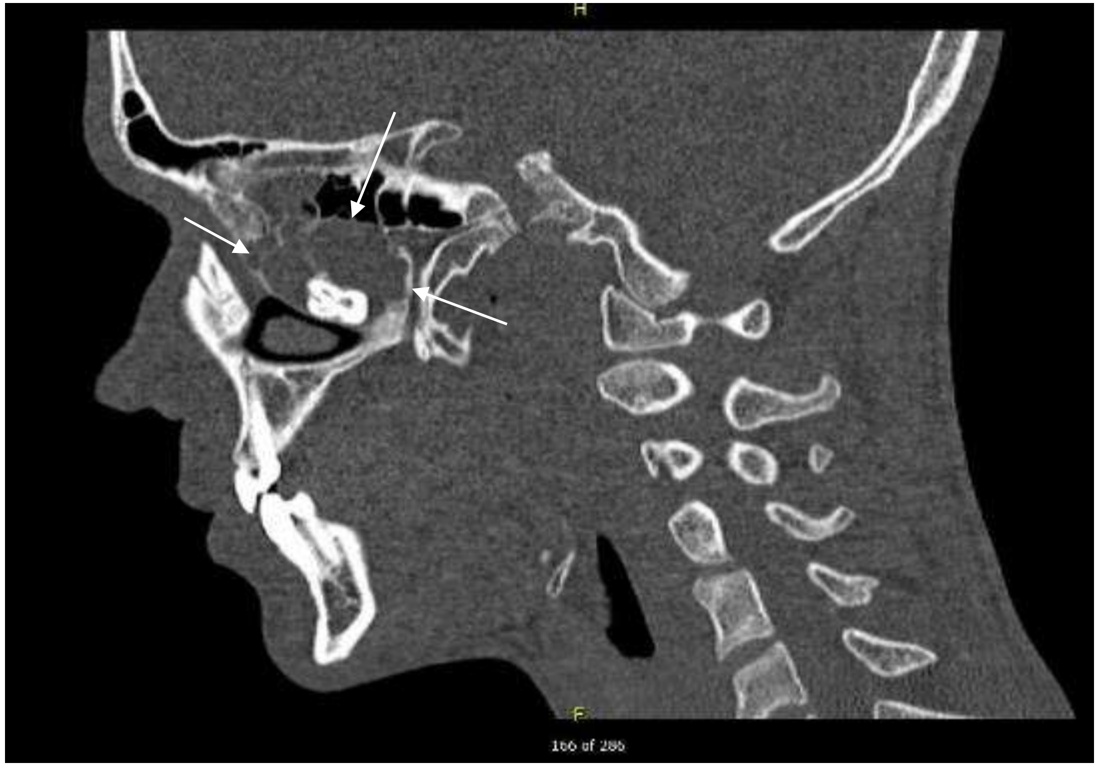

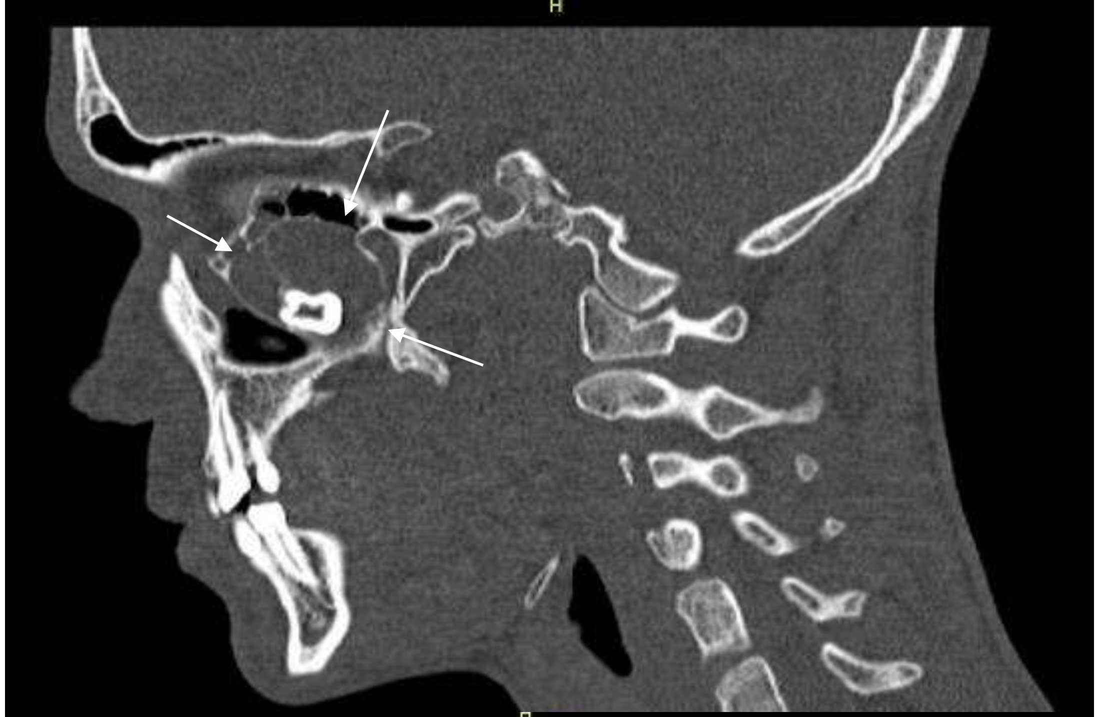

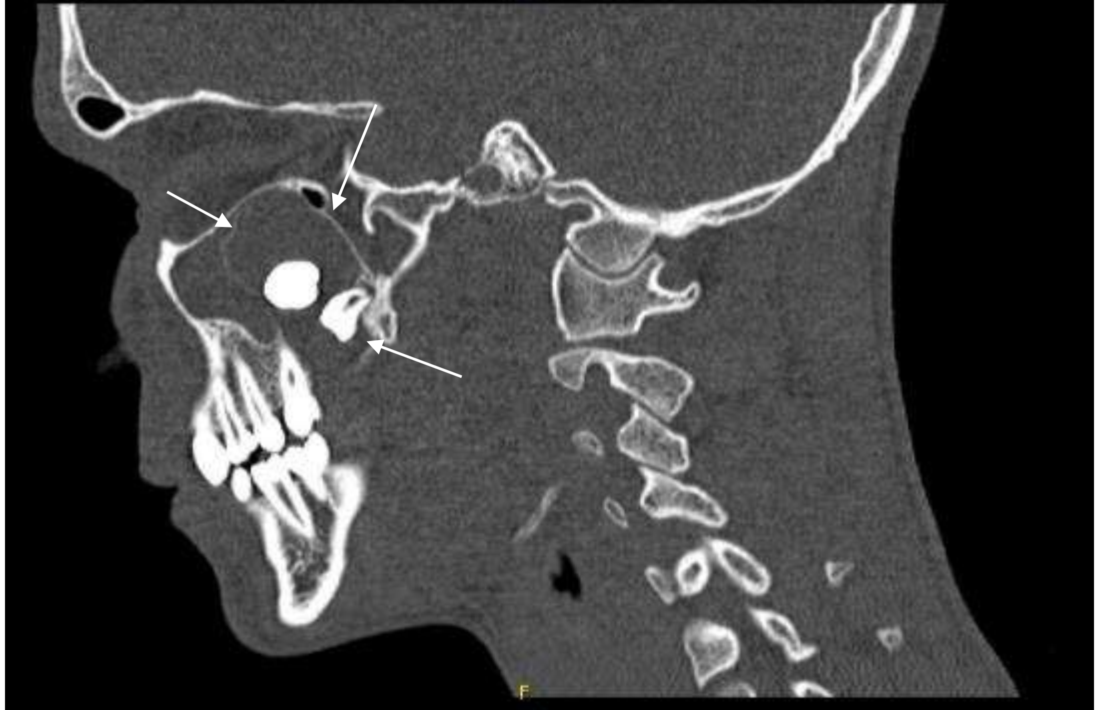

K1 – HE, CK17, bcl2 (x1)

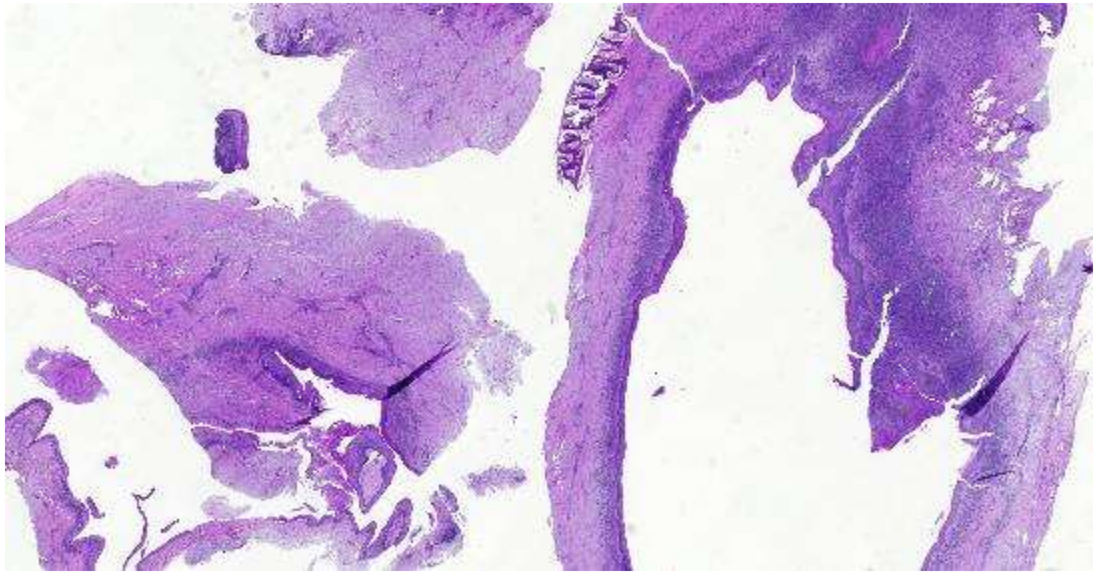

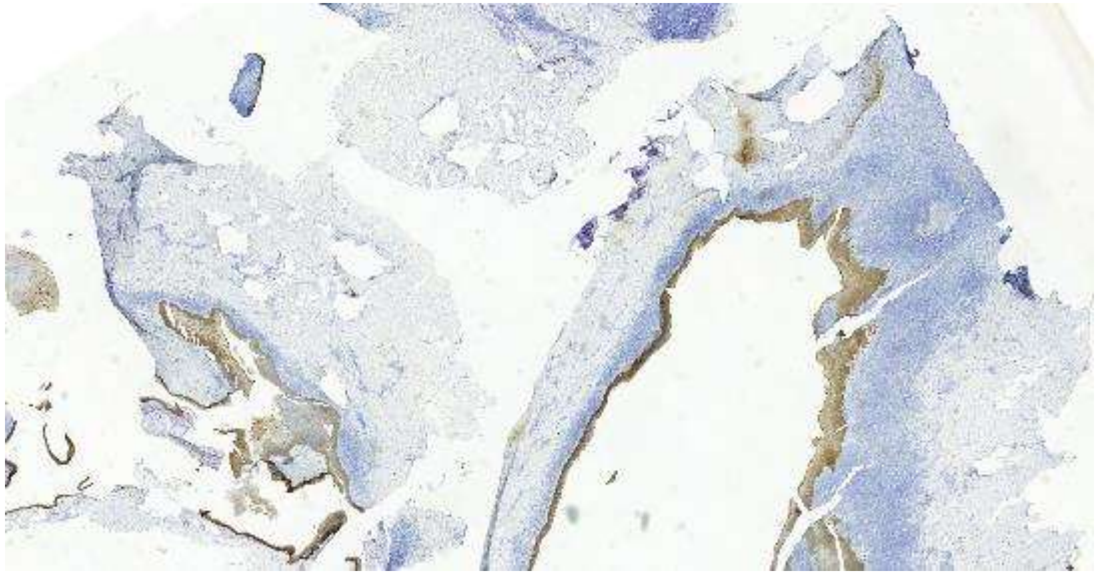

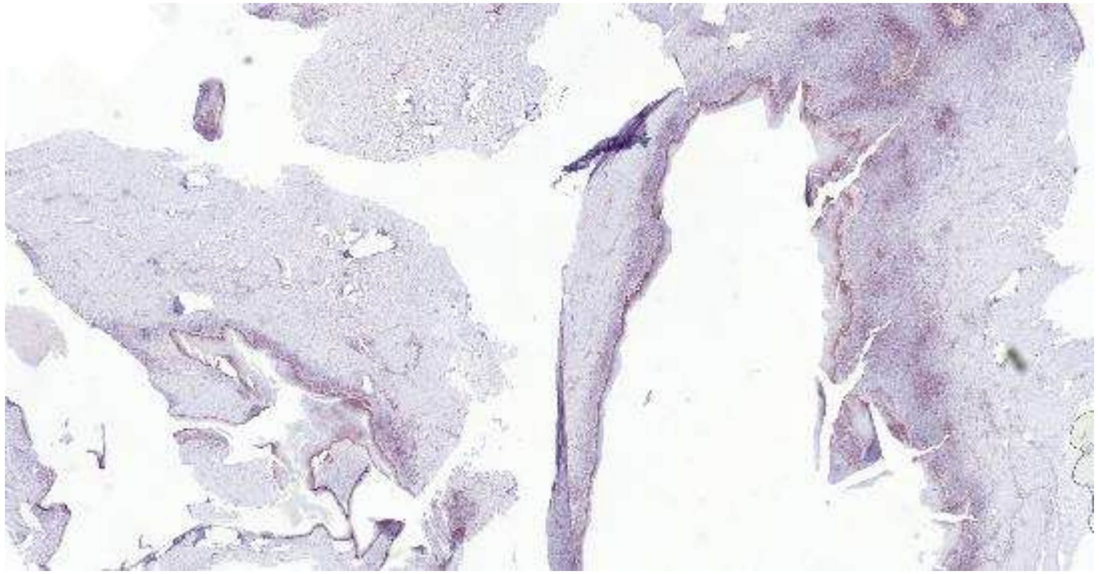

K1 – HE, CK17, bcl2 (x20)

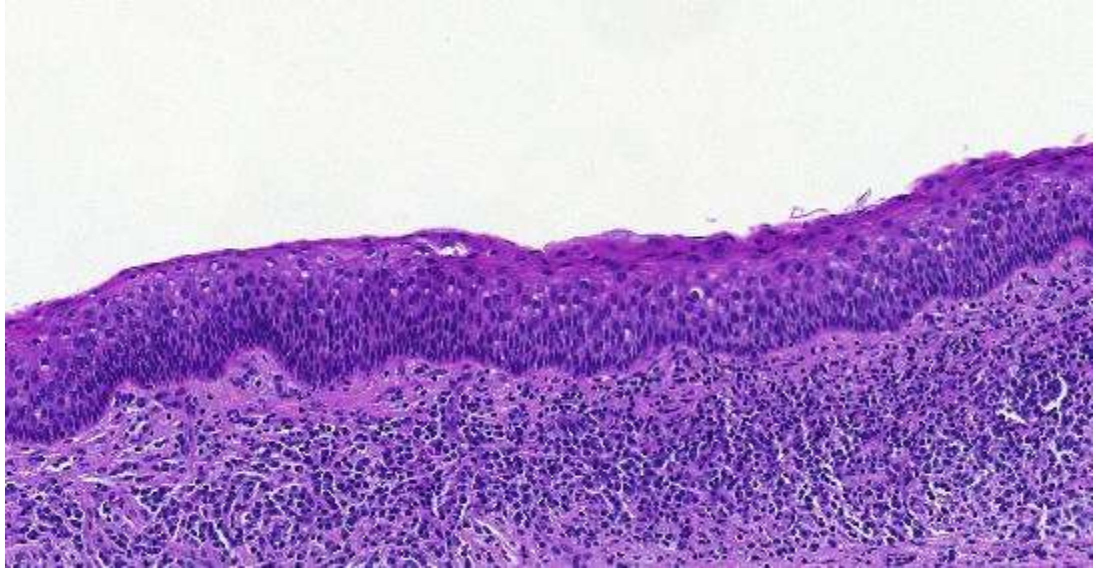

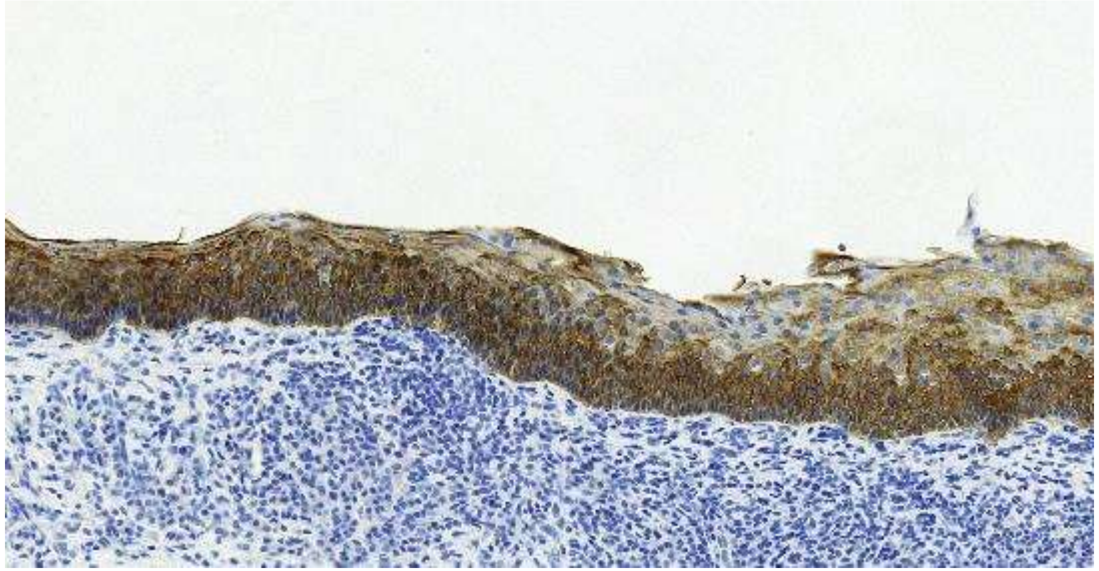

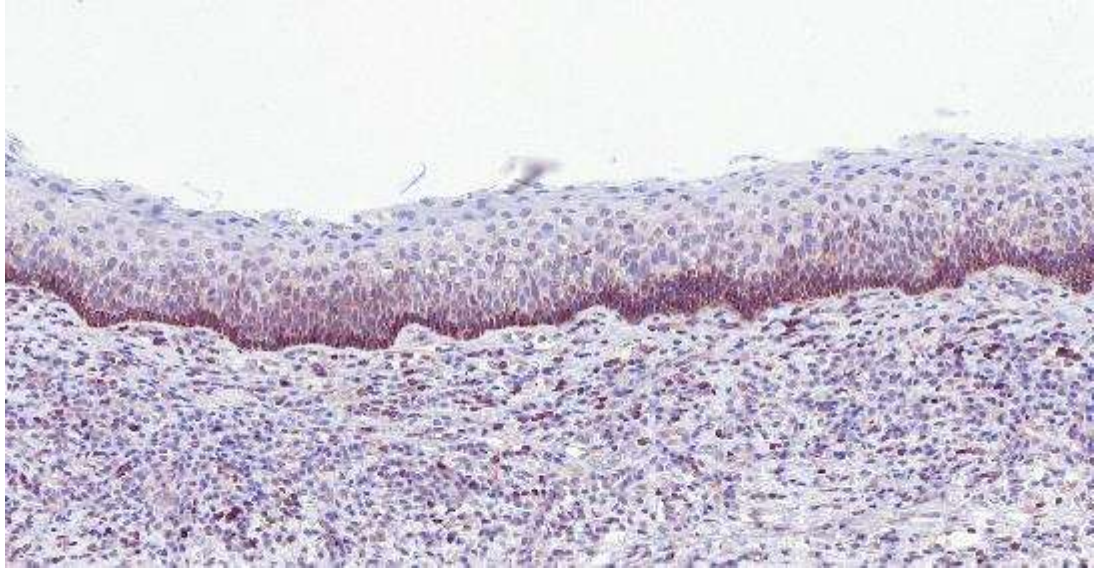

K1 – HE, CK17, bcl2 (x8)

Thick epithelium due to tangential sectioning + inflammation

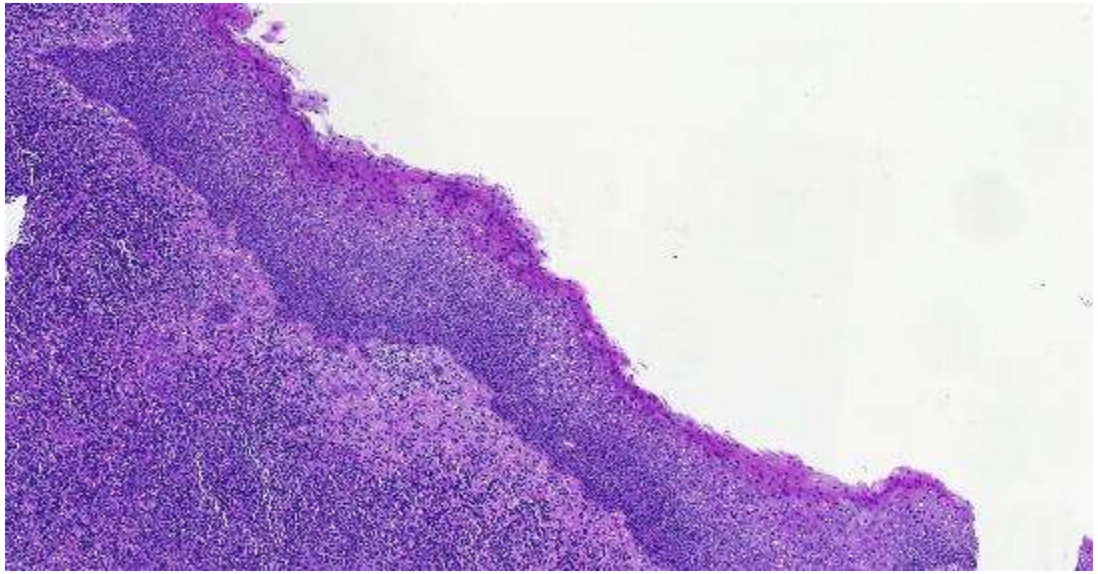

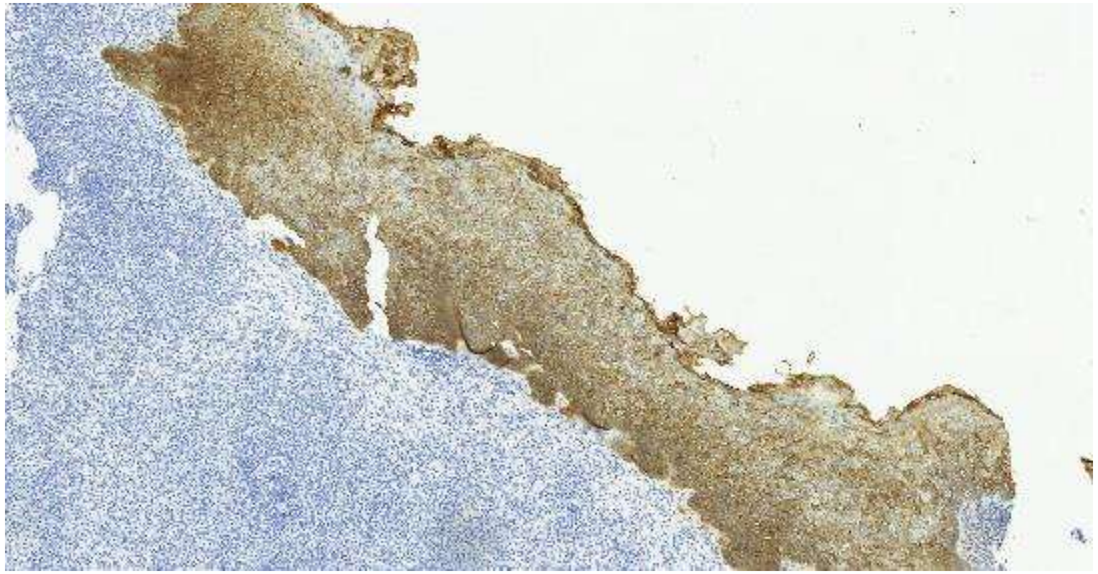

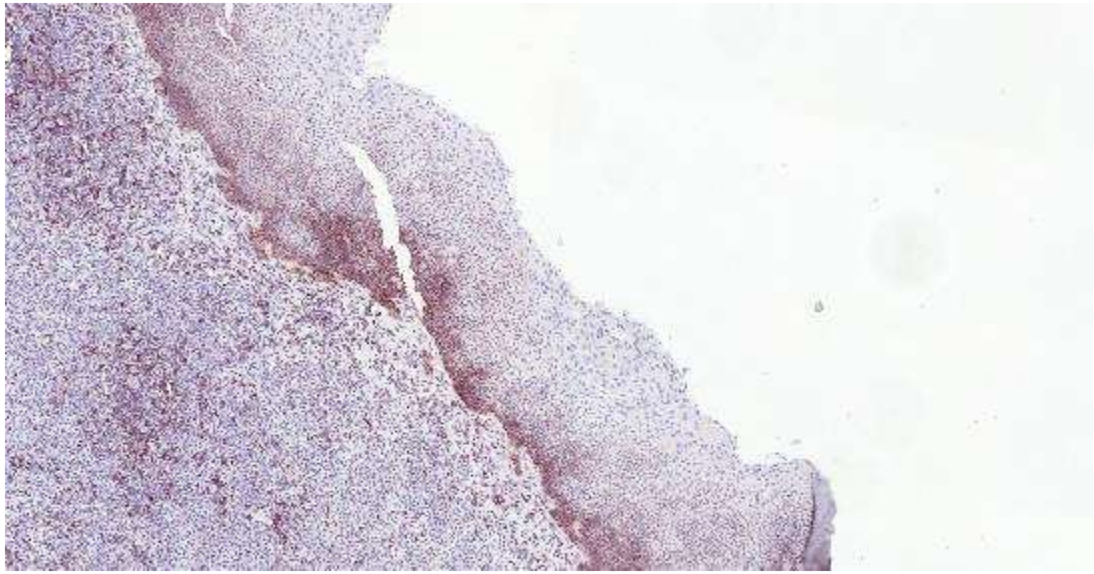

K1 – OKC - typical histology (x30)

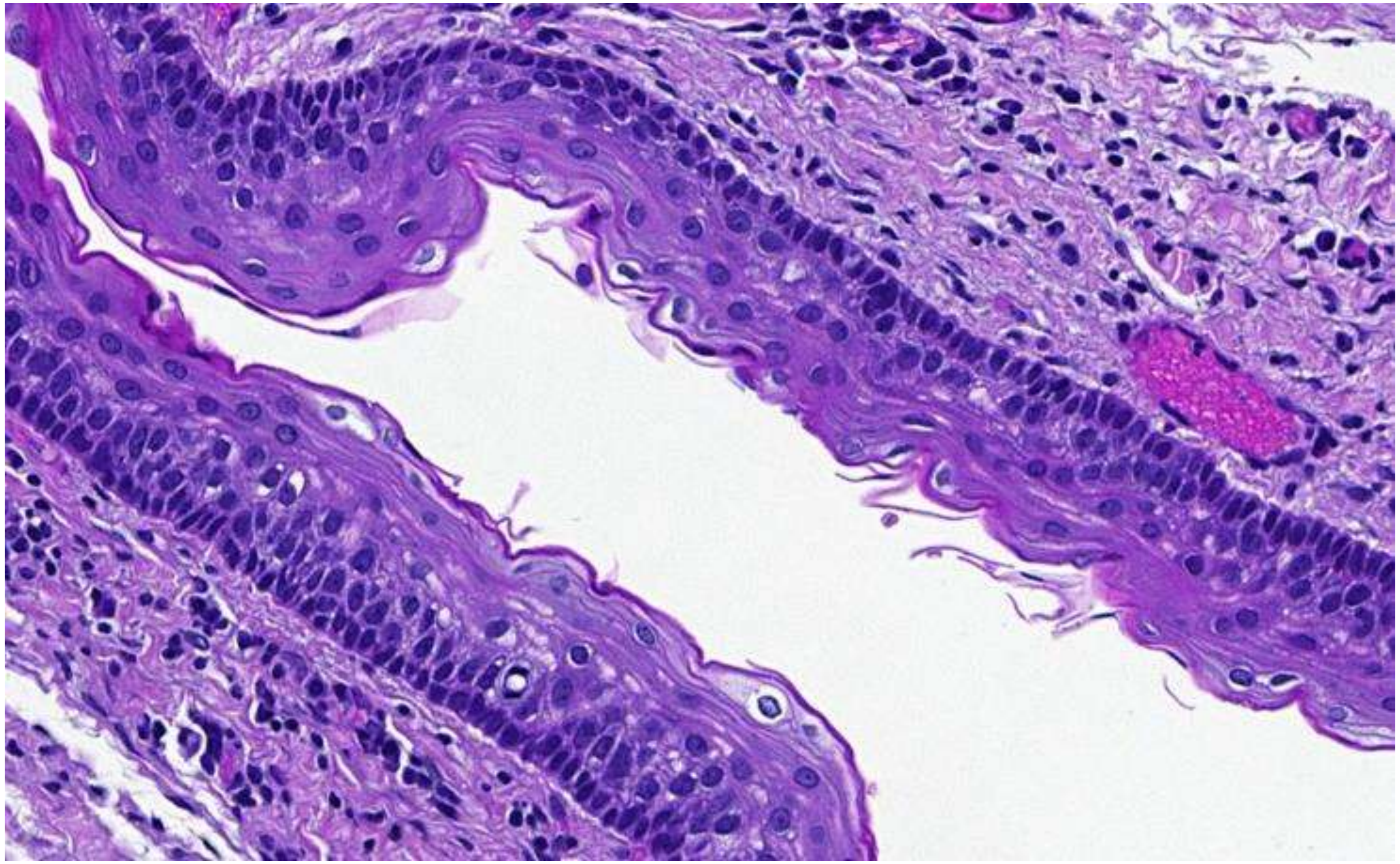

K1 – CK17 (x40) and bcl2 (x30)

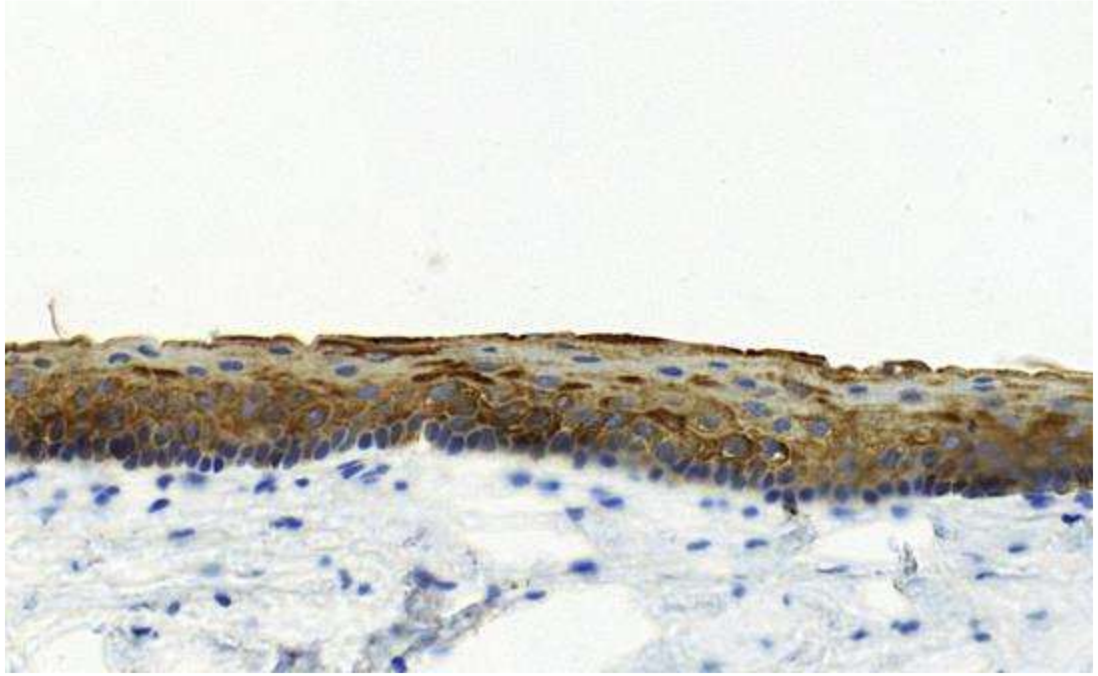

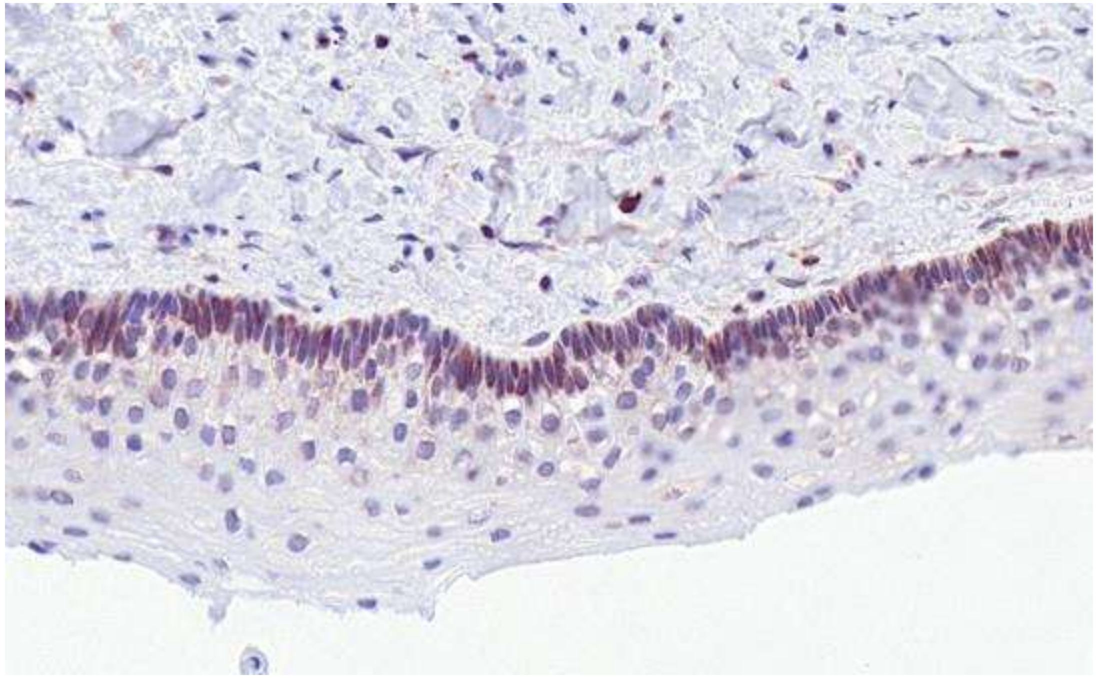

K1 – Conclusion

- H&E: thin (focally thick – tangentially sectioned; see image at x8), parakeratotic epithelium, with wavy surface, basal palisading, uneven epithelial-subepithelial contours
- Focal marked chronic inflammation of the cyst wall with focal acute component, focal thickening of the epithelium, same IHC
- Bcl2: basal / suprabasal positivity
- CK17: transepithelial positivity
- Histopathology against proposed clinical diagnosis: odontogenic keratocyst (OKC) with typical HE and IHC features
- Combined clinical and pathological opinion: OKC

Case K2

- 56-year-old female
- Dental hygiene/status: partly missing teeth, no further details recorded
- Radiology: 3.8x2.5 cm well circumscribed radiolucency in the angle and ramus of the mandible, with a fistula opening (and discharge) in the region of the missing tooth 38.
- Operation: Cystectomy
- Clinical diagnosis: residual cyst mentioned in previous records, sample sent as cyst from the mandible

K2 – OPT

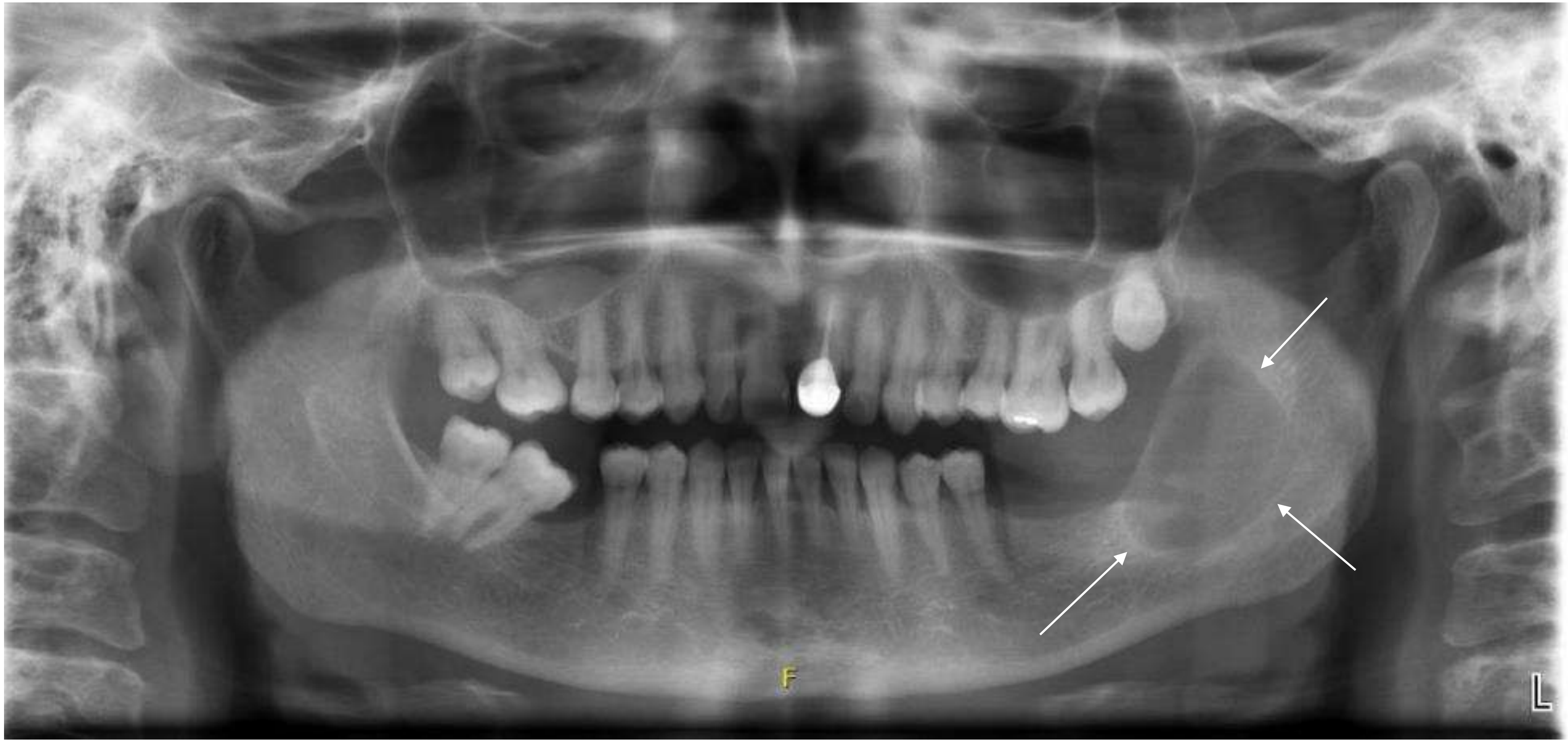

K2 – CT axial

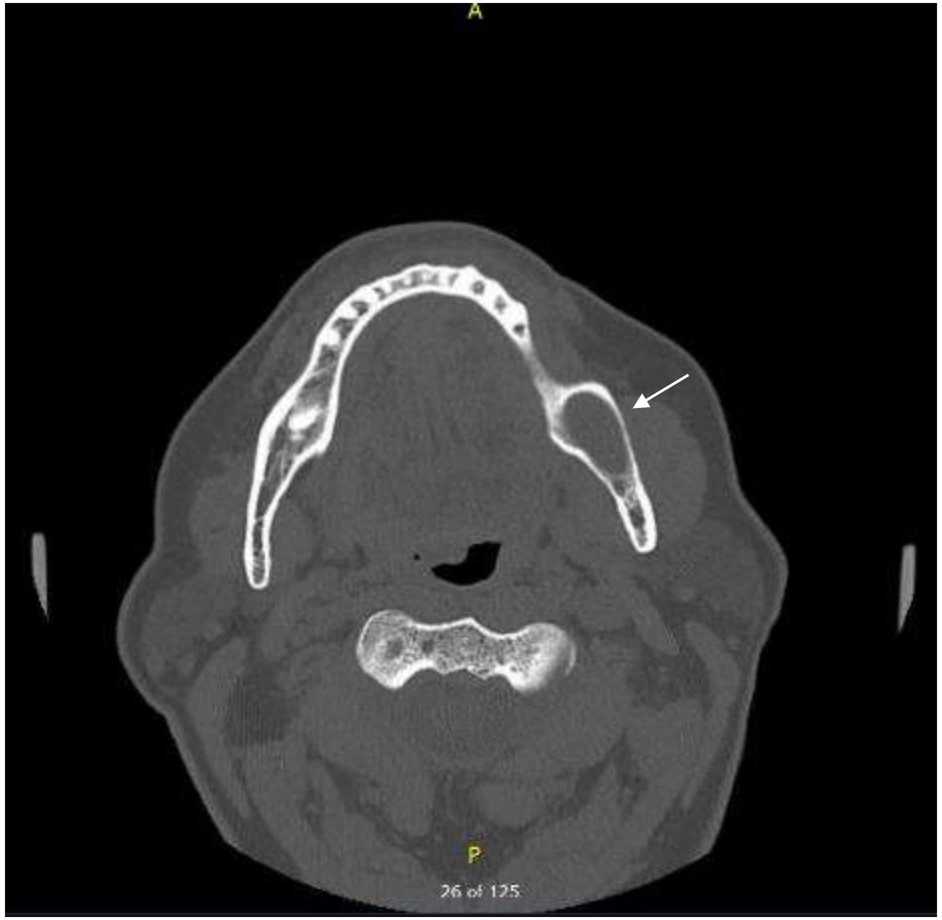

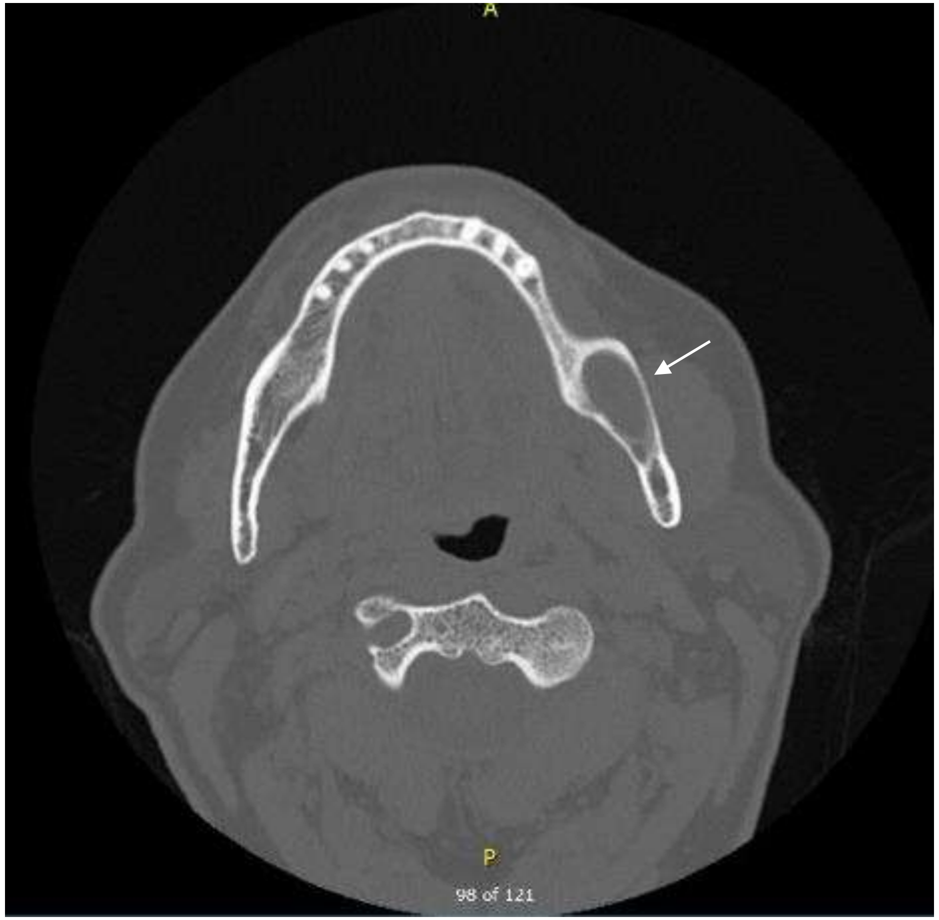

K2 – CT coronal

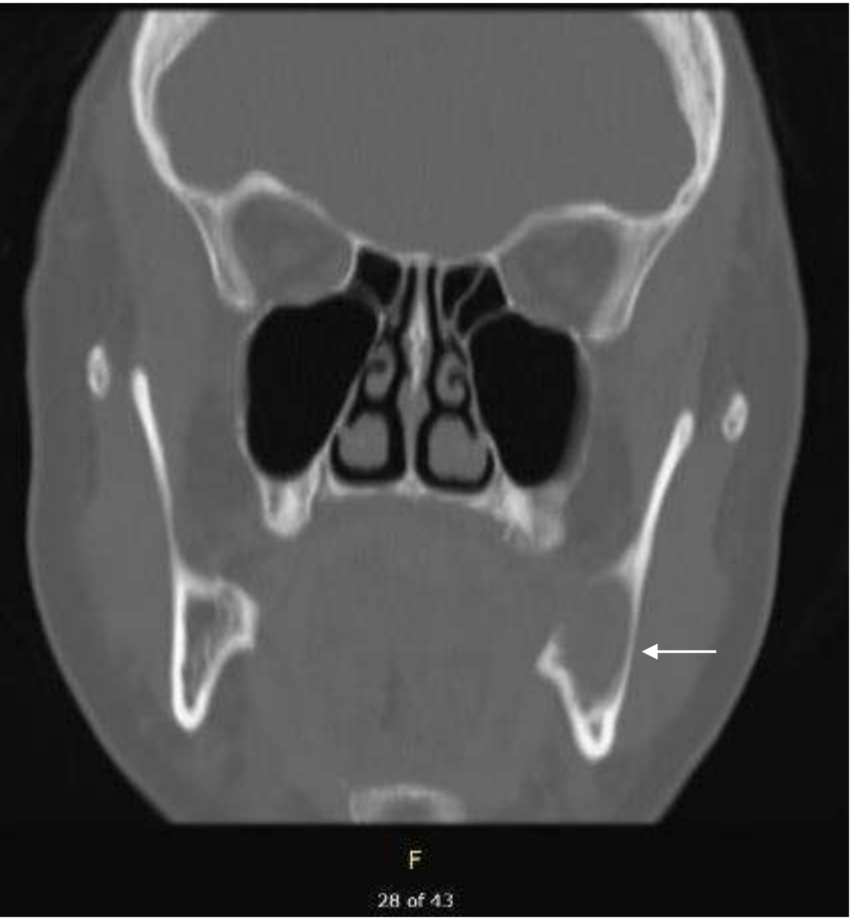

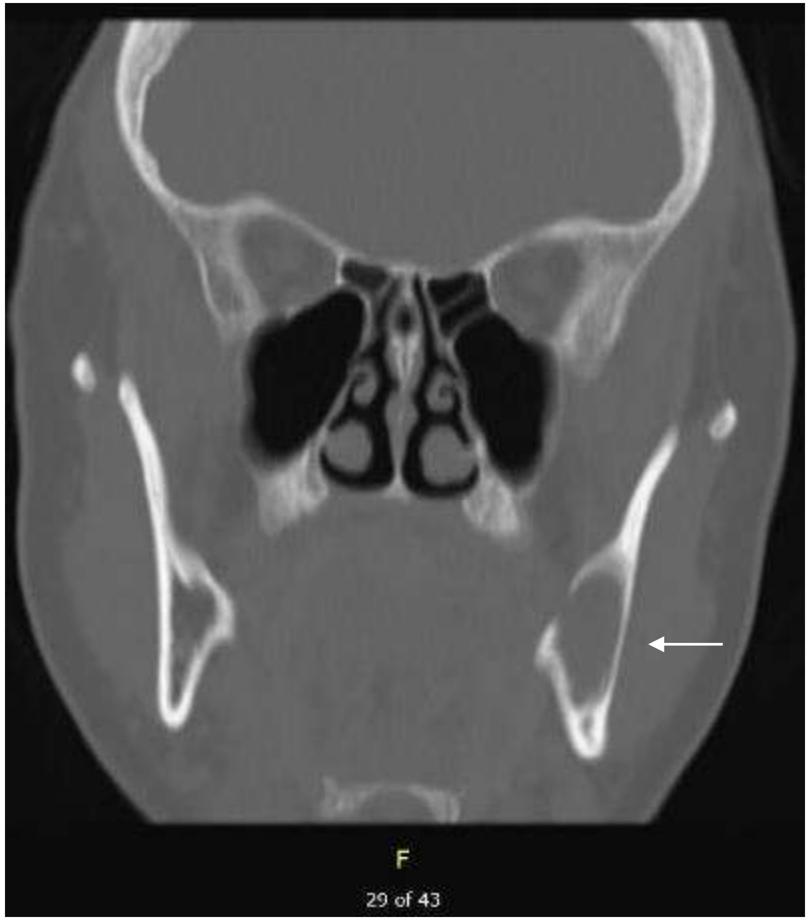

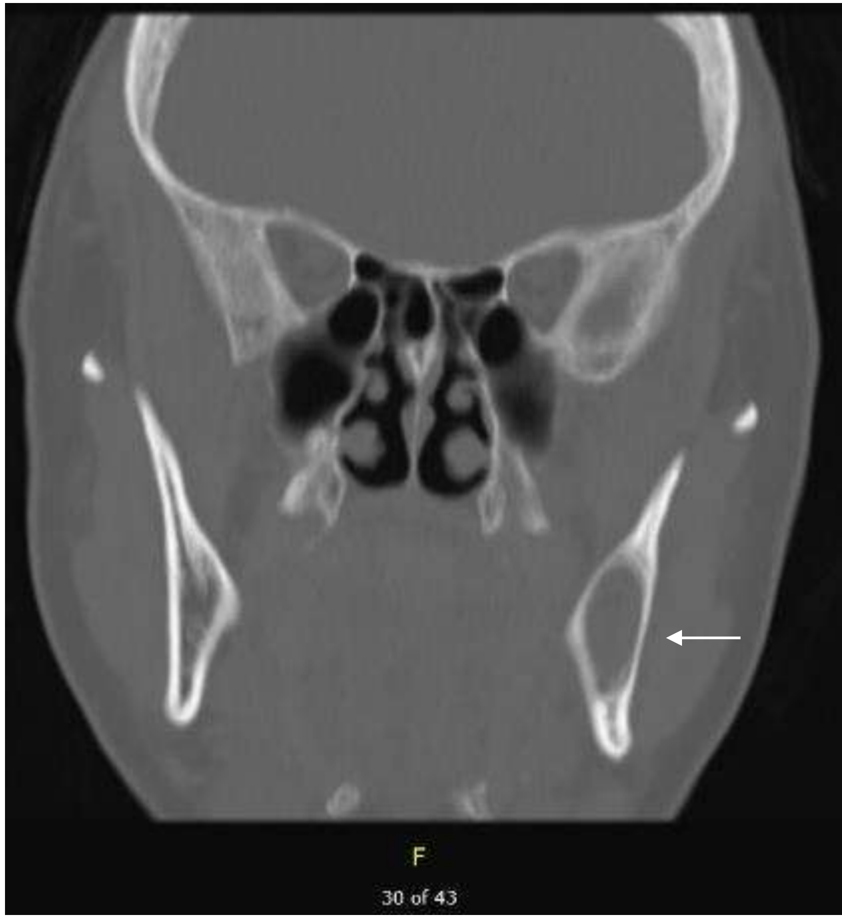

K2 – CT sagittal

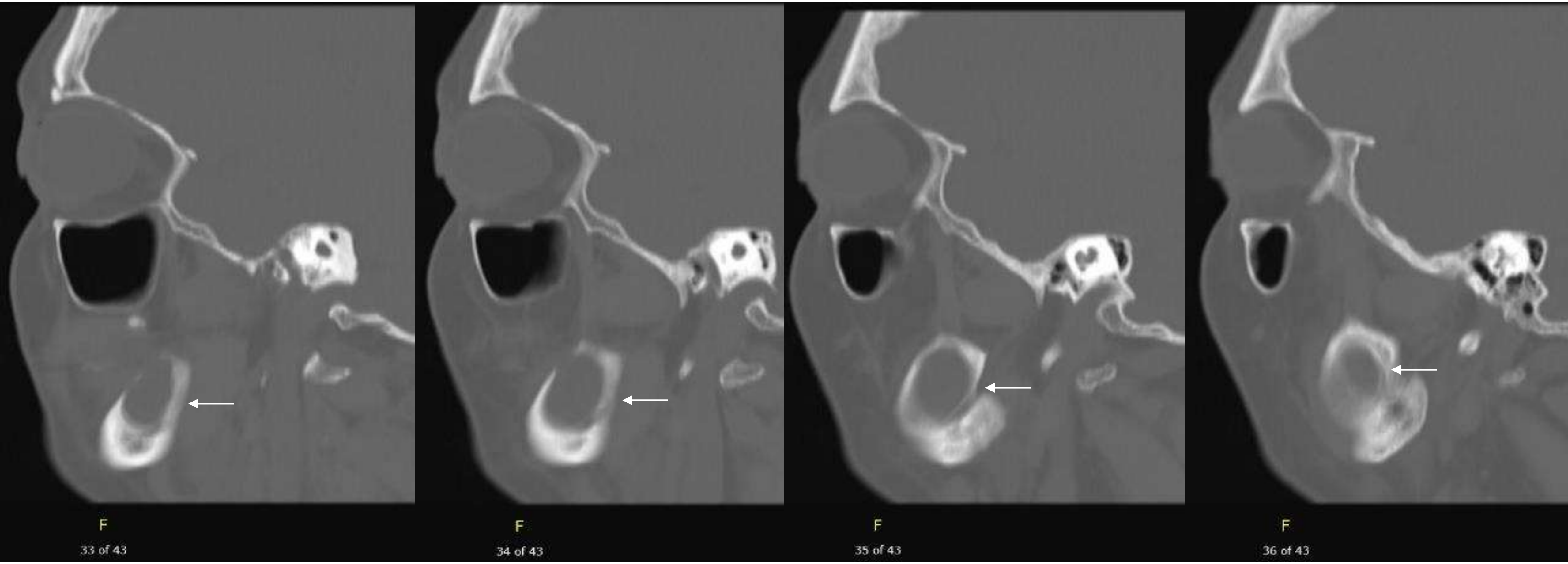

K2 – HE, CK17
bcl2, CK10 (x1)

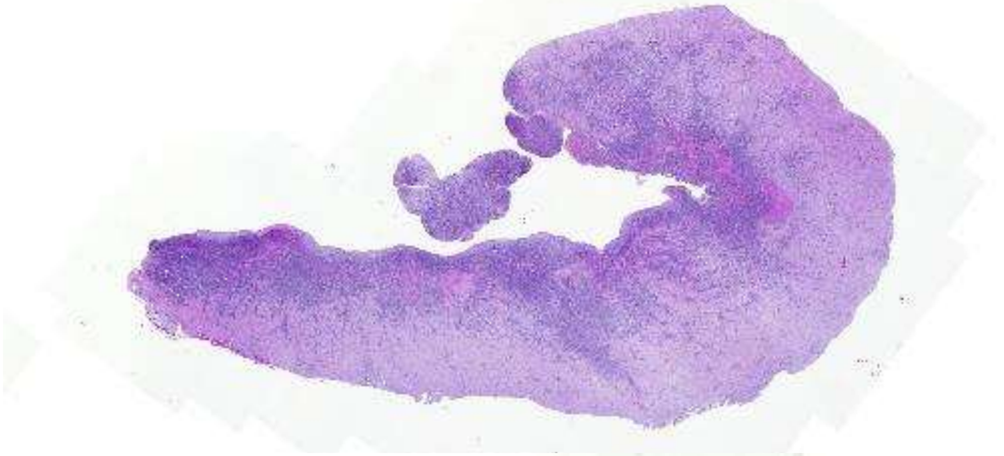

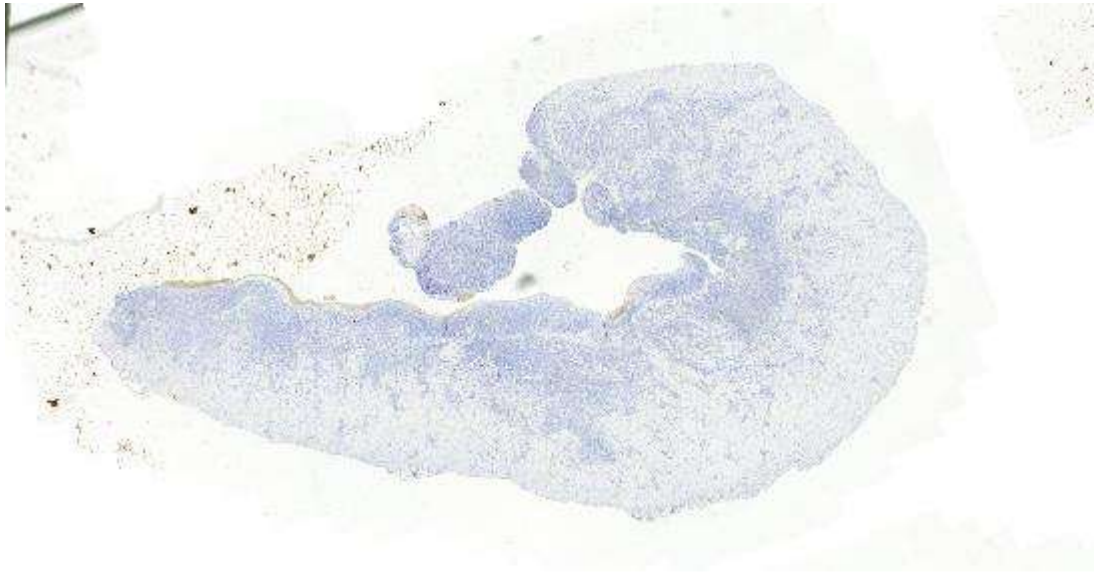

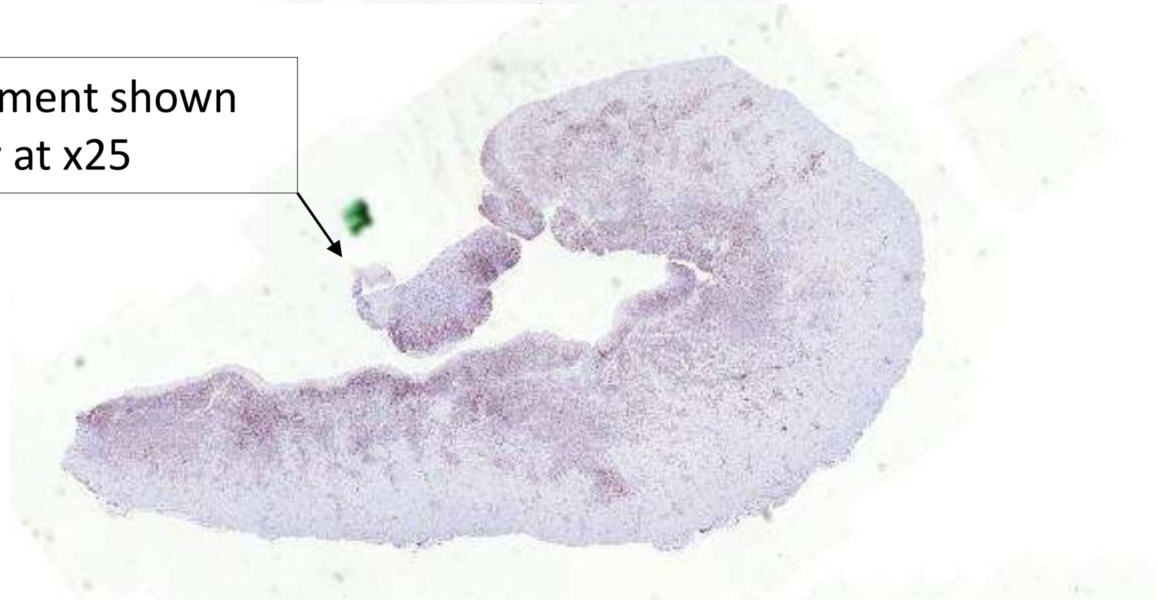

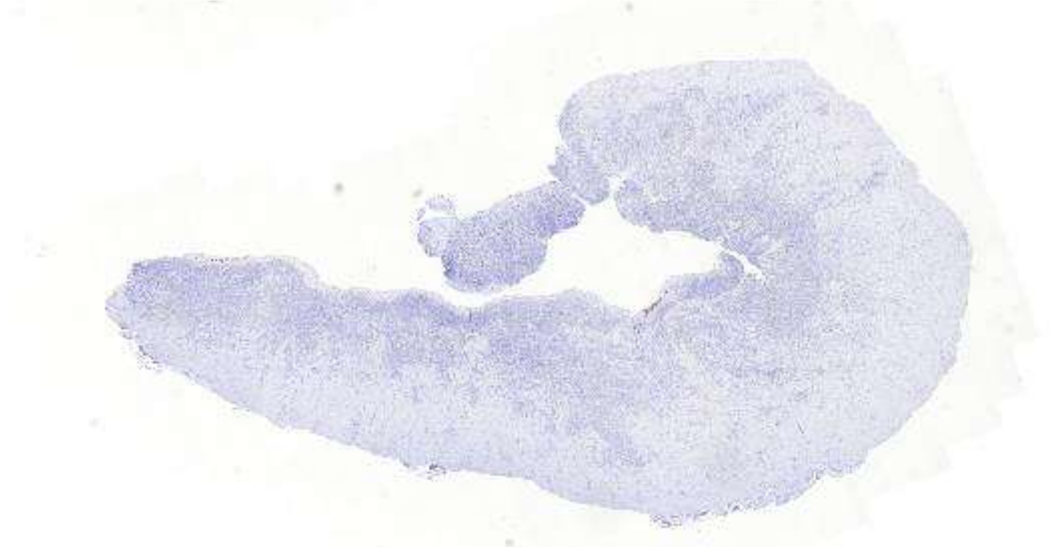

K2 – HE, CK17
bcl2, CK10 (x10) inset: bcl2 x30

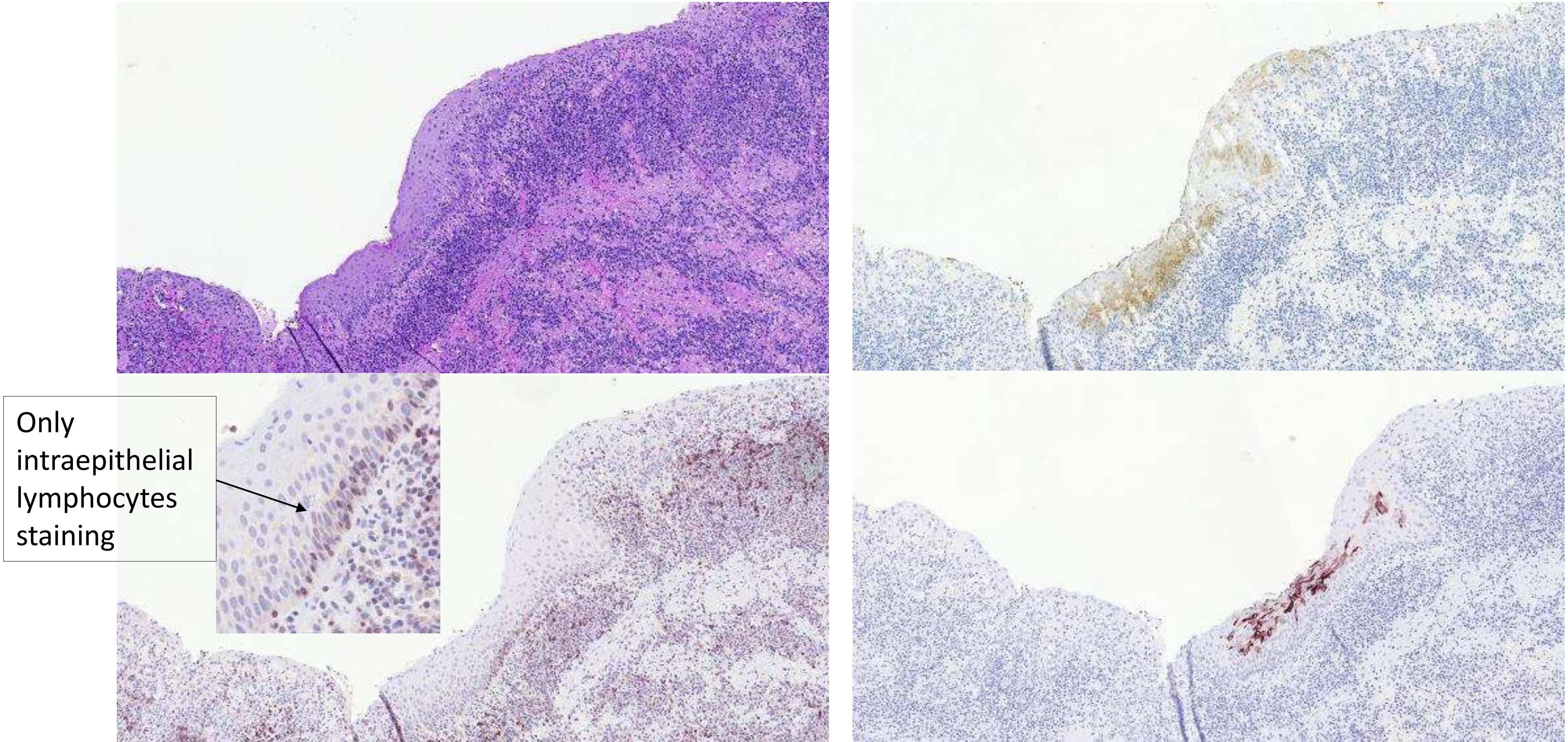

K2 – HE, CK17
bcl2, CK10 (x12)

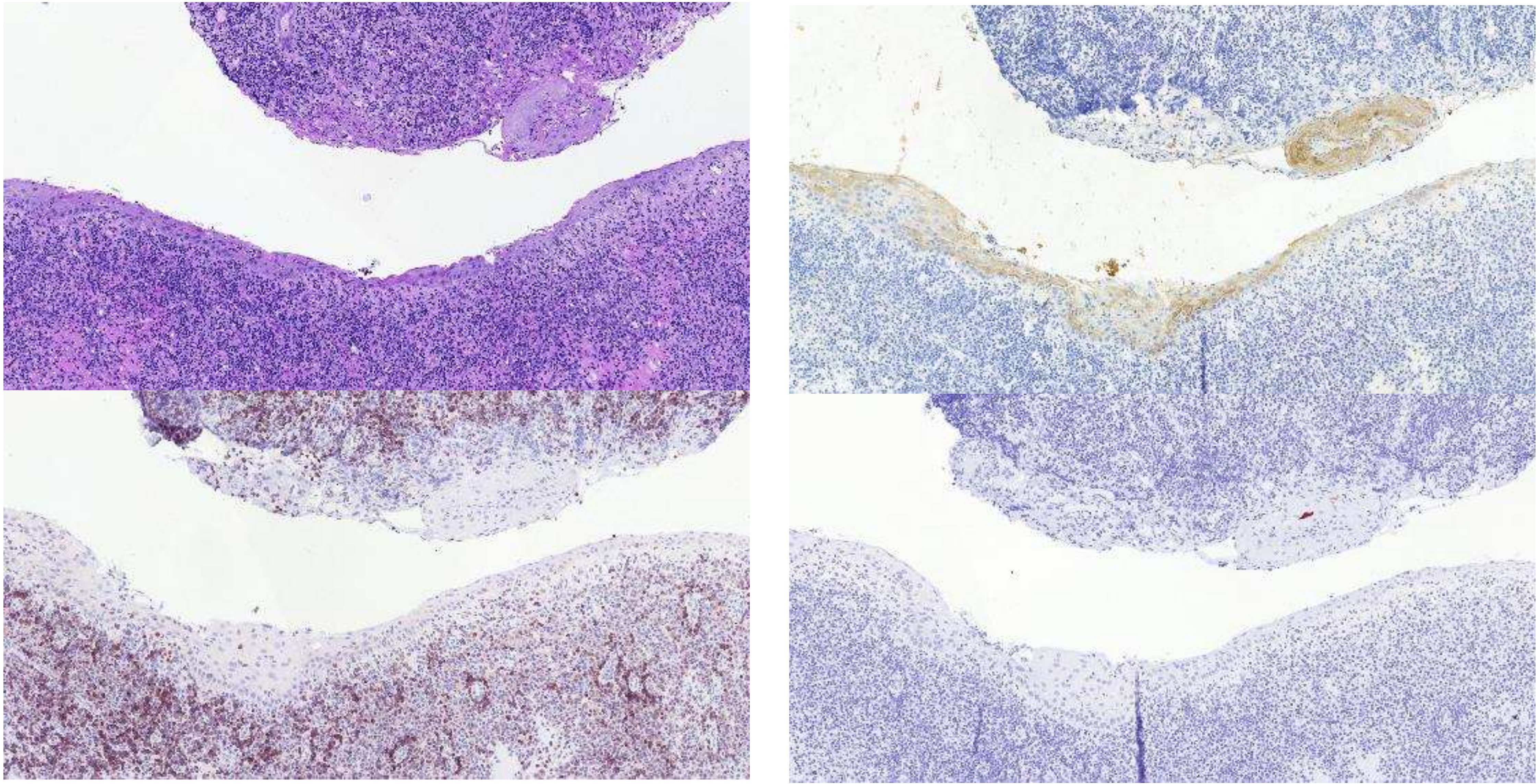

K2 – HE, CK17
bcl2, CK10 (x25)

Highlighted fragment with typical OKC IHC staining

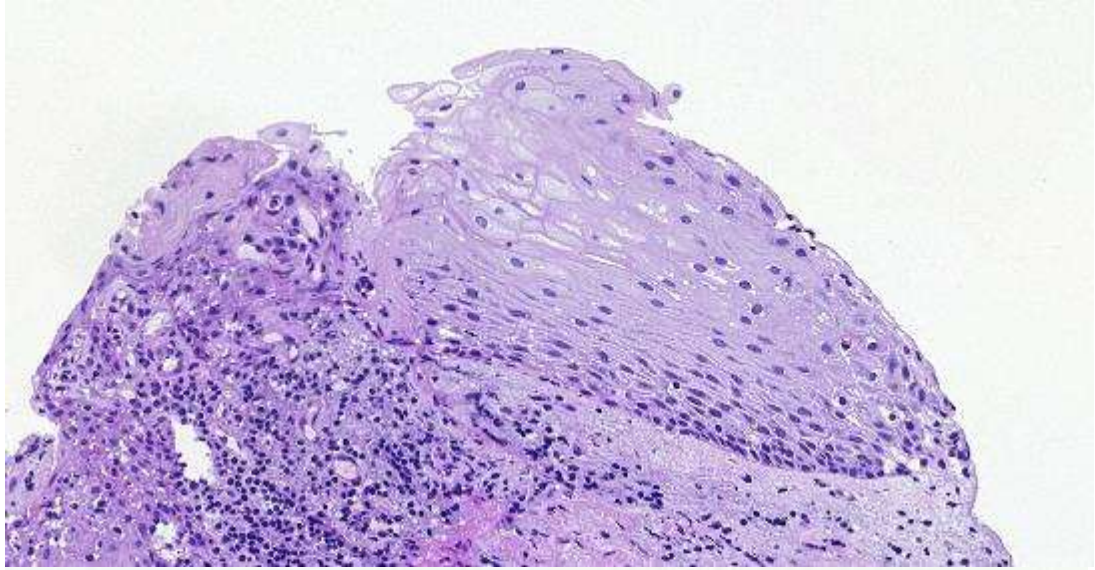

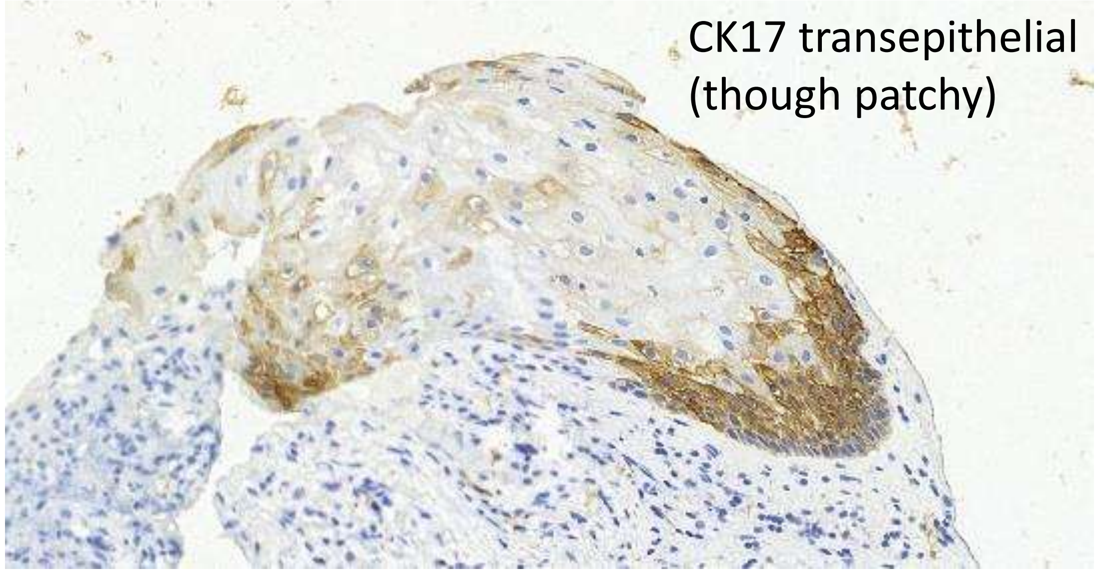

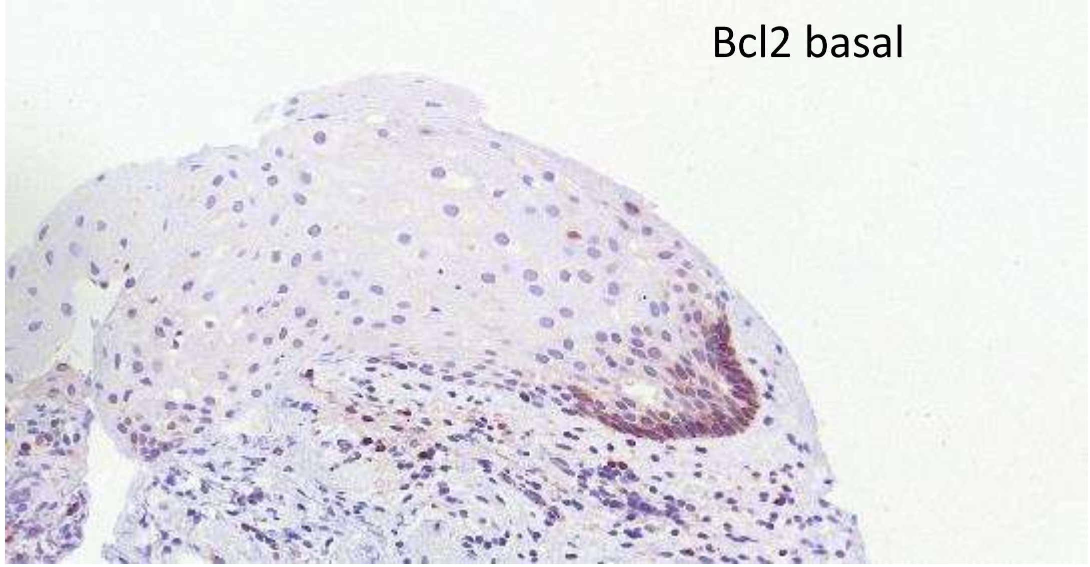

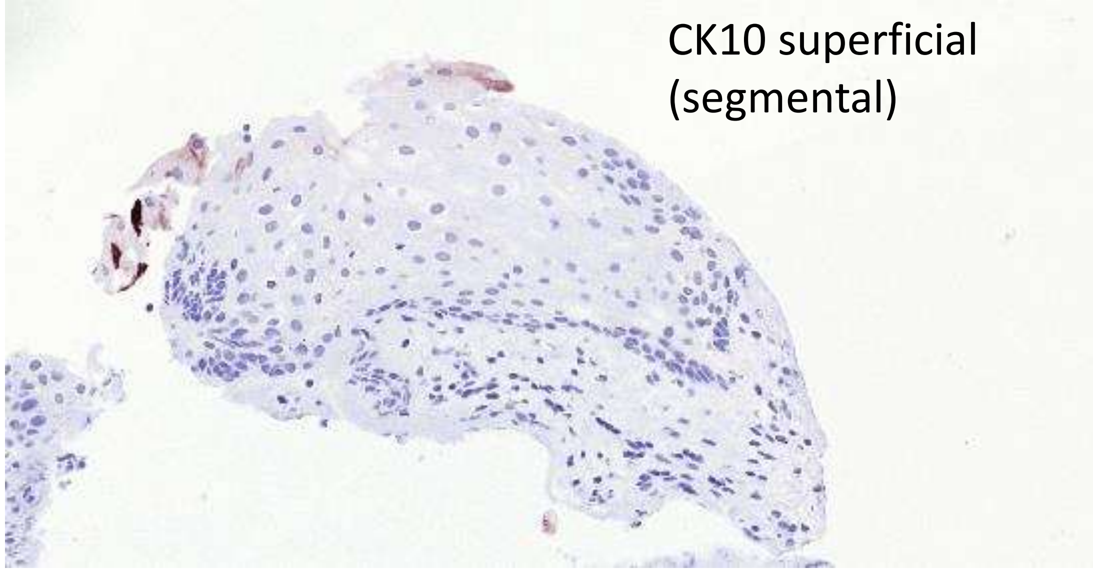

K2 – HE, CK17
bcl2, CK10 (x10)

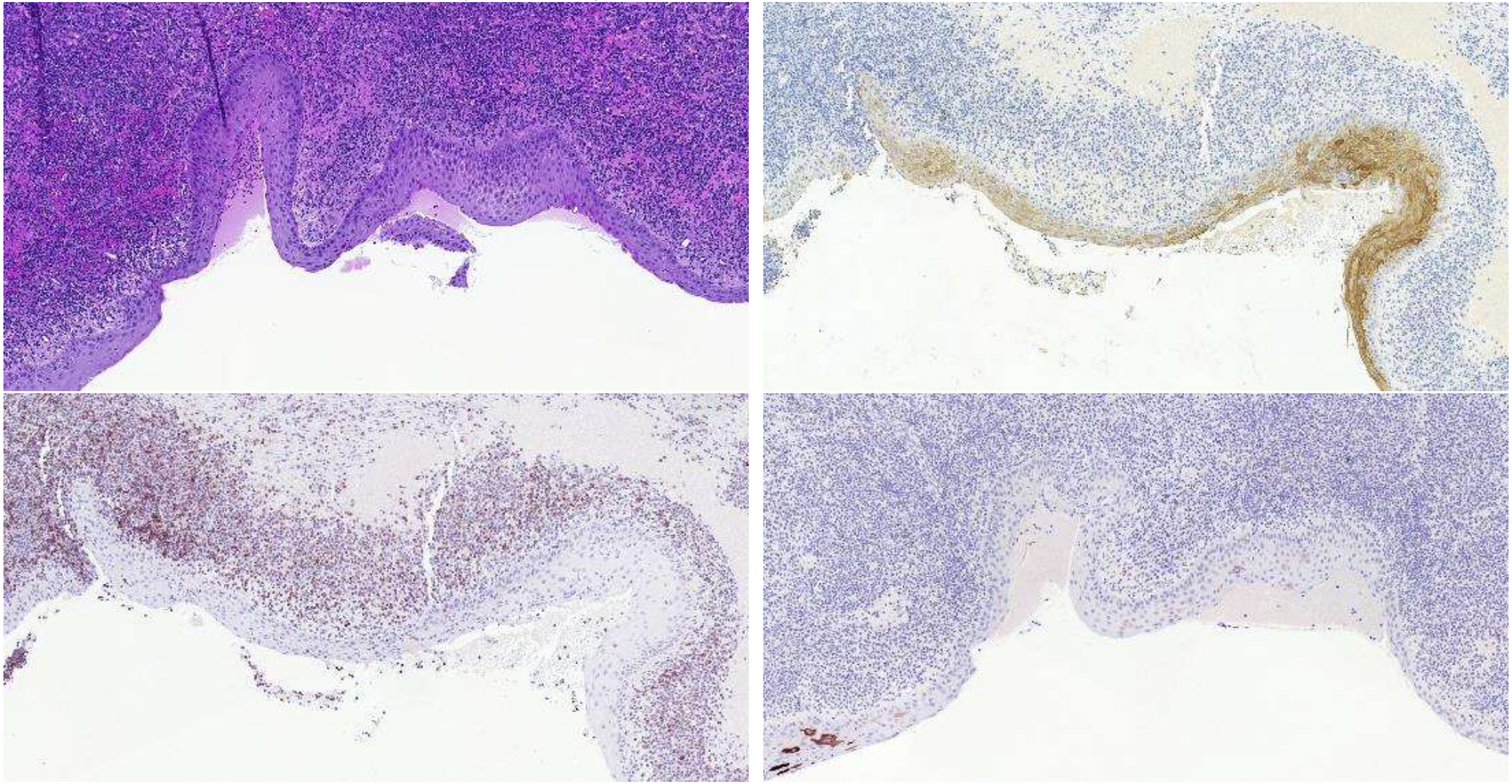

K2 - Surprise on revision / recut: HE, CK17, bcl2 (x35)

1.1-mm-long segment of parakeratotic epithelium with basal palisading and even epithelial stromal interface, some reversed polarity (nuclei located in the upper rather than the basal half of the basal cells; ↑). Transepithelial CK17 and basal/suprabasal bcl2 staining are all proofs of an OKC

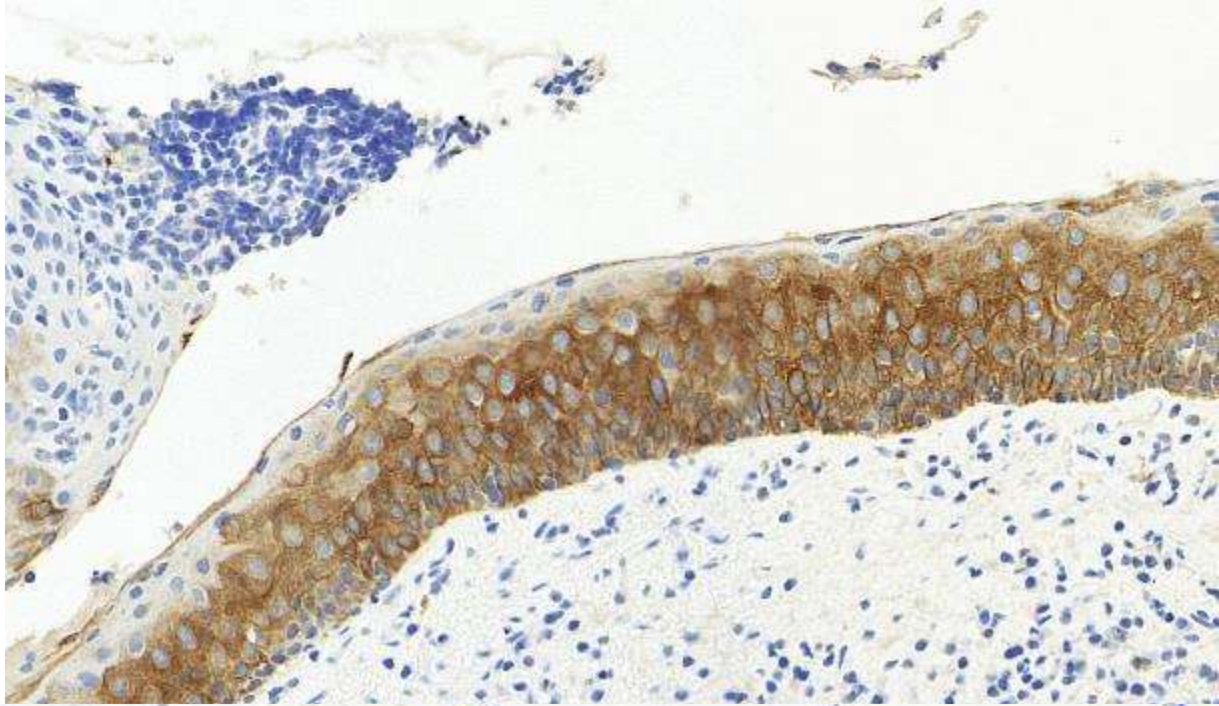

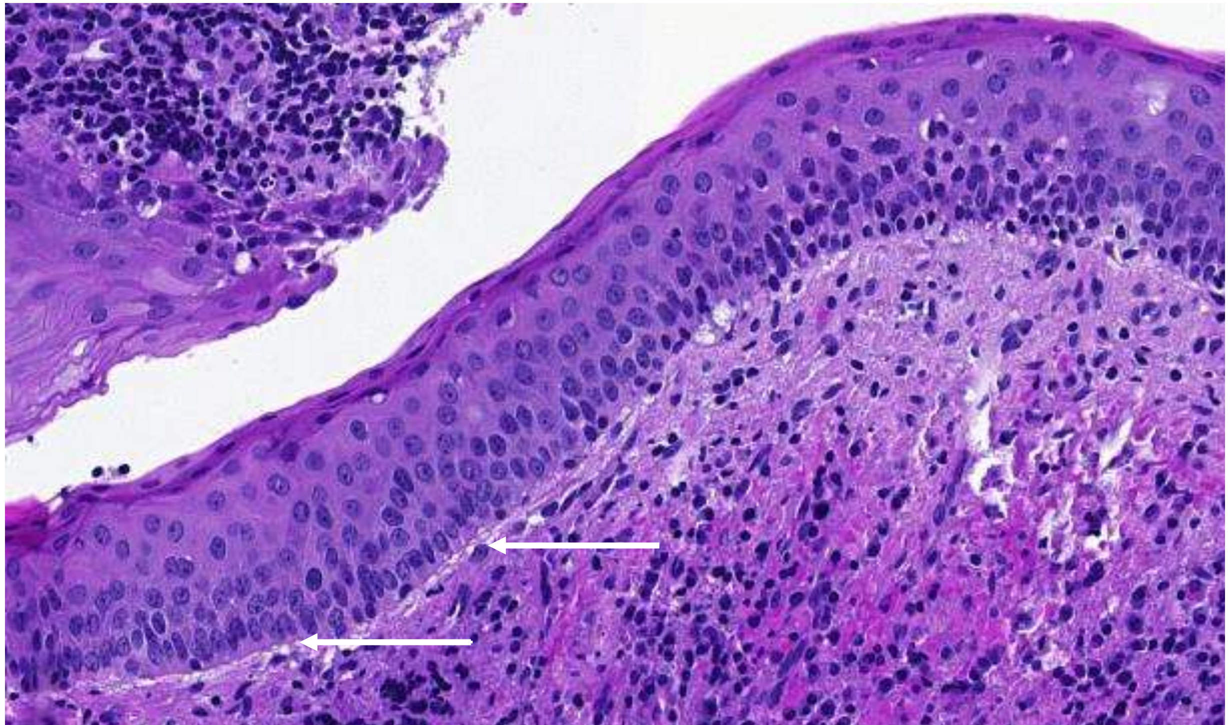

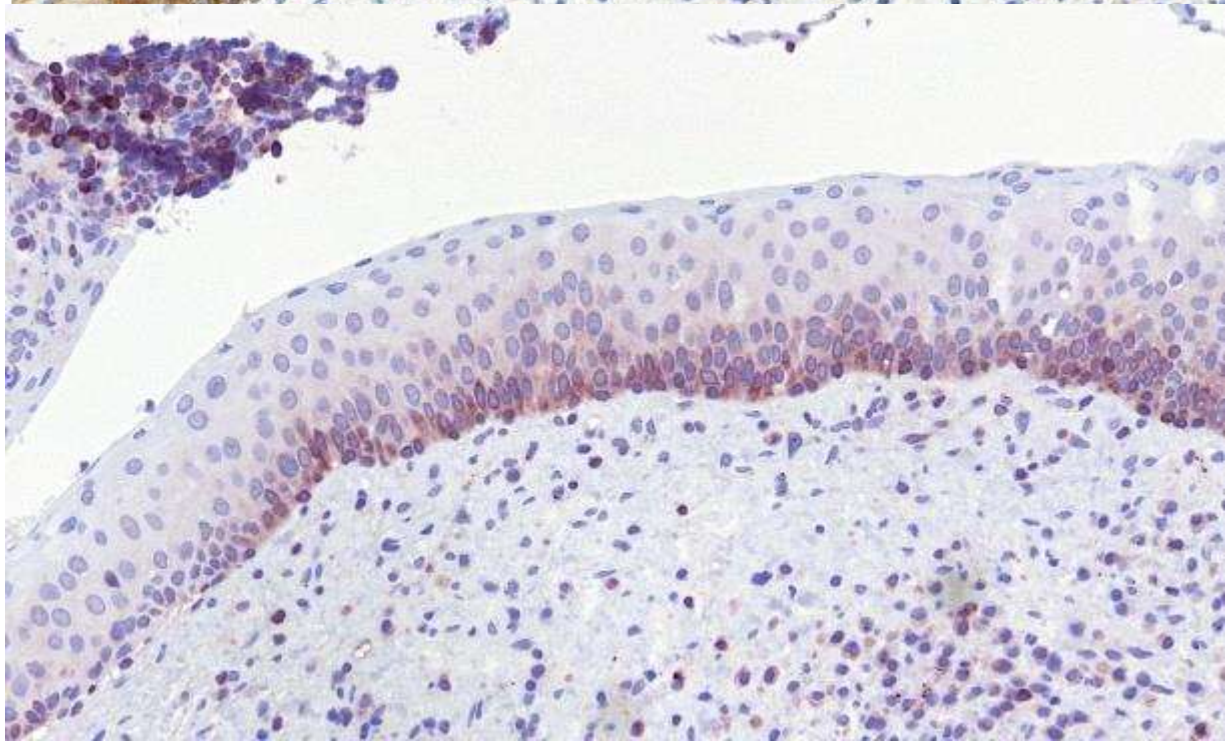

K2 – Conclusion

- H&E: relatively thick squamous epithelium, with parakeratosis, with wavy surface, only focal uncertain palisading highlighted by bcl2 IHC (see x25 magnification image), uneven epithelial-subepithelial contours
- Diffuse mixed inflammation
- Bcl2: negative, focally basal/suprabasal (see x25 magnification image)
- CK17: transepithelial (though patchy) positivity
- CK10: focal superficial positivity
- Histopathology: odontogenic cyst with some features of OKC; not fully diagnostic of OKC, but most likely to represent OKC with secondary inflammation - supportive IHC.
- Combined clinical and pathological opinion: inflamed OKC; patient followed as for OKC

Case K3

- 31-year-old male
- History: orthodontic interventions
- Dental hygiene/status: good; partly missing, partly treated teeth
- Radiology: 1 cm well circumscribed radiolucency distal to tooth partly impacted 48.
- Operation: removal of the cyst and tooth 48.
- Clinical diagnosis: follicular (dentigerous) cyst; keratocyst?

K3 – OPT

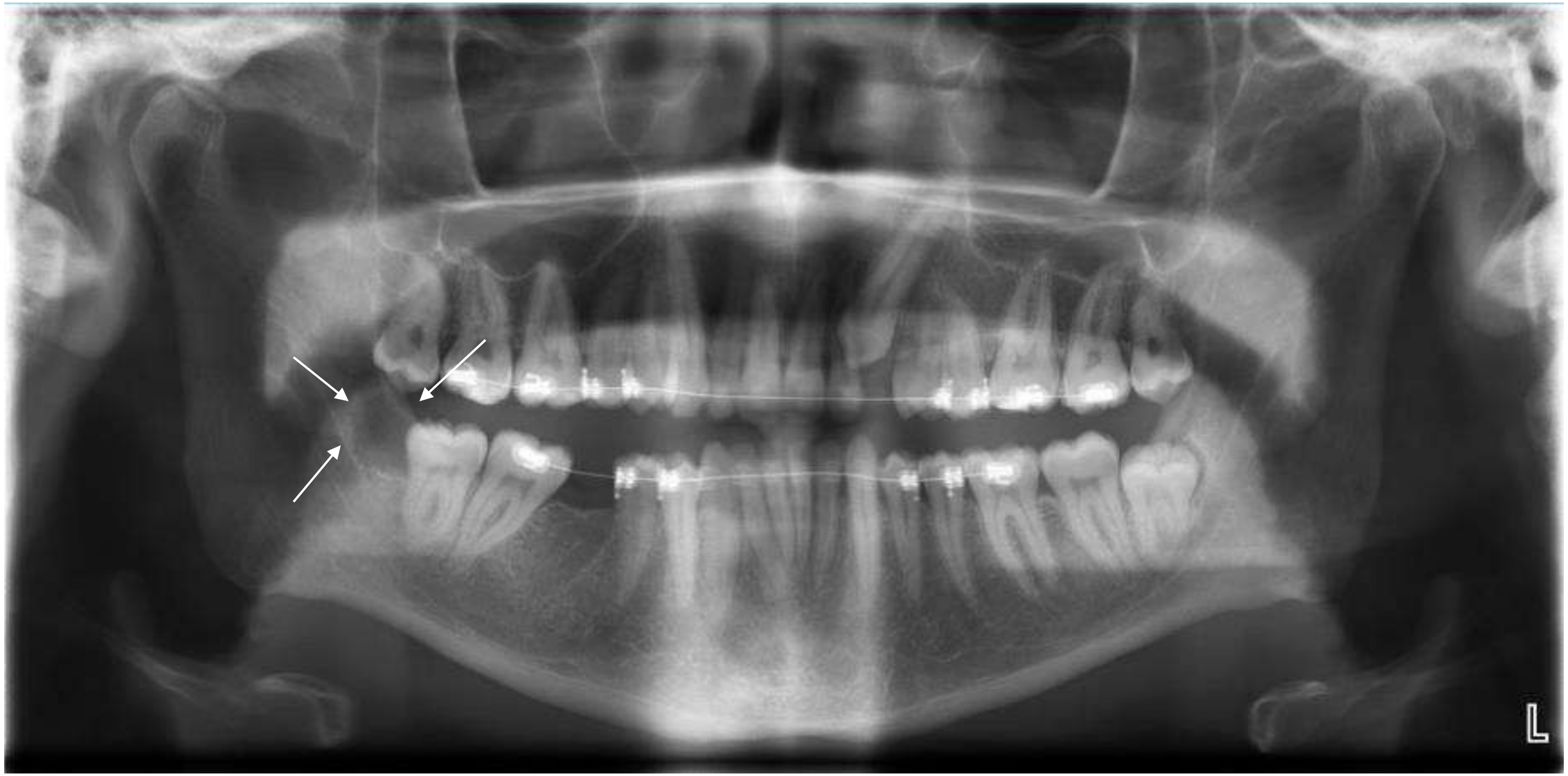

K3 – HE, CK17 (x5)
bcl2 (x5) (x18) – no basal staining

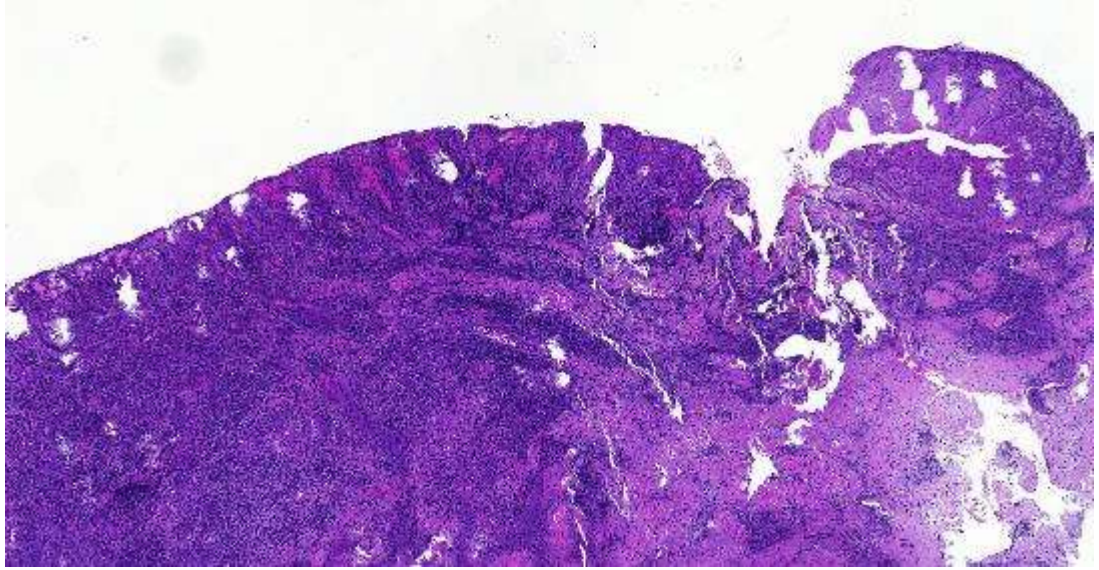

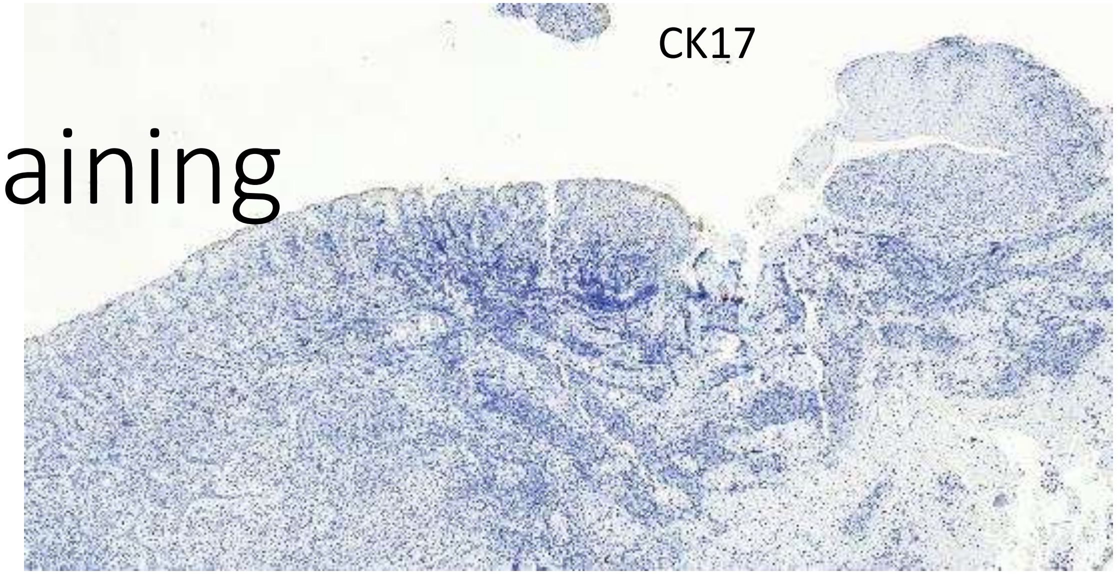

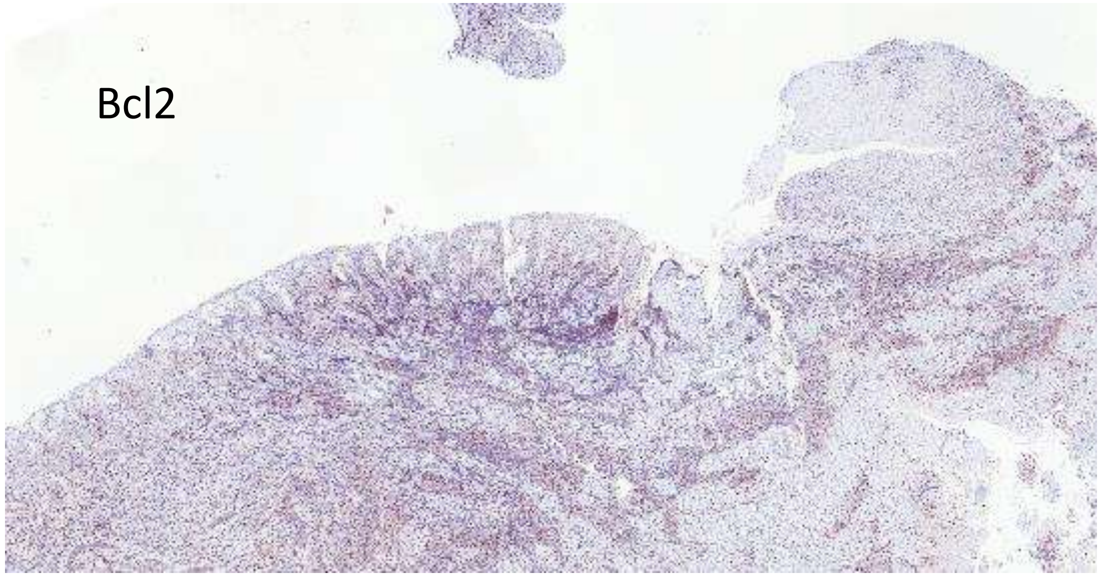

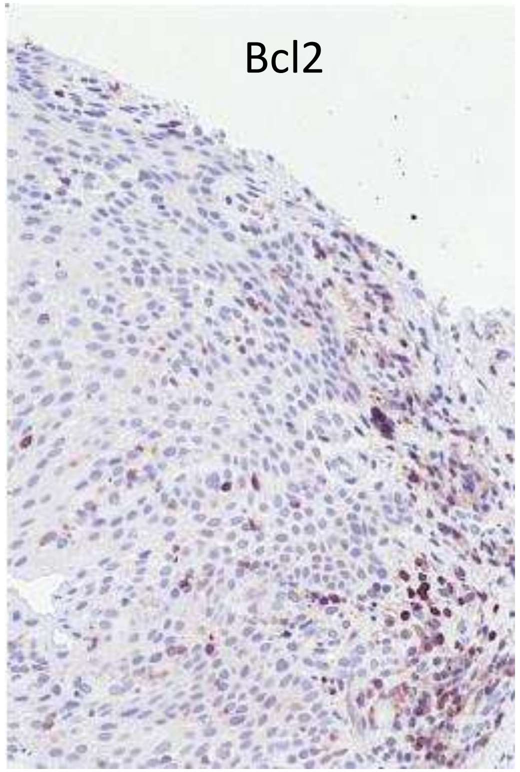

CK17 (x20) negative area

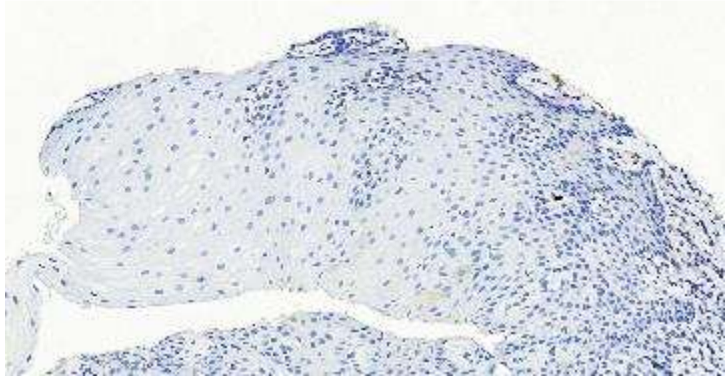

K3 – HE, CK17, bcl2 (x20)

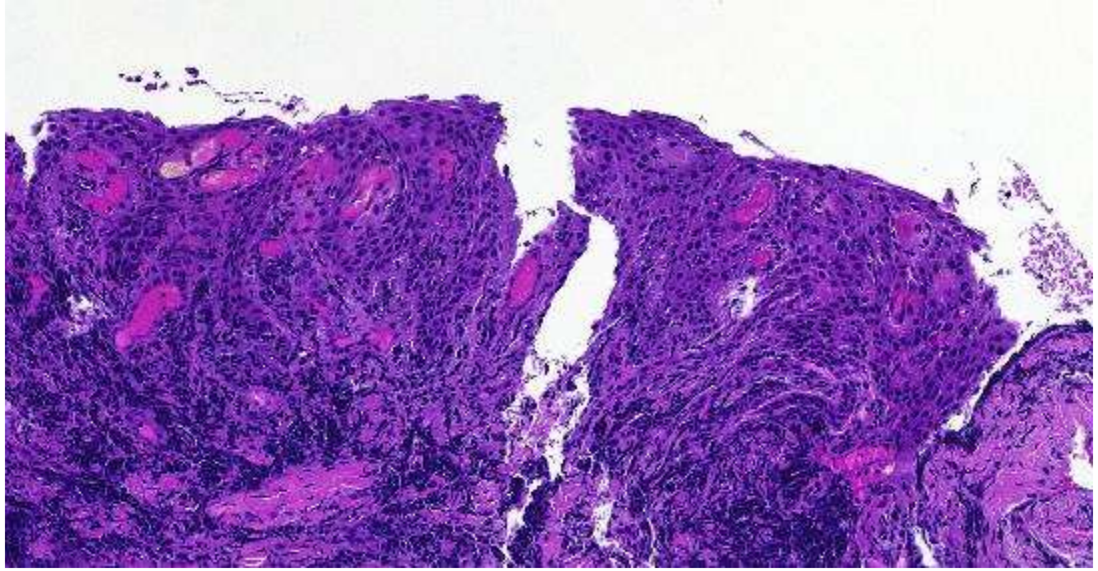

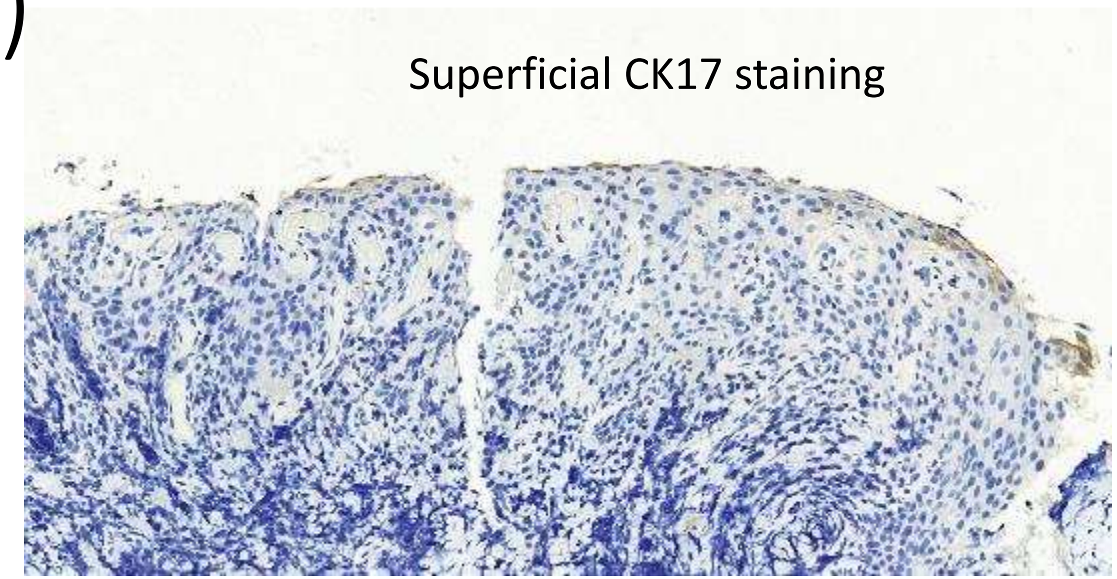

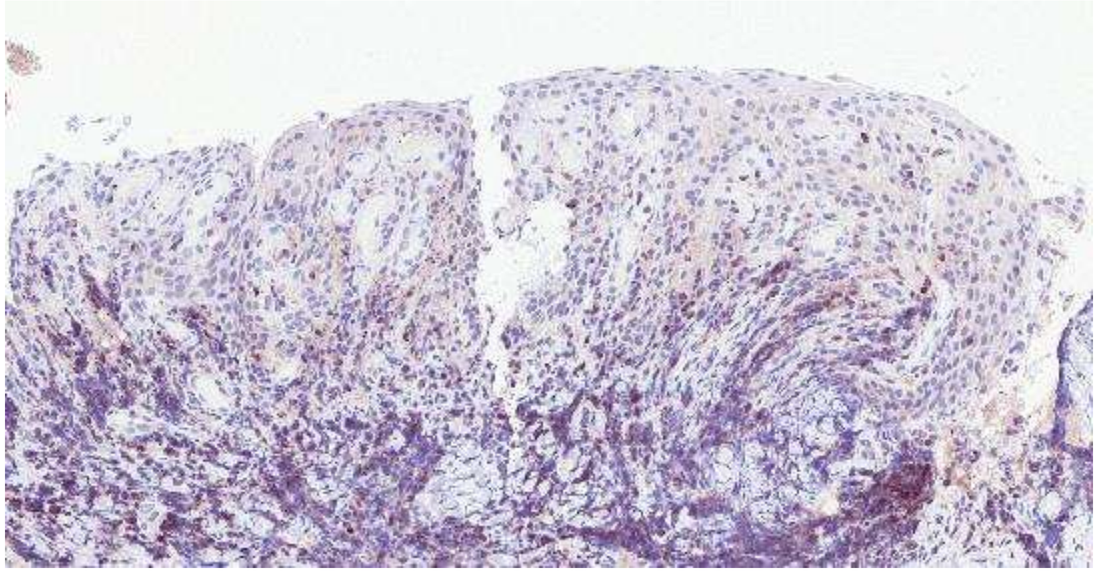

K3 – Conclusion

- H&E: relatively thick squamous epithelium, without parakeratosis, no wavy surface, no basal palisading, uneven epithelial-subepithelial contours
- Diffuse mixed inflammatory infiltrate of the cyst wall
- Bcl2: negative
- CK17: superficial cell layer staining / negative
- Histopathology: inflammatory odontogenic cyst N.O.S. with no features of OKC
- Combined clinical and pathological opinion: dentigerous cyst with secondary inflammation

Case K4

- 41-year-old male
- Dental hygiene/status: moderately neglected; partly missing, fillings in teeth 11, 12, 13, 14 and 16 (performed after the discovery of the lesion); periodontitis
- Left sided swelling of the face, intraoral incision followed by serous fluid flow
- CT: thin walled 46x20x45 mm cystic lesion with fluid density, cortical bone destruction and mucosal thickening in the left maxillary sinus. Unicystic radiolucent lesion between teeth 11 and 16 on panorama.
- Operation: cystectomy (trough lateral maxillary window) + apicectomy of teeth 11 - 14 and extraction of tooth 16
- Clinical diagnosis: maxillary cyst

K4 – OPT

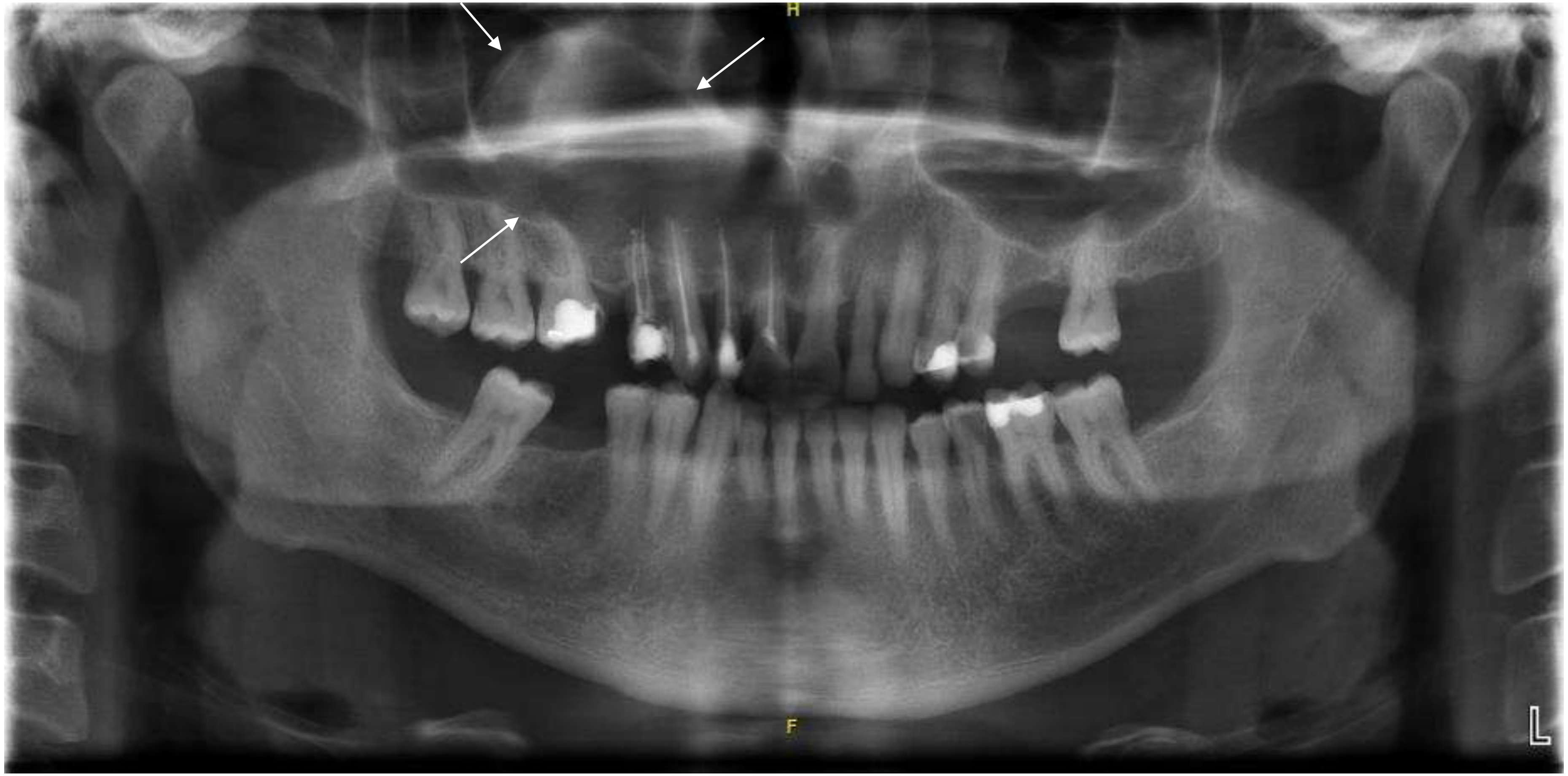

K4 – CT Coronal (Slices 15-20-23/47)

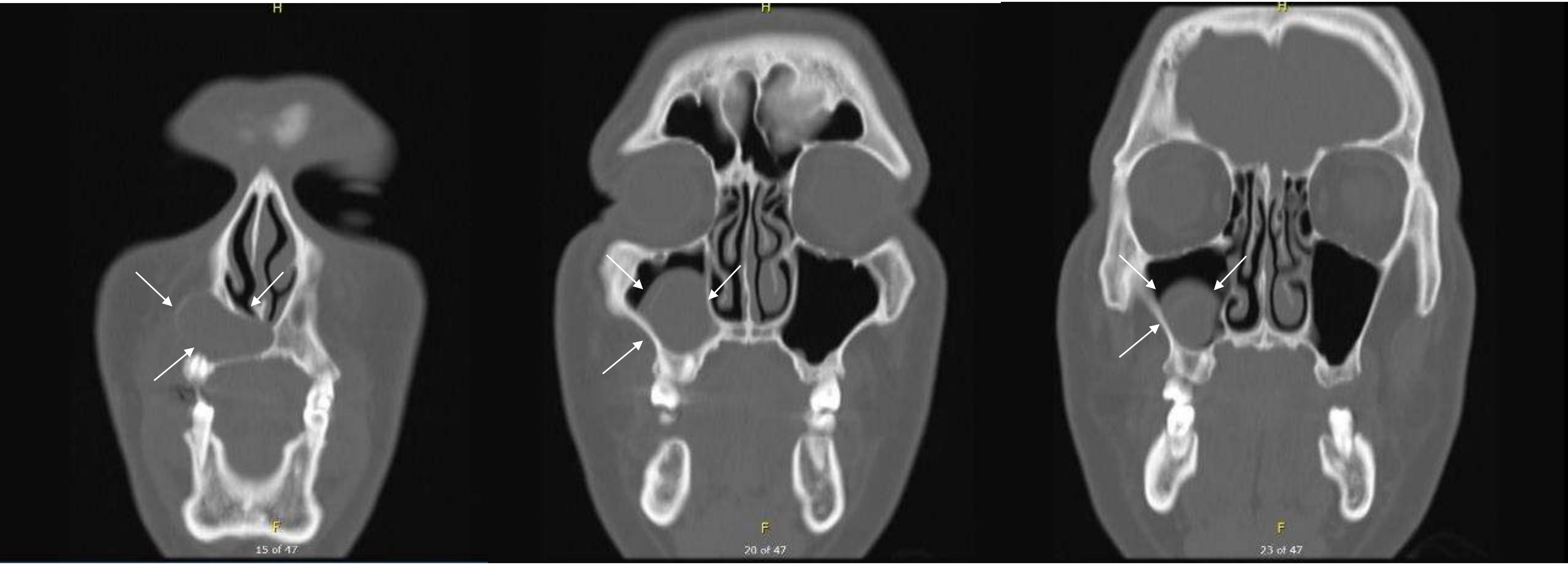

K4 – CT sagittal and axial

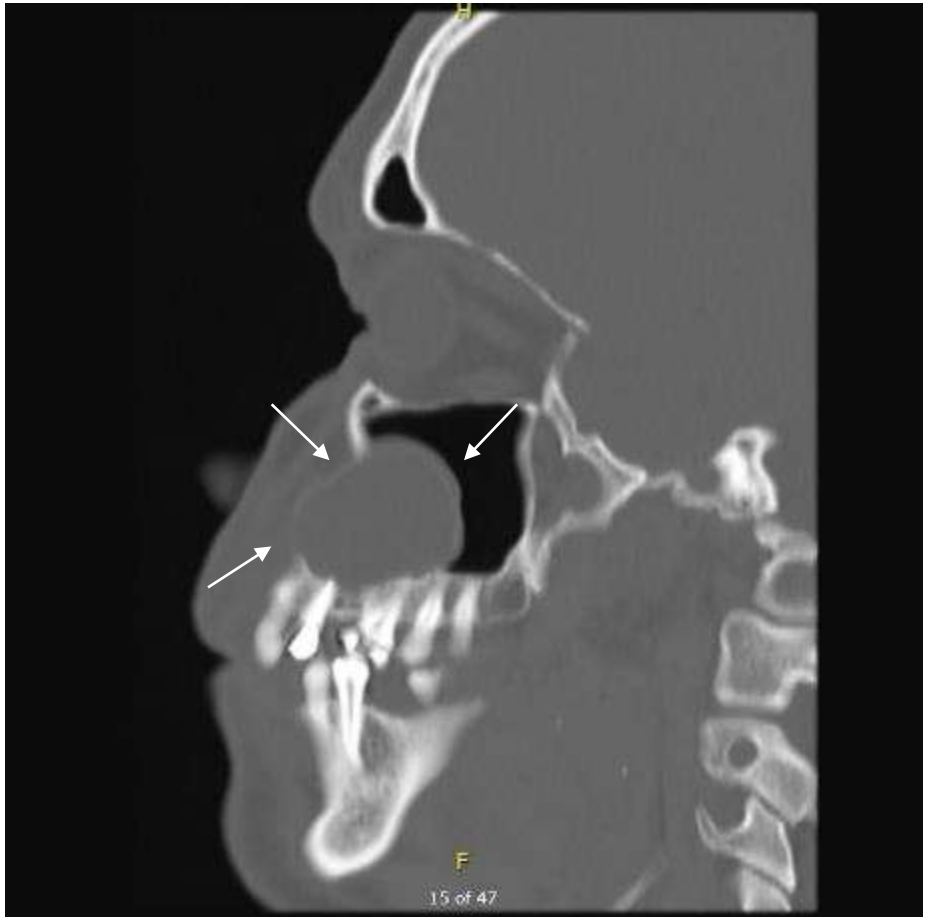

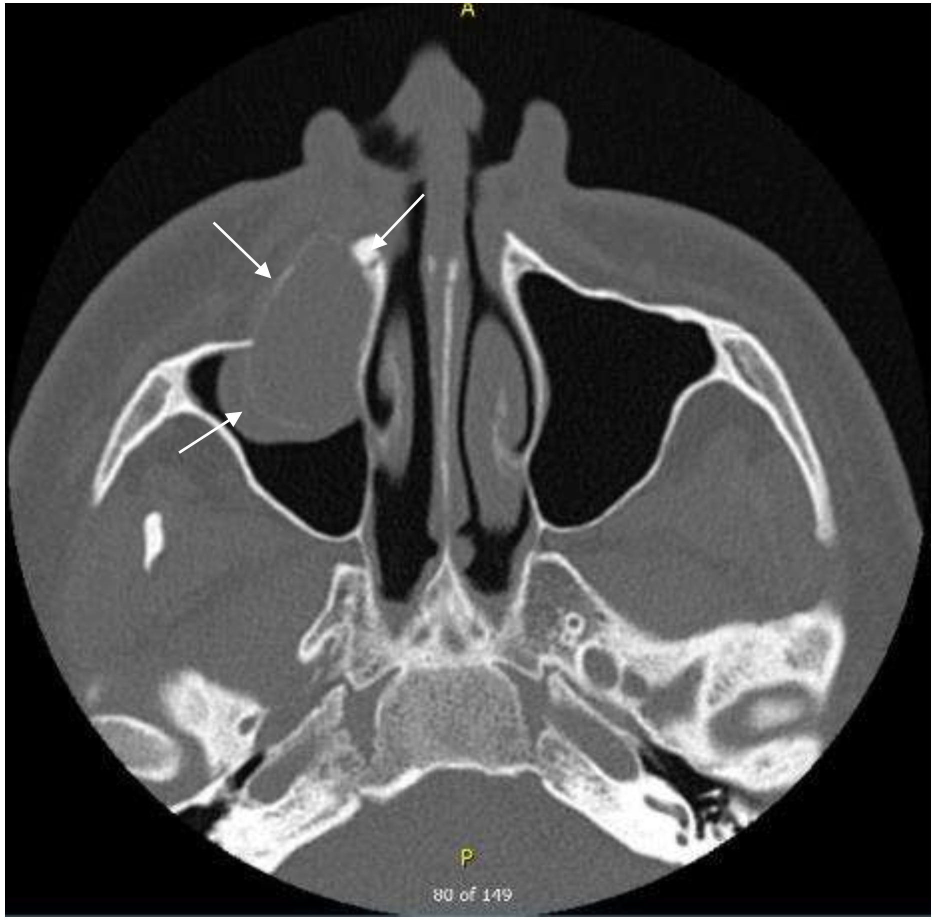

K4 – HE, CK17, bcl2 (x0.9)

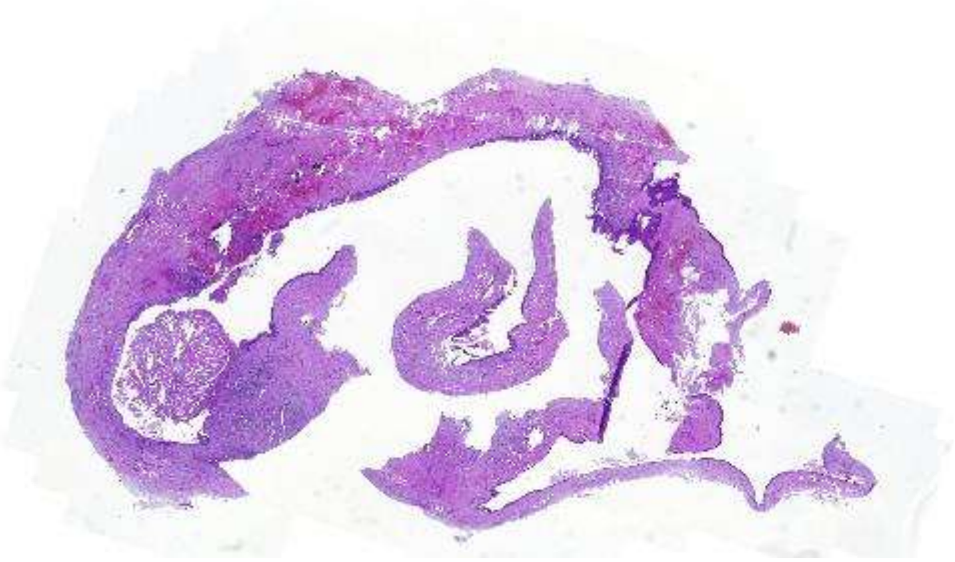

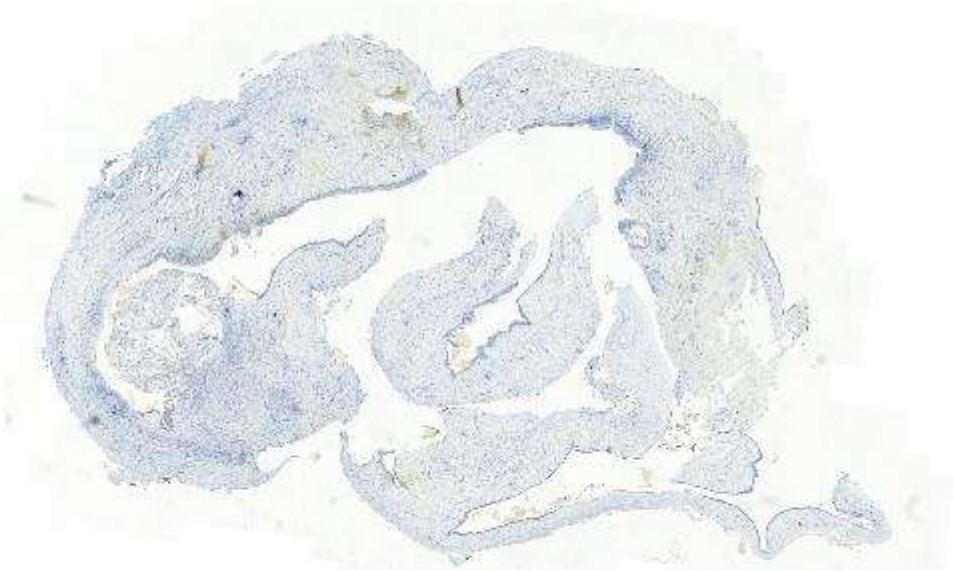

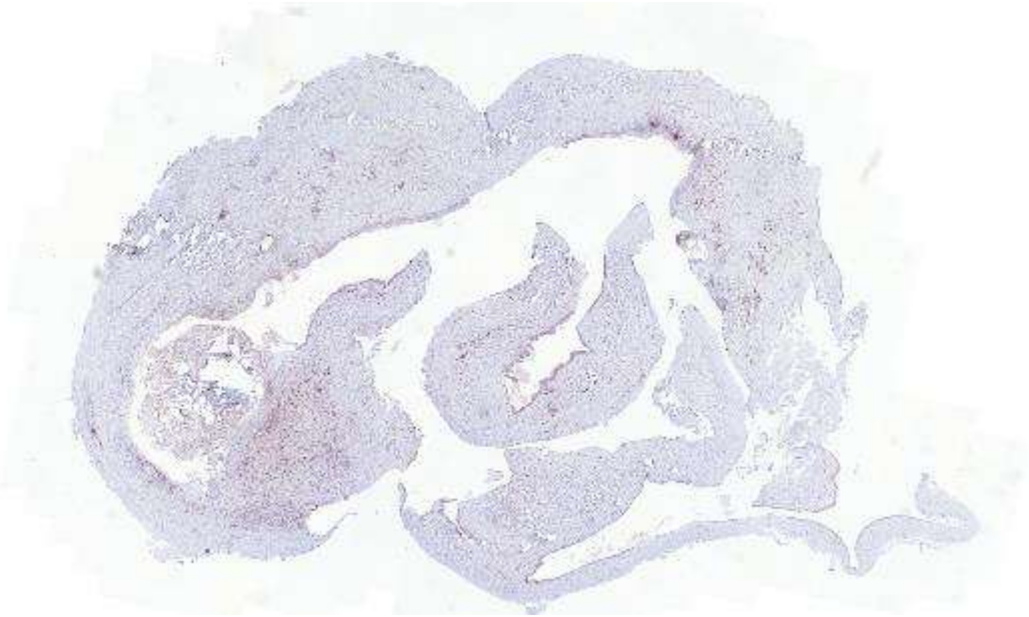

K4 – HE, CK17, bcl2 (x25)

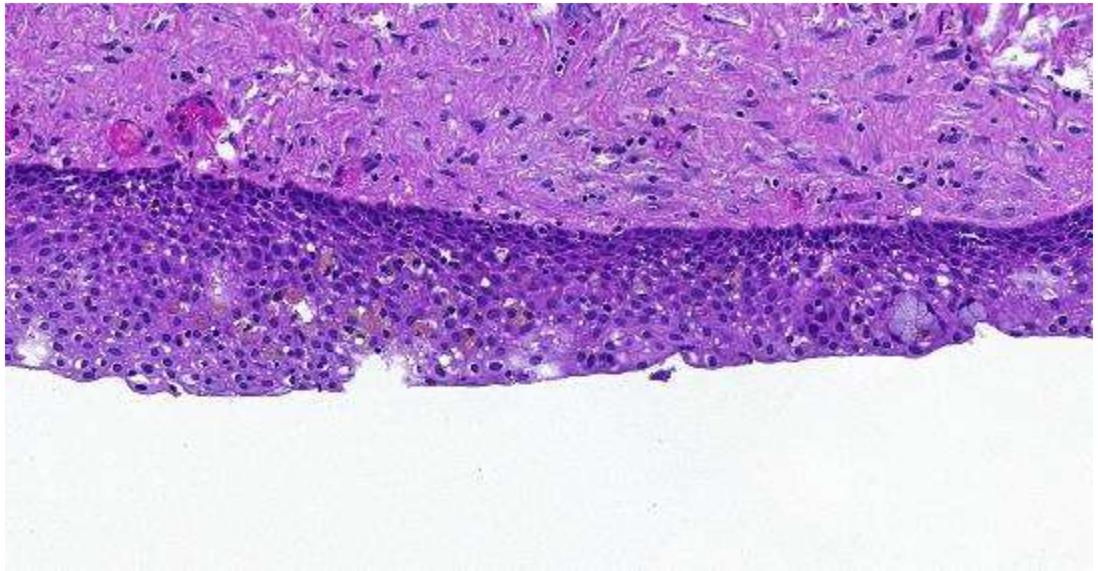

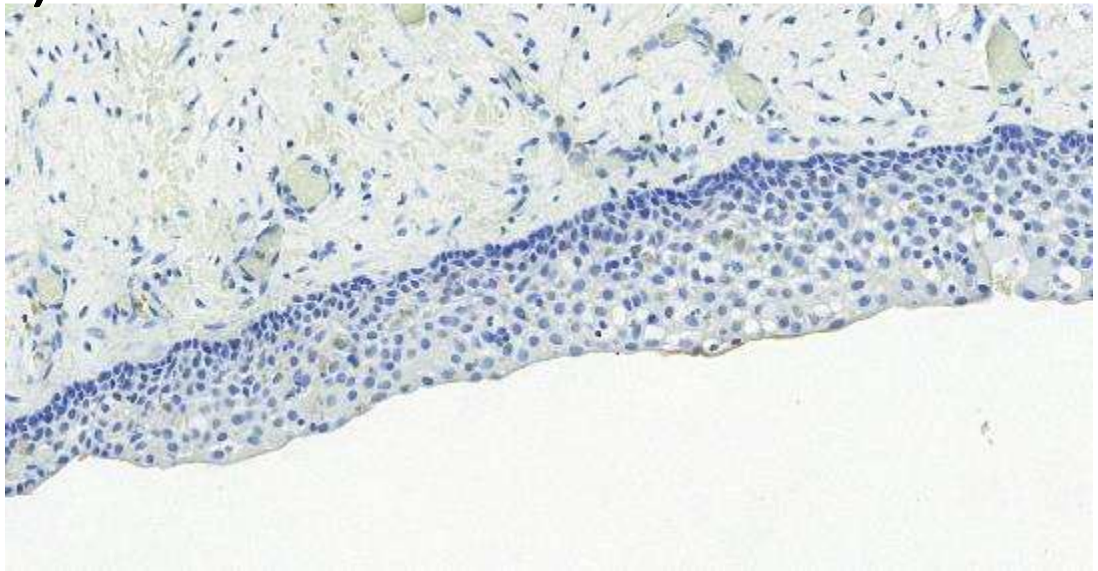

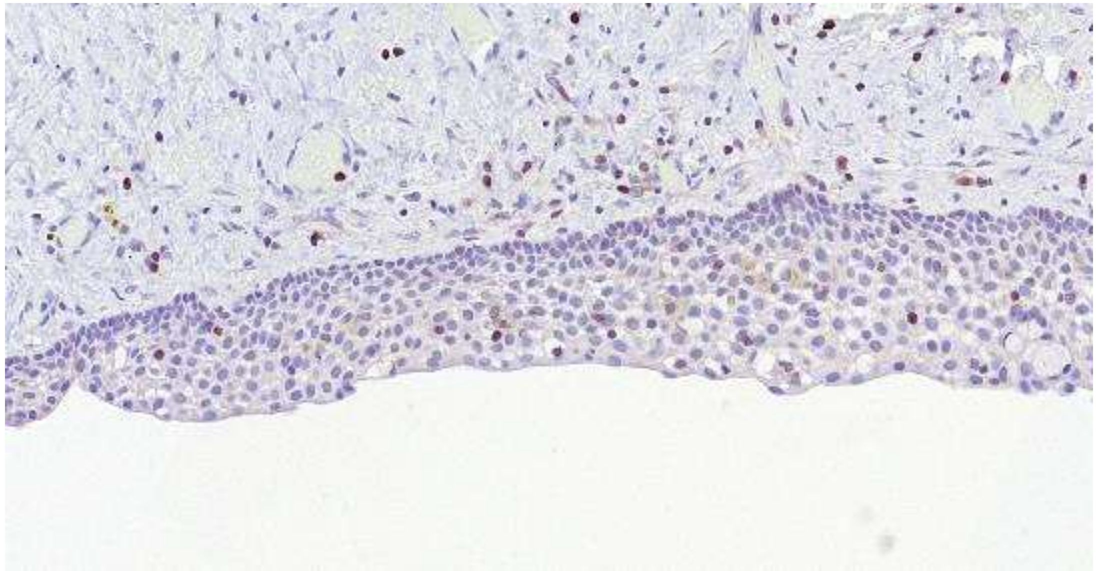

K4 – HE, CK17, bcl2 (x10) – mucous metaplasia

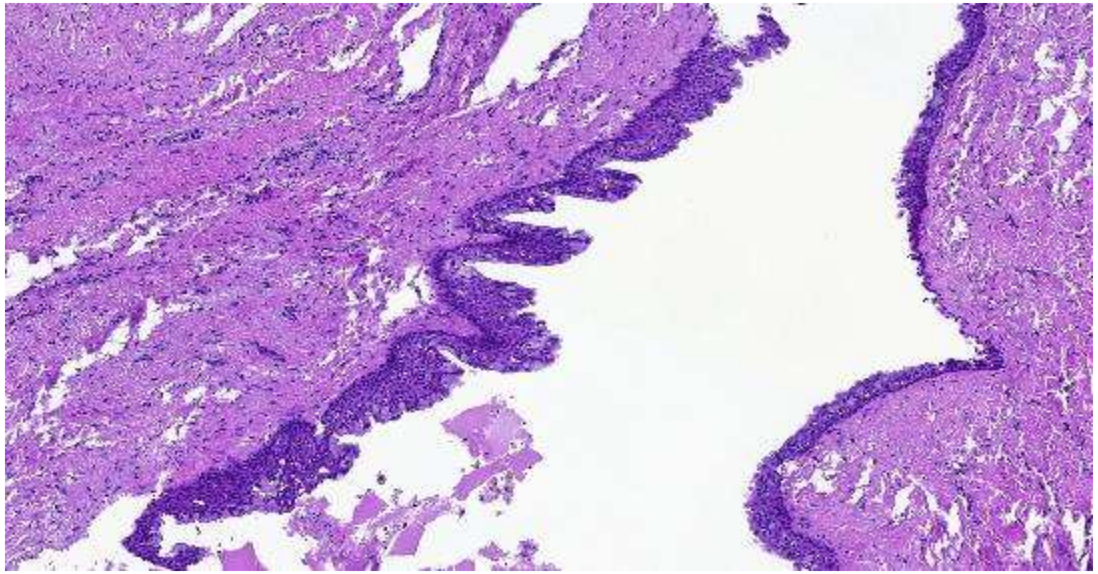

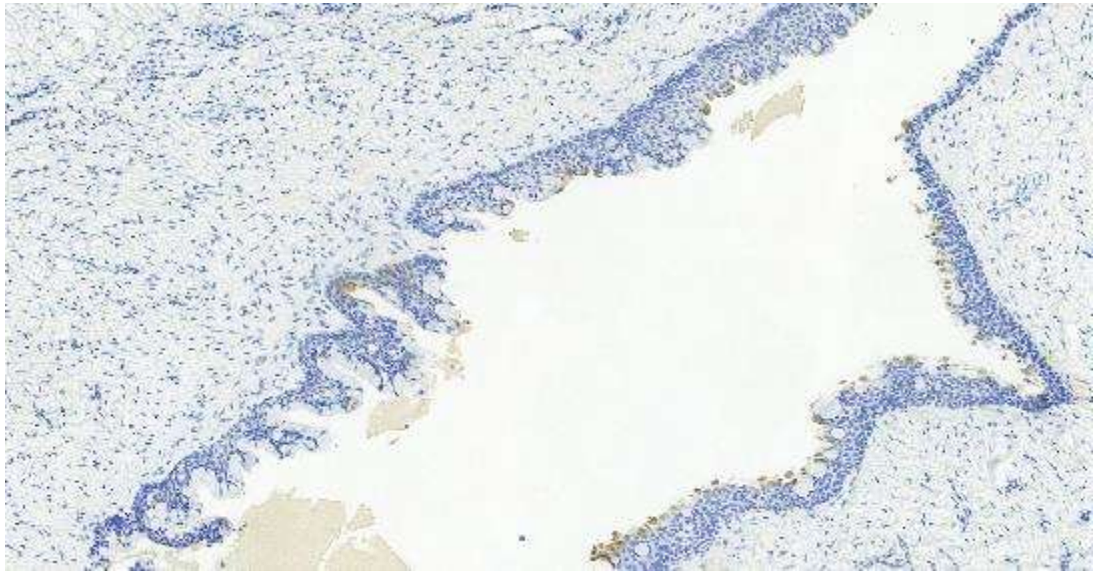

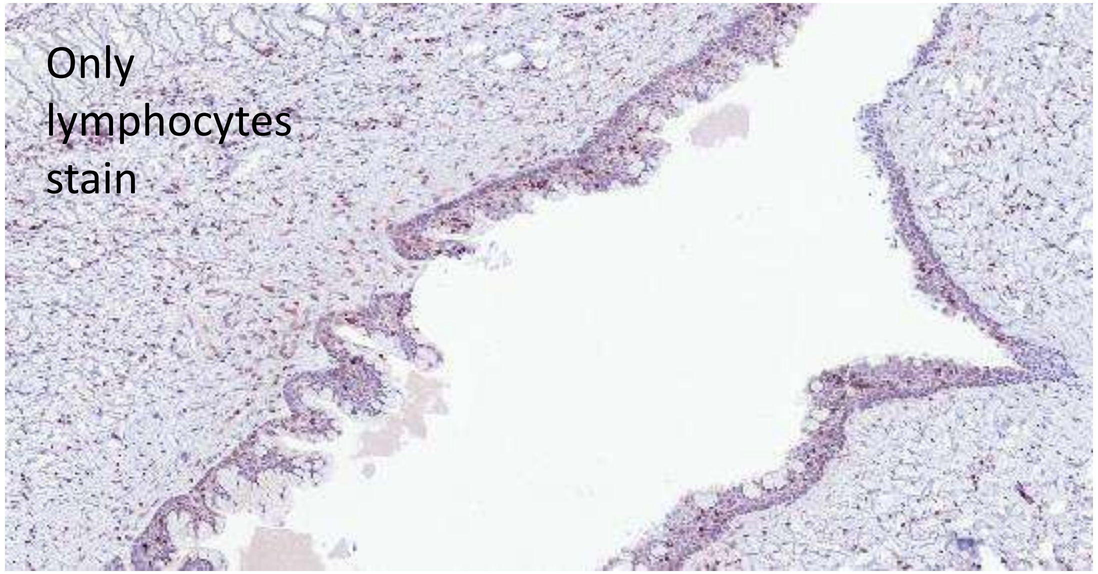

K4 – Conclusion

- H&E: relatively thick squamous epithelium, without parakeratosis, no wavy surface, no basal palisading, uneven (minimal, focally even) epithelial-subepithelial contours; mucous metaplasia
- Focal chronic inflammation, with protruding cholesterol granuloma; larger areas without inflammation
- Bcl2: negative
- CK17: negative, focal superficial staining, scanty spinous cells also stain
- Histopathology: unspecified probably odontogenic cyst
- Combined clinical and pathological opinion: consistent with mature residual cyst

Case K5

- 61-year-old female
- Dental hygiene: no data
- Retrospective anamnesis: resection of dental root and cystectomy about 10 years ago; recurrent cyst
- Operation: removal of tooth 22 and cystectomy (unicystic lesion without daughter cysts, „content not reminiscent of keratocyst”)
- Clinical diagnosis: „maxillary cyst, periapical”

K5 – CT

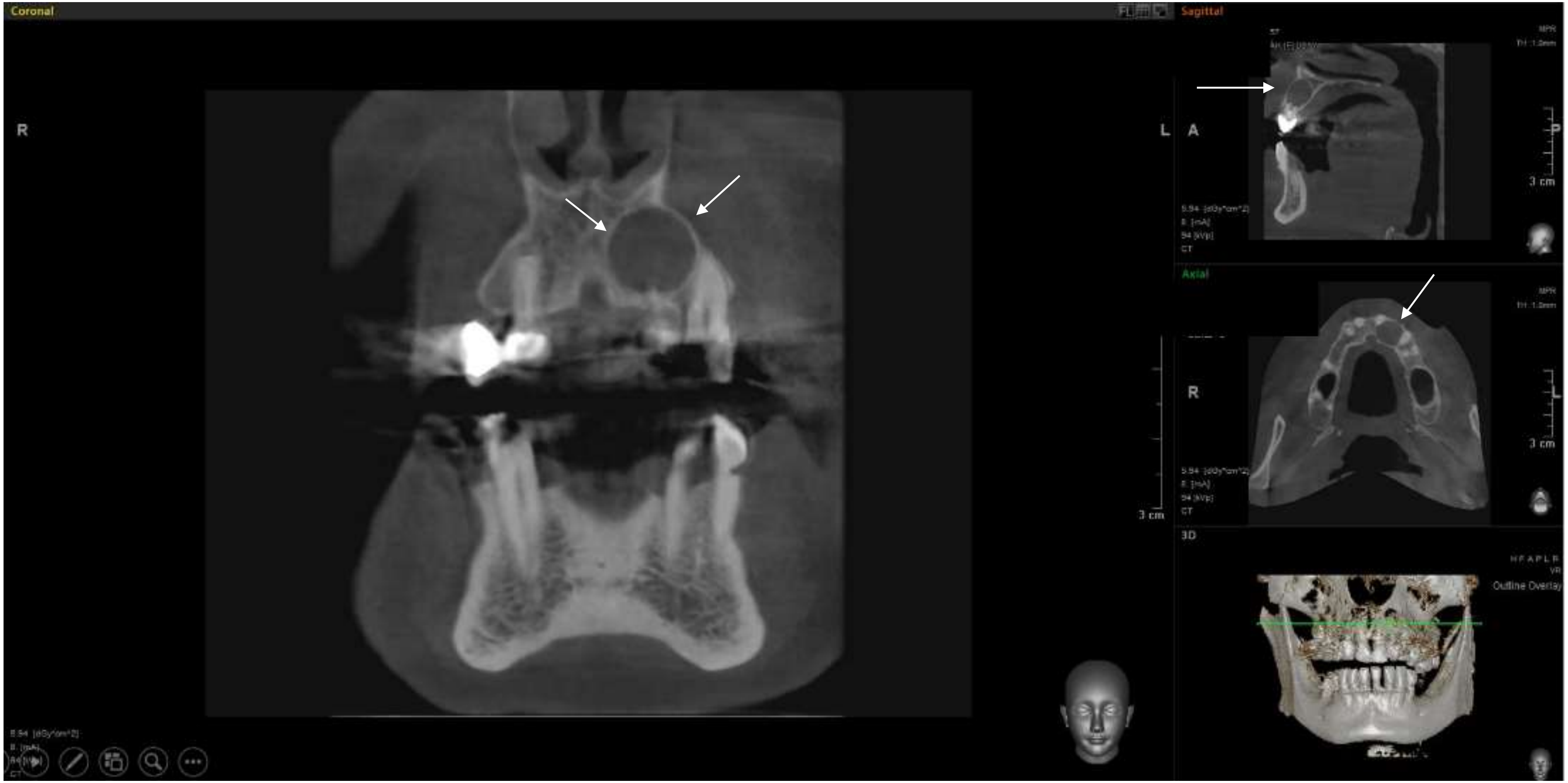

Coronal
R
L
3 cm
Sagittal
A
P
3 cm
Axial
R
L
3 cm
3D
Outline Overlay

K5 – HE, CK17, bcl2 (x0.8)

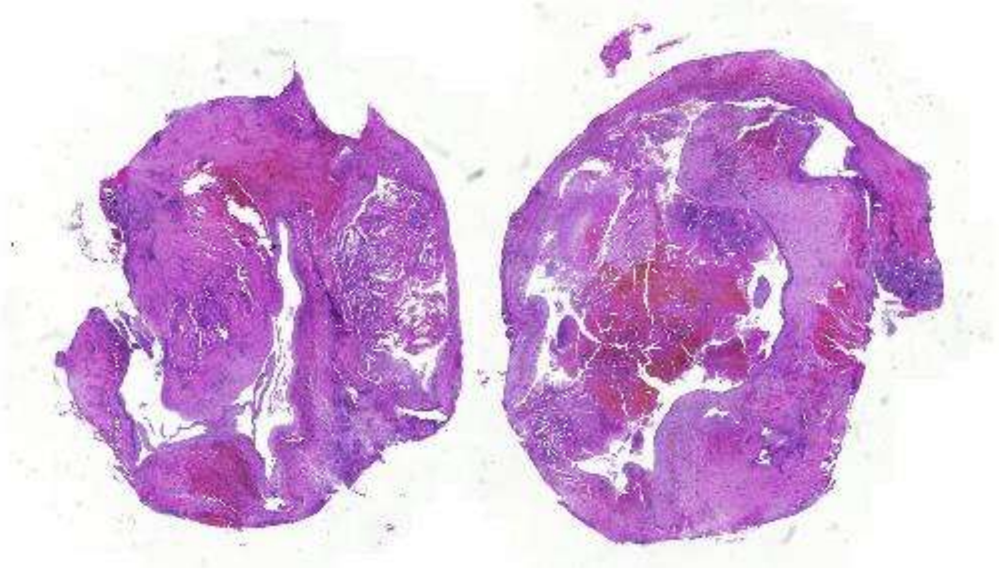

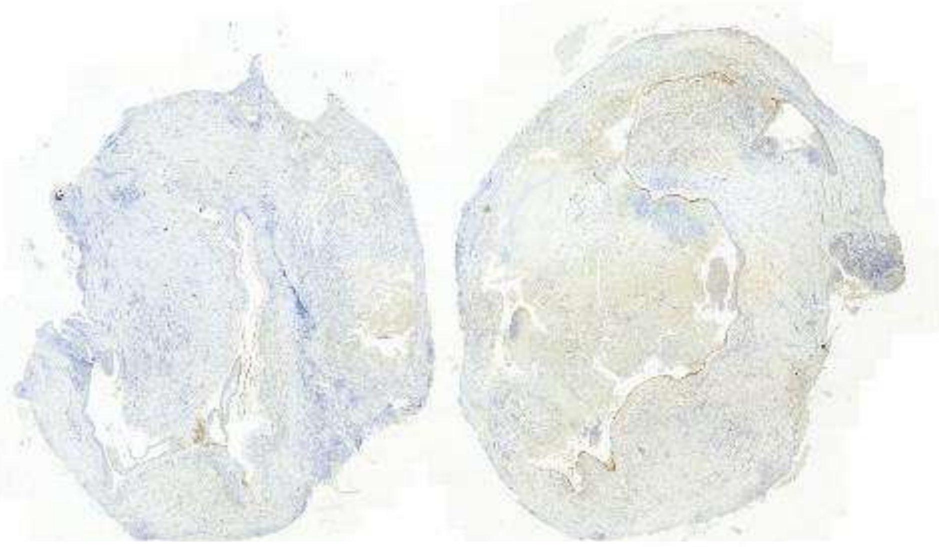

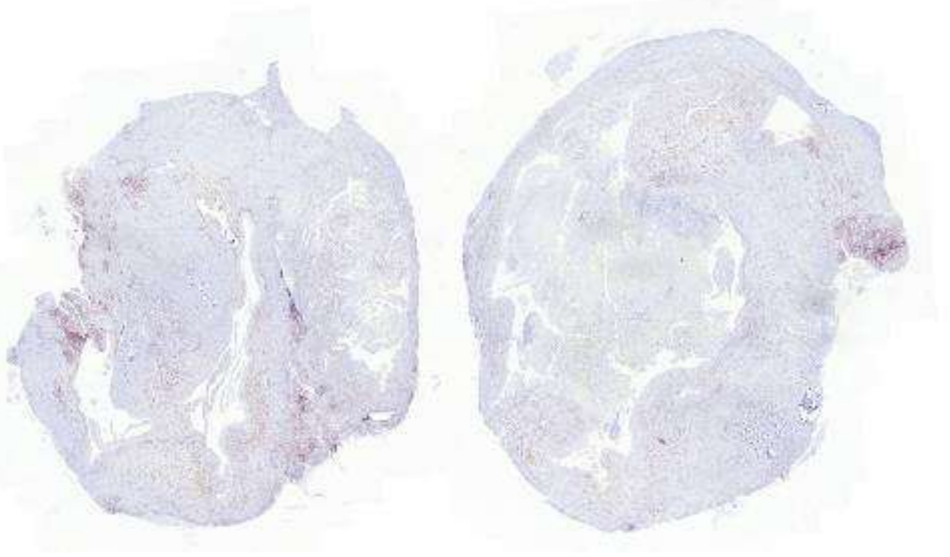

K5 – CK17 (x5) various staining patterns

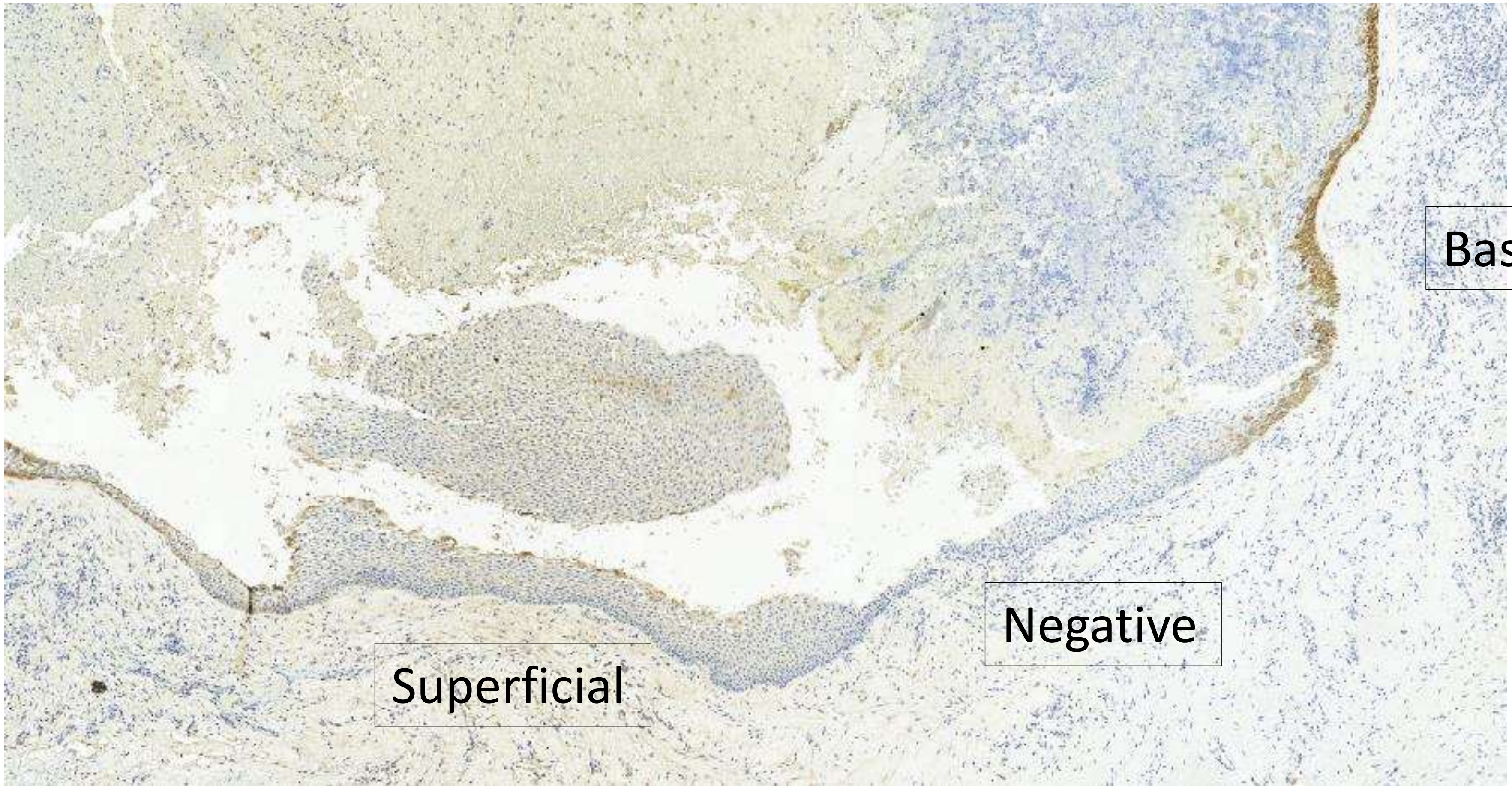

K5 – Corresponding HE & bcl2 (x5)

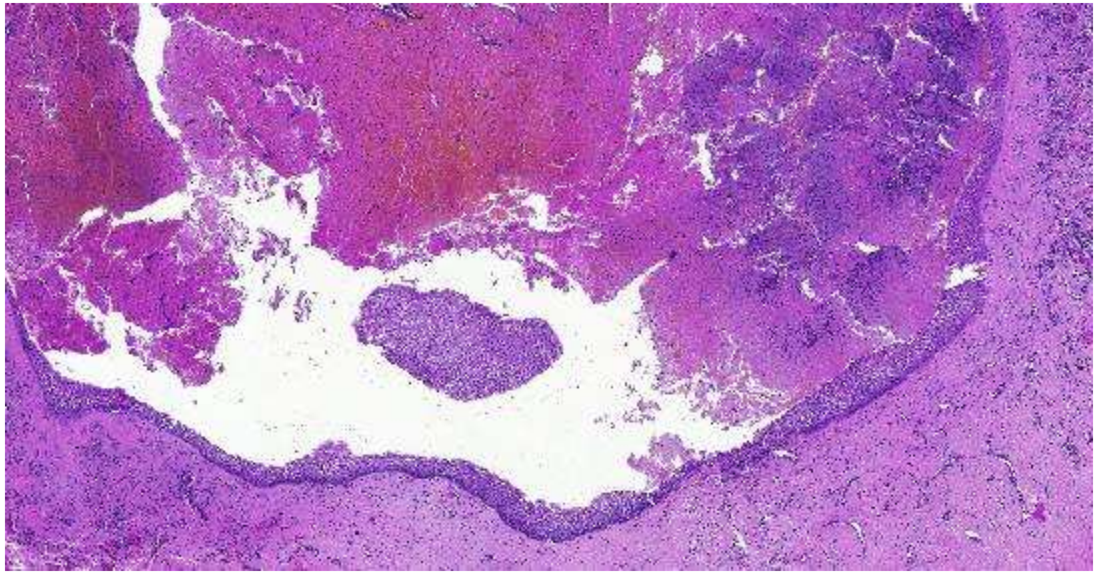

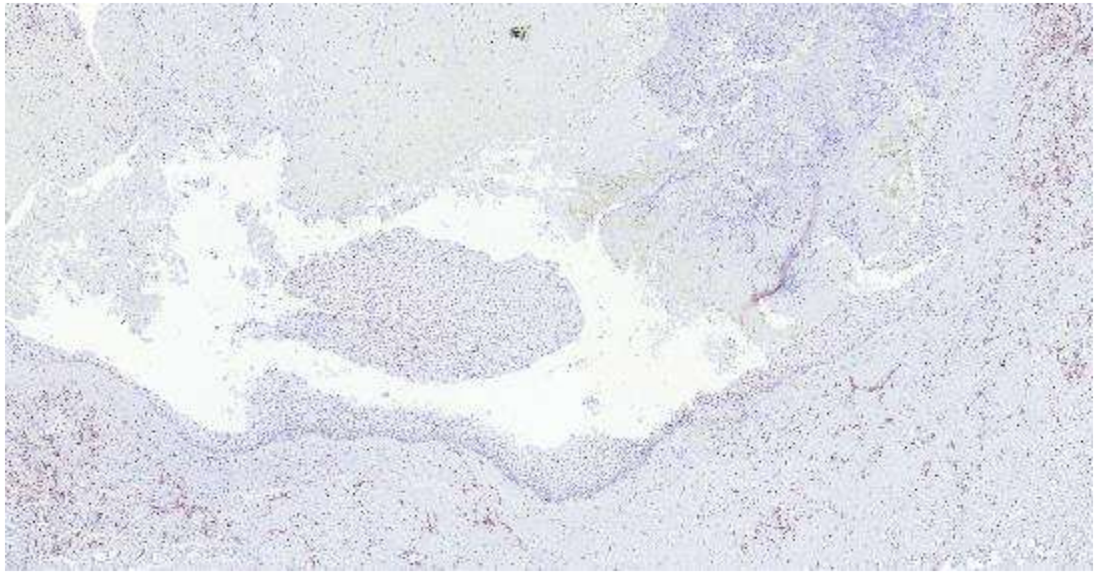

K5 – HE, CK17, bcl2 (x20) basal CK17+ & bcl2- area

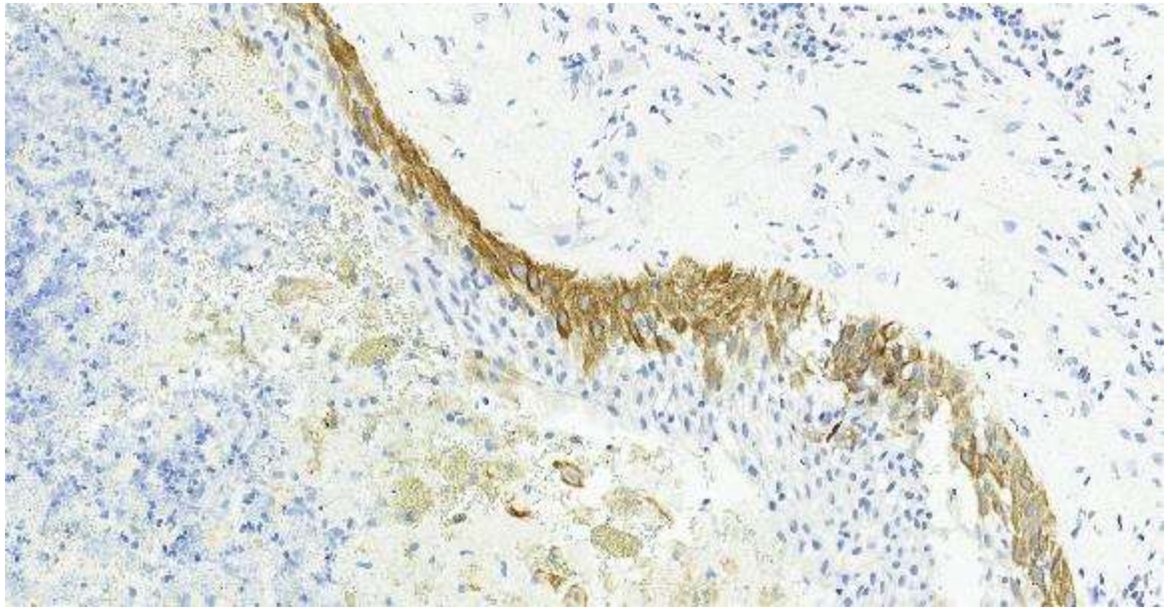

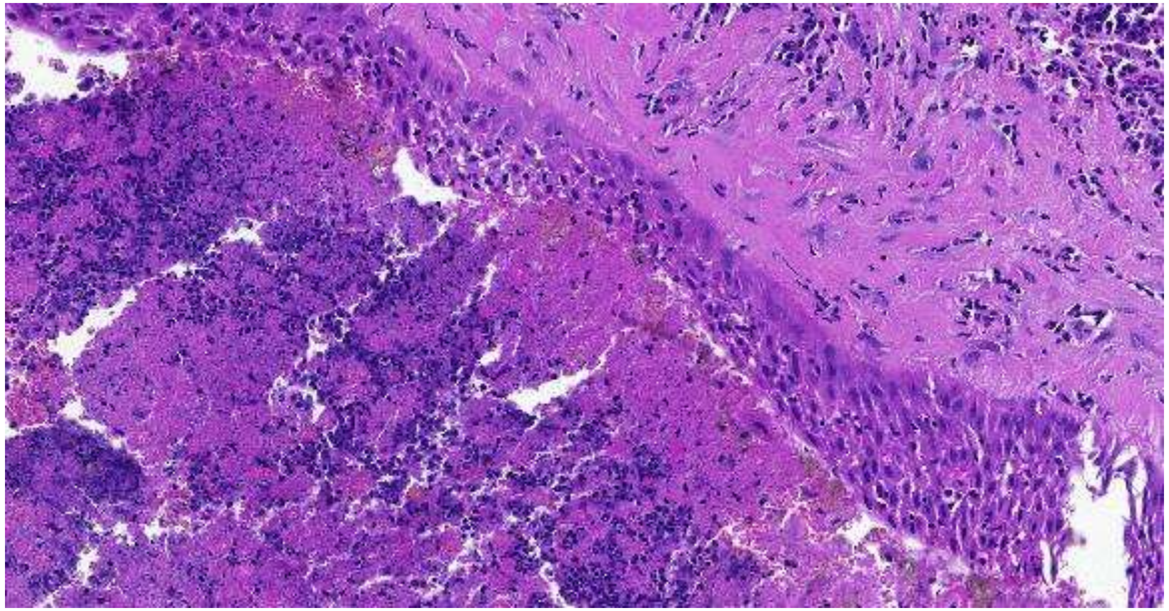

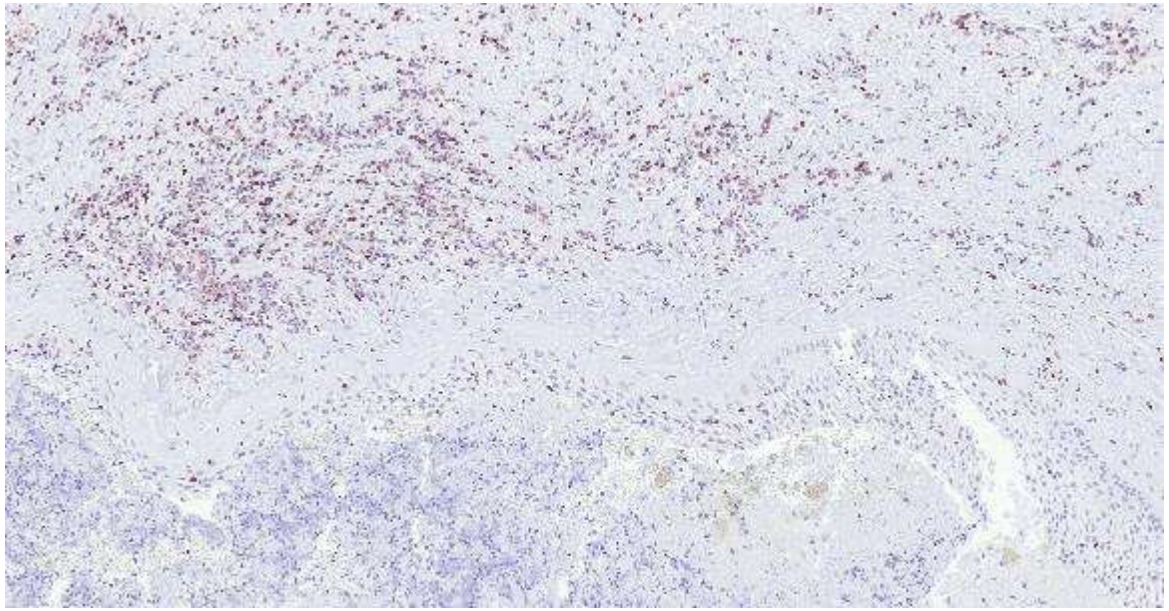

K5 – HE, CK17, bcl2 (x10) transepithelial CK17+

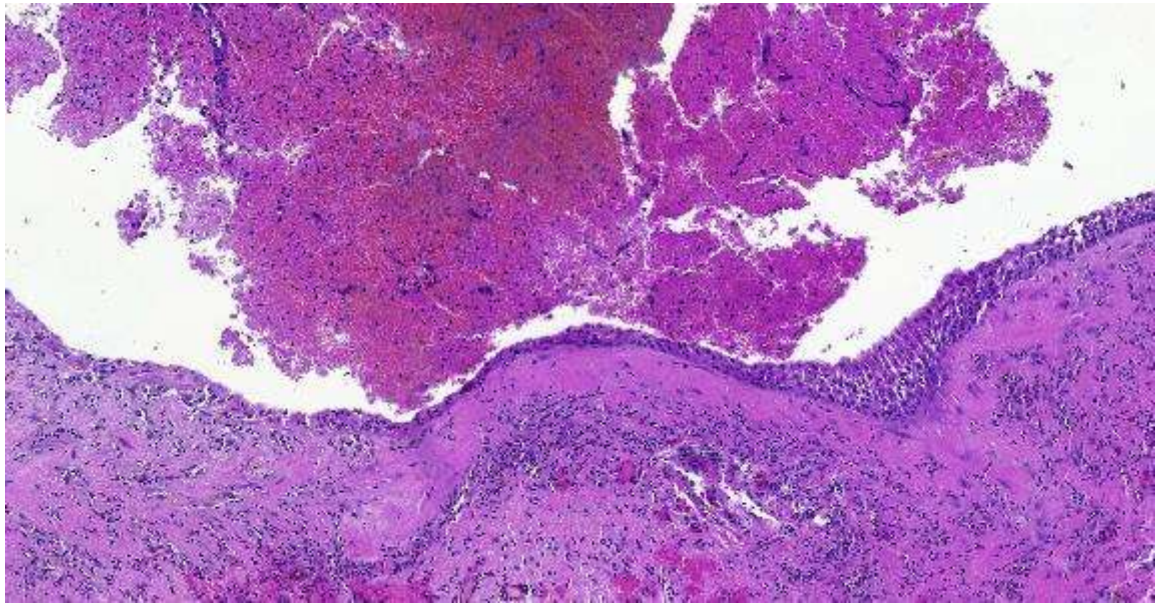

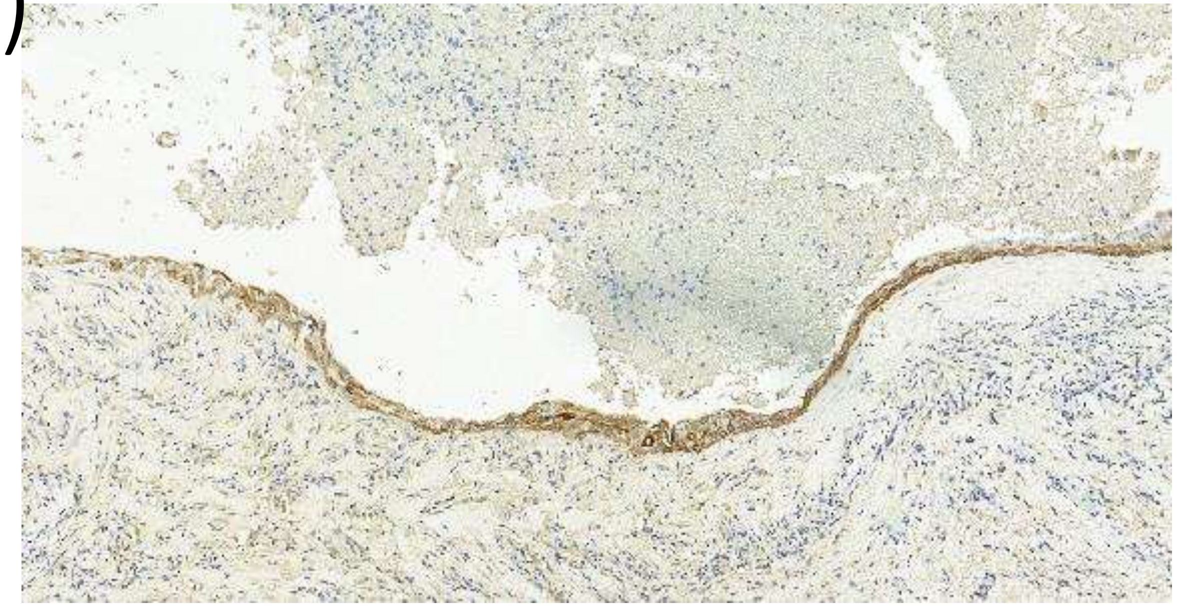

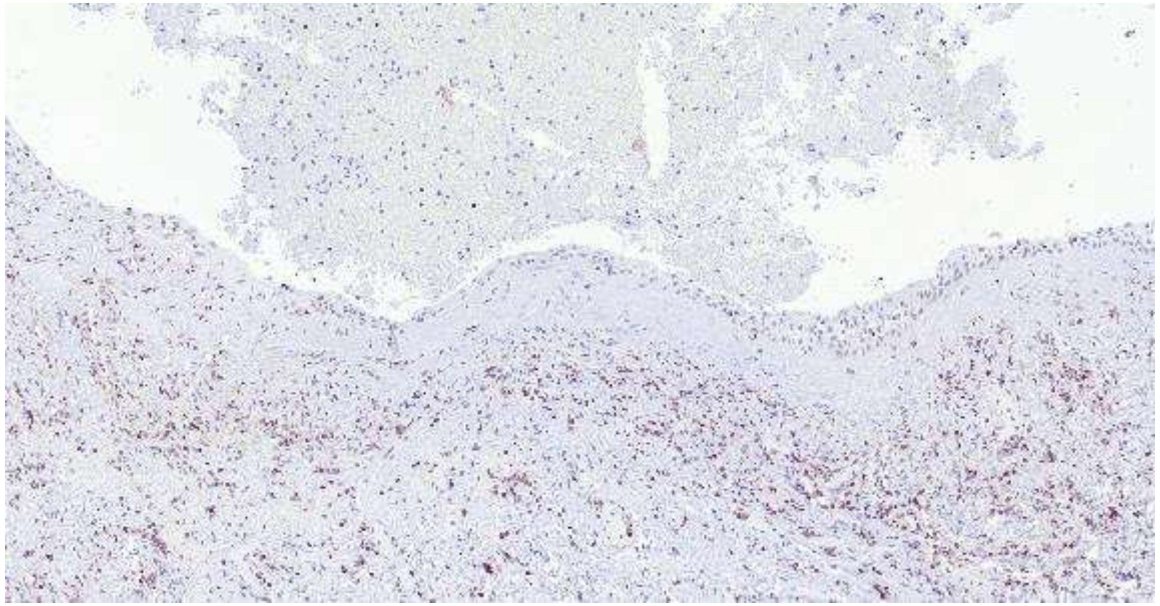

K5 – Conclusion

- H&E: relatively thin (focally thick) squamous epithelium, without keratosis, no wavy surface, no basal palisading, even epithelial-subepithelial contours
- Moderate chronic inflammation of the cyst wall with haemorrhage, haemosiderin and cholesterol granuloma; few neutrophils, too
- Bcl2: negative
- CK17: dominantly positive (various: basal/suprabasal – basal & superficial – transepithelial) + focally negative
- Histopathology: radicular cyst
- Combined clinical and pathological opinion: radicular cyst

Case K6

- 31-year-old female
- Dental hygiene/status: moderately neglected; partly missing, unclean teeth
- Radiology: 18x18 mm well demarcated radiolucent lesion in region of teeth 21-23.
- Operation: cystectomy in one piece
- Clinical diagnosis: residual cyst

K6 – OPT

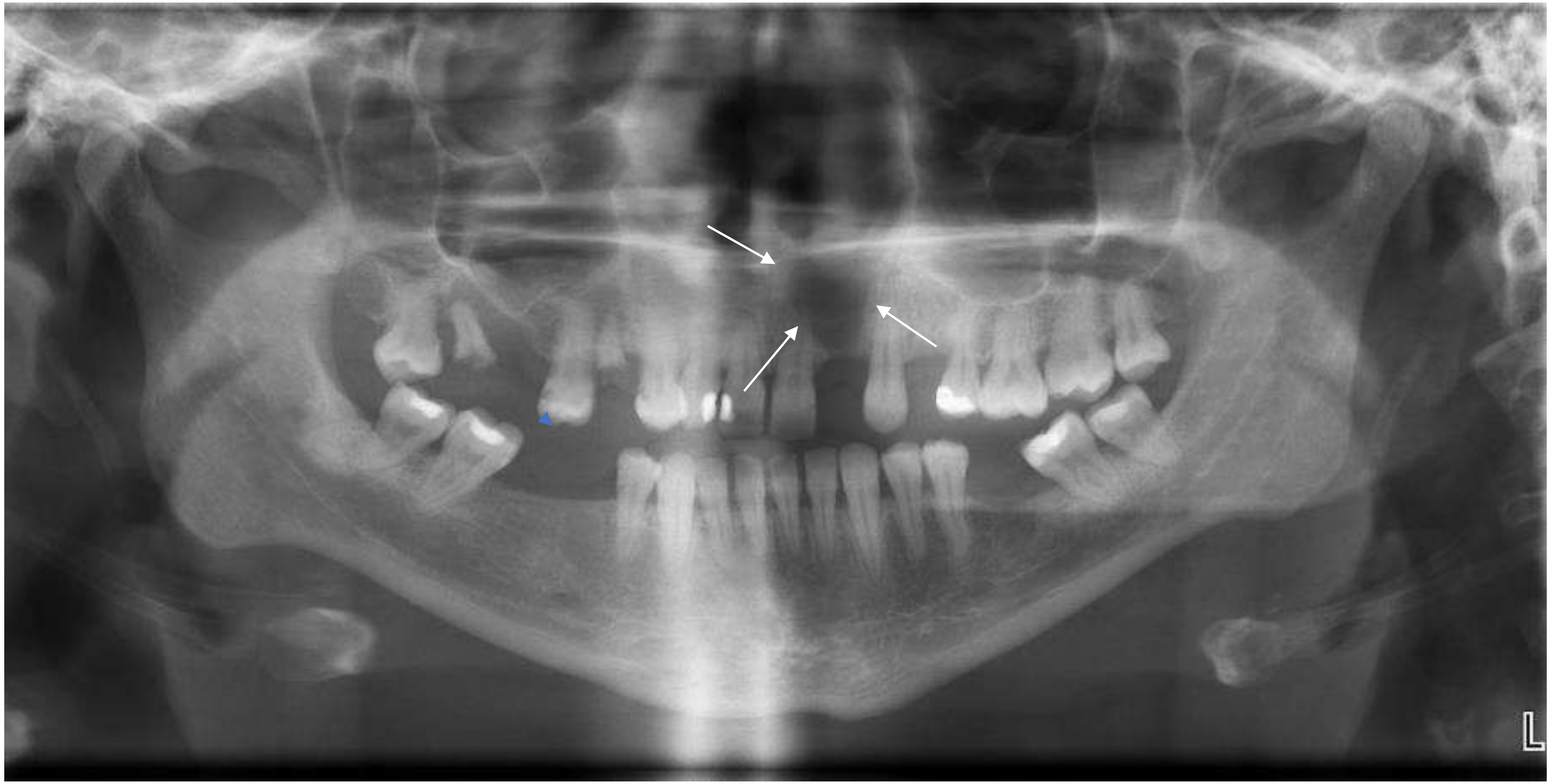

K6 – HE, CK17, bcl2 (x10) very focal superficial CK17+ & focal basal bcl2+

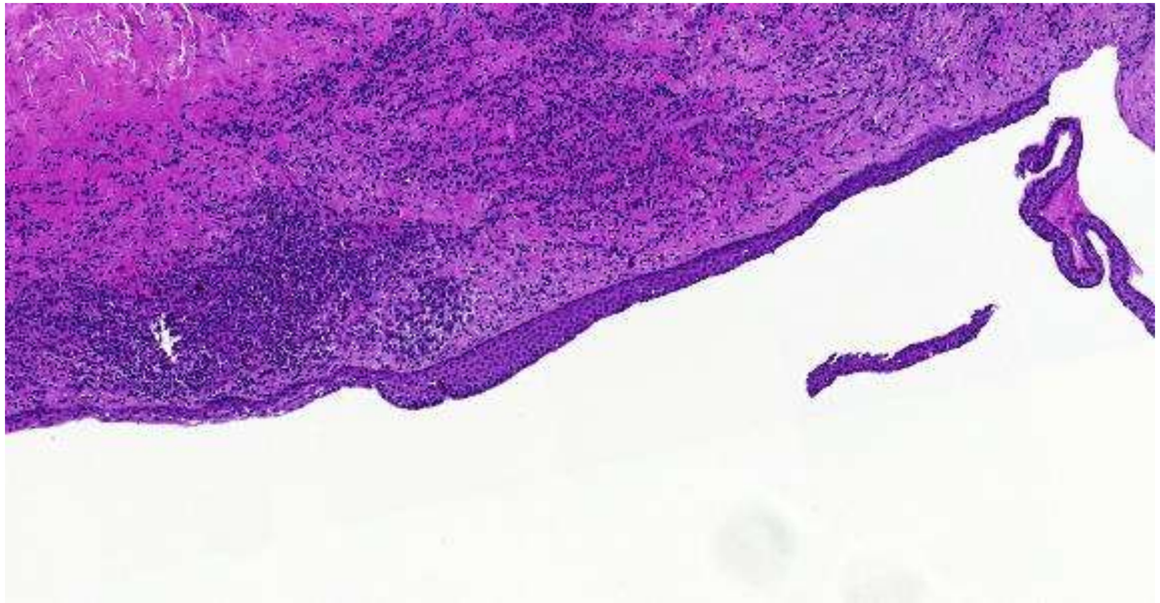

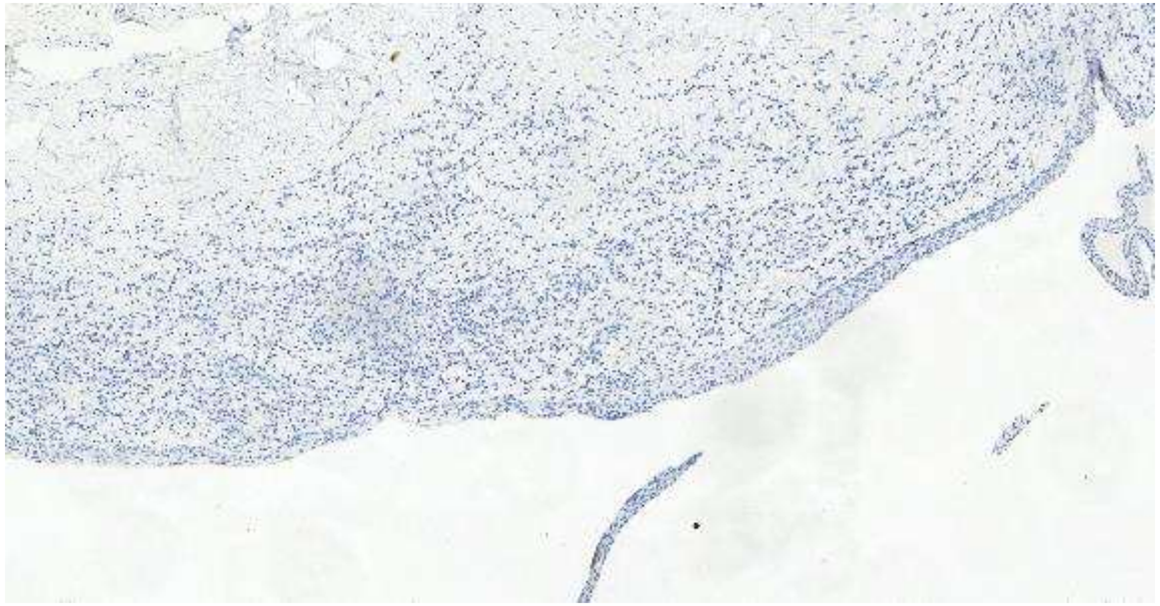

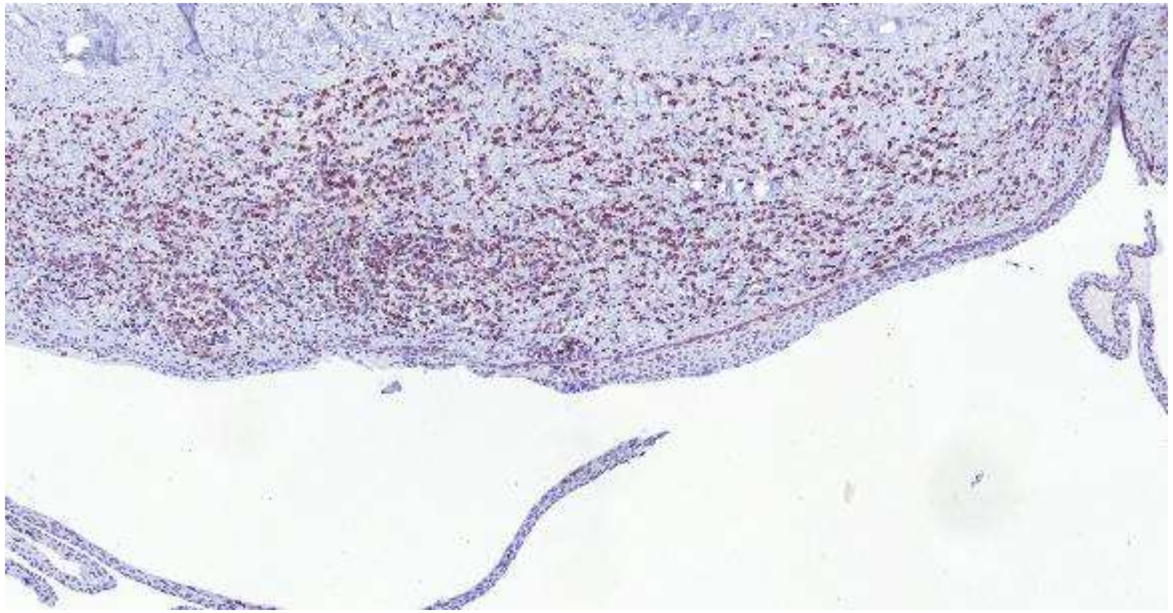

K6 – HE, CK17, bcl2 (x40) very focal superficial CK17+ & focal basal bcl2+

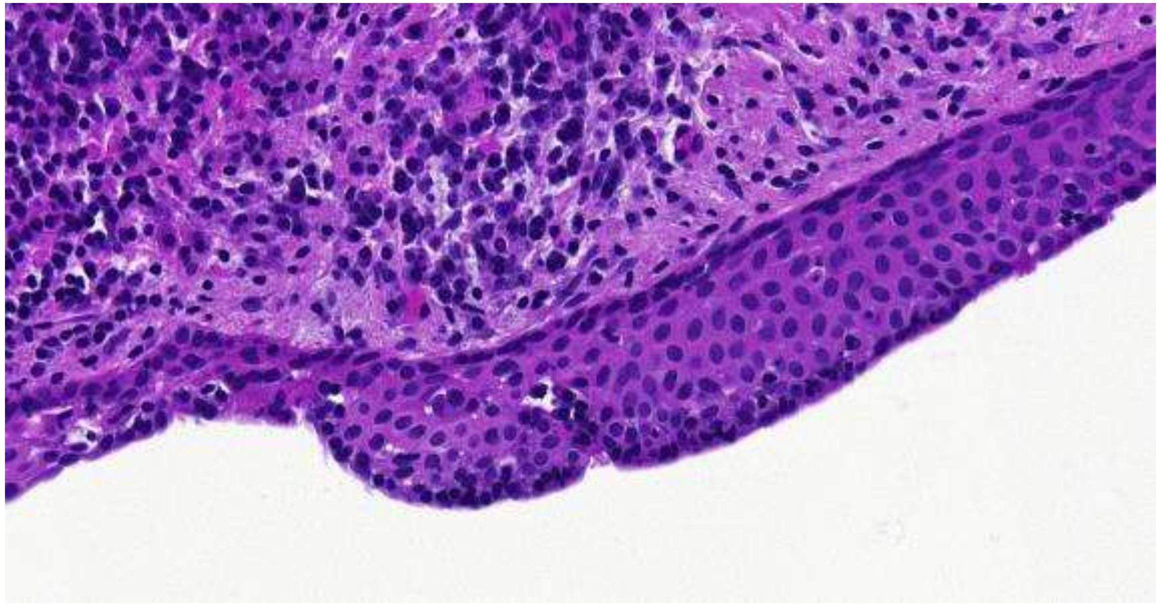

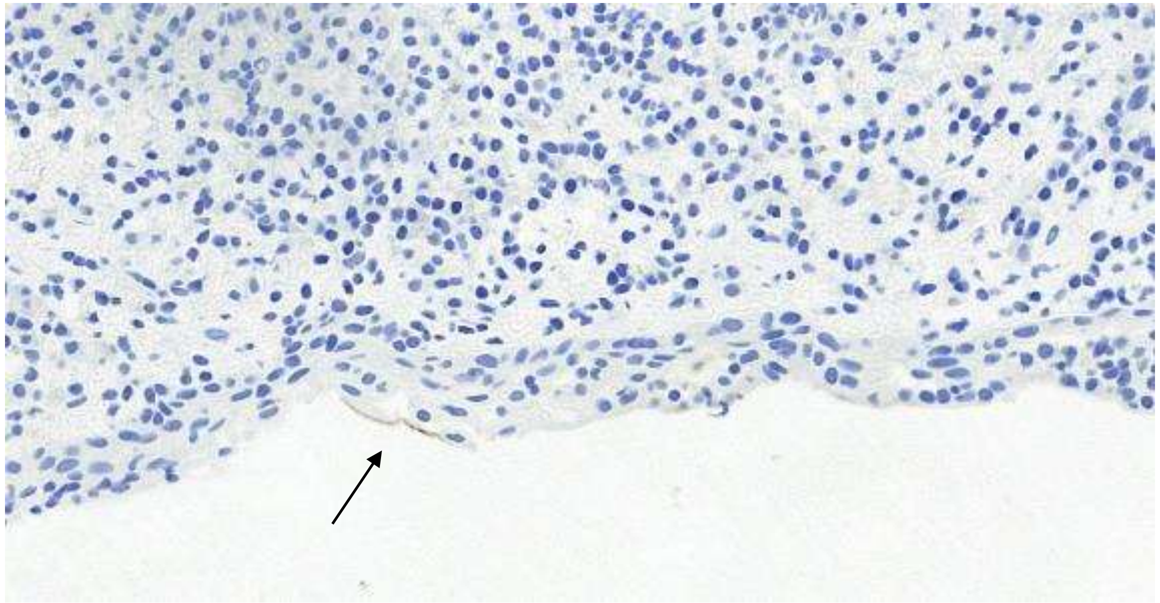

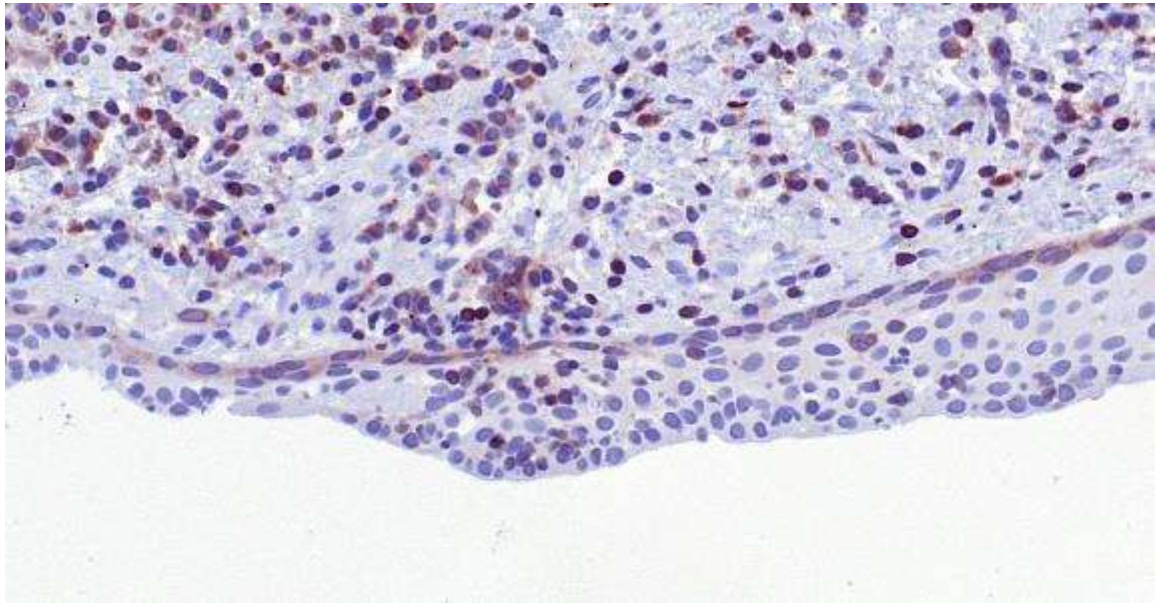

K6 – HE, CK17, bcl2 (x20) focal superficial CK17+ & bcl2 negative

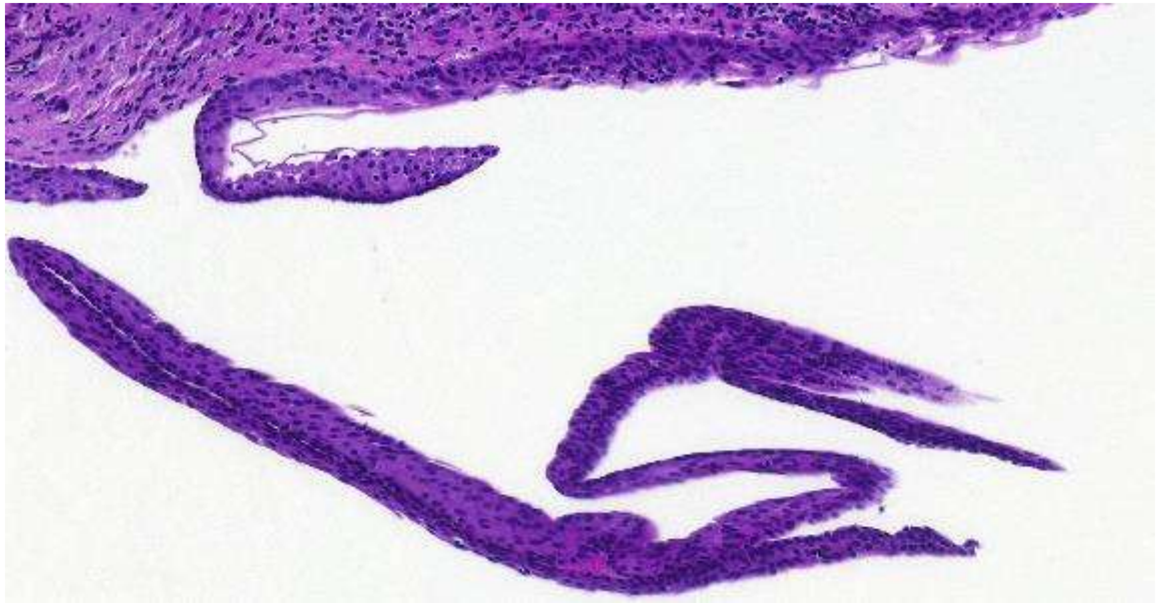

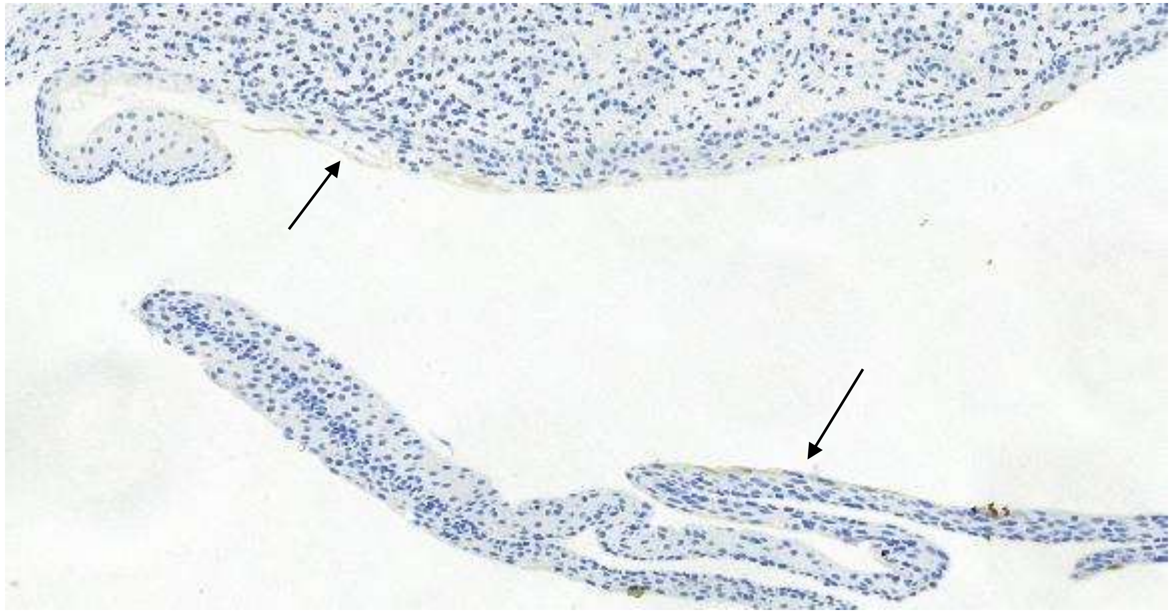

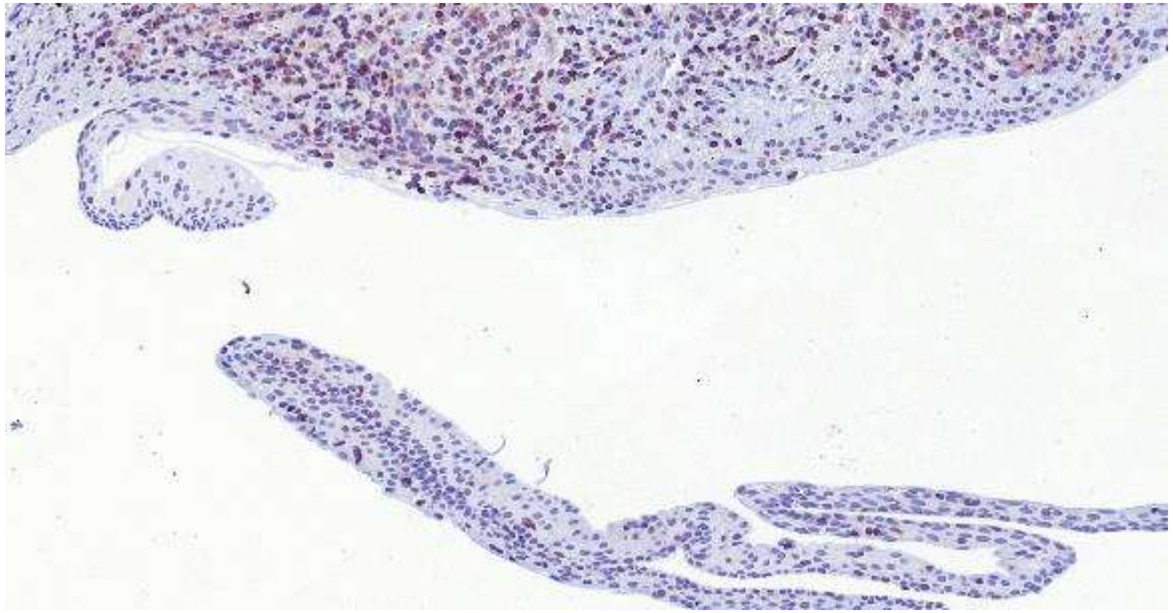

K6 – Focal wavy surface, lack of parakeratosis (x40)

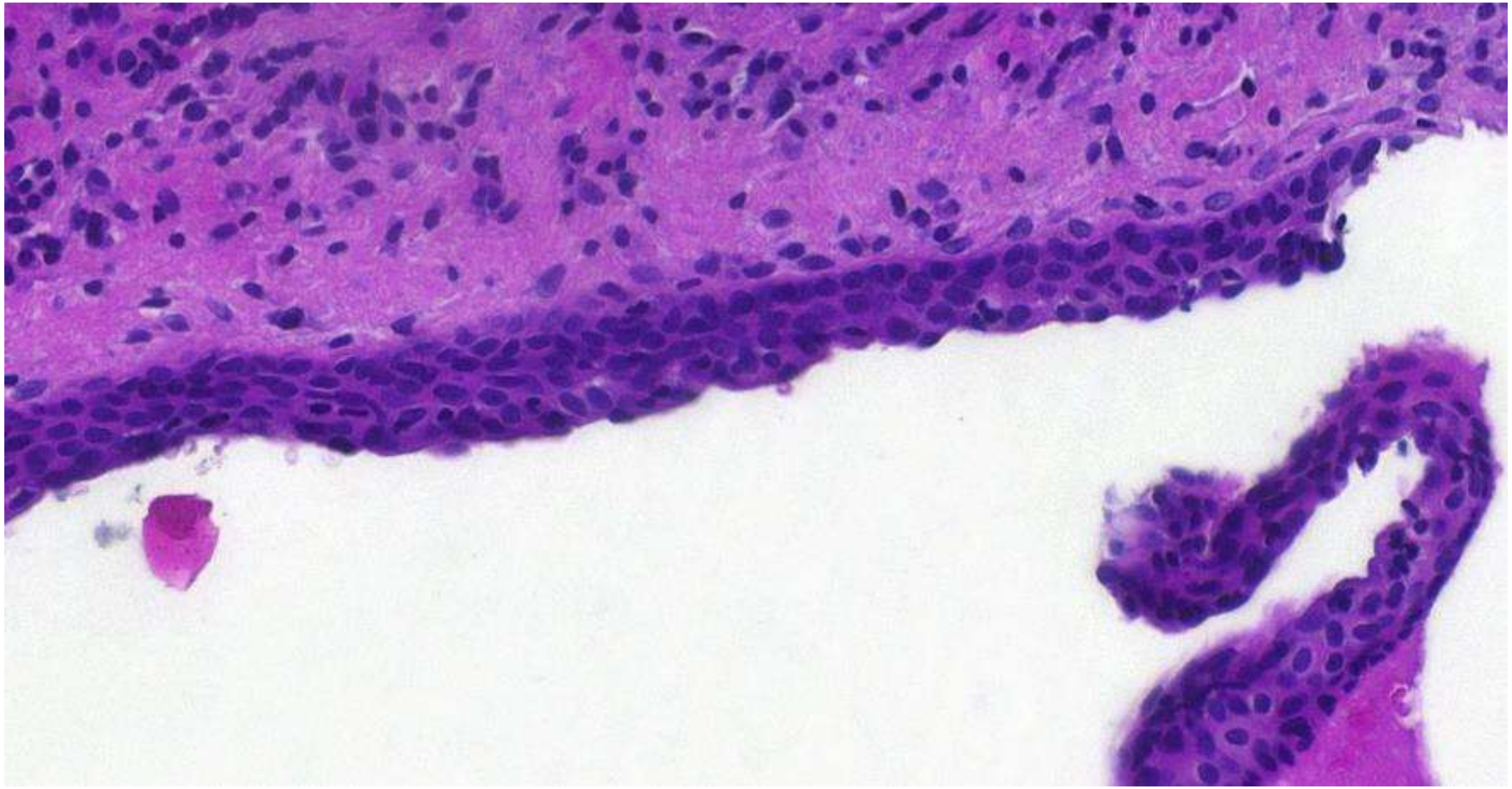

K6 – Keratosis? (x40)

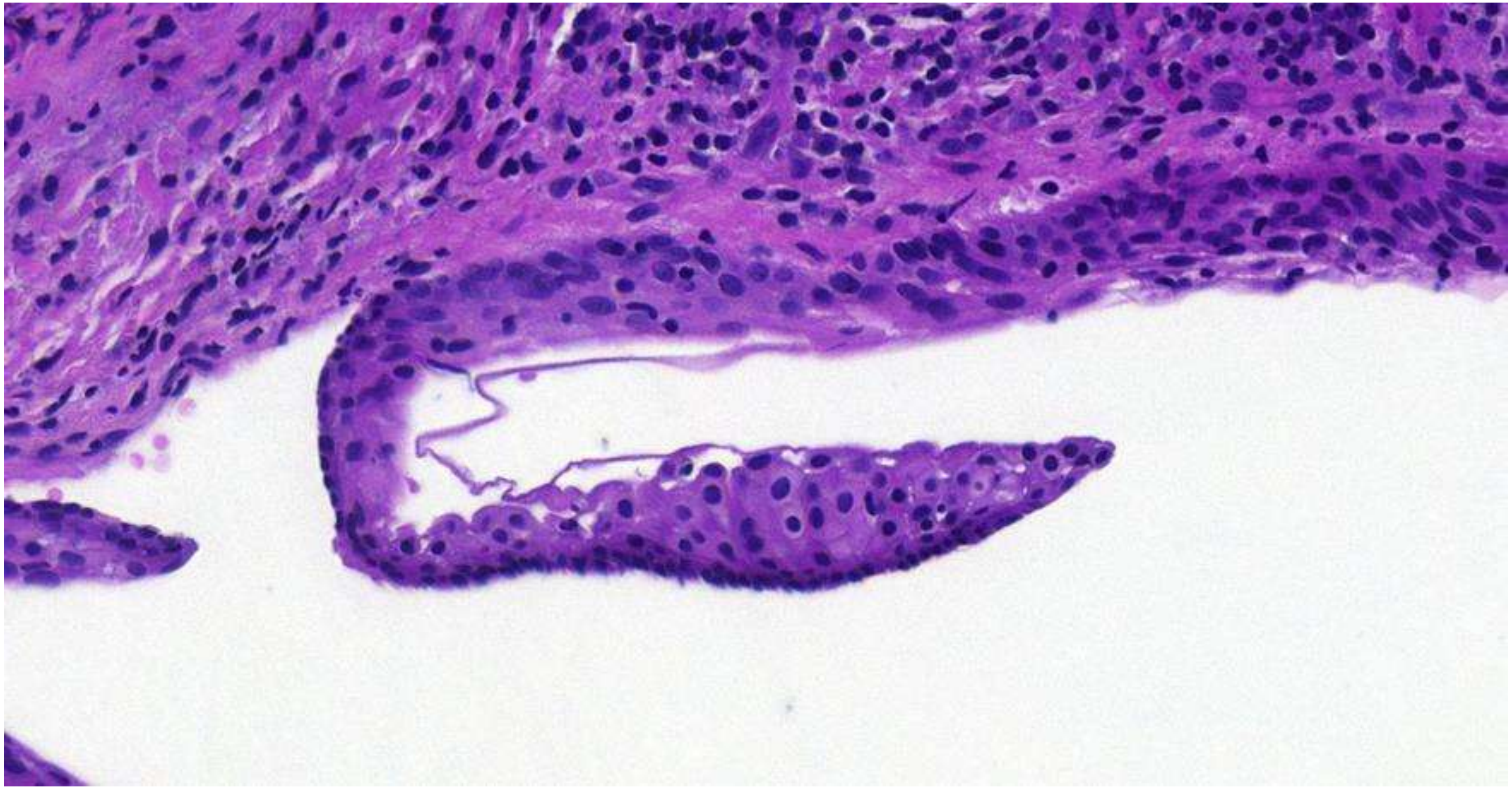

K6 – Lack of basal palisading but basal bcl2 staining

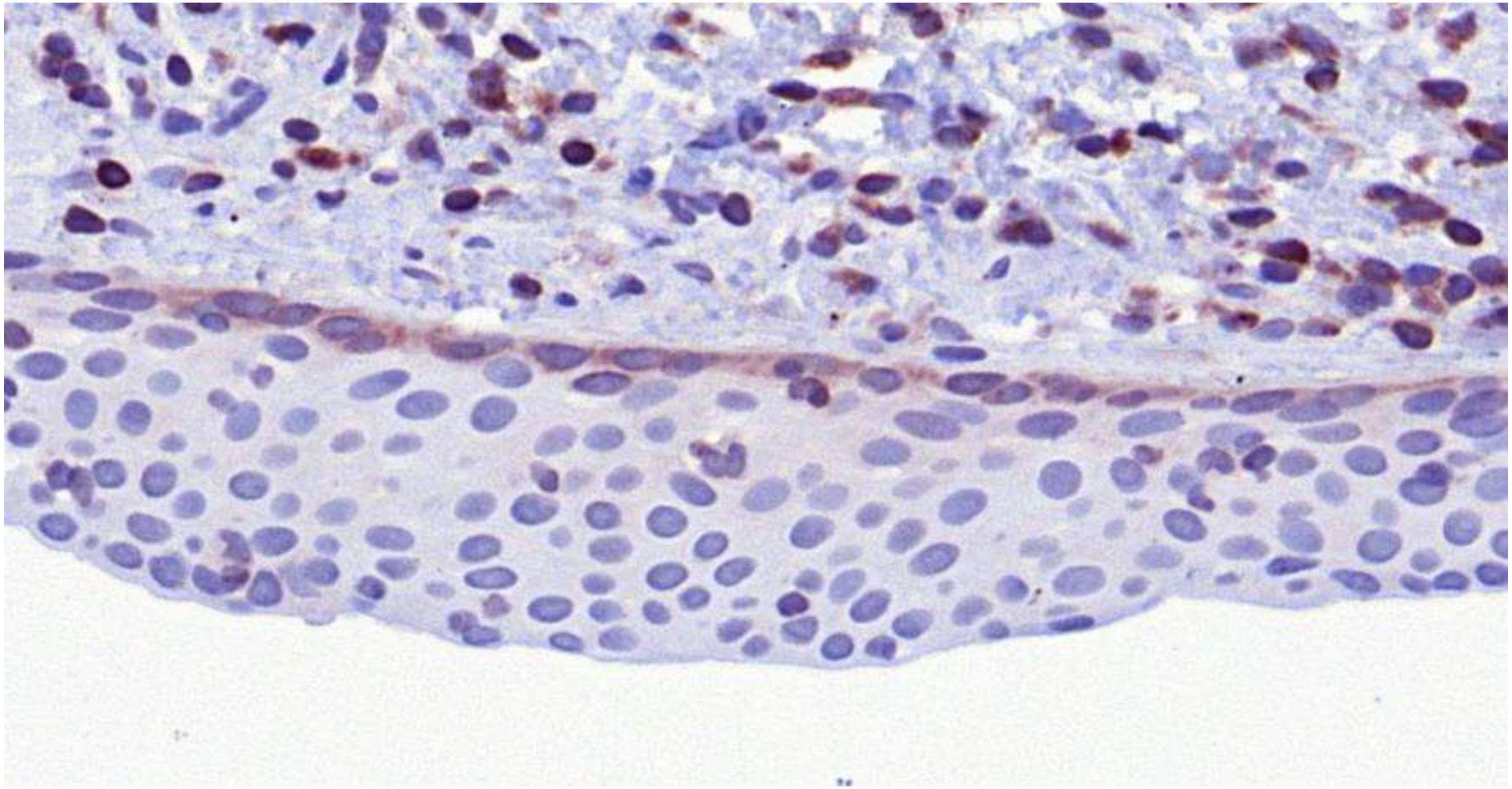

K6 – Denuded epithelium without basal bcl2 staining; few intraepithelial lymphocytes are positive (x50)

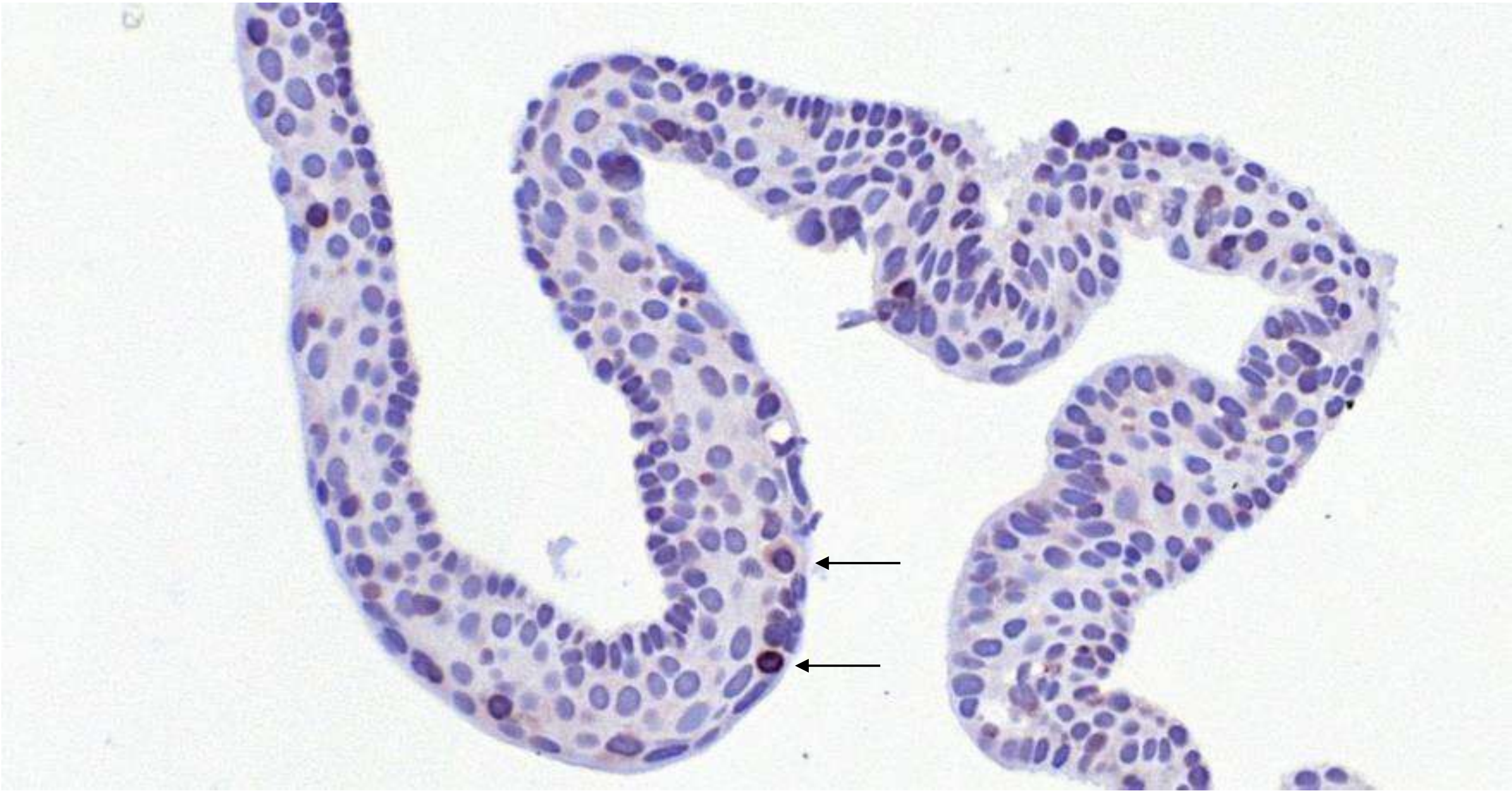

K6 – Conclusion

- H&E: relatively thin squamous epithelium (largely detached), uncertain parakeratosis, focally wavy surface, no basal palisading, even epithelial-subepithelial contours
- Moderate chronic inflammation of the cyst wall
- Bcl2: negative, but focally the flat basal cells are positive contiguously
- CK17: focal superficial cells staining
- Histopathology: residual cyst
- Combined clinical and pathological opinion: residual cyst

Case K7

- 31-year-old female
- Dental hygiene/status: normal; maintained and treated, crowded teeth
- History: the lesion was detected on radiography 2 years previously, the lesion enlarged, paraesthesia of the tongue was noted and increased lately.
- Radiology: 20x15 mm well demarcated radiolucent lesion between teeth 43 and 44, fistula orifice at the neck of tooth 44.
- Operation: excochleation of the fistula orifice and infundibulum; excisional biopsy of the cyst wall, during which the whole cyst comes out… (installation of a cyst obturator)
- Clinical diagnosis: mandibular cyst between vital teeth 43 and 44

K7 OPT

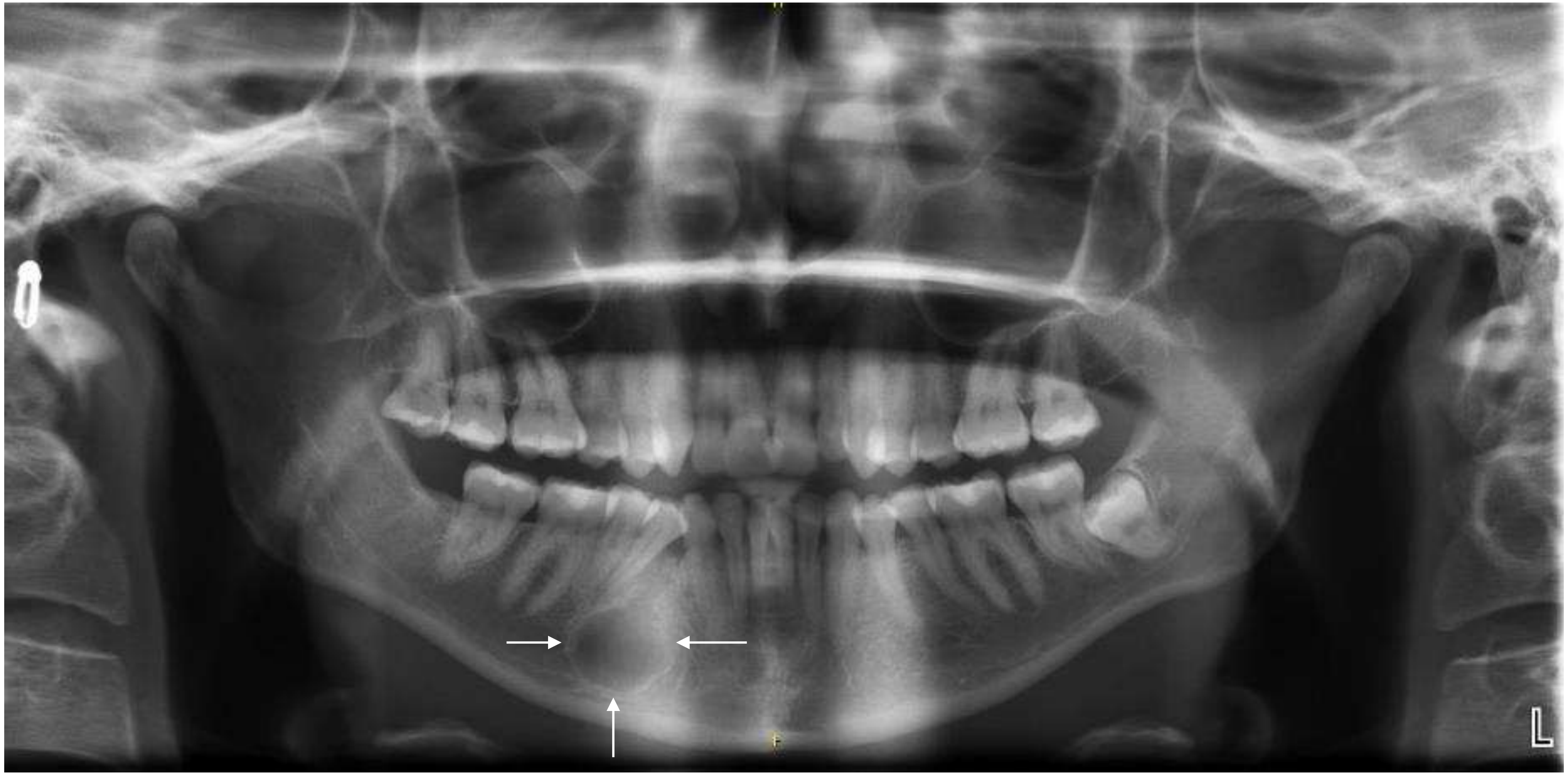

OPT detail magnified

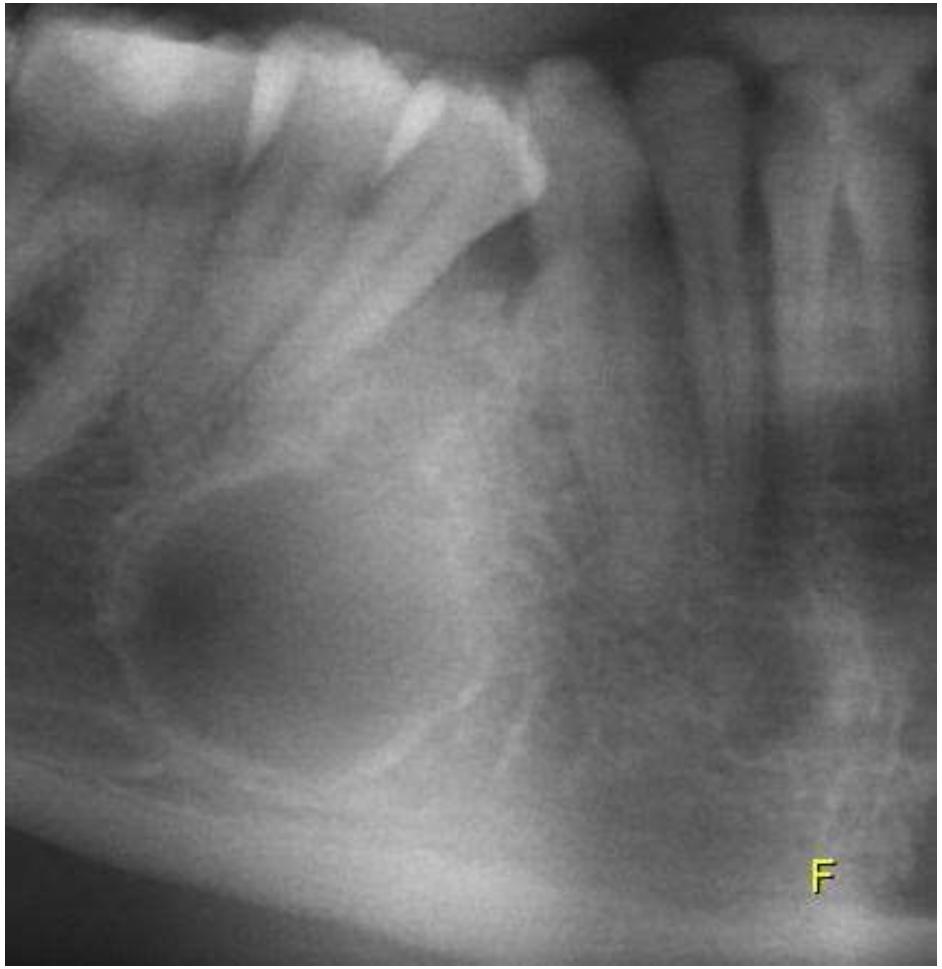

K7 – HE, CK17
bcl2, CK10 (x1.2)

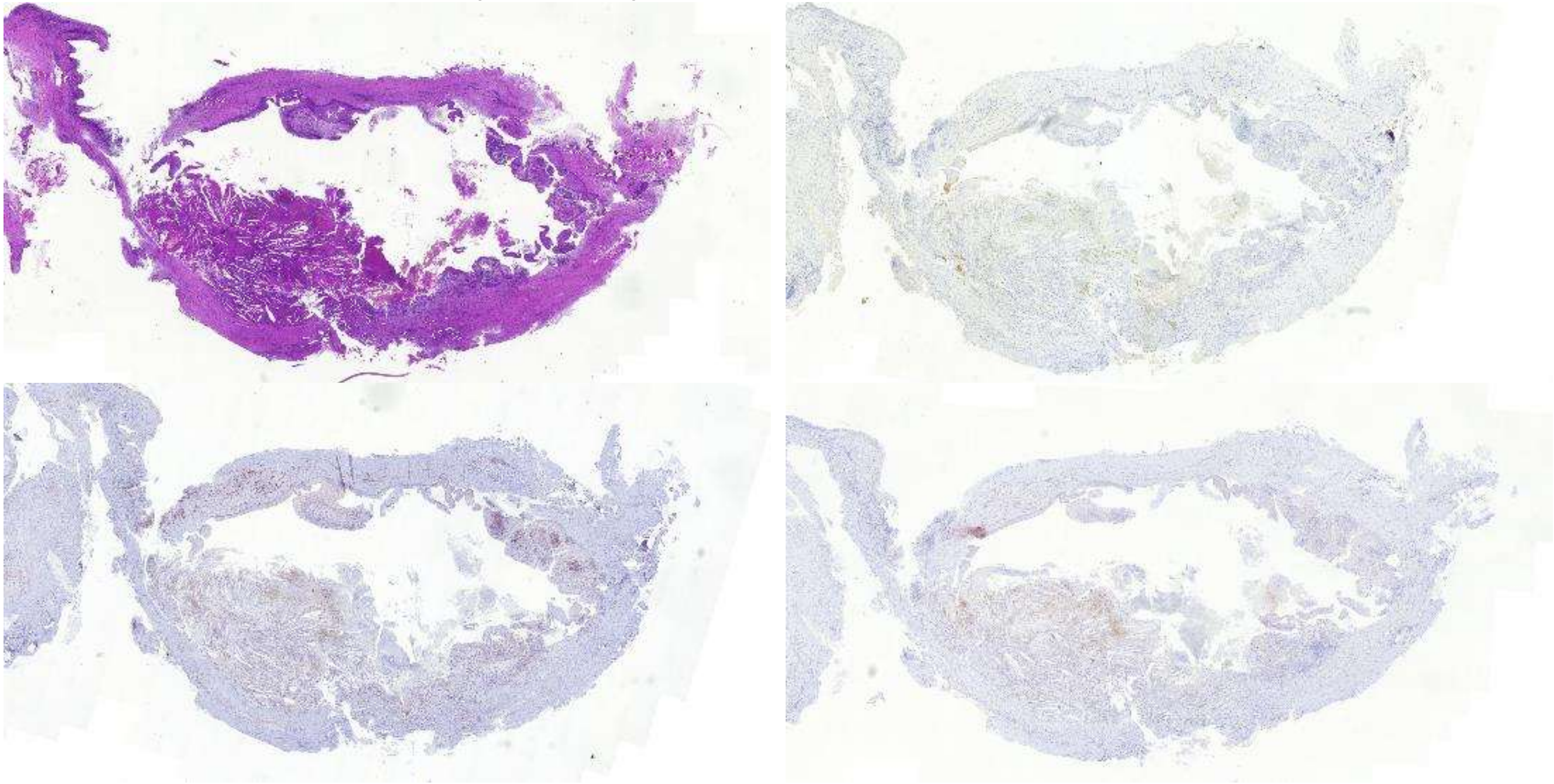

K7 – HE, CK17 focal transepithelial (**), superficial (→) and negative (*)
bcl2, CK10 (x5)

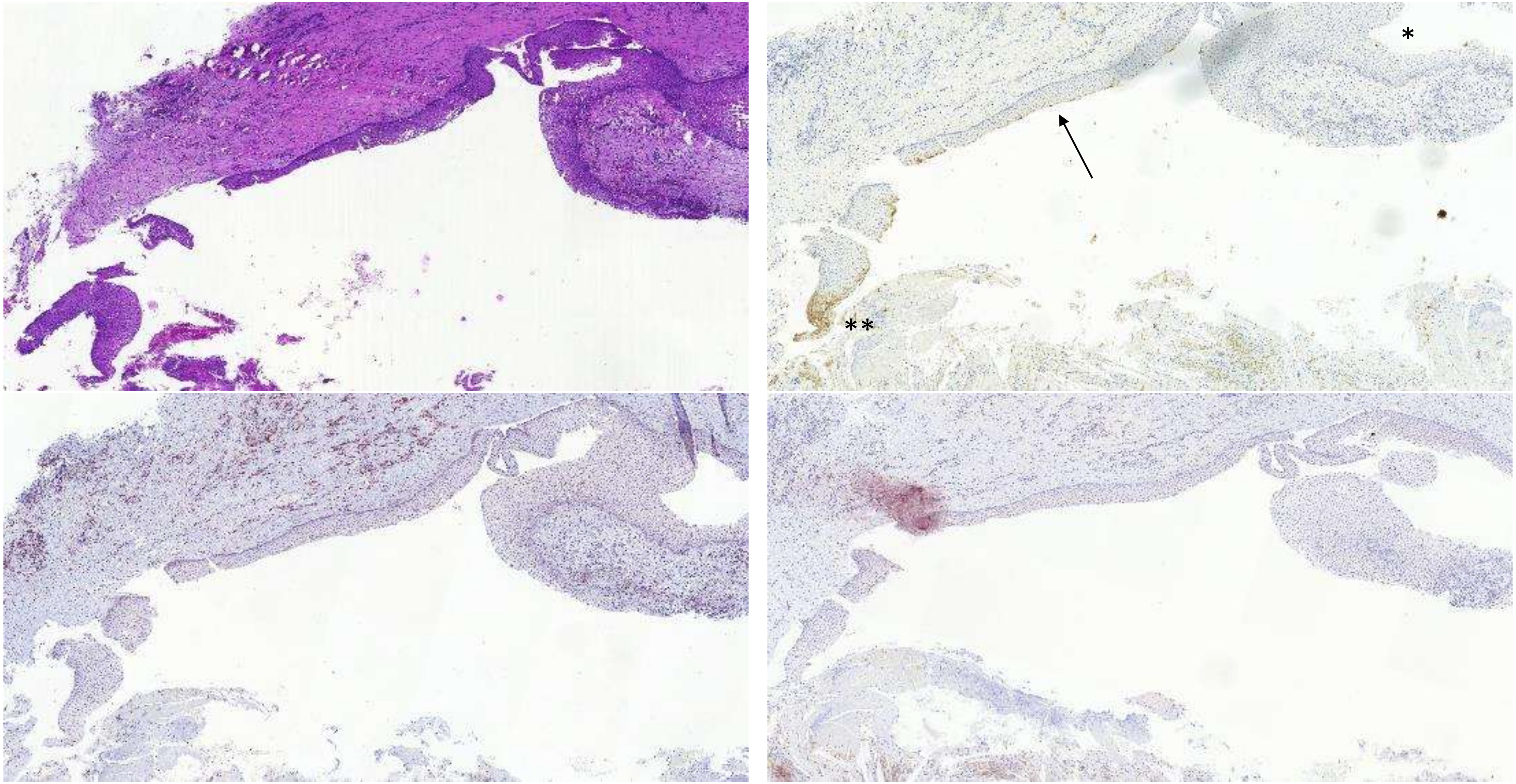

K7 – HE, CK17 (focal transepithelial) (*) bcl2 (negative), CK10 (negative) (x10)

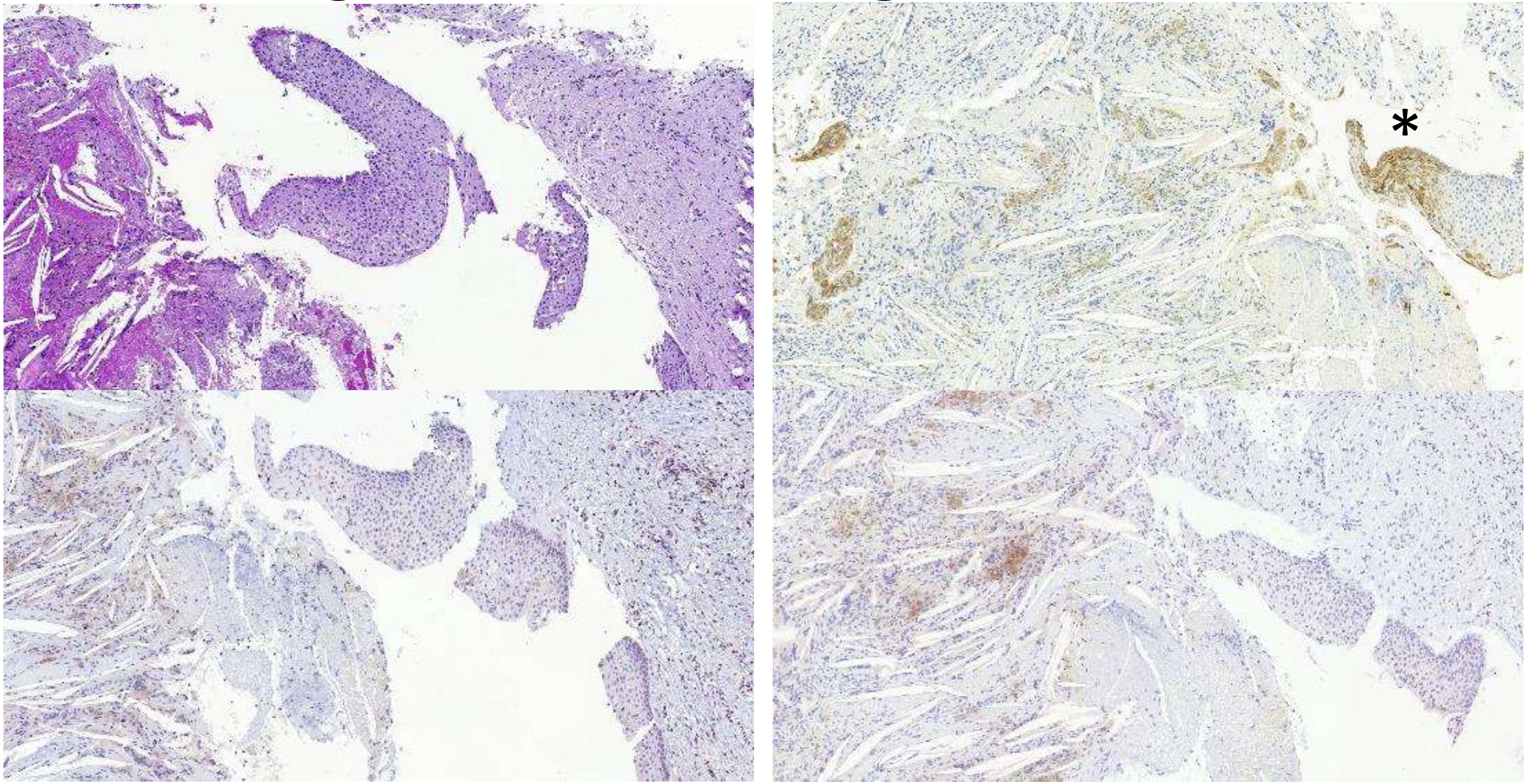

K7 – HE, CK17 (negative)
bcl2 (basal staining), CK10 (x10)(negative)

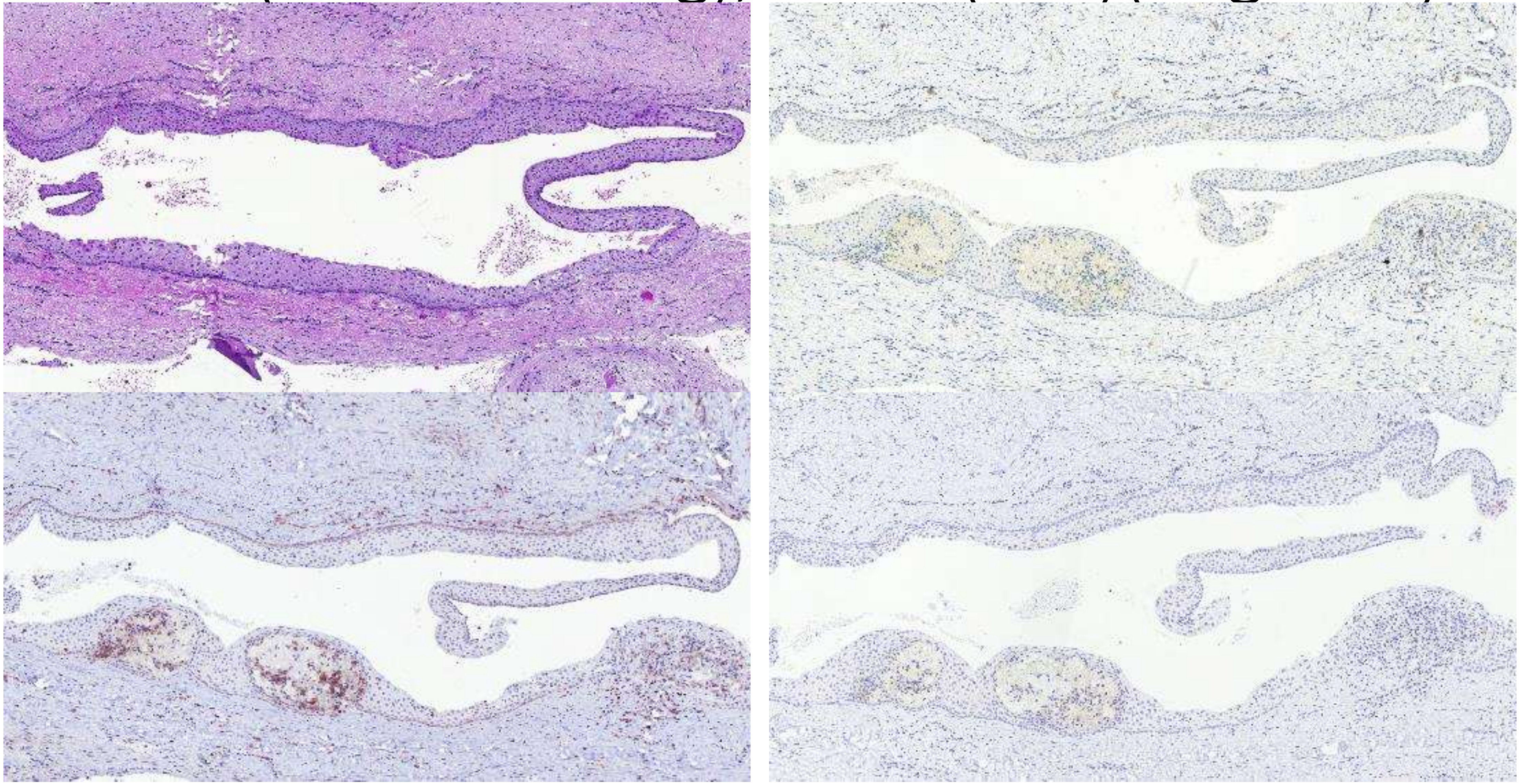

K7 – HE (x25)

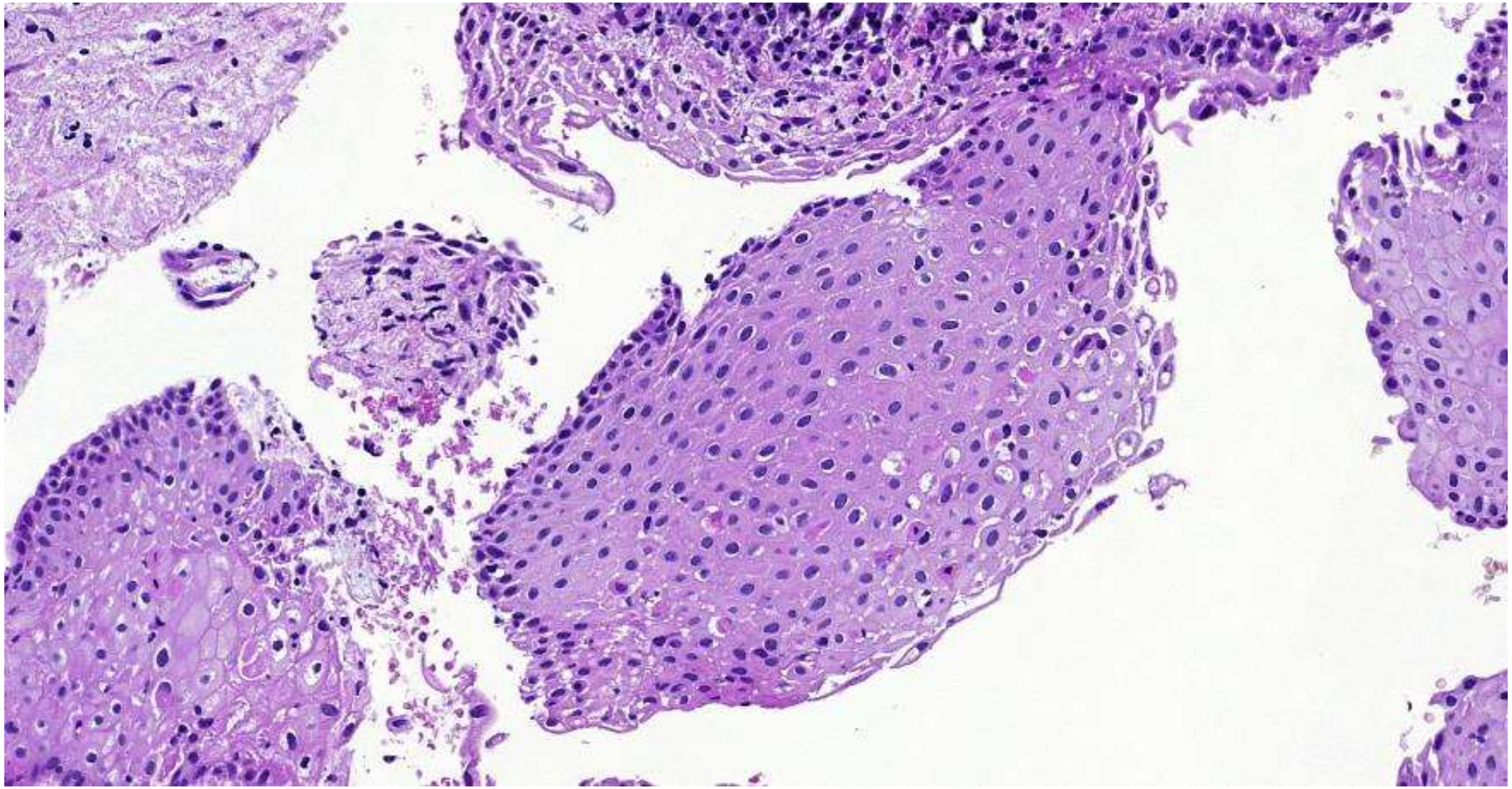

K7 – HE (x25) – wavy surface (x40), with no basal palisading

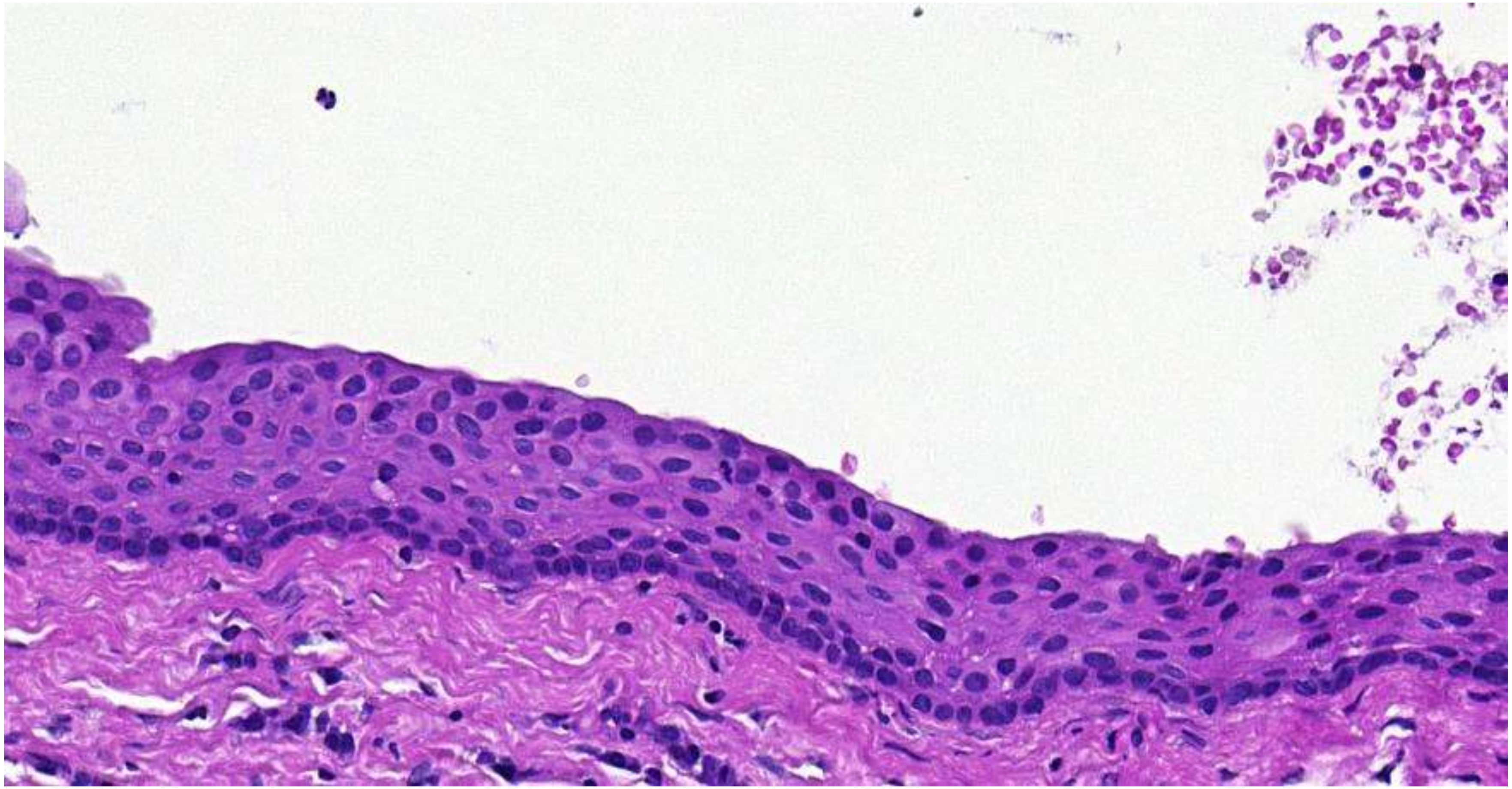

K7 – bcl2 (x40) faint basal staining, wavy surface, no basal palisading

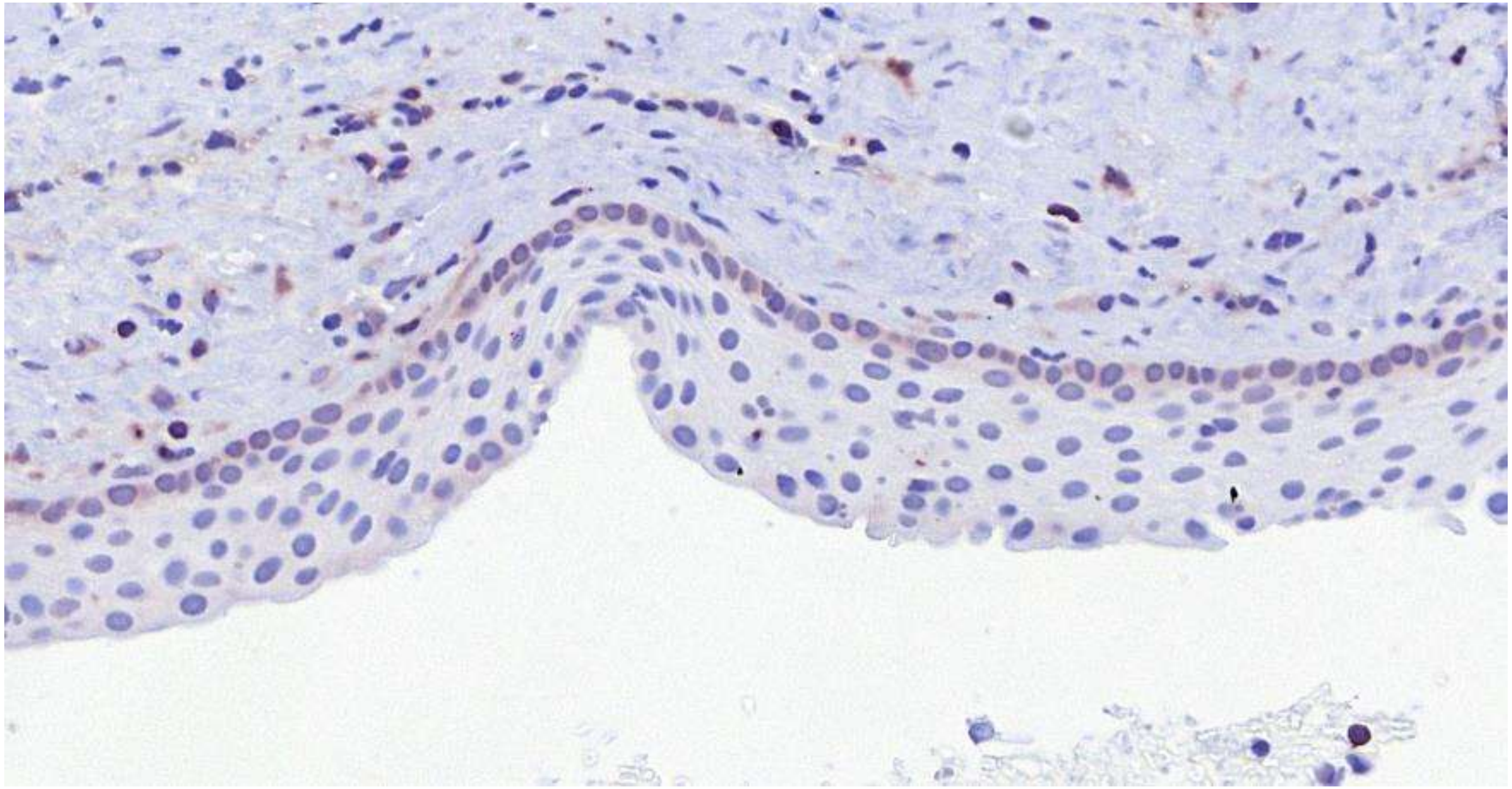

K7 – Conclusion

- H&E: segmentally thin and thick squamous epithelium, no parakeratosis, focally wavy surface (thin segment), no basal palisading, even epithelial-subepithelial contours on thin segment, uneven on thick segment
- Inflammation of the cyst wall beneath the thick epithelium and cholesterol granuloma; no inflammation in the thin epithelial segment
- Bcl2: negative, but flat basal cells staining (of unknown significance)
- CK17: few superficial cells staining; minimal transepithelial
- CK10: negative (few spinous cells sparsely positive)
- Histopathology: inflammatory cyst (radicular cyst)
- Combined clinical and pathological opinion: lateral periodontal cyst

Case K8

- 30-year-old male
- Dental hygiene/status: poor; partly conserved, untreated, multiple roots in the alveoli
- Radiology: Homogeneous radiolucent lesion at the apex of residual root of tooth 48.
- Operation: removal of the dental root and cyst
- Clinical diagnosis: radicular cyst

K8 – OPT

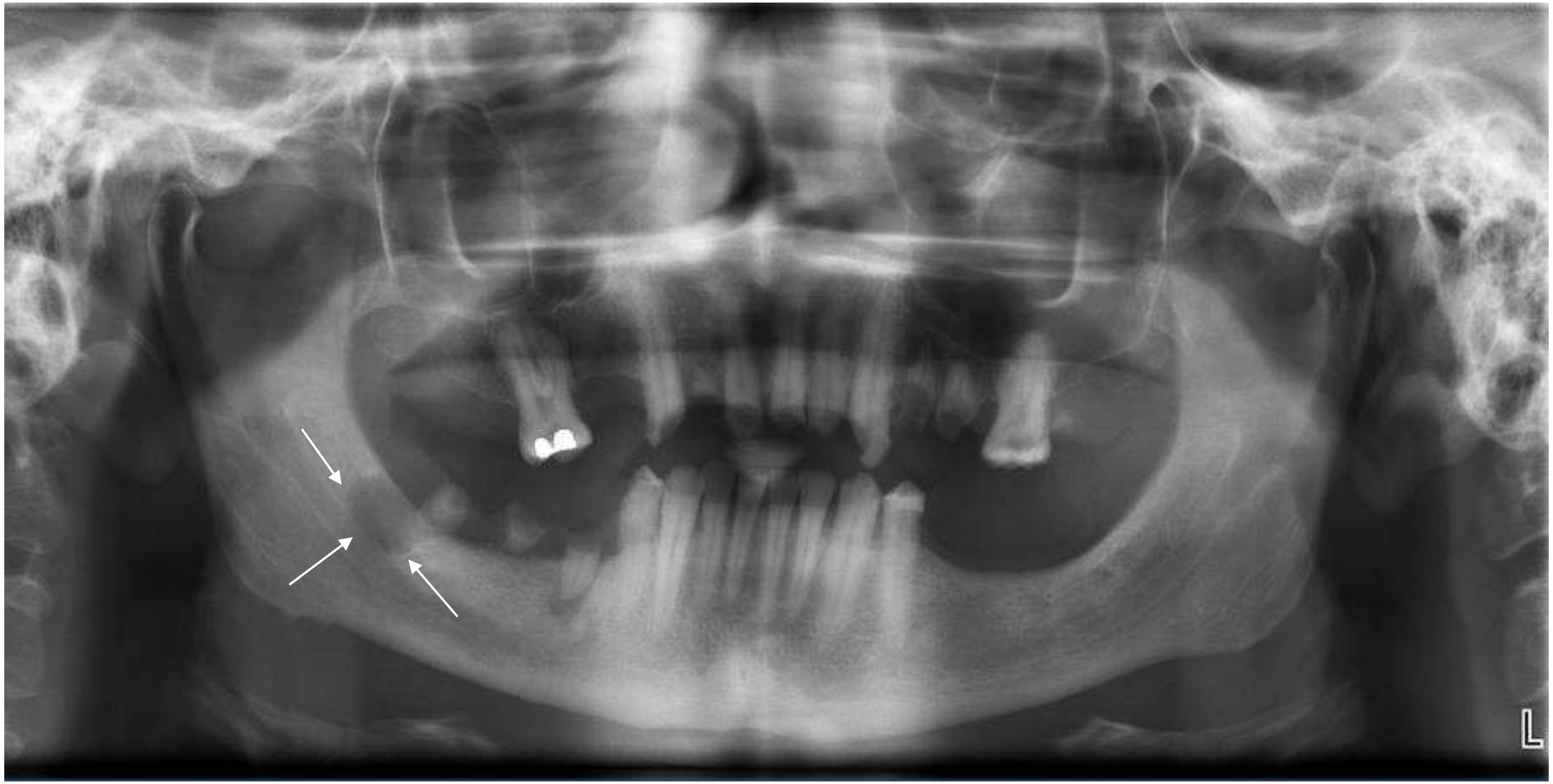

K8 – HE, CK17, bcl2 (x5)

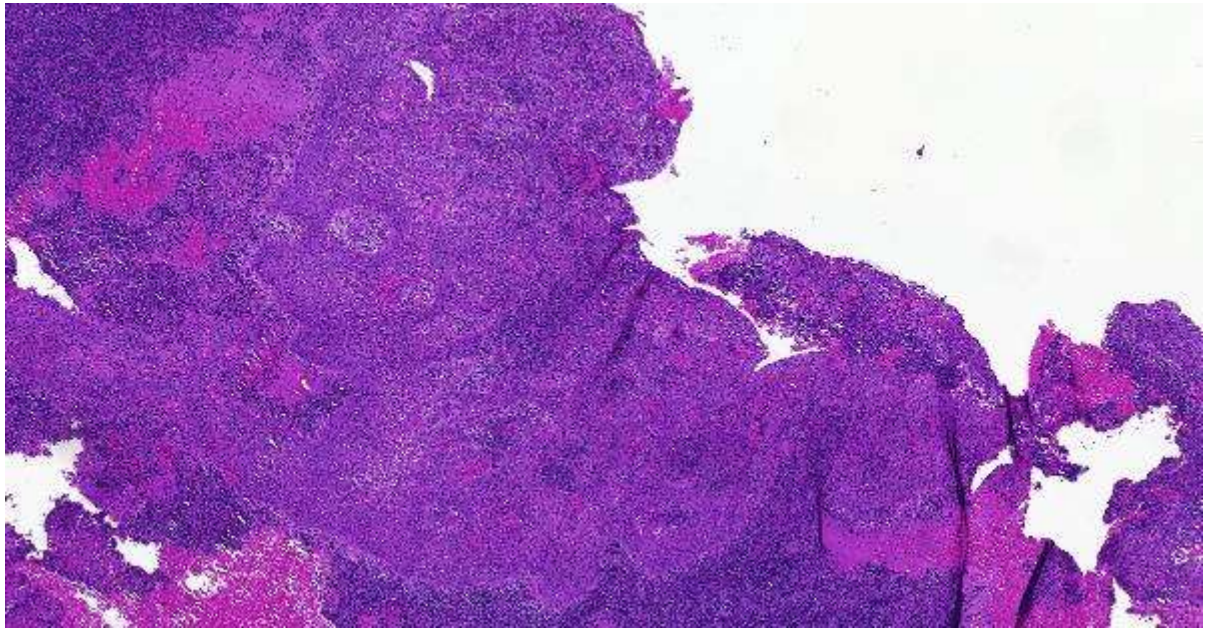

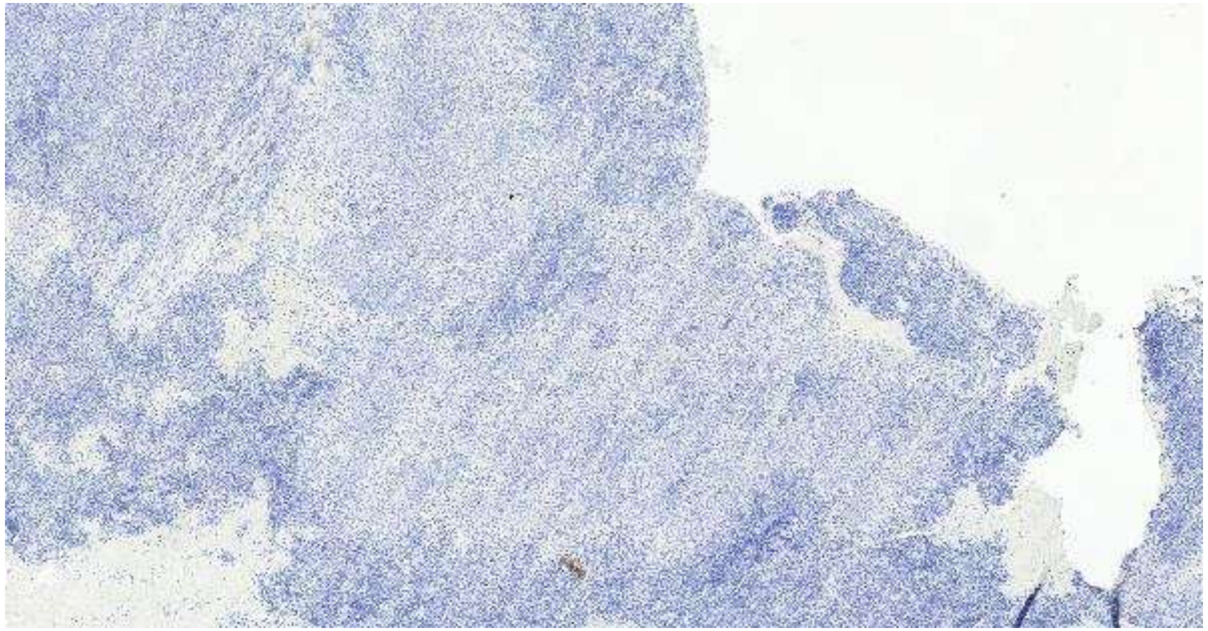

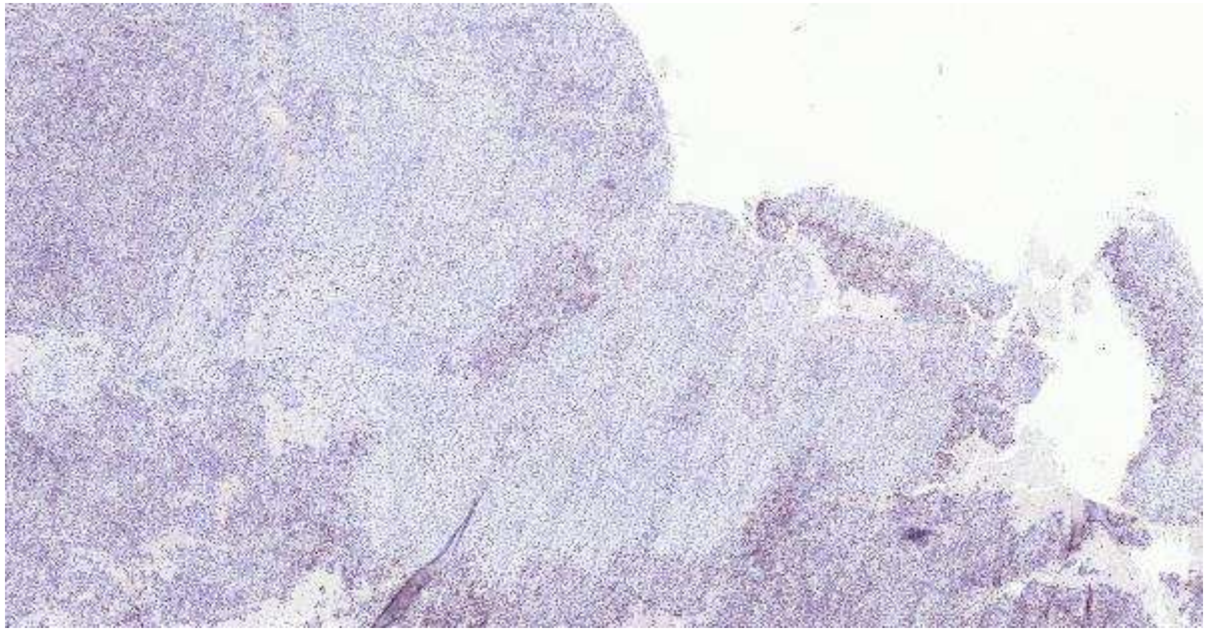

K8 – bcl2 (x20)

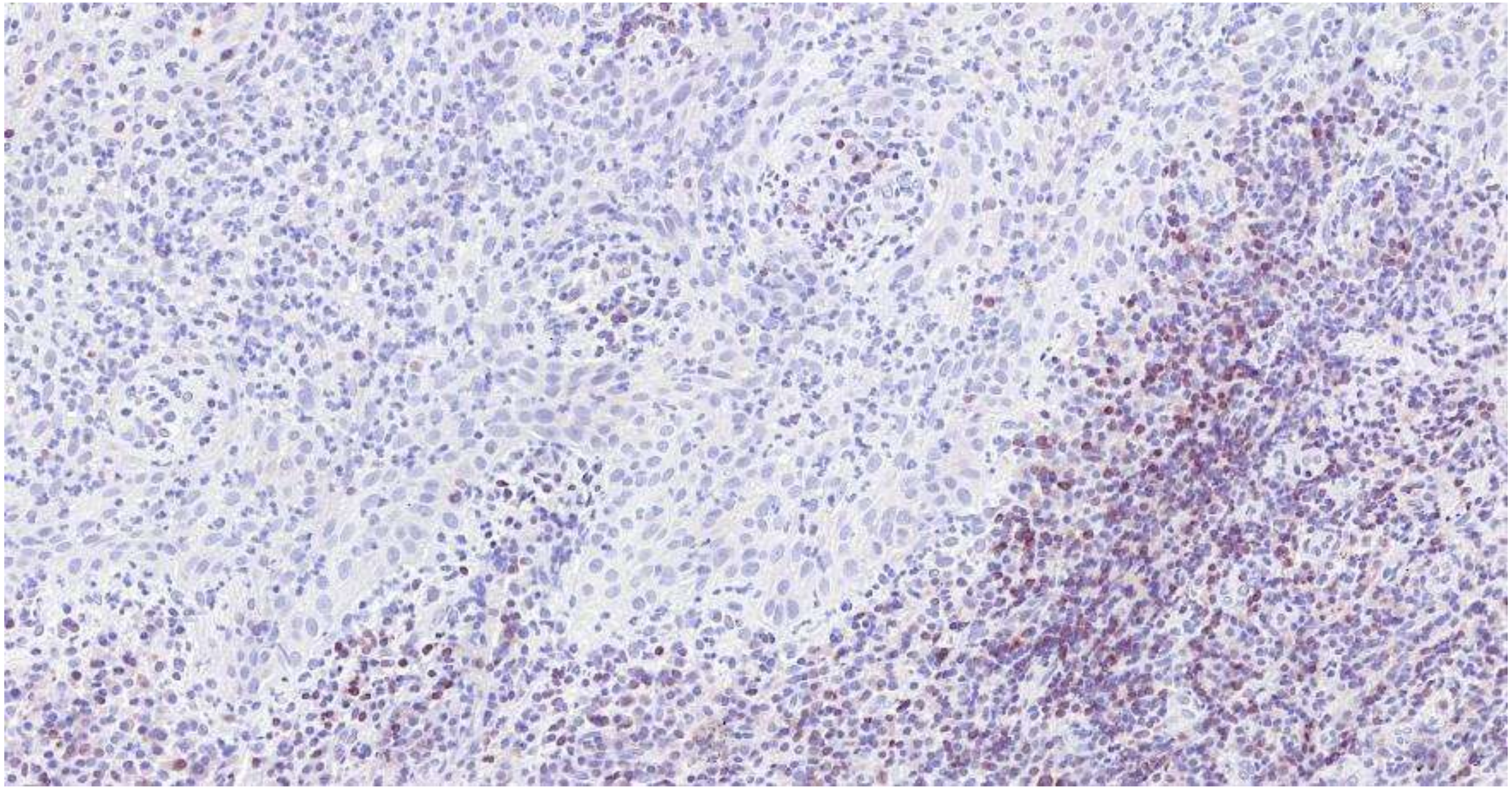

K8 – Conclusion

- H&E: relatively thick squamous epithelium, no parakeratosis, no wavy surface, no basal palisading, uneven epithelial-subepithelial contours
- Mixed inflammatory infiltrate of the cyst wall
- Bcl2: negative
- CK17: negative
- Histopathology: inflammatory odontogenic cyst (radicular cyst)
- Combined clinical and pathological opinion: radicular cyst

Case K9

- 72-year-old female
- Dental hygiene/status: normal; partly missing, partly replaced, partly treated; vestibular mucosa in area of teeth 11 & 21 painful on pression; periodontitis
- Radiology: Radiolucent lesion at the apex of teeth 11-22.
- Operation: removal of the upper bridge, teeth 12, 21 and cyst
- Clinical diagnosis: maxillary cyst

K9 – OPT

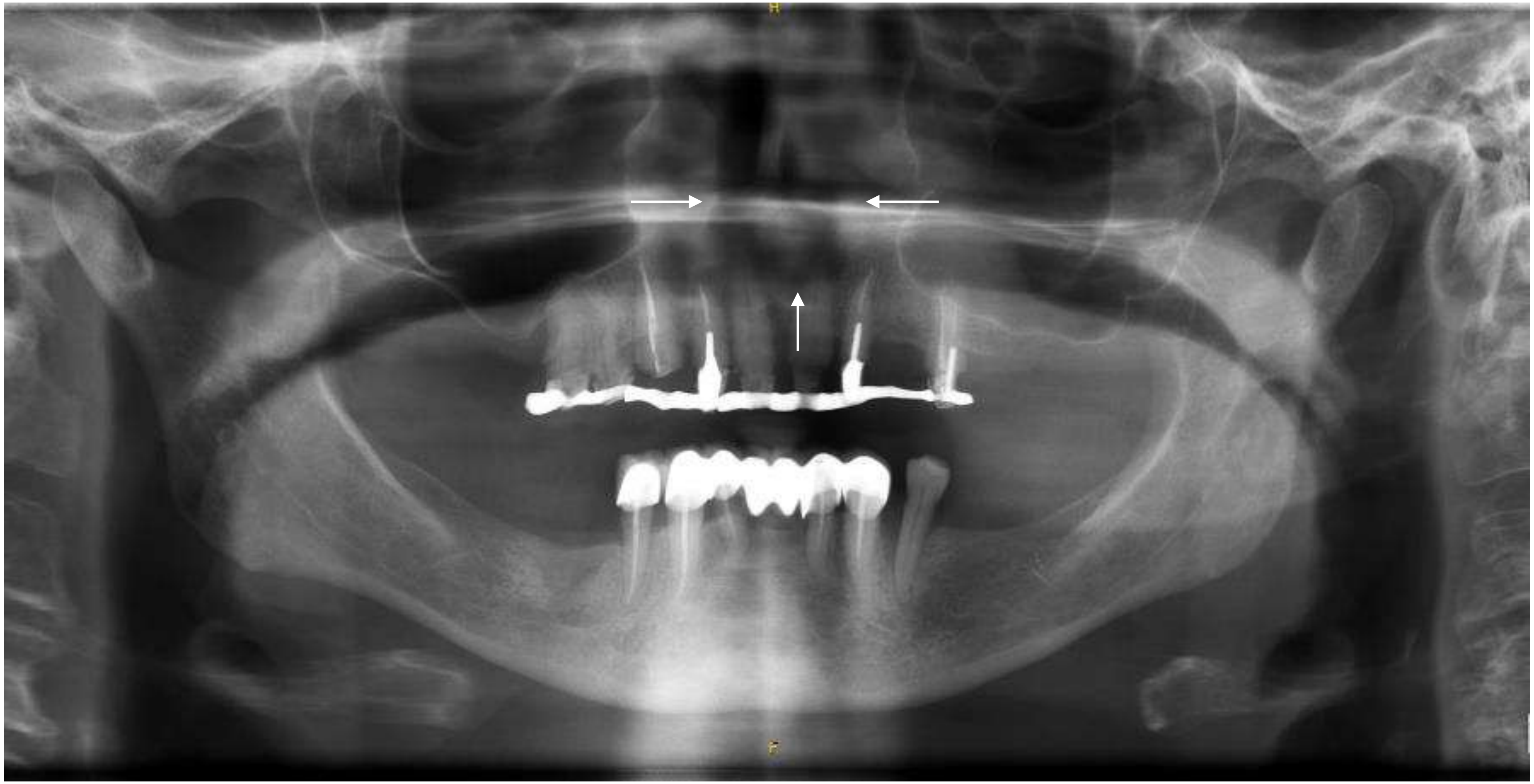

K9 – OPT zoomed

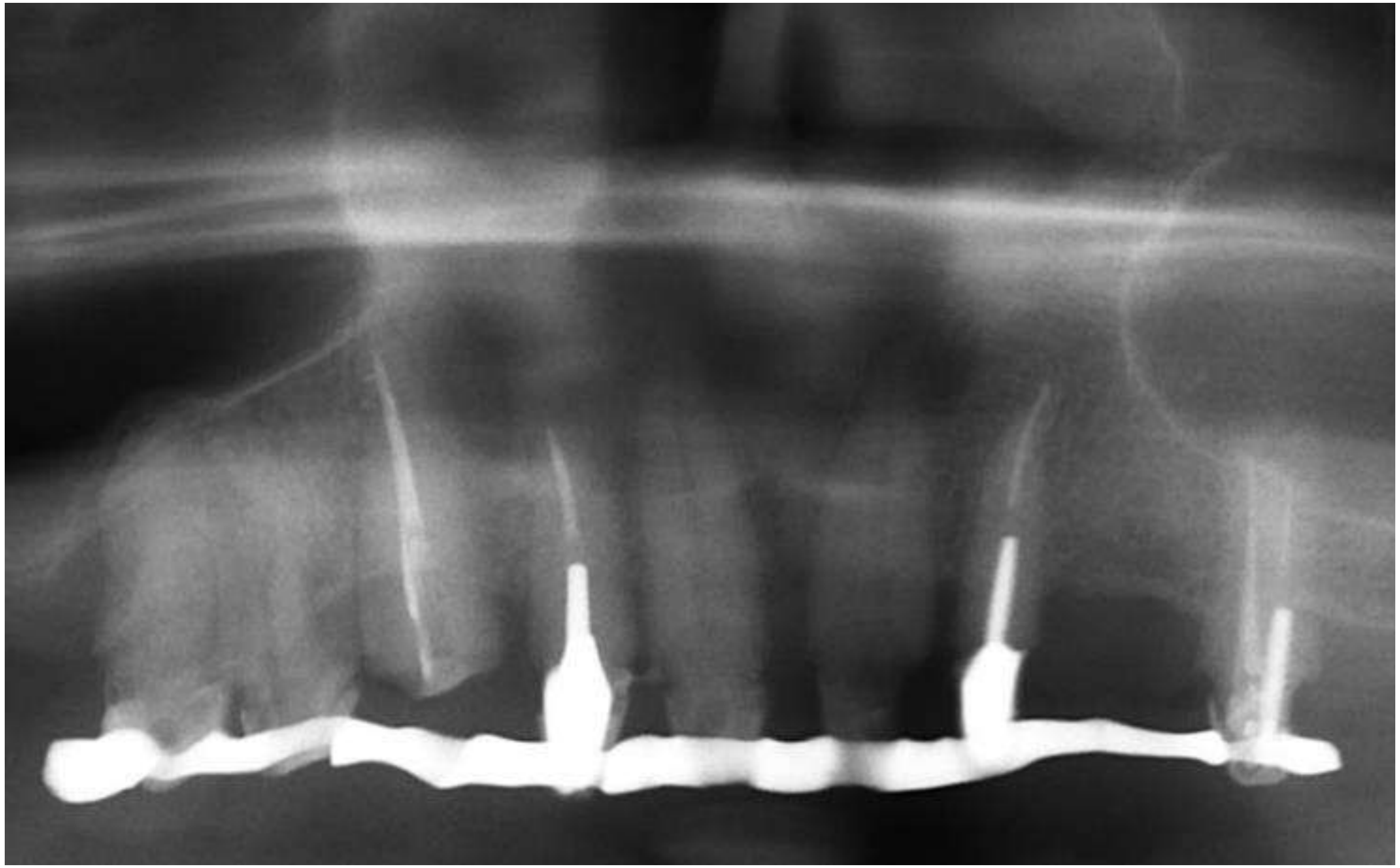

K9 – HE x4

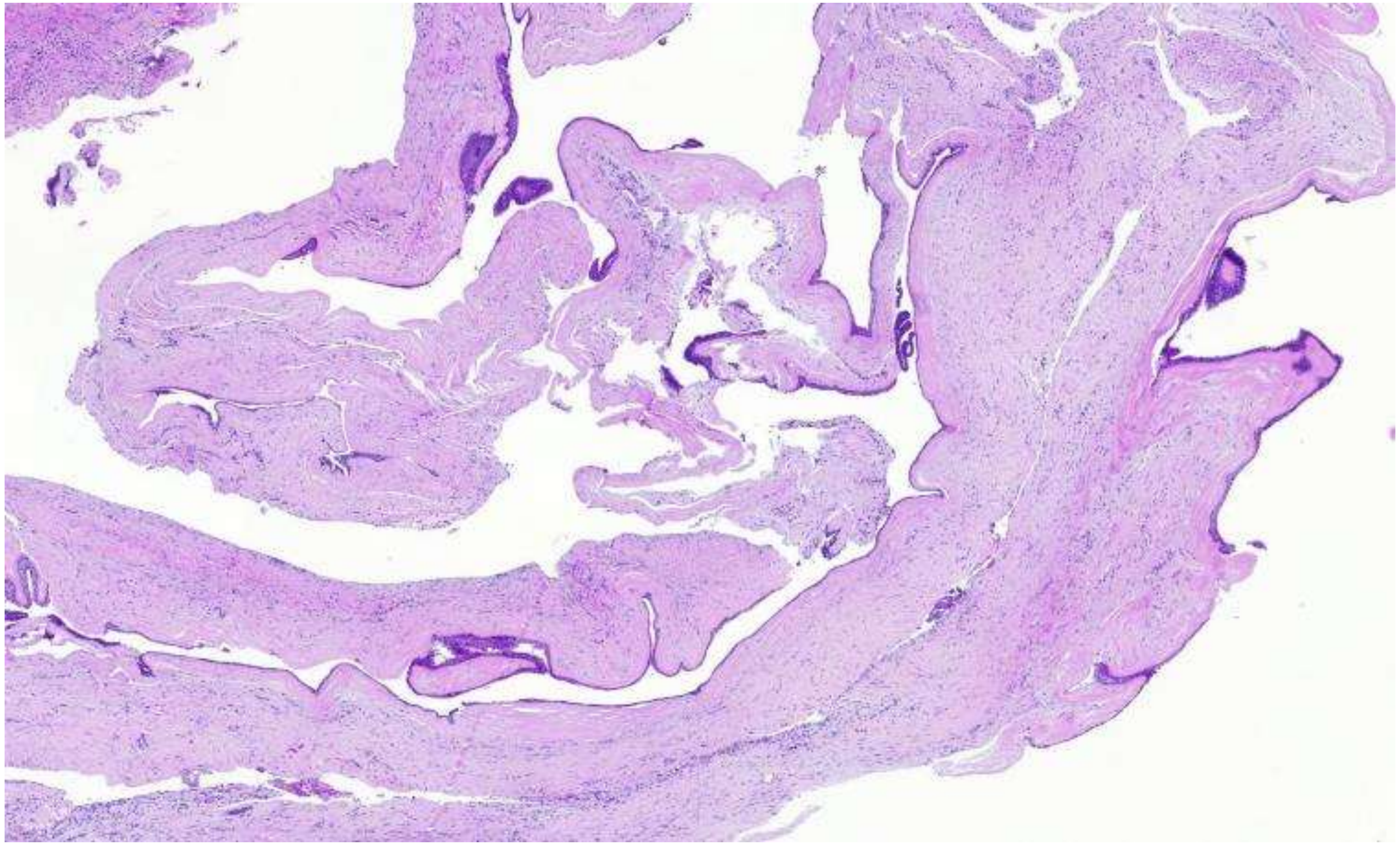

K9 – HE x10

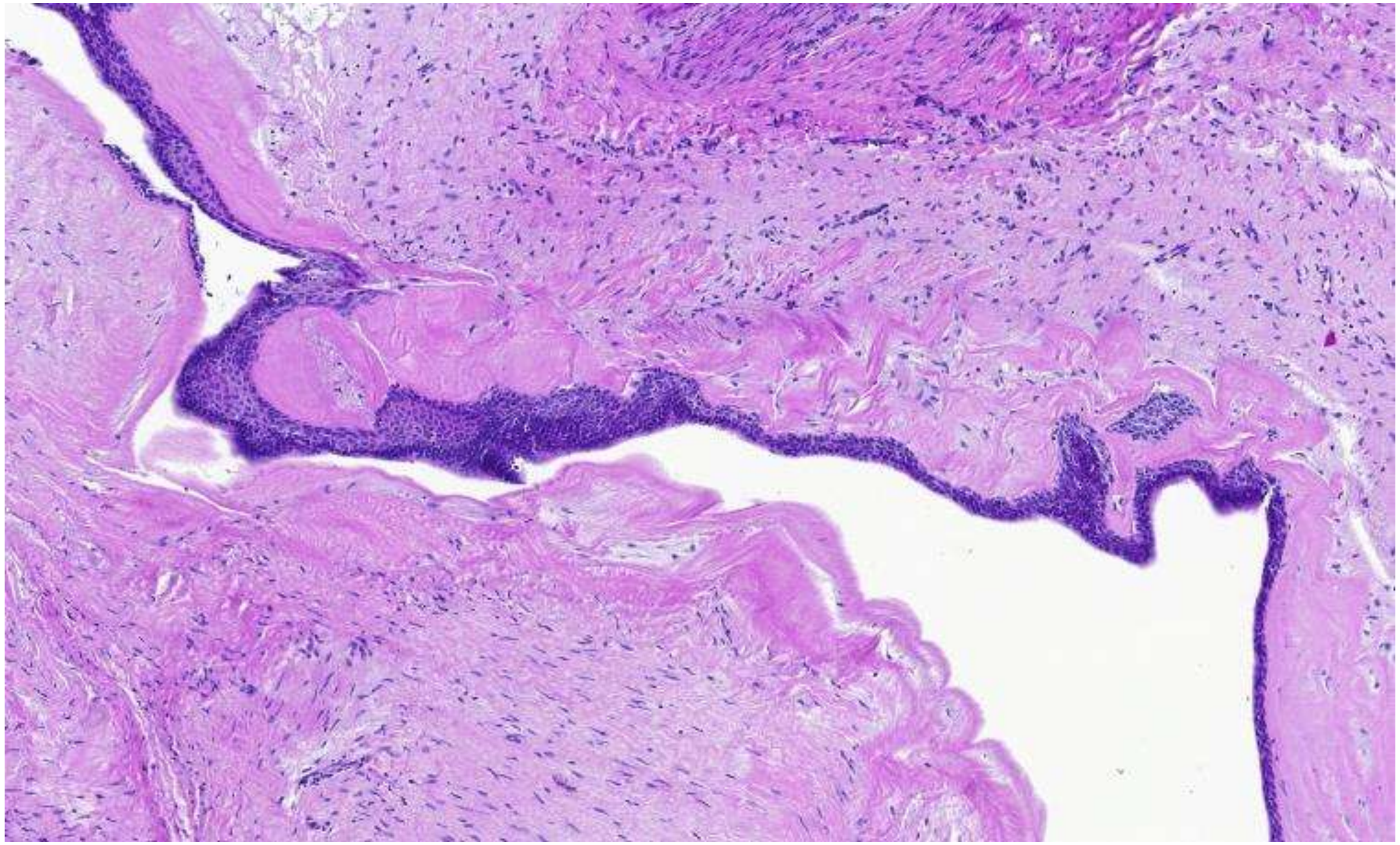

K9 – HE x15

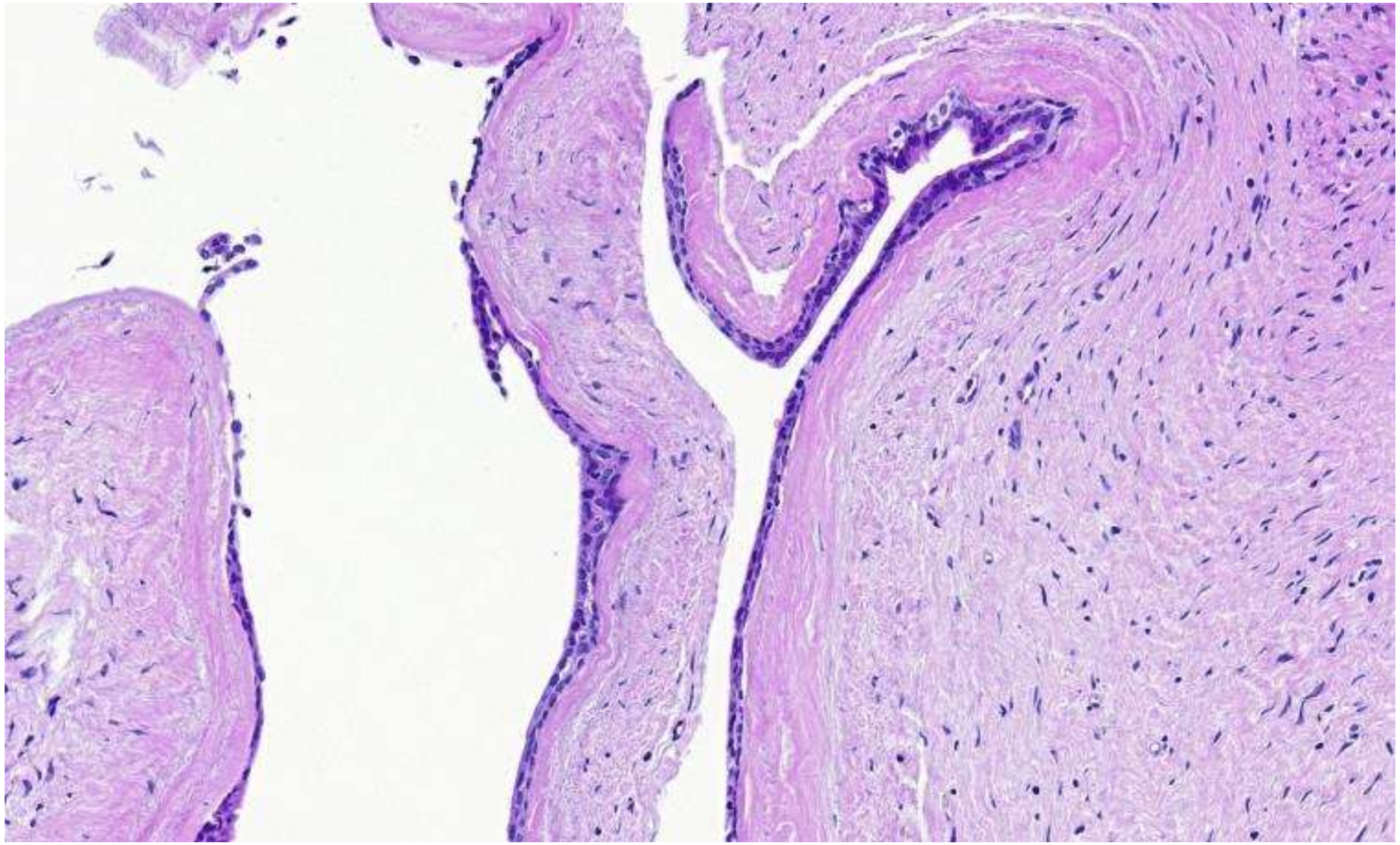

K9 – HE, CK17, bcl2 (x1)

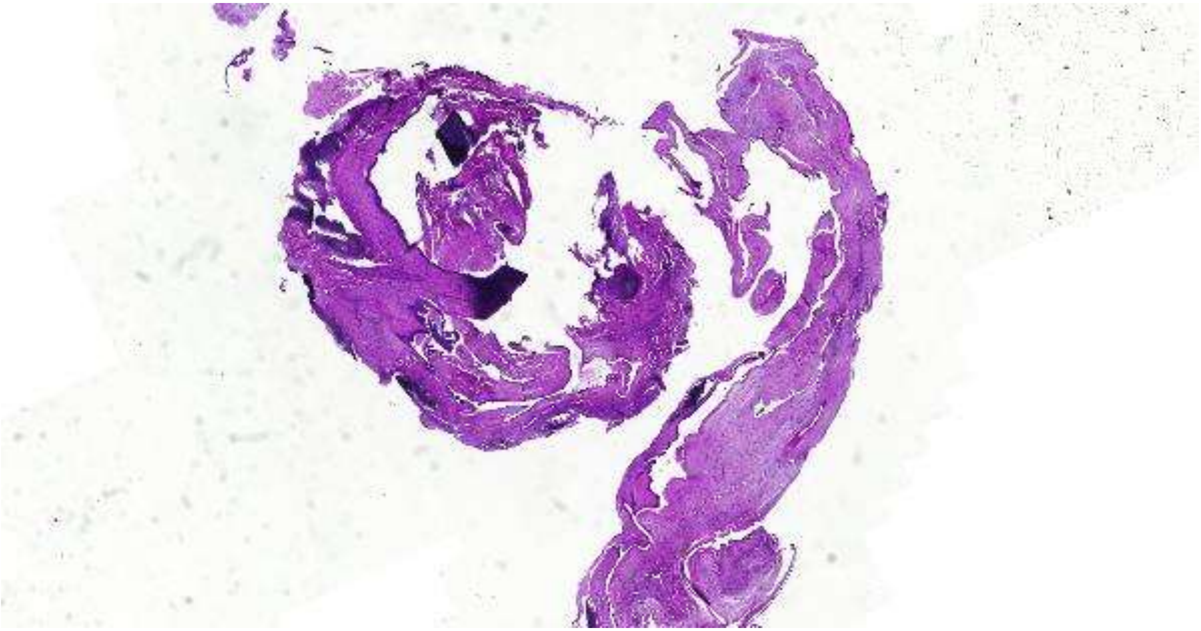

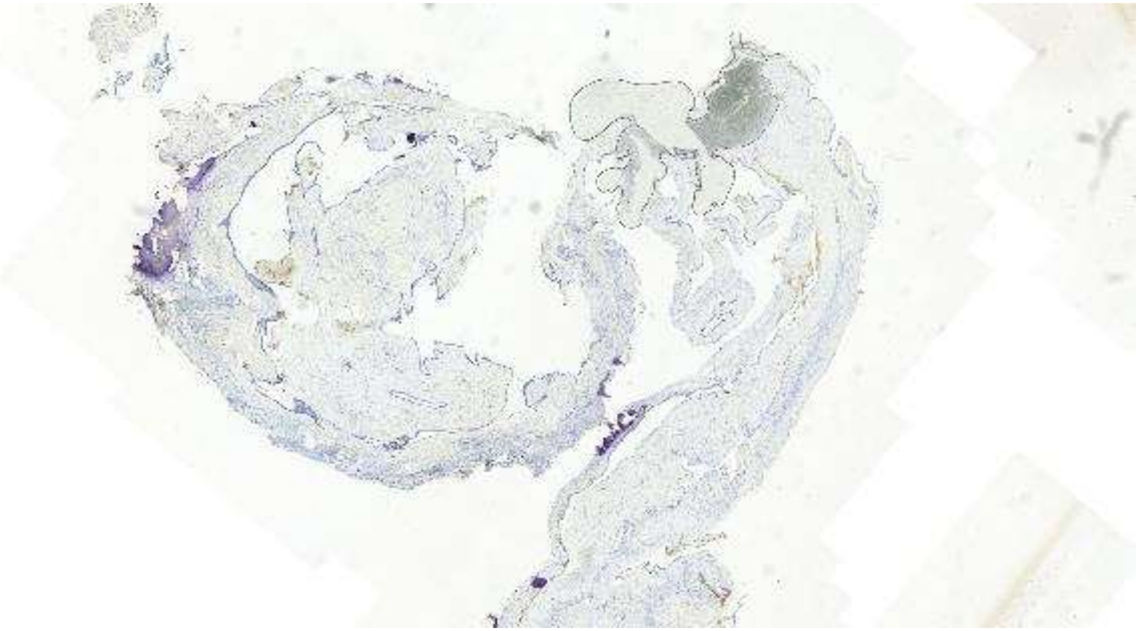

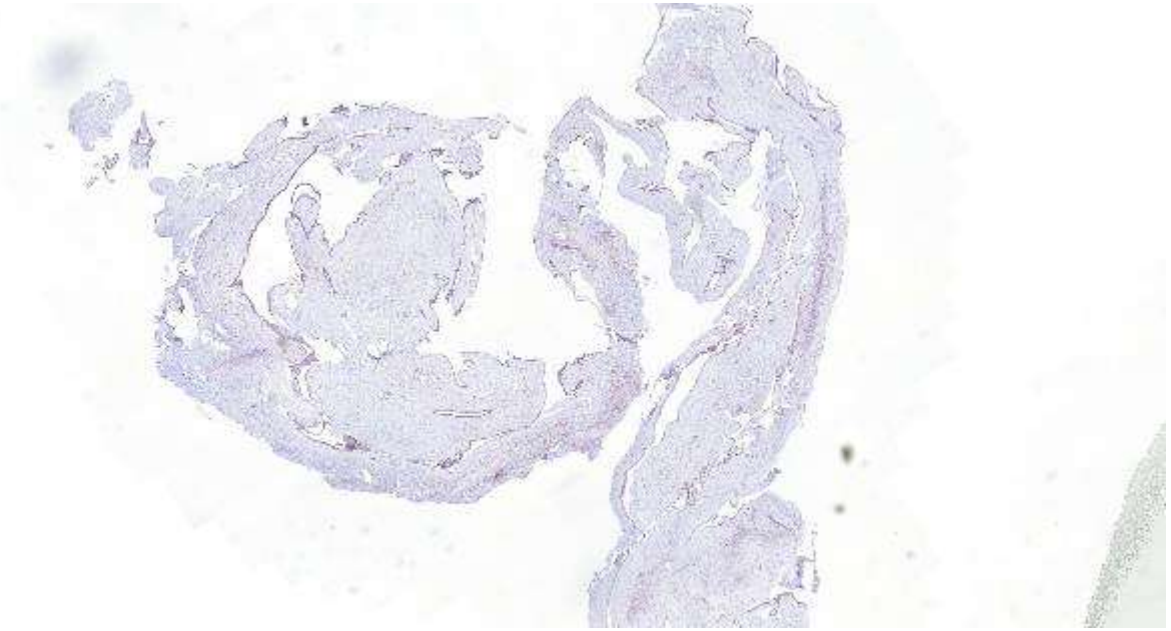

K9 – HE, CK17, bcl2 (x10)

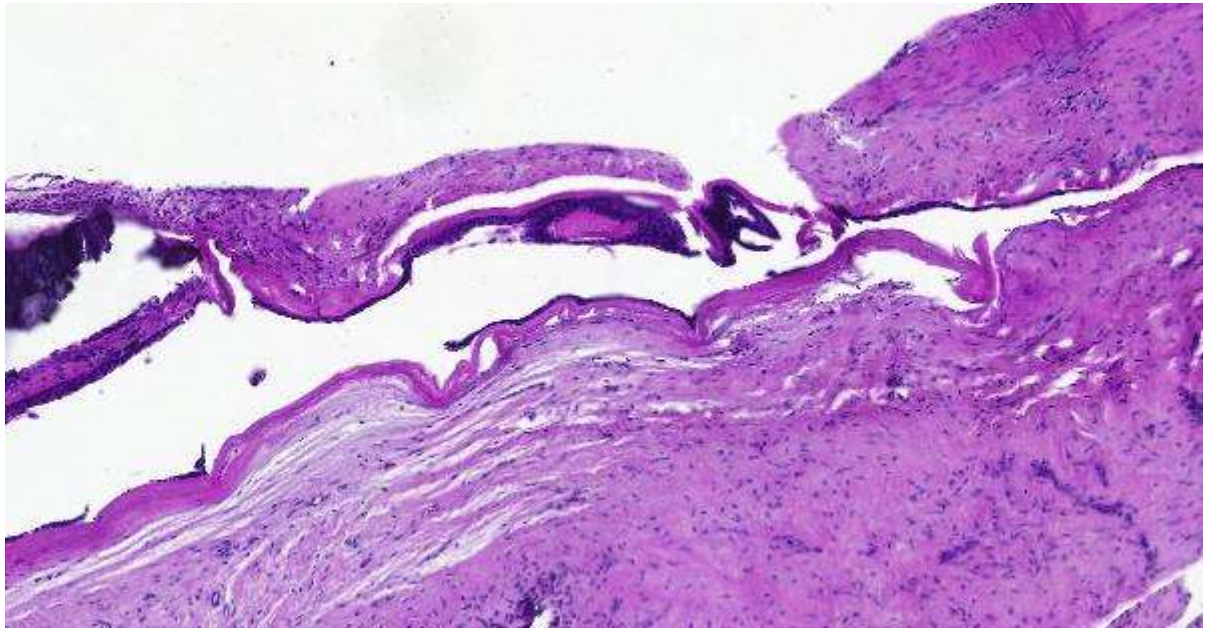

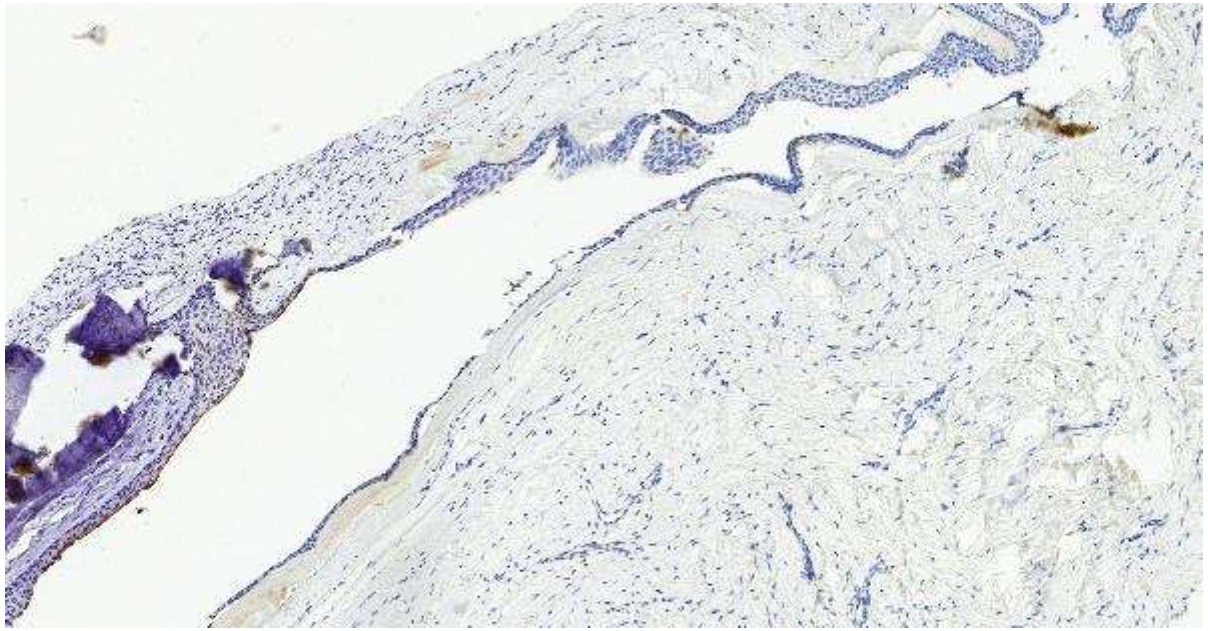

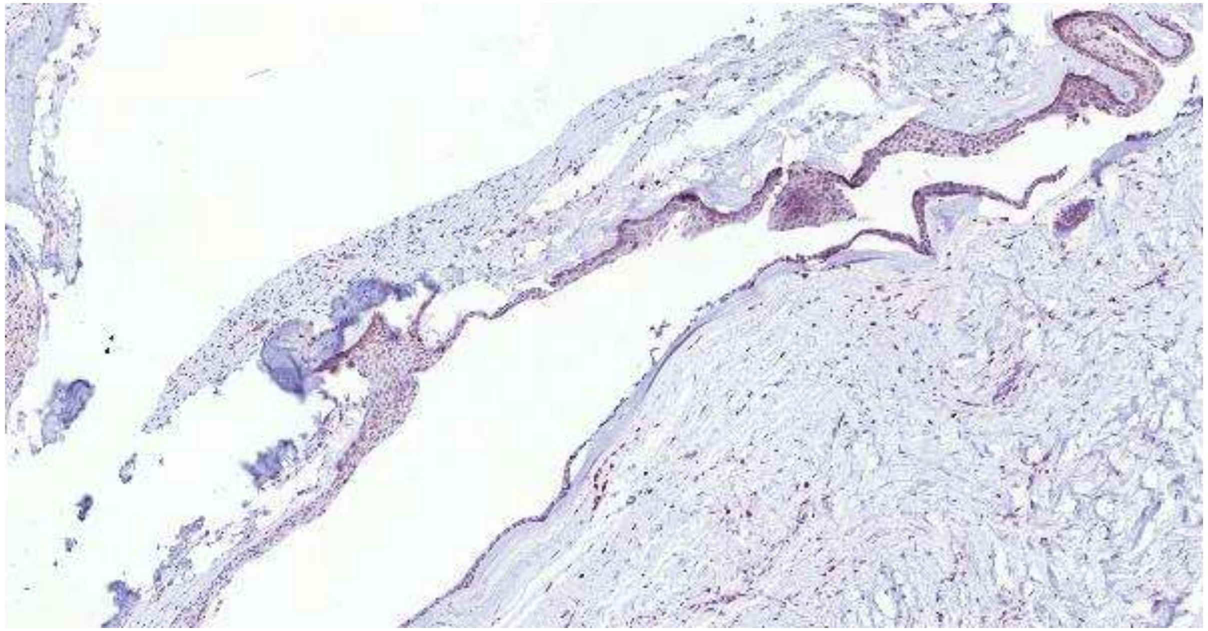

K9 – CK17, bcl2 (x10)

focal transepithelial CK17 positivity (↑) – weak focal basal bcl2 positivity (↑)

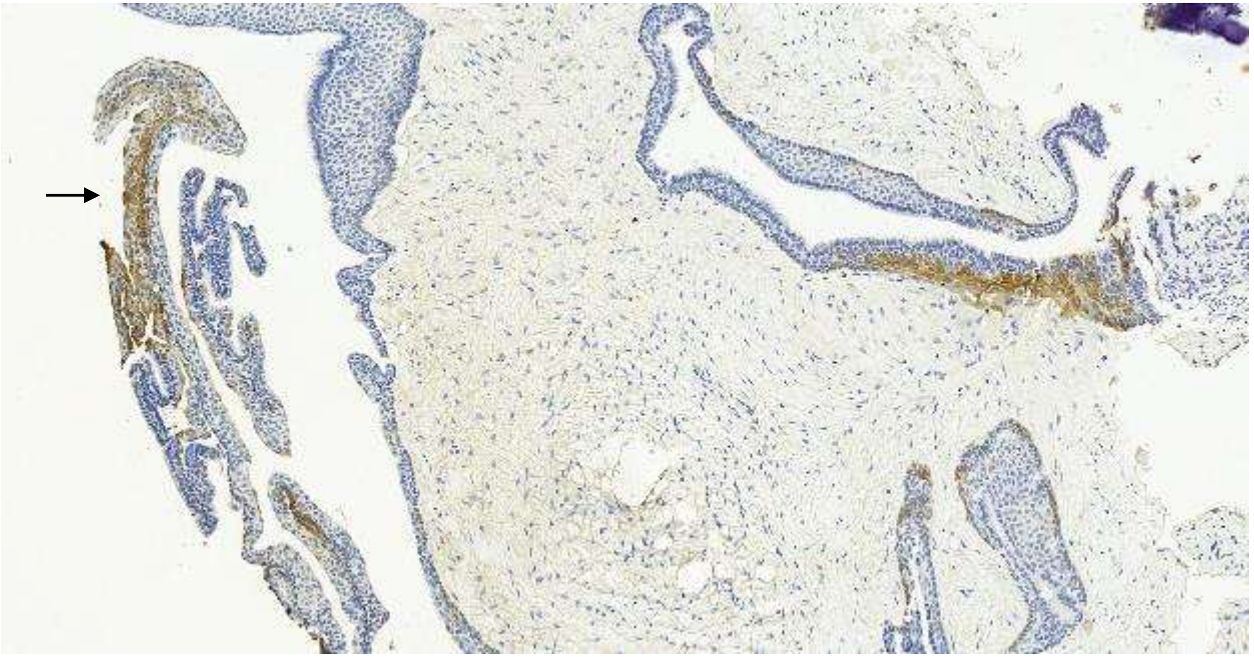

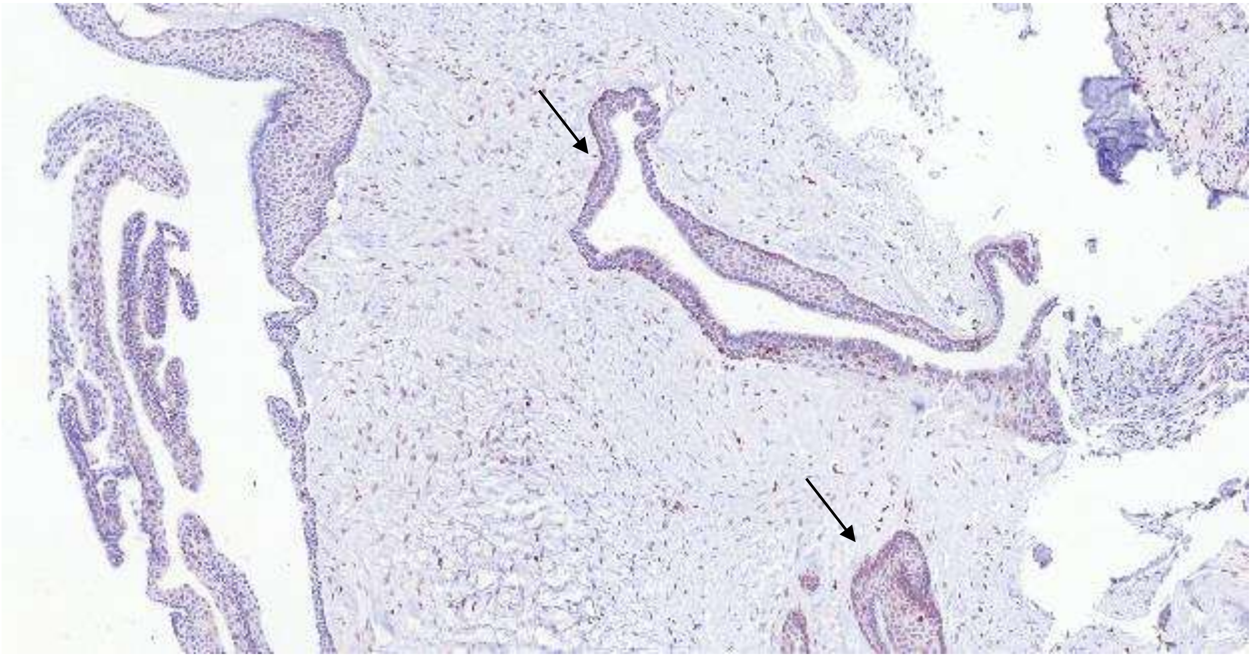

K9 – CK17, bcl2 (x20)

focal superficial CK17 staining (↑); weak basal bcl2 staining

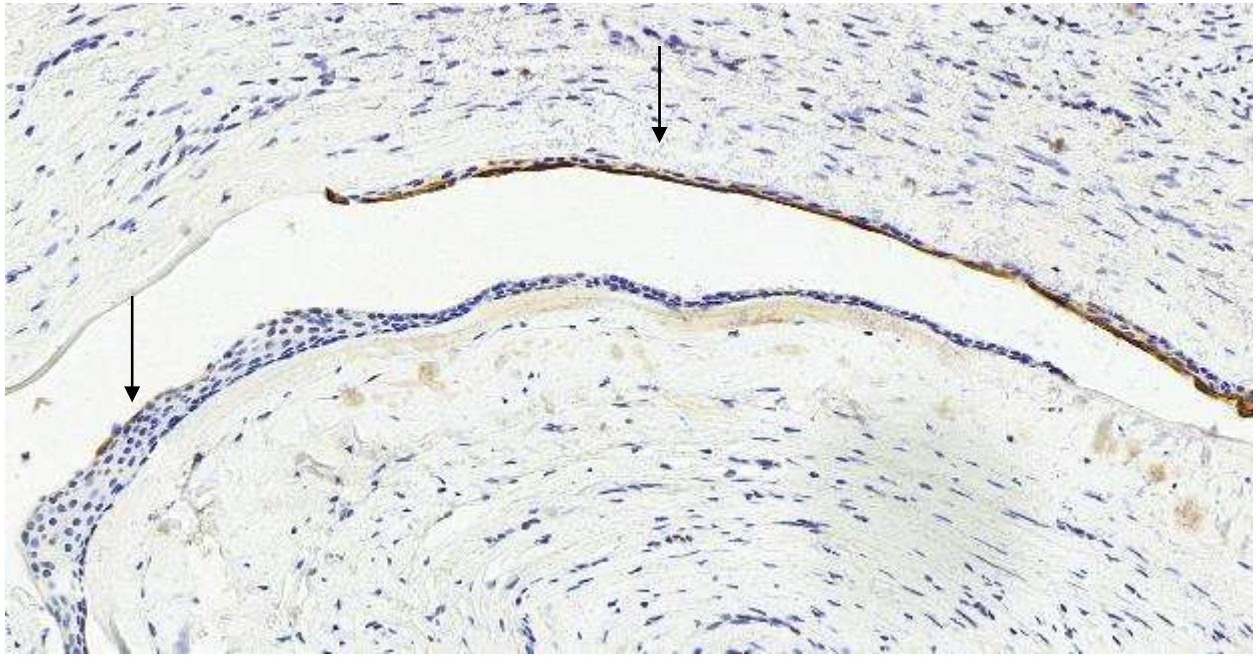

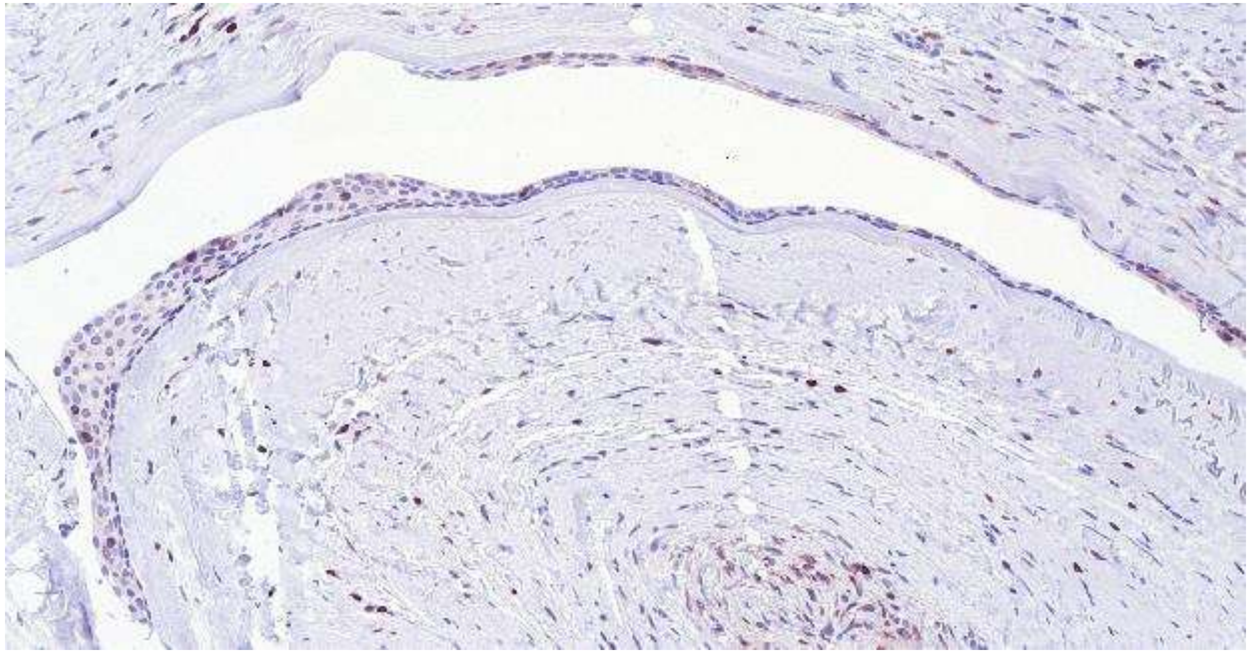

K9 – bcl2 (x40) - weak basal bcl2 staining

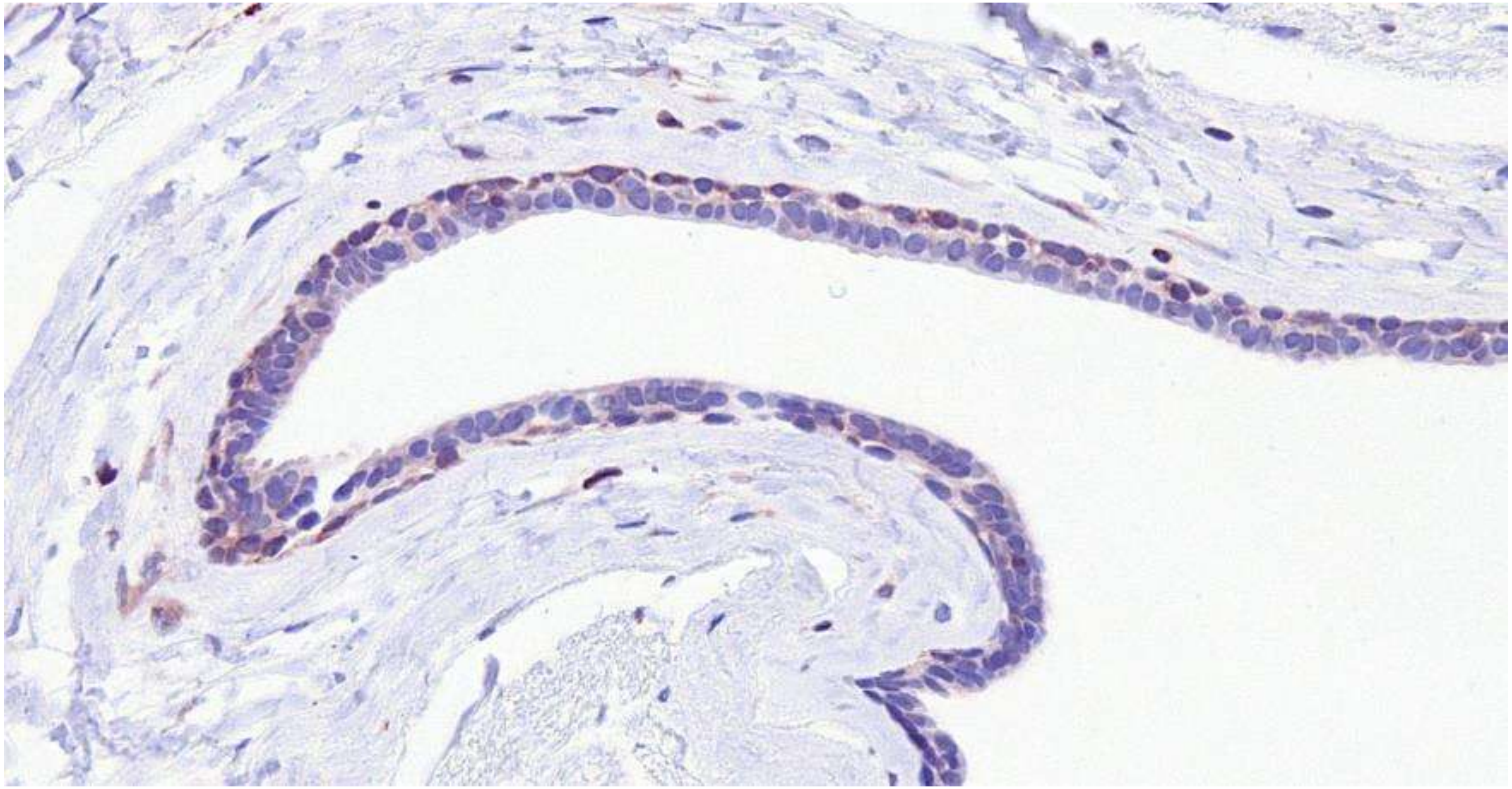

K9 – Conclusion

- H&E: relatively thin (2-5 cells) squamous epithelium with focal thickening (up to 10 cells), no parakeratosis, no wavy surface, no basal palisading, even epithelial-subepithelial contours
- No inflammation of the cyst wall
- Bcl2: negative, but areas with staining of flat basal cells
- CK17: mainly negative, focally superficial & focally transepithelial positivity
- Histopathology: original diagnosis: maxillary cyst N.O.S.; revised diagnosis: botryoid odontogenic cyst
- Combined clinical and pathological opinion: botryoid odontogenic cyst

Case K10

- 28-year-old male
- Dental hygiene/status: normal; partly missing, partly replaced, treated; vestibular mucosa in area of tooth 12 hyperaemic and painful on pression
- Radiology: Radiolucent lesion at the apex of filled teeth 13-21
- Operation: removal of the cyst with resection of root apex of teeth 13-21
- Clinical diagnosis: maxillary cyst (periapical cyst mentioned elsewhere in the charts)

K10 – OPT

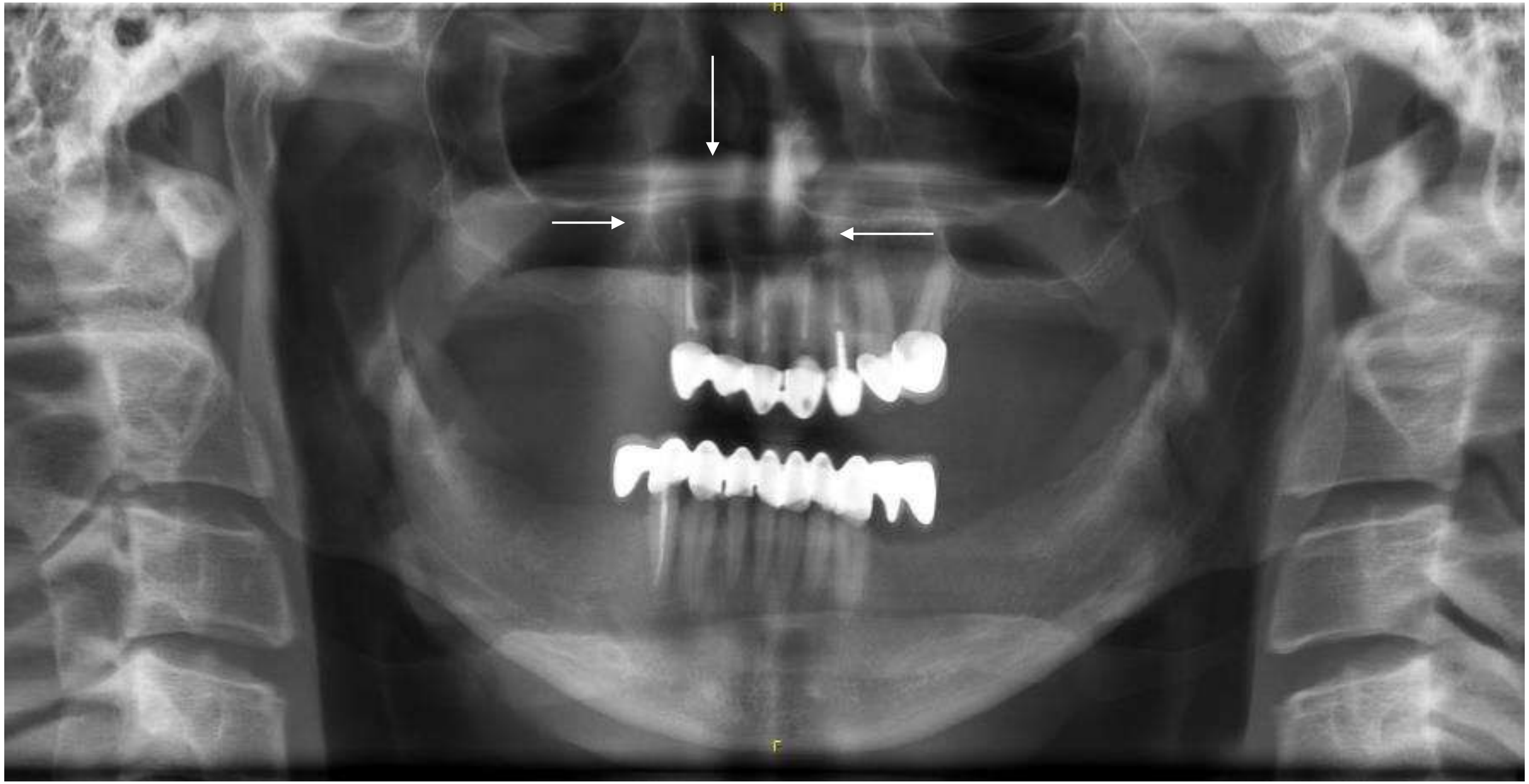

K10 – HE, CK17, bcl2 (x1.8)

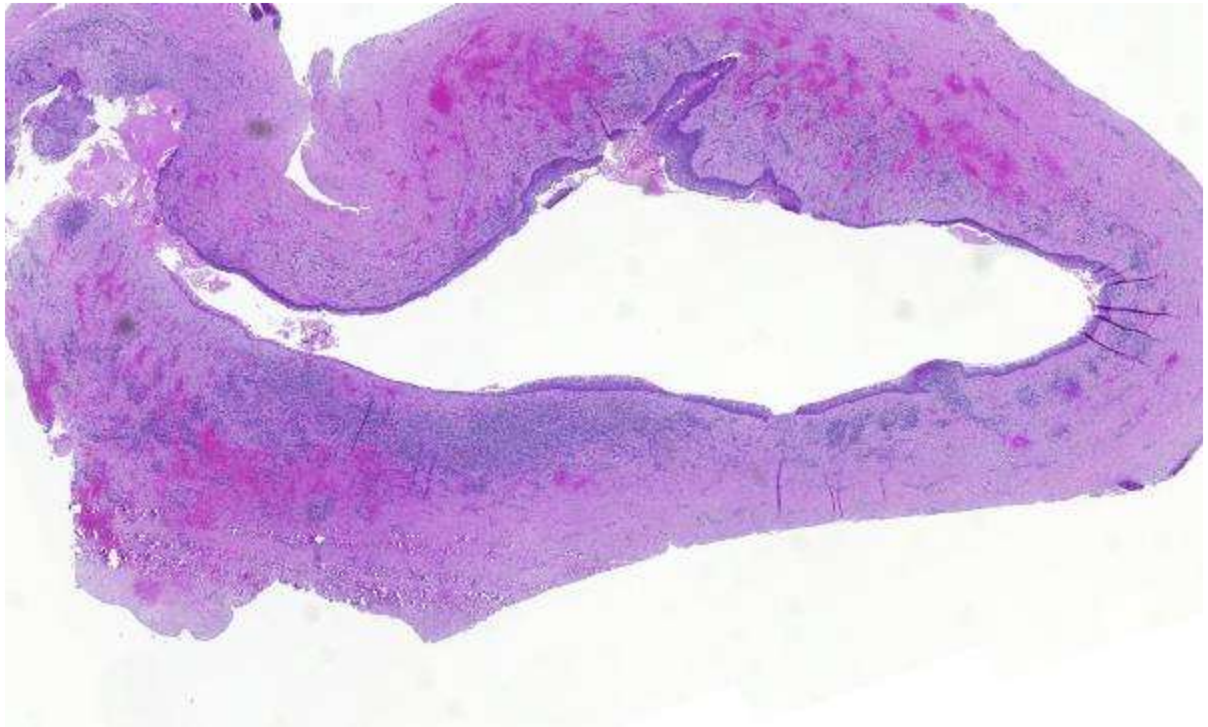

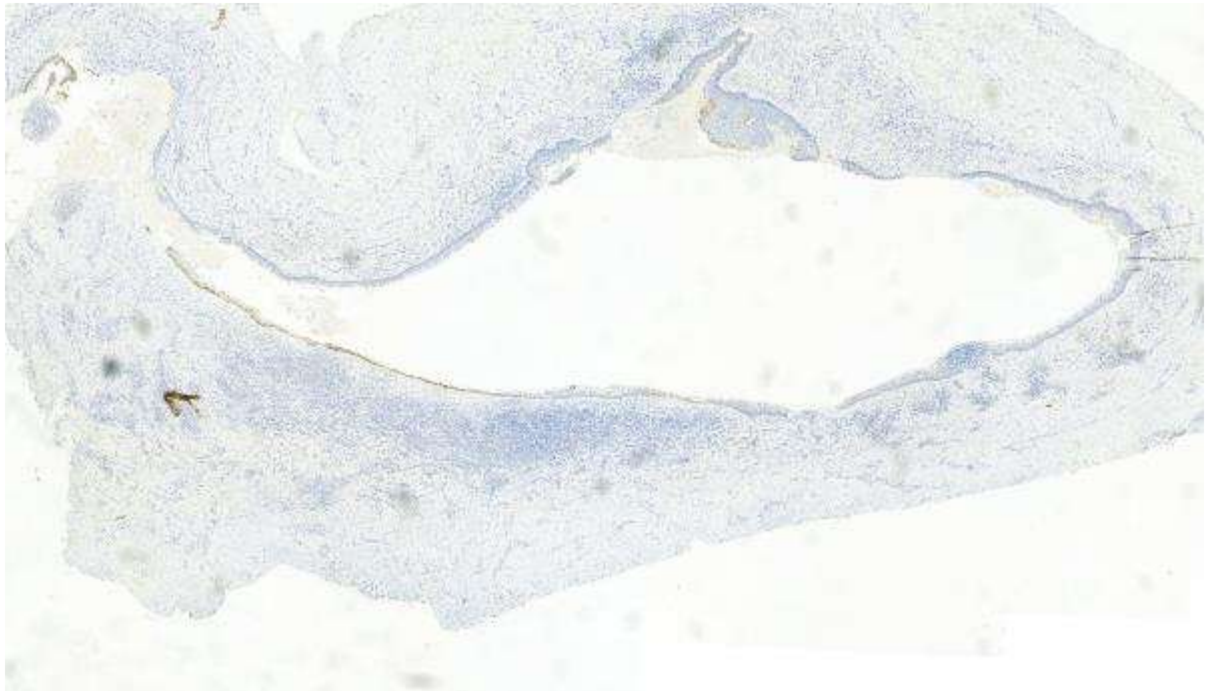

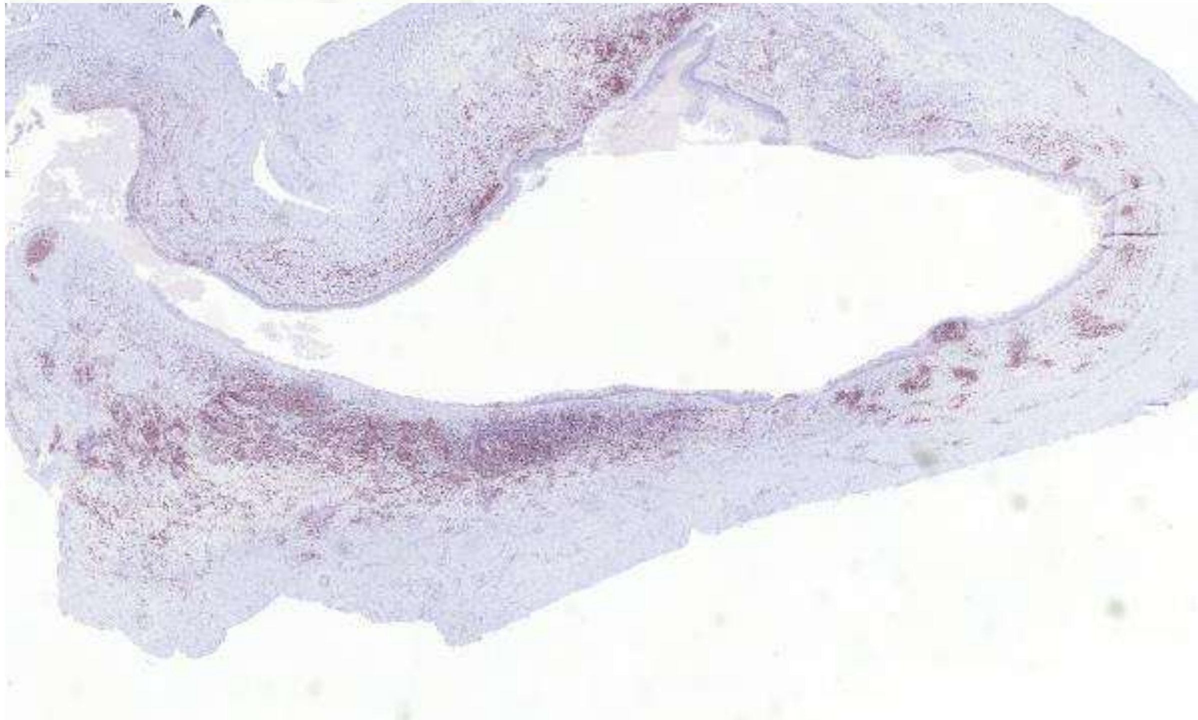

K10 – HE, CK17, bcl2 (x10)

Focal transepithelial CK17 staining (↑),
negativity of mucinous metaplasia (*)

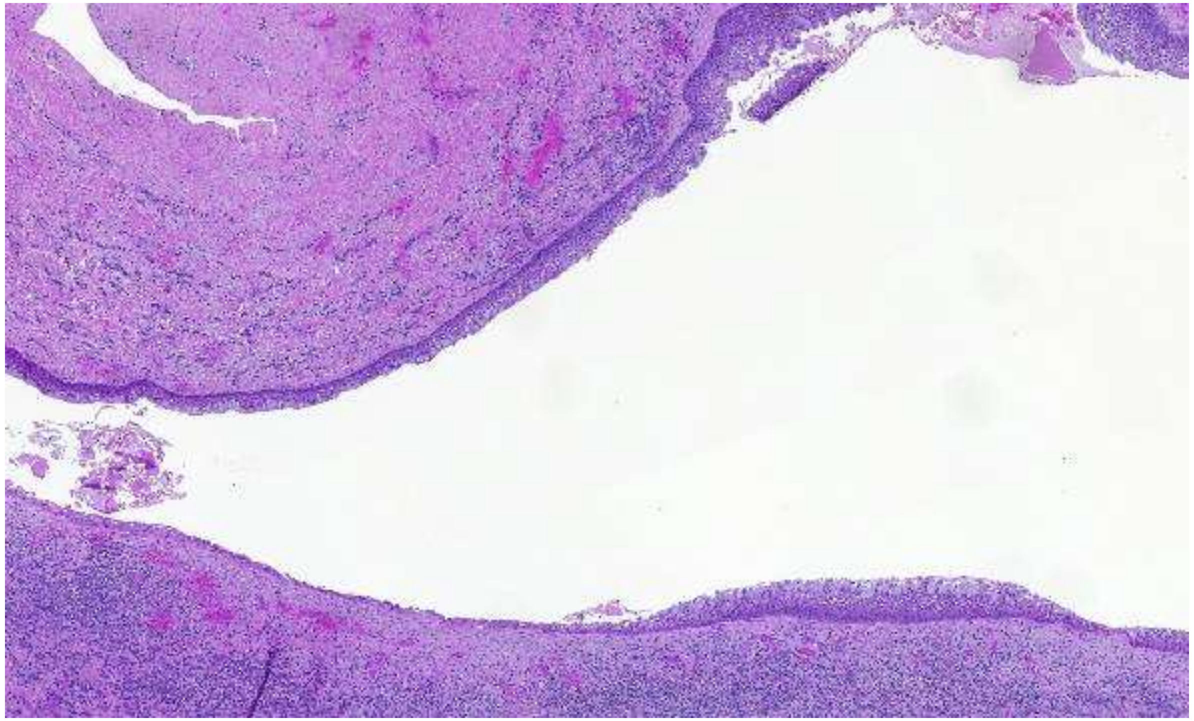

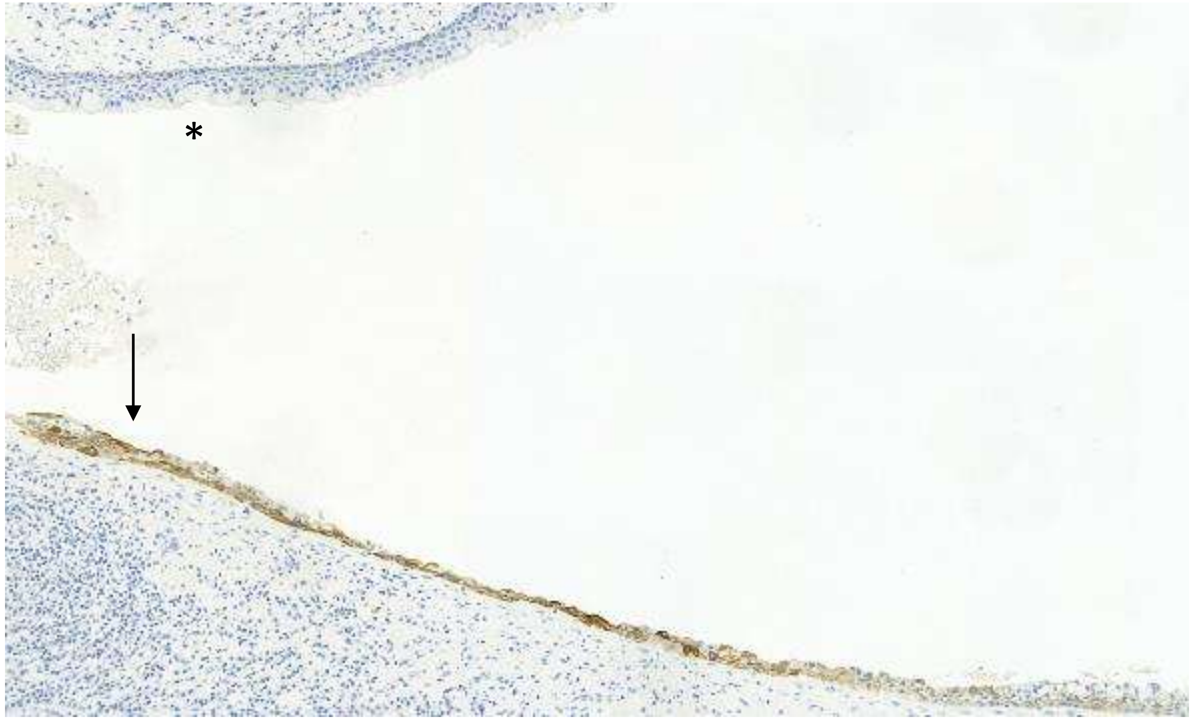

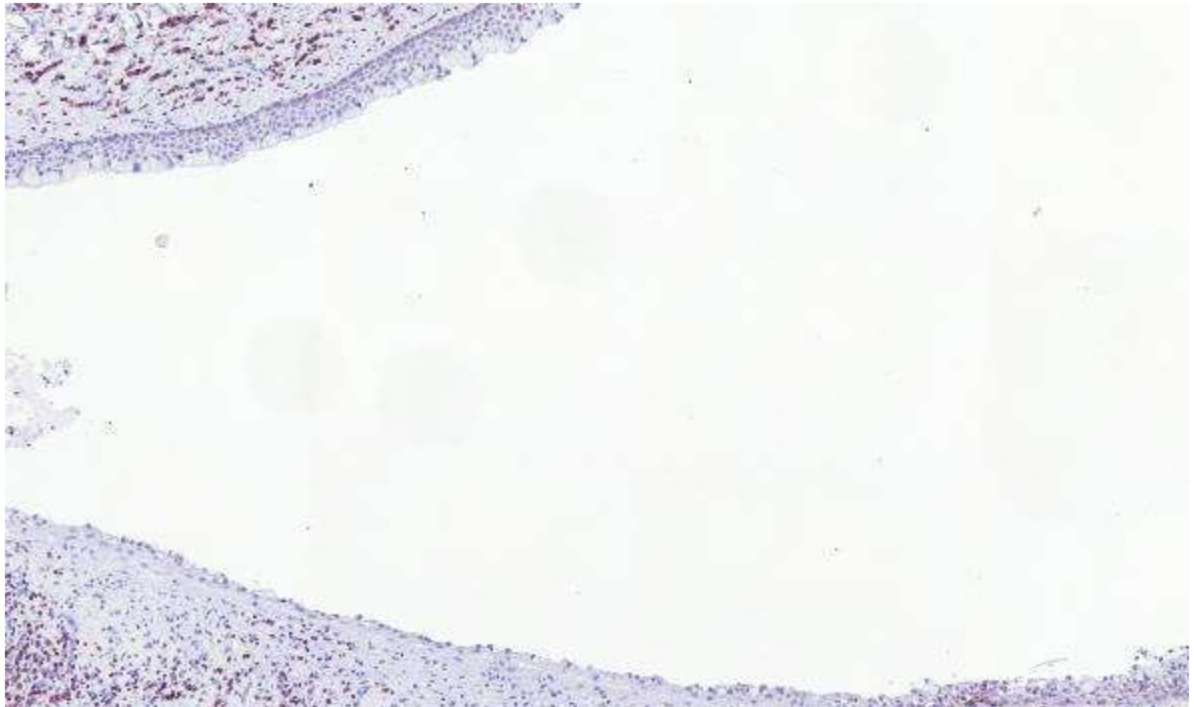

K10 – HE, CK17, bcl2 (x12)

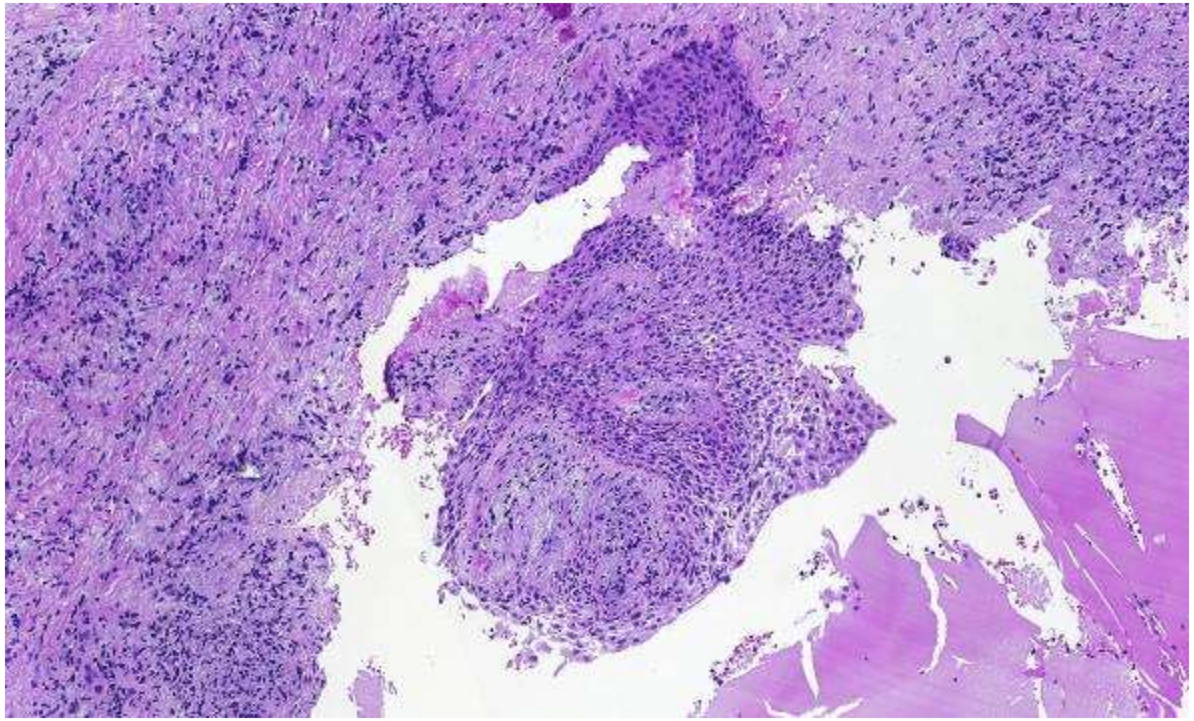

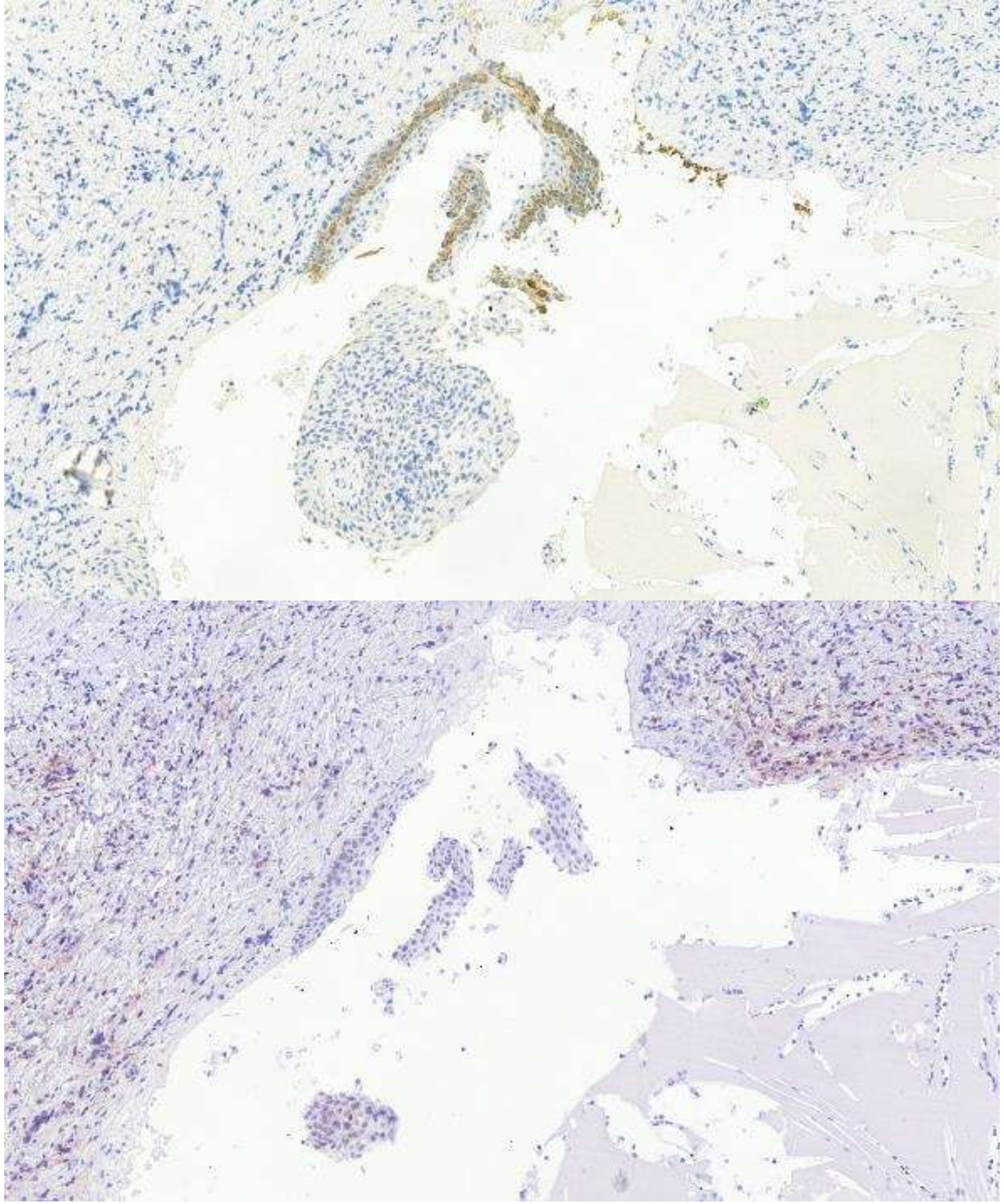

K10 – HE, CK17, bcl2 (x10)

Focal superficial CK17 positivity sparing mucinous cells (↑)

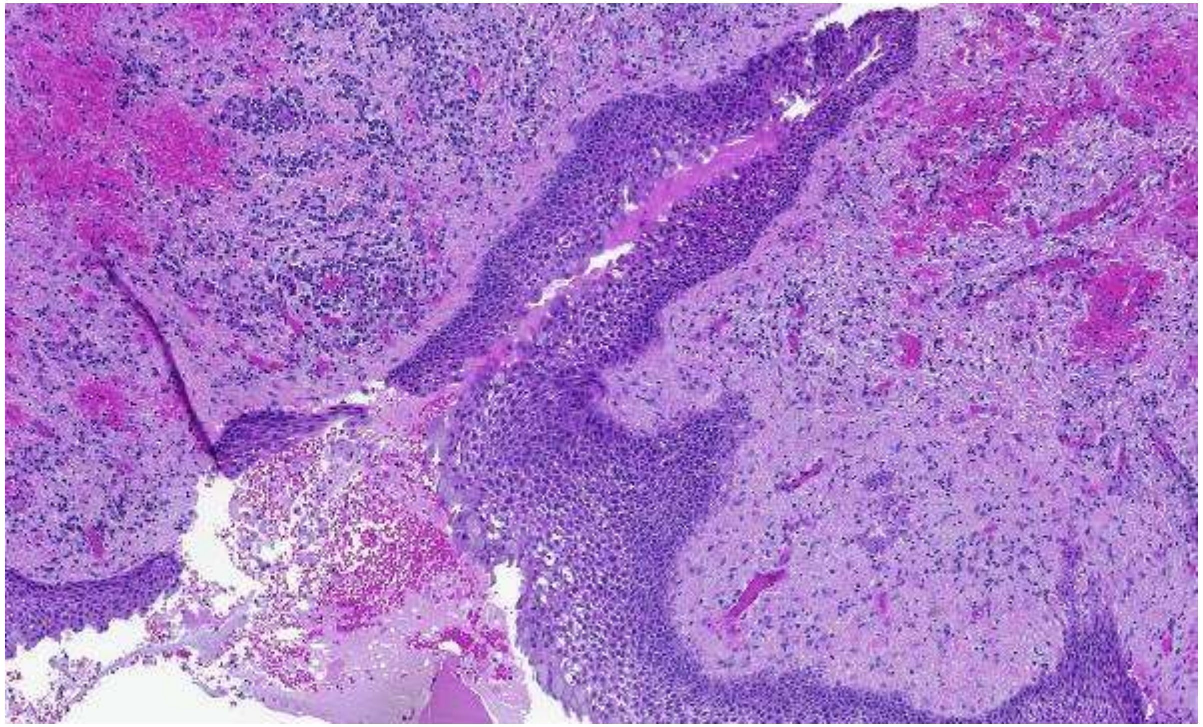

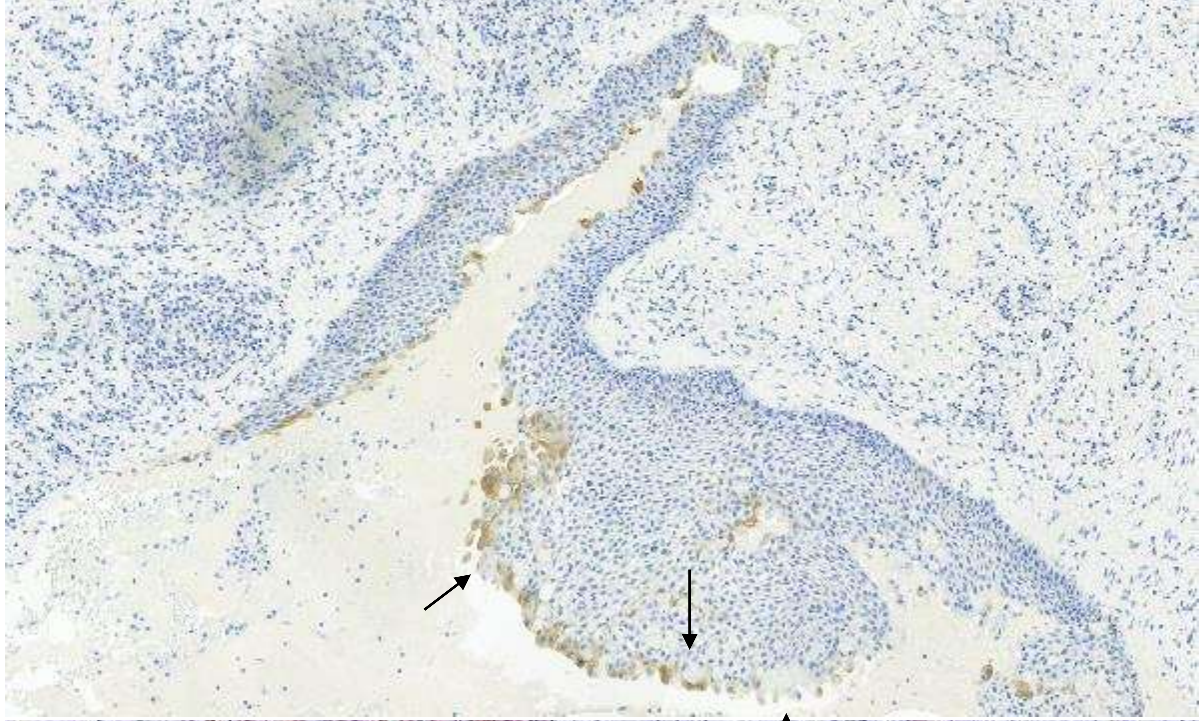

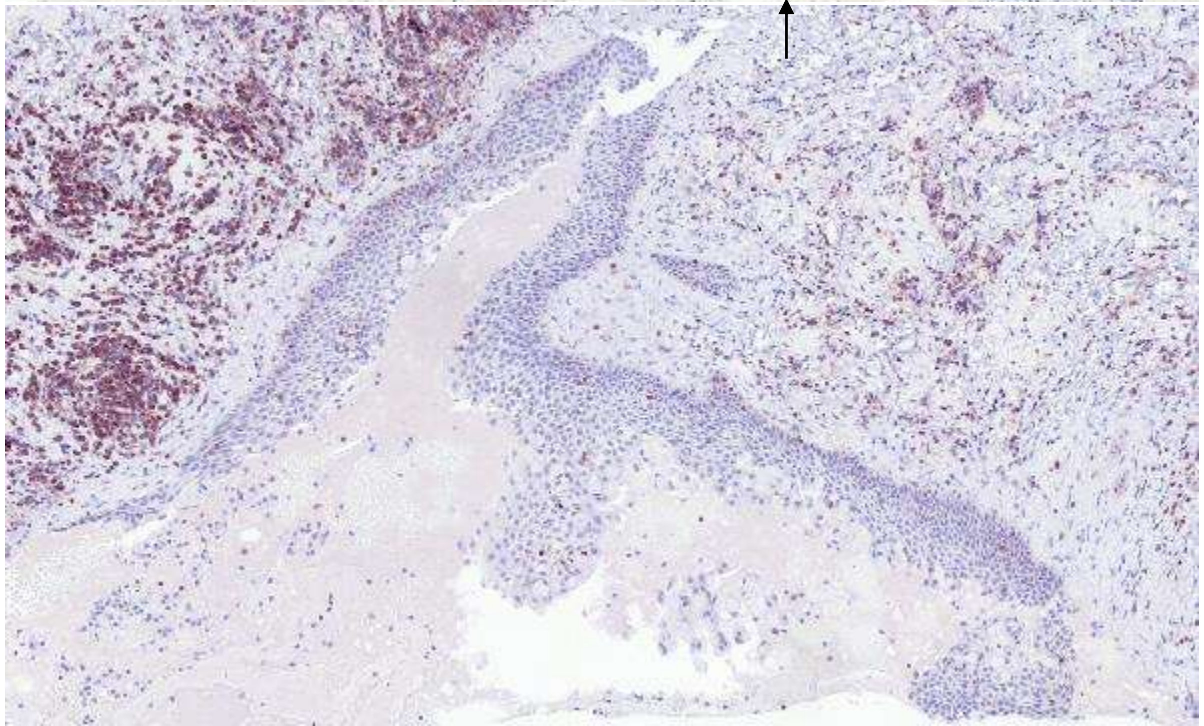

K10 – Conclusion

- H&E: relatively thick squamous epithelium, no parakeratosis, no wavy surface, no basal palisading, even epithelial-subepithelial contours; respiratory epithelium and goblet cell metaplasia
- Chronic inflammation of the cyst wall
- Bcl2: negative
- CK17: dominantly negative, focally basal and minor area with transepithelial positivity
- Histopathology: inflammatory cyst (N.O.S.)
- Combined clinical and pathological opinion: radicular cyst

Case K11

- 56-year-old female (cyst 1 of the same patient as Case K12)
- Dental hygiene/status: partly missing, partly replaced teeth, removable upper denture, mobile bridge over teeth 33, 32 and 42, 43 – all with probable pouches; vestibular mucosa in area of tooth 12 hyperaemic and painful on pression
- Radiology: 1x1 cm large radiolucency around the apex of dental root 42 also involving the root of tooth 43…
- Operation: removal of the bridge, residual movable roots and both cysts
- Clinical diagnosis: mandibular cyst of region of teeth 42-43

K11 – OPT1

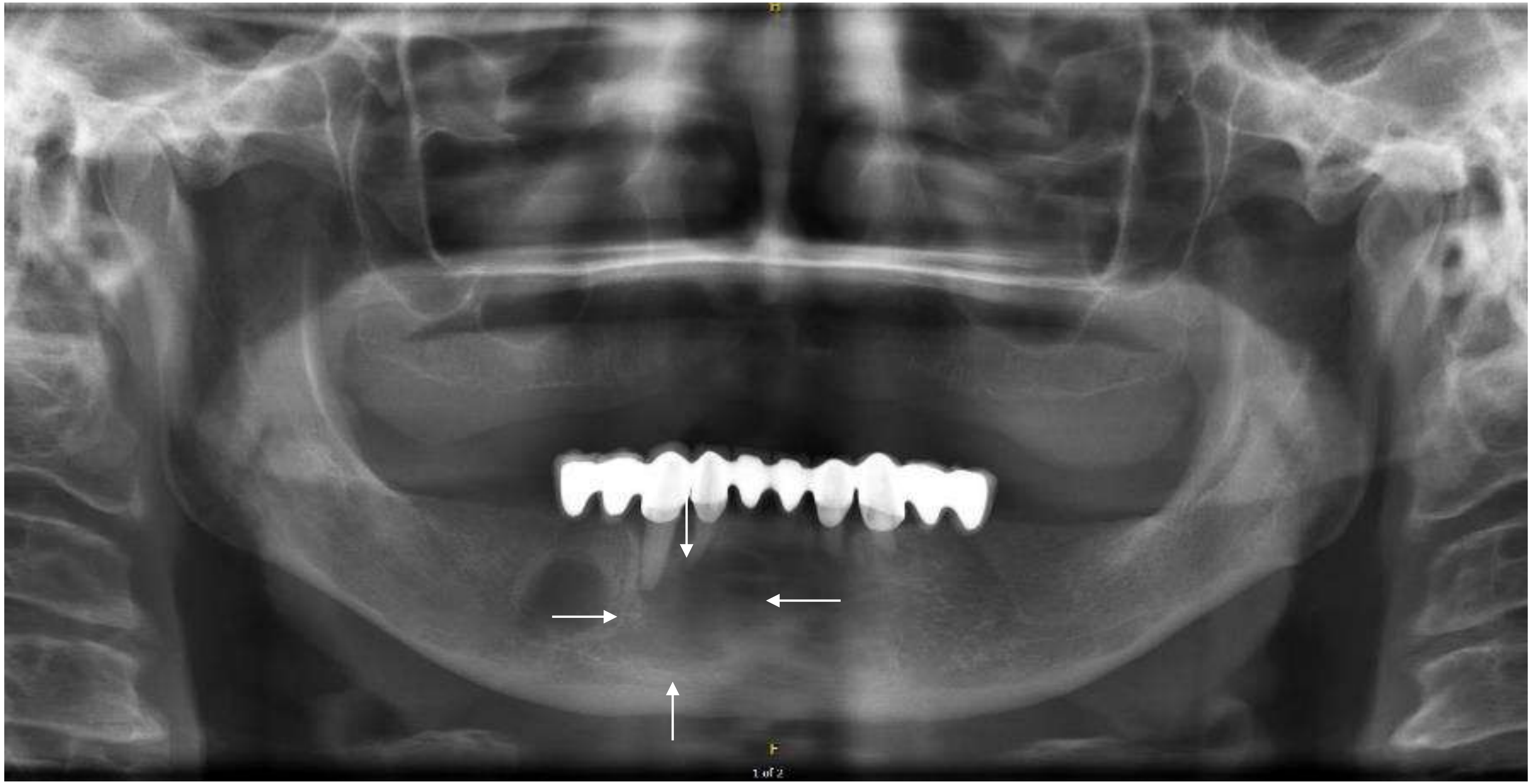

K11 – OPT2

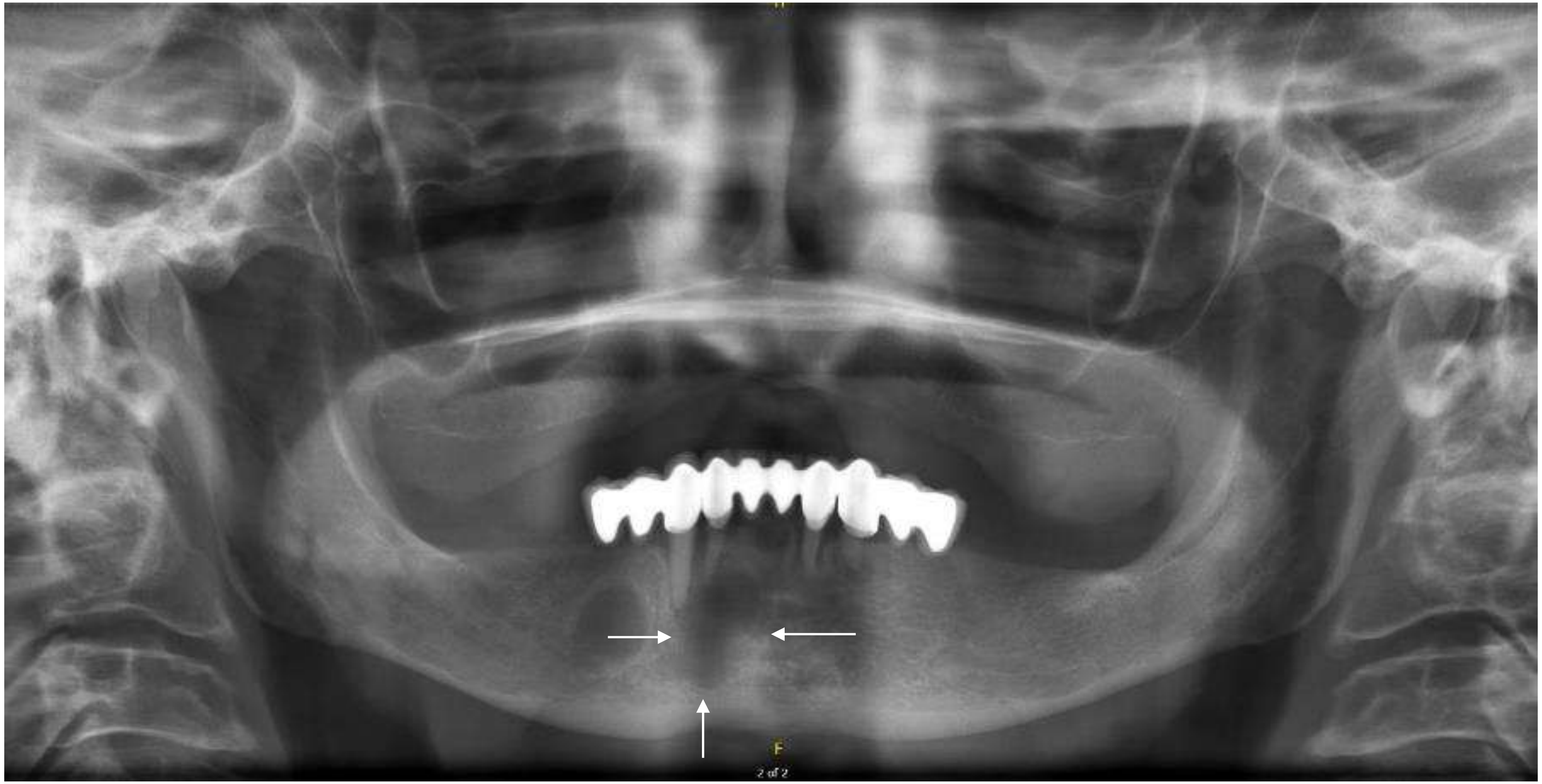

K11 – HE, CK17, bcl2 (x1)

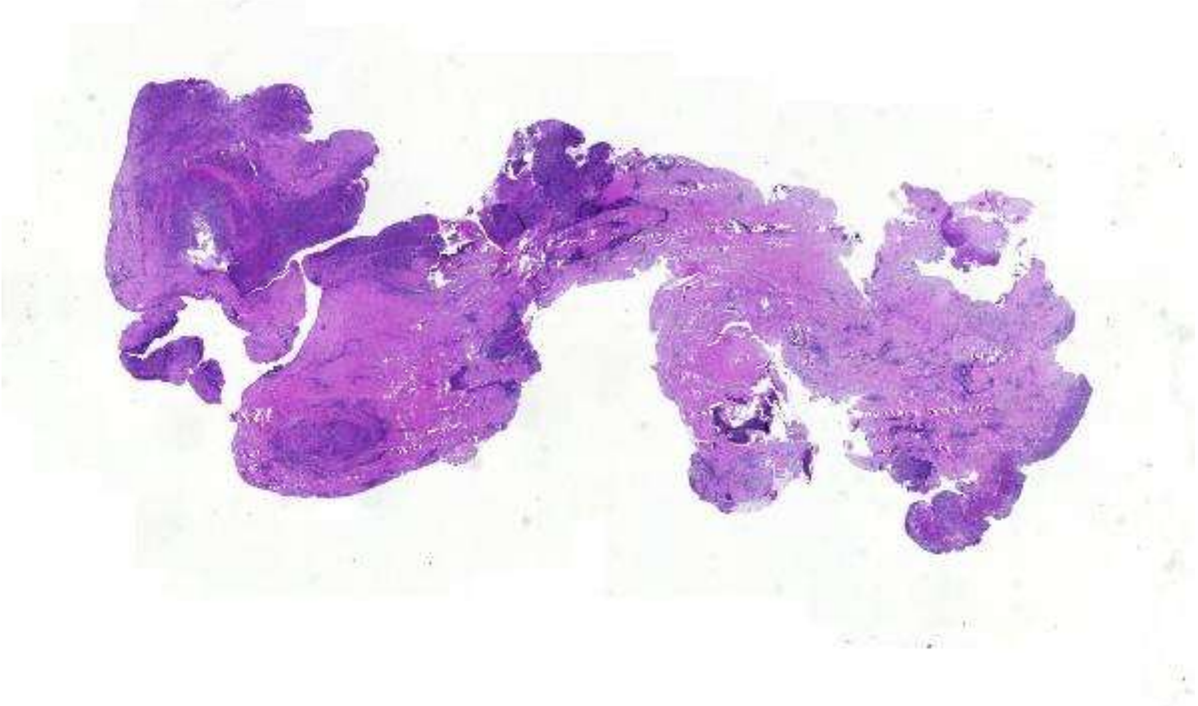

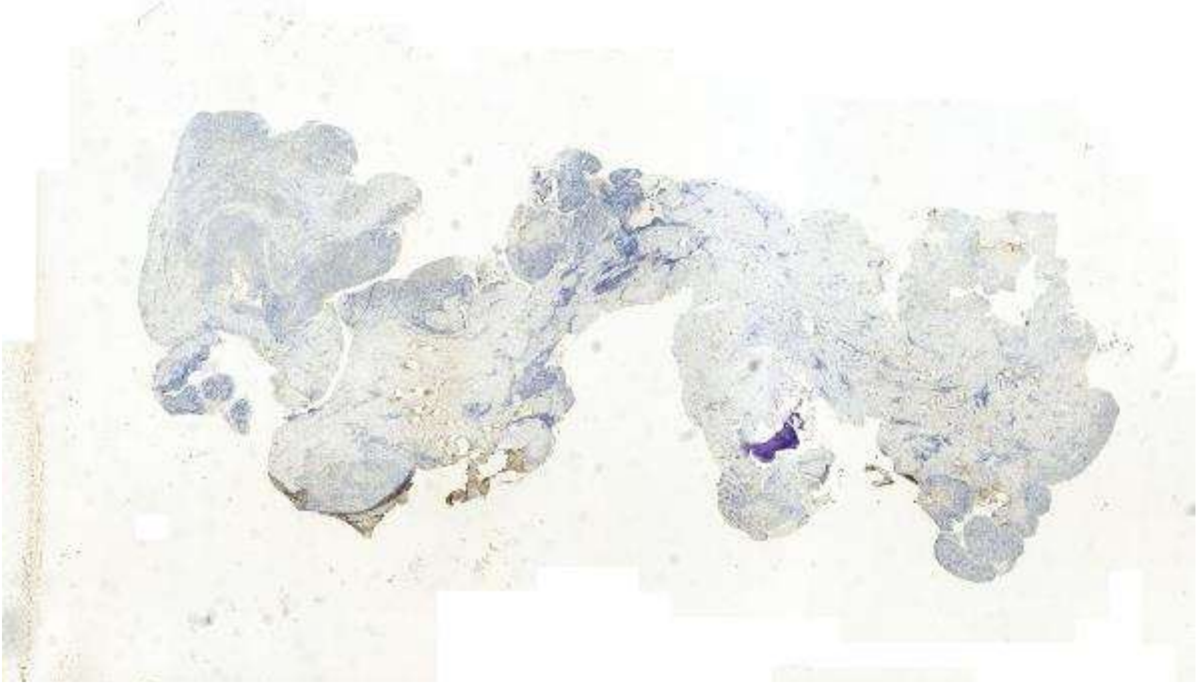

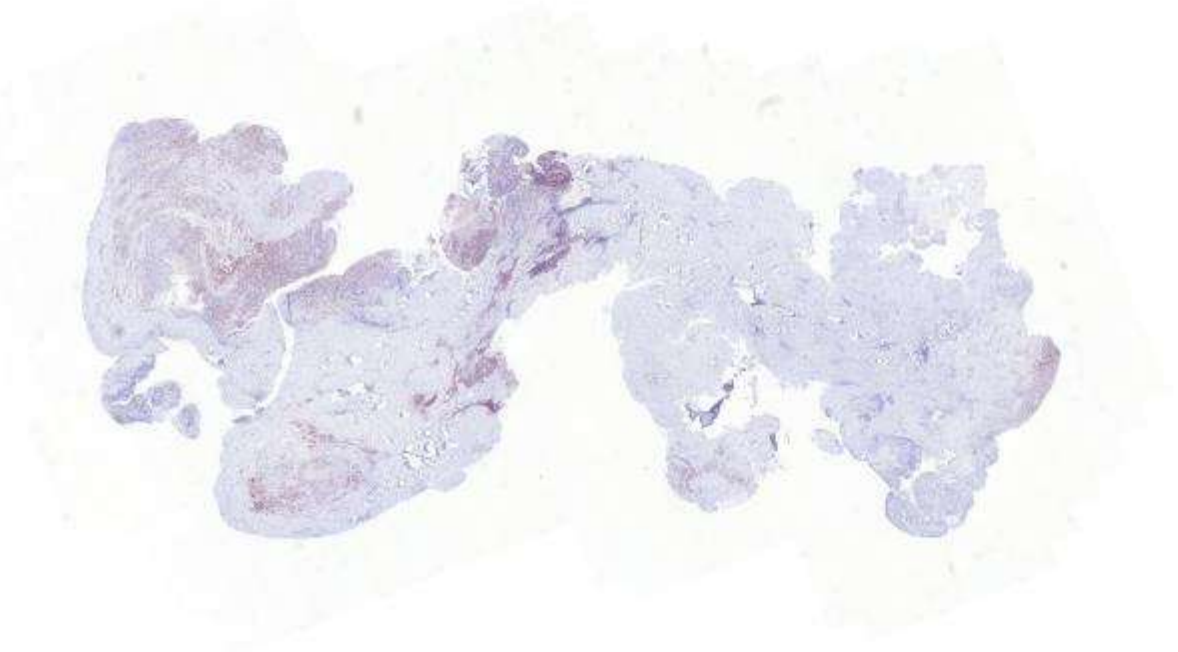

K11 – HE, CK17, bcl2 (x5)

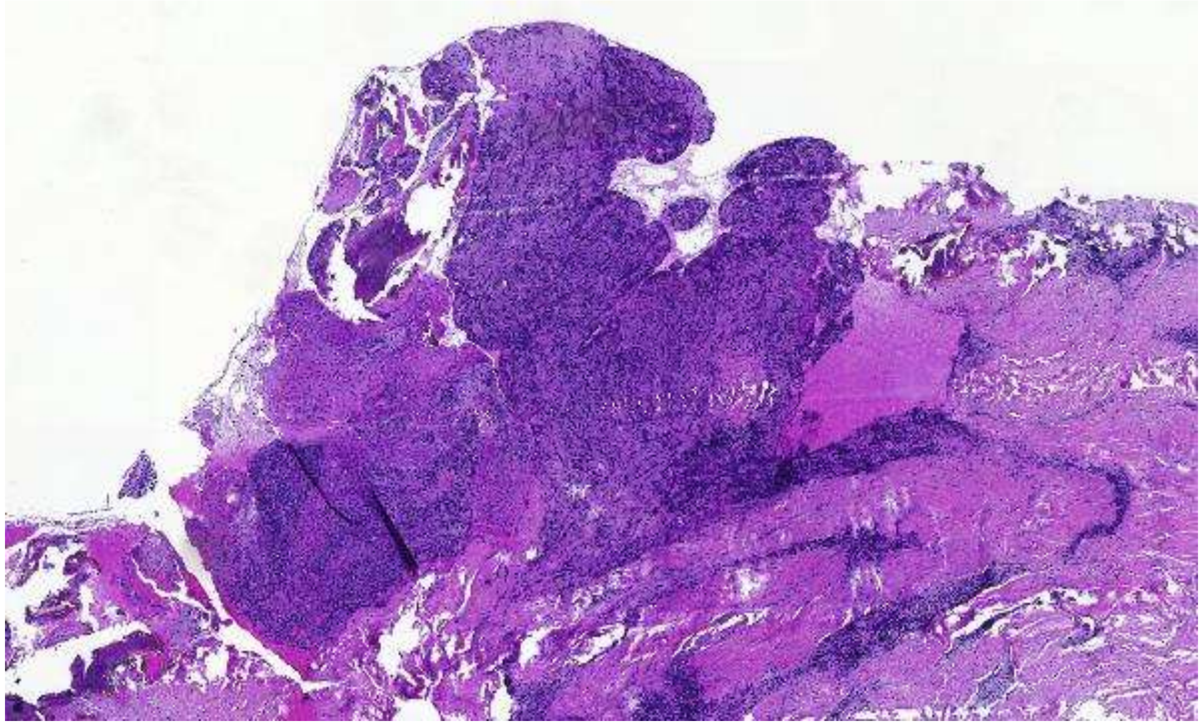

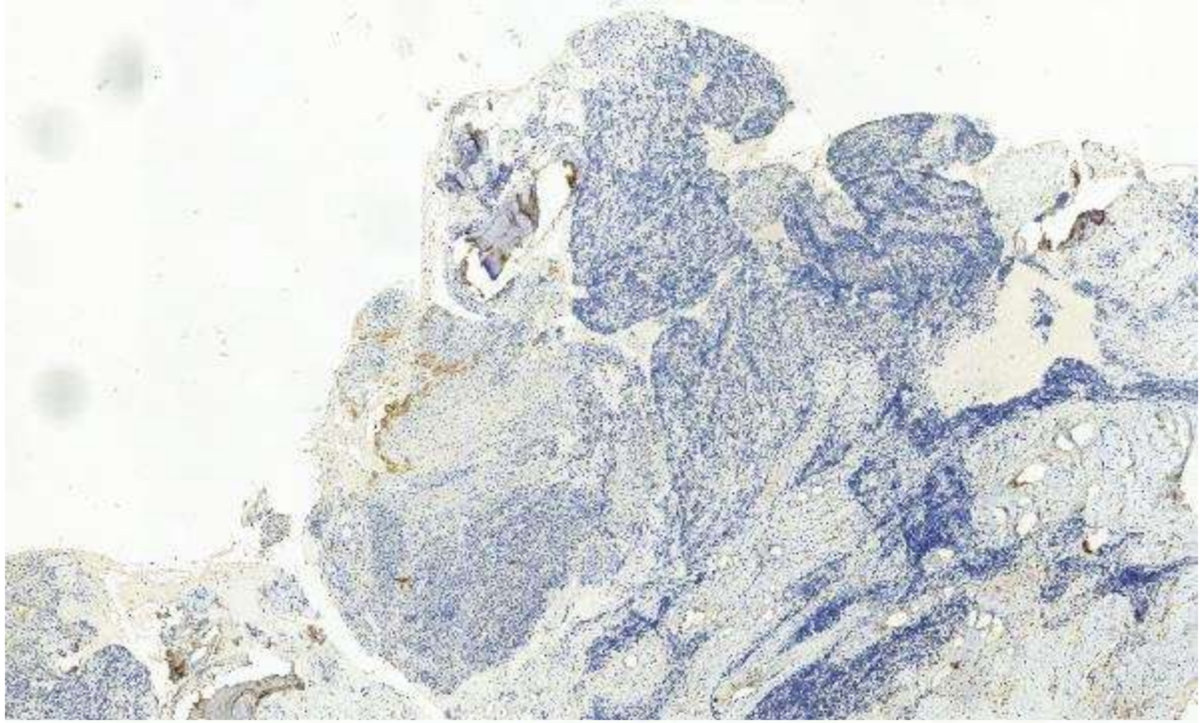

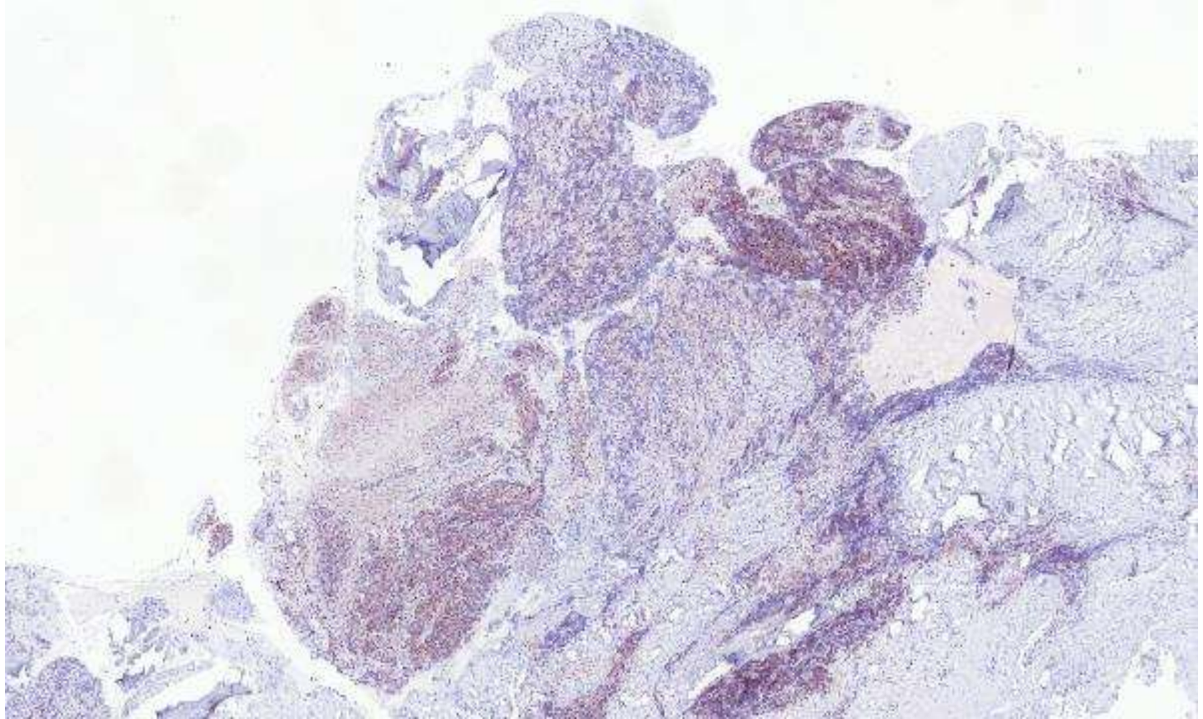

K11 – HE, CK17, bcl2 (x10)

Focal superficial CK17 staining

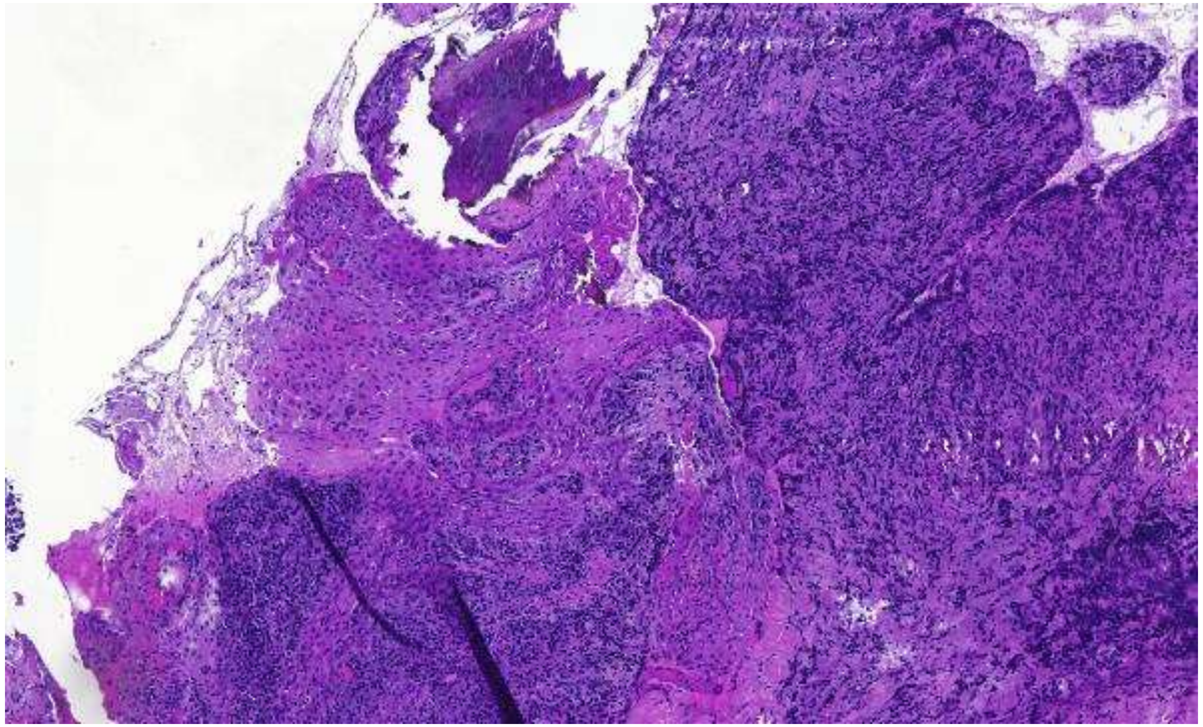

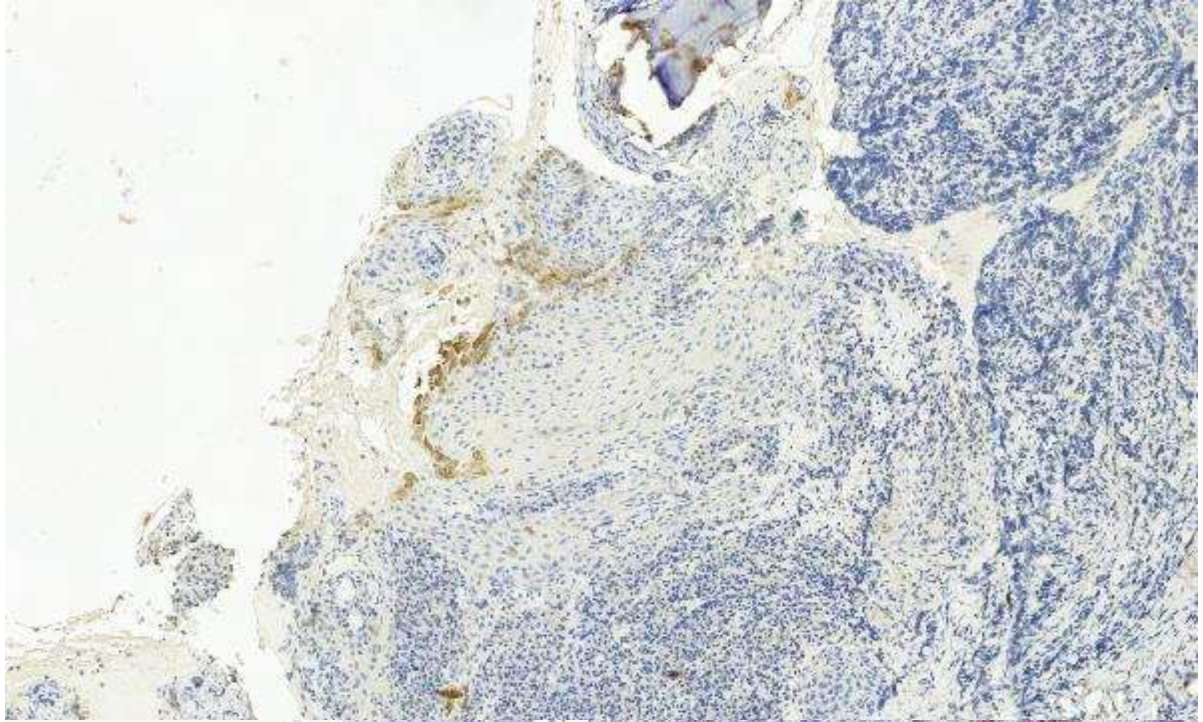

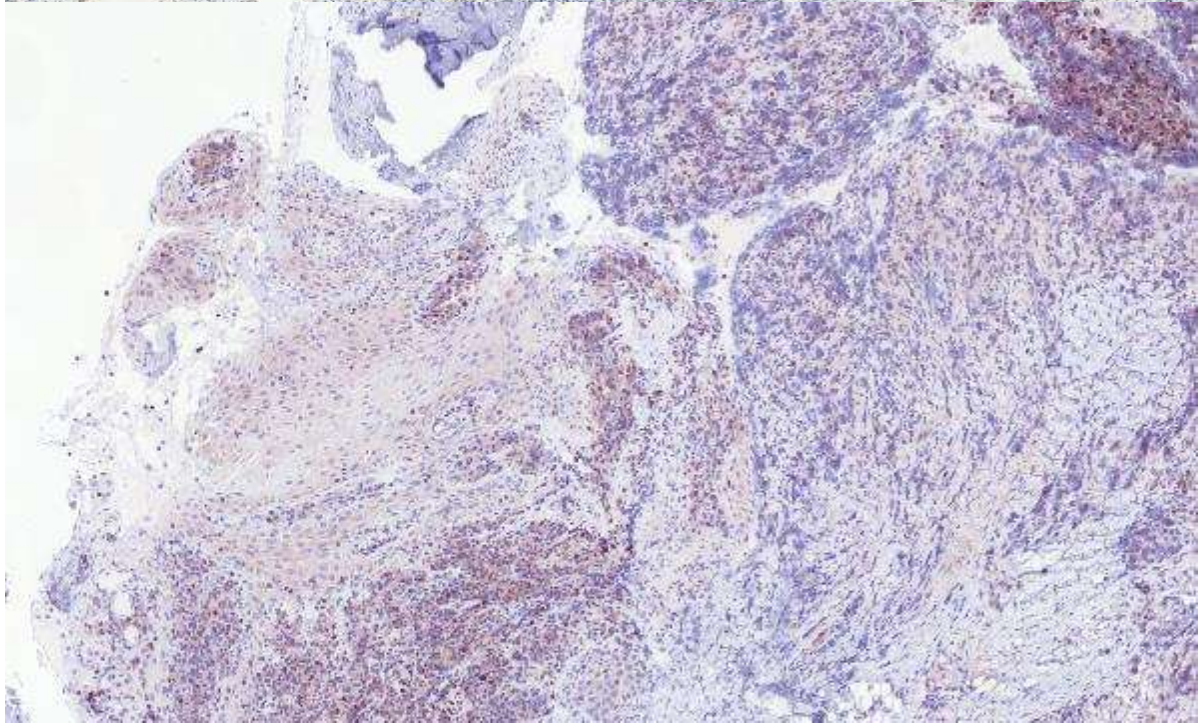

K11 – HE, CK17, bcl2 (x8)

Negative CK17 and bcl2 IHC

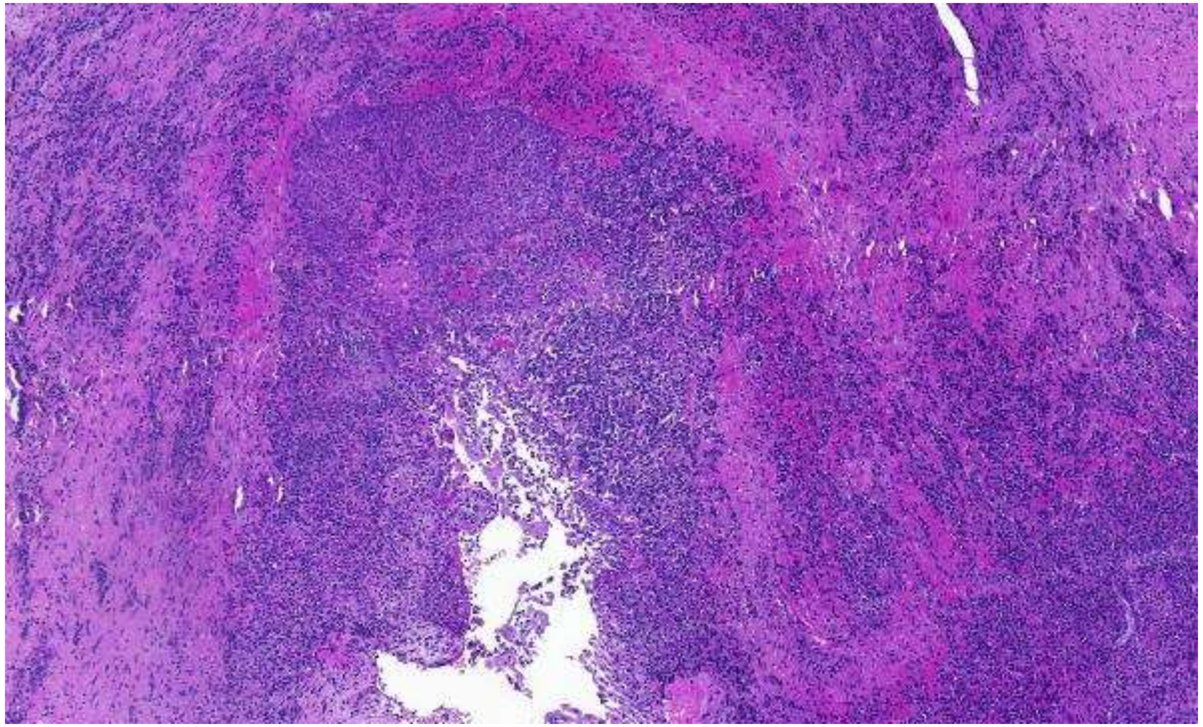

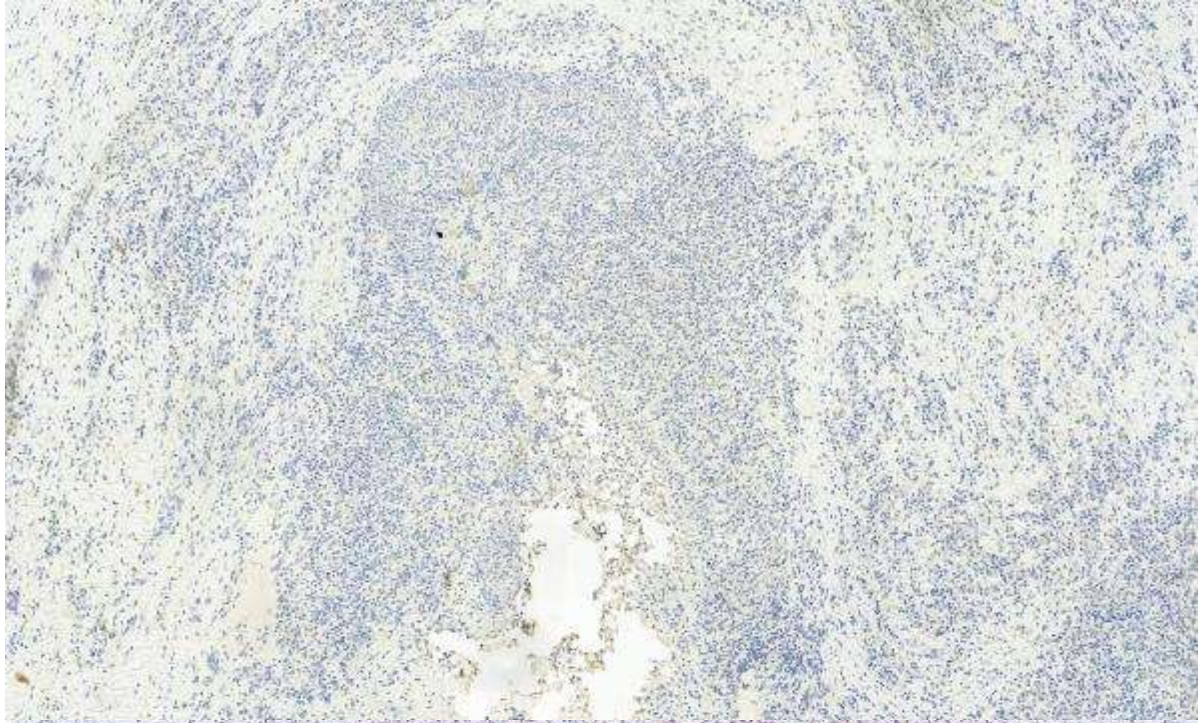

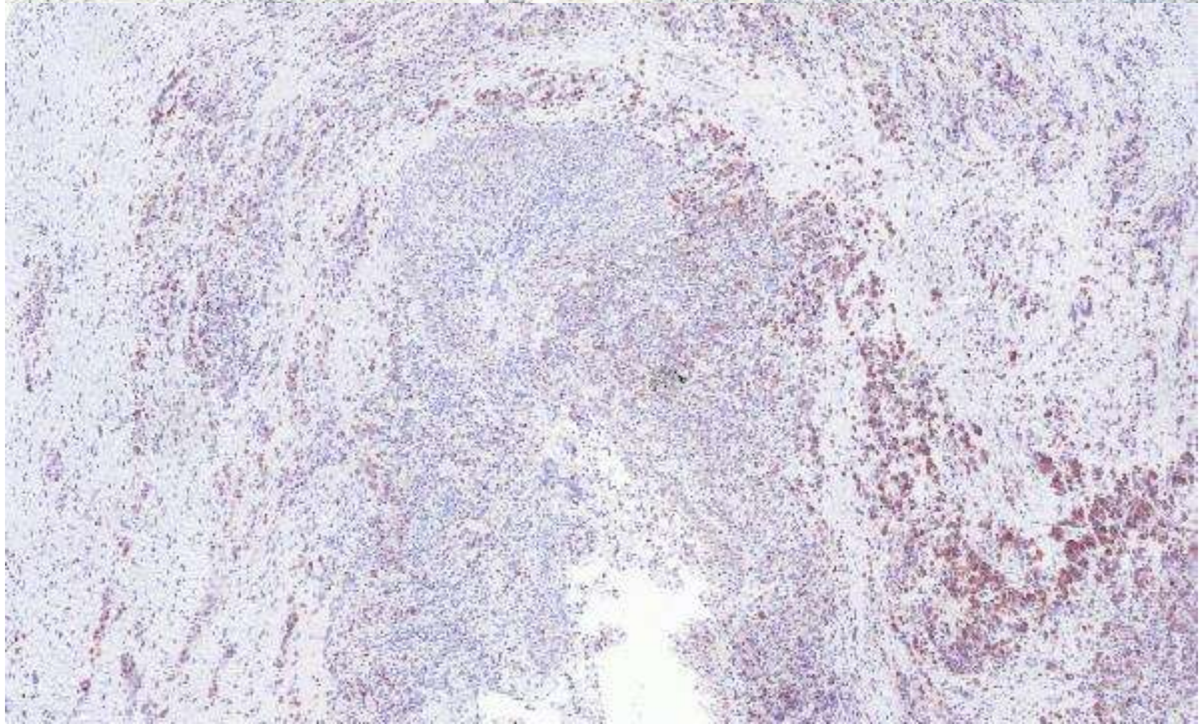

K11 – Conclusion

- H&E: relatively thick squamous epithelium, no parakeratosis, no wavy surface, no basal palisading, even epithelial-subepithelial contours;
- Marked mixed inflammation of the cyst wall
- Bcl2: negative
- CK17: dominantly negative with focal superficial positivity
- Histopathology: radicular cyst
- Combined clinical and pathological opinion: radicular cyst

Case K12

- 56-year-old female (cyst 2 of the same patient as Case K11)
- Dental hygiene/status: partly missing, partly replaced teeth, removable upper denture, mobile bridge over teeth 33, 32 and 42, 43 – all with probable pouches; vestibular mucosa in area of tooth 12 hyperaemic and painful on pression
- Radiology: … and similar radiolucency in the region of missing teeth 44 and 45
- Operation: removal of the bridge, residual movable roots and both cysts
- Clinical diagnosis: mandibular cyst of region of teeth 44-45

K12 – OPT-1

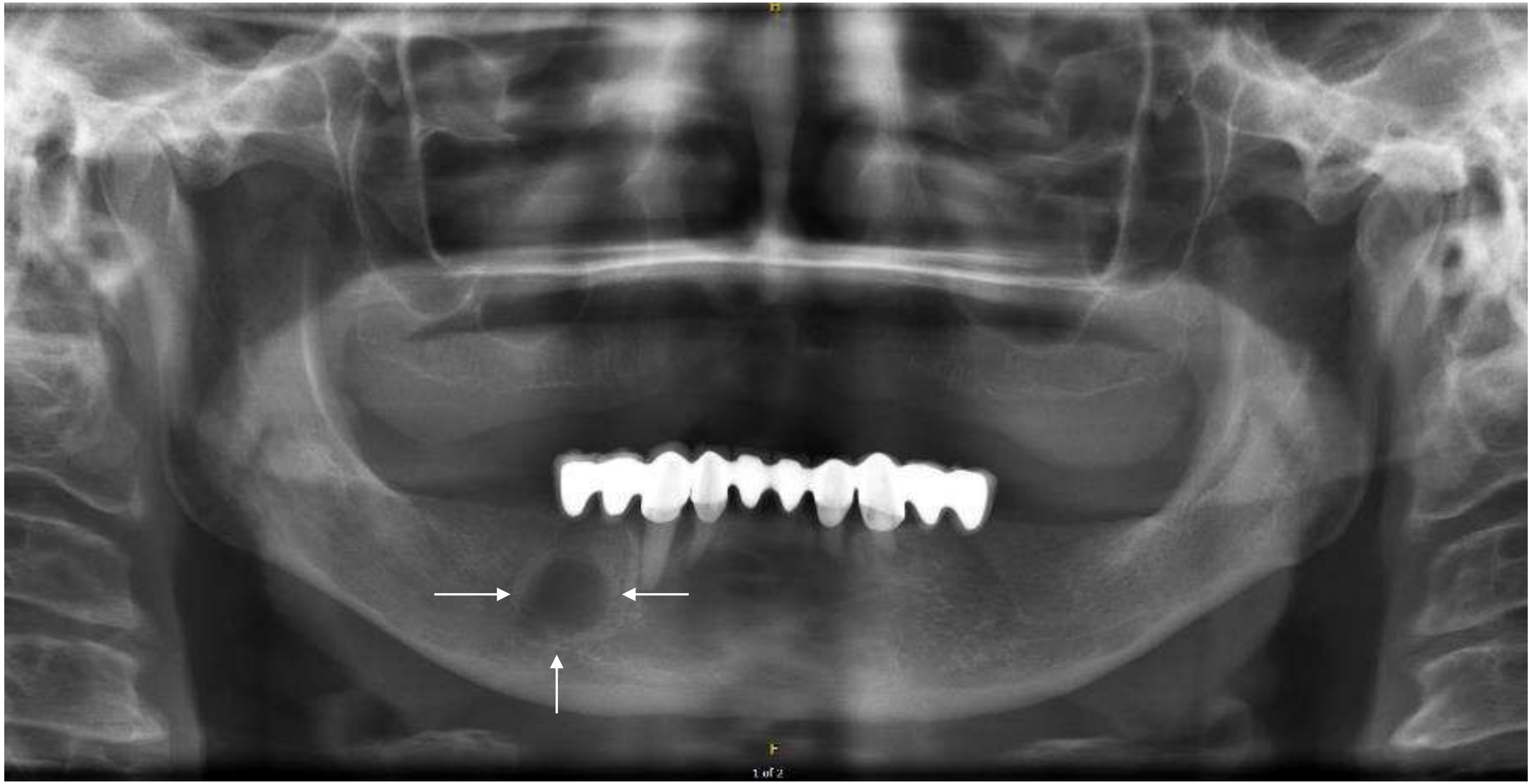

K12 – OPT-2

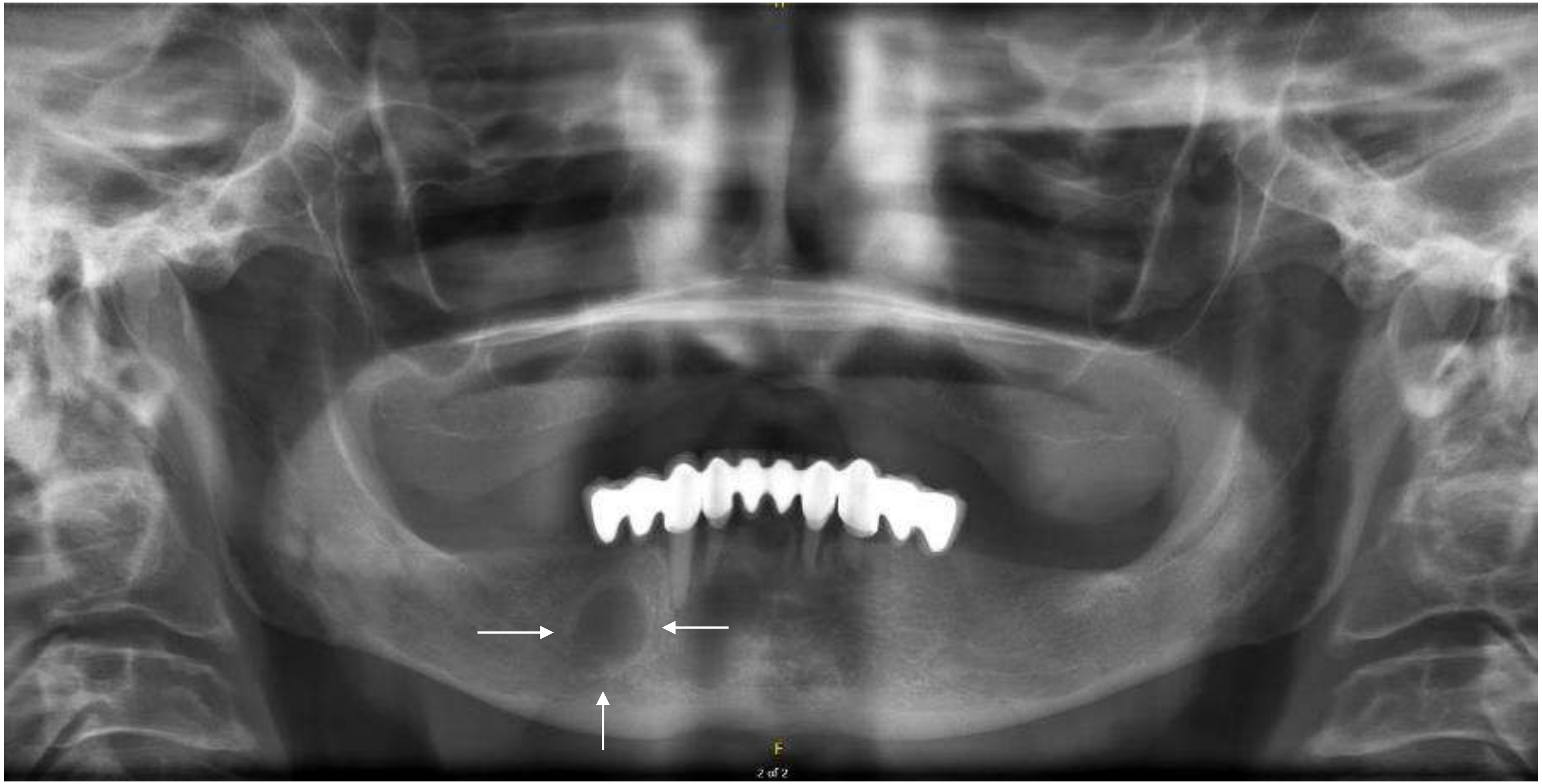

K12 – HE, CK17, bcl2 (x1.4)

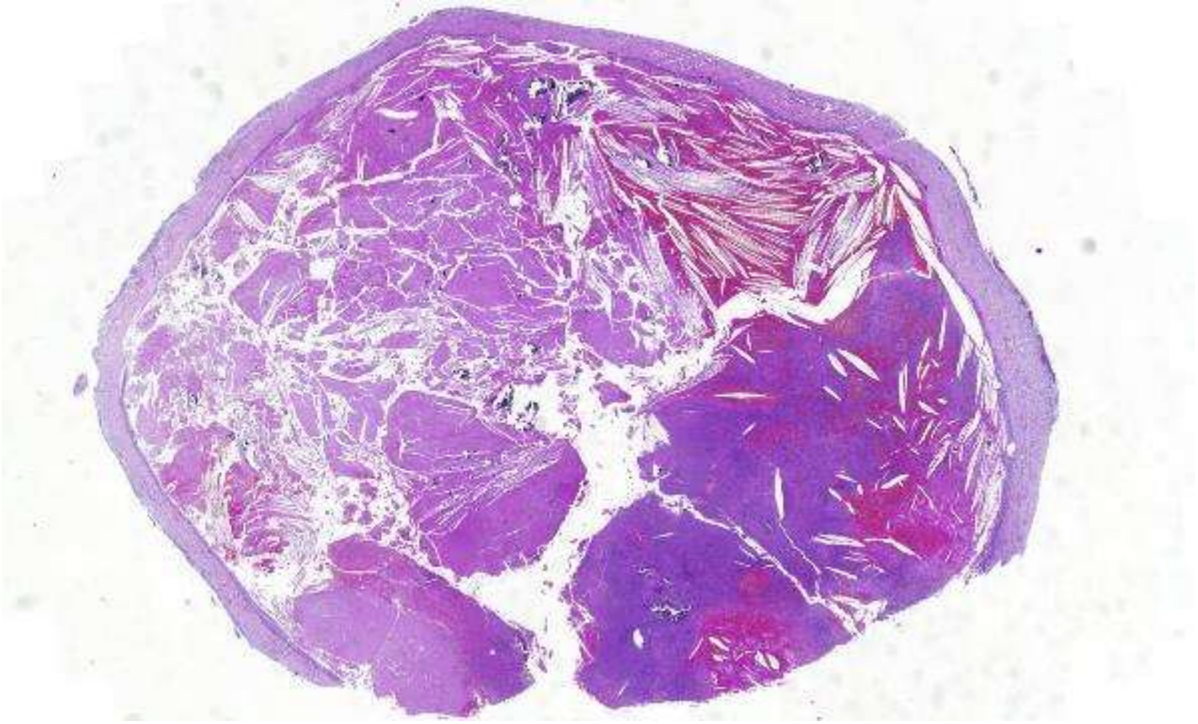

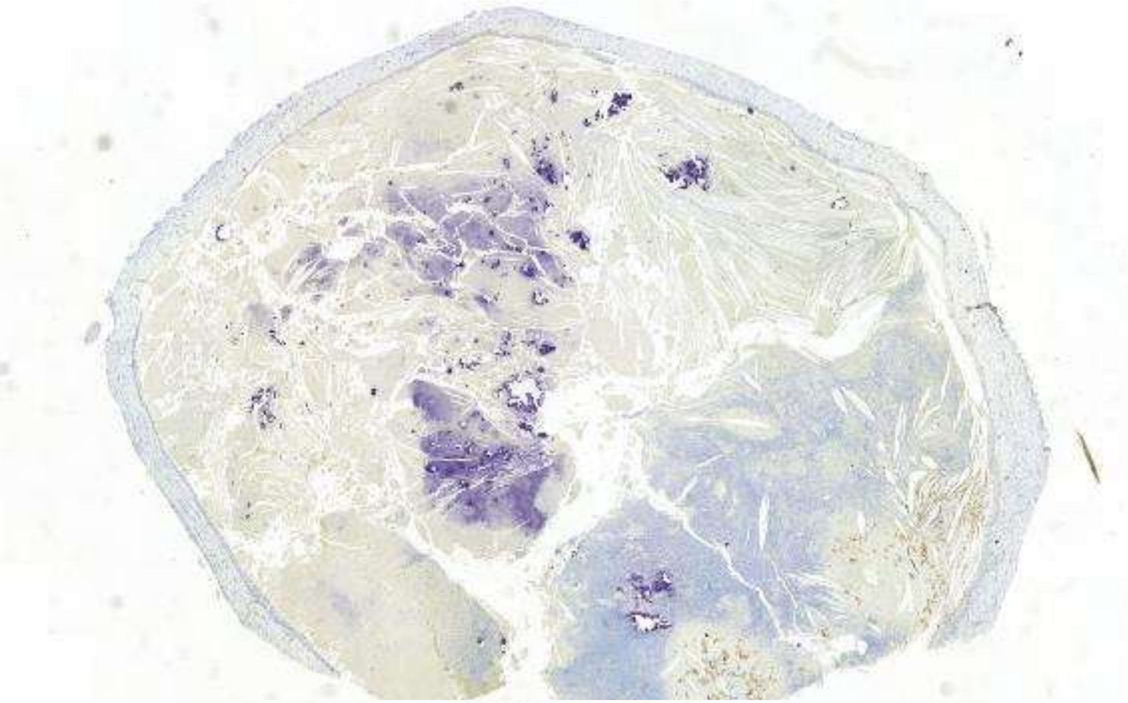

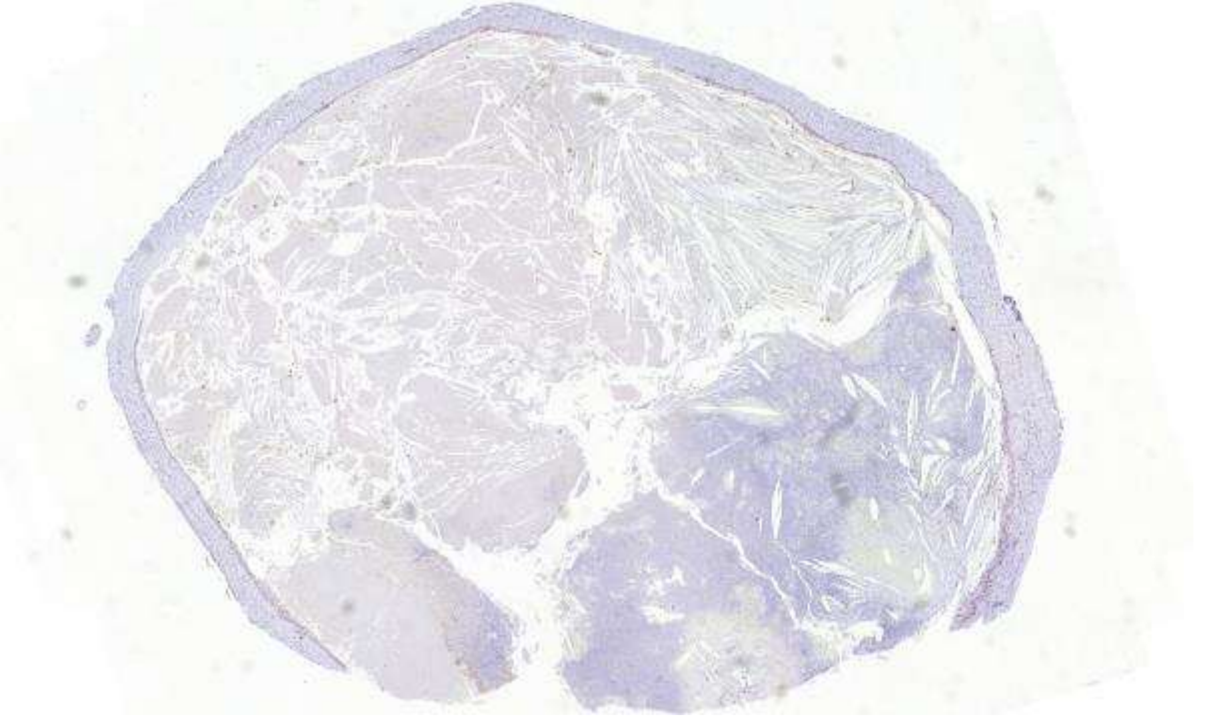

K12 – HE, CK17, bcl2 (x10)

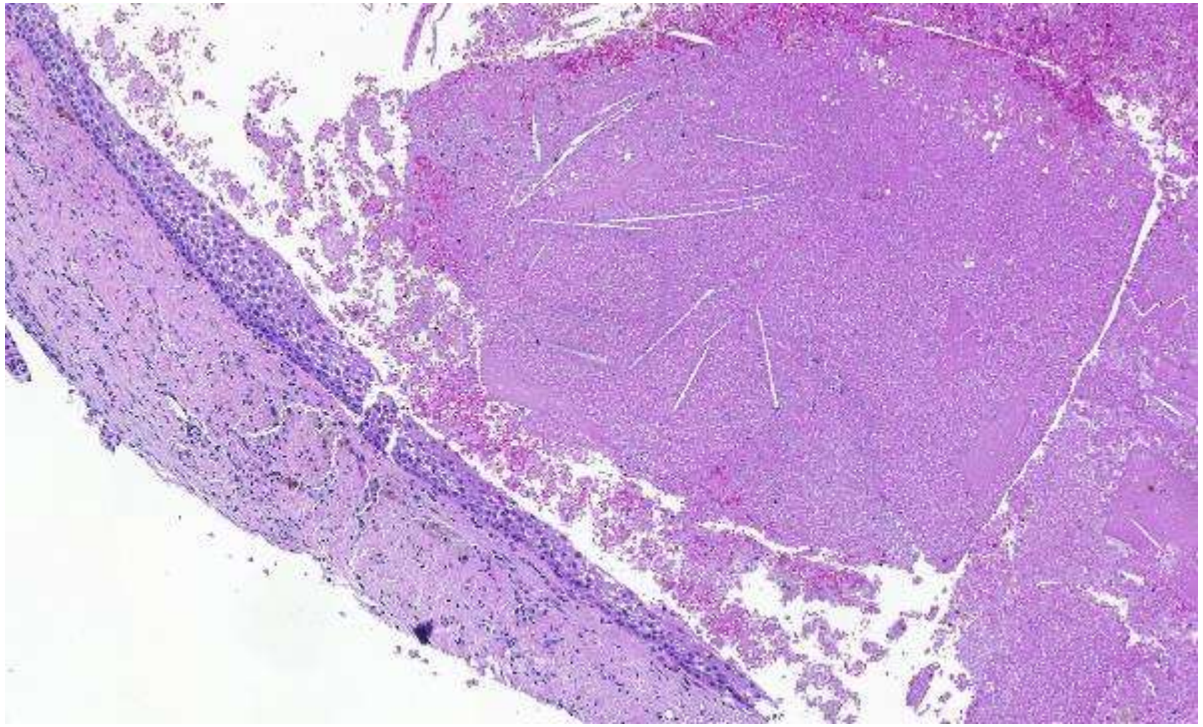

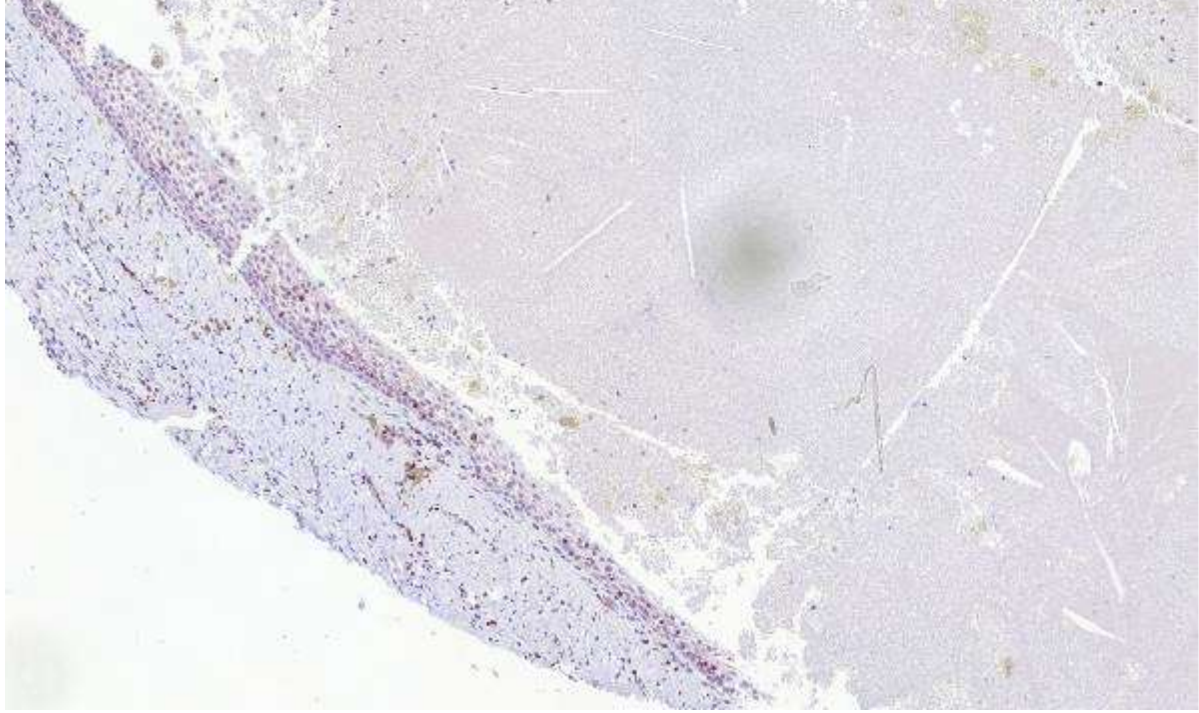

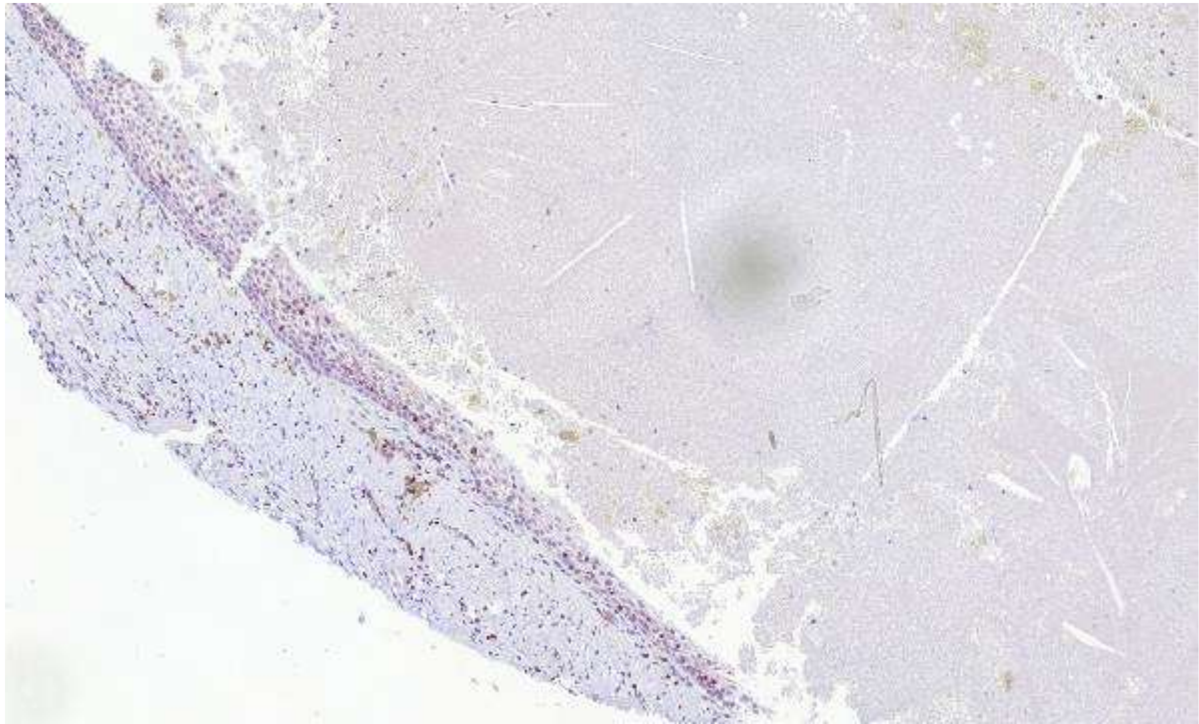

K12 – HE, CK17, bcl2 (x20)

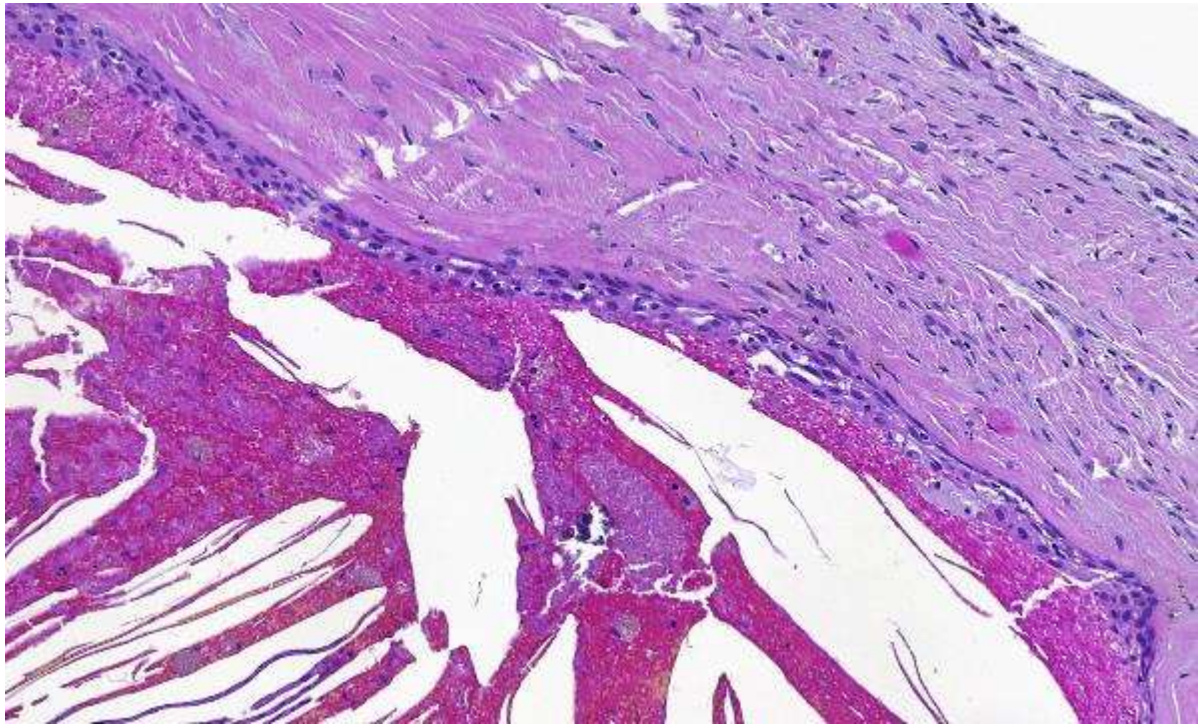

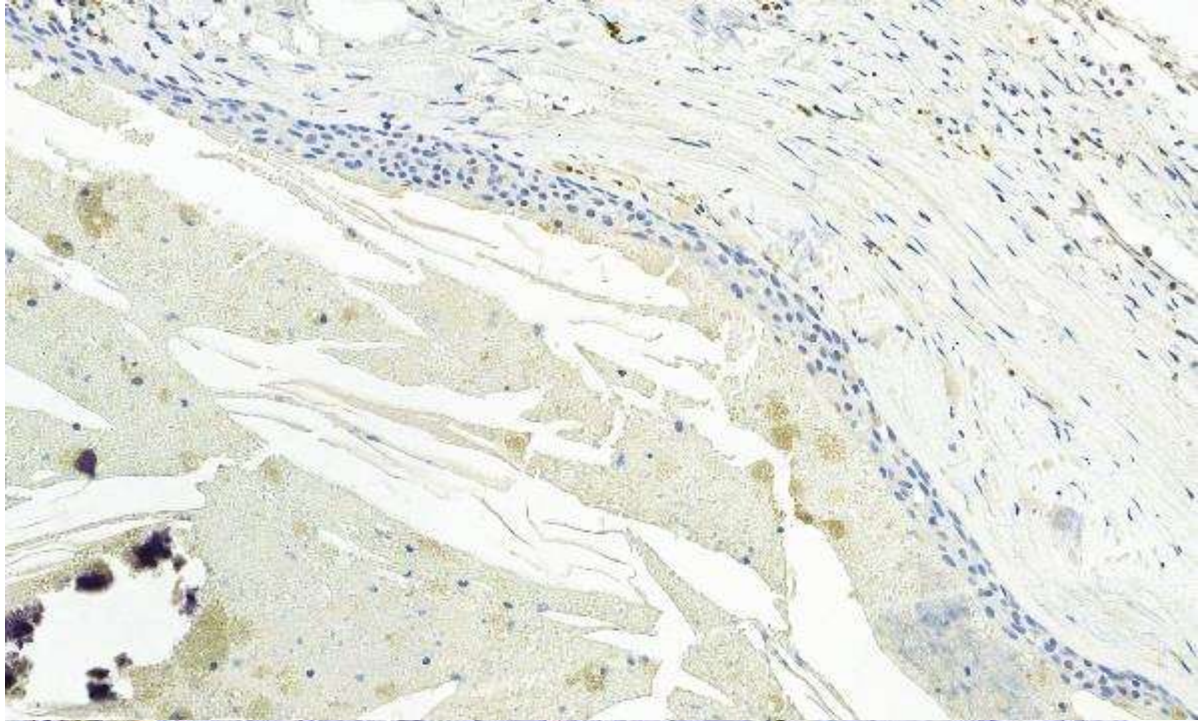

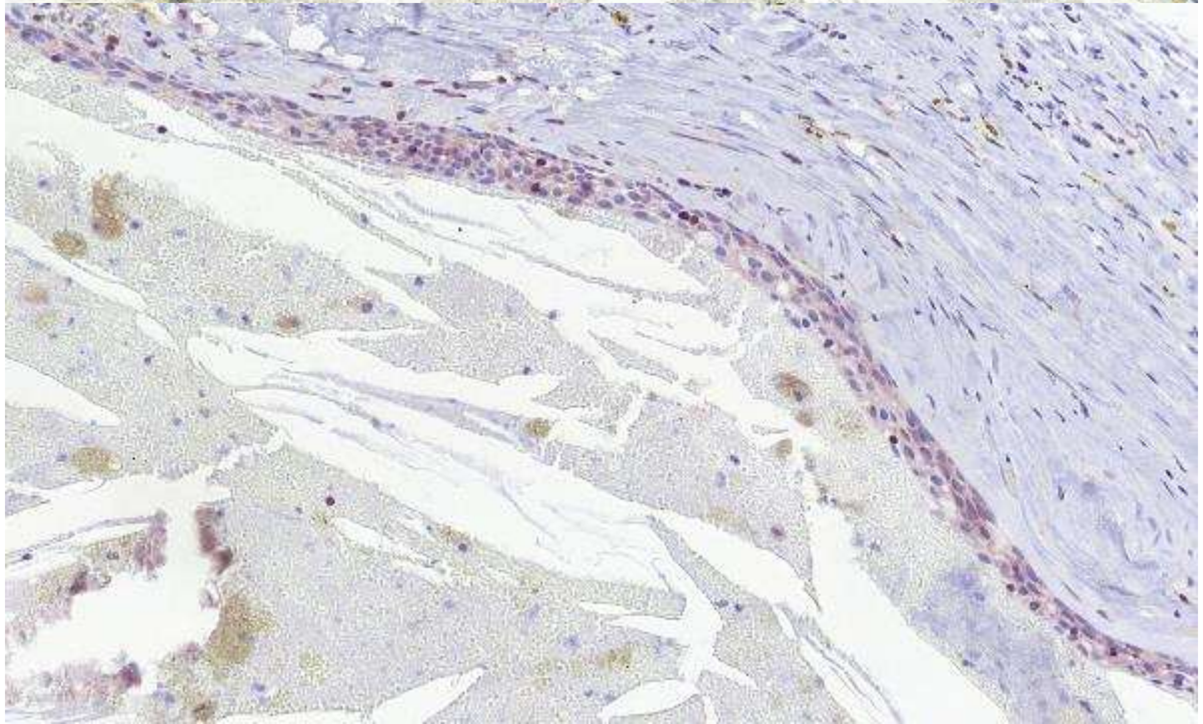

K12 – bcl2 negativity with lymphocytes staining

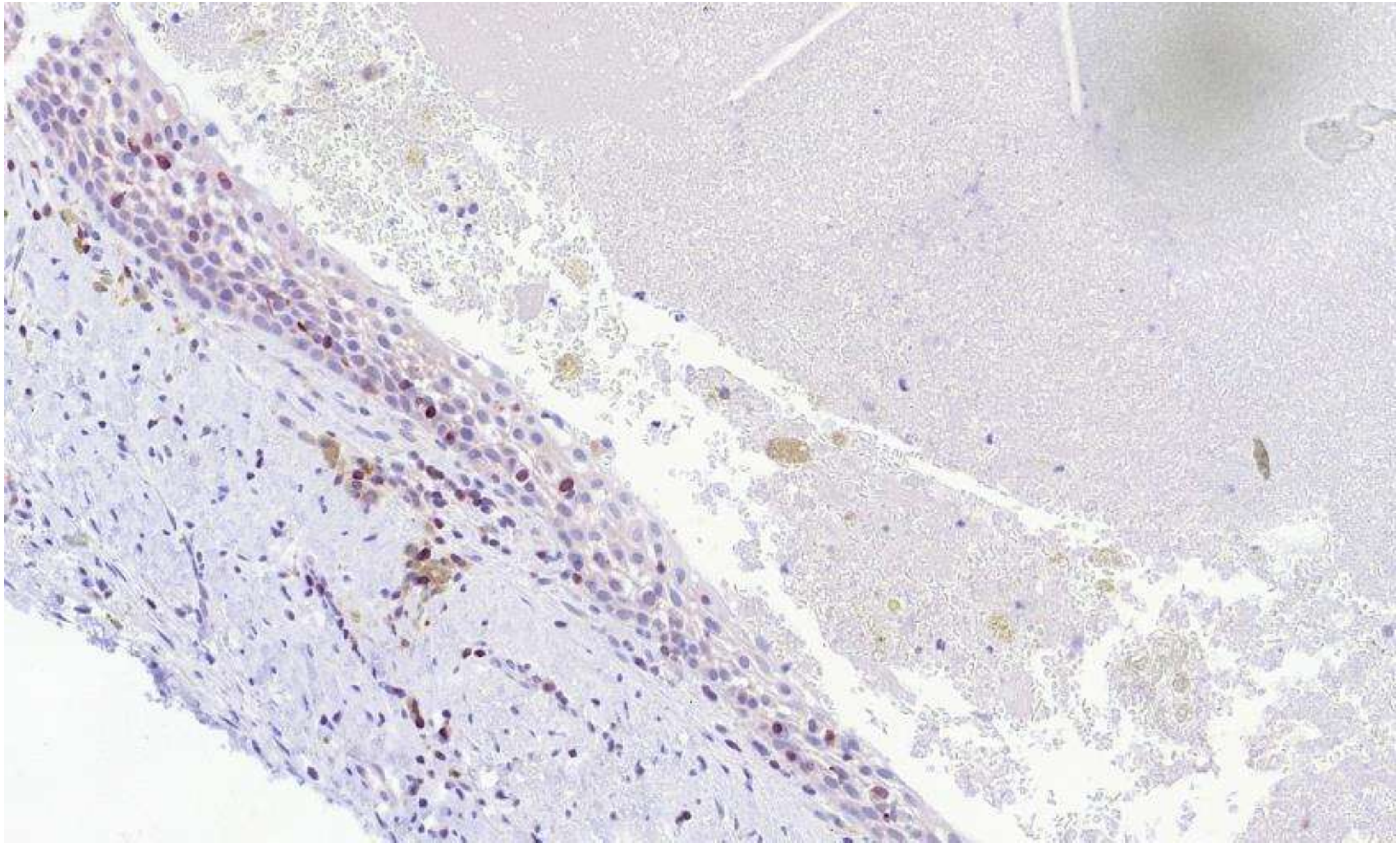

K12 – Conclusion

- H&E: relatively thick squamous epithelium (with focal thinning), no parakeratosis, no wavy surface, no basal palisading, uneven epithelial-subepithelial contours
- Minimal focal chronic inflammation of the cyst wall
- Bcl2: negative
- CK17: dominantly negative, with a few superficial cells staining
- Histopathology: residual cyst
- Combined clinical and pathological opinion: (mature) residual cyst

Case K13

- 53-year-old male
- Dental hygiene/status: partly missing, partly treated teeth; periodontitis
- Radiology: Radiolucent lesion suggestive of dentigerous cyst at tooth 38; tenderness of the overlying vestibular mucosa
- Operation: removal of the cyst and tooth 38 together
- Clinical diagnosis: follicular cyst

K13 – CT

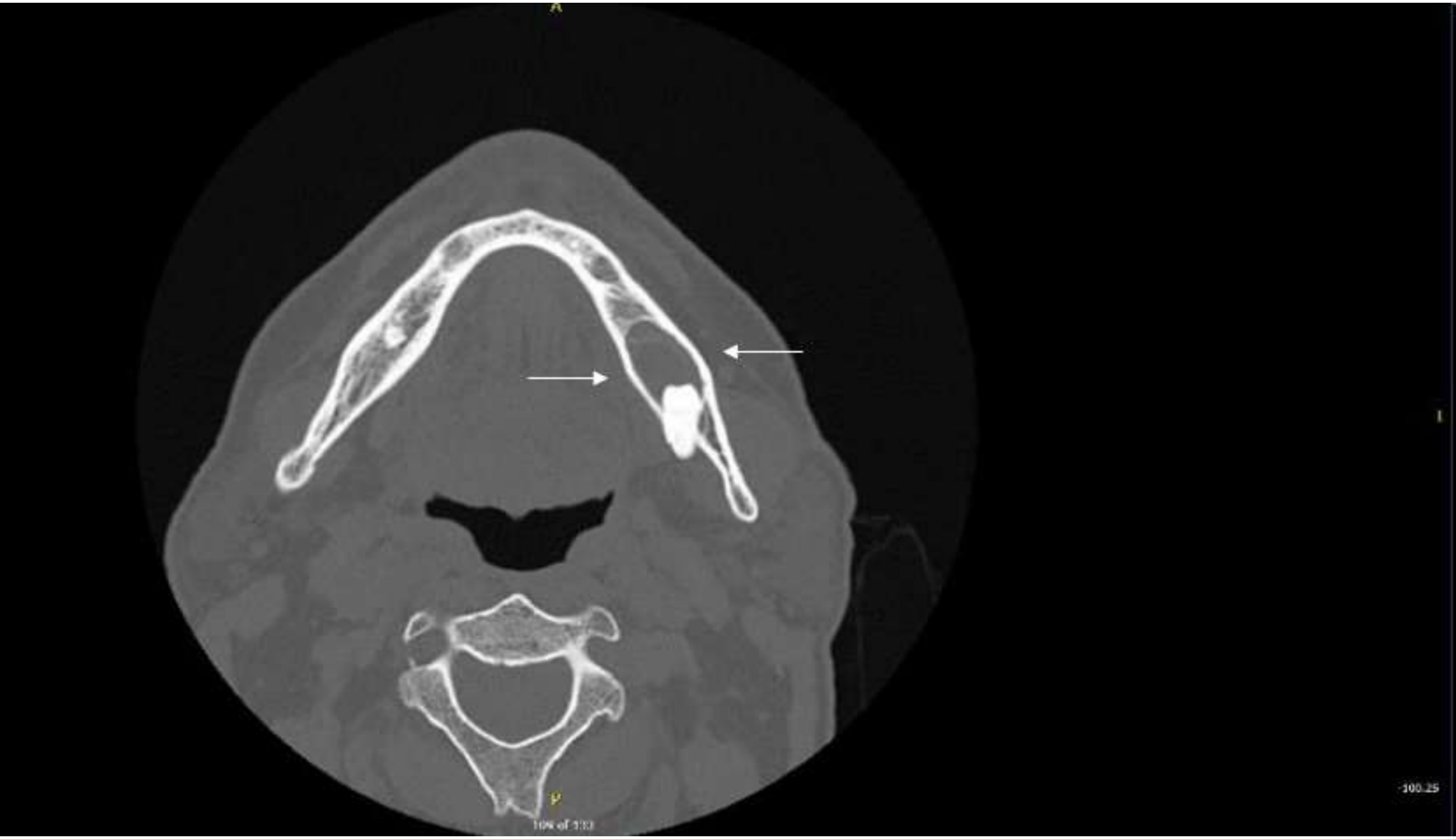

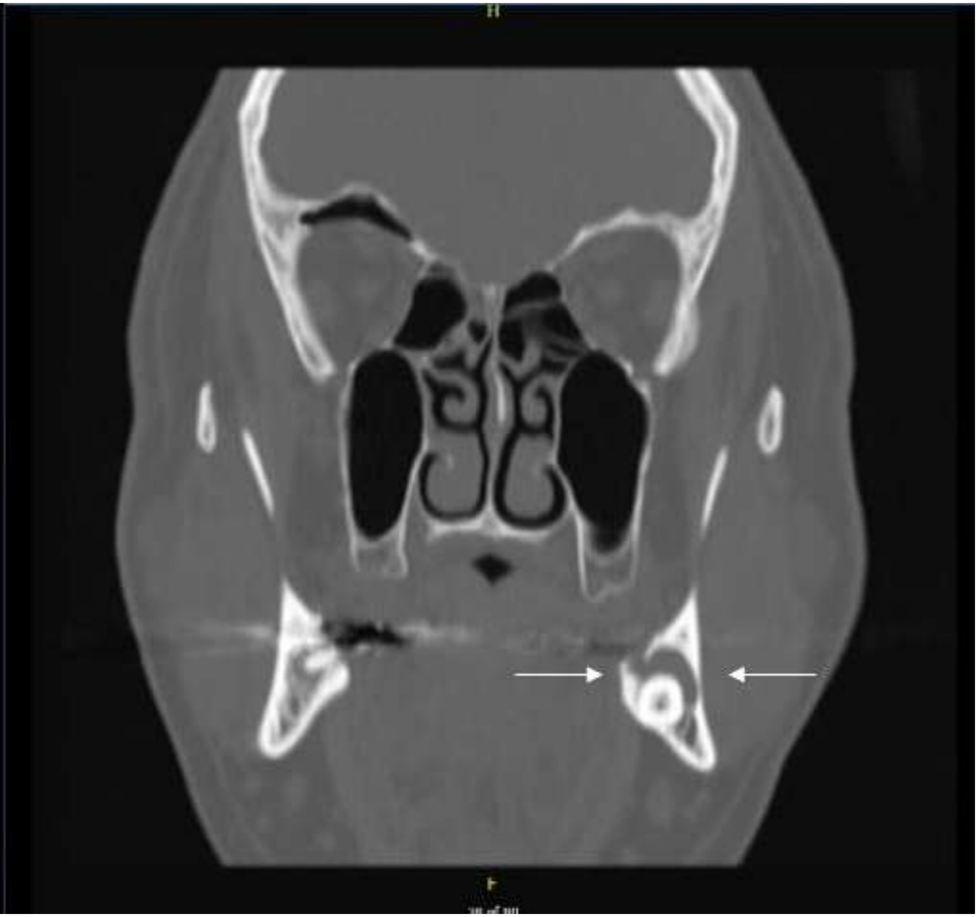

K13 – HE, CK17, bcl2 (x5)

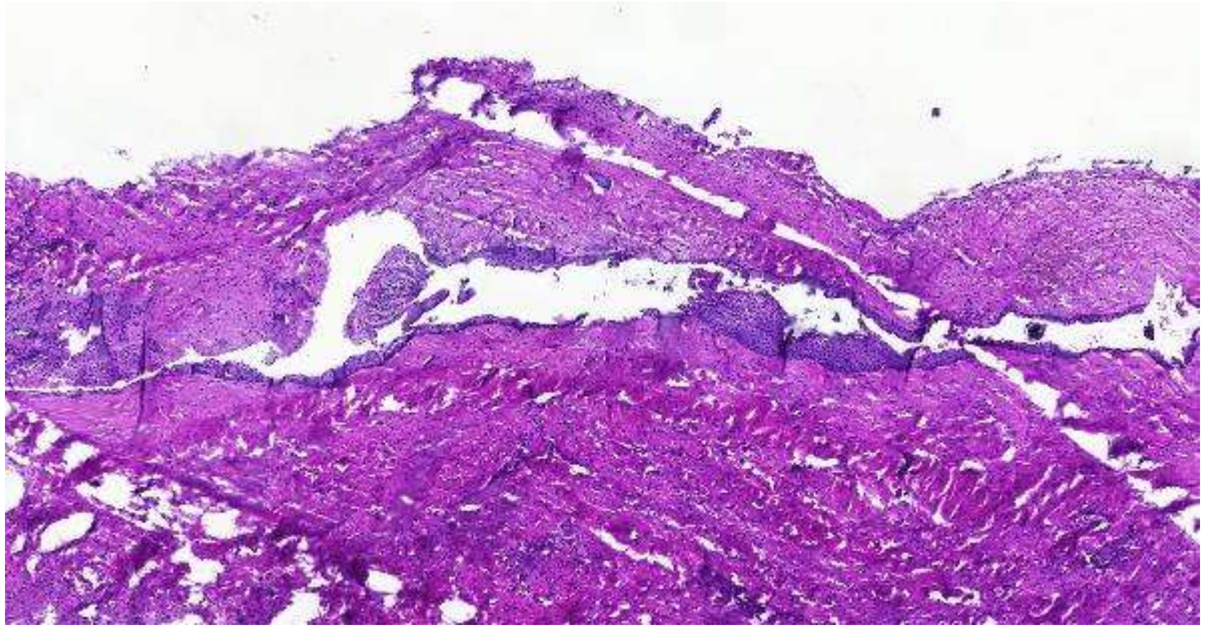

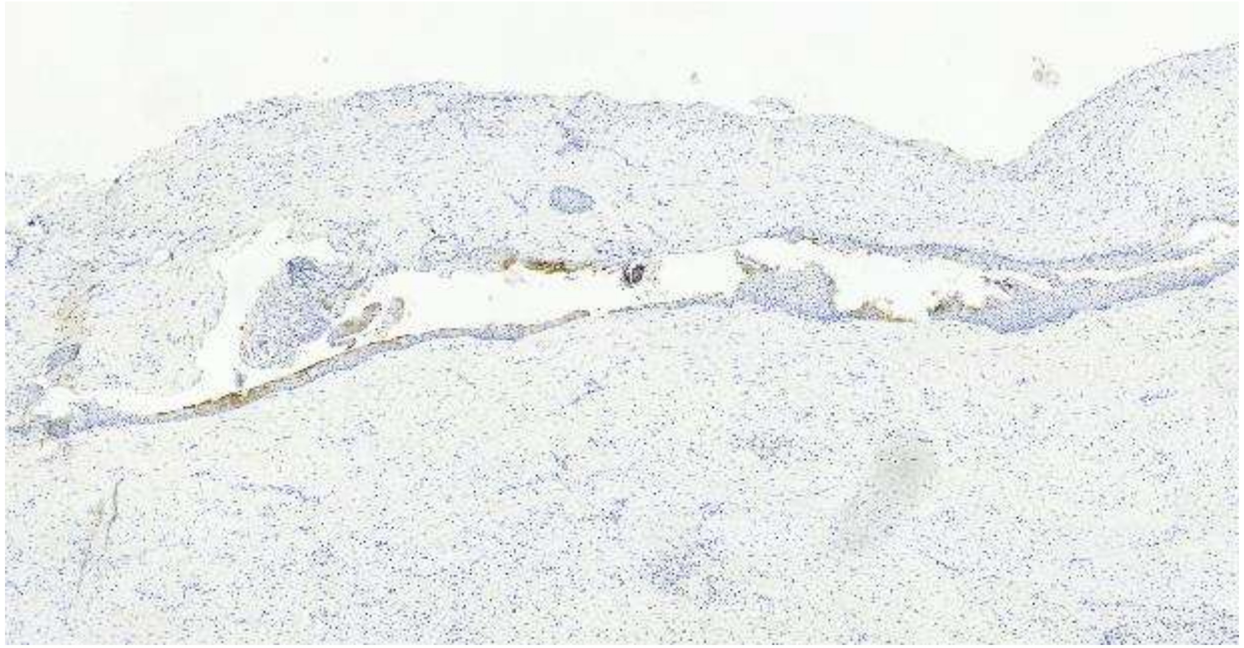

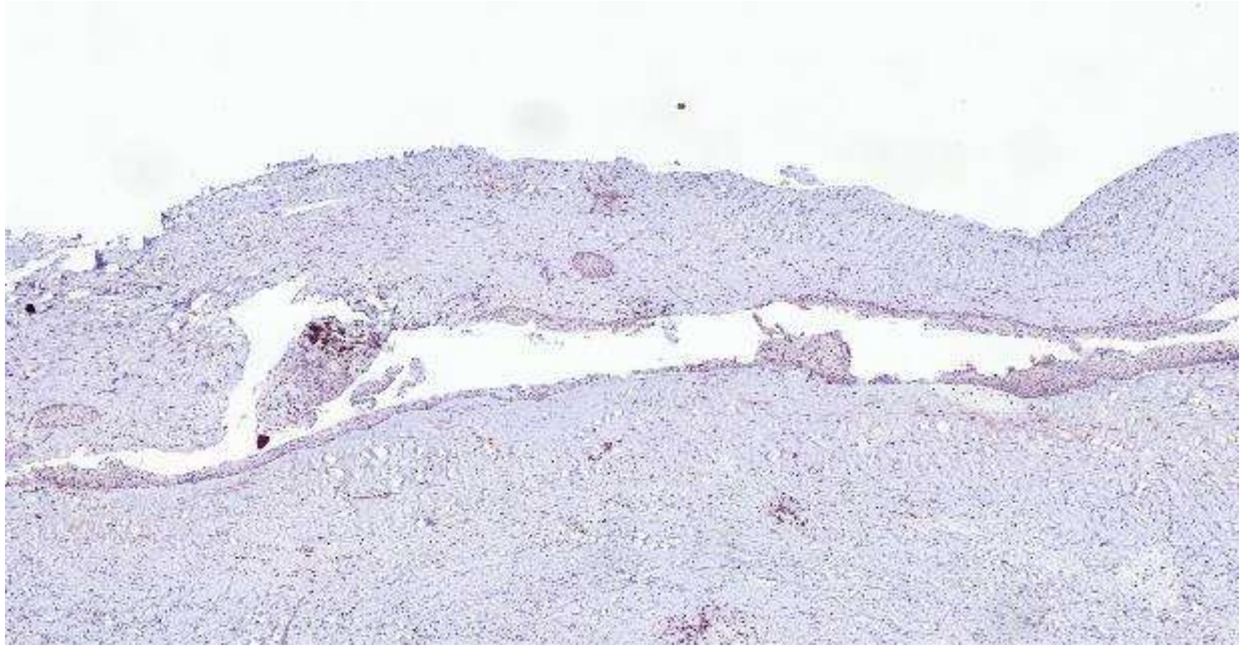

K13 – HE, CK17, bcl2 (x10)

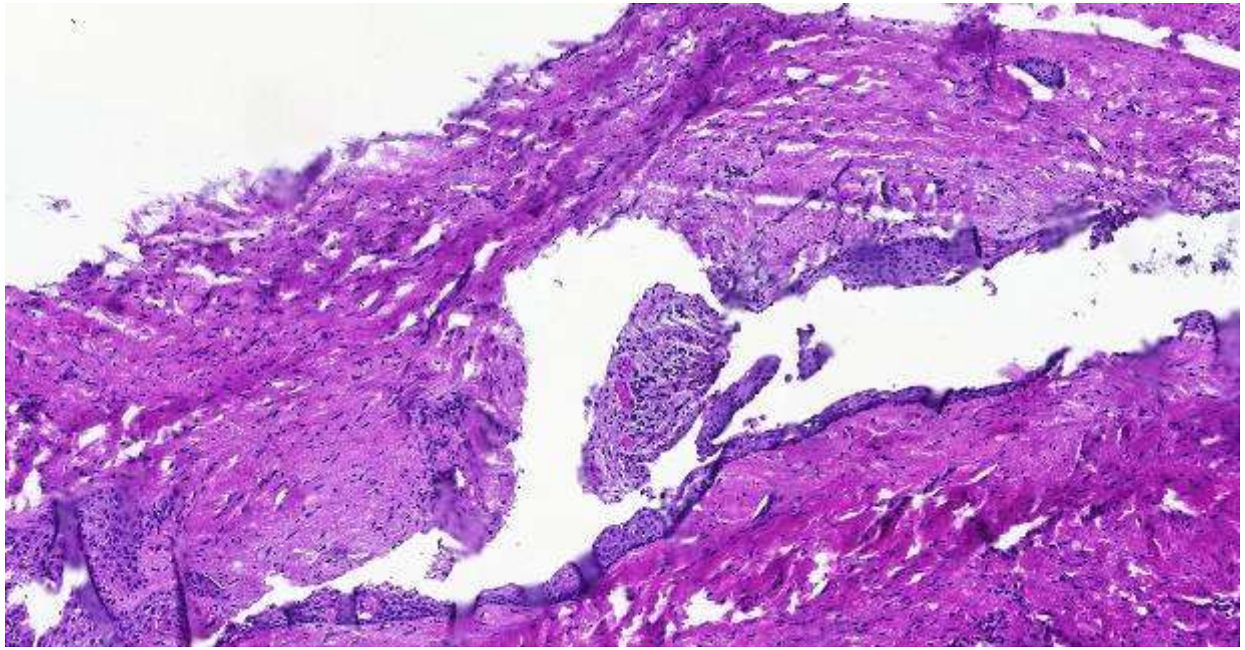

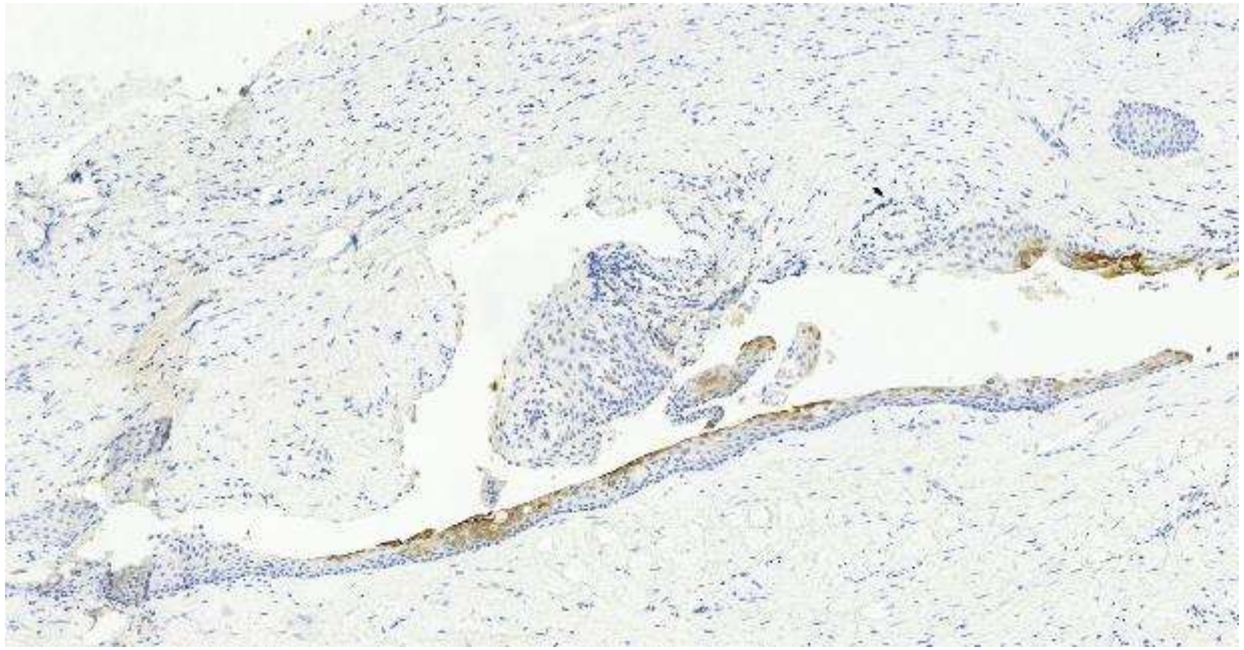

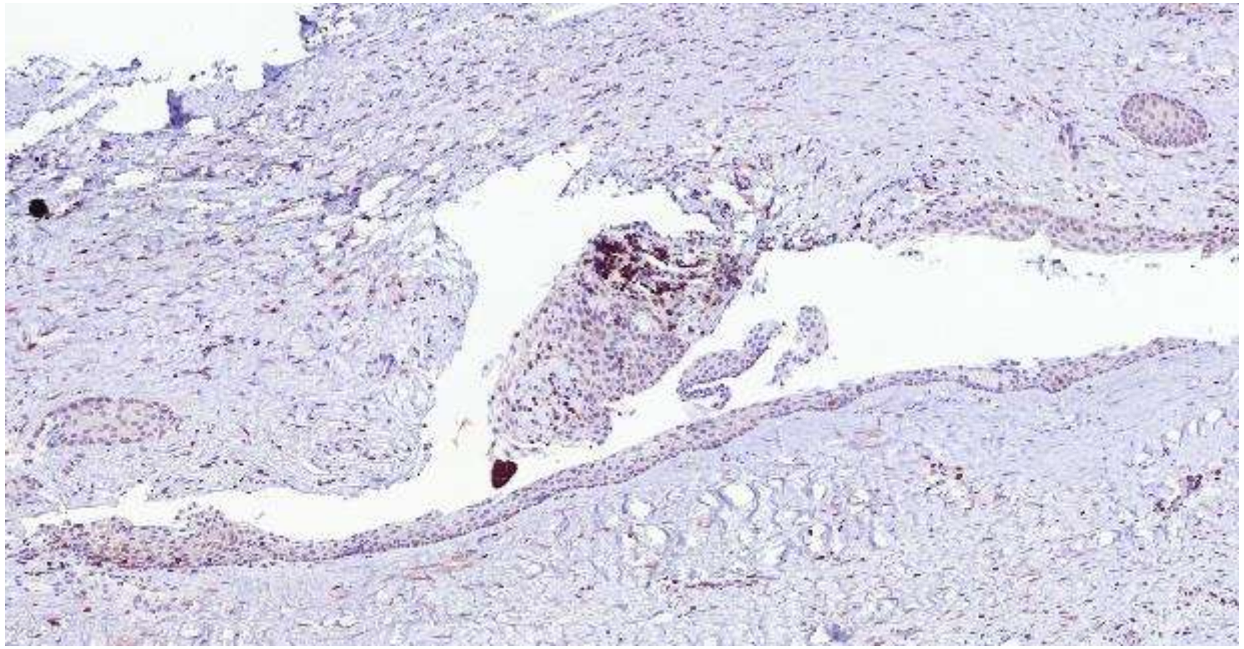

K13 – HE, CK17, bcl2 (x8)

mucous metaplasia (→)

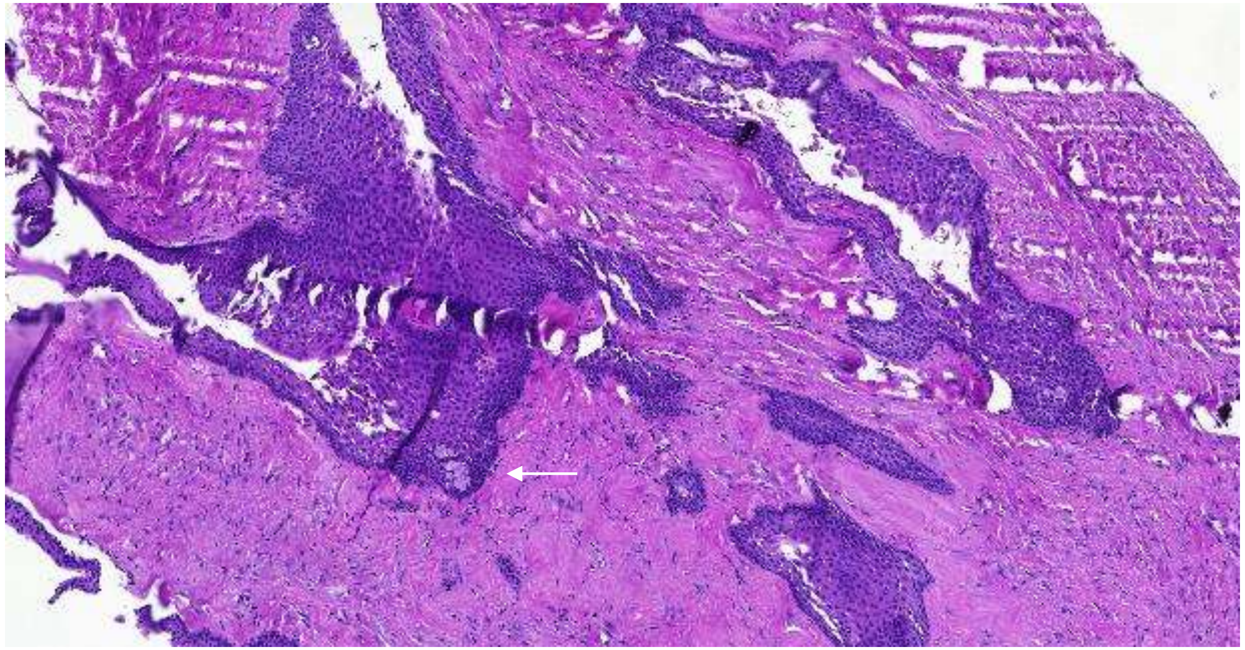

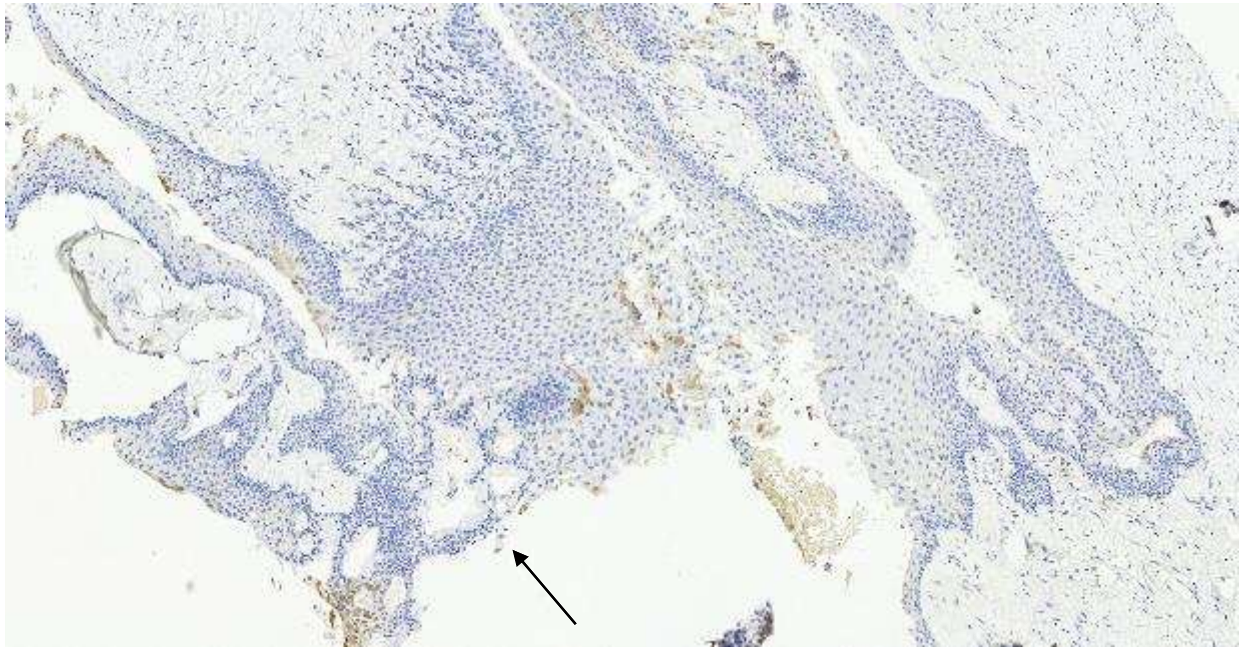

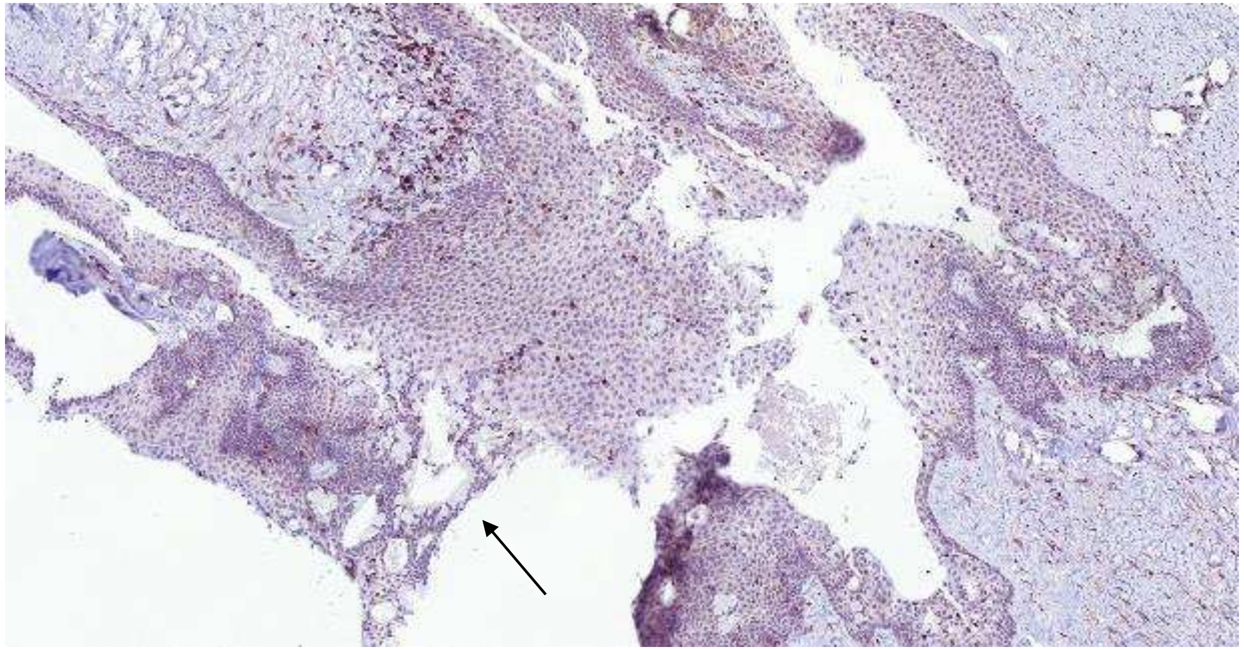

K13 – Conclusion

- H&E: relatively thick squamous epithelium, no parakeratosis, no wavy surface, no basal palisading, uneven to even epithelial-subepithelial contours; mucous metaplasia
- No inflammation
- Bcl2: negative (background darker)
- CK17: focal superficial staining
- Histopathology: dentigerous (follicular) cyst
- Combined clinical and pathological opinion: dentigerous (follicular) cyst

Case K14

- 40-year-old female
- Dental hygiene/status: poor; partly conserved, partly missing, untreated teeth with multiple caries
- Radiology: corticated radiolucent lesion of 45 mm in size in the right maxillary sinus
- Operation: removal of teeth 15, 13-11, 21, 23-24 and the cyst with conservation of the sinus and the basis of the nose
- Clinical diagnosis: maxillary cyst (radicular cyst mentioned elsewhere in the charts)

K14 – OPT

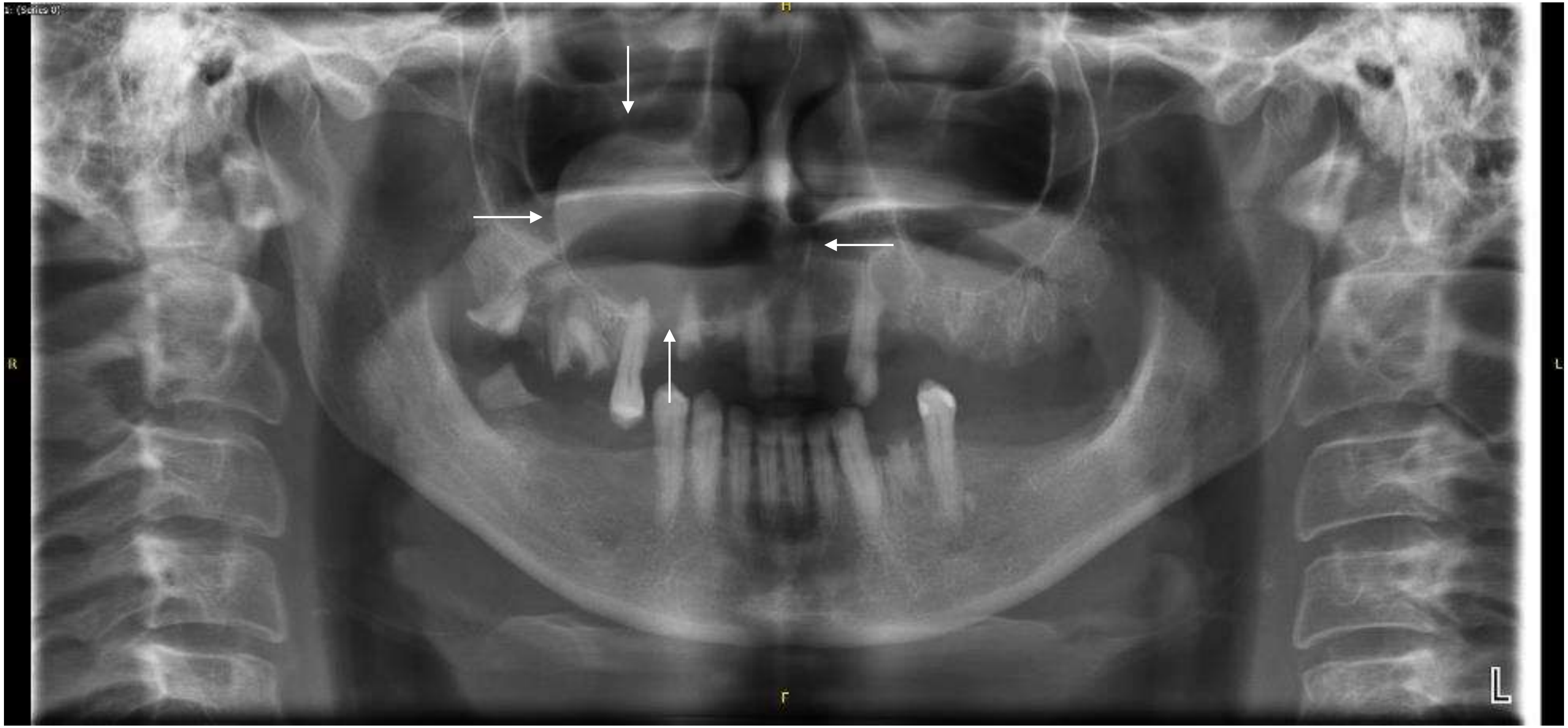

K14 – CT axial (153, 150, 138 of 241 slices)

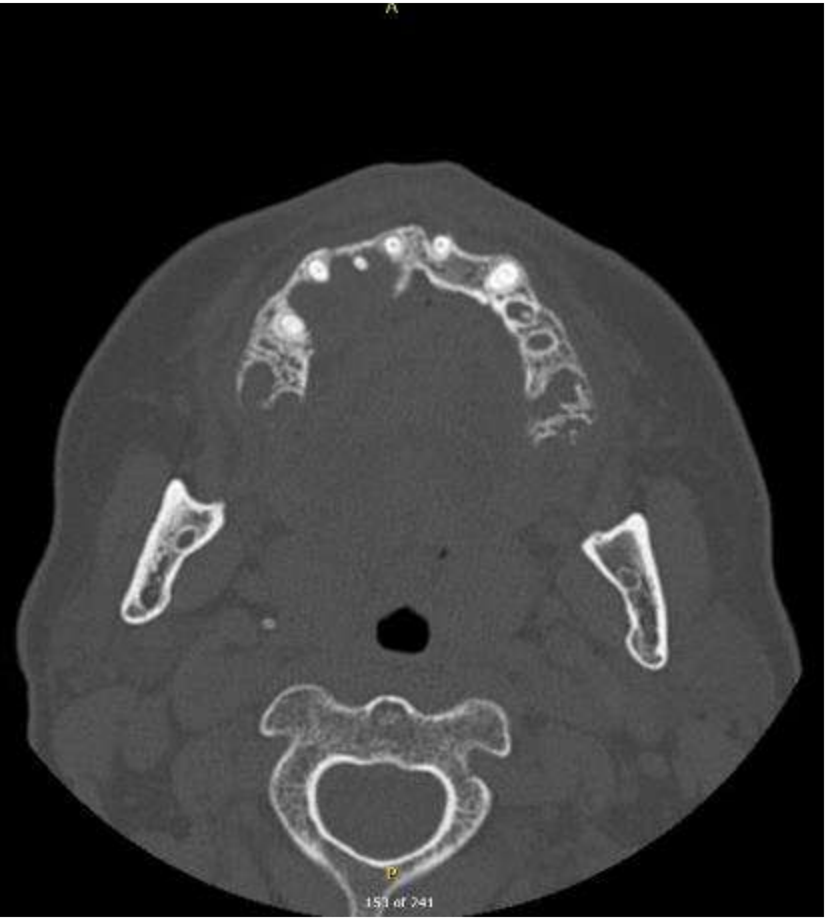

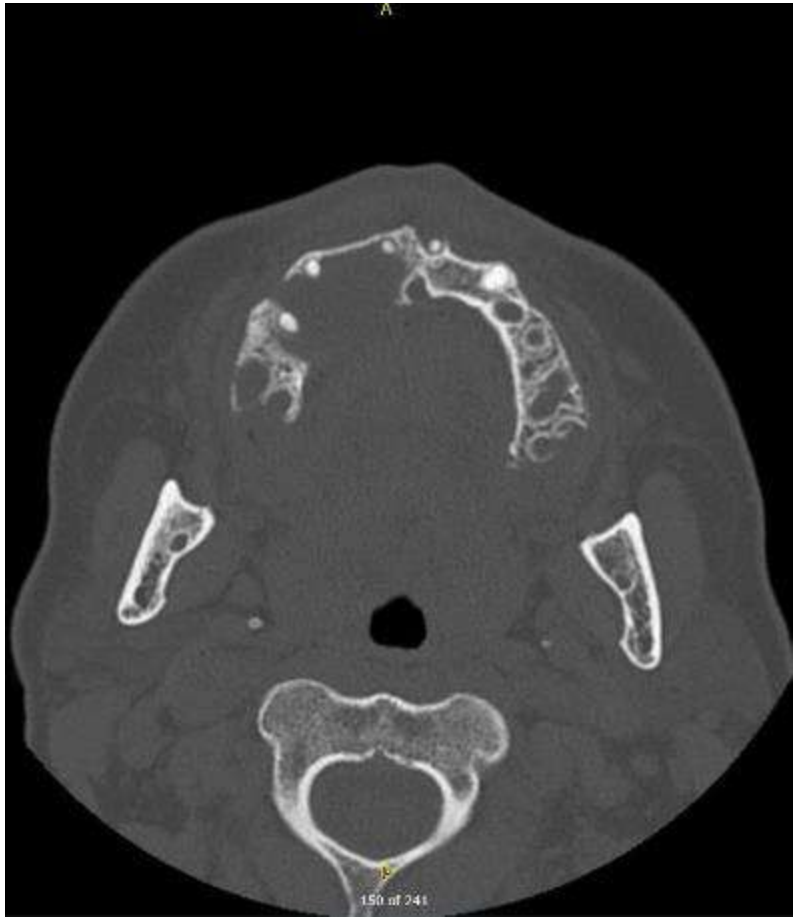

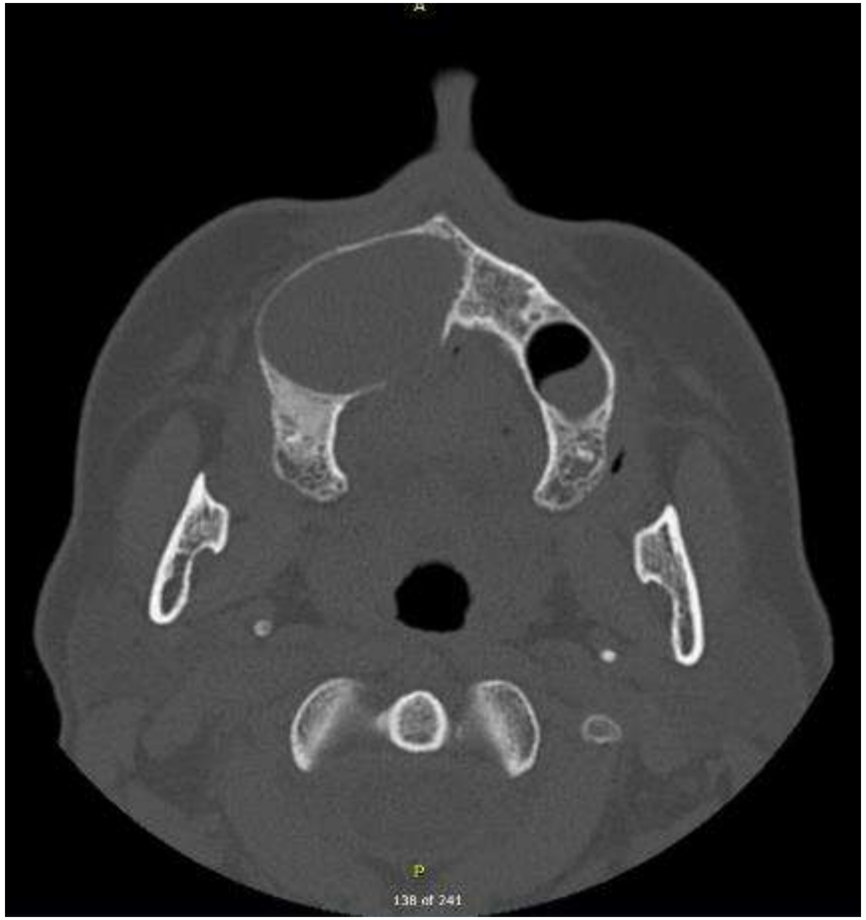

K14 – CT coronal (65, 69, 73, 86 of 213 slices)

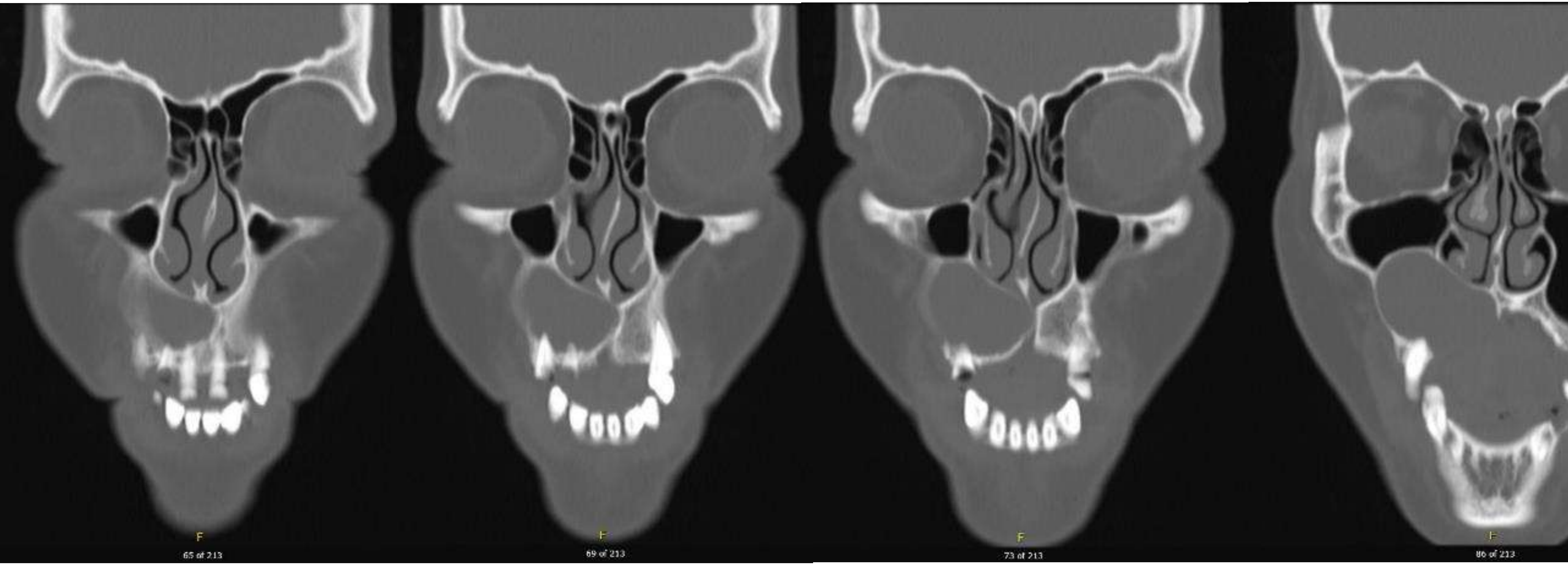

K14 – CT sagittal (124, 132, 141 of 239 slices)

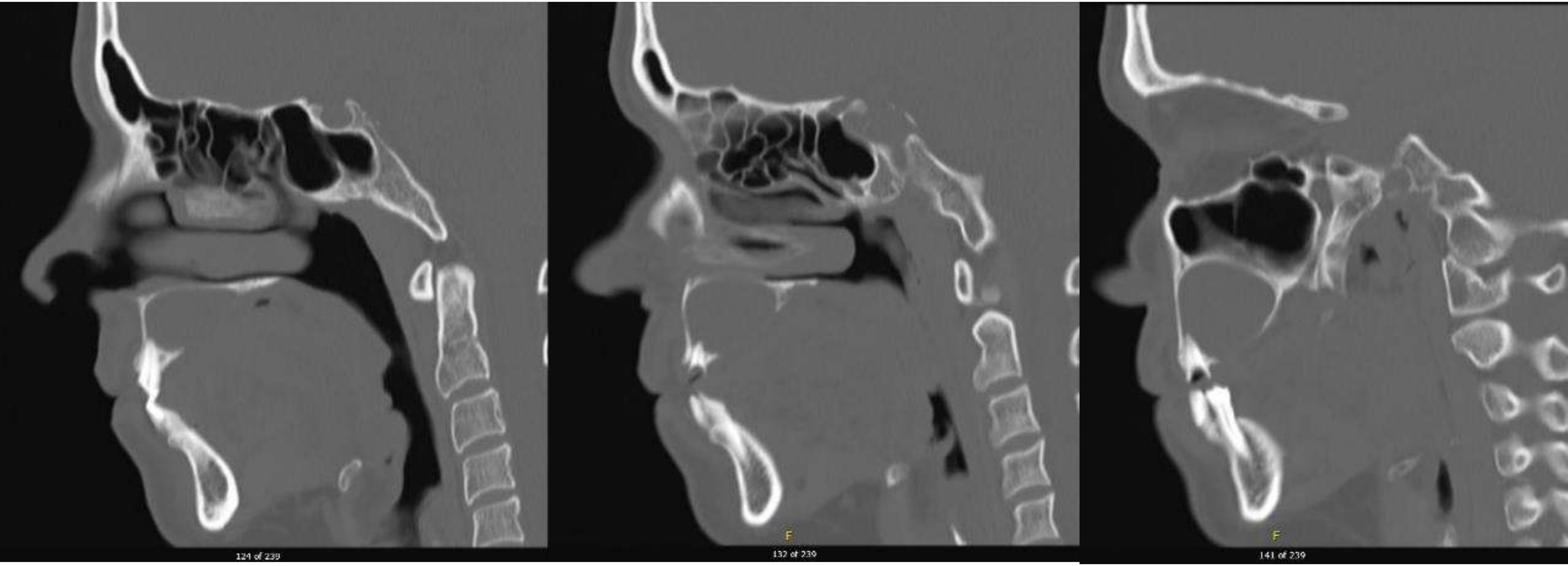

K14 – HE, CK17, bcl2 (x0.8)

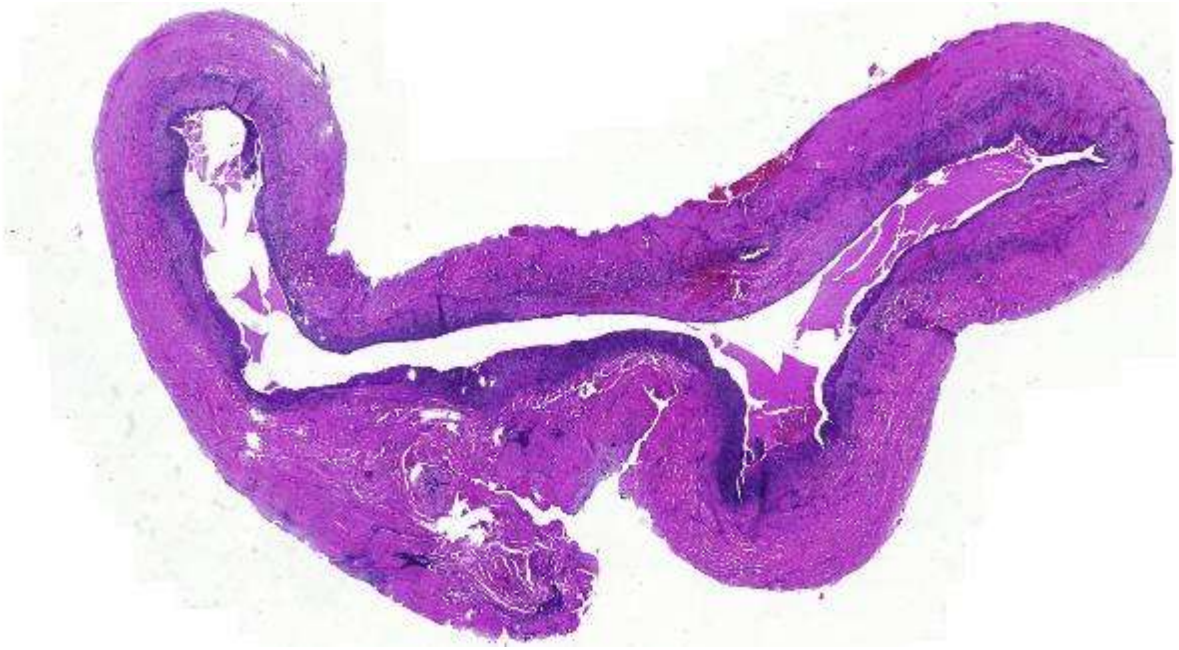

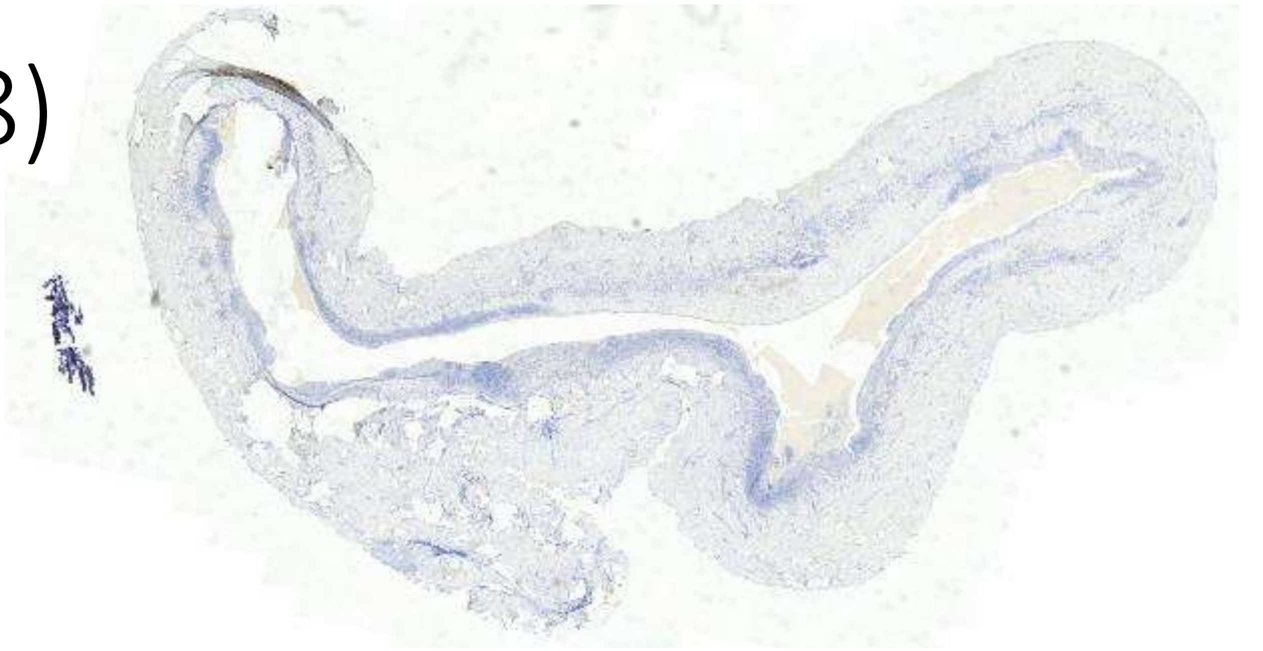

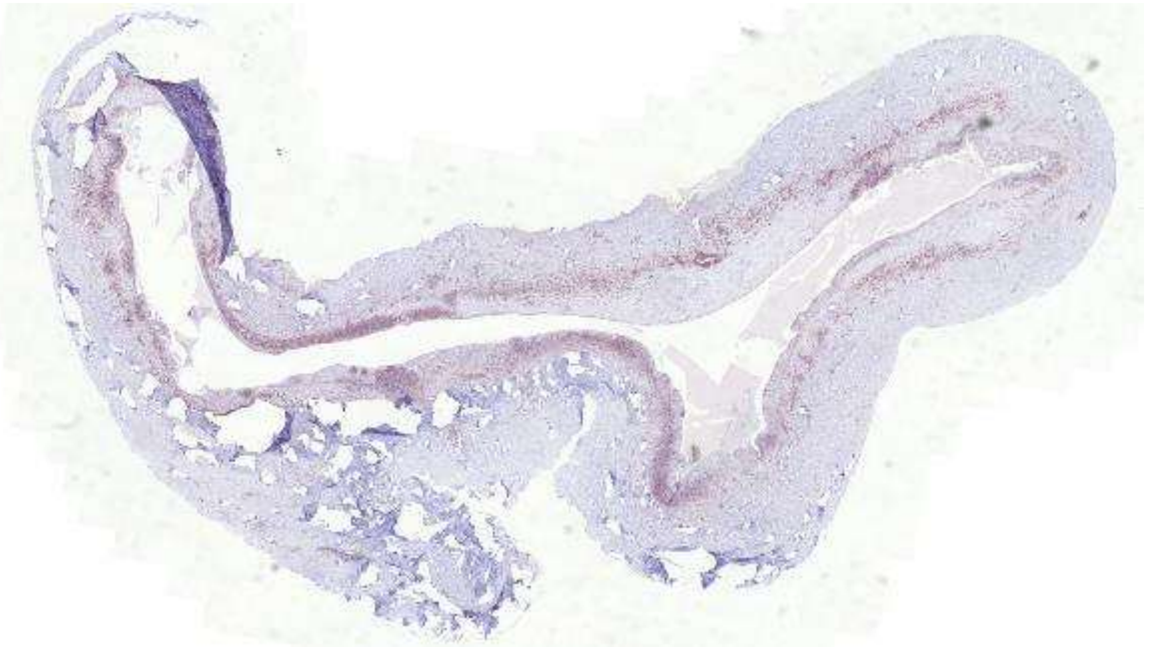

K14 – HE, CK17, bcl2 (x5)

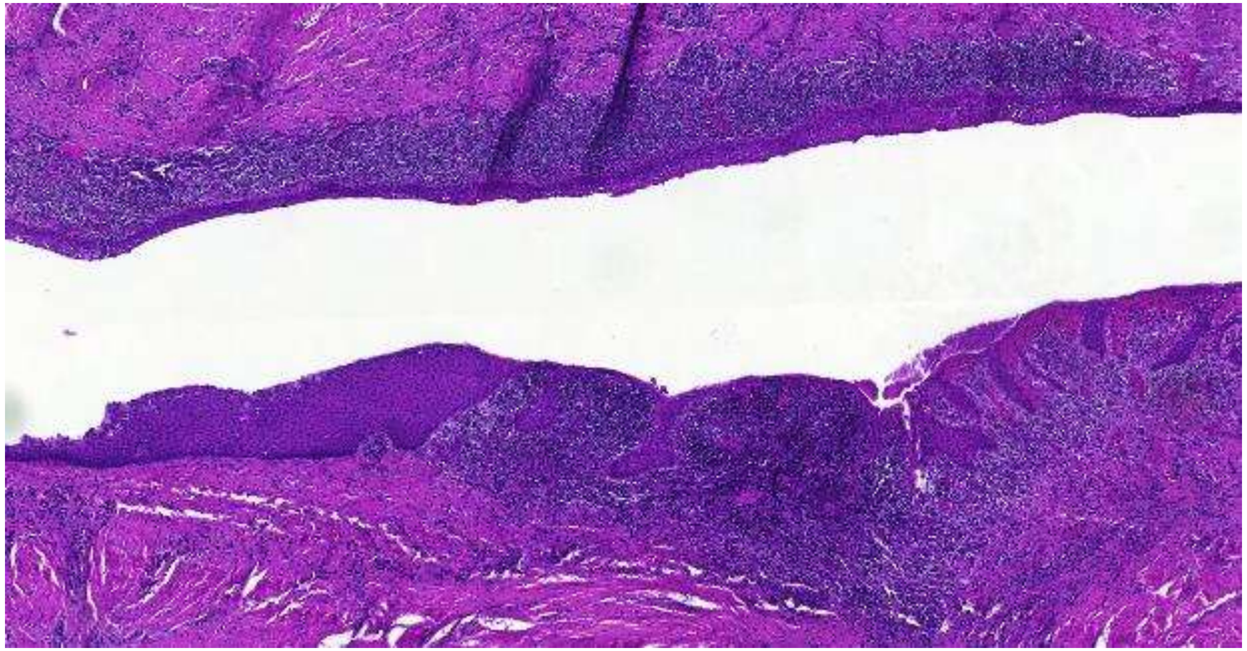

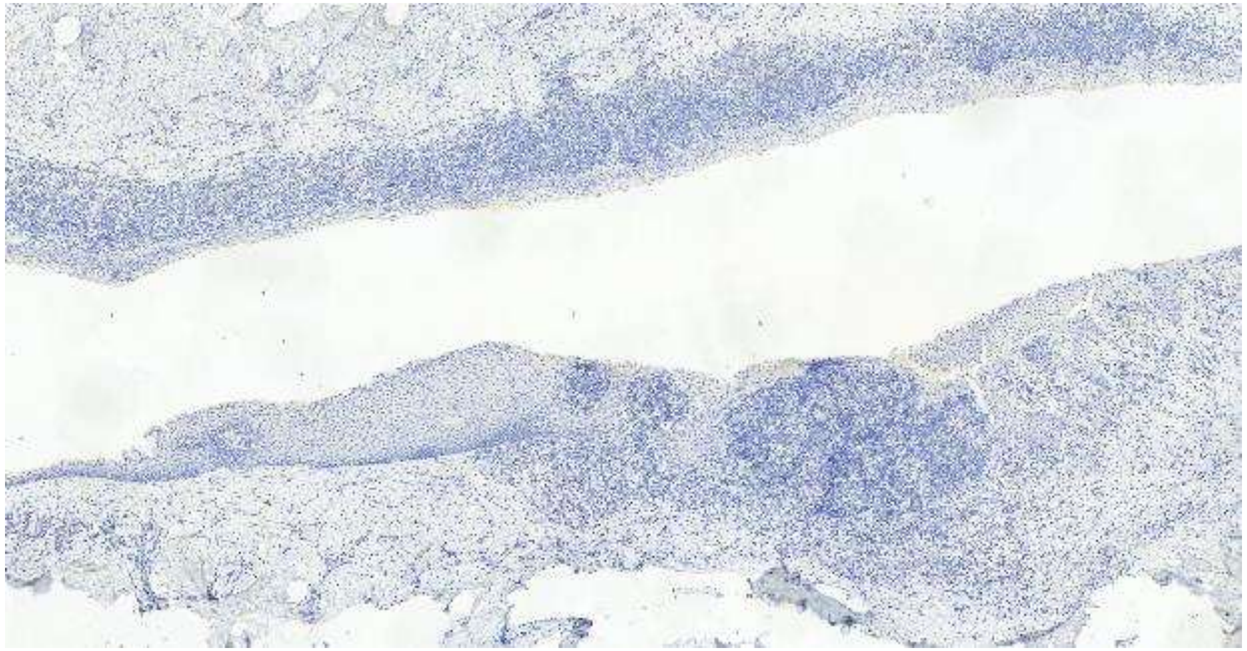

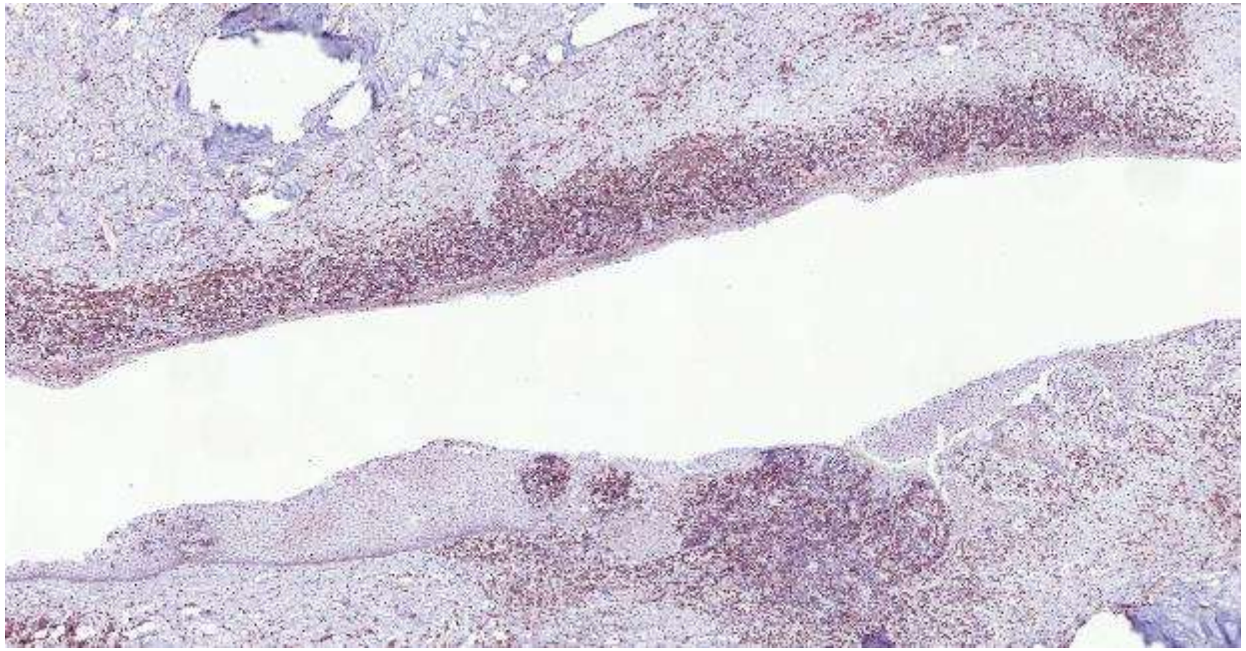

K14 – HE, CK17, bcl2 (x20)

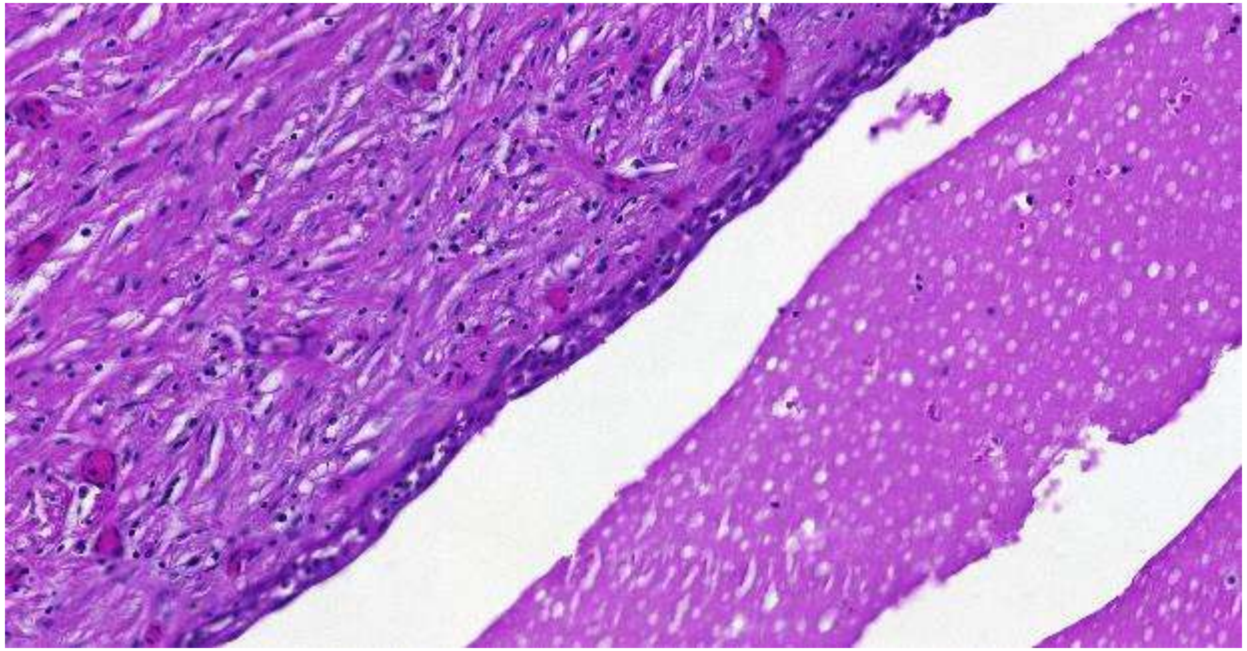

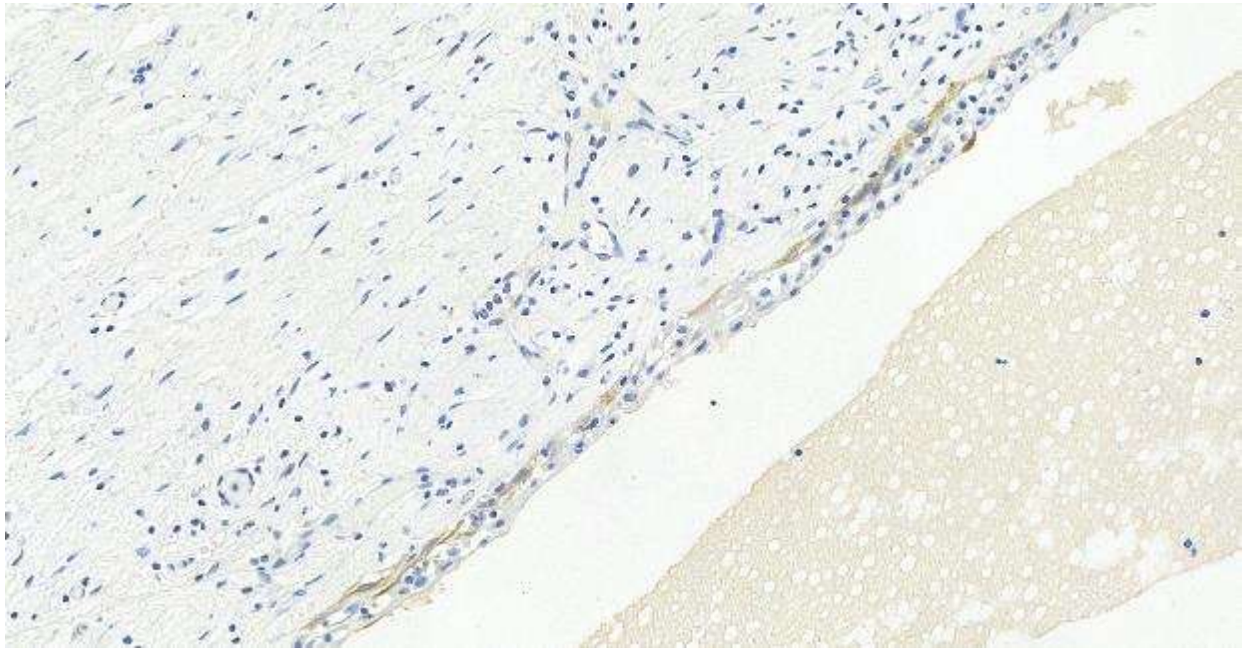

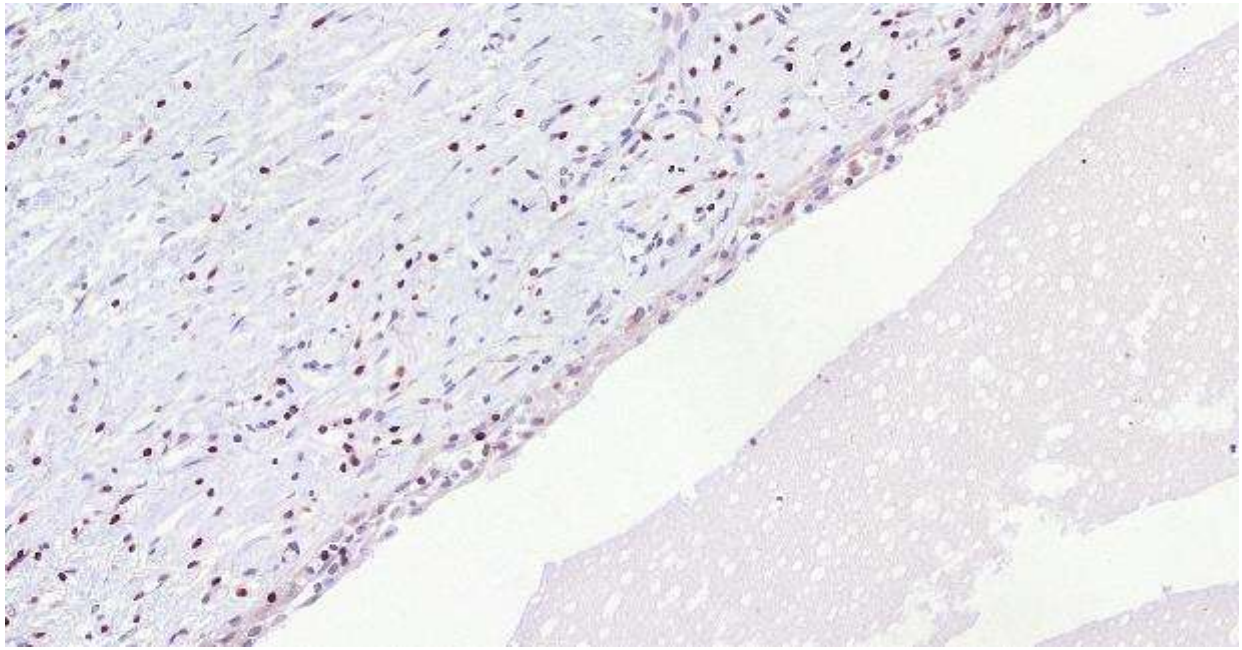

K14 – bcl2 (x25 & x30)

Faint focal basal (/suprabasal) staining versus background – note the intense staining of lymphocytes

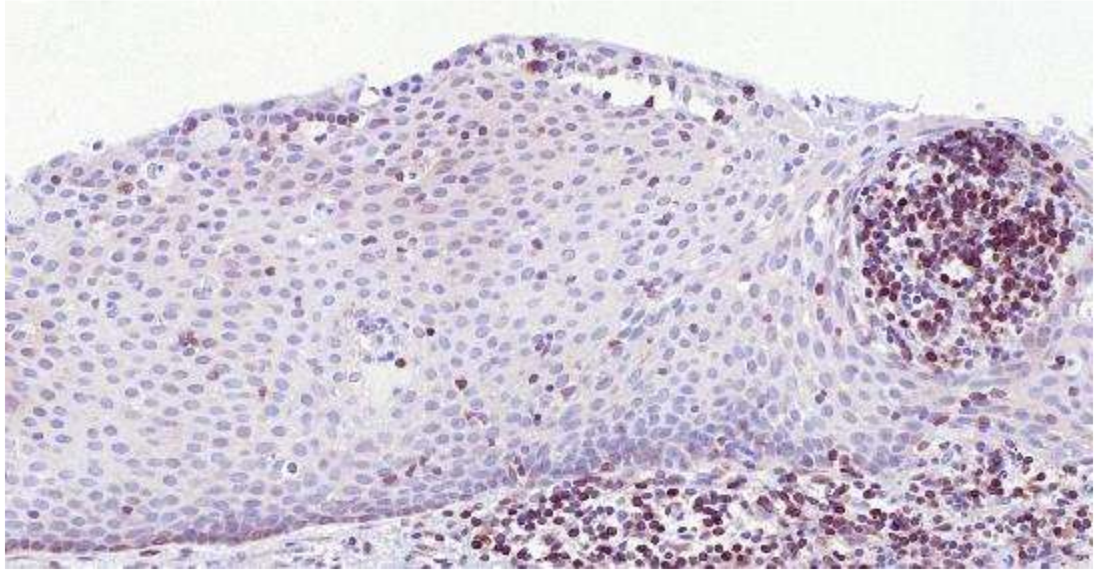

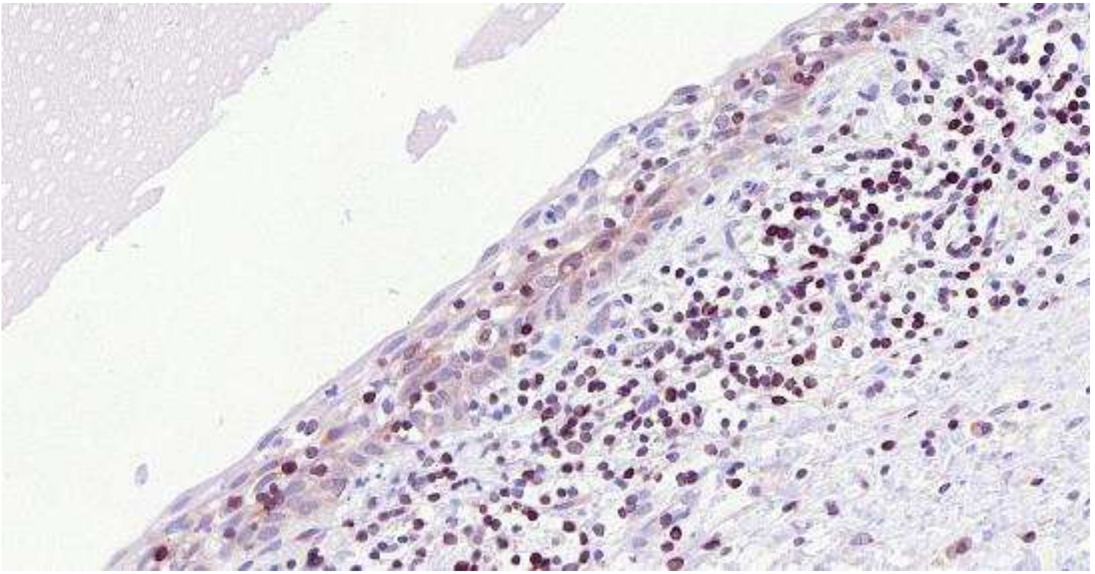

K14 – Conclusion

- H&E: partly thick, partly thin squamous epithelium, no parakeratosis, no wavy surface, no basal palisading, uneven (partly even) epithelial-subepithelial contours; focal mucous metaplasia
- Focal mixed inflammation of the cyst wall which is generally not infalmed
- Bcl2: negative (uncertain faint basal (/suprabasal) staining incomparable with control lymphocyte positivity – or non specific background staining)
- CK17: dominantly negative, minimal focal basal and/or superficial (nearly transepithelial) staining
- Histopathology: inflammatory odontogenic cyst
- Combined clinical and pathological opinion: large mature radicular cyst

Case K15

- 65-year-old female
- History: asymptomatic
- Dental hygiene: normal, partly conserved, treated teeth
- Radiology: Complete impaction of horizontal tooth 38; radiolucent lesion of 22 mm in greatest dimension around the crown of this tooth, expanding to the distal root of tooth 36.
- Operation: decoronation and removal (in two pieces) of tooth 38 followed by removal of the inflamed cyst wall
- Clinical diagnosis: dentigerous (follicular) cyst

K15 – OPT

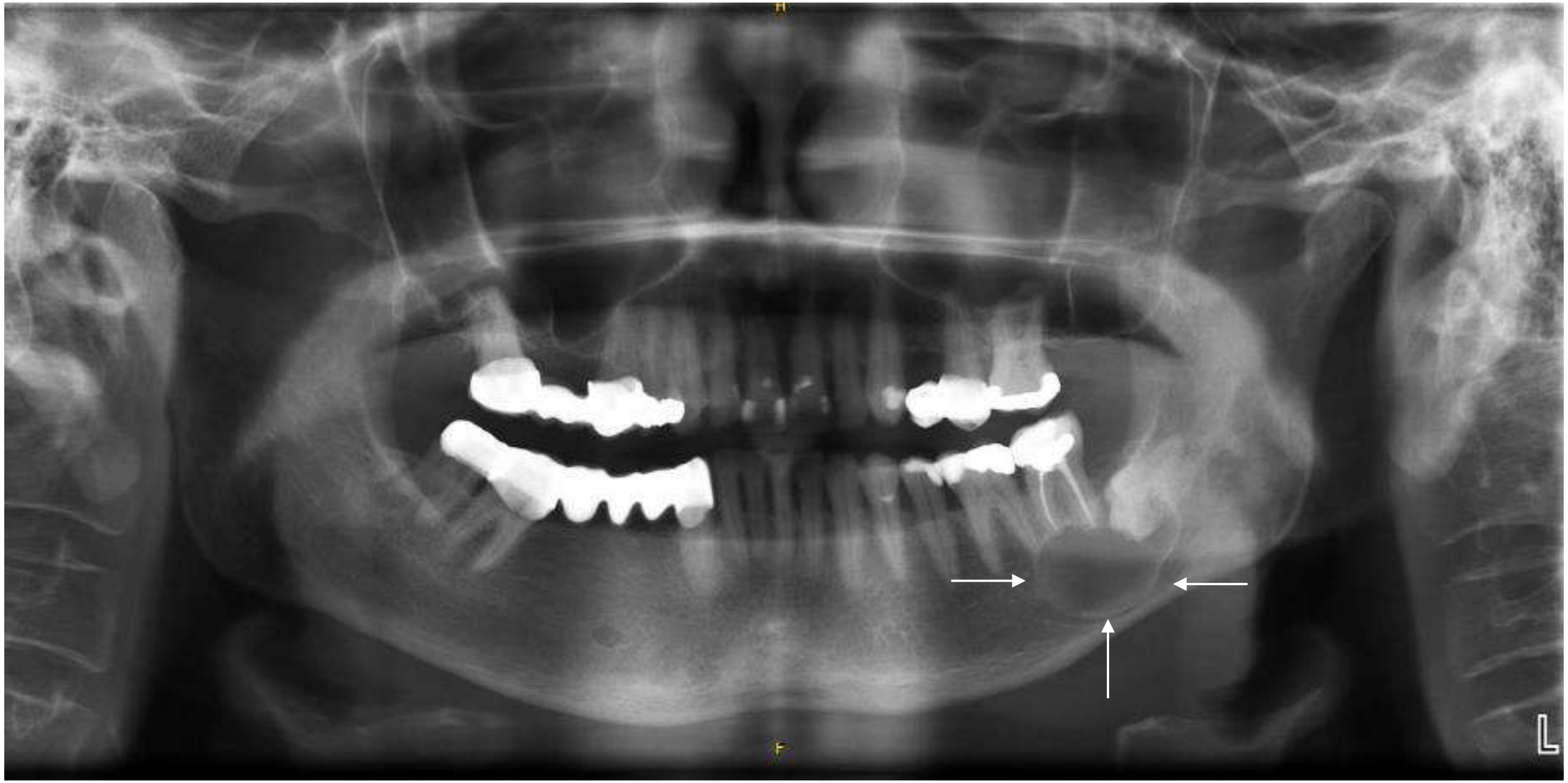

K15 – OPT magnified

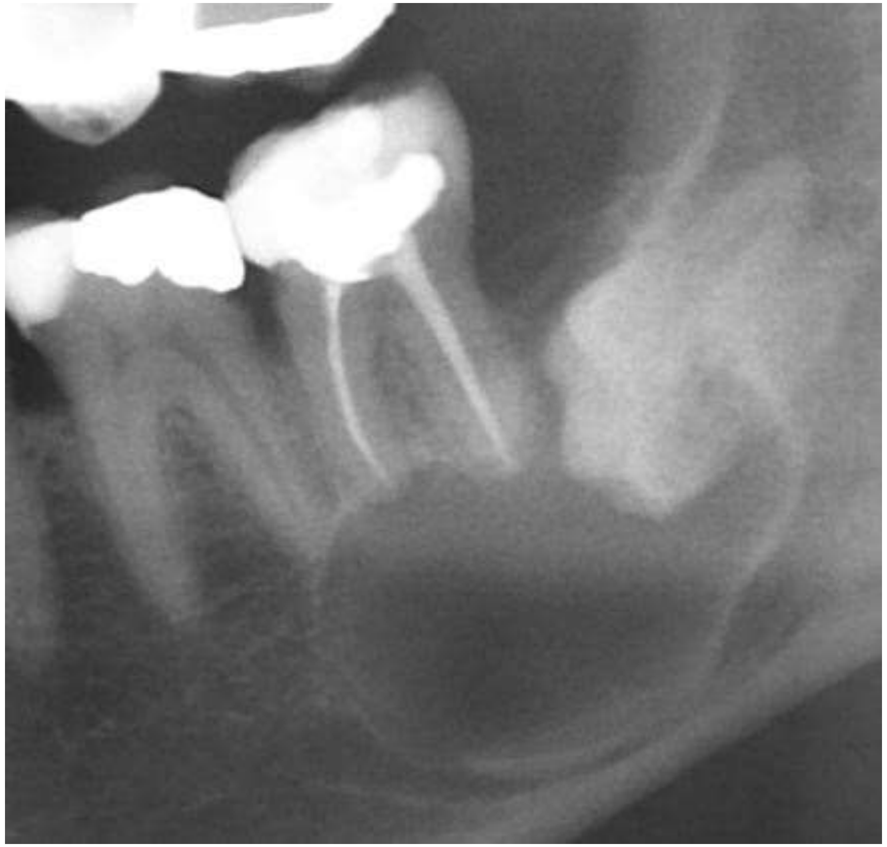

K15 – CT Summary

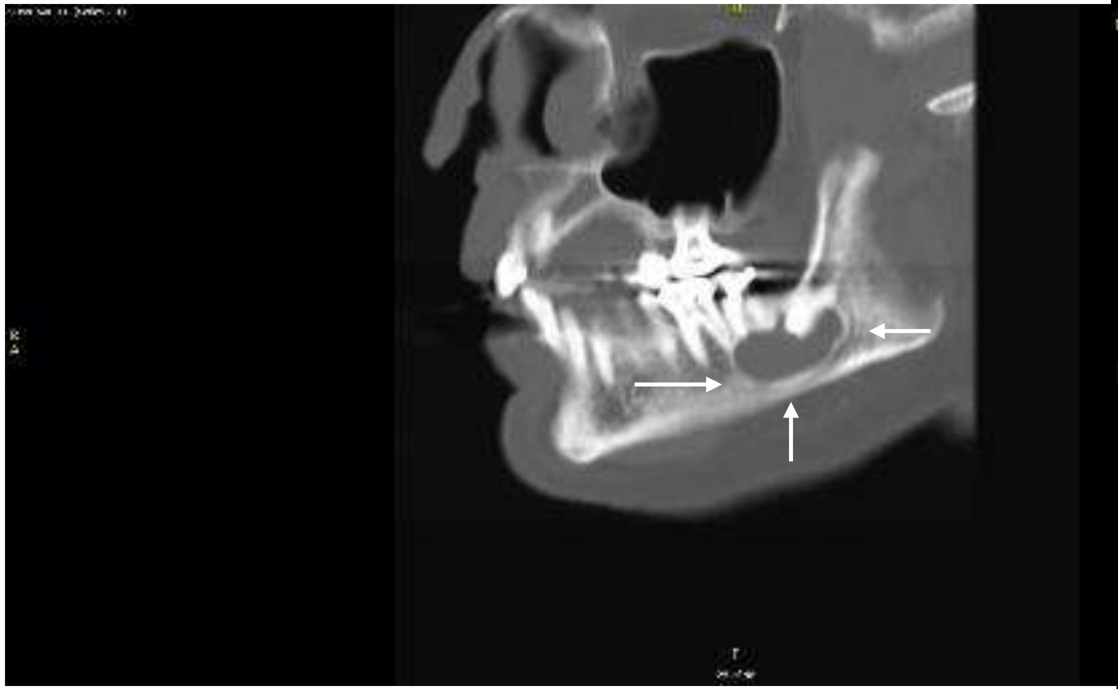

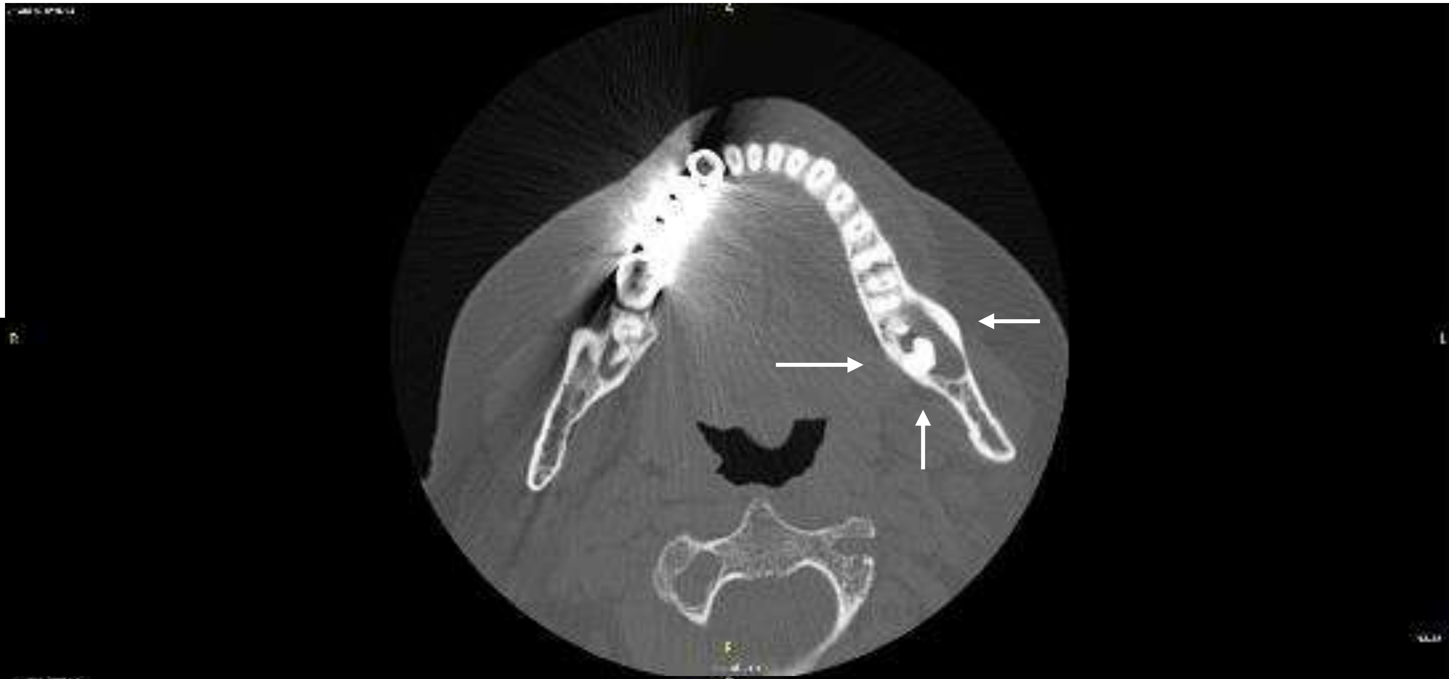

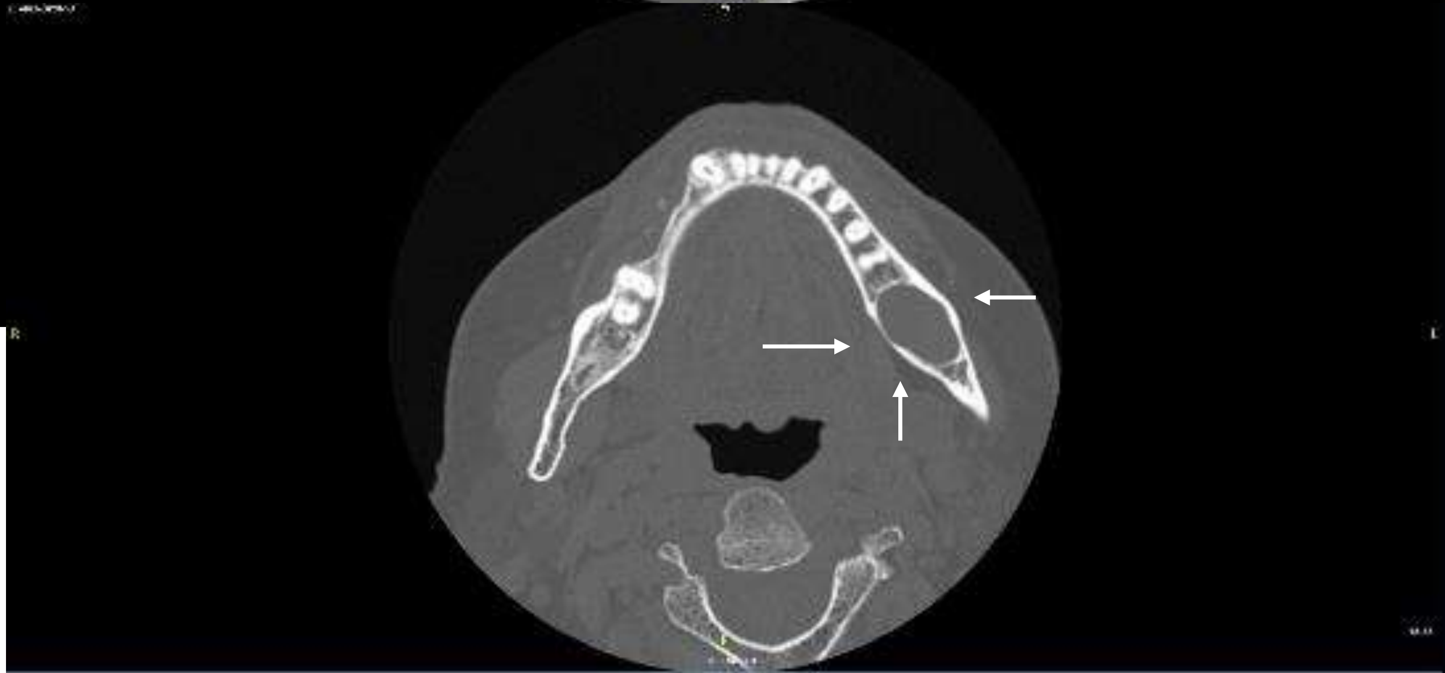

K15 – CT axial

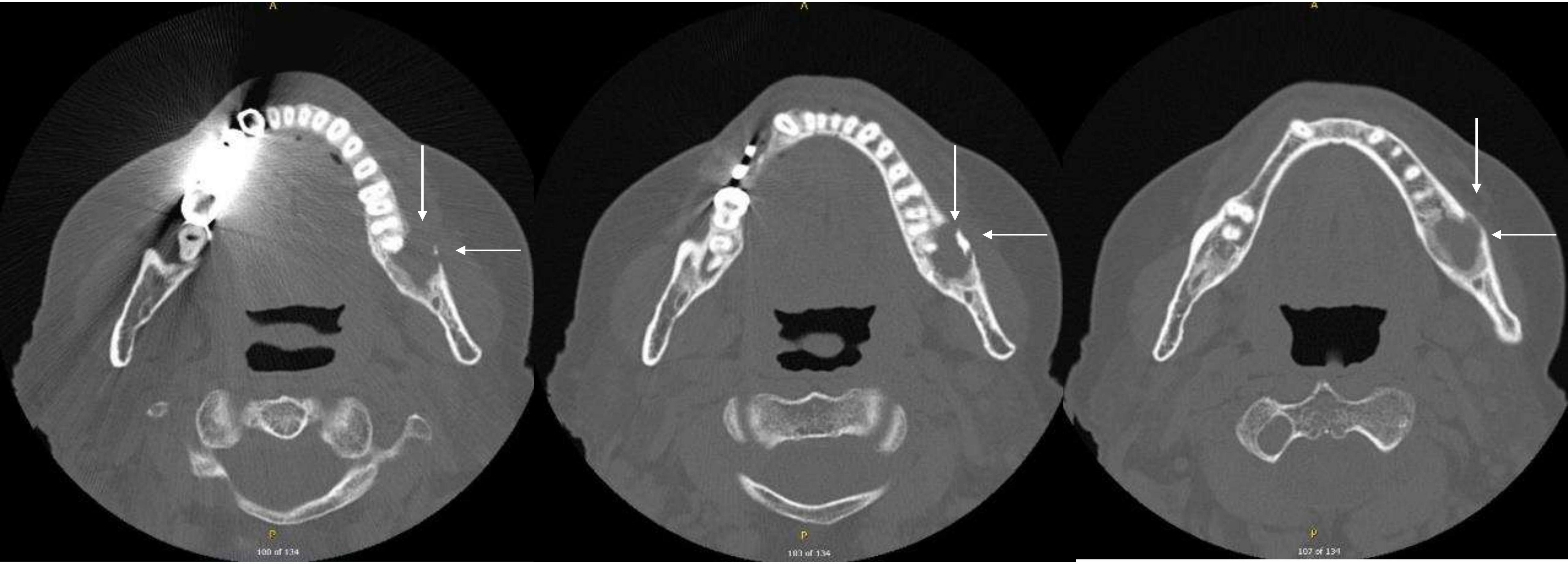

K15 – CT coronal

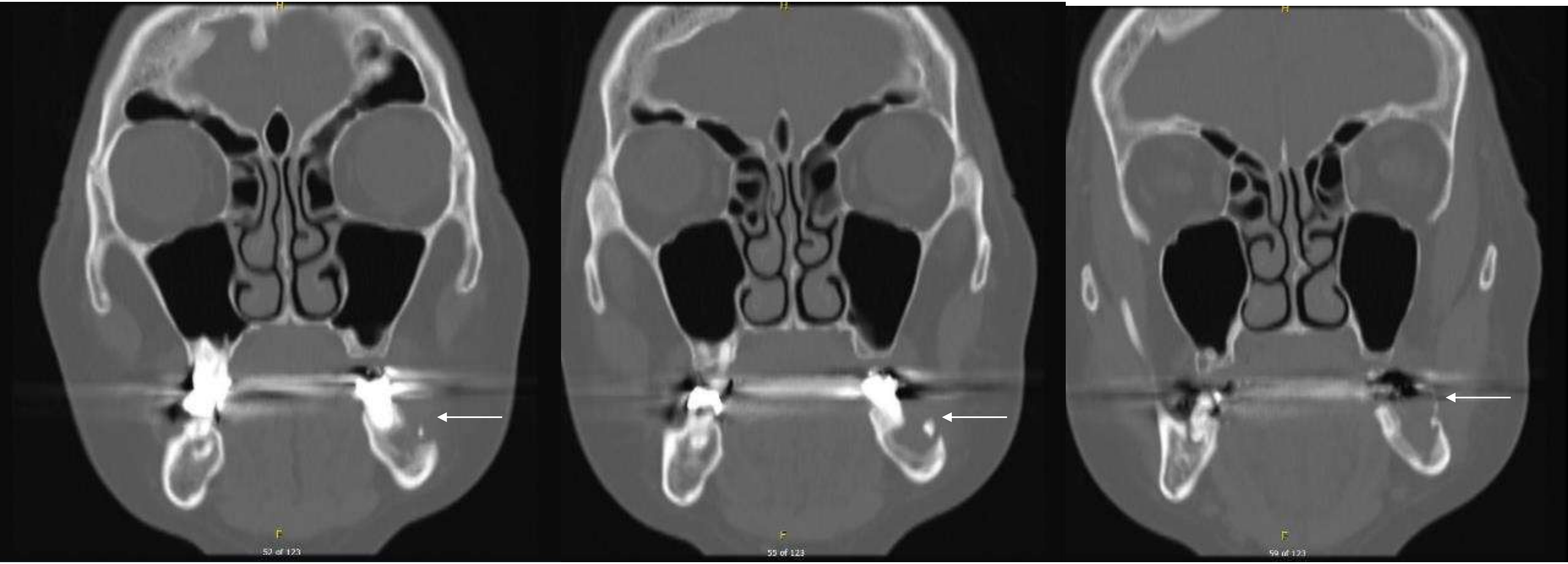

K15 – CT sagittal

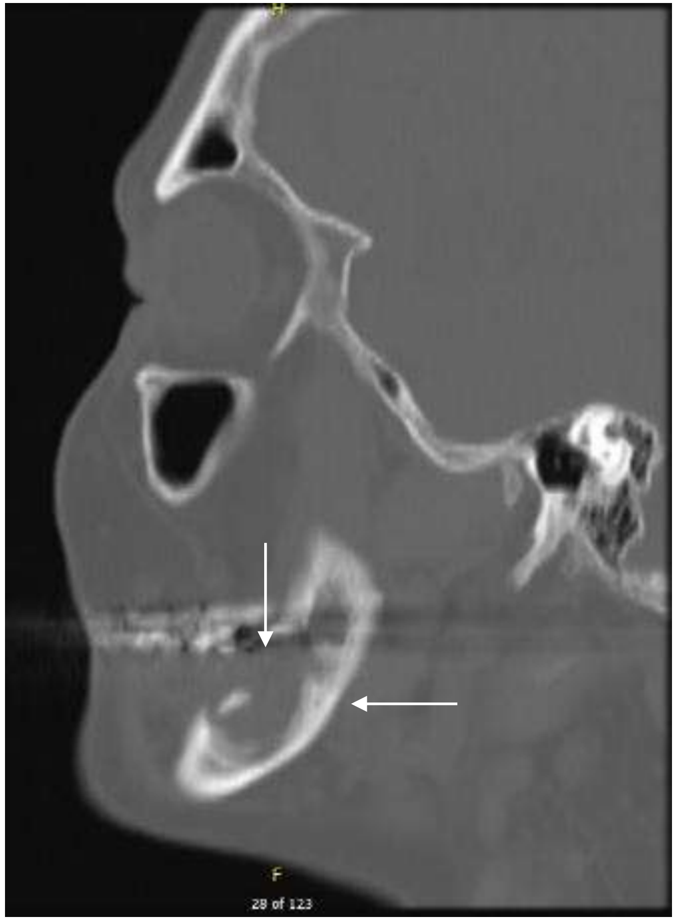

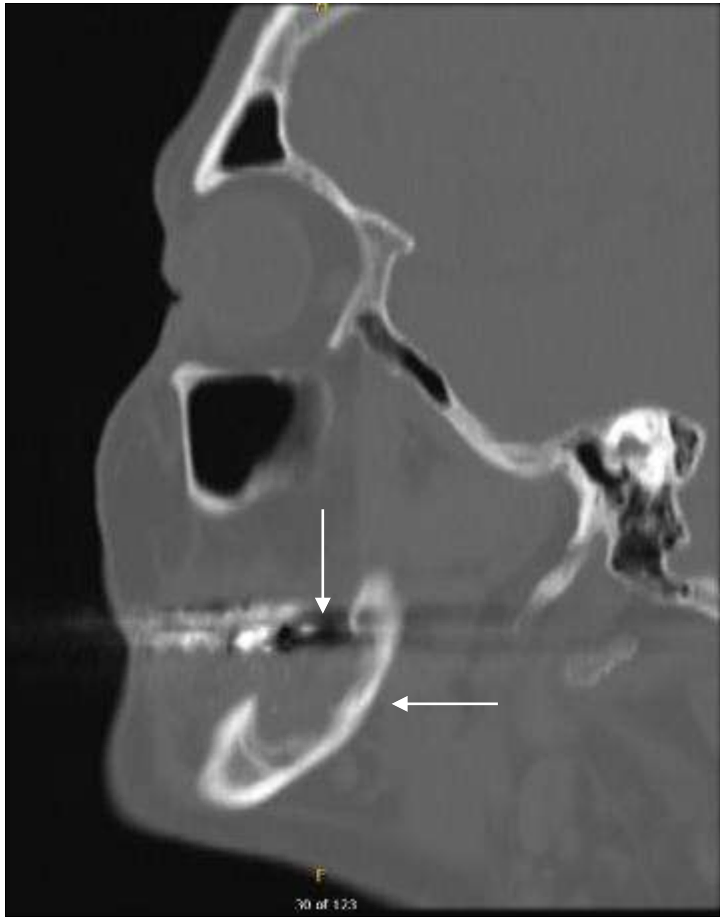

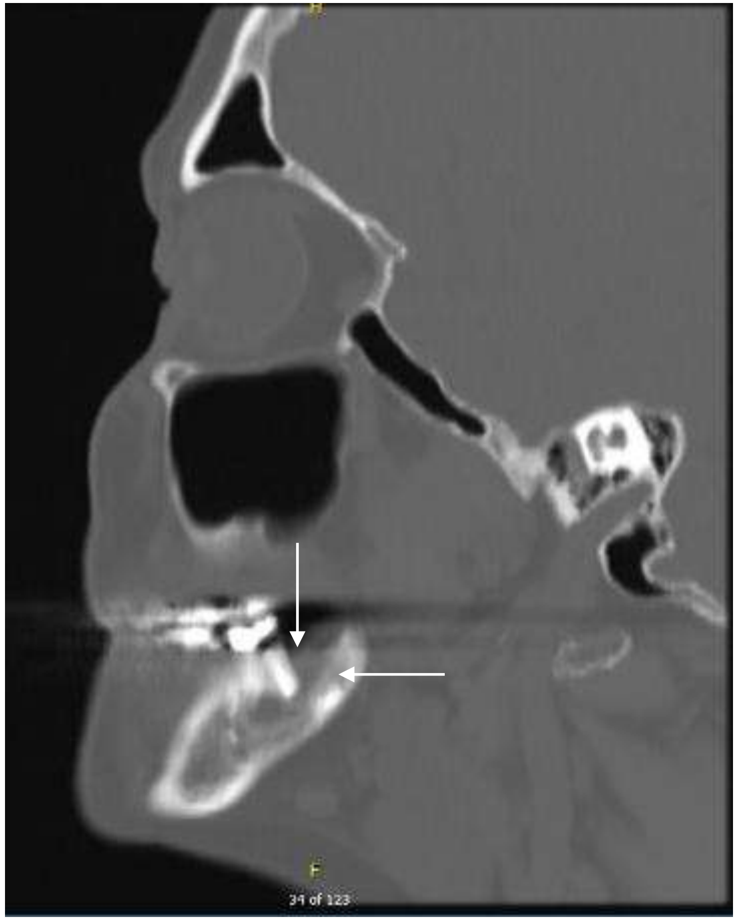

K15 – HE, CK17, bcl2 (x1.2)

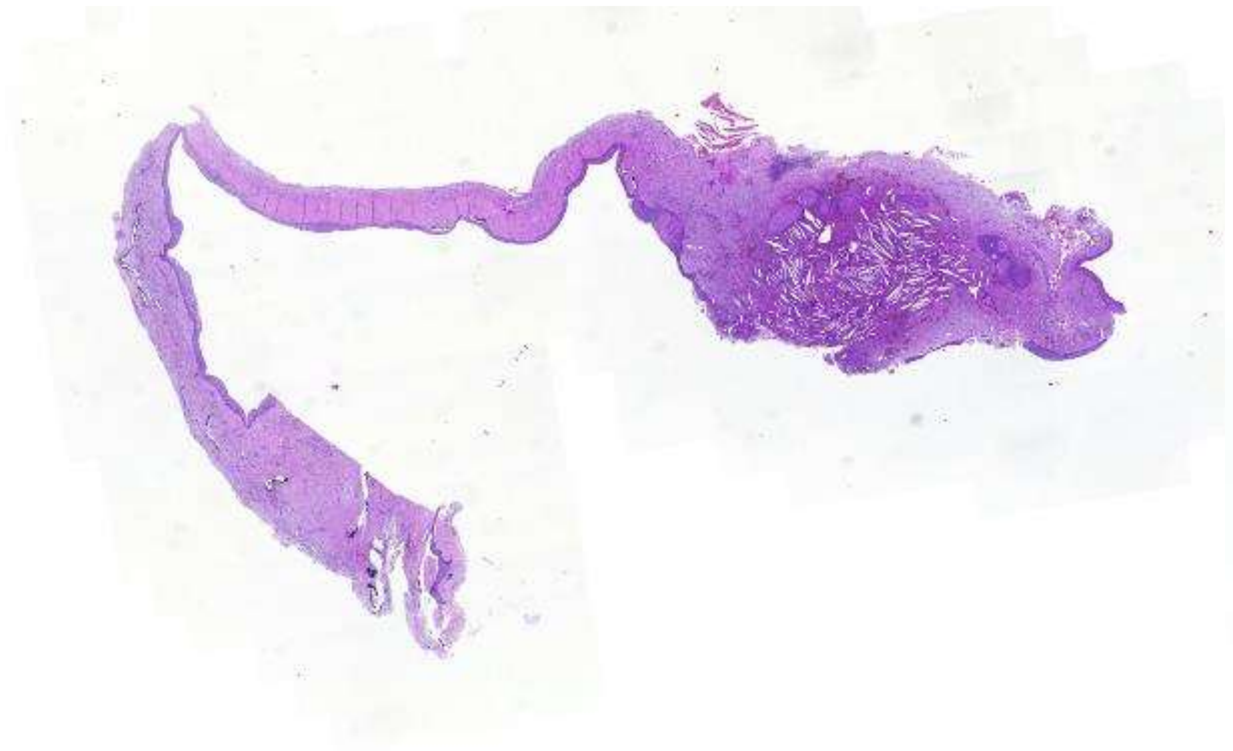

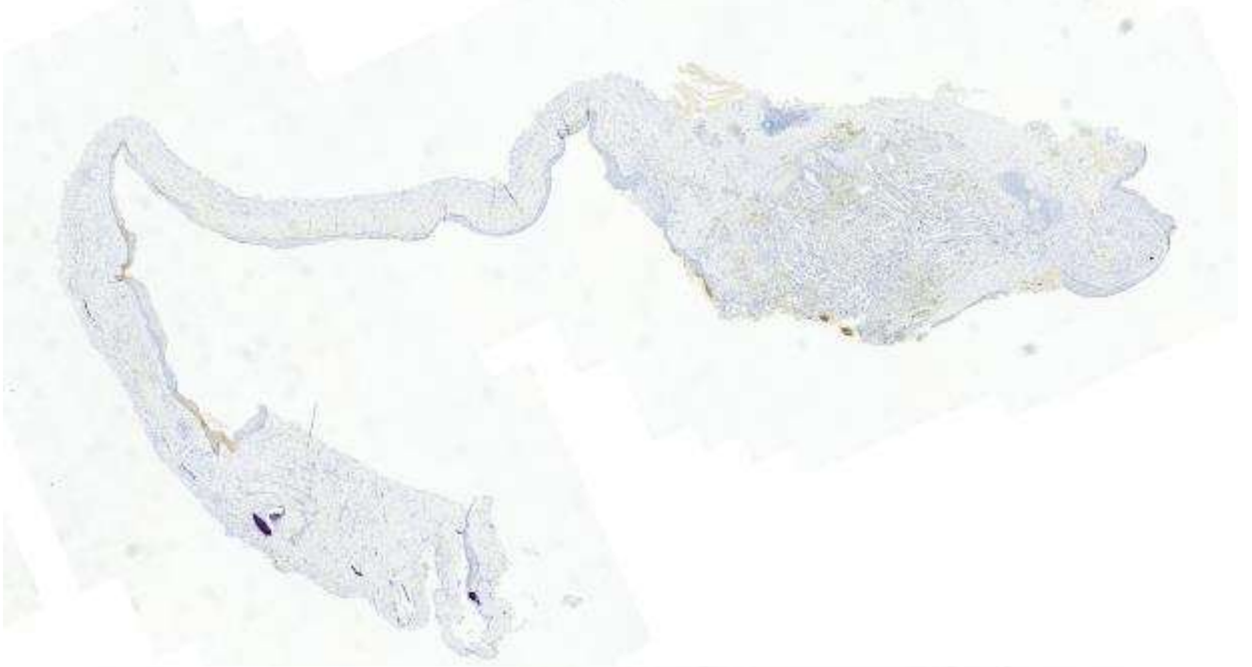

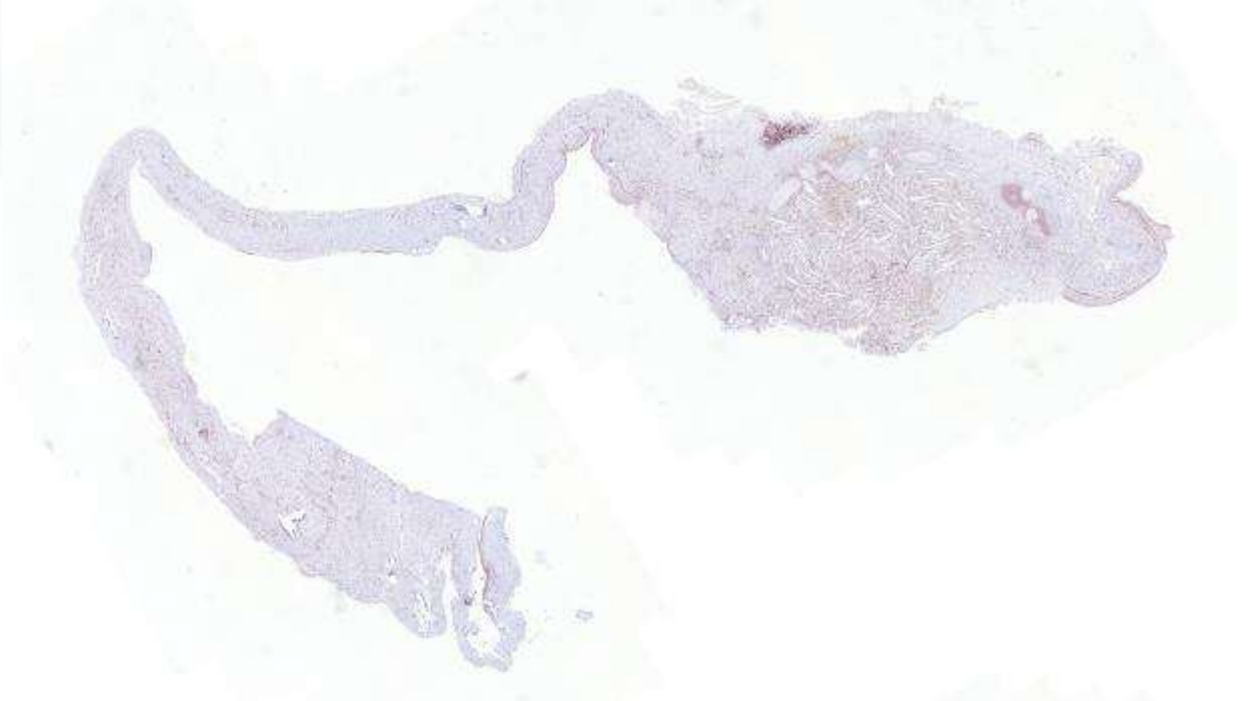

K15 – HE, CK17, bcl2 (x4)

Focal transepithelial CK17 staining (↑); negative bcl2

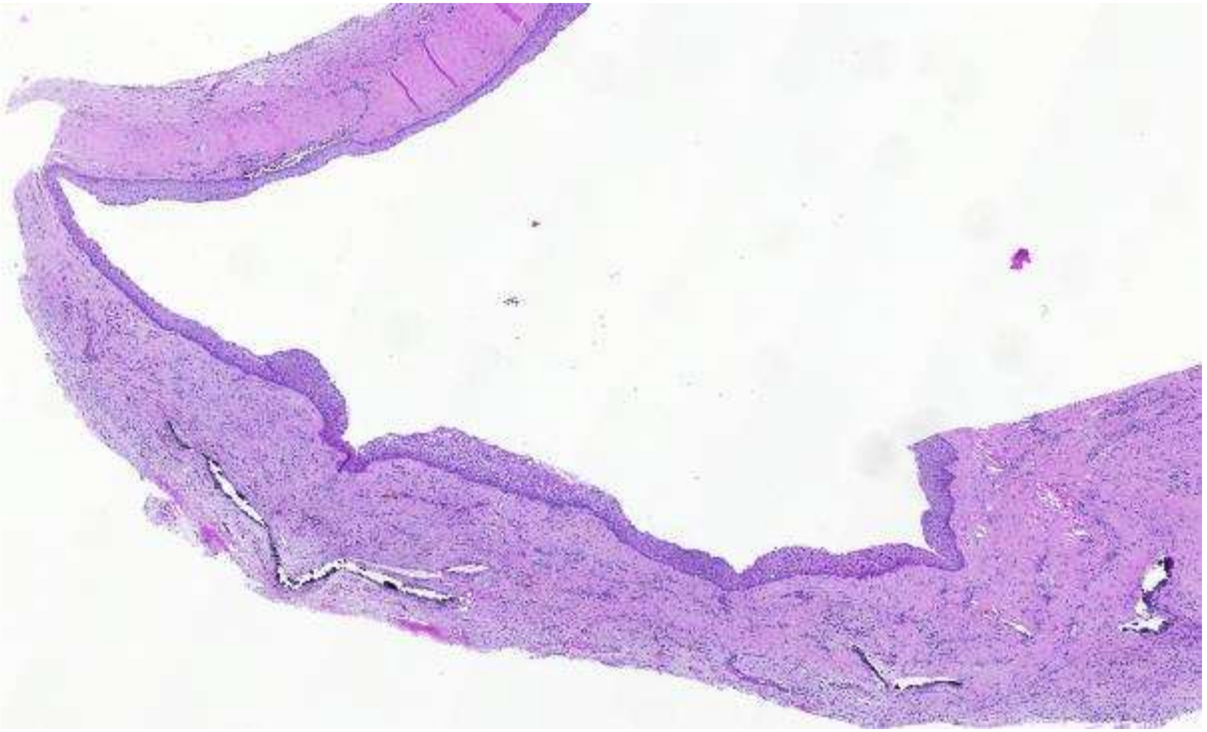

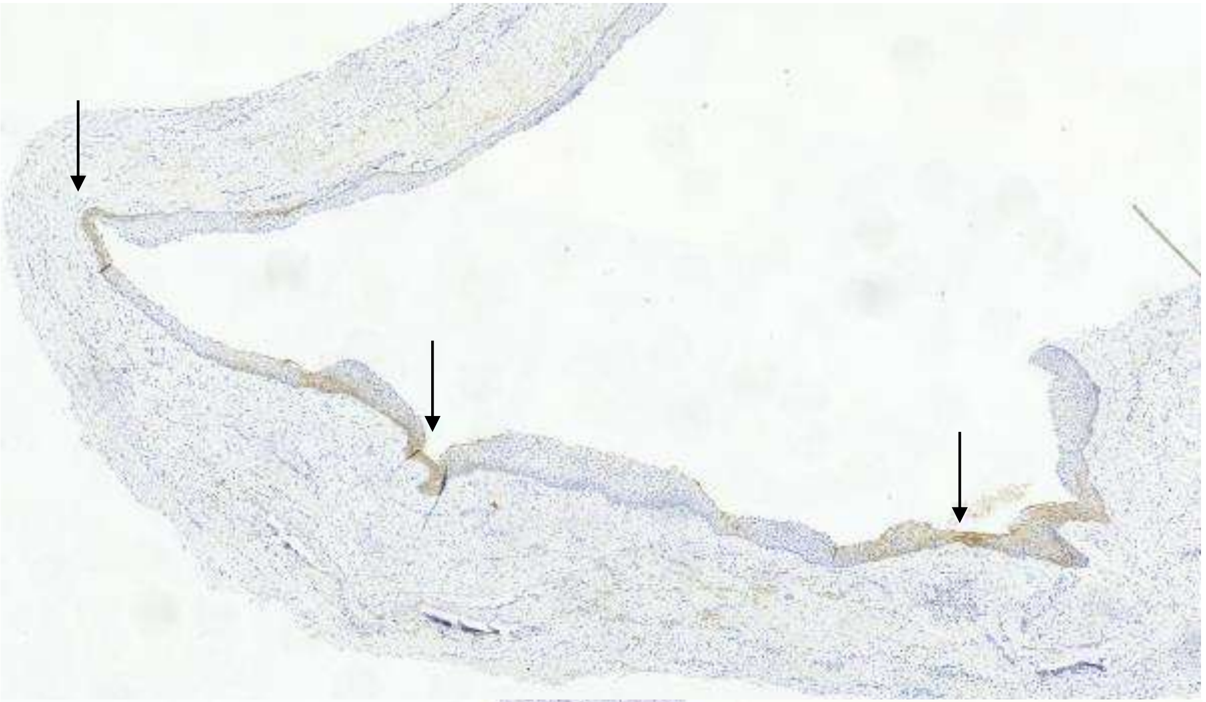

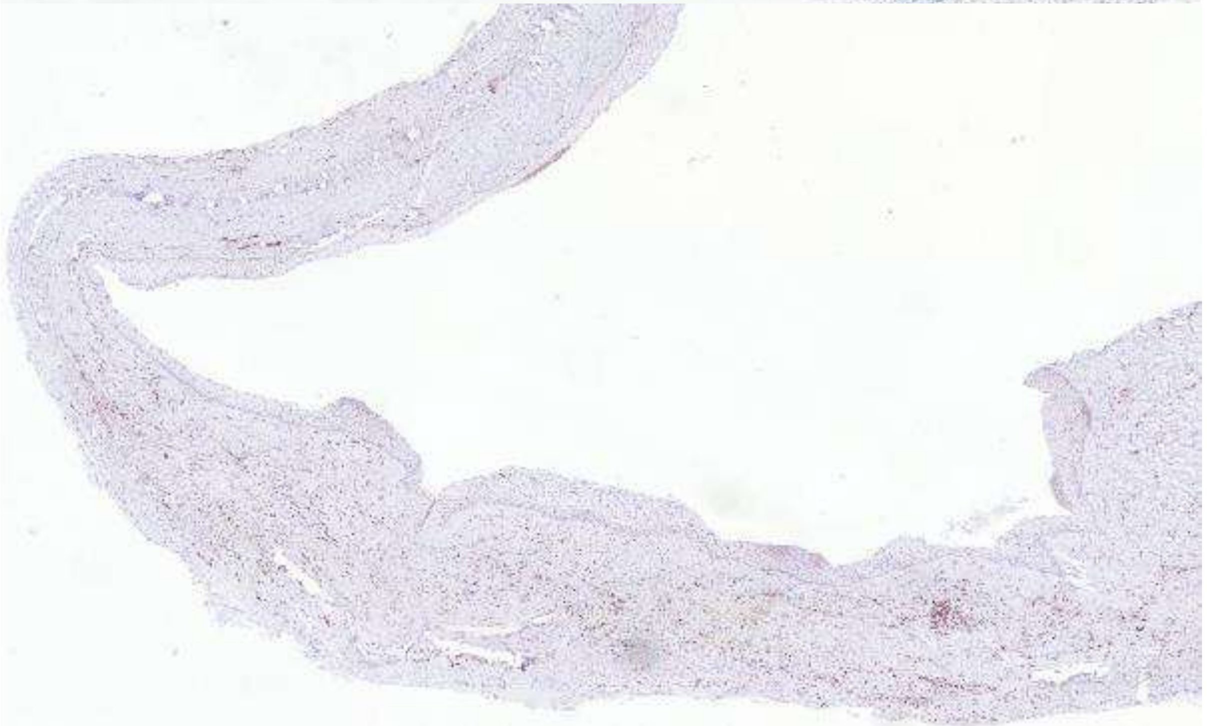

K15 – HE, CK17, bcl2 (x10)

Focal transepithelial (↑), basal (*), superficial CK17 staining (↓); negative bcl2

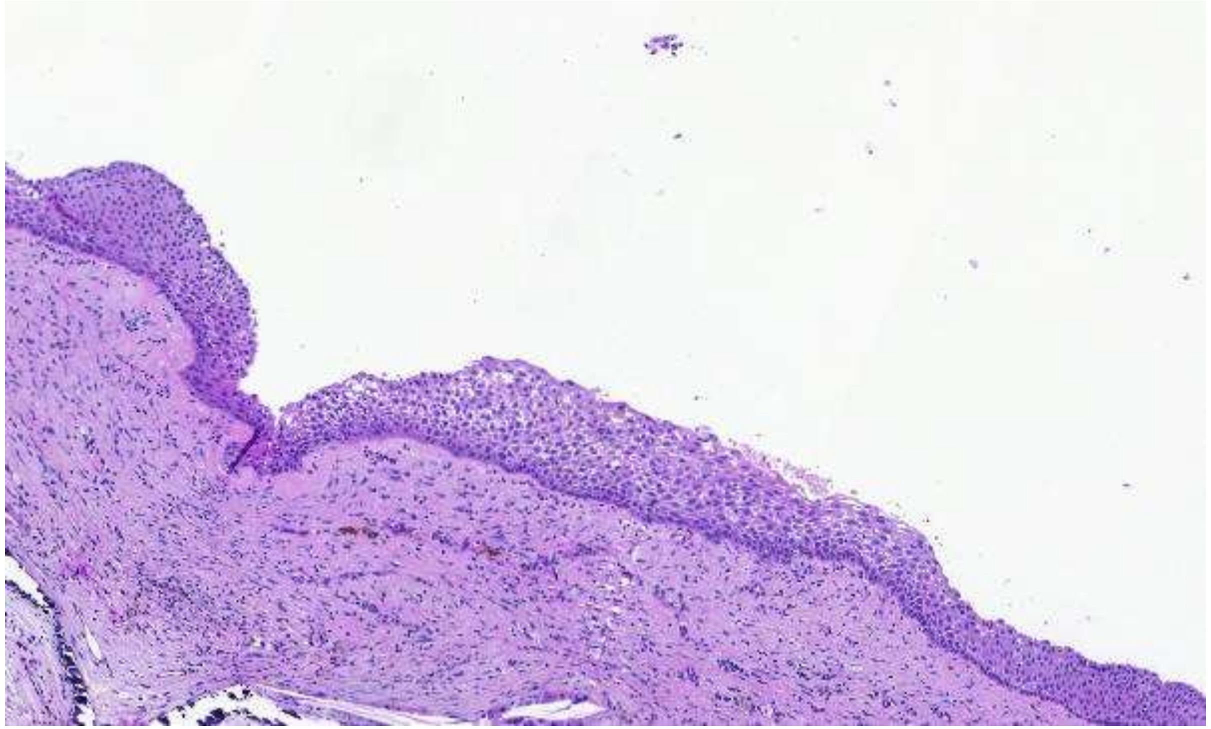

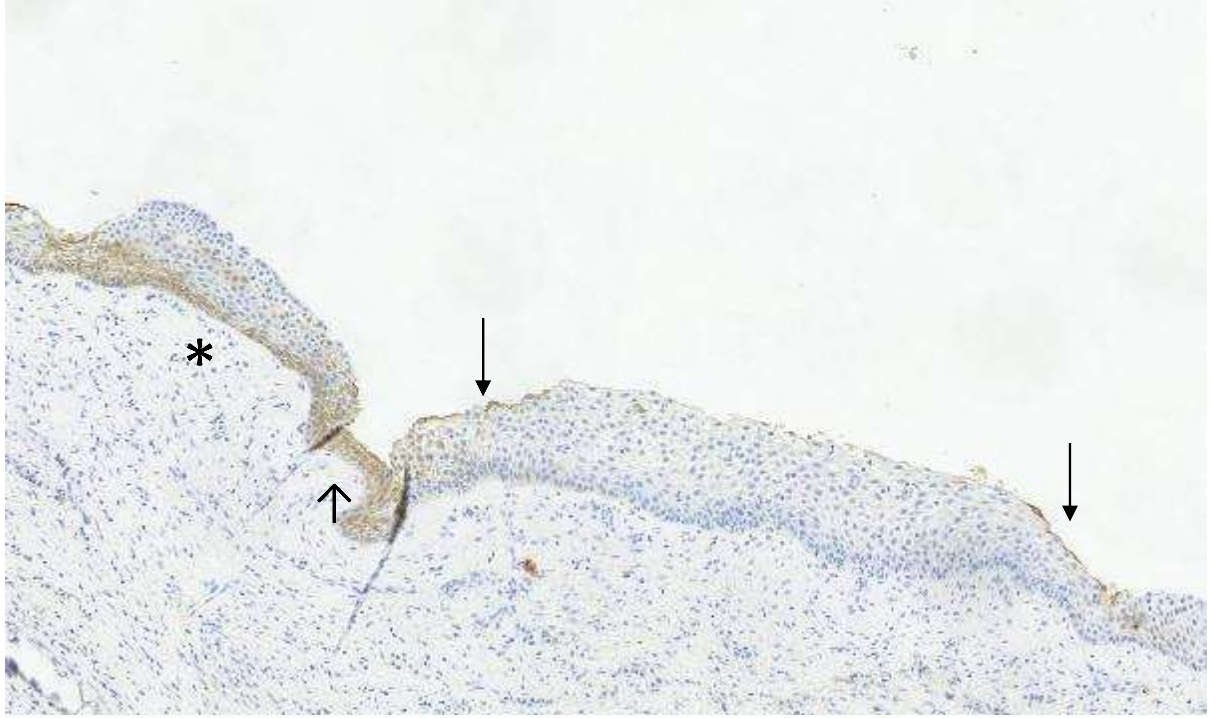

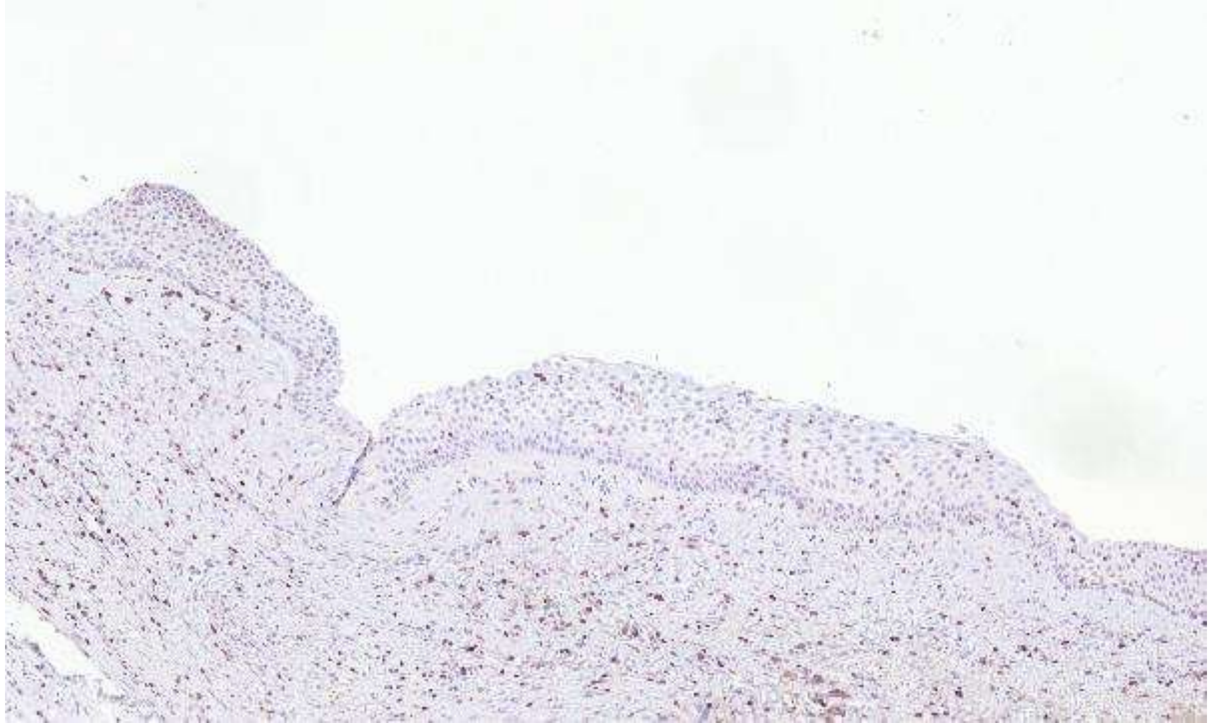

K15 – HE, CK17, bcl2 (x7)

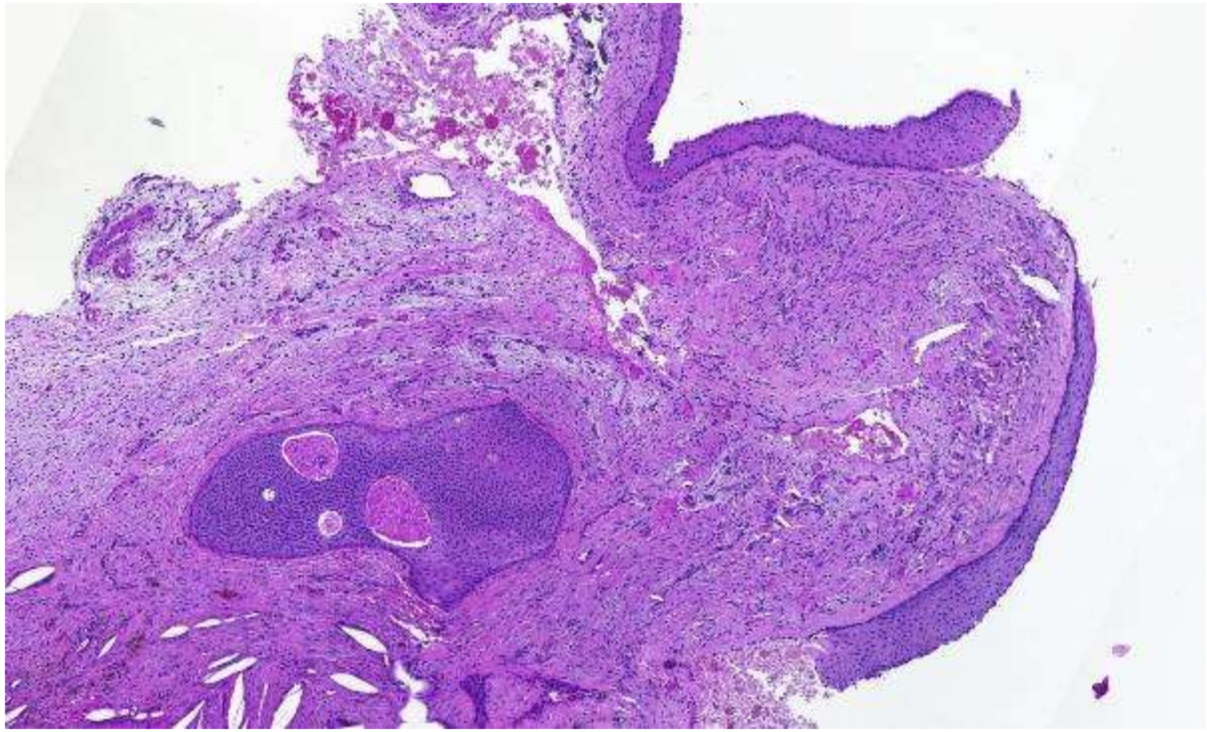

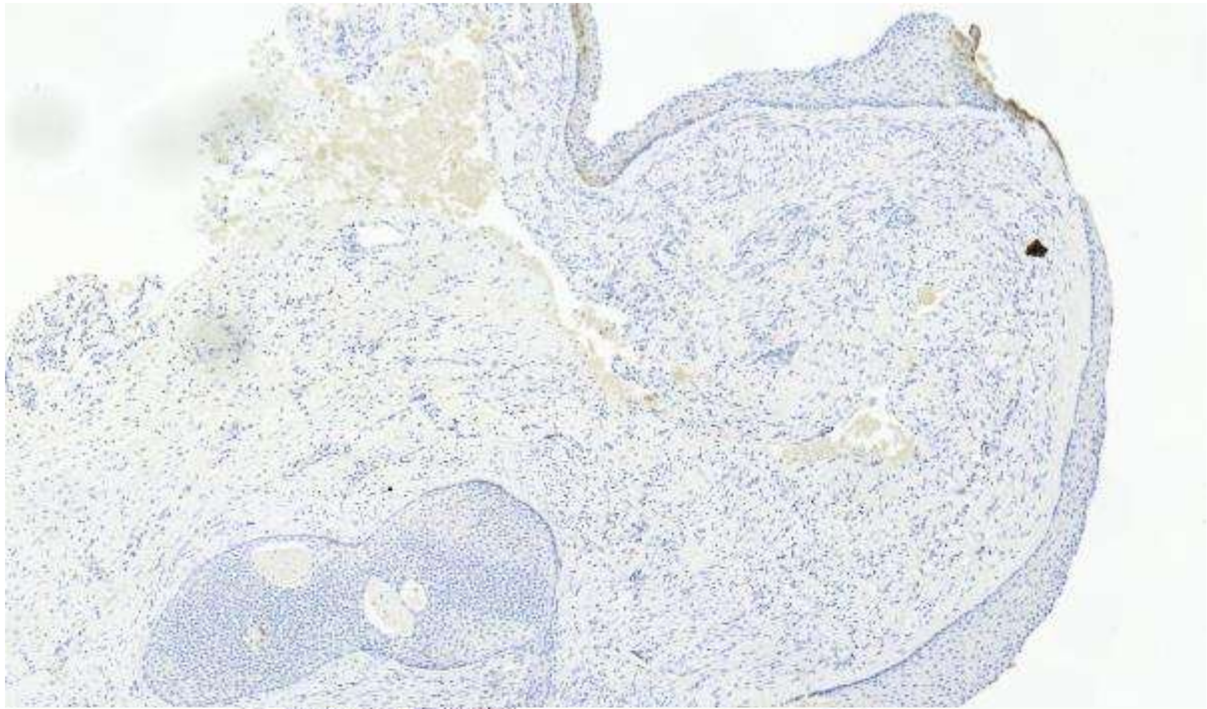

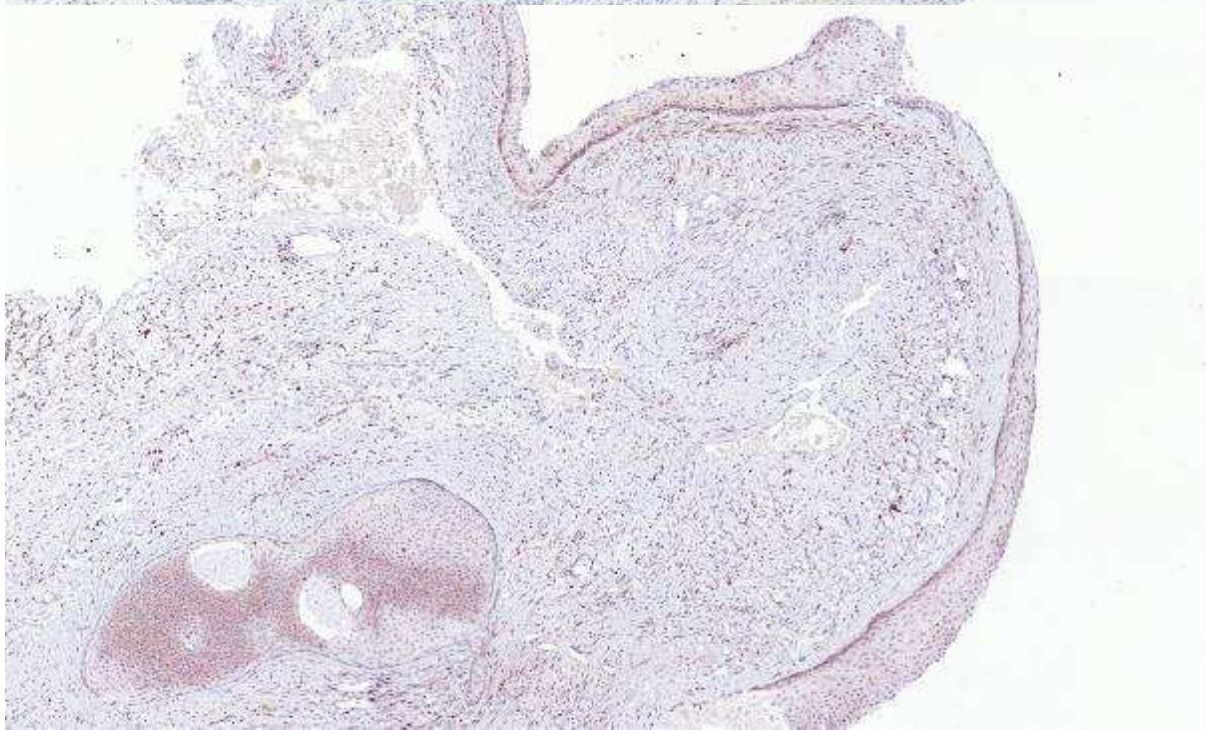

K15 – HE, CK17, bcl2 (x12)

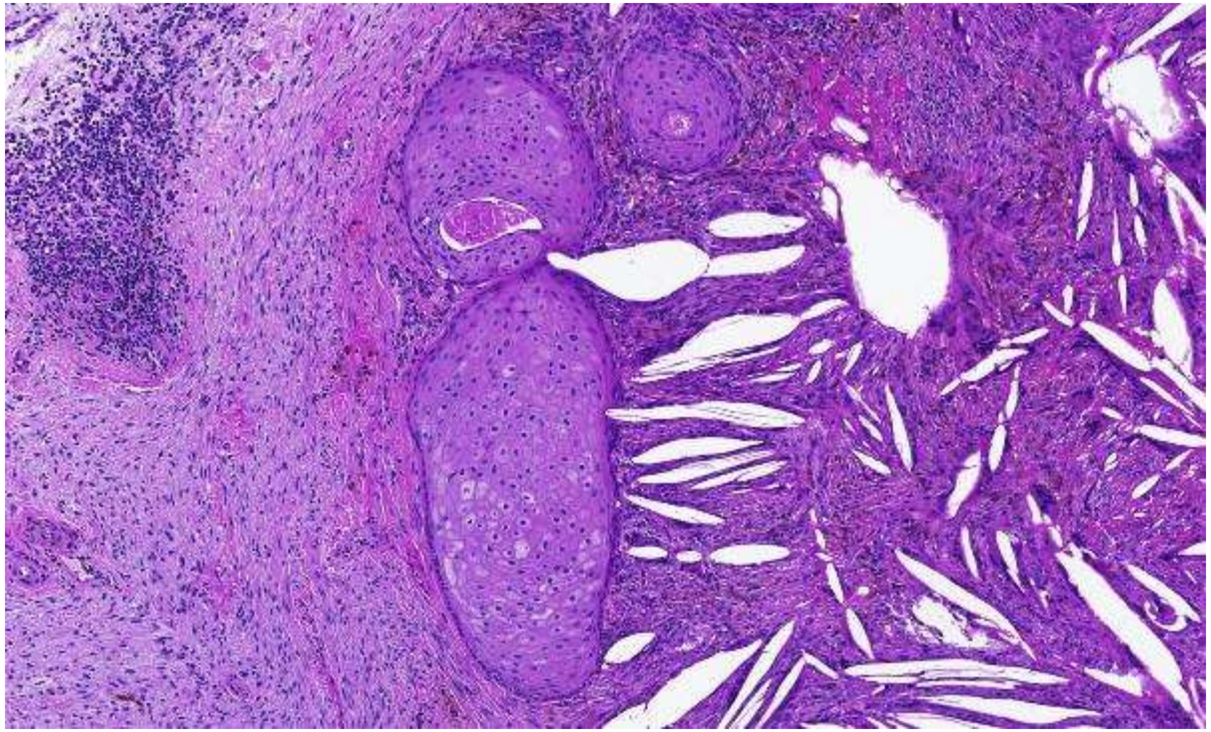

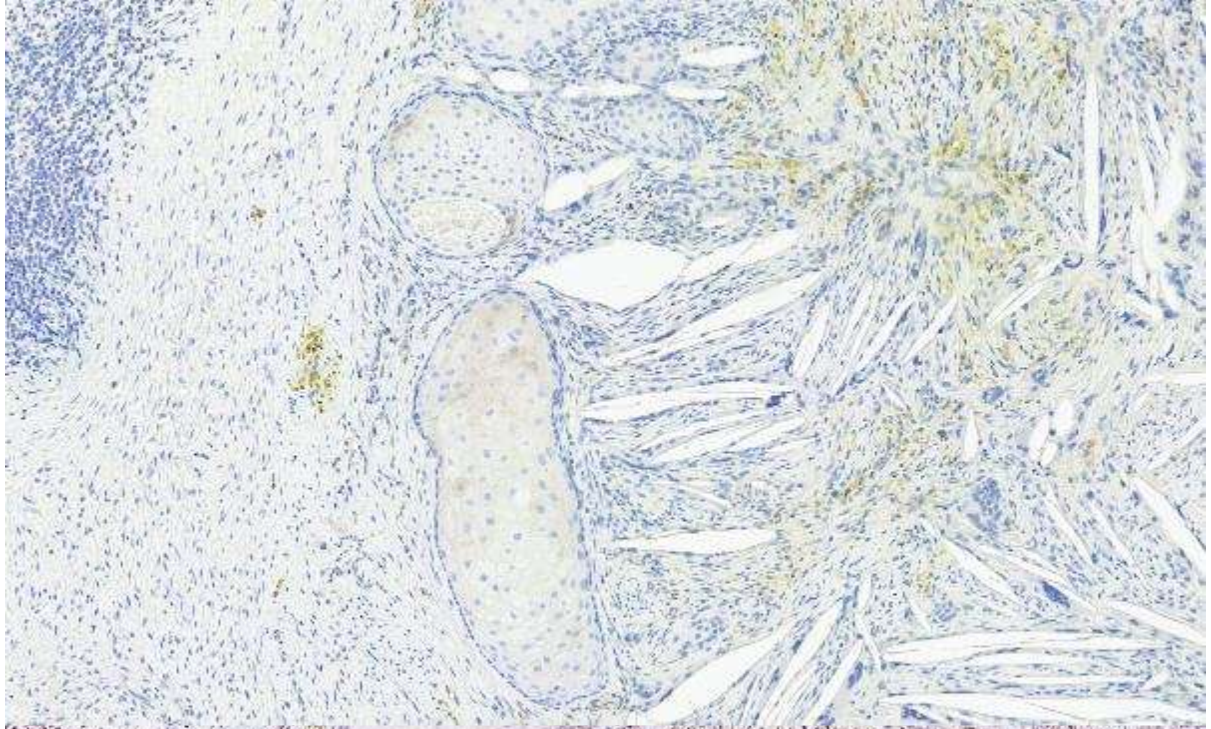

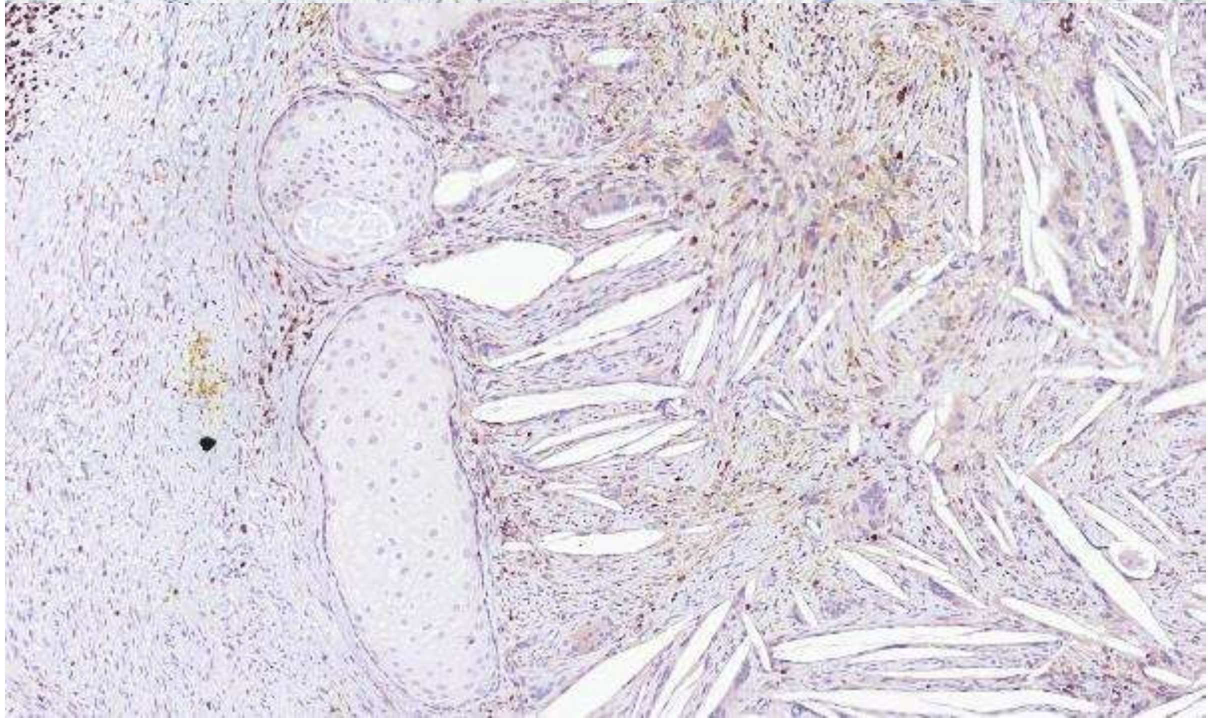

K15 - bcl2 x7

Partial basal staining, patchy staining in epithelial nest on the periphery of the cyst

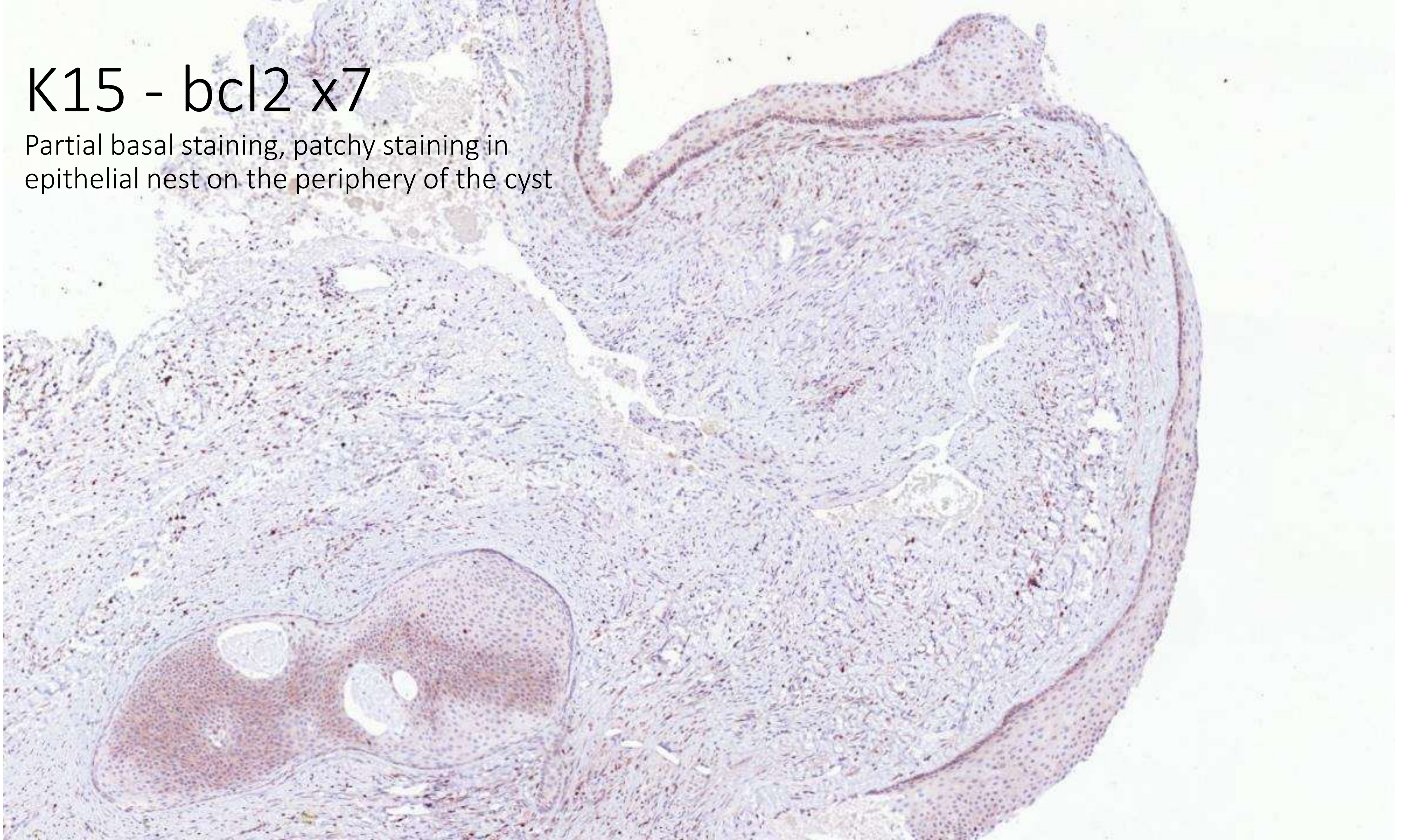

K15 – HE, CK17, bcl2 (x2)

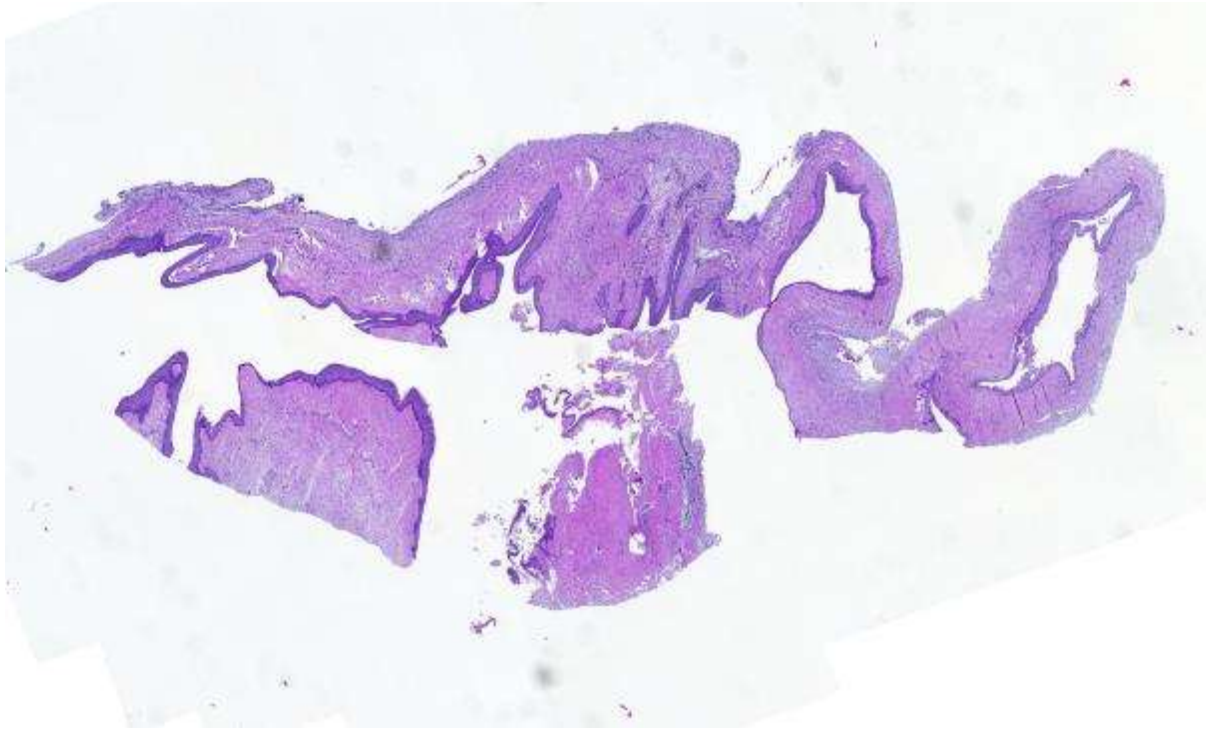

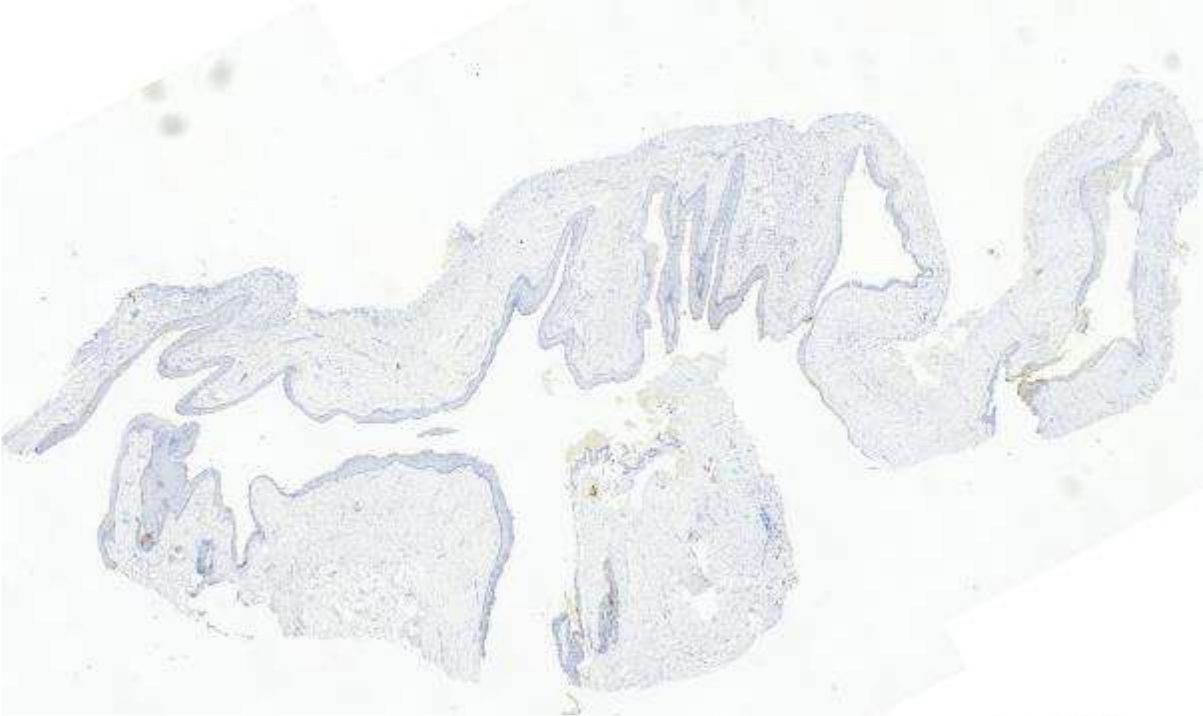

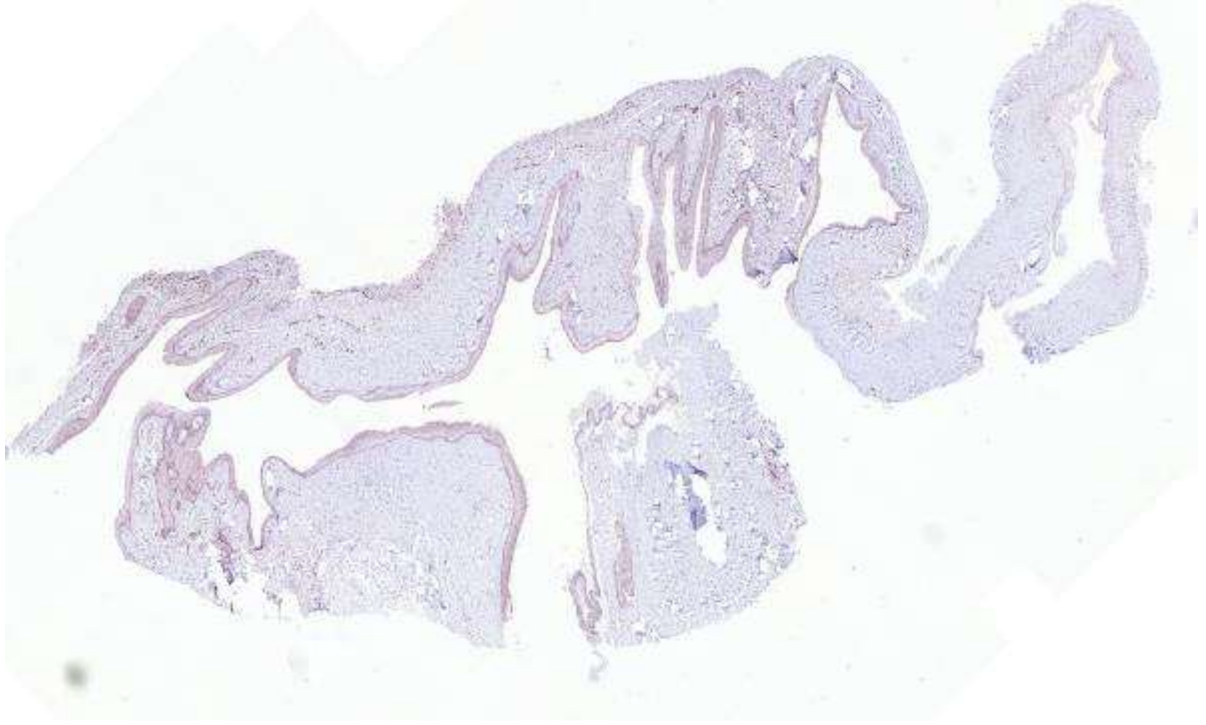

K15 – HE, CK17, bcl2 (x10)

Basal and superficial CK17 staining;
bcl2 negative (see next) but seemingly positive

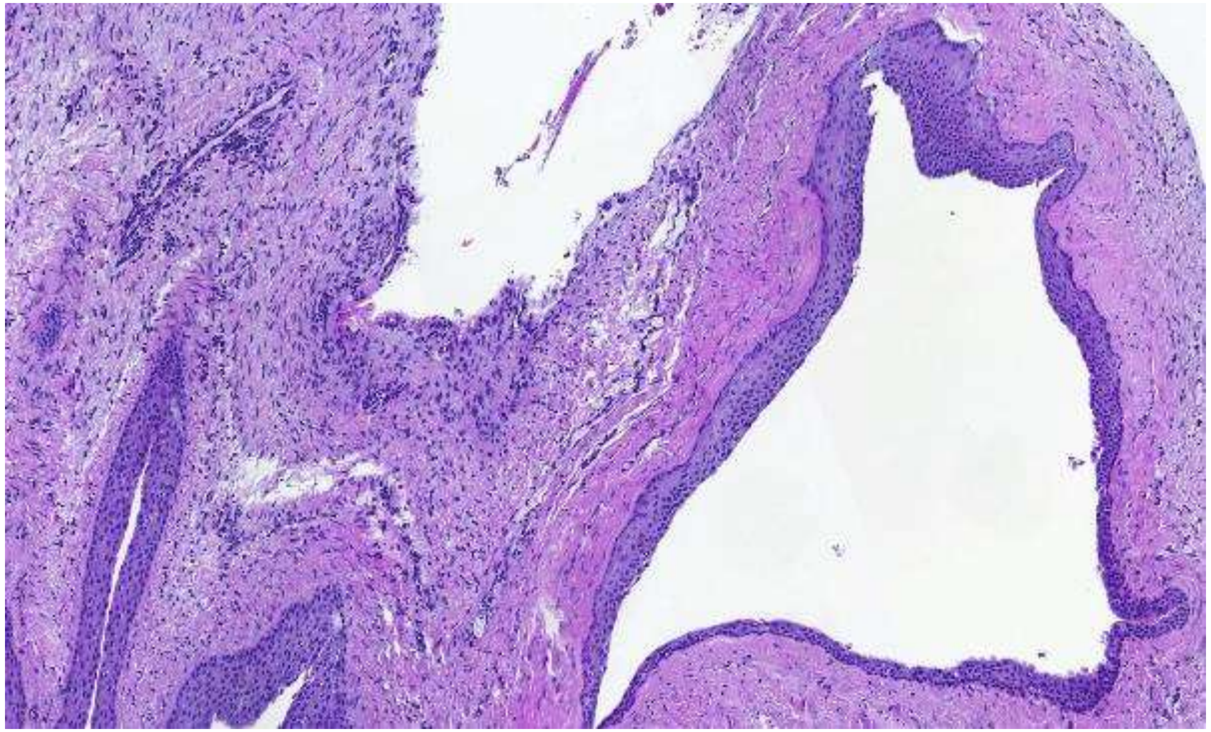

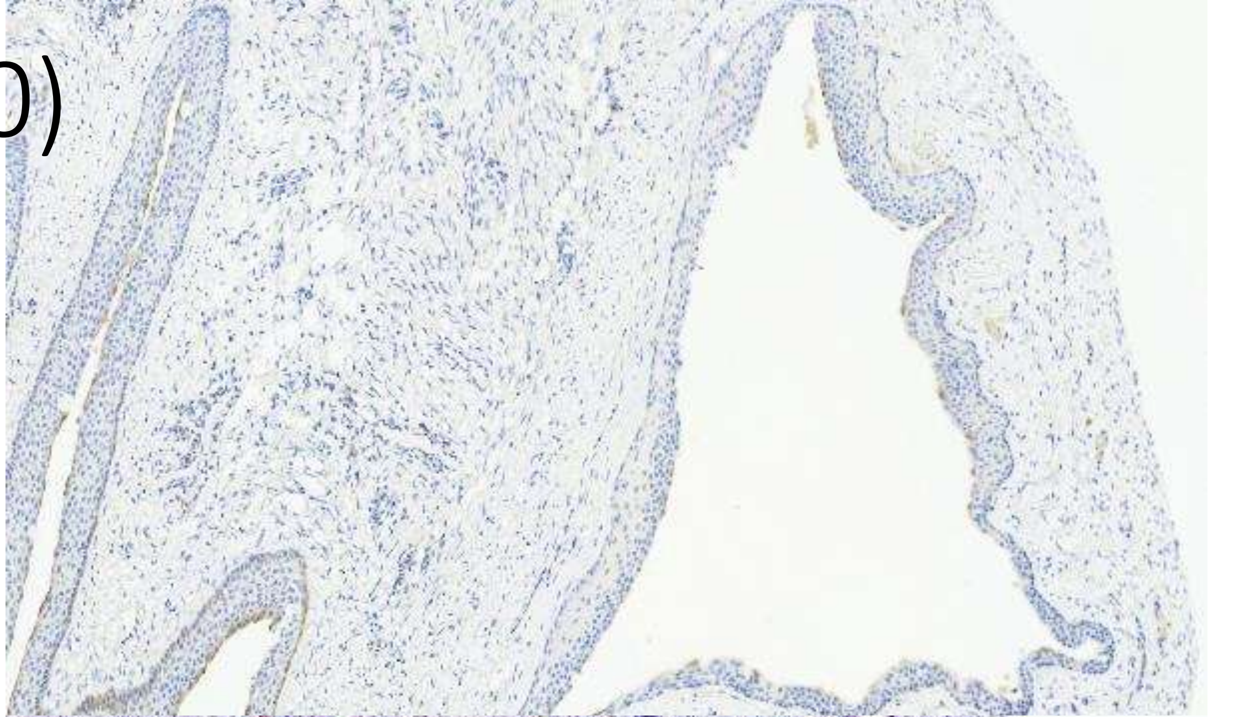

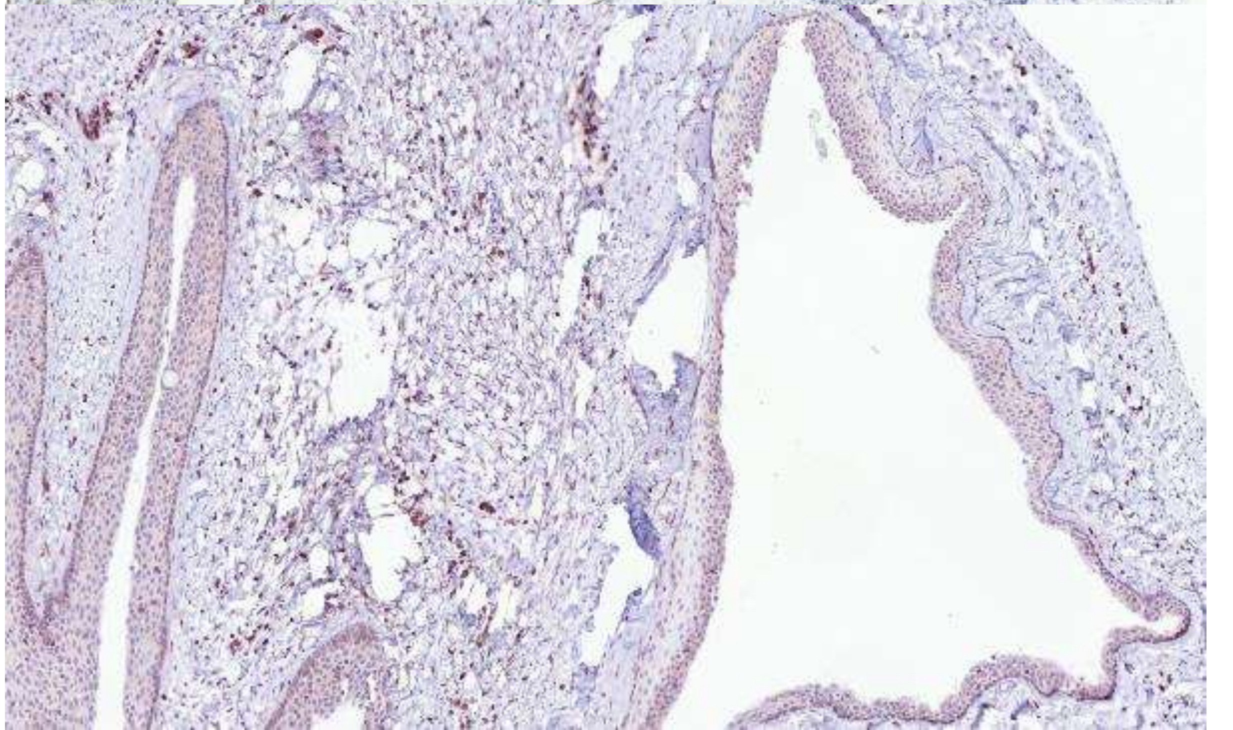

K15 - bcl2 negative versus weak superficial & basal or transepithelial (likely background) staining

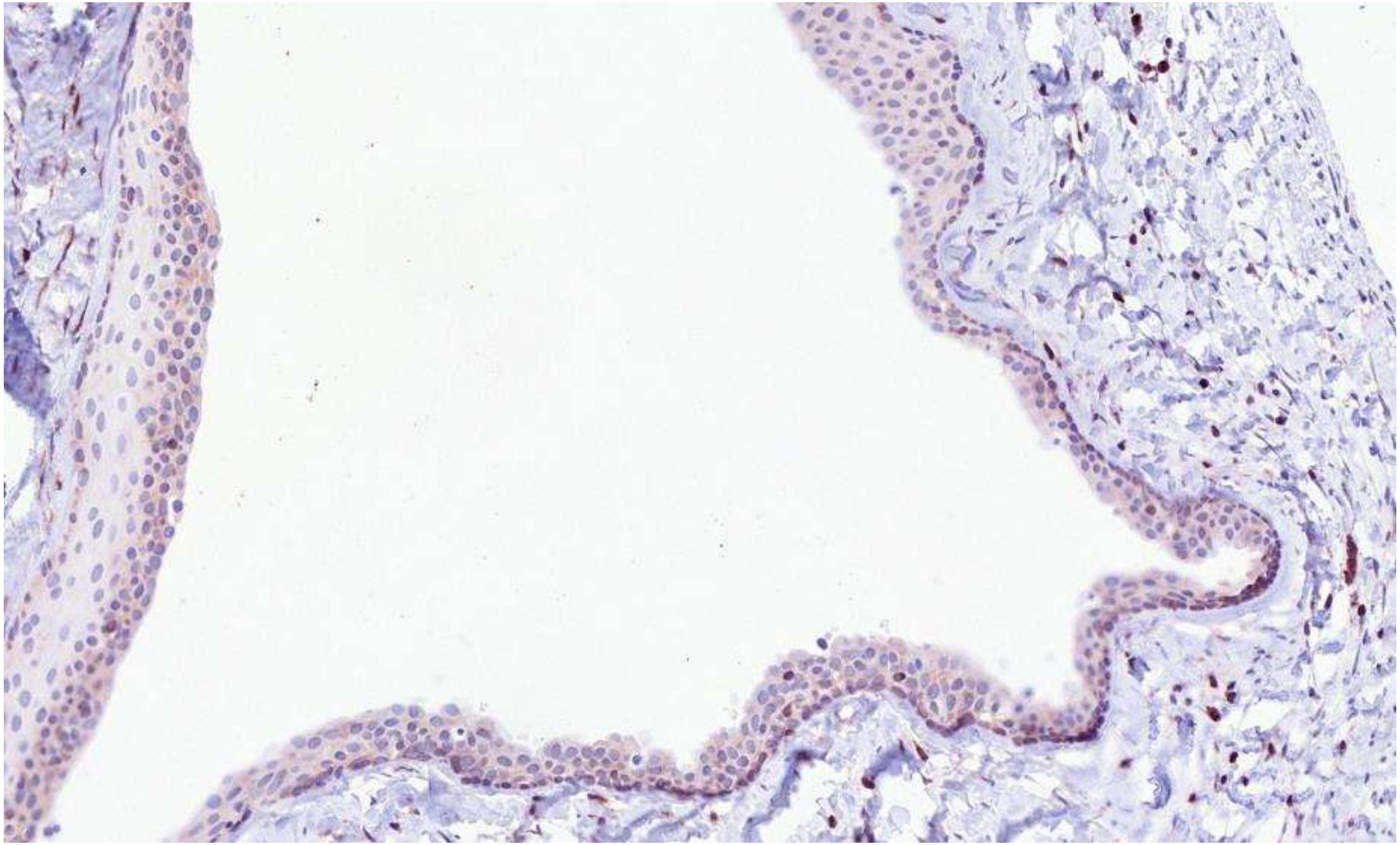

K15 - bcl2 negative versus weak superficial & basal or transepithelial (likely background) staining

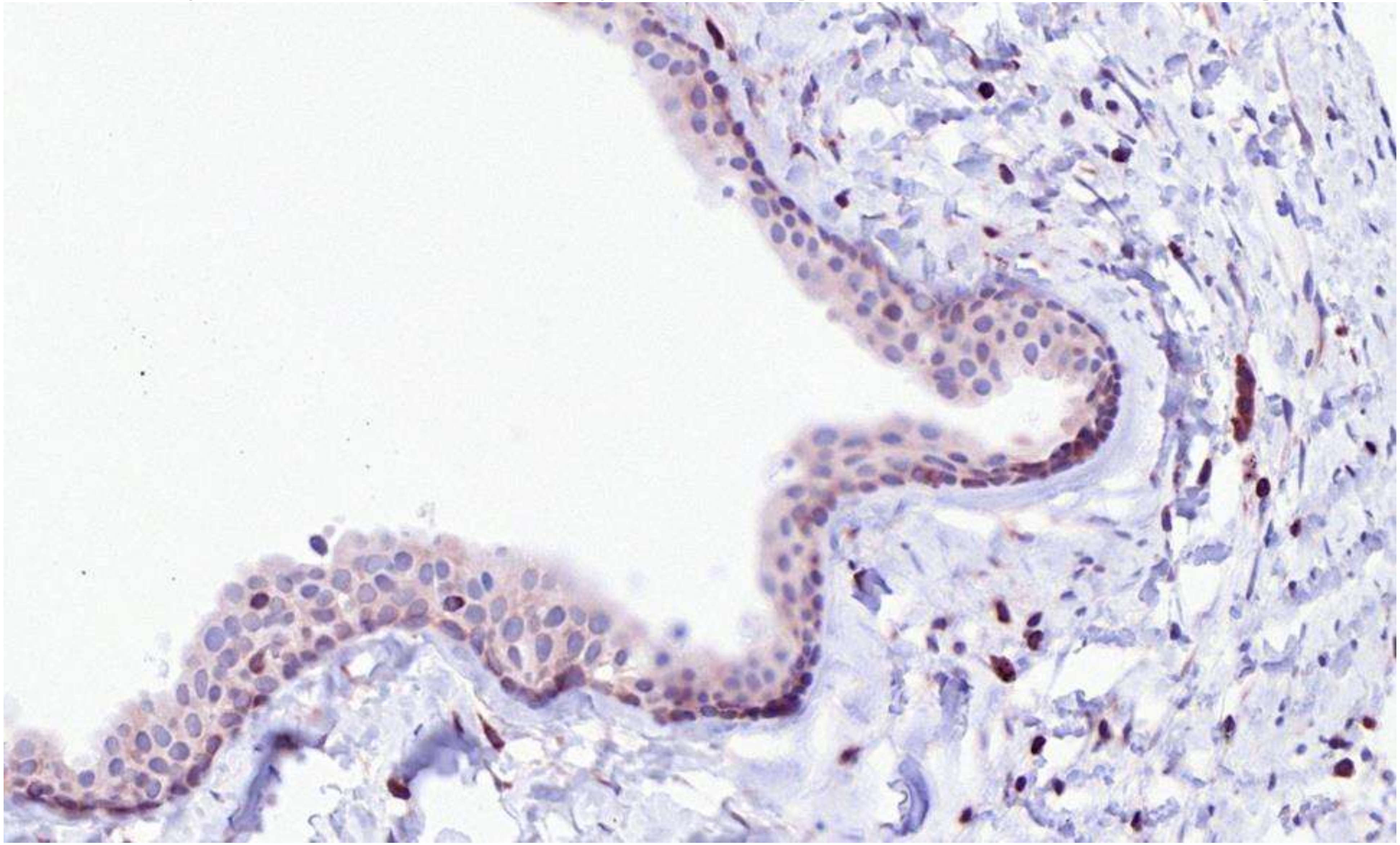

K15 – Conclusion

- H&E: relatively thin squamous epithelium, no obvious parakeratosis, no wavy surface, focal basal palisading, even epithelial-subepithelial contours; epithelial islands around the cyst
- Focal and minimal inflammation of the cyst wall associated with cholesterol granuloma
- Bcl2: basal/suprabasal, focal superficial, transepithelial or negative
- CK17: dominantly negative, with focal basal or transepithelial staining
- Histopathology: „inflammatory odontogenic cyst N.O.S.”
- Combined clinical and pathological opinion: dentigerous (follicular) cyst

Case – K16 (same patient as K30)

- 20-year-old female
- History: root filling of tooth 22 performed
- Dental hygiene/status: conserved, treated, crowded teeth
- Radiology: Sharp radiolucency between the root of teeth 22 and 23
- Operation: complete removal of the cyst
- Clinical diagnosis: globulomaxillary cyst

K16 – OPT

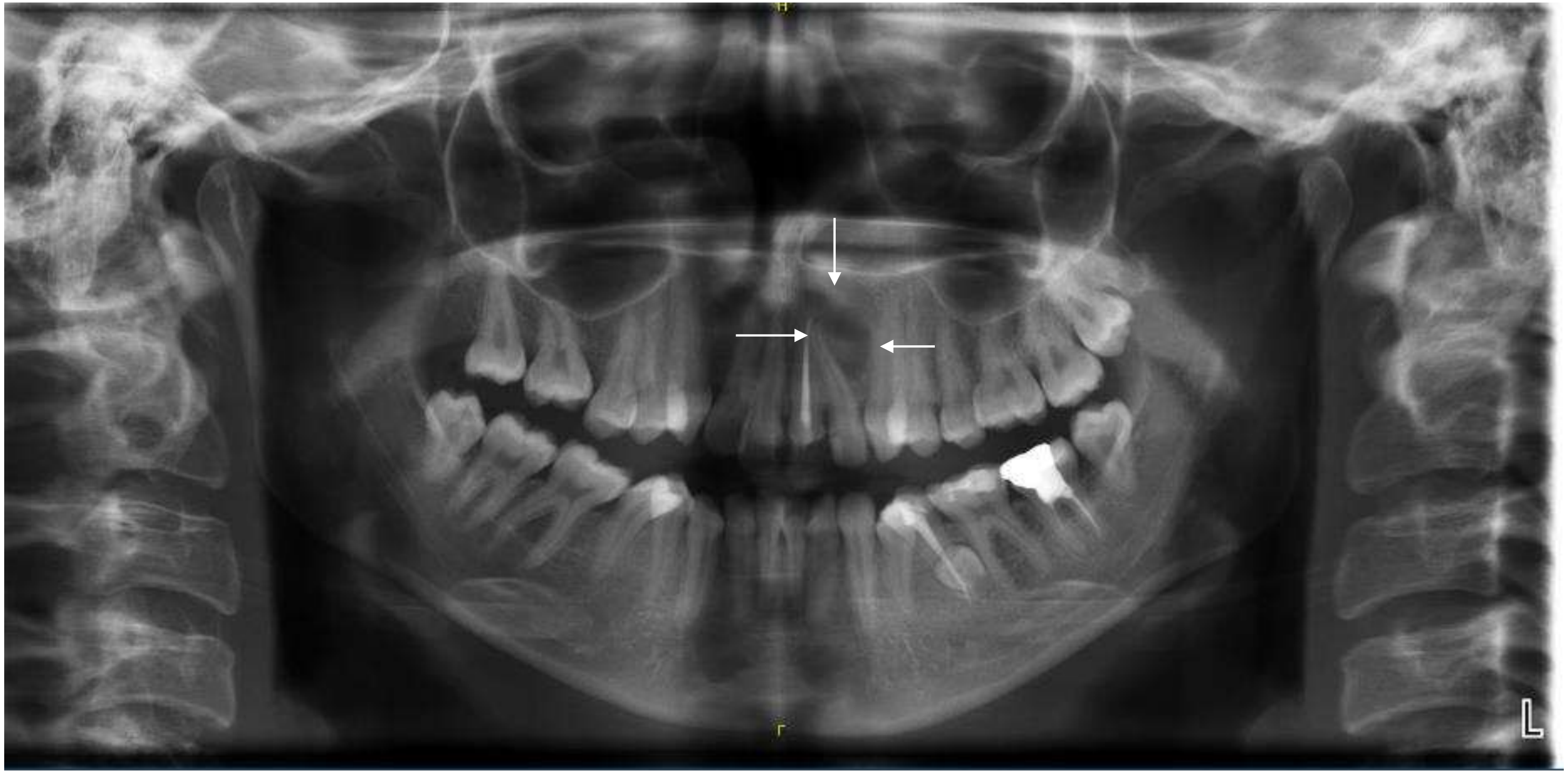

K16 – radiograph
(root filling of 22 between OPT and this image)

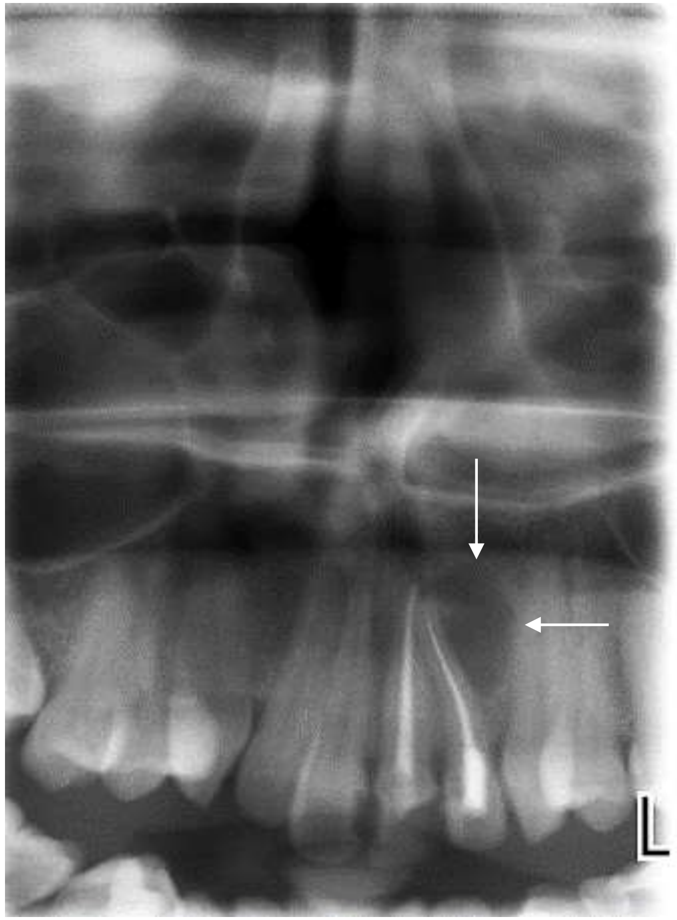

K16 – CT (coronal plane; anterior to posterior)

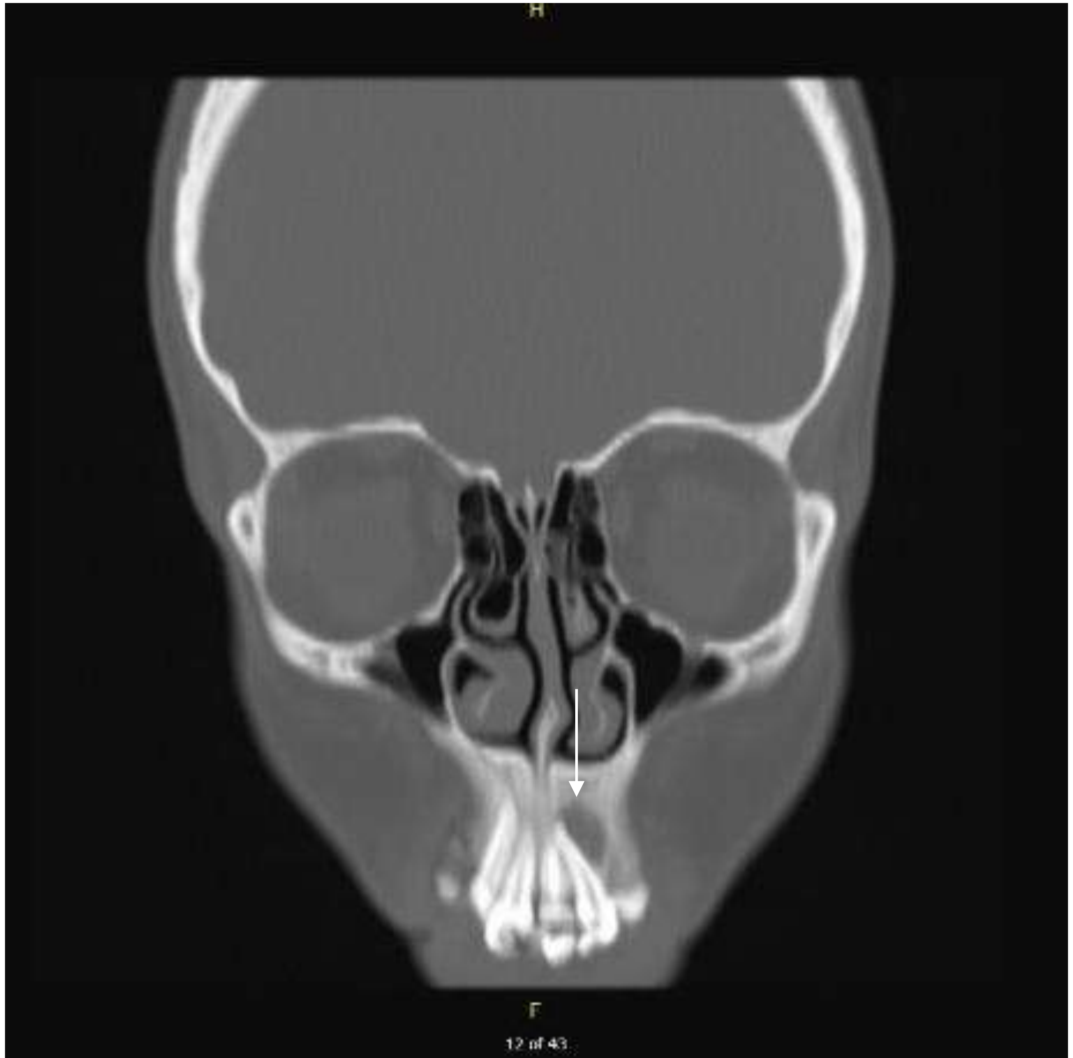

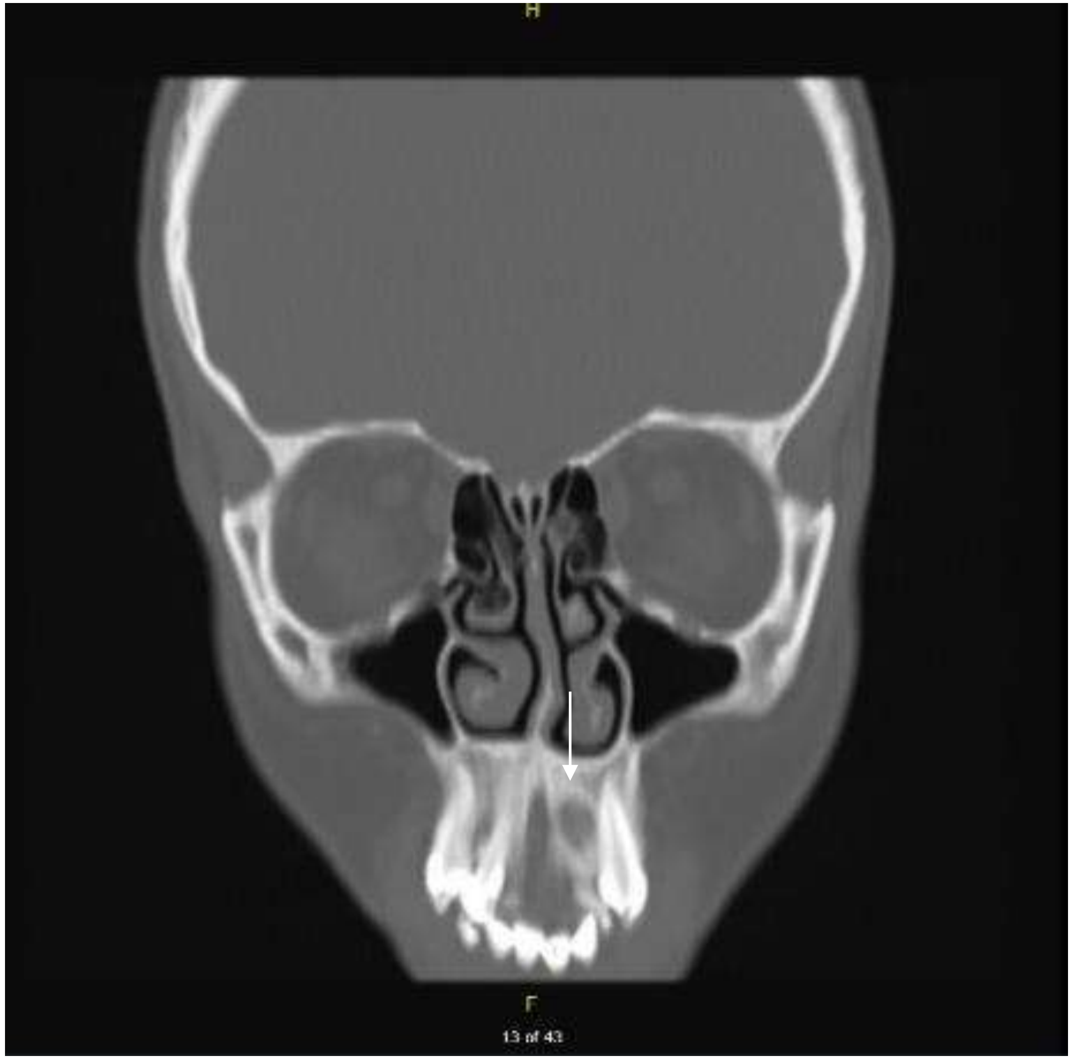

K16 – CT sagittal (right to left)

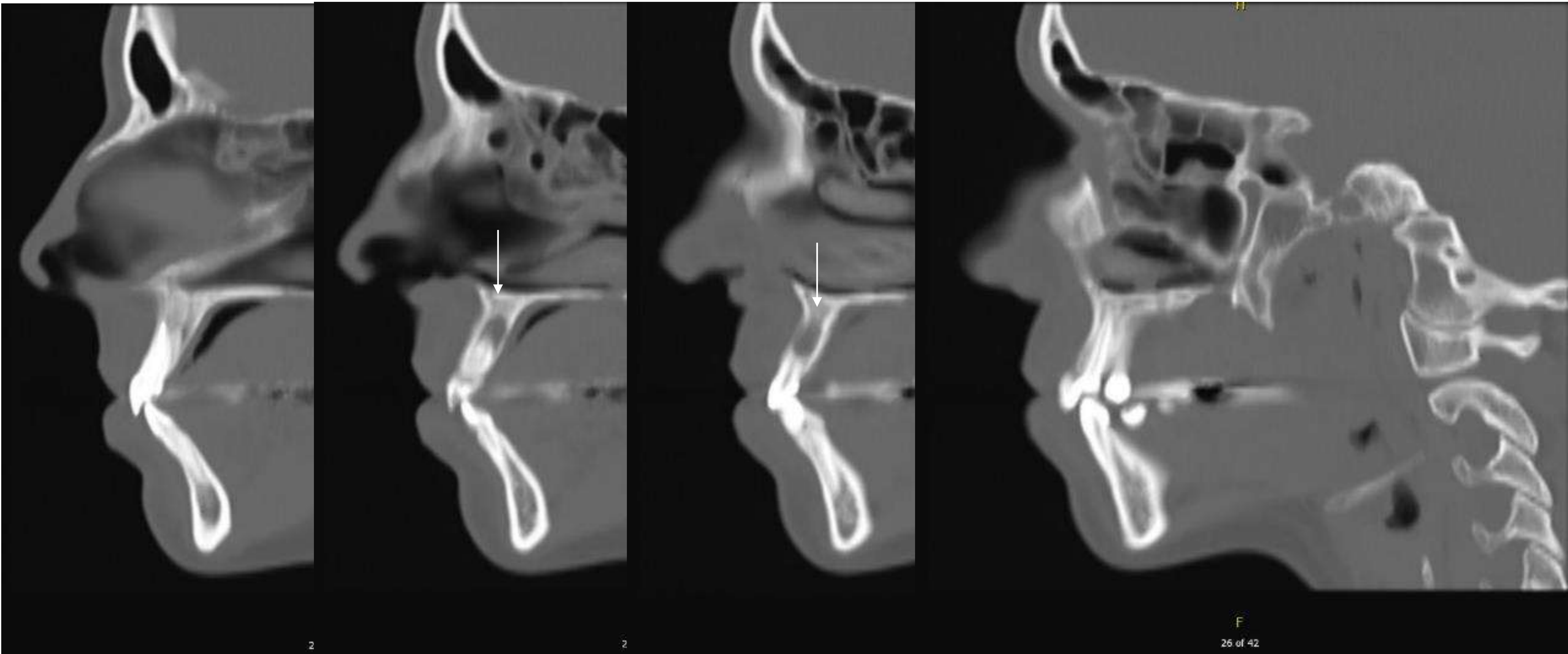

K16 – CT axial

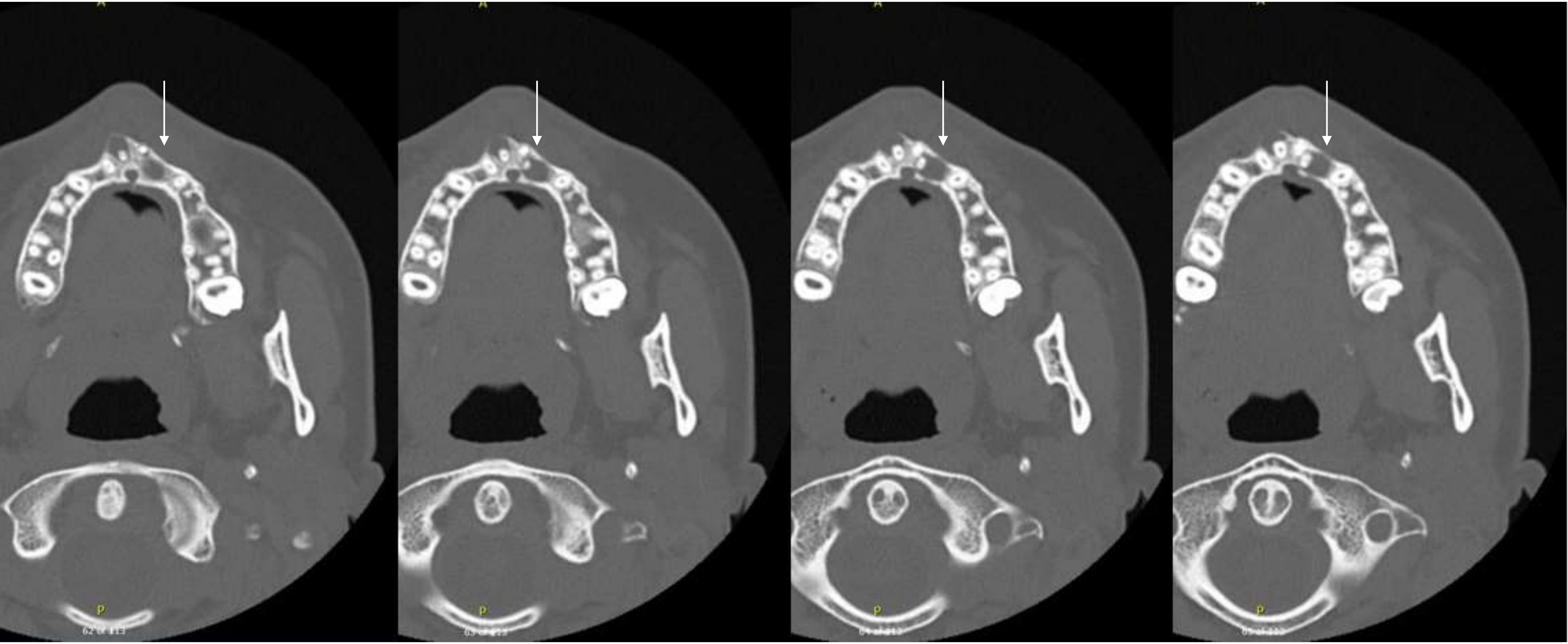

K16 – CT axial

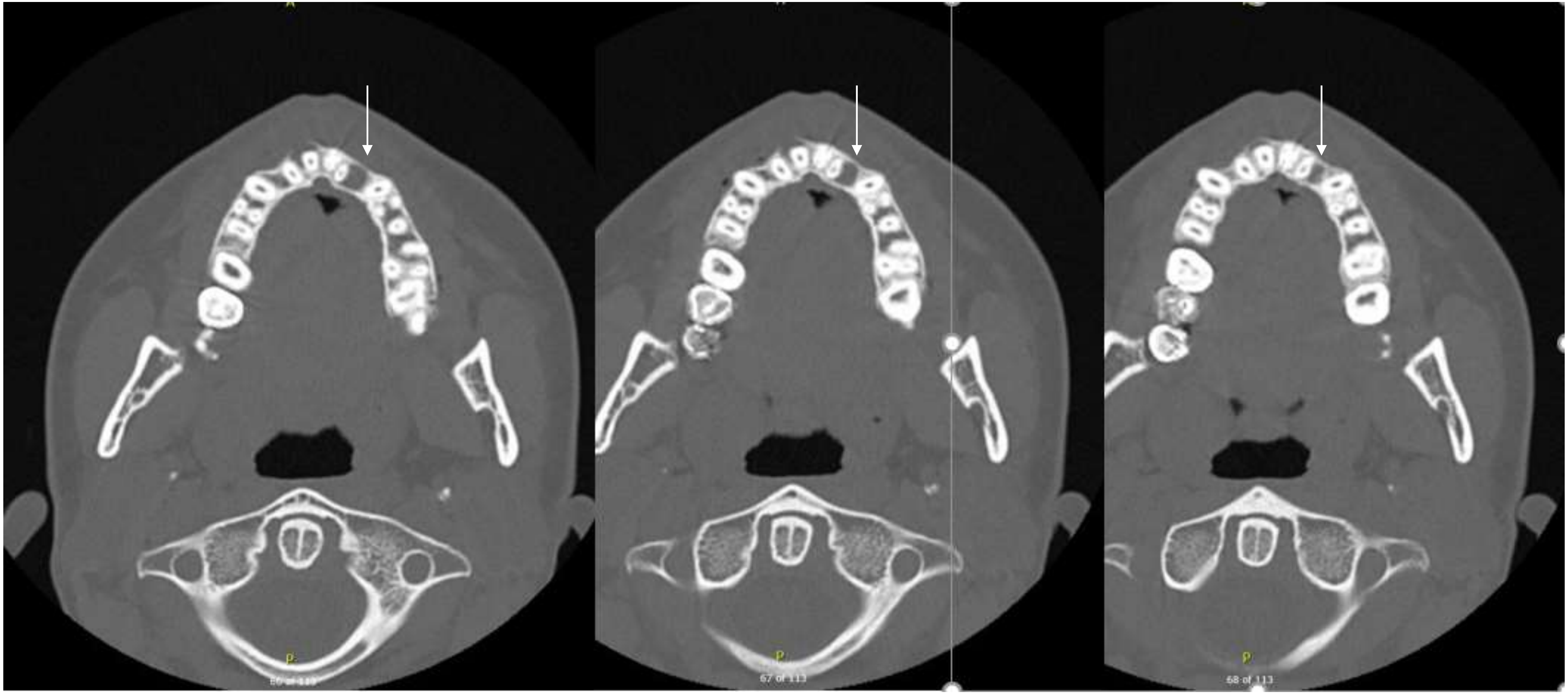

K16 – HE, CK17, bcl2 (x2)

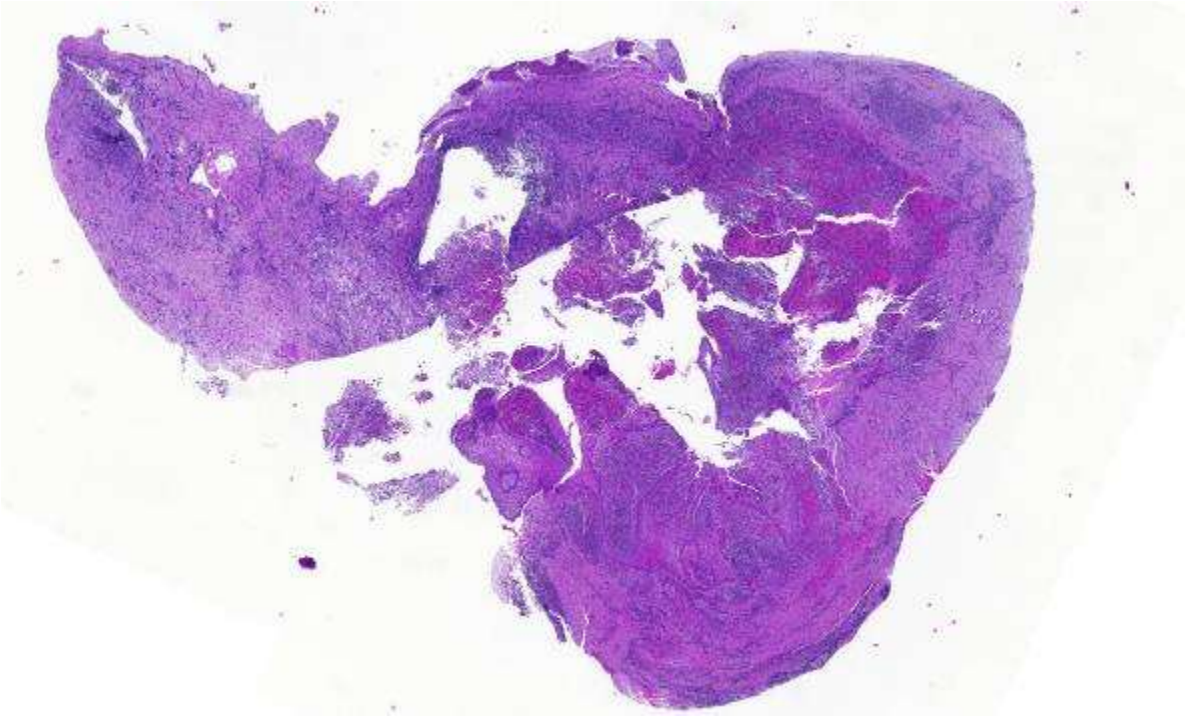

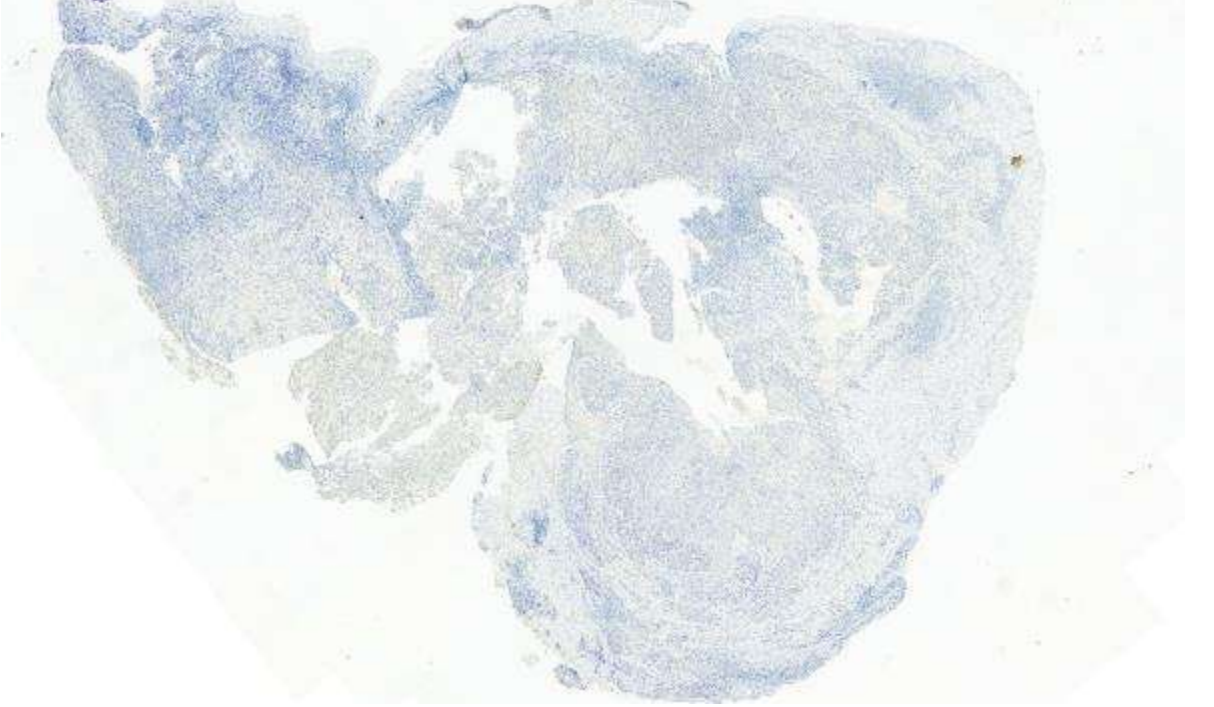

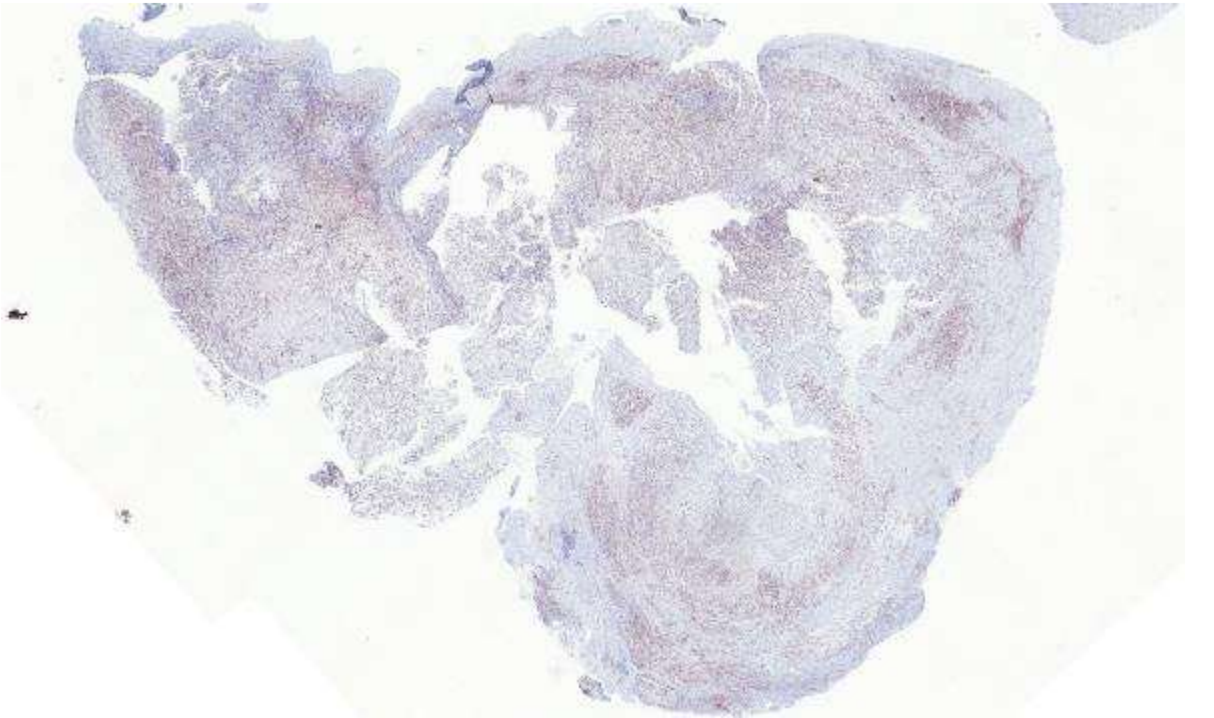

K16 – HE, CK17, bcl2 (x5)

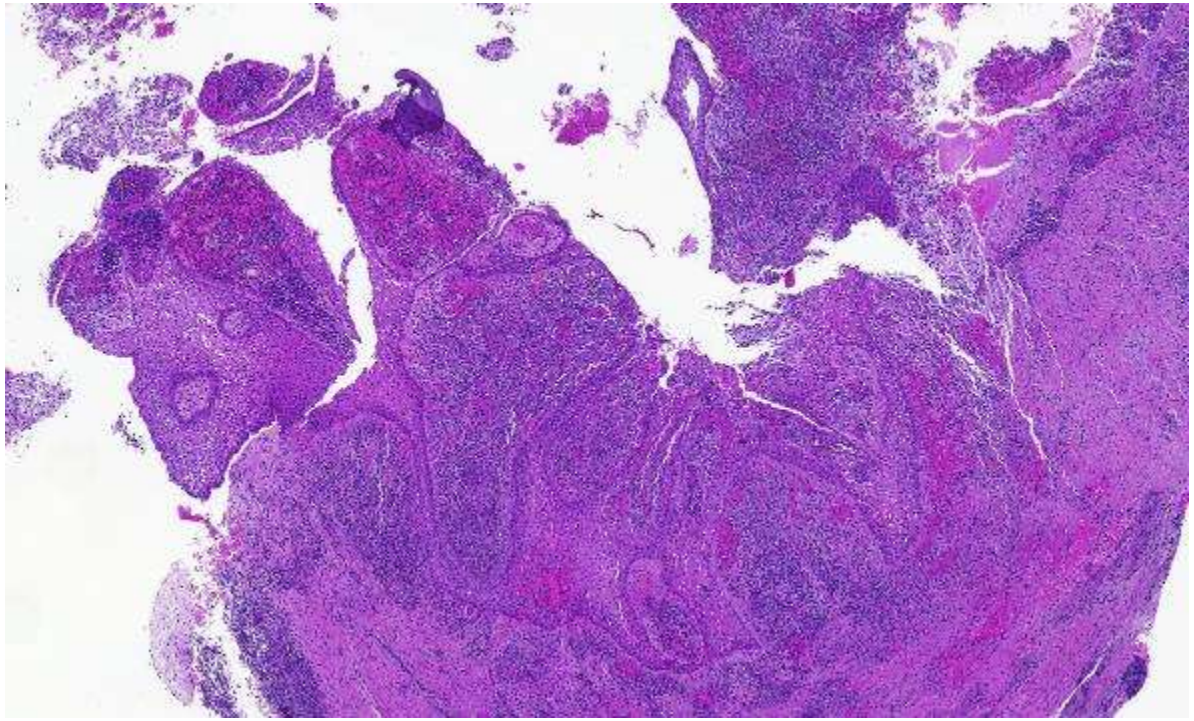

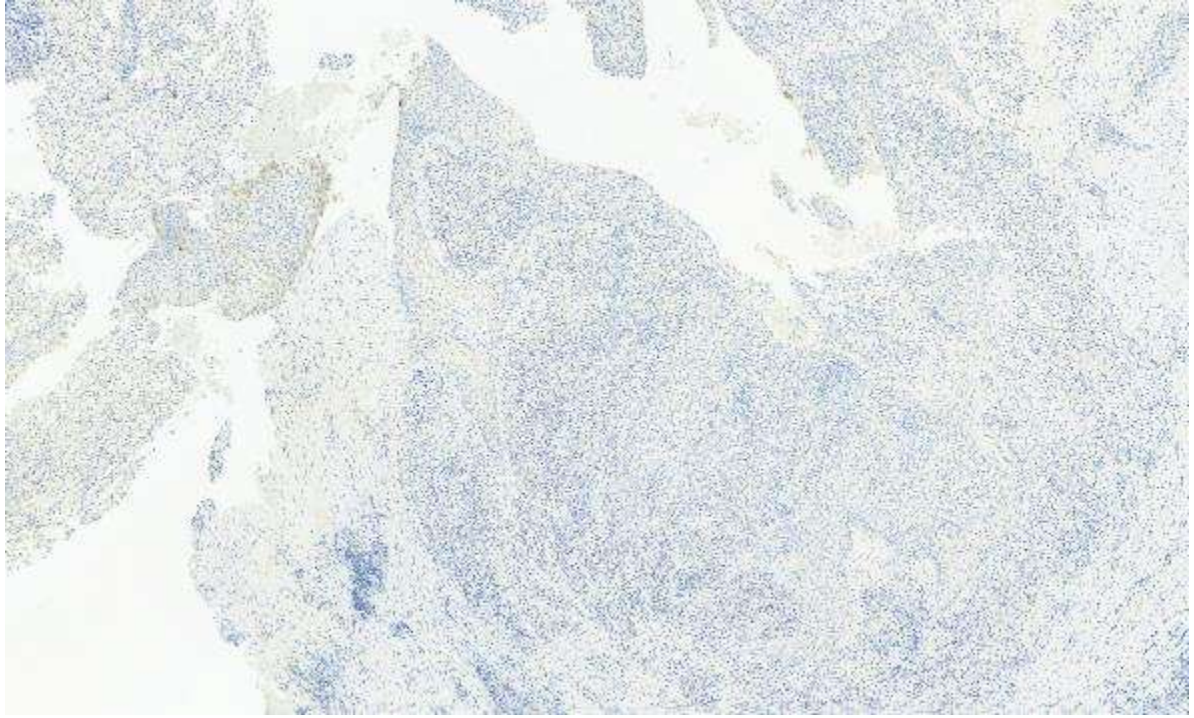

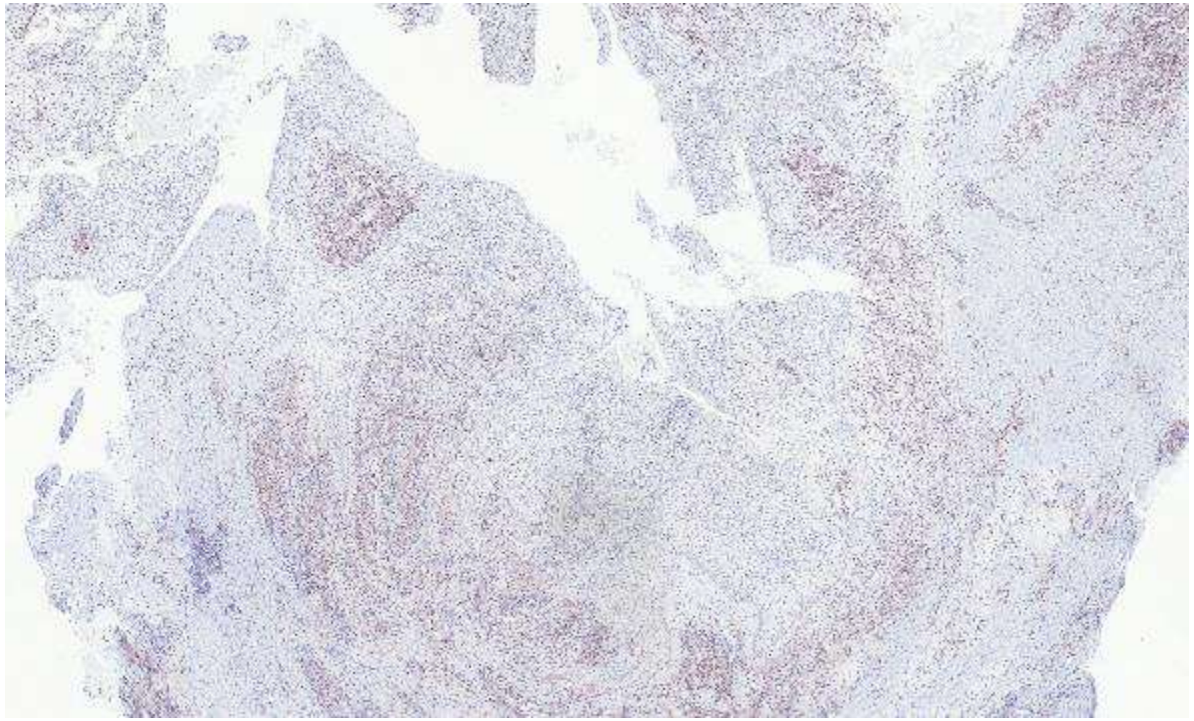

K16 – HE, CK17, bcl2 (x10)

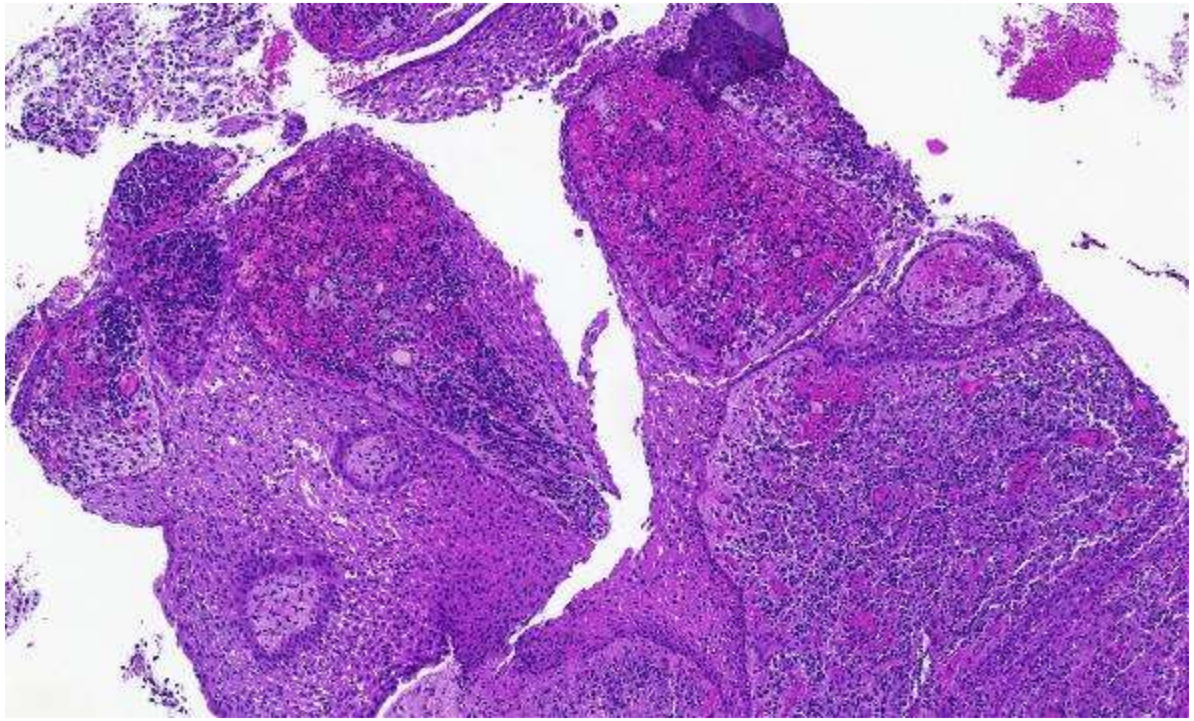

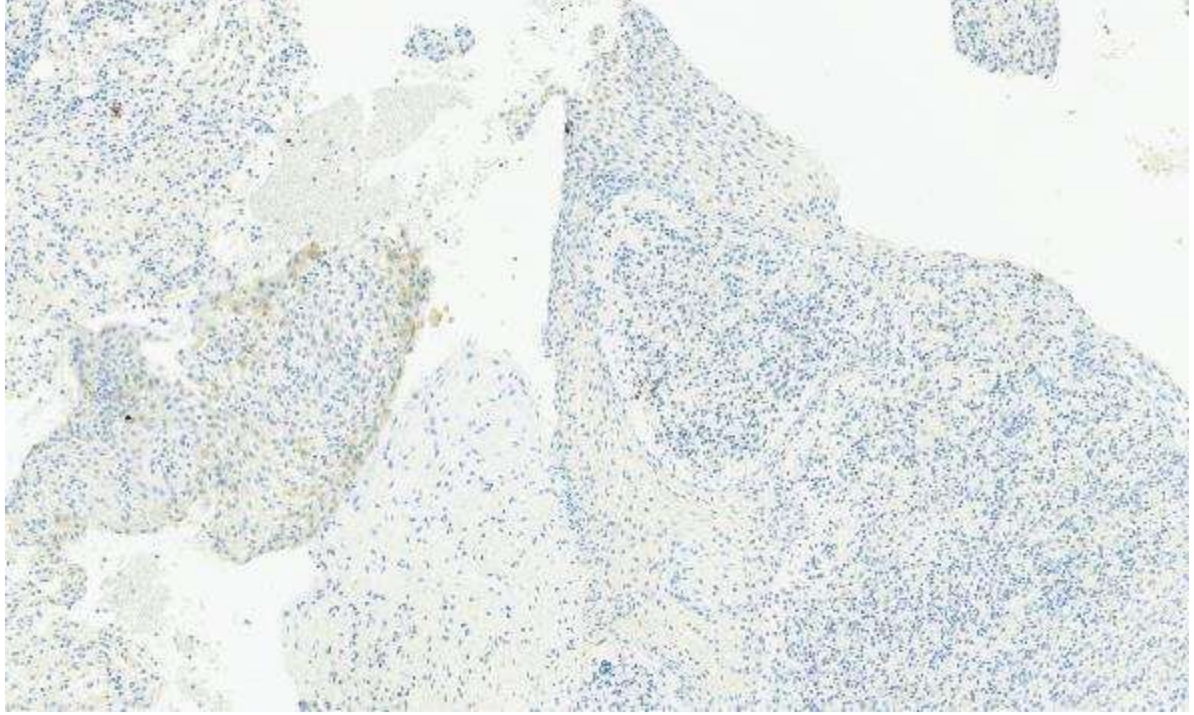

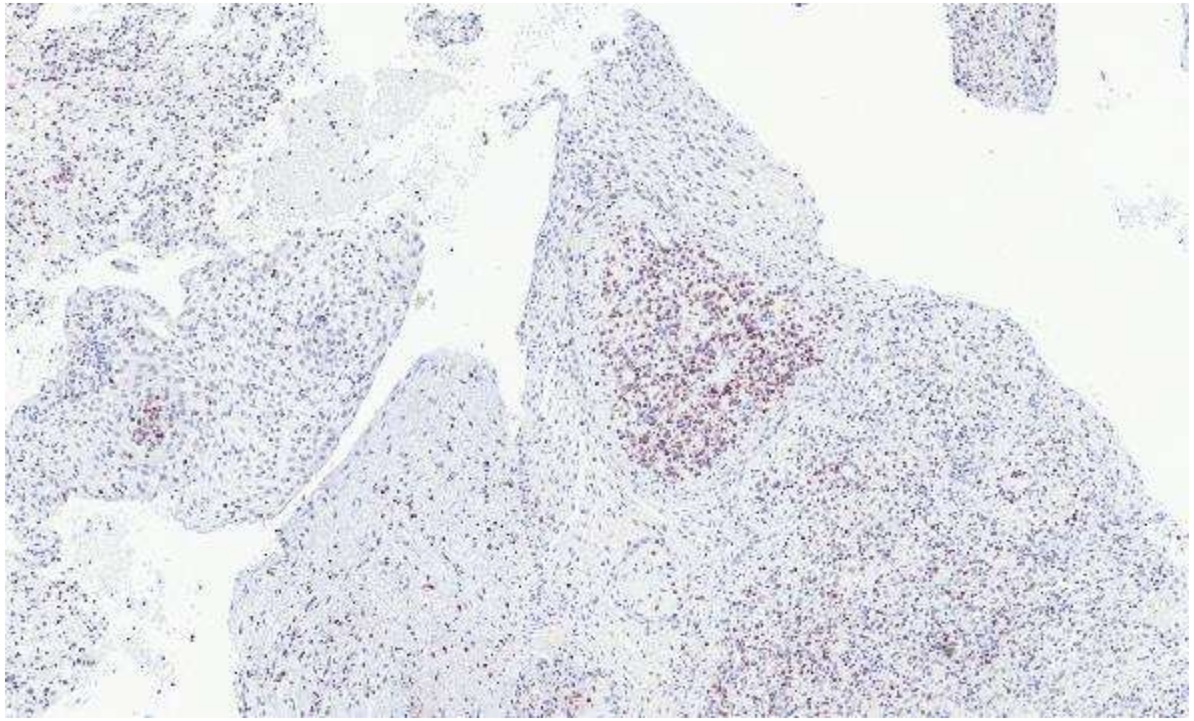

K16 – Conclusion

- H&E: relatively thick squamous epithelium, no parakeratosis, no wavy surface, no basal palisading, uneven epithelial-subepithelial contours
- Mixed inflammatory infiltrate of the cyst wall
- Bcl2: negative
- CK17: negative (focal are with superficial and patchy spinous staining)
- Histopathology: radicular cyst
- Combined clinical and pathological opinion: most likely to be an inflamed lateral periodontal cyst

Case K17

- 66-year-old male
- History: toothache (tooth 38)
- Dental hygiene/status: partly missing, untreated teeth including tooth 38 with caries; periodontitis
- Radiology: 2 cm maximum dimension radiolucent lesion at the apex of tooth 38
- Operation: removal of the partially impacted tooth 38, followed by removal of the cyst
- Clinical diagnosis: mandibular cyst

K17 – OPT

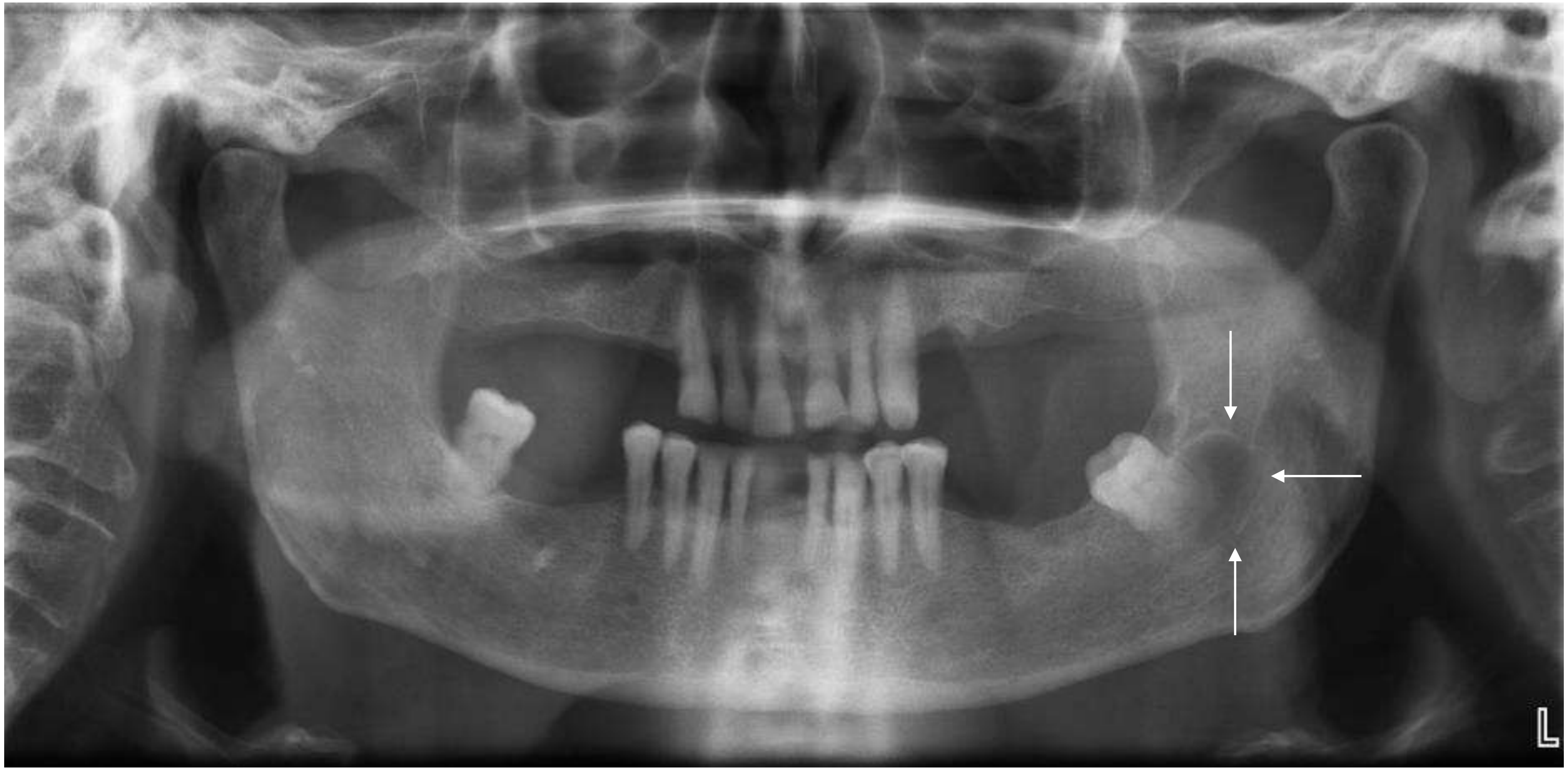

K17 – OPT zoomed

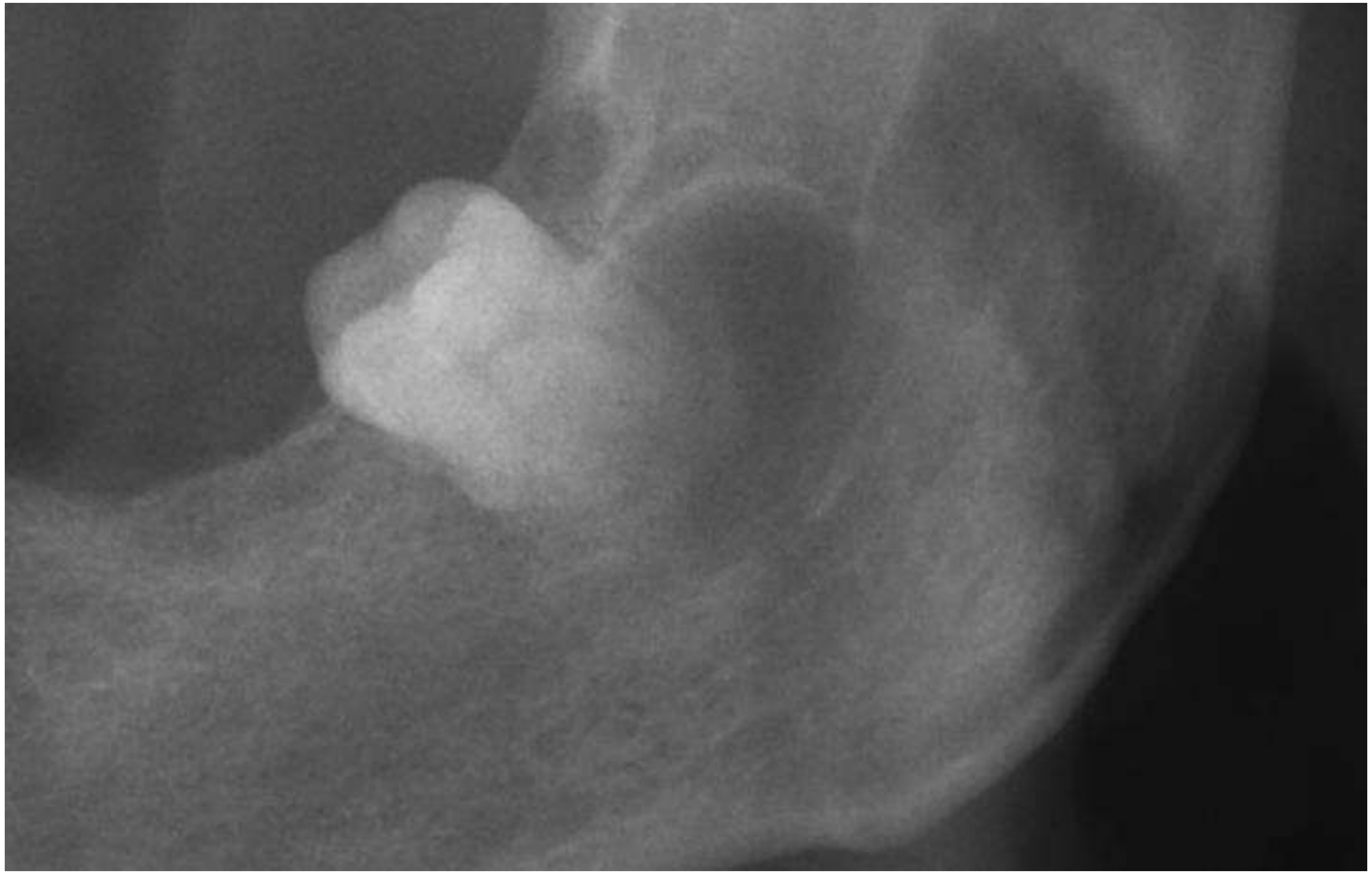

K17 – CT Coronal, posterior to anterior

35/138
40/138
45/138
49/138
51/138

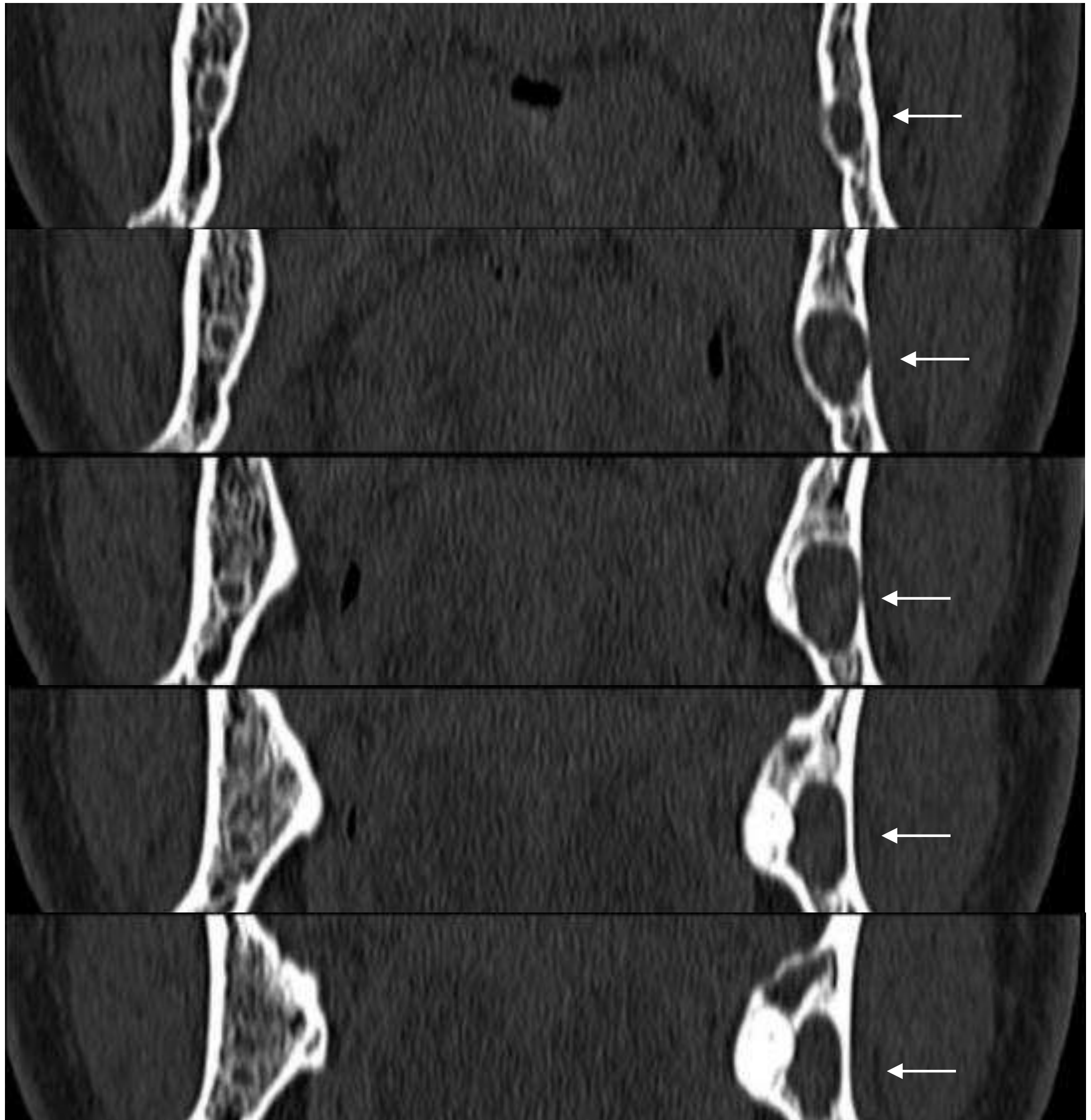

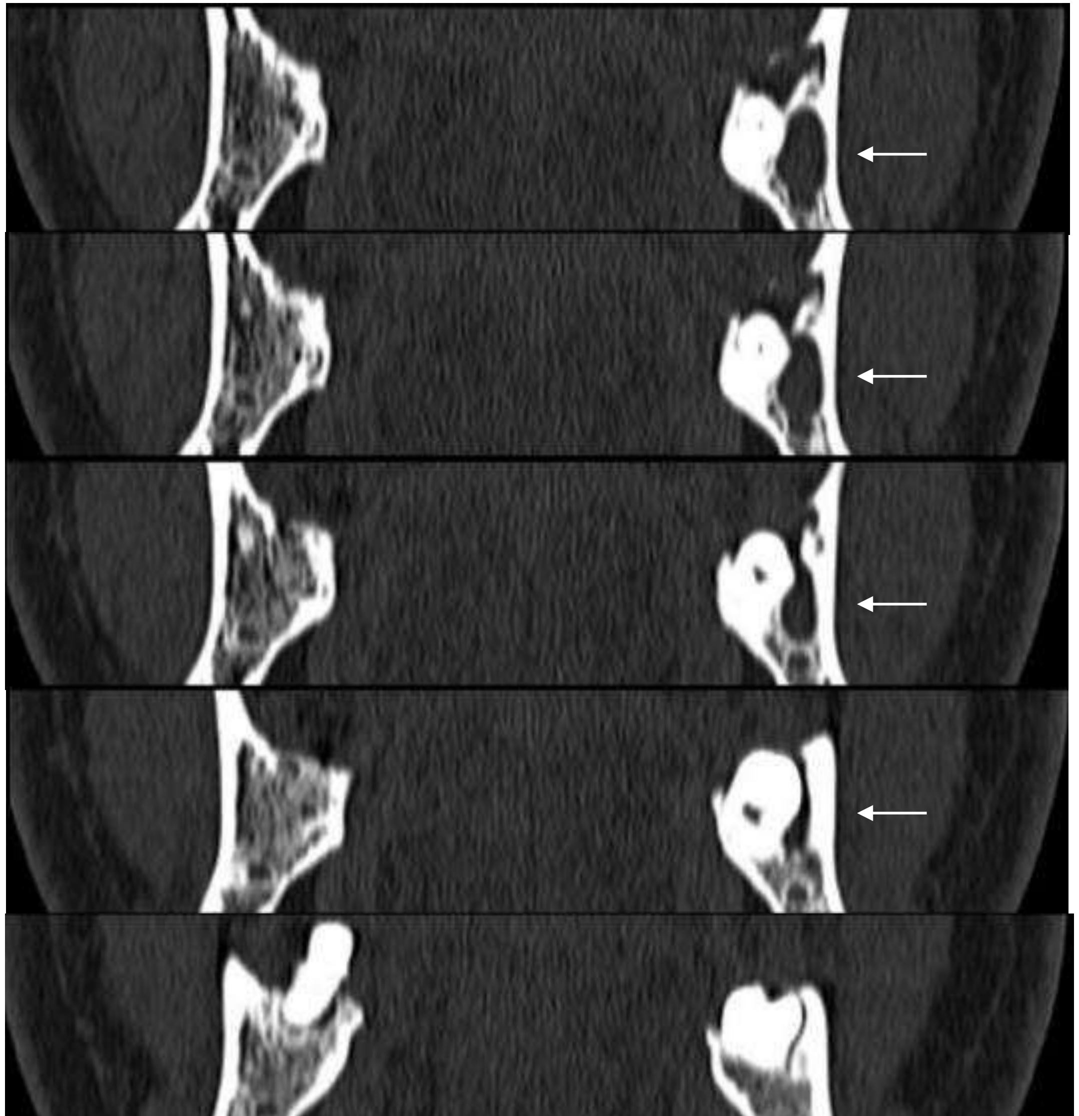

52/138
53/138
55/138
58/138
63/138

K17 – CT Sagittal, left to right 23-27/101 slices

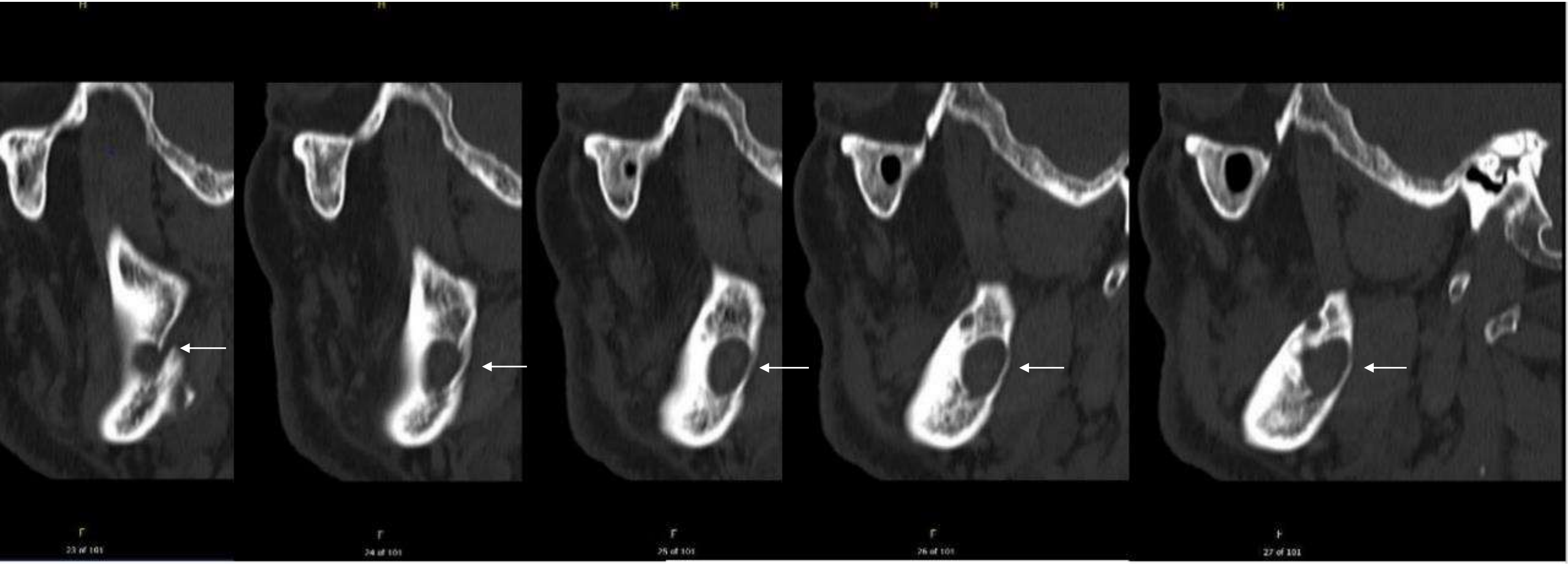

K17 – CT Sagittal, left to right 28-30/101 slices

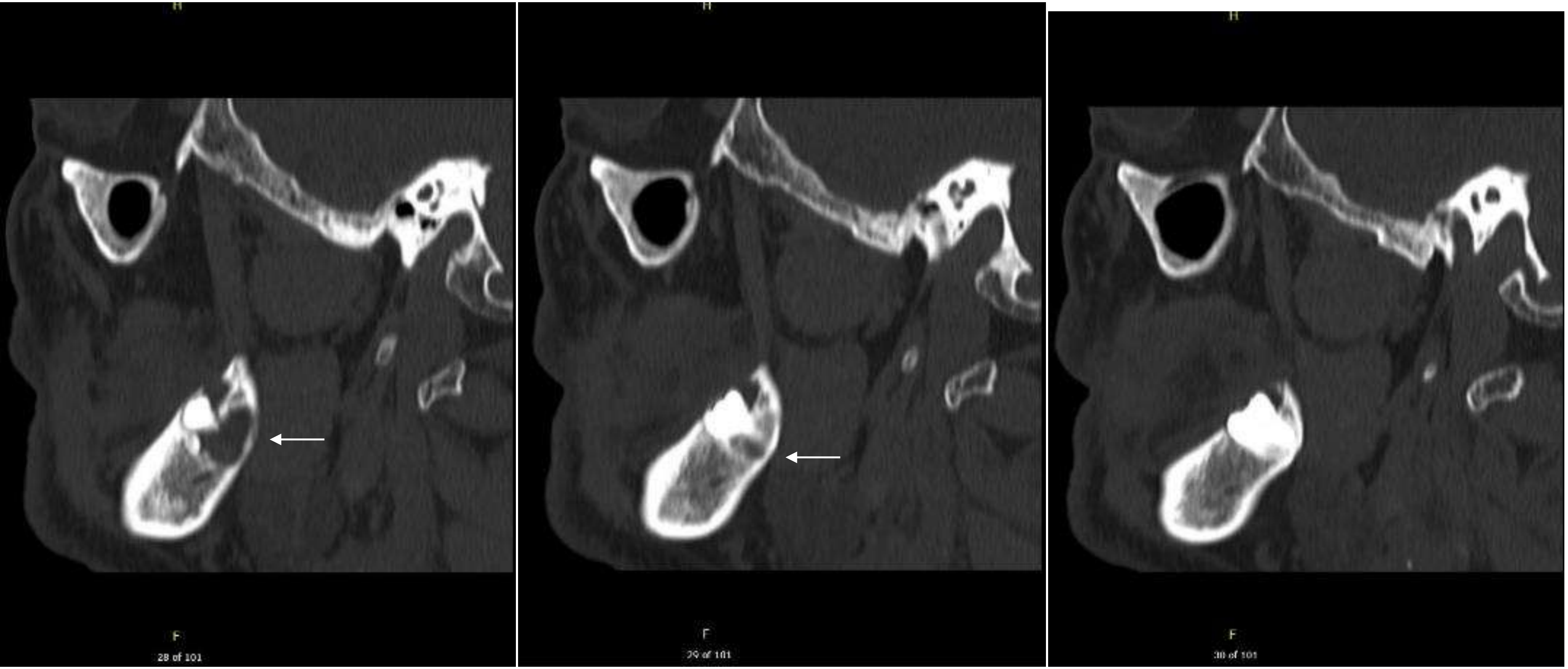

K17 – CT axial, caudal to cranial 37, 39, 41, 43/144 slices

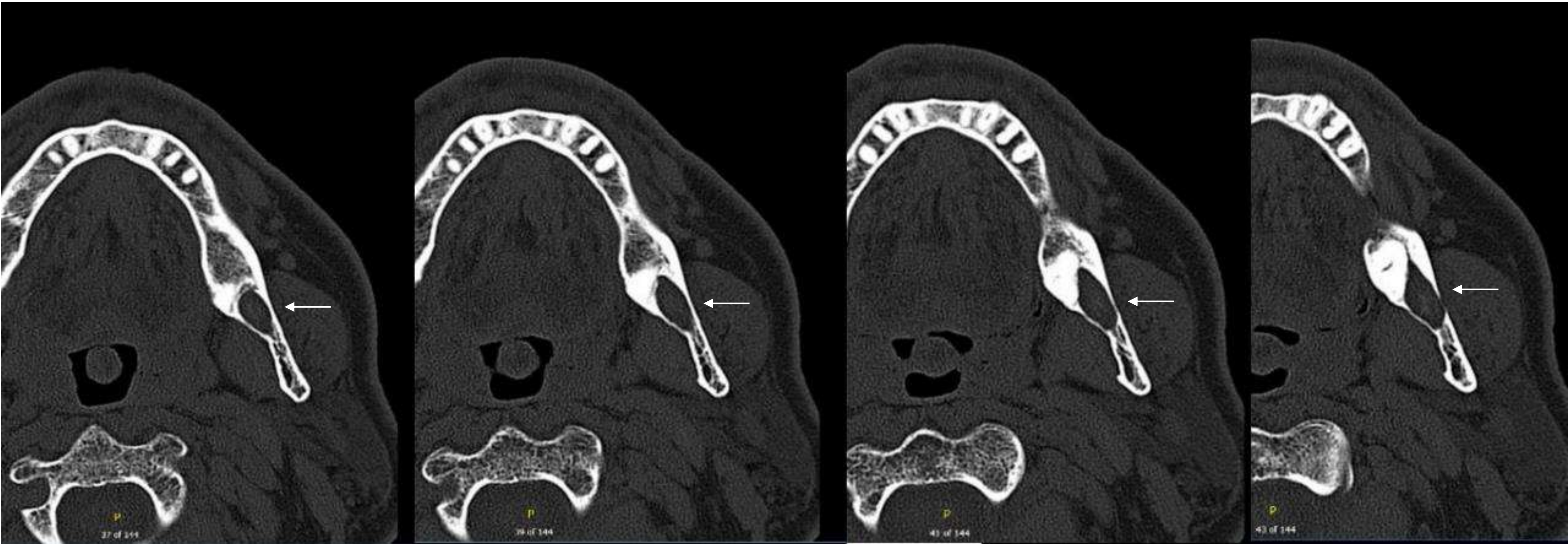

K17 – CT axial, caudal to cranial 47, 48, 49, 50/144 slices

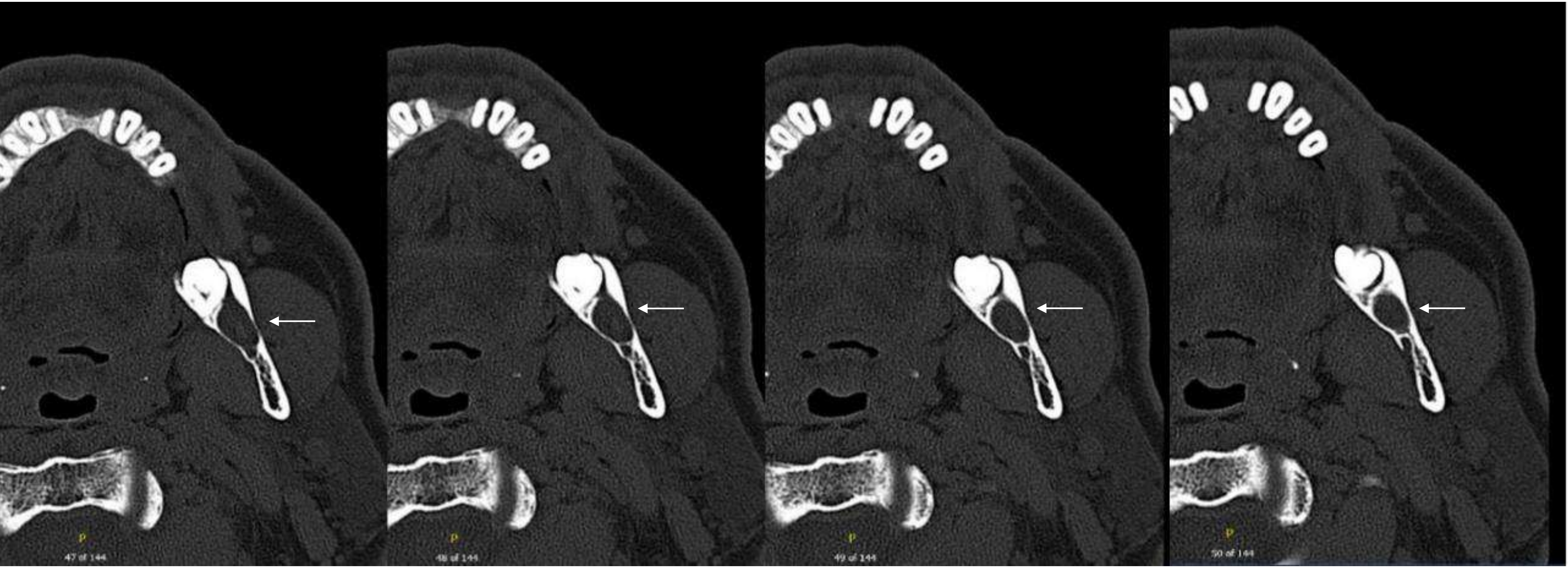

K17 – HE, CK17, bcl2 (x1.7)

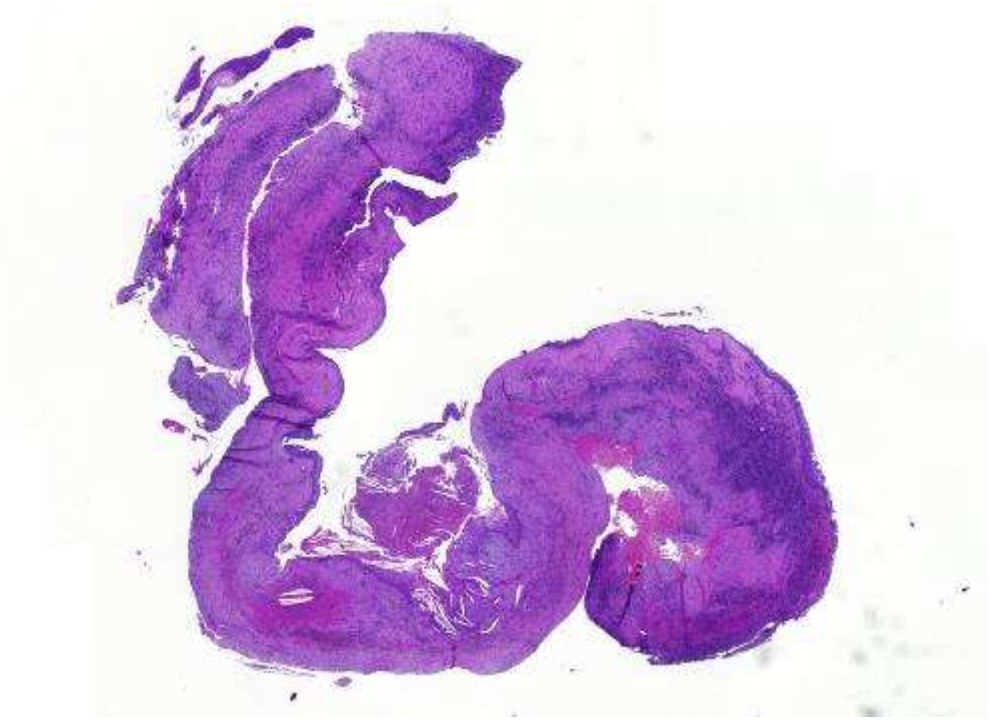

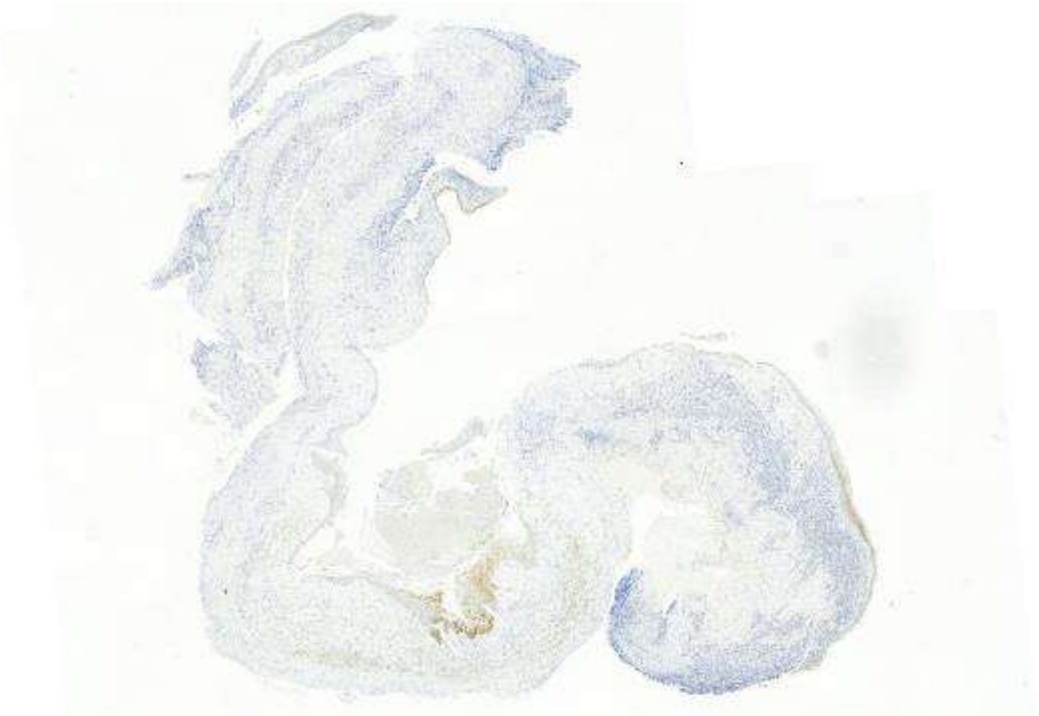

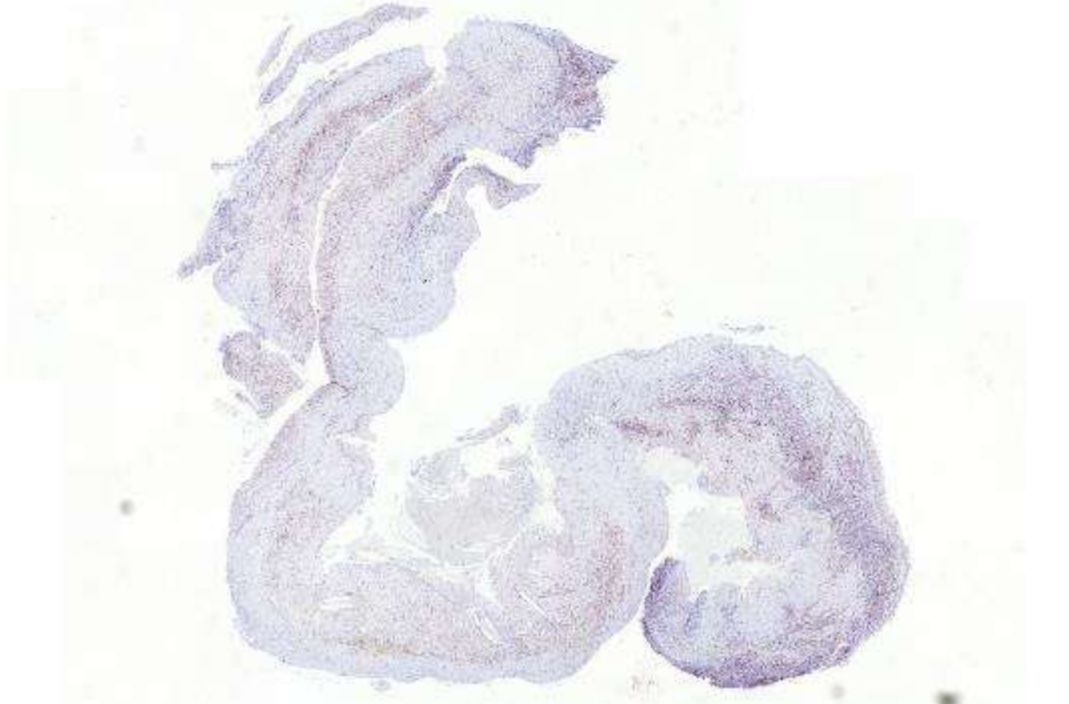

K17 – HE, CK17, bcl2 (x8)

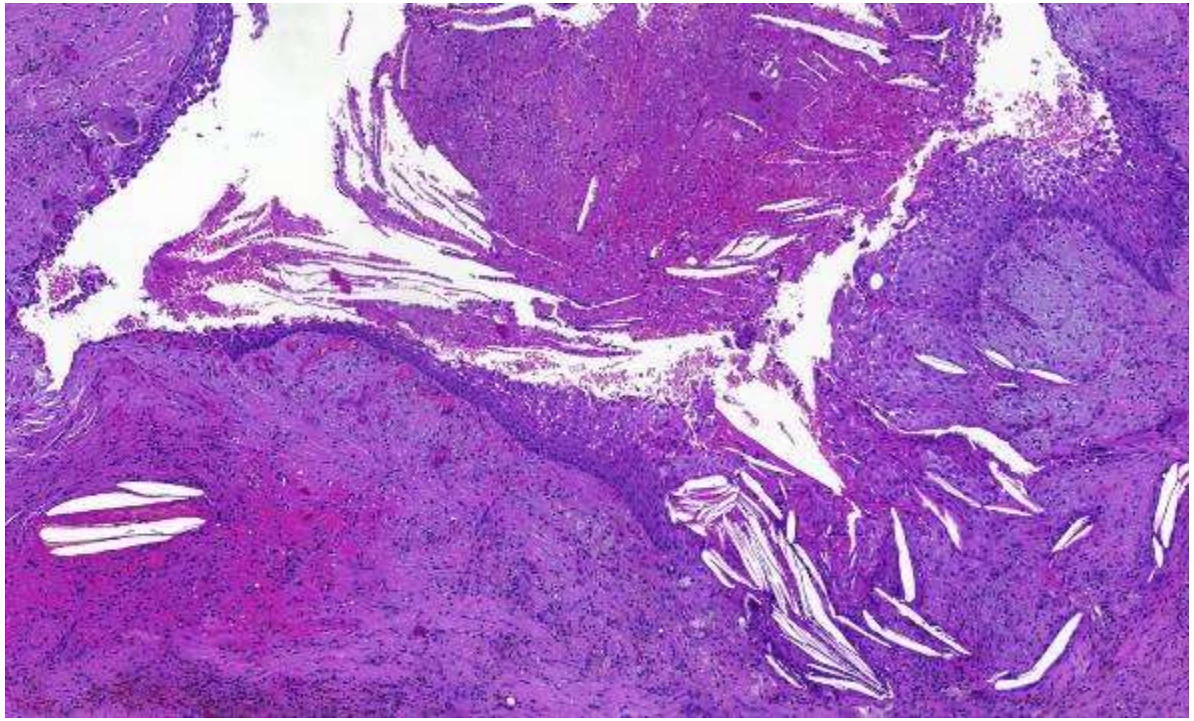

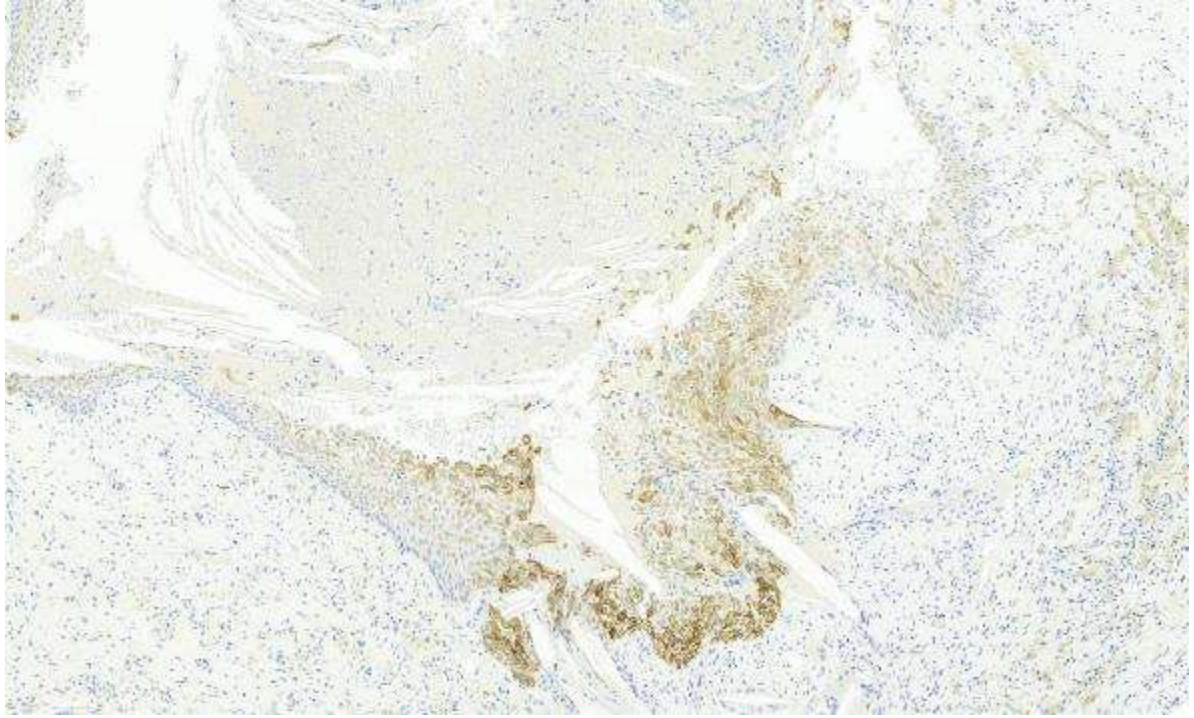

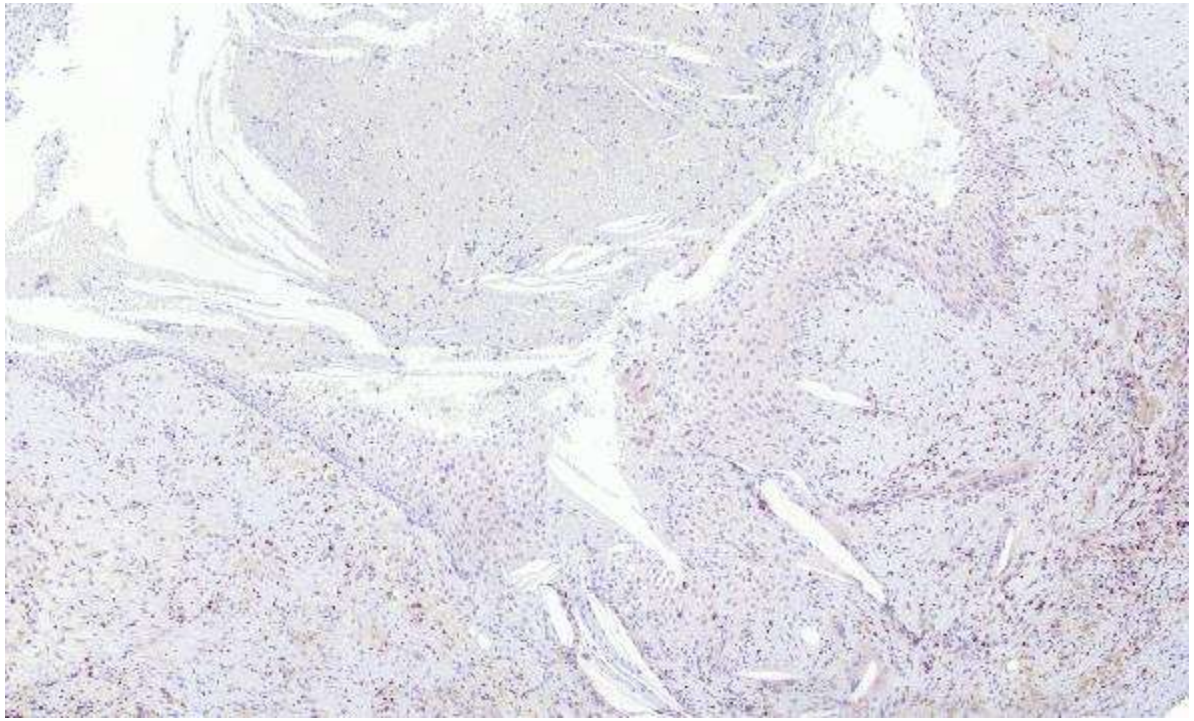

K17 – HE, CK17, bcl2 (x10)

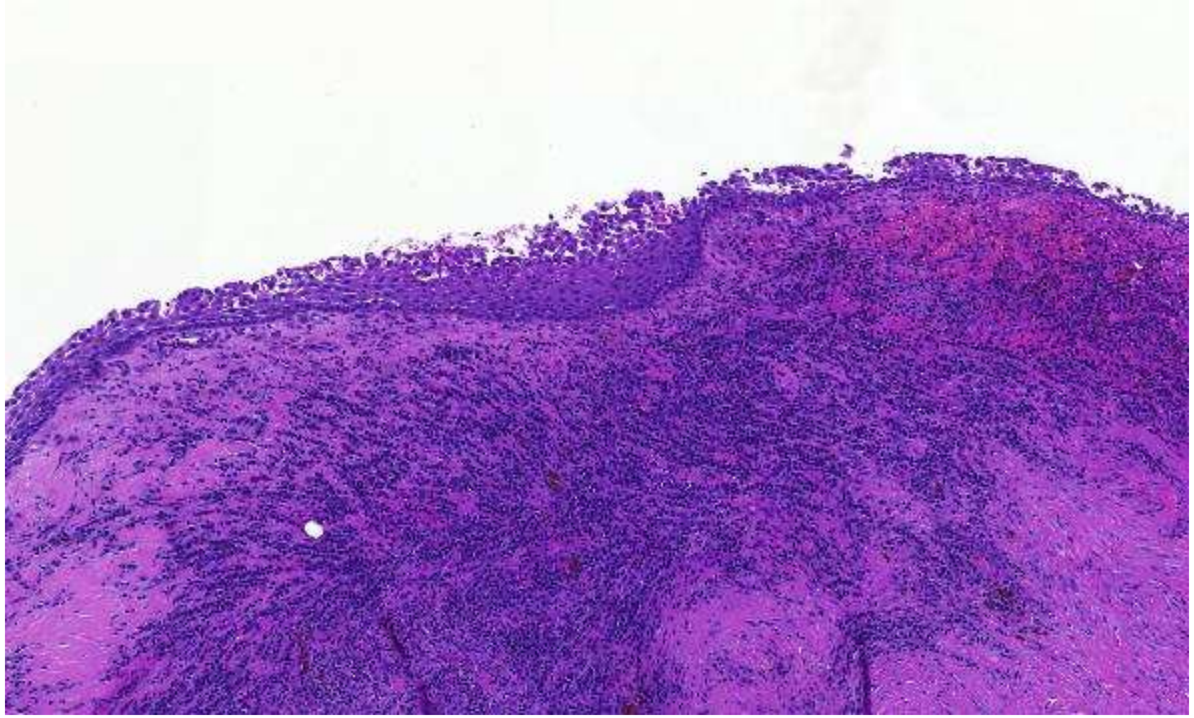

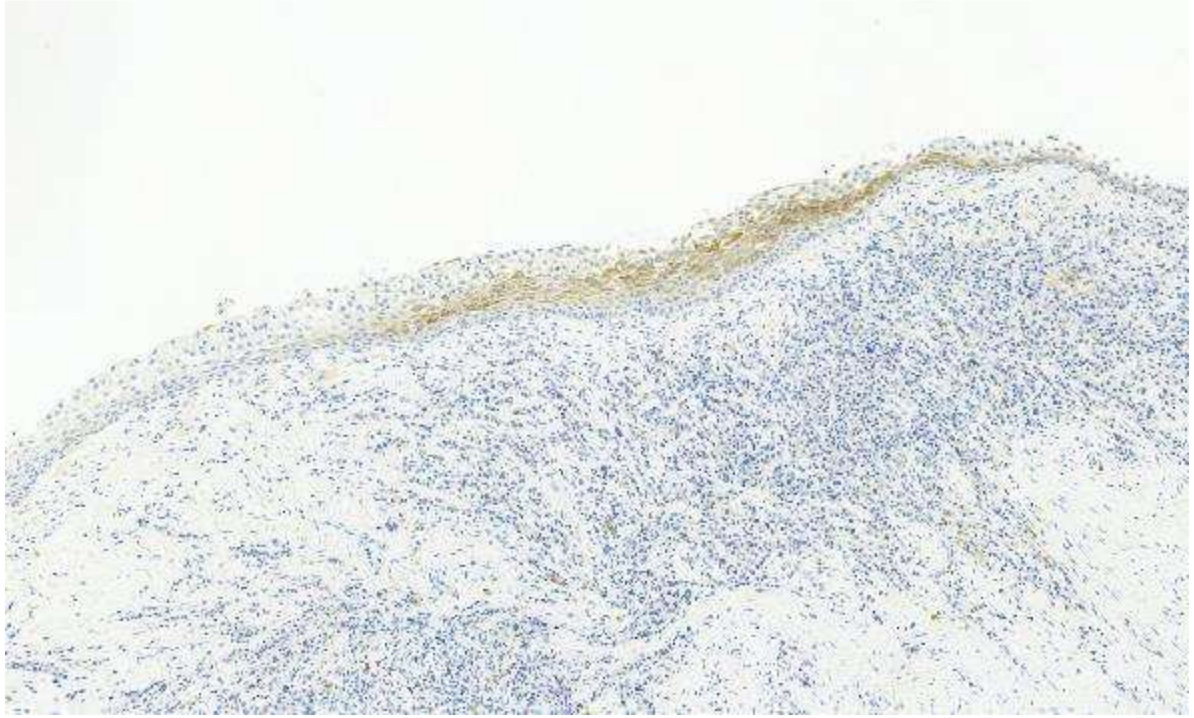

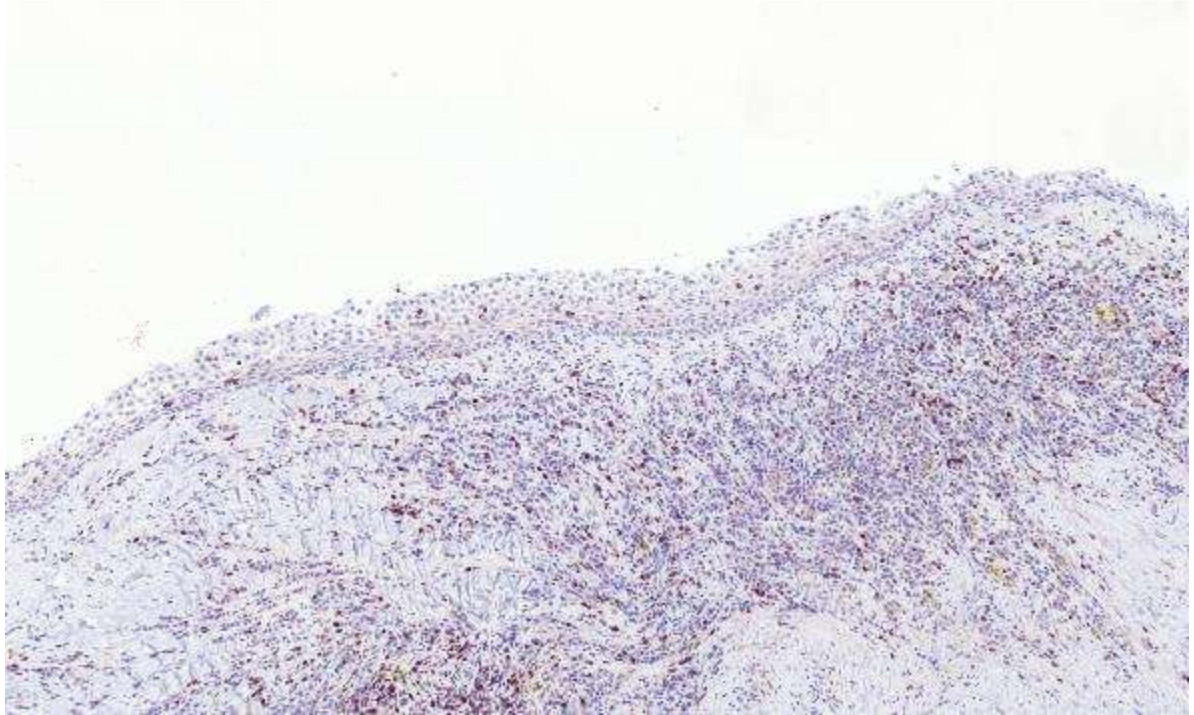

K17 – Conclusion

- H&E: relatively thick squamous epithelium (with focal thinning mostly due to desquamation), no parakeratosis, no wavy surface, no basal palisading, even epithelial-subepithelial contours
- Focal mixed inflammation of the cyst wall with cholesterol granuloma
- Bcl2: negative
- CK17: dominantly negative, with focal transepithelial staining with or without basal sparing
- Histopathology: inflammatory cyst, radicular cyst
- Combined clinical and pathological opinion: inflammatory collateral cyst / paradental cyst

Case K18

- 39-year-old male
- History: The patient noticed an enlarged lymph node in the mental region, removal of tooth 36 by the dentist was unsuccessful.
- Dental hygiene/status: partly missing, untreated teeth with the crown of tooth 36 missing.
- Radiology: Sharply circumscribed radiolucent lesion from the apex of tooth 36 to its base
- Operation: removal of the retained roots, followed by removal of the cyst
- Clinical diagnosis: mandibular cyst

K18 – OPT

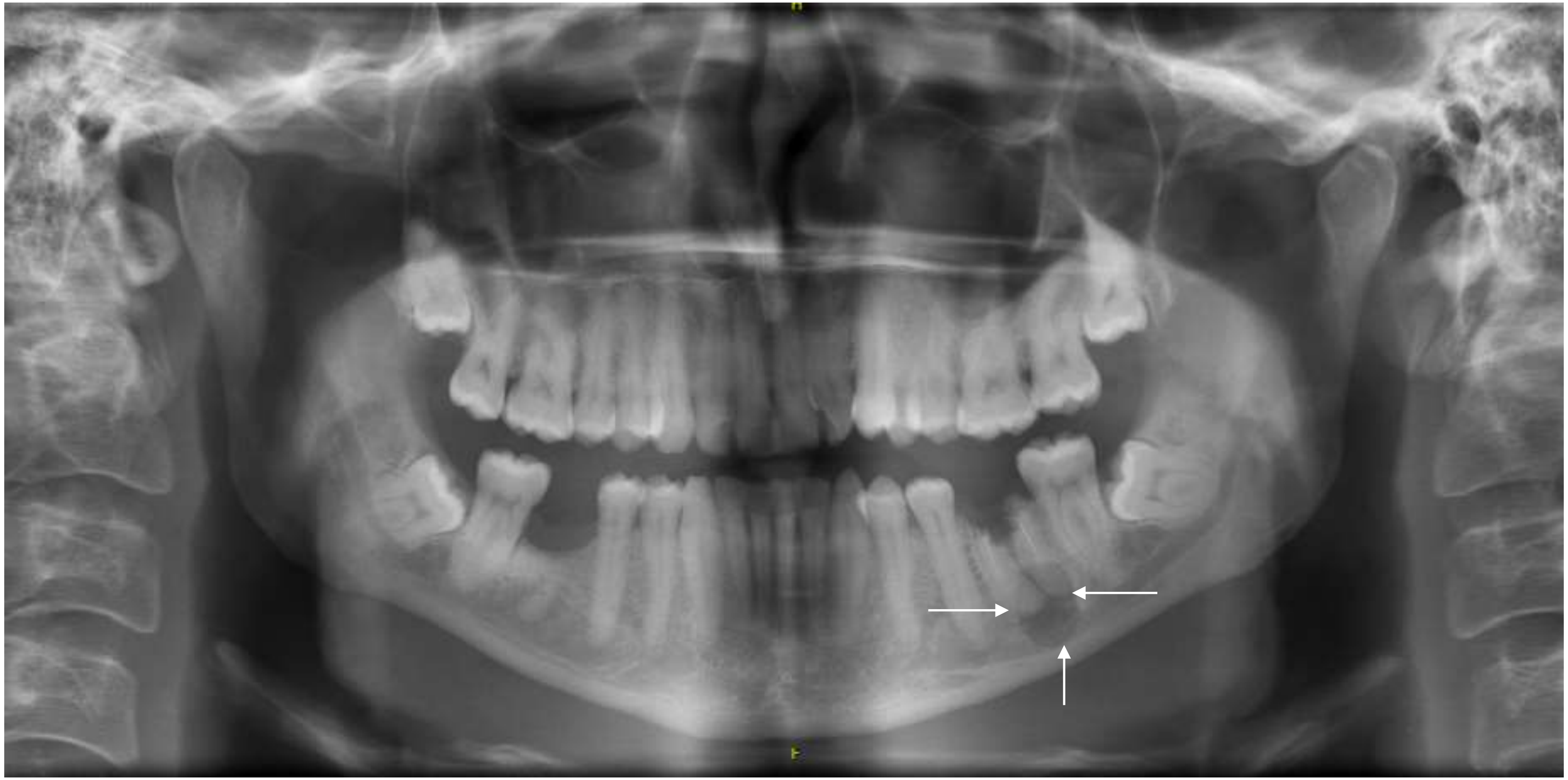

K18 – HE, CK17, bcl2 (x2)

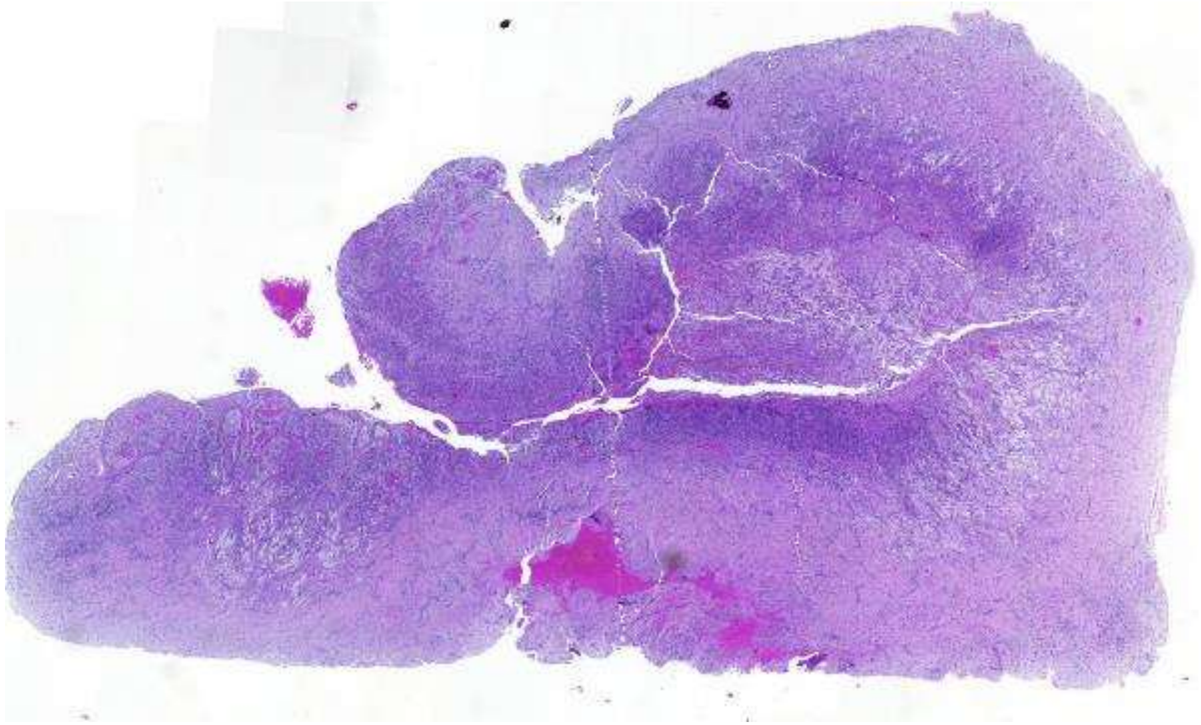

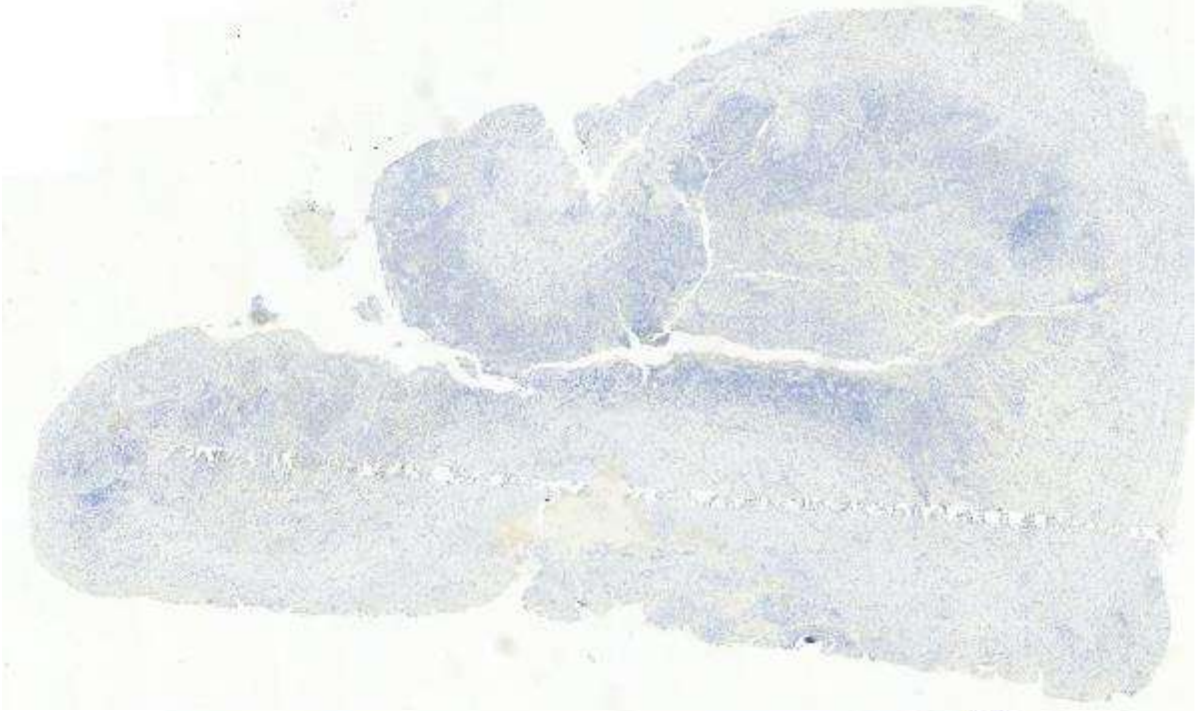

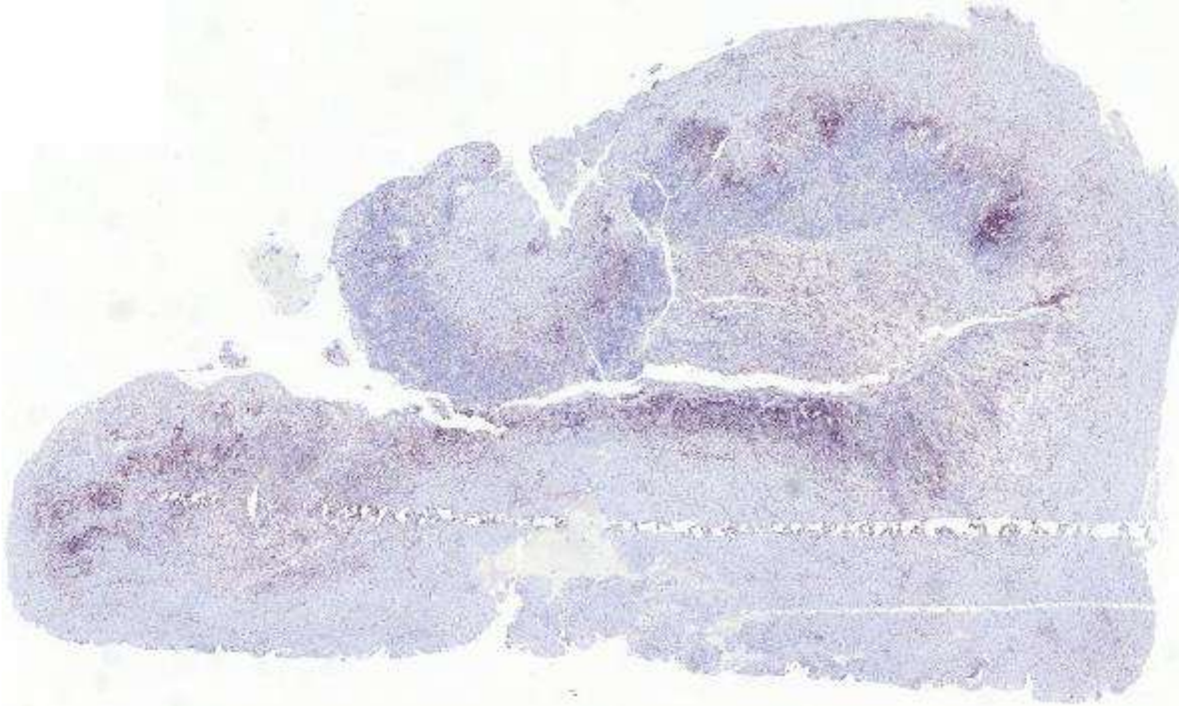

K18 – HE, CK17, bcl2 (x8)

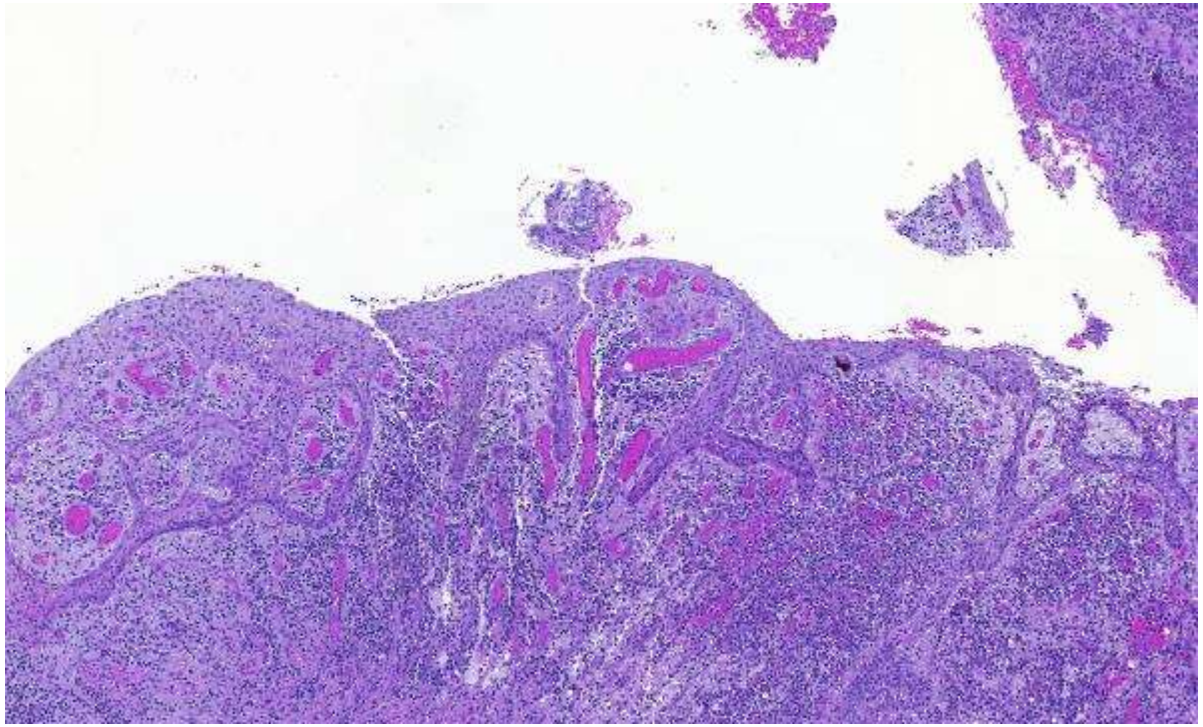

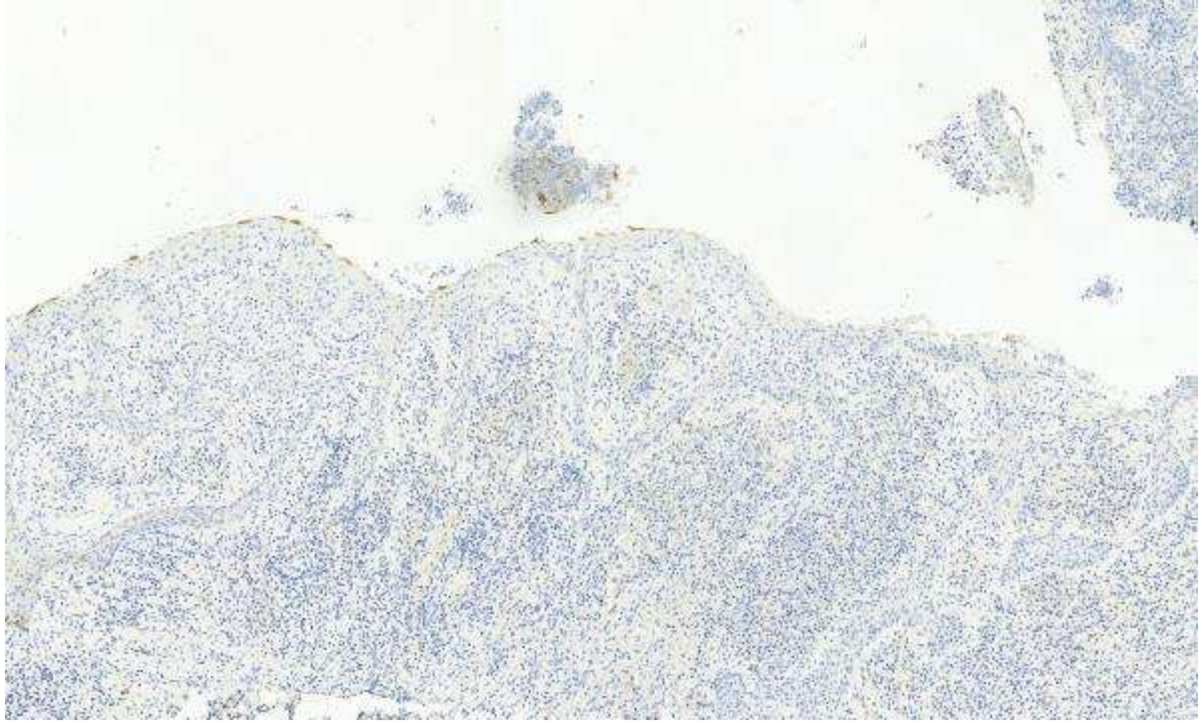

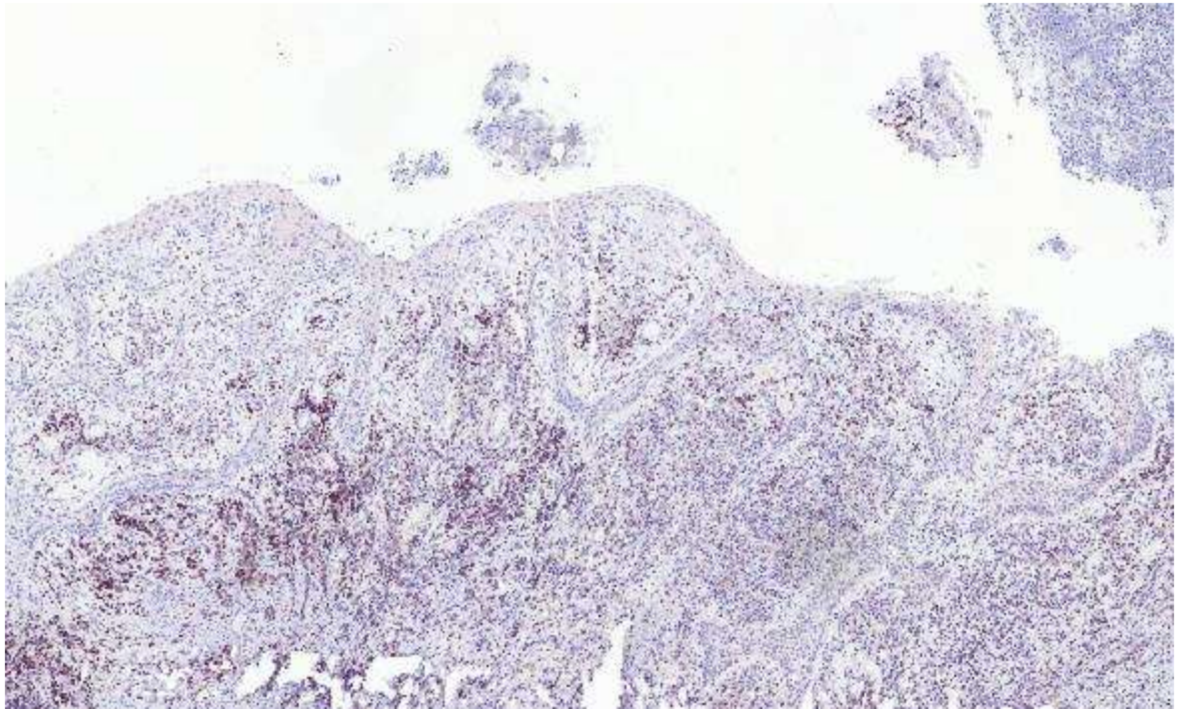

K18 – HE, CK17, bcl2 (x20)

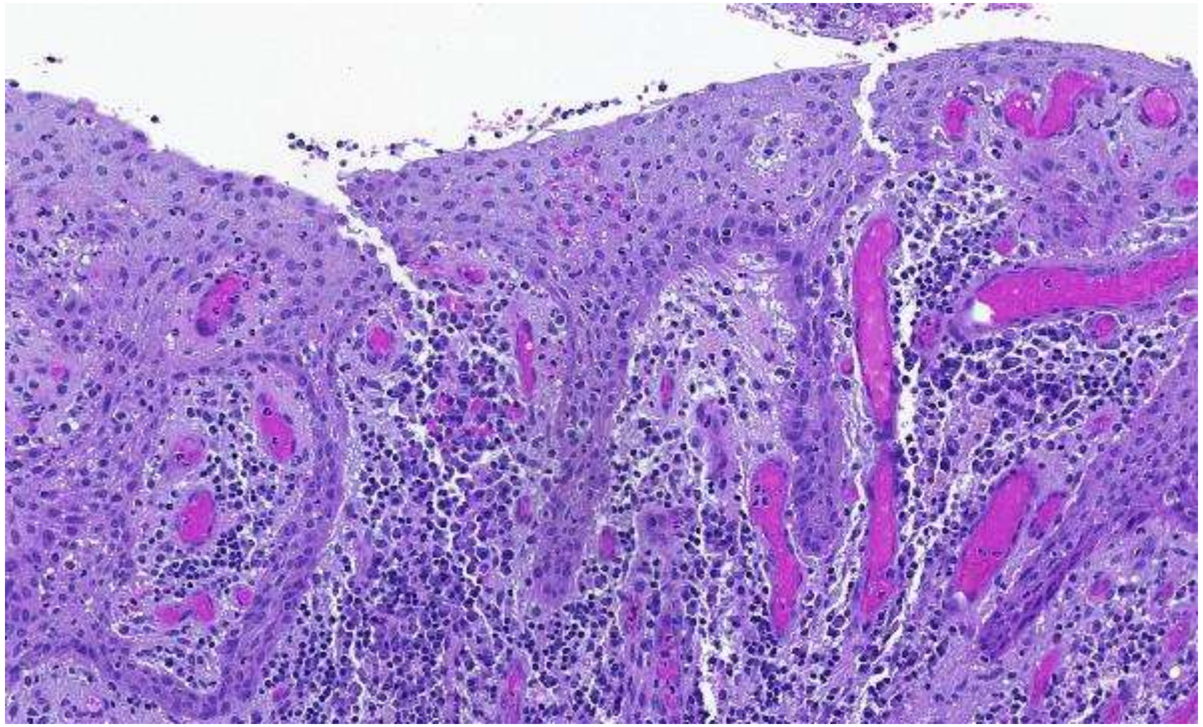

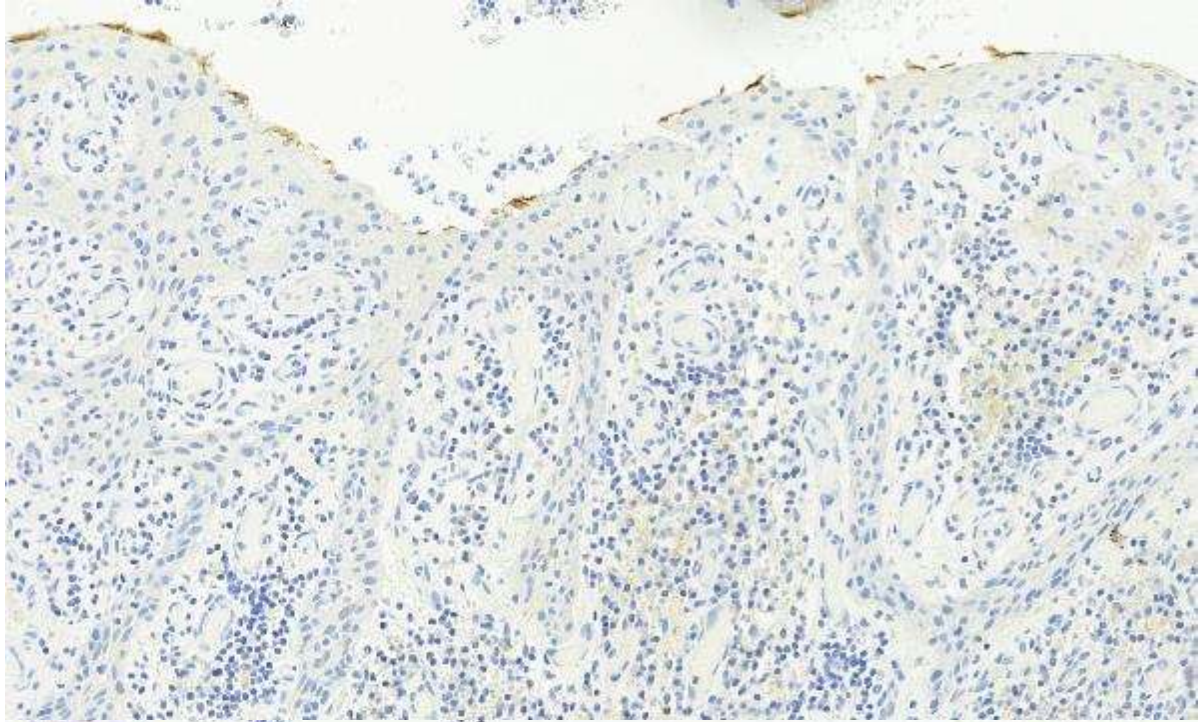

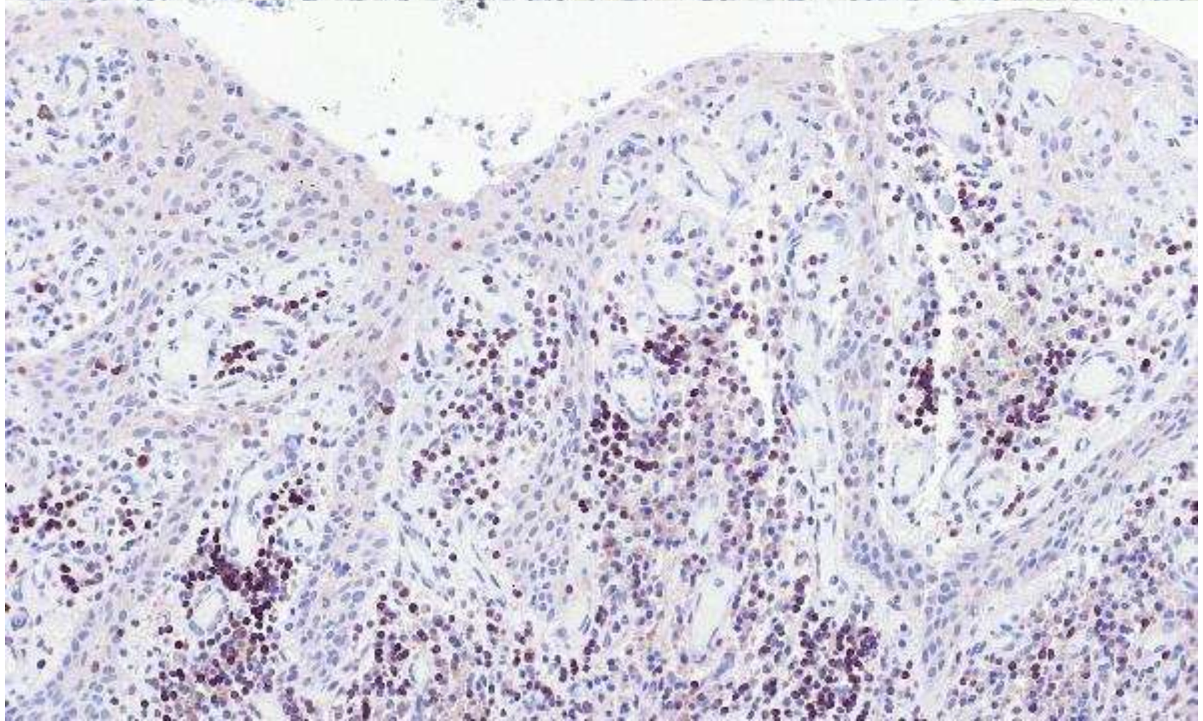

K18 – Conclusion

- H&E: relatively thick squamous epithelium, no parakeratosis, no wavy surface, no basal palisading, uneven epithelial-subepithelial contours
- Mixed inflammatory infiltrate of the cyst wall
- Bcl2: negative
- CK17: dominantly negative, with focal superficial staining
- Histopathology: radicular cyst
- Combined clinical and pathological opinion: radicular cyst

Case K19

- 58-year-old male
- History: The patient noticed a swelling, which was followed by the removal of a bridge and extraction of tooth (N.O.S.). He was referred for further management of a large mandibular lesion…
- Dental hygiene/status: partly missing, treated teeth; swelling at the vestibular inflexion at position of teeth 31-33.
- Radiology: Osteolytic lesion with scalloped contours between teeth 44-36 thinning the cortex of the mandible. Dental root at position of tooth 33(?)
- Operation: 1. Biopsy; 2. Excision of fistula orifice at position of tooth 32, removal of teeth 36, 31, 41 and 42, removal of the cyst through window between positions 34-32.
- Clinical diagnosis: keratocyst?

K19 – OPT

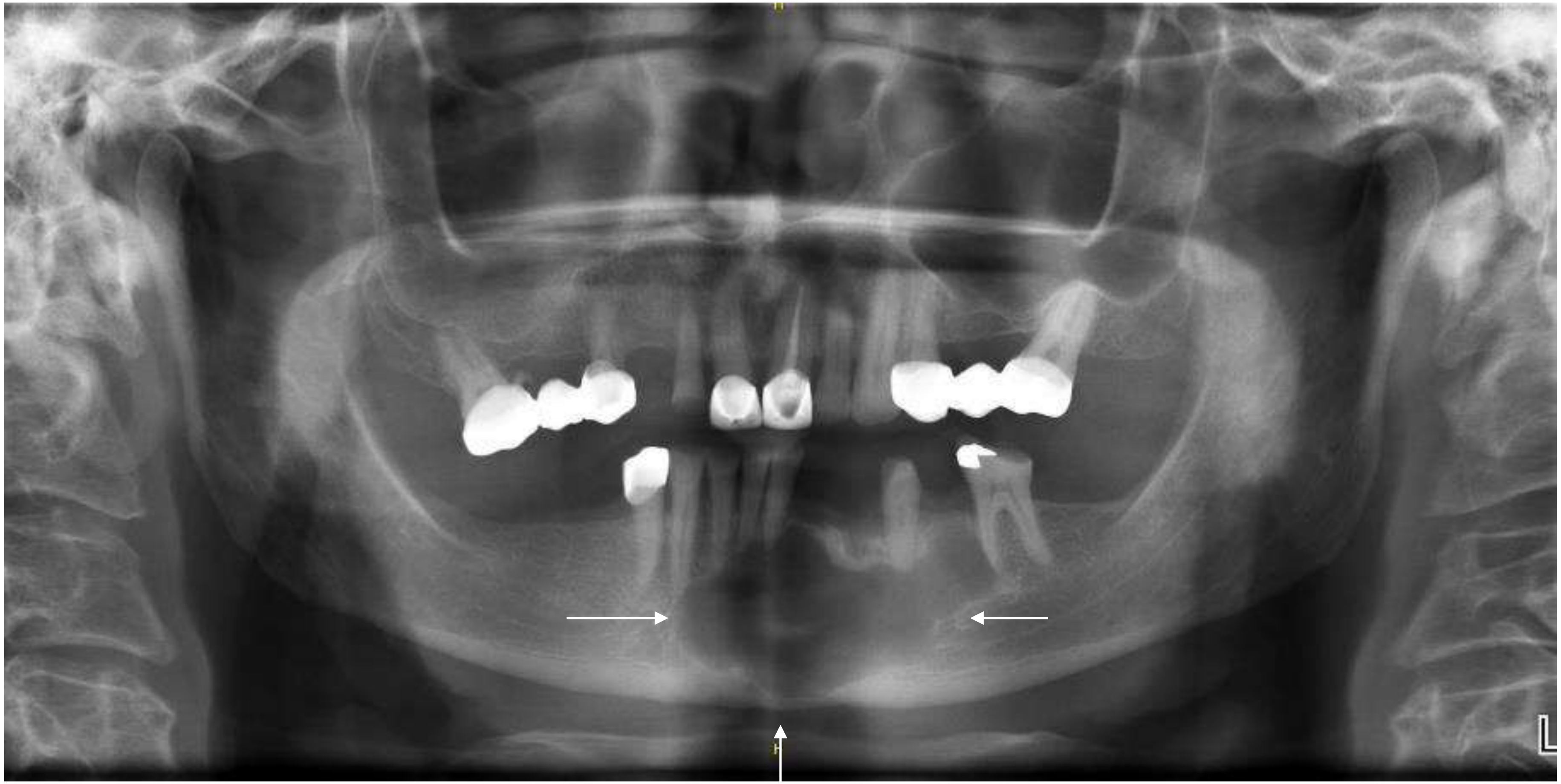

K19 – CT coronal

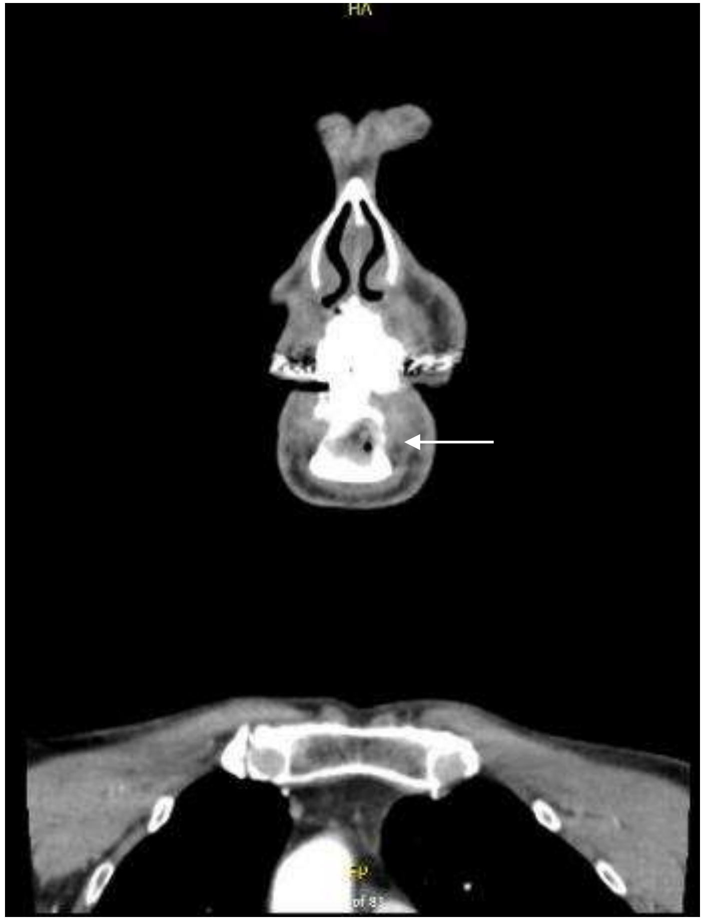

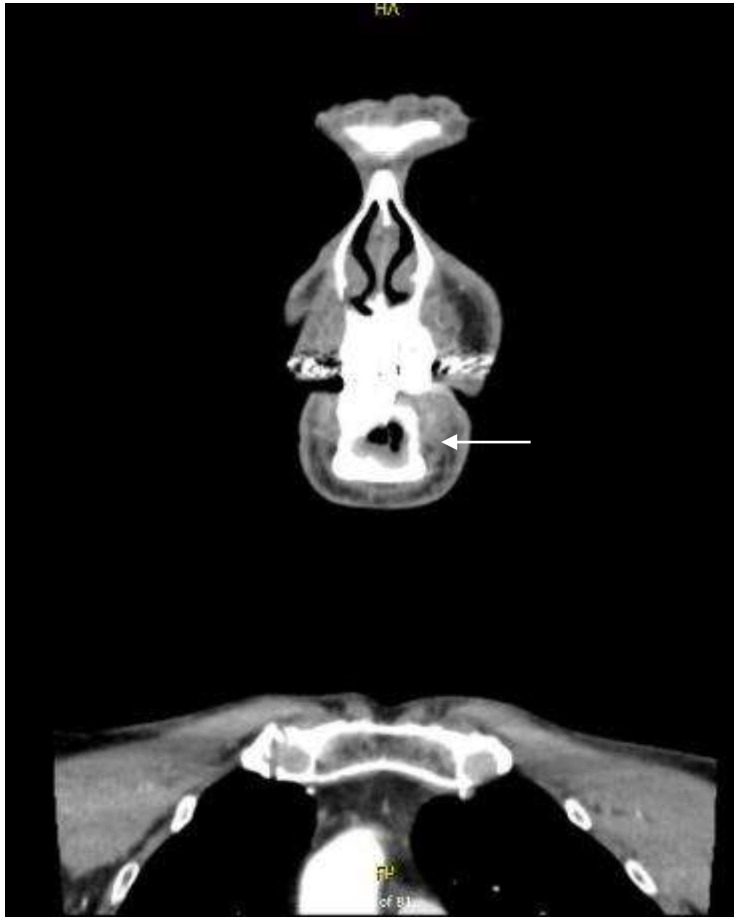

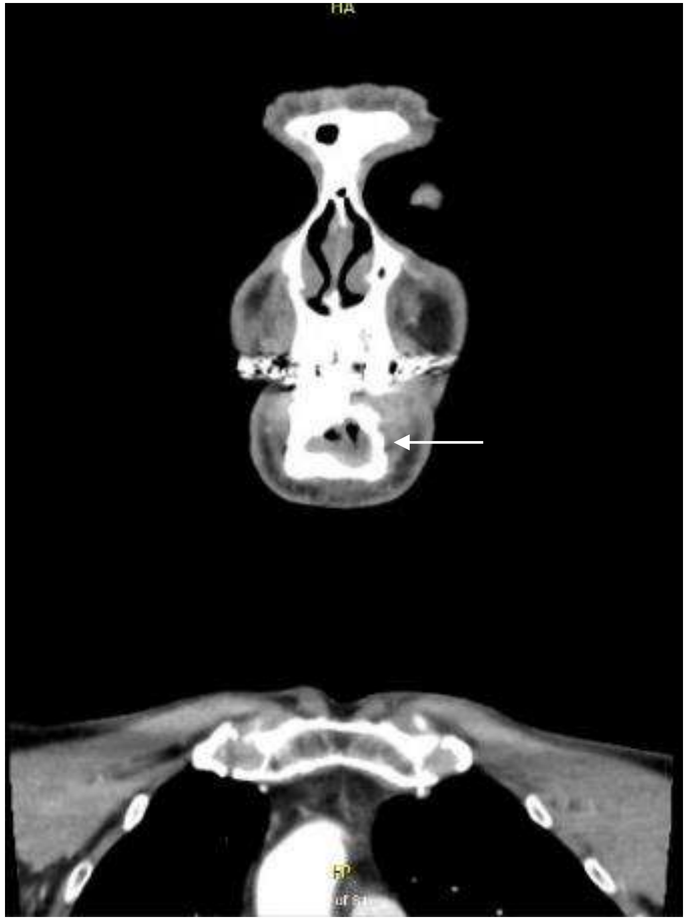

K19 CT – coronal

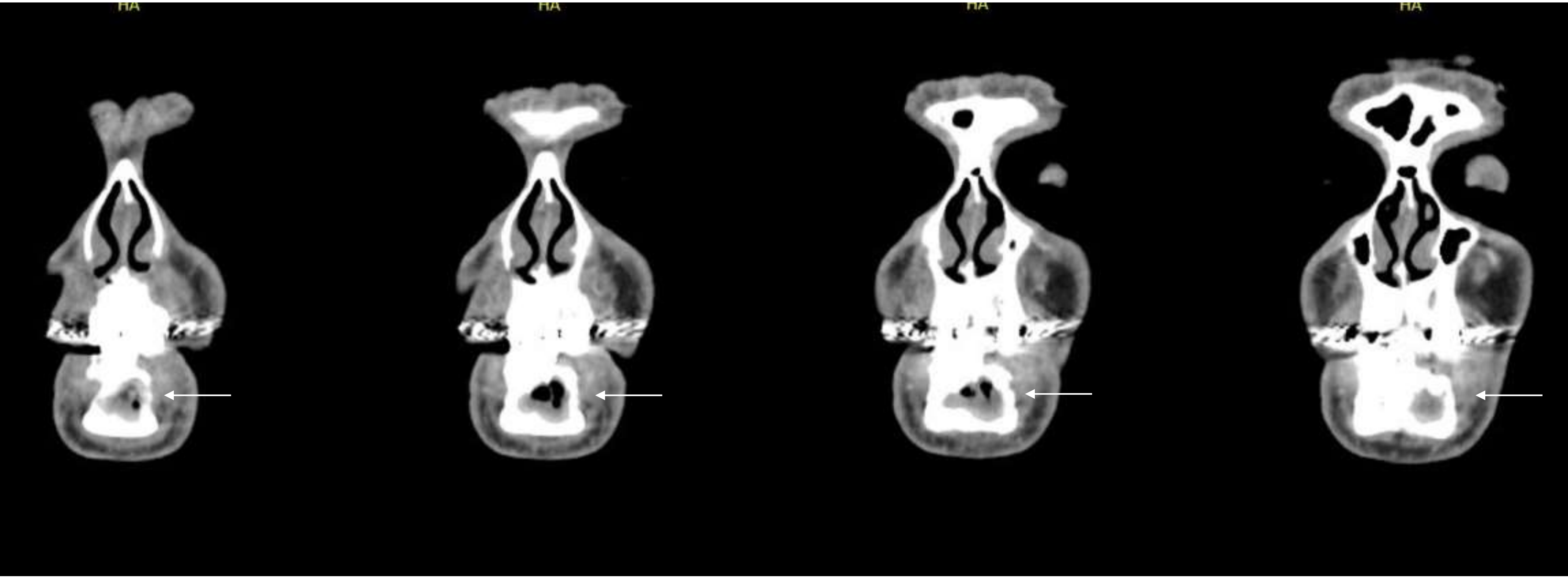

K19 – CT sagittal

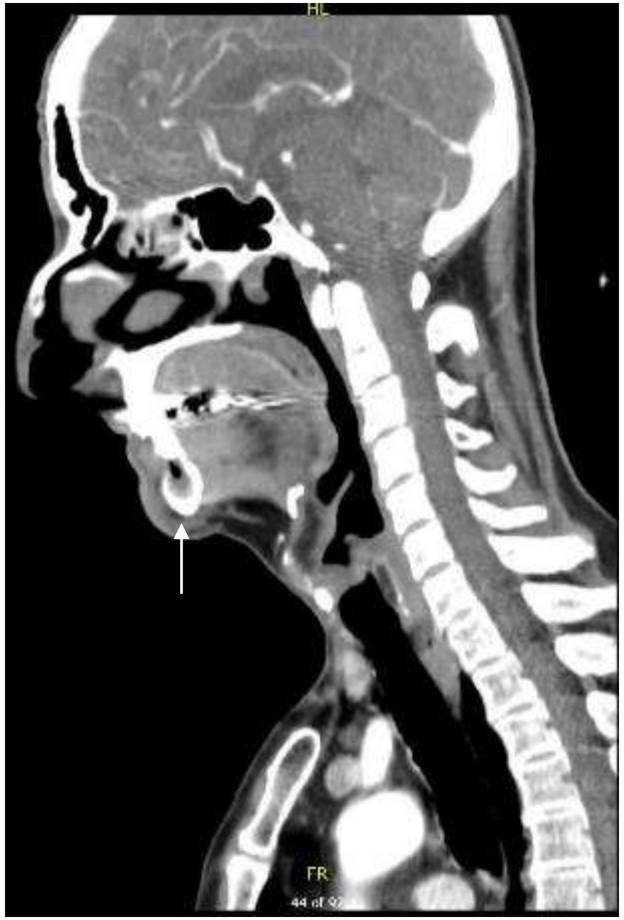

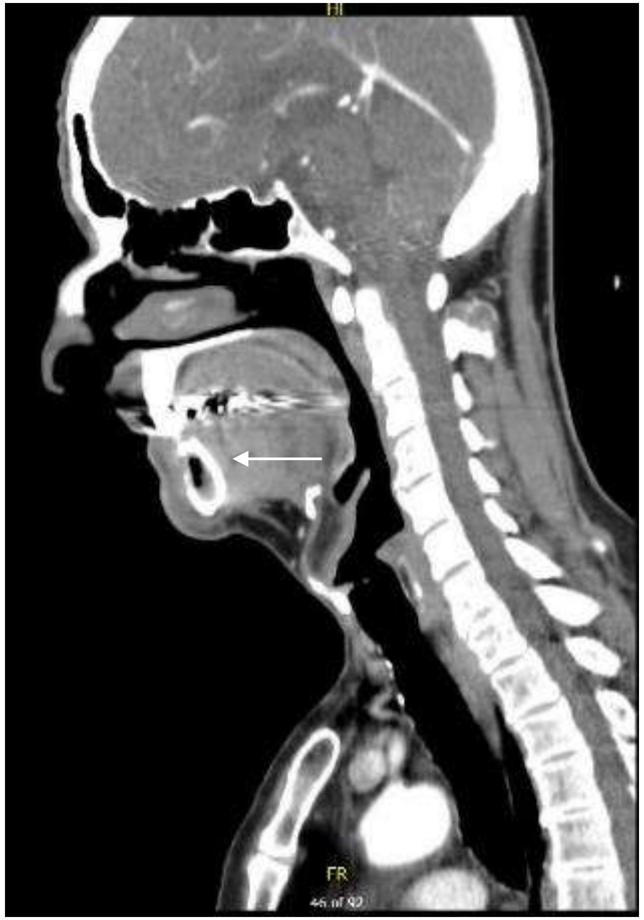

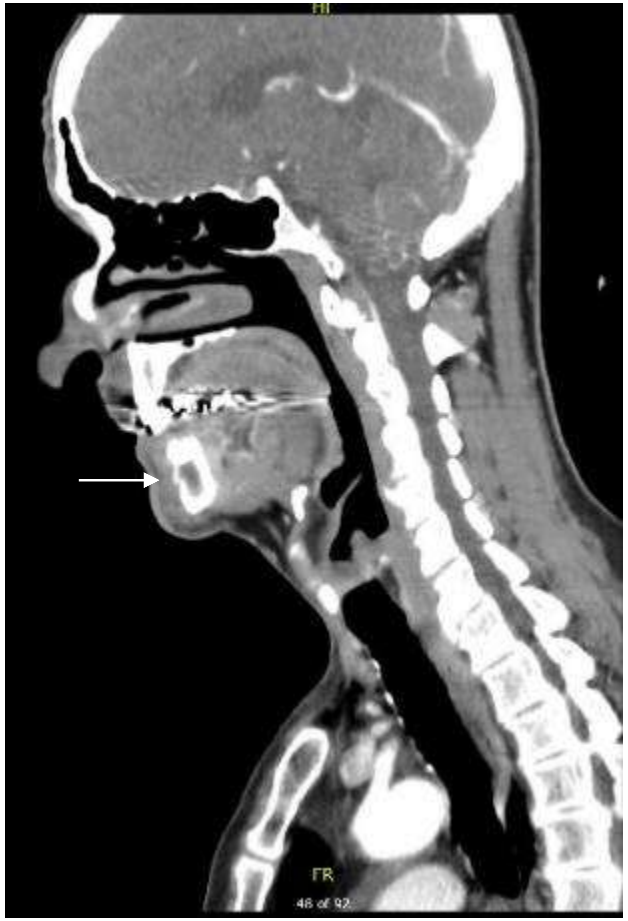

K19 – CT sagittal - slices 44, 46, 47 & 48 /92

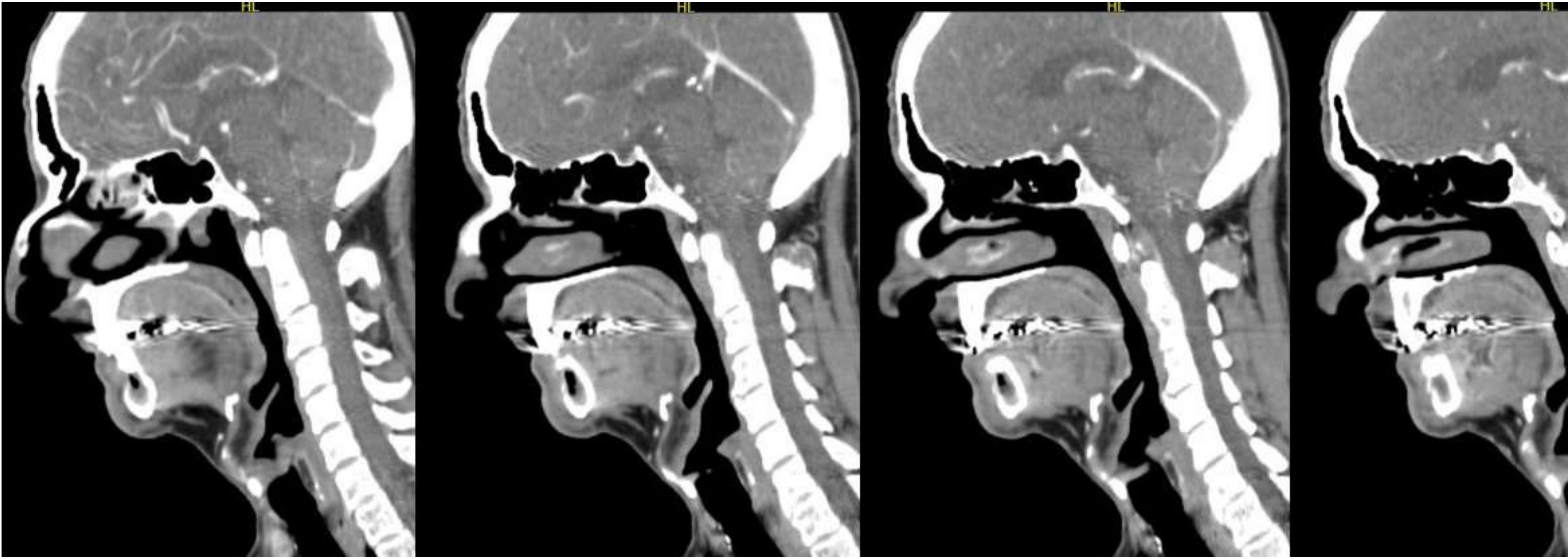

K19 – CT axial

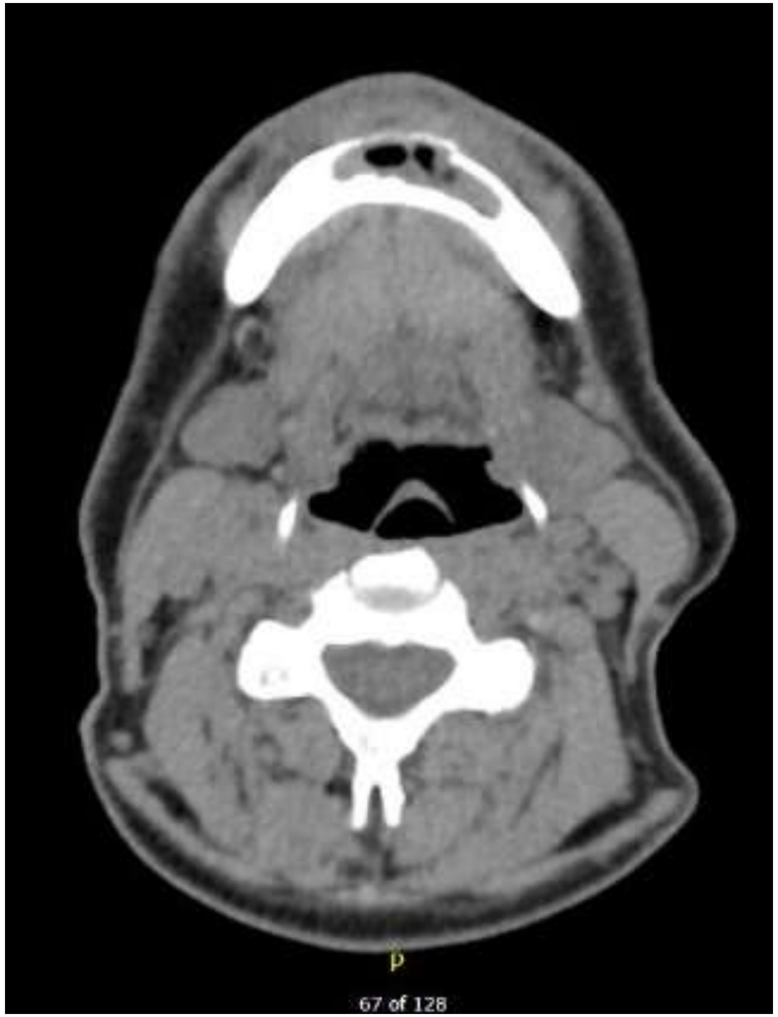

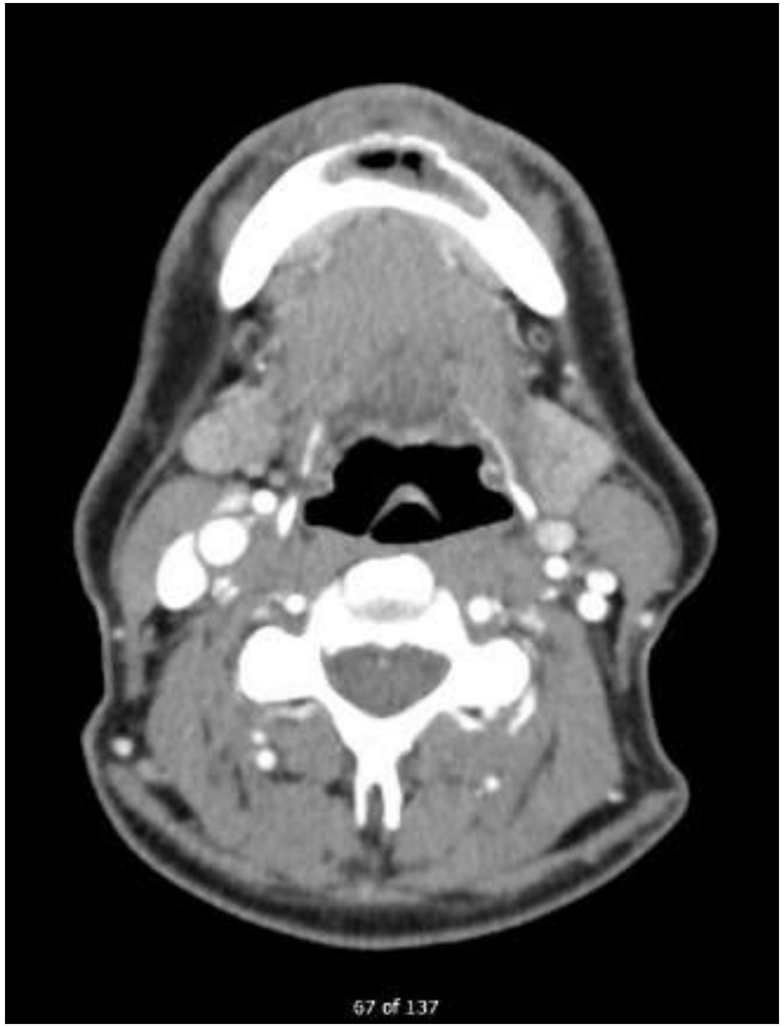

K19 – HE, CK17, bcl2 (x1.5)

Bone fragments requiring decalcination

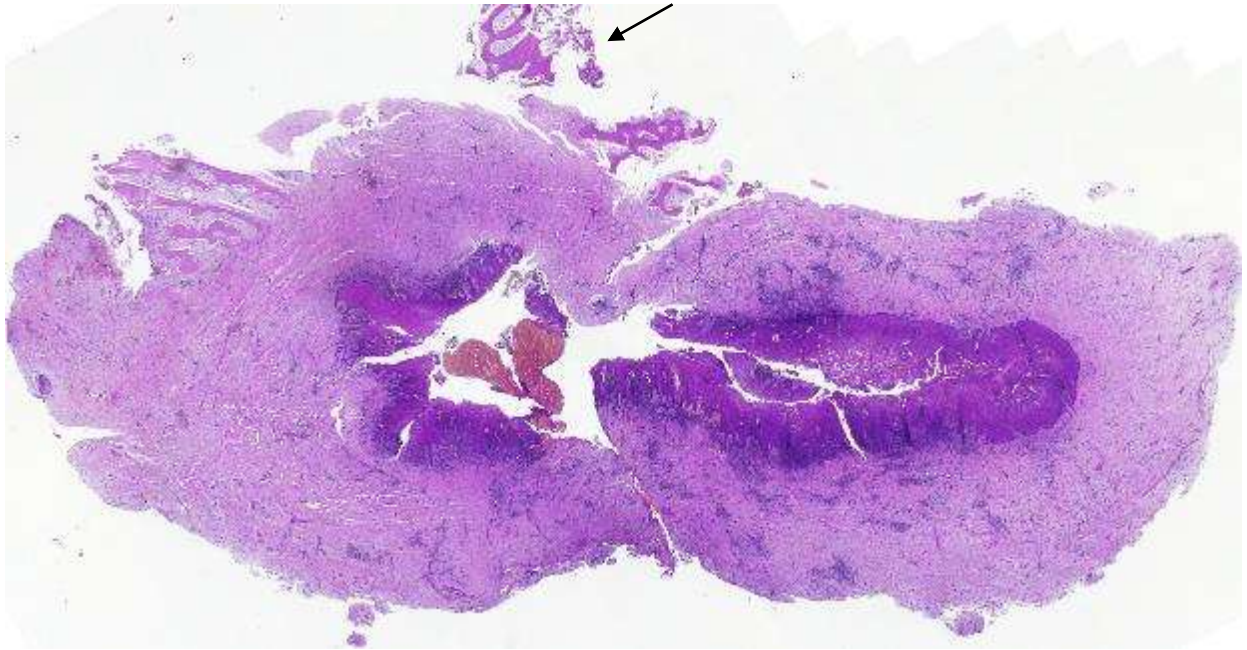

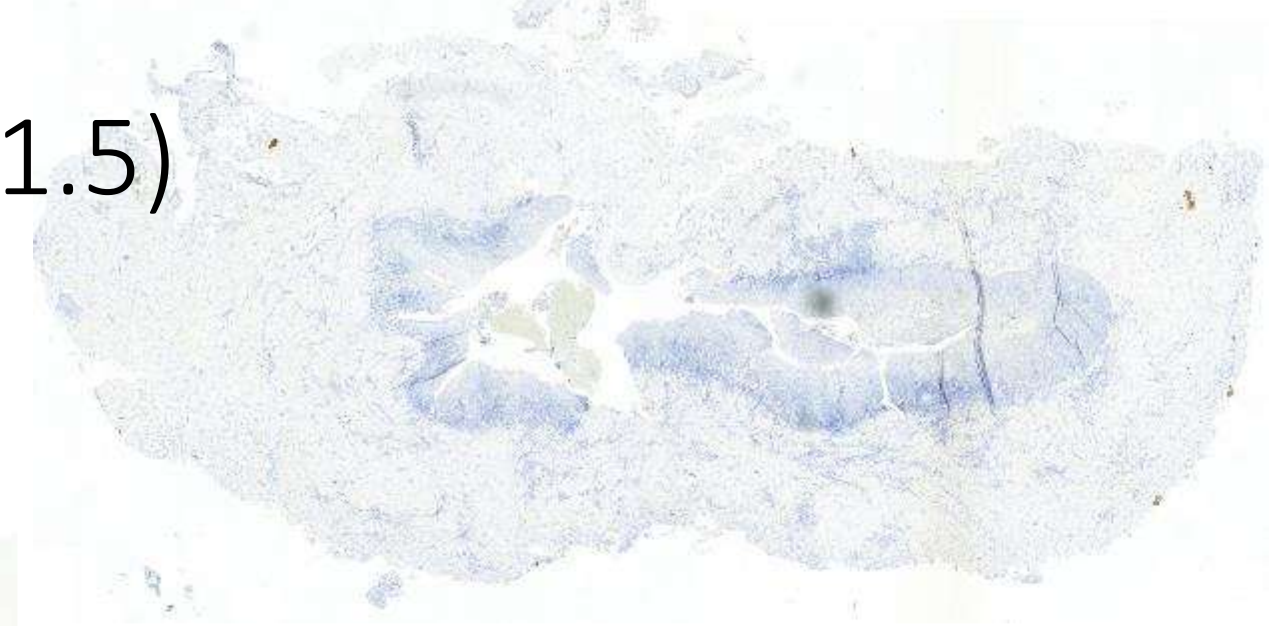

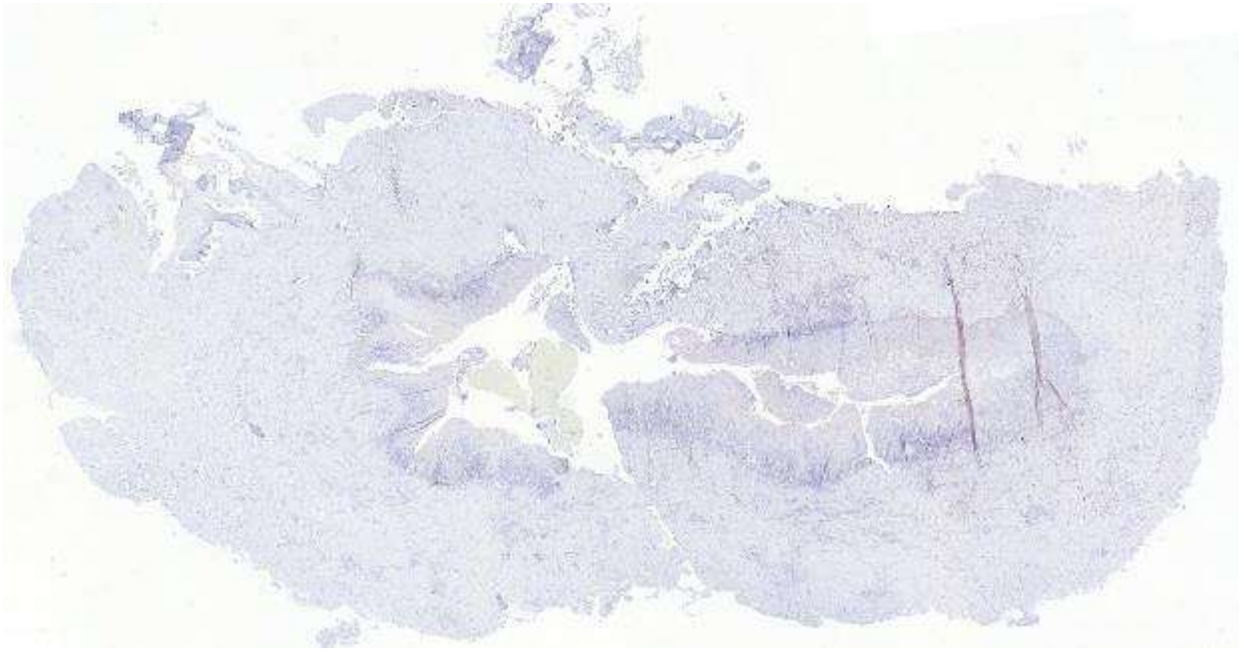

K19 – HE, CK17, bcl2 (x10)

Few basal cells staining next to subepithelial connective tissue papillae (↑)

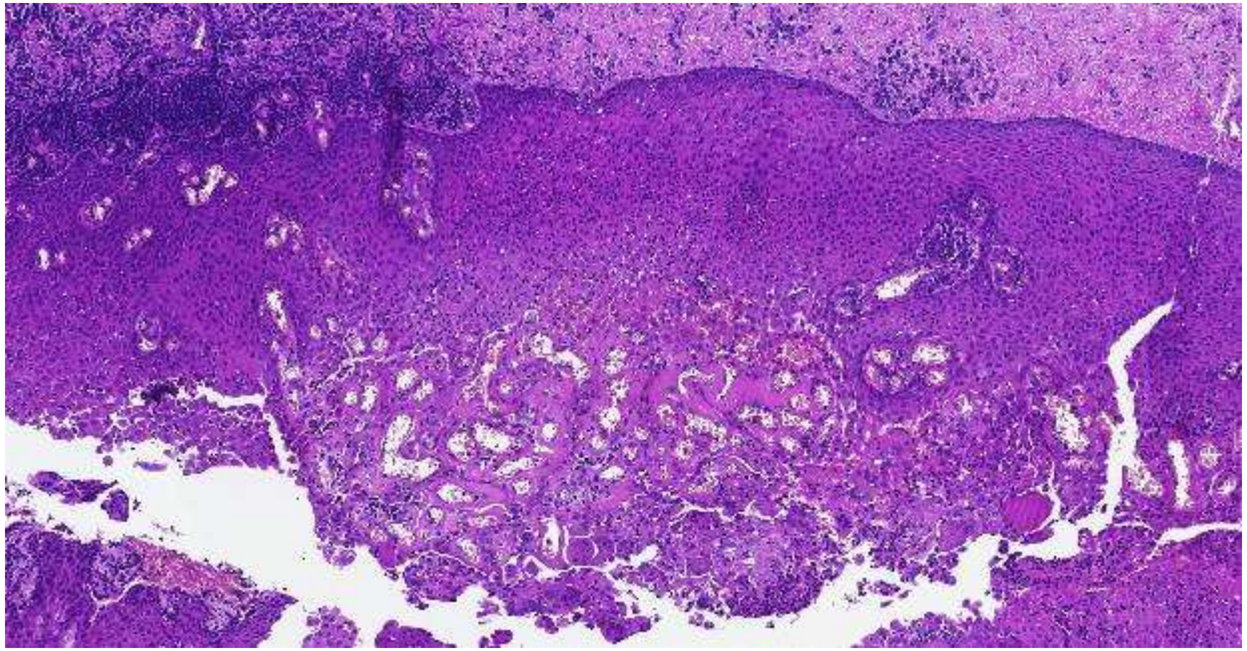

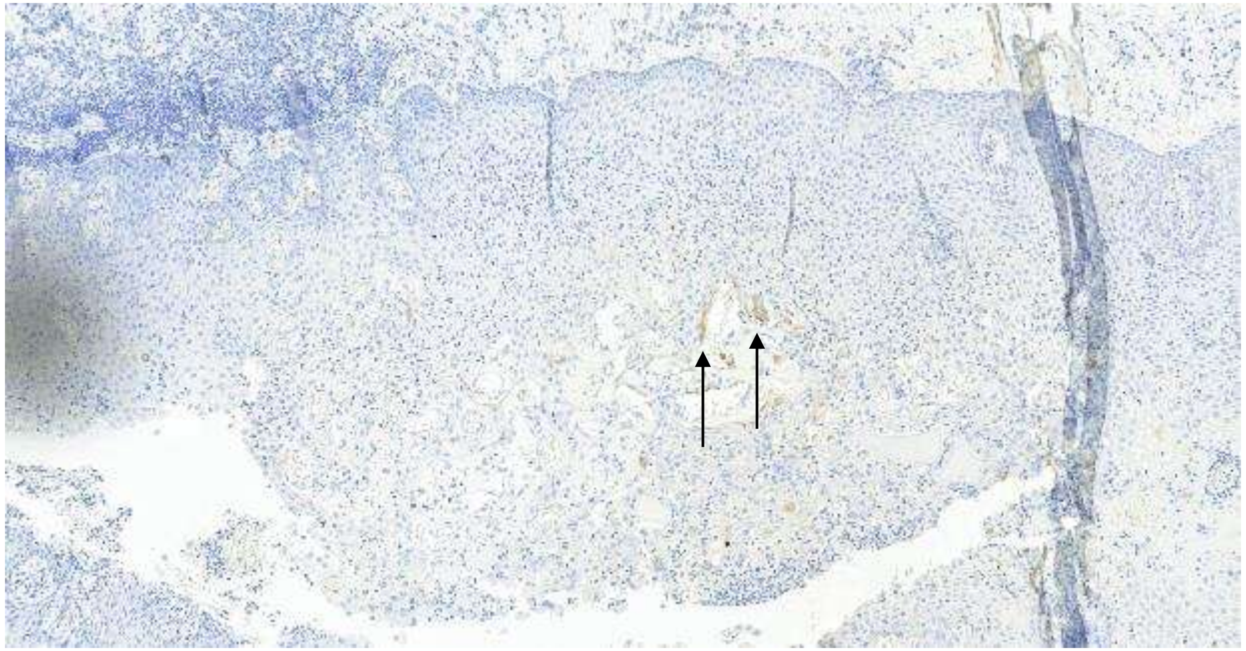

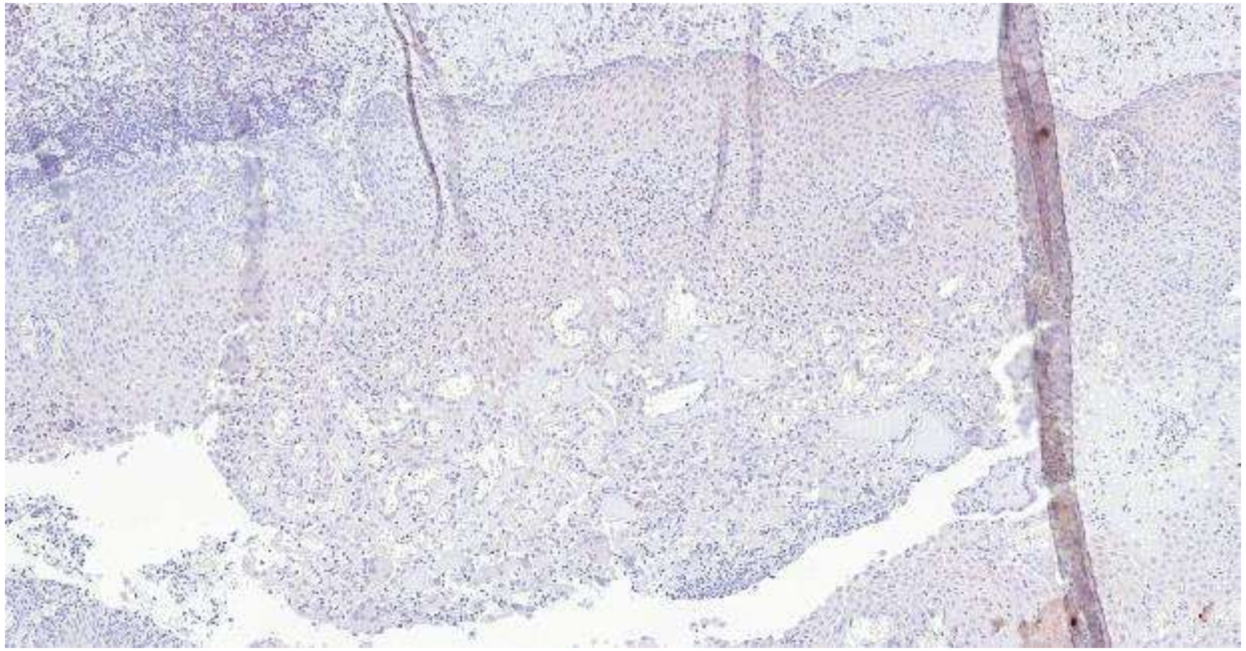

K19 – HE, CK17, bcl2 (x18)

Note faint bcl2 staining of lymphocytes probably due to decalcination (↑)

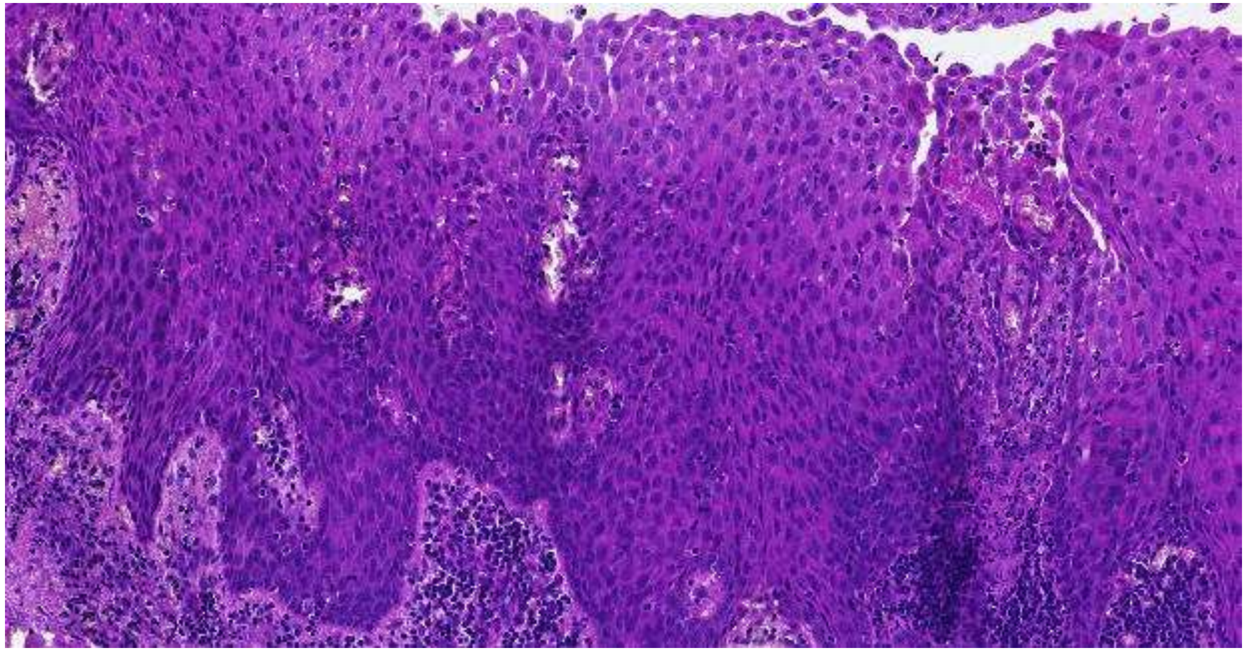

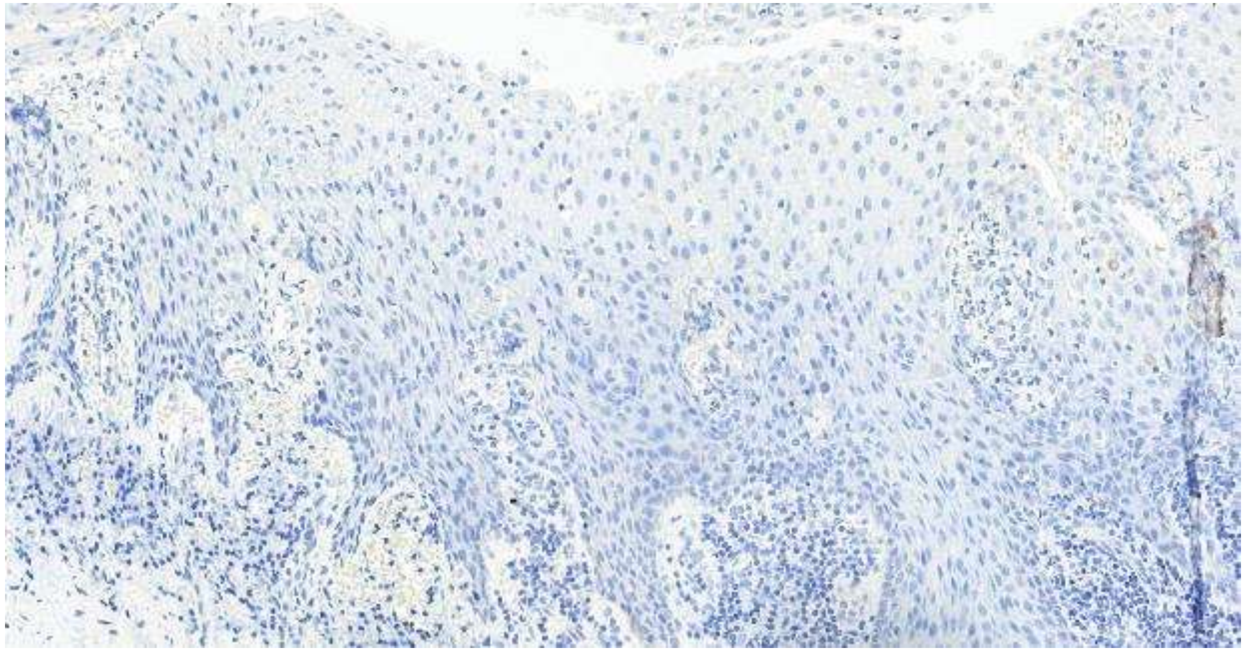

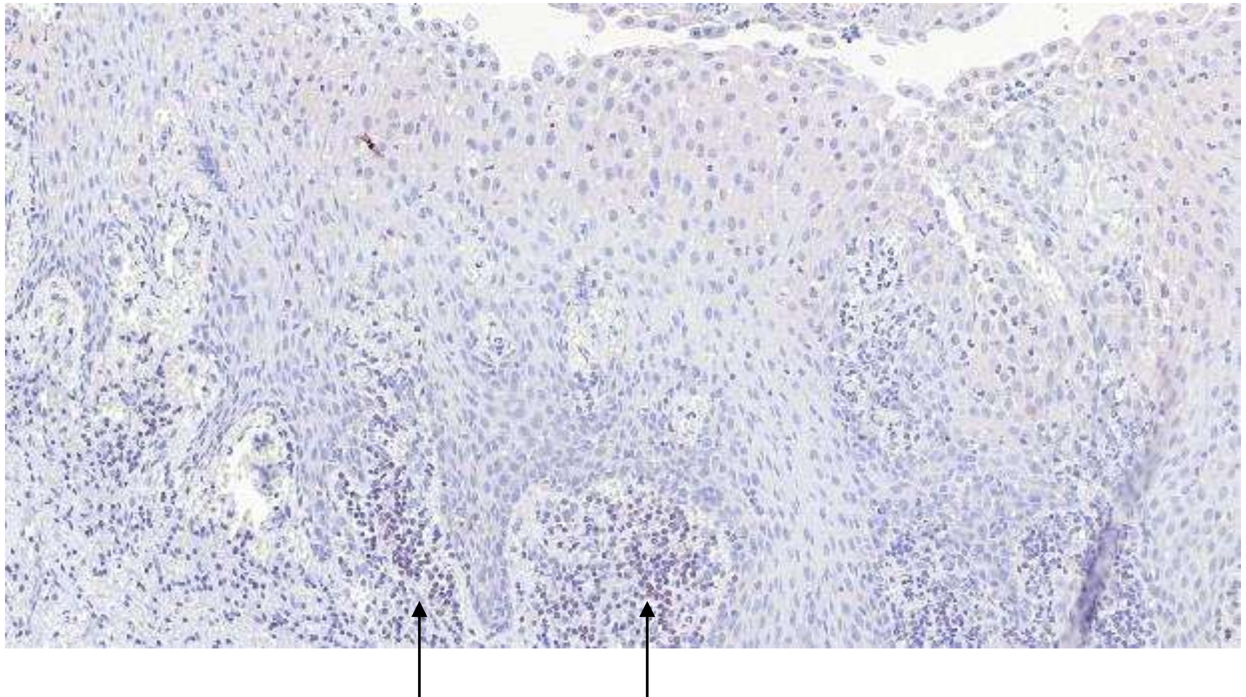

K19 – HE, CK17, bcl2 (x5)

Previous biopsy: focal superficial CK17 positivity & bcl2 negativity with good internal lymphocyte control

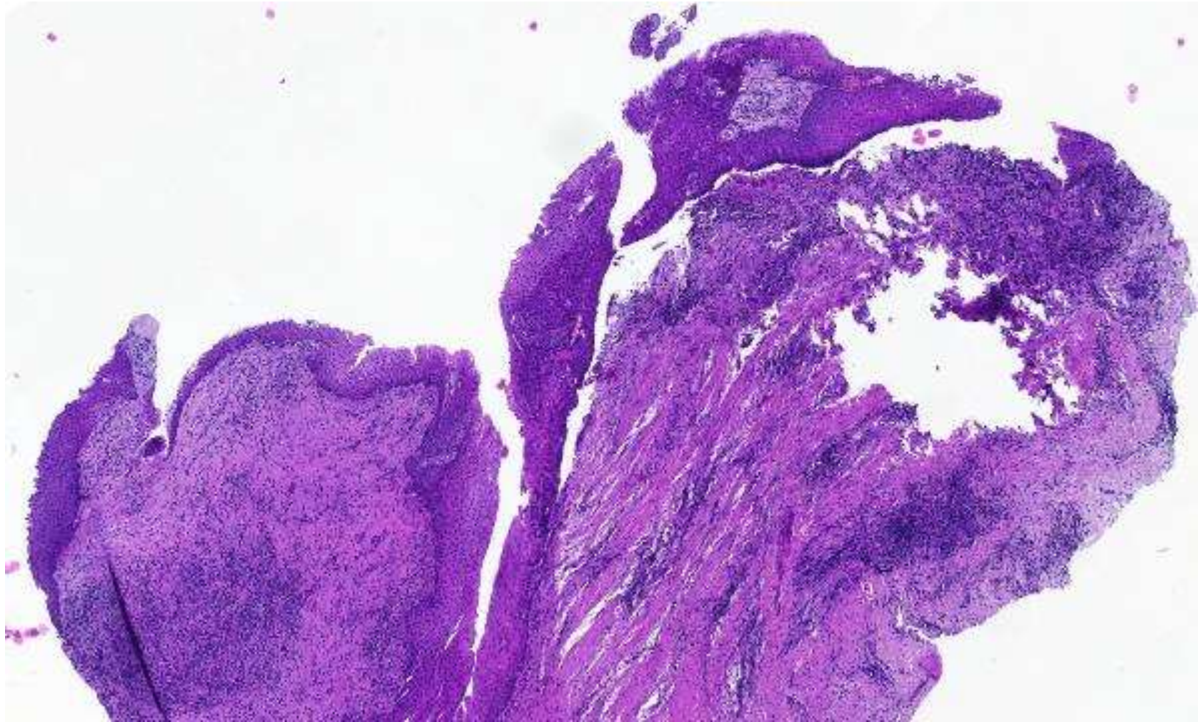

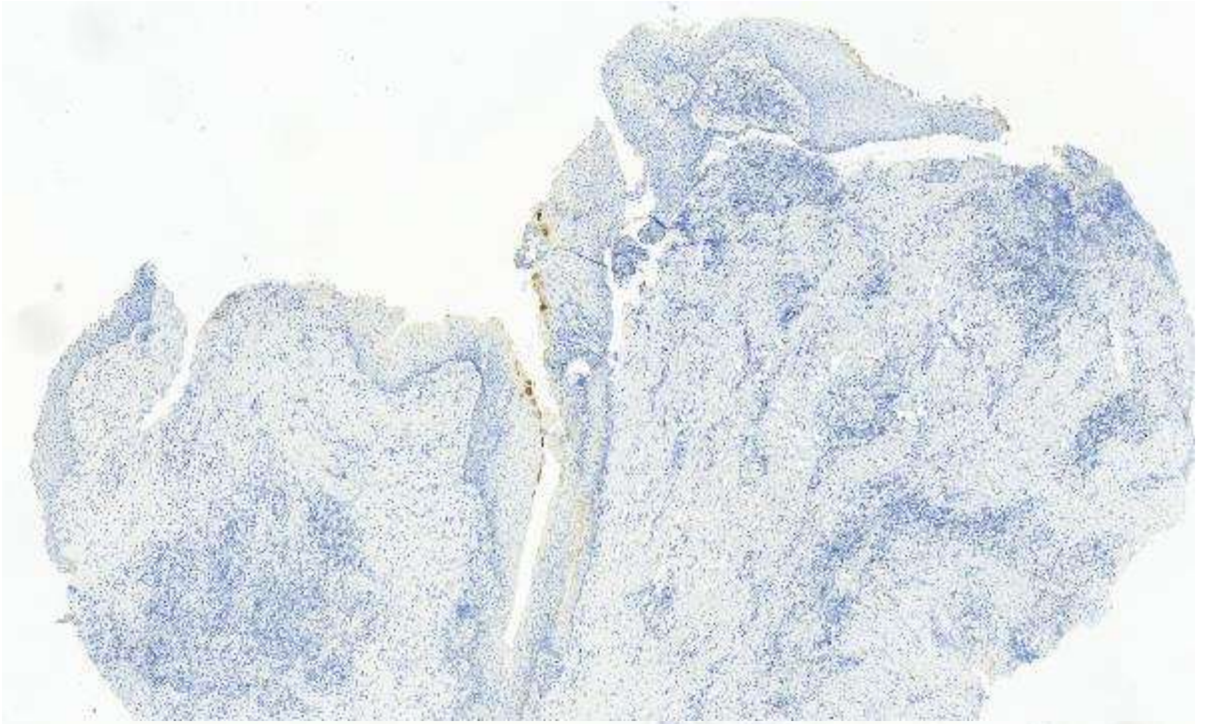

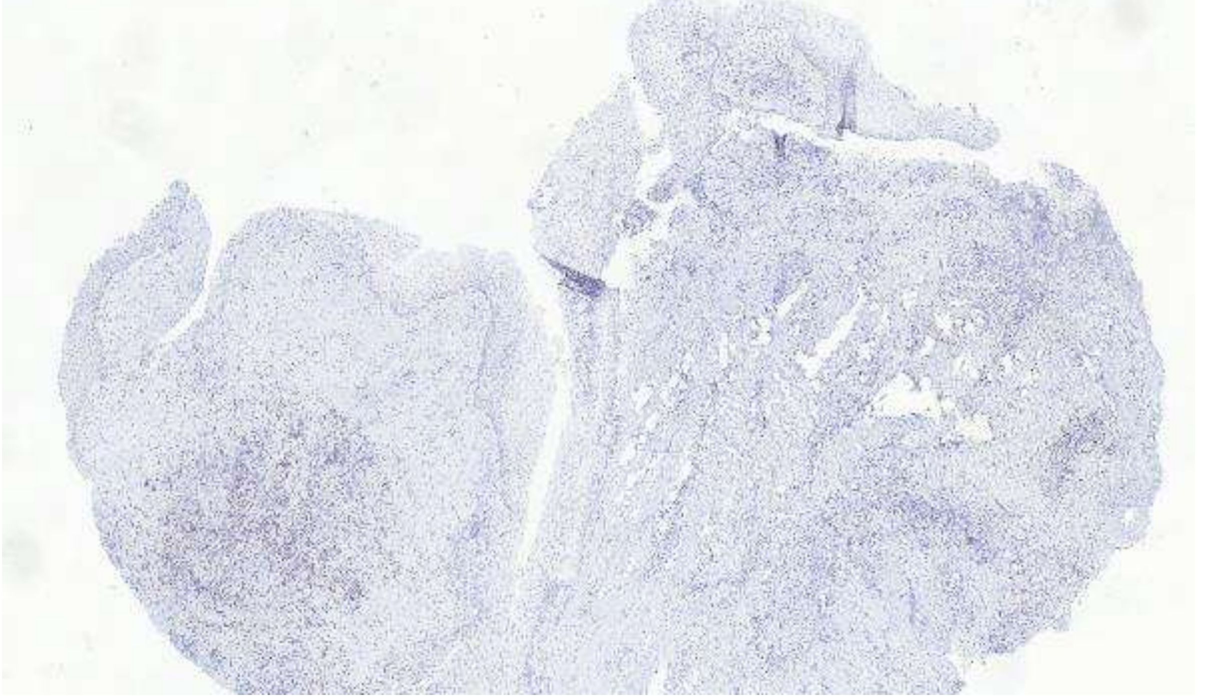

K19 – bcl2 (x20) - negative

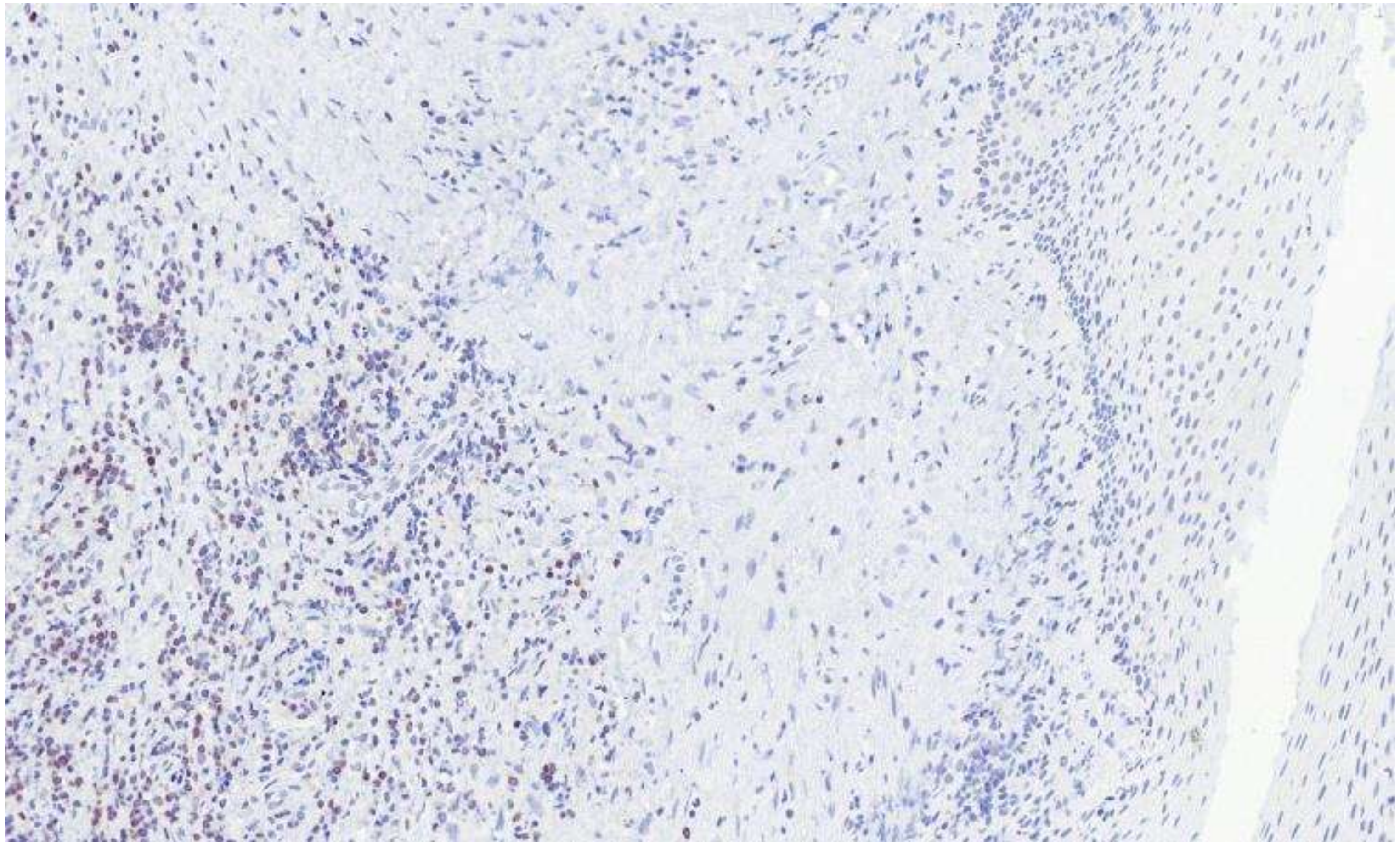

K19 – Conclusion

- H&E: relatively thick (focally thin in biopsy sample) squamous epithelium, no parakeratosis, no wavy surface, no obvious basal palisading, uneven (partly even in biopsy specimen) epithelial-subepithelial contours
- Prominent chronic inflammation of the cyst wall with areas of mixed inflammatory infiltrate including neutrophils
- Bcl2: negative (weak control staining of lymphocytes on decalcinated material)
- CK17: dominantly negative, with focal superficial staining and a few basal cells staining next to subepithelial connective tissue papillae
- Histopathology: inflammatory cyst (NOS) on biopsy („neither proving OKC nor excluding OKC with secondary epithelial changes”) ; inflammatory cyst /radicular cyst on cystectomy specimen
- Combined clinical and pathological opinion: most consistent with inflamed OKC

Case – K20

- 41-year-old female
- History: The patient noticed the swelling of the palatum more than half a year before, this had been incised and the root treatment of tooth 12 was also started. The swelling returned several times and surgical drainage was also done.
- Dental hygiene/status: partly missing, treated teeth, roots of teeth 21, 11 through 14 are filled, crowns include temporary fillings. The cyst seen on radiology is palpable.
- Radiology: Unilocular radiolucent lesion between teeth 15 and 11 (+ osteolytic lesions at the apex of roots of teeth 16 and 18)
- Operation: Removal of the cyst through window between positions teeth 15-11 and resection of roots of teeth 14-11.
- Clinical diagnosis: „maxillary cyst” (NOS)

K20 – OPT2

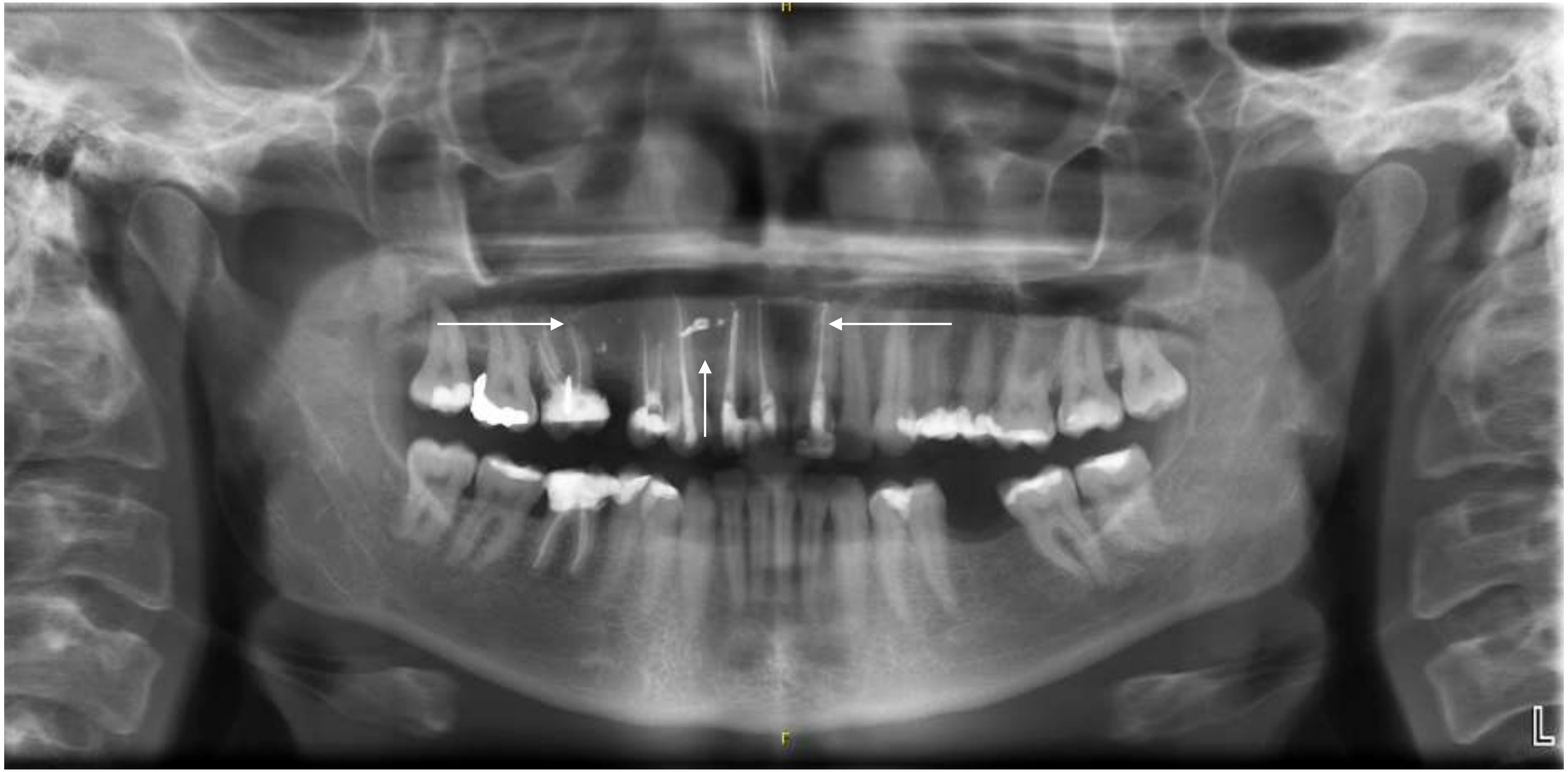

K20 – cholesterol clefts

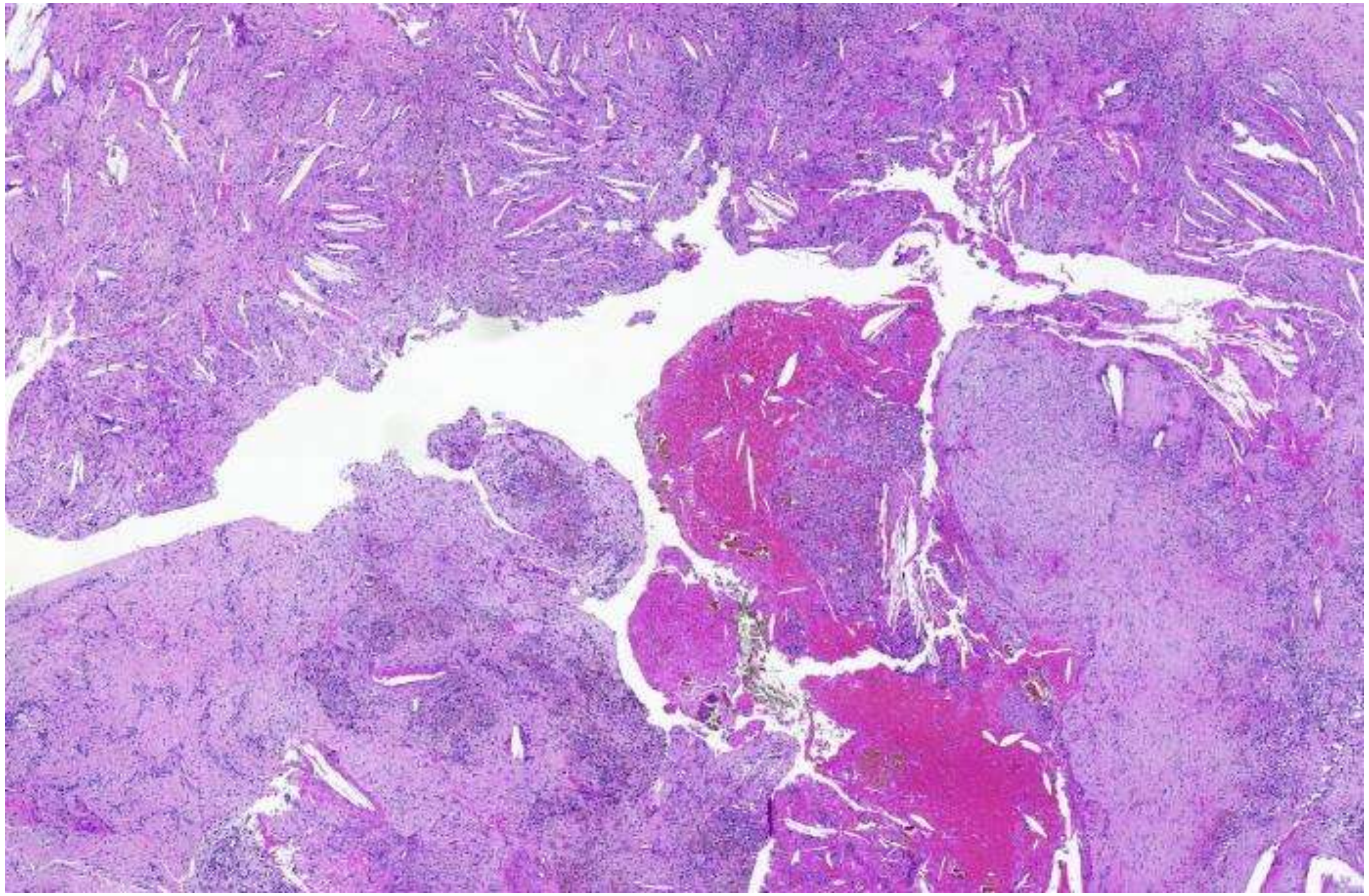

K20 – Calcium (HE x1 & x1.5) x10 & x20

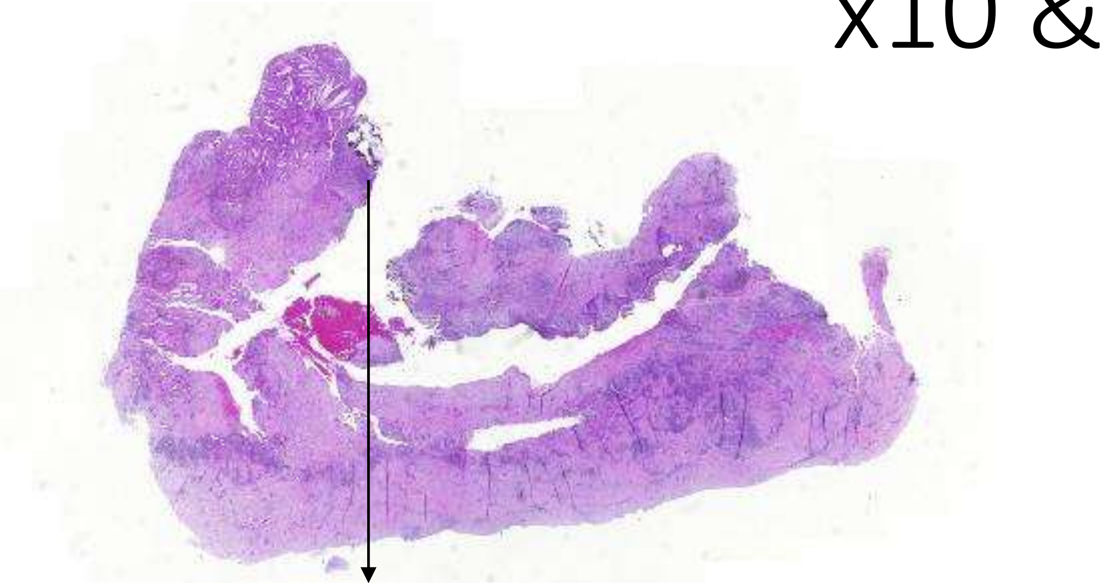

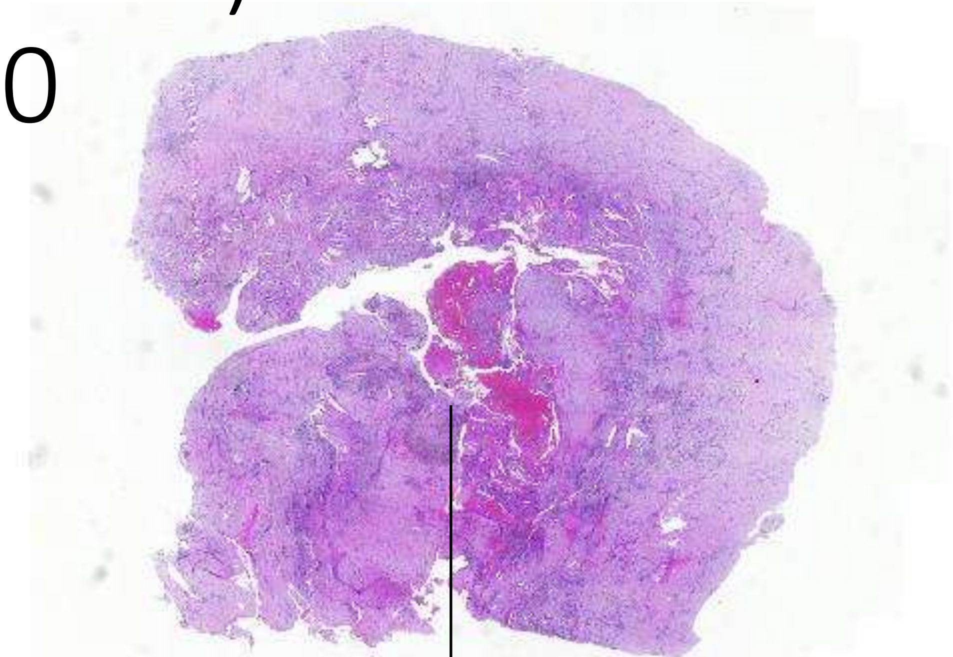

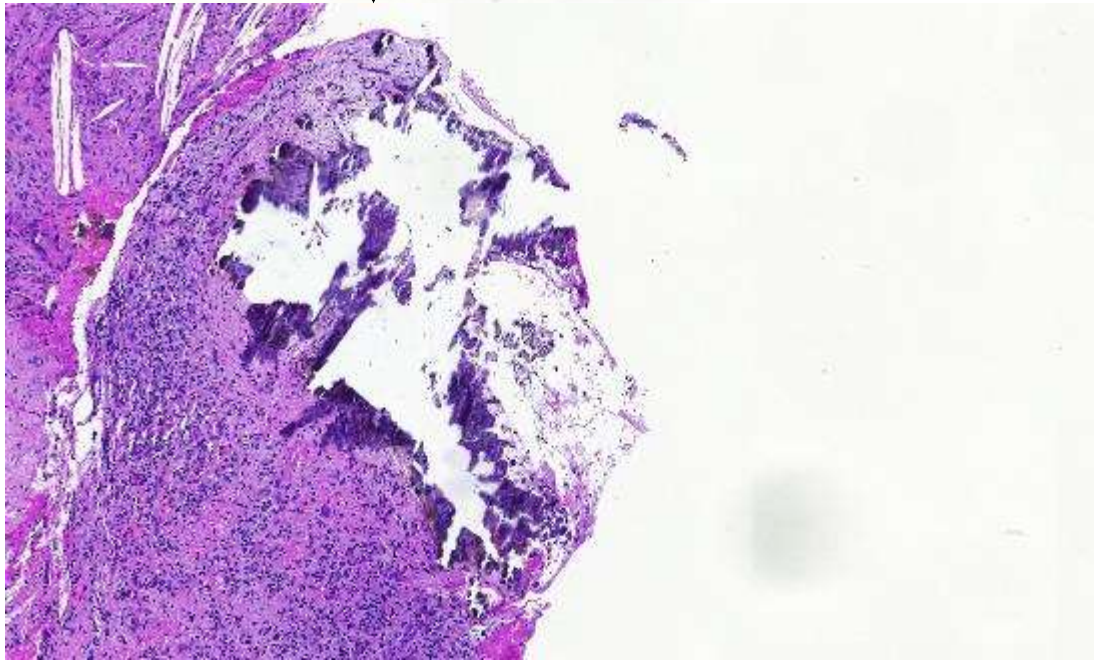

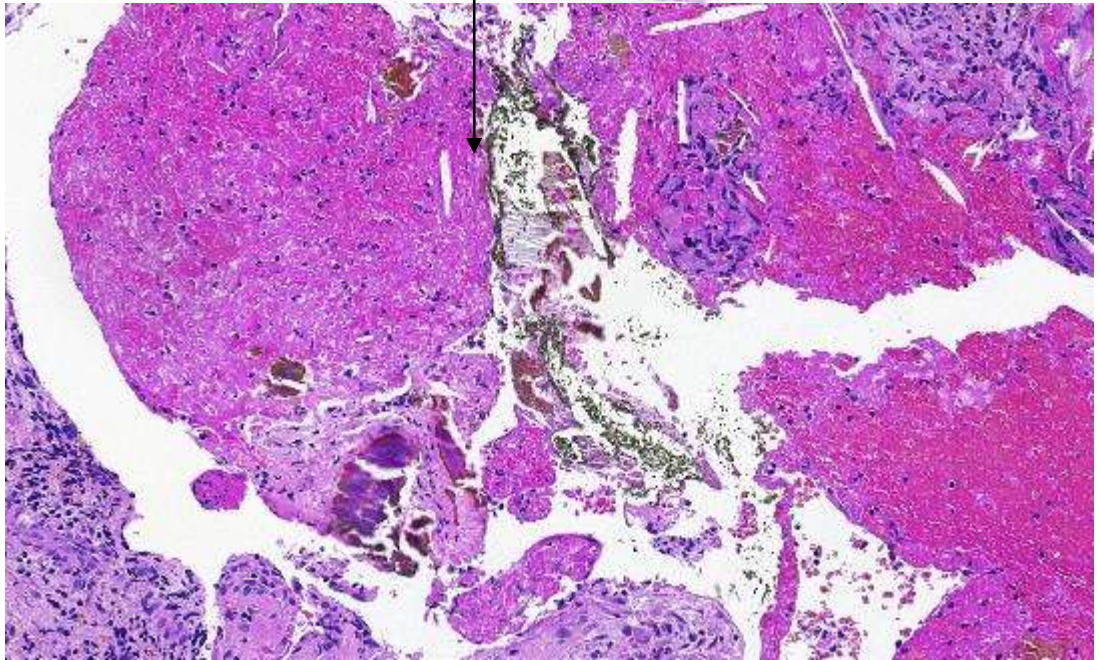

K20 – HE x5 & x20 ghost cells

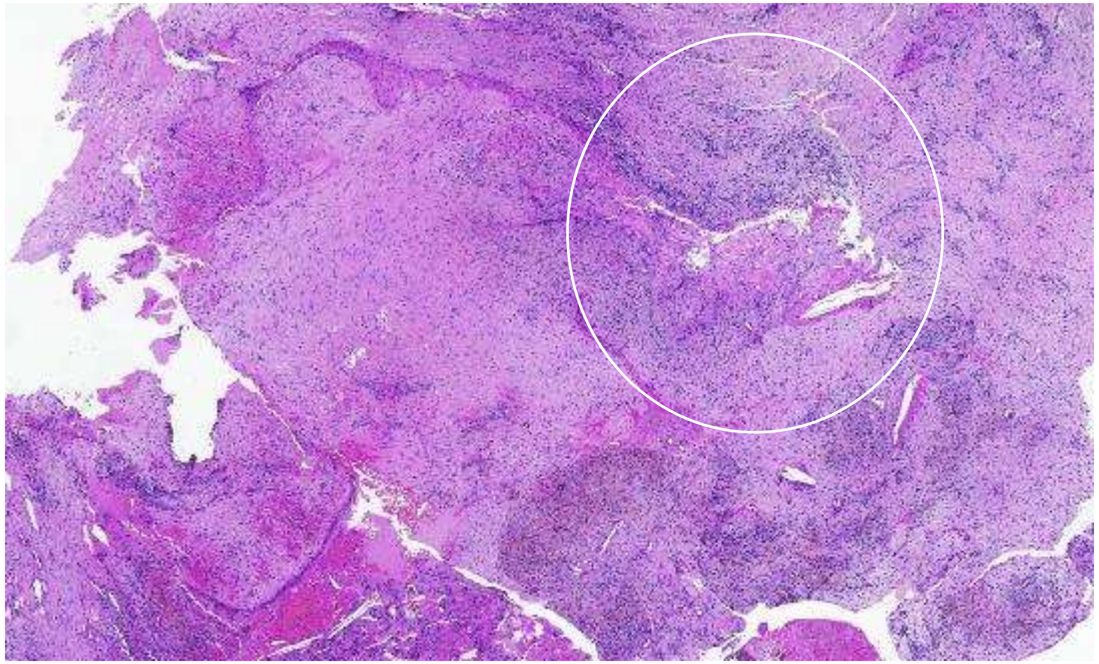

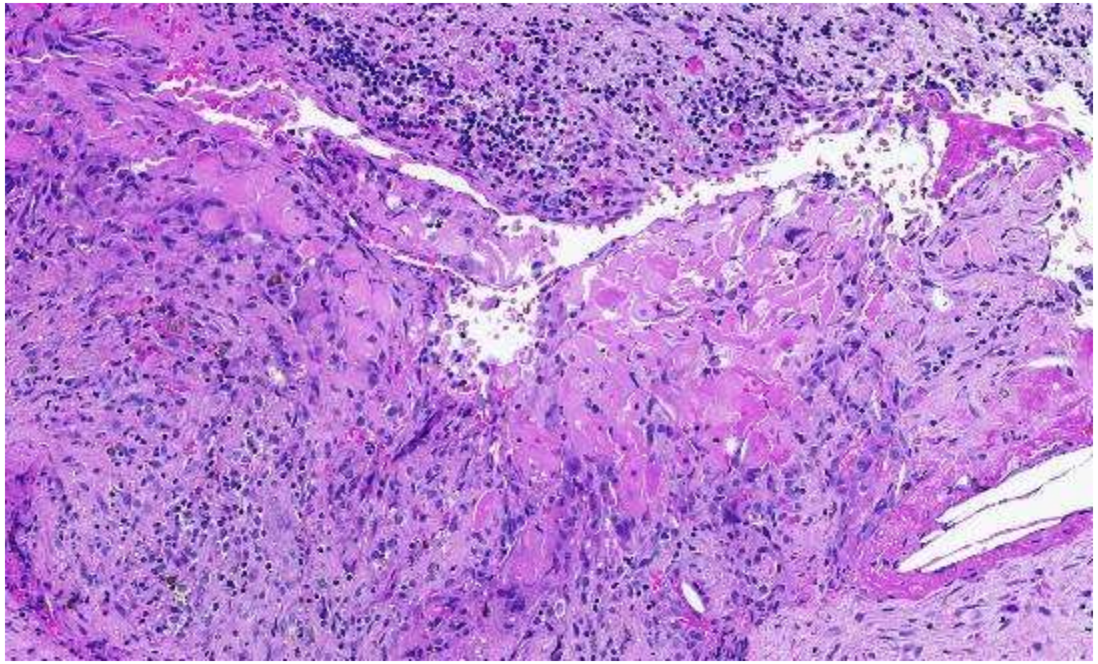

K20 – HE x35 ghost-like cells

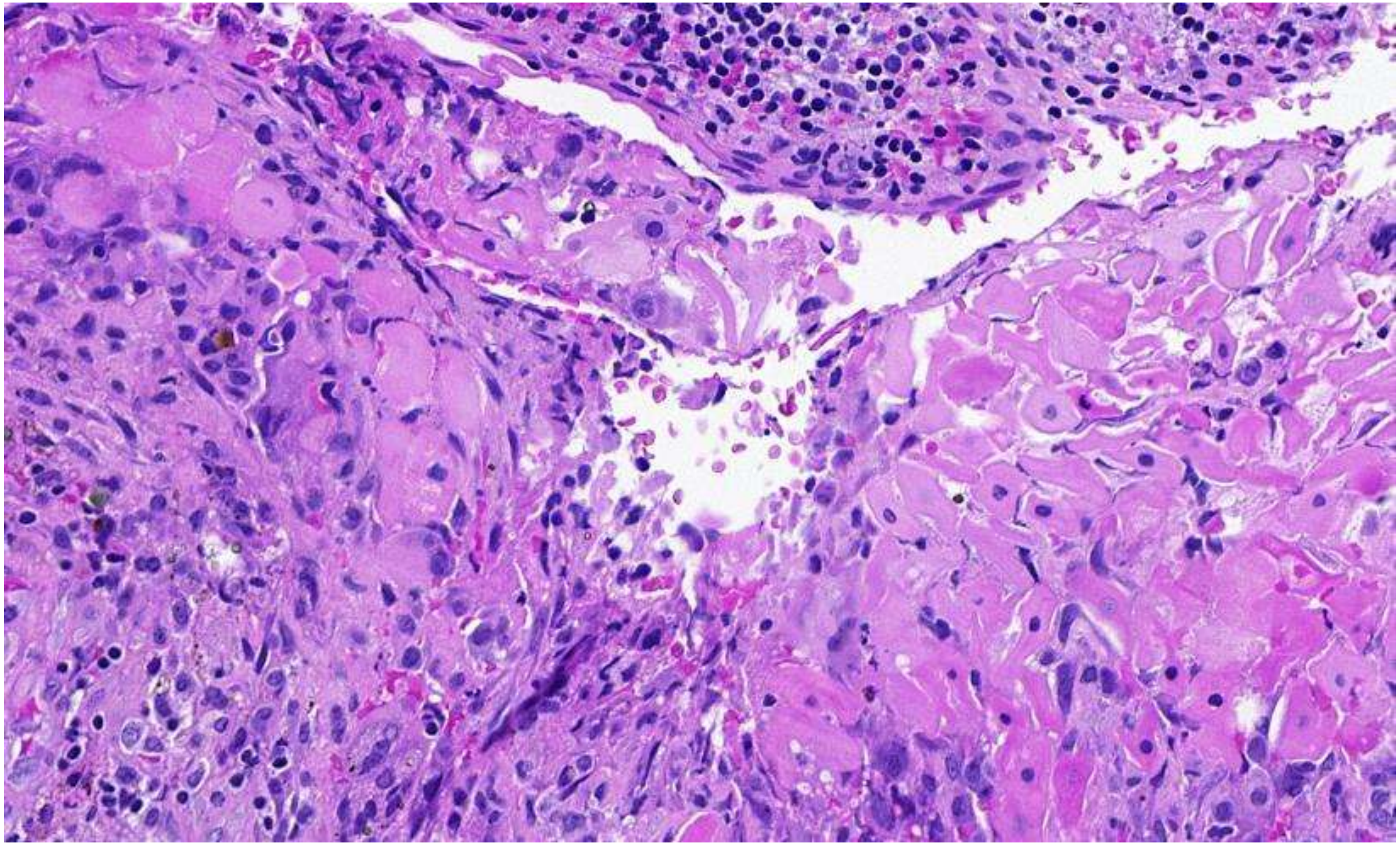

K20 – HE, CK17, bcl2 (x10)

Focal transepithelial CK17 staining (↑)

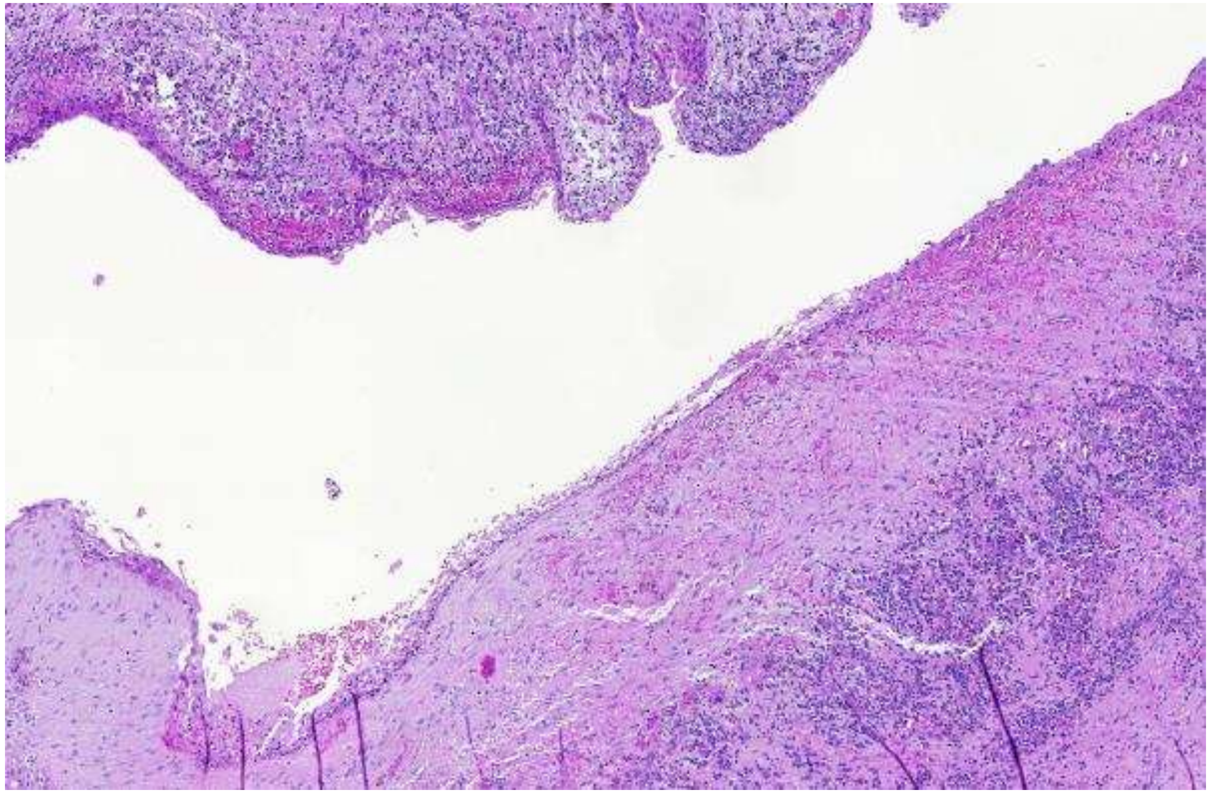

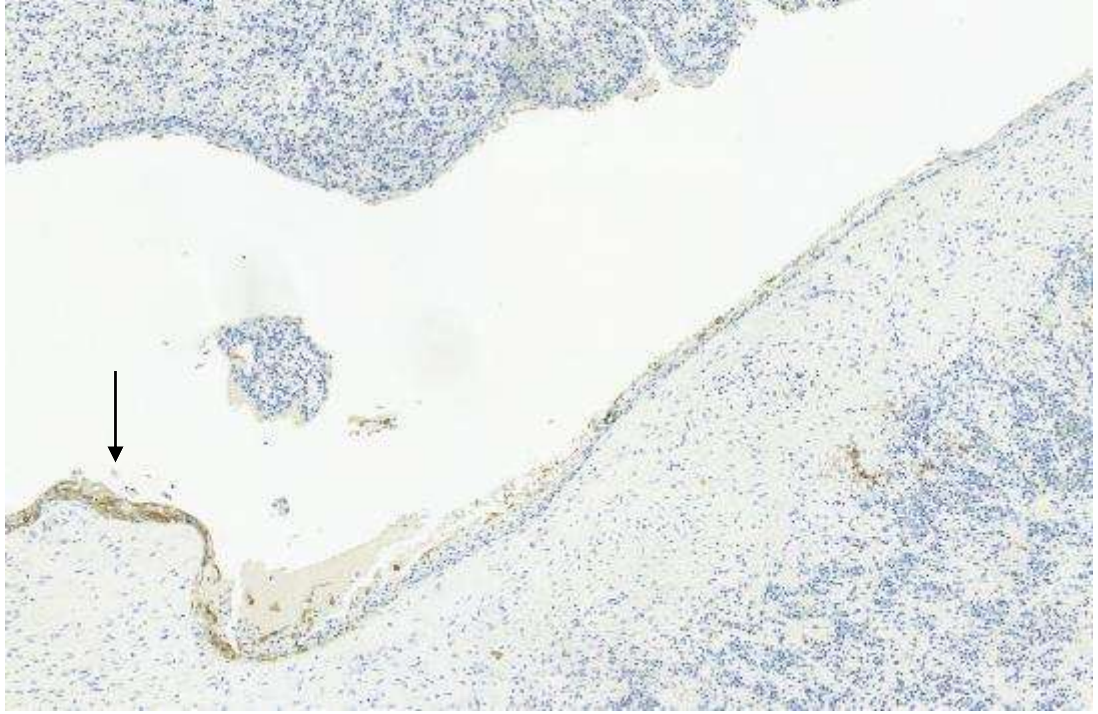

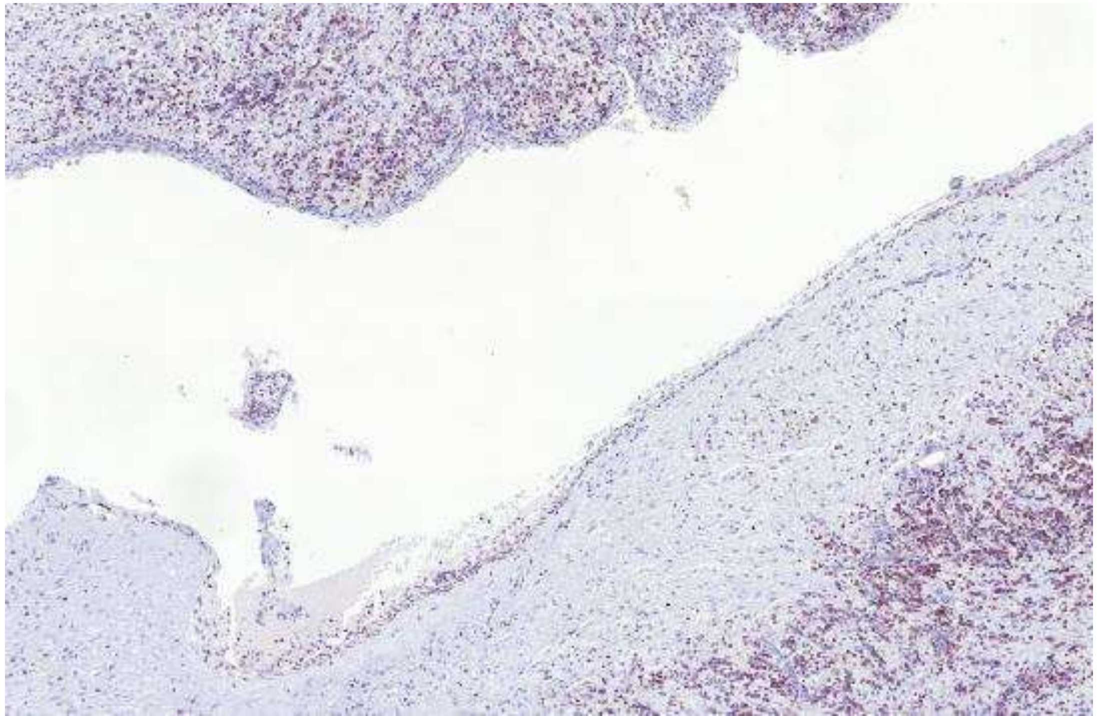

K20 – HE, CK17, bcl2 (x16)

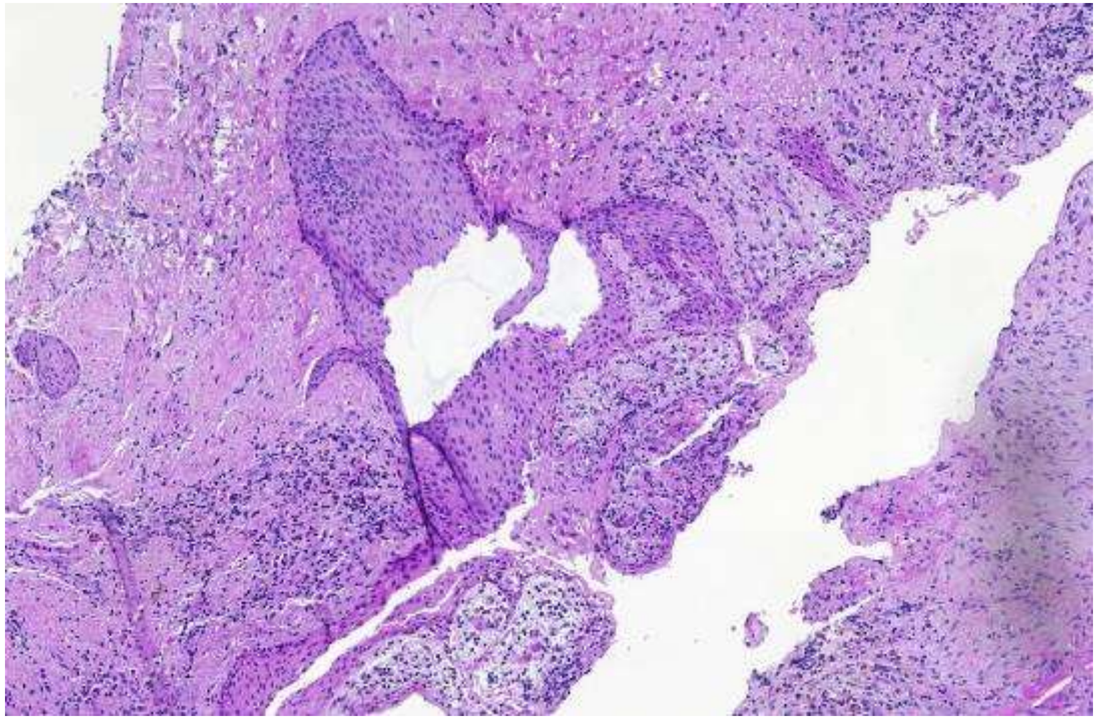

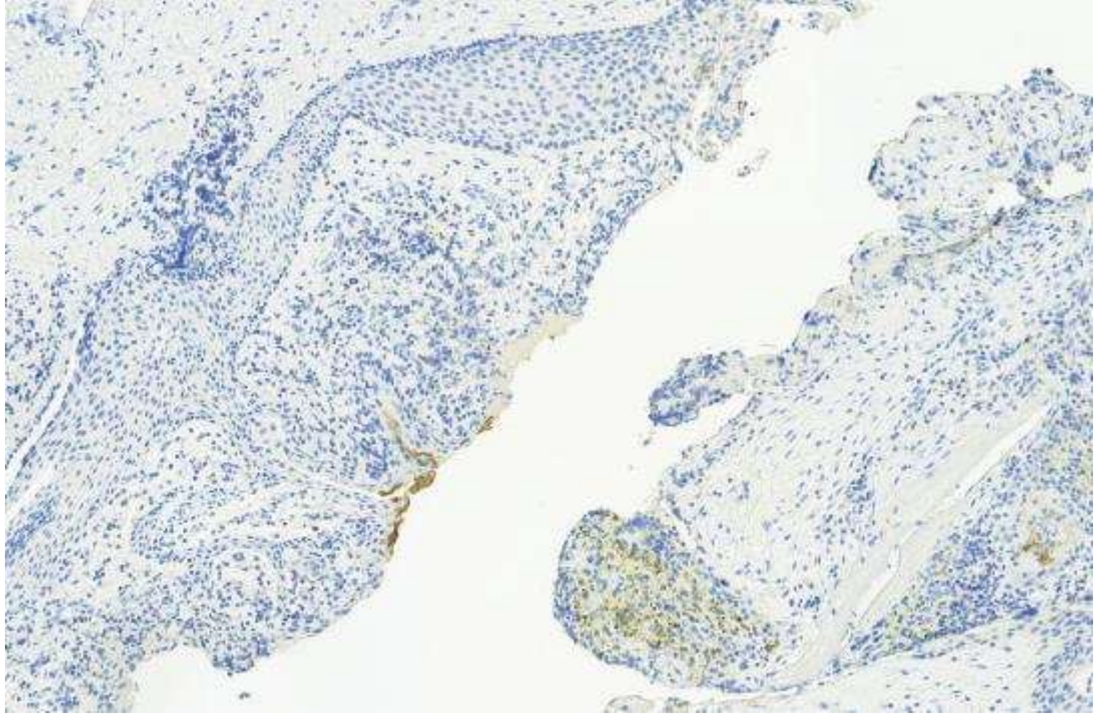

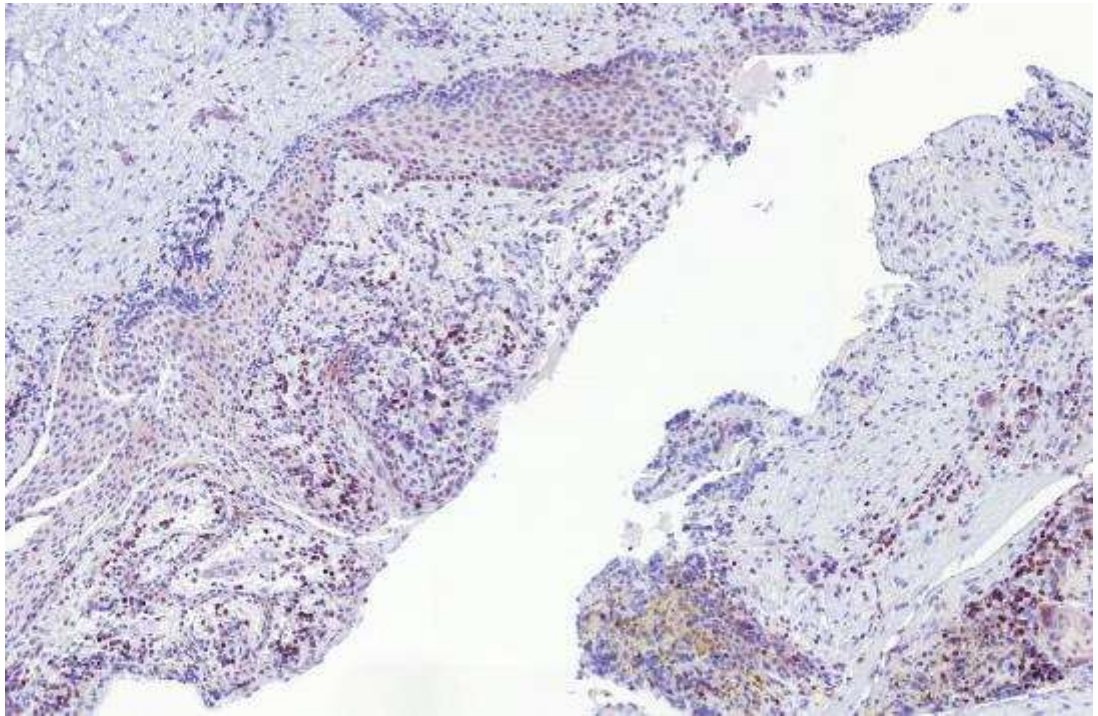

K20 – HE, CK17, bcl2 (x40)

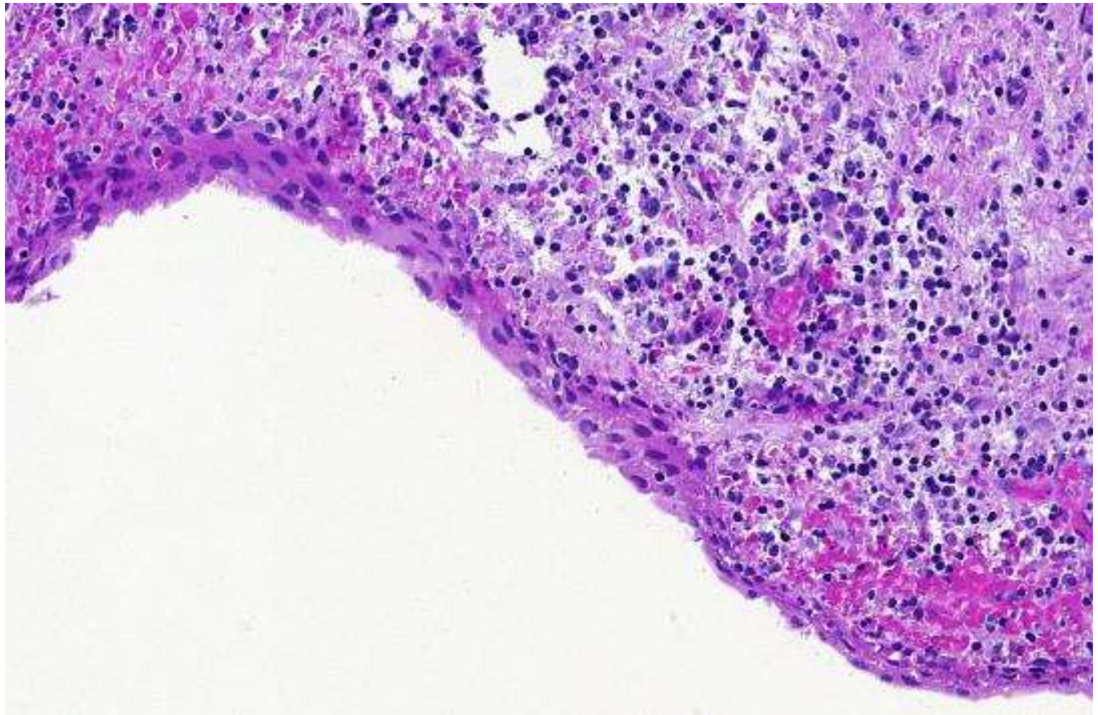

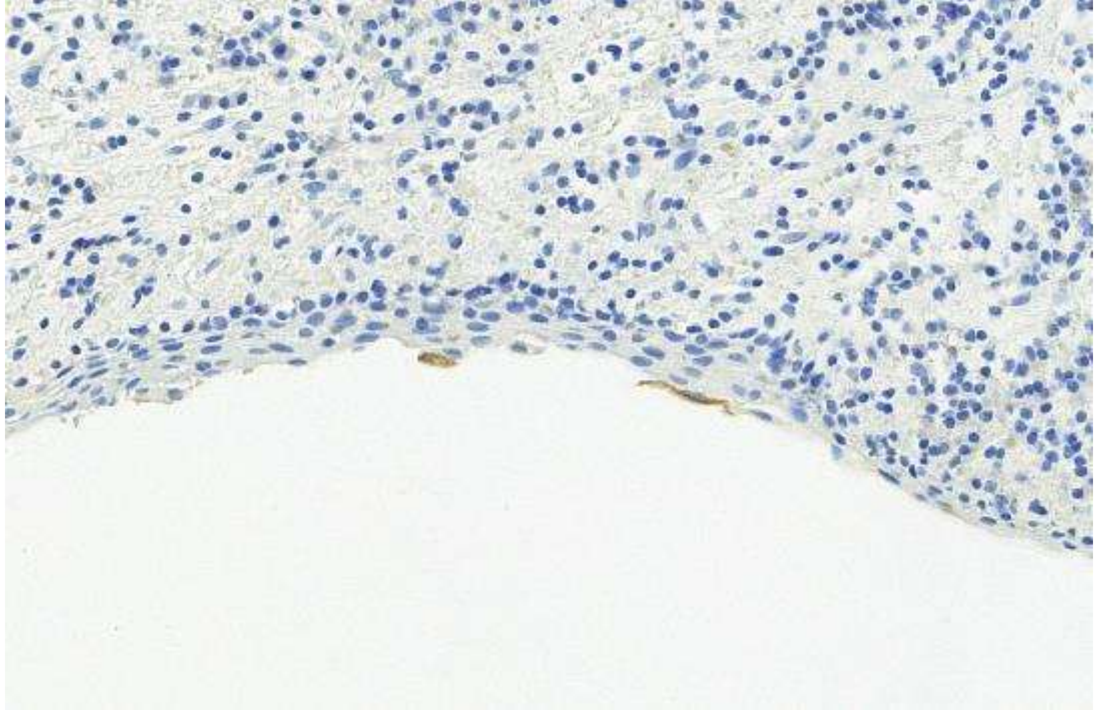

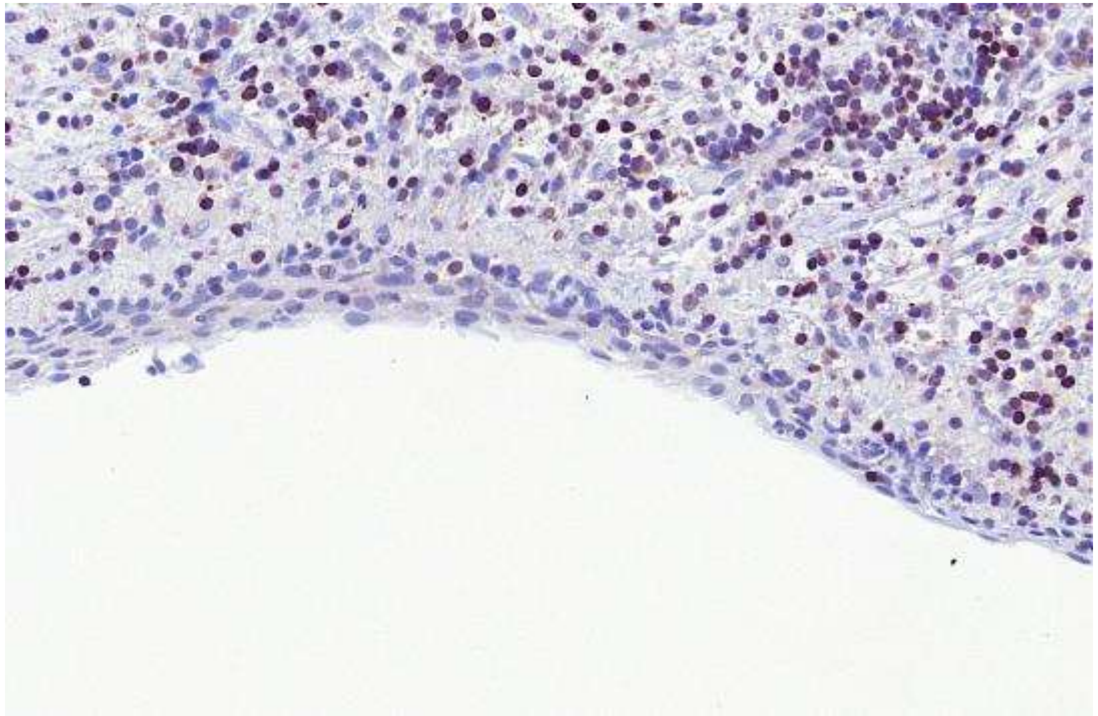

K20 – Conclusion

- H&E: relatively thick (focally thin) squamous epithelium, no parakeratosis, no wavy surface, no basal palisading, even to uneven epithelial-subepithelial contours
- Moderate chronic inflammation of the cyst wall with cholesterol clefts and granuloma, filling material debris
- Bcl2: negative
- CK17: focal superficial staining with small area of transepithelial or spinous layer staining.
- Histopathology: inflammatory (radicular) cyst
- Combined clinical and pathological opinion: (most likely to represent) fused radicular cysts

Case K21

- 49-year-old male
- Dental hygiene: normal.
- Radiology: Periapical 2-cm lesion (cyst) corresponding to teeth 11, 12, and 13, protruding towards the maxillary sinus and nasal base.
- Operation: Removal of the cyst with apical resection and retrograde root filling of teeth 11, 12, 13.
- Clinical diagnosis: periapical (inflammatory) cyst

K21 – OPT

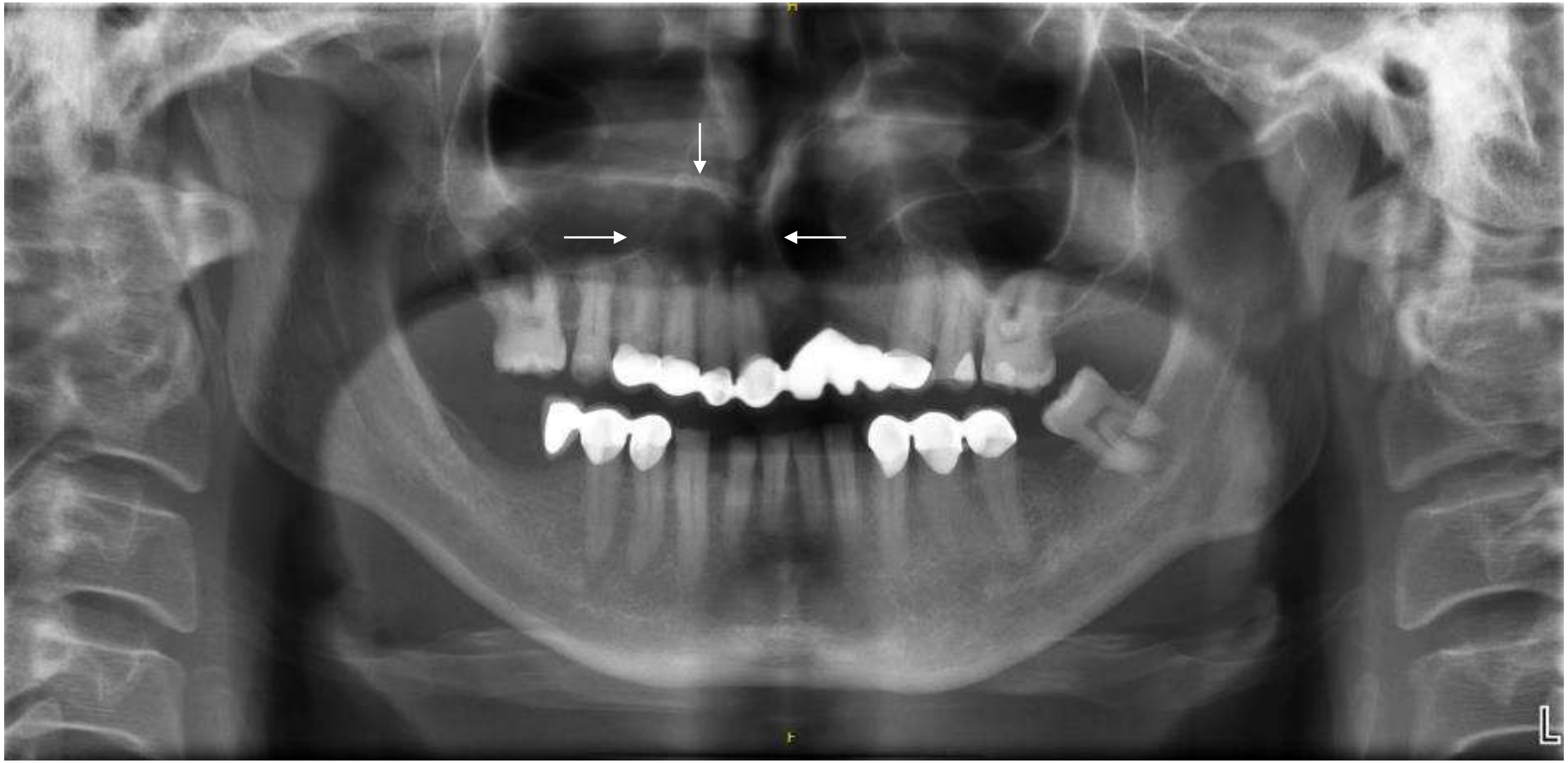

K21 – OPT magnified

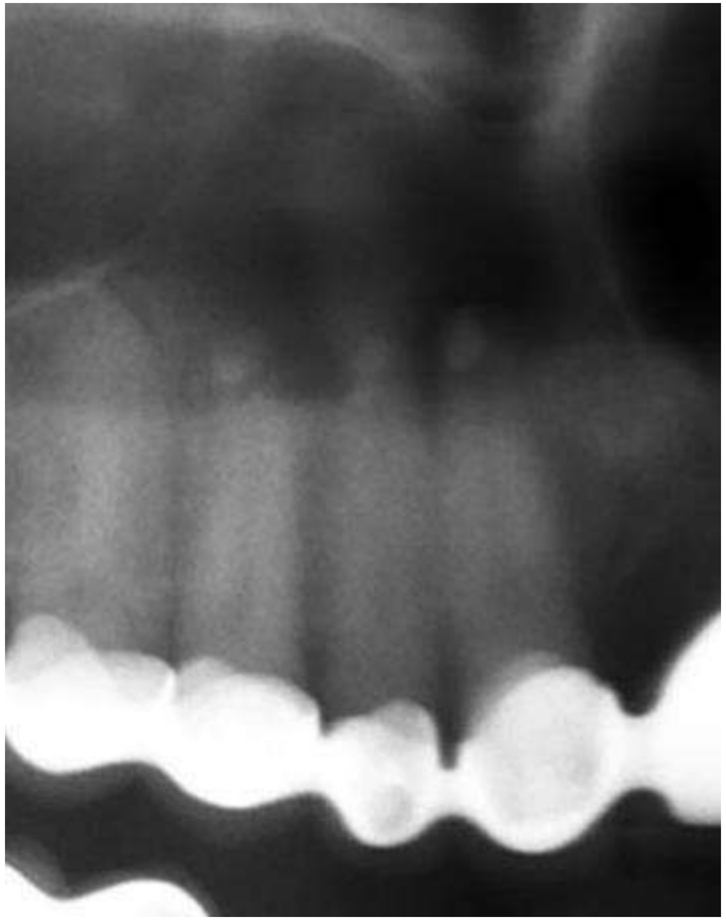

K21 – HE, CK17, bcl2 (x1.2)

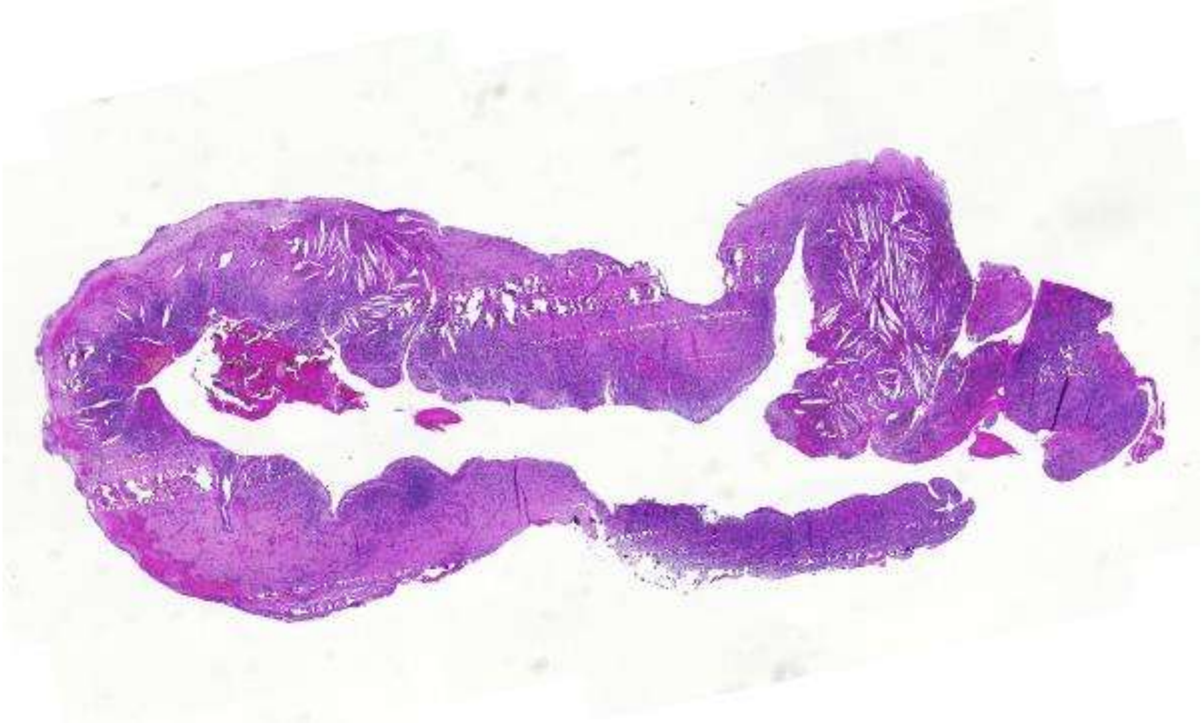

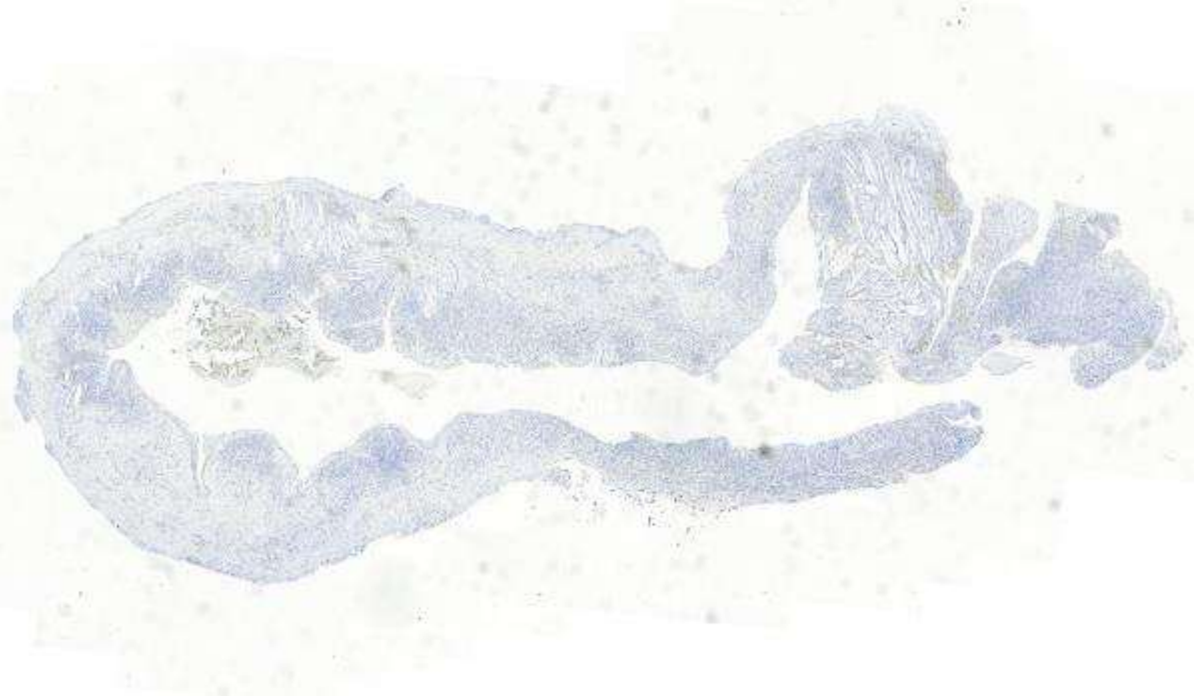

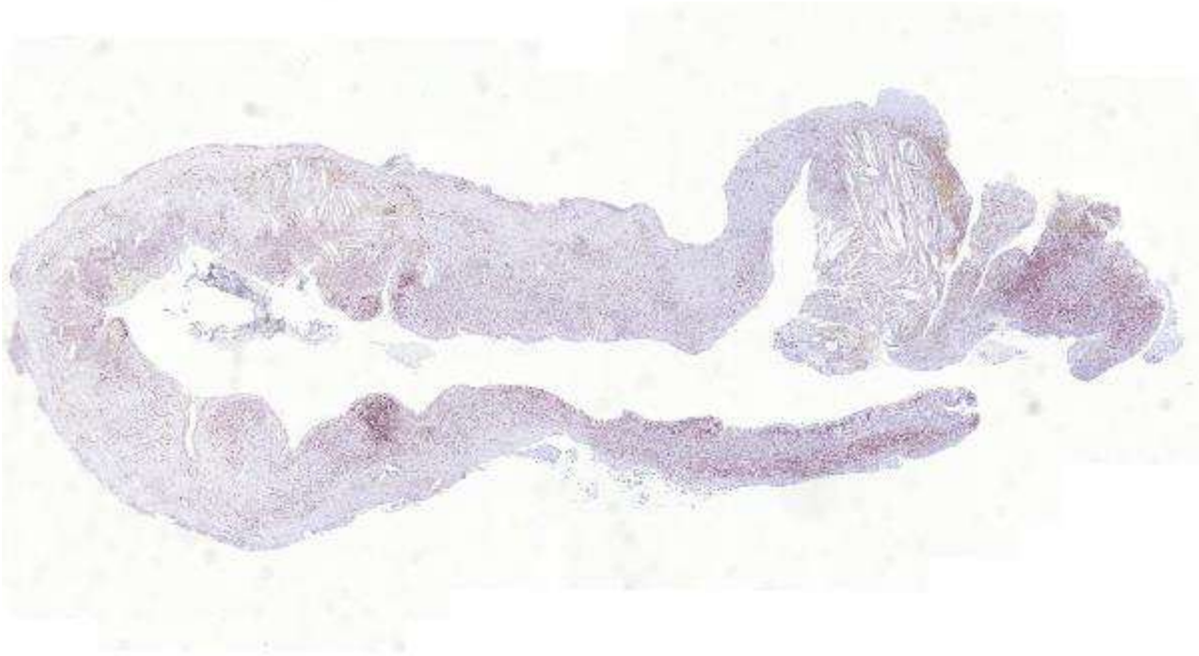

K21 – HE, CK17, bcl2 (x10)

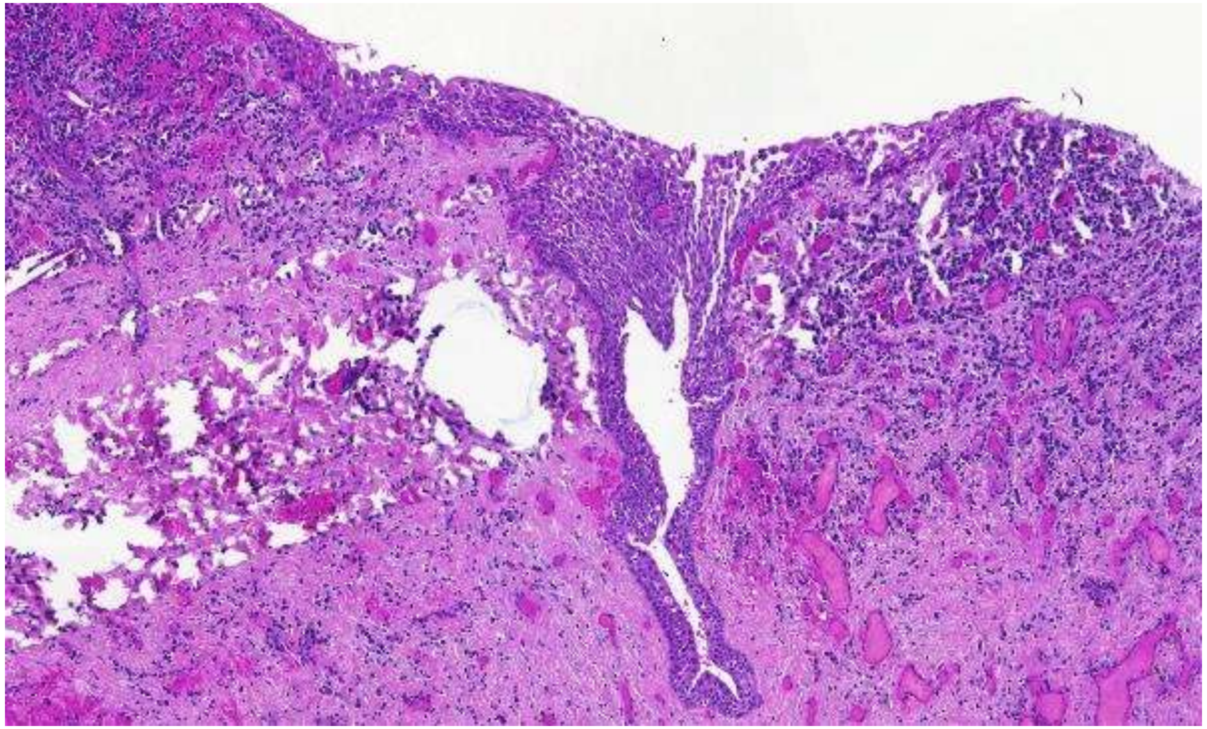

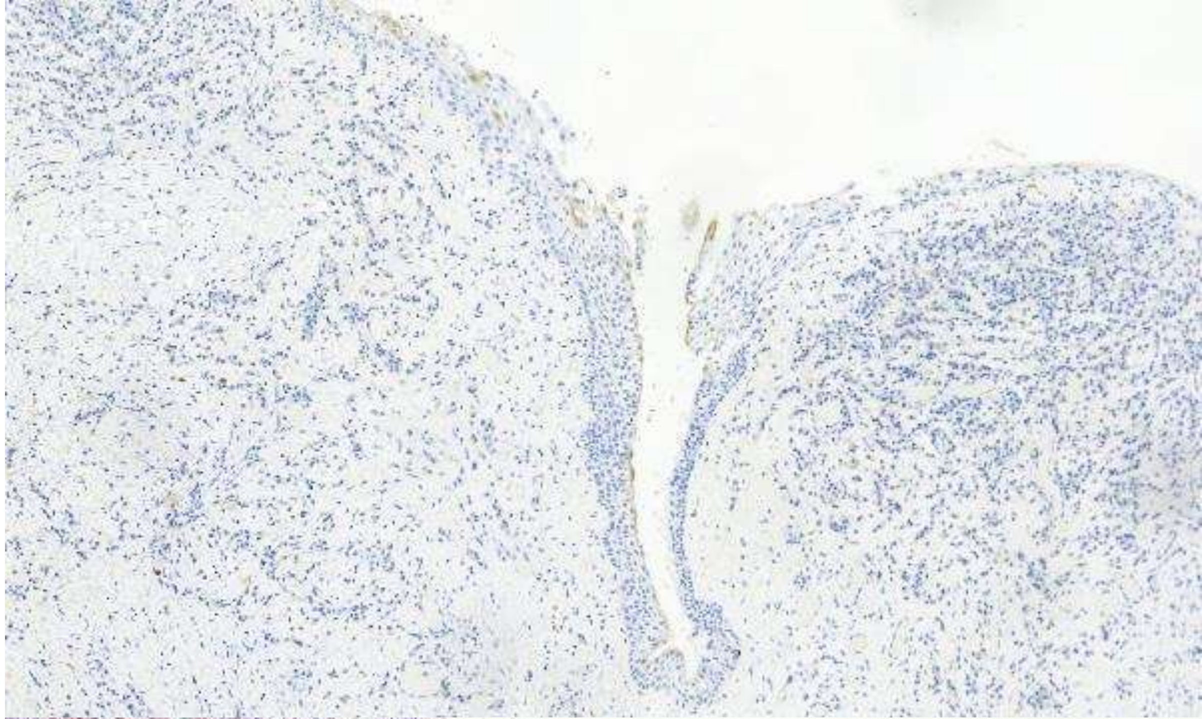

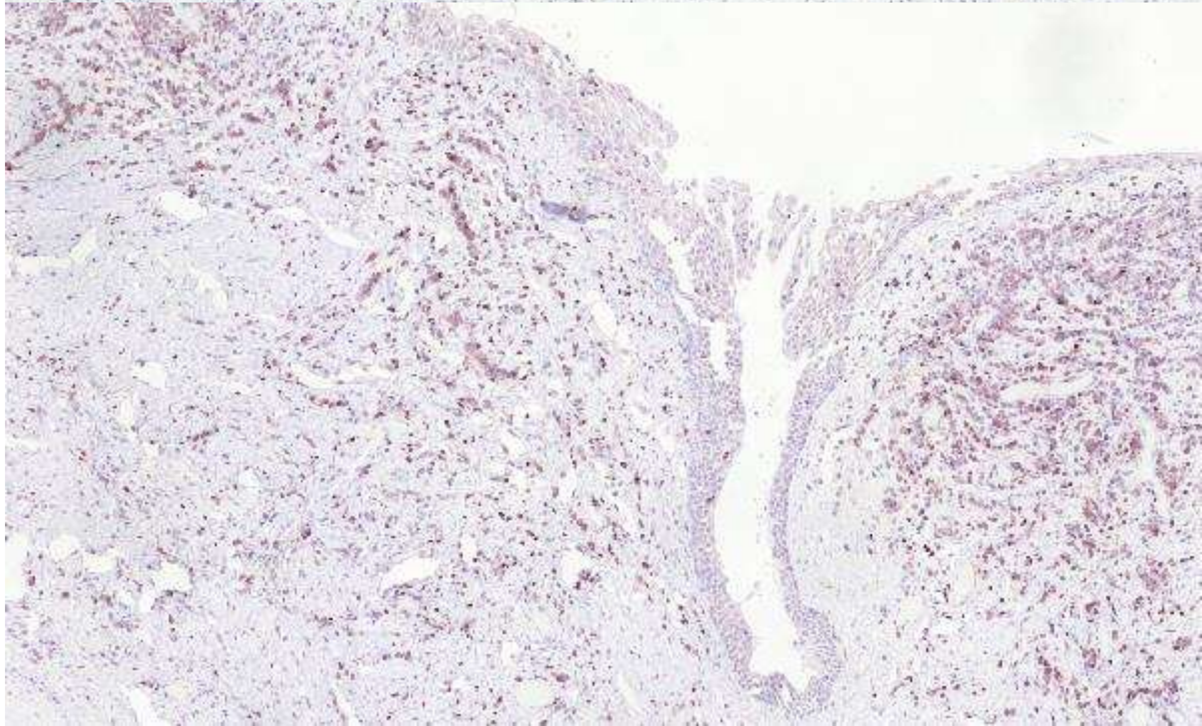

K21 – HE, CK17, bcl2 (x20)

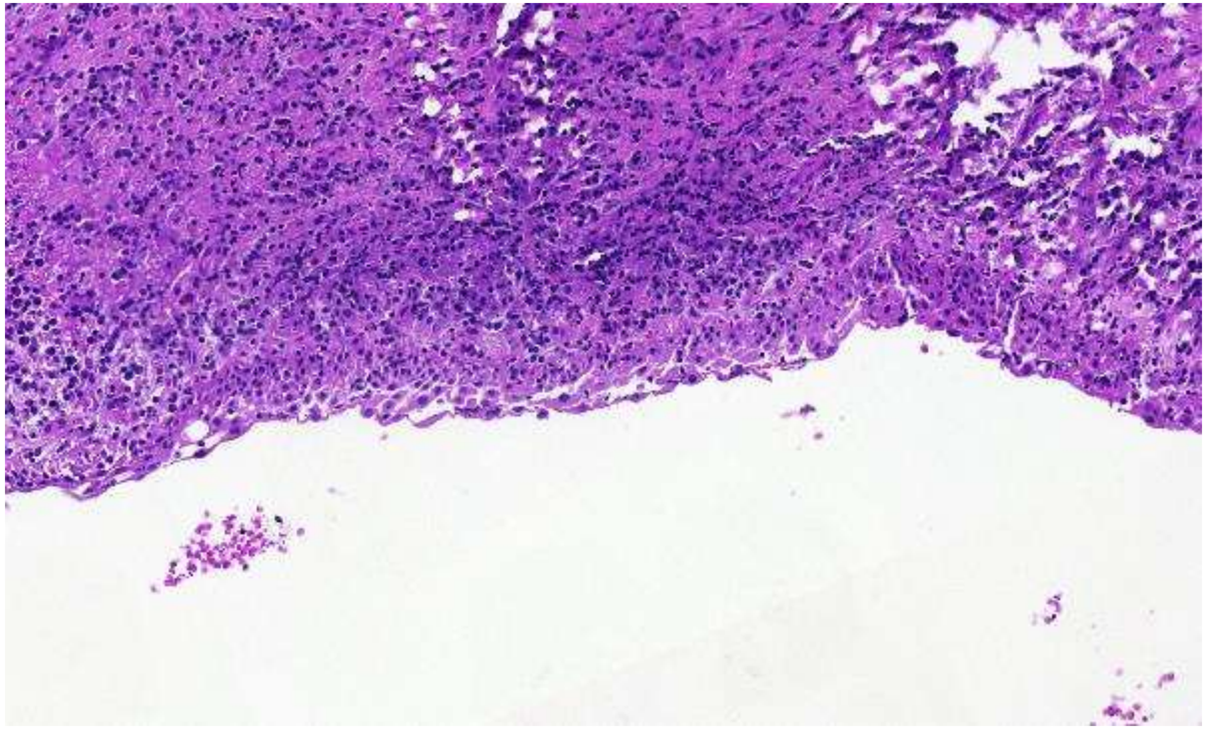

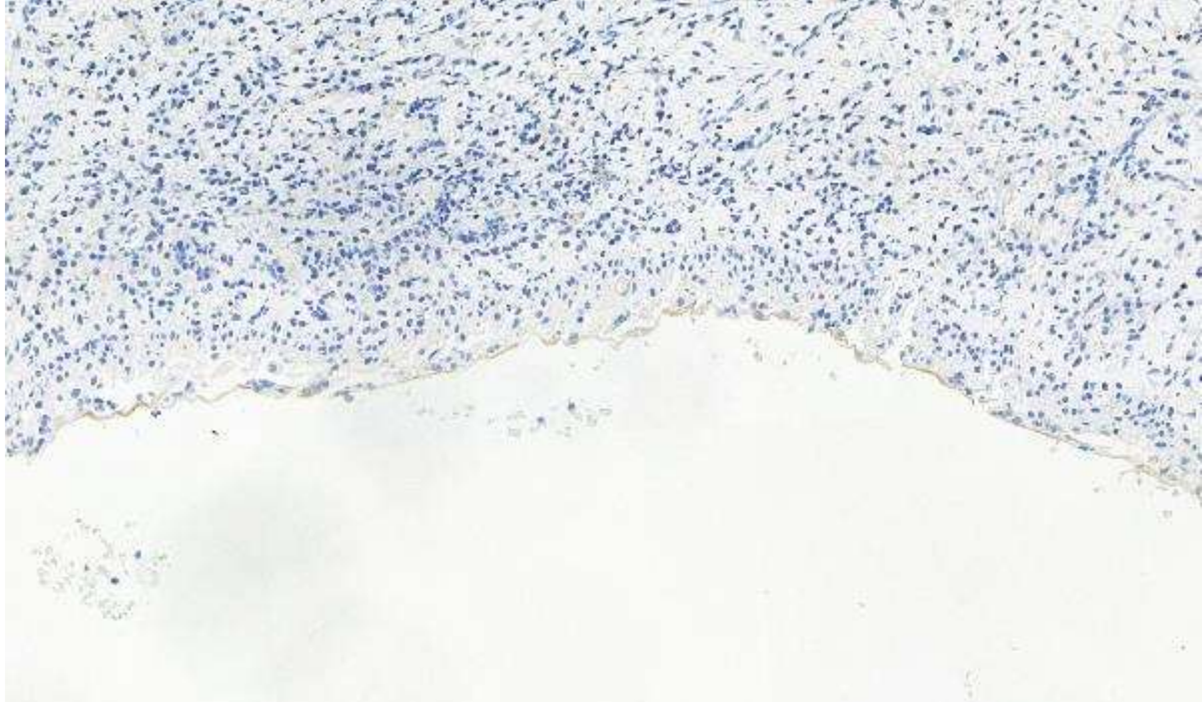

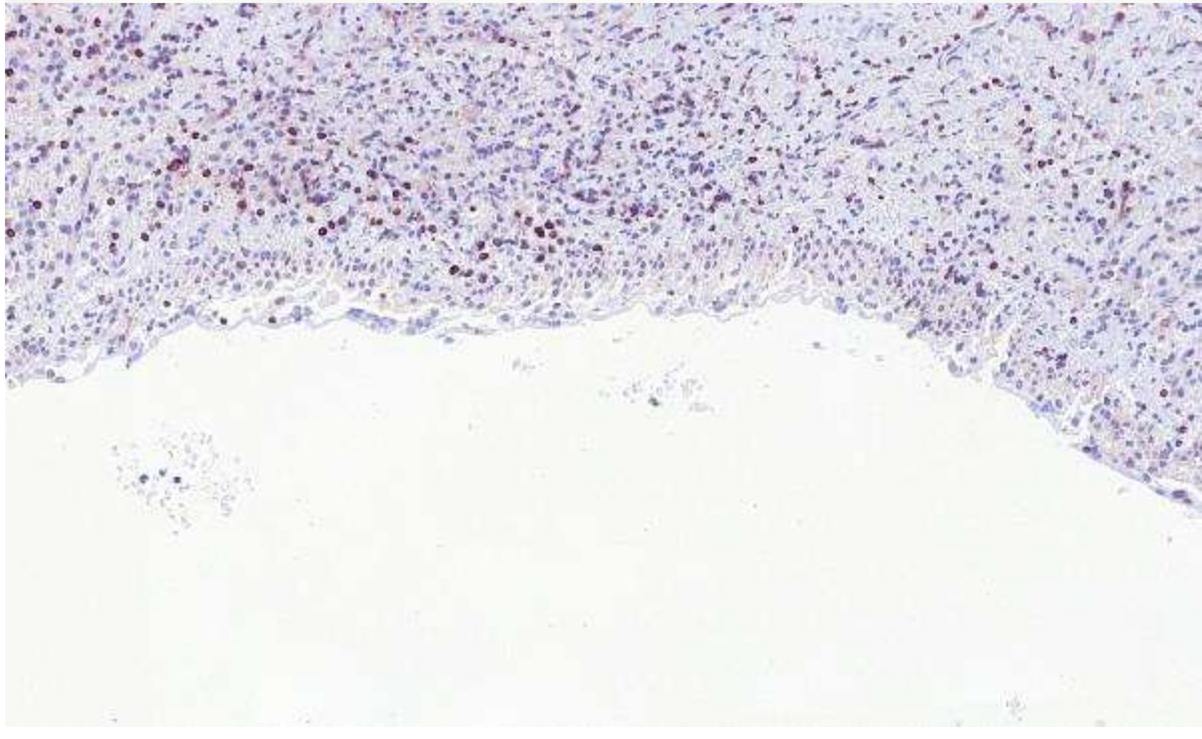

K21 – Conclusion

- H&E: relatively thin squamous epithelium, no parakeratosis, wavy surface not assessable, no basal palisading, even to uneven epithelial-subepithelial contours
- Inflammation (with mixed inflammatory infiltrate) of the cyst wall with cholesterol clefts and granuloma
- Bcl2: negative
- CK17: superficial cells staining or negative
- Histopathology: radicular cyst
- Combined clinical and pathological opinion: radicular cyst

Case K22

- 42-year-old female
- Dental hygiene/status: partly missing, treated teeth
- Radiology: Unilocular radiolucent lesion 1 cm in diameter at the apex of tooth 11 (with root filling)
- Operation: Cystectomy
- Clinical diagnosis: „maxillary cyst” (NOS)

K22 – OPT

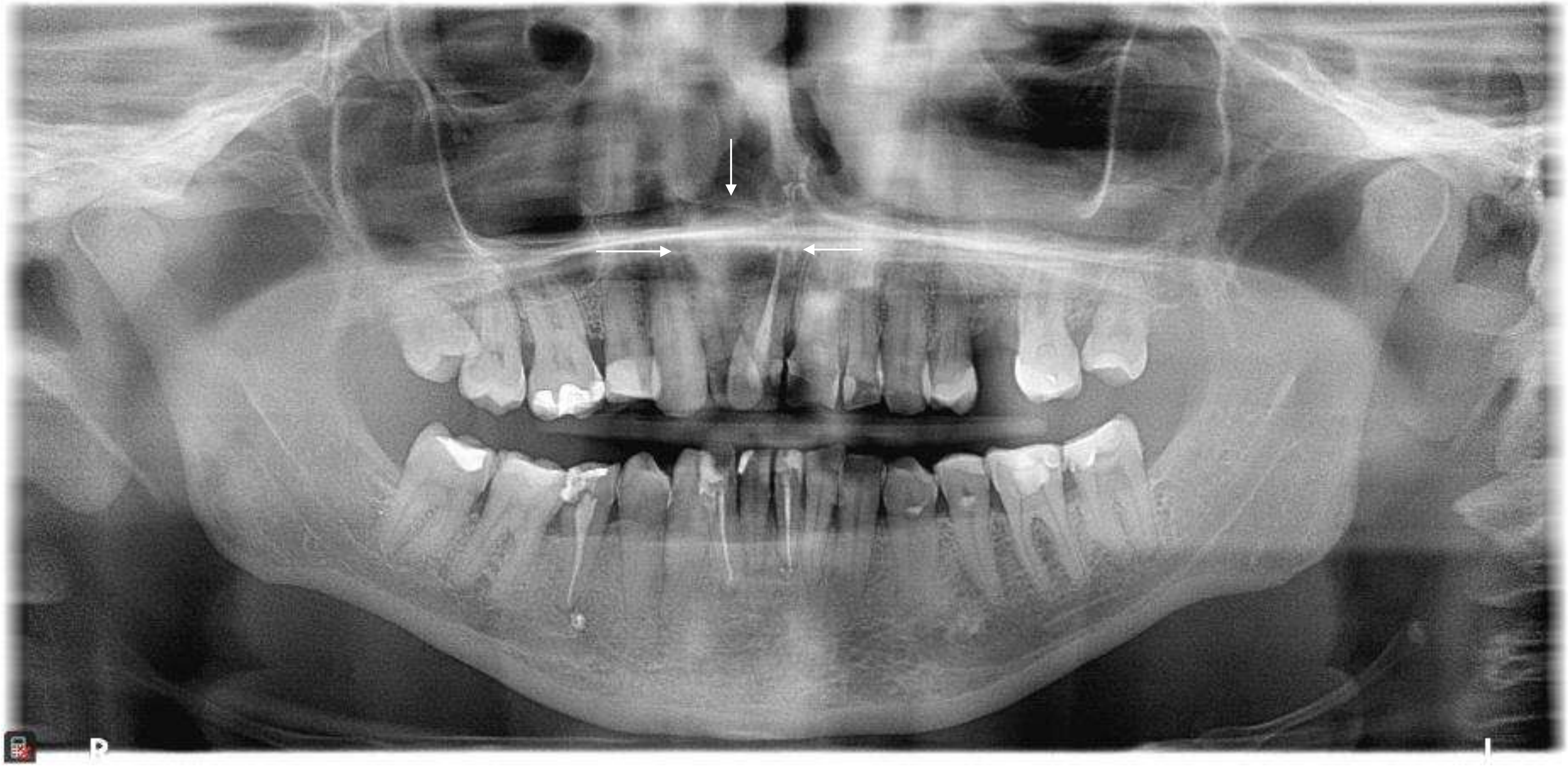

K22 – OPT detail magnified

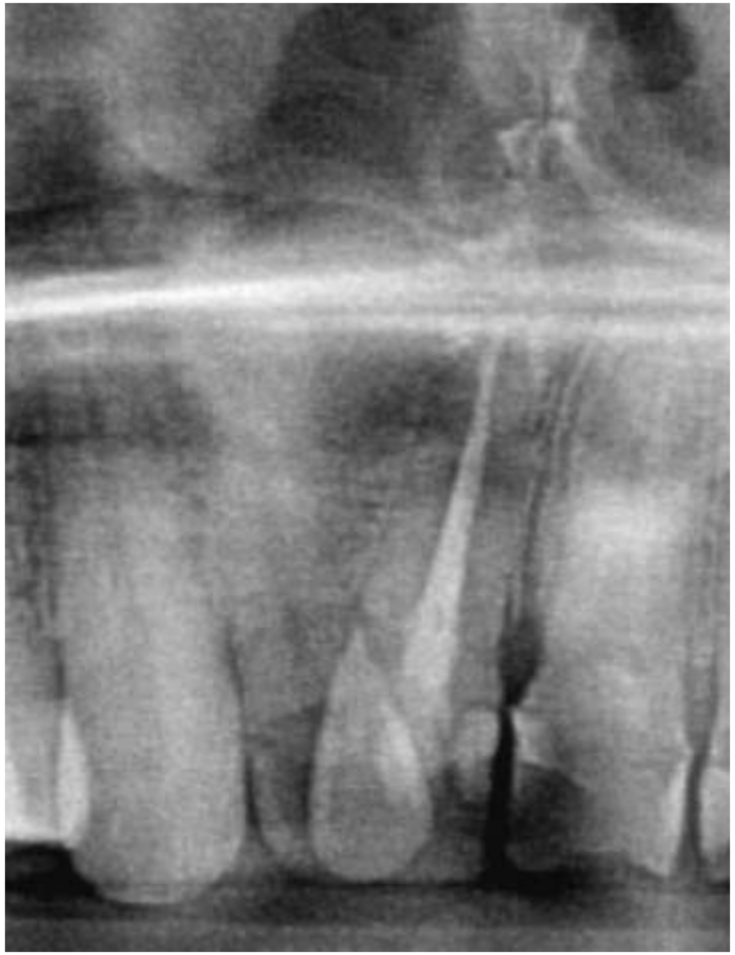

K22 – HE, CK17, bcl2 (x1)

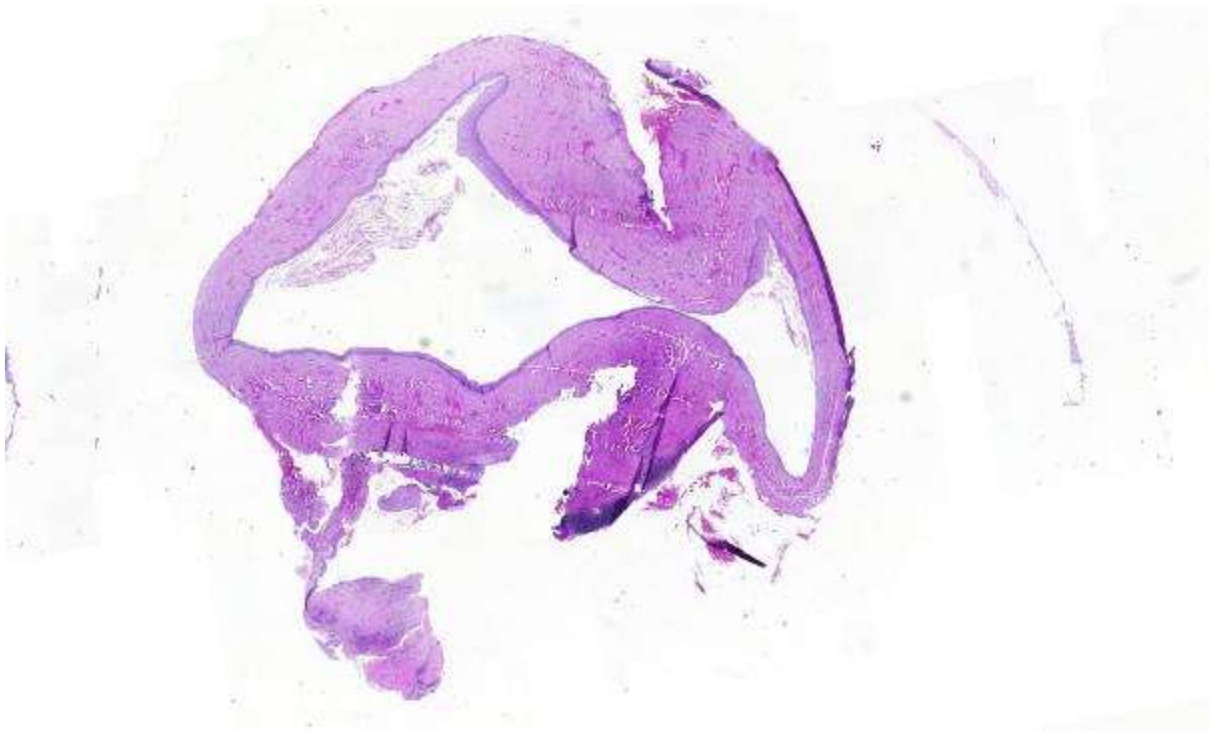

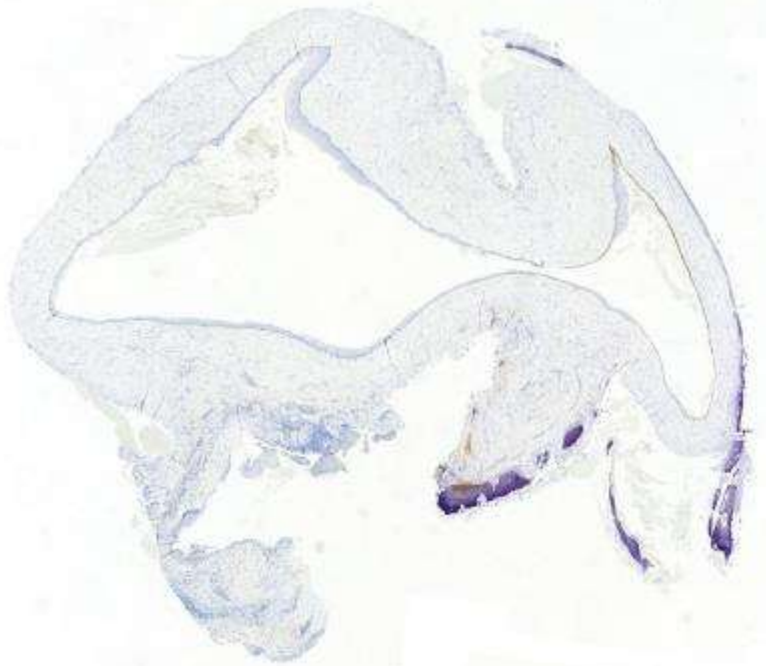

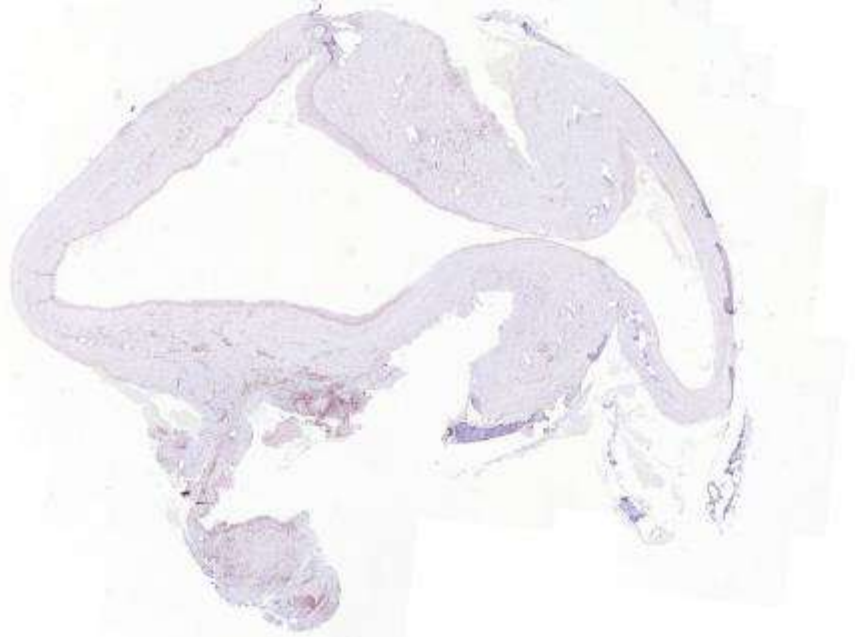

K22 – HE, CK17, bcl2 (x1.2)

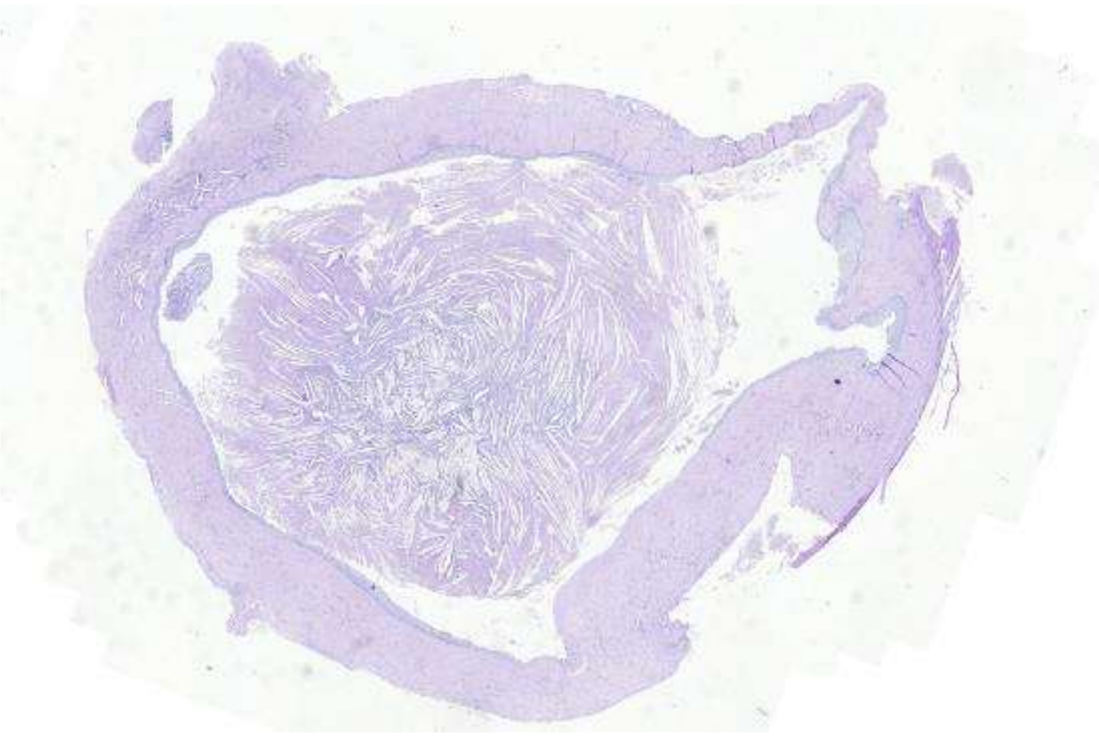

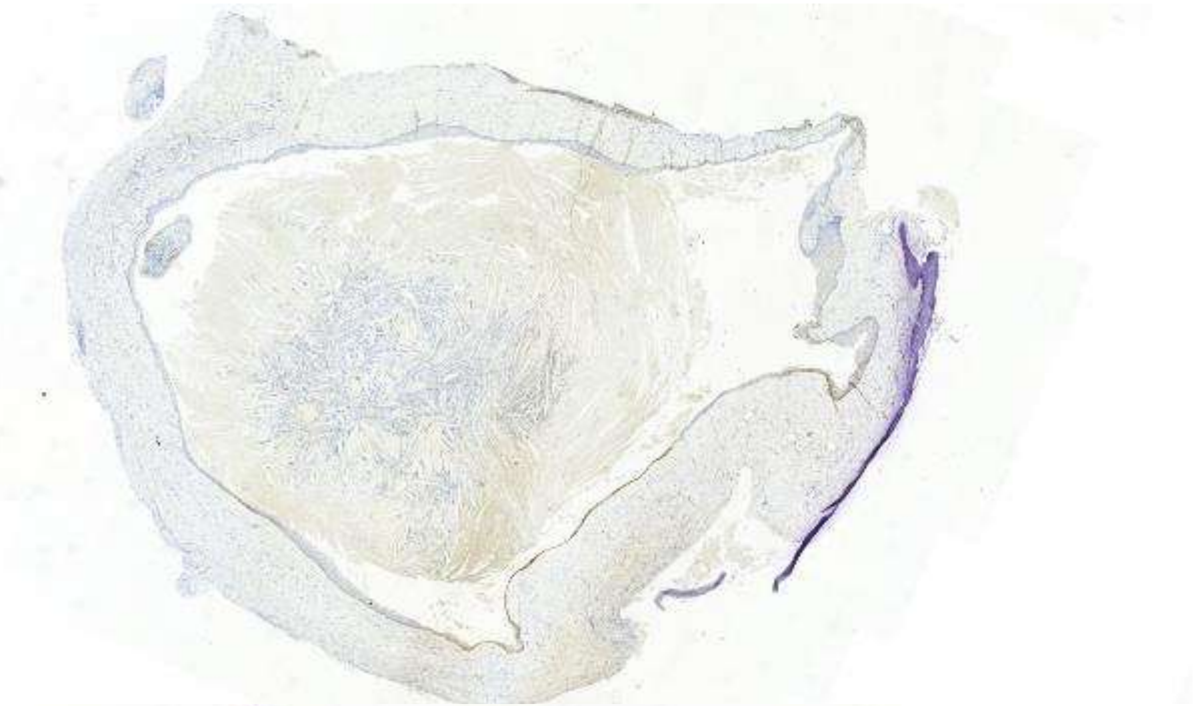

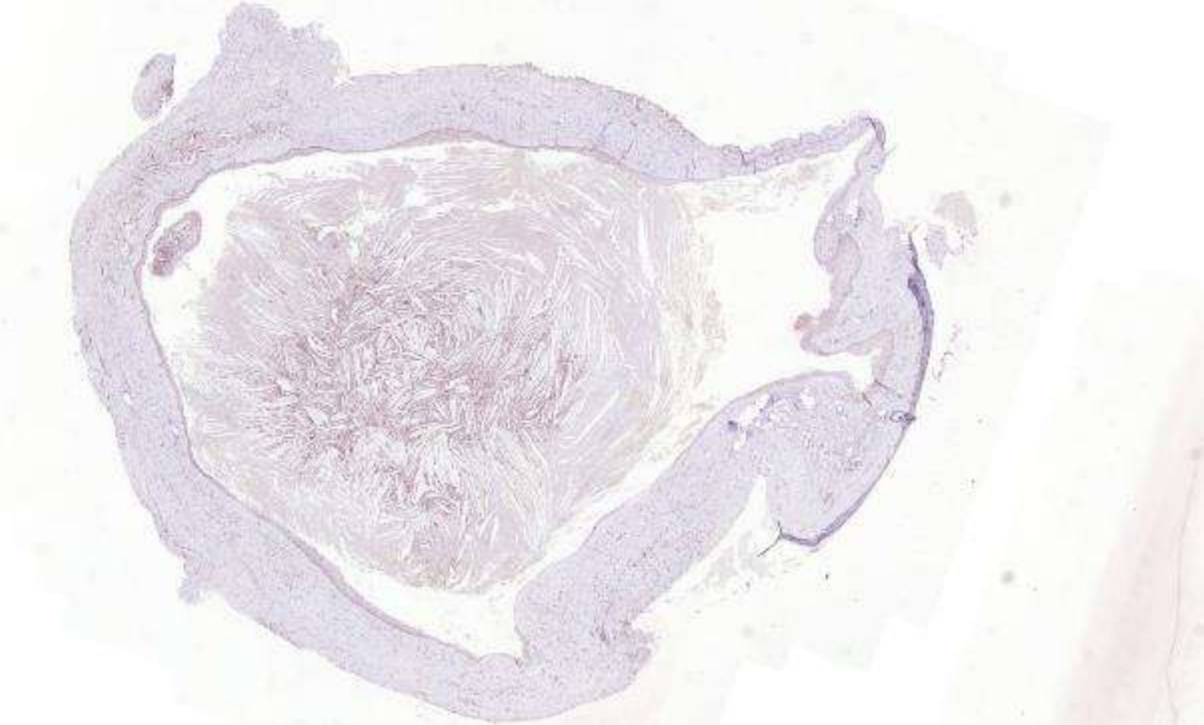

K22 – CK17 (x10) negative(*), transepithelial (↑), basal(↑↑)

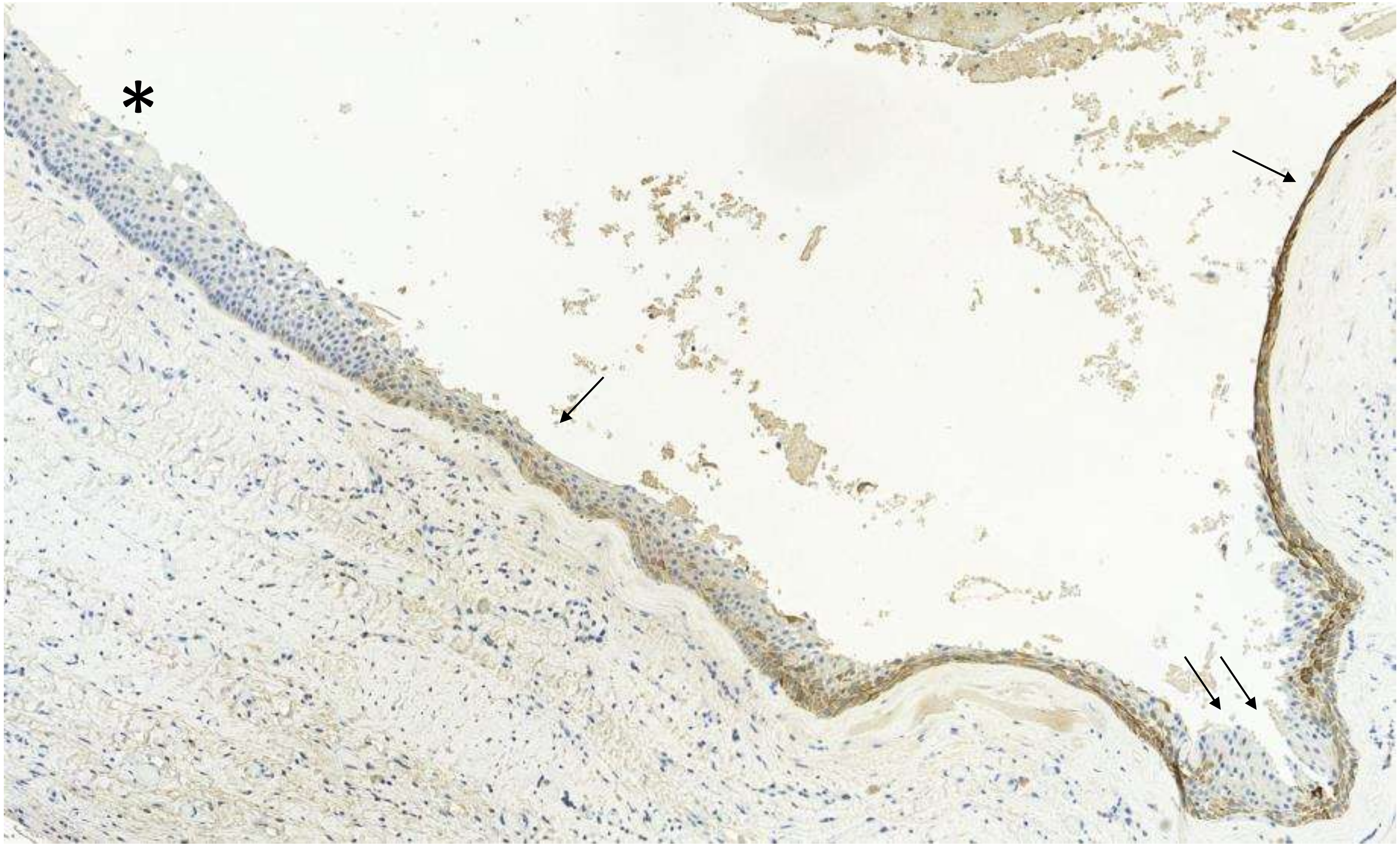

K22 – HE, bcl2 corresponding to previous CK17 (x10)

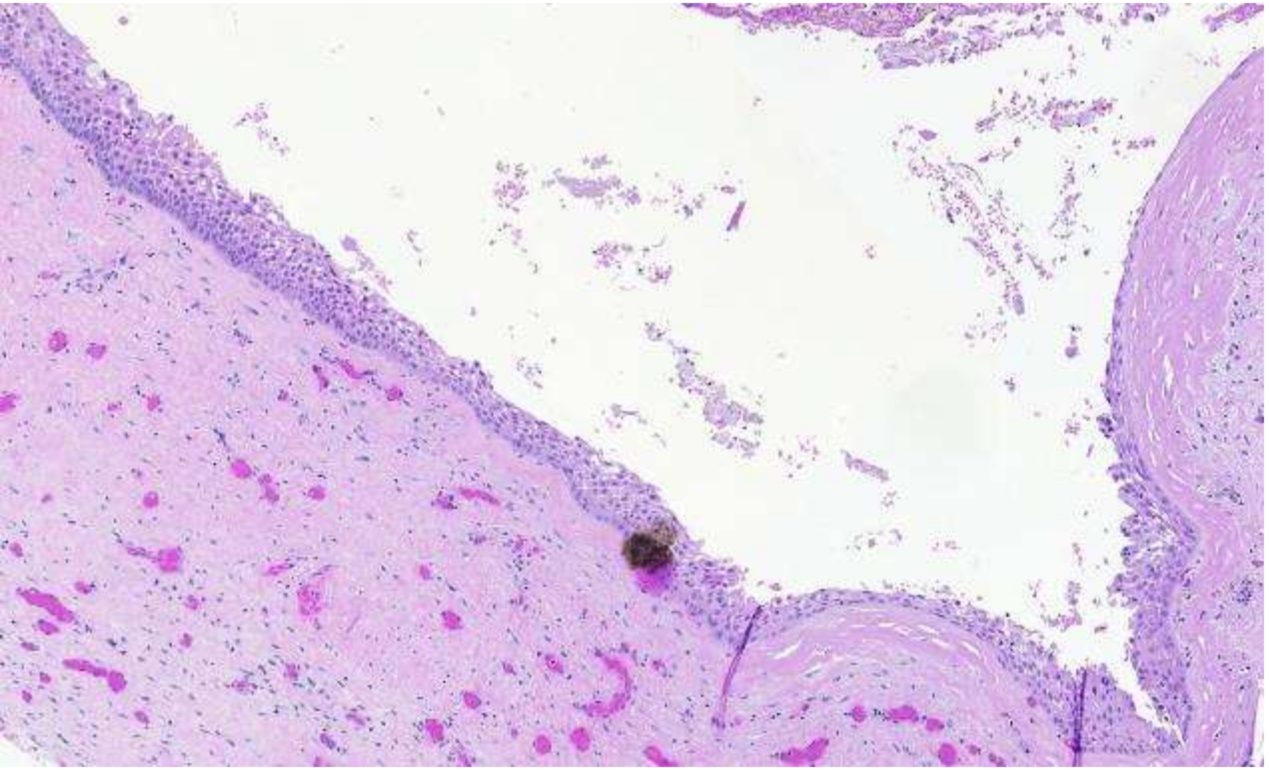

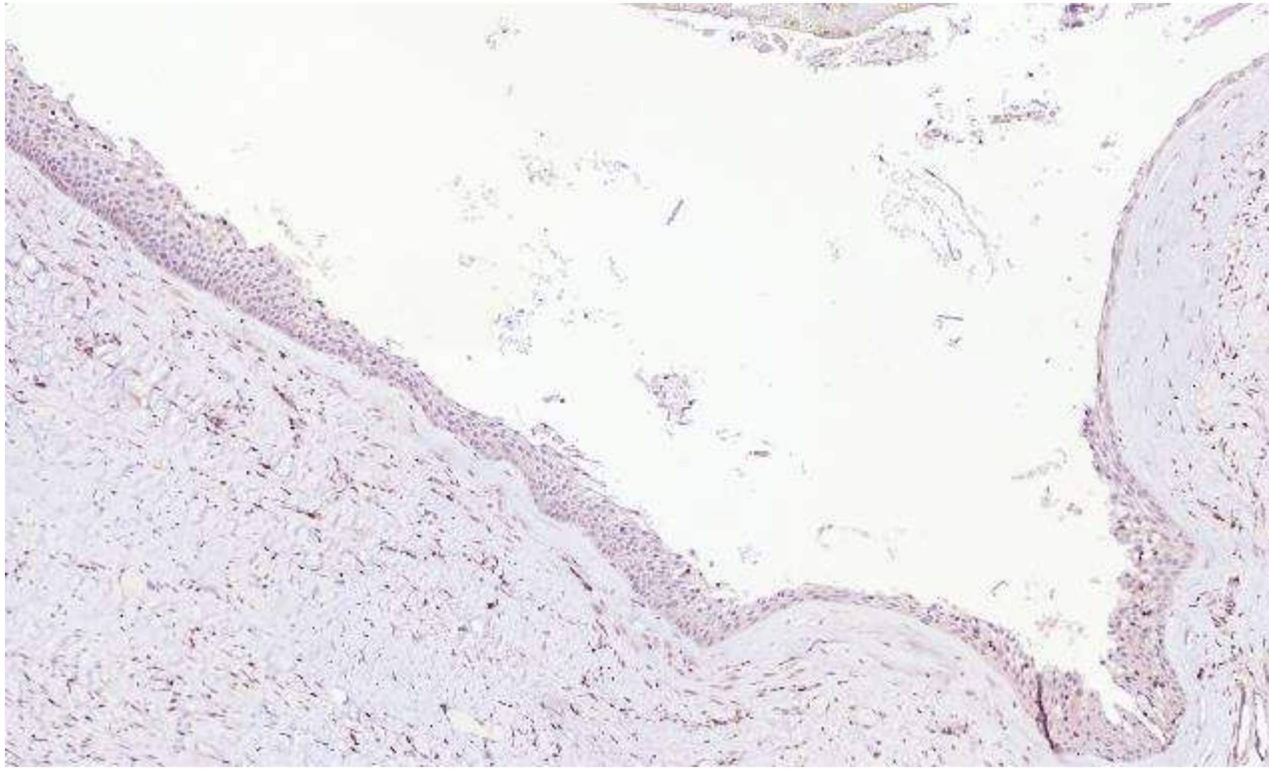

K22 – HE, CK17, bcl2 (x20)

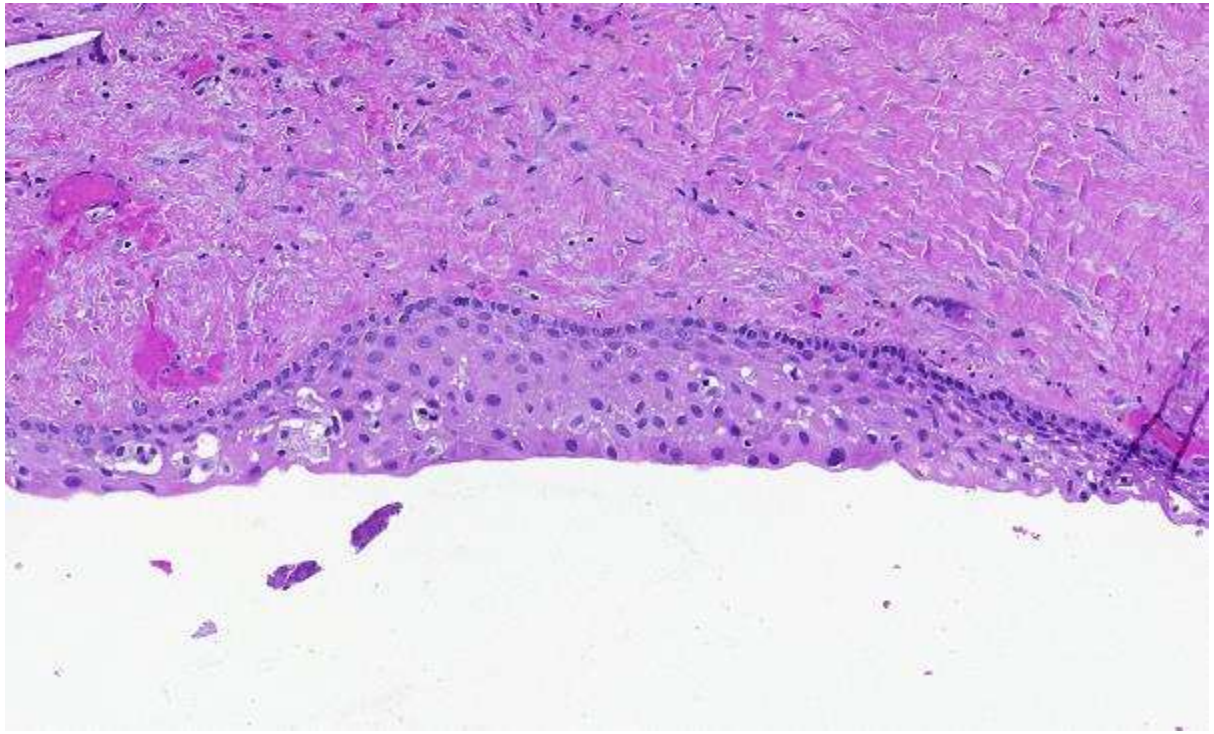

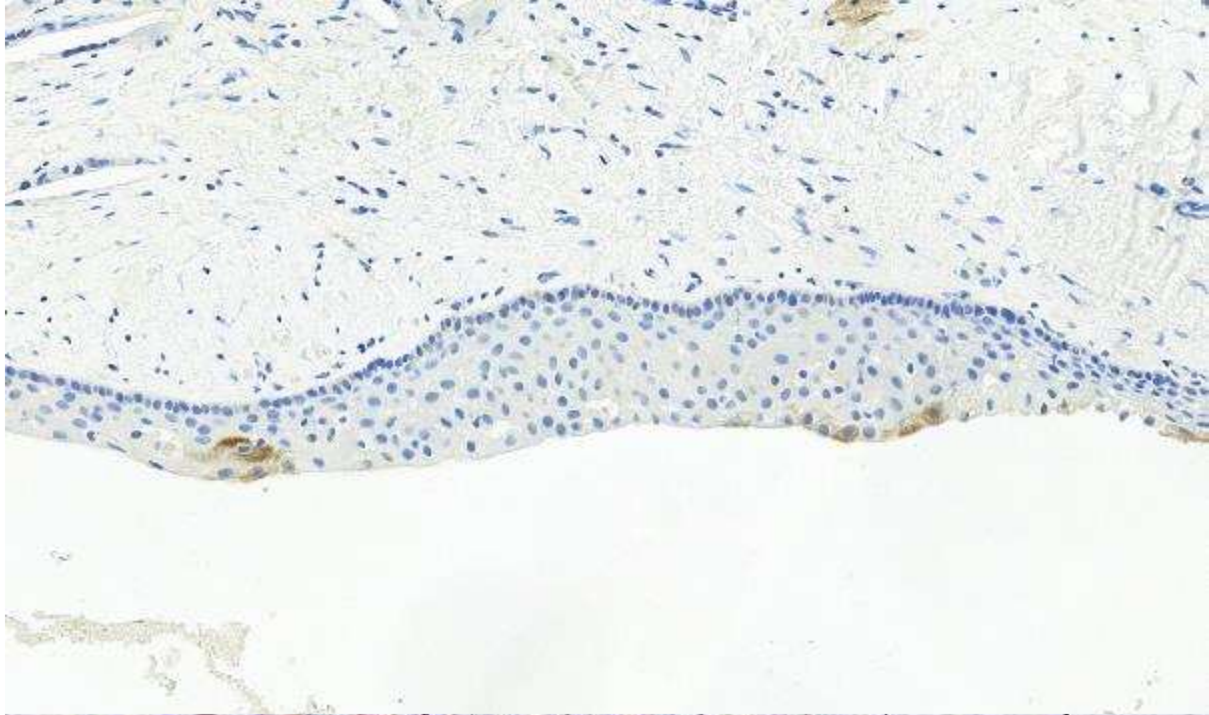

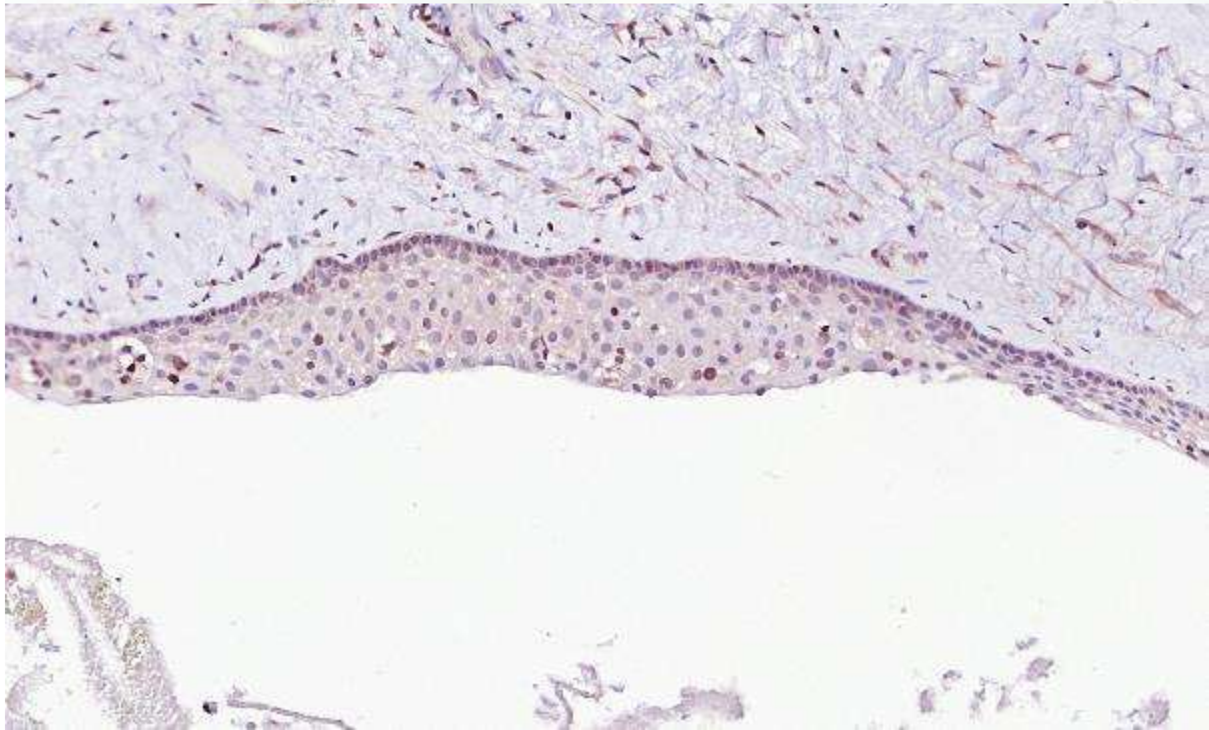

K22 – Conclusion

- H&E: relatively thick (focally thin) squamous epithelium, no parakeratosis, no wavy surface, no basal palisading, even to uneven epithelial-subepithelial contours
- Not inflamed, but cholesterol clefts in some areas of the cyst wall
- Bcl2: negative
- CK17: dominantly negative, with focal superficial, basal or transepithelial staining
- Histopathology: non-inflammatory odontogenic cyst
- Combined clinical and pathological opinion: lateral periodontal cyst

Case K23

- 54-year-old male
- Dental hygiene/status: poor hygiene; partly missing, treated teeth
- Radiology: Unilocular radiolucent lesion (barely visible on OPT) of around 1 cm in diameter at the apex of tooth 15 expanding to the base of the maxillary sinus.
- Operation: Cystectomy
- Clinical diagnosis: „maxillary cyst” (NOS)

K23 – OPT

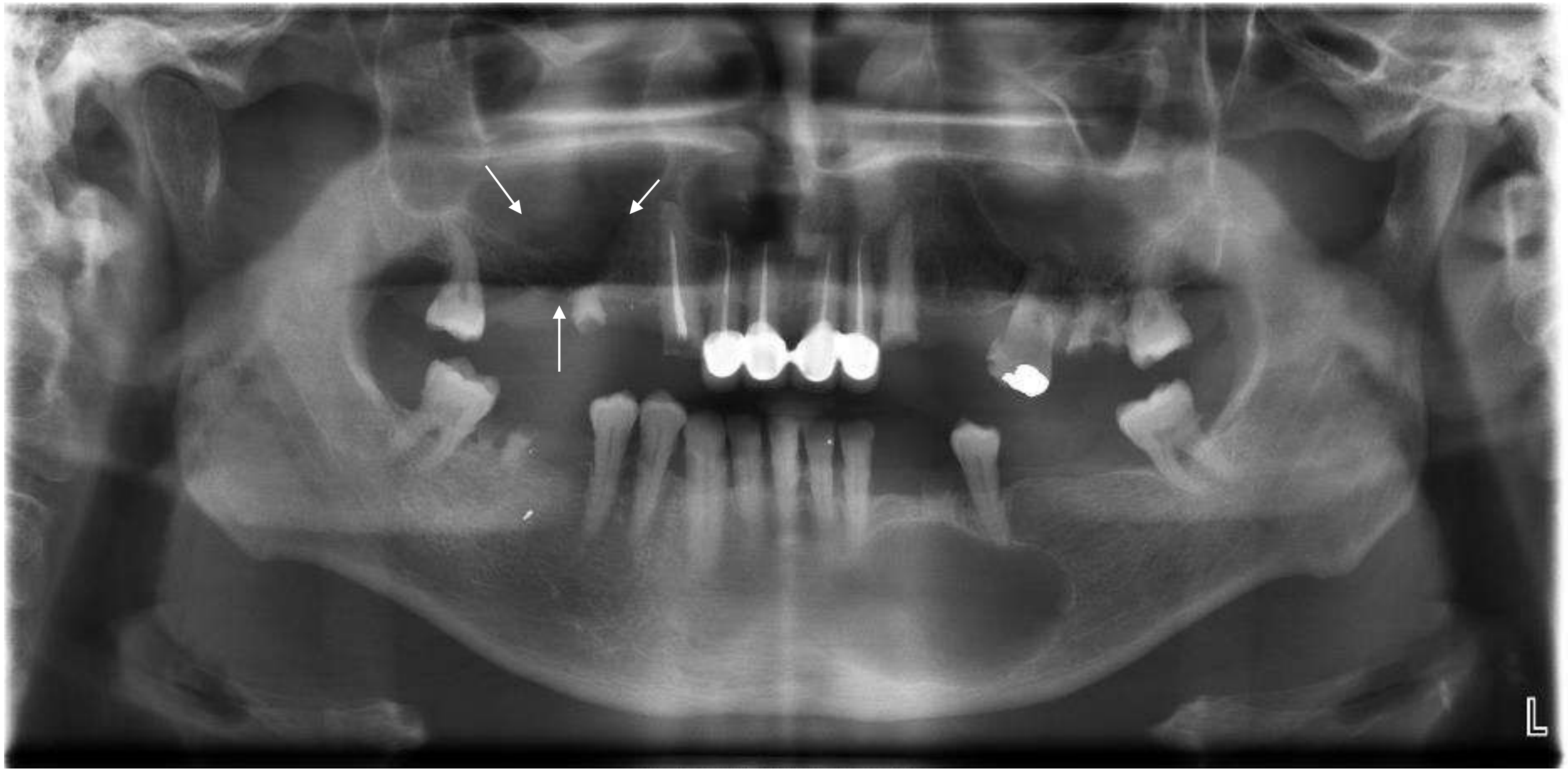

K23 – CT

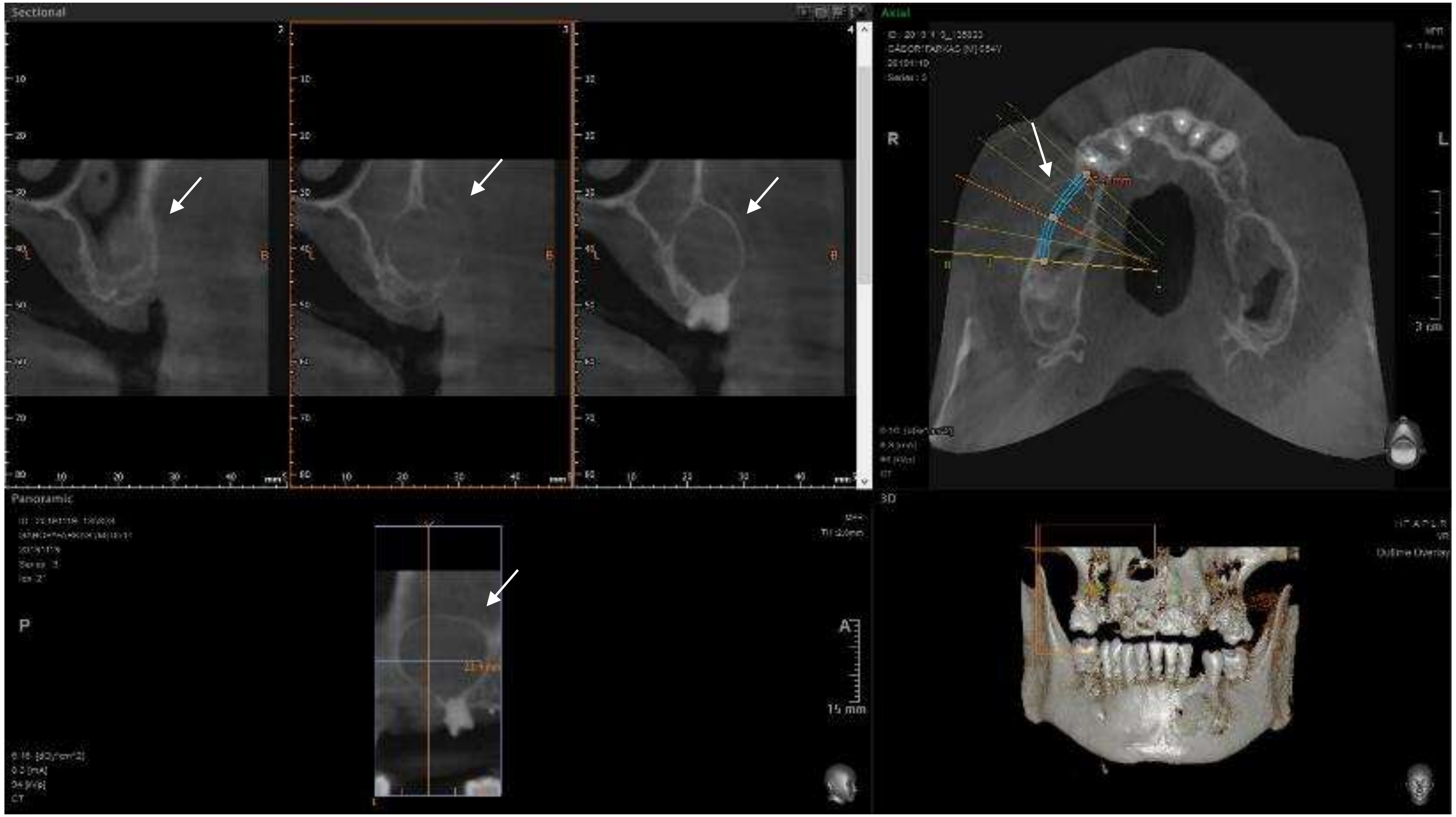

K23 – HE, CK17, bcl2 (x0.8)

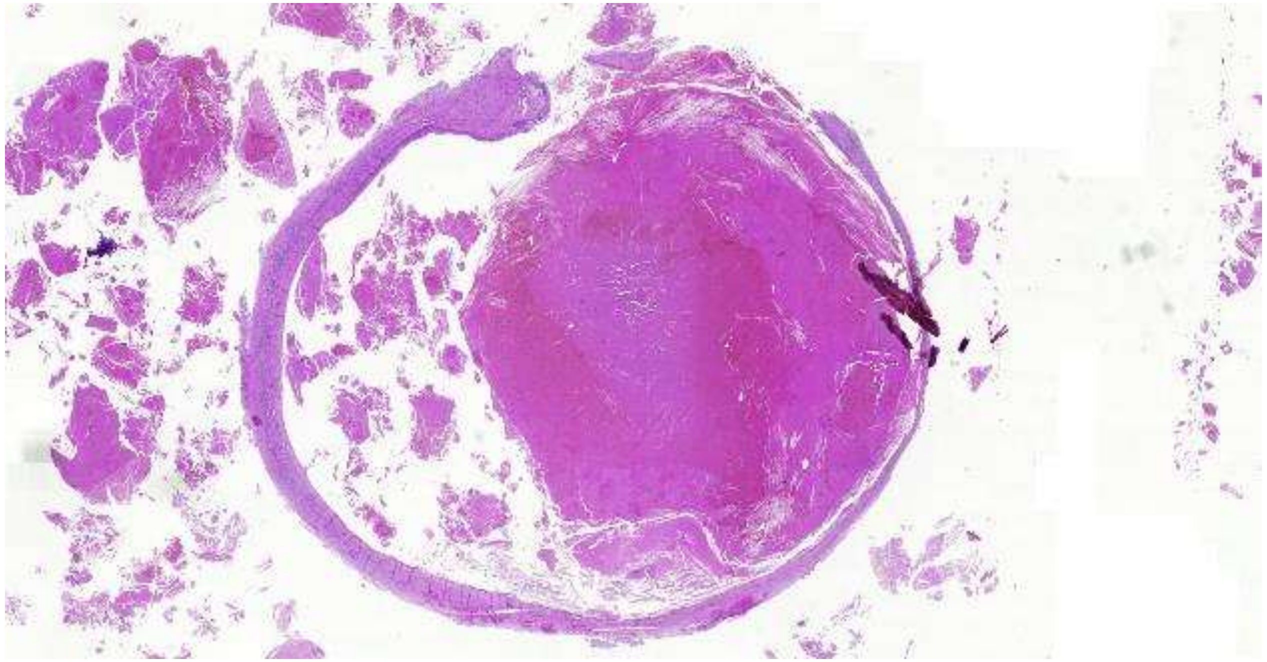

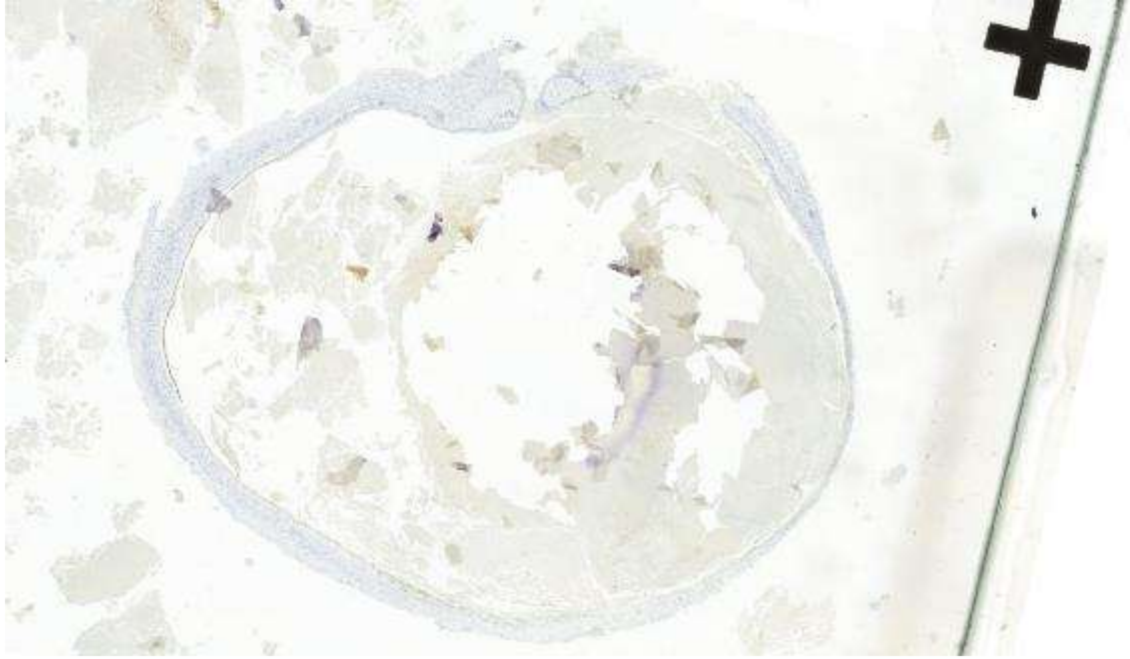

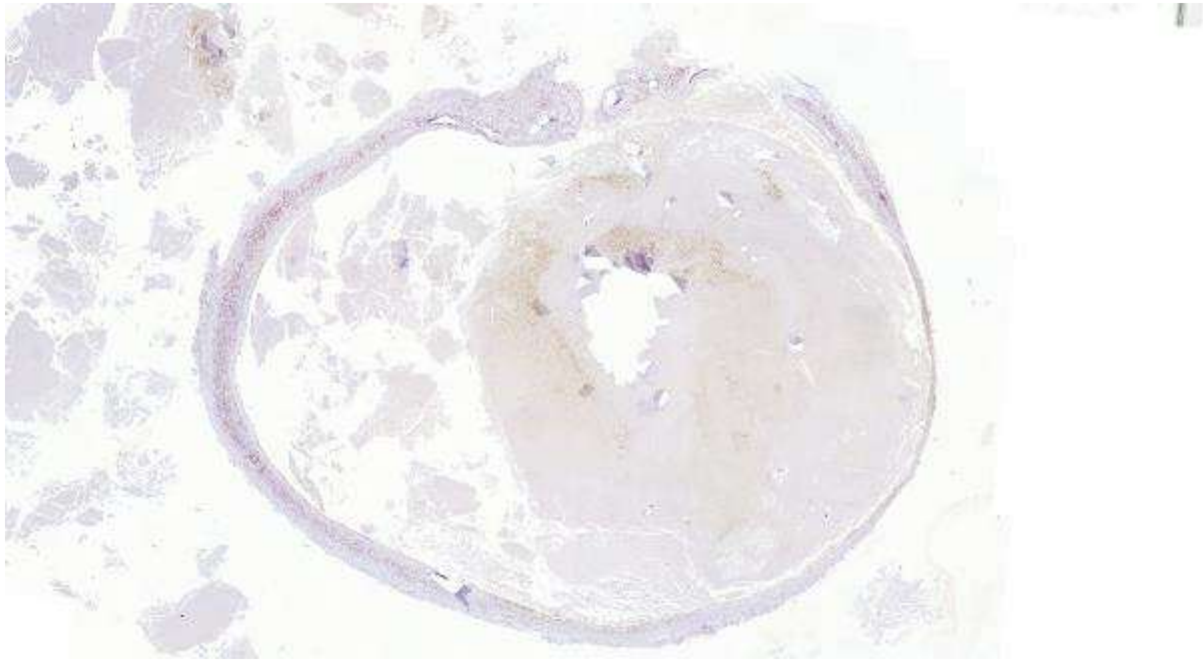

K23 – HE, CK17, bcl2 (x10)

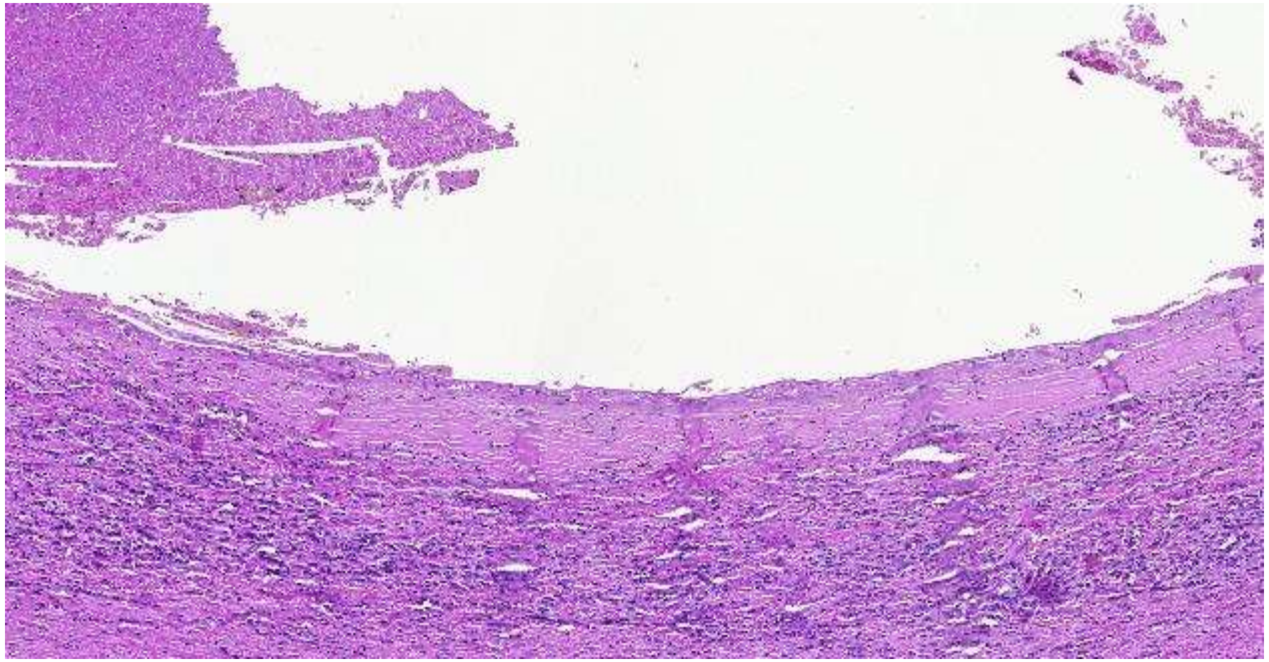

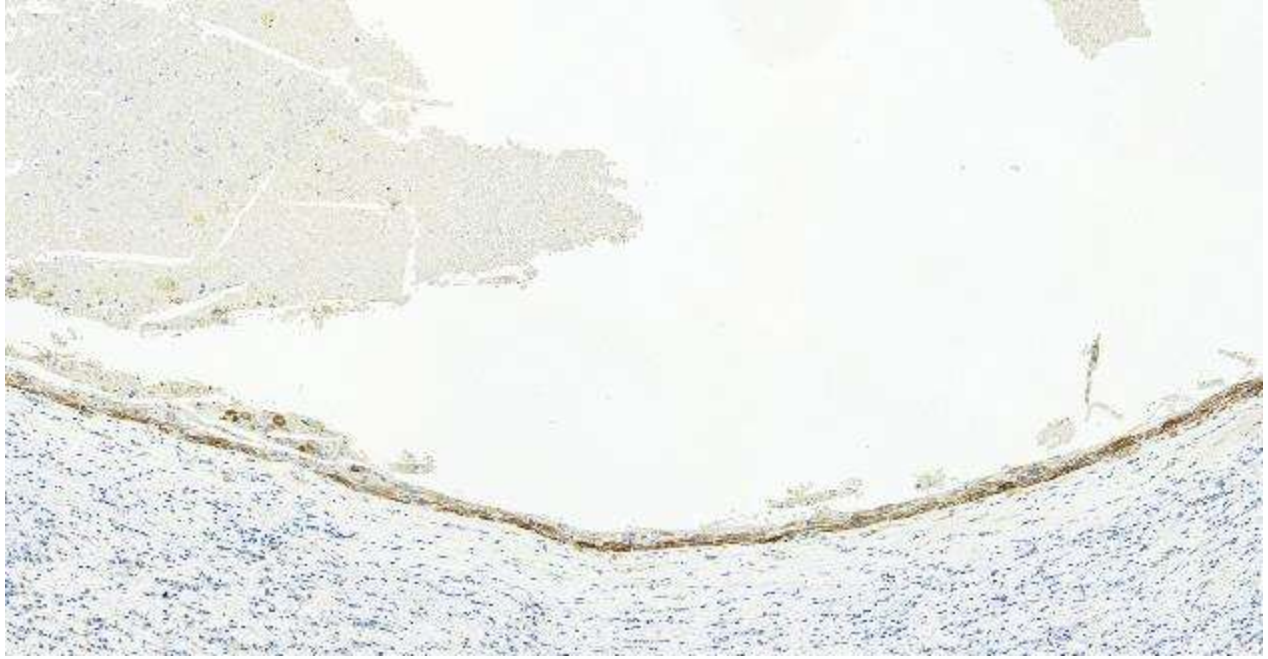

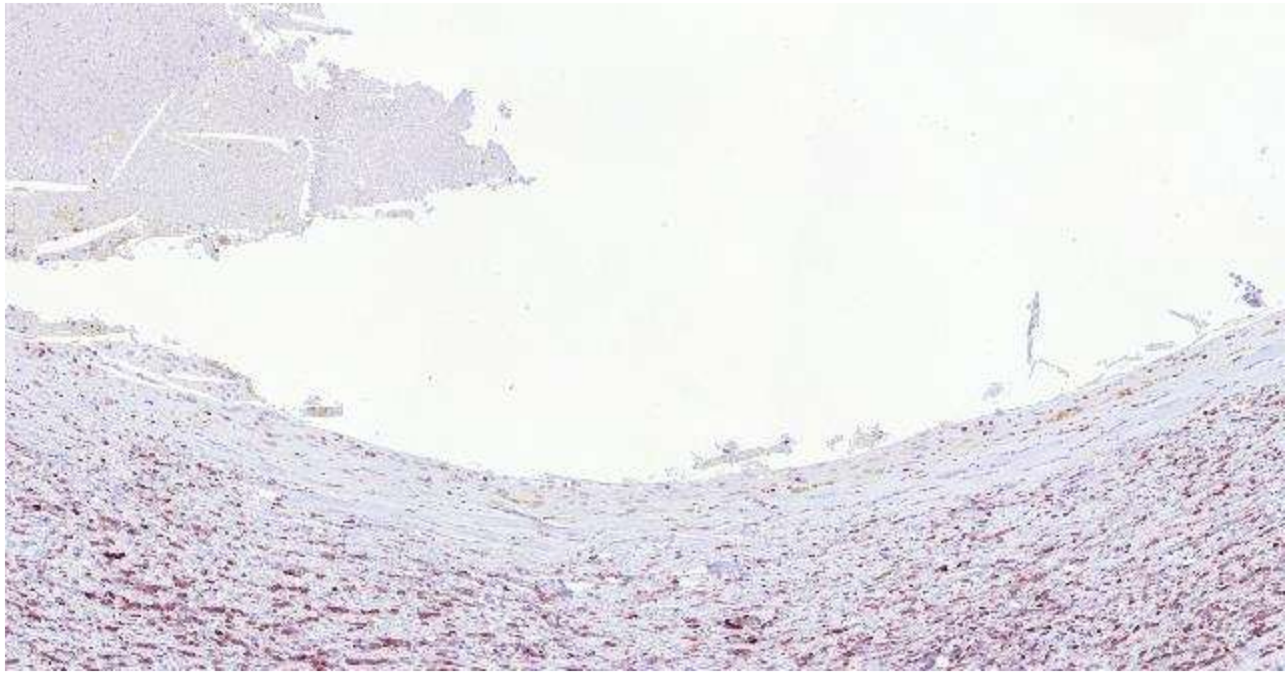

K23 – HE, CK17, bcl2 (x10)

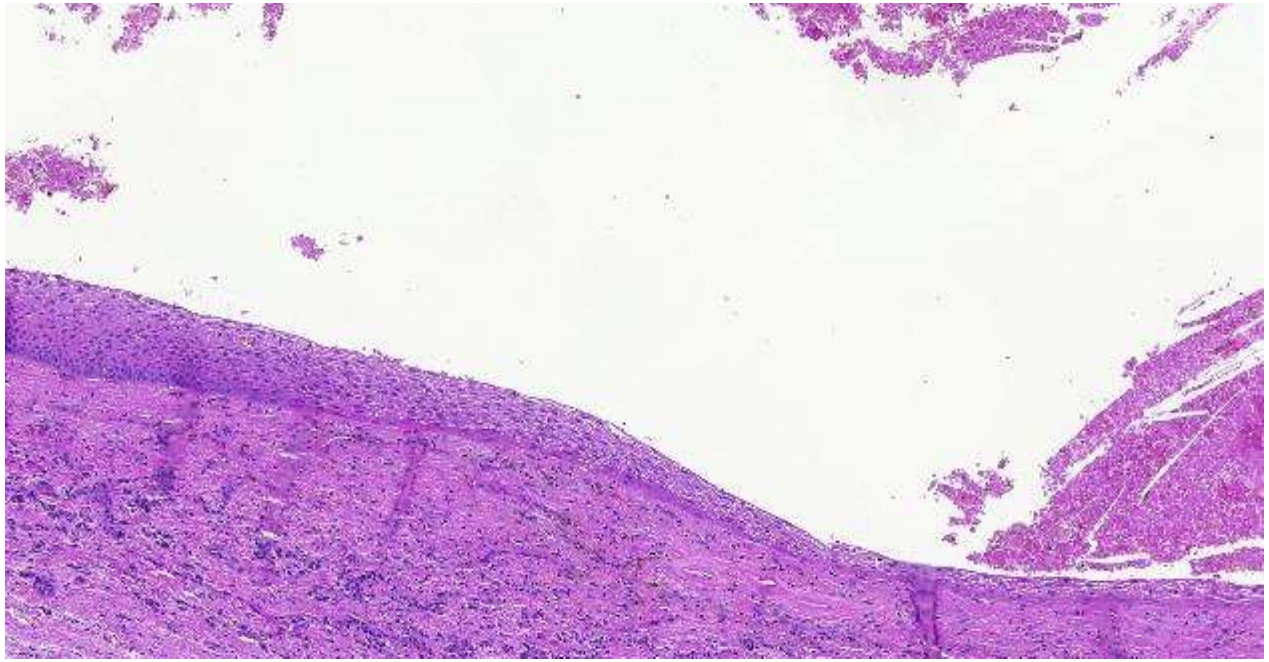

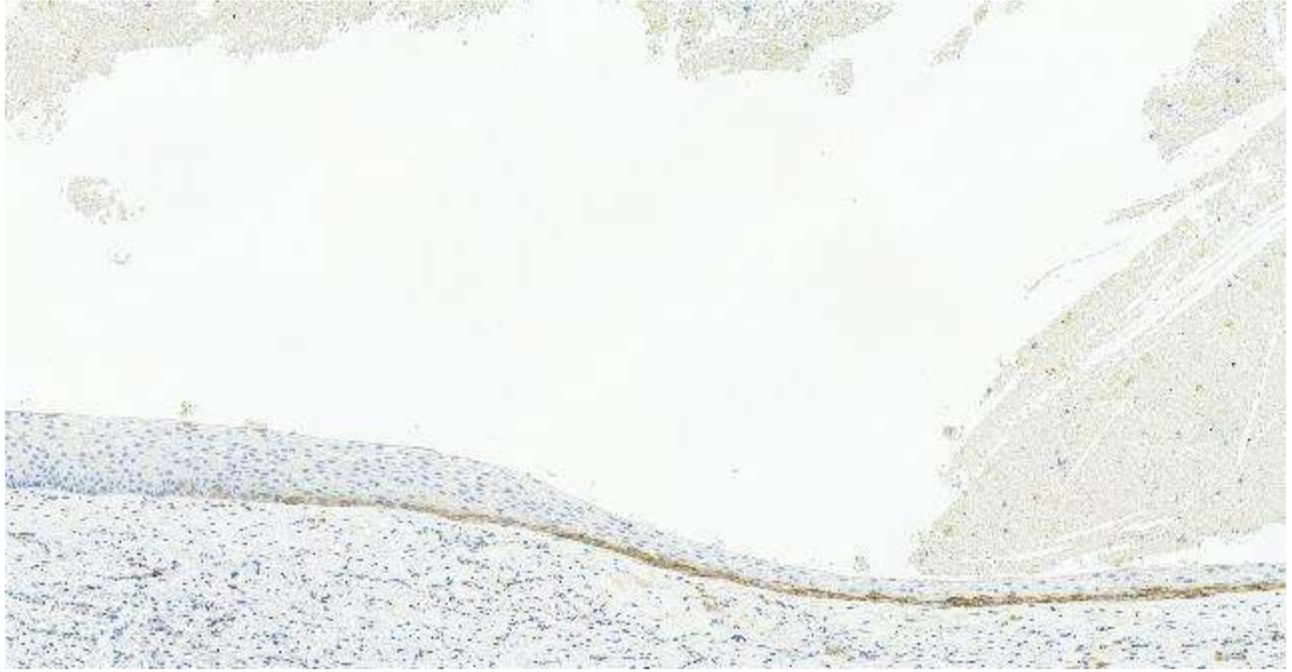

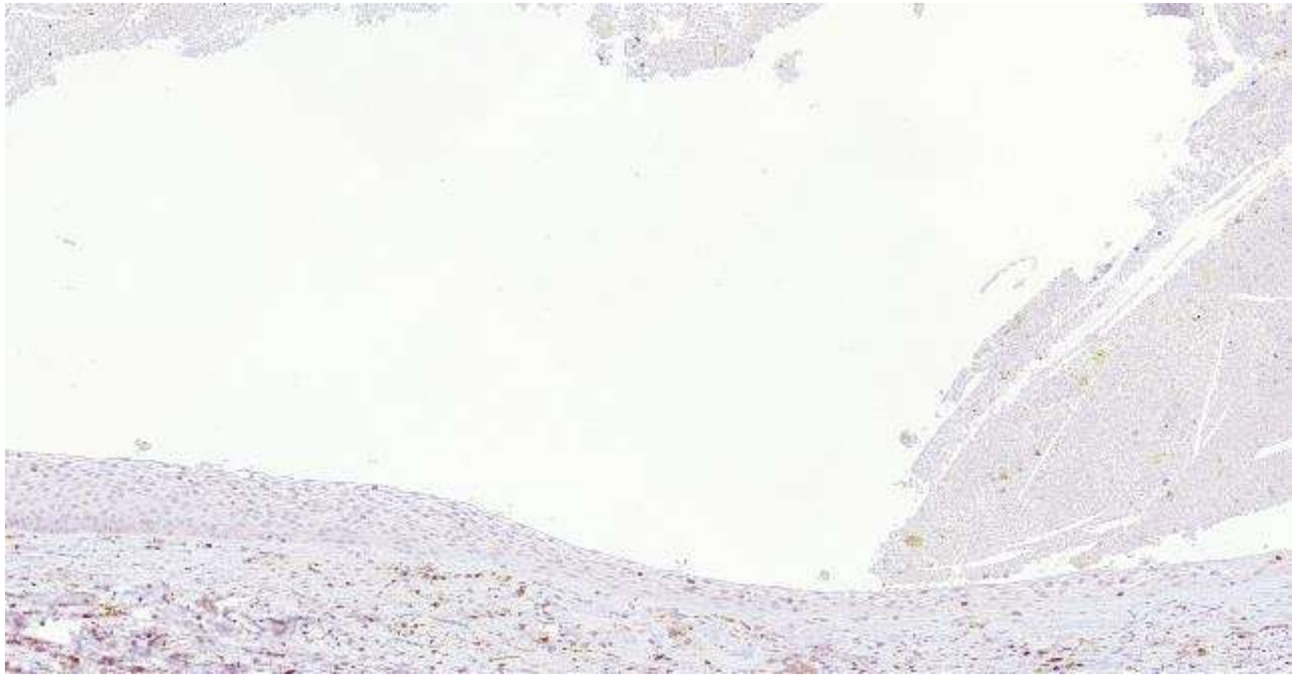

K23 – HE, CK17, bcl2 (x10)

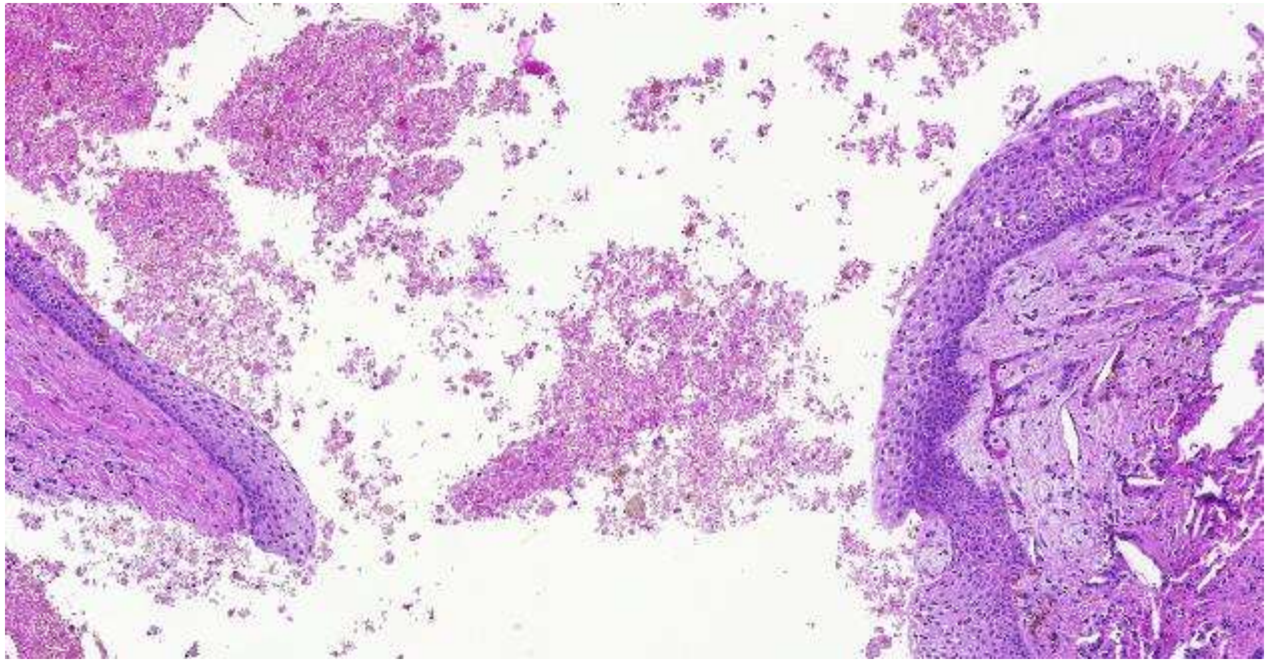

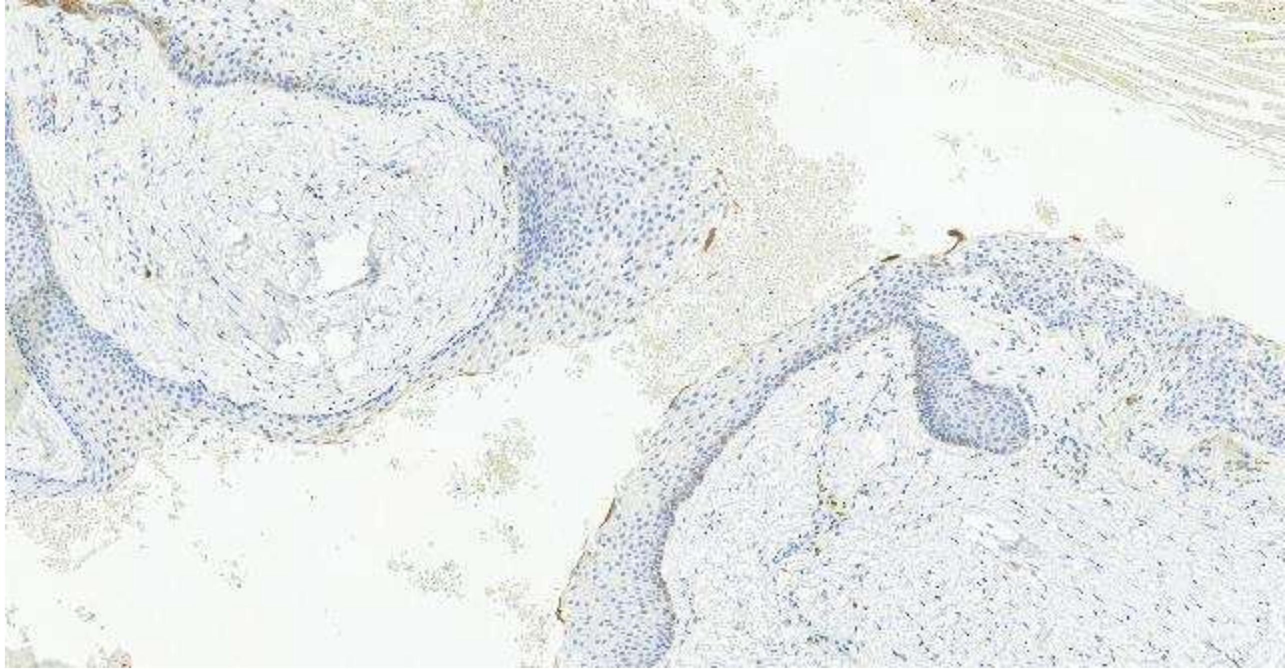

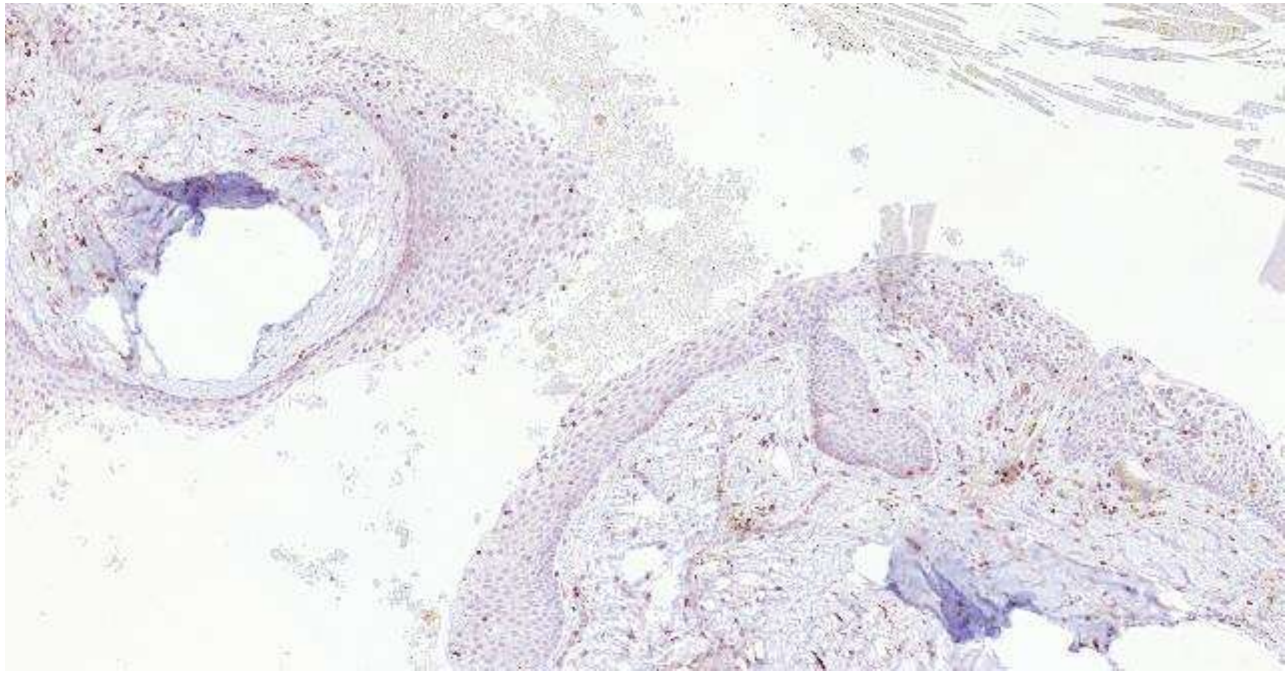

K23 – Conclusion

- H&E: relatively thick (and areas of thin) non-keratinizing squamous epithelium, no parakeratosis, no wavy surface, no basal palisading, even epithelial-subepithelial contours
- Mild chronic inflammation of the cyst wall, intracystic cholesterol granuloma
- Bcl2: negative
- CK17: focal superficial or basal staining with small area of transepithelial at the site of epithelial thinning.
- Histopathology: inflammatory (radicular) cyst
- Combined clinical and pathological opinion: radicular cyst

Case K24

- 47-year-old male (same as K25 and K26)
- Dental hygiene/status: poor hygiene; partly missing, dominantly untreated, partly treated teeth, teeth 11, 12, 15, 16, 21, 22, 23, 26, 32, 33 without crowns, roots in the alveoli; bridge over 31-43.
- Radiology: 16 mm large unilocular radiolucent lesion between teeth 11 and 12 extending to the nasal base
- Operation: Removal of roots of teeth 11, 12, and 15, 16 and the cysts; opening of the maxillary sinus - modified Caldwell-Luc operation
- Clinical diagnosis: „maxillary cyst” (periapical)

K24 – OPT

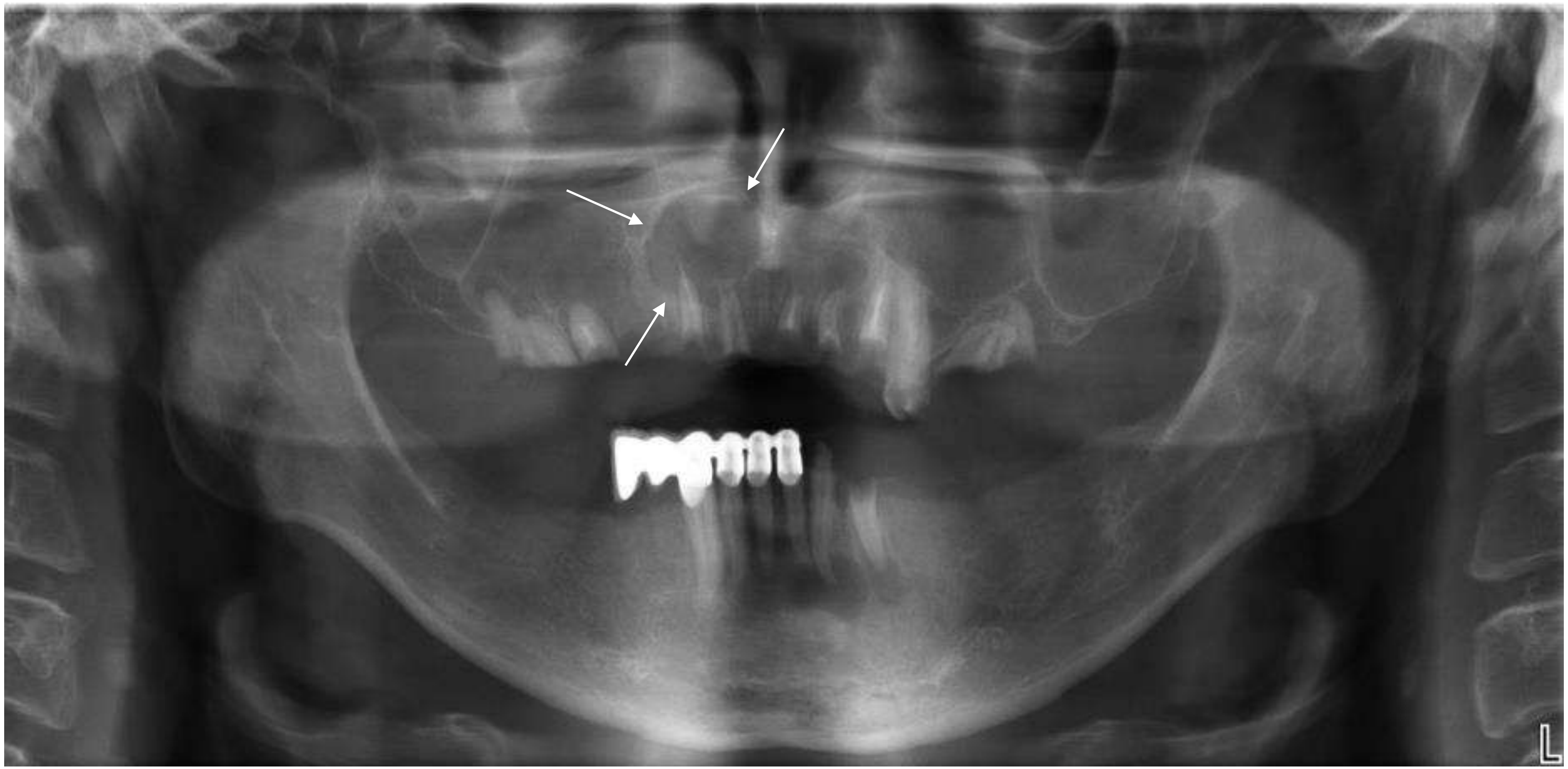

K24 – HE, CK17, bcl2 (x2)

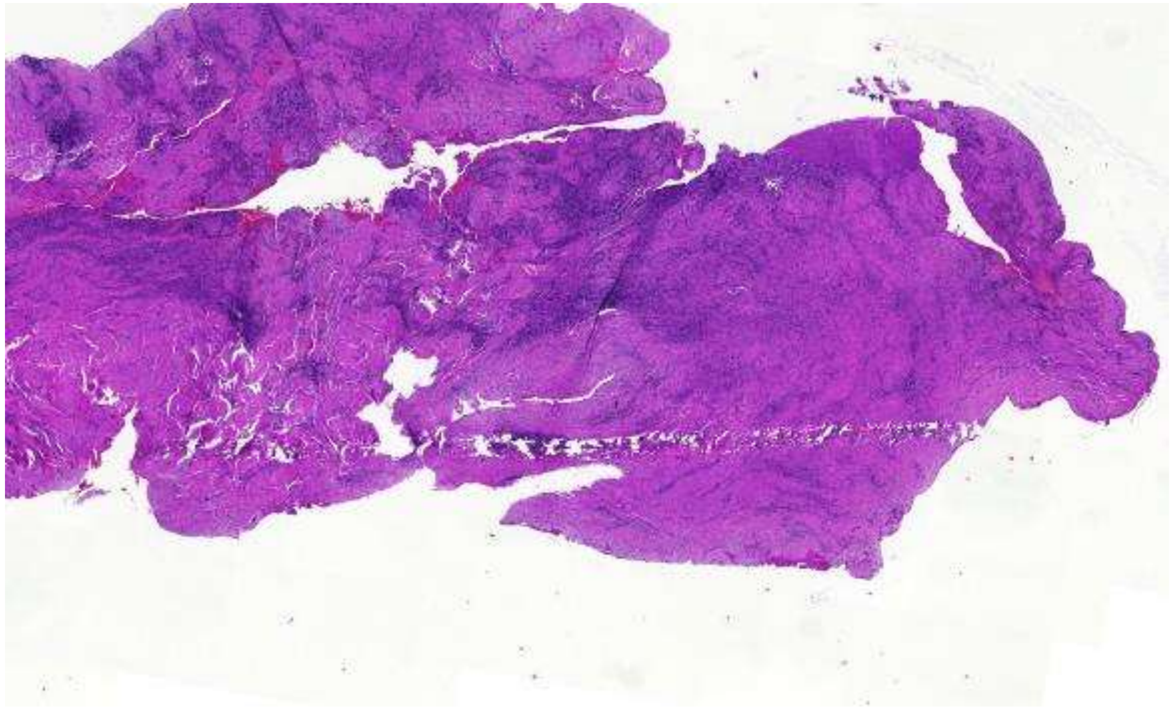

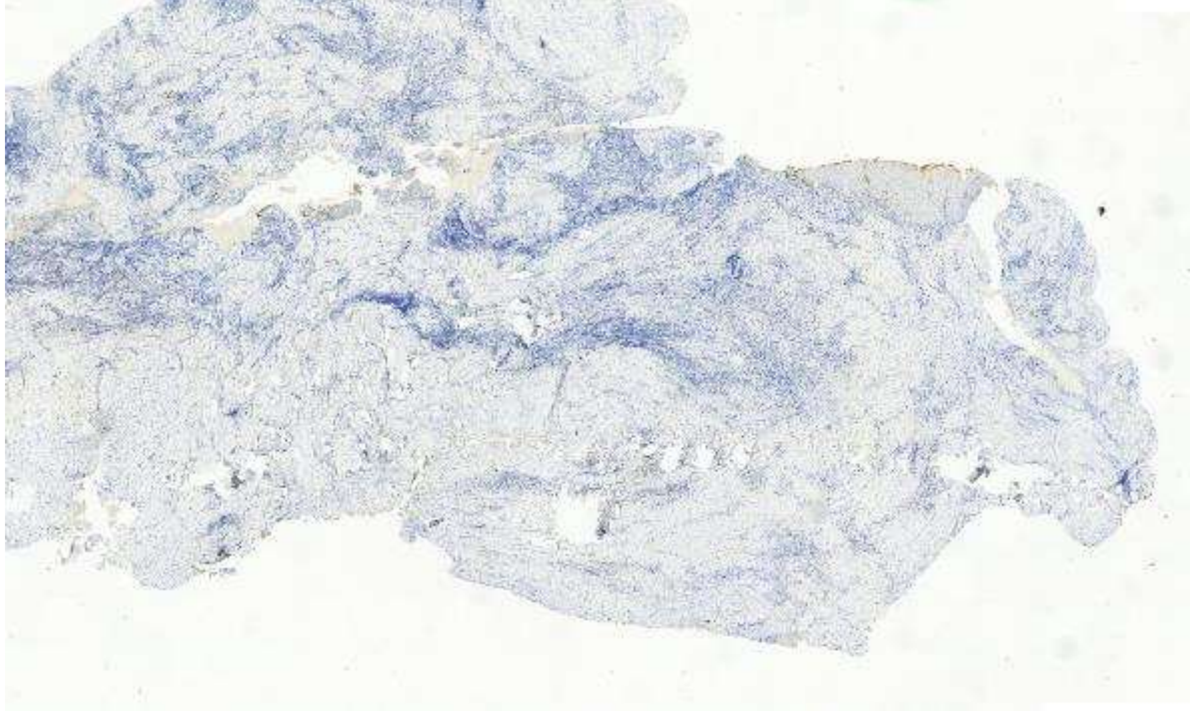

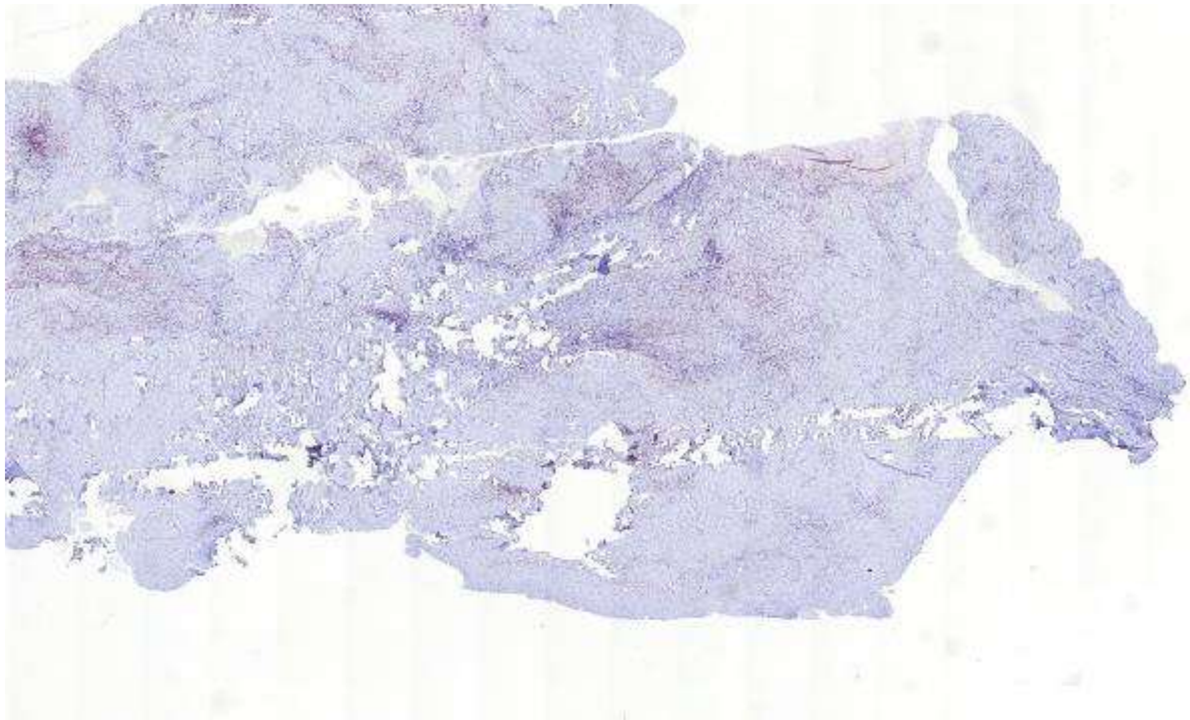

K24 – HE, CK17, bcl2 (x10)

Superficial (↑↑) and focal basal (↑) CK17 staining, the latter also appreciated at the tip of the papillae

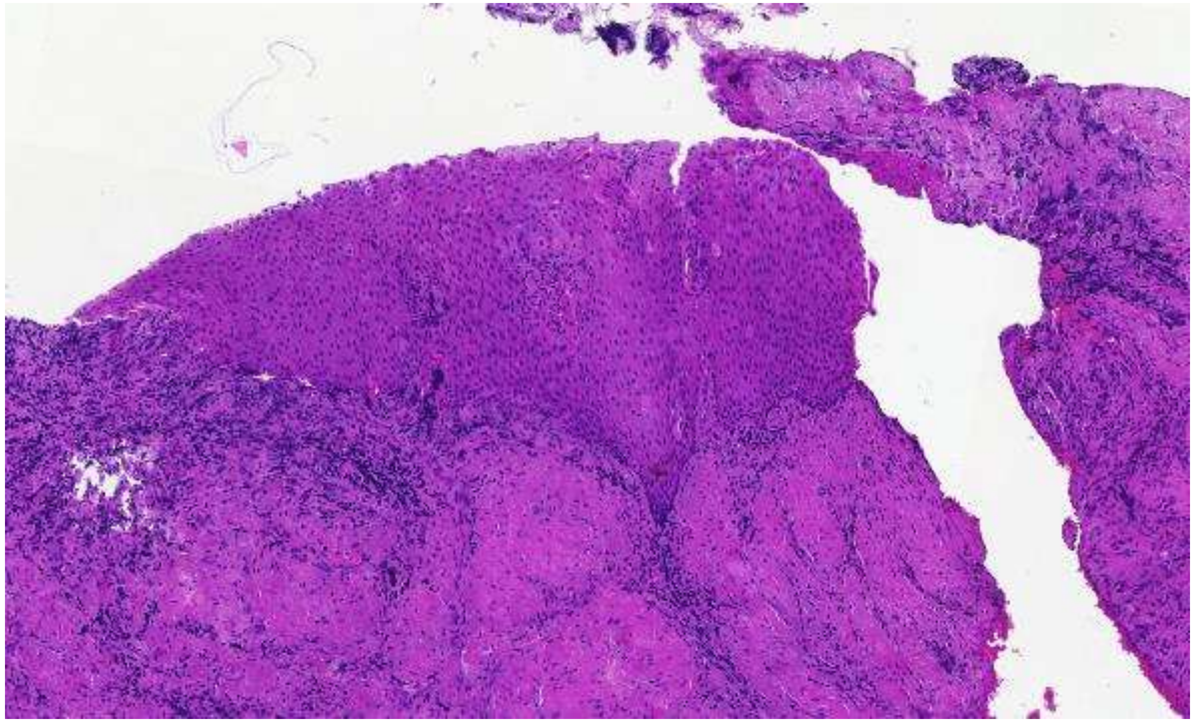

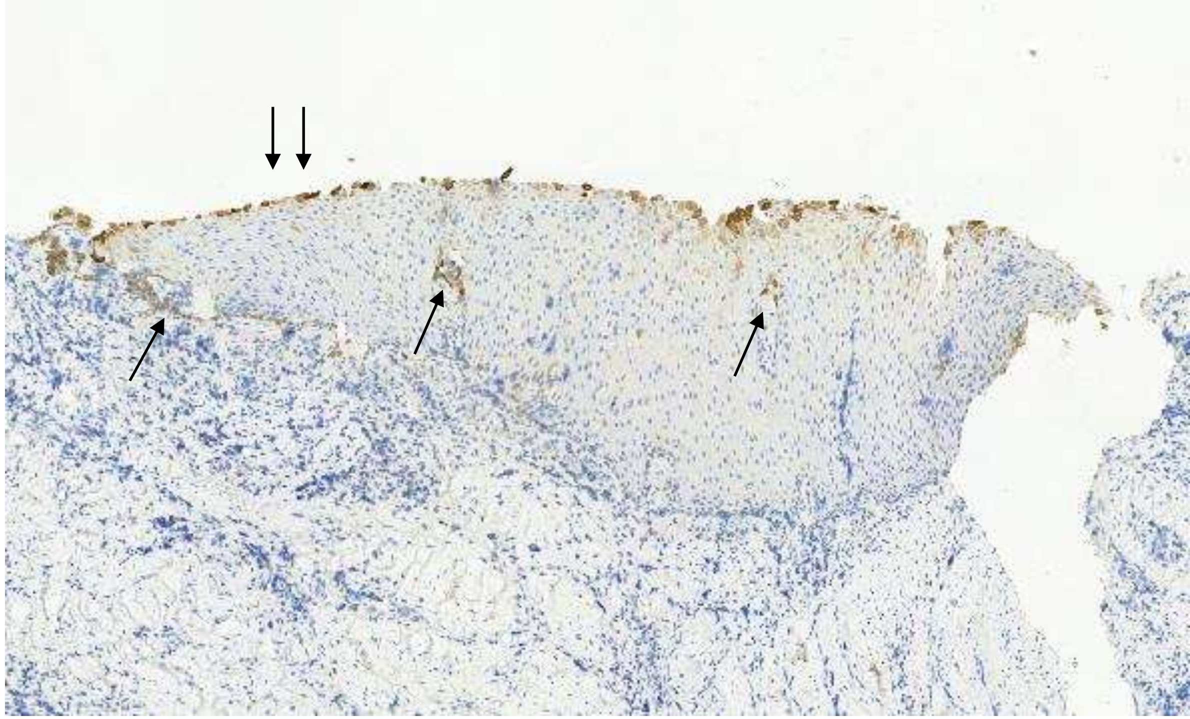

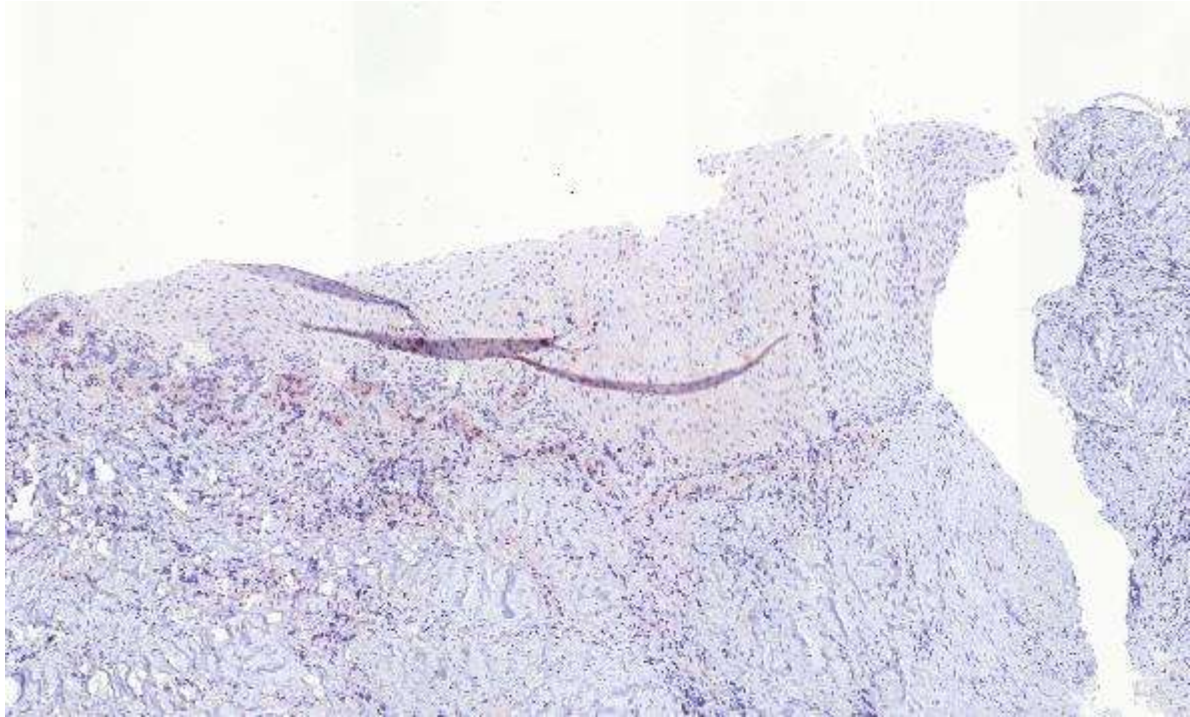

K24 – HE, CK17, bcl2 (x20)

Focal superficial and upper spinous CK17 staining

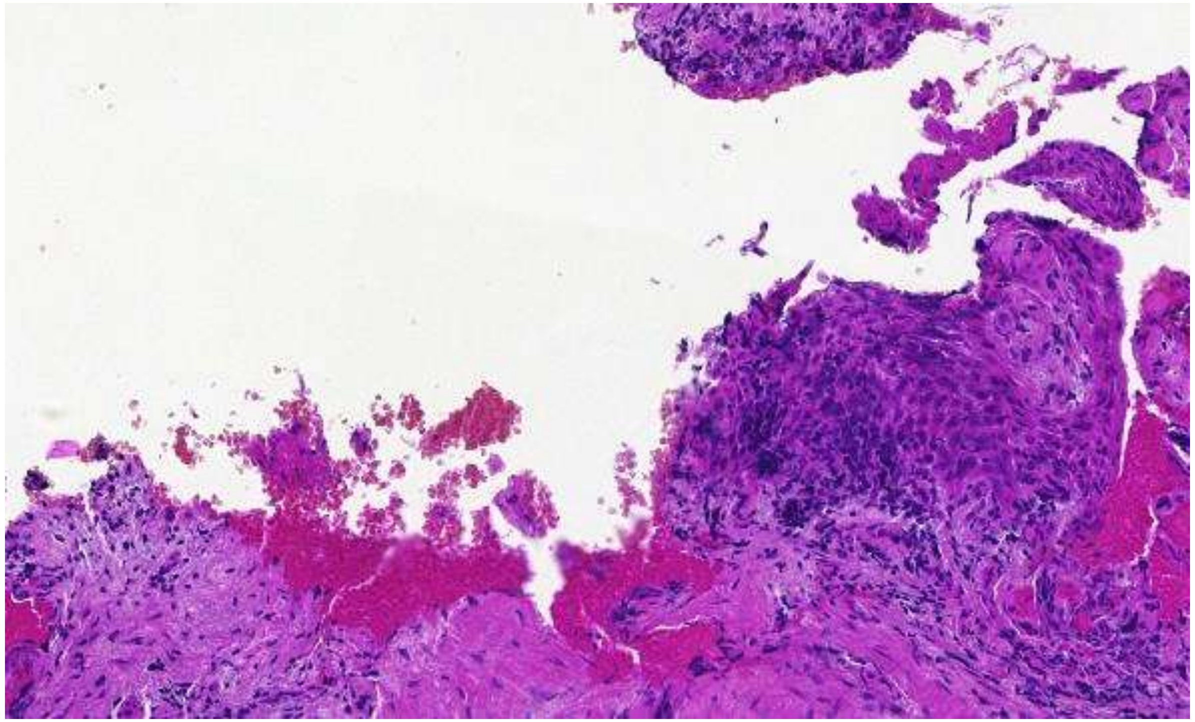

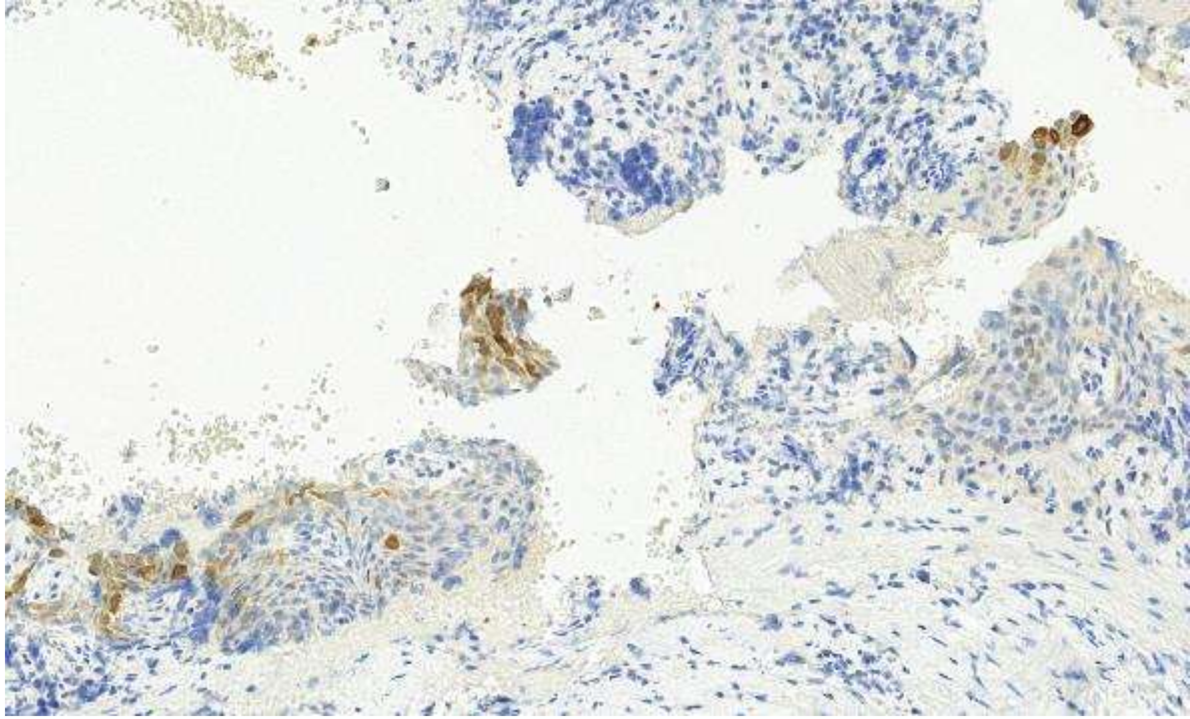

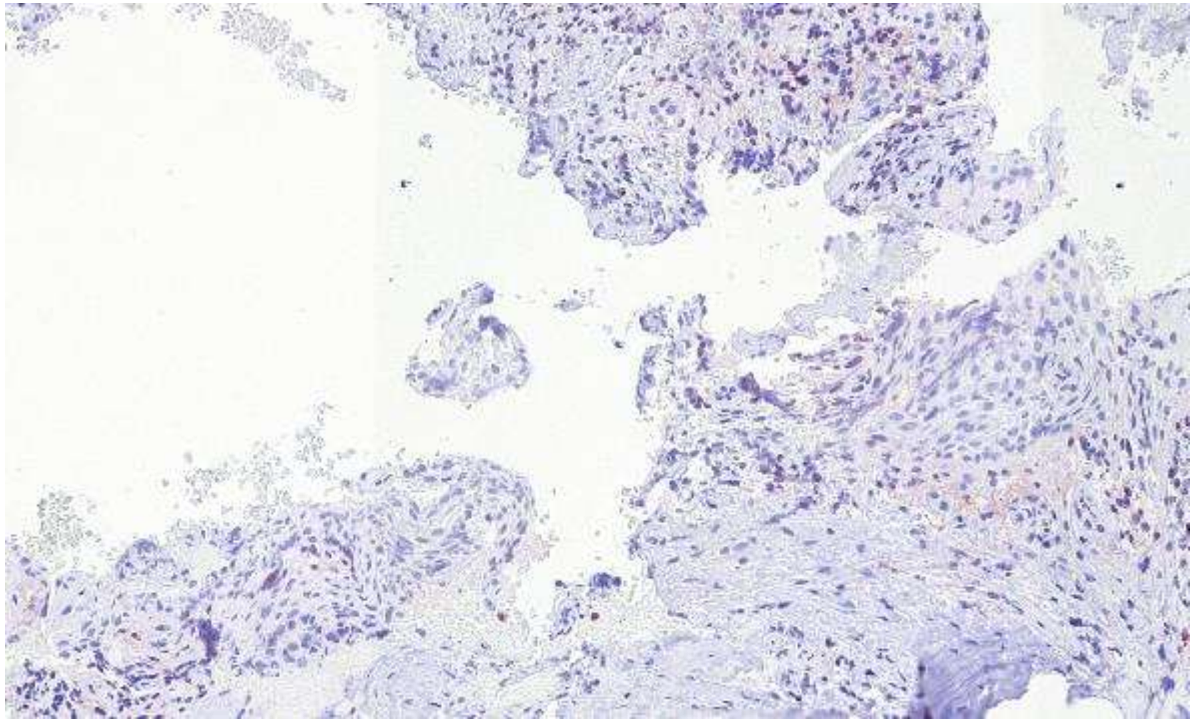

K24 – HE, CK17, bcl2 (x15)

Weak superficial CK17 staining

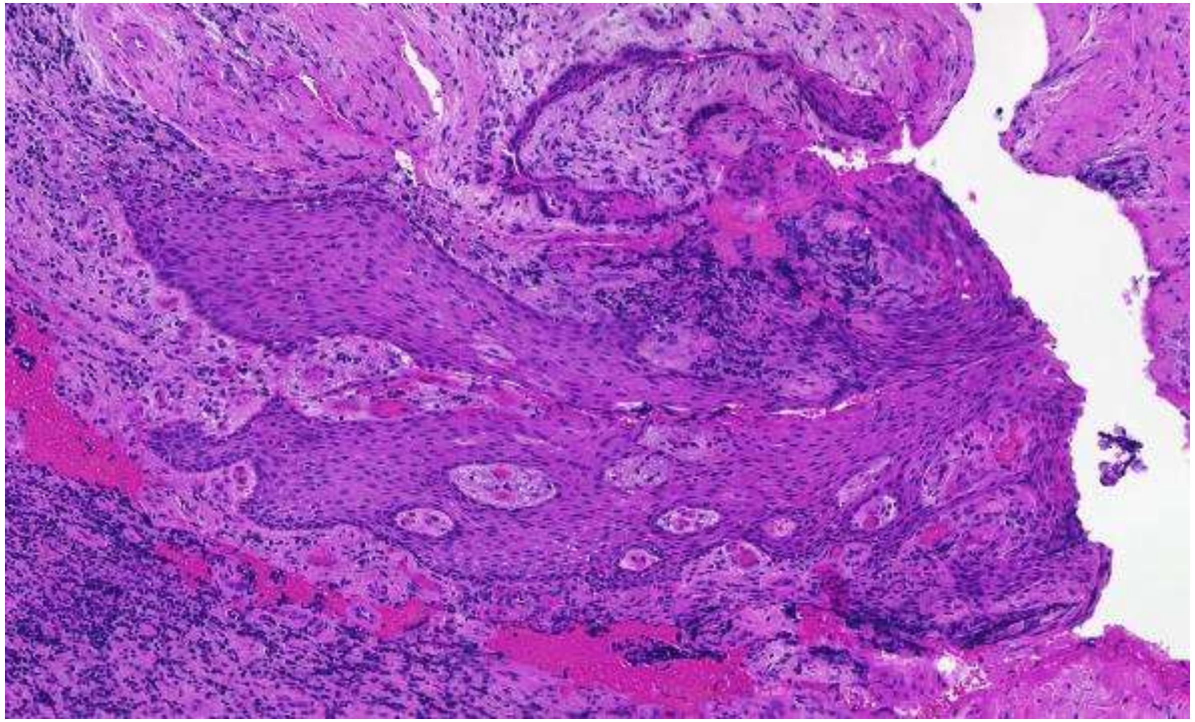

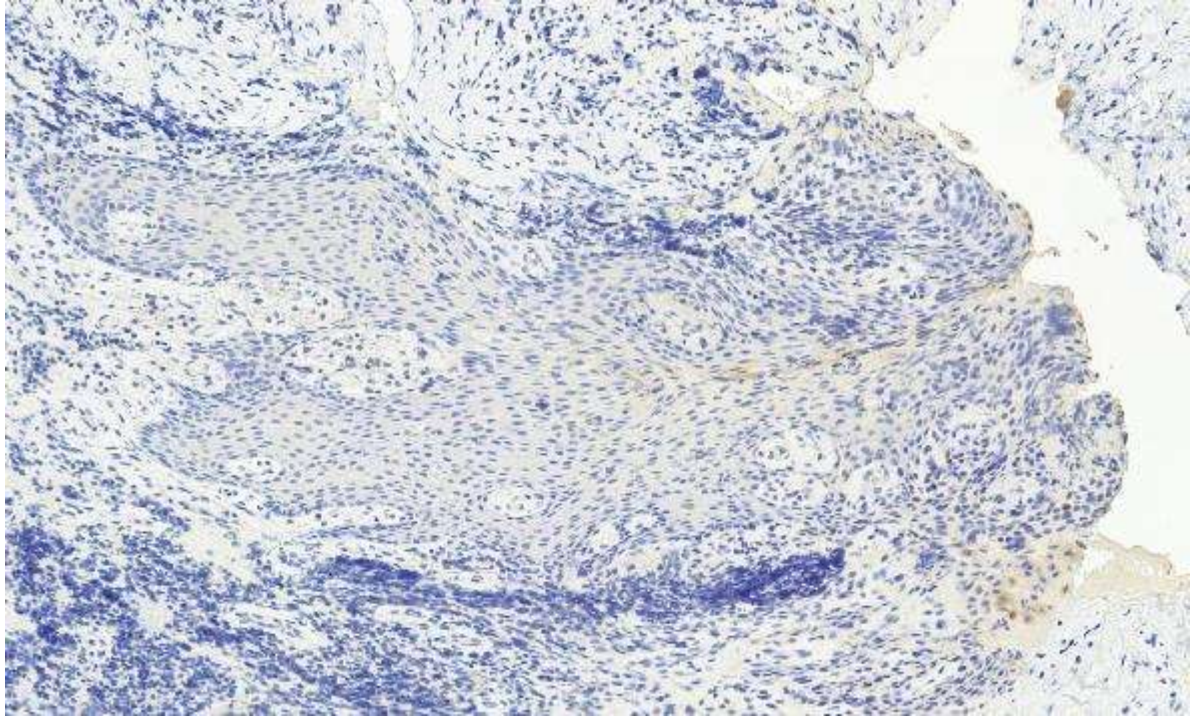

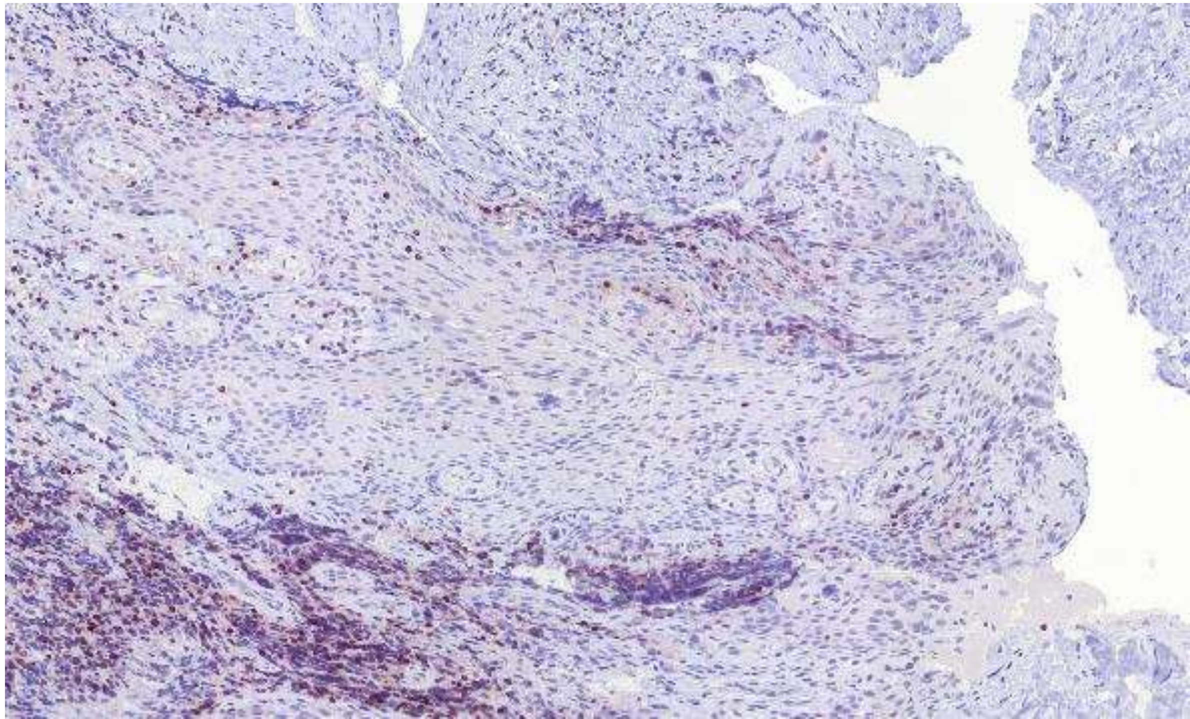

K24 – Conclusion

- H&E: relatively thick (mostly denuded) squamous epithelium, no parakeratosis, no wavy surface, no basal palisading, uneven epithelial-subepithelial contours
- Inflammation of the cyst wall
- Bcl2: negative
- CK17: mainly superficial staining with small area of basal or upper spinous layer staining.
- Histopathology: inflammatory cyst (N.O.S.)
- Combined clinical and pathological opinion: radicular cyst

Case K25

- 47-year-old male (same as K24 and K26)
- Dental hygiene/status: poor hygiene; partly missing, dominantly untreated, partly treated teeth, teeth 11, 12, 15, 16, 21, 22, 23, 26, 32, 33 without crowns, roots in the alveoli; bridge over 31-43.
- Radiology: Sharply circumscribed 16 mm large radiolucent lesion at the roots of tooth 15
- Operation: Removal of roots of teeth 11, 12, and 15, 16 and the cysts; opening of the maxillary sinus - modified Caldwell-Luc operation
- Clinical diagnosis: „maxillary cyst” (periapical)

K25 – OPT

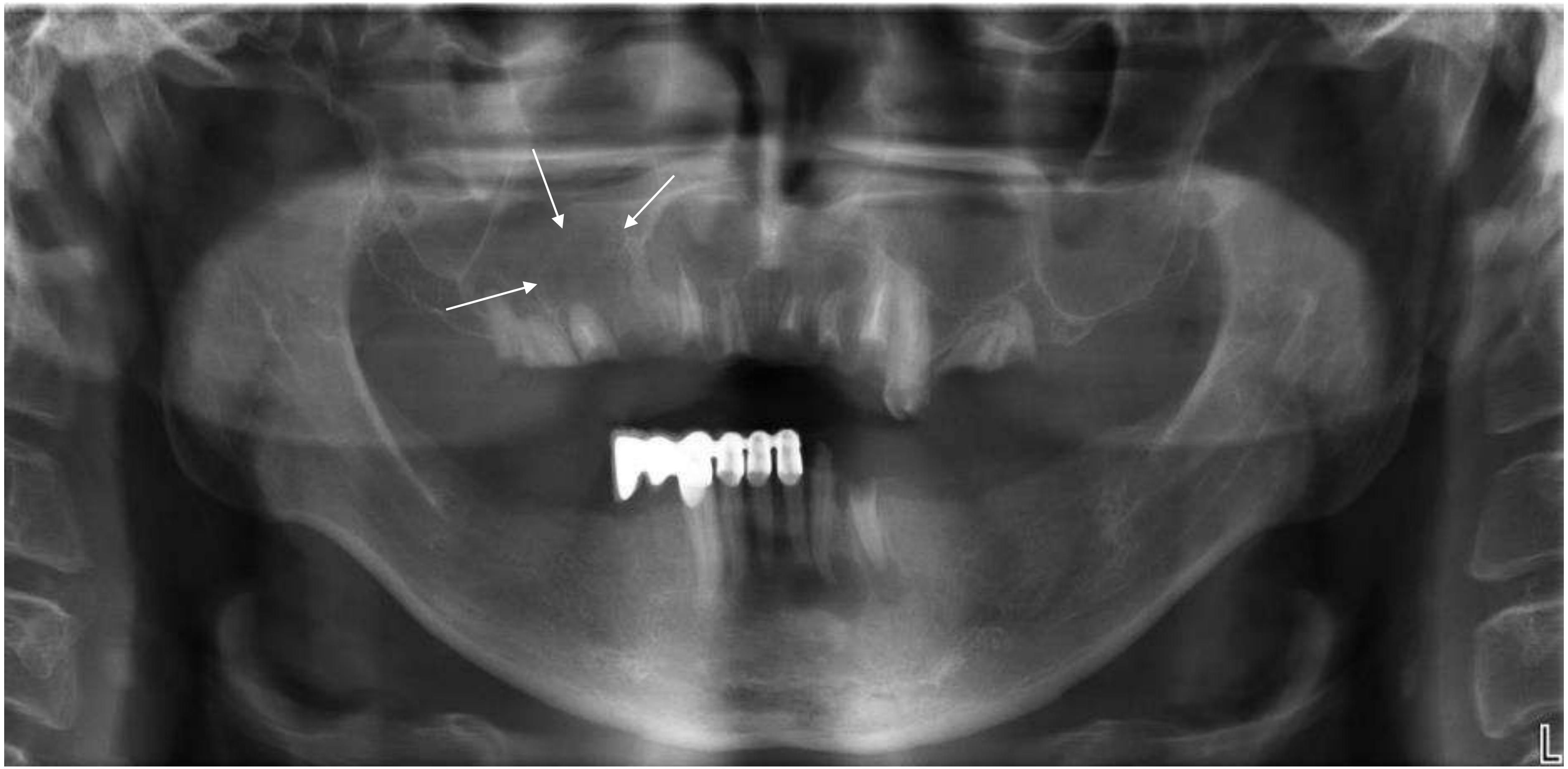

K25 – HE, CK17, bcl2 (x1)

Inflammation

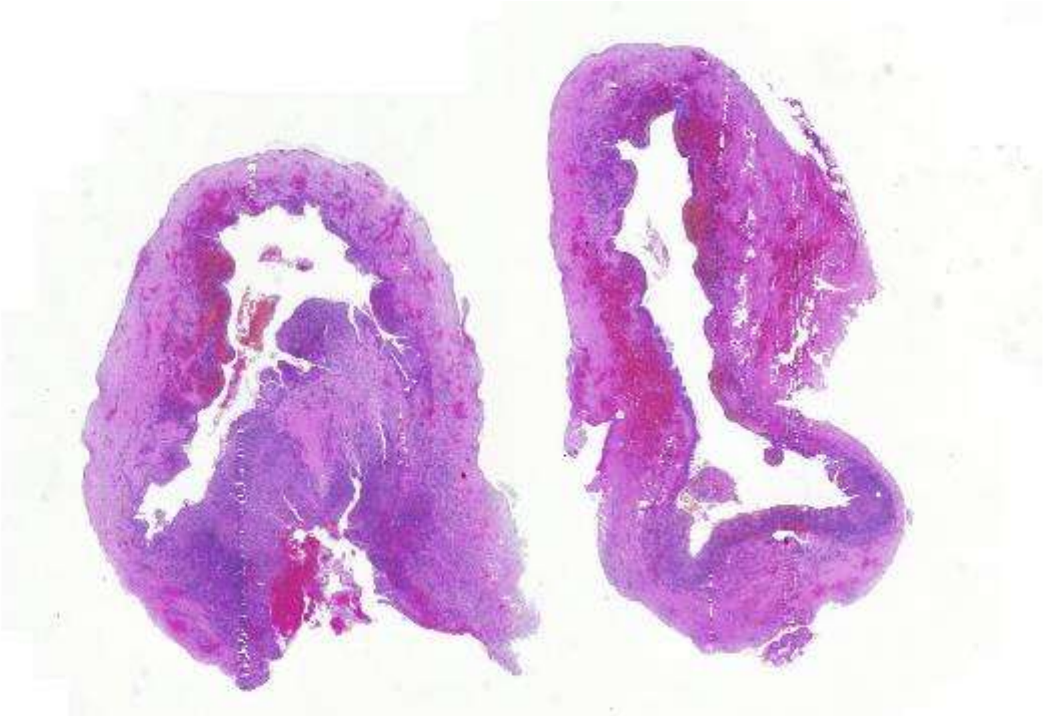

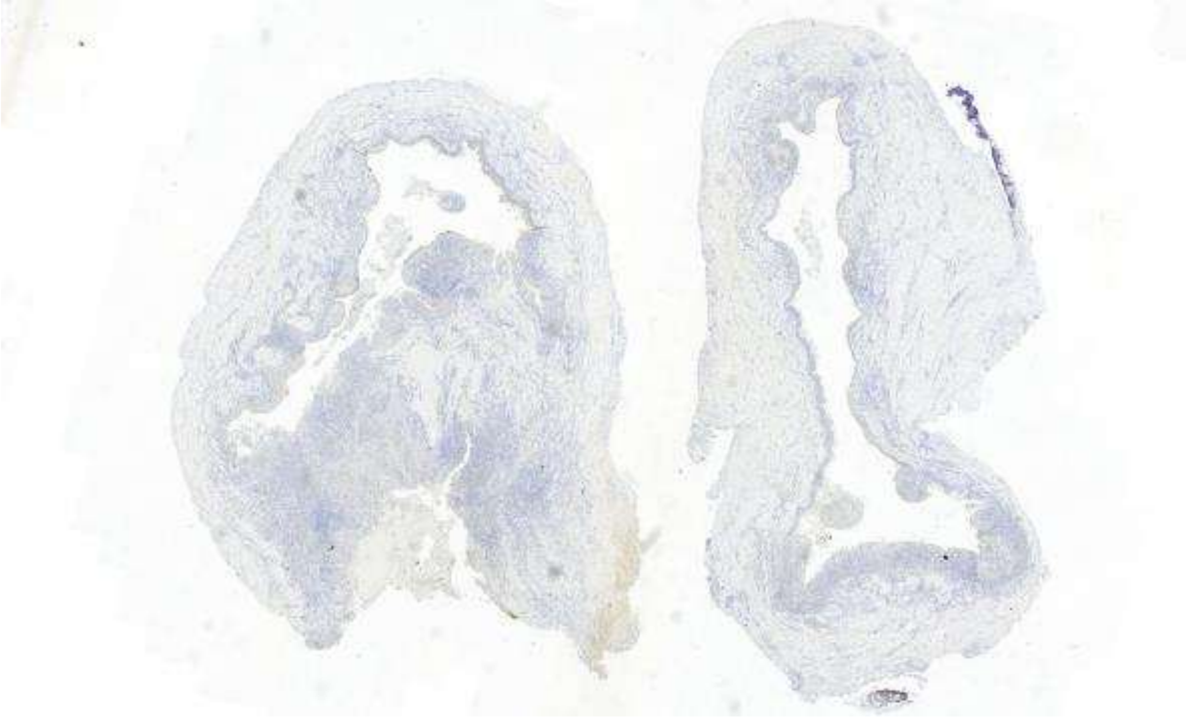

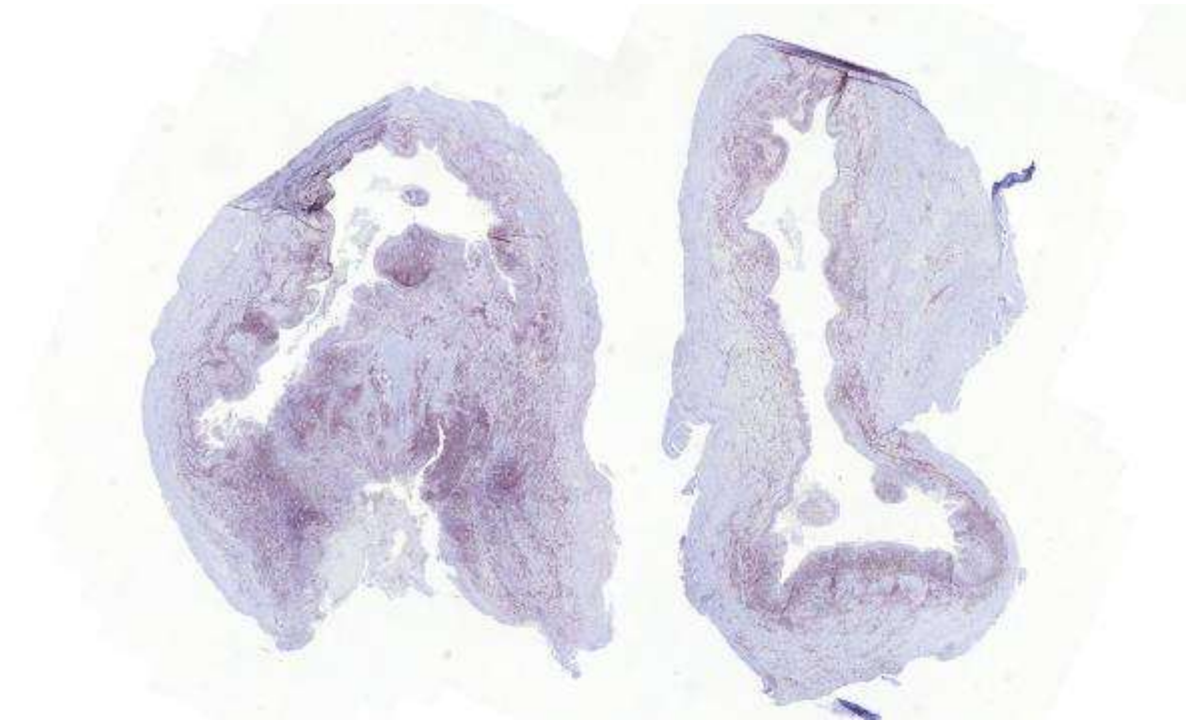

K25 – HE, CK17, bcl2 (x10)

Thin squamous (with superficial (↑↑) and/or basal (↑) CK17 staining) and thicker epithelium with mucous component negative for CK17 (bottom)

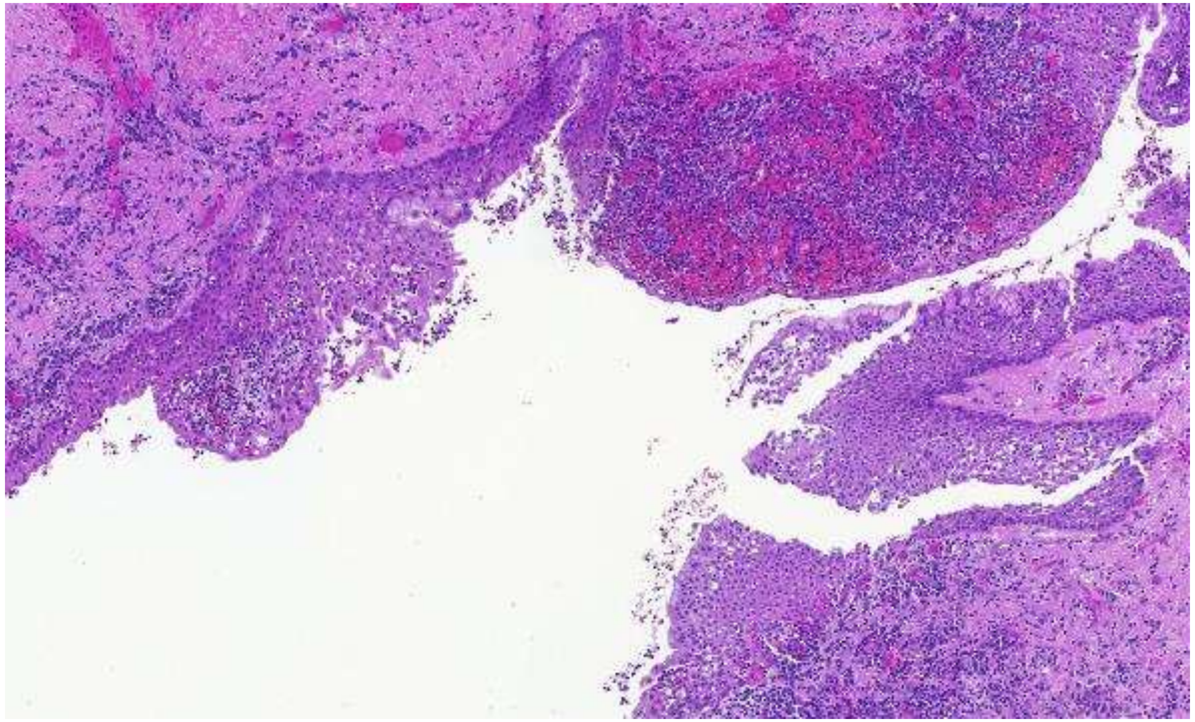

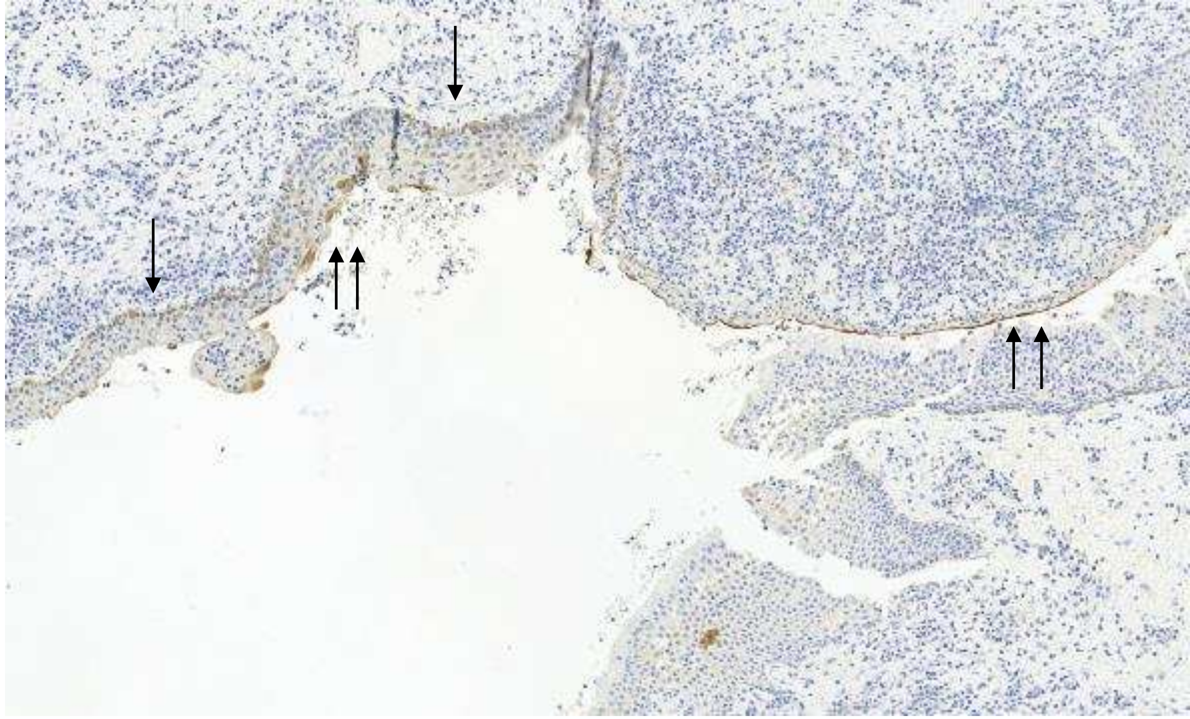

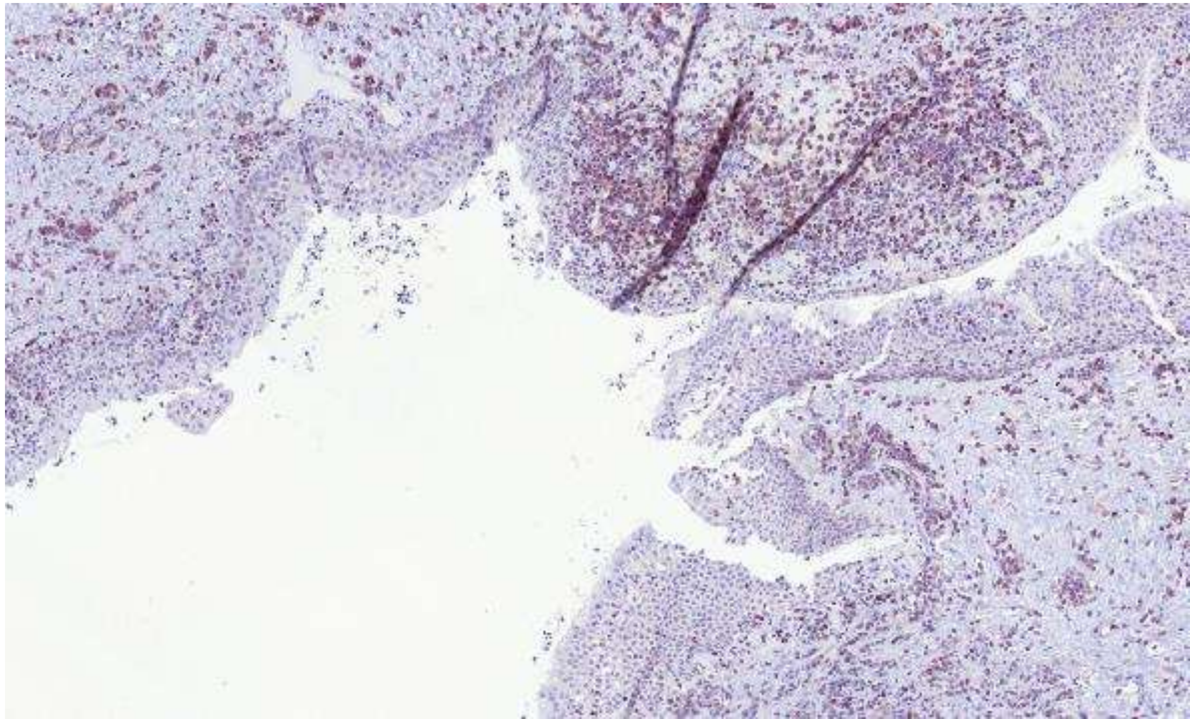

K25 – HE, CK17, bcl2 (x20)

Squamous (with focal superficial (↑) CK17 staining) and ciliated epithelium (↑↑) negative for CK17

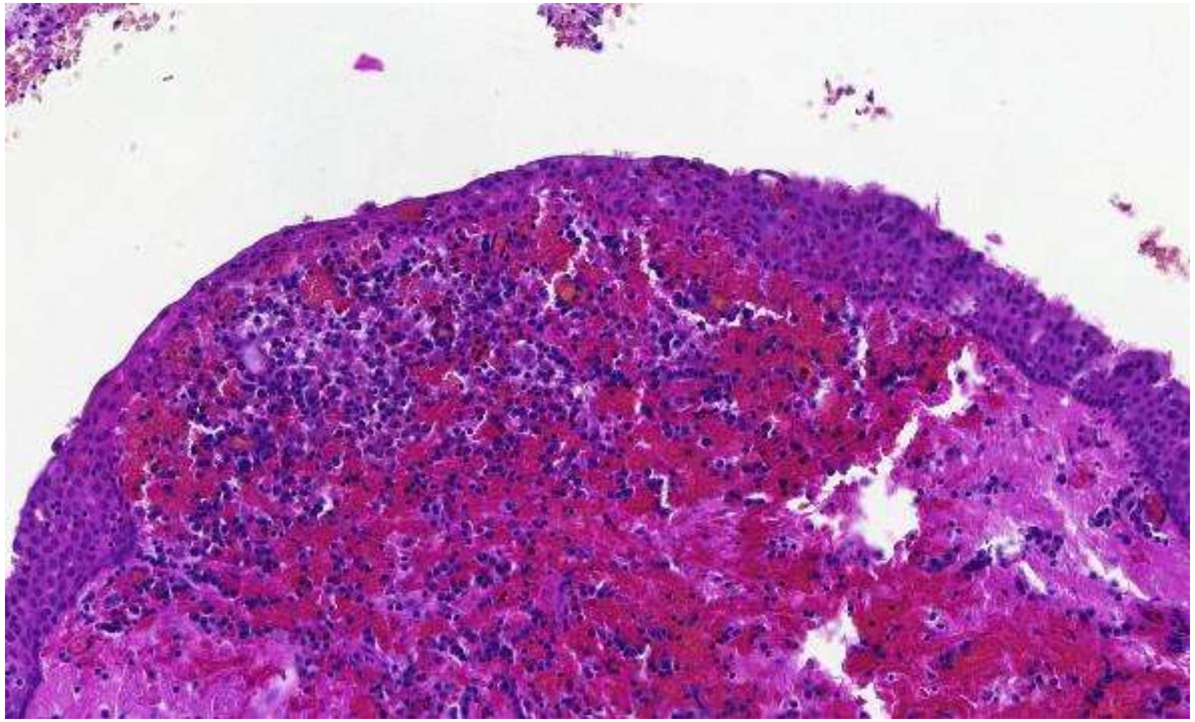

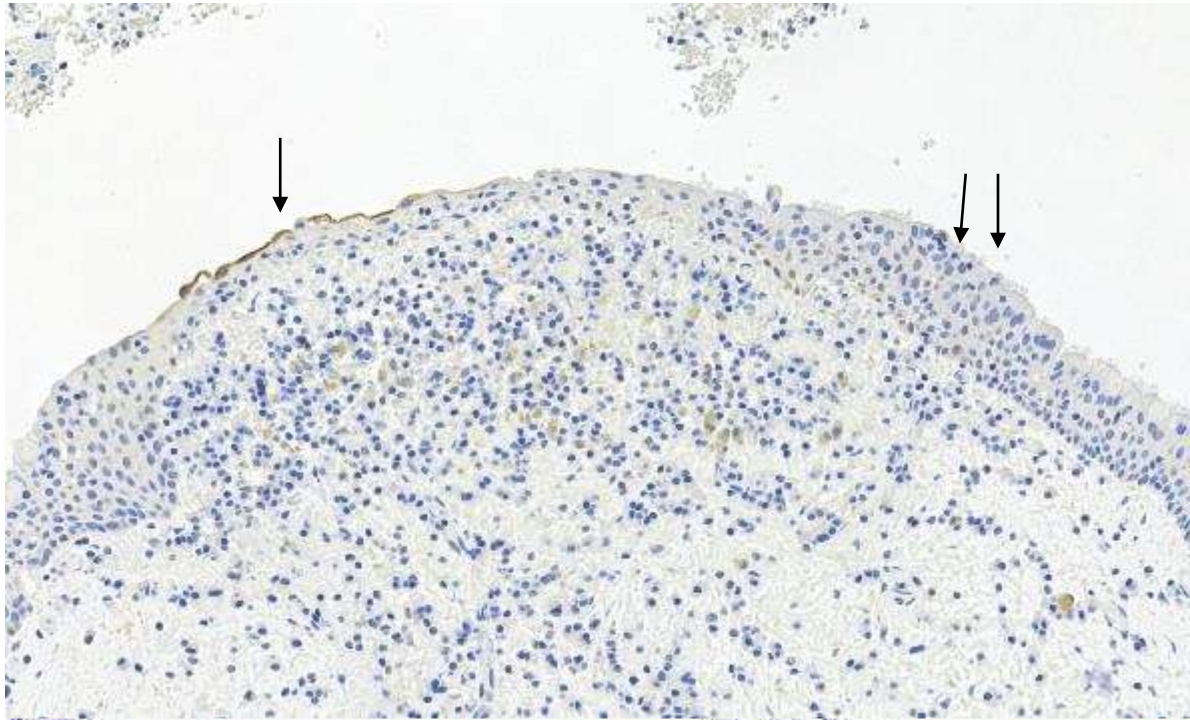

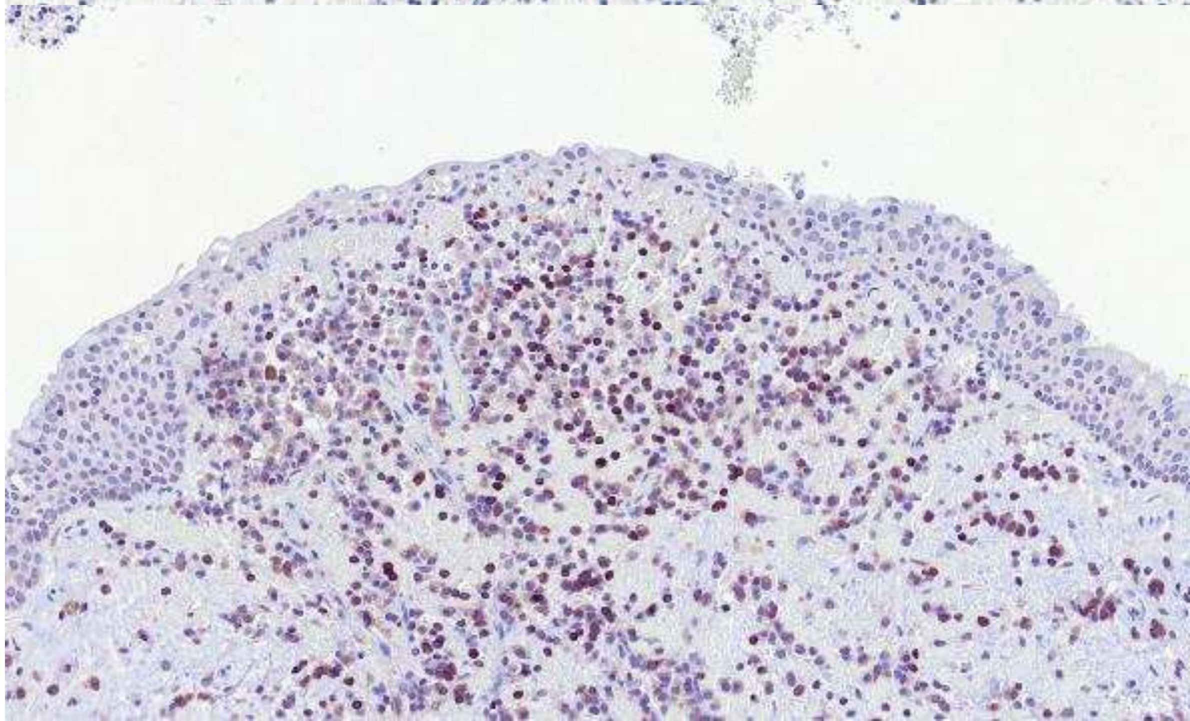

K25 – Conclusion

- H&E: Thin squamous epithelium with focal thickening and mucinous and ciliated metaplasia, no parakeratosis, no wavy surface, no basal palisading, uneven epithelial-subepithelial contours
- Focally mixed, dominantly chronic inflammation of the cyst wall
- Bcl2: negative
- CK17: superficial and/or basal staining
- Histopathology: inflammatory cyst (N.O.S.)
- Combined clinical and pathological opinion: radicular cyst

Case K26

- 47-year-old male (same as K24 and K25)
- Dental hygiene: poor, partly missing, dominantly untreated, partly treated teeth, teeth 11, 12, 15, 16, 21, 22, 23, 26, 32, 33 without crowns, roots in the alveoli; bridge over 31-43.
- Radiology: 7 mm large unilocular radiolucent lesion at the residual root of tooth 16 or 17.
- Operation: Removal of roots of teeth 11, 12, and 15, 16 and the cysts; opening of the maxillary sinus - modified Caldwell-Luc operation
- Clinical diagnosis: „maxillary cyst” (periapical)

K26 – OPT

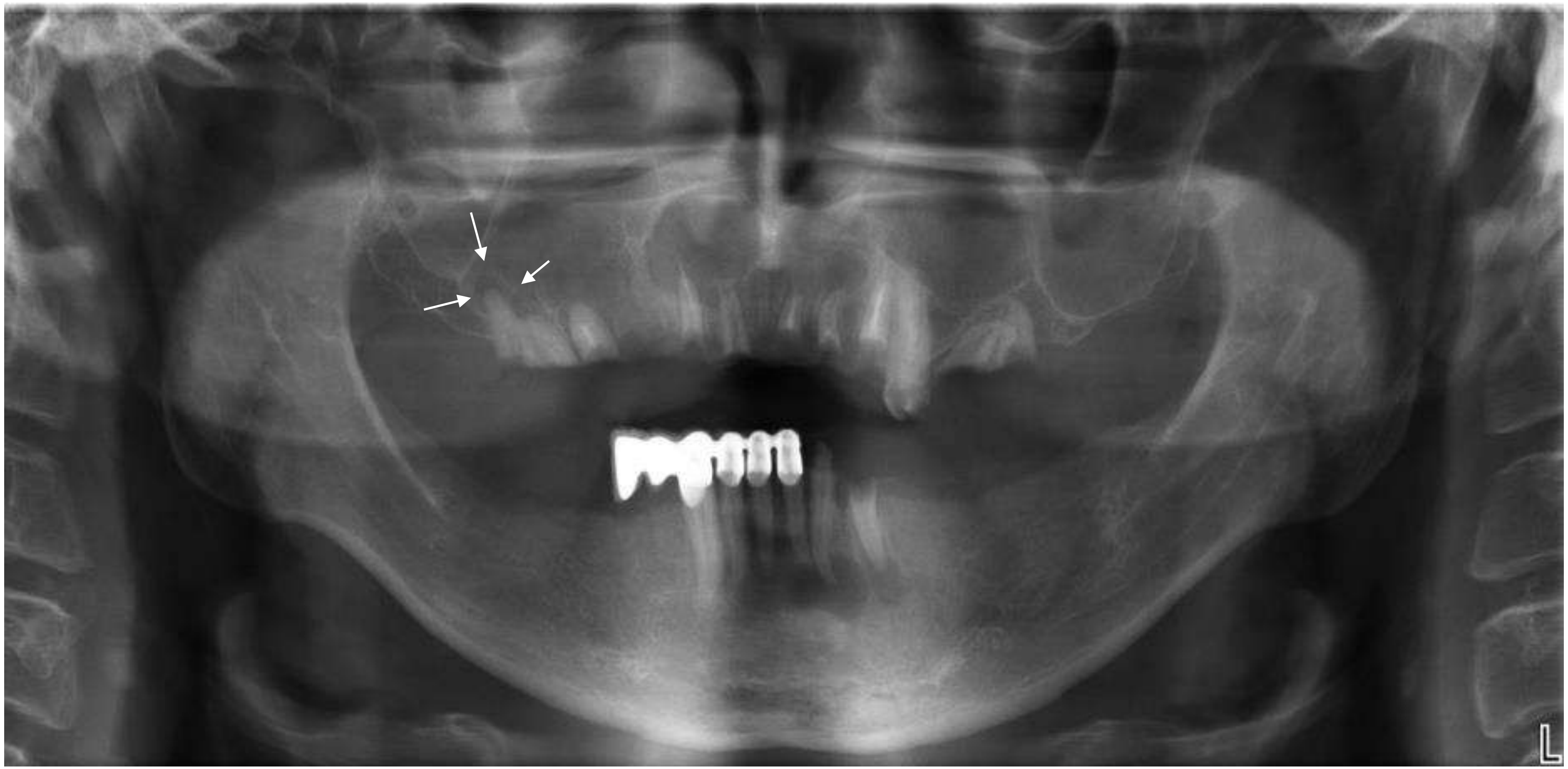

K26 – HE, CK17, bcl2 (x1.5)

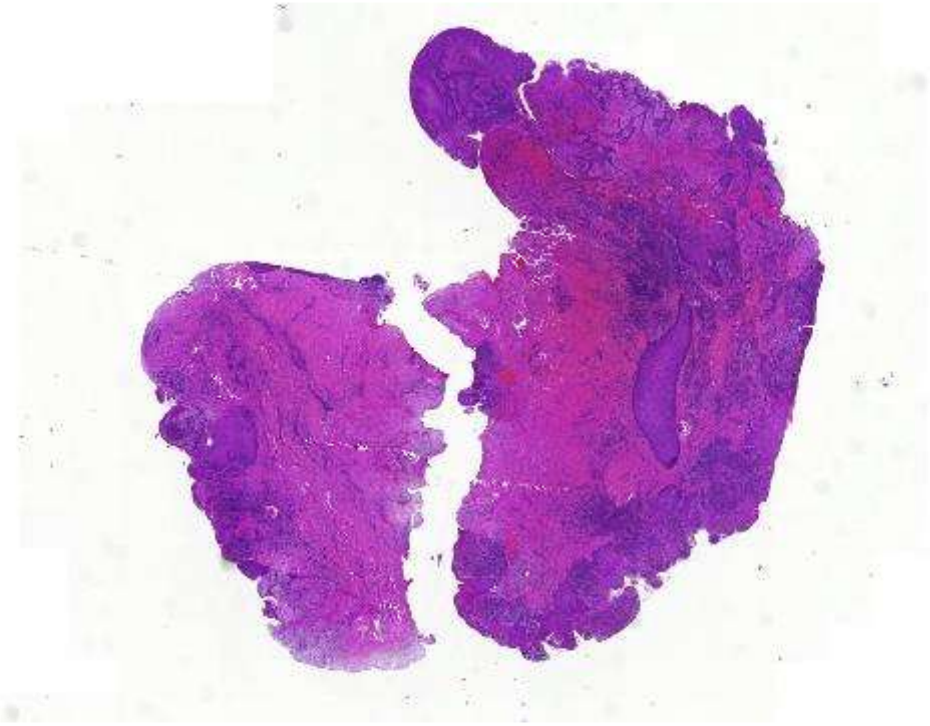

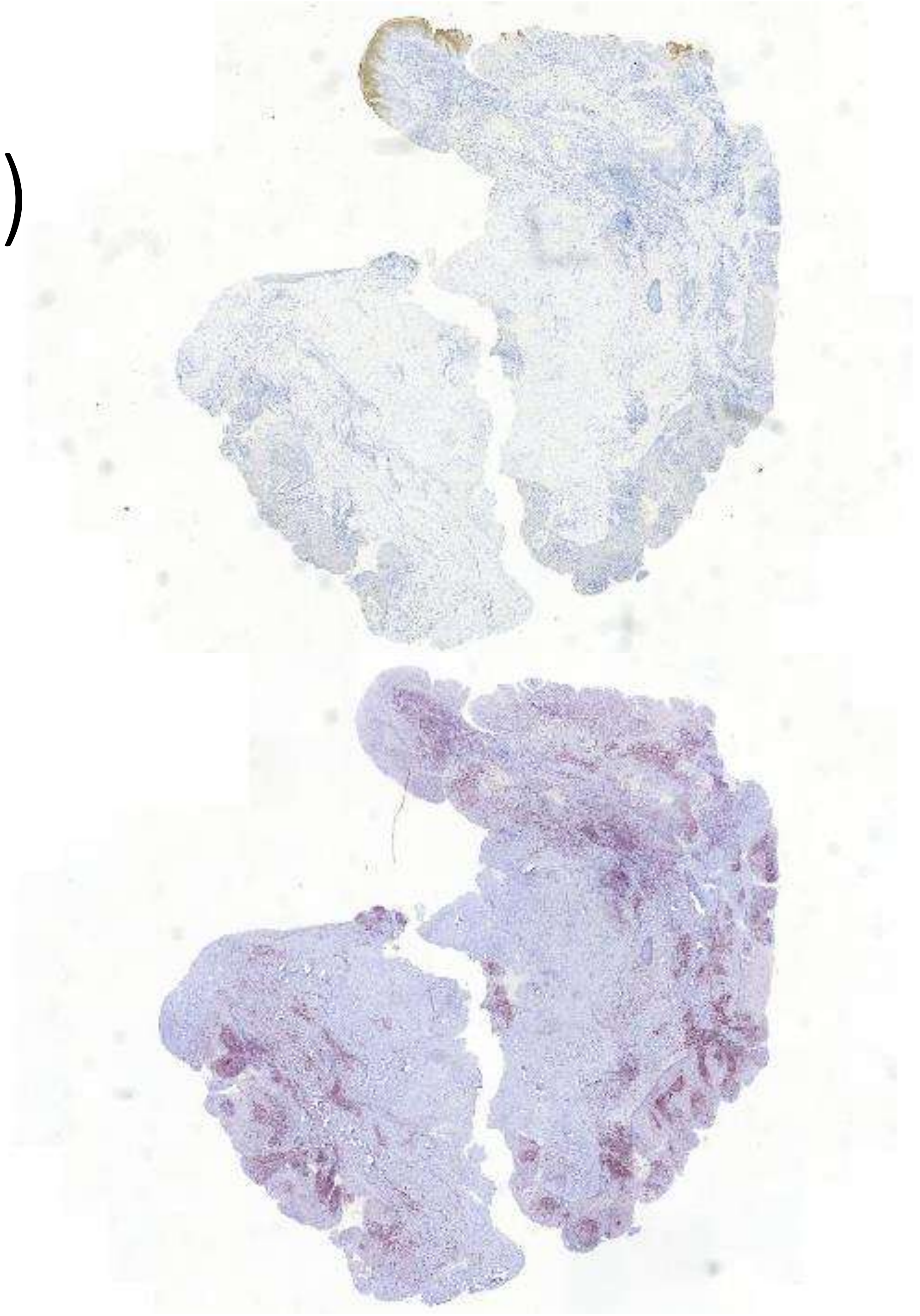

K26 – HE, CK17, bcl2 (x8)

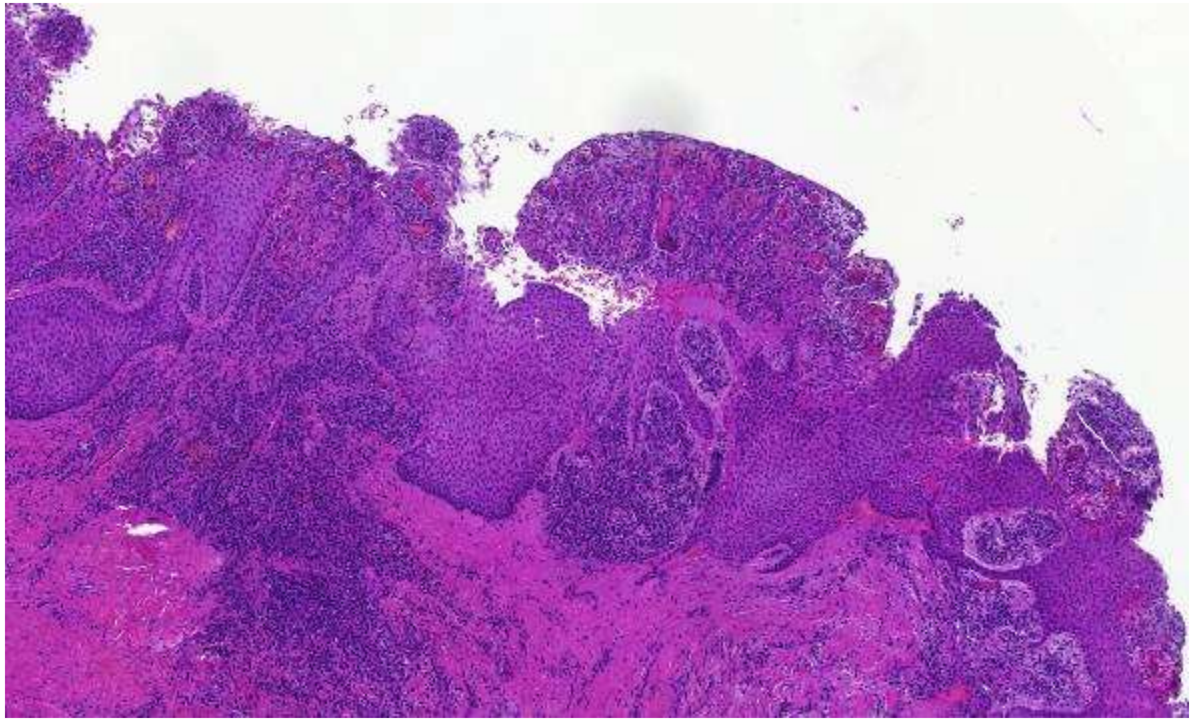

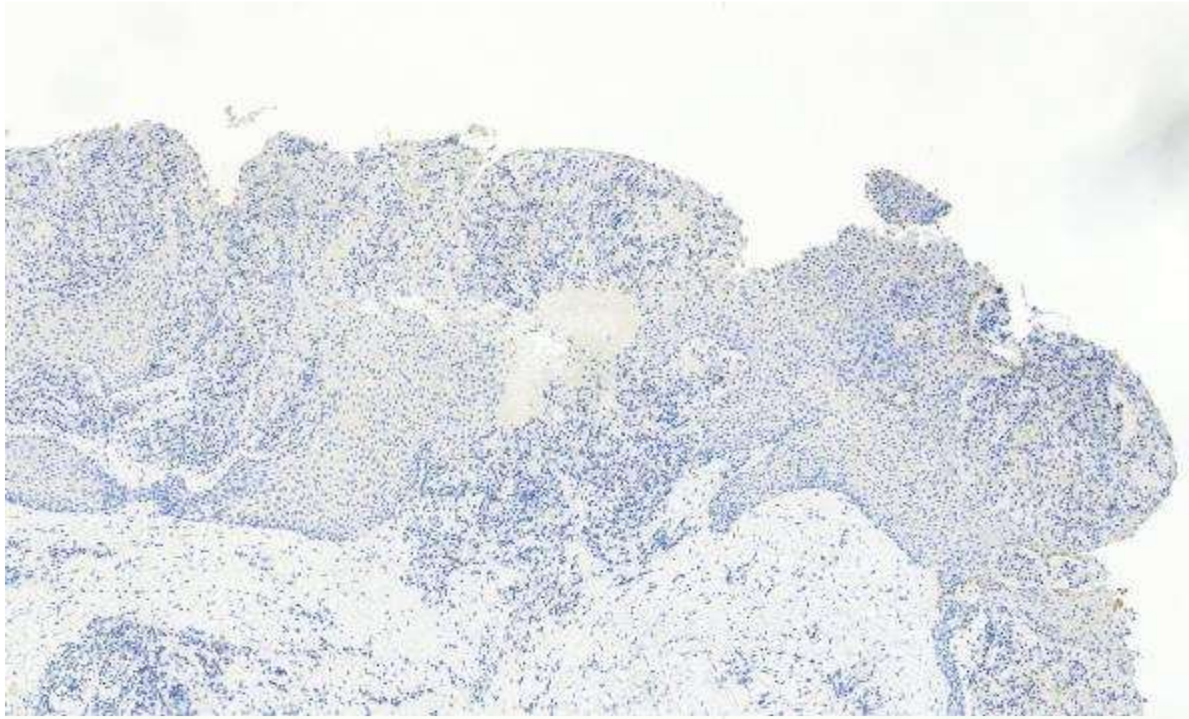

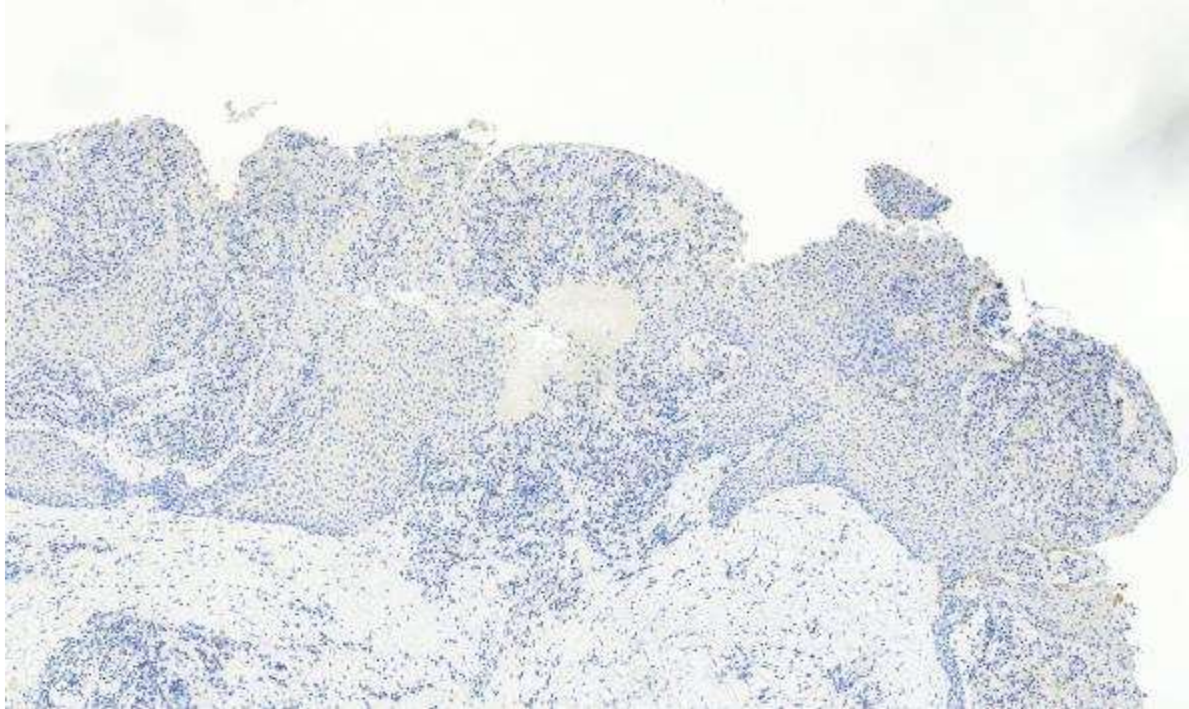

K26 – CK17 few superficial cells with weak staining (x10)

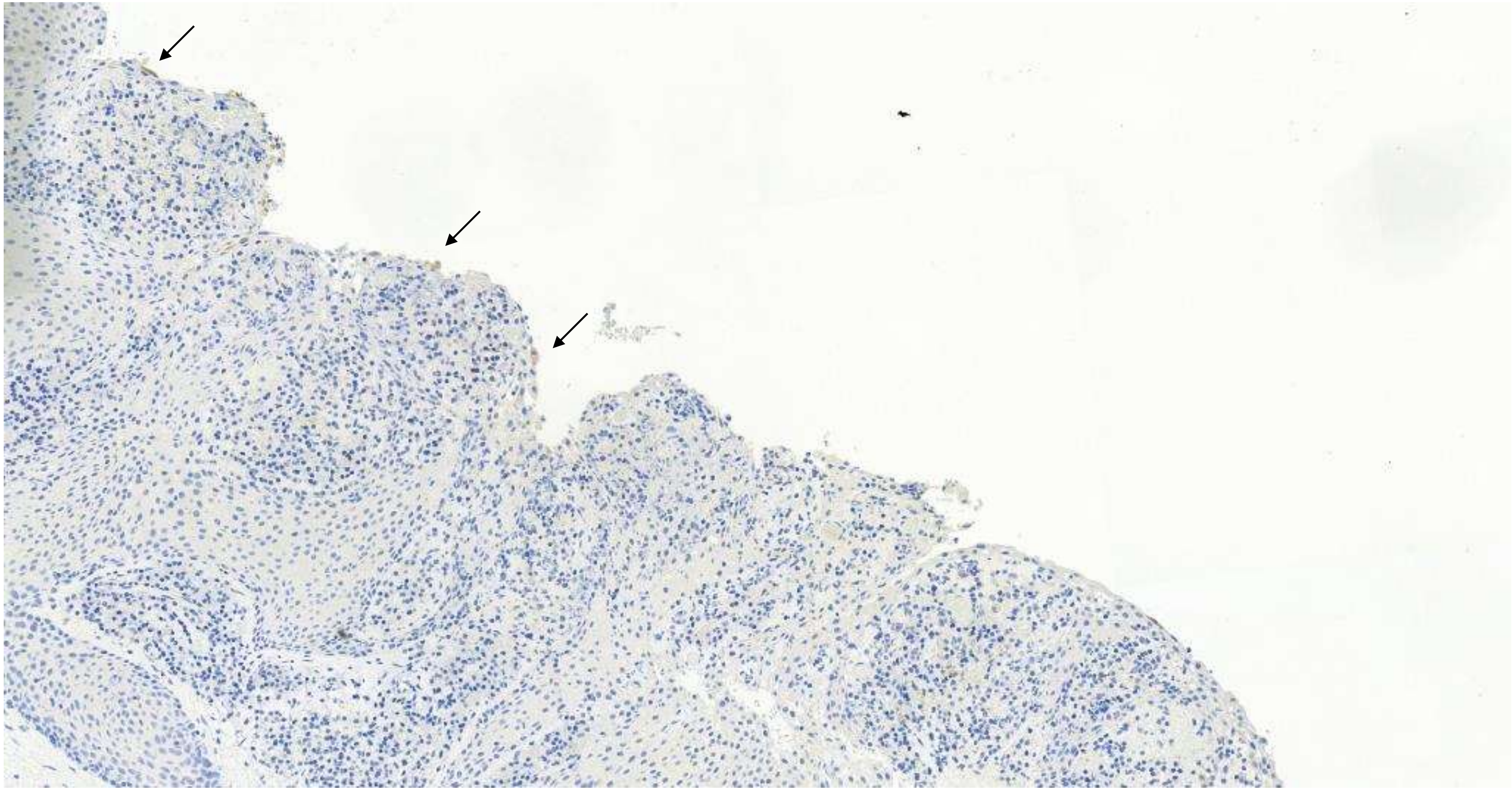

K26 – HE, CK17, bcl2 (x10)

Transepithelial CK17 pattern

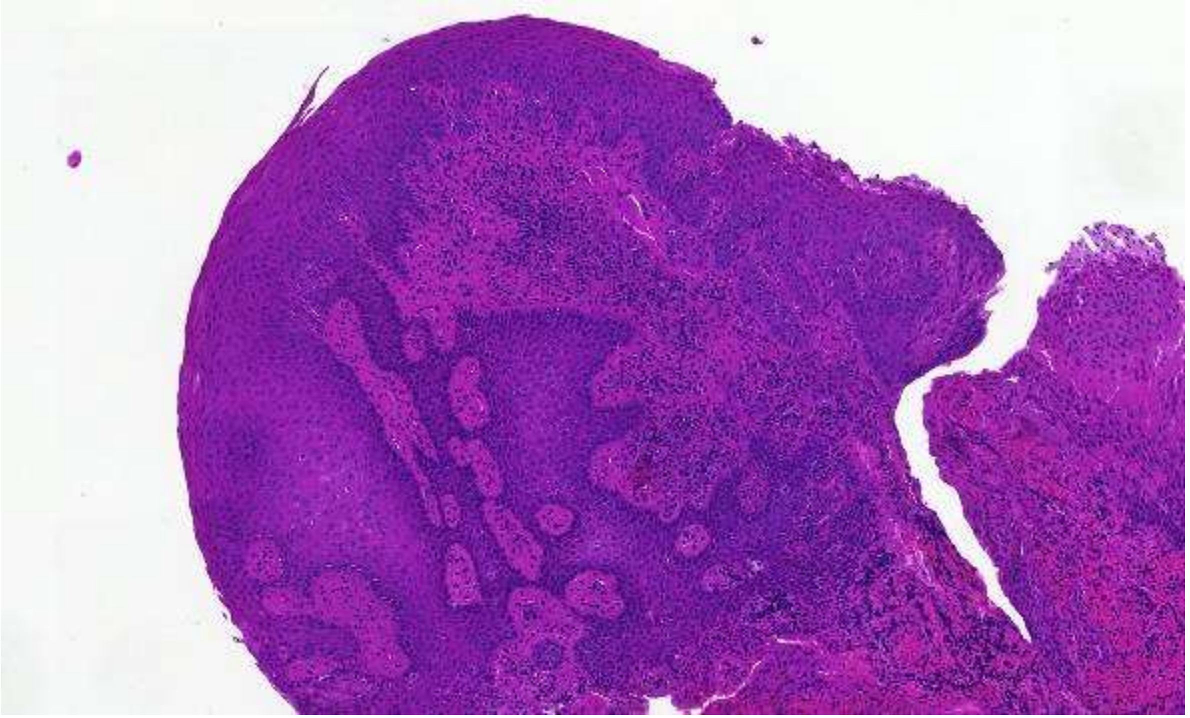

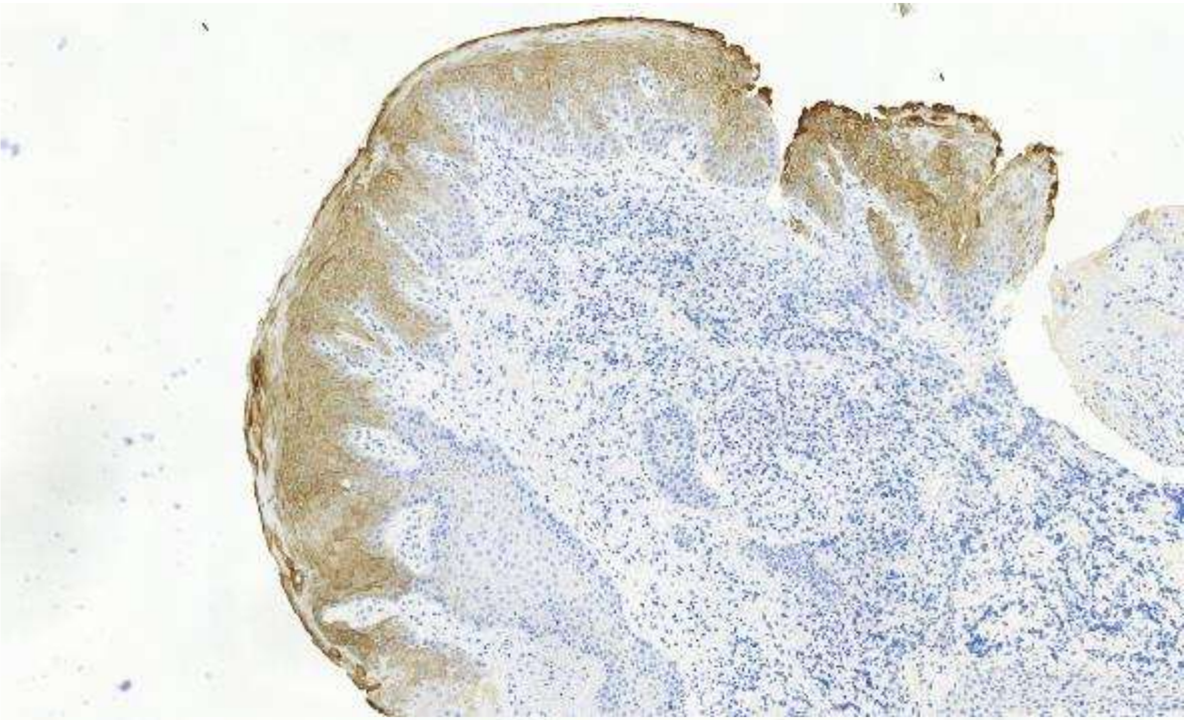

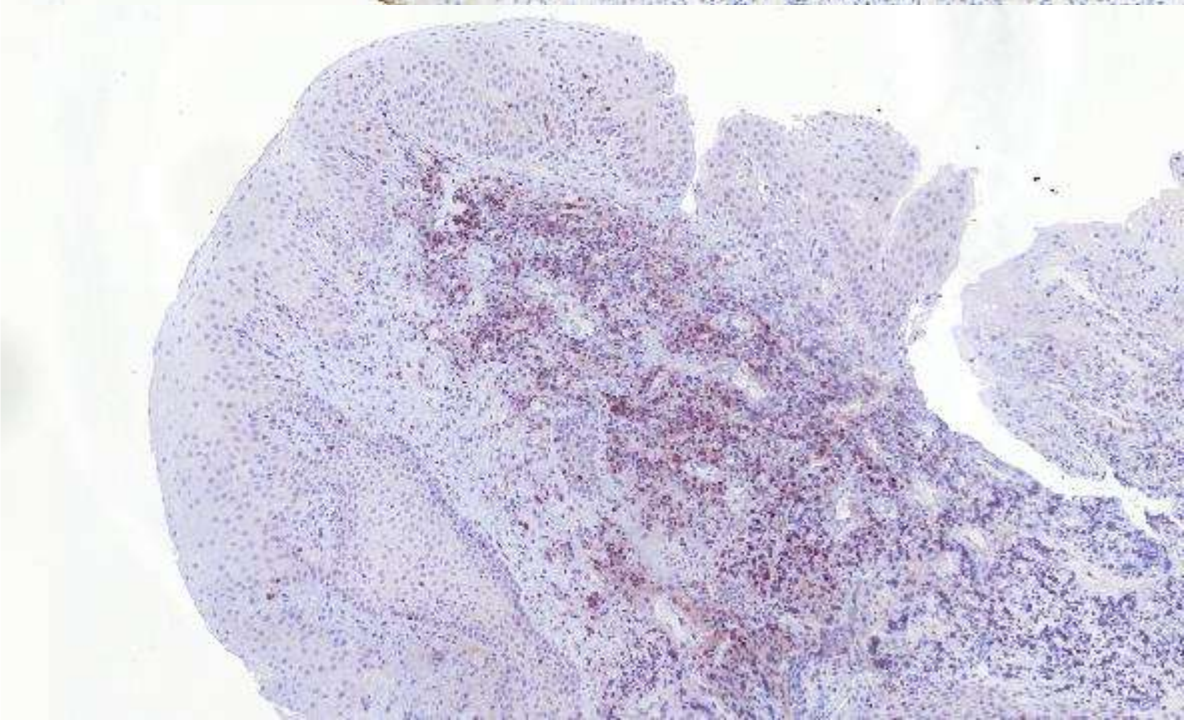

K26 – Conclusion

- H&E: Thick non-keratinizing squamous epithelium, no parakeratosis, no wavy surface, no basal palisading, uneven epithelial-subepithelial contours
- Mixed inflammatory infiltrate of the cyst wall
- Bcl2: negative
- CK17: negative, rare superficial cells staining, focal transepithelial staining with strong superficial, weaker spinous and areas of basal sparing
- Histopathology: inflammatory cyst (N.O.S.)
- Combined clinical and pathological opinion: radicular cyst

Case K27

- 43-year-old female
- Dental hygiene/status: normal hygiene; maintained, treated teeth, impacted 3rd molars
- Radiology: 13 mm large unilocular radiolucent lesion at the root of tooth 22 with incomplete root filling.
- Operation: Resection of the apex of the root of tooth 22, removal of the cyst
- Clinical diagnosis: periapical cyst

K27 – OPT

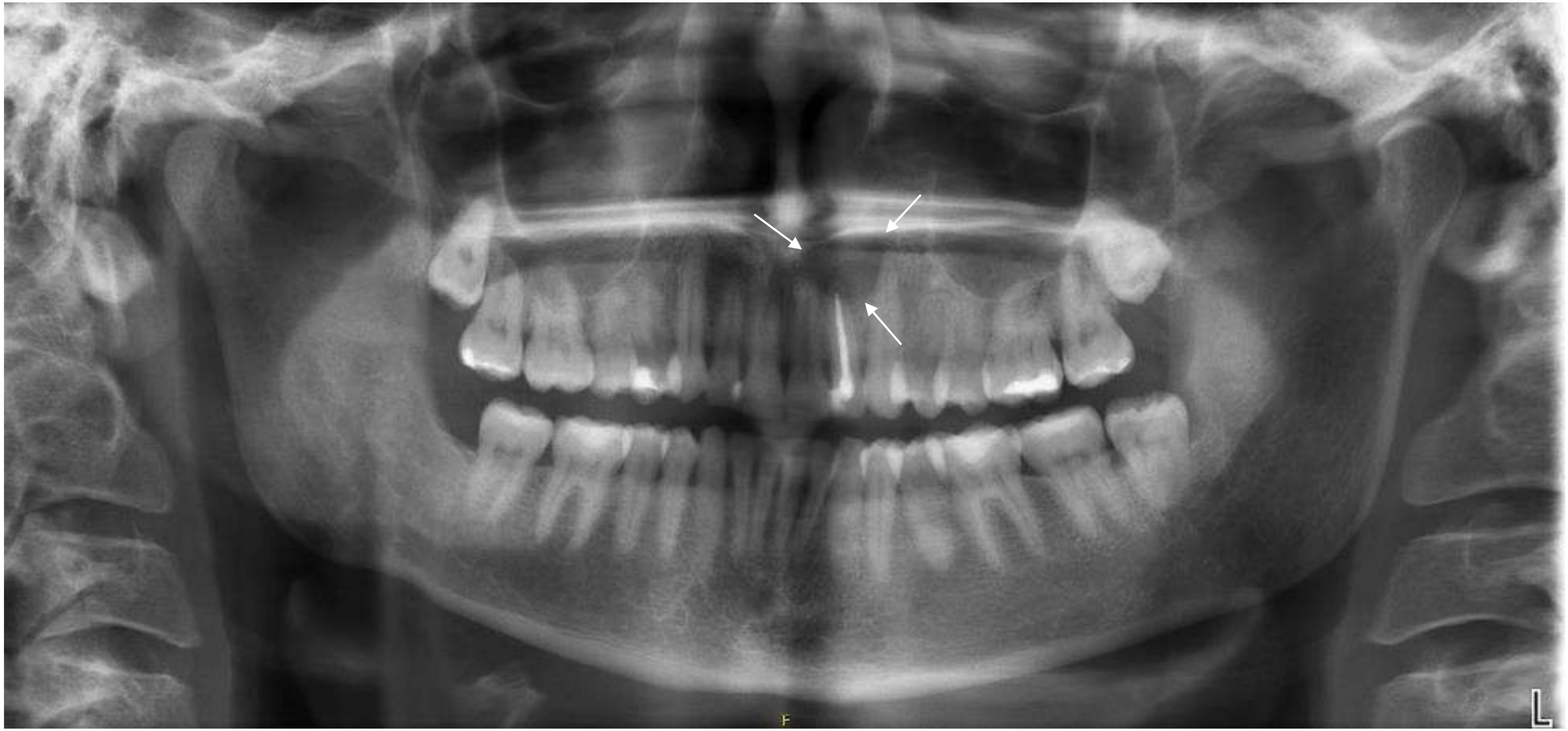

K27 – HE, CK17, bcl2 (x10)

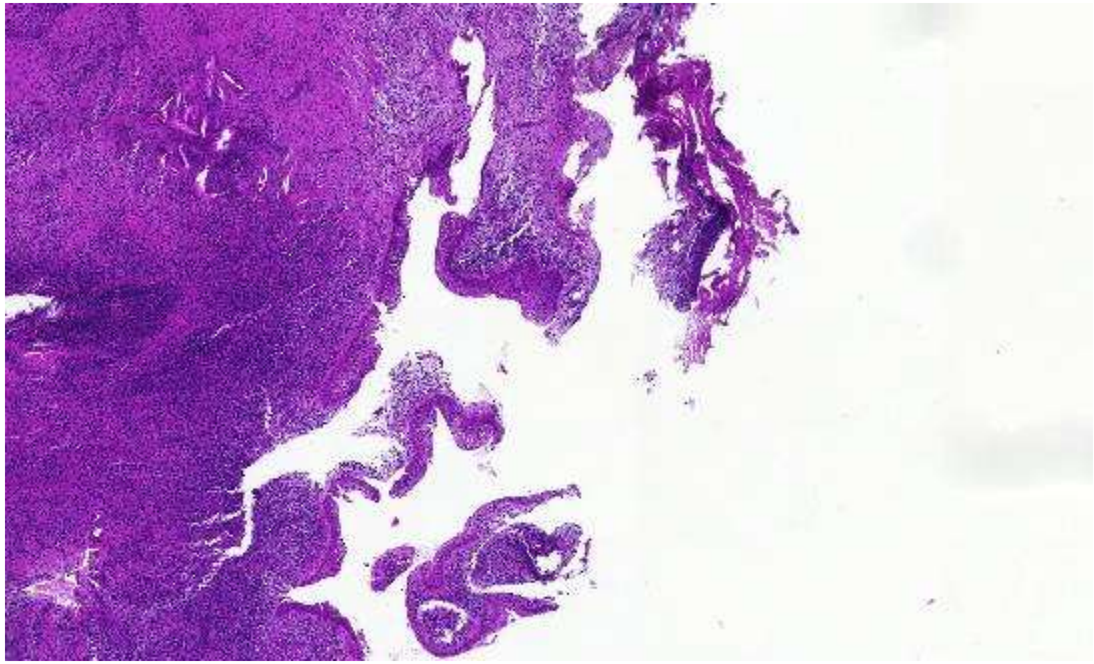

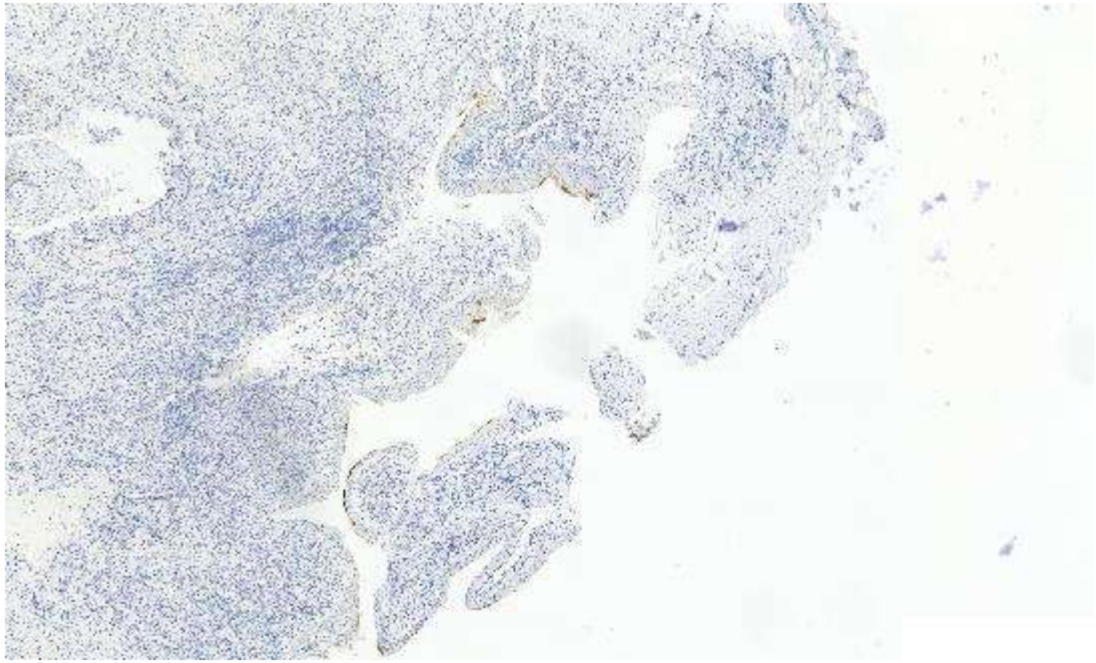

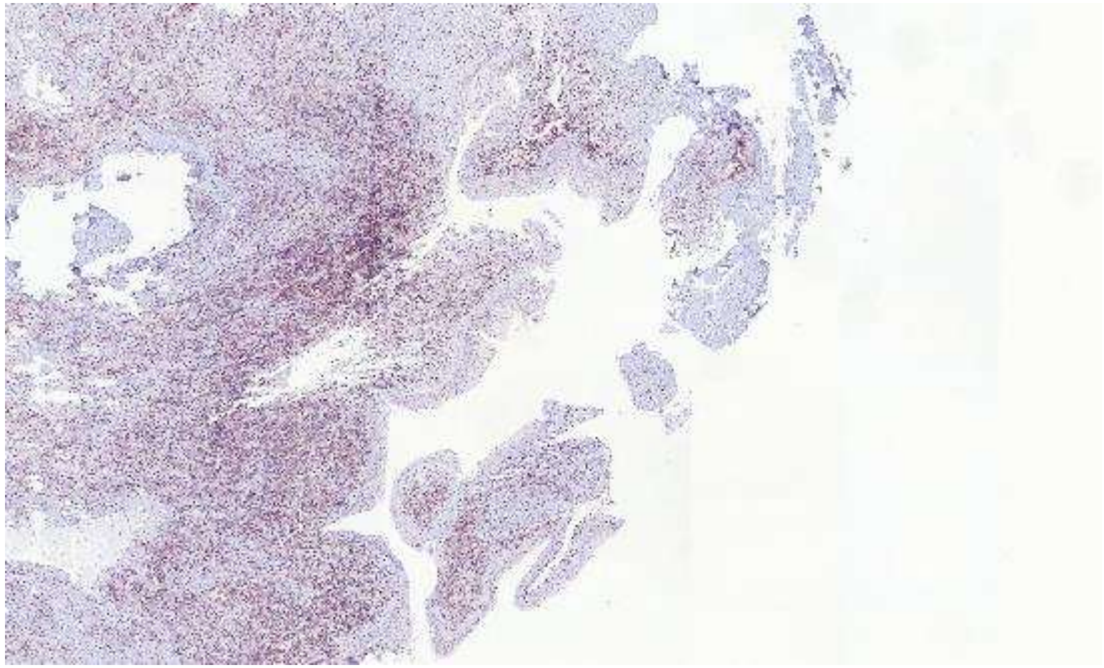

K27 – HE, CK17, bcl2 (x20)

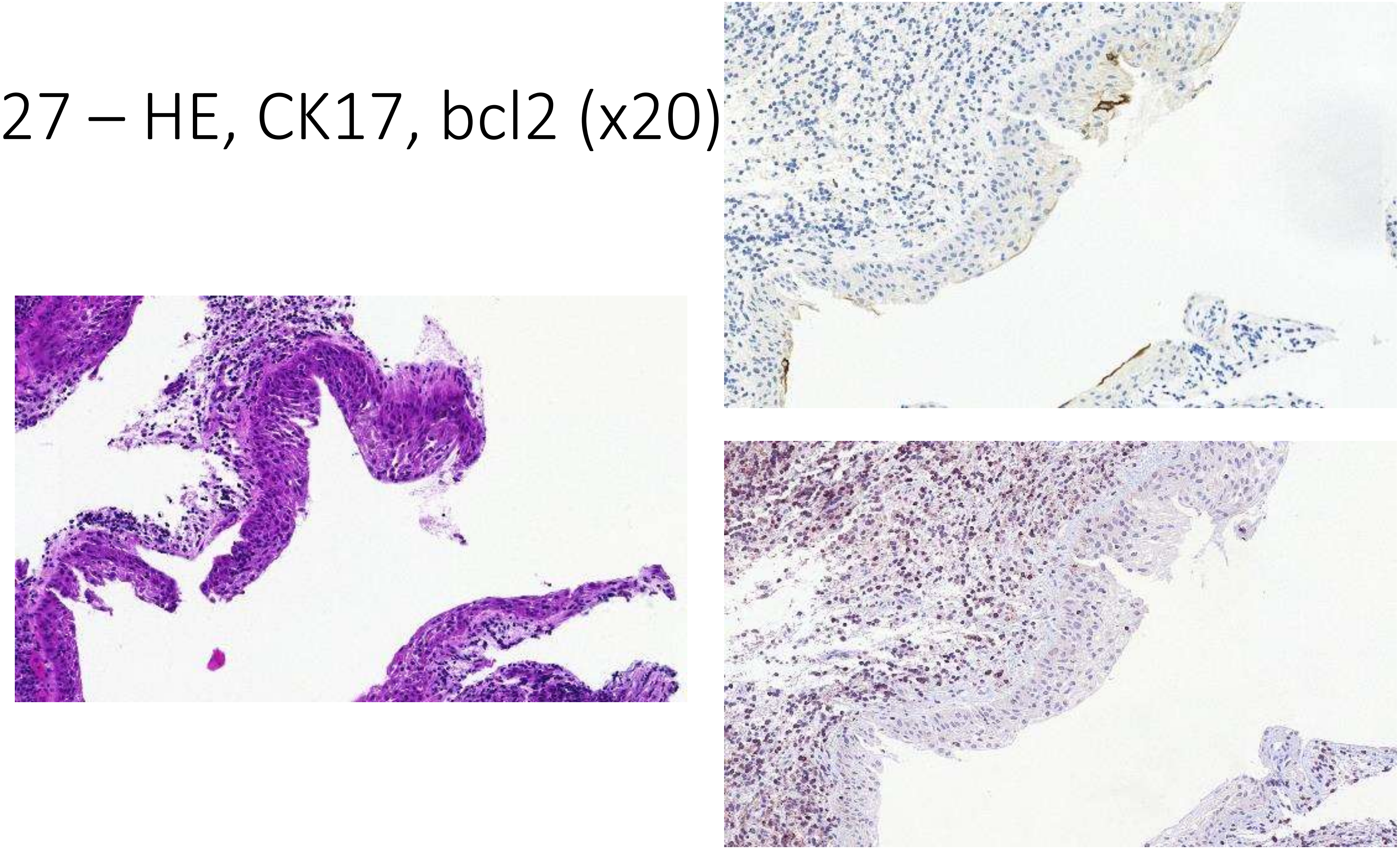

K27 – Conclusion

- H&E: relatively thick (focally thin) squamous epithelium, no parakeratosis, no wavy surface, no basal palisading, even to uneven epithelial-subepithelial contours
- Moderate to marked chronic inflammation of the cyst wall with cholesterol clefts in some areas
- Bcl2: negative
- CK17: focal superficial staining
- Histopathology: inflammatory (radicular) cyst
- Combined clinical and pathological opinion: radicular cyst

Case K28

- 56-year-old male
- Dental hygiene: missing teeth replaced by movable denture
- Radiology: 21 mm large cyst around the crown of the retained and misplaced tooth 13
- Operation: Removal of tooth 13 and the cyst around it
- Clinical diagnosis: maxillary cyst N.O.S. on the histopathology request form; dentigerous (follicular) cyst mentioned in previous clinical charts

K28 – OPT

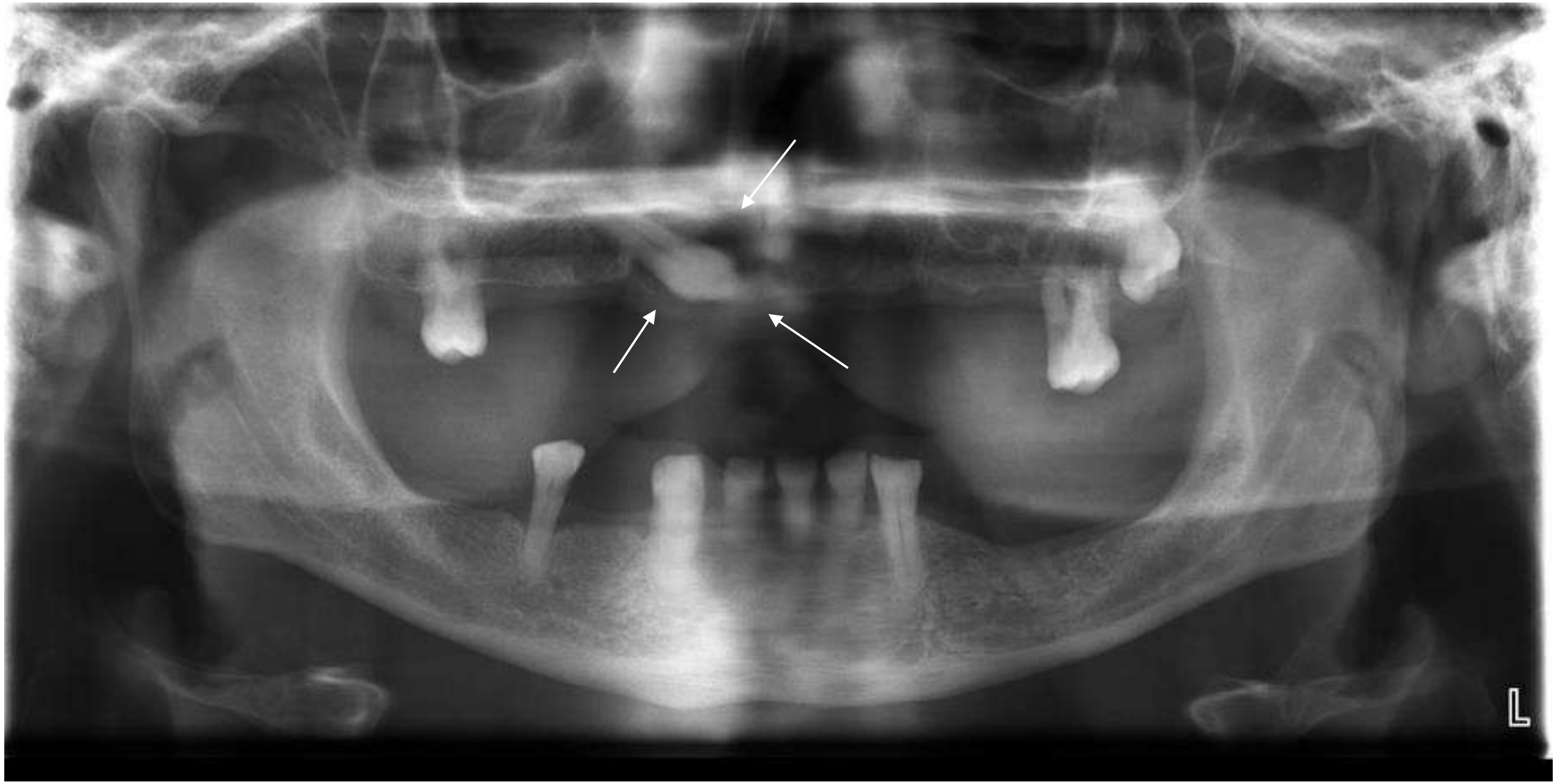

K28 – HE, CK17, bcl2 (x10)

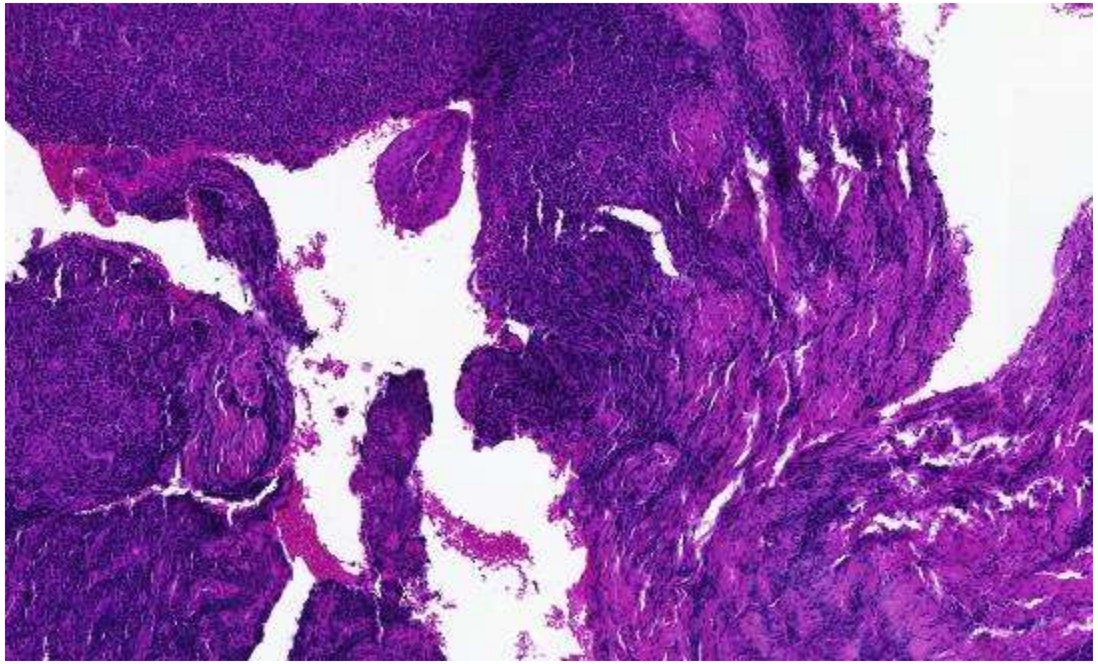

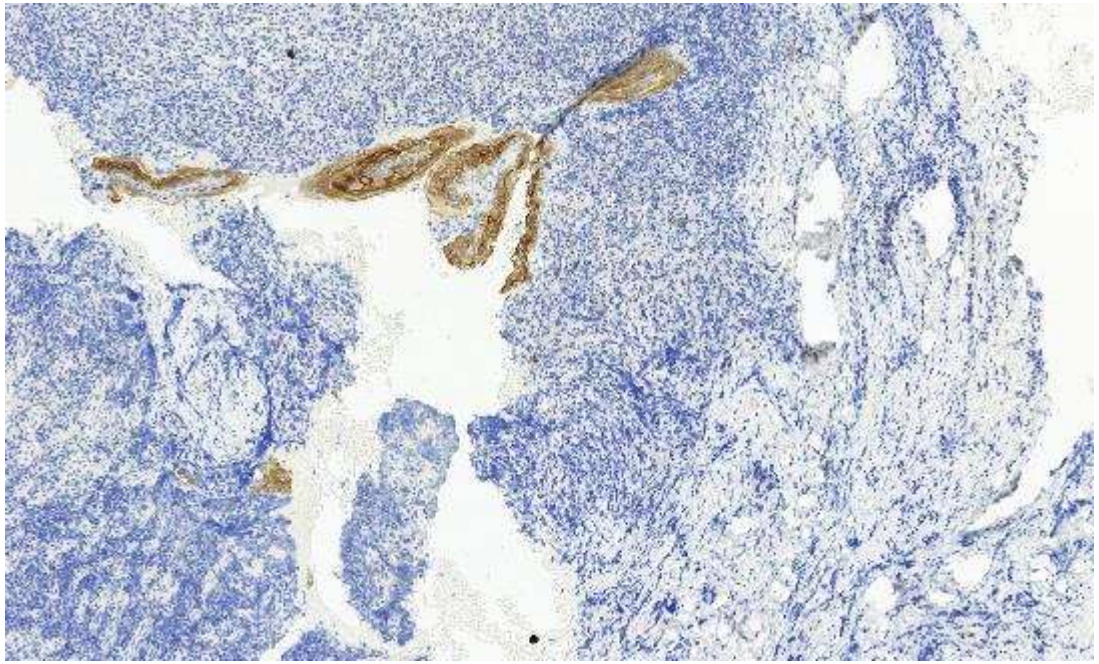

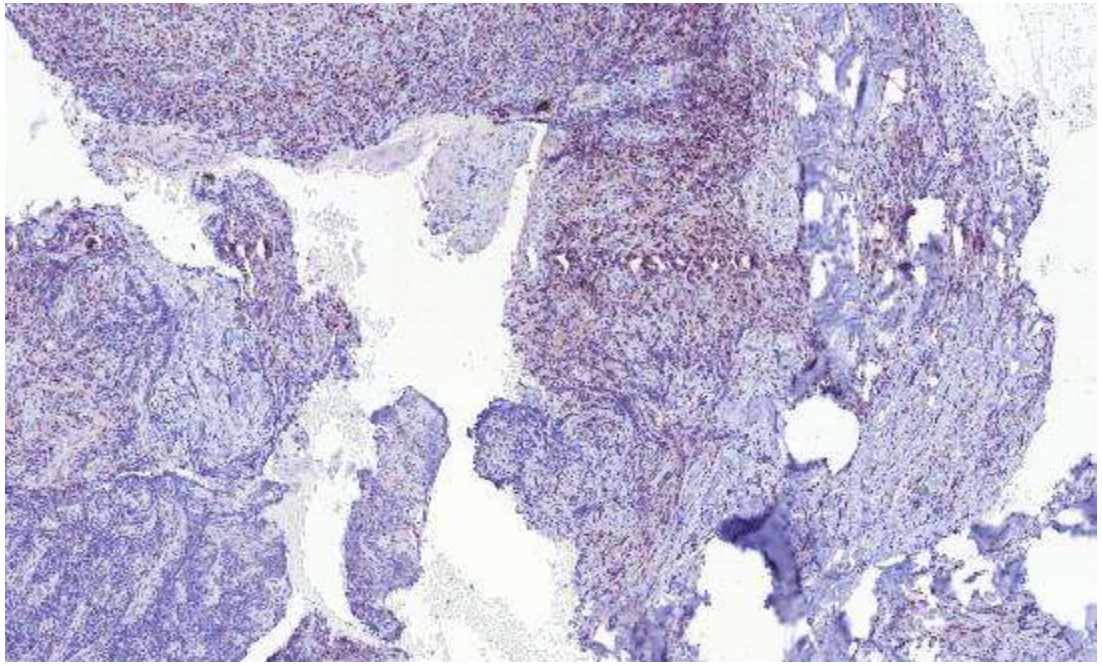

K28 – HE, CK17, bcl2 (x20)

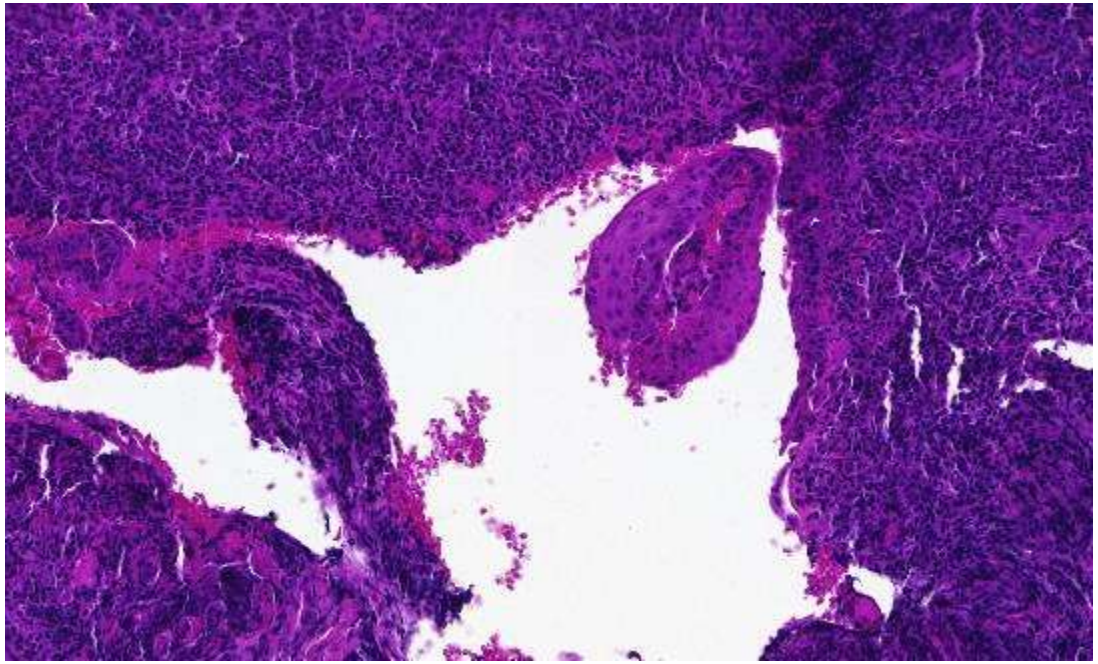

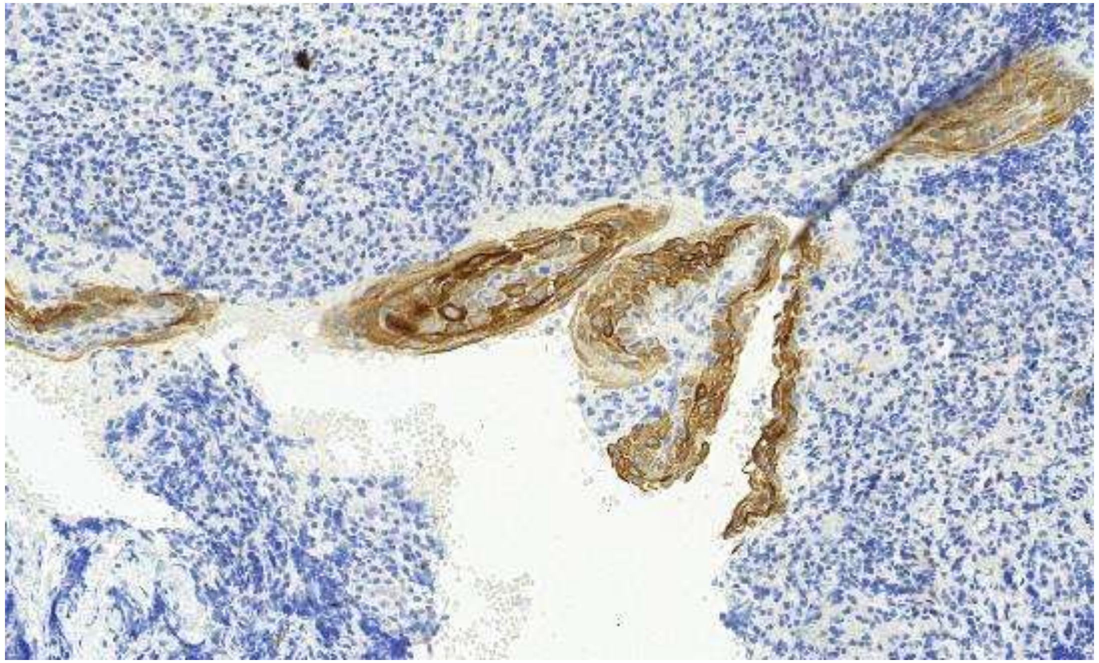

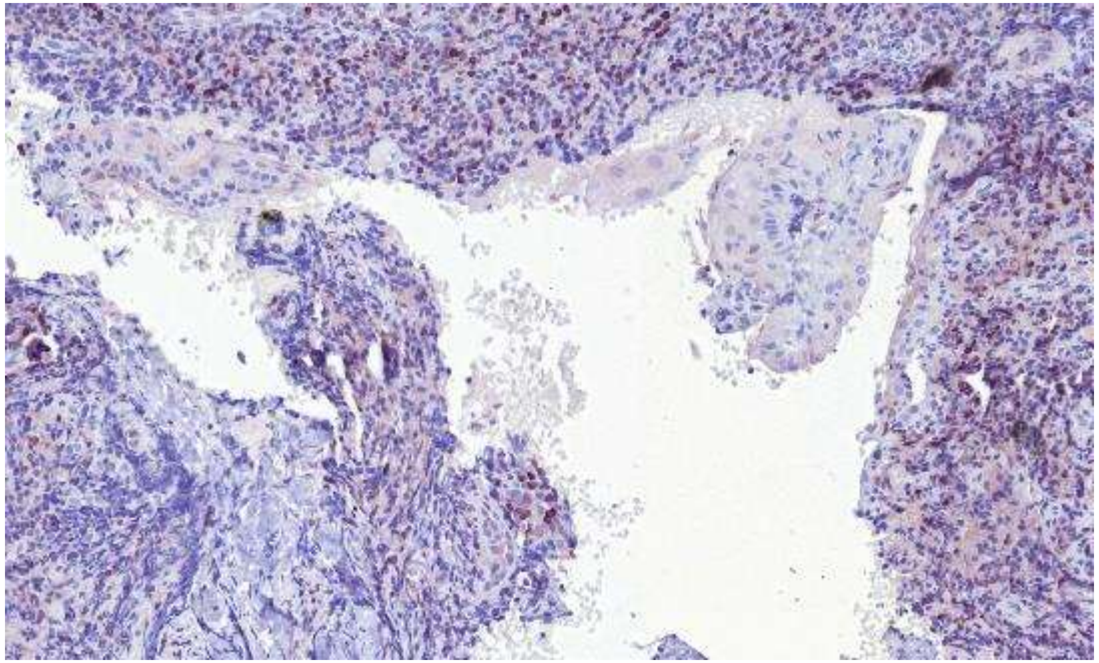

K28 – HE, CK17, bcl2 (x20)

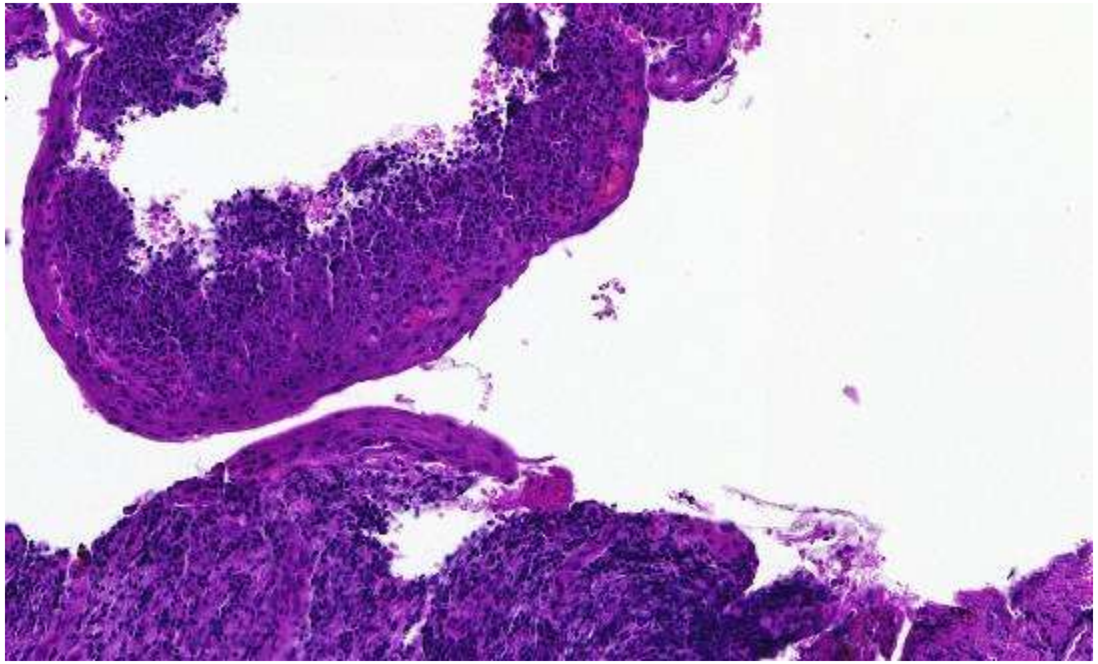

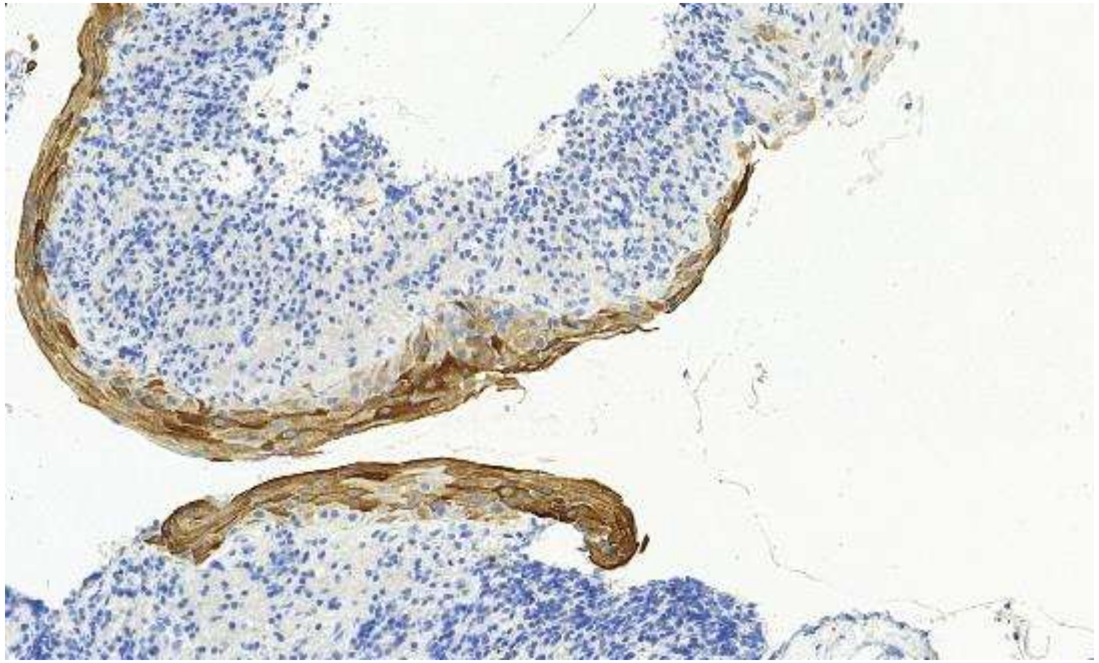

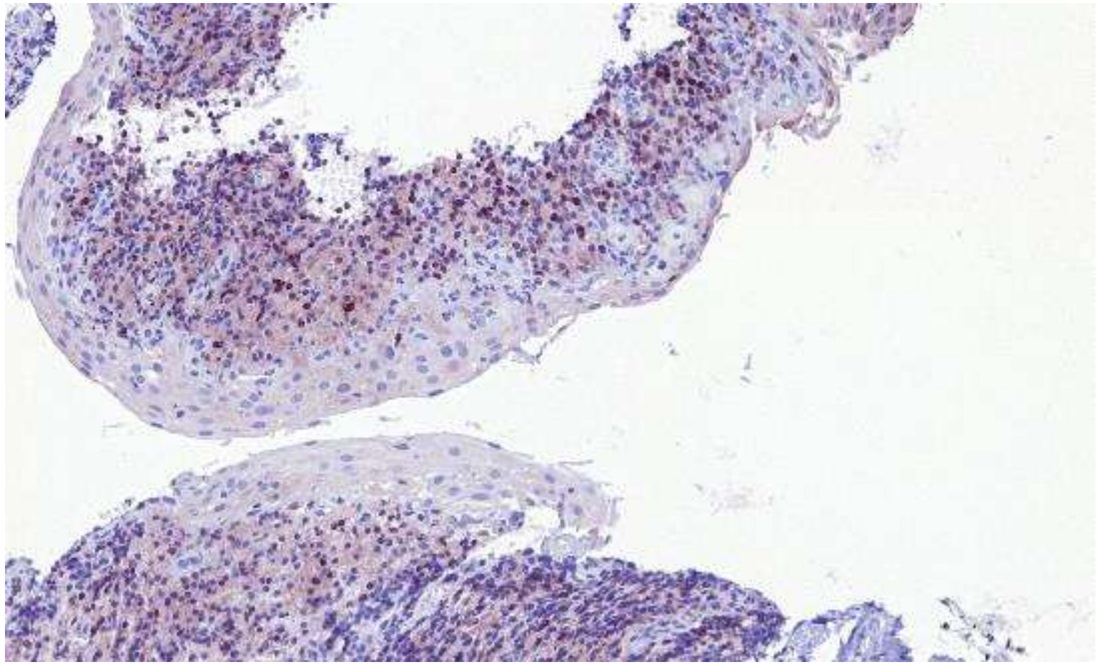

K28 – Conclusion

- H&E: relatively thin non-keratinizing squamous epithelium, no parakeratosis, no wavy surface, no basal palisading, even epithelial-subepithelial contours
- Prominent mixed inflammation of the cyst wall
- Bcl2: negative
- CK17: transepithelial
- Histopathology: inflamed keratocyst (wrong wording)
- Combined clinical and pathological opinion: dentigerous (follicular) cyst with secondary inflammation

Case K29

- 60-year-old male
- Dental hygiene: partly maintained, untreated teeth; a mass palpable on the vestibular mucosa between teeth 11-22, pus can be expressed
- Radiology: 2-3 cm large cyst at the apex of the residual root of tooth 22
- Operation: Removal of the residual roots of teeth 21-23, along with the cyst
- Clinical diagnosis: periapical cyst

K29 – OPT

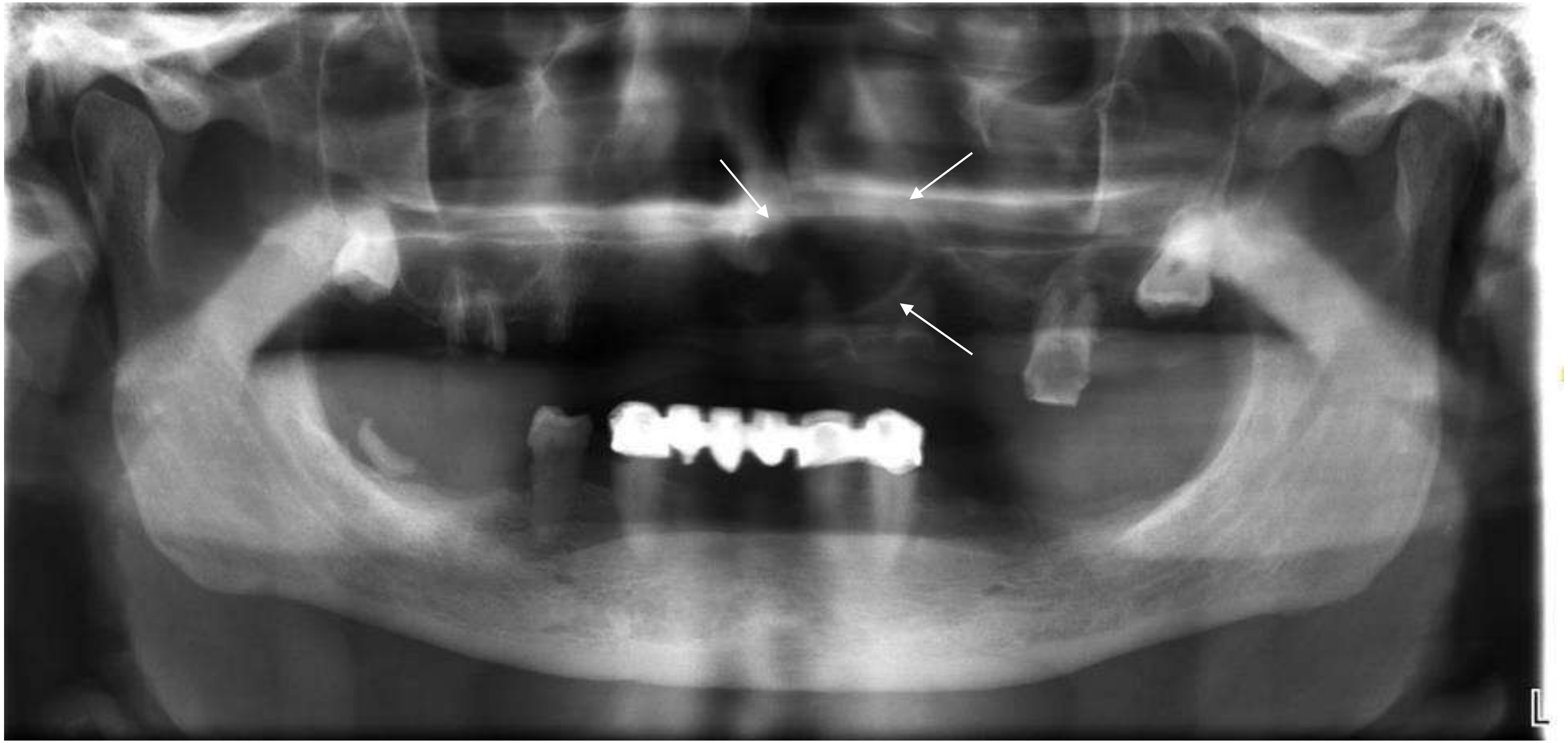

K29 – HE, CK17, bcl2 (x1)

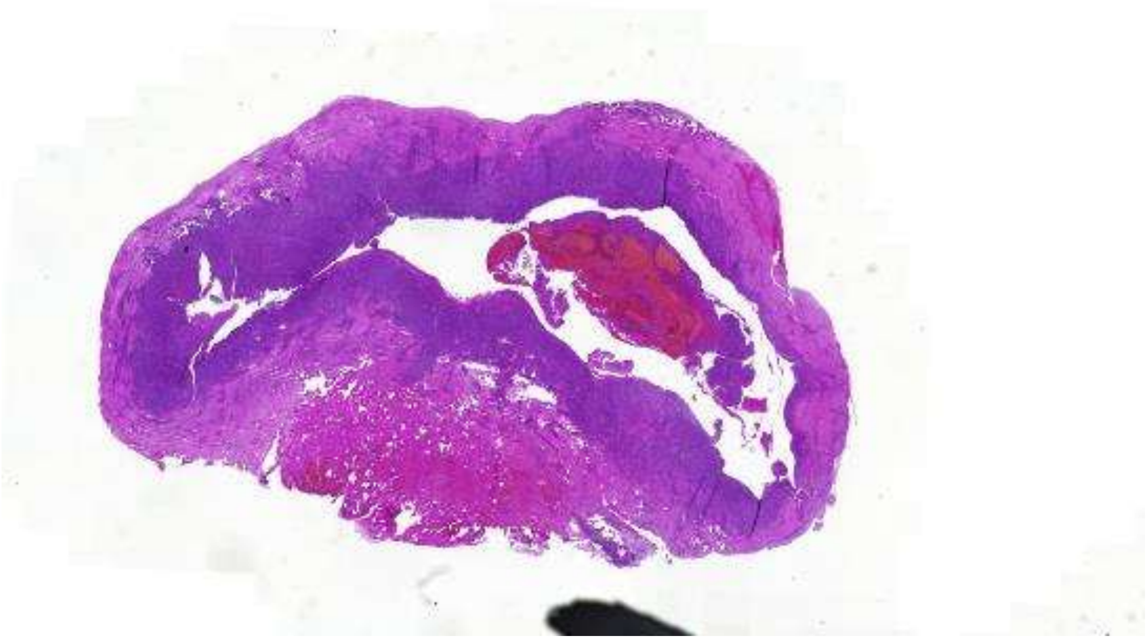

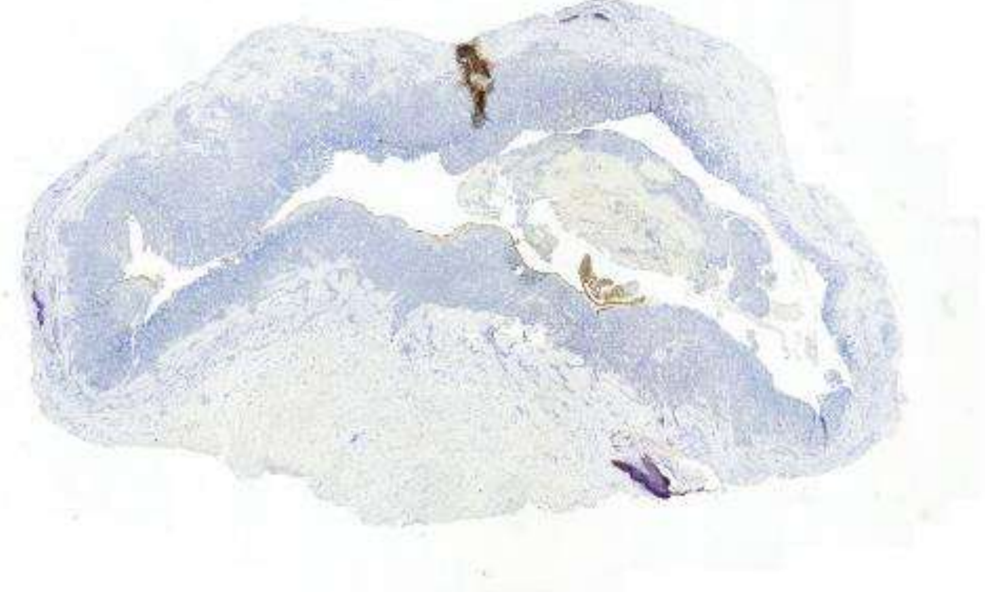

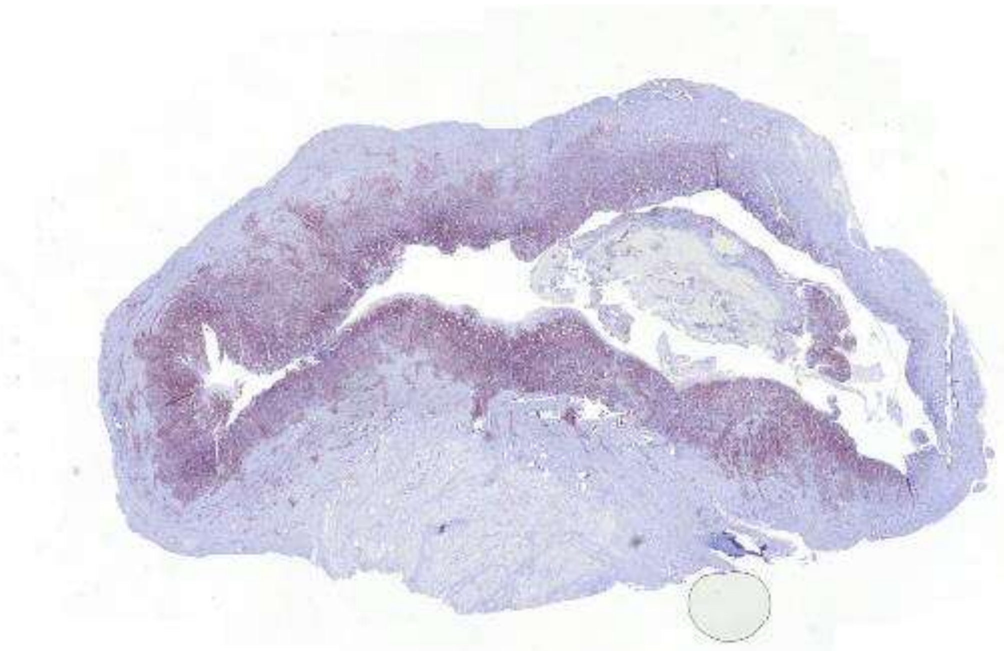

K29 – HE, CK17, bcl2 (x20)

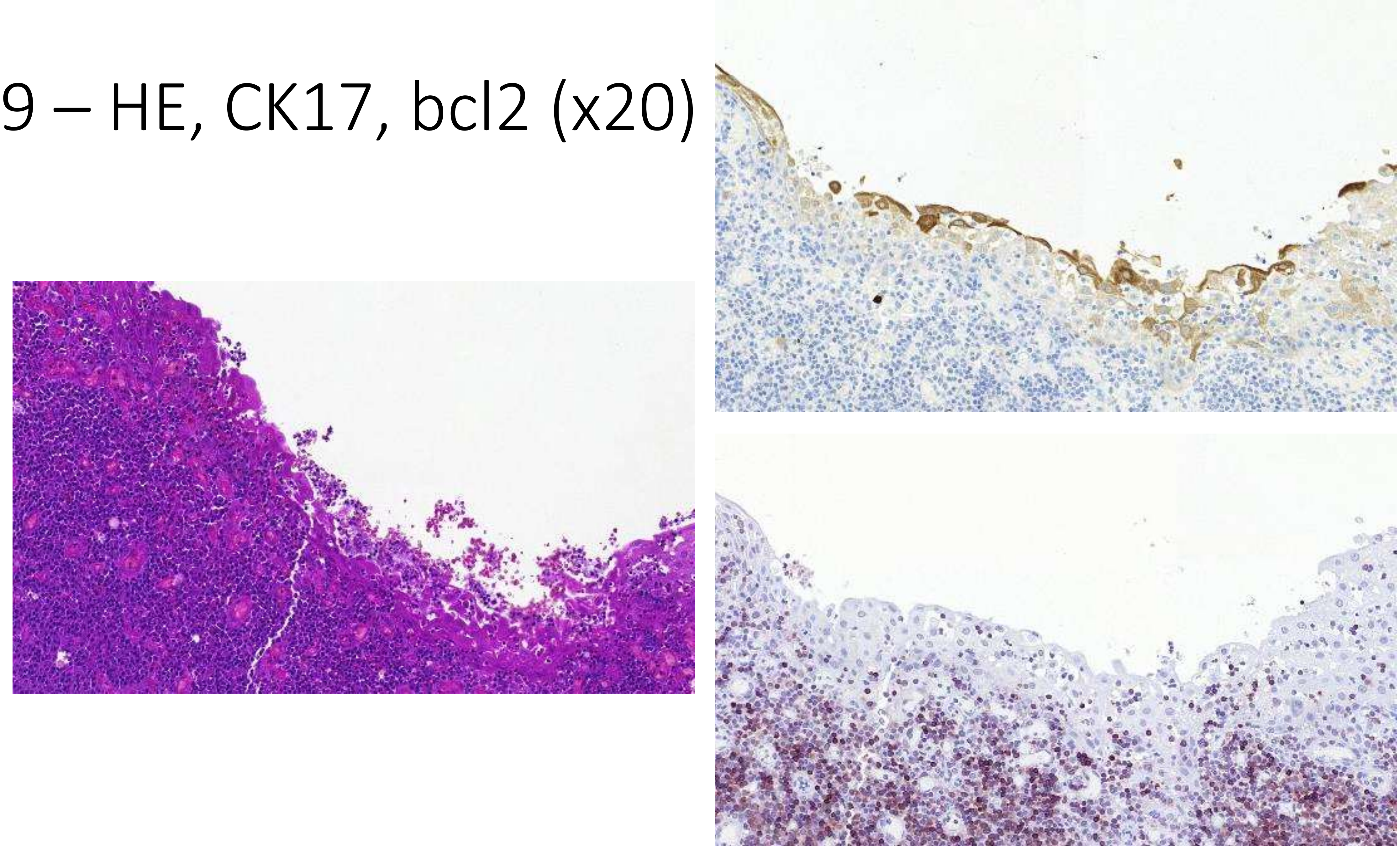

K29 – HE, CK17, bcl2 (x10)

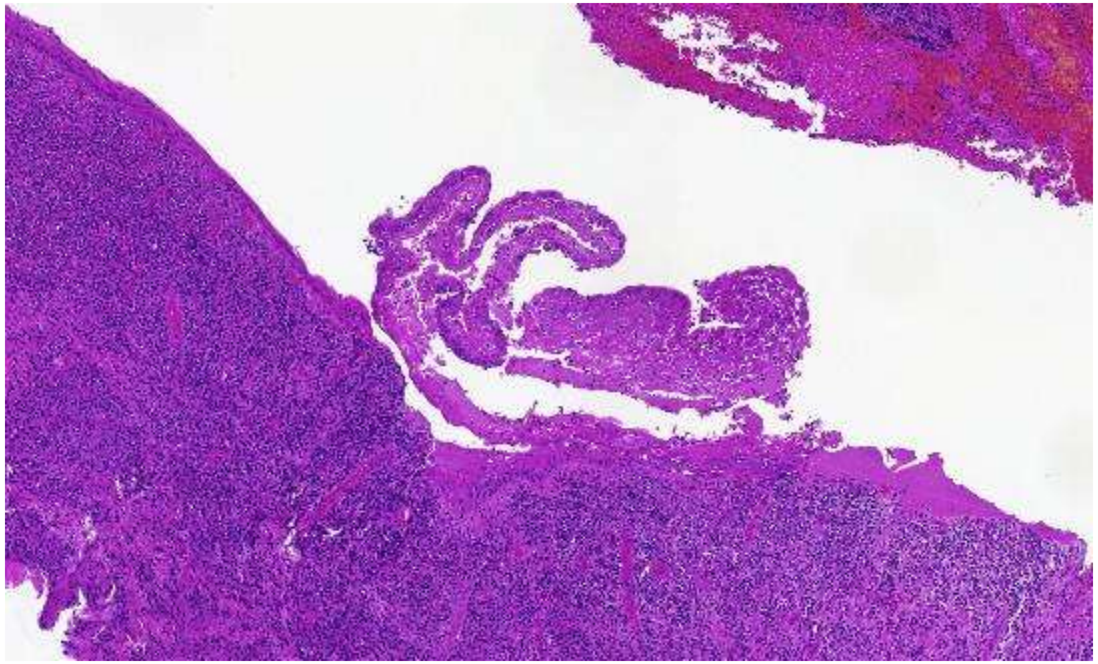

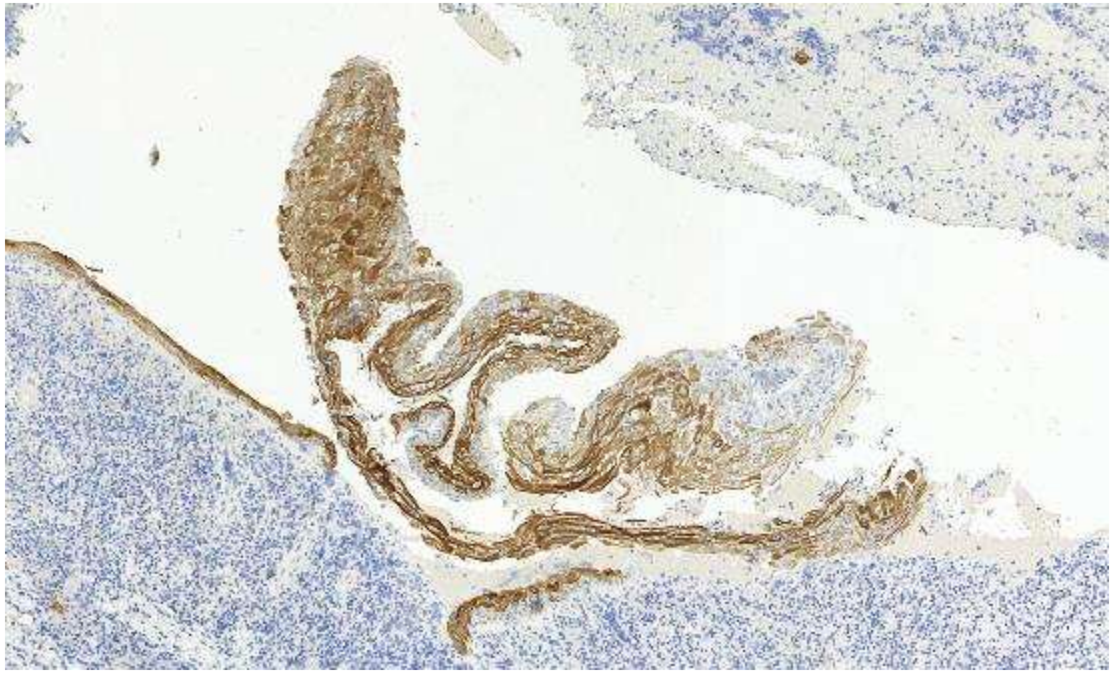

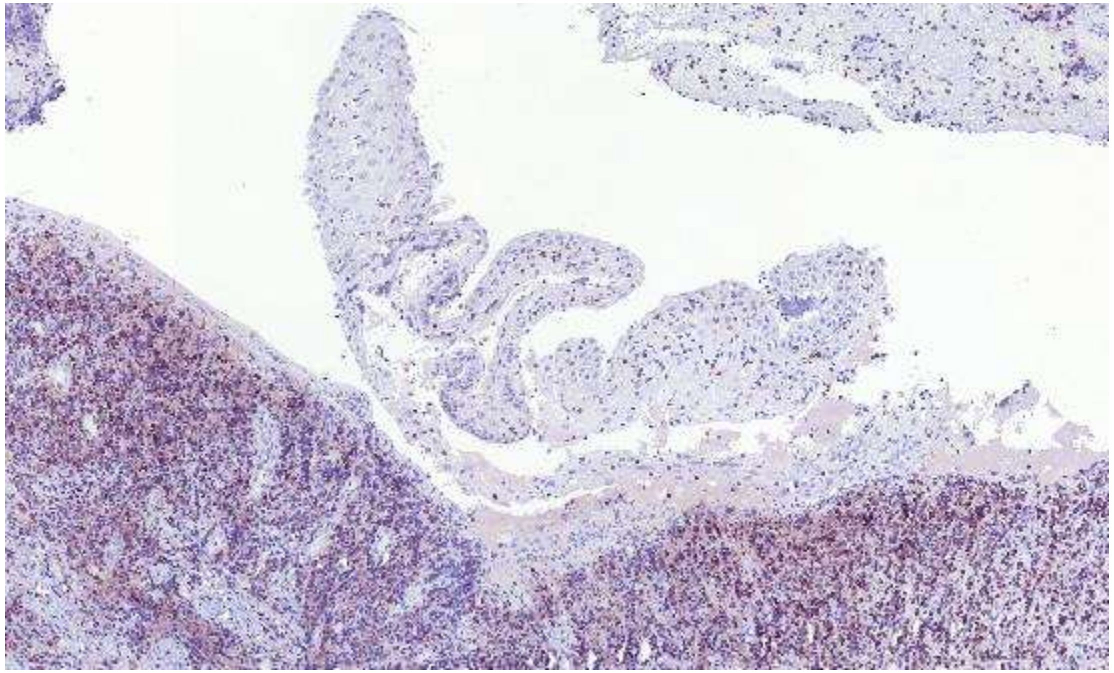

K29 – Conclusion

- H&E: relatively thick (focally thin) squamous epithelium and denuded areas, no parakeratosis, no wavy surface, no basal palisading, even to uneven epithelial-subepithelial contours
- Marked mixed inflammation of the cyst wall
- Bcl2: negative
- CK17: partly superficial, partly transepithelial
- Histopathology: inflammatory cyst; radicular cyst
- Combined clinical and pathological opinion: radicular cyst

Case K30 (same patient as K16, operated later for this second cyst)

- 20-year-old female
- Dental hygiene/status: Maintained, crowded, treated teeth
- Radiology: cyst periapical to tooth 11 suspected on OPT, confirmed on CT with thinning of the cortical bone
- Operation: Cystectomy
- Clinical diagnosis: „globulomaxillary cyst" or radicular cyst

K30 – OPT

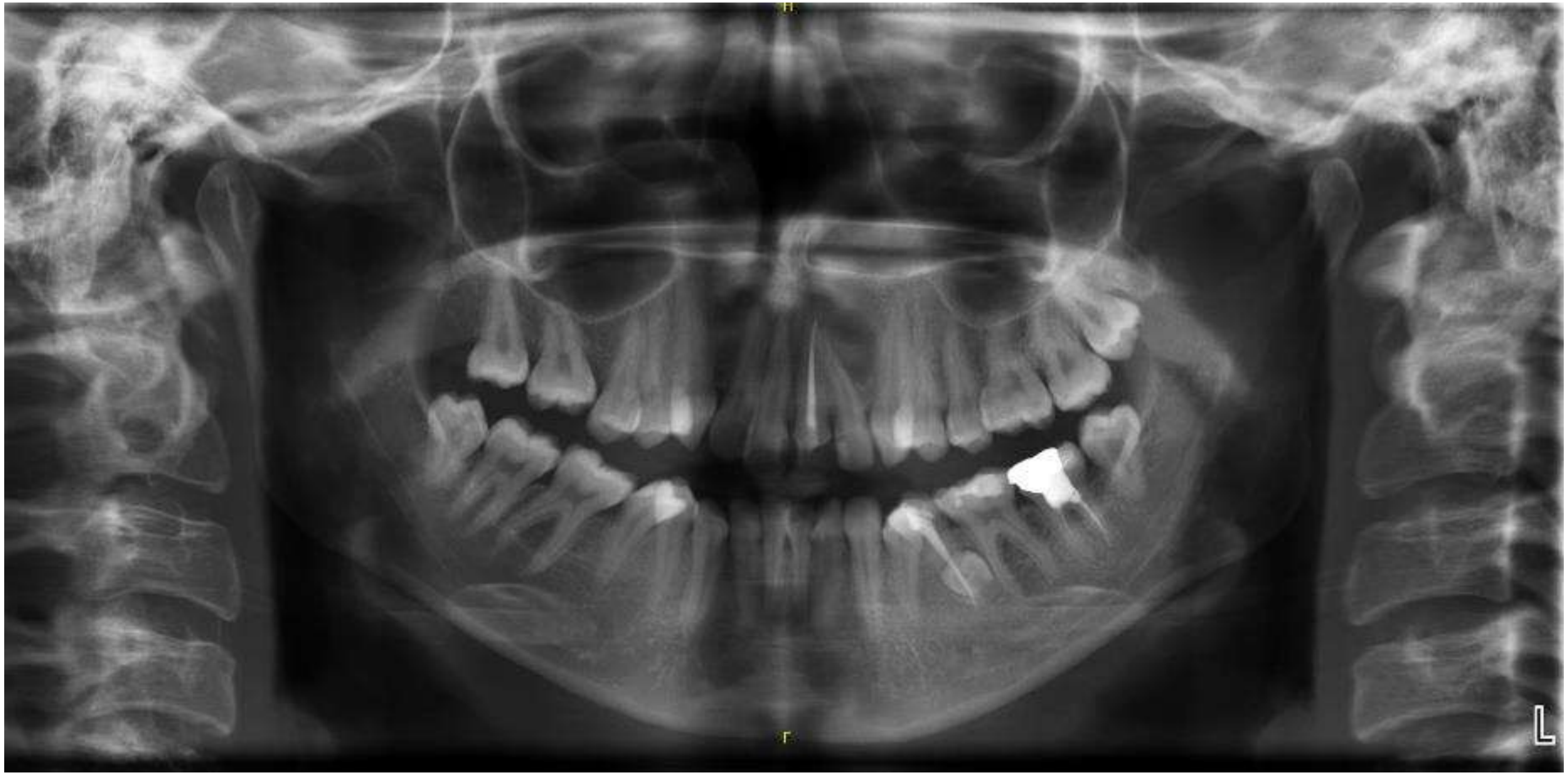

K30 – Radiograph

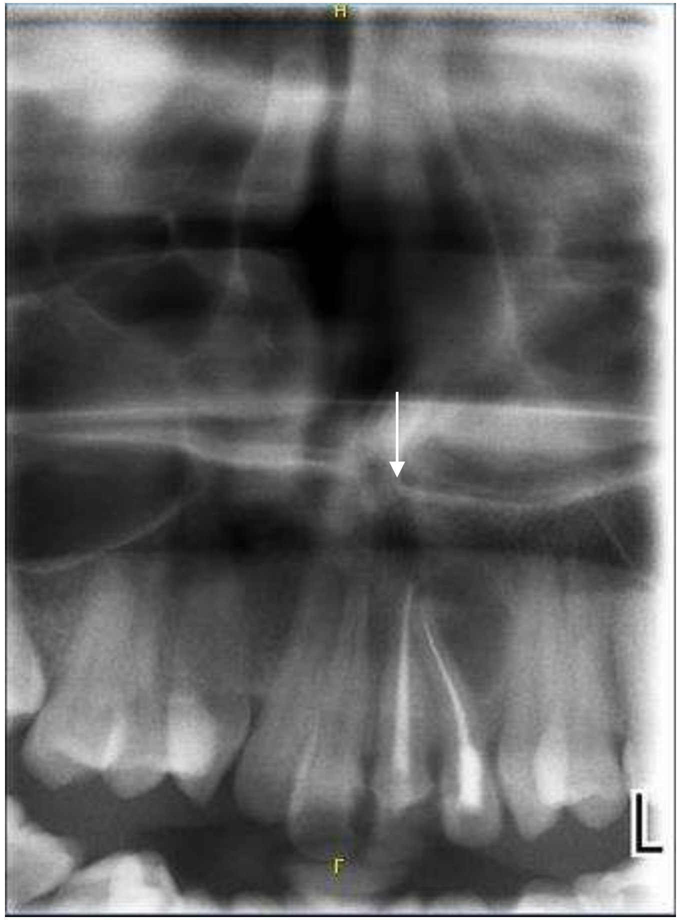

K30 – CT (coronal plane; anterior to posterior)

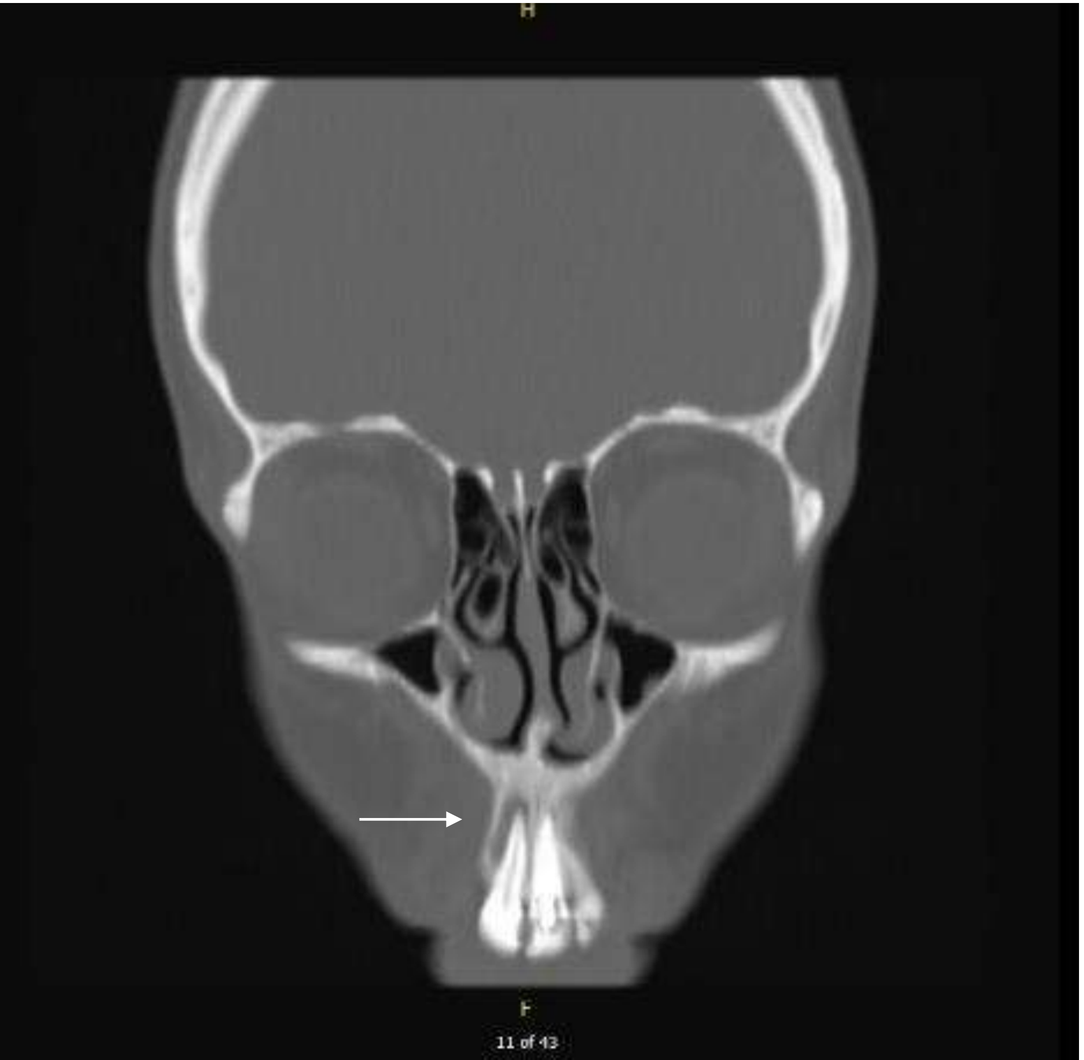

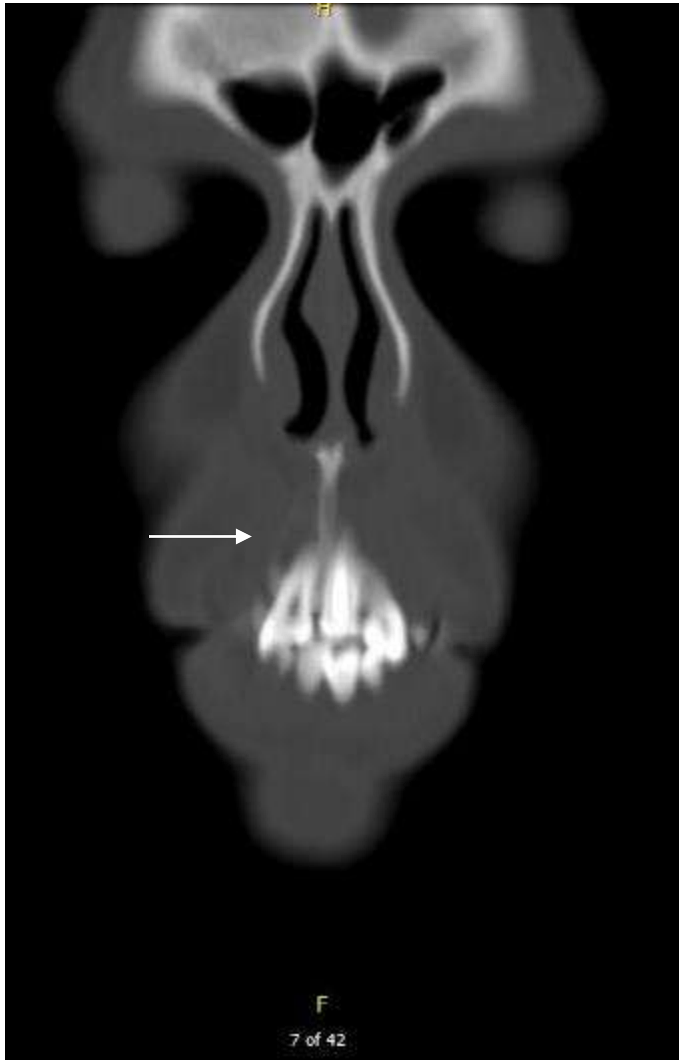

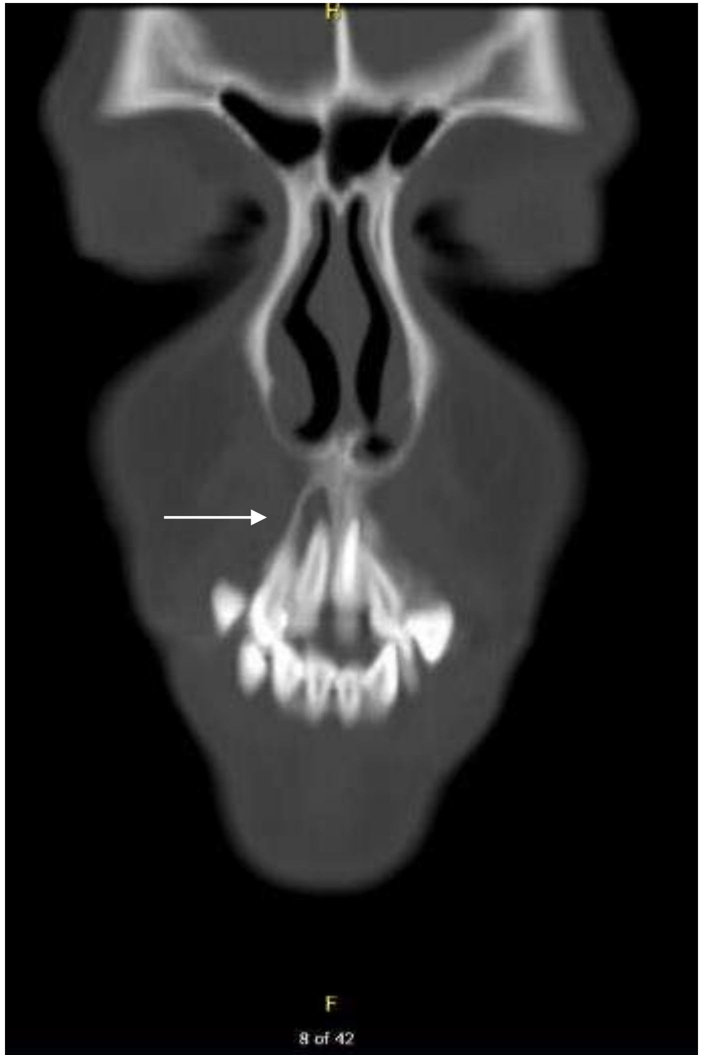

K30 – CT sagittal (right to left)

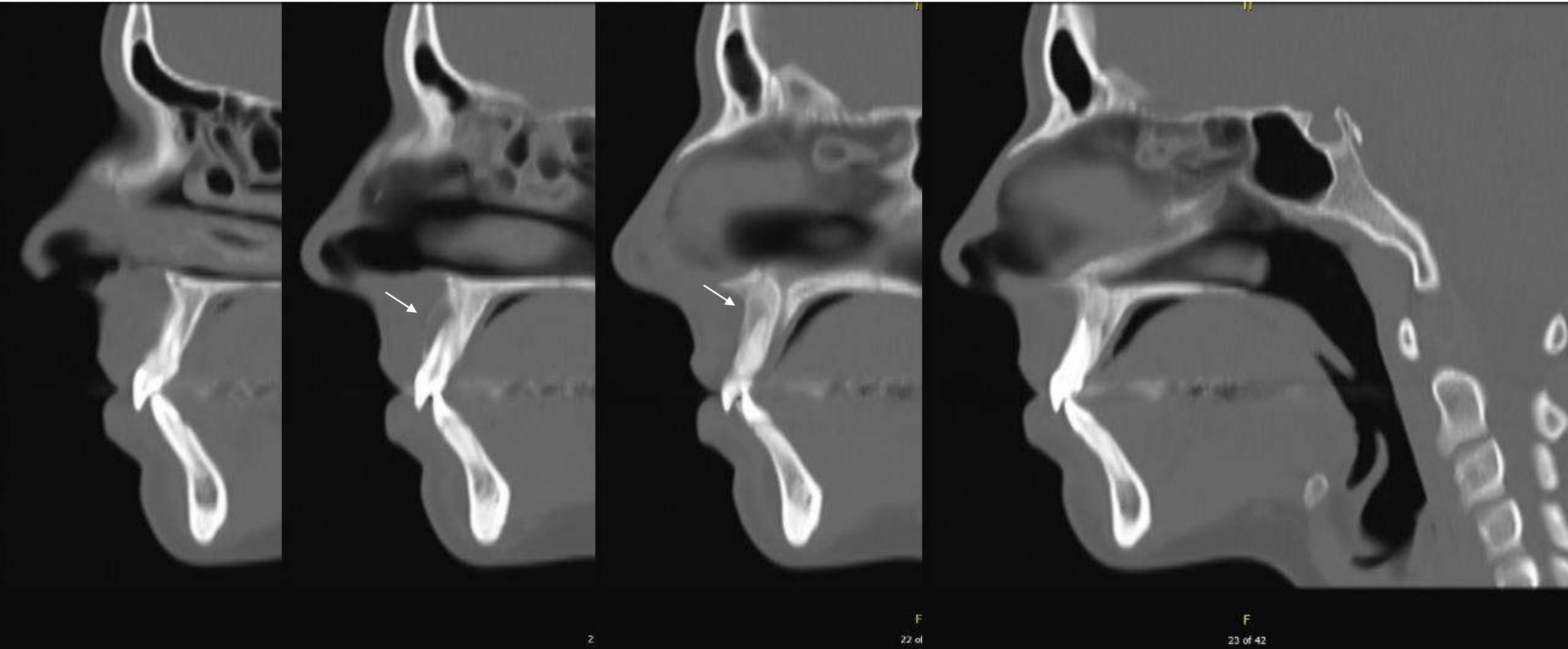

K30 – CT axial

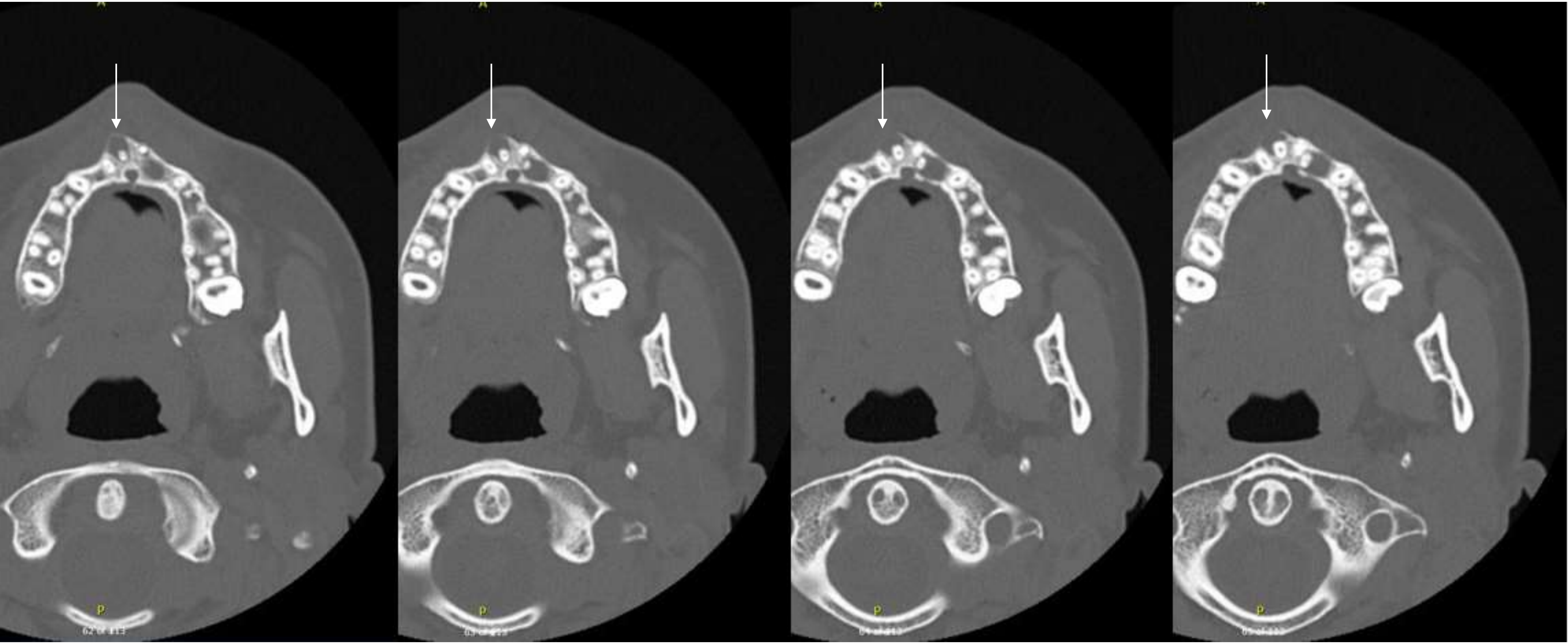

K30 – CT axial

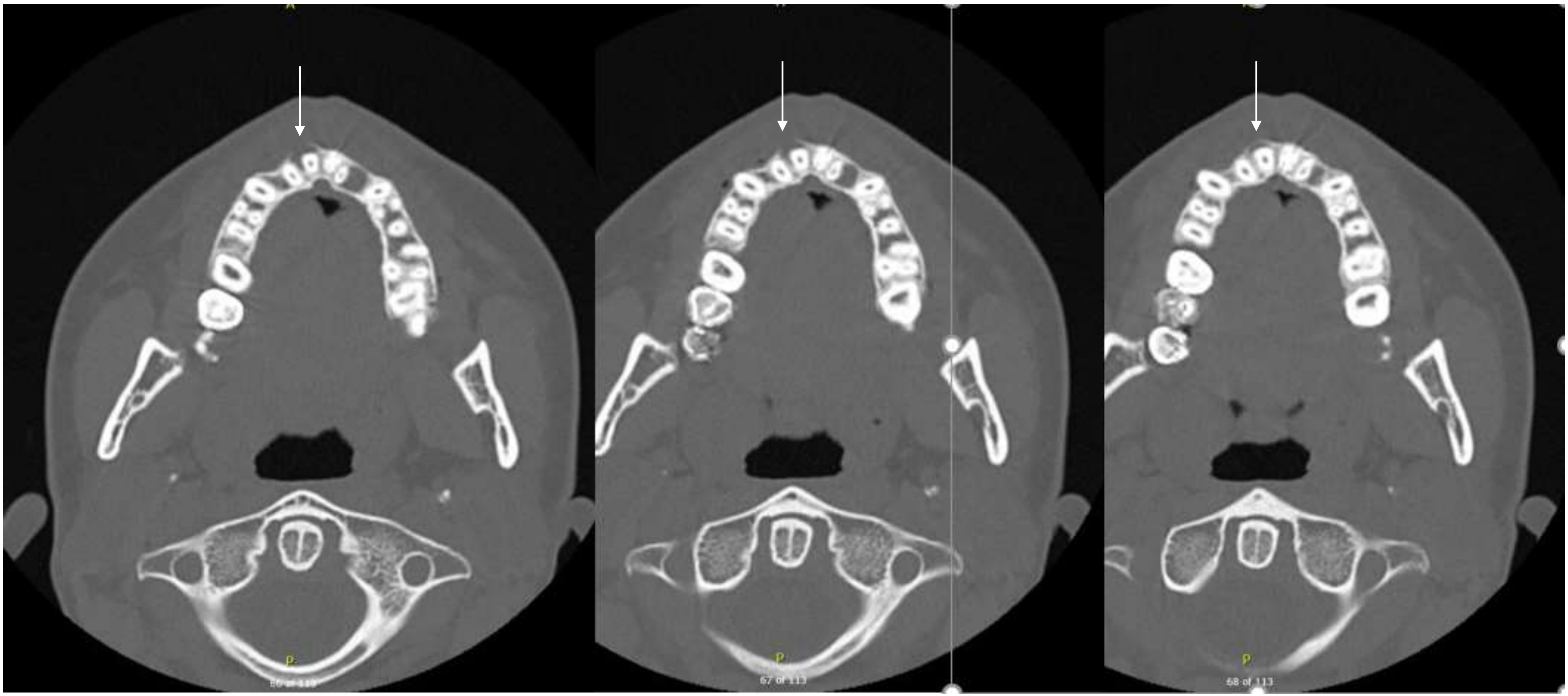

K30 – HE, CK17, bcl2 (x10)

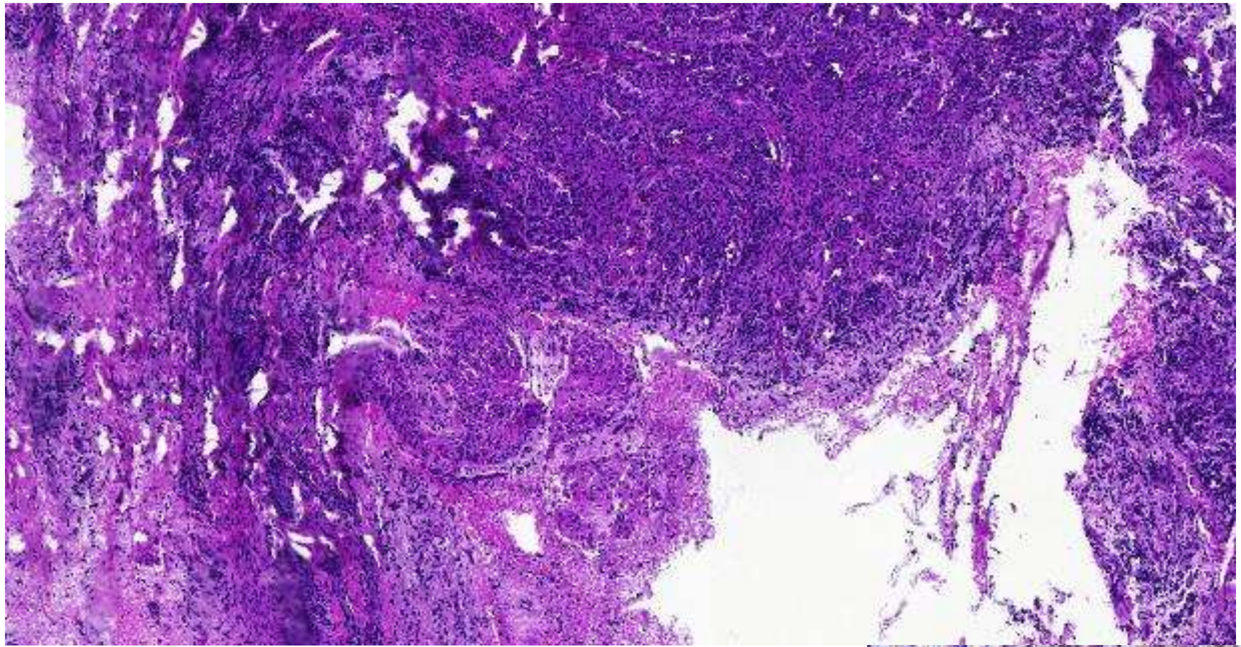

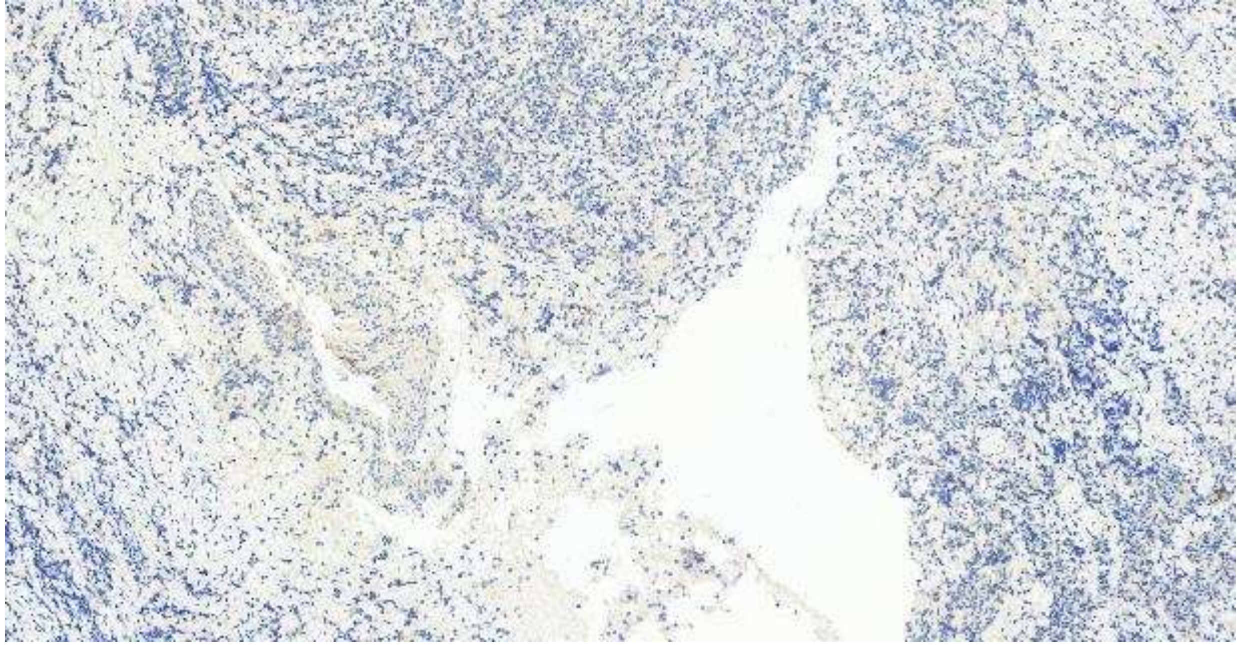

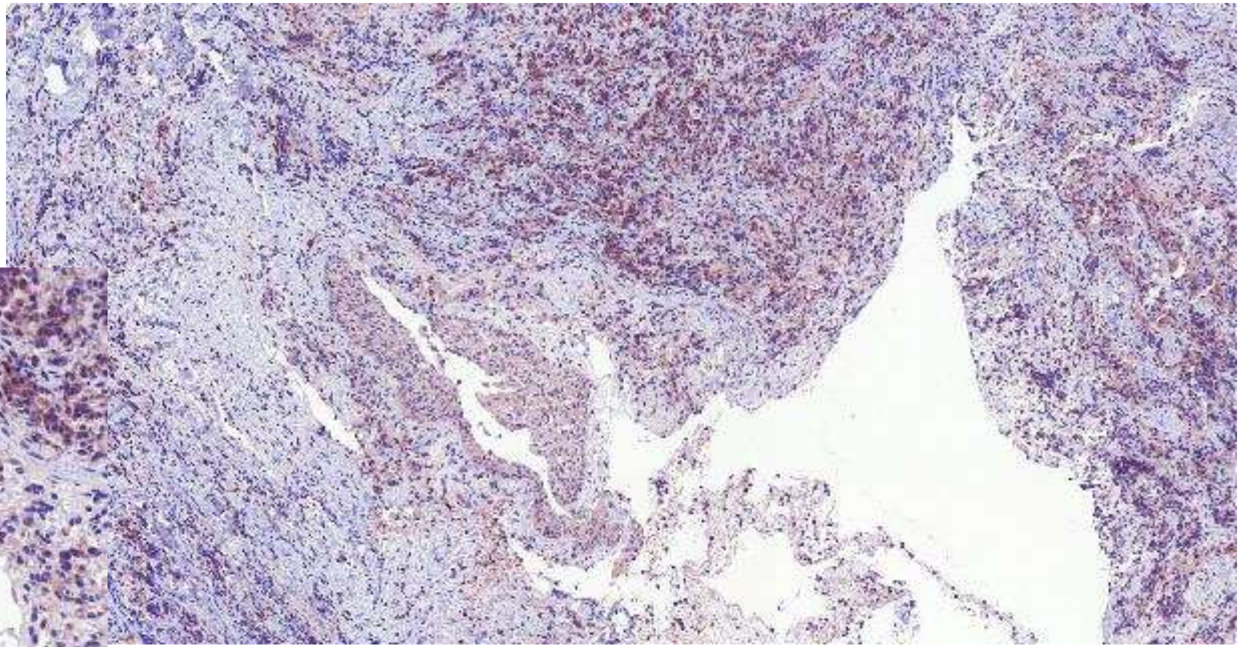

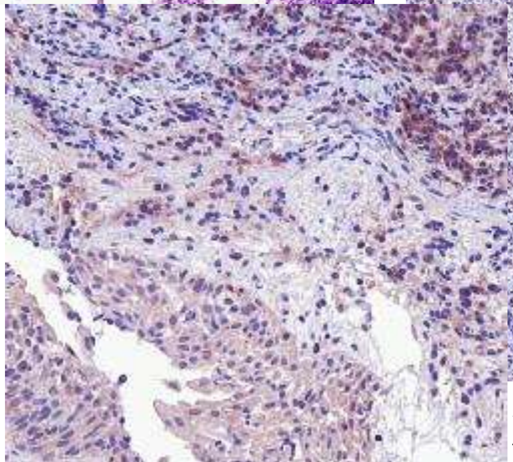

x25

K30 – HE, CK17, bcl2 x10

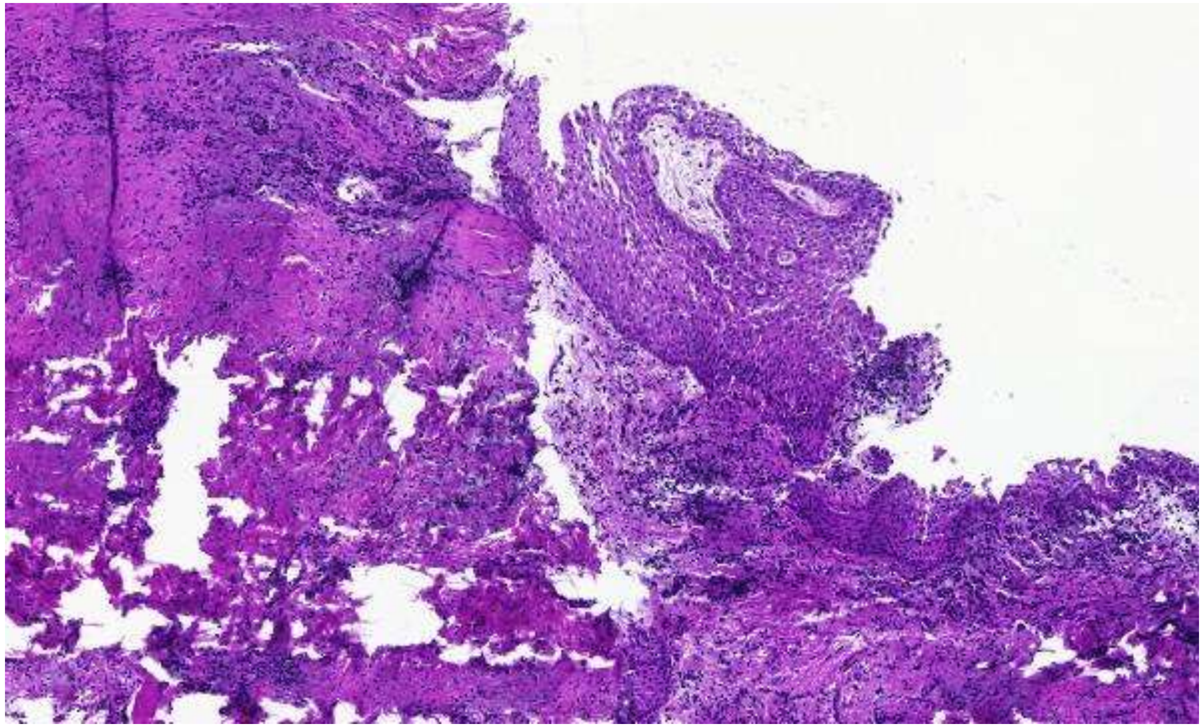

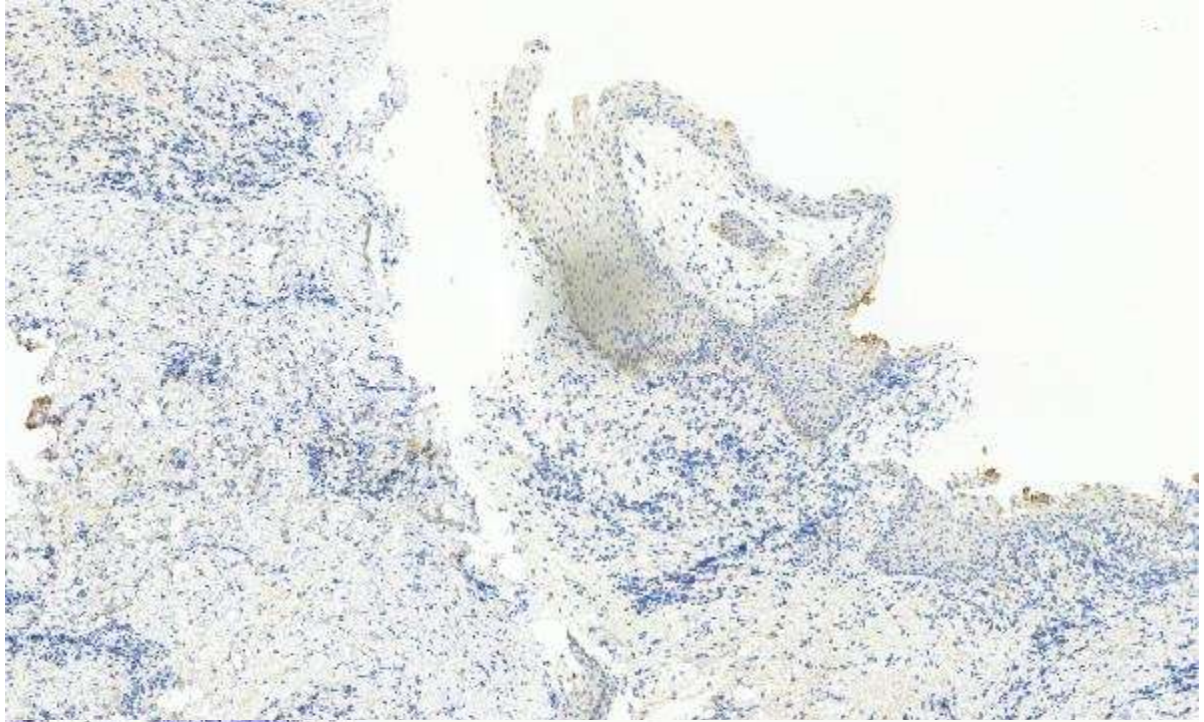

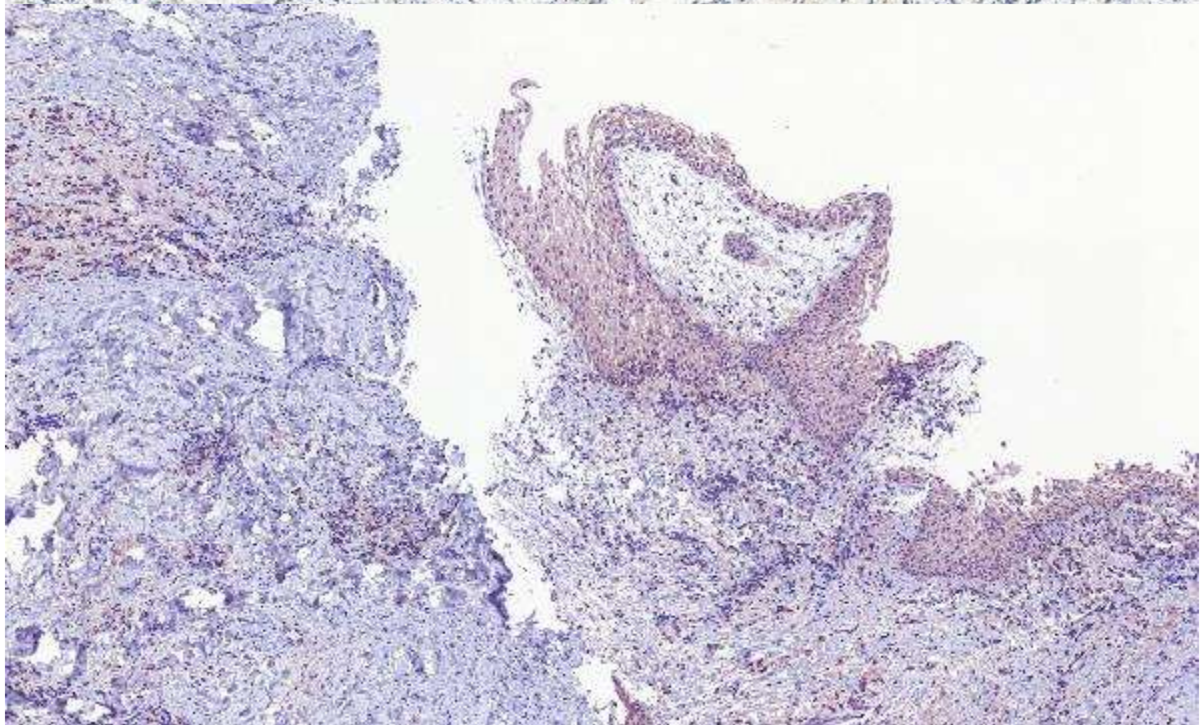

K30 – Conclusion

- H&E: relatively thick squamous epithelium and denuded areas, no parakeratosis, no wavy surface, no basal palisading, uneven epithelial-subepithelial contours
- Marked mixed inflammation of the cyst wall
- Bcl2: negative (with transepithelial background coloration)
- CK17: negative, focal superficial staining
- Histopathology: inflammatory cyst (N.O.S.)
- Combined clinical and pathological opinion: radicular cyst

Case K31

- 66-year-old male
- History: symptomless
- Dental hygiene/status hygiene: normal; partly replaced, partly missing teeth. Periodontitis. Terminally missing teeth distal to 33 and 43; tooth 33 is mobile, pus is oozing out from a pocket, on compression. No fistula detected.
- Radiology: Chance finding on OPT: Circumscribed homogeneous decrease in transparency (radiolucency) corresponding to teeth 34,35, reaching the root of tooth 33; horizontal bone loss in the region of teeth 31, 32, 33, 41, 42 and 43, and vertical bone loss at tooth 33.
- Operation: removal of the mobile tooth 33, followed by the removal of the cyst, which was not covered by bone buccally; the cyst was first detached from the periosteum, then mobilized from the bony base, then removed
- Clinical diagnosis: Cyst? Keratocyst? Ameloblastoma? (not well founded differential diagnostic list)

K31 – OPT

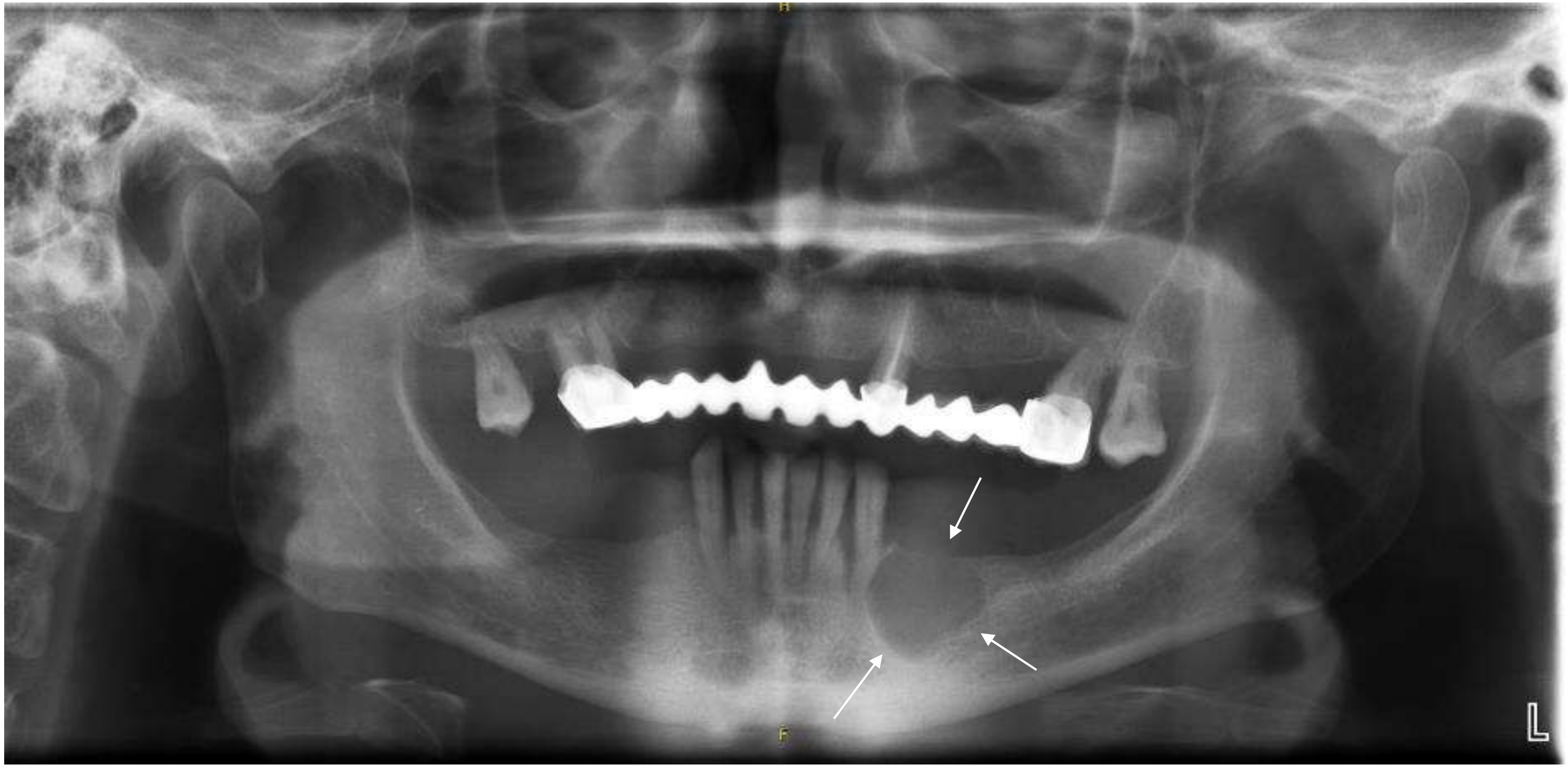

K31 – CT, coronal plane

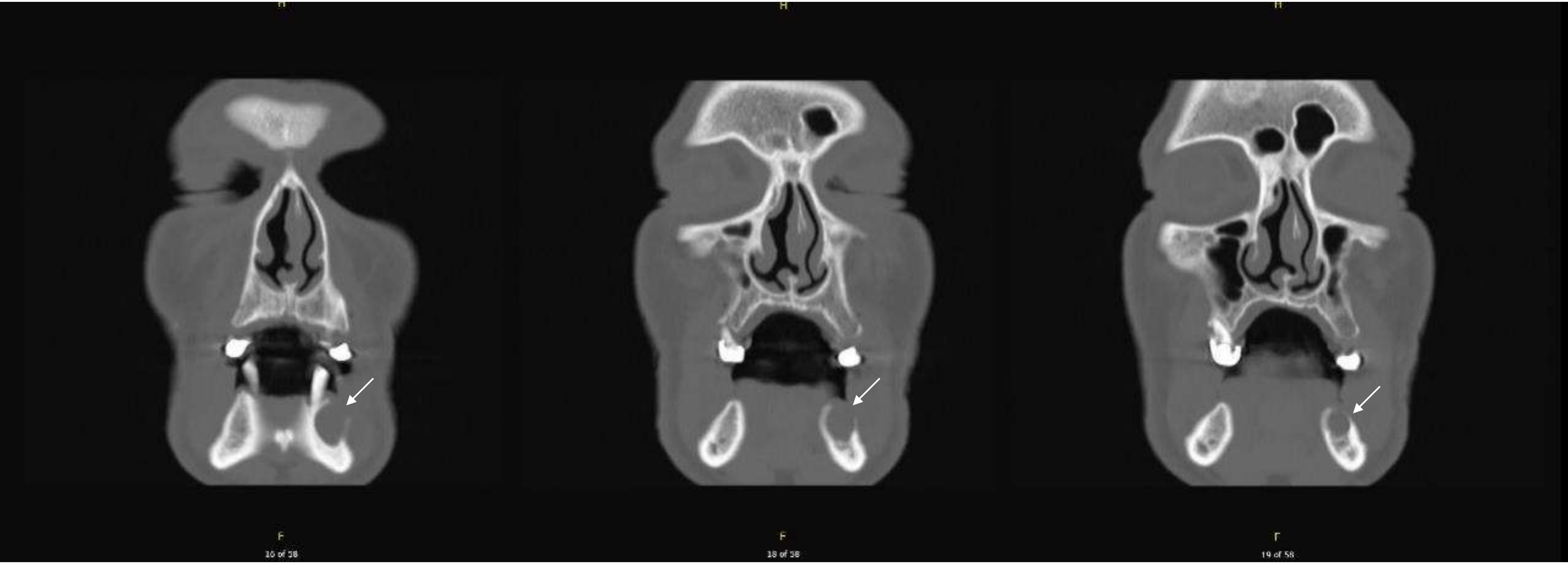

K31 – CT, oblique sagittal plane

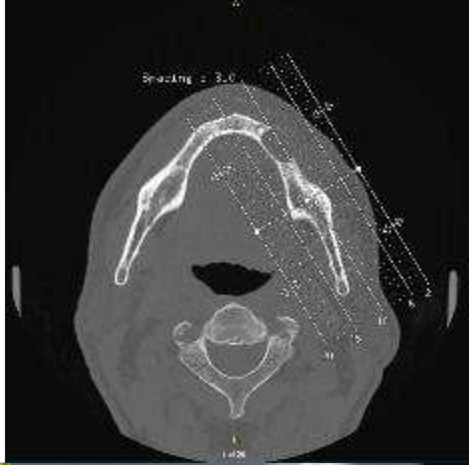

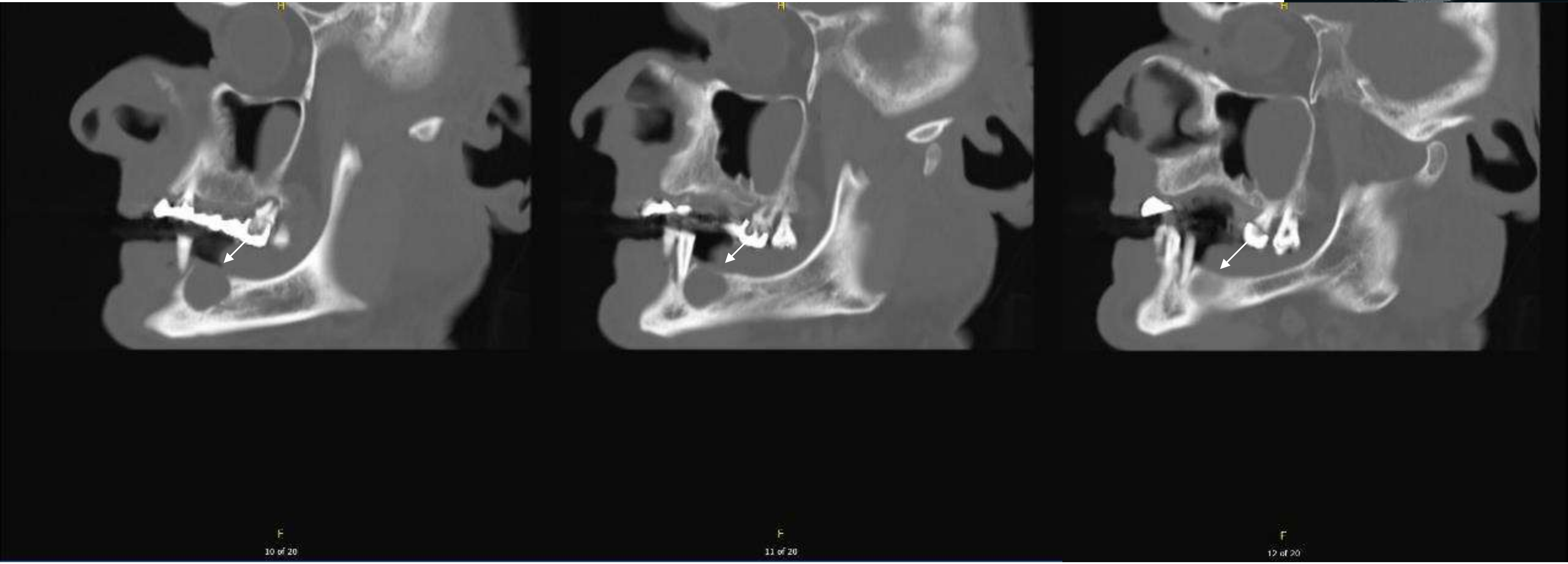

K31 – HE, CK17, bcl2 (x1)

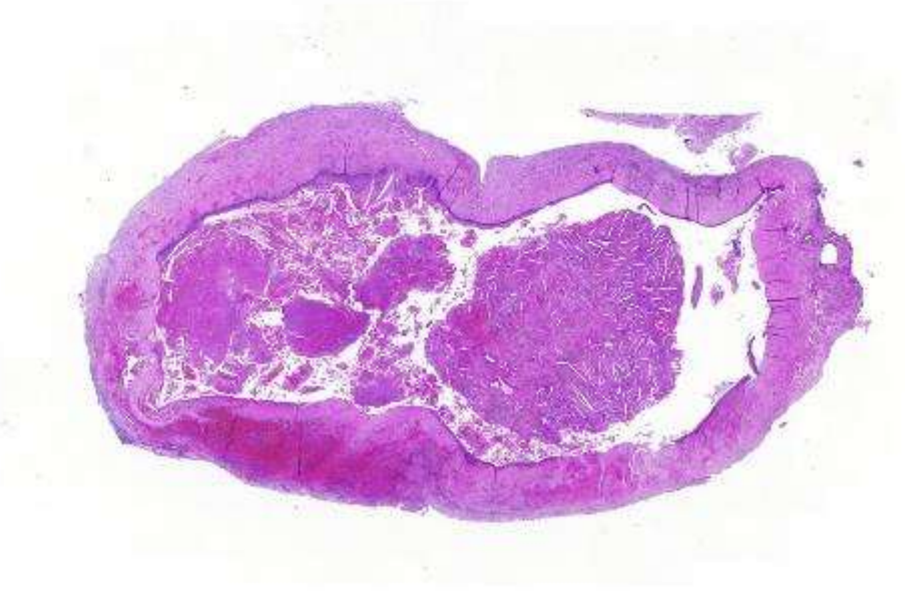

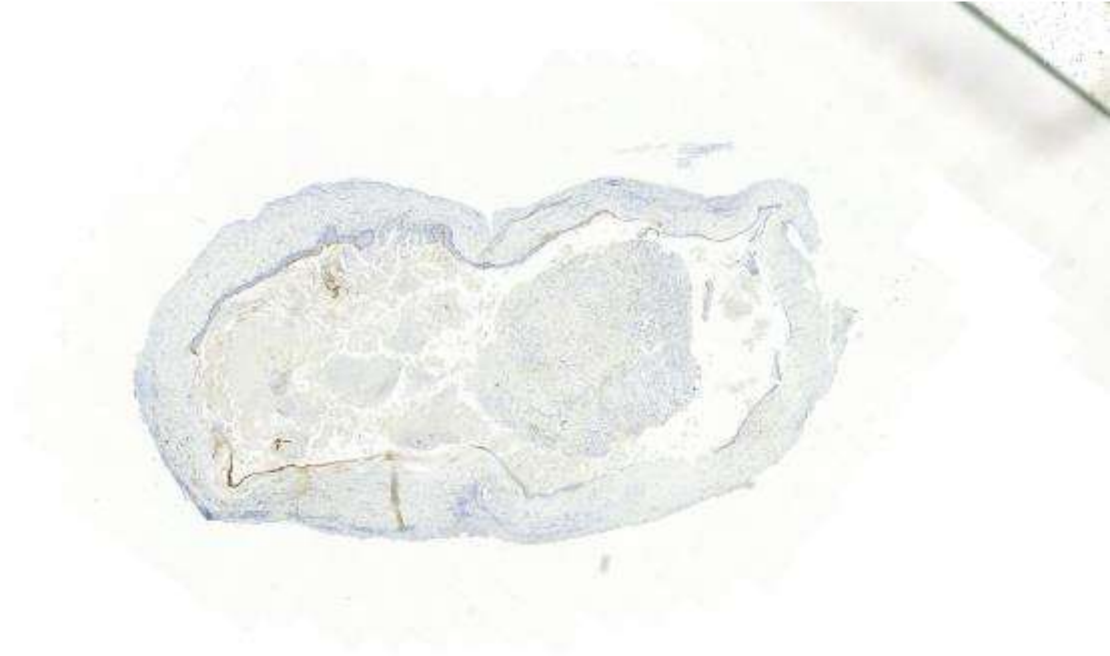

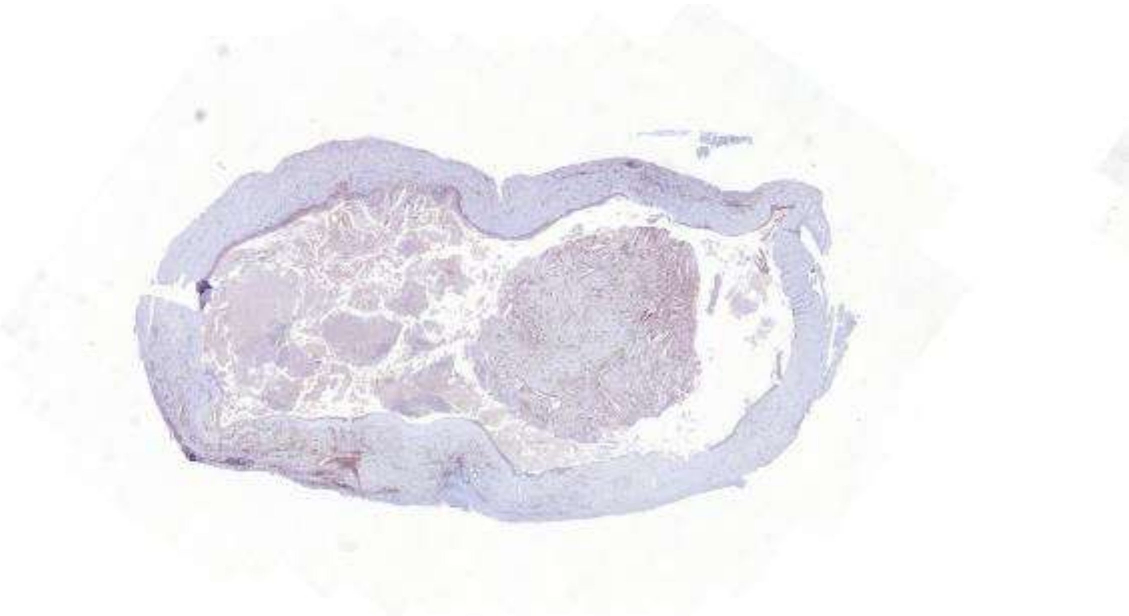

K31 – HE, CK17, bcl2 (x5)

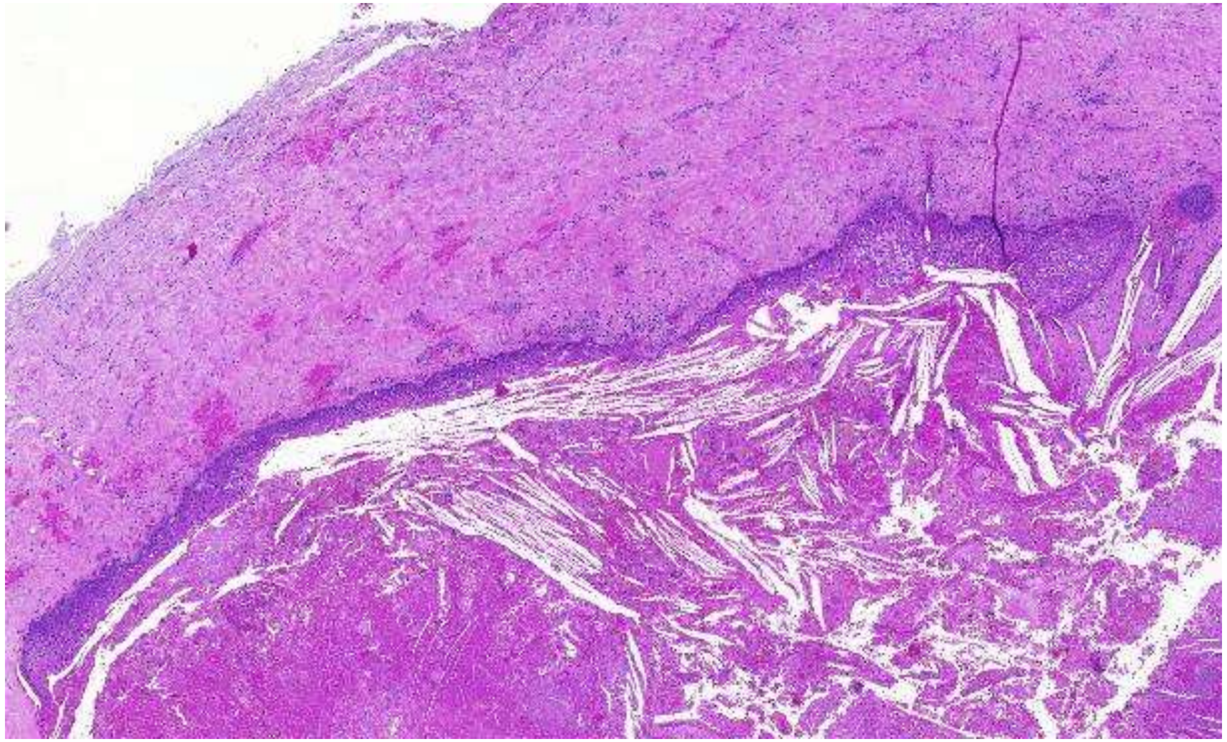

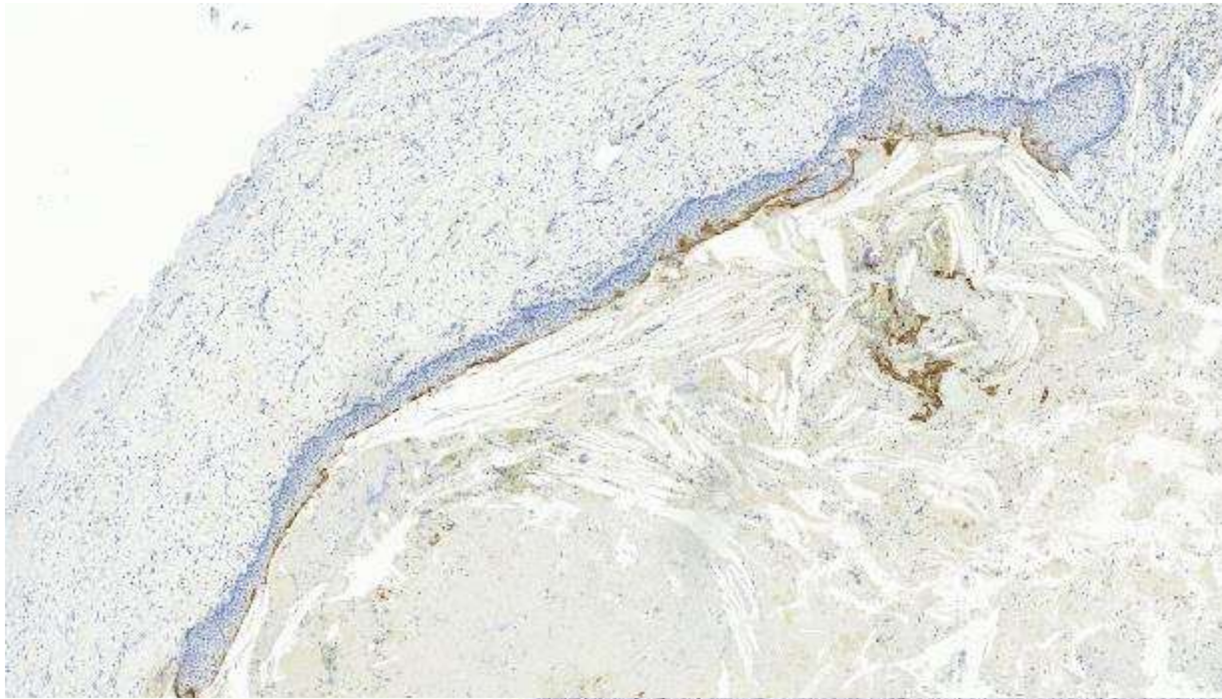

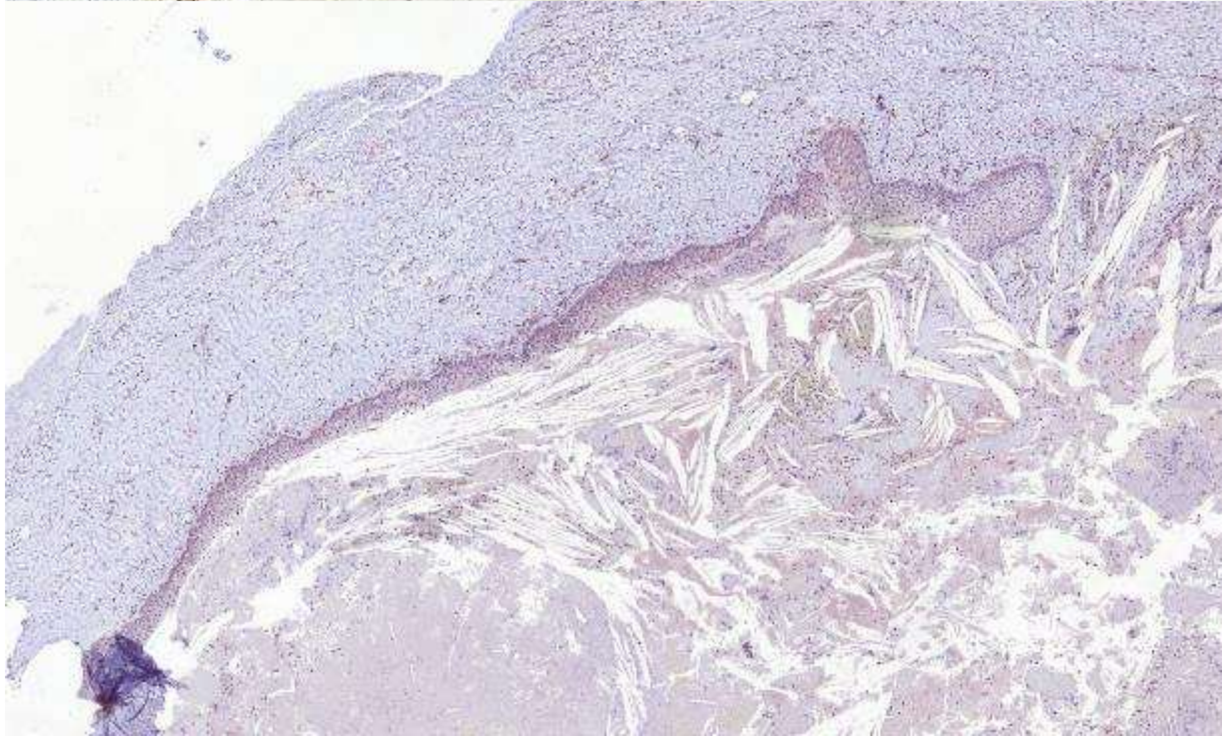

K31 – HE, CK17, bcl2 (x10)

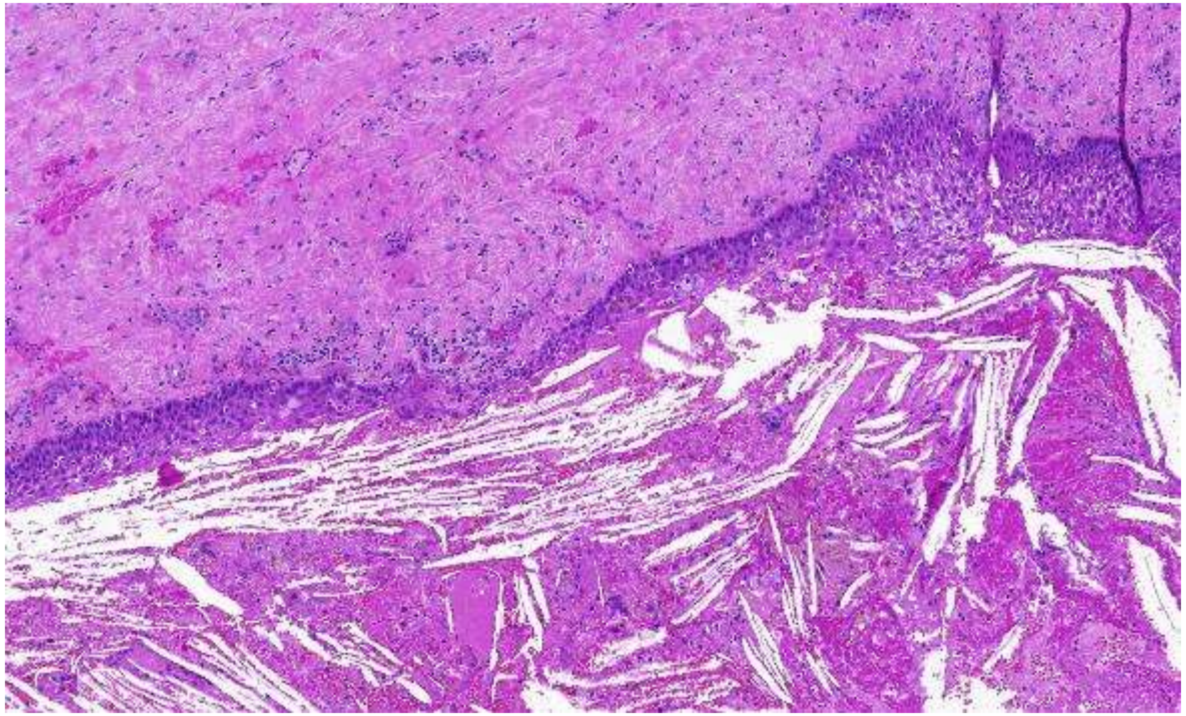

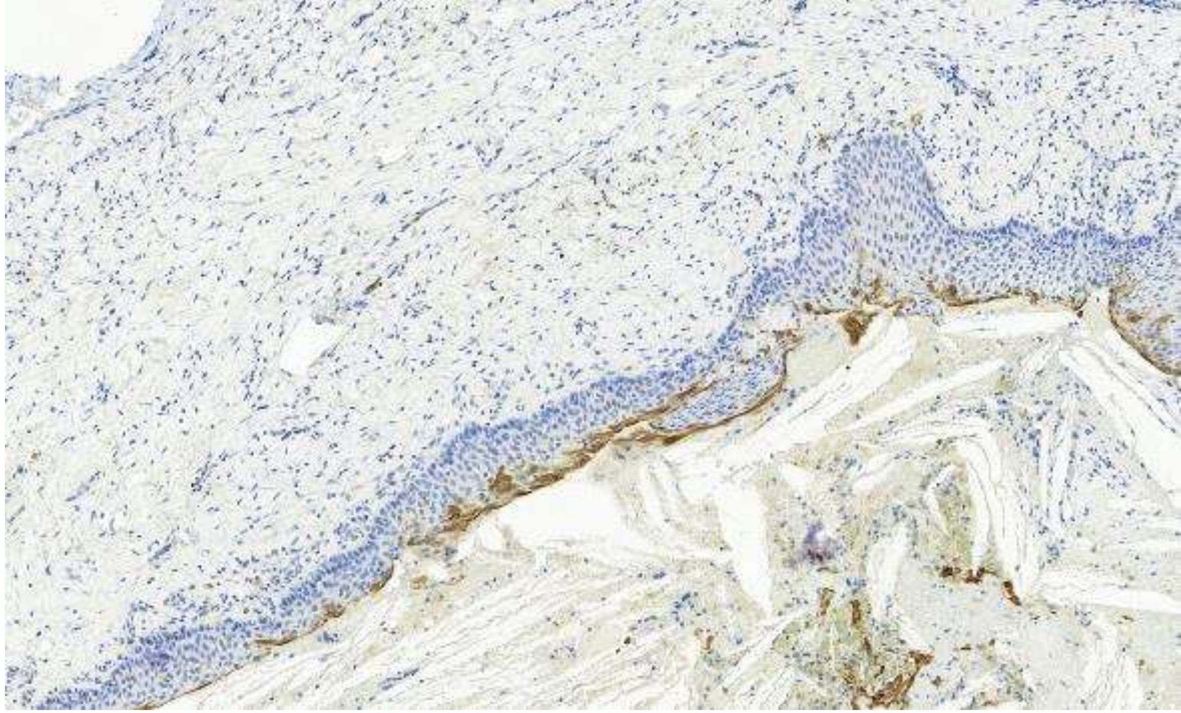

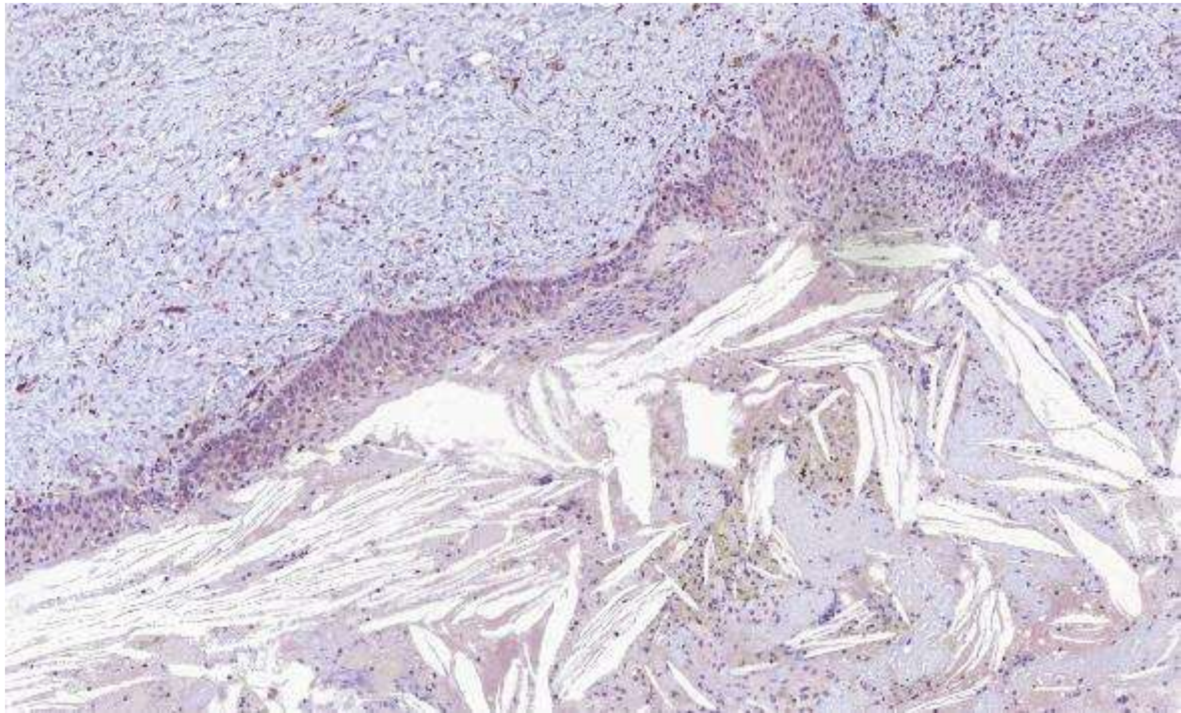

K31 – CK17 (x5, x10)

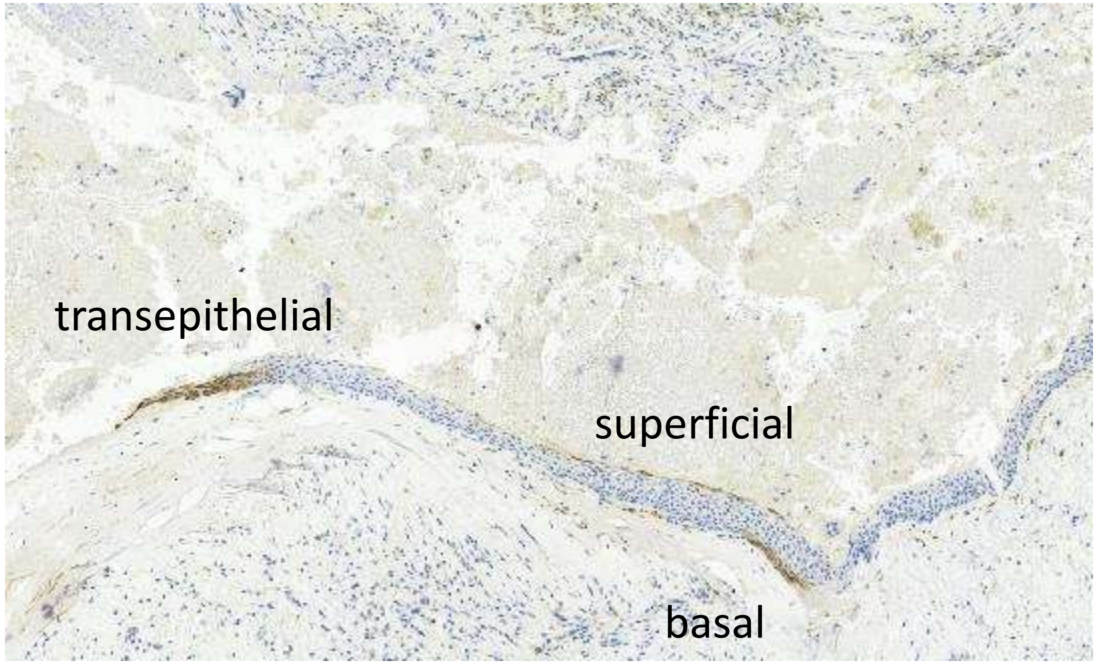

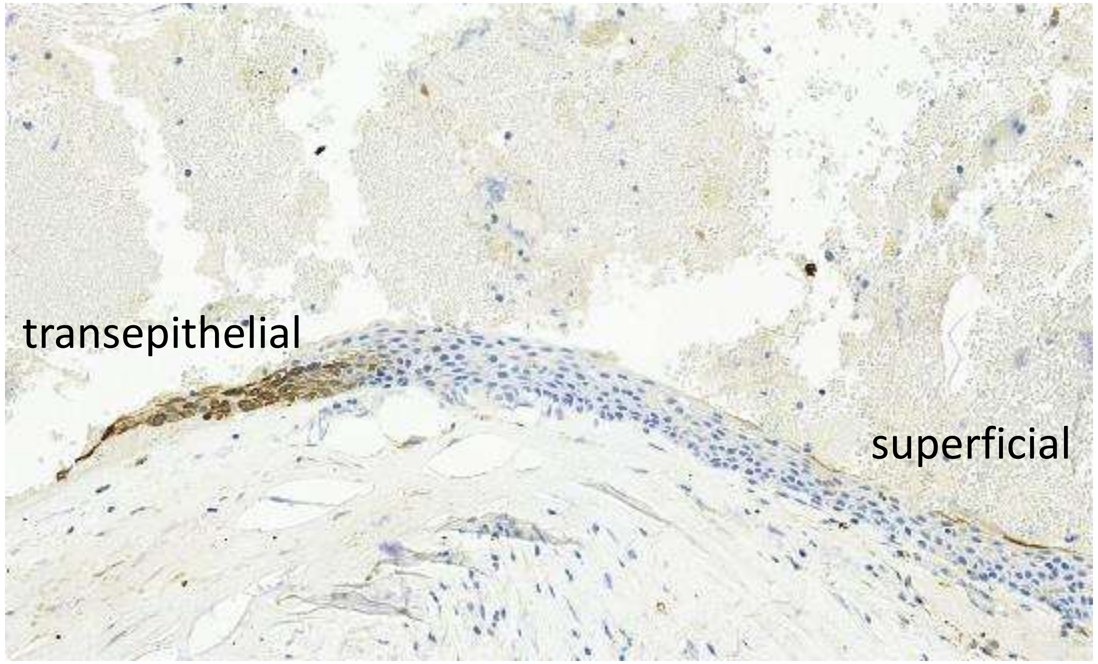

K31 – bcl2 x20

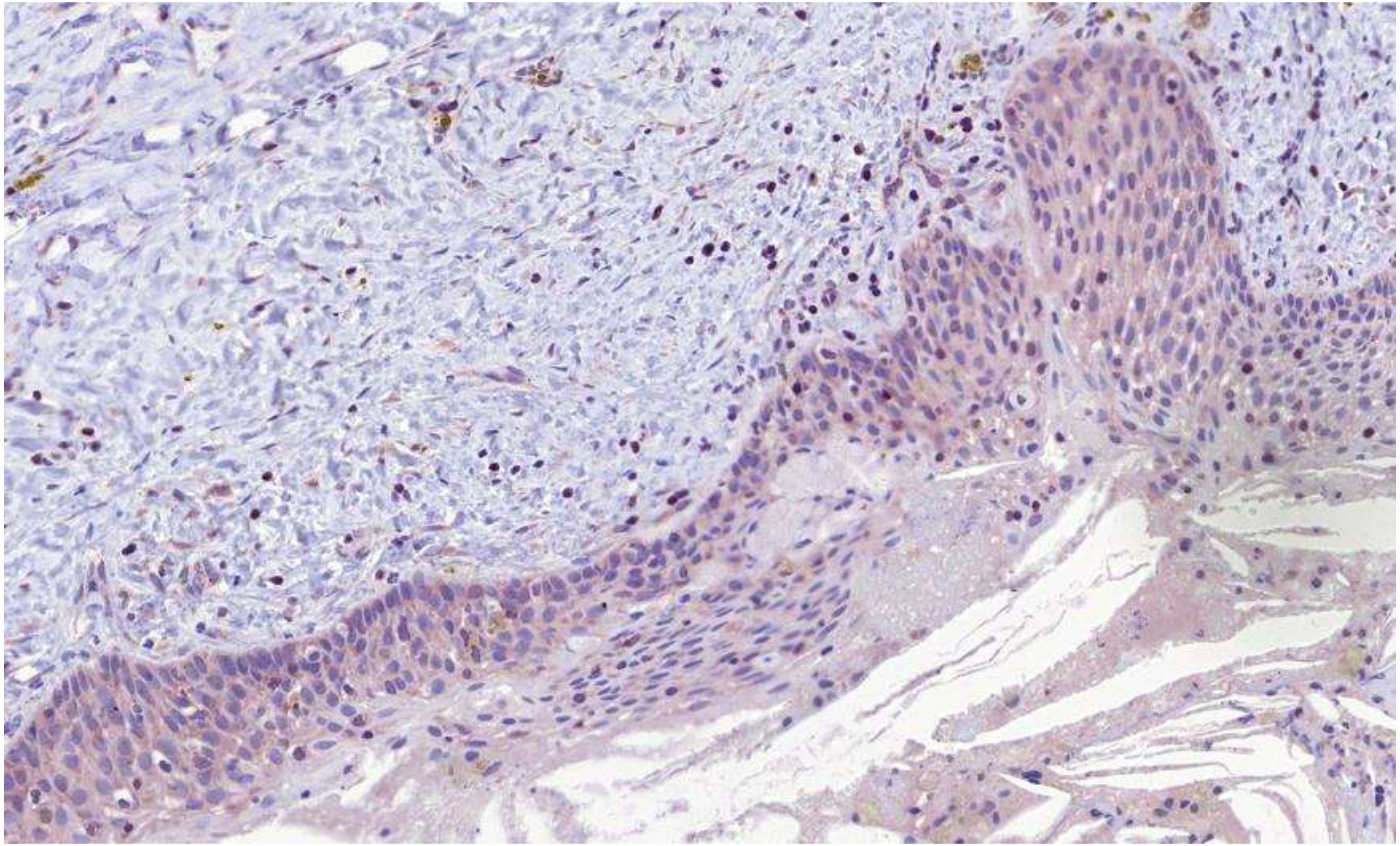

K31 – Conclusion

- H&E: relatively thick squamous epithelium (focally thin), no parakeratosis, no wavy surface, no basal palisading, even epithelial-subepithelial contours; focal mucous metaplasia
- Focal chronic inflammation in the cyst wall, cholesterol clefts and related tissue reaction inside the cyst and minimally also outside
- Bcl2: negative (ignored faint positivity)
- CK17: superficial staining with negative areas, focal basal or transepithelial staining
- Histopathology: inflammatory cyst consistent with a residual cyst
- Combined clinical and pathological opinion: consistent with inflammatory odontogenic cyst / residual cyst

Case K32

- 38-year-old male
- Dental hygiene/status: partly missing, retained roots, multiple teeth with caries, tartar
- Radiology: 2x2 cm larger well-circumscribed radiolucency around the root of tooth 13. Cyst located at the apex of tooth 13
- Operation: removal of multiple dental roots, and in toto removal of the maxillary cyst of tooth 13
- Clinical diagnosis: cyst N.O.S.

K32 – OPT

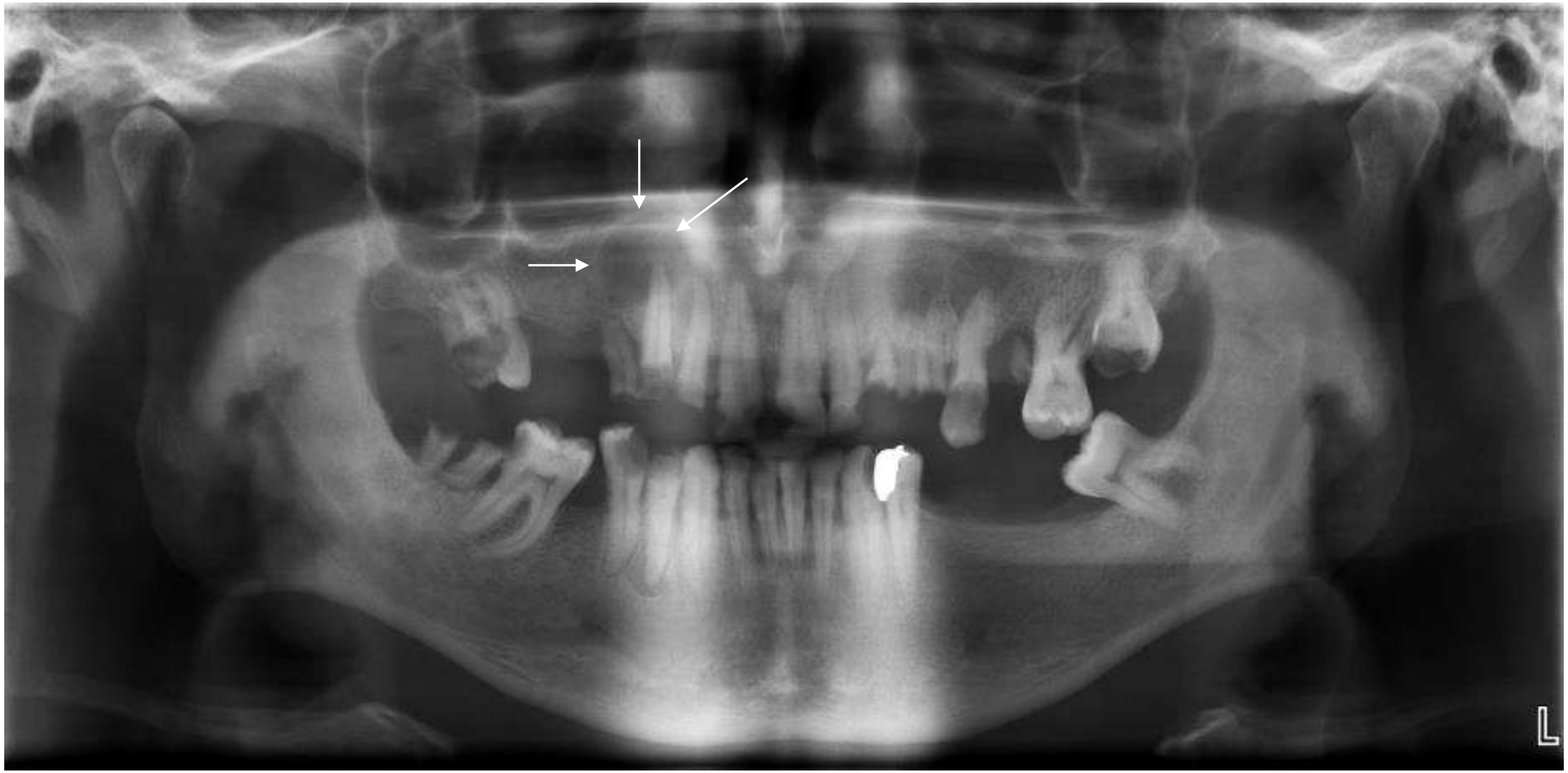

K32 – HE, CK17, bcl2 (x15)

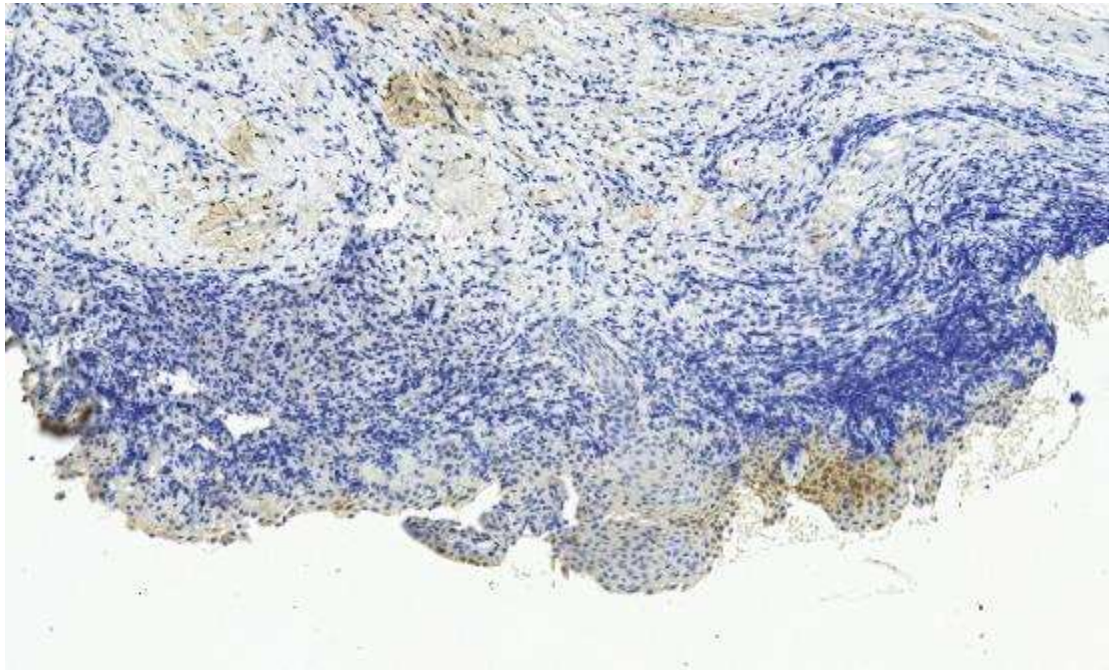

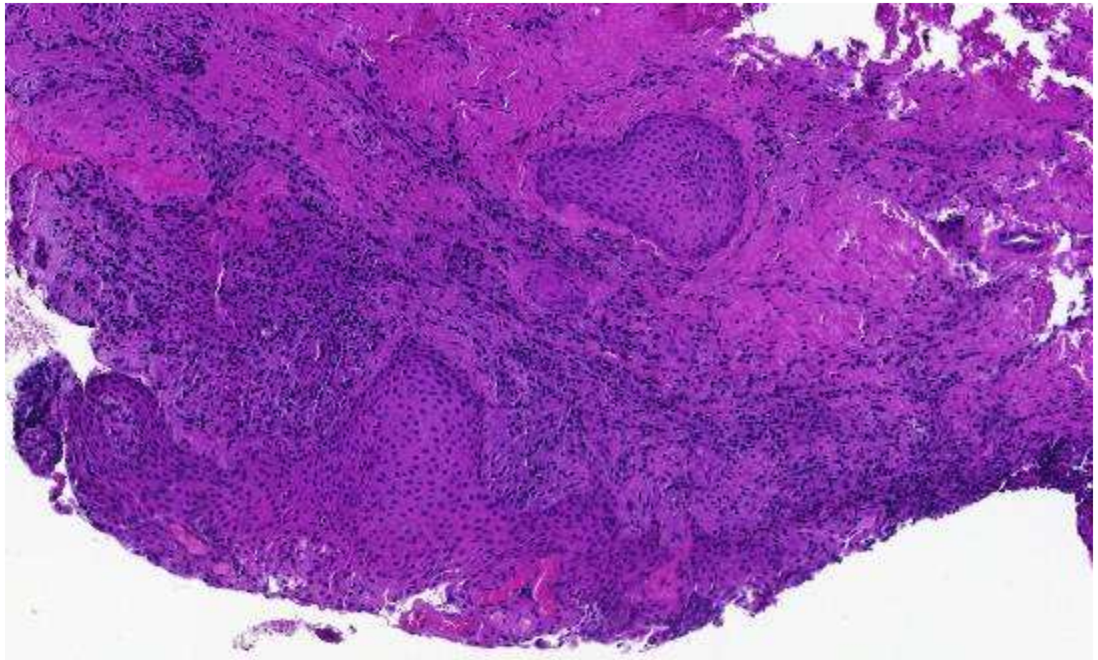

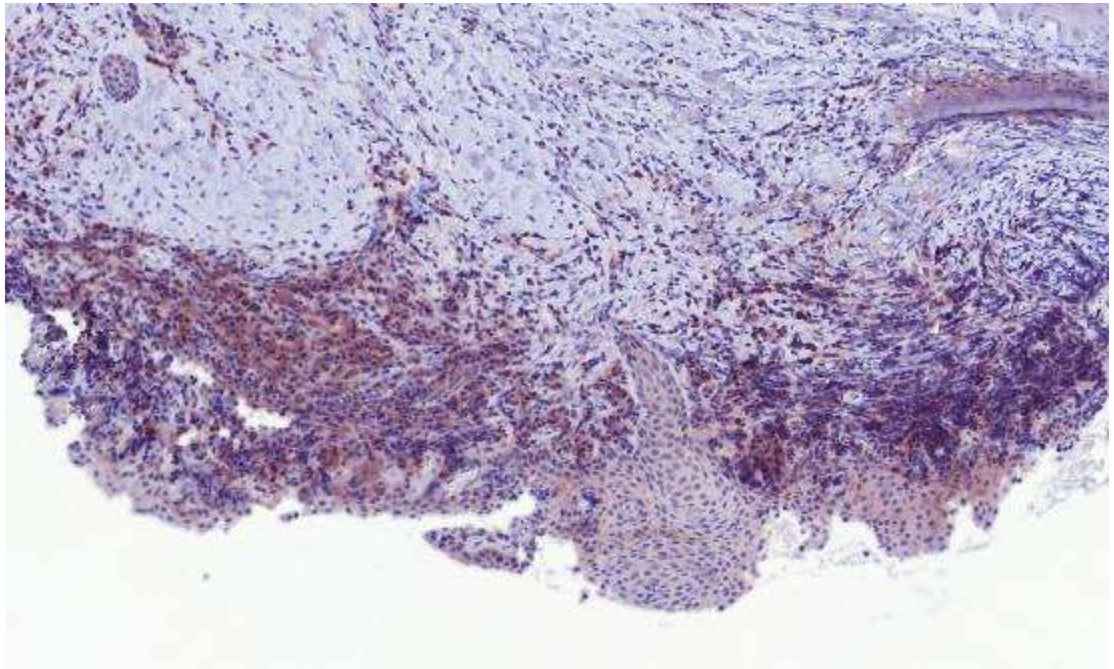

K32 – bcl2 (x30)

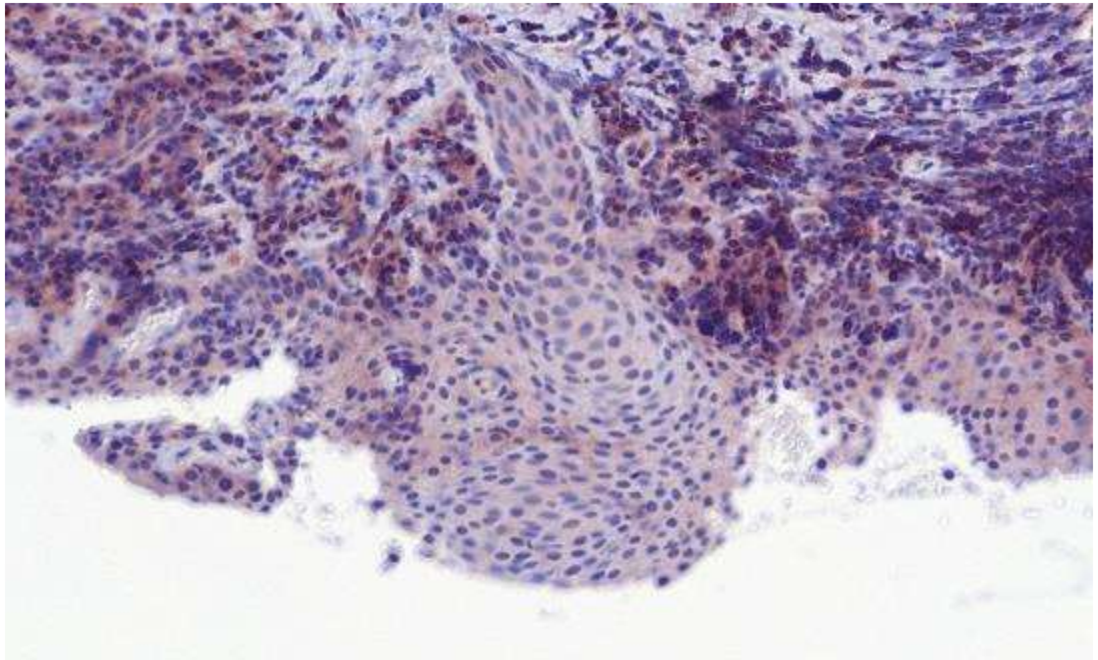

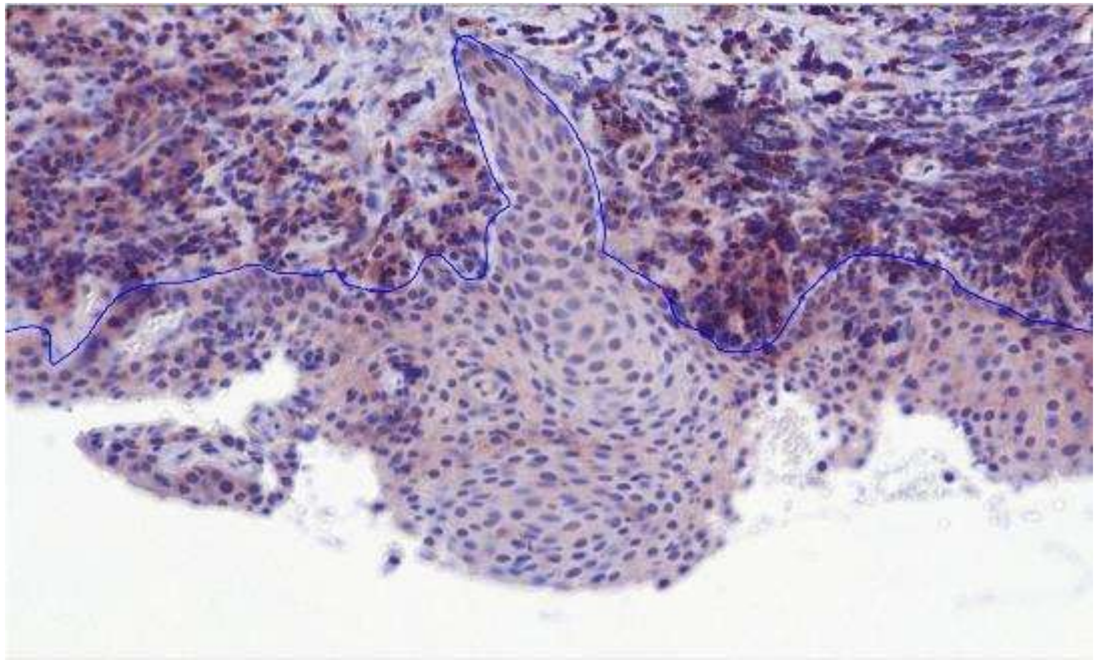

K32 – Conclusion

- H&E: largely denuded; relatively thick, non-keratinizing epithelium, no parakeratosis, no wavy surface, no basal palisading, uneven epithelial-subepithelial contours
- Marked chronic inflammation of the cyst wall
- Bcl2: negative
- CK17: negative, partly superficial, partly involving the spinous cell layer
- Histopathological diagnosis: inflammatory odontogenic cyst / radicular cyst
- On clinical grounds & histopathology: radicular cyst

Case KR1

- 76-year-old female
- Dental hygiene/status: partly conserved, partly missing and unreplaced teeth, parodontopathy; tooth 11 is mobile (grade I), crown of tooth 12 is missing, root in the alveolus
- Radiology: 1 cm large radiolucency at the apex of retained root of tooth 12
- Operation: removal of tooth 11 (along with tooth 42) and root 12; oozing pus from the wound, blunt separation of the cyst wall from the bone and removal (cystectomy)
- Clinical diagnosis: radicular cyst

KR1 – OPT

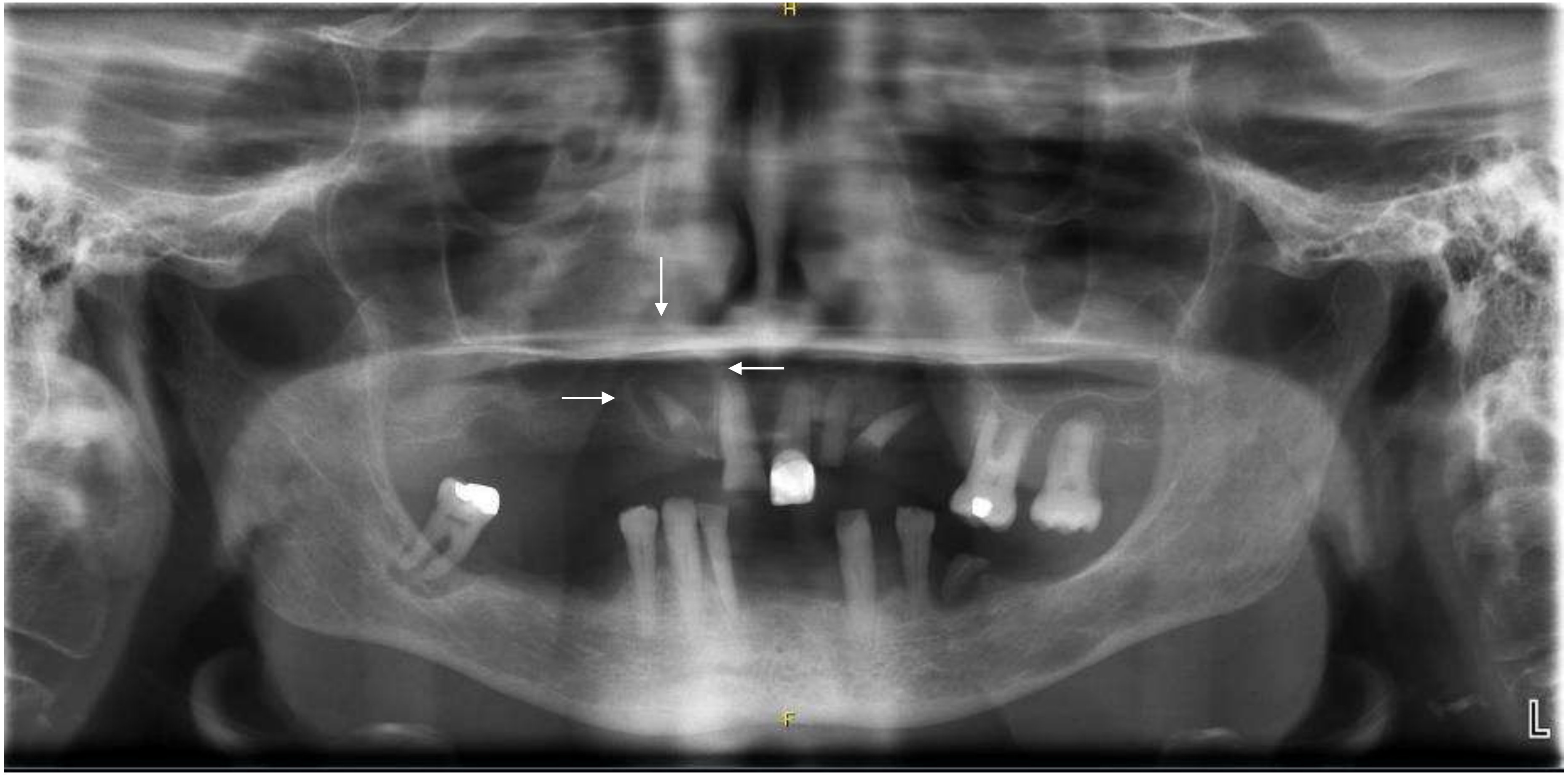

KR1 – HE, CK17, bcl2 (x1)

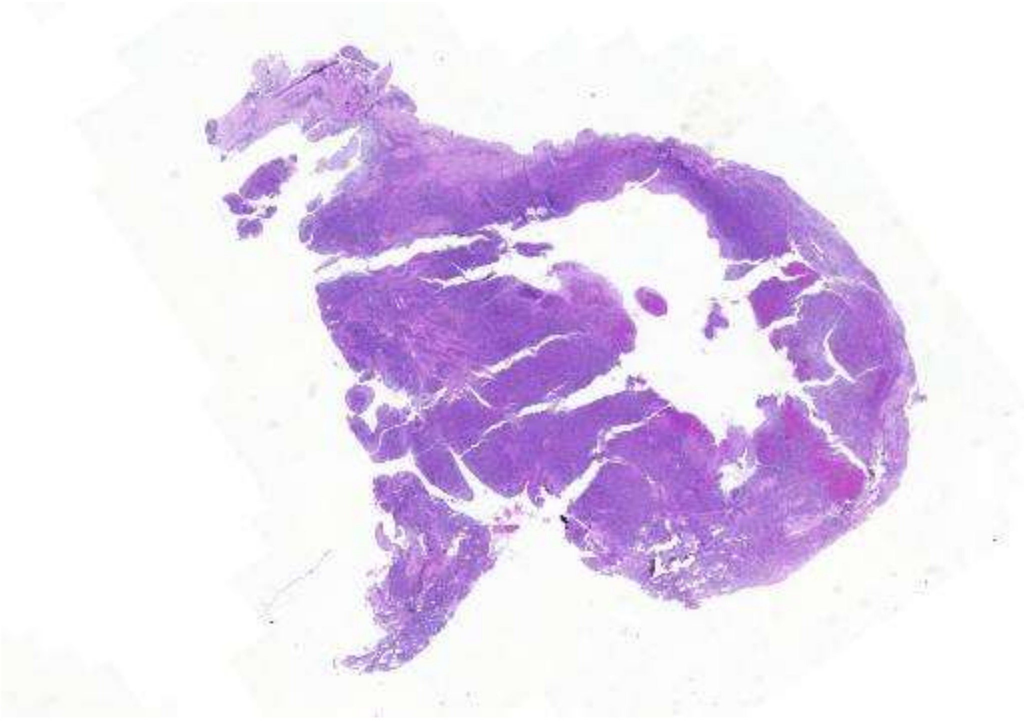

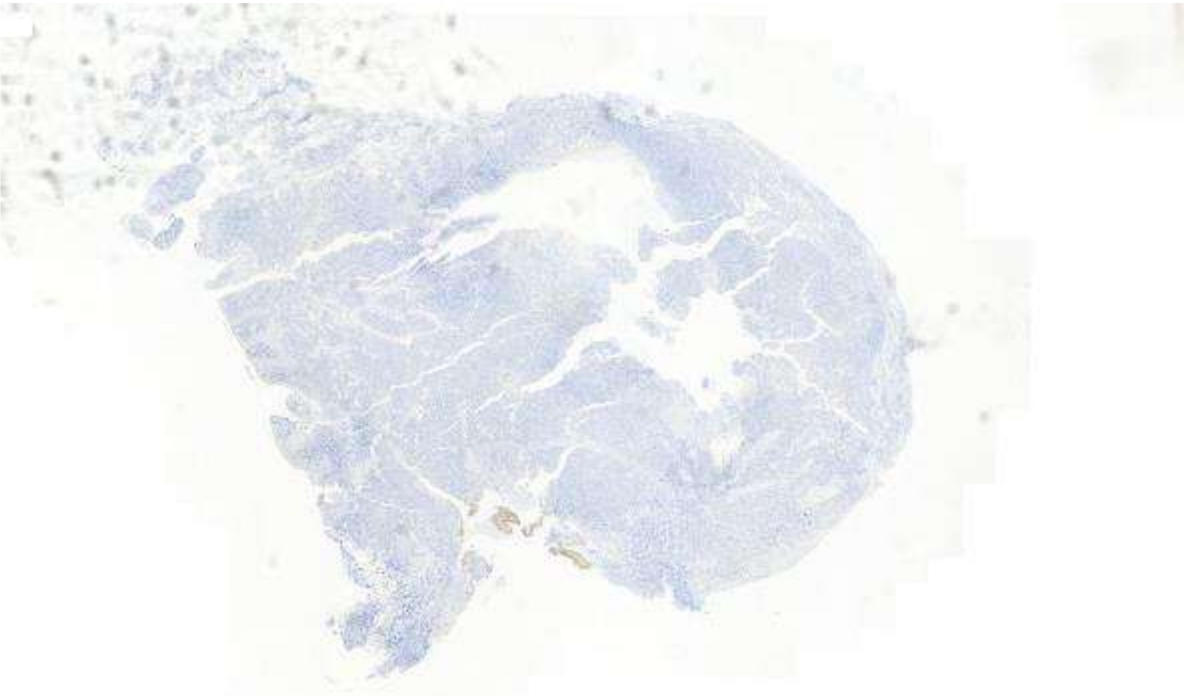

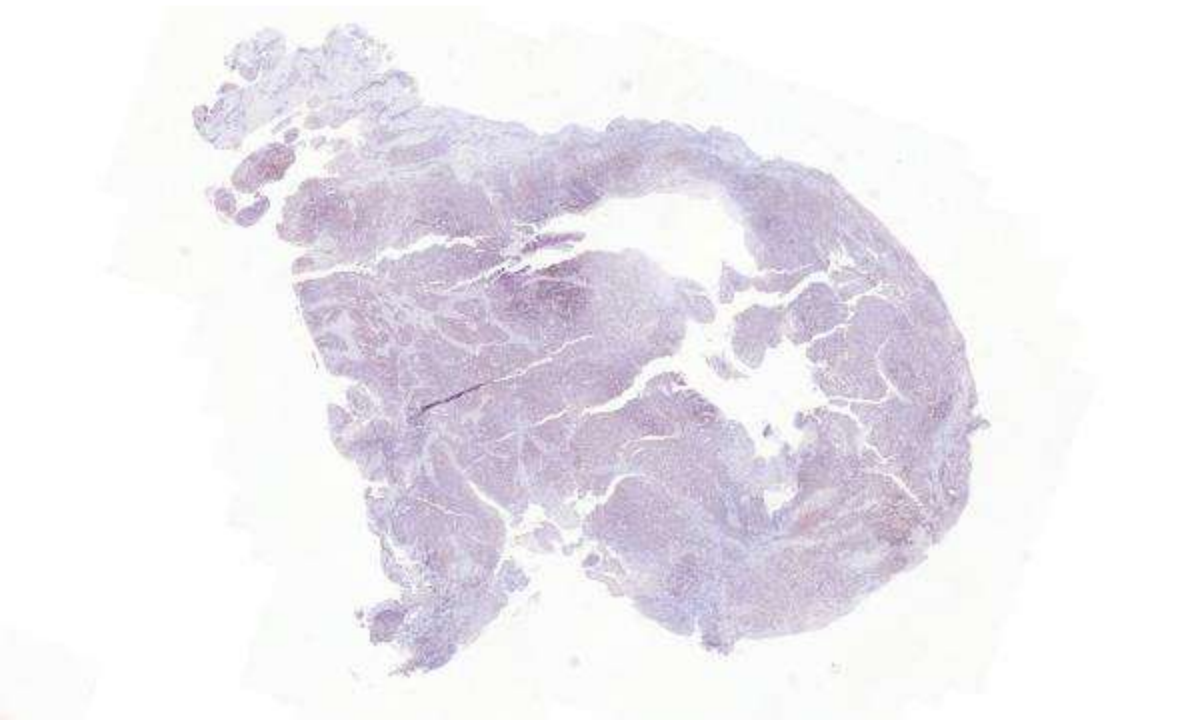

KR1 – HE, CK17, bcl2 (x5)

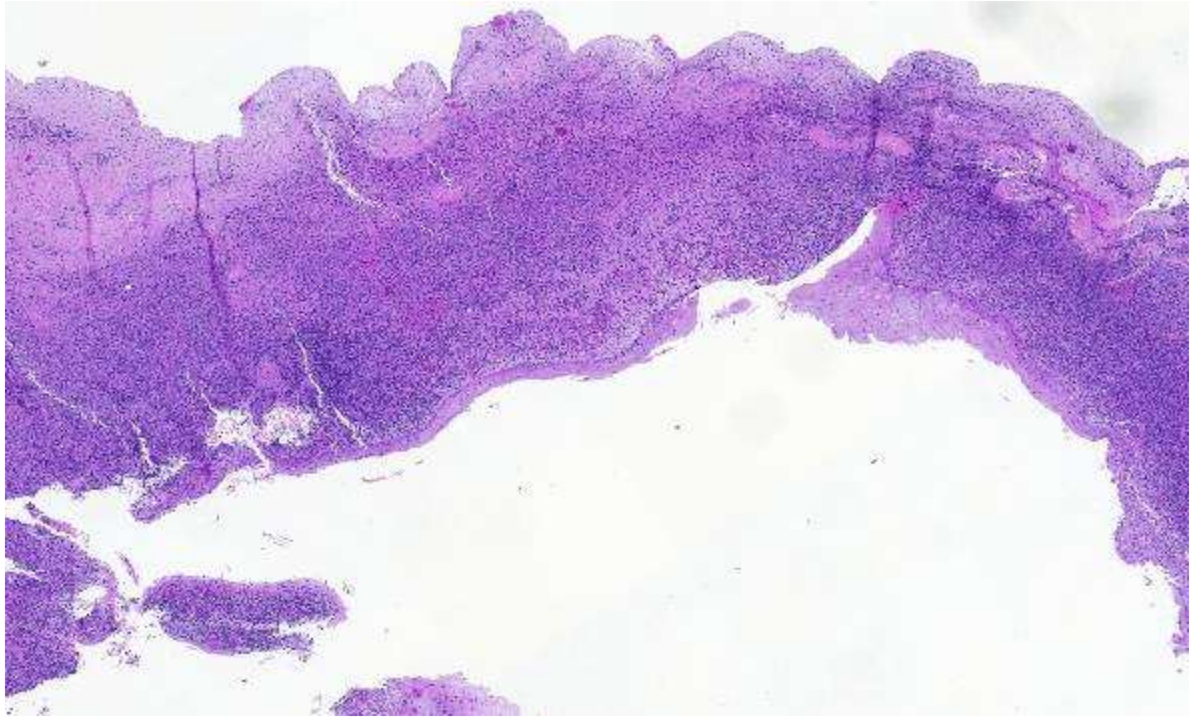

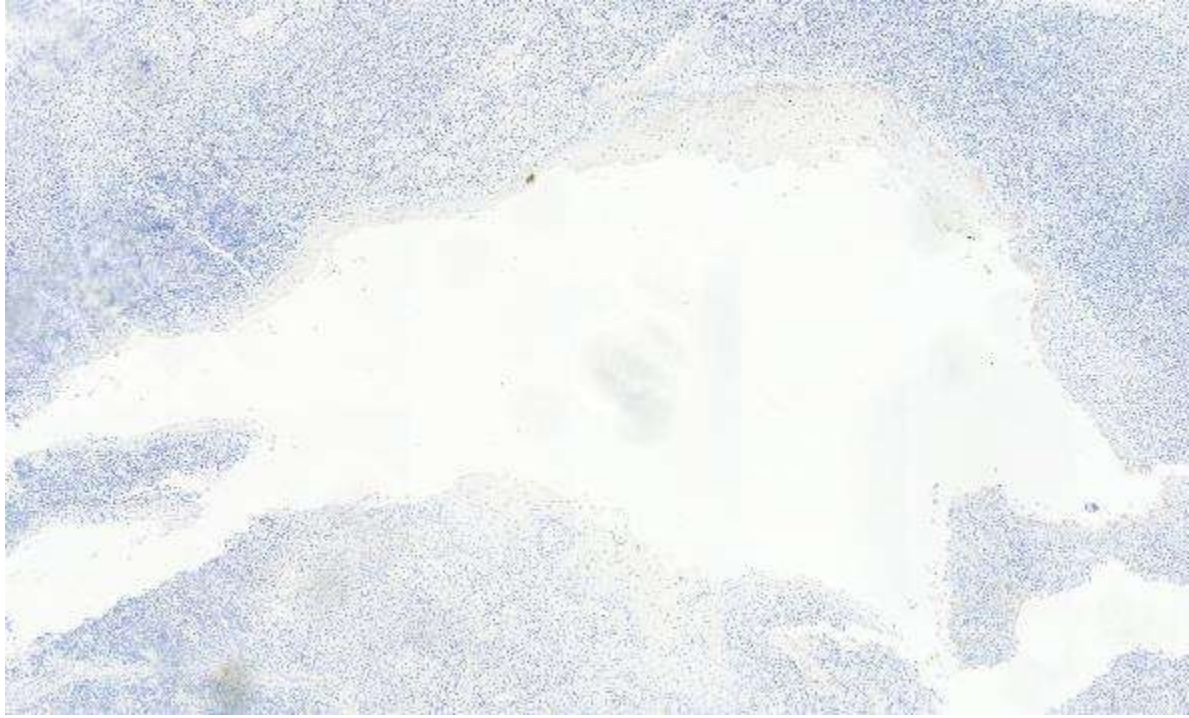

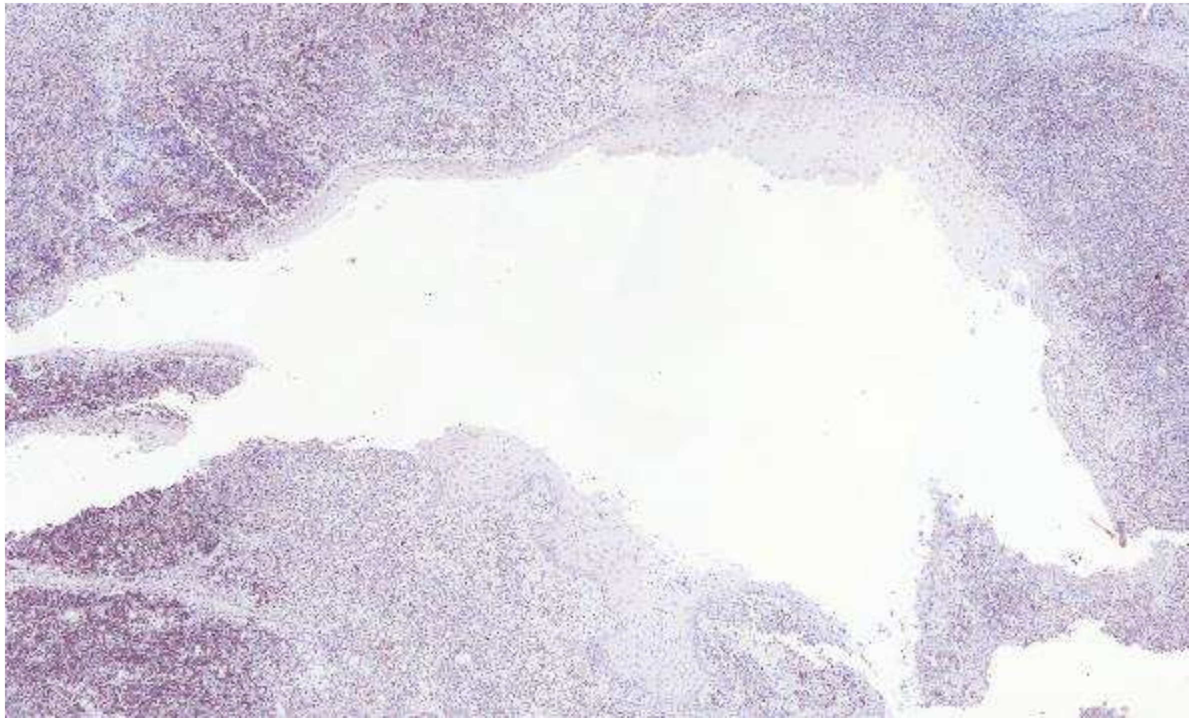

KR1 – HE, CK17, bcl2 (x10)

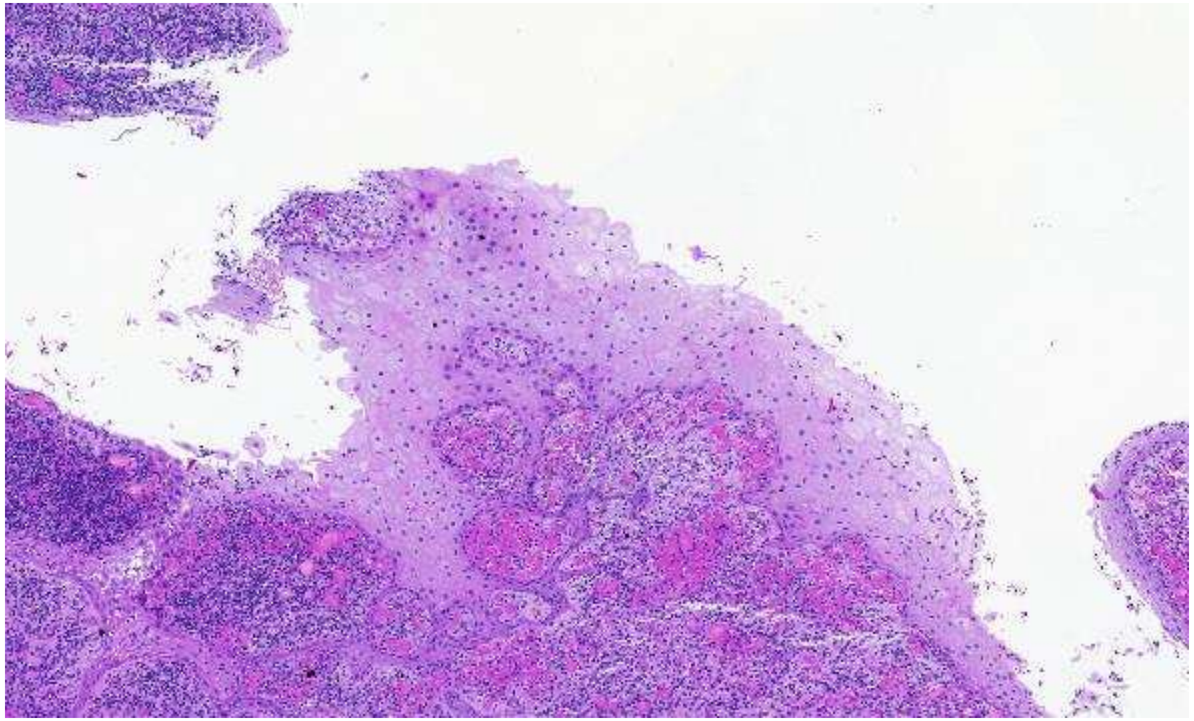

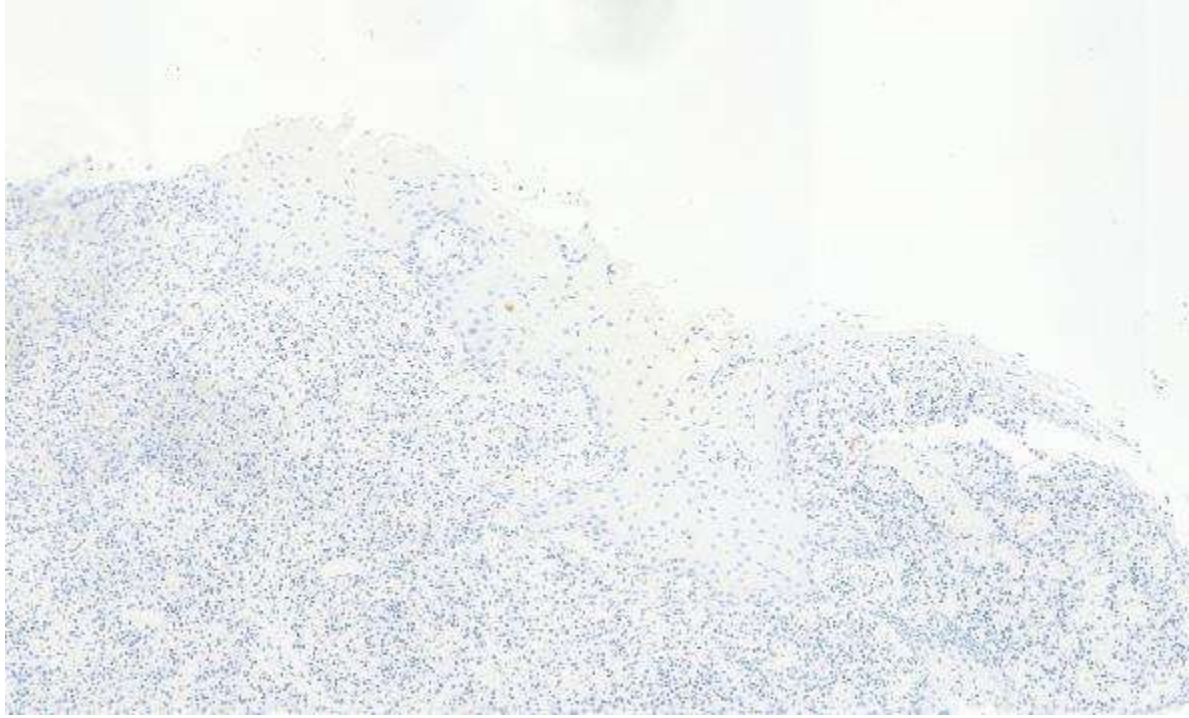

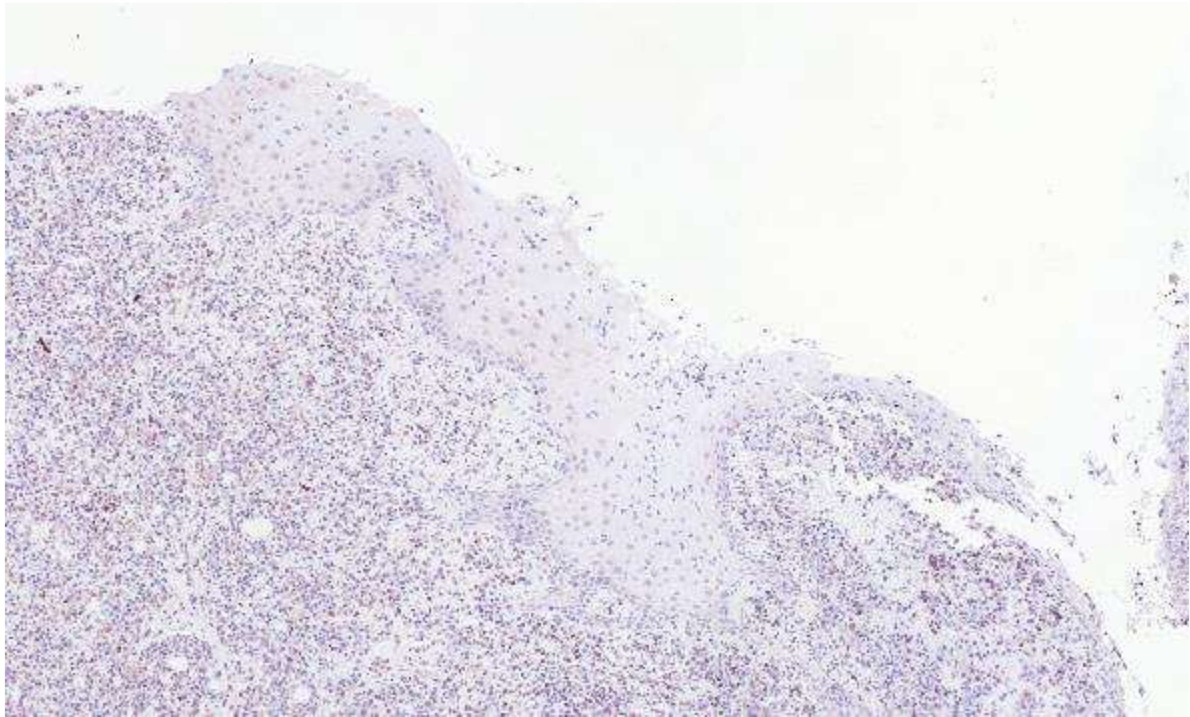

KR1 – HE, CK17, bcl2 (x10)

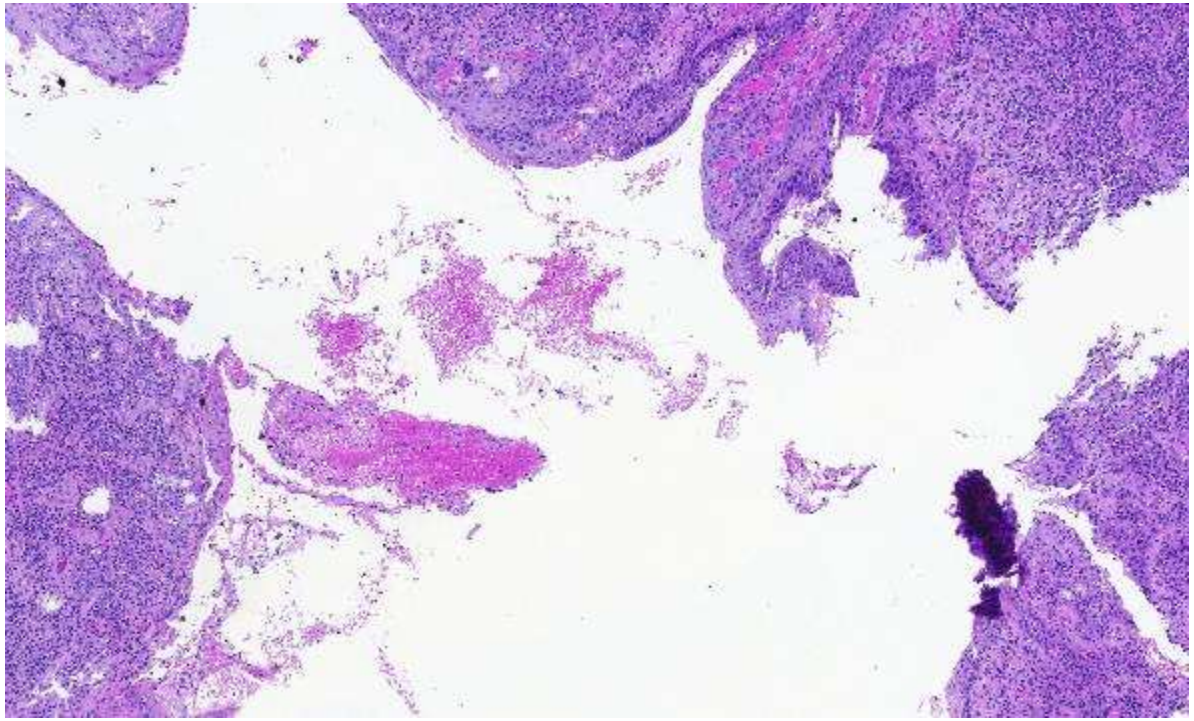

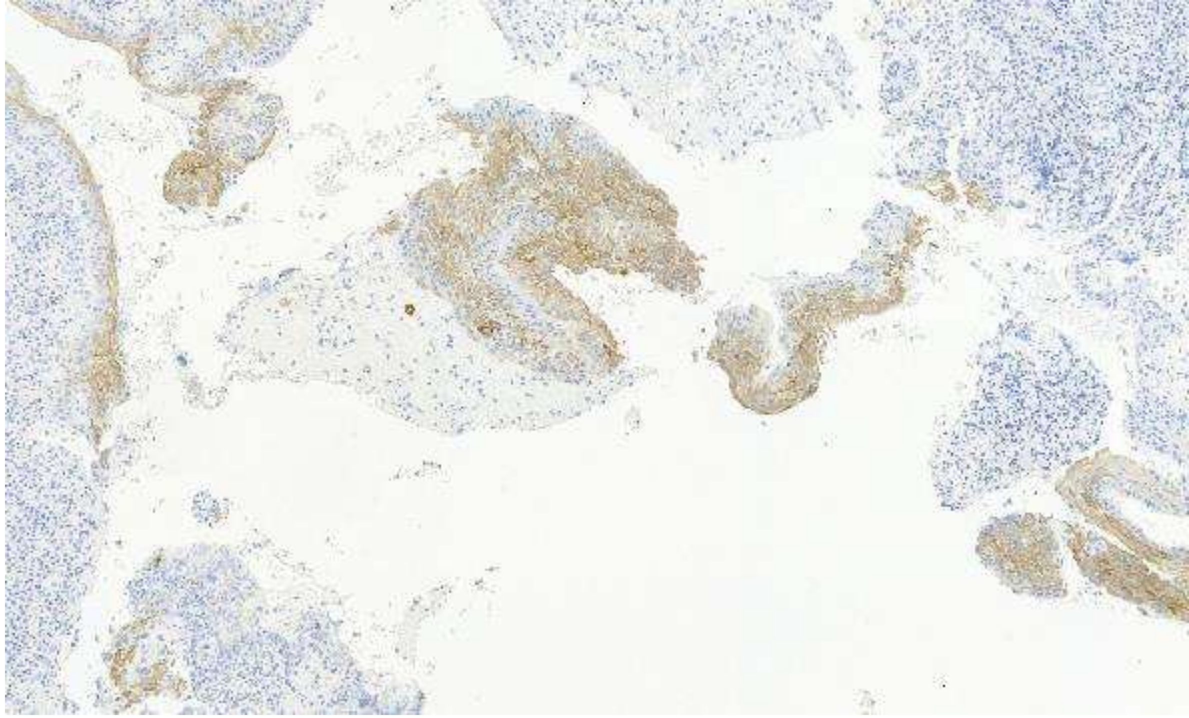

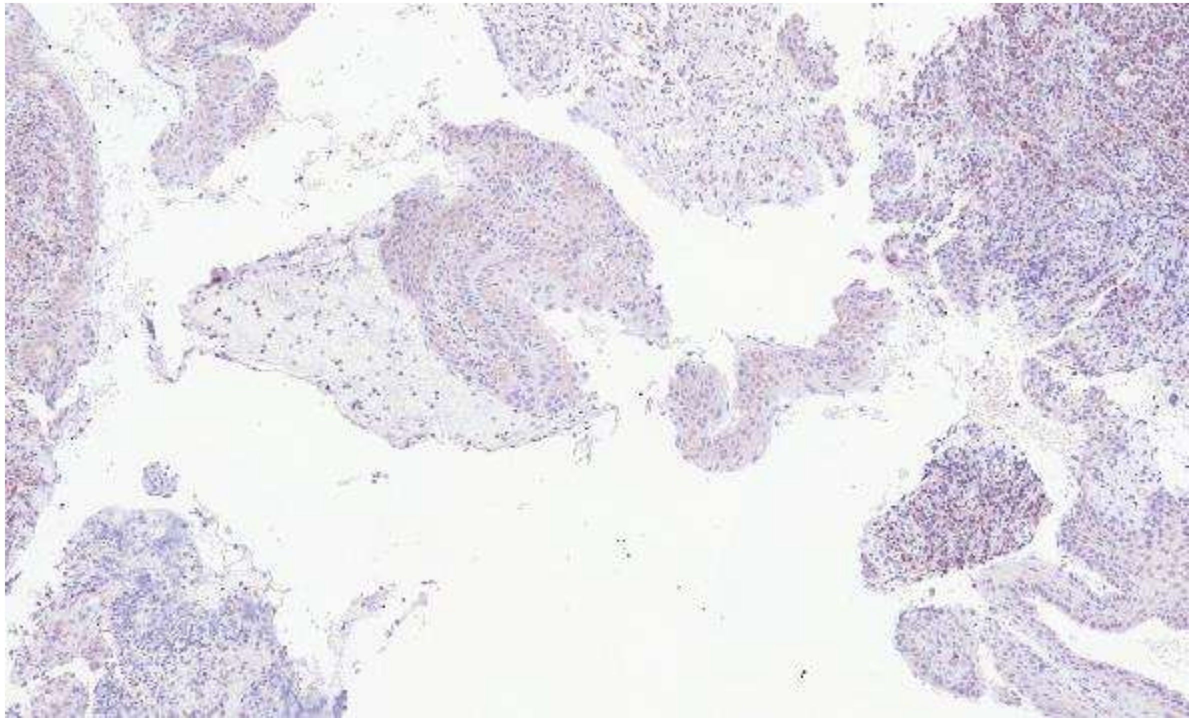

KR1 – Conclusion

- H&E: partly thick, partly thin squamous epithelium, no parakeratosis, focally wavy surface, no basal palisading, uneven to even epithelial-subepithelial contours
- Inflamed the cyst wall with mixed inflammatory infiltrate
- Bcl2: negative
- CK17: dominantly negative / with focal superficial to transepithelial staining dominantly with (or without) basal sparing
- Histopathology: radicular cyst
- Combined clinical and pathological opinion: radicular cyst

Case KR2

- Deleted because no evaluable amount of epithelium was present in the specimen

Case KR3

- 36-year-old female
- Dental hygiene/status: poor hygiene; maintained, condensed teeth, partial impaction of 18 and 28, complete impaction and horizontal position of 38 and 48. Preauricular tenderness corresponding to the mandibular condyle on both sides.
- Radiology: 5.5 mm radiolucency beneath the crown of tooth 38
- Operation: seropurulent excudate oozing out at incision, decoronation of impacted tooth 38, dissection and removal followed by the removal of the pericoronal cyst
- Clinical diagnosis: follicular (dentigerous) cyst

KR3 – OPT

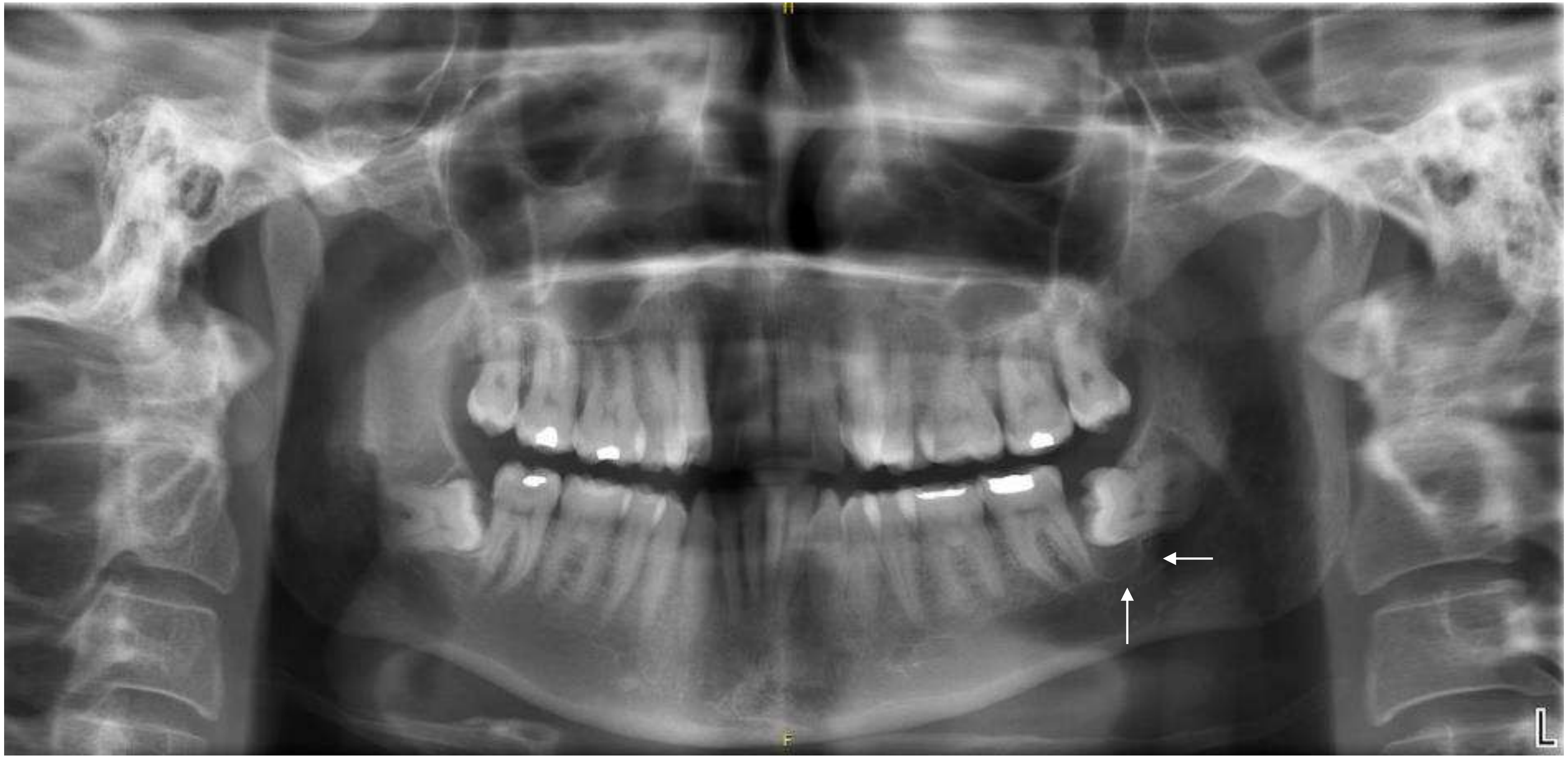

KR3 – OPT magnified

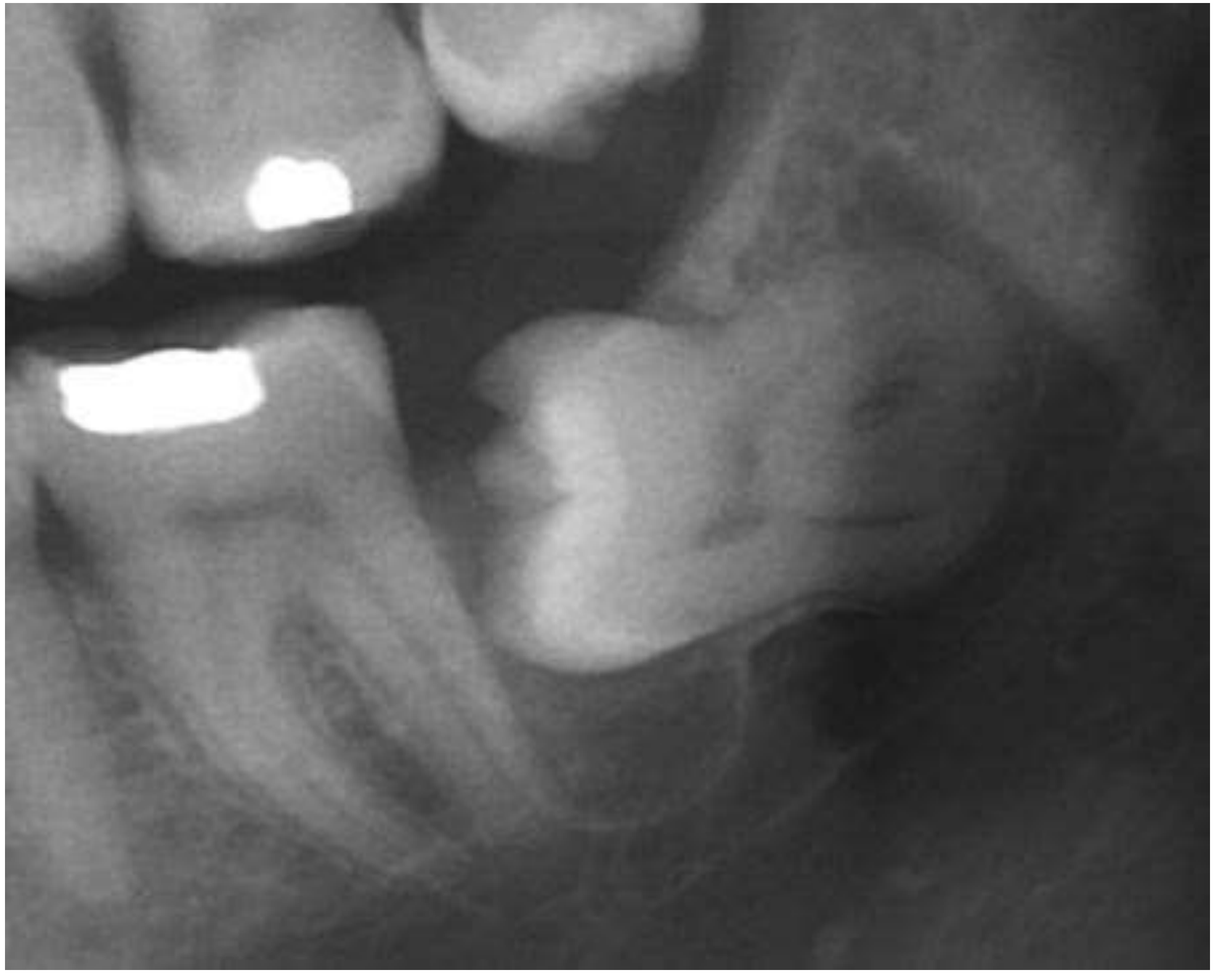

KR3 – CT coronal (slices 98-100-102/286)

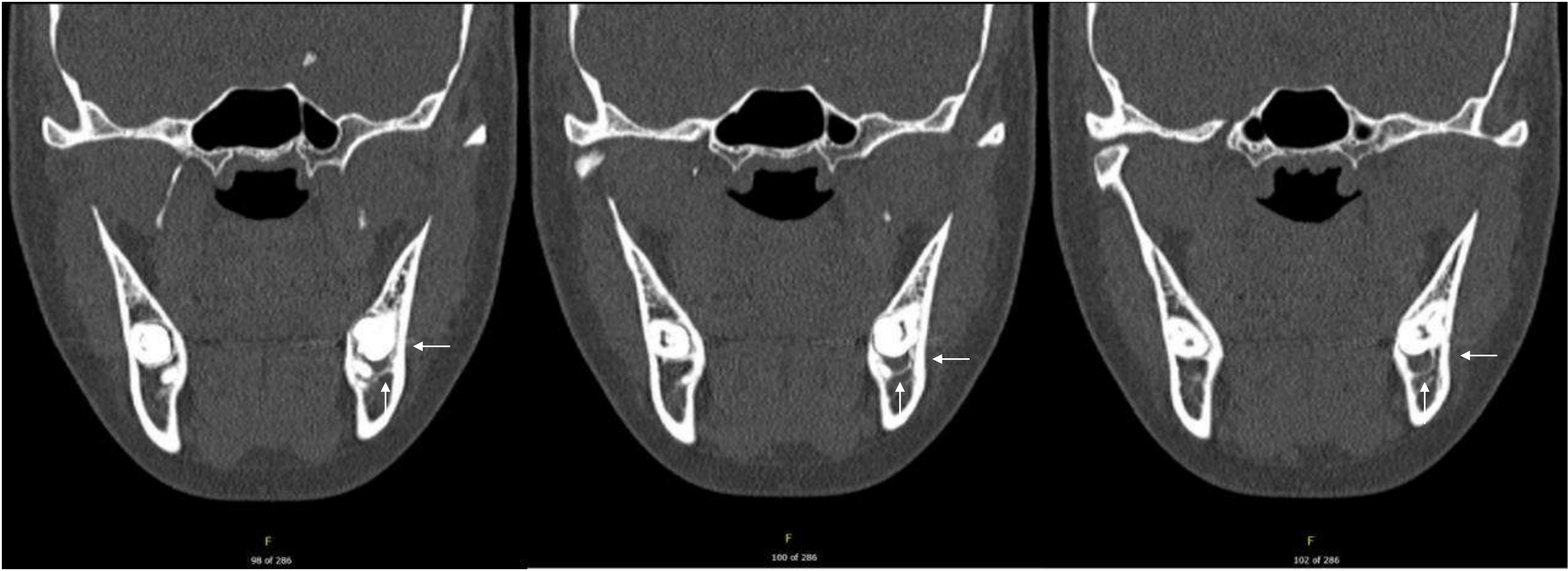

KR3 – CT axial (slices 68-71-73-75/211)

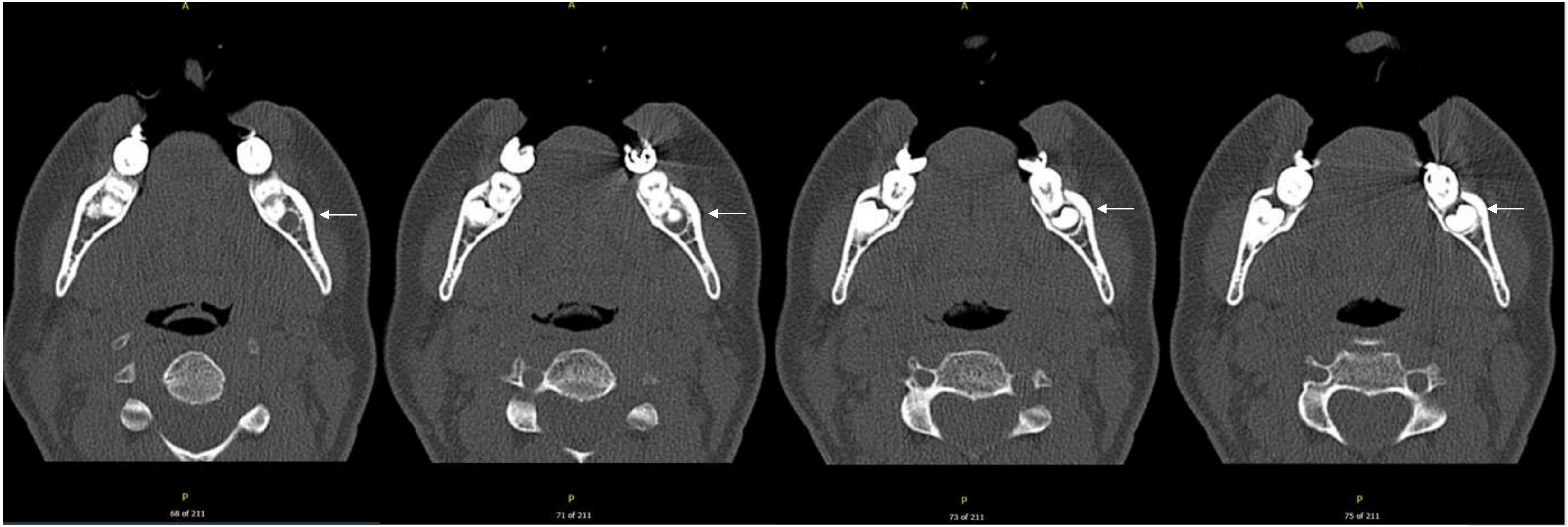

KR3 – CT sagittal (slices 100-102/286)

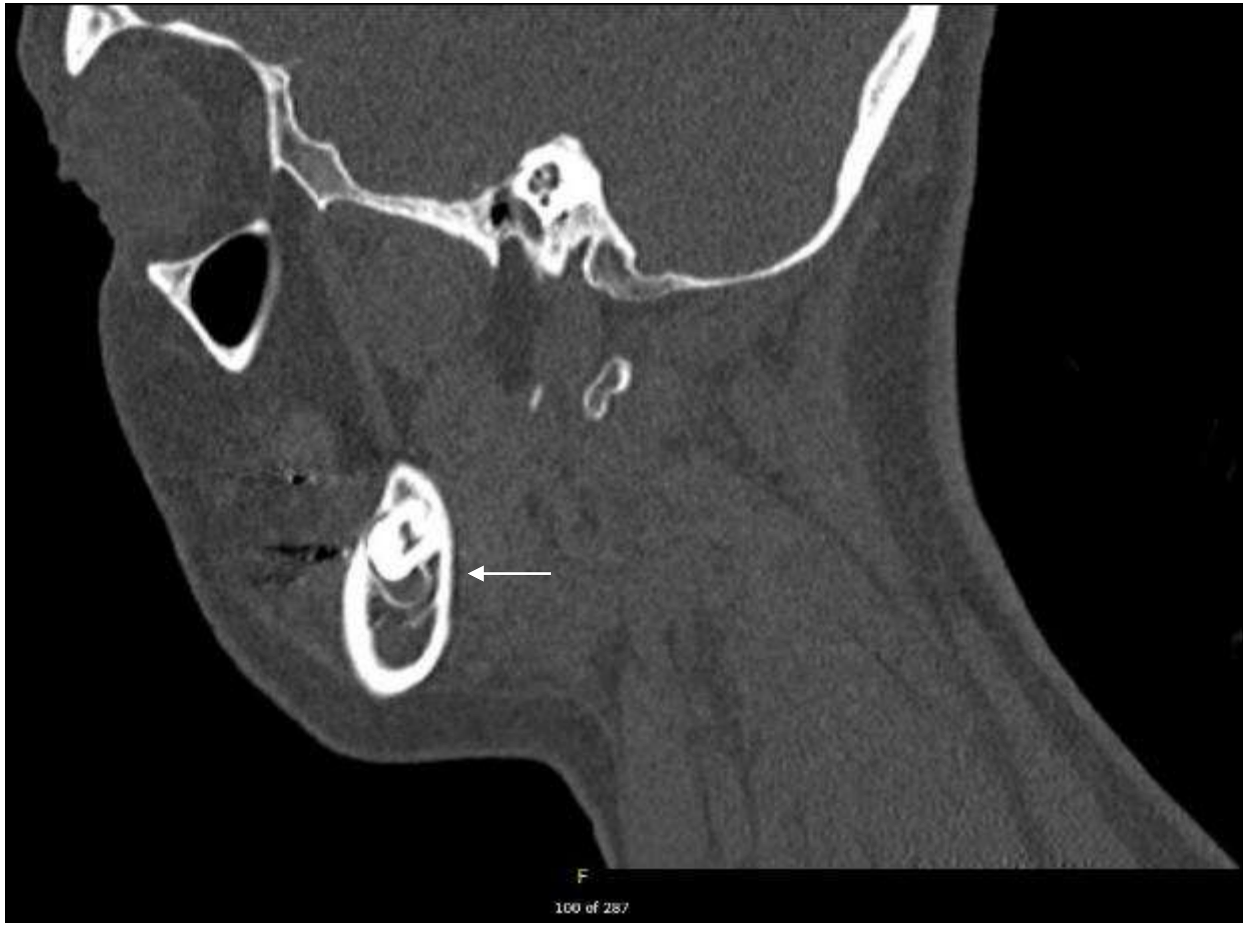

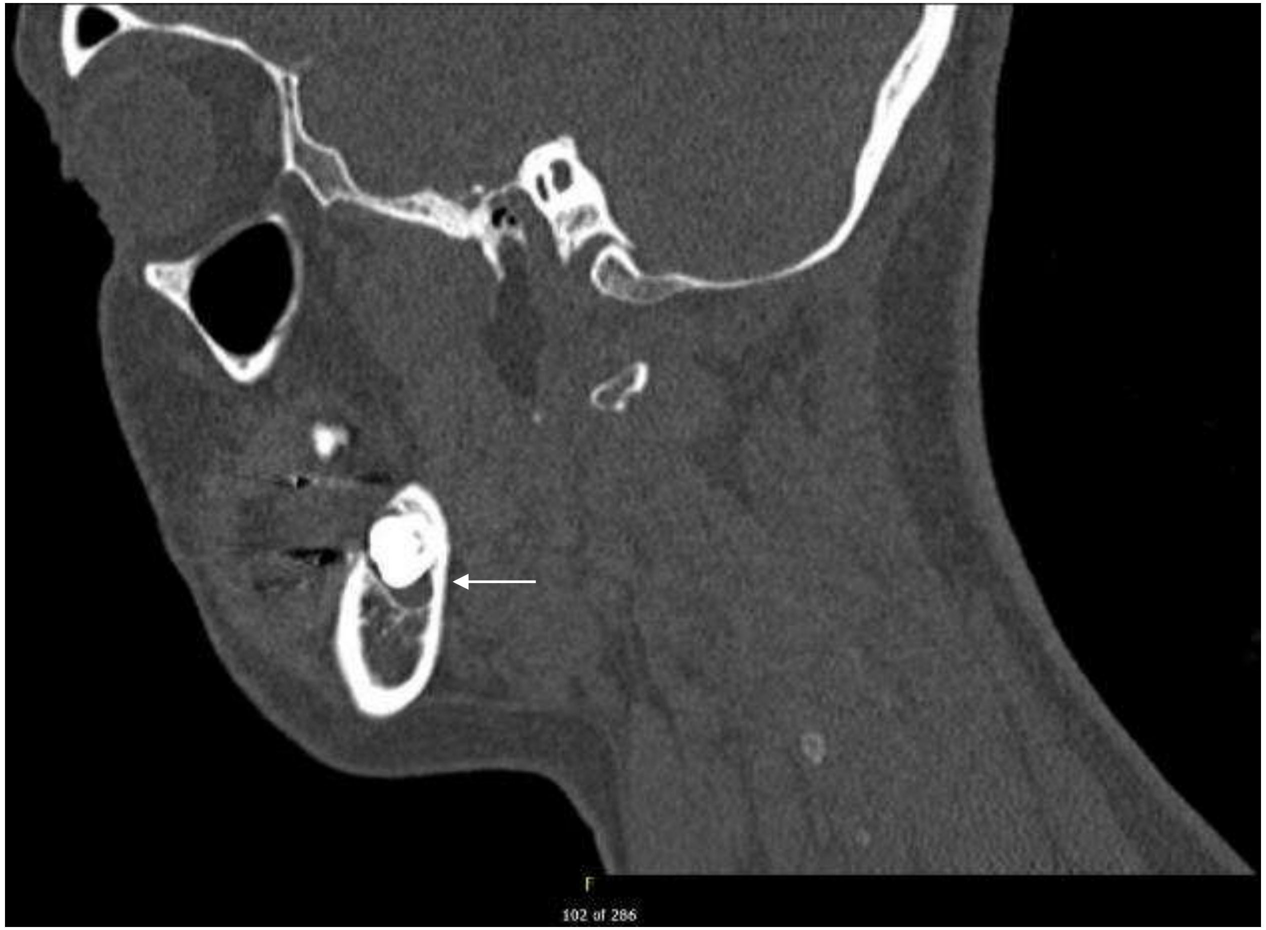

KR3 – HE (x20, x35)

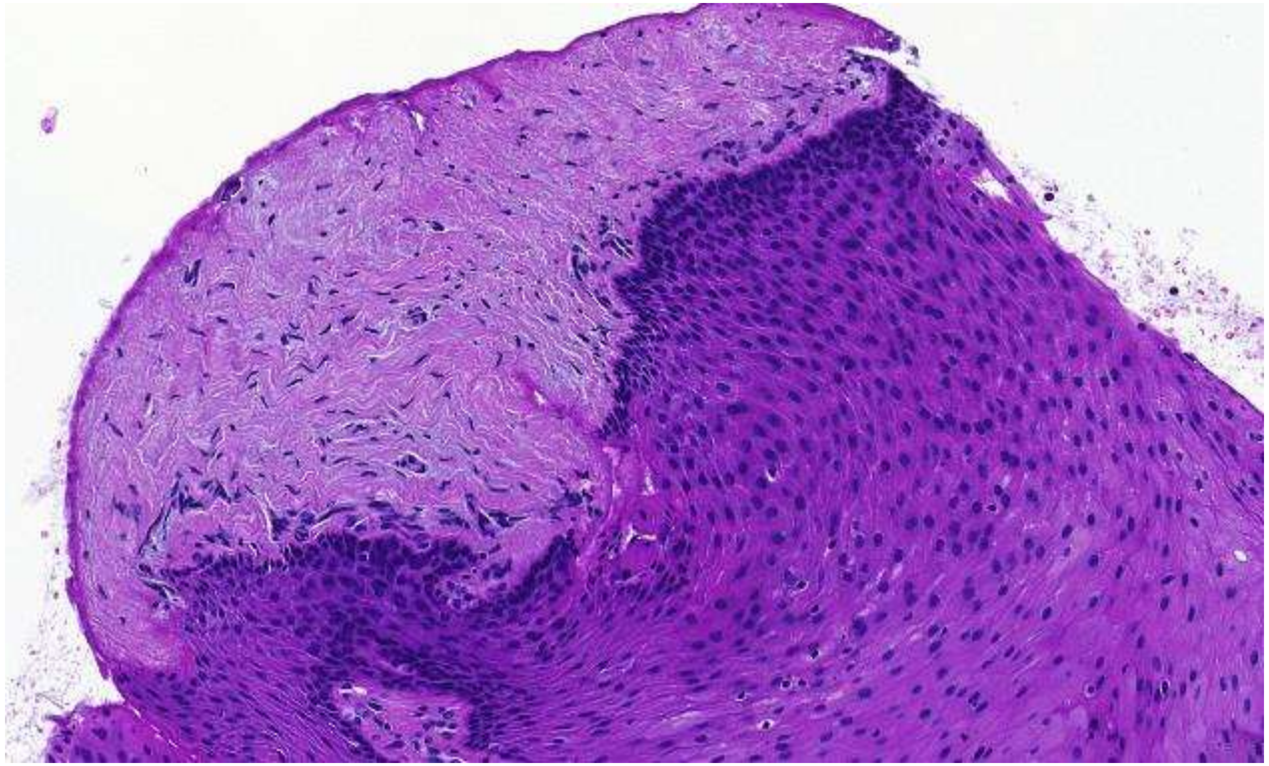

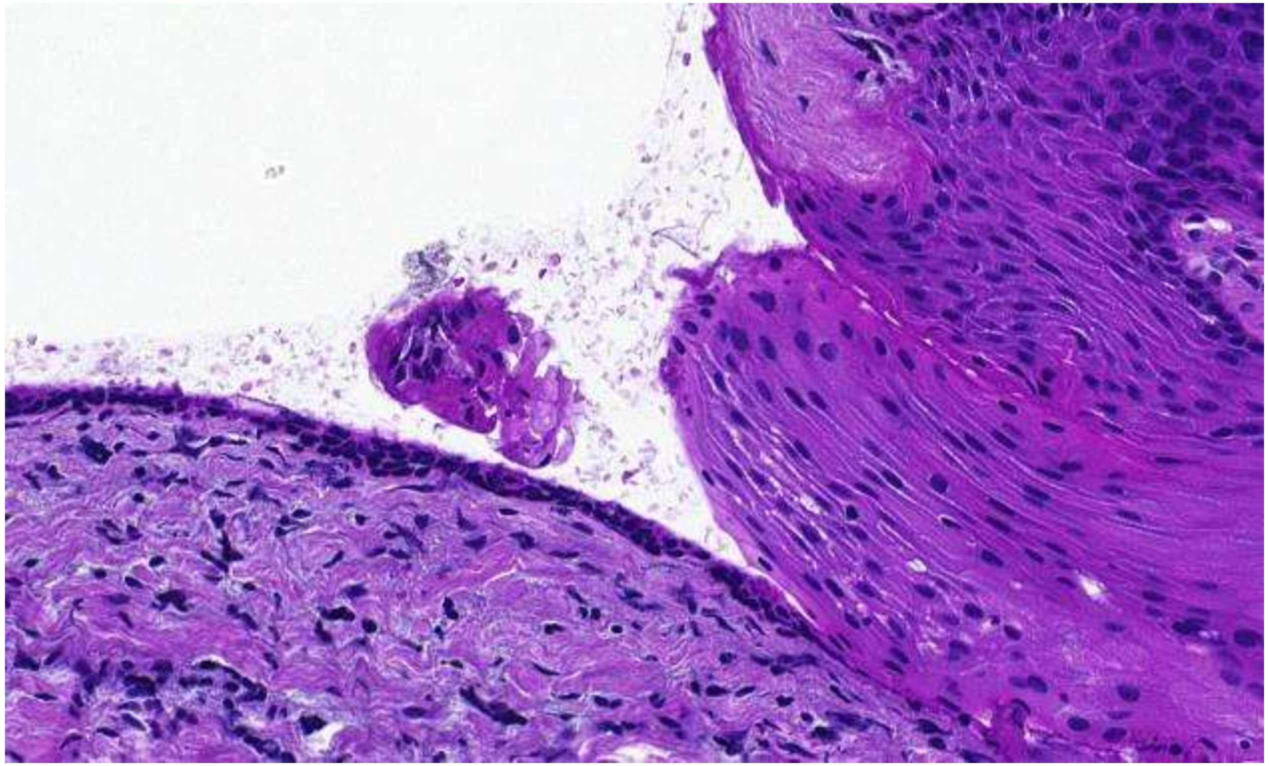

KR3 – CK17 (x20, x35)

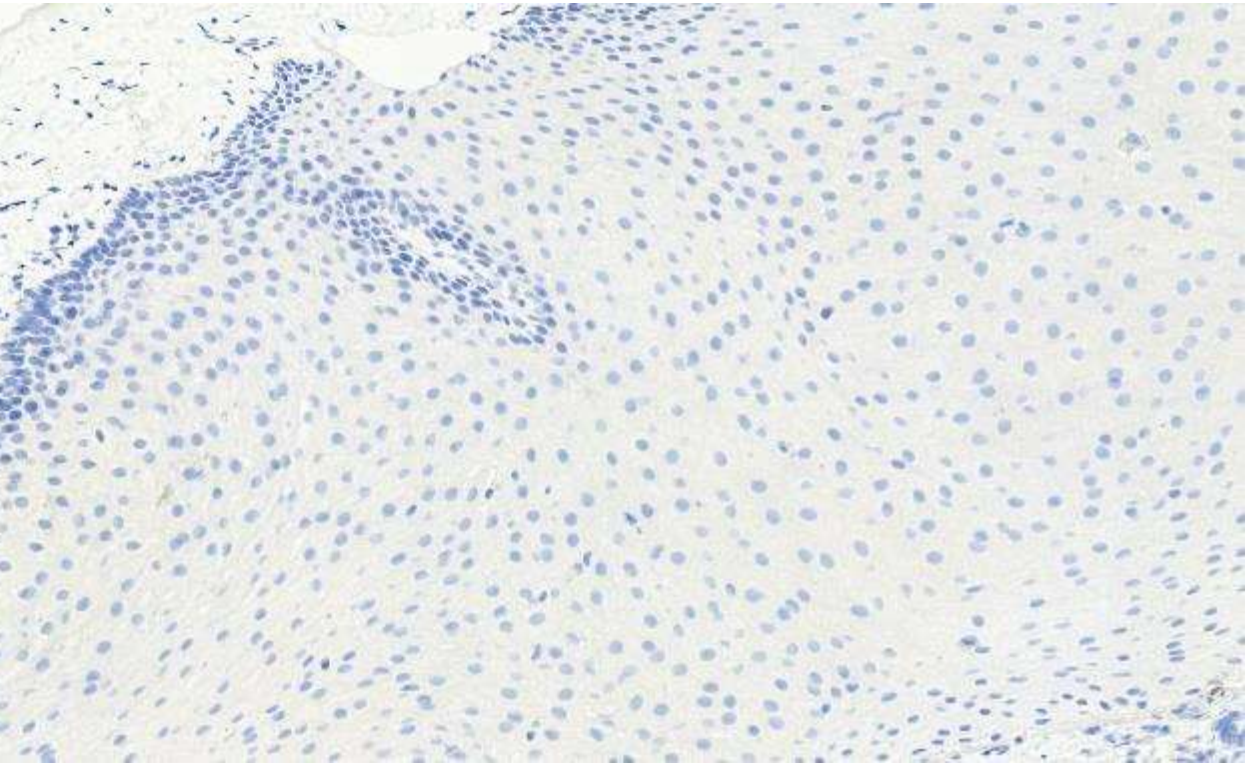

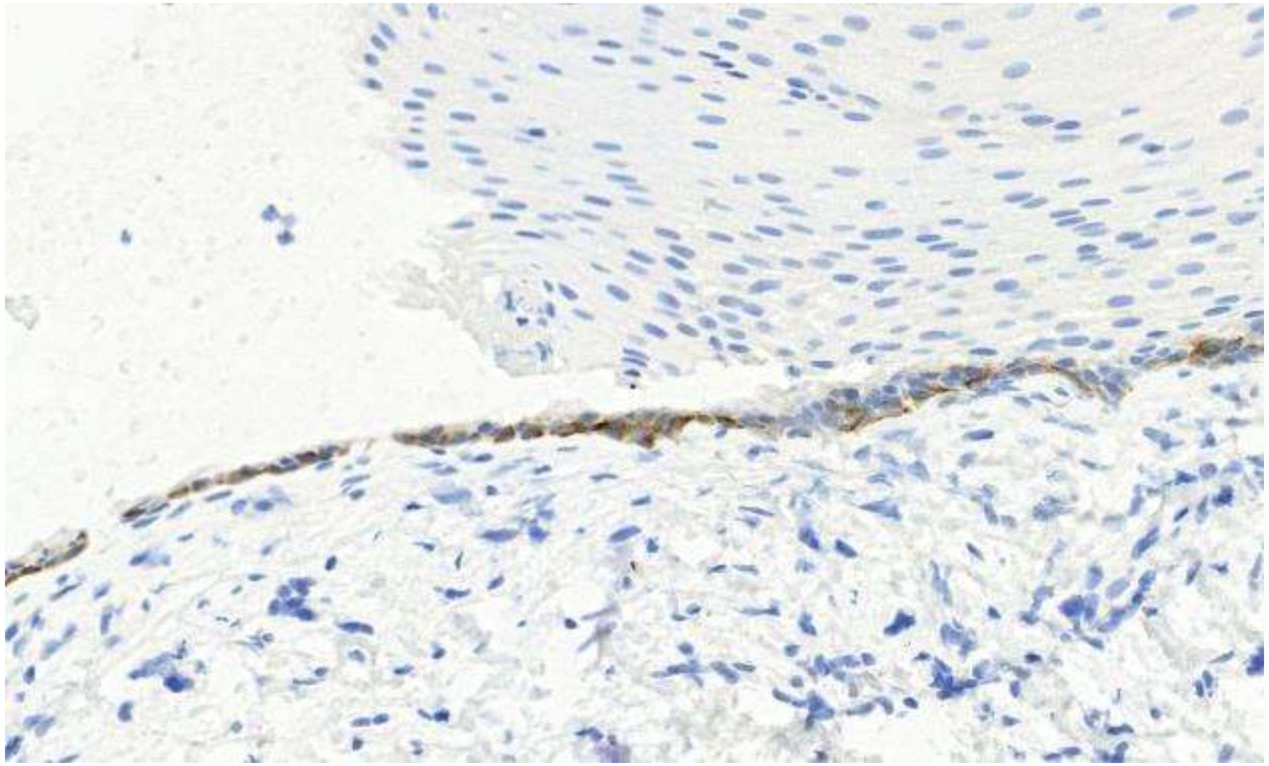

KR3 – bcl2 (x20, x35) focal weak basal staining

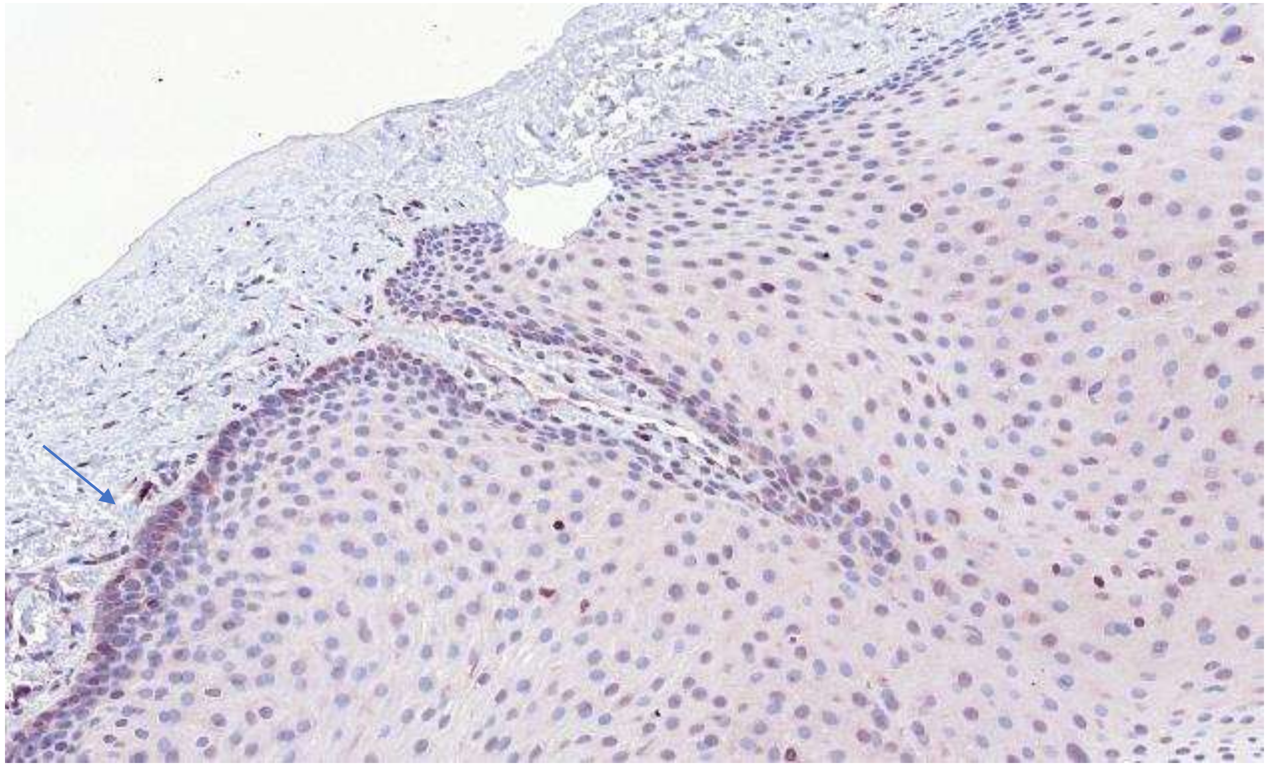

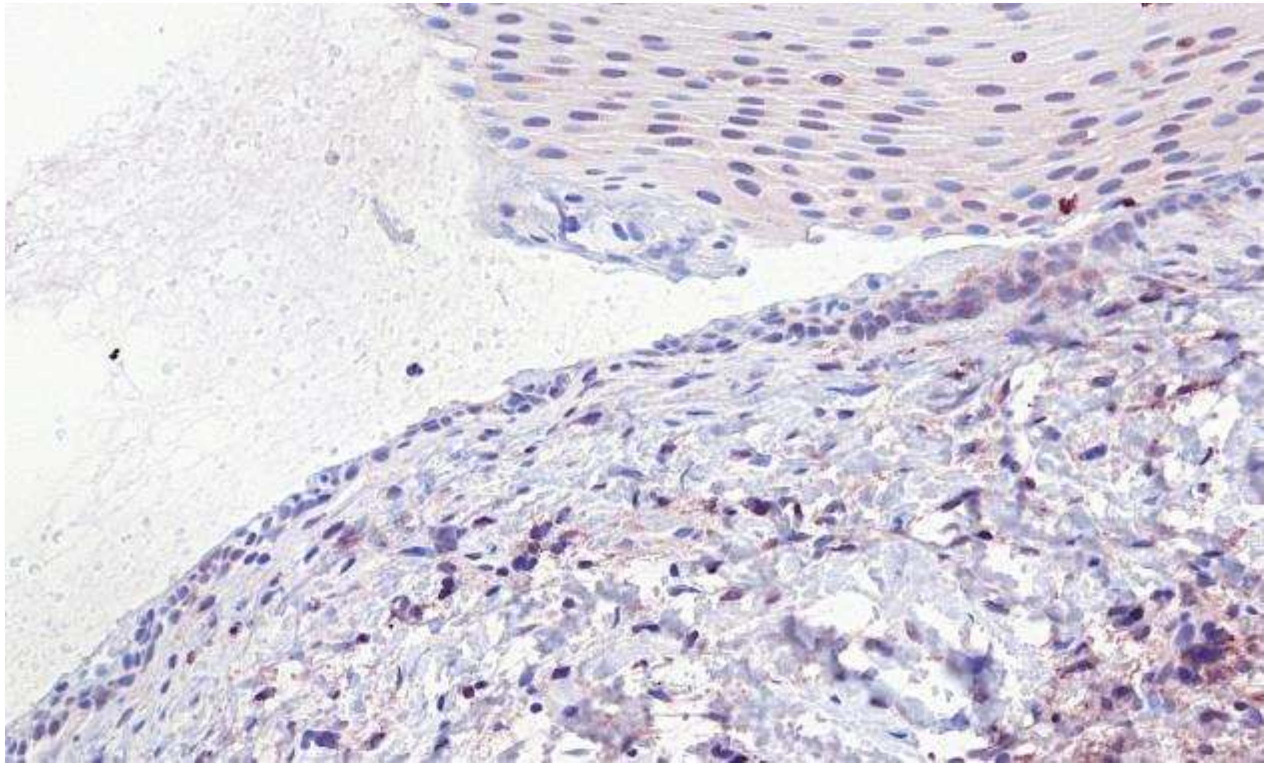

KR3 – Conclusion

- H&E: relatively thick squamous non-kertinizing epithelium, no parakeratosis, no wavy surface, no basal palisading, uneven epithelial-subepithelial contours with rete pegs
- Non-inflamed cyst, with minimal focal chronic inflammation of the cyst wall away from the epithelium
- Bcl2: negative, focal weak basal staining
- CK17: negative, minimal focal basal staining at a nearly completely denuded focus
- Histopathology: inflammatory odontogenic cyst (radicular cyst)
- Combined clinical and pathological opinion: dentigerous (follicular) cyst

Case KR4

- 33-year-old male
- Dental hygiene/status: poor hygiene; missing crowns of teeth 17,16,26,27,44 with roots in the alveoli, extensive caries of teeth 11, 21, 34, 43, caries of teeth 12, 22. Gingival hyperaemia.
- Radiology: periapical radiolucent lesion of tooth 44
- Operation: removal of residual roots and cystectomy with blunt preparation
- Clinical diagnosis: periapical cyst

KR4 – OPT

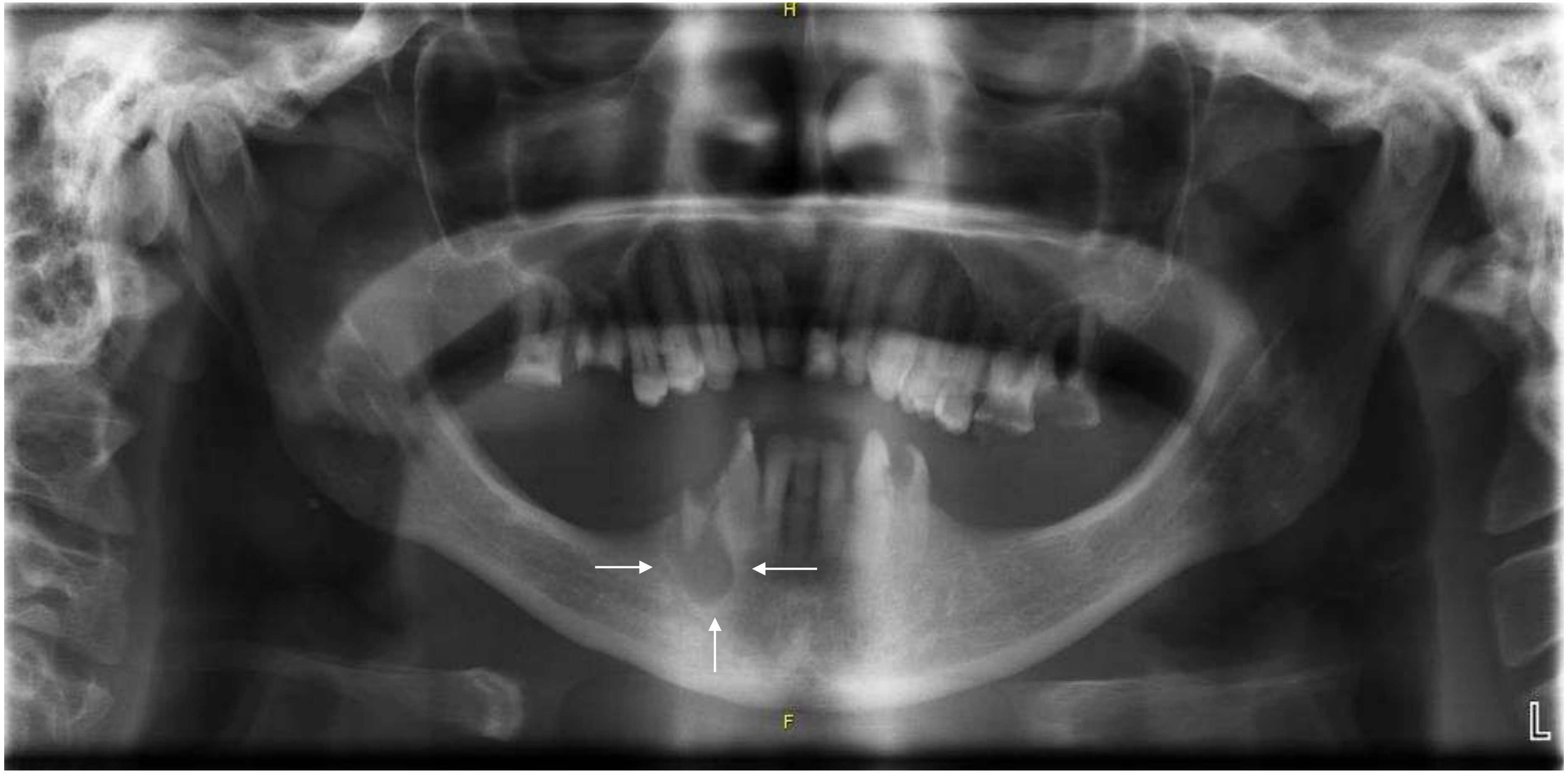

KR4 – OPT magnified

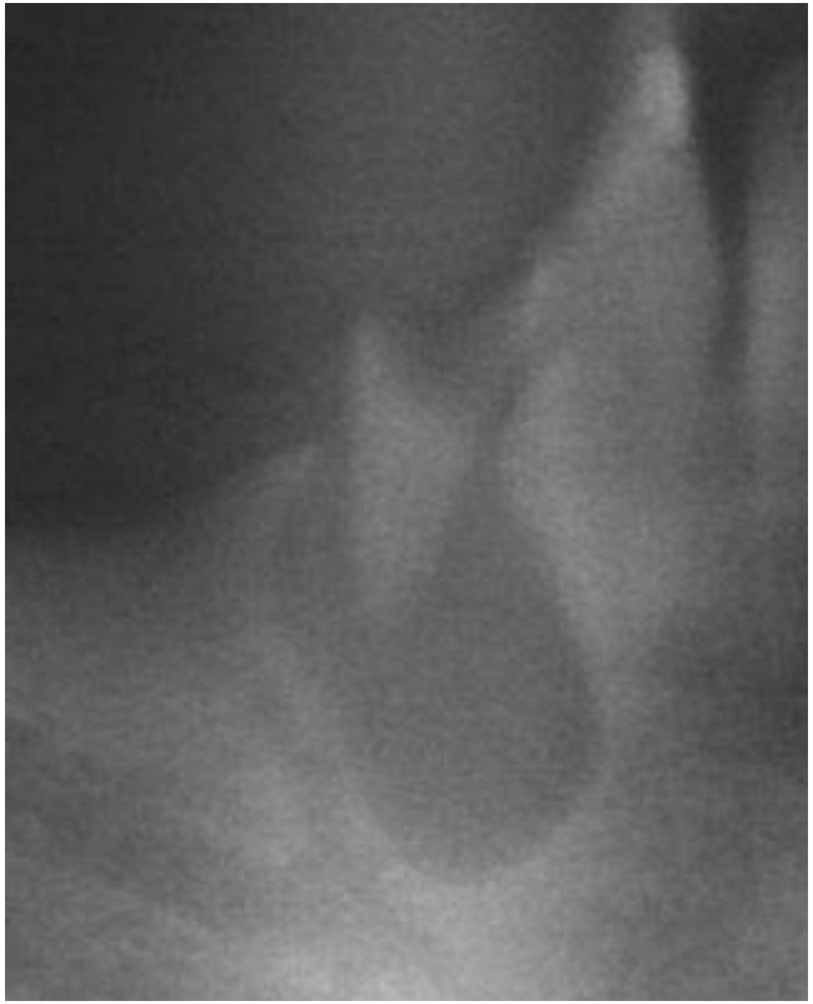

KR4 – HE, CK17, bcl2 (x1.4)

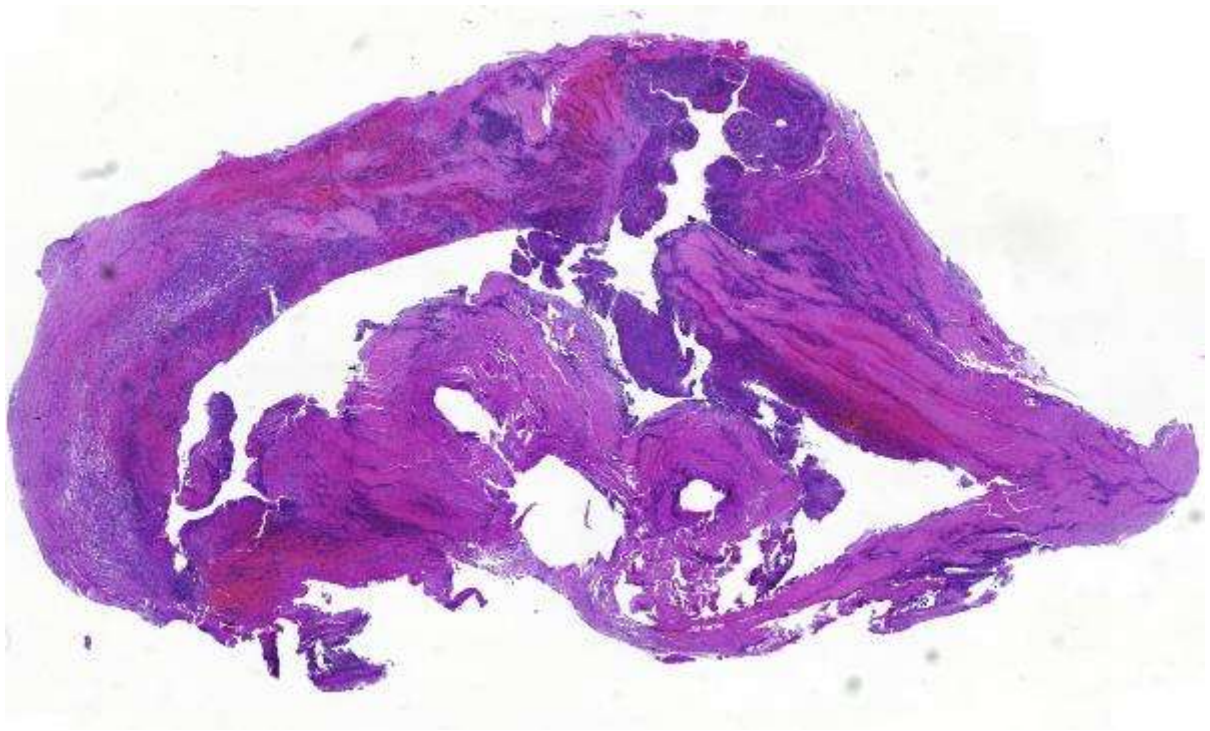

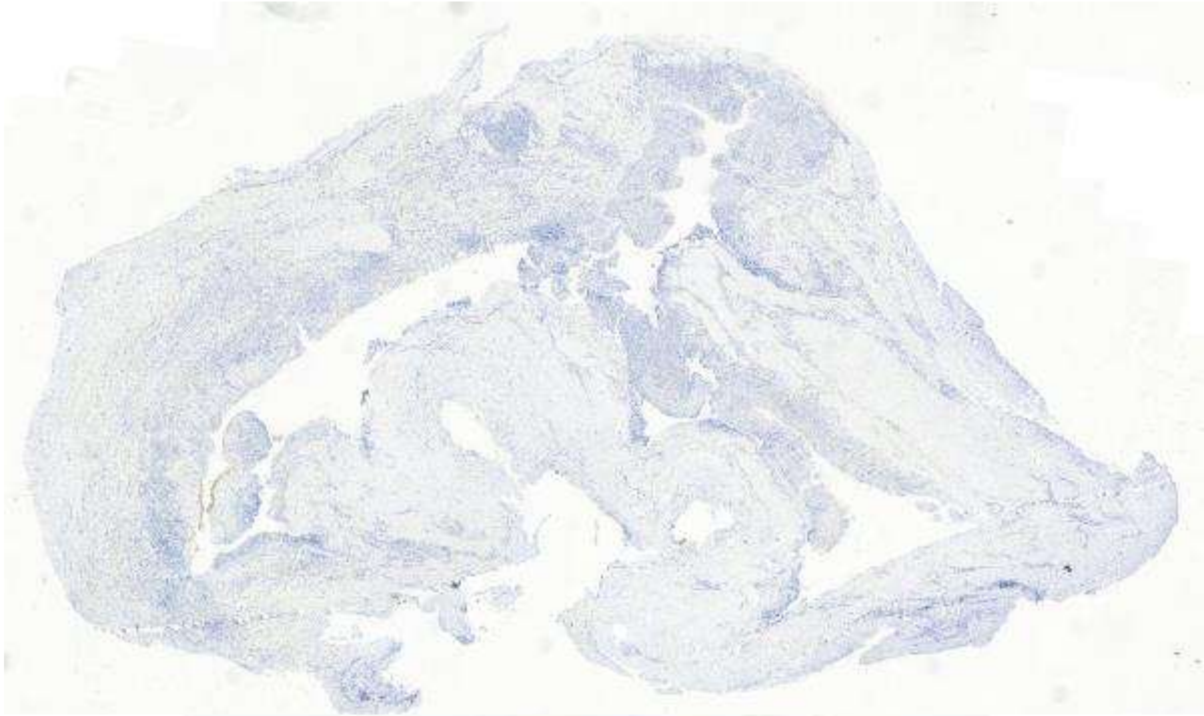

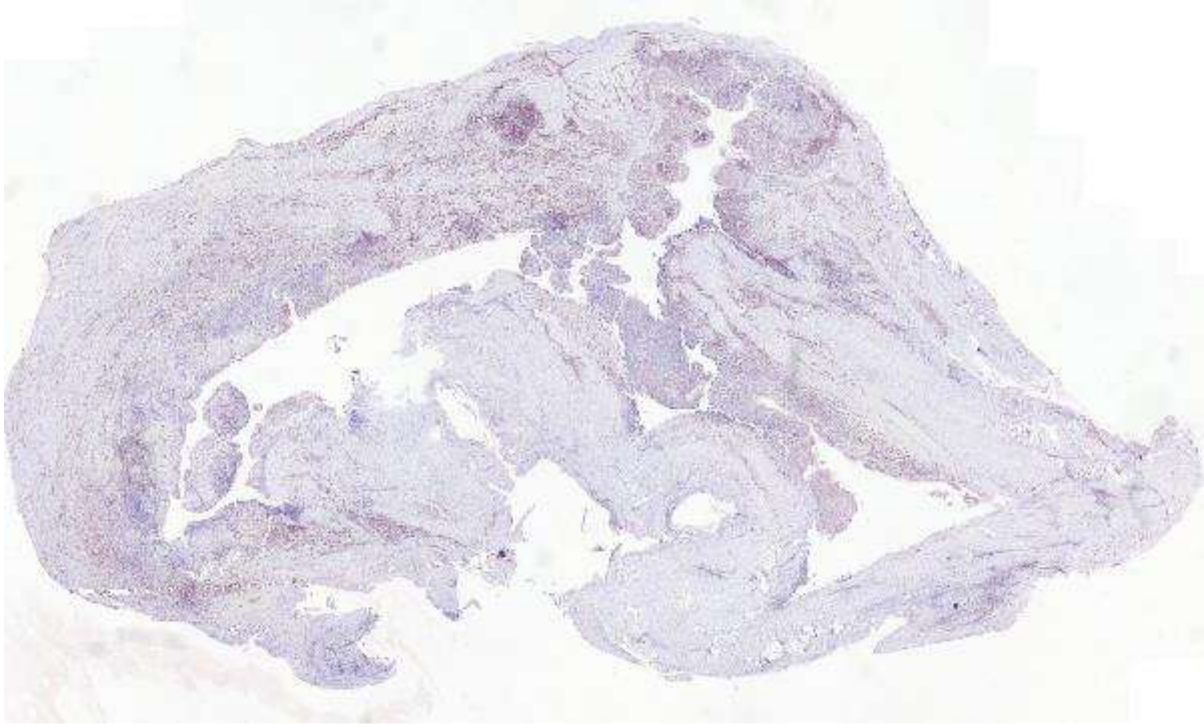

KR4 – HE, CK17, bcl2 (x5)

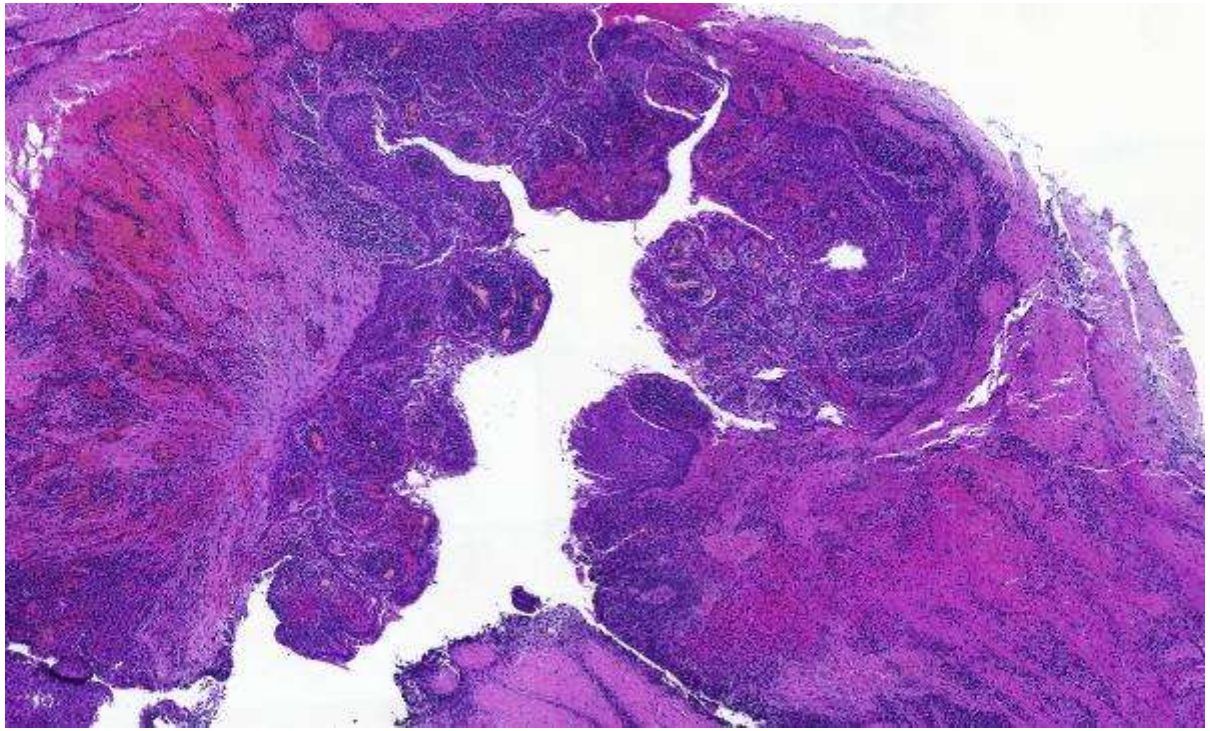

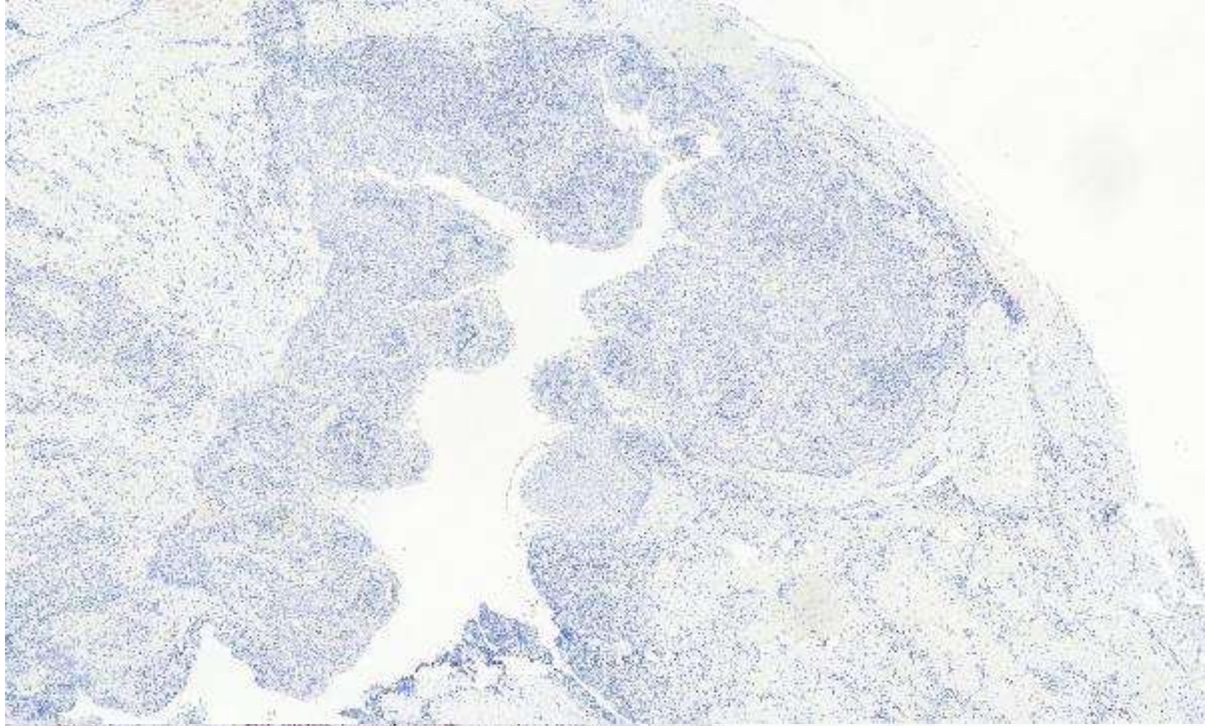

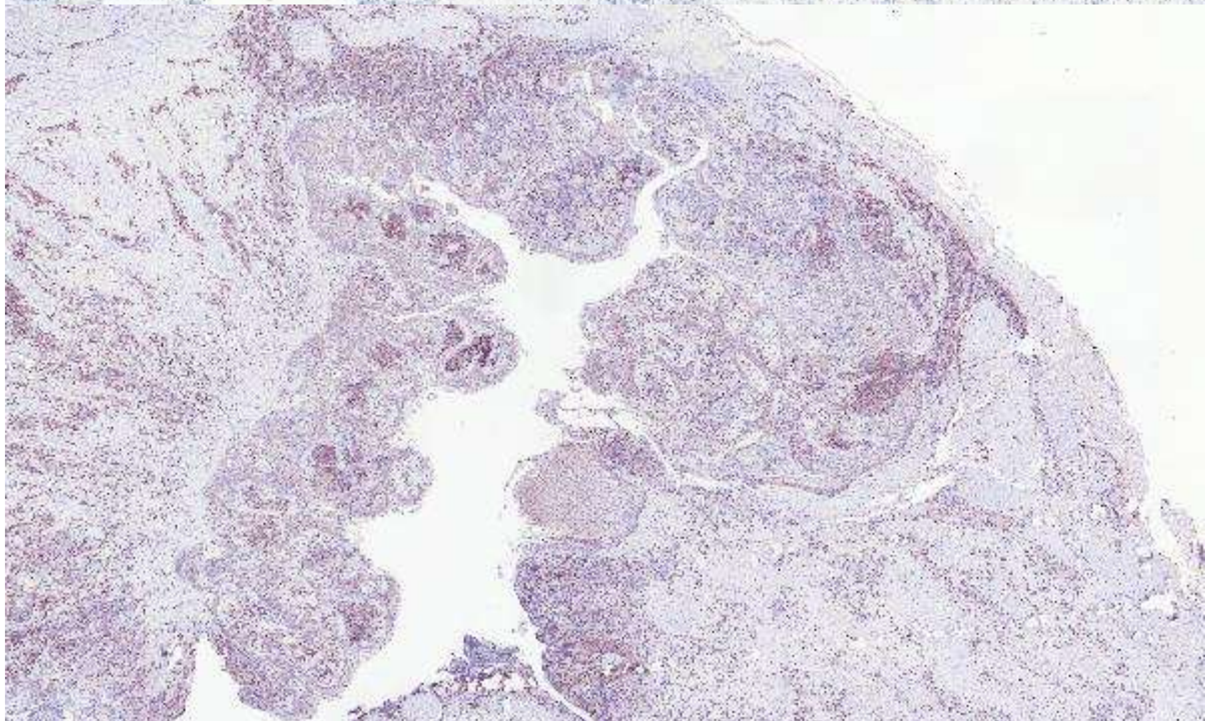

KR4 – HE, CK17, bcl2 (x20)

Minimal focal superficial CK17 staining

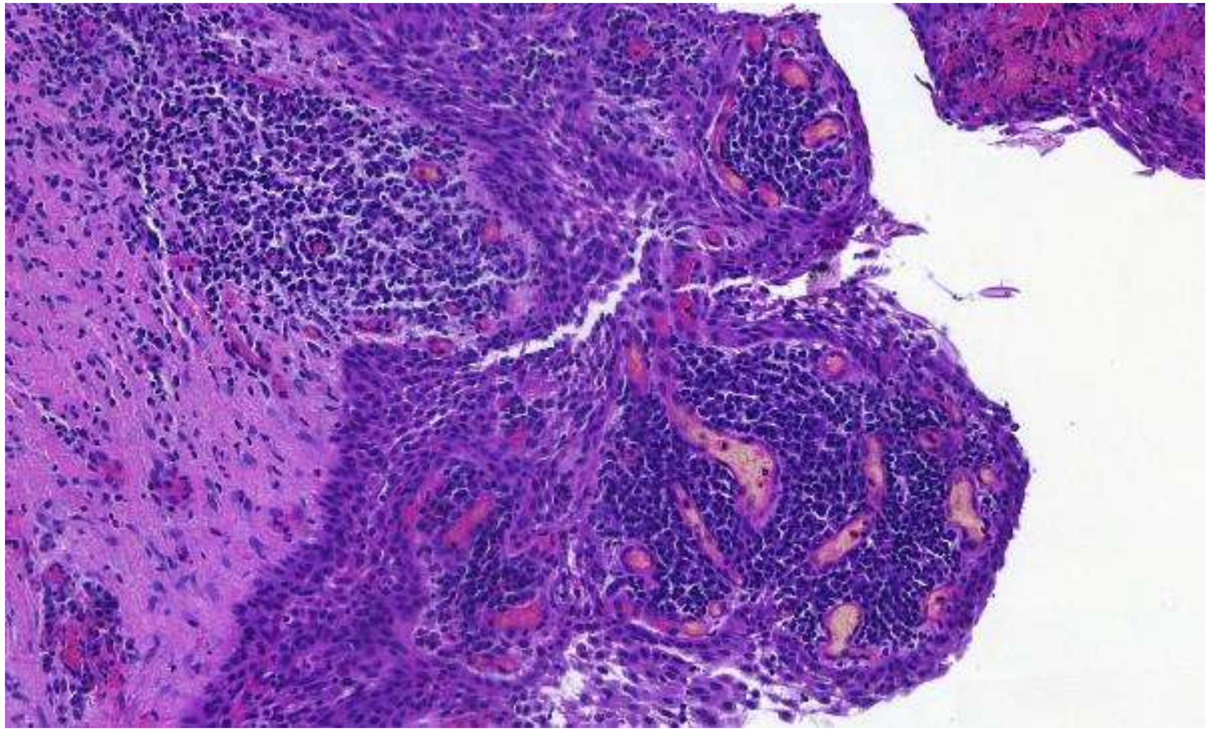

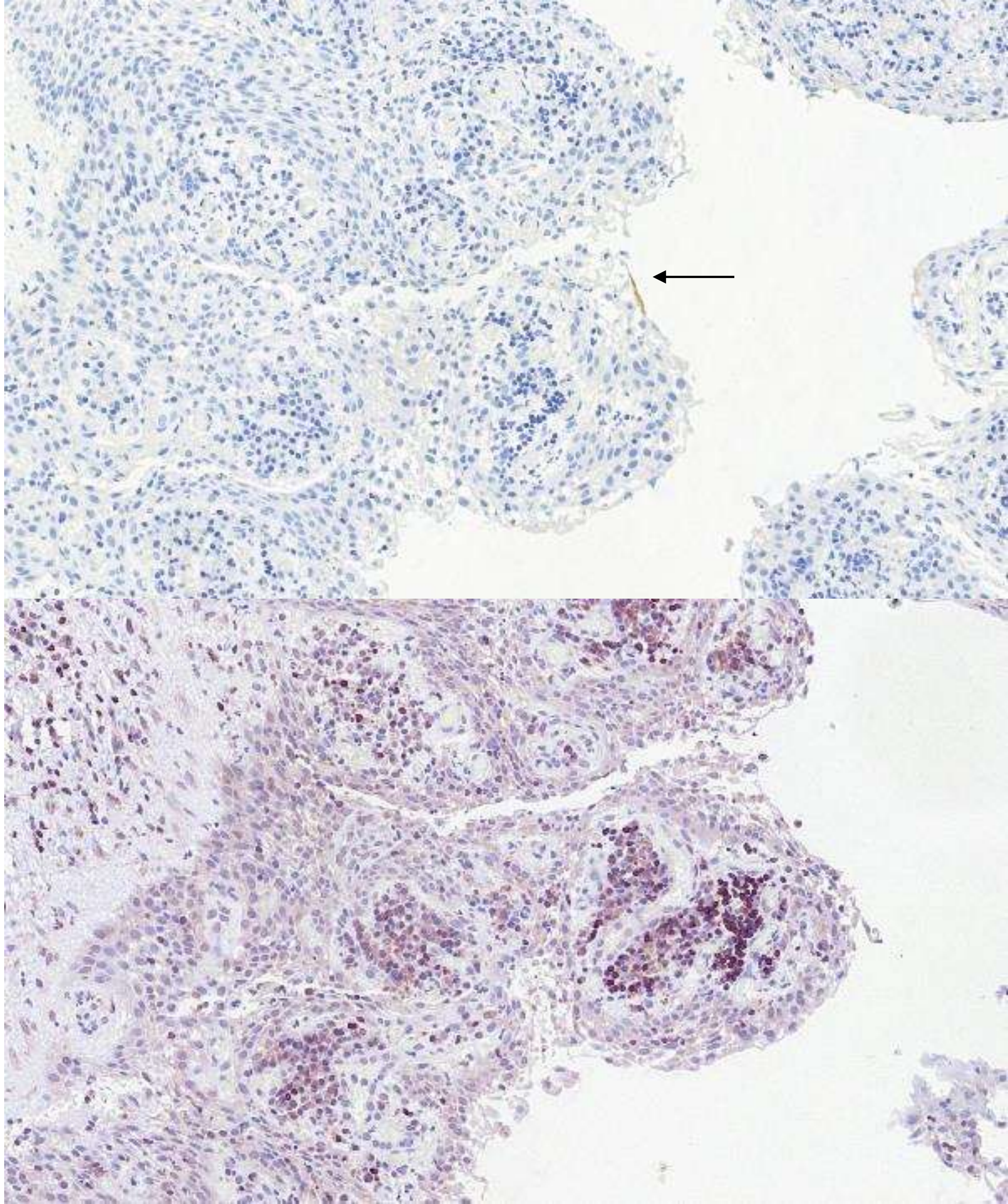

KR4 – HE, CK17, bcl2 (x1.4)

Focal transepithelial CK17 staining

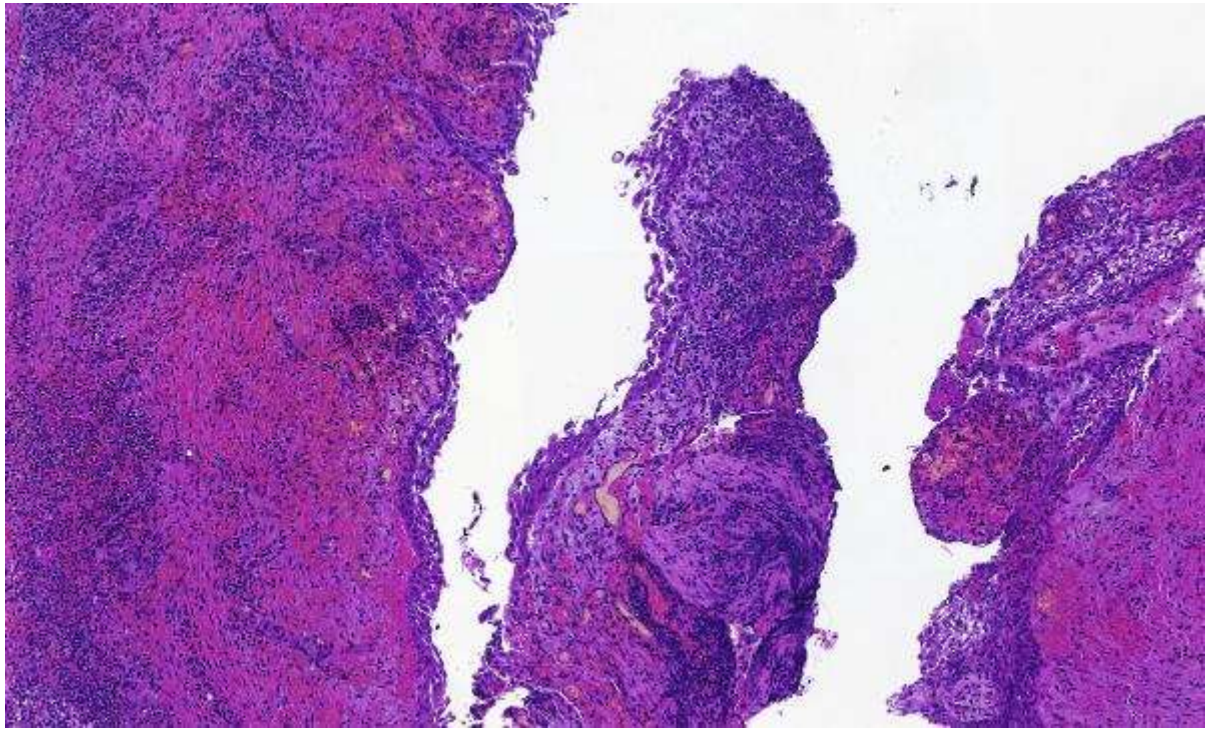

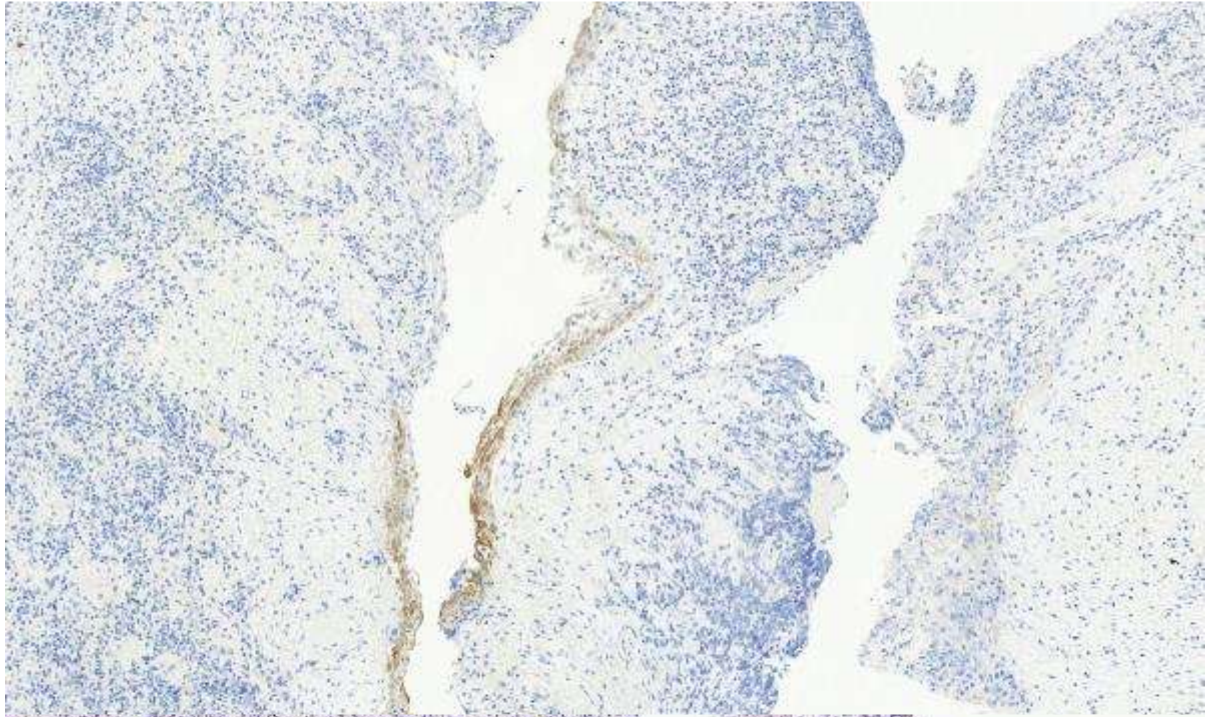

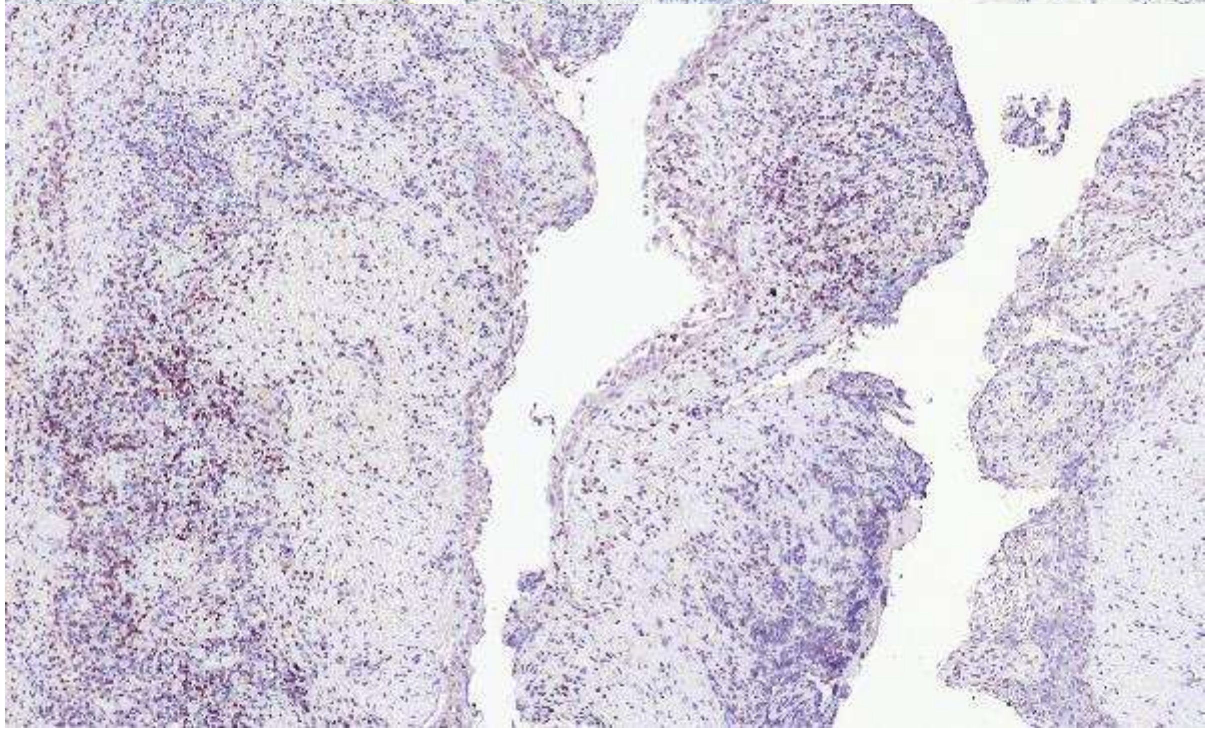

KR4 – Conclusion

- H&E: relatively thick (focally thin) non-keratinizing squamous epithelium, no parakeratosis, no wavy surface, no basal palisading, uneven (partly even) epithelial-subepithelial contours ; focal mucinous metaplasia
- Focal mixed inflammatory infiltration of the cyst wall
- Bcl2: negative
- CK17: dominantly negative, focal transepithelial staining in thinner epithelium, minimal superficial
- Histopathology: inflammatory odontogenic cyst, radicular cyst
- Combined clinical and pathological opinion: radicular cyst

Case KR5

- 43-year-old female
- Dental hygiene/status: partly missing, treated teeth; teeth 11 and 21 are not mobile. At the level of tooth 21 a soft lesion protrudes 5 mm from the mucosal level.
- Radiology: corticated radiolucent lesion of 45 mm in size in the right maxillary sinus
- Operation: In toto removal of the cyst, resection of roots 11, 21 and retrograde root filling
- Clinical diagnosis: maxillary cyst

KR5 – OPT

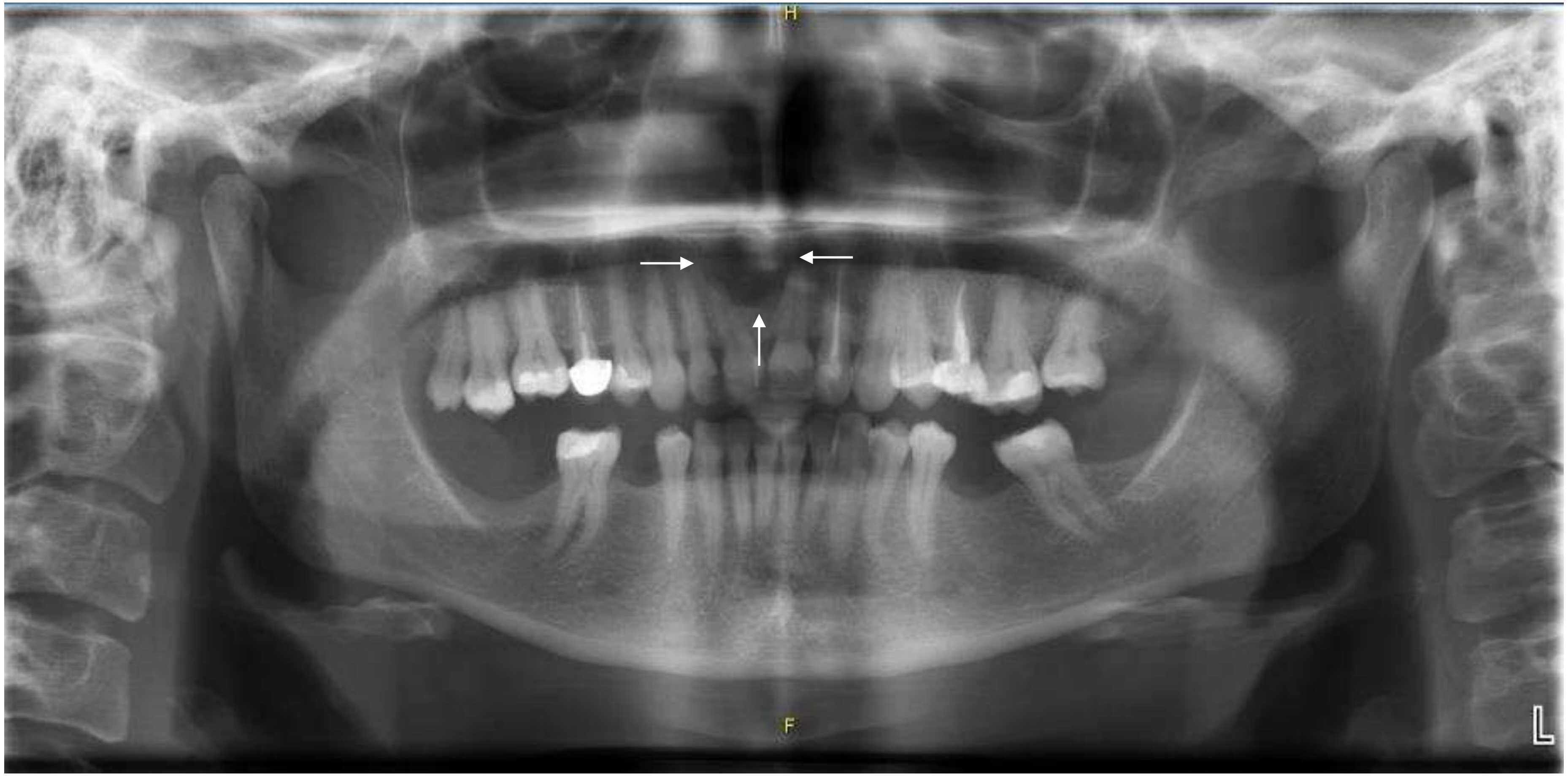

KR5 – OPT magnified

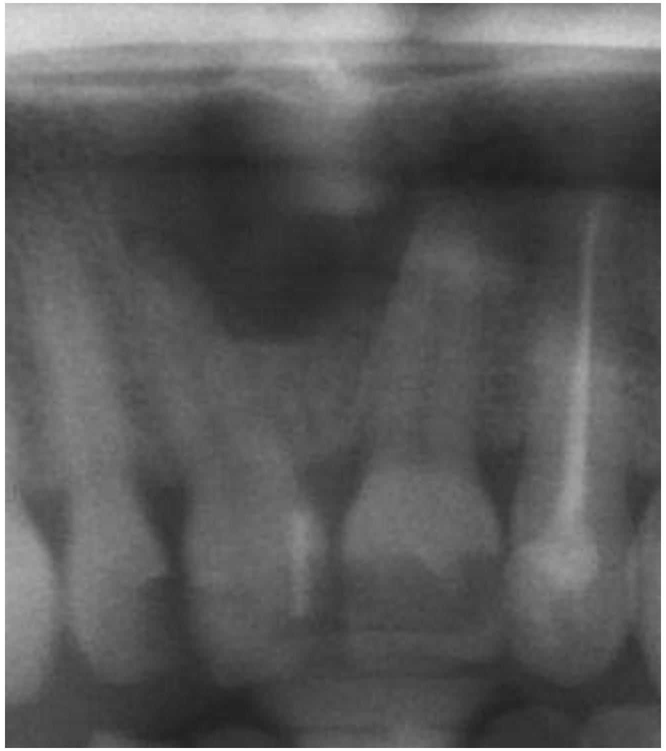

KR5 – HE, CK17, bcl2 (x1.6)

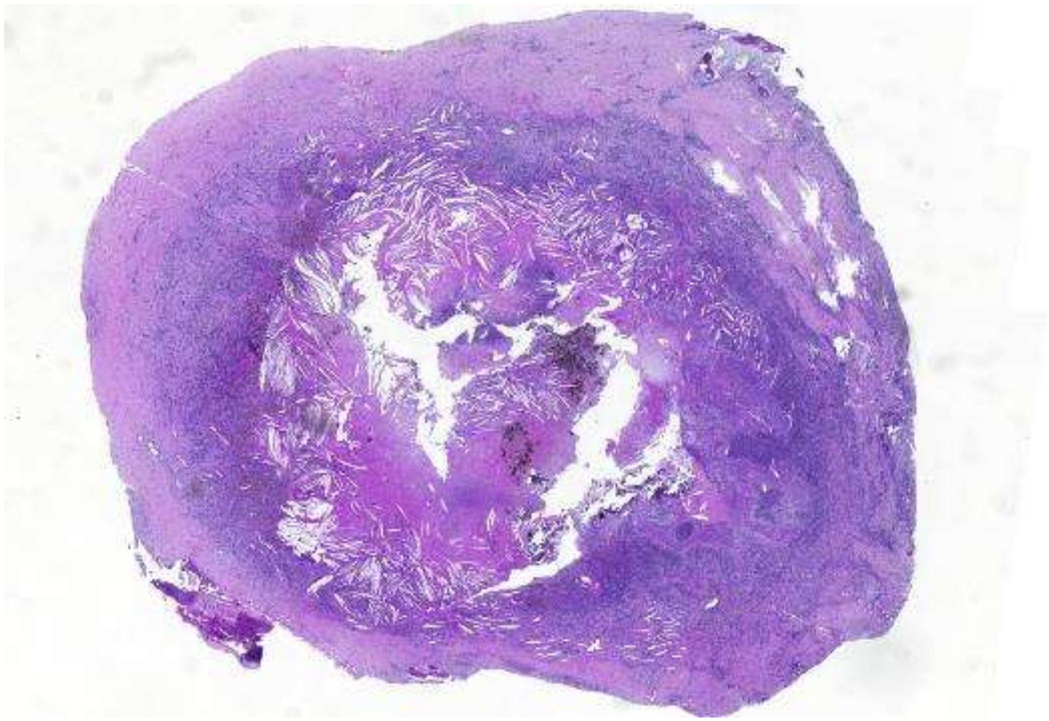

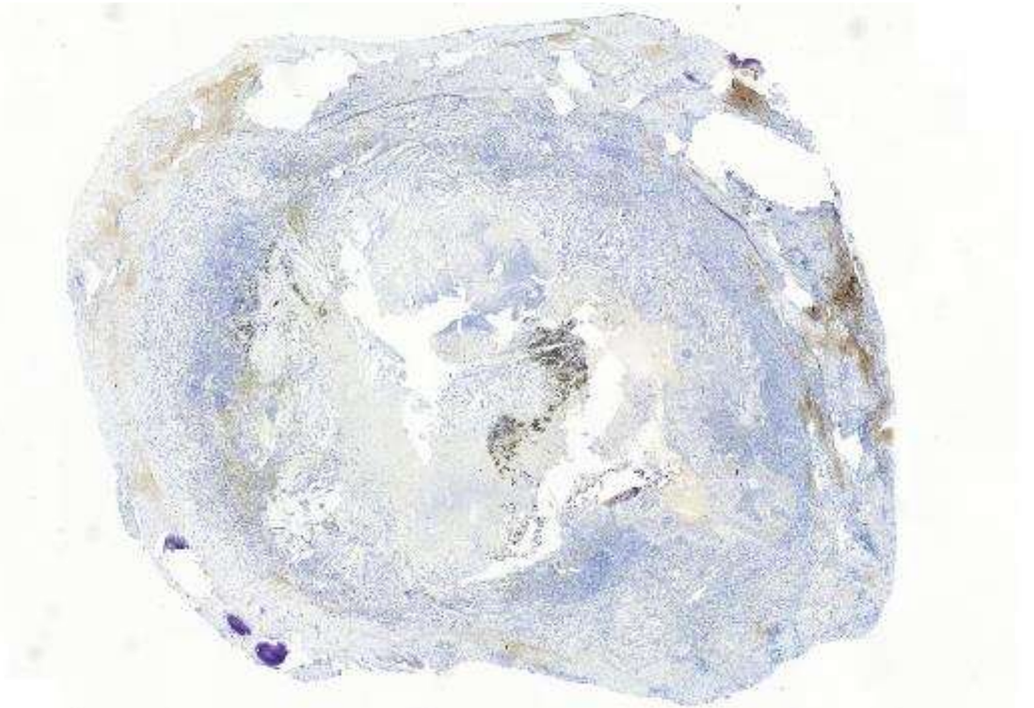

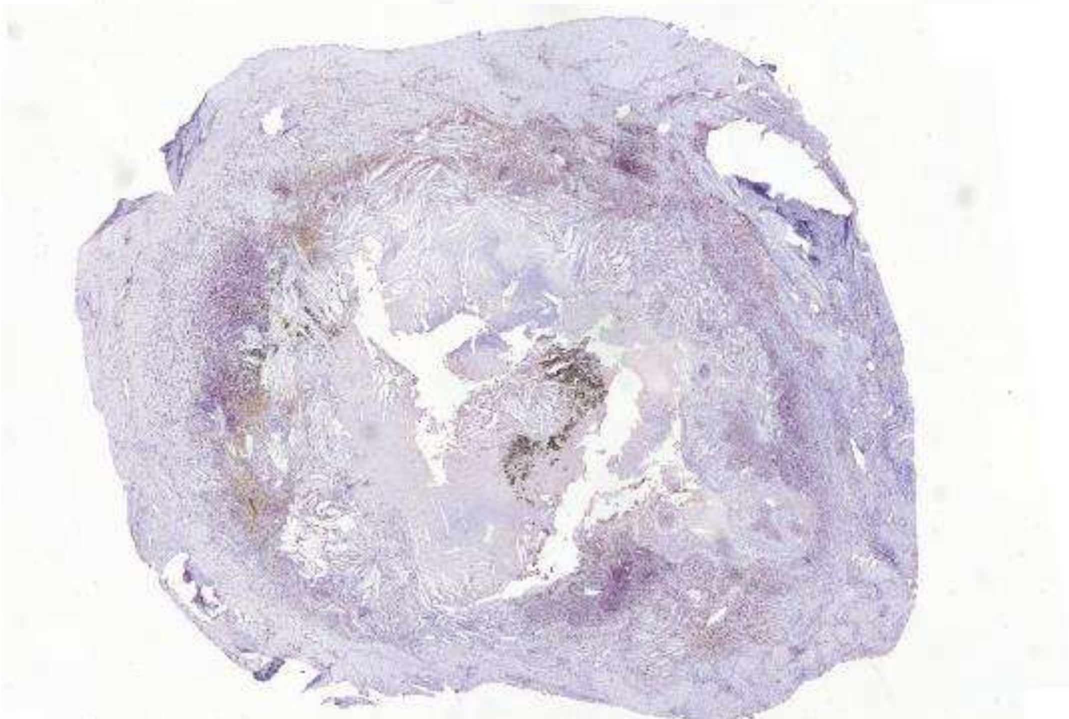

KR5 – HE, CK17, bcl2 (x5)

root filling material

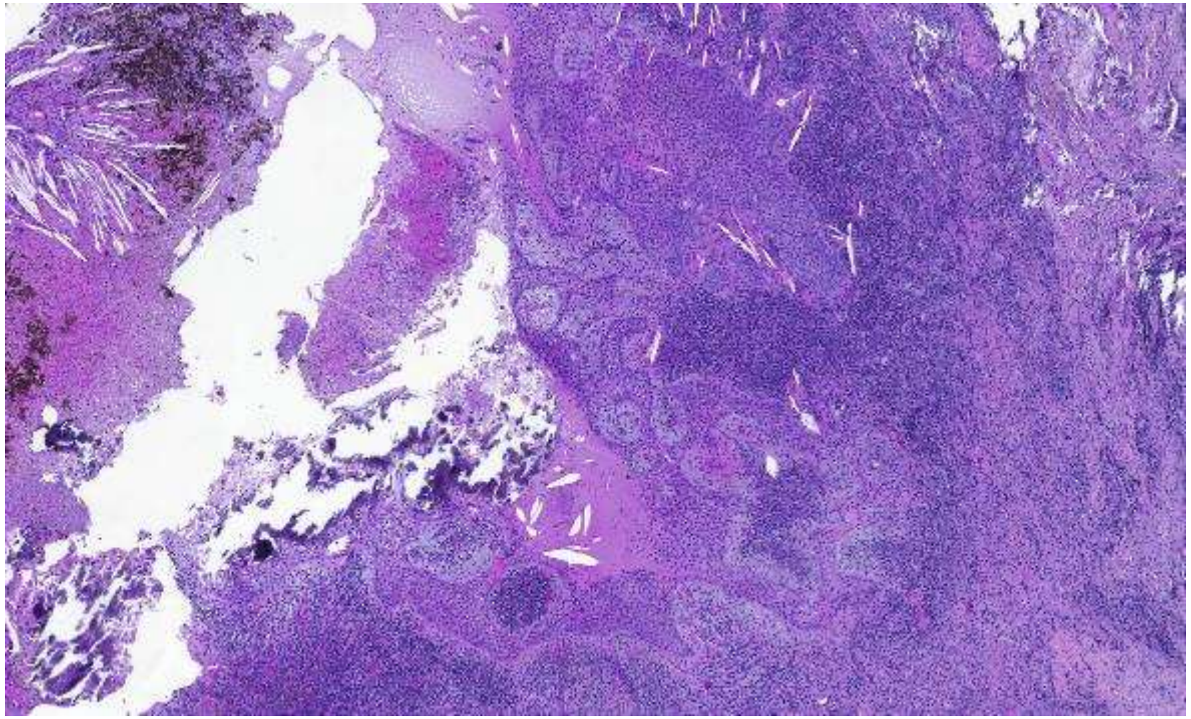

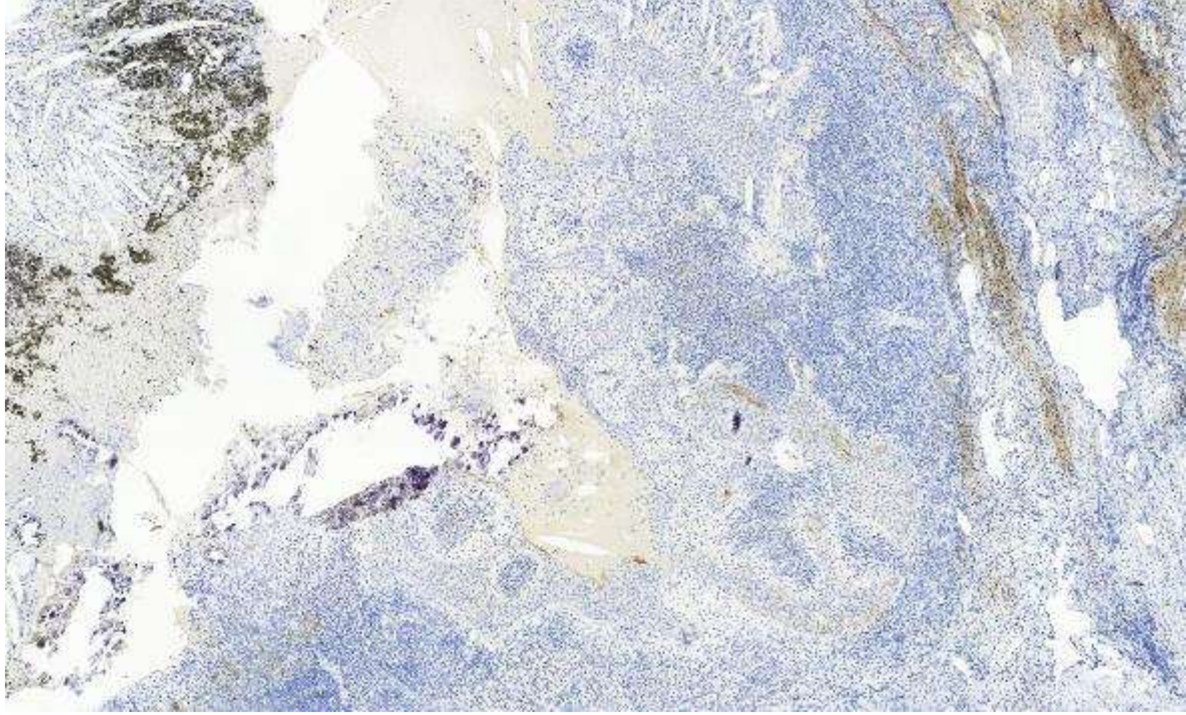

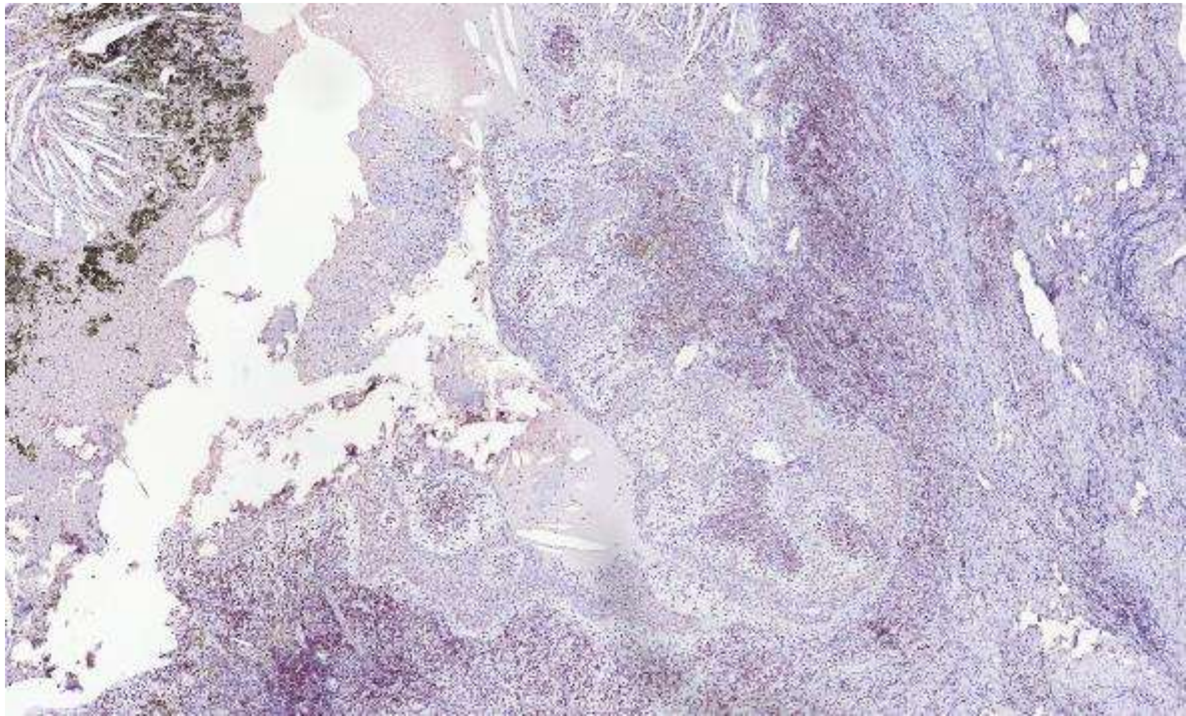

KR5 – HE, CK17, bcl2 (x10)

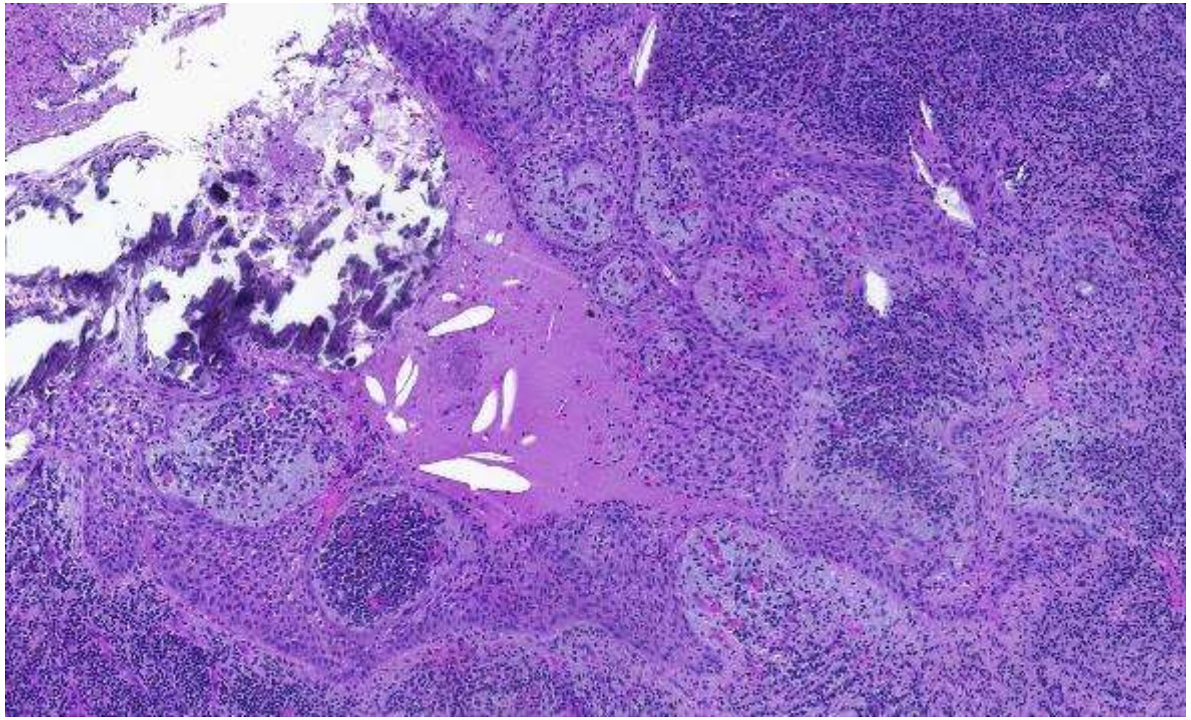

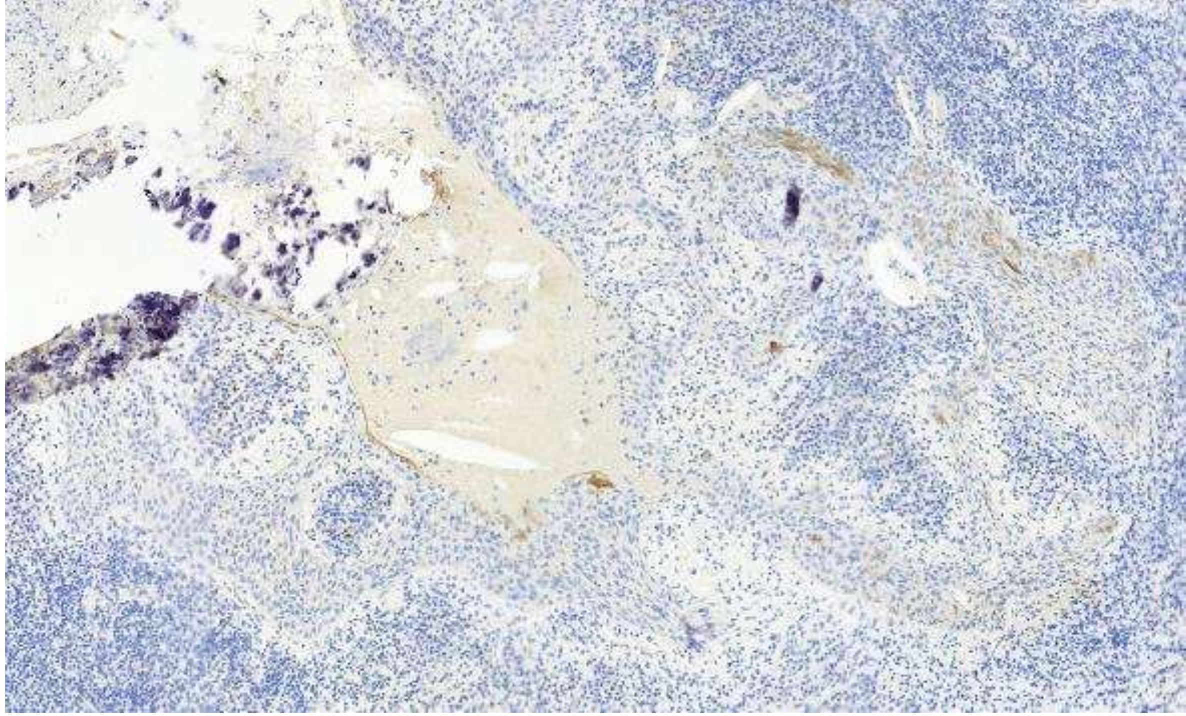

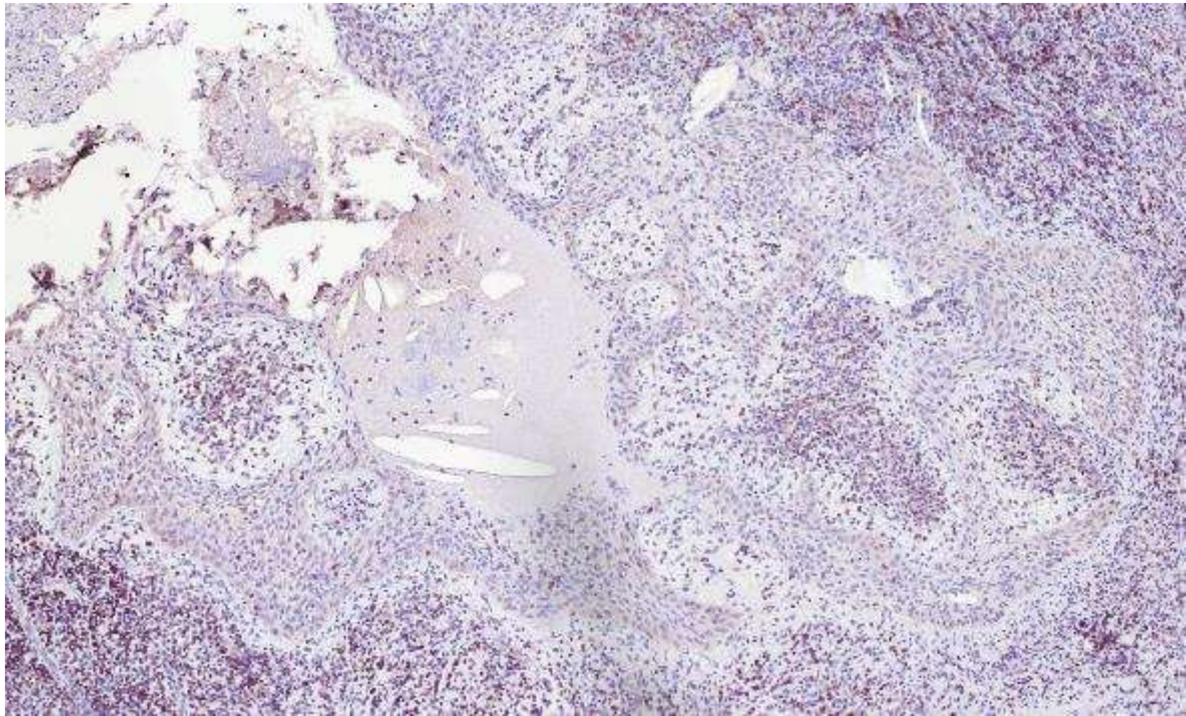

KR5 – Conclusion

- H&E: partly thick, partly thin squamous epithelium, no parakeratosis, no wavy surface, no basal palisading, uneven (partly even) epithelial-subepithelial contours; focal mucous metaplasia
- Focal inflammation of the cyst wall with mixed infiltrate but lymphocytes and plasma cells predominating; filling material debris
- Bcl2: negative (uncertain faint basal (/suprabasal) staining incomparable with control lymphocyte positivity – or non specific background staining)
- CK17: dominantly negative, minimal focal basal and/or superficial (nearly transepithelial) staining
- Histopathology: inflammatory odontogenic cyst, radicular cyst
- Combined clinical and pathological opinion: radicular cyst (inflamed nasopalatine duct cyst considered; there were no data on palatal extension and filling material debris shifted the diagnosis toward radicular cyst.)

Case KR6

- 49-year-old male
- Dental hygiene/status: normal hygiene; crowded teeth, delayed eruption of teeth 18, 28, 38; tooth 38 in horizontal impaction
- Radiology: radiolucent lesion around the crown of tooth 38
- Operation: decoronation of tooth 38, removal of the tooth in two pieces, and removal of the cyst touching the root of tooth 37
- Clinical diagnosis: dentigerous (follicular) cyst

KR6 – OPT

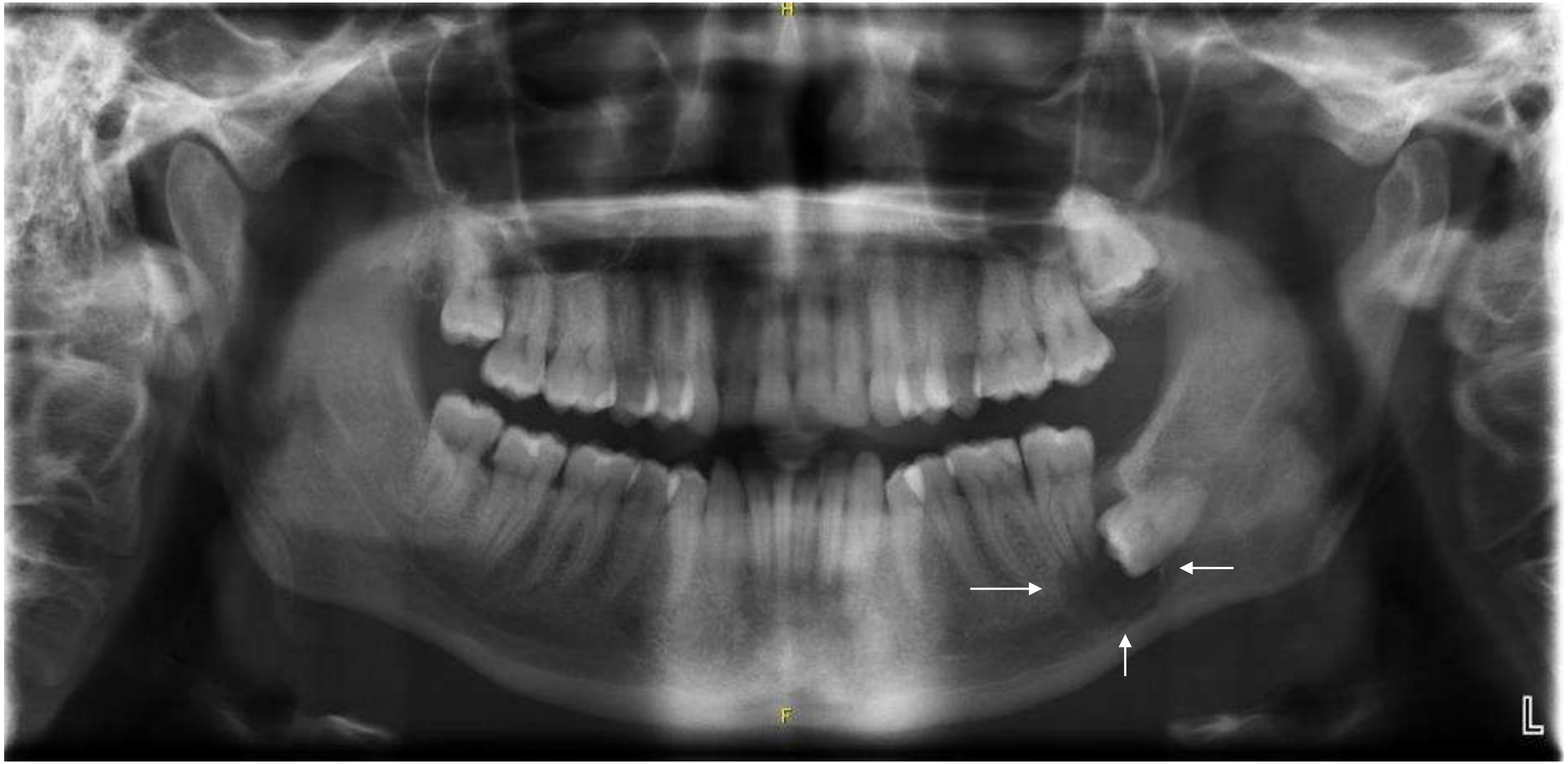

KR6 – HE, CK17, bcl2 (x1)

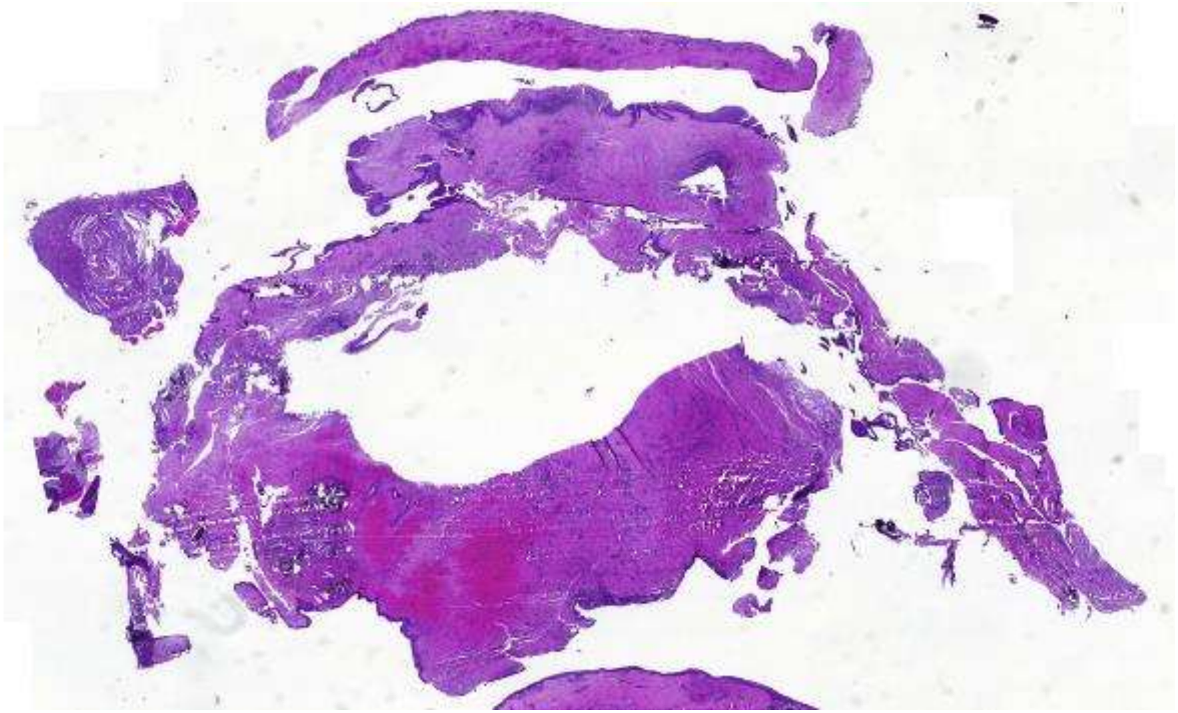

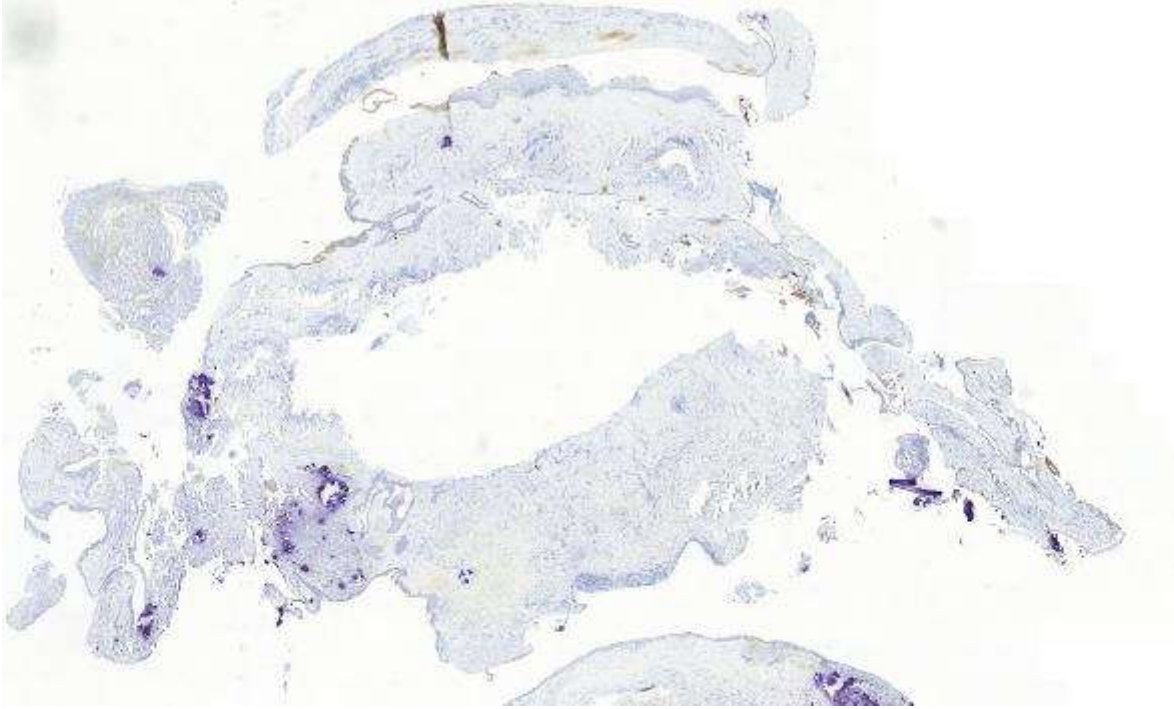

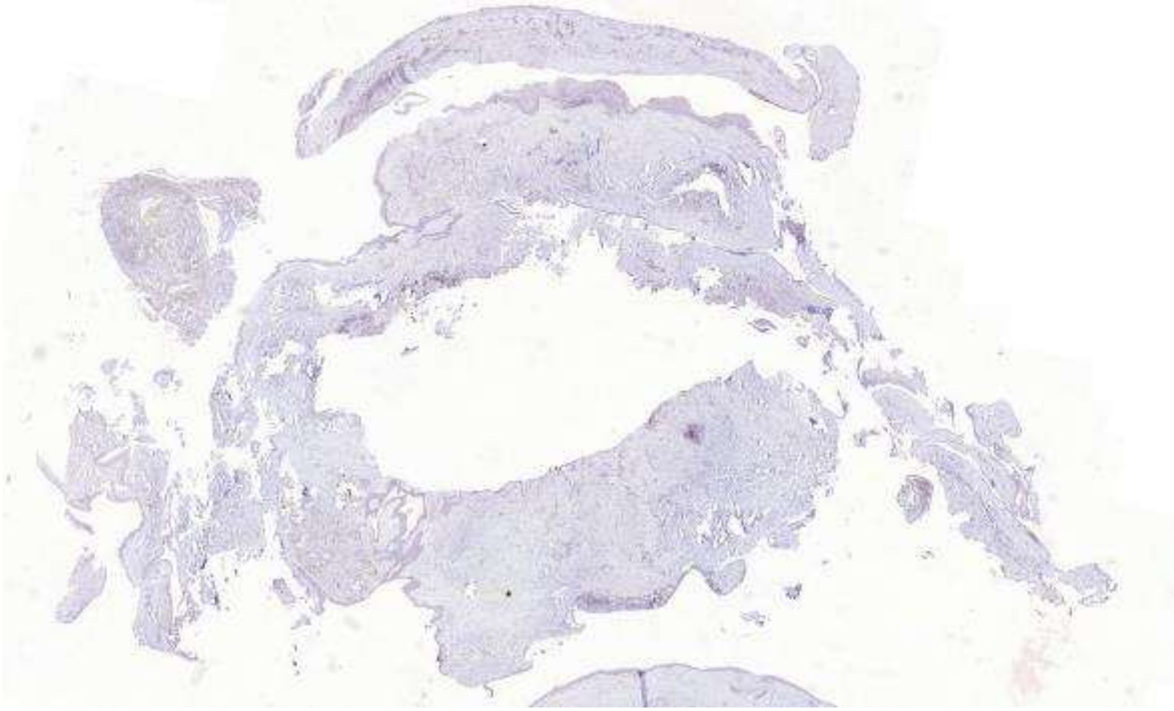

KR6 – HE, CK17, bcl2 (x5)

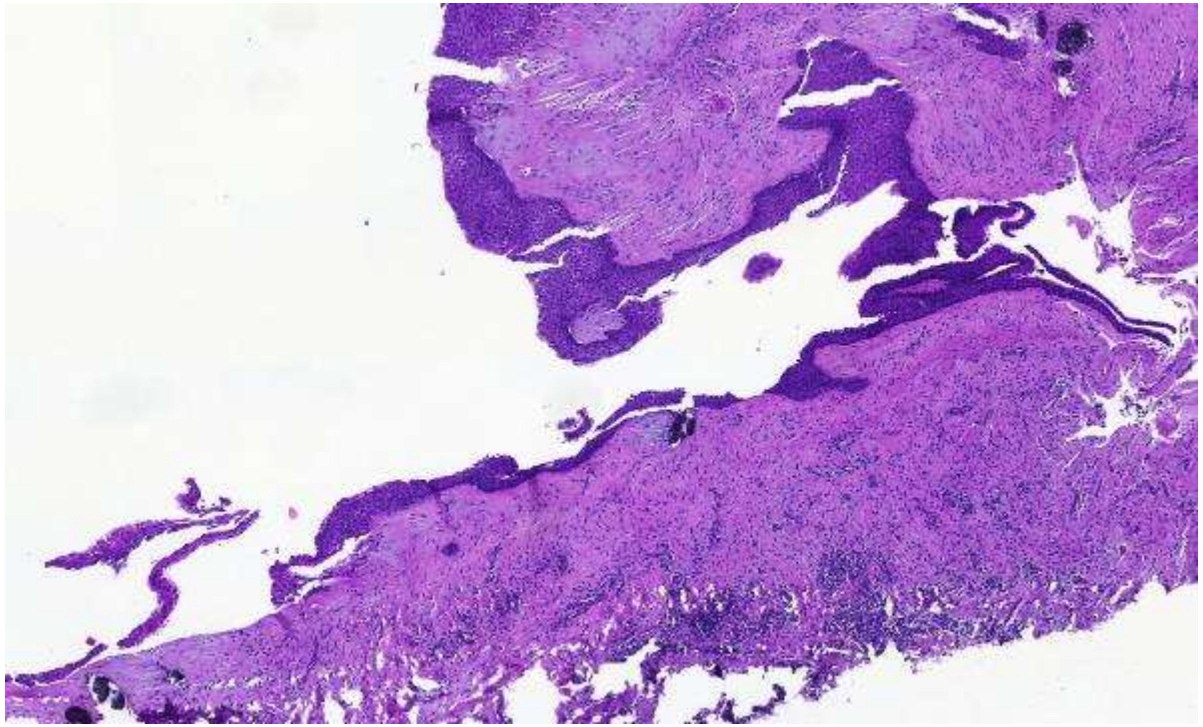

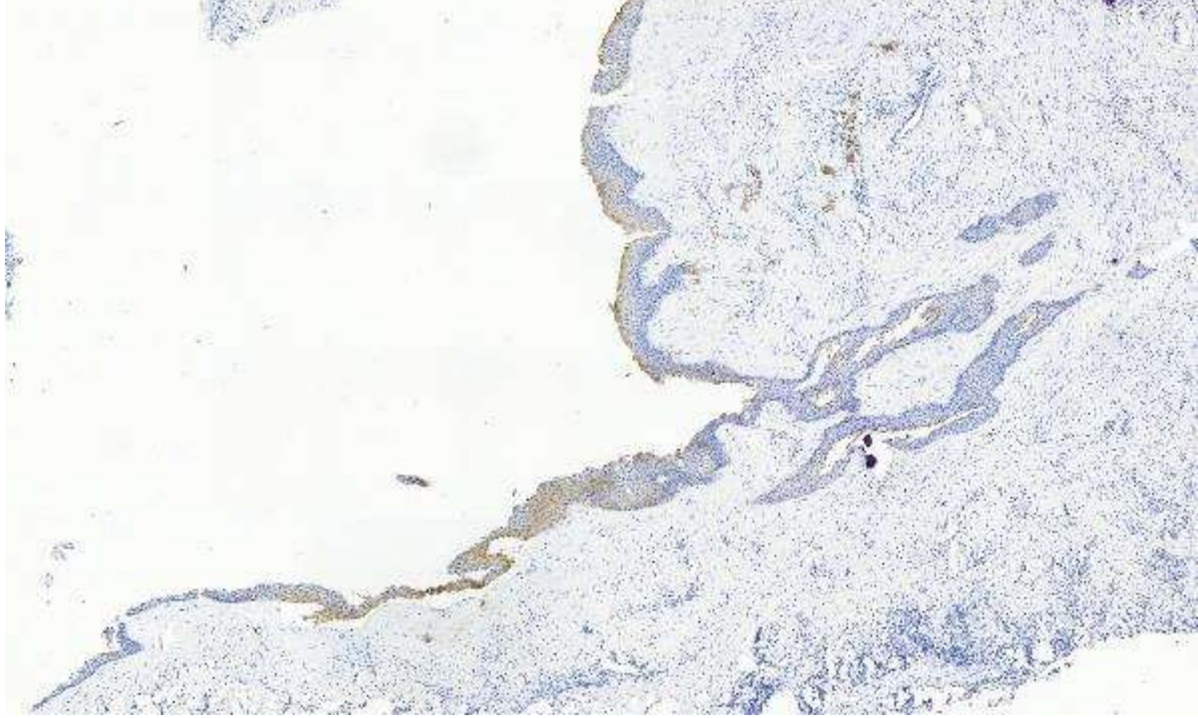

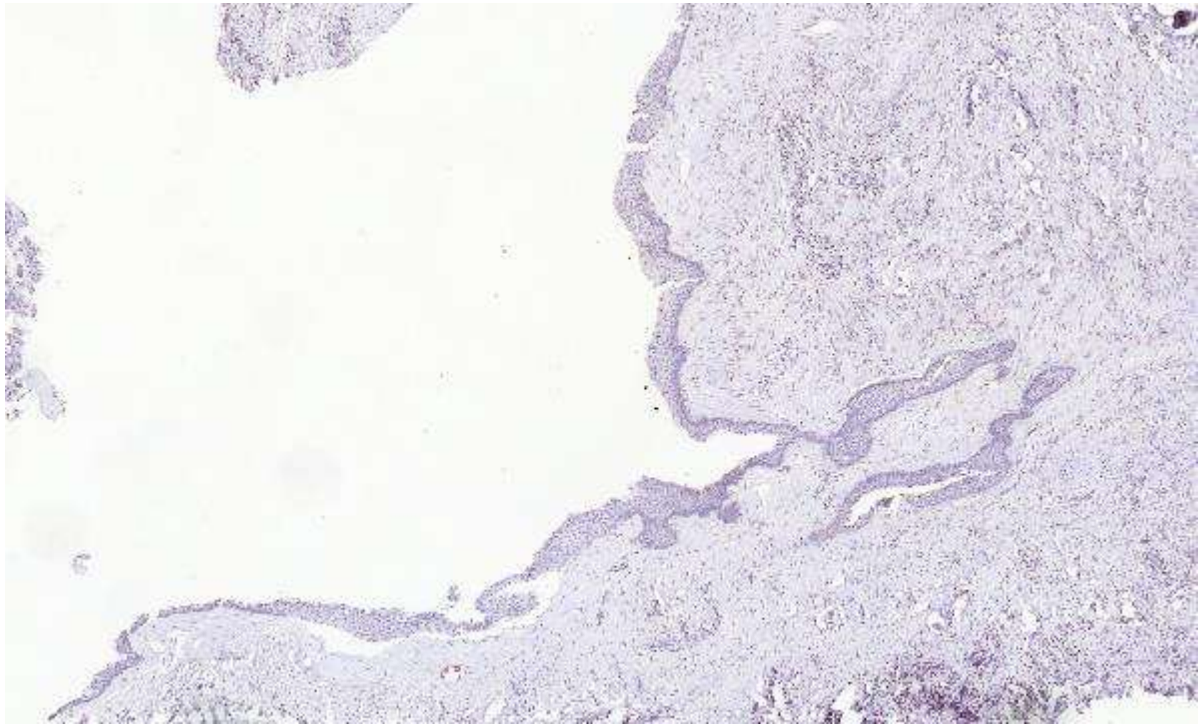

KR6 – HE, CK17, bcl2 (x5)

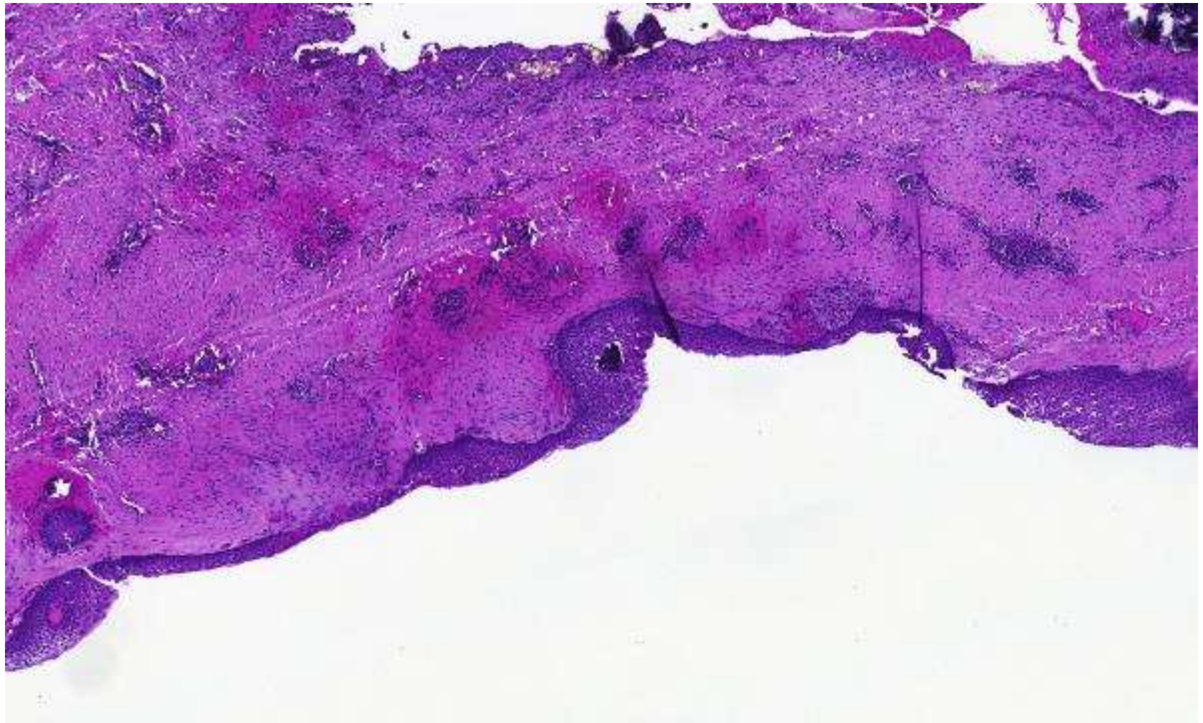

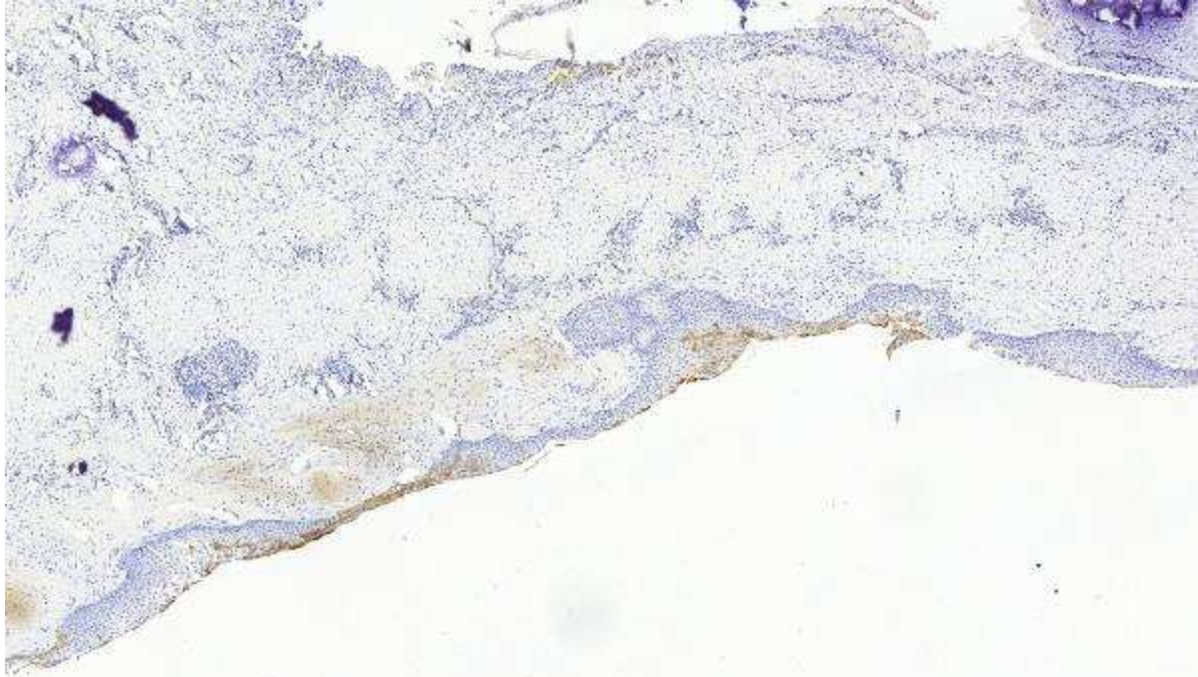

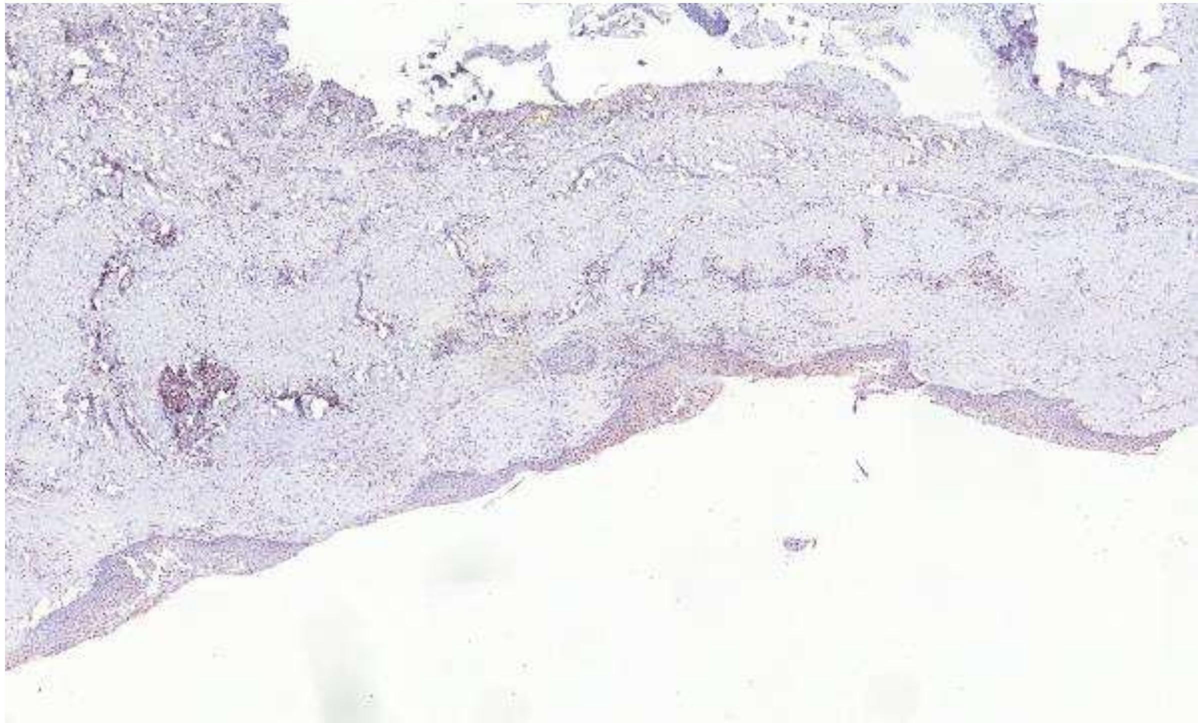

KR6 – HE, CK17, bcl2 (x10)

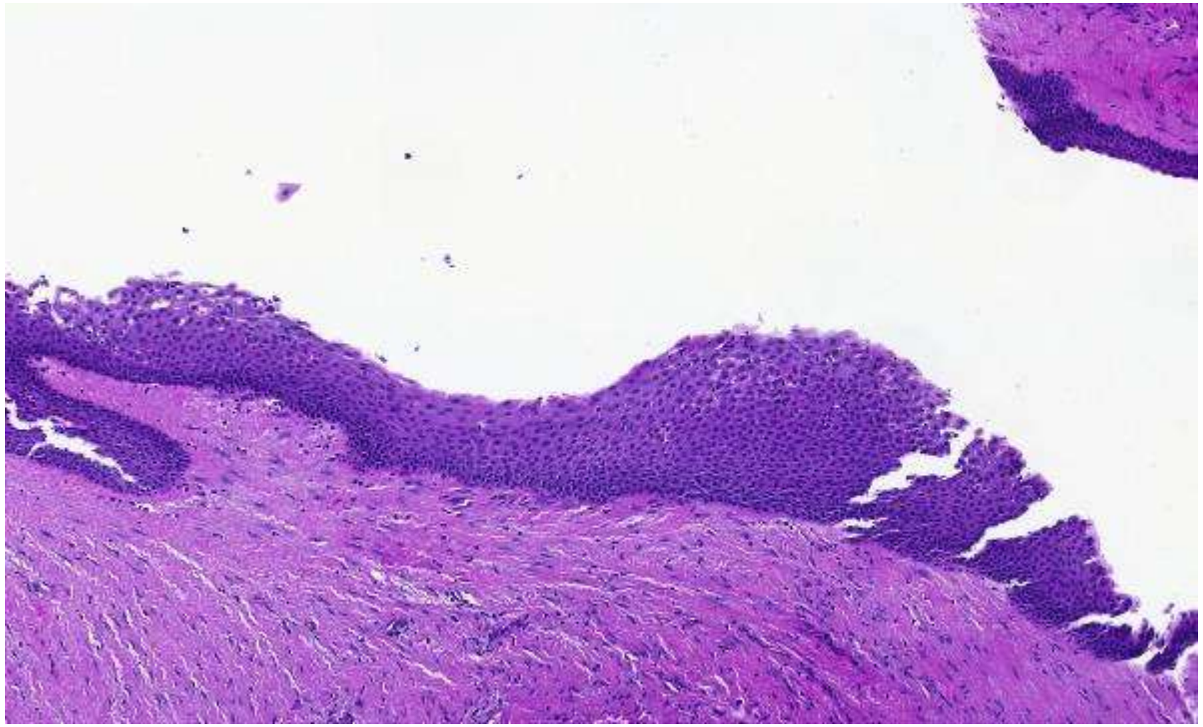

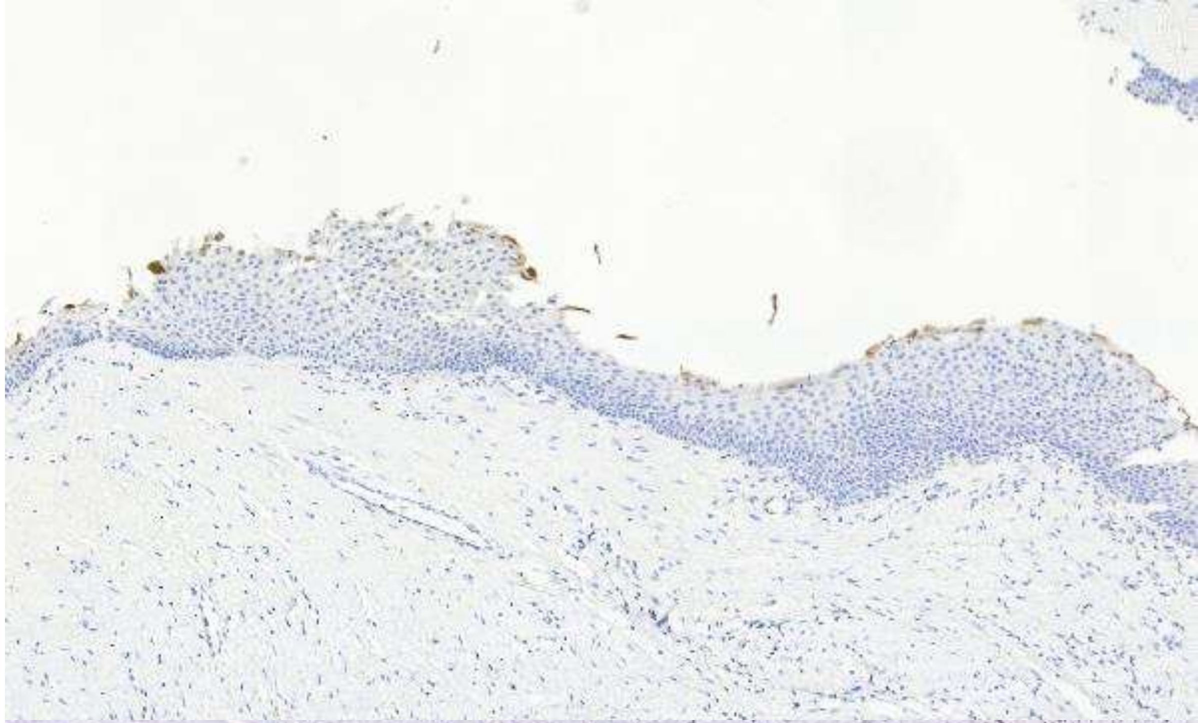

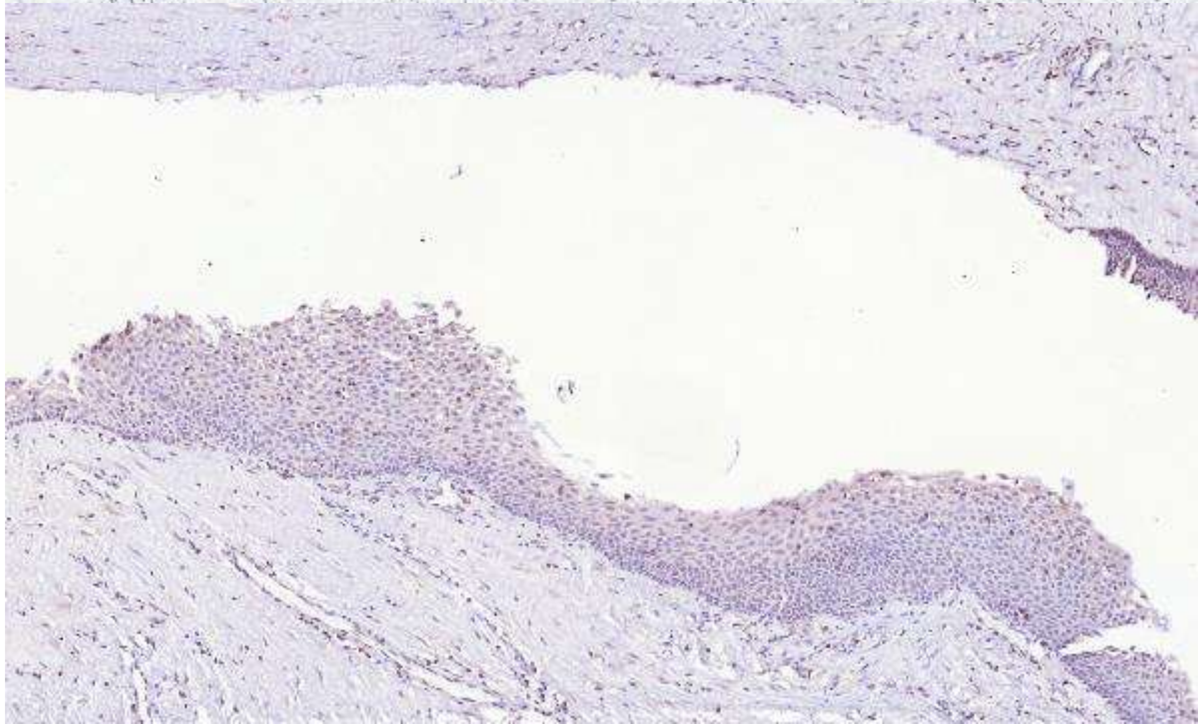

KR6 – Conclusion

- H&E: partly thick, partly thin (2-3 cell layers) non-keratinizing squamous epithelium, no parakeratosis, no wavy surface, no basal palisading, even epithelial-subepithelial contours
- Generally no inflammation of the cyst wall, with smaller foci of lymphocytes and focal cholesterol granuloma
- Bcl2: negative
- CK17: dominantly thin superficial, with focal weak transepithelial staining
- Histopathology: dentigerous (follicular) cyst
- Combined clinical and pathological opinion: dentigerous (follicular) cyst

Case KR7

- 60-year-old female
- Dental hygiene/status: normal hygiene; partly missing, treated teeth
- Radiology: partial impaction of tooth 38, 1x1 cm radiolucency distal to its crown
- Operation: removal of tooth 38 and cystectomy
- Clinical diagnosis: Cyst? Dental follicle?

KR7 – OPT

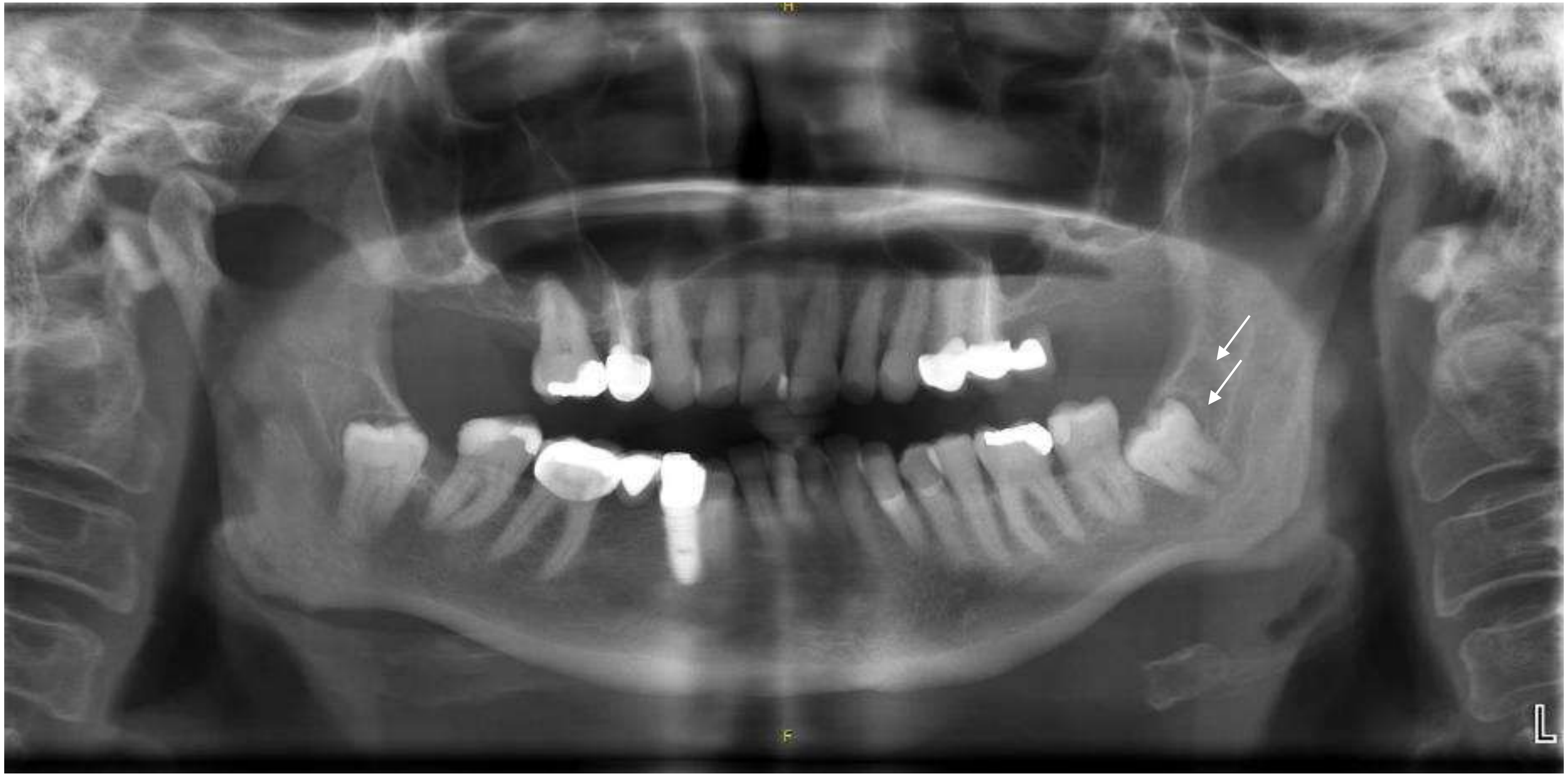

KR7 – OPT magnified (follicle)

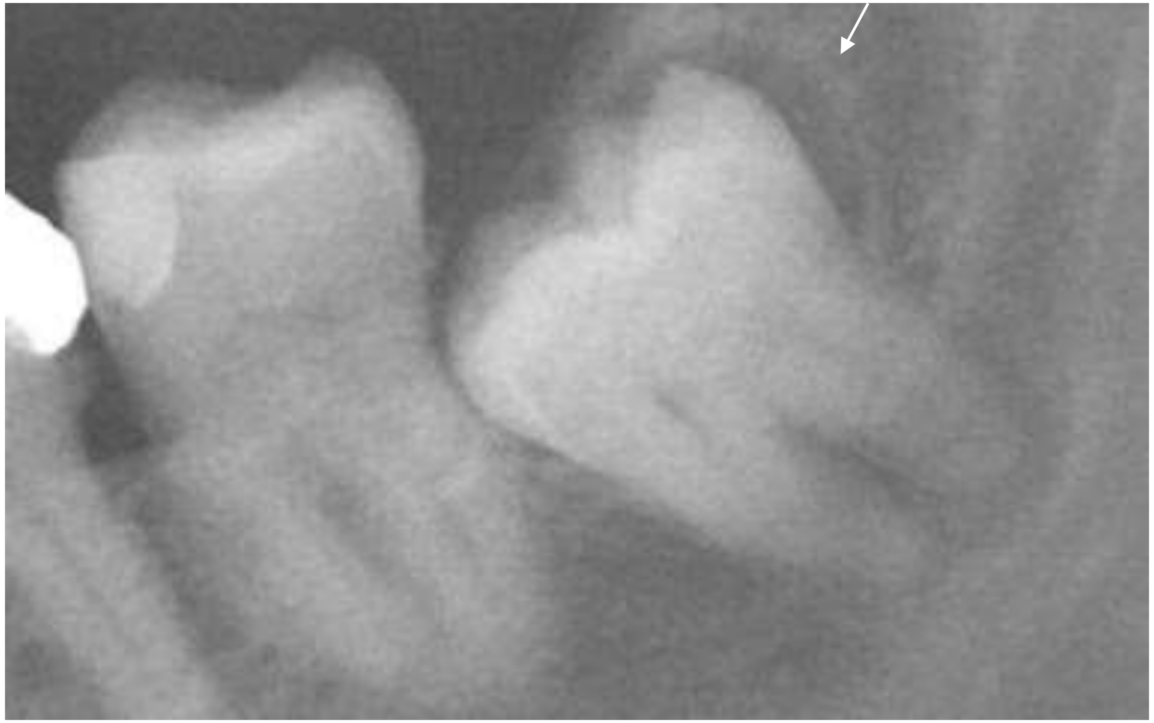

KR7 – HE (x1.3) Non-inflamed area (bottom) and inflamed area (top right)

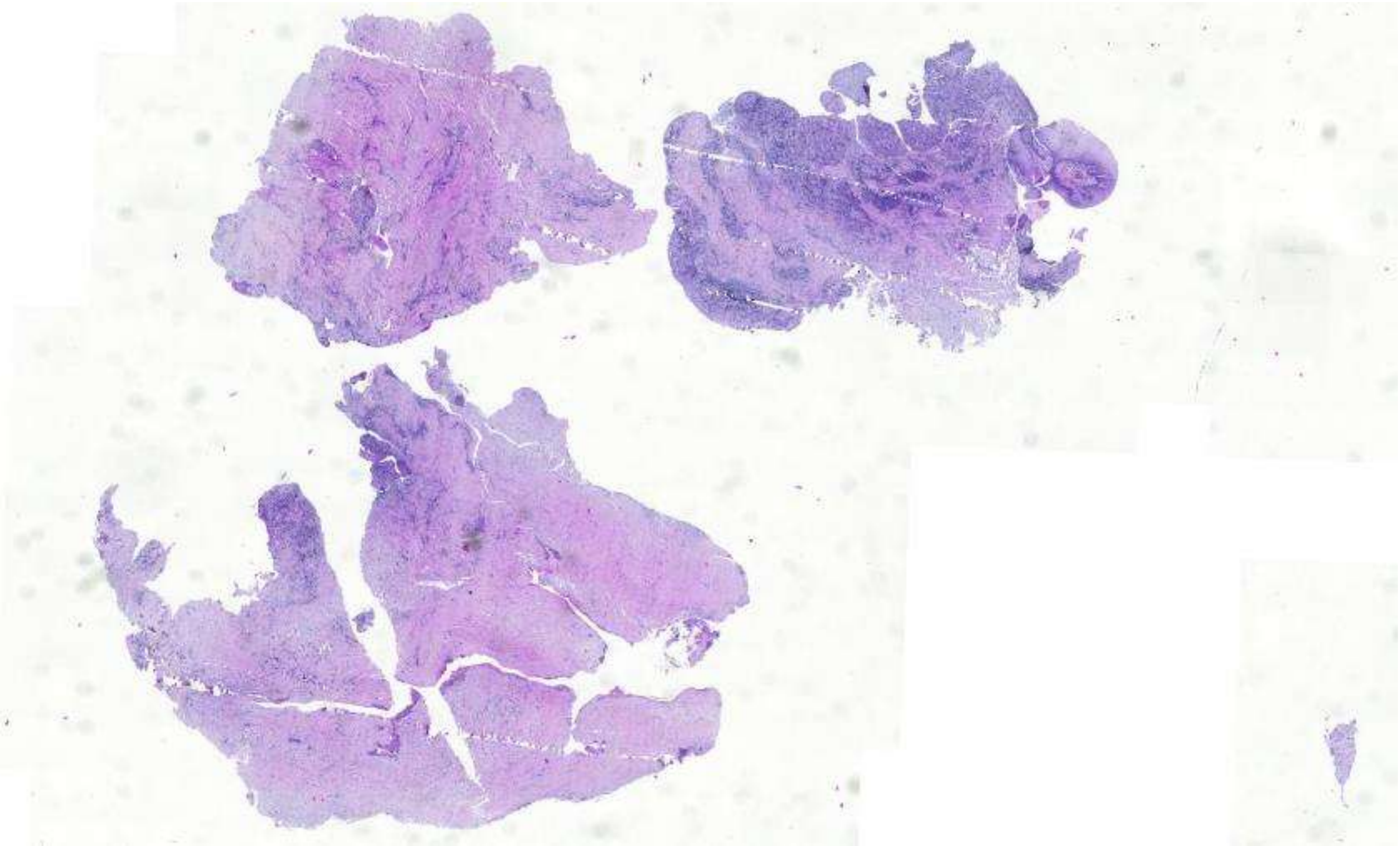

KR7 – HE, CK17, bcl2 (x12)

Non-inflamed area

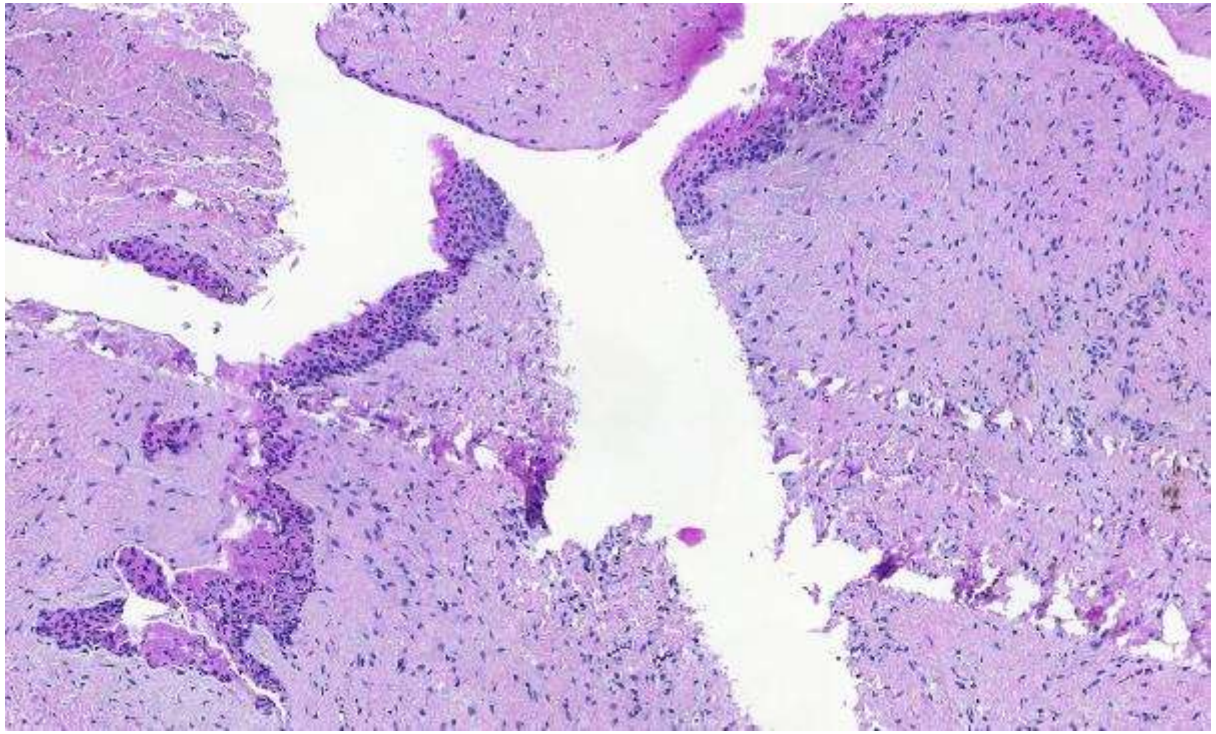

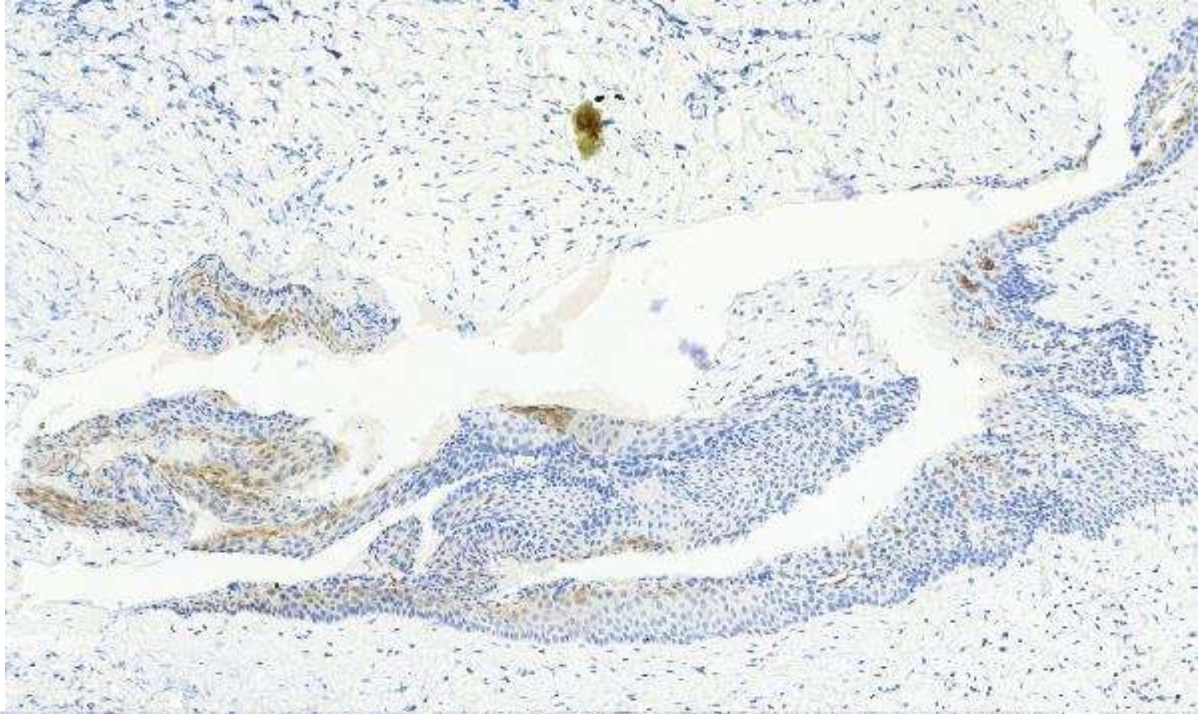

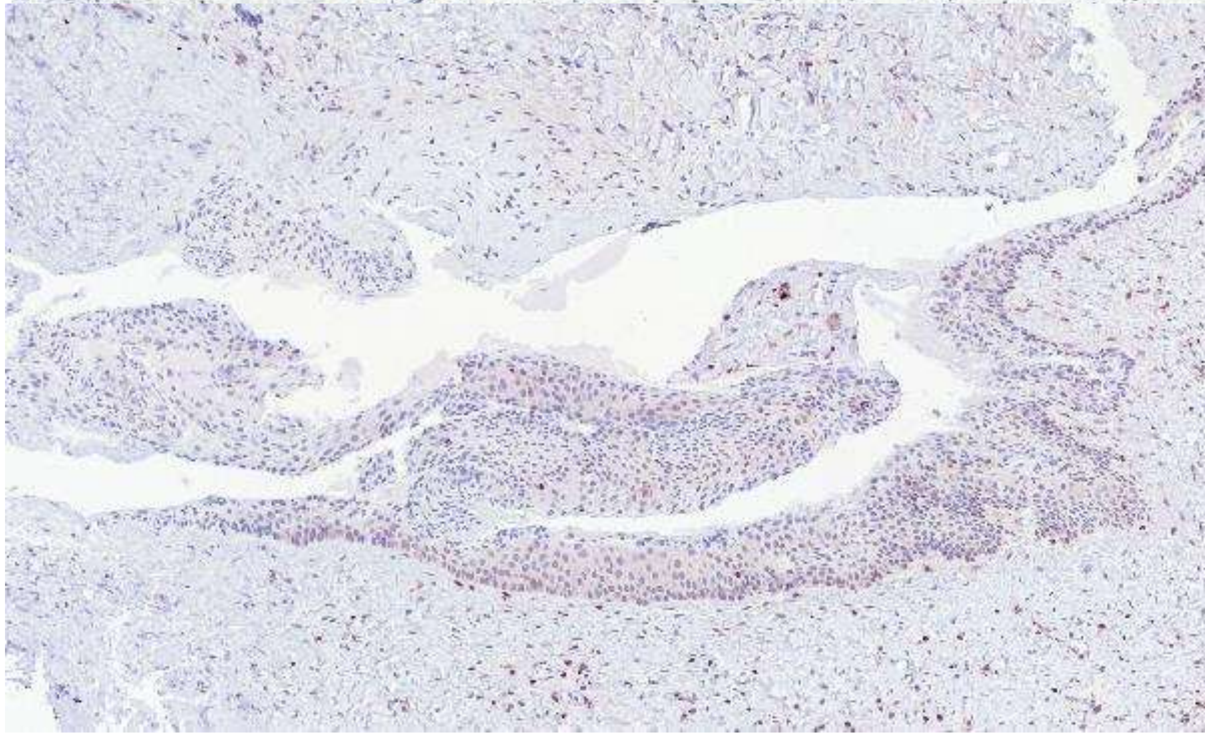

KR7 – CK17, bcl2 (x20) non inflamed area

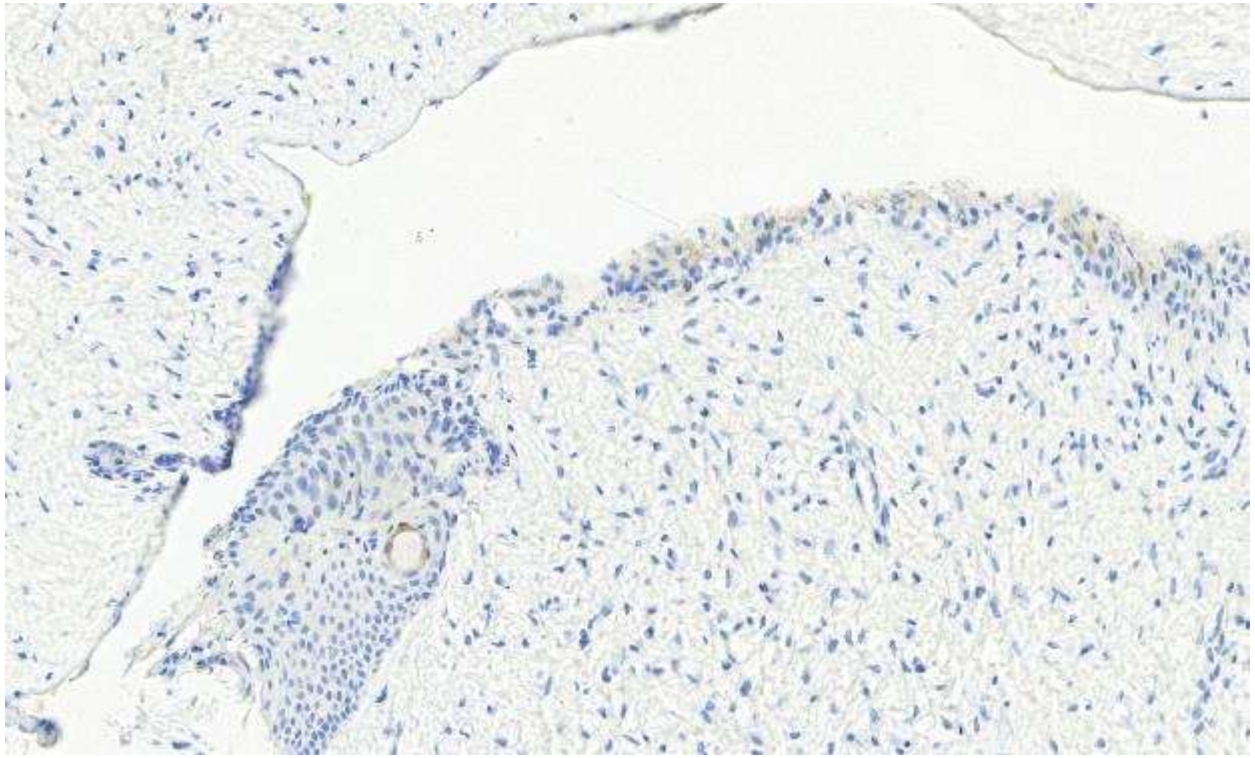

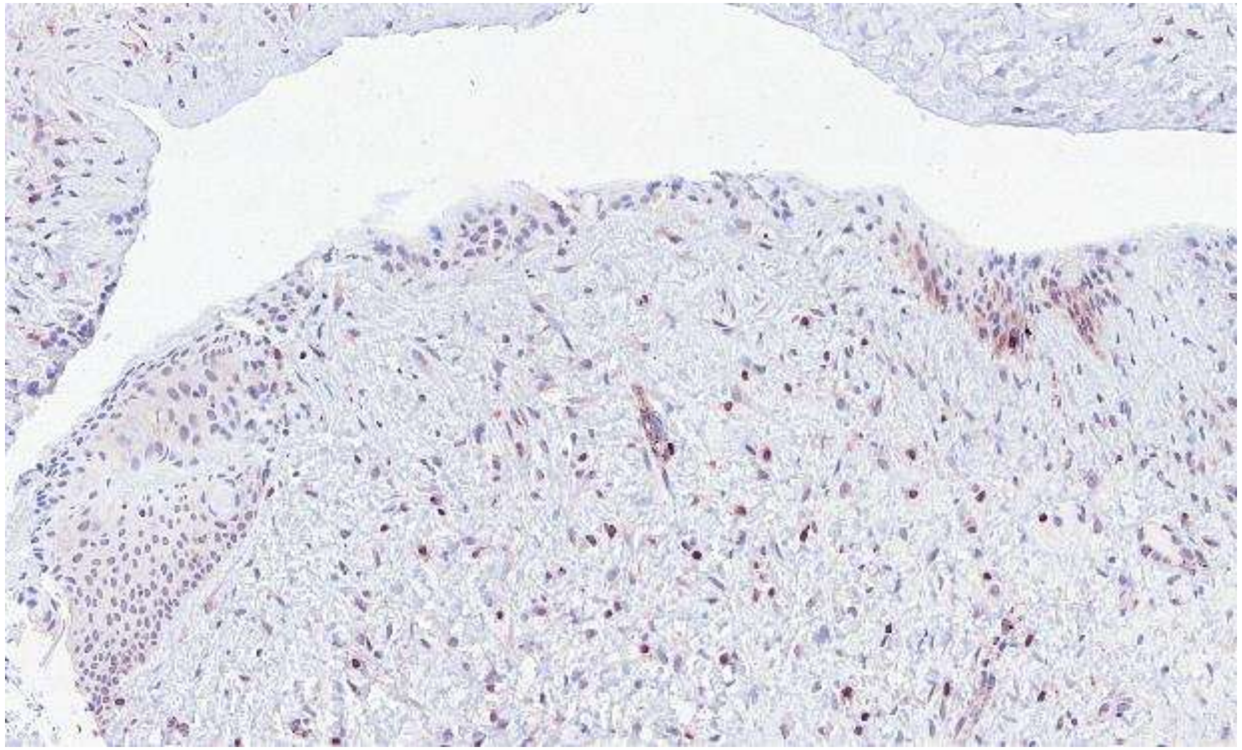

KR7 – HE, CK17, bcl2 (x5)

Inflamed area, minimal transepithelial CK17 staining (*) and focal superficial staining (↑)

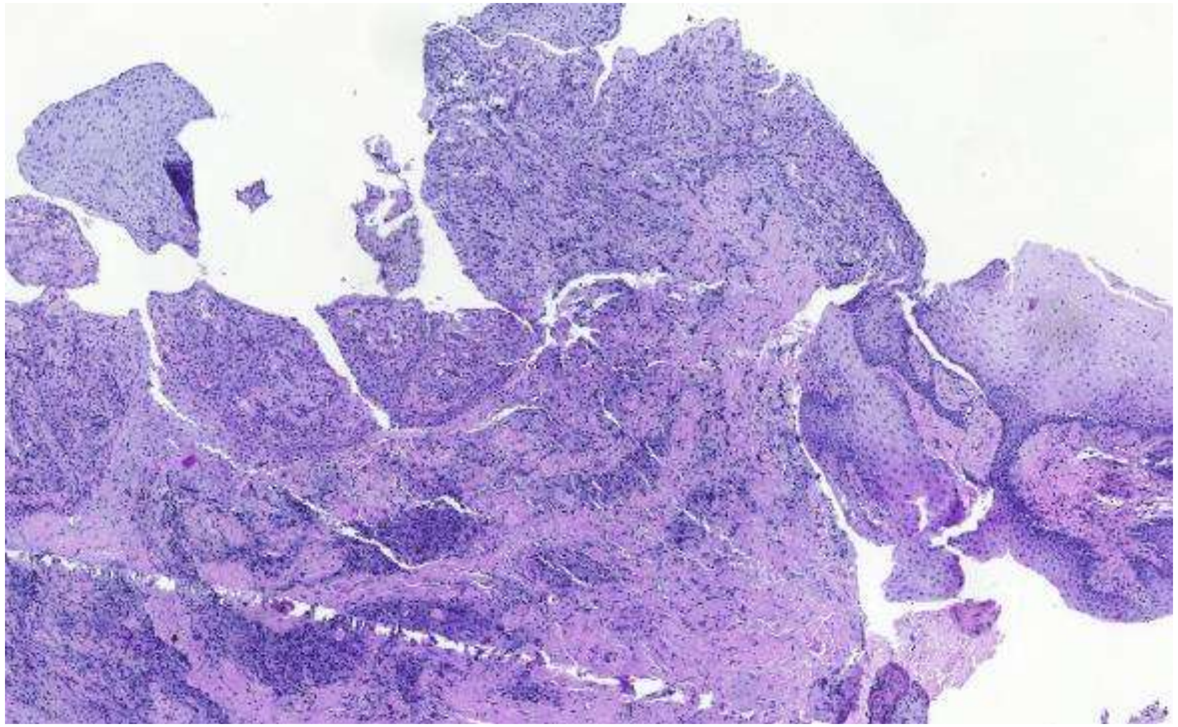

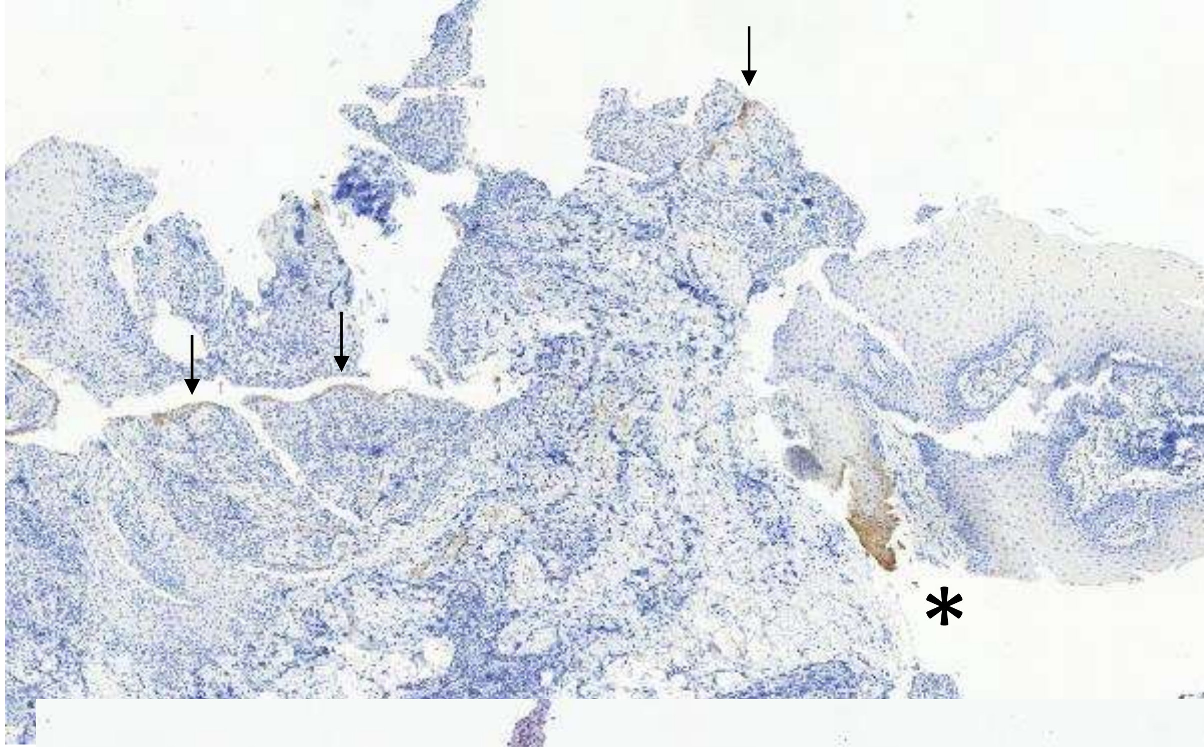

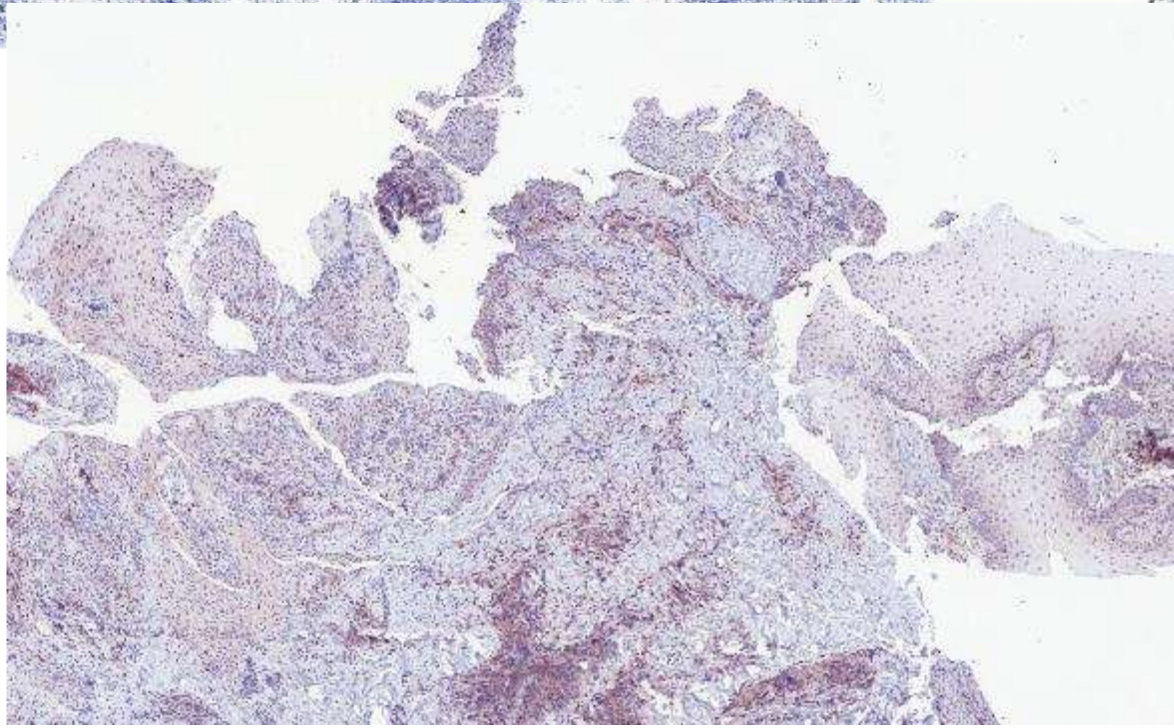

KR7 – Conclusion

- H&E: relatively thick squamous epithelium (non-inflamed areas with thin epithelium), no parakeratosis, no wavy surface, no basal palisading, uneven epithelial-subepithelial contours
- Focal mixed inflammatory infiltrate of the cyst wall (at the site of epithelial thickening)
- Bcl2: negative
- CK17: dominantly negative, minimal focal superficial, spinous or transepithelial staining
- Histopathology: inflammatory odontogenic cyst (radicular cyst?)
- Combined clinical and pathological opinion: dentigerous (follicular) cyst (+ follicle)

Case KR8

- 33-year-old female
- Dental hygiene/status: partly missing, partly treated teeth, crown of teeth 16 and 12 missing, roots in the alveoli; vestibular mucosa at teeth 11, 12 is slightly protruding and tender
- Radiology: 15-mm-diameter radiolucency corresponding to teeth 11, 12, also seen on periapical radiogram
- Operation: resection of root 11, removal of tooth 12 and the cyst, excochleation of granulation tissue
- Clinical diagnosis: periapical cyst

KR8 – OPT

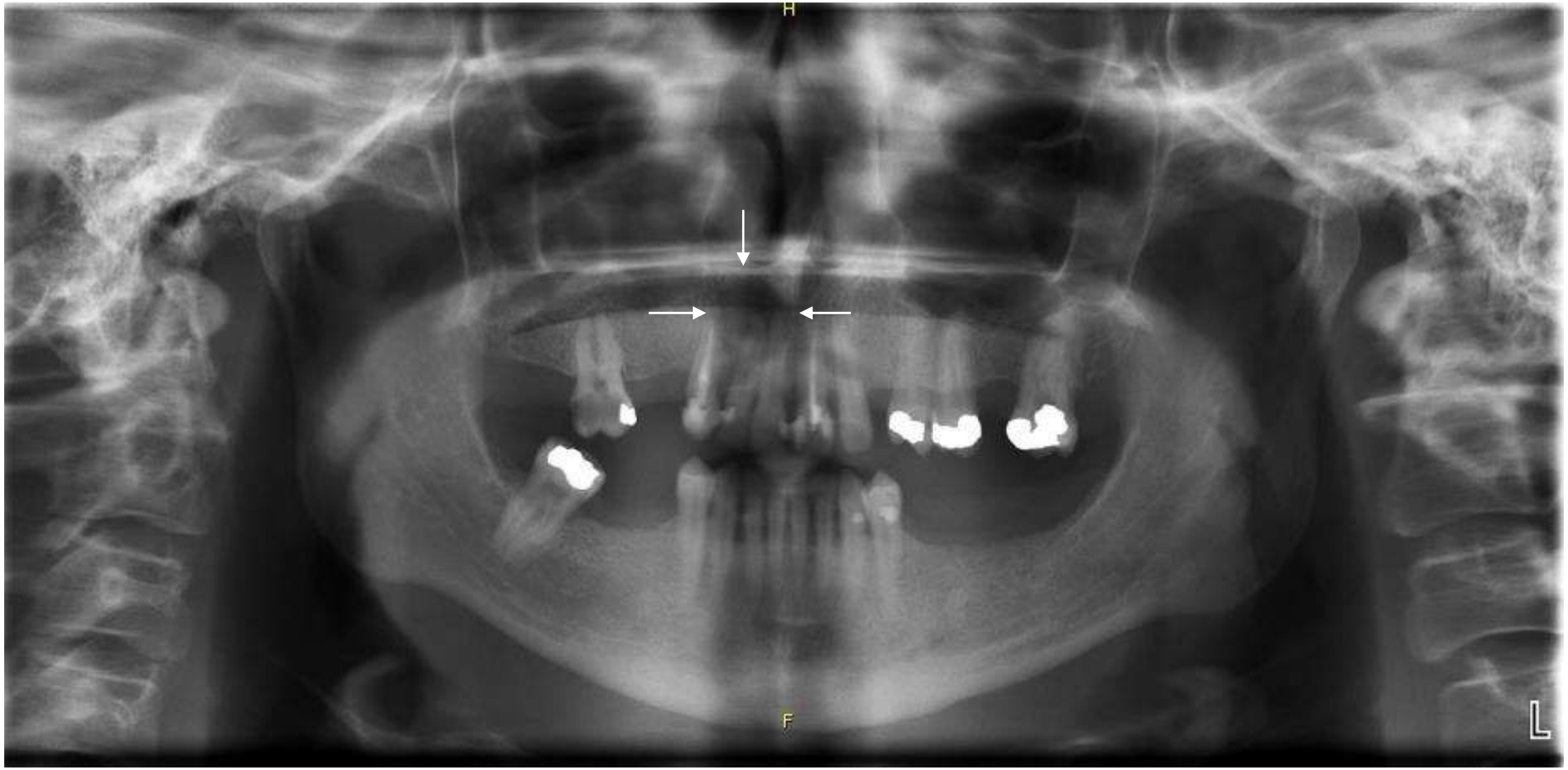

KR8 – OPT magnified

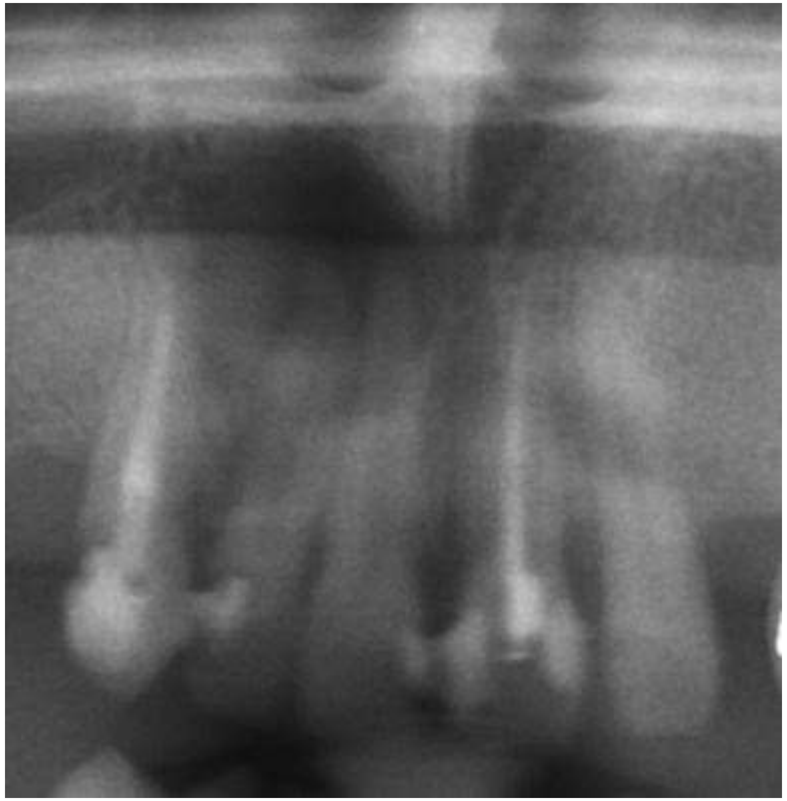

KR8 – HE, CK17, bcl2 (x1)

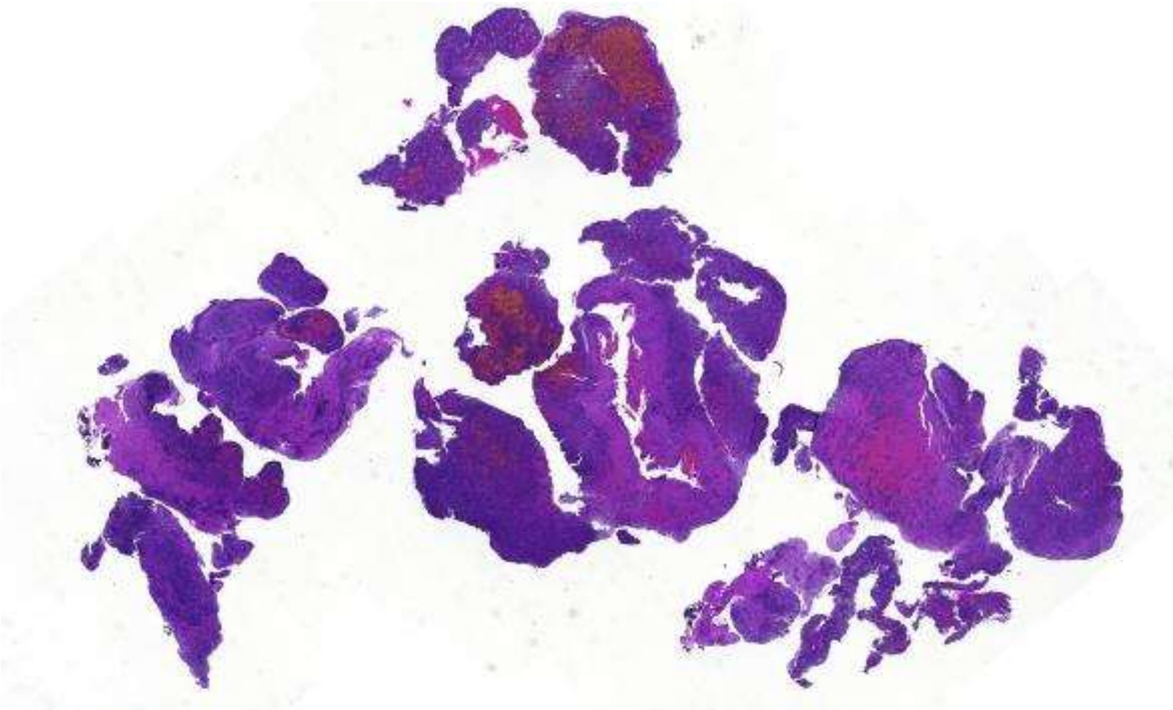

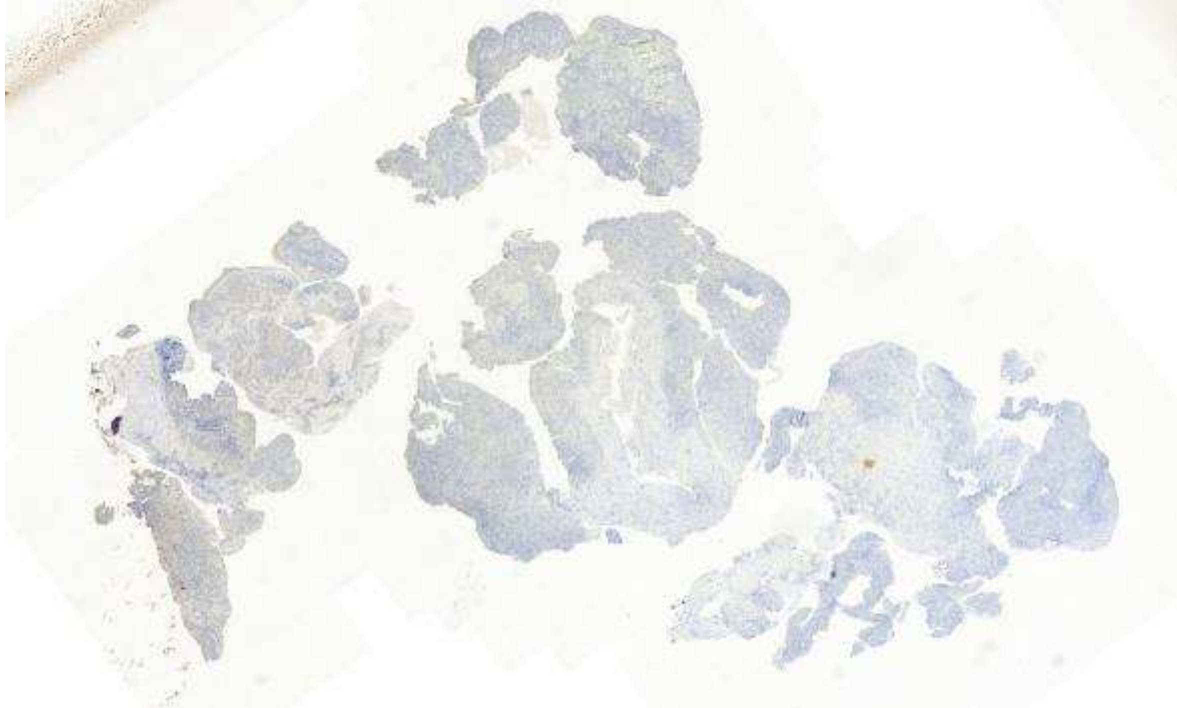

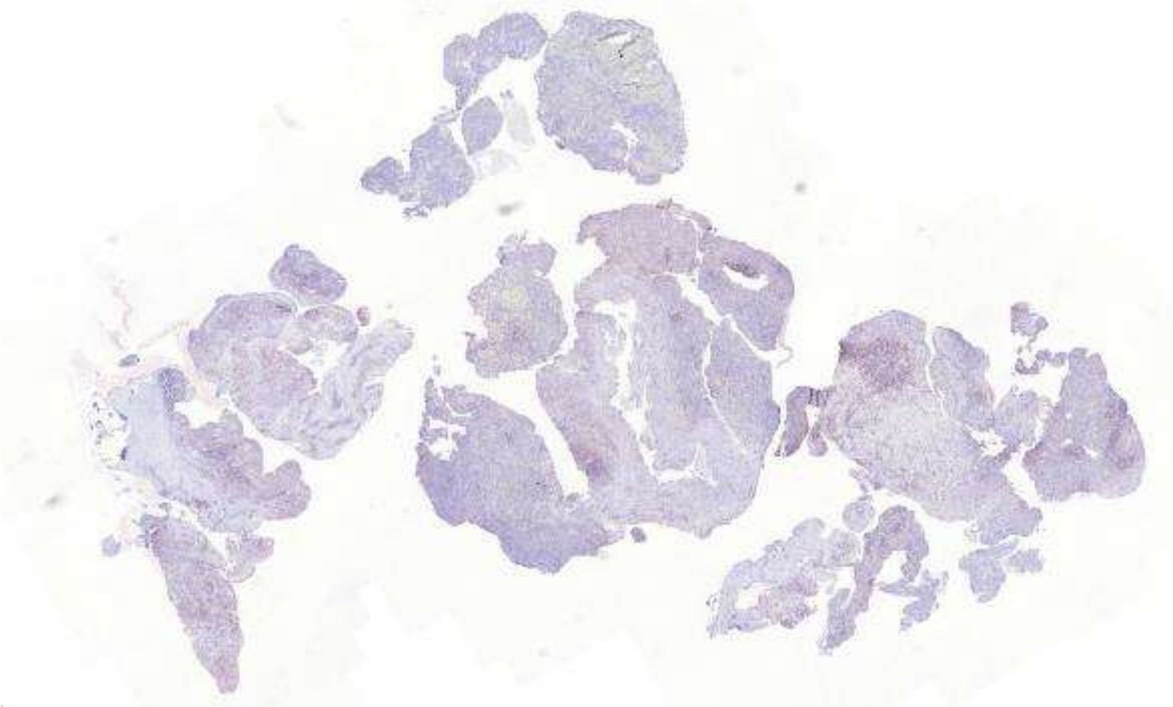

KR8 – HE, CK17, bcl2 (x12)

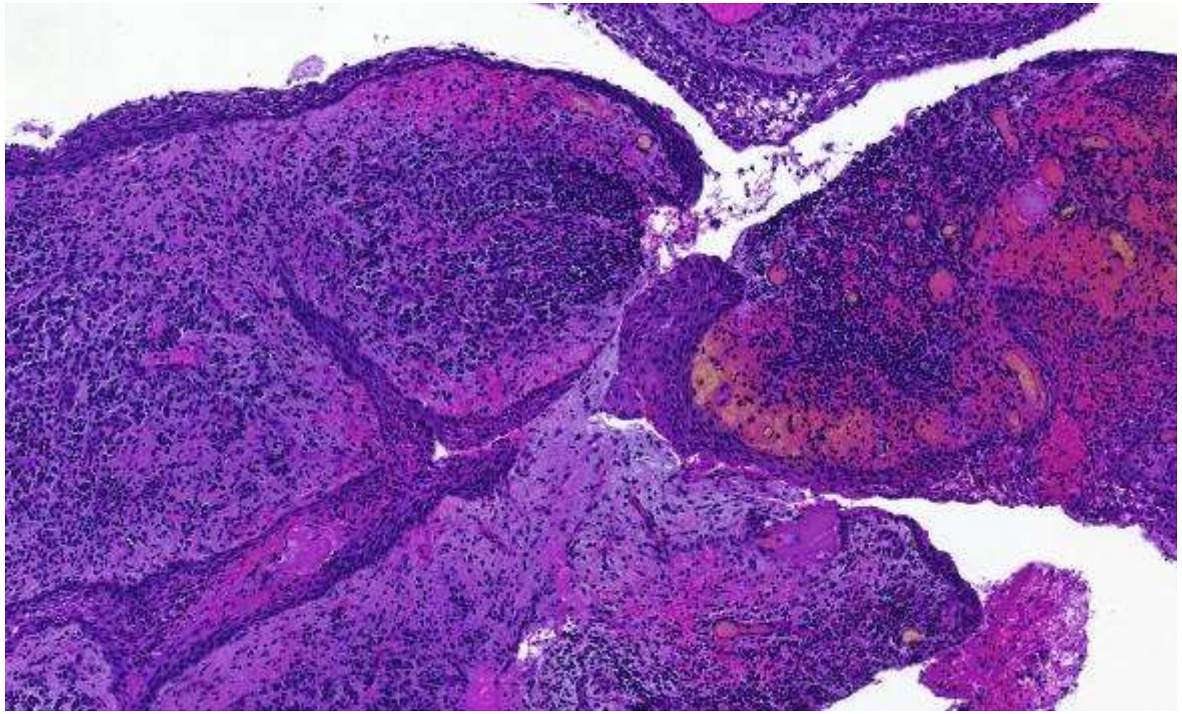

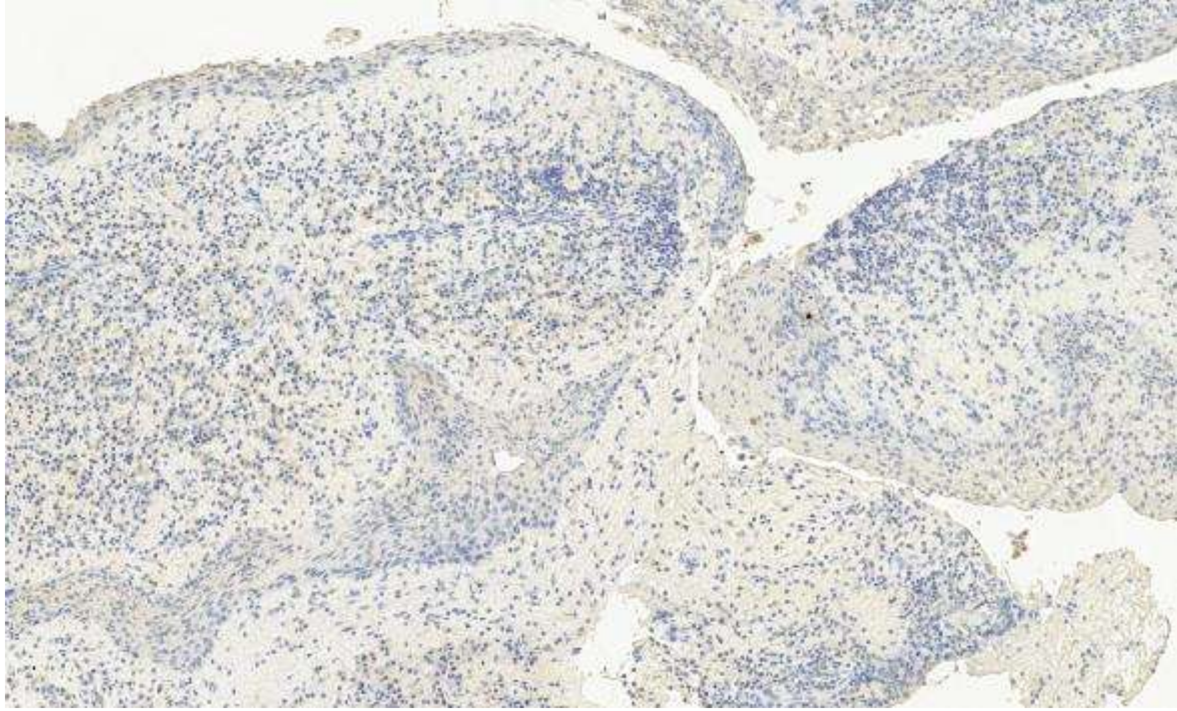

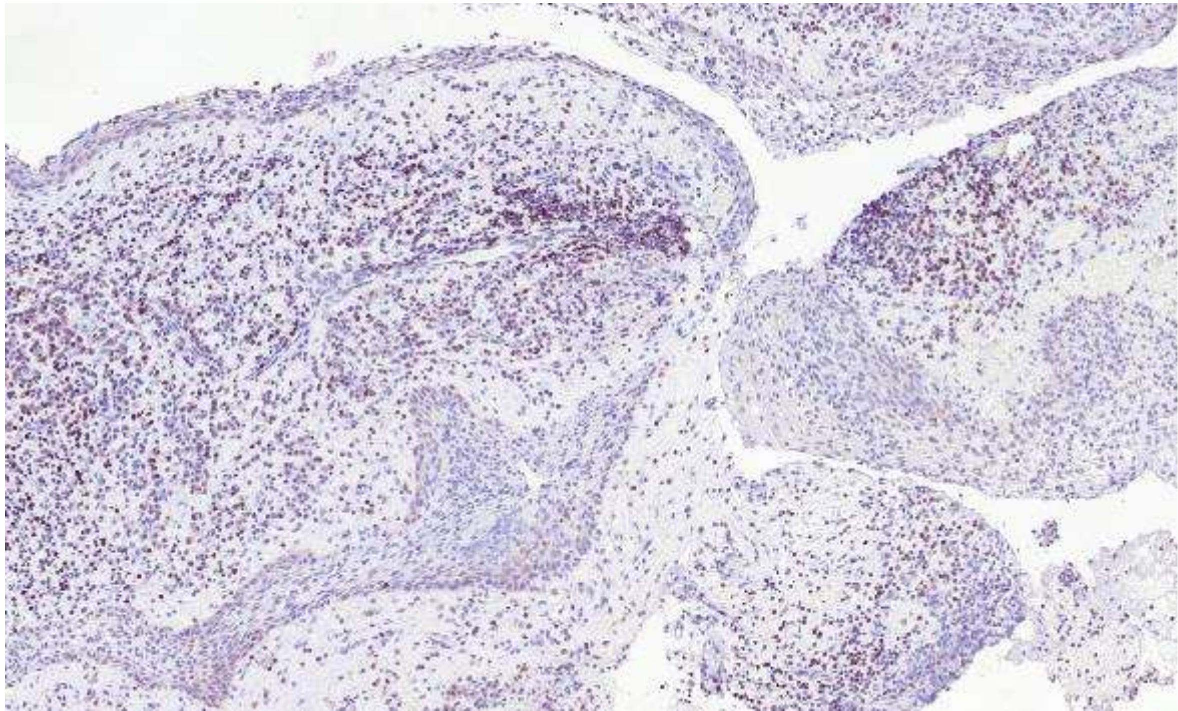

KR8 – HE, CK17, bcl2 (x30)

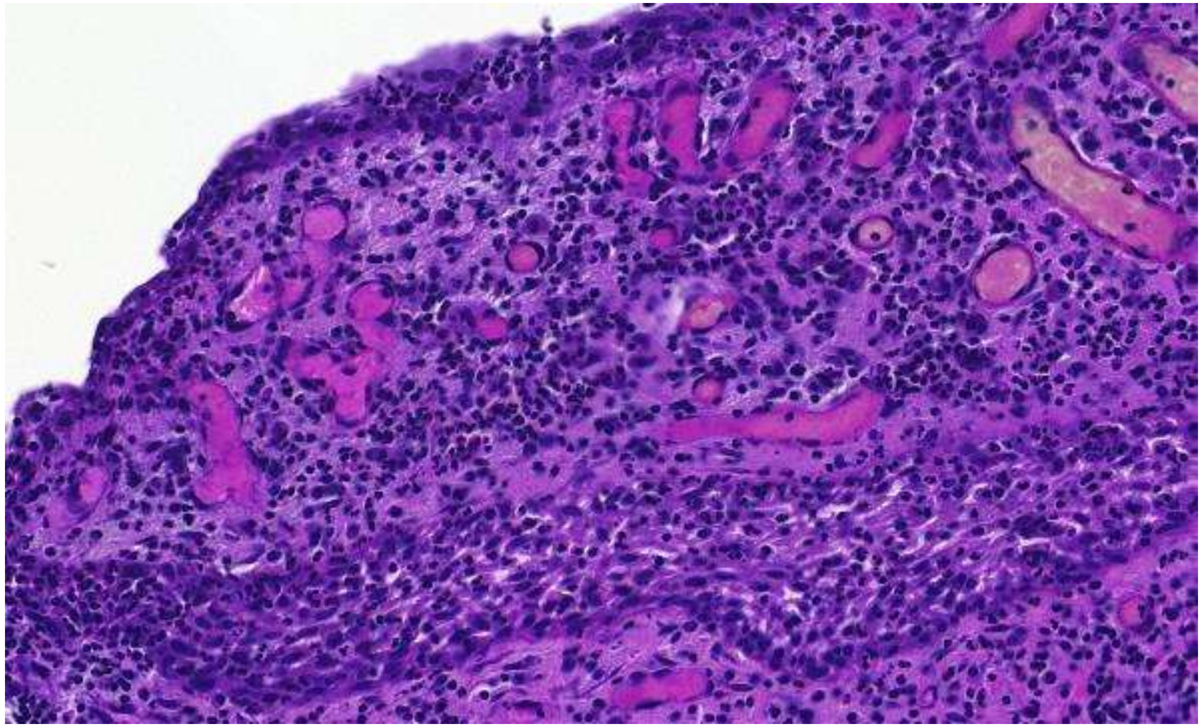

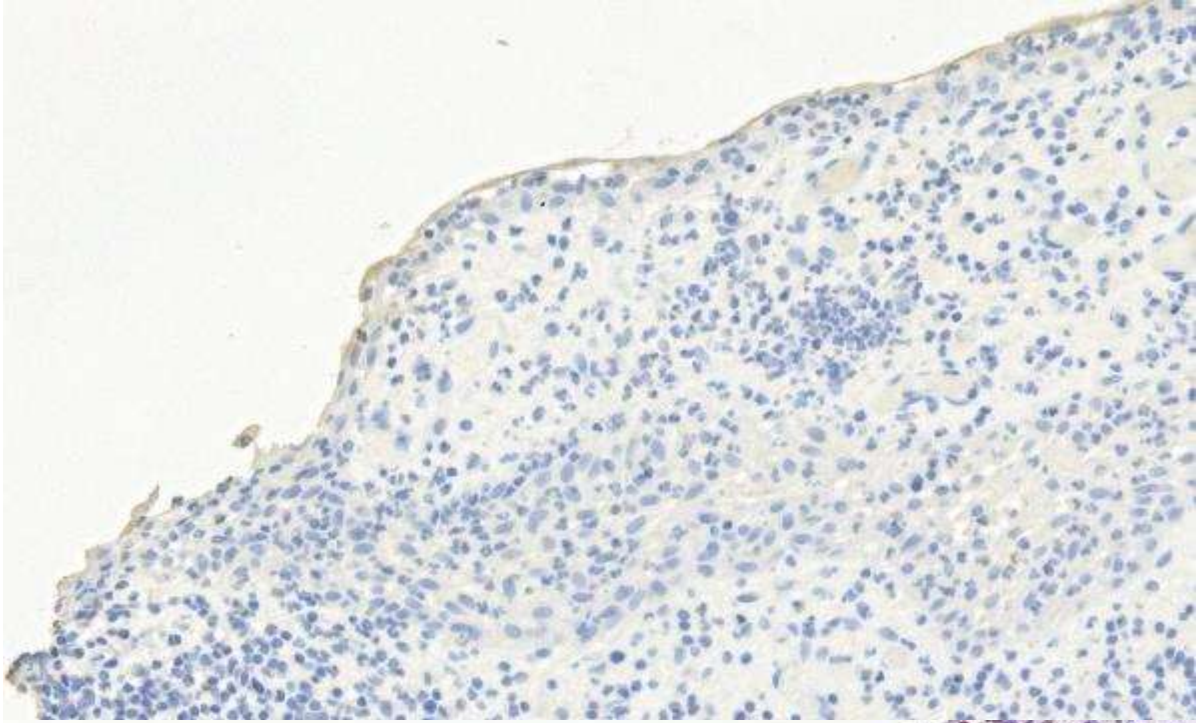

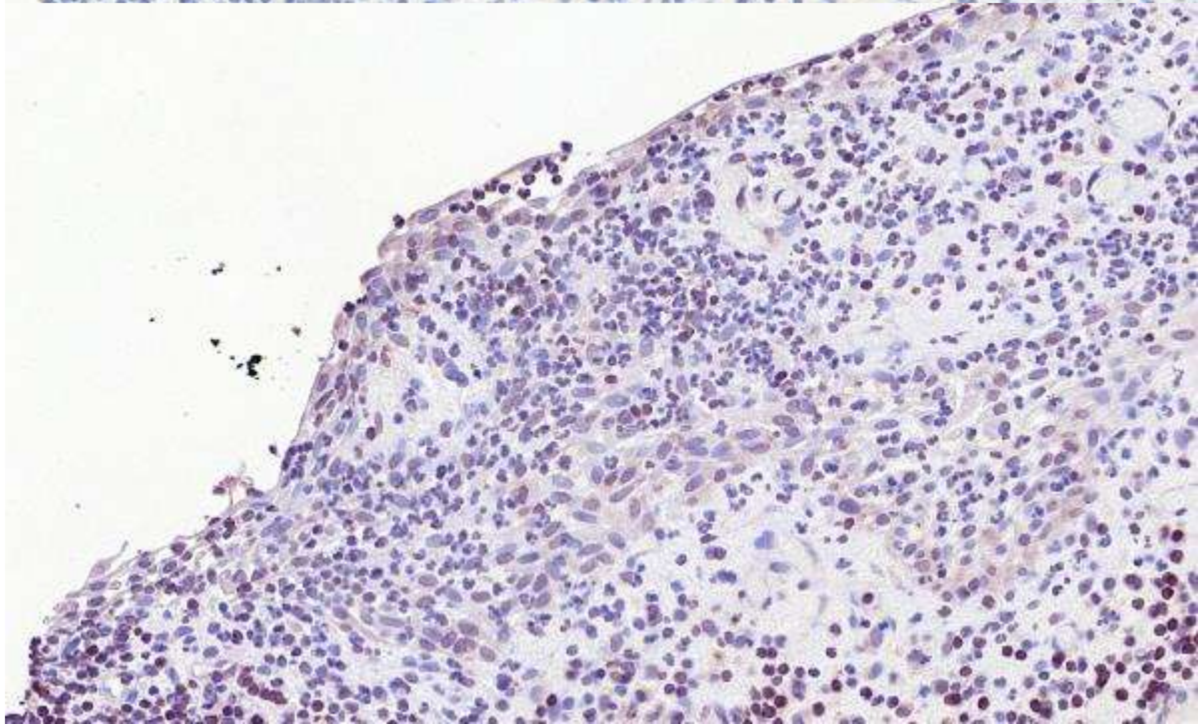

KR8 – Conclusion

- H&E: partly thick, partly thin squamous epithelium, no parakeratosis, no wavy surface, no basal palisading, uneven (partly even) epithelial-subepithelial contours
- Mixed inflammatory infiltrate of the cyst wall with massive neutrophilic component
- Bcl2: negative (uncertain faint basal (/suprabasal) staining incomparable with control lymphocyte positivity – or non specific background staining)
- CK17: dominantly negative, focal superficial staining
- Histopathology: inflammatory odontogenic cyst, radicular cyst
- Combined clinical and pathological opinion: radicular cyst

Case KR9

- 62-year-old male
- History: surgical removal of an OKC from the left maxilla 14 months before
- Dental hygiene/status: not recorded
- Radiology: CT: hypodense 6.8-mm-large cystic lesion associated with a disruption of the ventral wall of the left maxillary sinus, which itsef is partly filled with a soft tissue density lesion
- Operation: in toto removal of the cyst (corresponding to the place of missing teeth 22-24) accroaching to the basal mucosa of the left maxillary sinus and protruding into the maxillary sinus
- Clinical diagnosis: recurrent OKC

KR9 – OPT before the removal of the original OKC 14 months ago

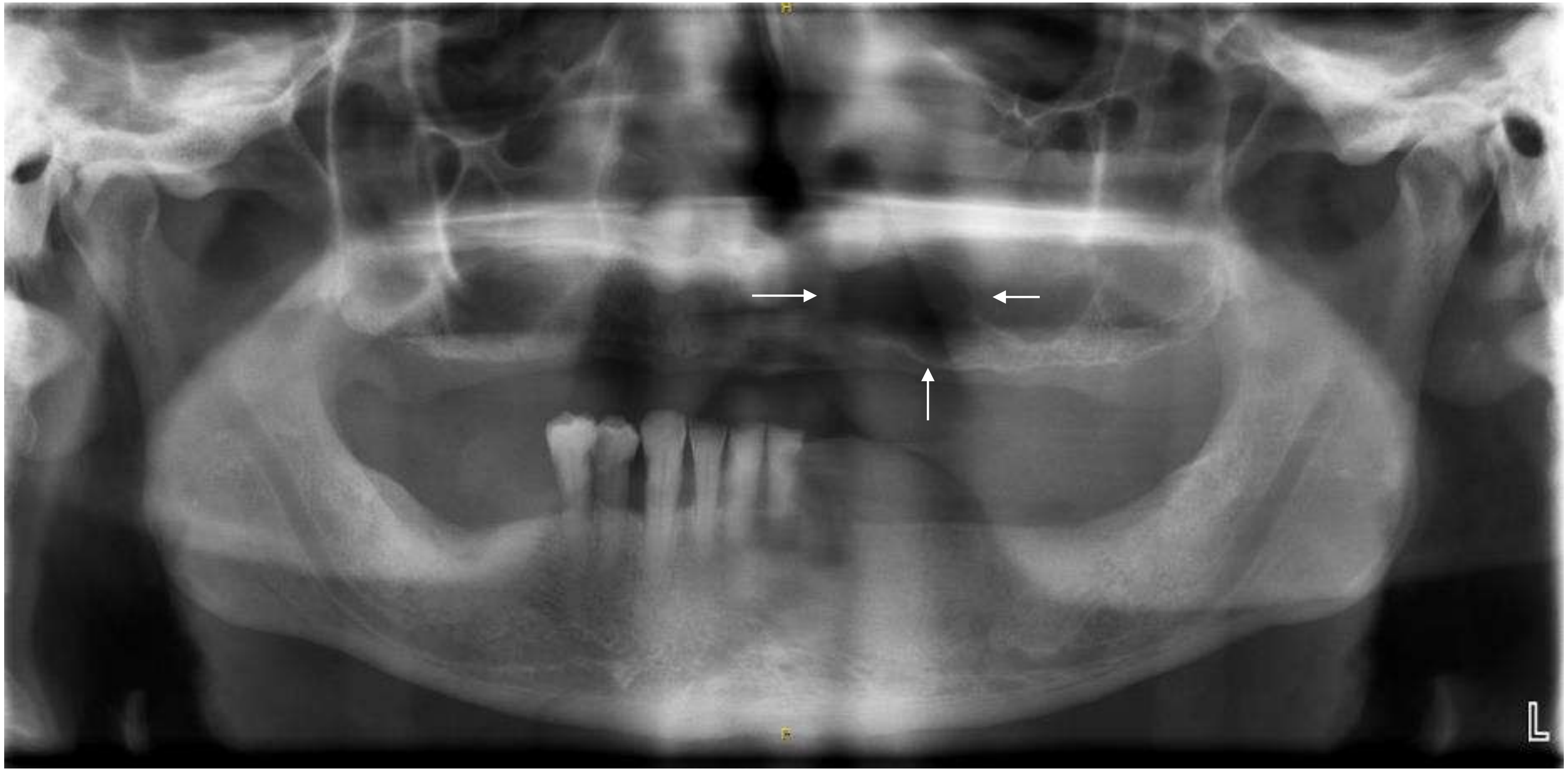

KR9 – CT coronal

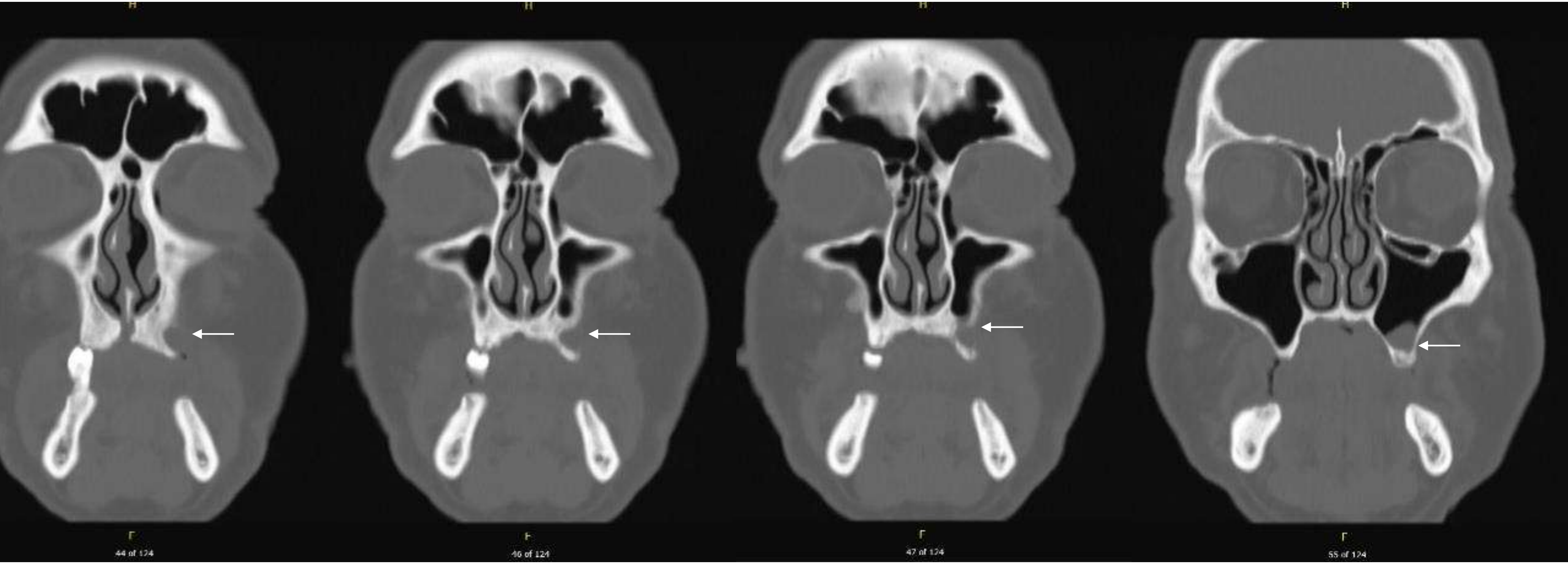

KR9 – CT sagittal

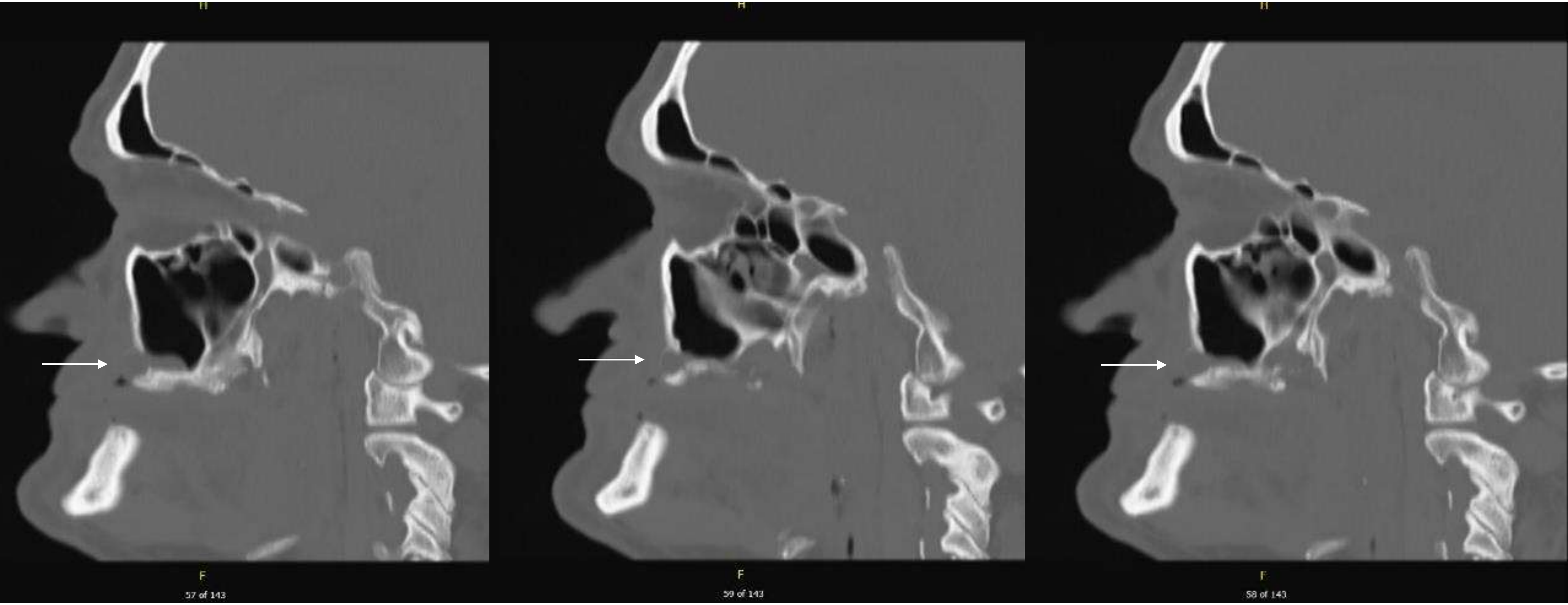

KR9 – CT axial

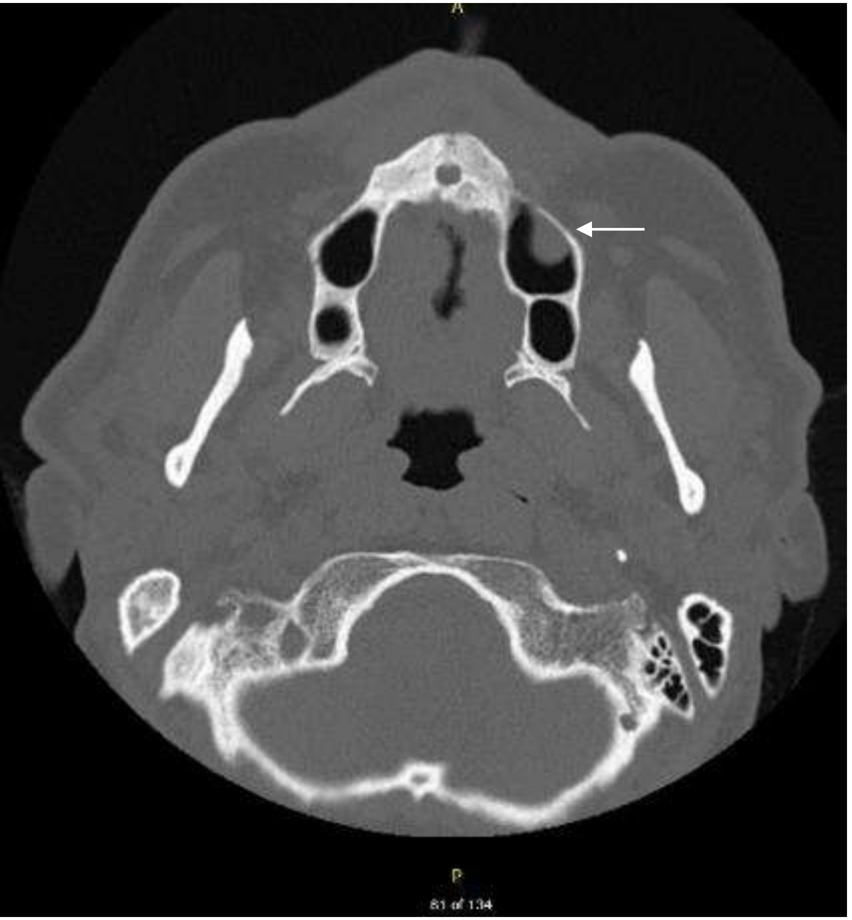

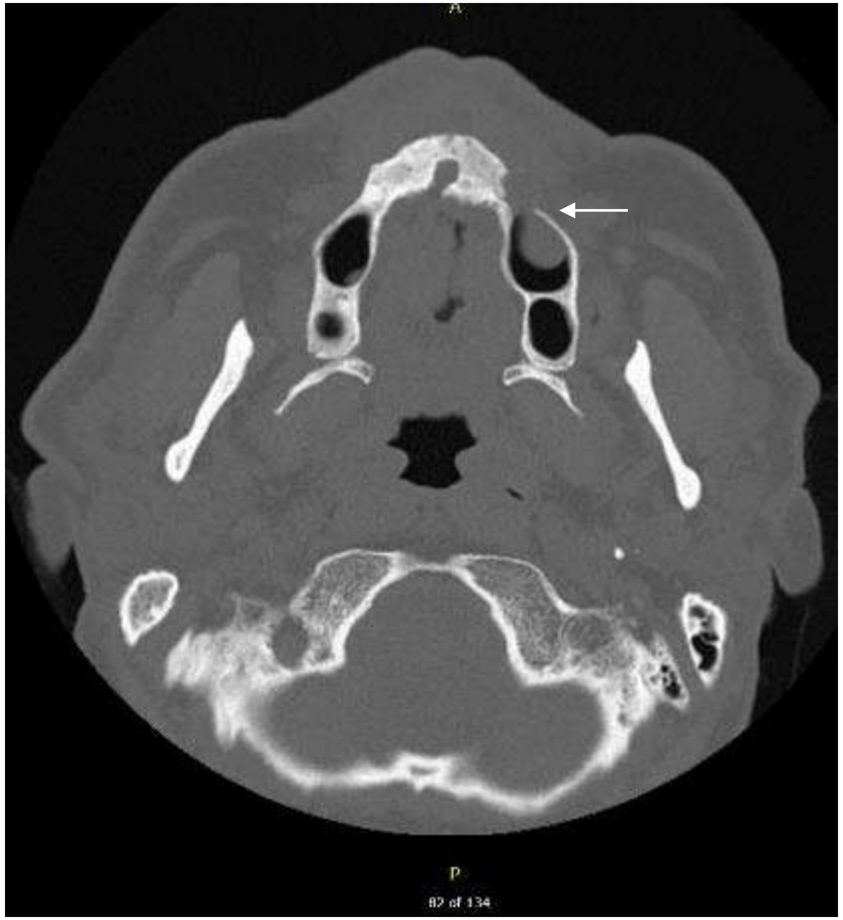

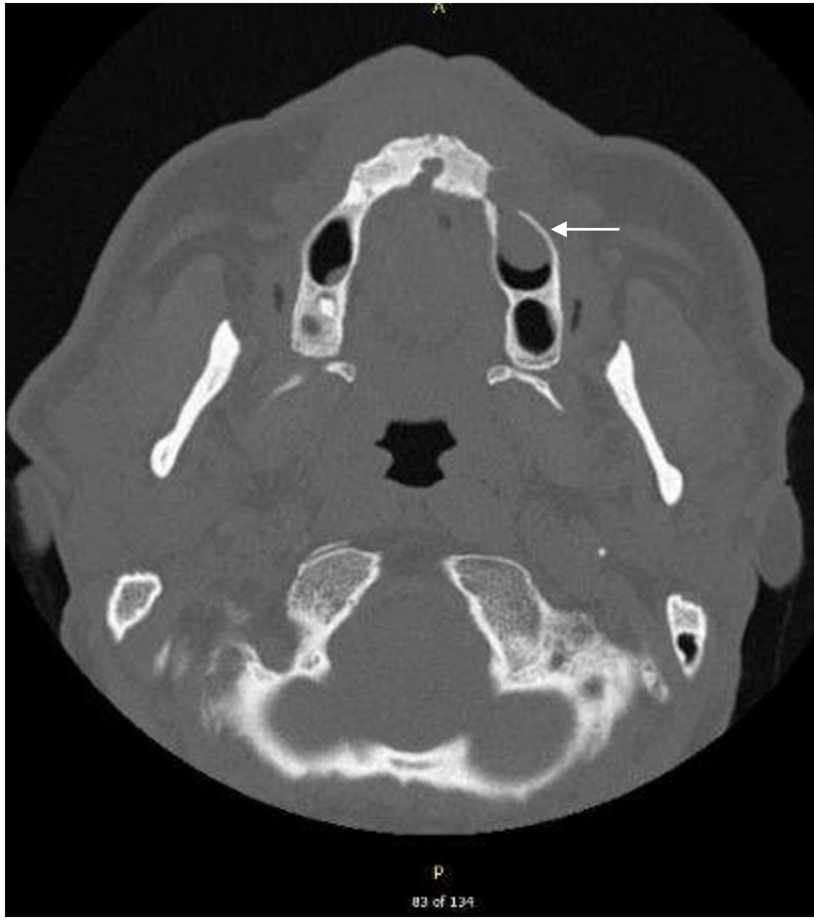

KR9 – HE, CK17, bcl2 (x3)

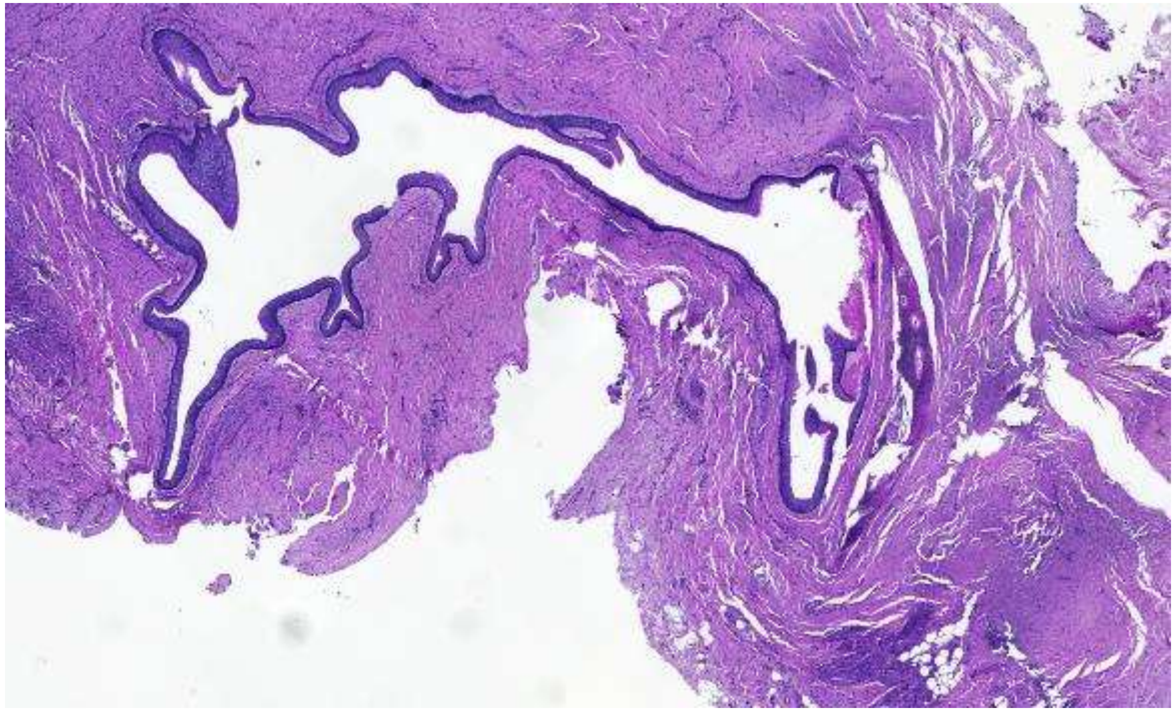

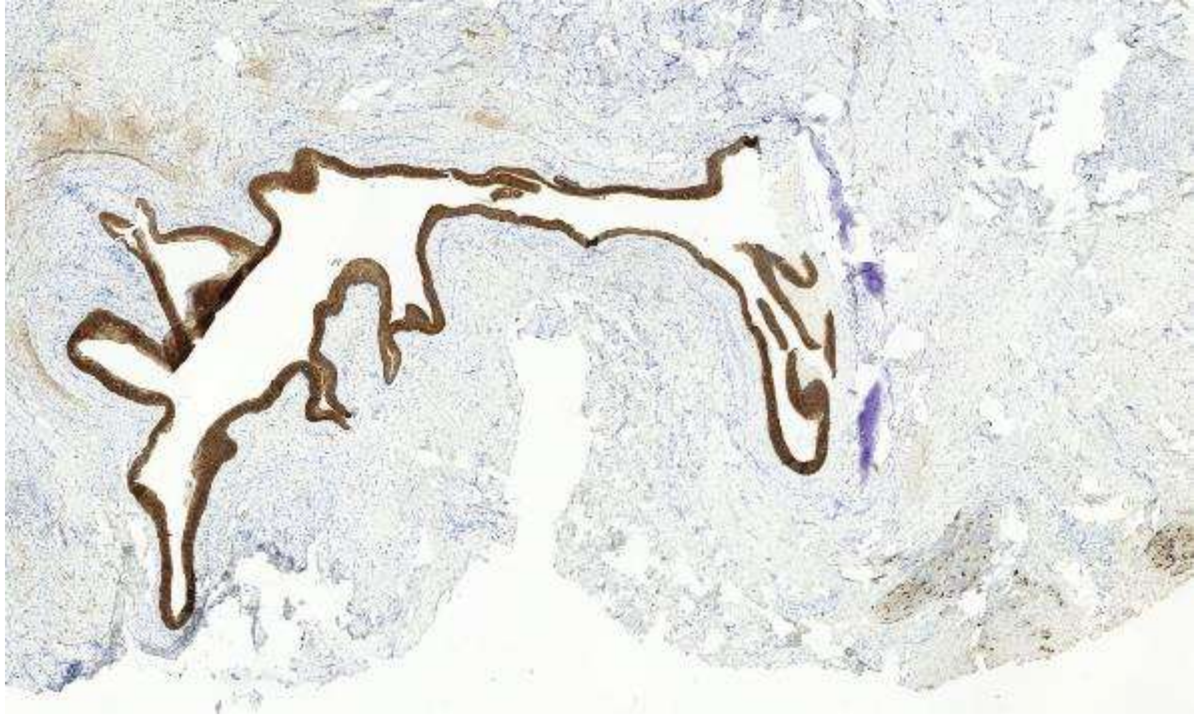

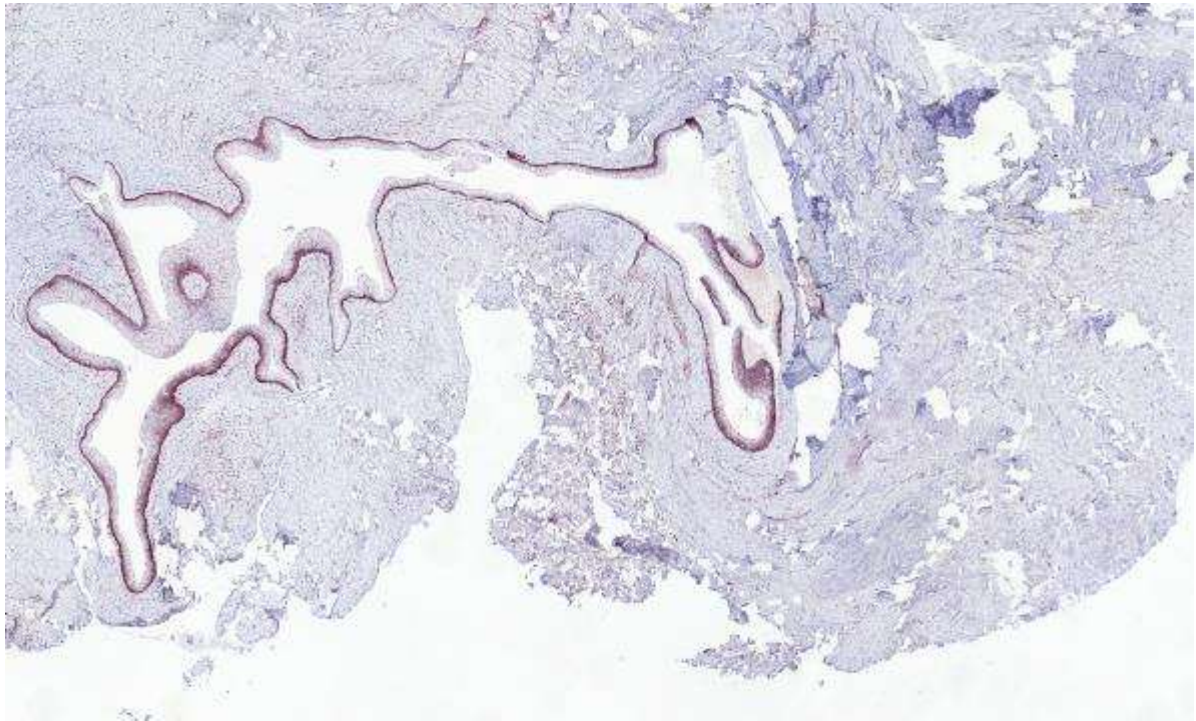

KR9 – HE, CK17, bcl2 (x10)

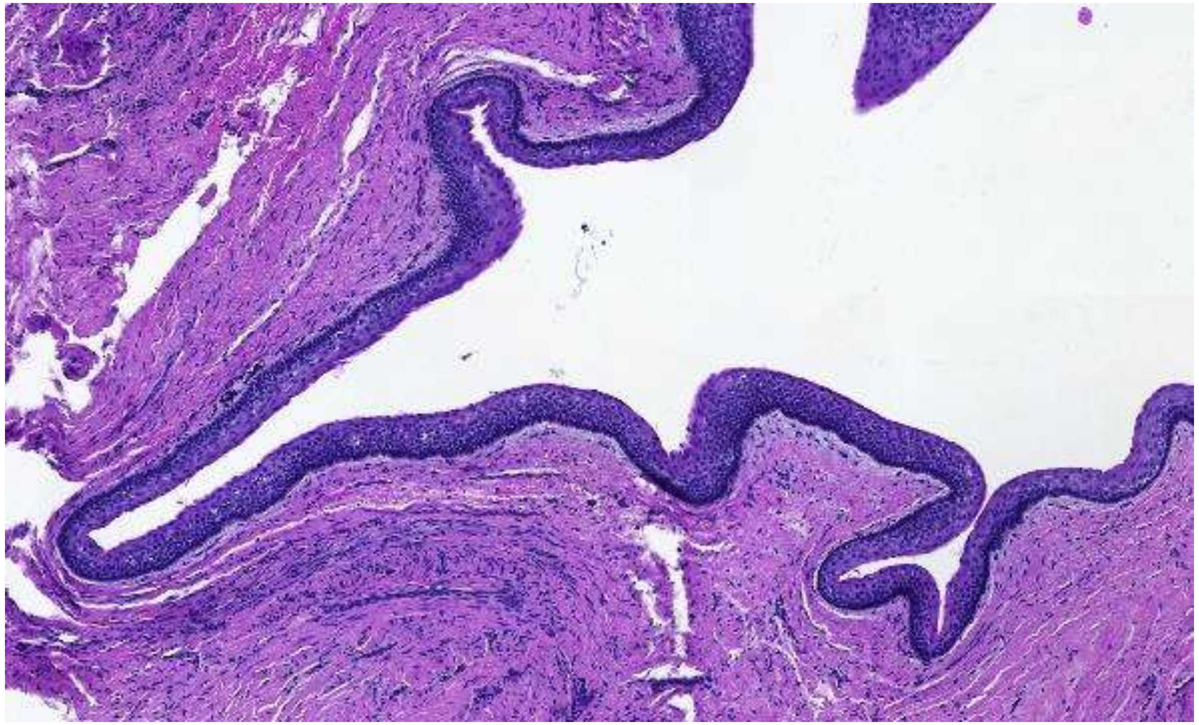

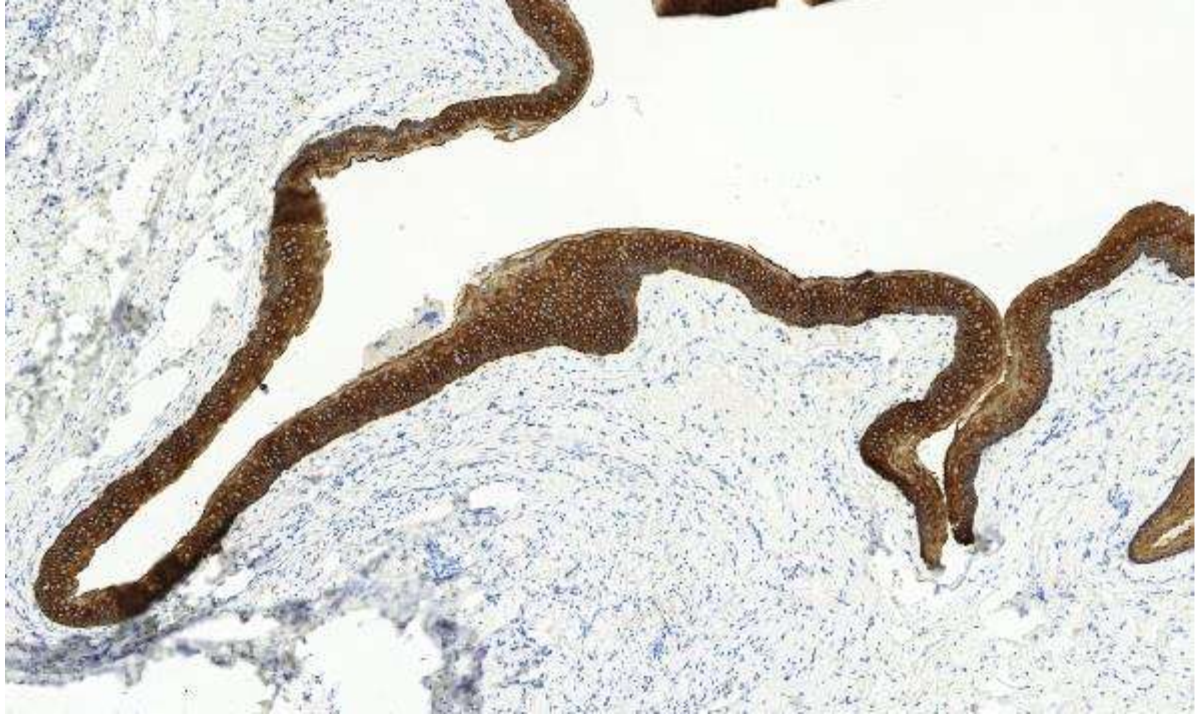

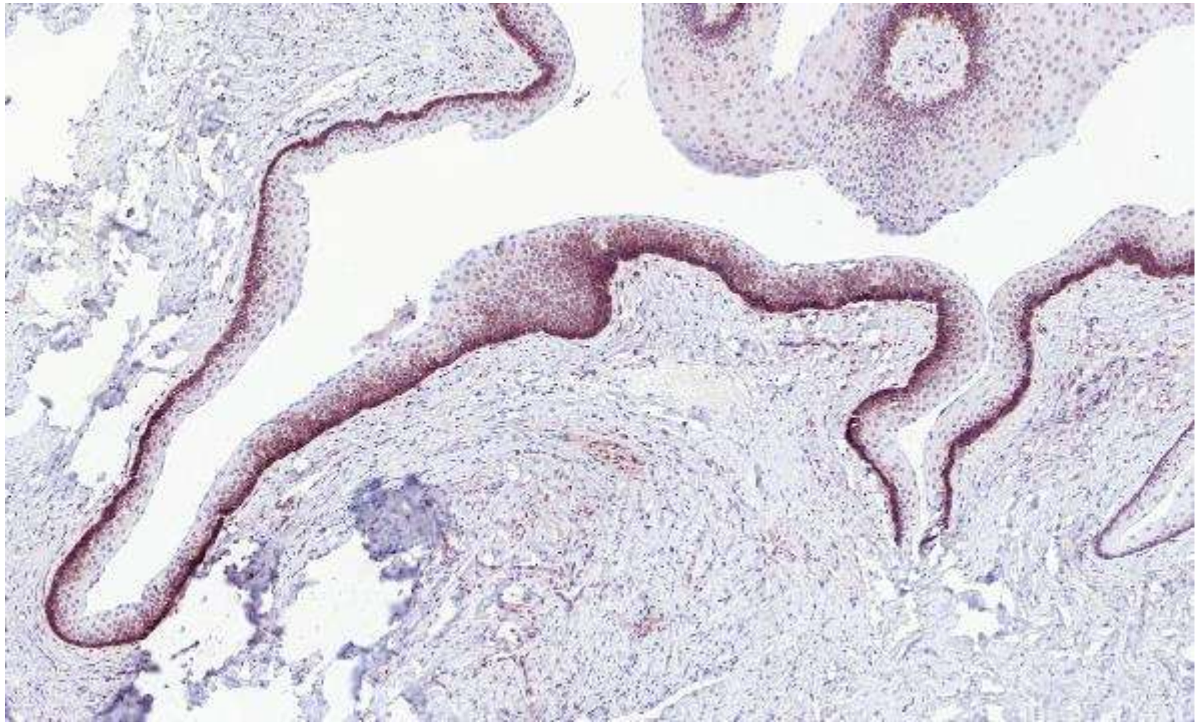

KR9 – HE, CK17, bcl2 (x25)

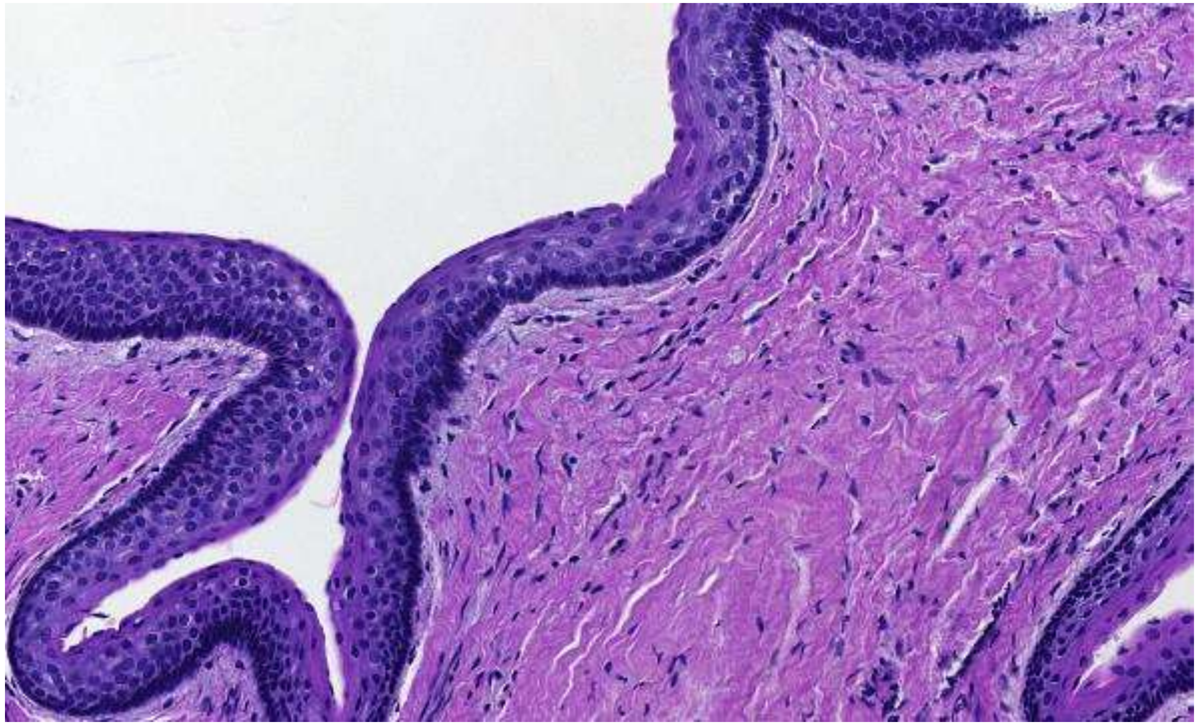

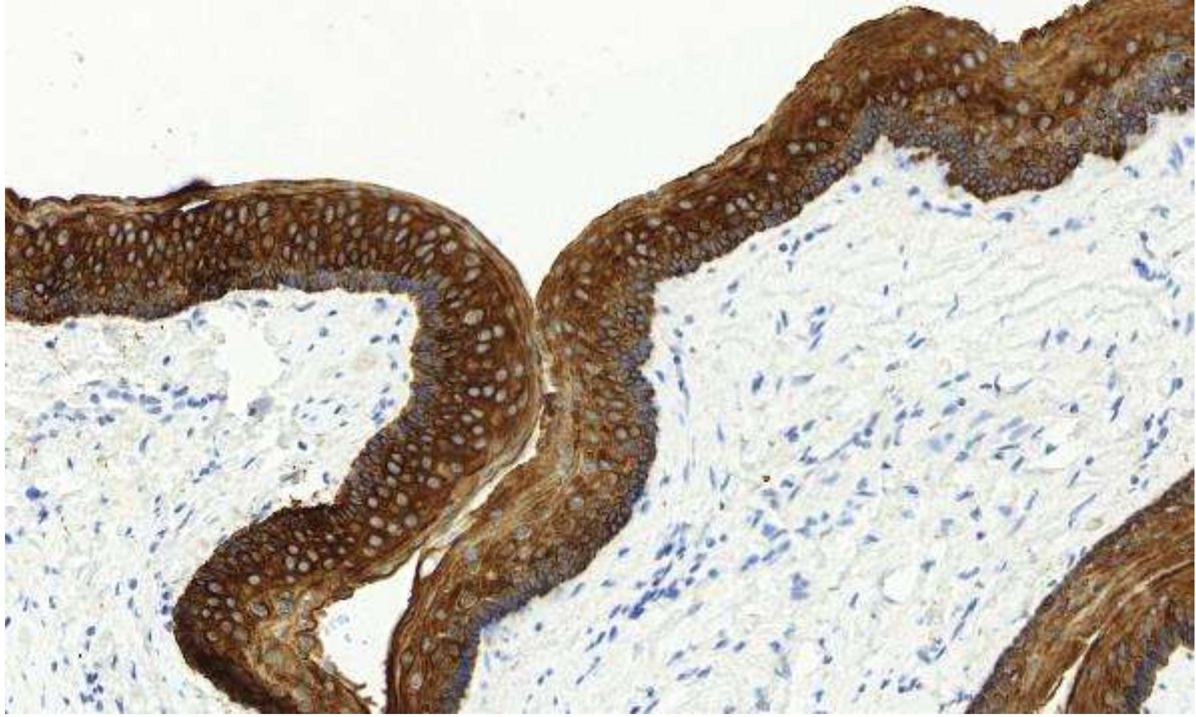

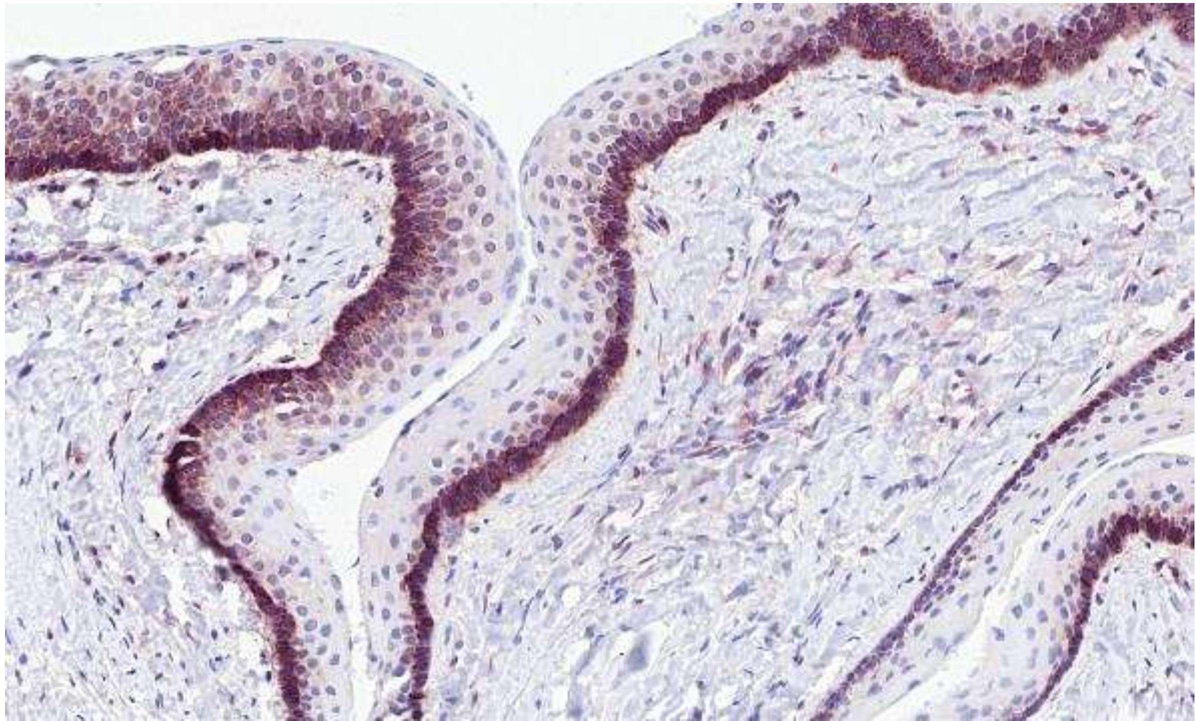

KR9 – Conclusion

- H&E: relatively thin squamous epithelium with parakeratosis, focal wavy surface, basal palisading, even epithelial-subepithelial contours
- Focal inflammation of the cyst wall
- Bcl2: strong diffuse basal /suprabasal staining
- CK17: strong diffuse transepithelial staining
- Histopathology: recurrent OKC
- Combined clinical and pathological opinion: recurrent OKC

Case KR10

- 36-year-old male
- Dental hygiene/status: normal, conserved, treated teeth
- Radiology: impacted 18 displaced cranially, large cyst around
- Operation: removal of the cyst and tooth 18 in toto; 2.5x2x1 cm cyst adhering to the neck of the tooth
- Clinical diagnosis: dentigerous cyst / OKC?

KR10 – OPT

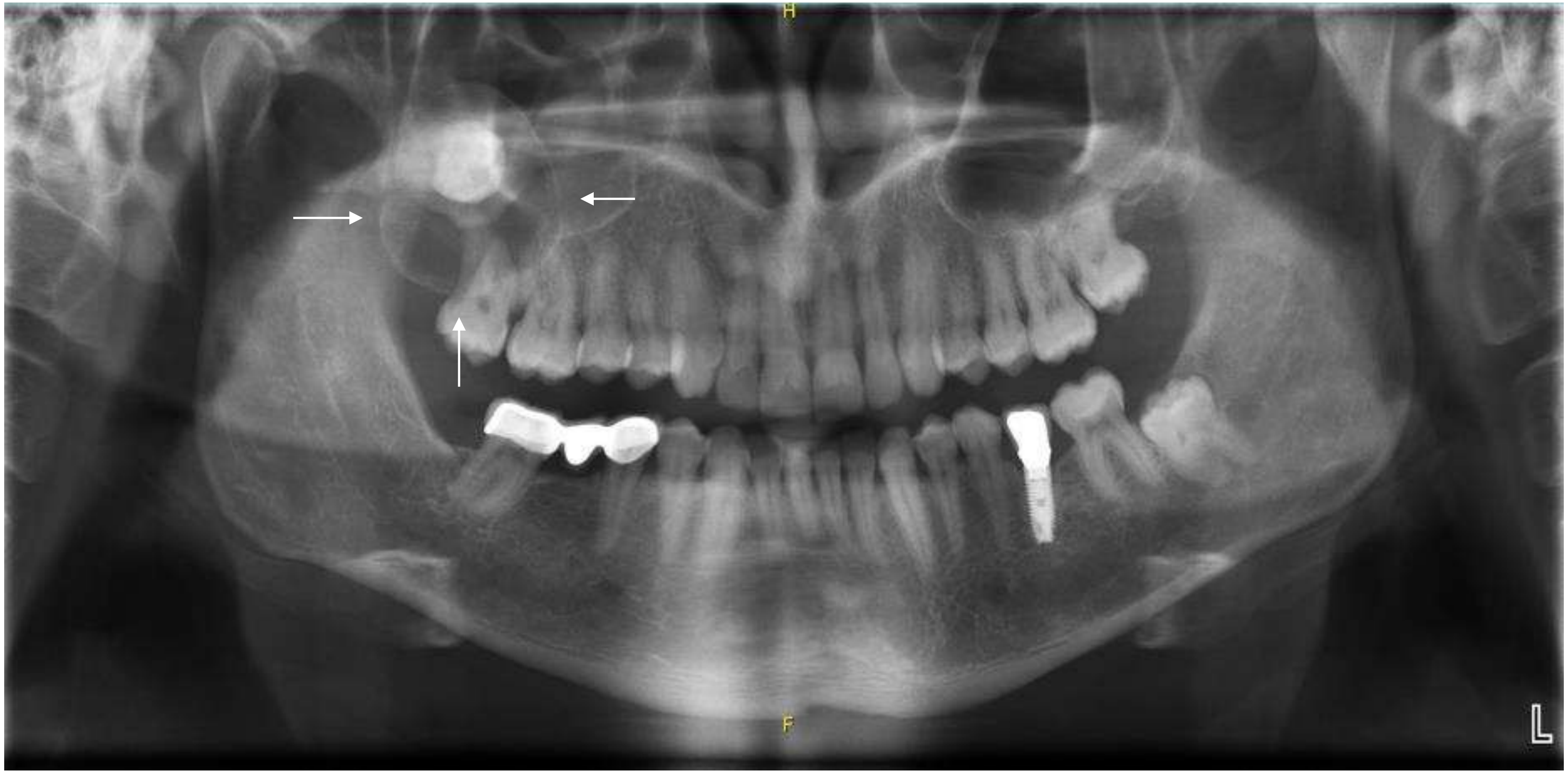

KR10 – HE, CK17, bcl2 (x0.9)

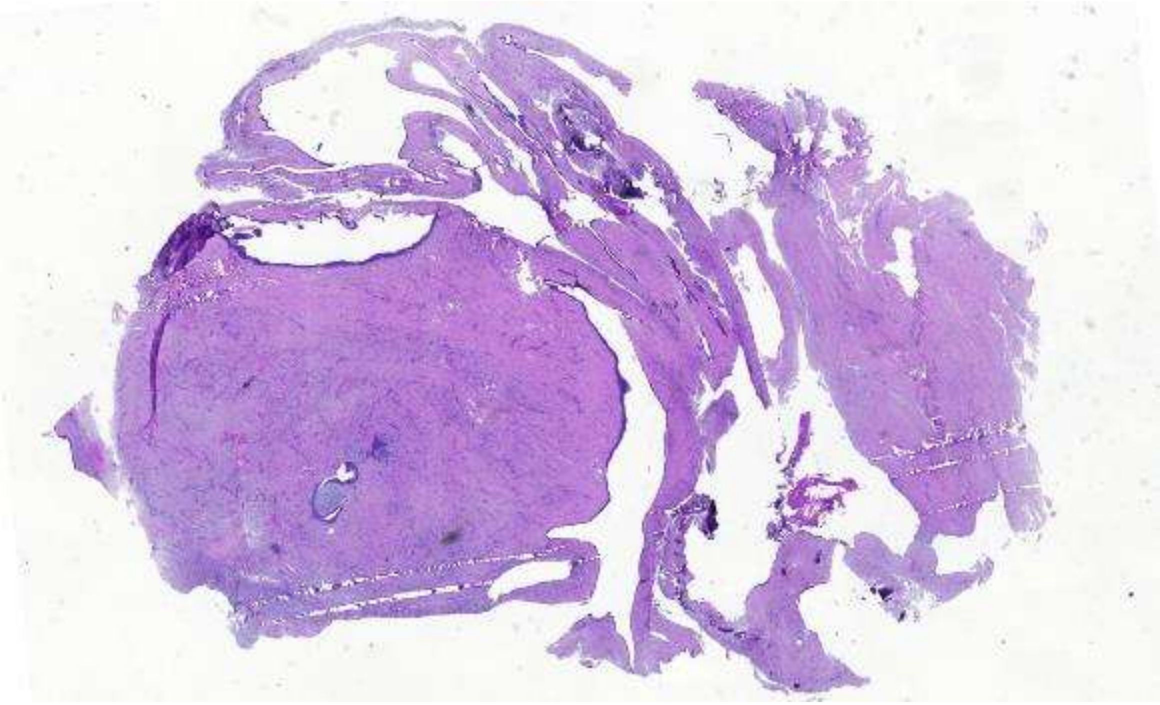

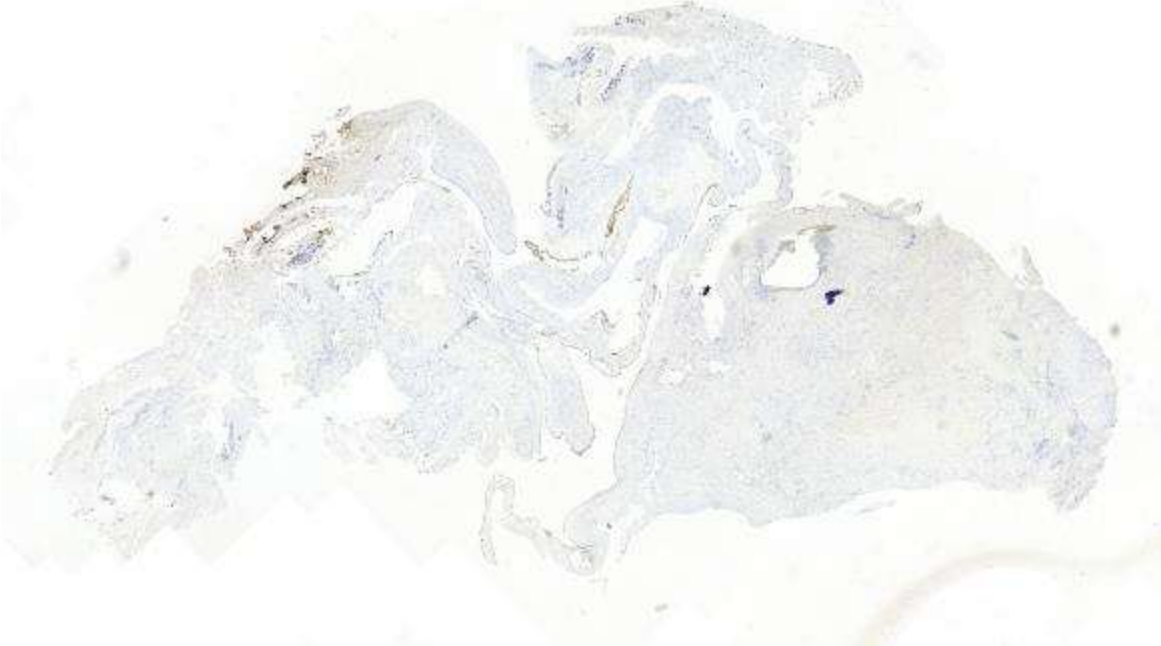

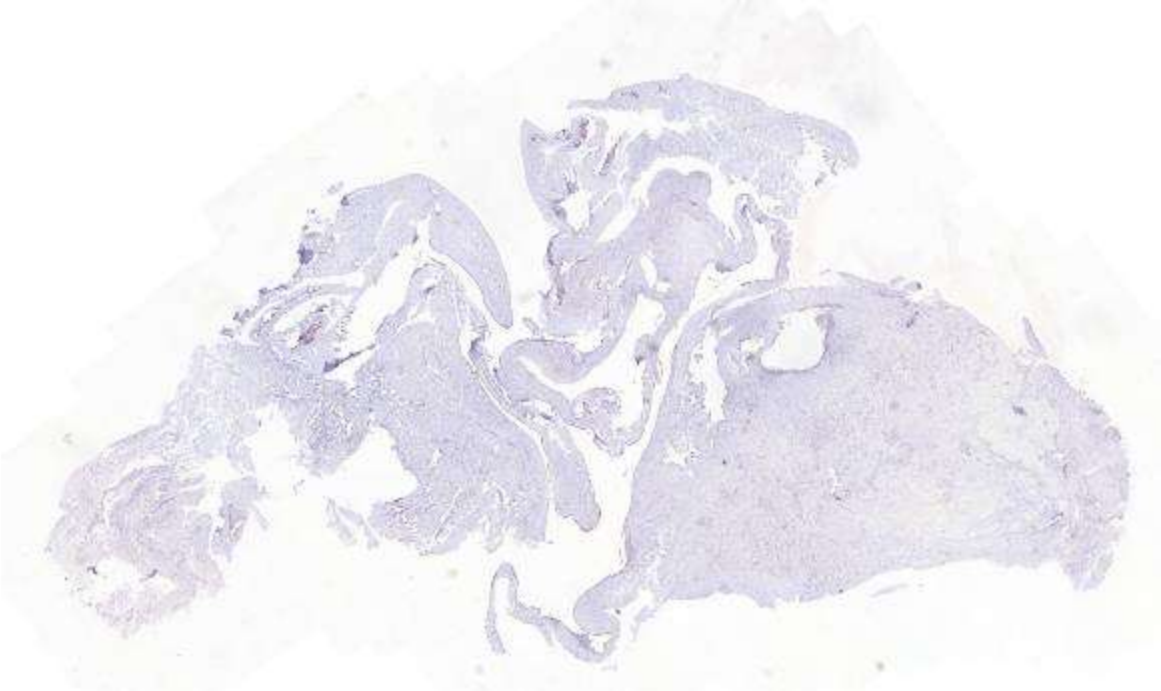

KR10 – HE (x15), CK17 (x5) bcl2 (x5)

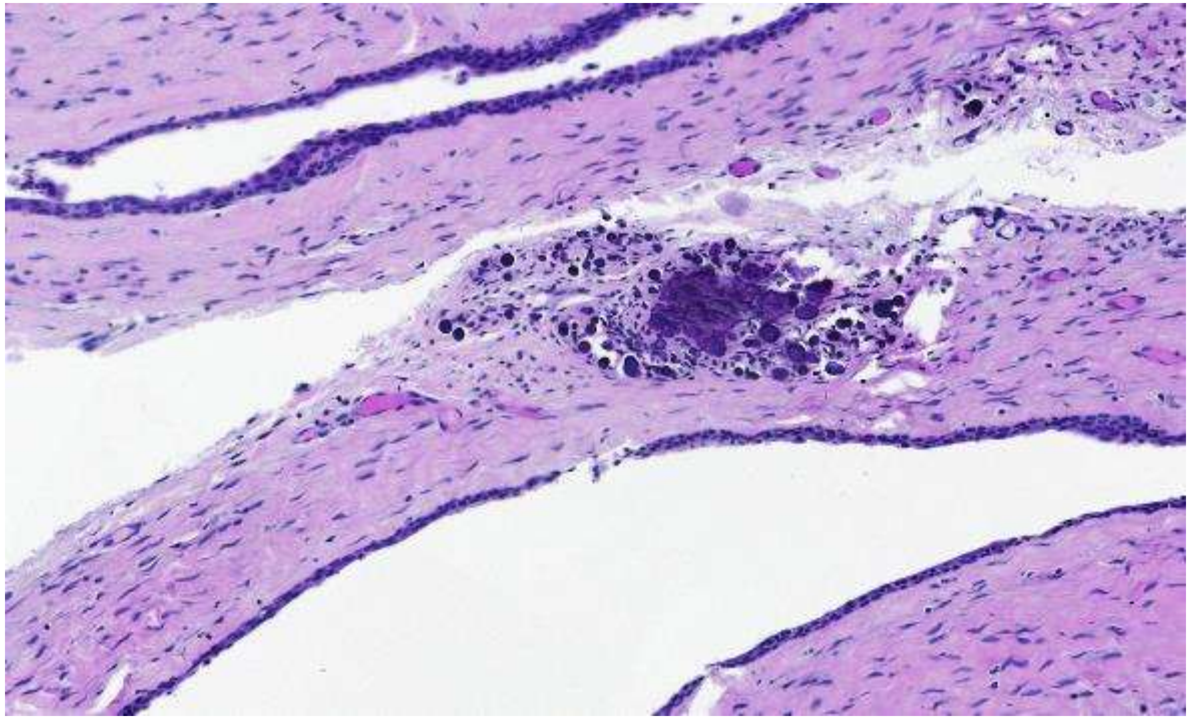

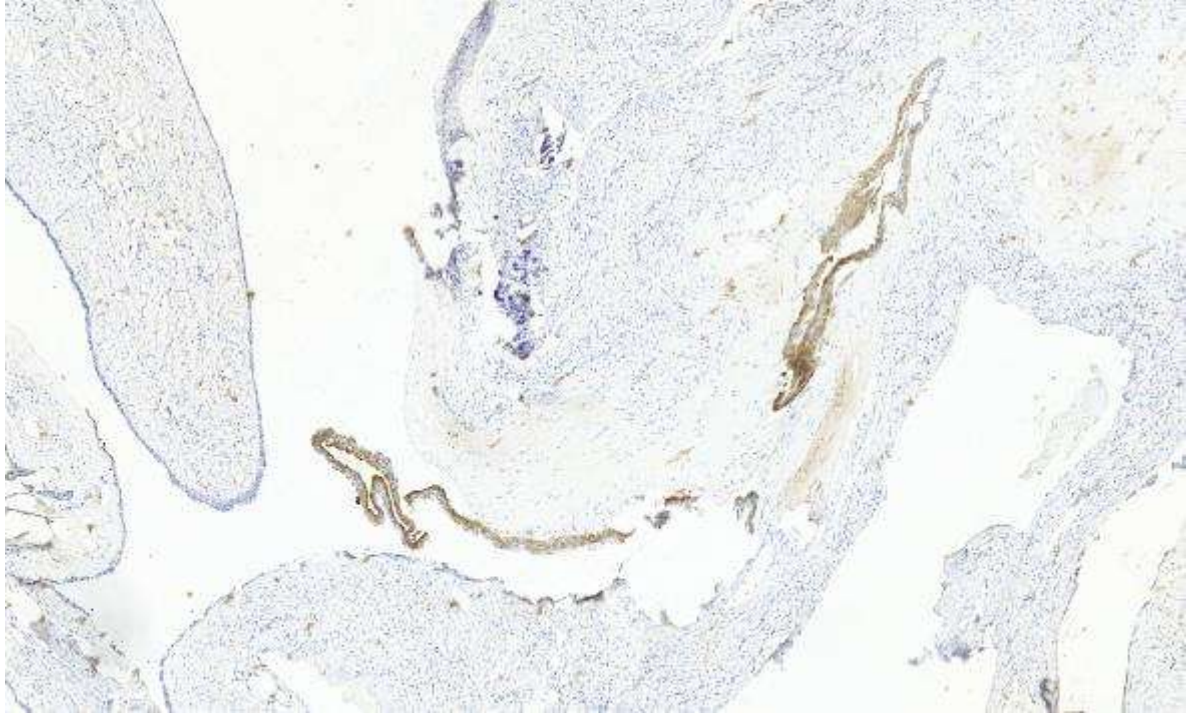

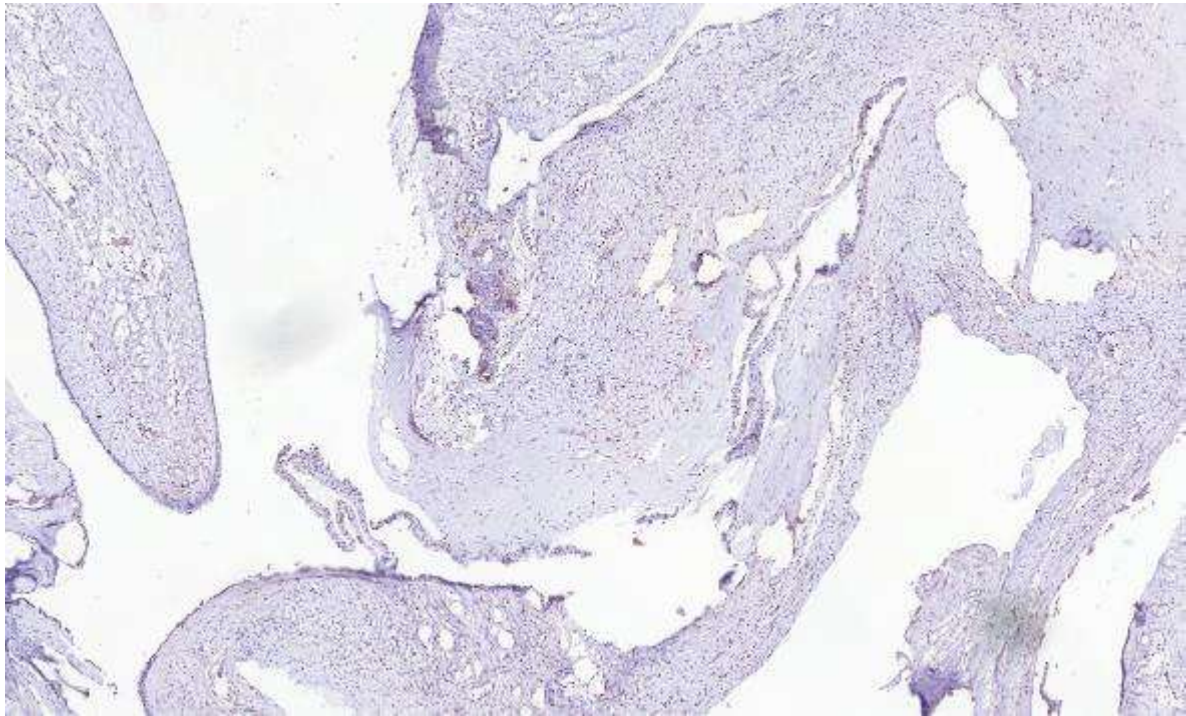

KR10 – CK17 (x20) superficially positive /negative

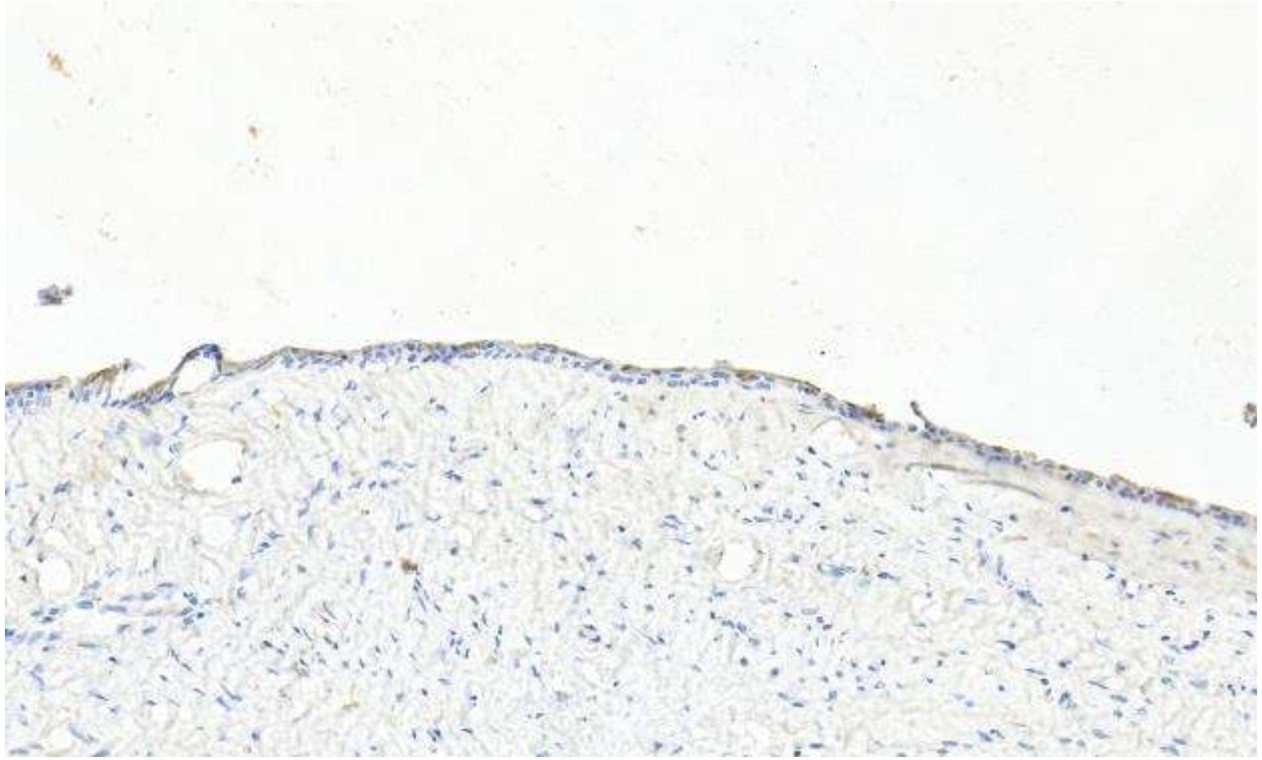

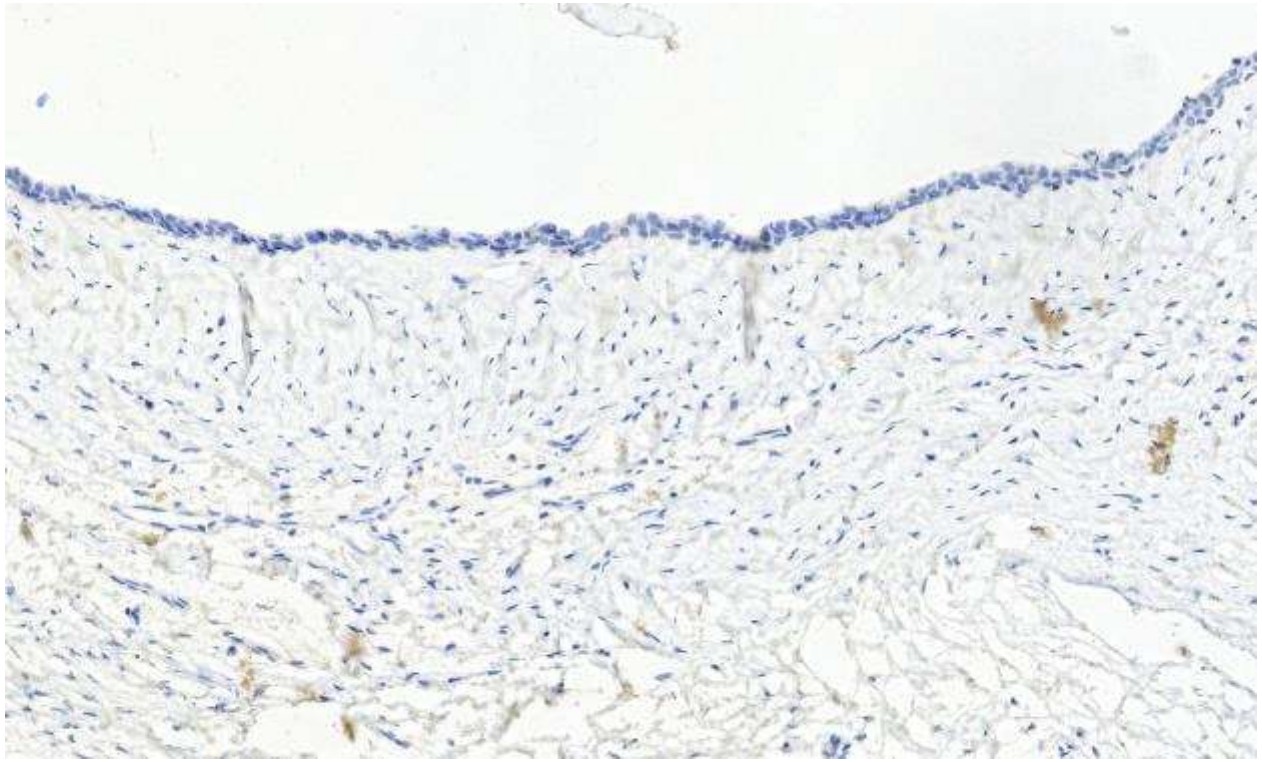

KR10 – HE (x2 & x5) inflammation

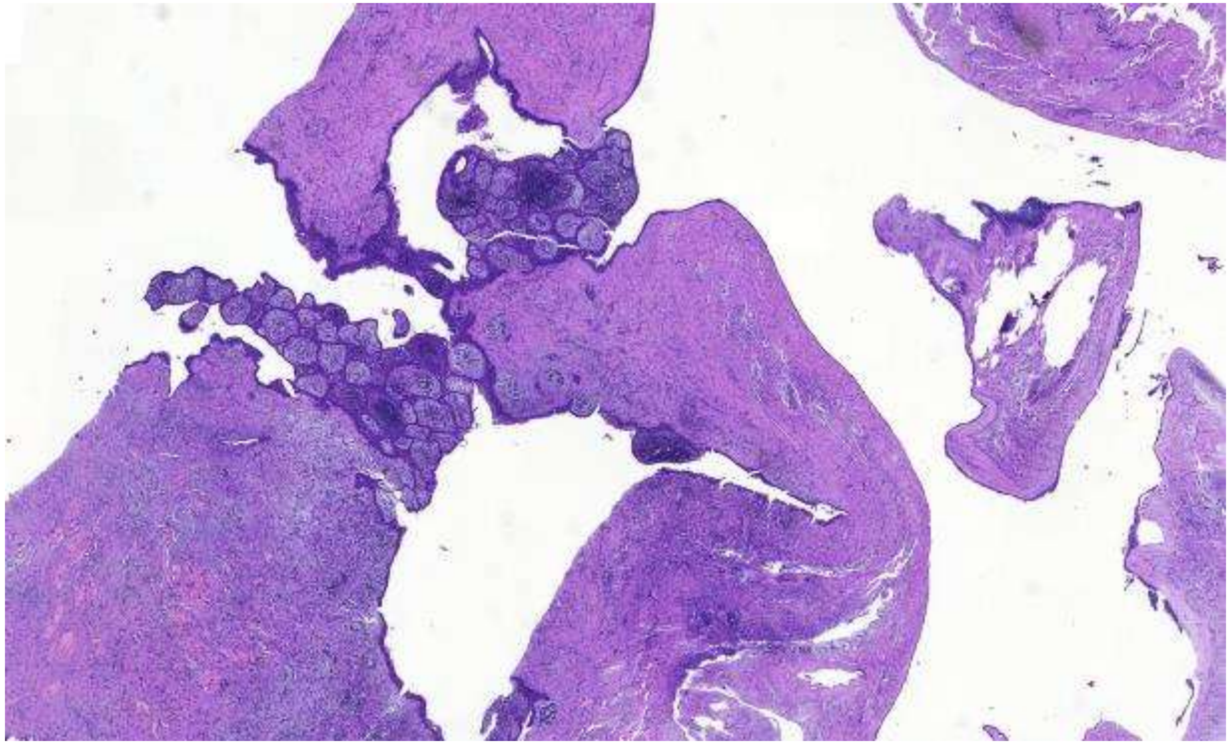

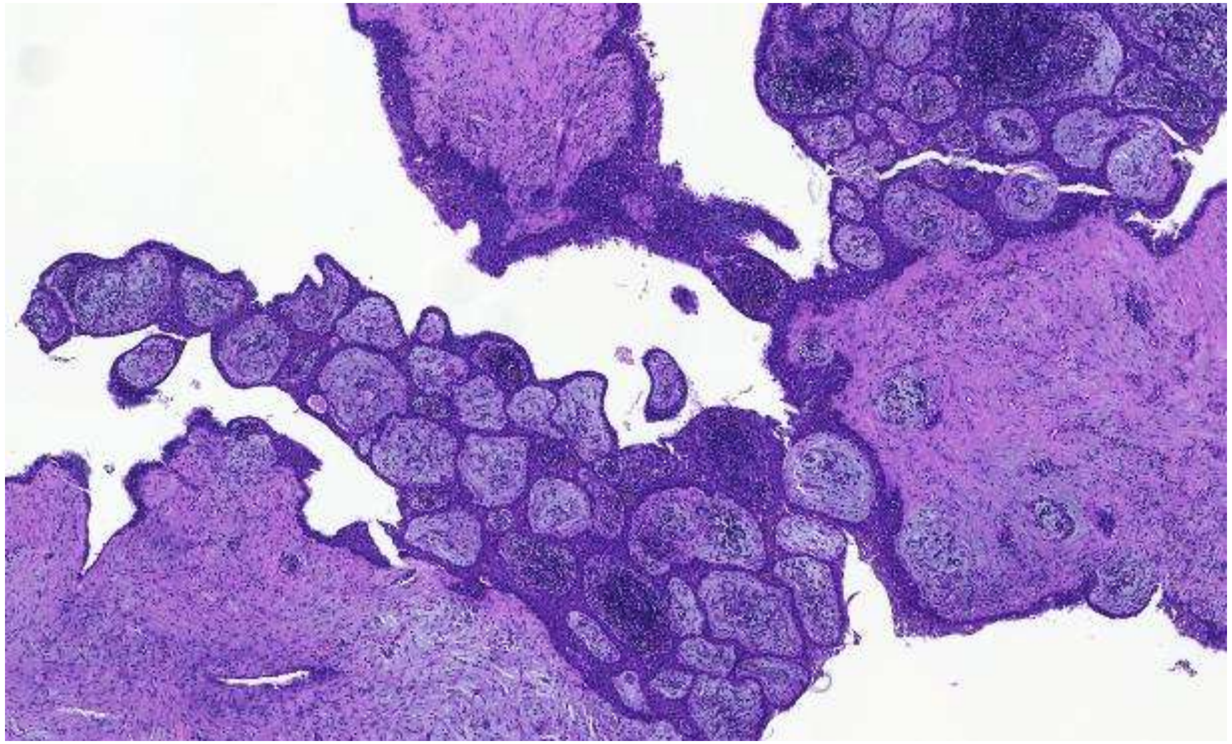

KR10 – Conclusion

- H&E: dominantly very thin (2 cell layers), partly slightly thicker (at the site of inflammation) epithelium, no parakeratosis, no wavy surface, no basal palisading, even epithelial-subepithelial contours; minimal surface mucous epithelium (metaplasia)
- Focal mixed inflammation and oedema of the cyst wall which is basically not inflamed (with lympocytes and plasma cells predominating and neutrophils being present only focally)
- Bcl2: negative
- CK17: dominantly negative, focal superficial /and upper spinous /or transepithelial staining
- Histopathology: radicular cyst (wrong diagnosis)
- Combined clinical and pathological opinion: dentigerous (follicular) cyst

Case KR11

- 59-year-old female
- Dental hygiene / status: normal hygiene; partly missing, partly replaced teeth; bone hard swelling and tenderness of vestibular mucosa at the site of tooth 38.
- Radiology: 33x21 mm well demarcated radiolucency around tooth 38 deplacing the cortical bone; further cysts periapically at tooth 32, at the place of tooth 23 (residual?) and periapically at tooth 21.
- Operation: removal of the cyst and tooth 38 together
- Clinical diagnosis: dentigerous (follicular) cyst

KR11 – OPT

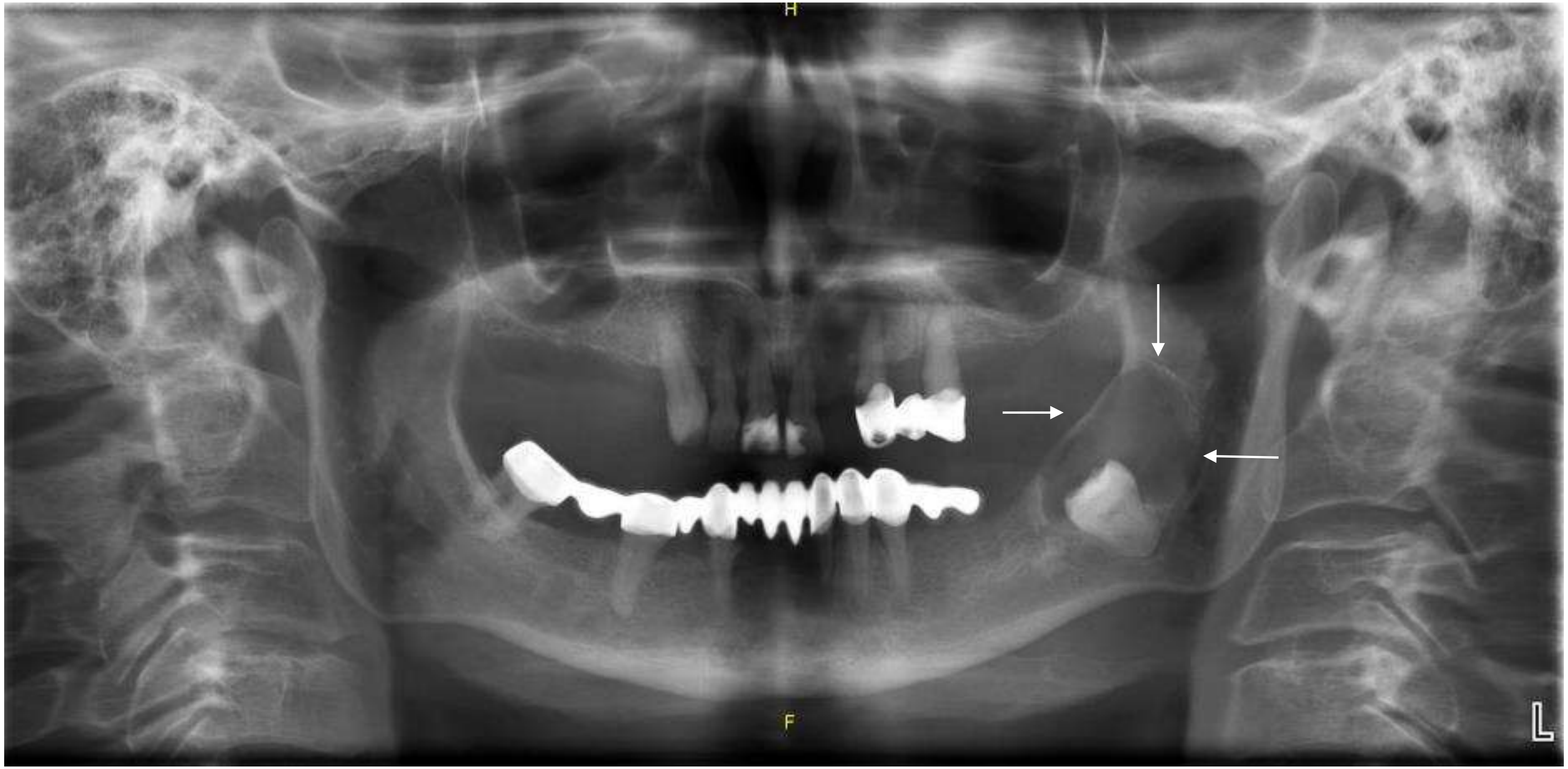

KR11 – CT axial

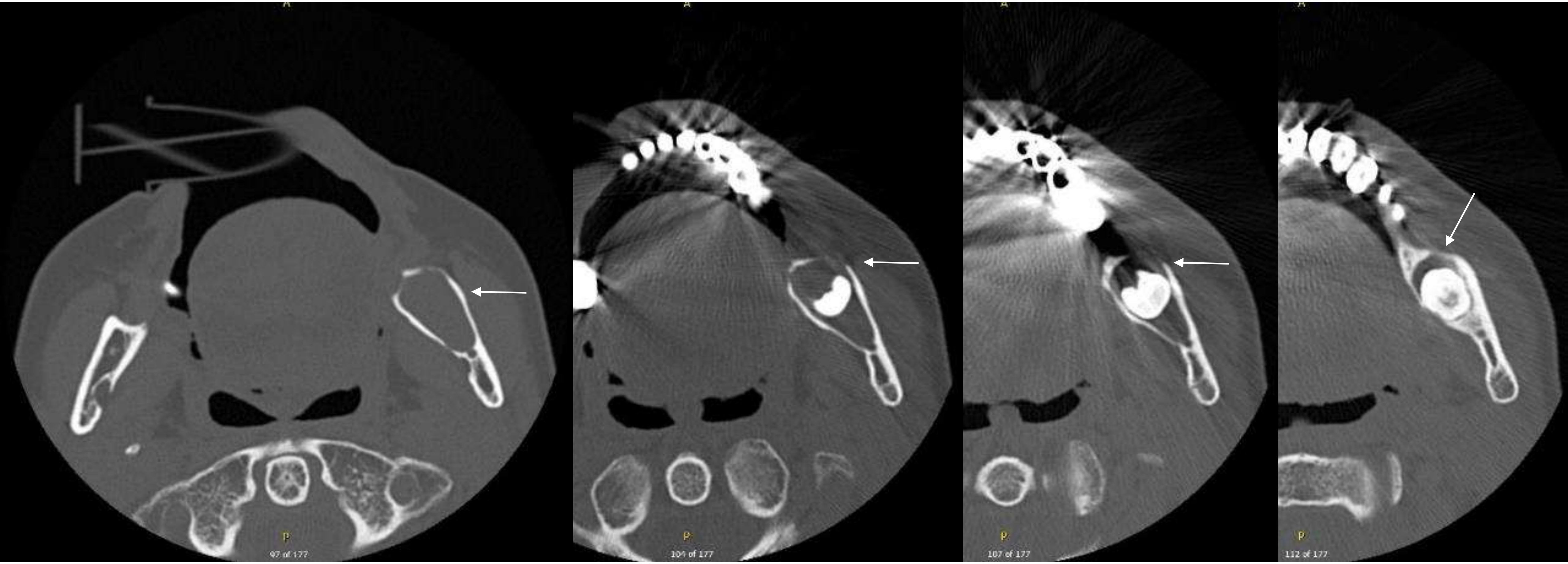

KR11 – CT coronal

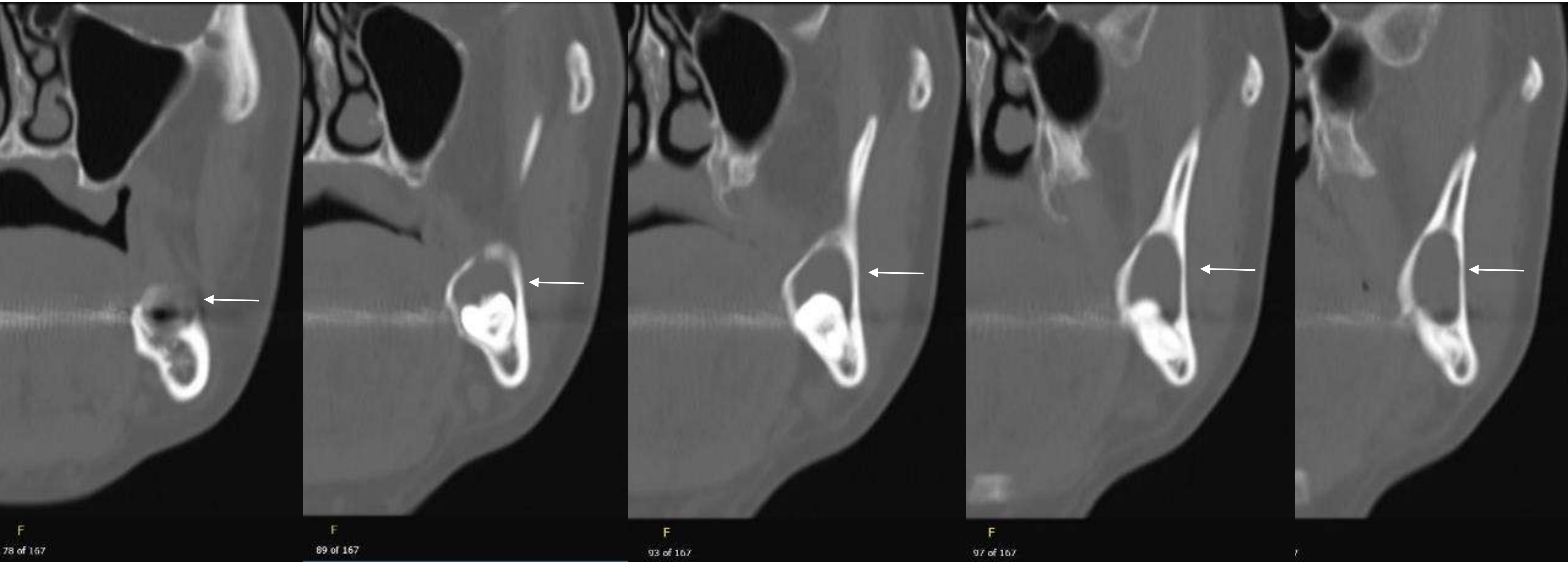

KR11 – CT sagittal

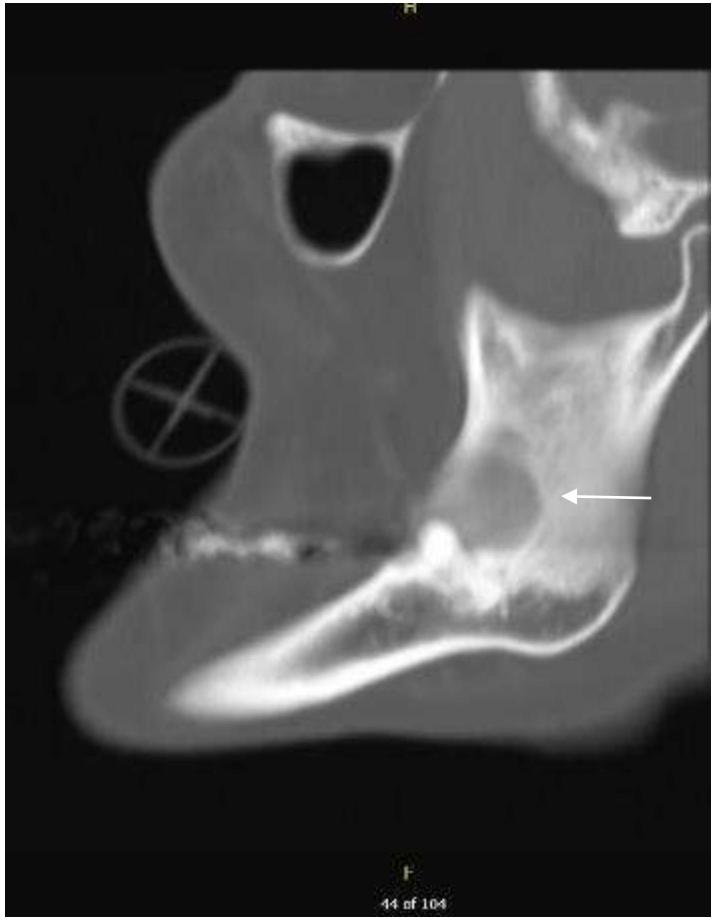

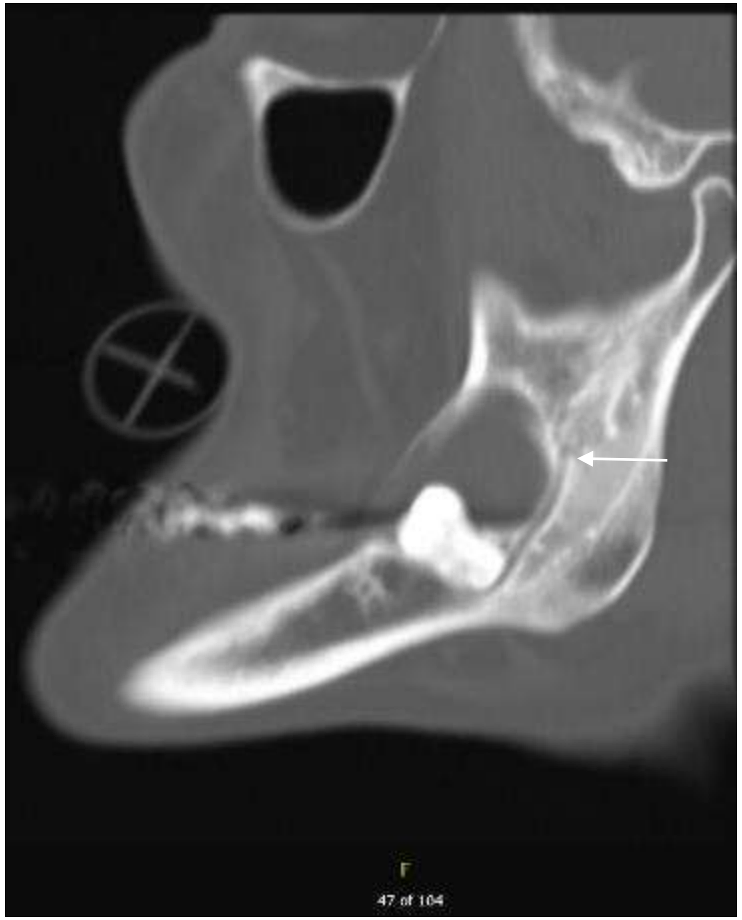

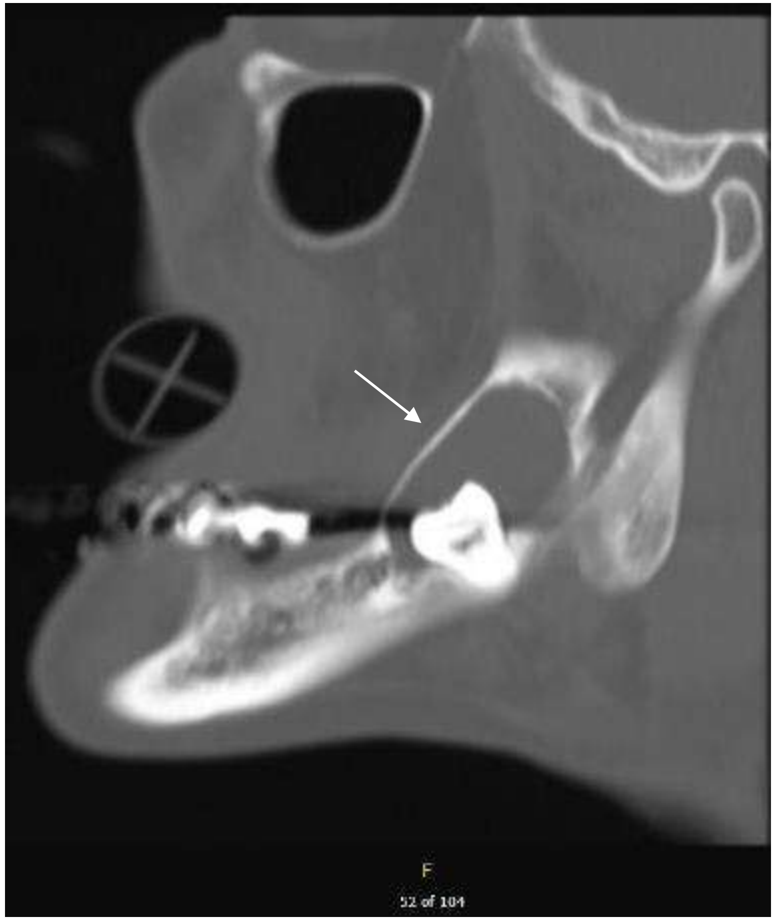

KR11 – HE, CK17, bcl2 (x1)

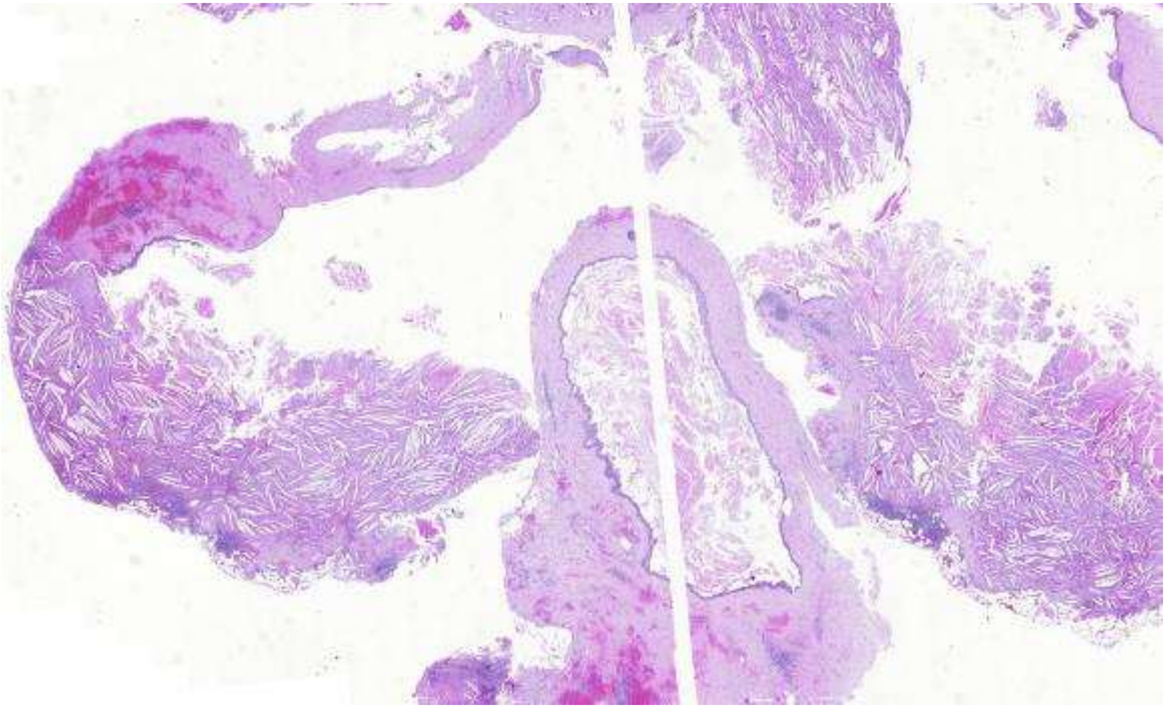

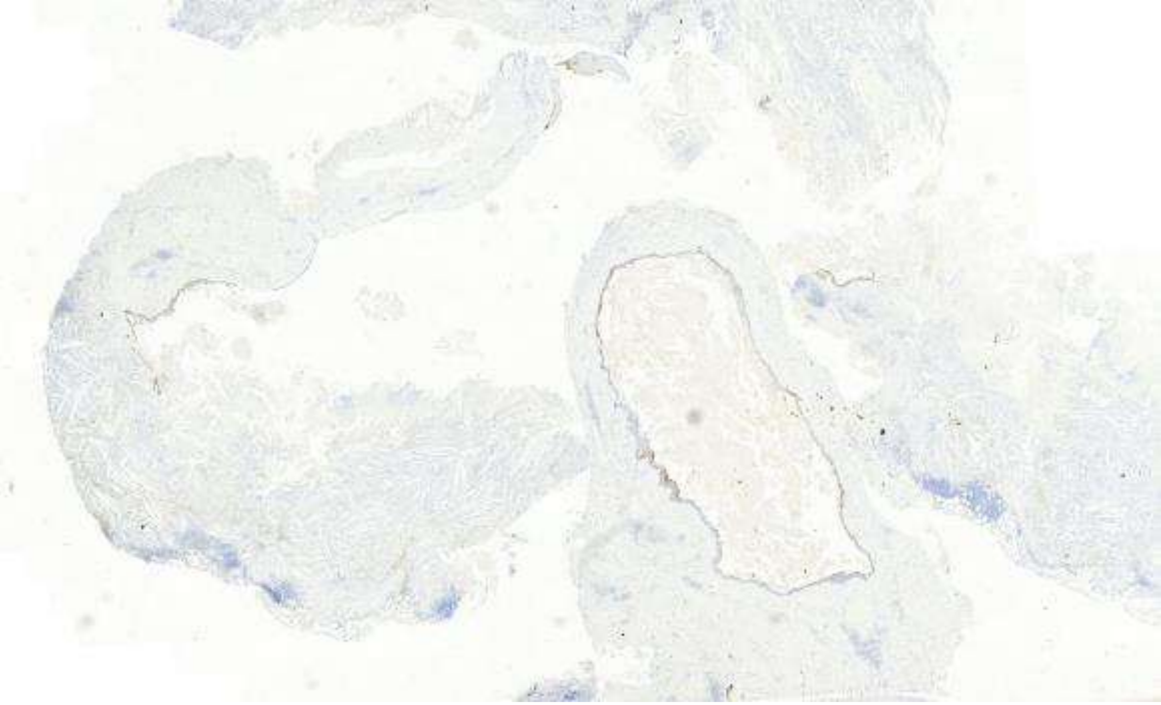

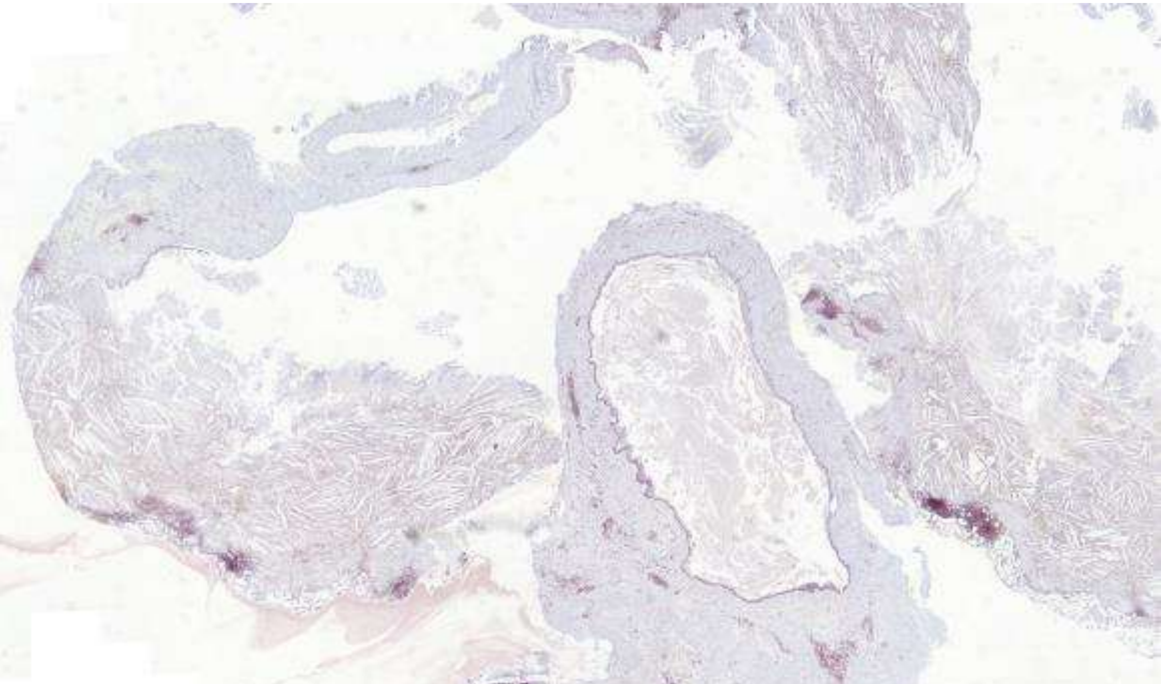

KR11 – HE, CK17, bcl2 (x15)

Basal/suprabasal (↑) to transepithelial CK17 staining,
bcl2- negativity

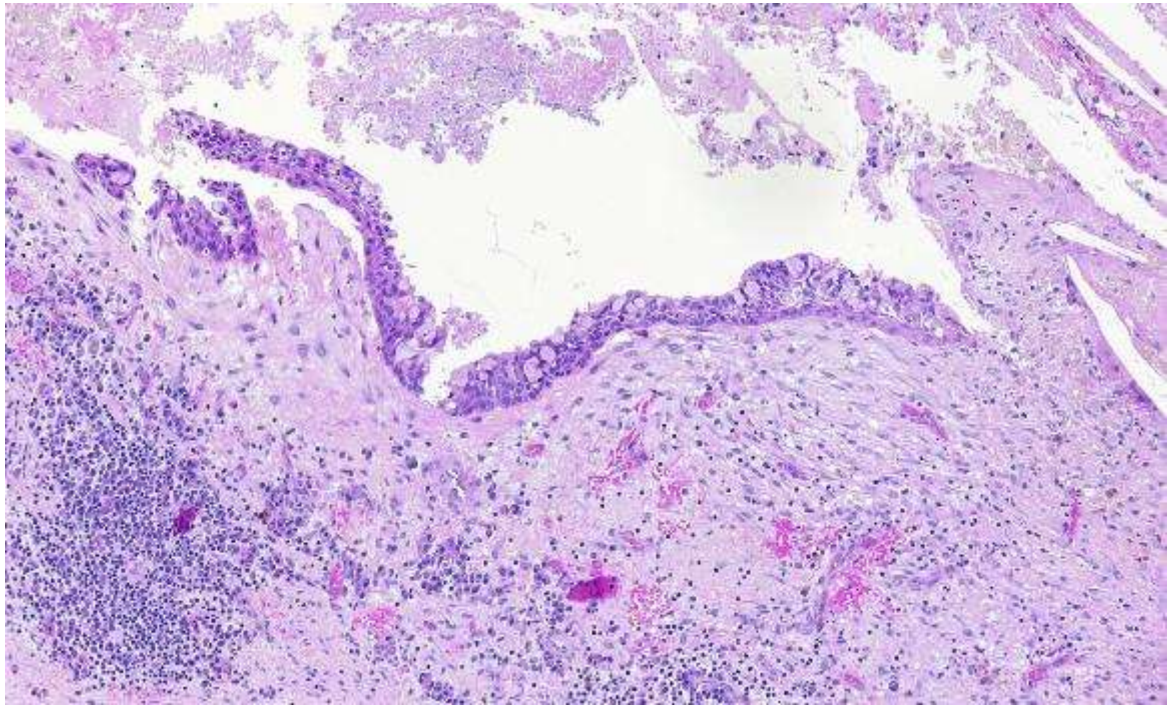

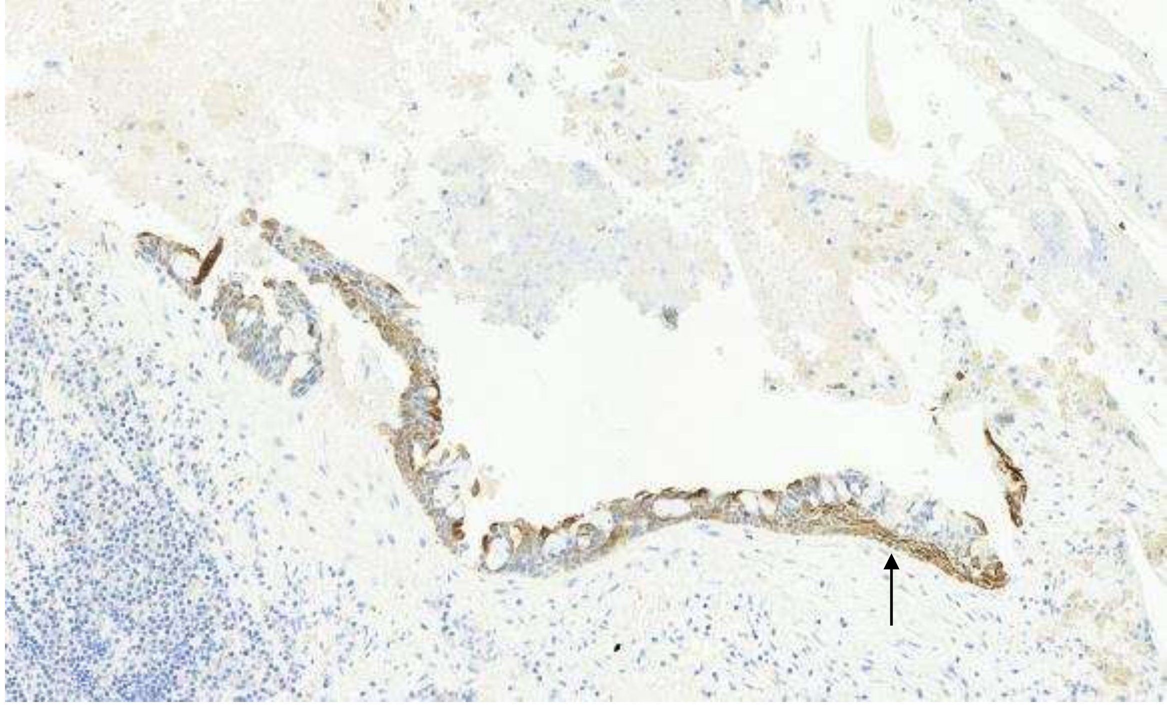

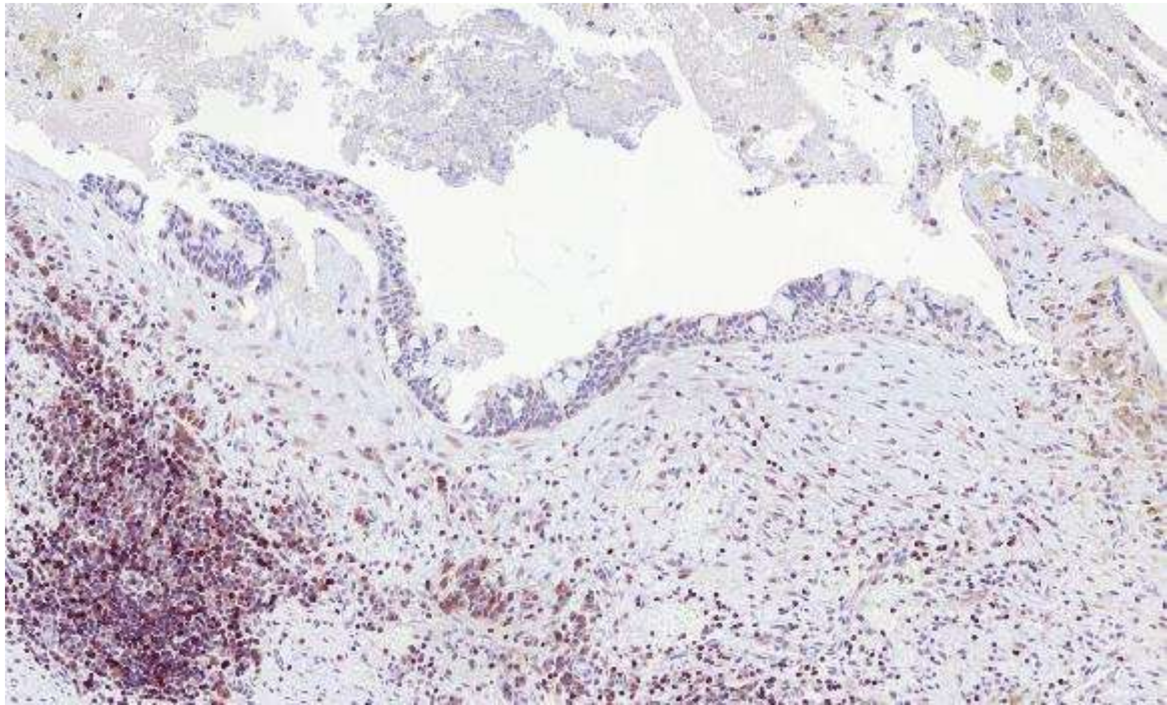

KR11 – HE, CK17, bcl2 (x35)

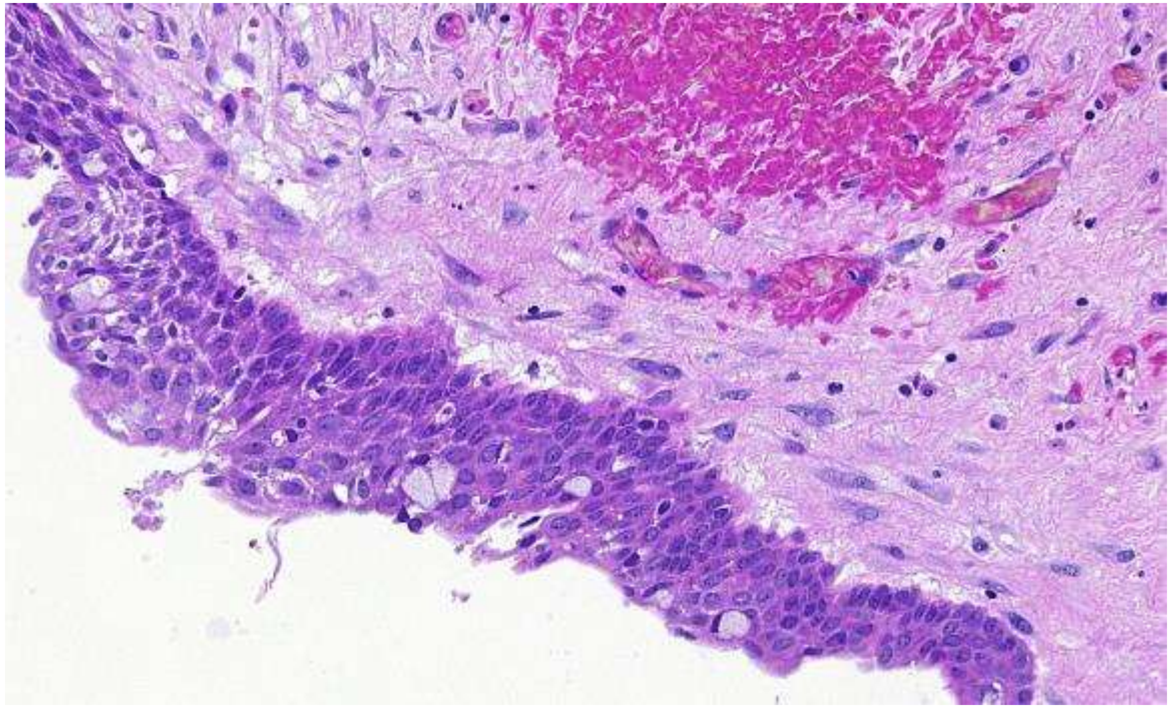

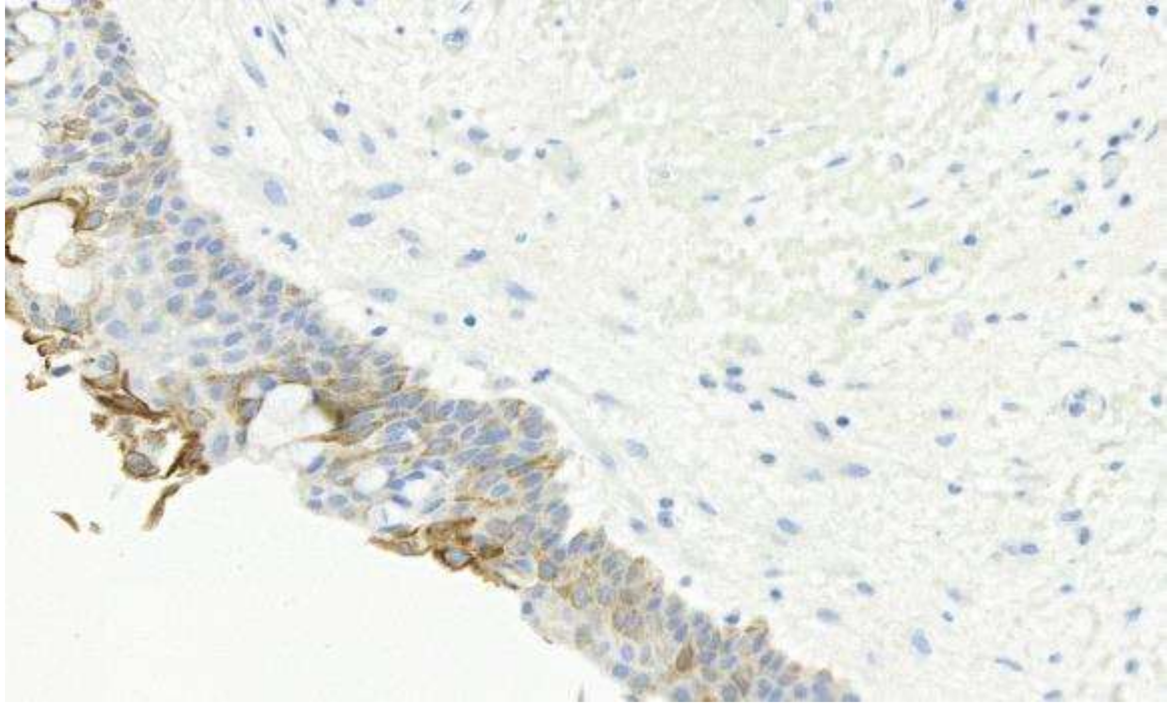

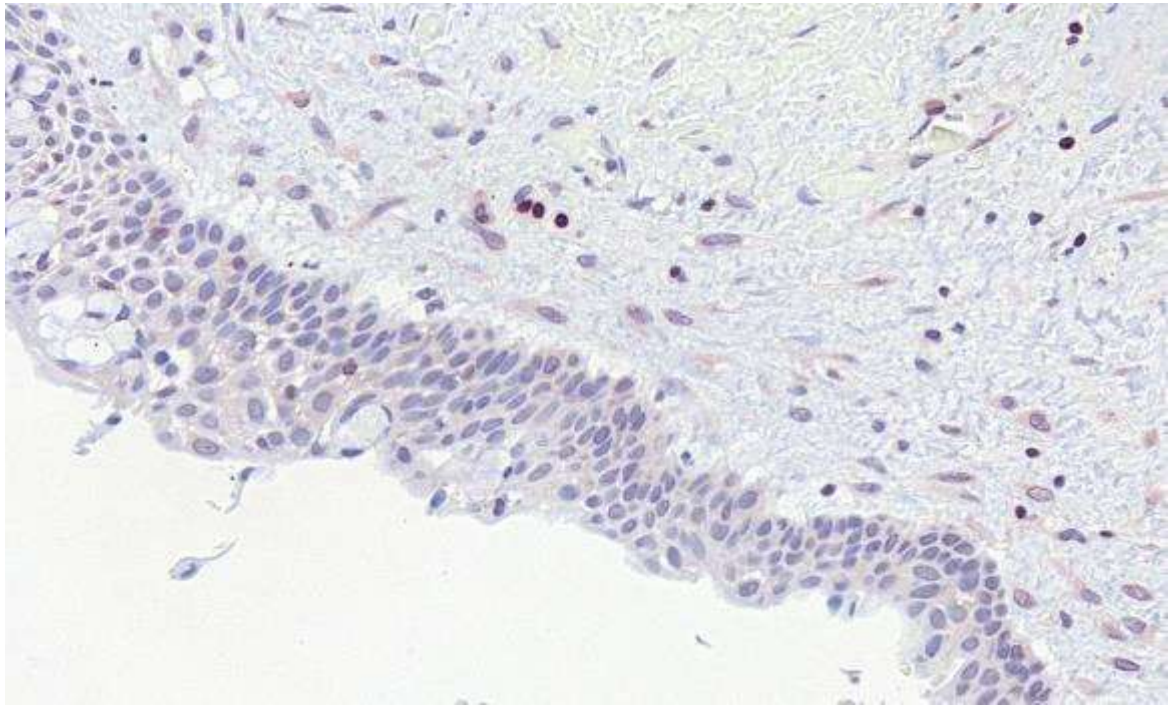

KR11 – HE, CK17, bcl2 (x40)

Focal CK17 negativity

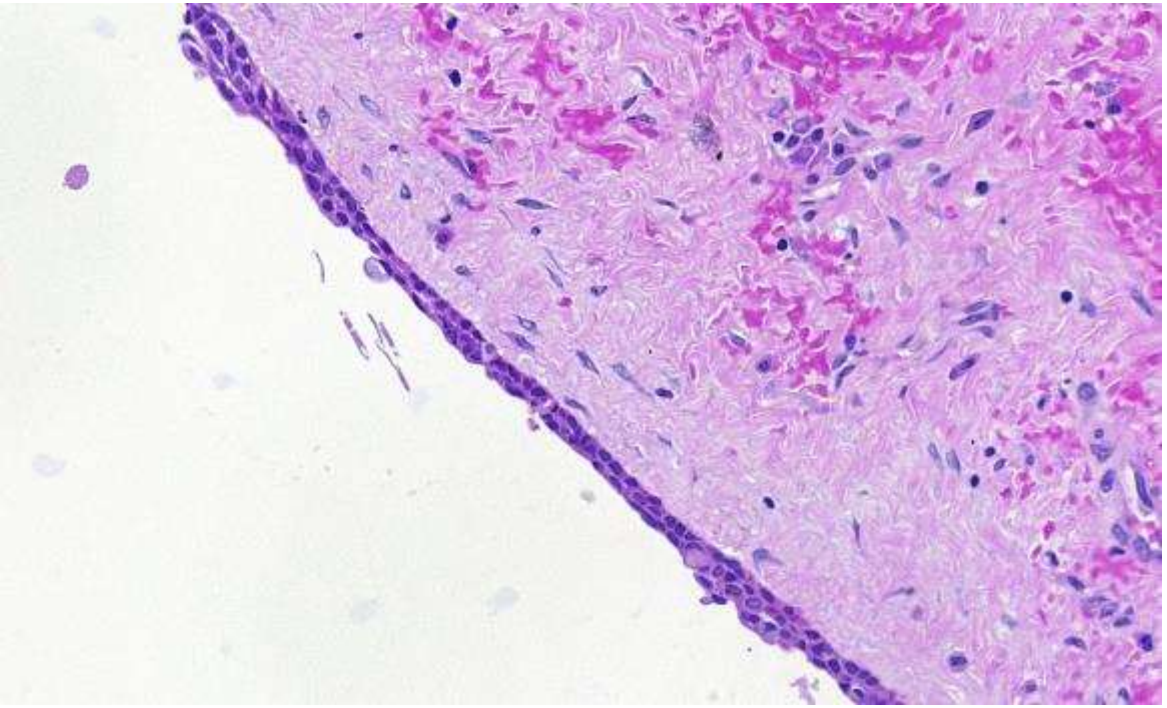

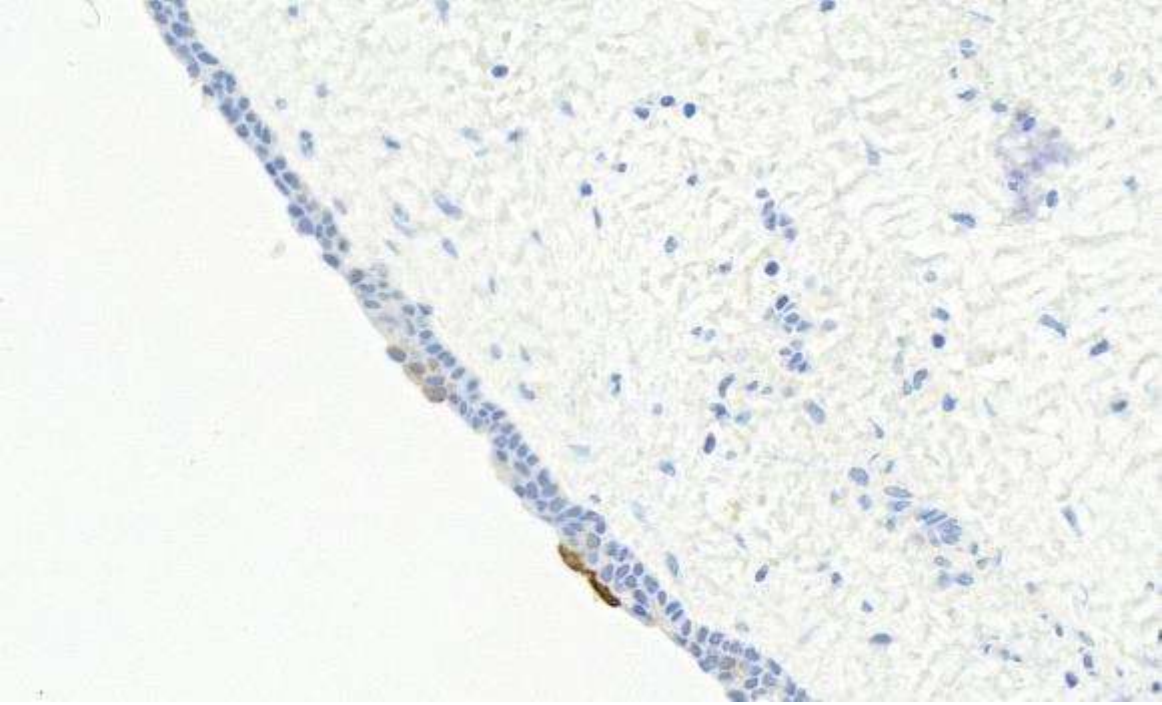

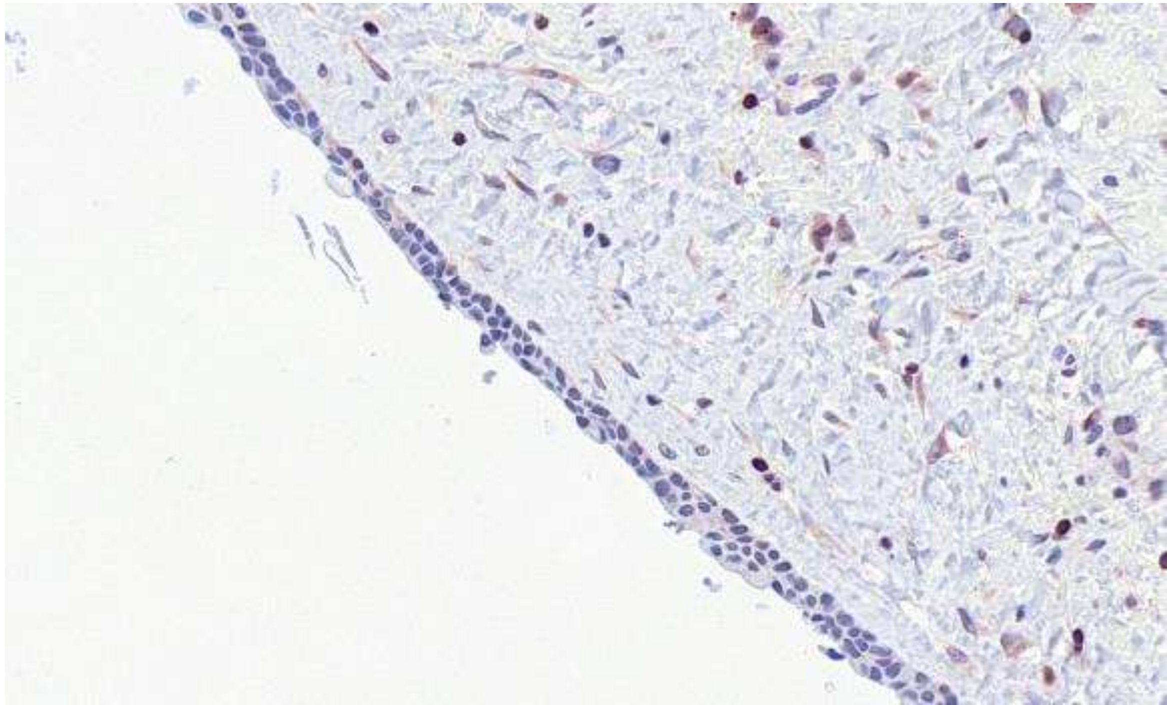

KR11 – Conclusion

- H&E: partly thick (squamous or with mucinous superficial layer), partly thin epithelium (with mucinous superficial layer), no parakeratosis, no wavy surface, no basal palisading, even epithelial-subepithelial contours; mucous metaplasia
- Cholesterol granuloma related focal chronic inflammation of the cyst wall
- Bcl2: negative / basal staining (focally at areas of mucous metaplasia / epithelium)
- CK17: focally negative / focal basal – suprabasal /or superficial /or transepithelial staining
- Histopathology: inflammatory / inflamed odontogenic cyst with squamous and mucous epithelium
- Combined clinical and pathological opinion: dentigerous (follicular) cyst with prominent mucous metaplasia

Case KR12

- 59-year-old male
- Dental hygiene/status: poor hygiene; partly conserved, partly missing, untreated teeth with numerous crowns missing and roots in the alveoli; fluctuating vestibular swelling corresponding to the place of teeth 11-15.
- Radiology: CT: only tooth 17 identified in the relevant quadrant; near tooth 17, there is an air-bubble containing, soft tissue density lesion protruding into the maxillary sinus (with bone destruction, soft tissue swelling), but separated from it by a sclerotic margin. Periostitis. Cervical lymphadenopathy.
- Operation: 1. cystostomy (incision and drainage); 2. cystectomy
- Clinical diagnosis: maxillary cyst

KR12 – OPT

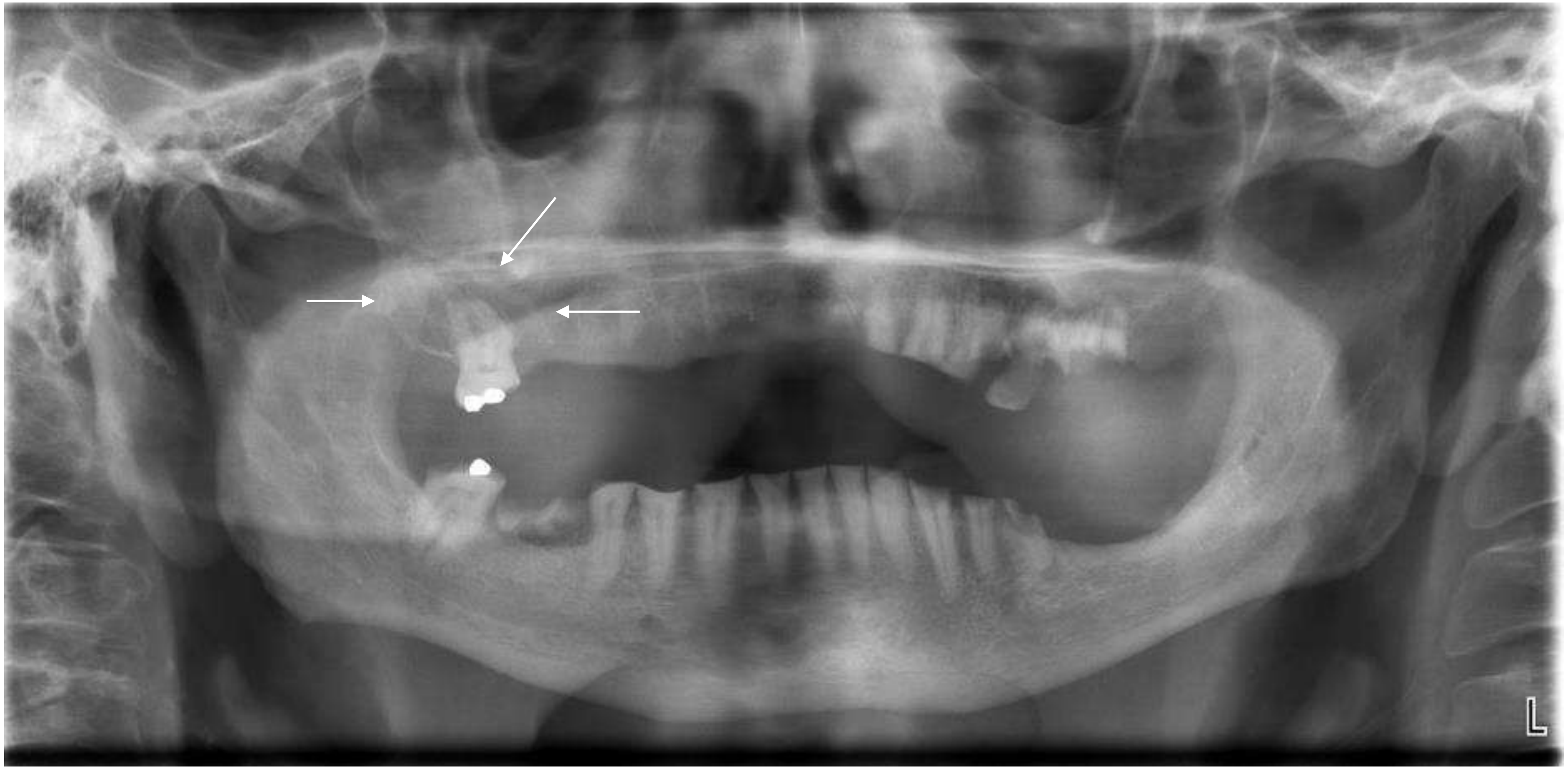

KR12 – OPT negative

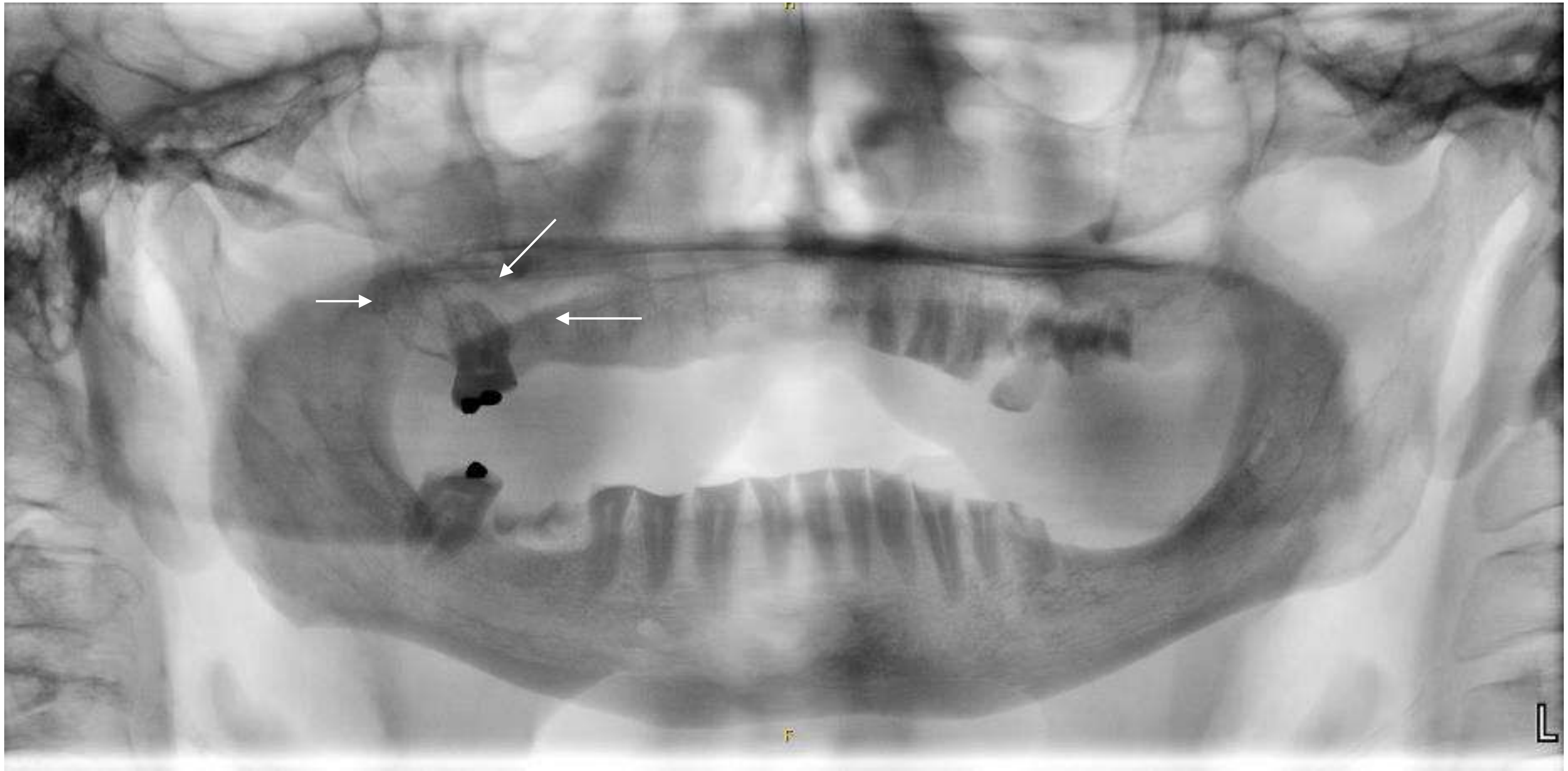

KR12 – CT axial (76, 81 and 84 / 135 slices)

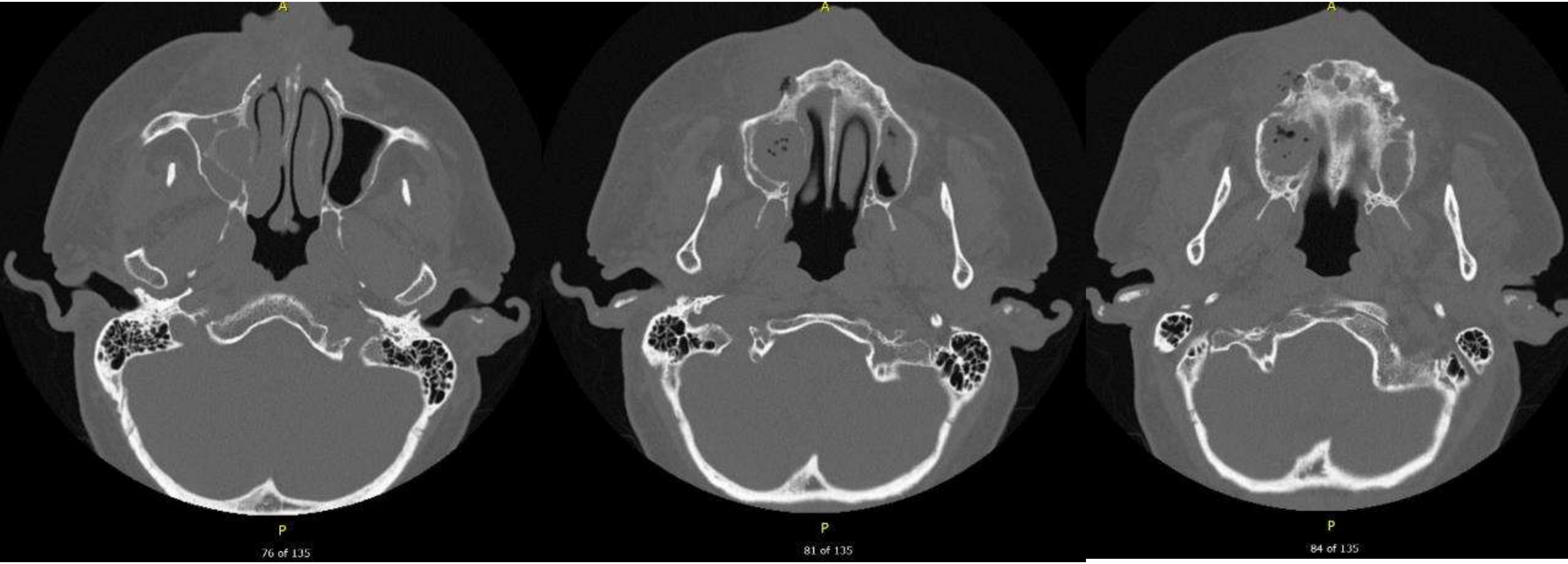

KR12 – CT coronal (15-18 / 67 slices)

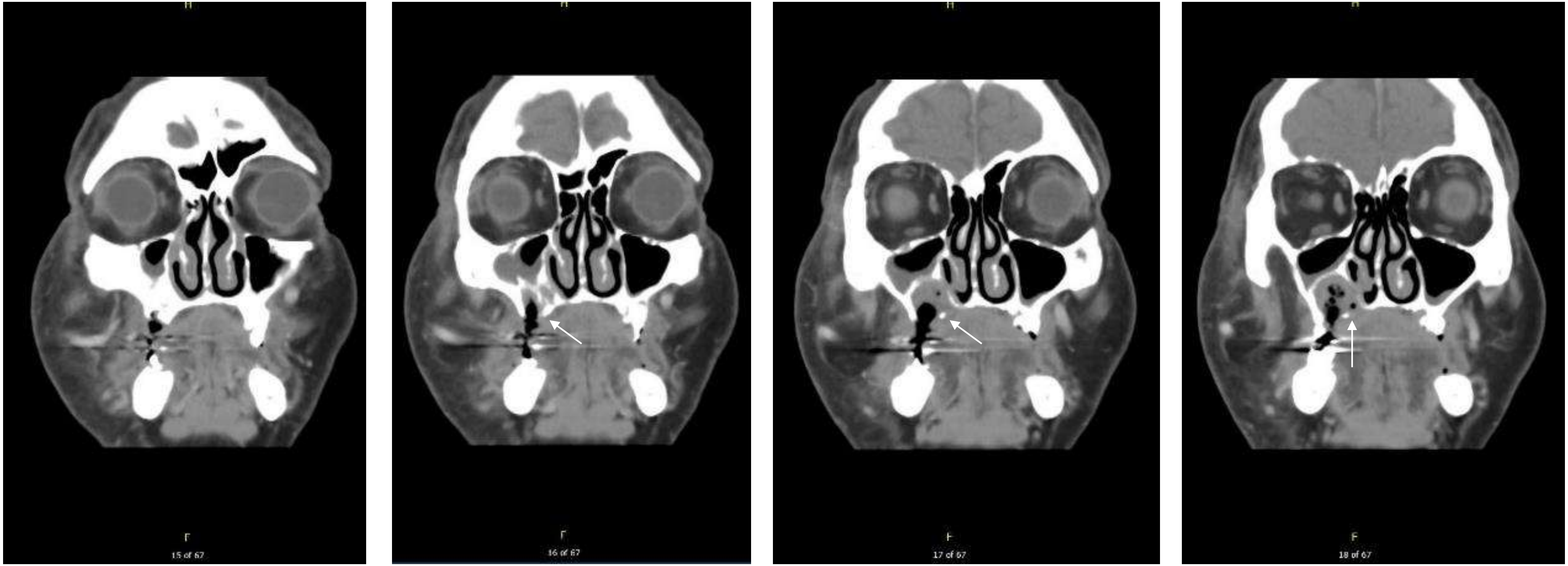

KR12 – CT sagittal (35 & 36 / 60 slices)

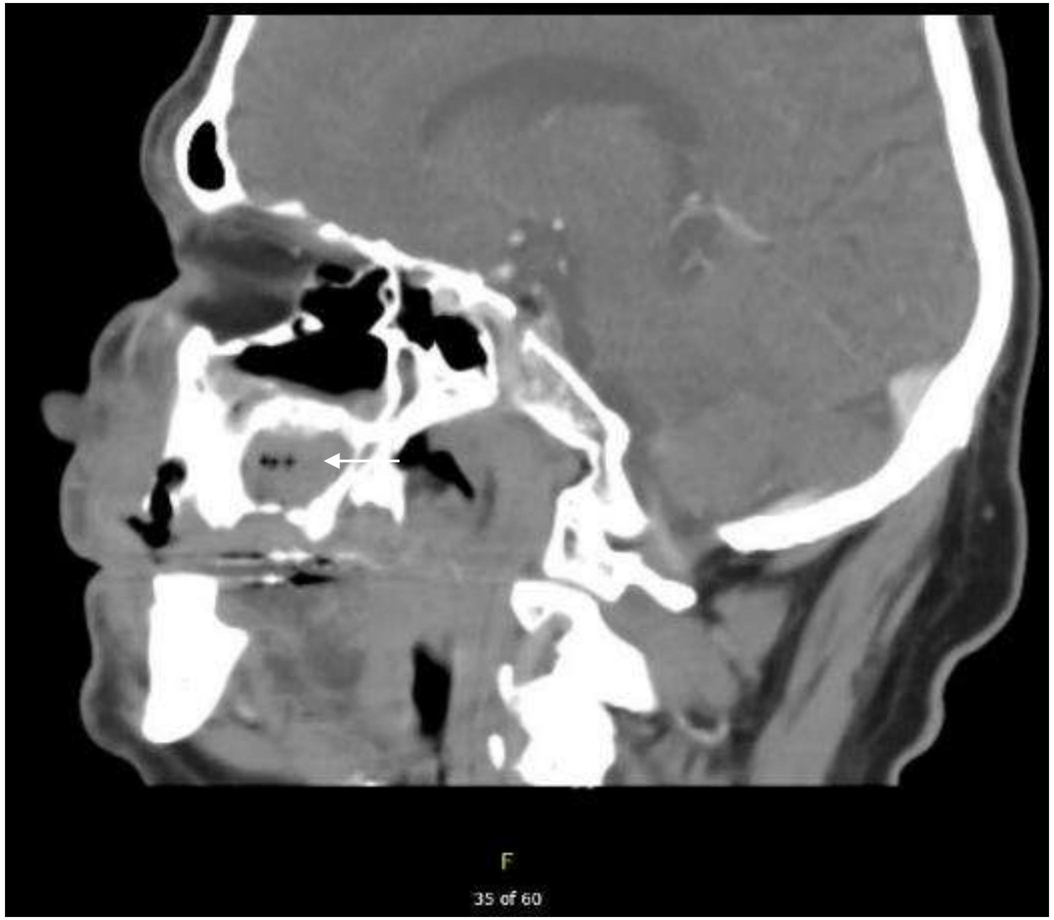

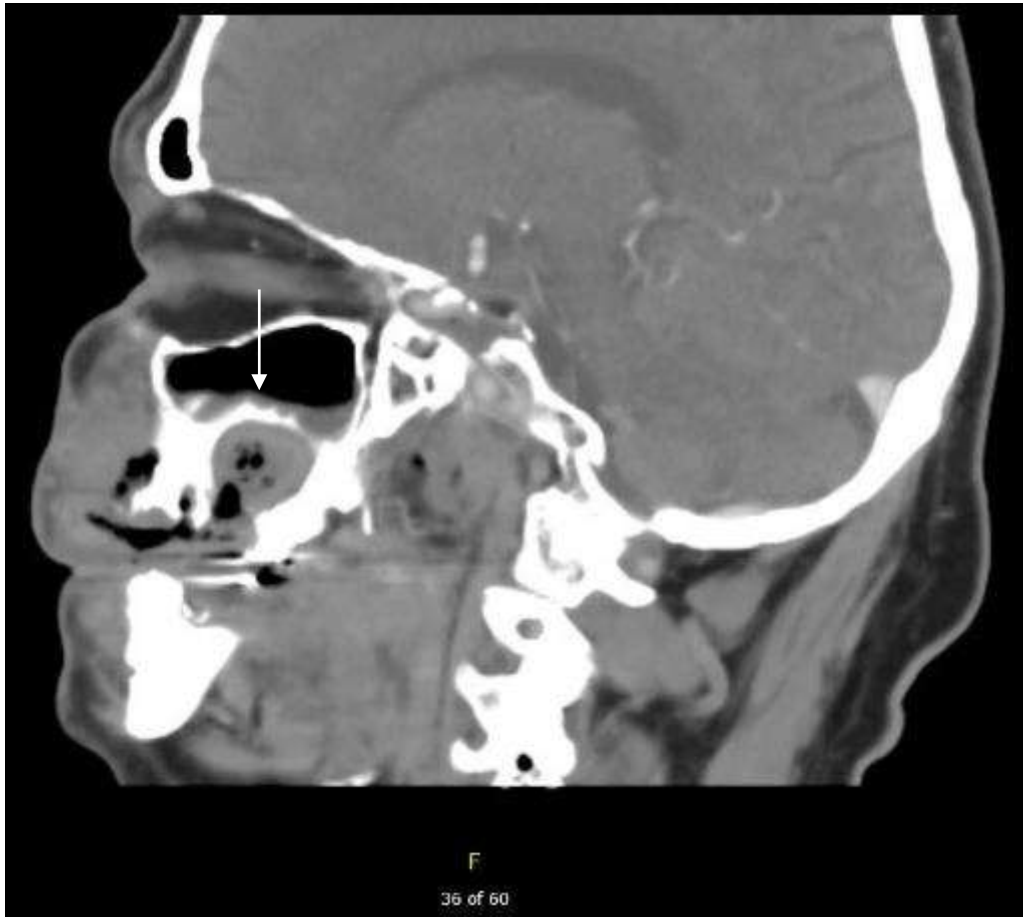

KR12 – CT sagittal (38 & 39 / 60 slices)

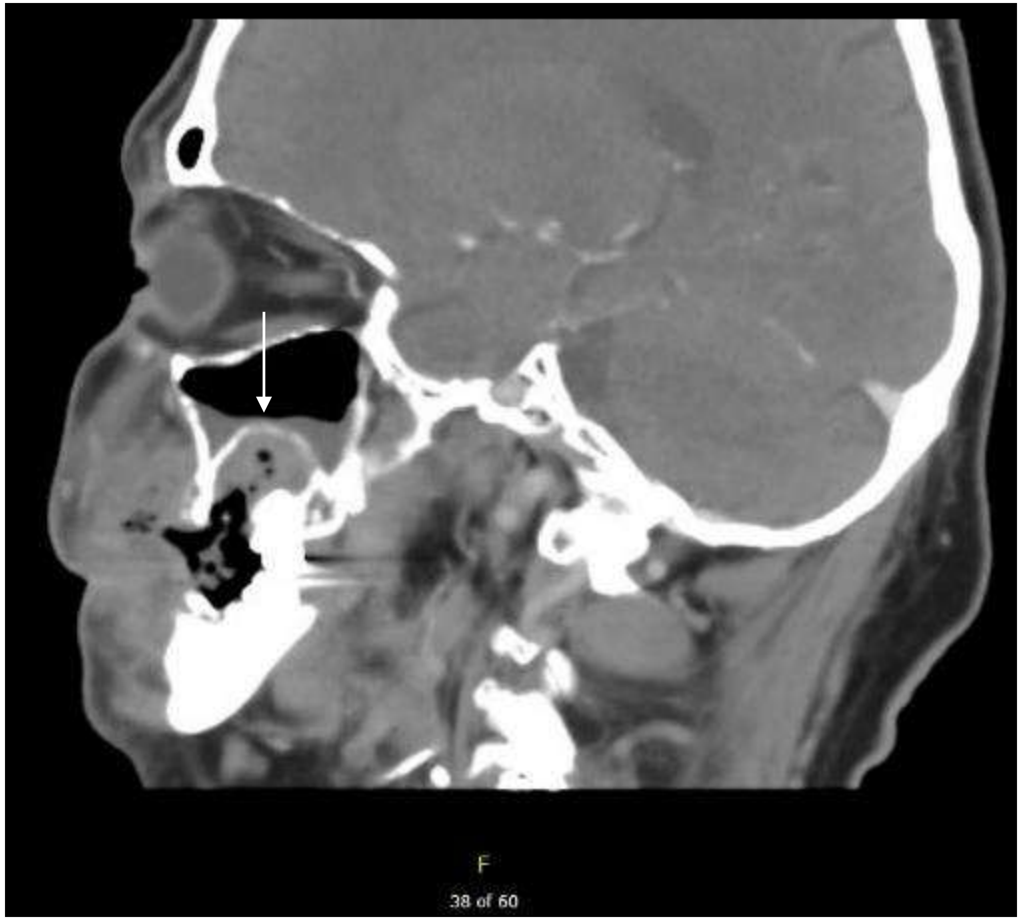

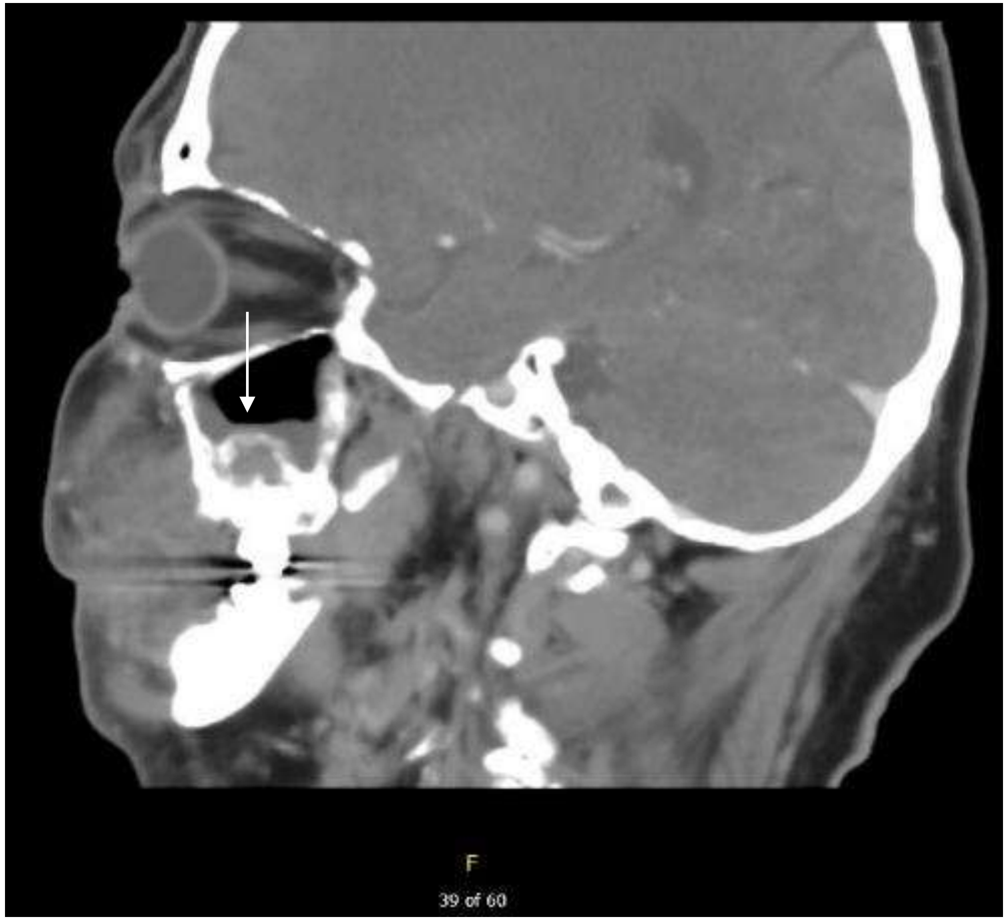

KR12 – HE, CK17, bcl2 (x1)

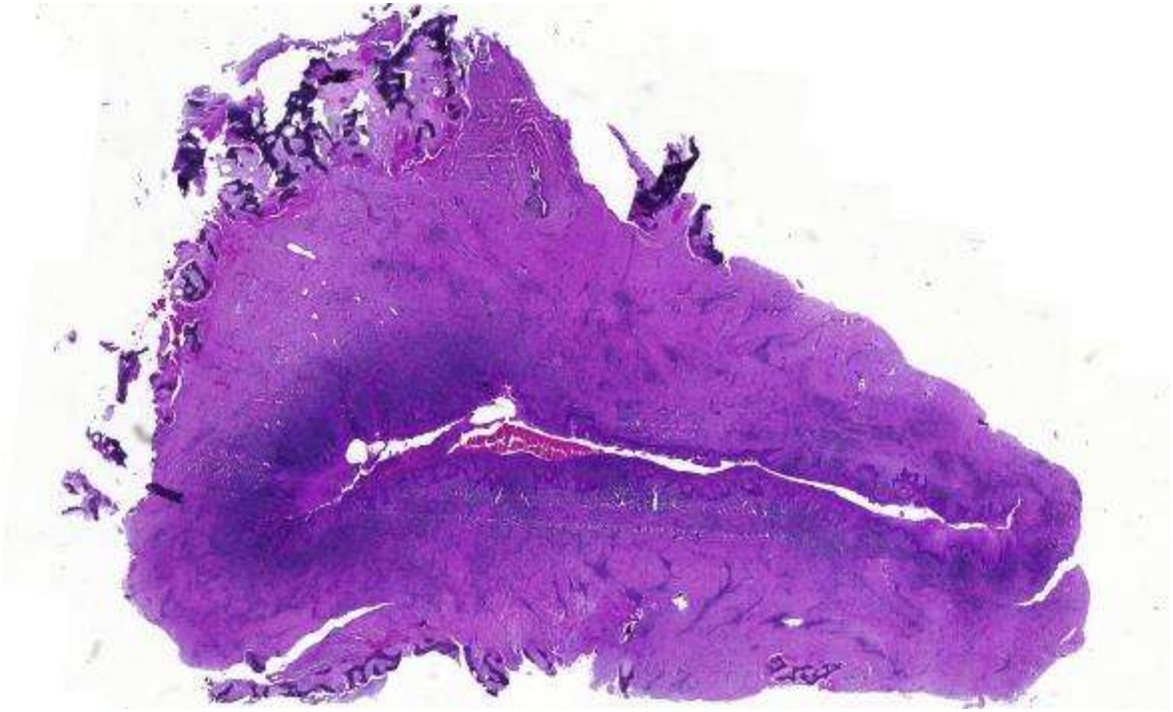

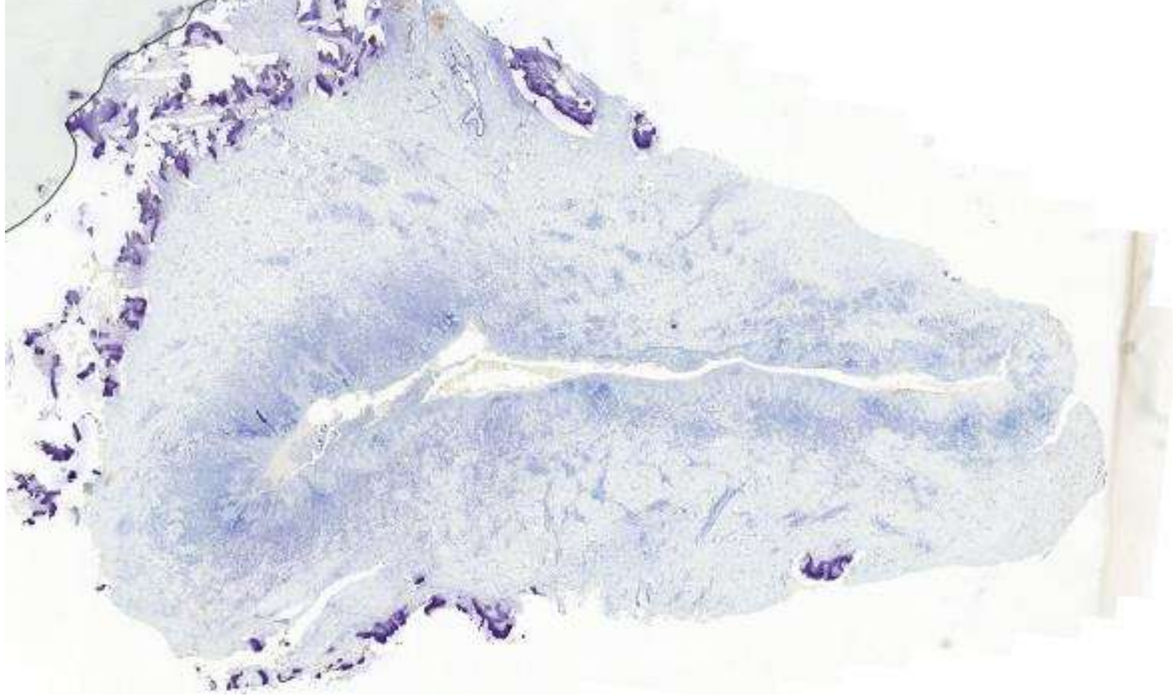

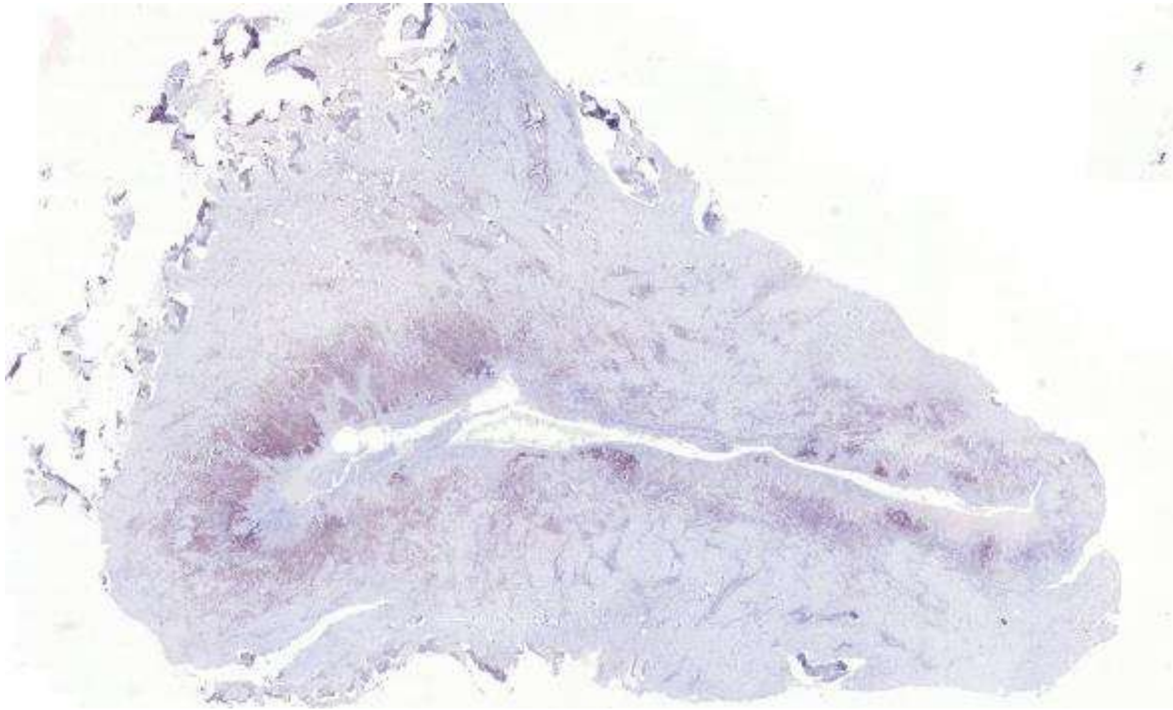

KR12 – HE, CK17, bcl2 (x5)

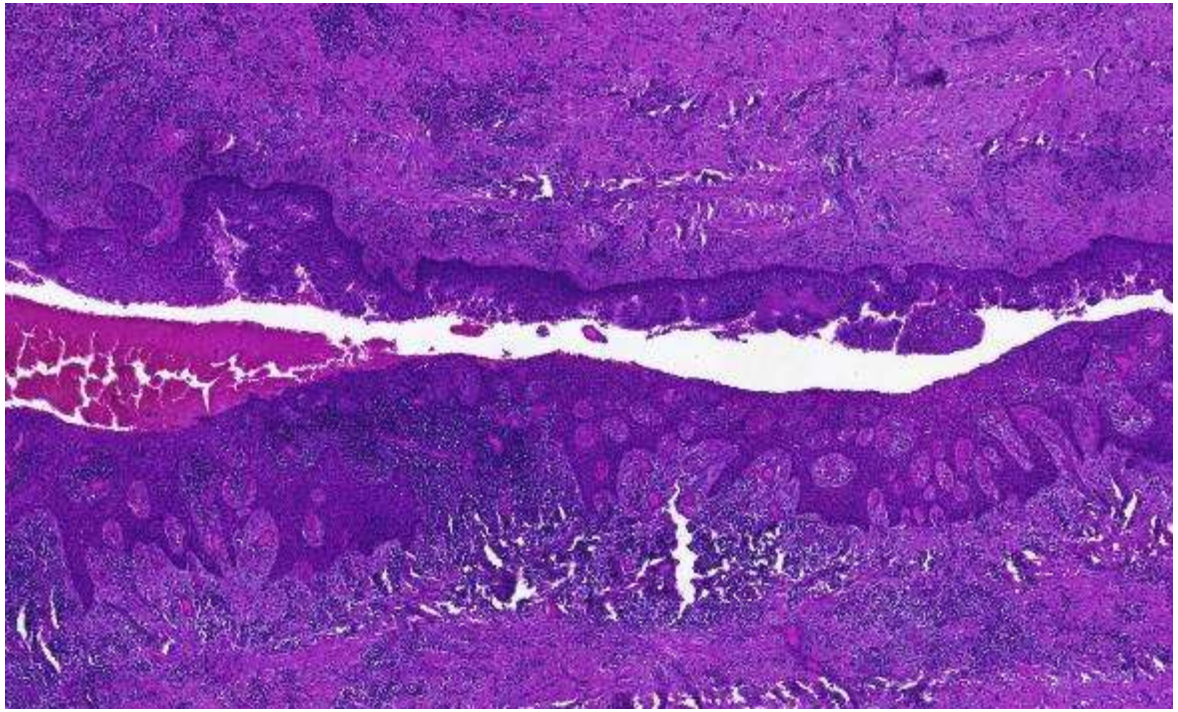

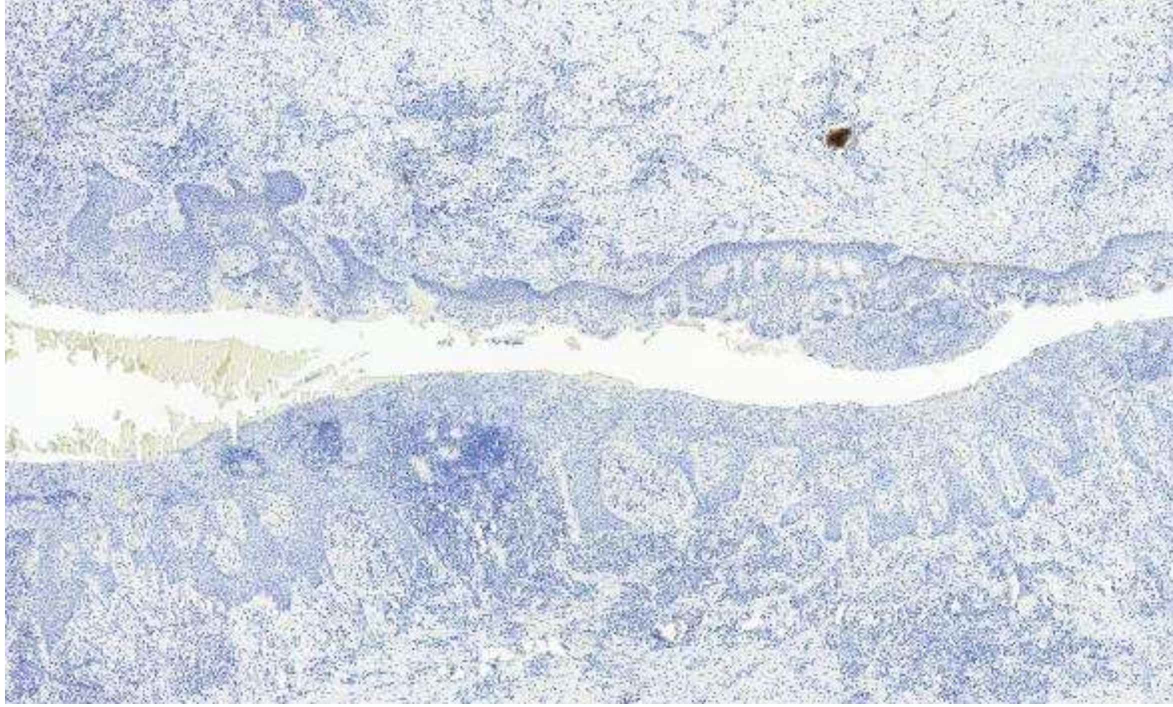

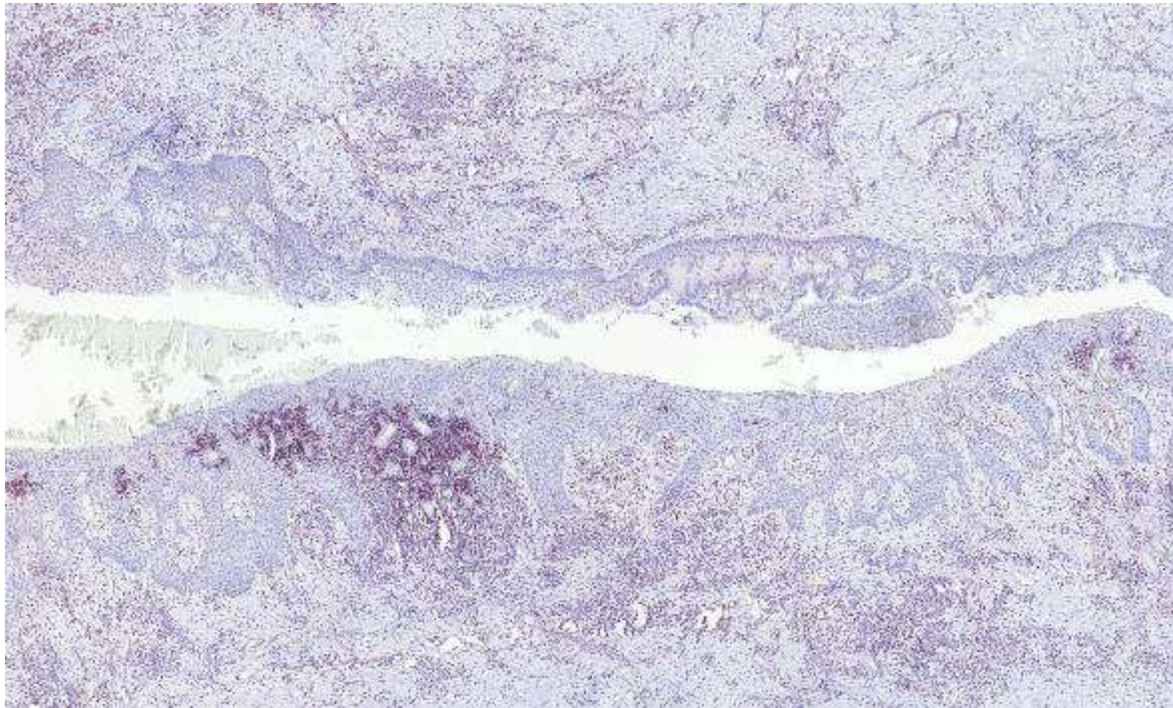

KR12 – HE, CK17, bcl2 (x10)

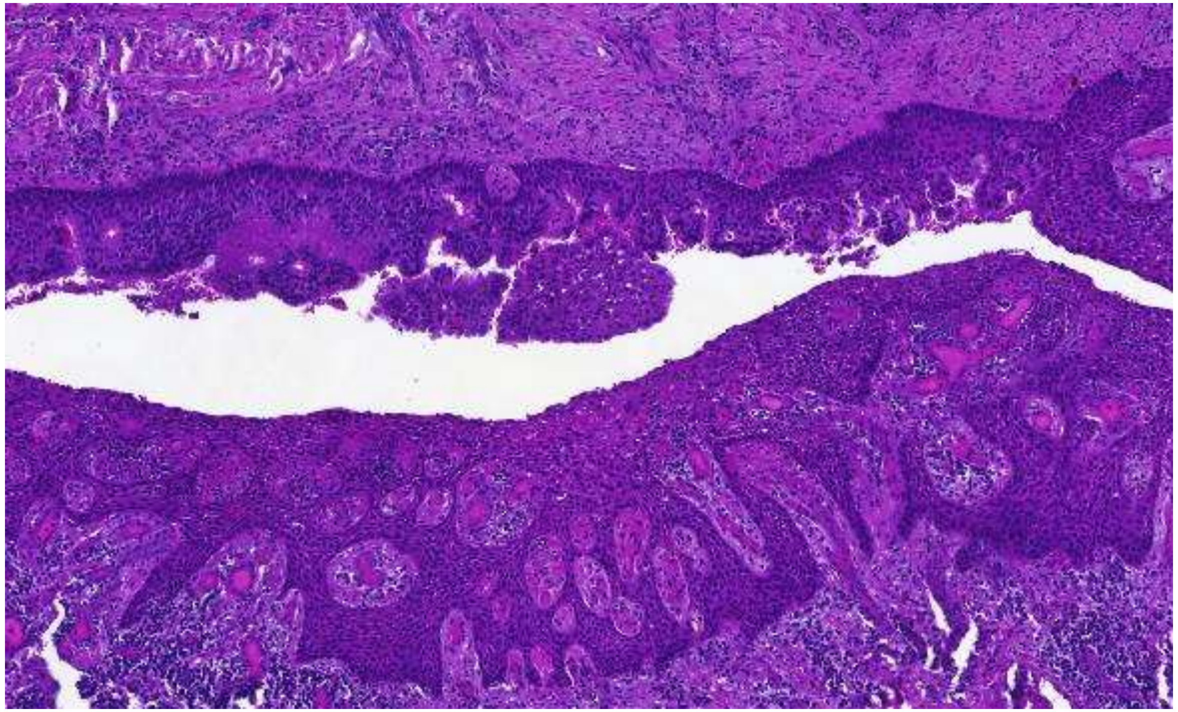

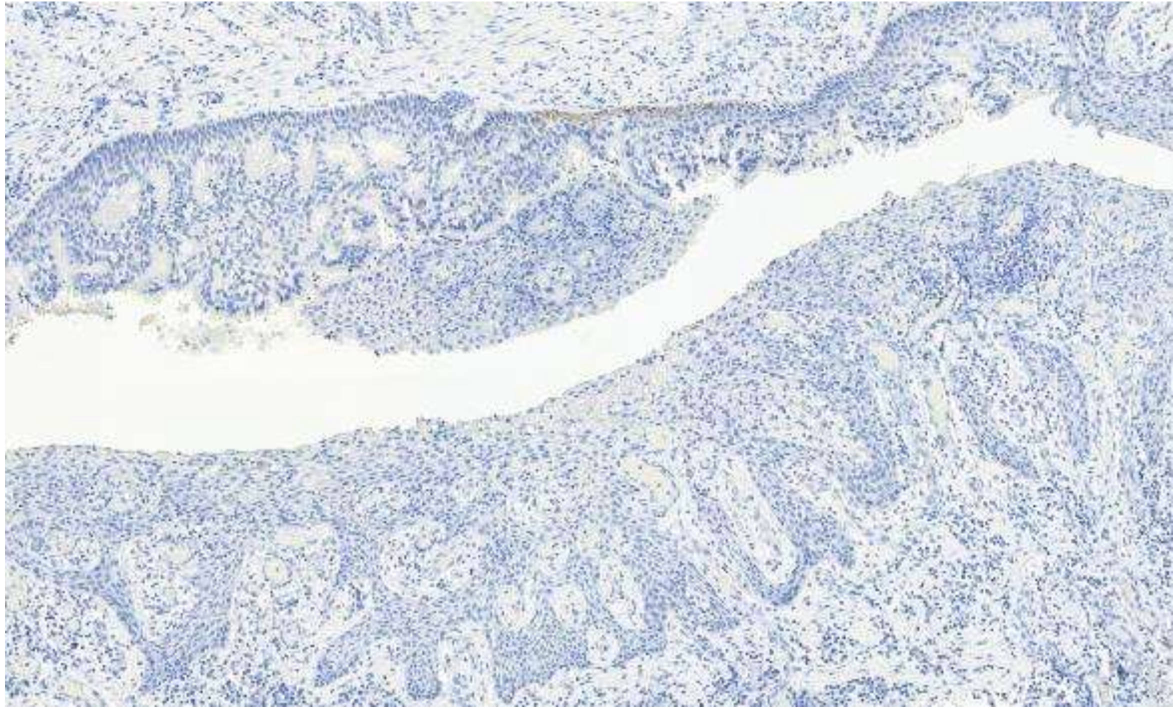

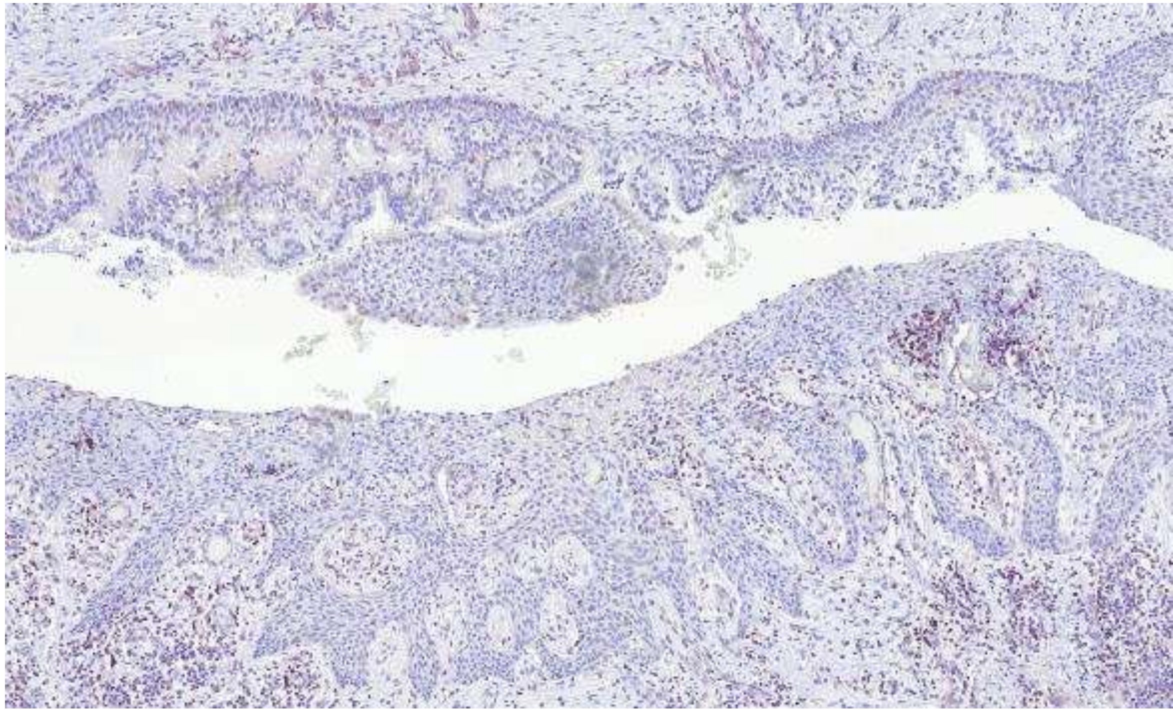

KR12 – HE, CK17, bcl2 (x1)

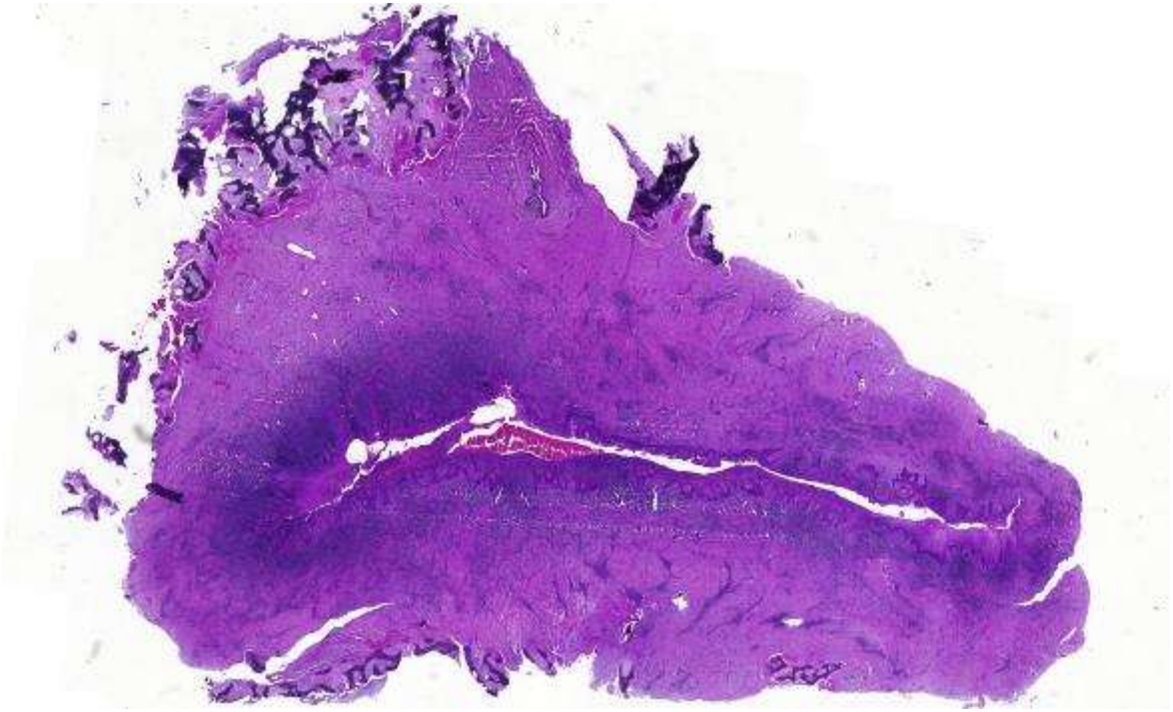

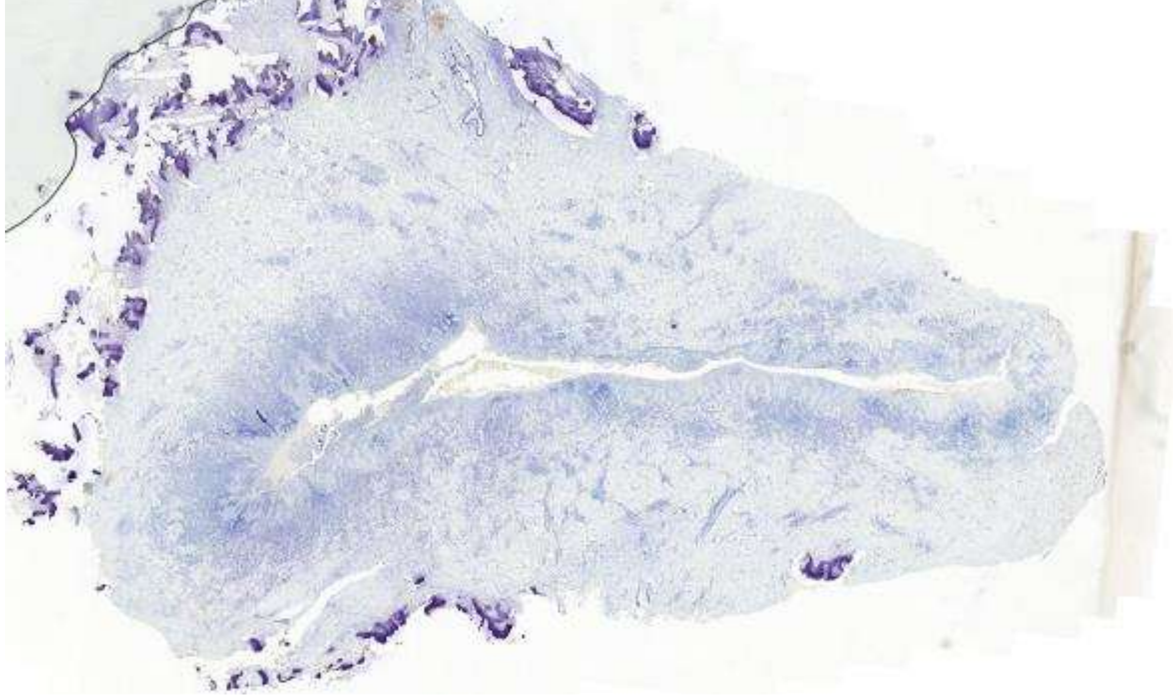

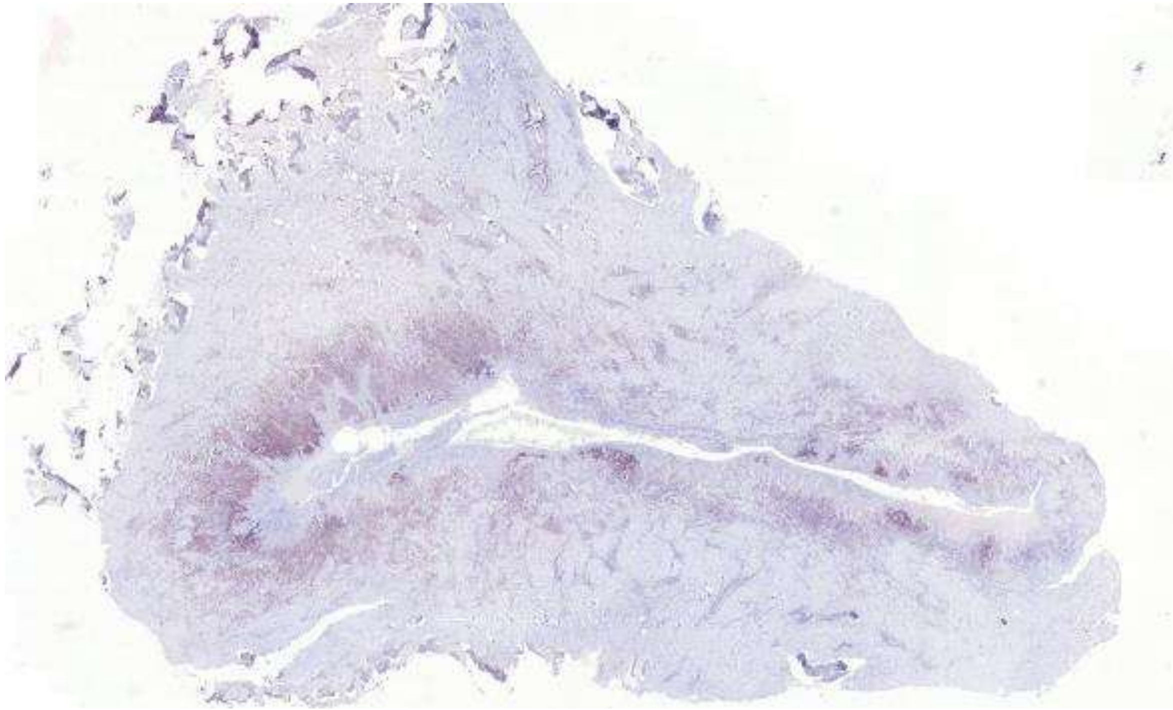

KR12 – Conclusion

- H&E: relatively thick squamous epithelium, no parakeratosis, no wavy surface, no basal palisading, uneven epithelial-subepithelial contours
- Focal mixed inflammation of the cyst wall with pus in the lumen
- Bcl2: negative
- CK17: dominantly negative, minimal focal superficial (with some upper spinous cells) staining
- Histopathology: inflammatory odontogenic cyst, radicular/residual cyst
- Combined clinical and pathological opinion: radicular cyst

Case KR13 (Same patient as KR19, but different cyst)

- 48-year-old male
- Dental hygiene/status: poor / partly conserved, partly missing teeth; tooth 41 removed recently; multiple teeth with caries including 42
- Radiology: circumscribed radiolucent lesion of 22 mm in size corresponding to teeth 31-42
- Operation: in to removal of the cyst, apical root resection of tooth 42 teeth with root filling
- Clinical diagnosis: „periapical cyst” (residual cyst mentioned in the charts before)

KR13 – OPT

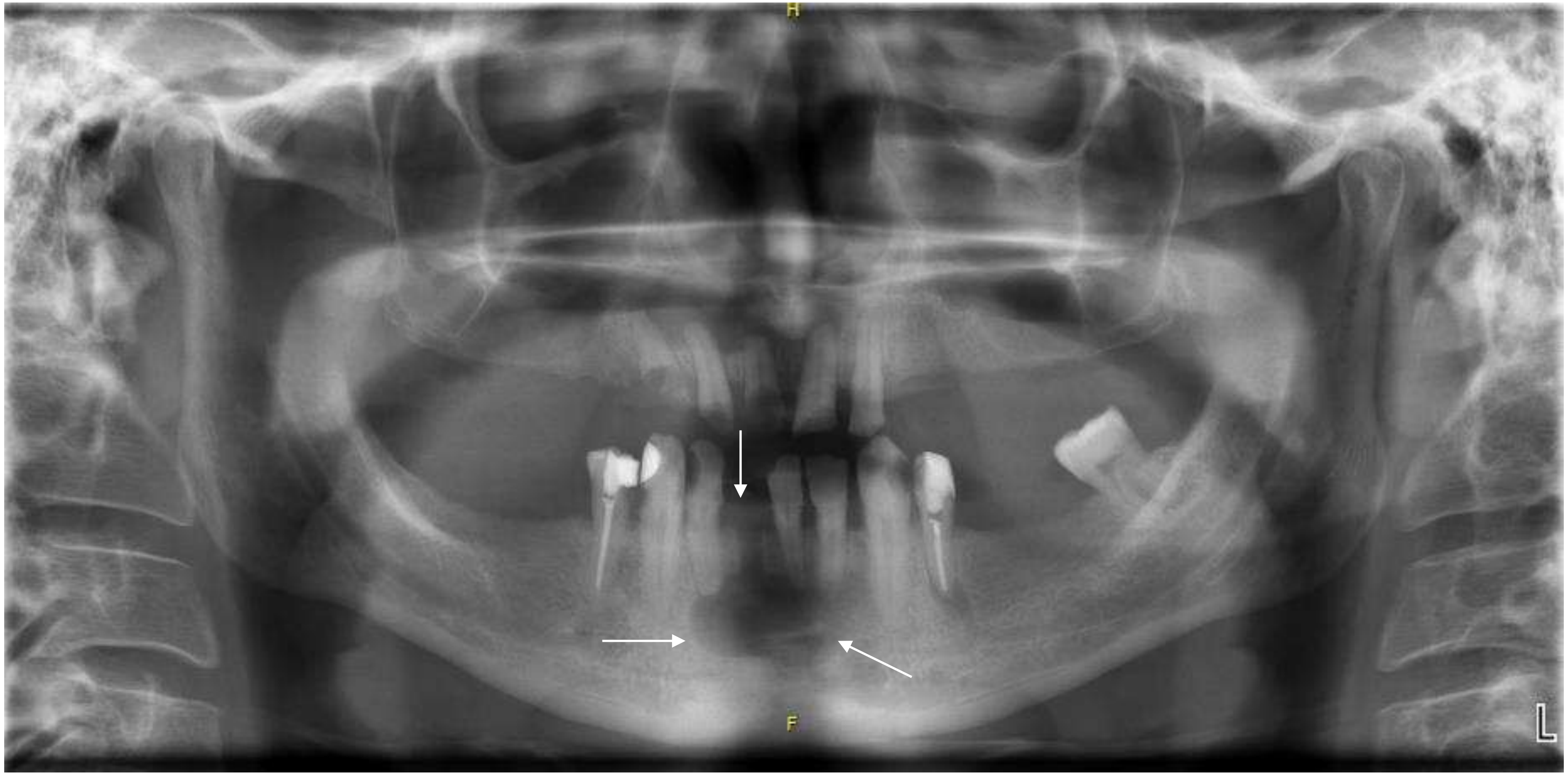

KR13 – HE, CK17, bcl2 (x1.3)

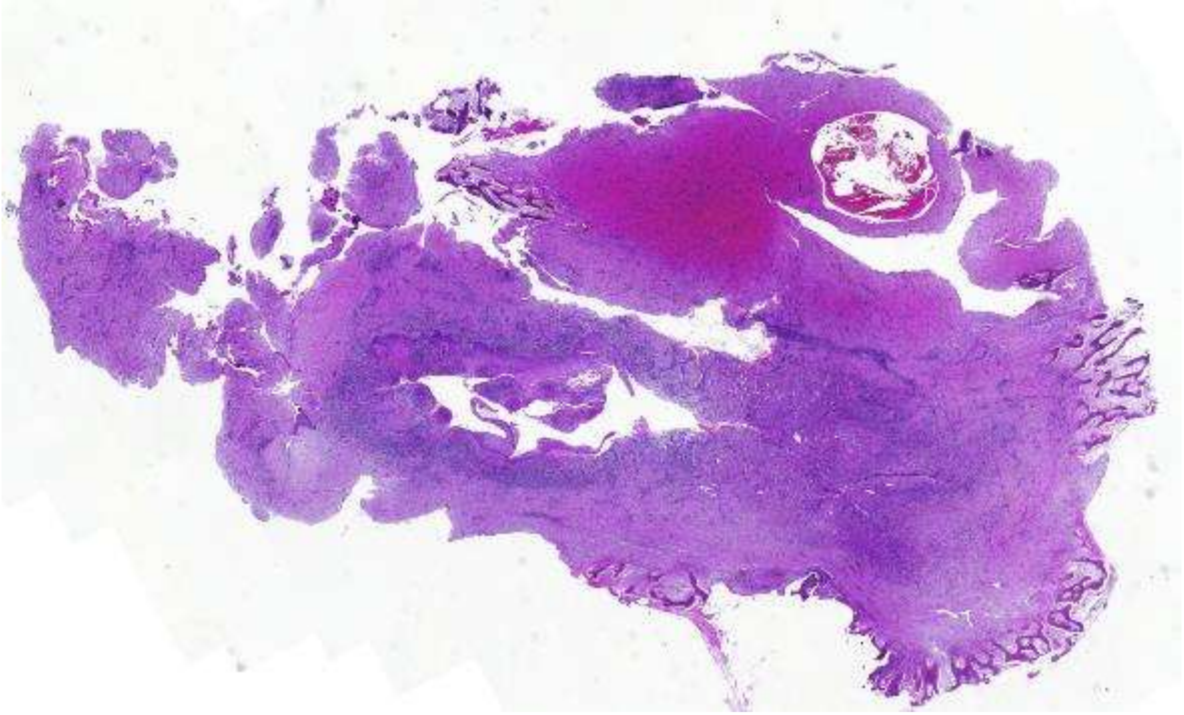

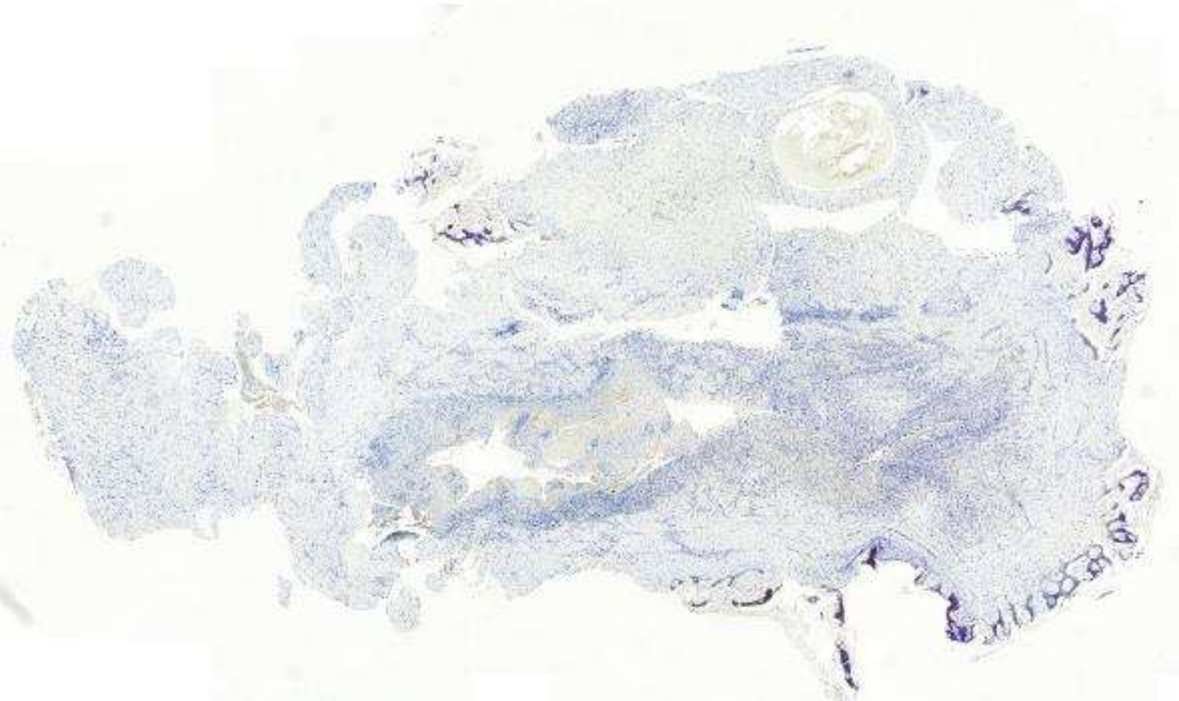

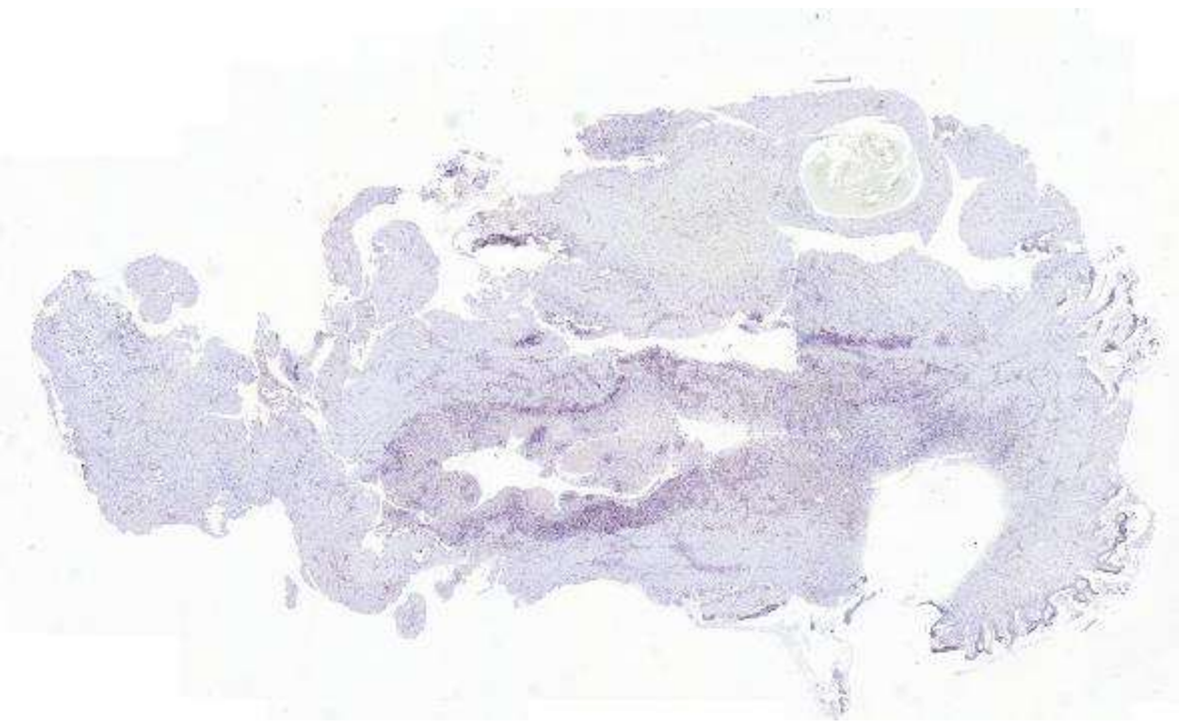

KR13 – HE, CK17, bcl2 (x4)

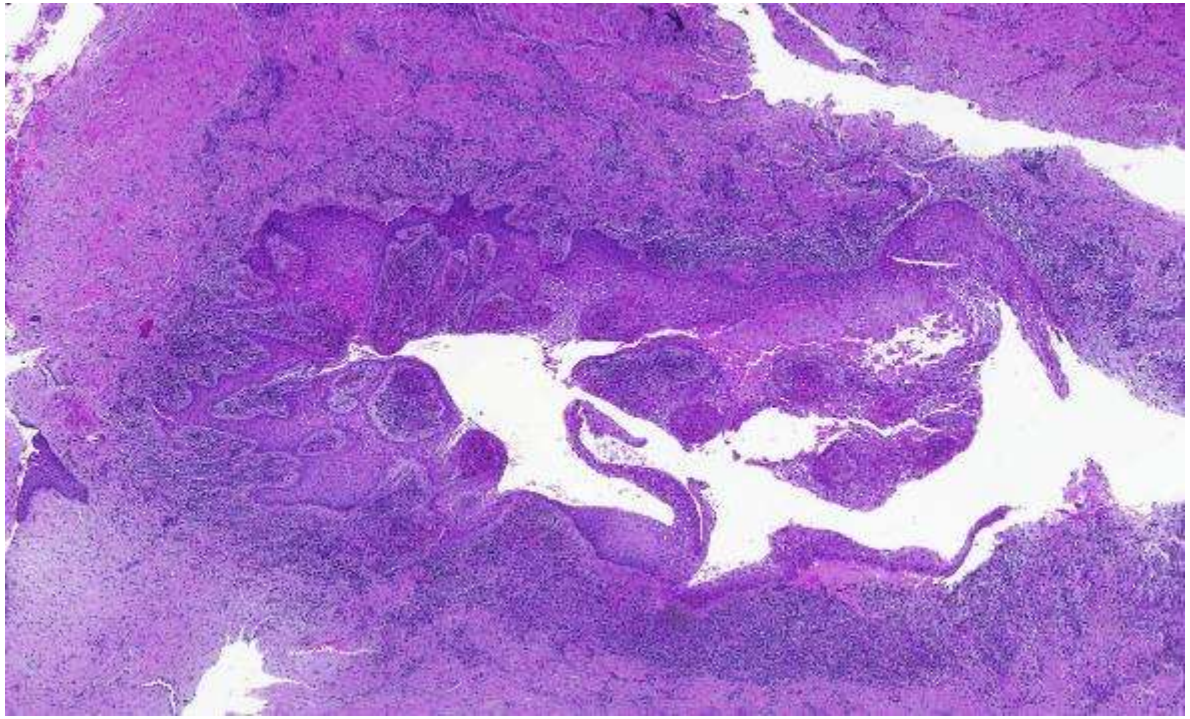

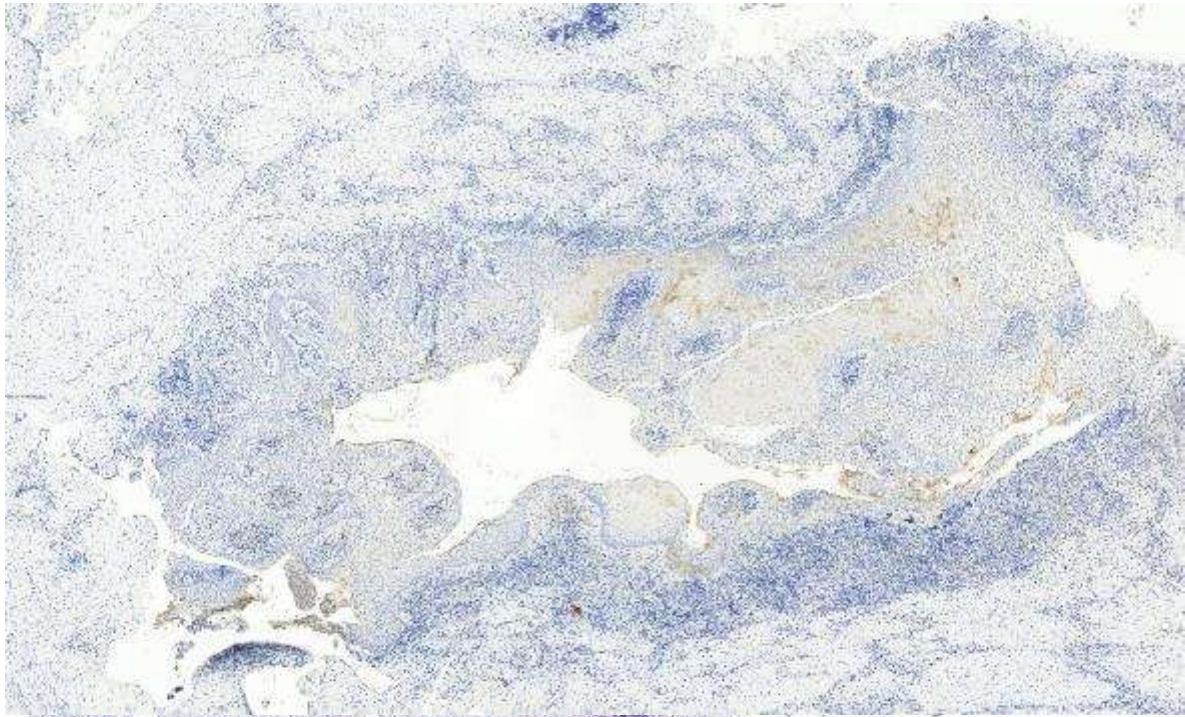

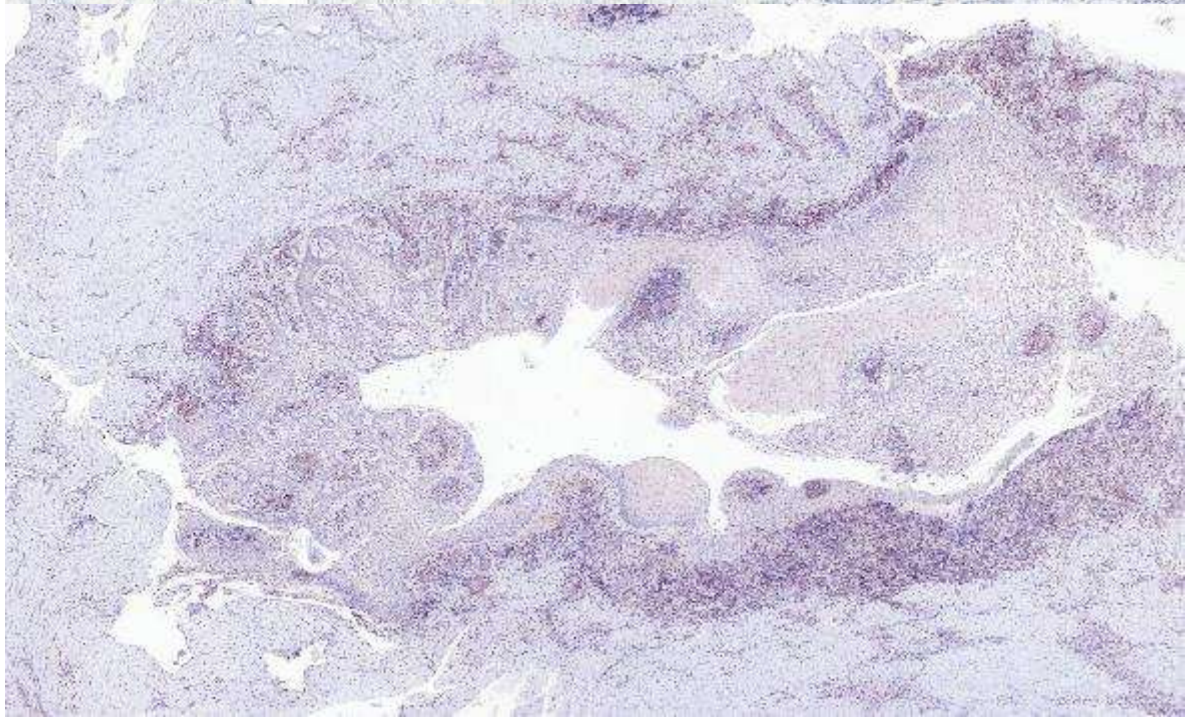

KR13 – HE, CK17, bcl2 (x10)

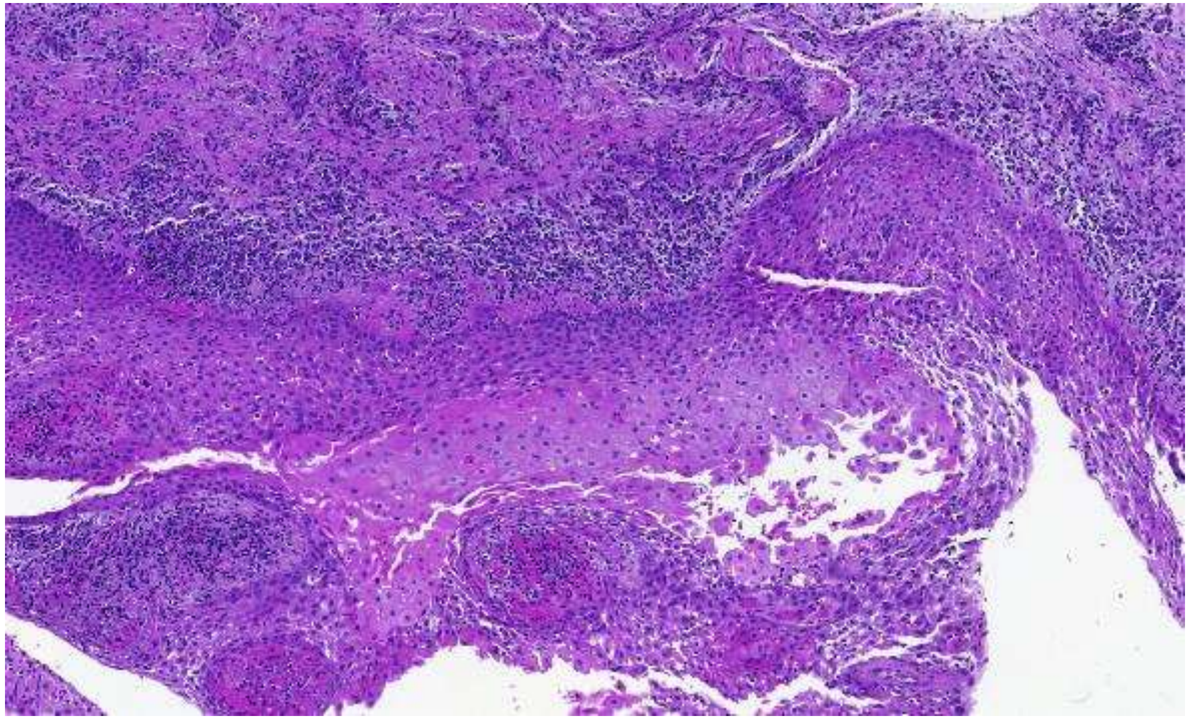

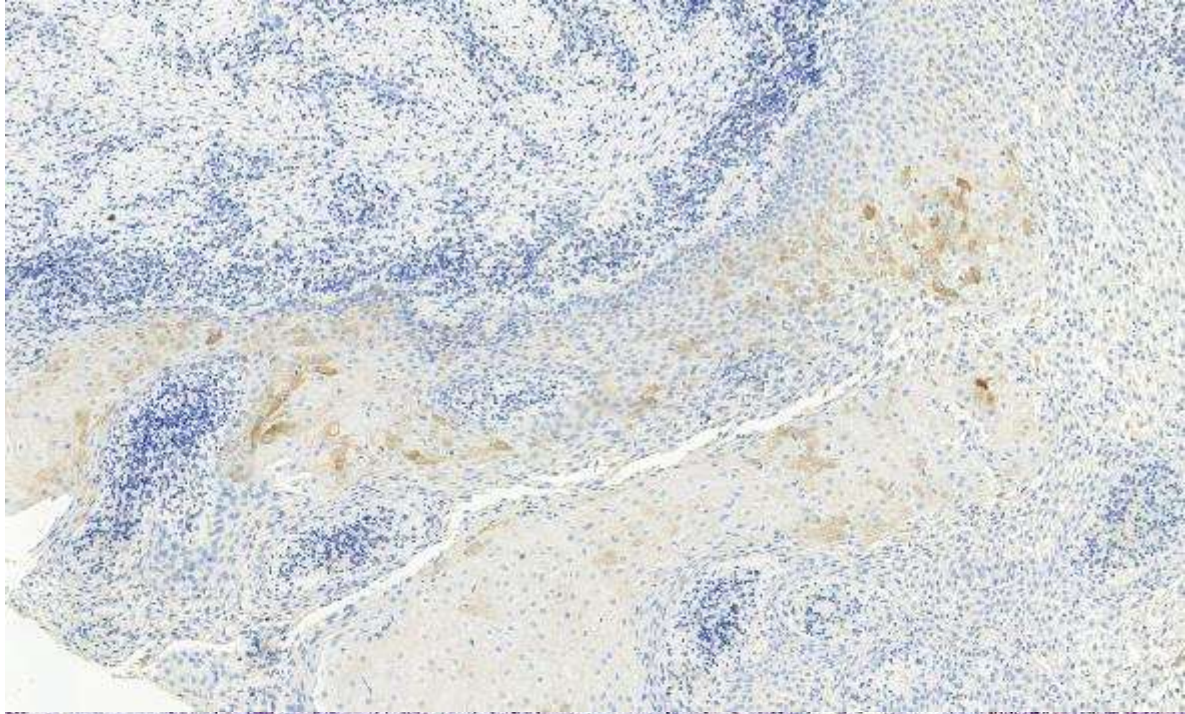

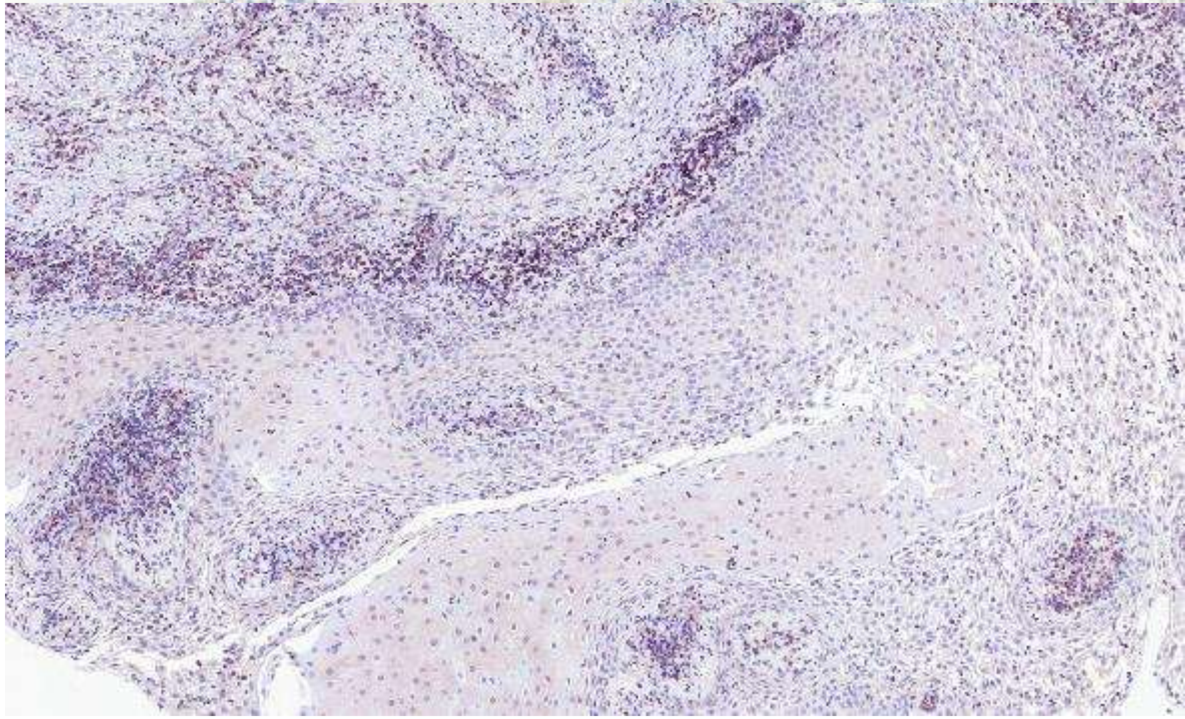

KR13 – HE, CK17, bcl2 (x10)

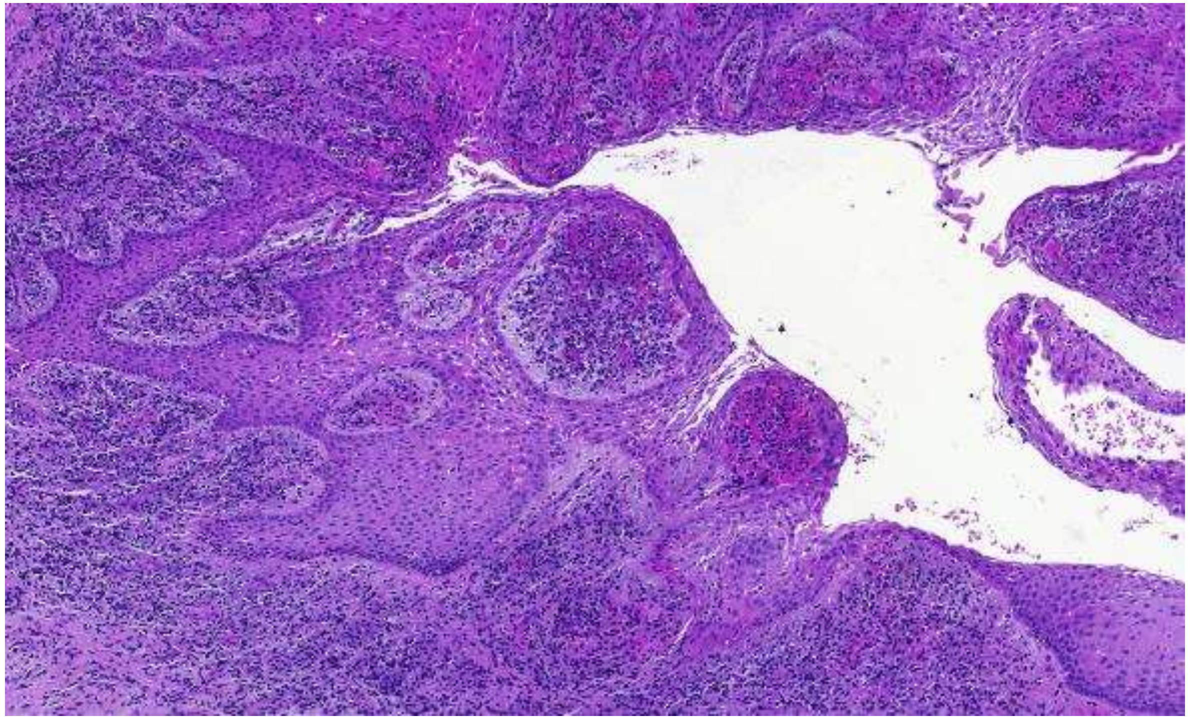

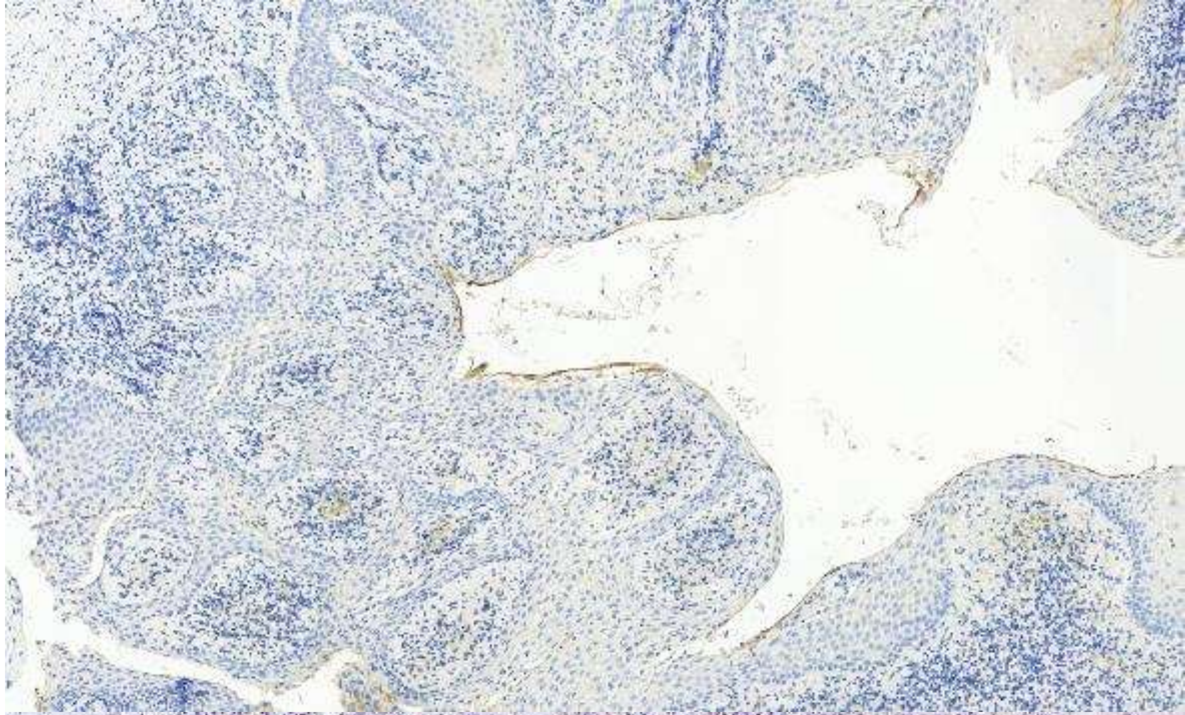

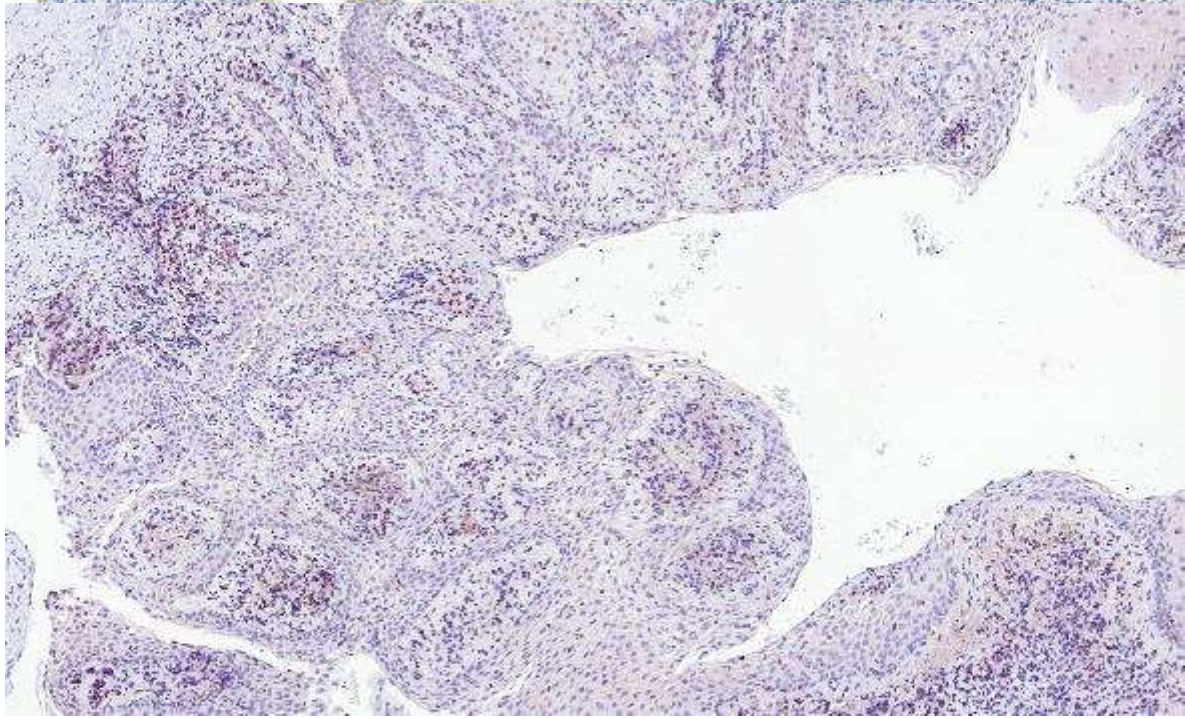

KR13 – Conclusion

- H&E: relatively thick squamous epithelium with scattered dyskeratocytes, no parakeratosis, no wavy surface, no basal palisading, uneven epithelial-subepithelial contours
- Mixed inflammation of the cyst wall with mononuclear cells predominating
- Bcl2: negative
- CK17: dominantly superficial staining with few upper to middle spinous cells labelled
- Histopathology: apical cyst
- Combined clinical and pathological opinion: residual cyst

Case KR14

- 51-year-old male
- Dental hygiene/status: normal; partly missing, partly replaced, partly treated teeth
- Radiology: 2-cm-diameter periapical radiolucency at teeth 11-13
- Operation: cystectomy and apical resection of teeth 12, 11 with retrograde root filling (tooth 13 uninvolved)
- Clinical diagnosis: radicular cyst

KR14 – OPT

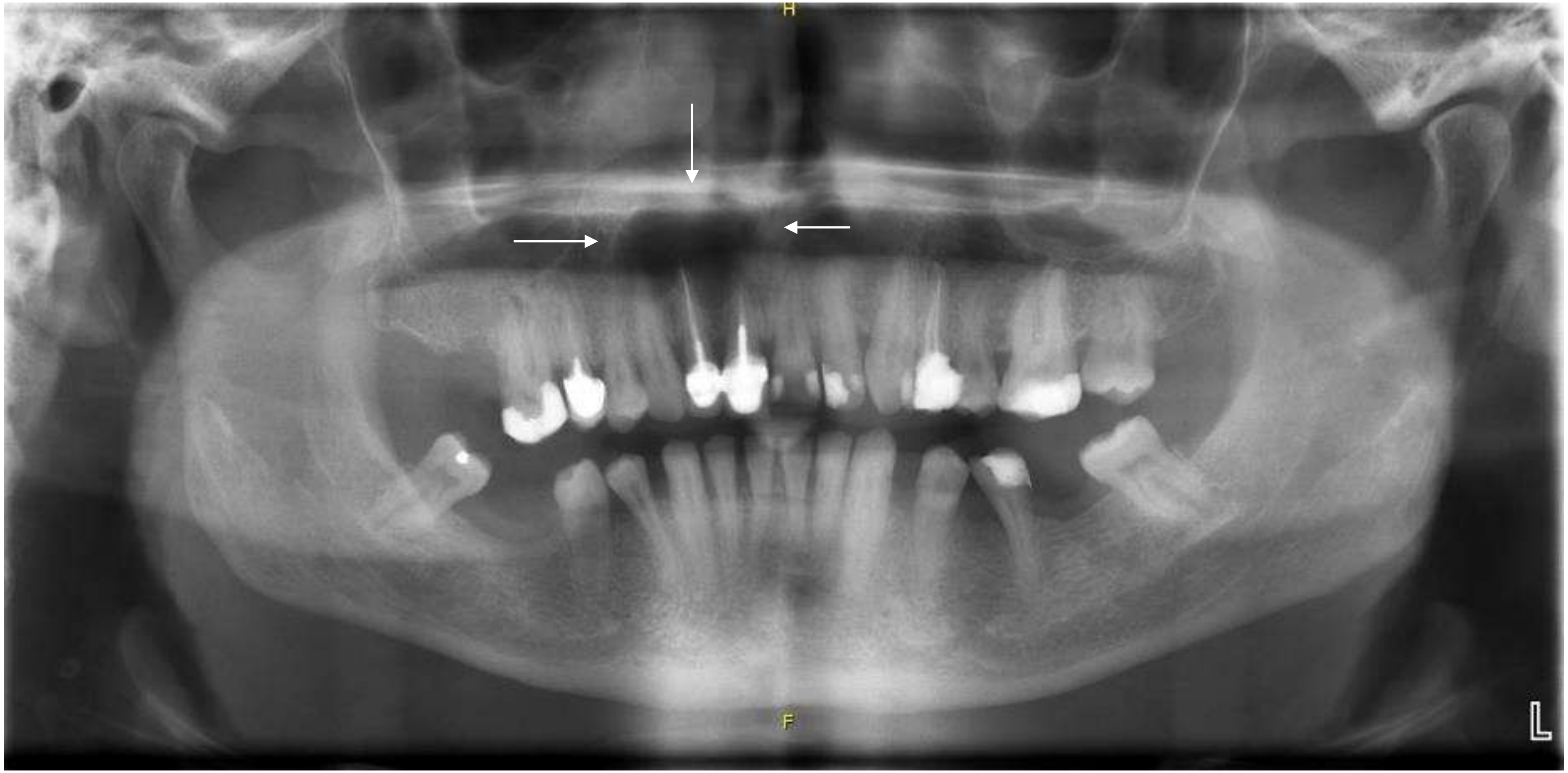

KR14 – OPT magnified

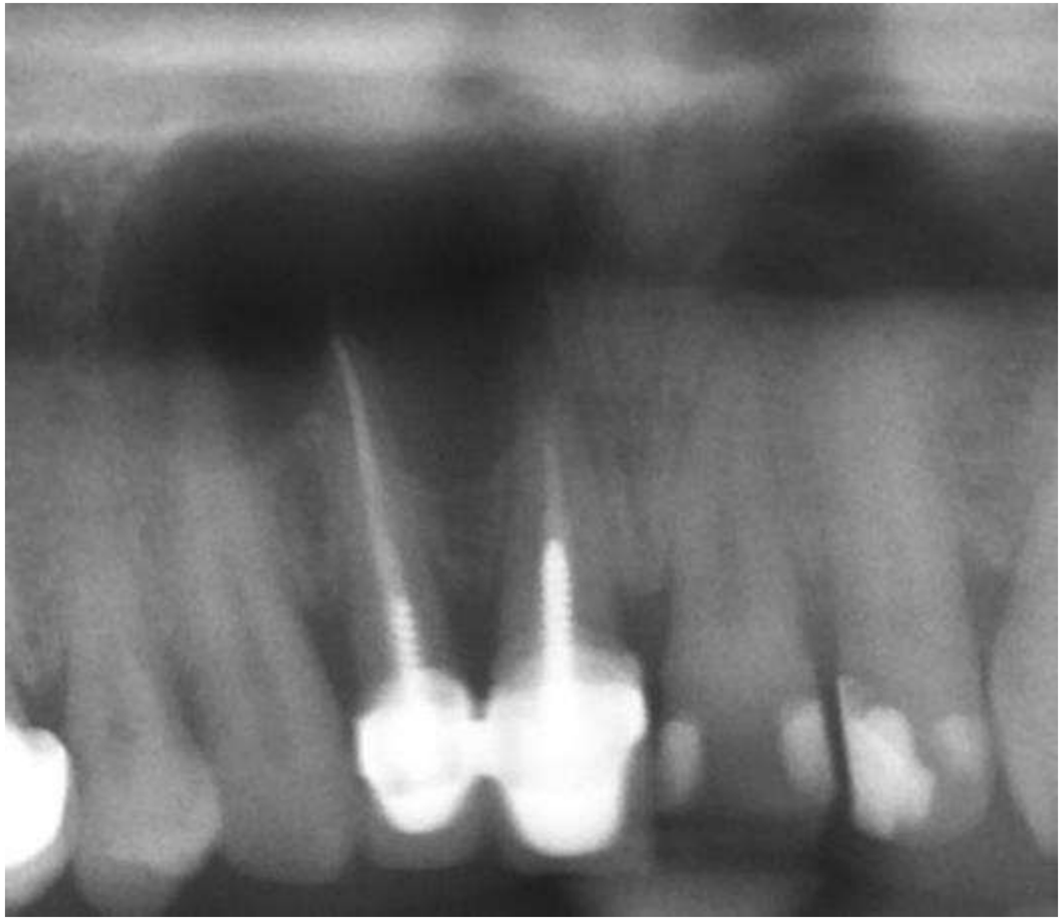

KR14 – HE x2.3

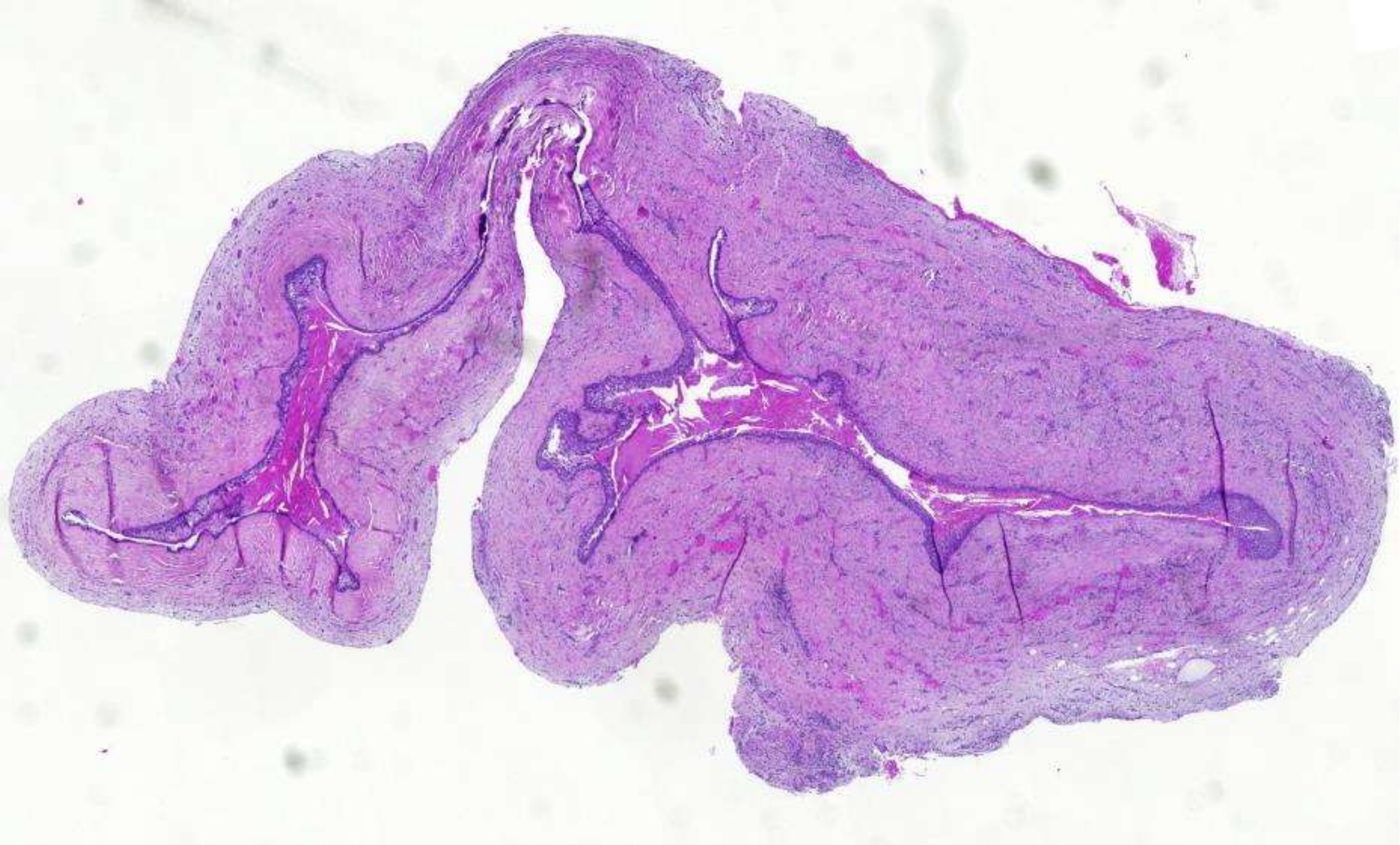

KR14 – Corresponding CK17 & bcl2 (x2)

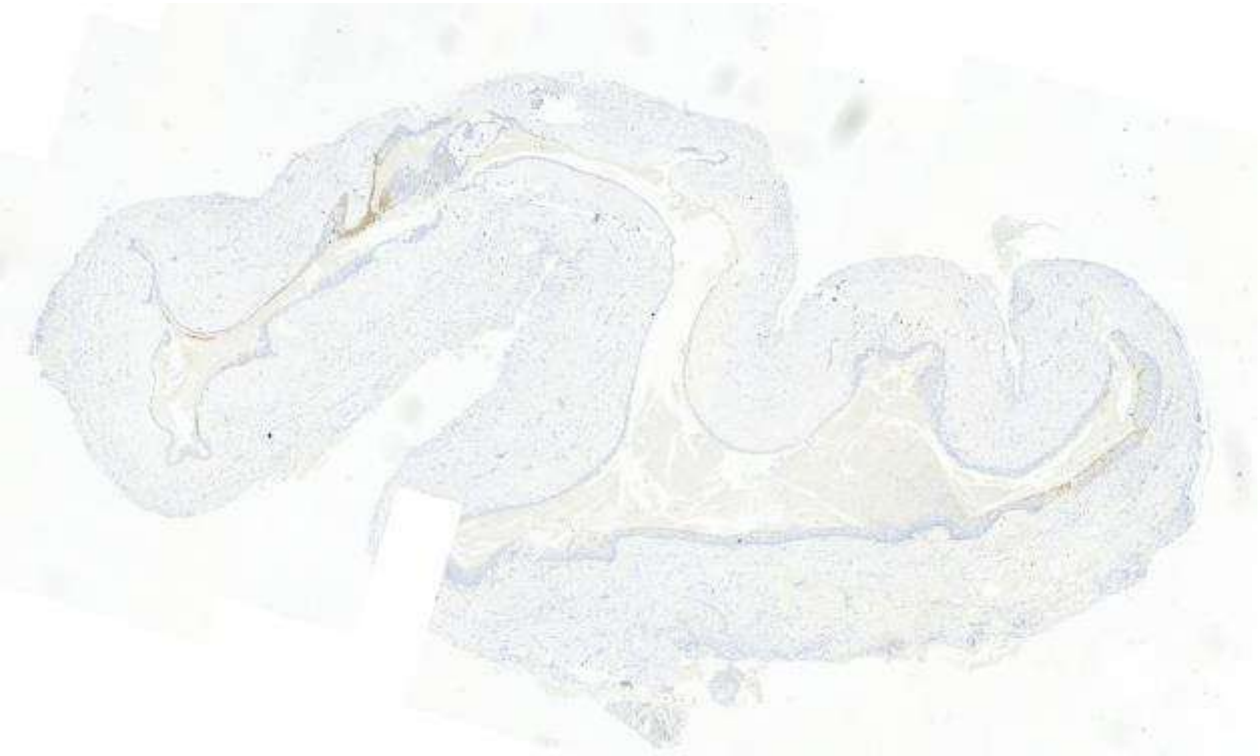

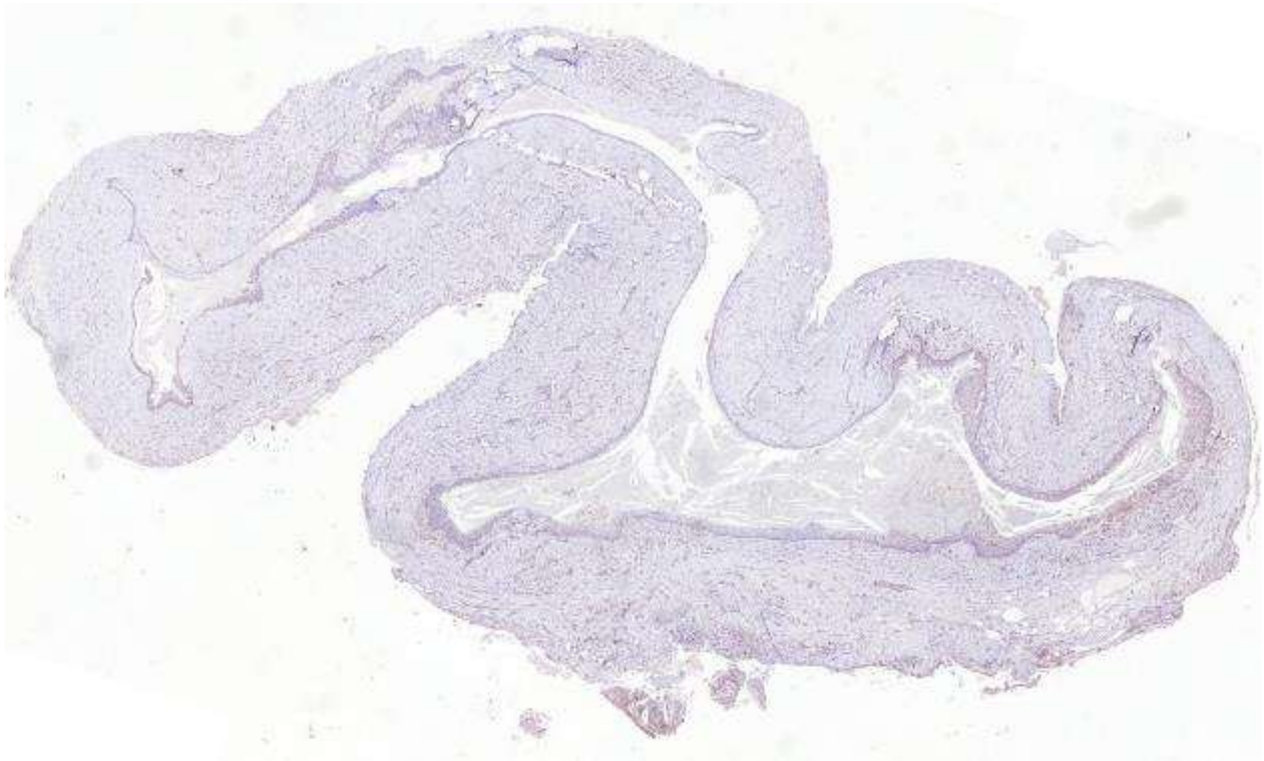

KR14 – HE, CK17, bcl2 (x10)

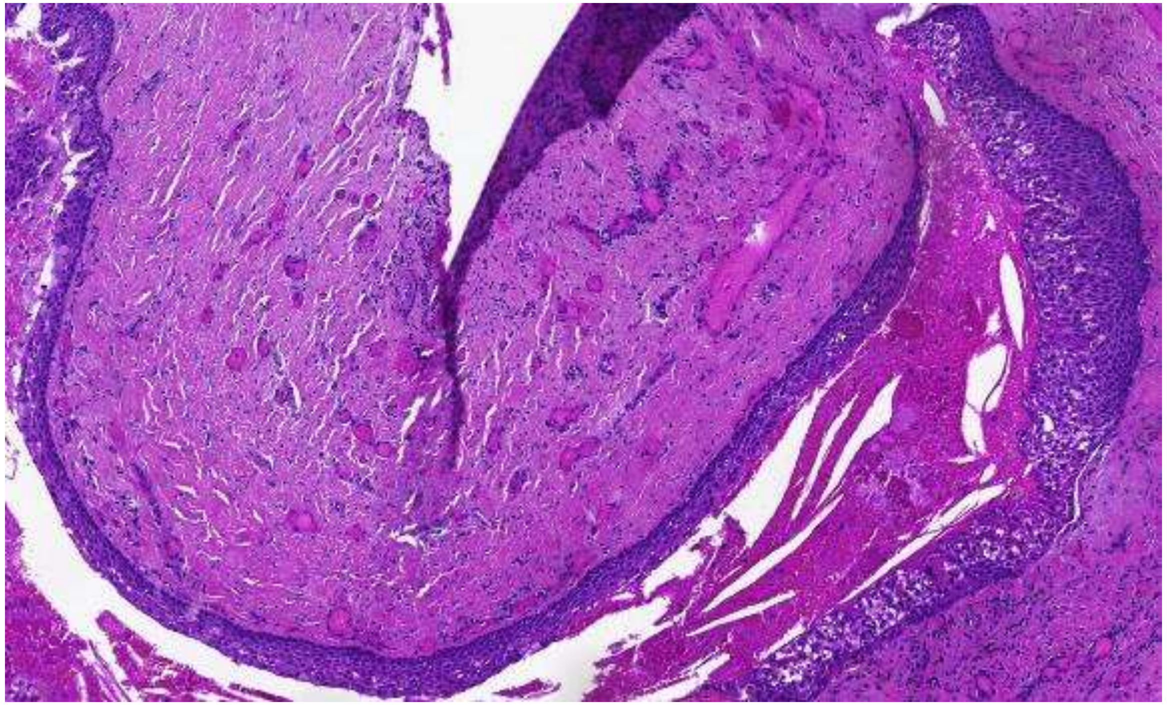

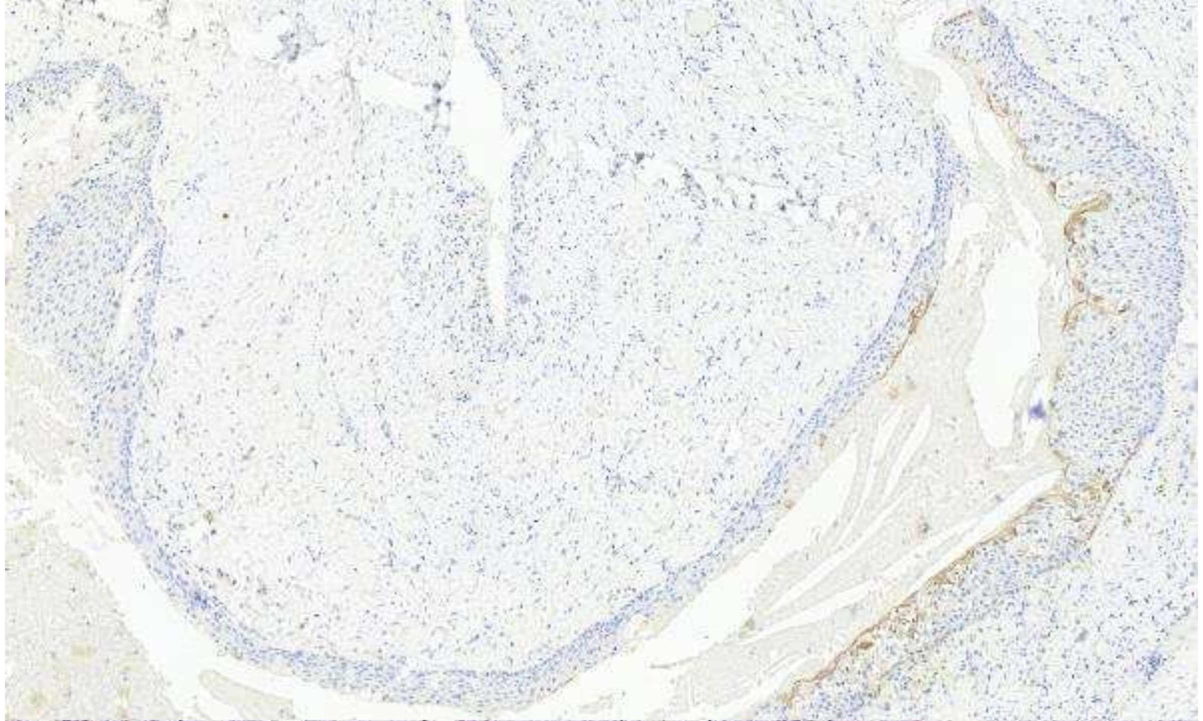

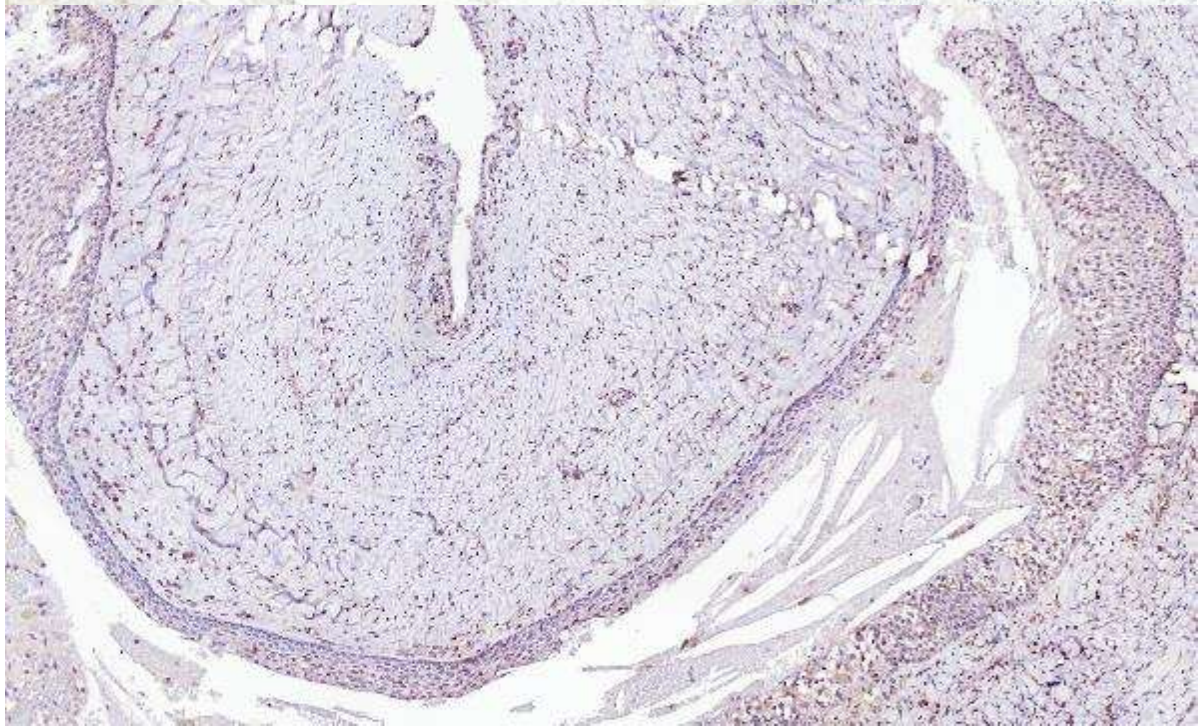

KR14 – HE, CK17, bcl2 (x10)

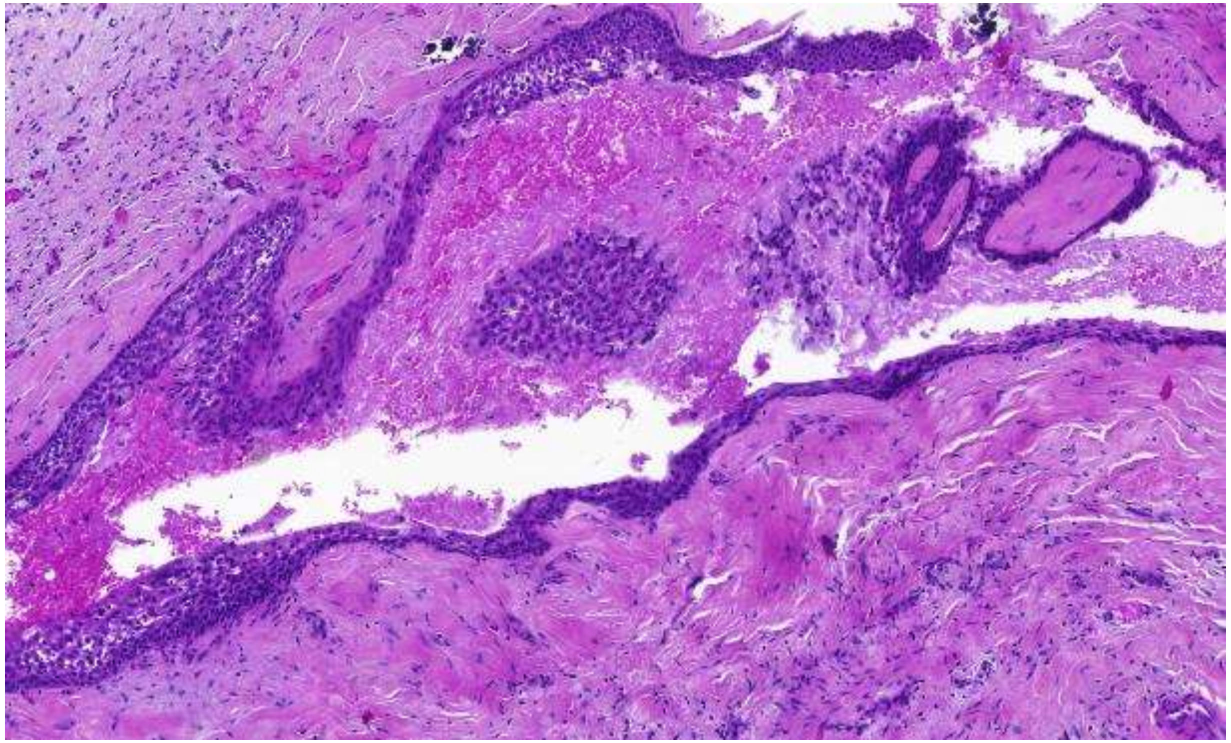

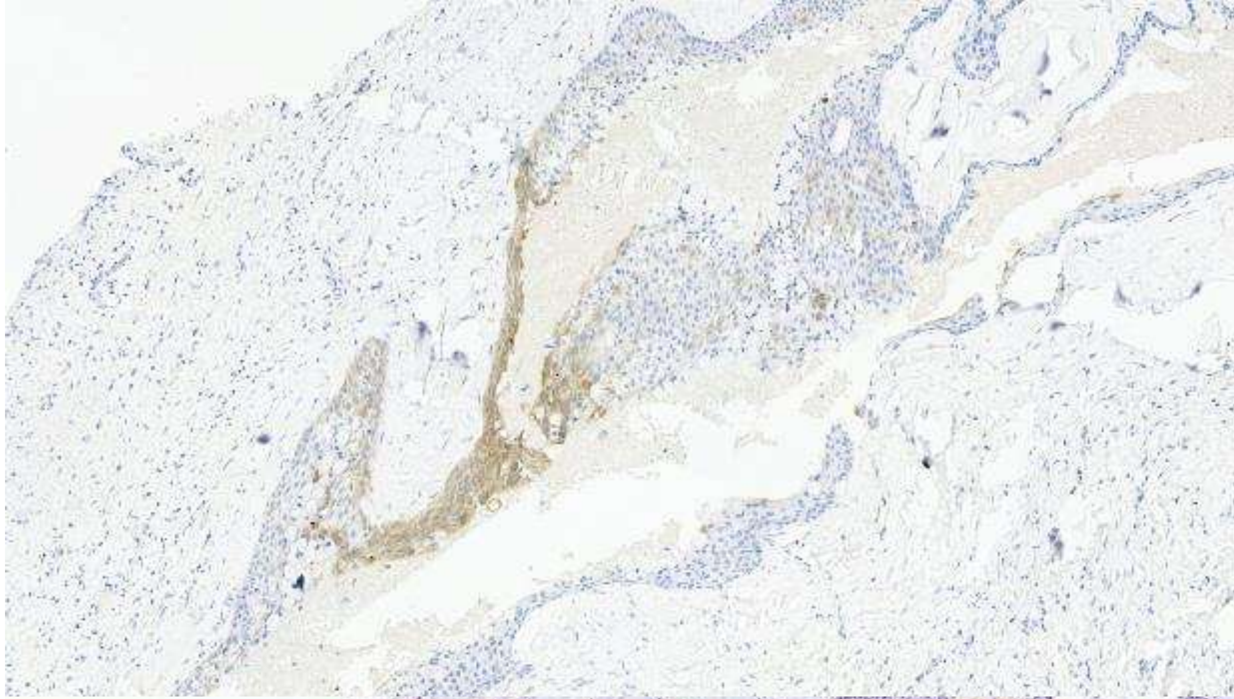

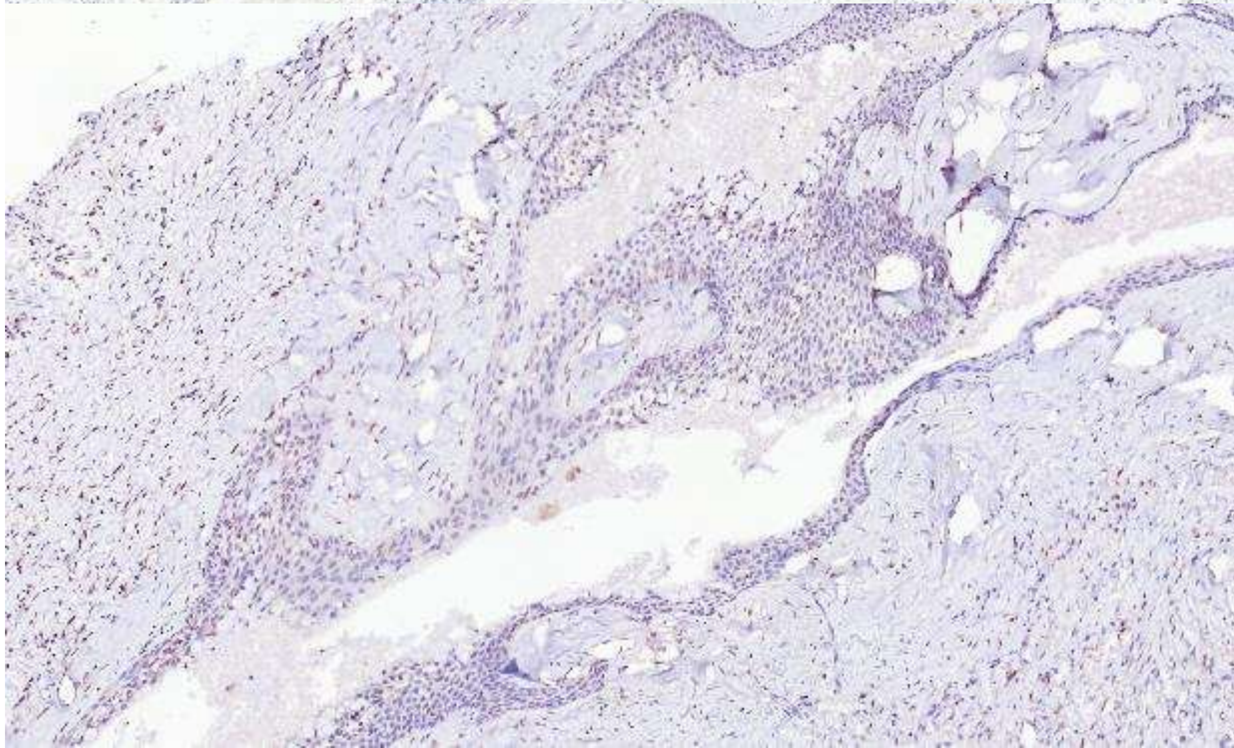

KR14 – HE x1.9

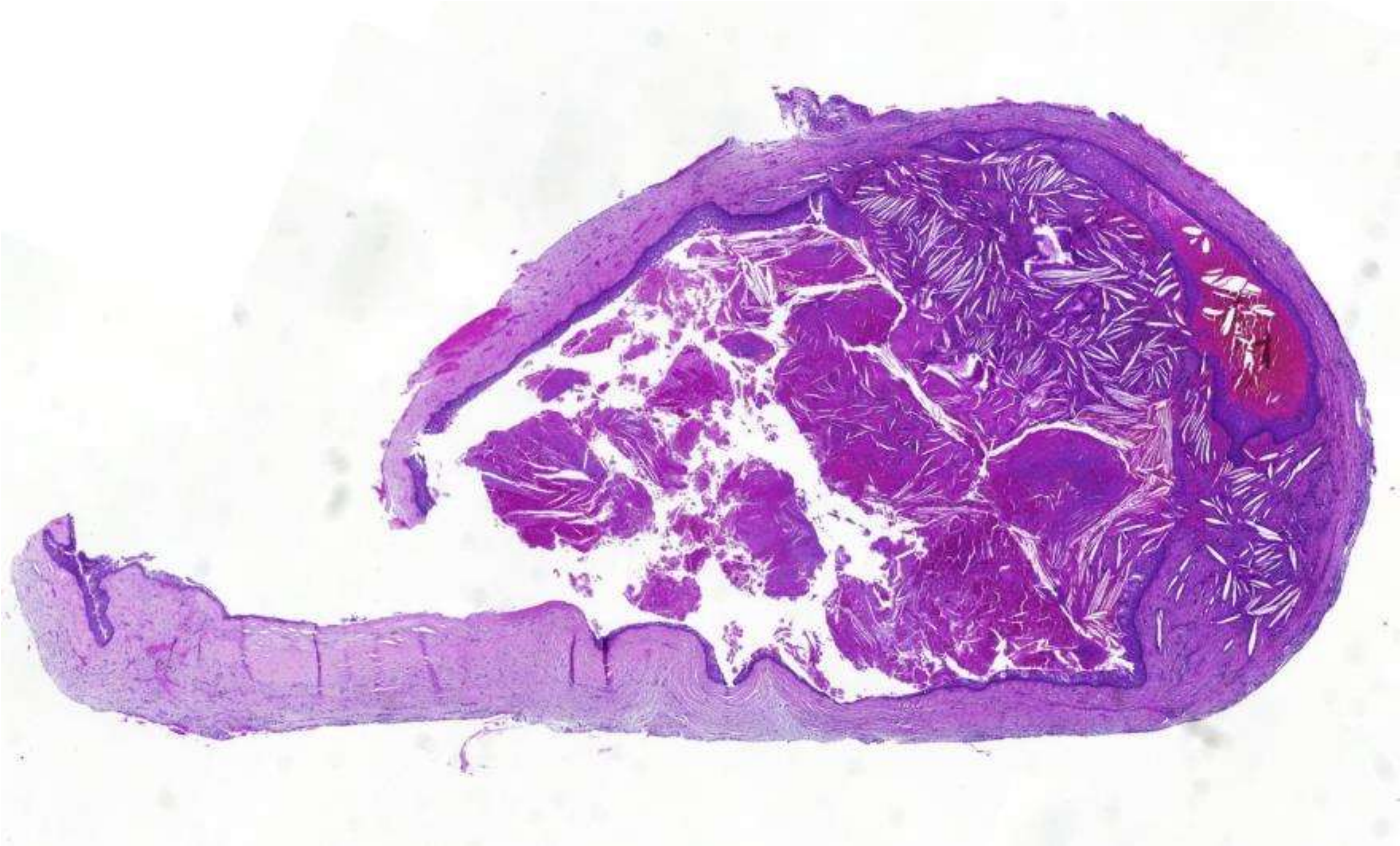

KR14 – Corresponding CK17 & bcl2 (x2)

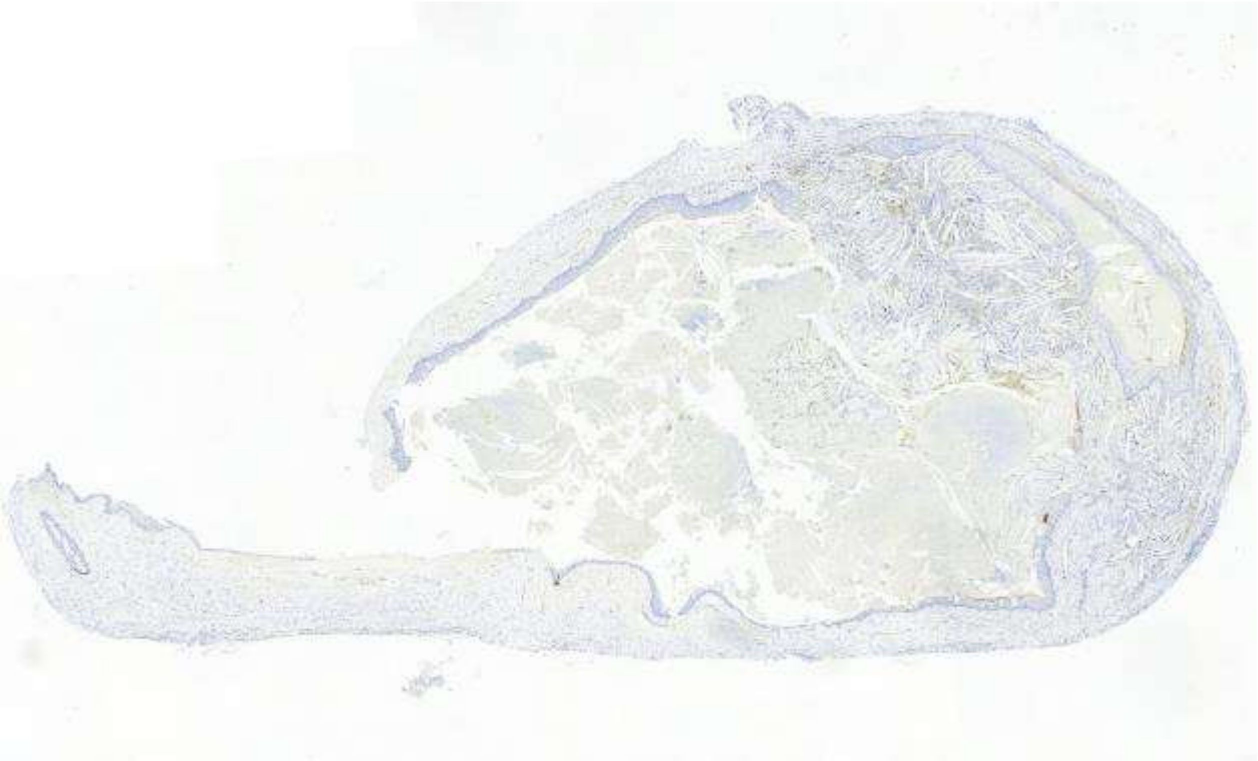

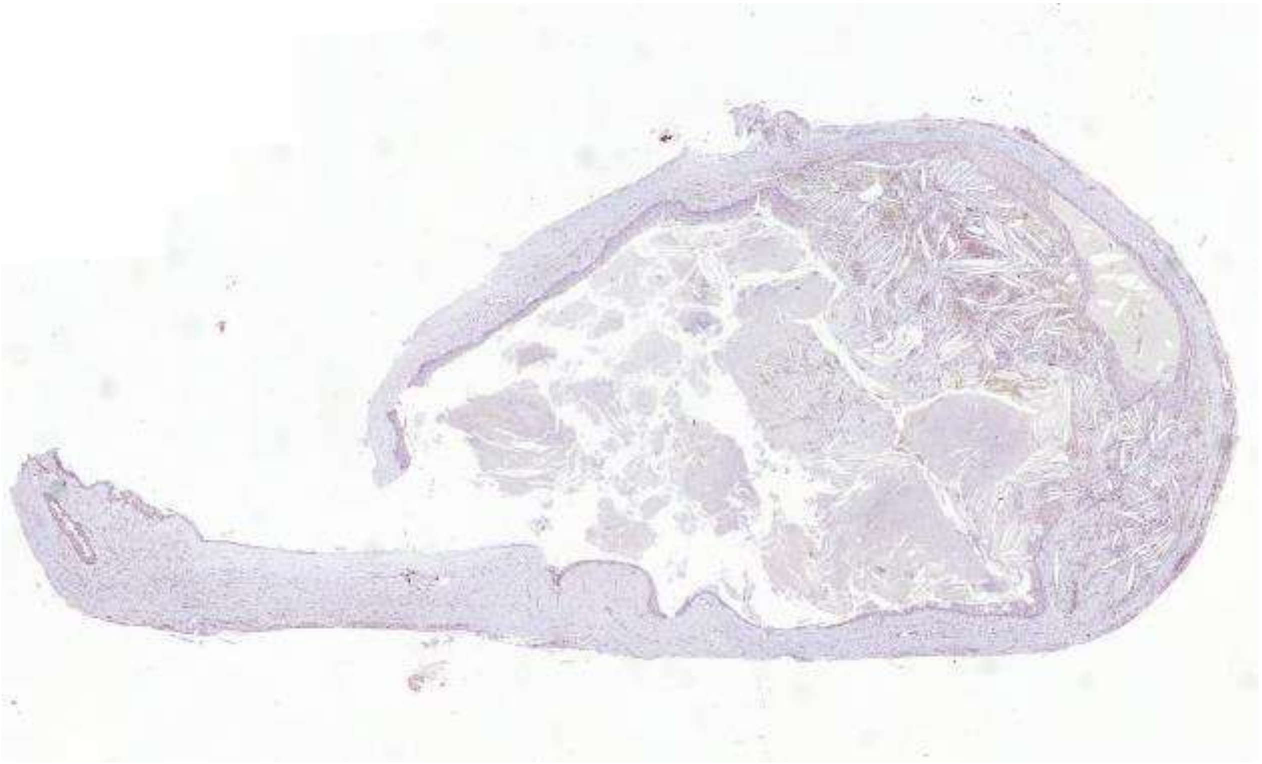

KR14 – HE, CK17, bcl2 (x10)

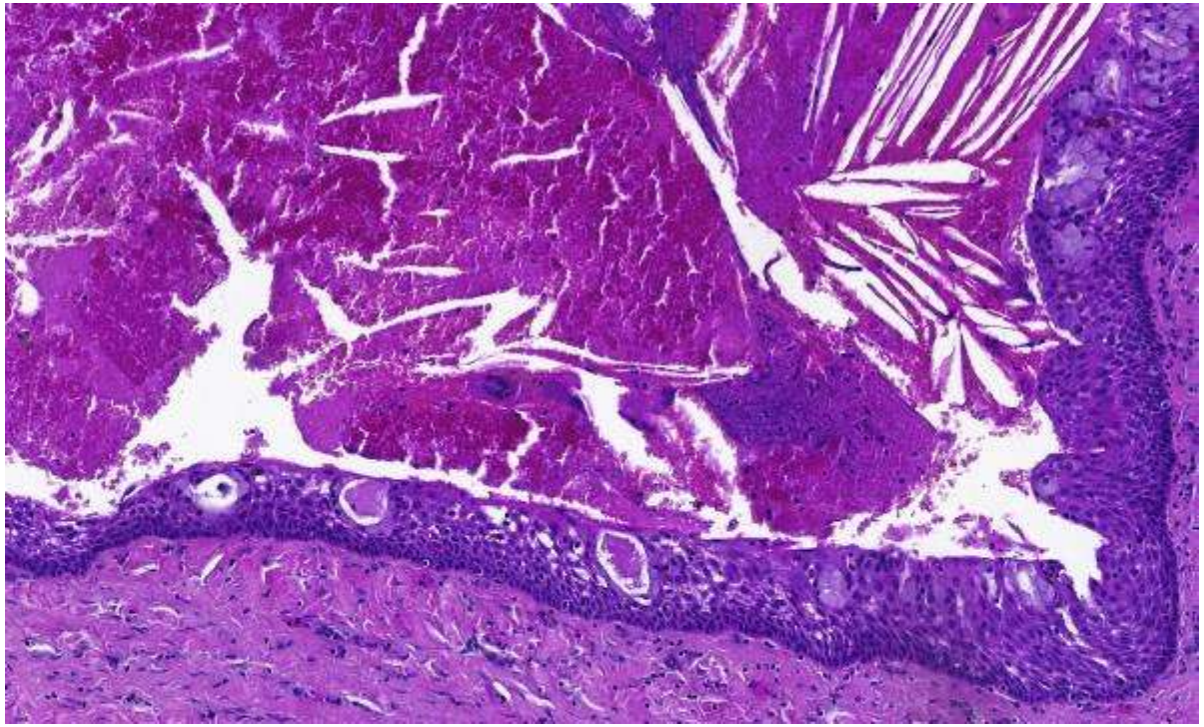

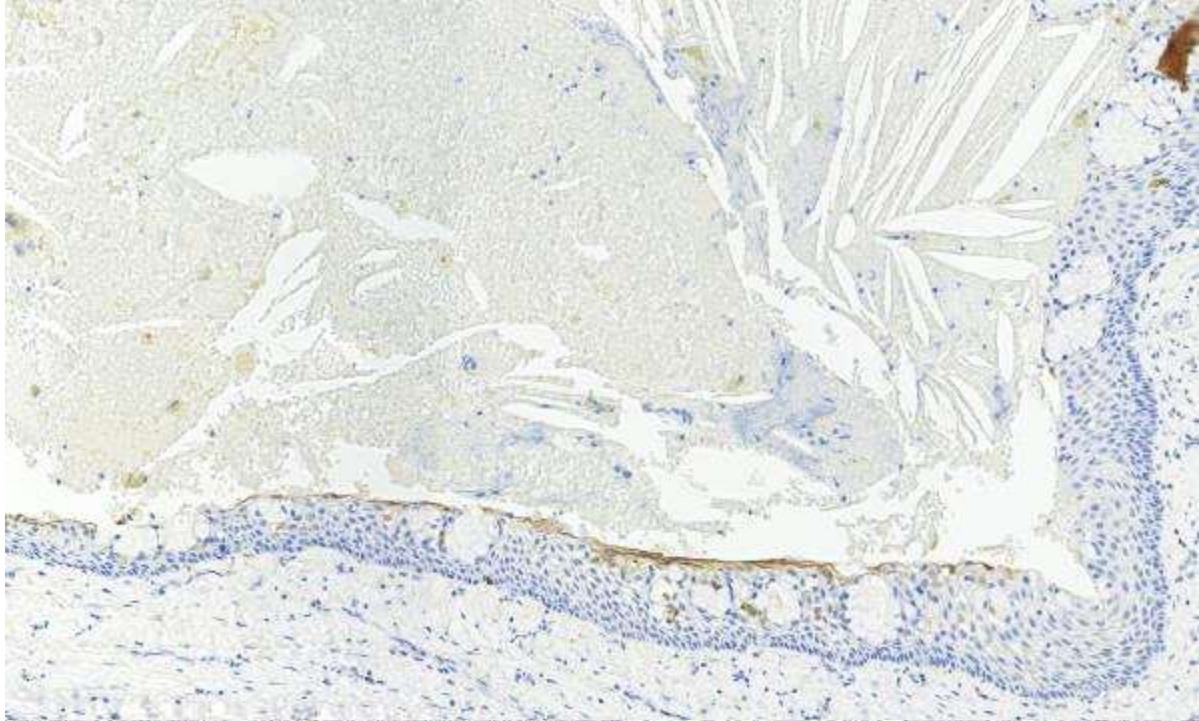

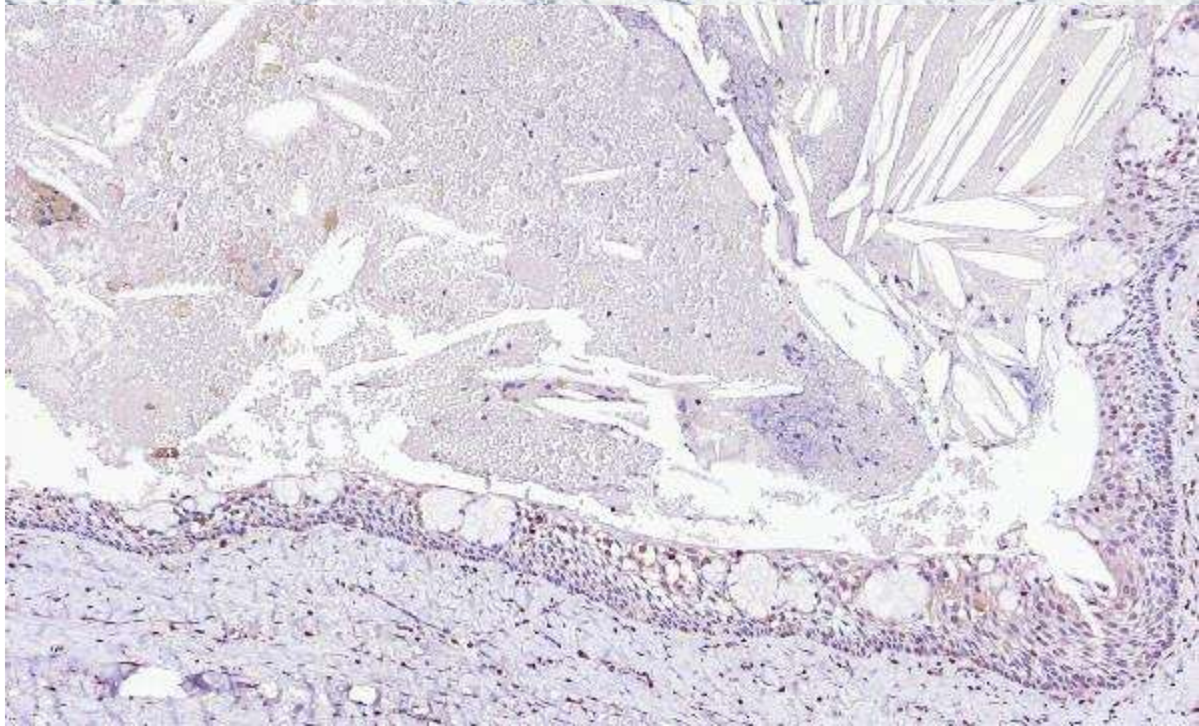

KR14 – Conclusion

- H&E: varying thickness epithelium, predominantly with superficial mucous cells (prominent mucous metaplasia), squamous elsewhere; focal glandular epithelial microcysts (duct-like intraepithelial structures); no parakeratosis, no wavy surface, no basal palisading, dominantly even epithelial-subepithelial contours
- No inflammation of the cyst wall, but an area of cholesterol granuloma (mainly intracystic, but breaking out of the cyst) and minor chronic inflammation associated with this
- Bcl2: negative
- CK17: dominantly negative, focal superficial (with occasional upper spinous cells) or transepithelial staining
- Histopathology: radicular cyst
- Combined clinical and pathological opinion: radicular cyst (probably two fused)

Case KR15

- 44-year-old male
- Dental hygiene/status: partly conserved, partly missing teeth, roots of teeth 11 an 18 in the alveoli; vitality of tooth 27 is lost.
- Radiology: radiolucent lesion of 20 mm in size in the left maxillary sinus at position of tooth 27. Note the maintained lower wall of the maxillary sinus!
- Operation: removal of the cyst; the material received was fragmented
- Clinical diagnosis: cyst (N.O.S.), residual cyst mentioned elsewhere in the charts

KR15 – OPT (not obvious)

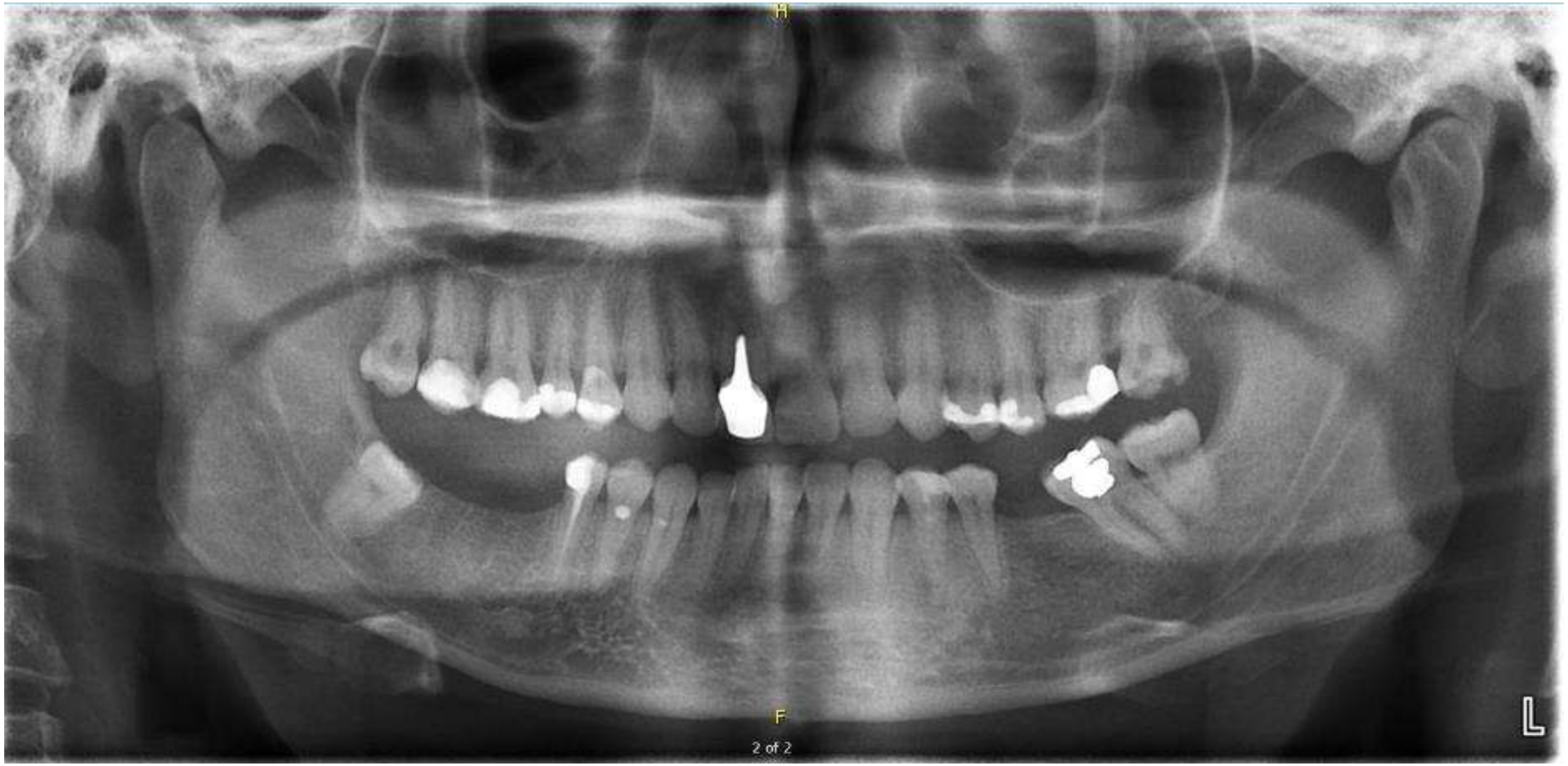

KR15 – CT axial

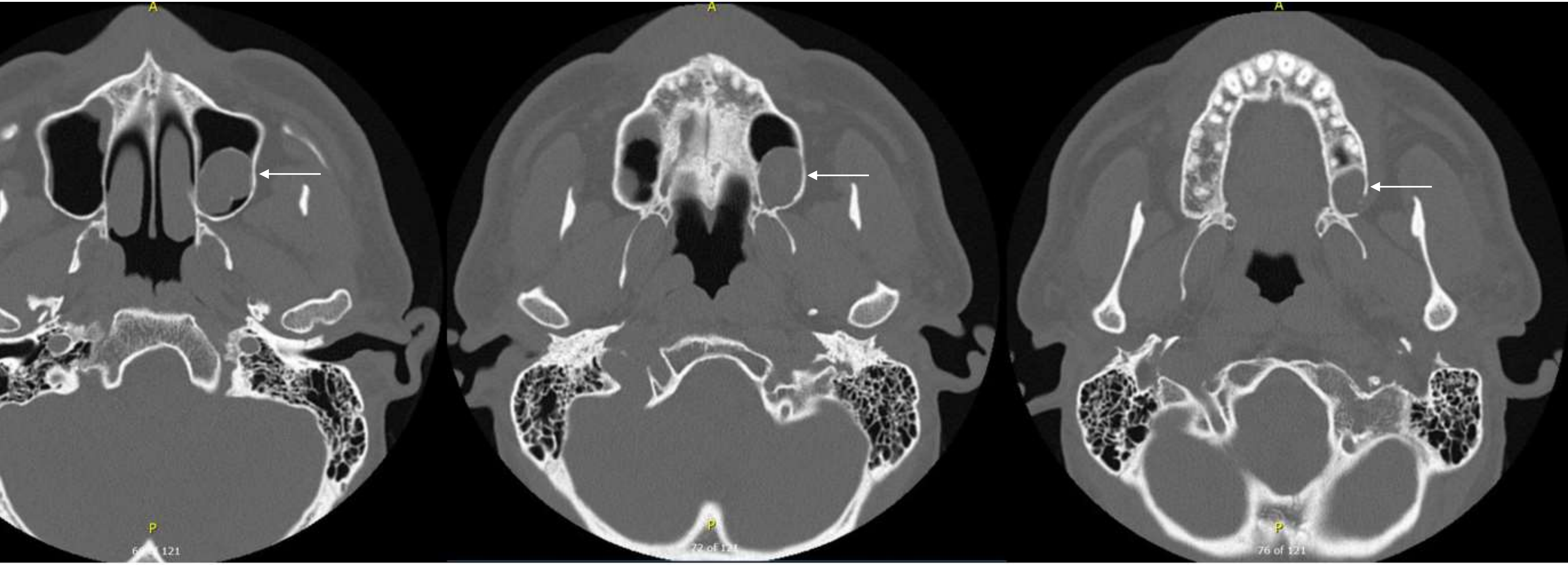

KR15 – CT coronal

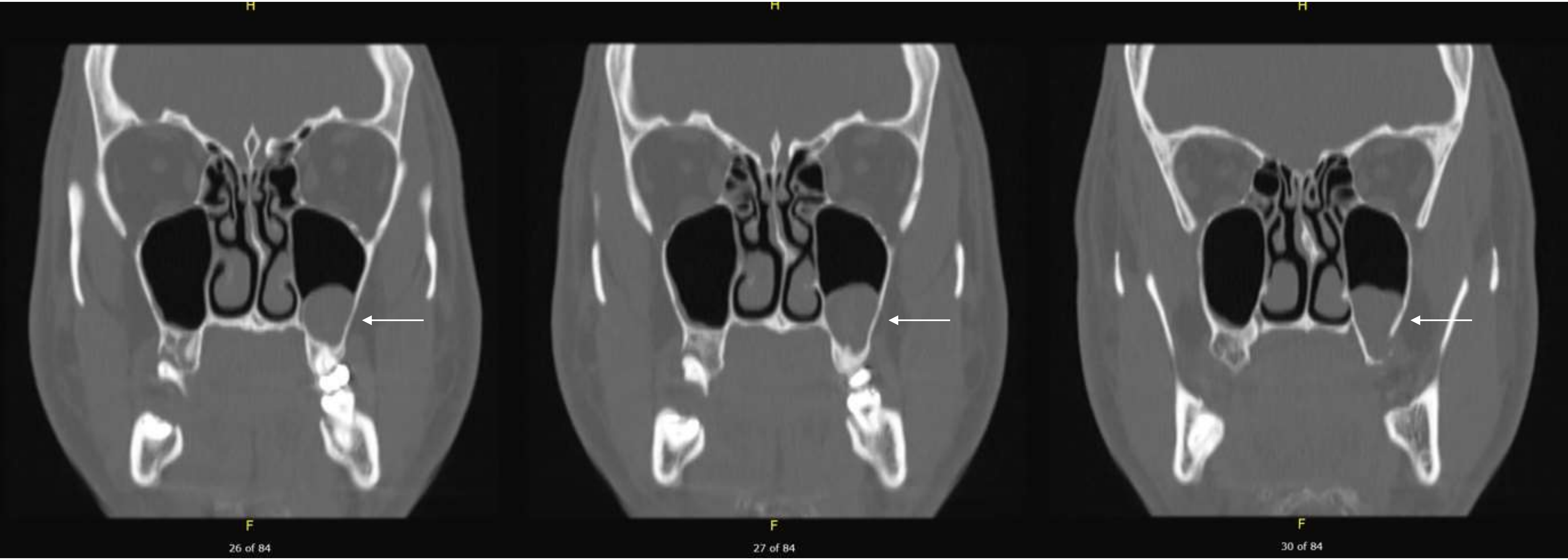

KR15 – CT sagittal

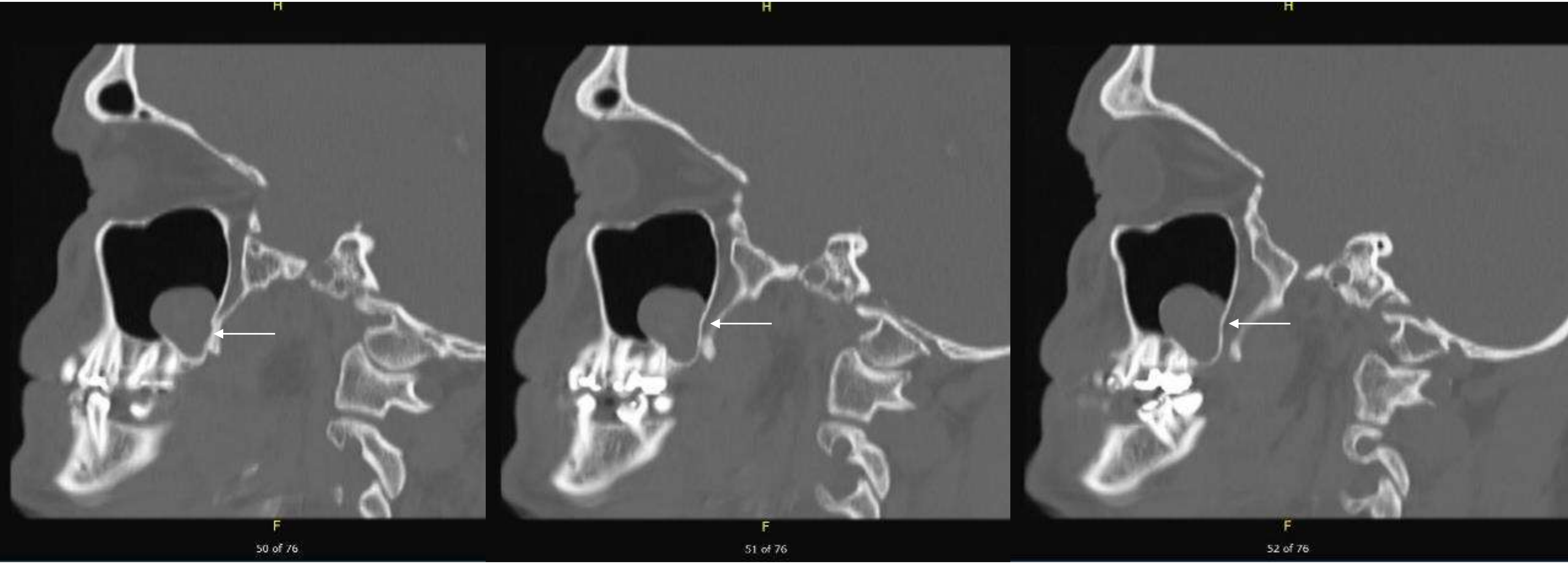

KR15 –
HE, CK17, bcl2 (x1)

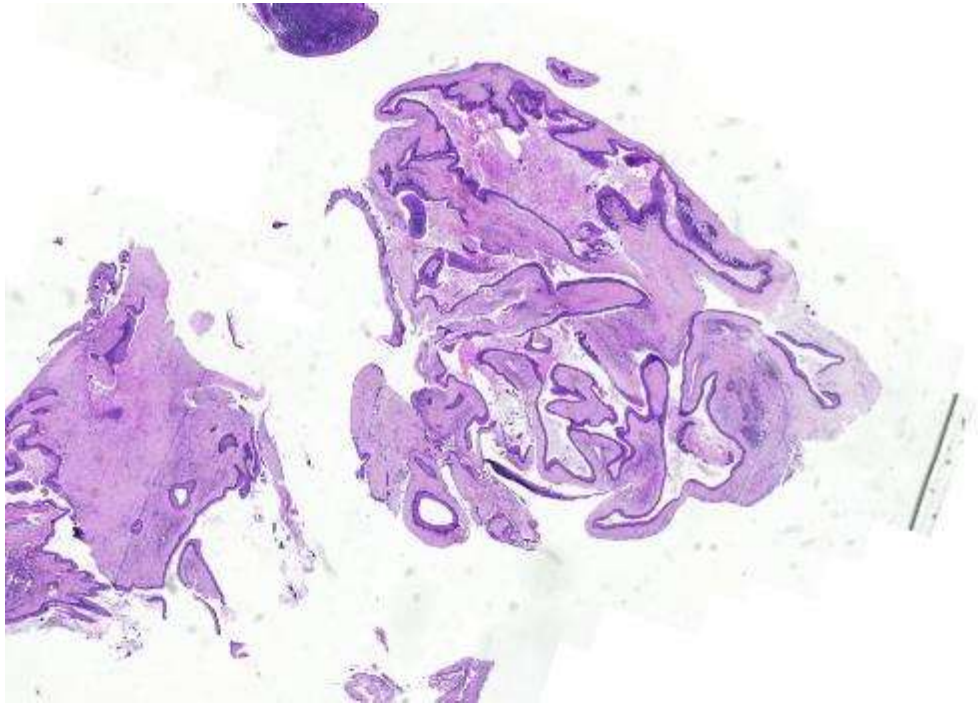

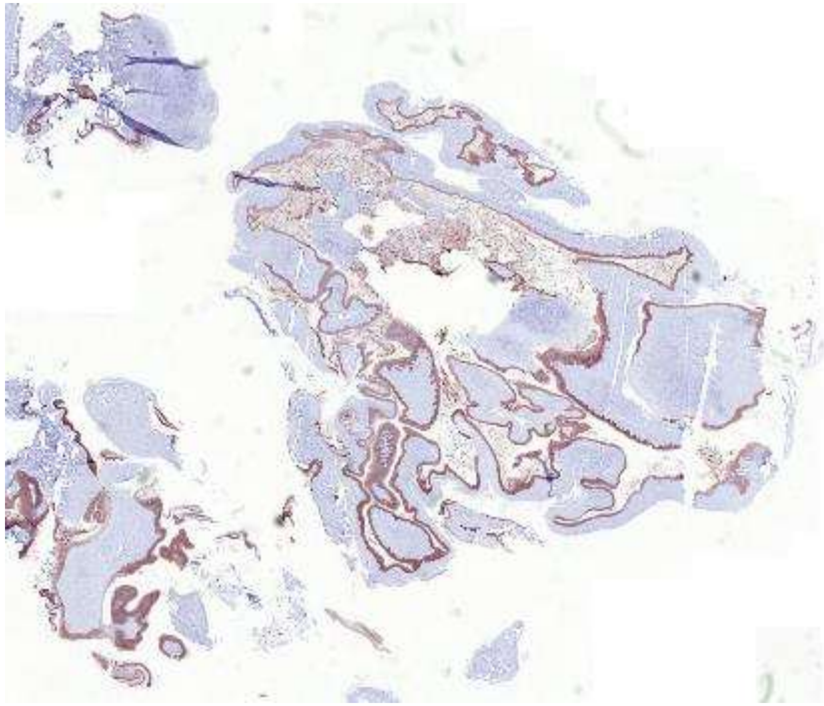

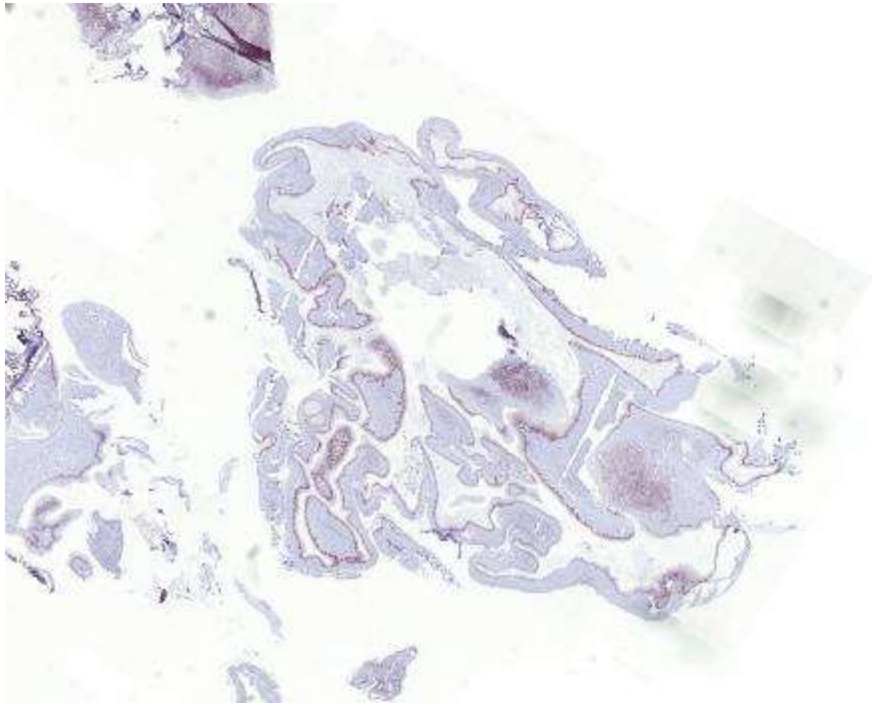

KR15 – CK10, CK19 (x1)

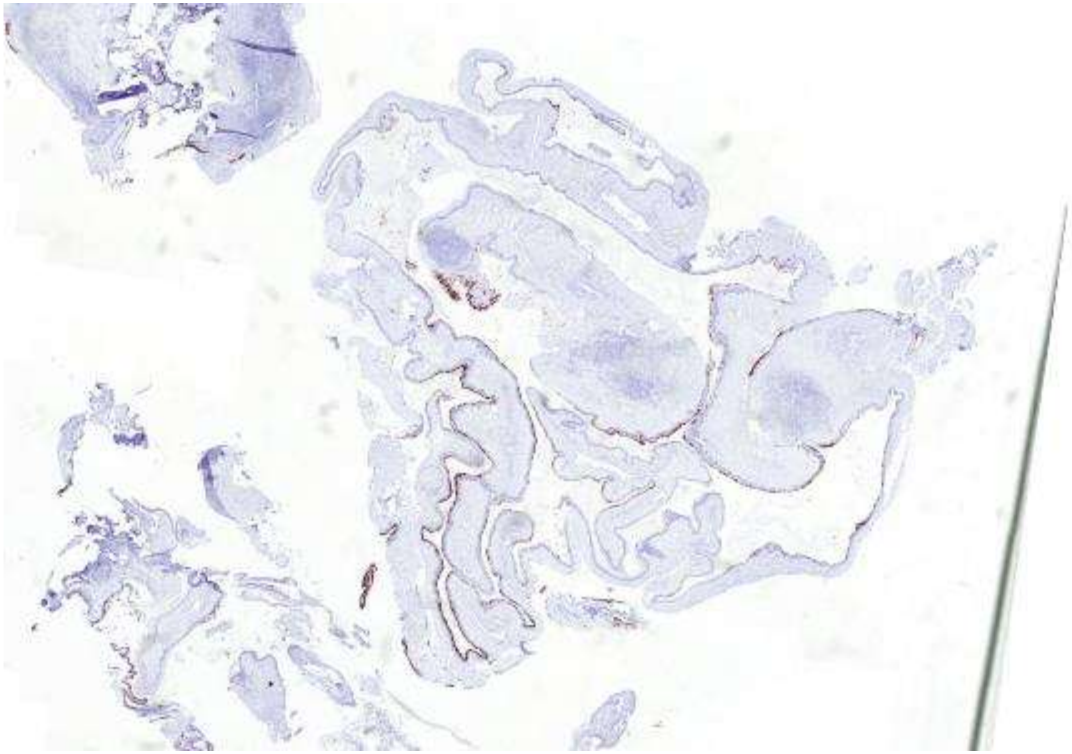

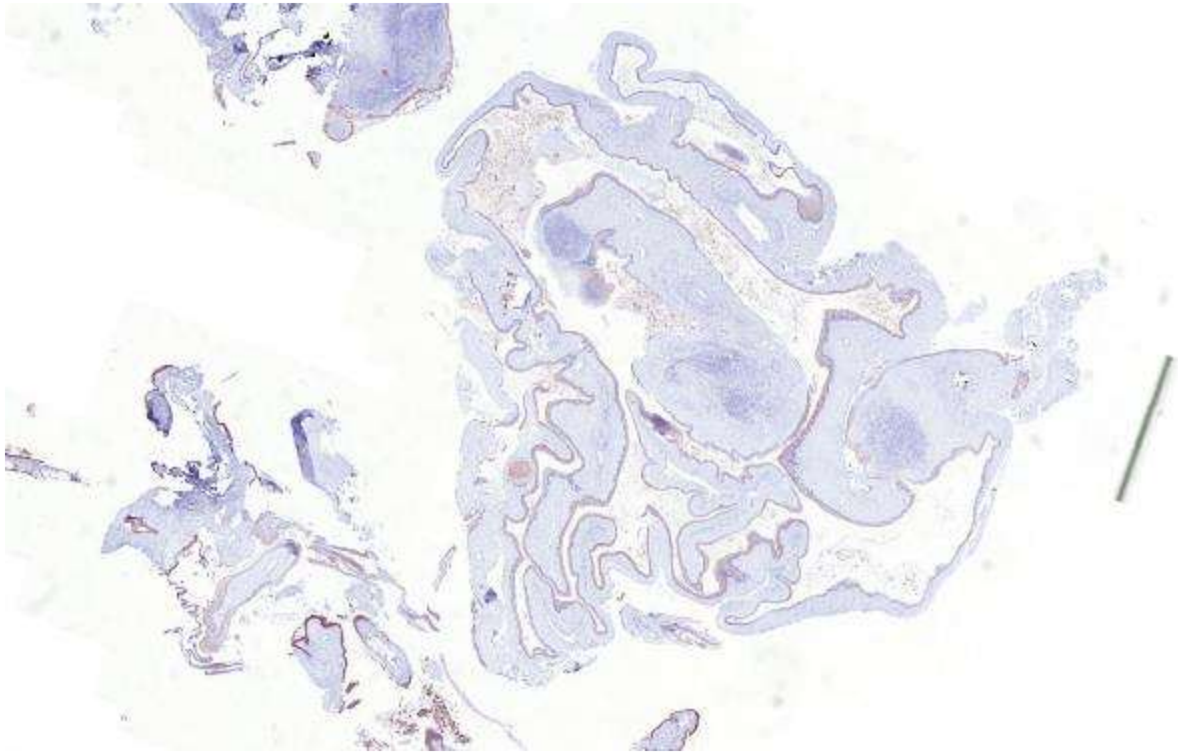

KR15 –
HE, CK17, bcl2 (x5)

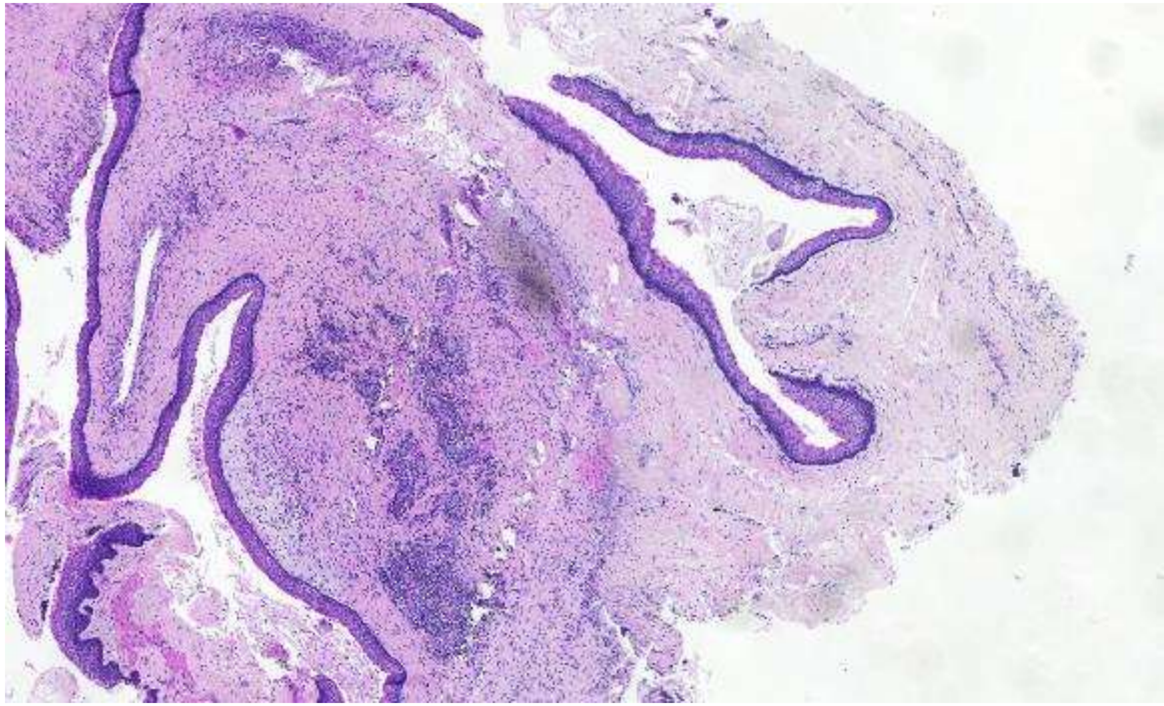

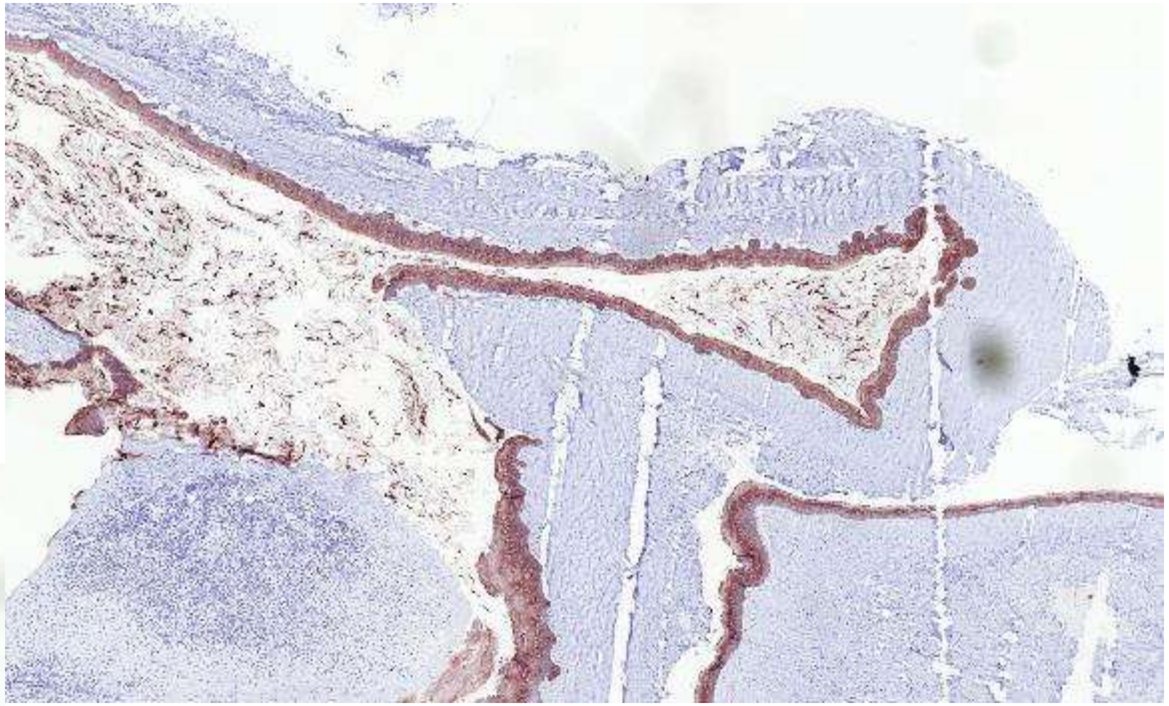

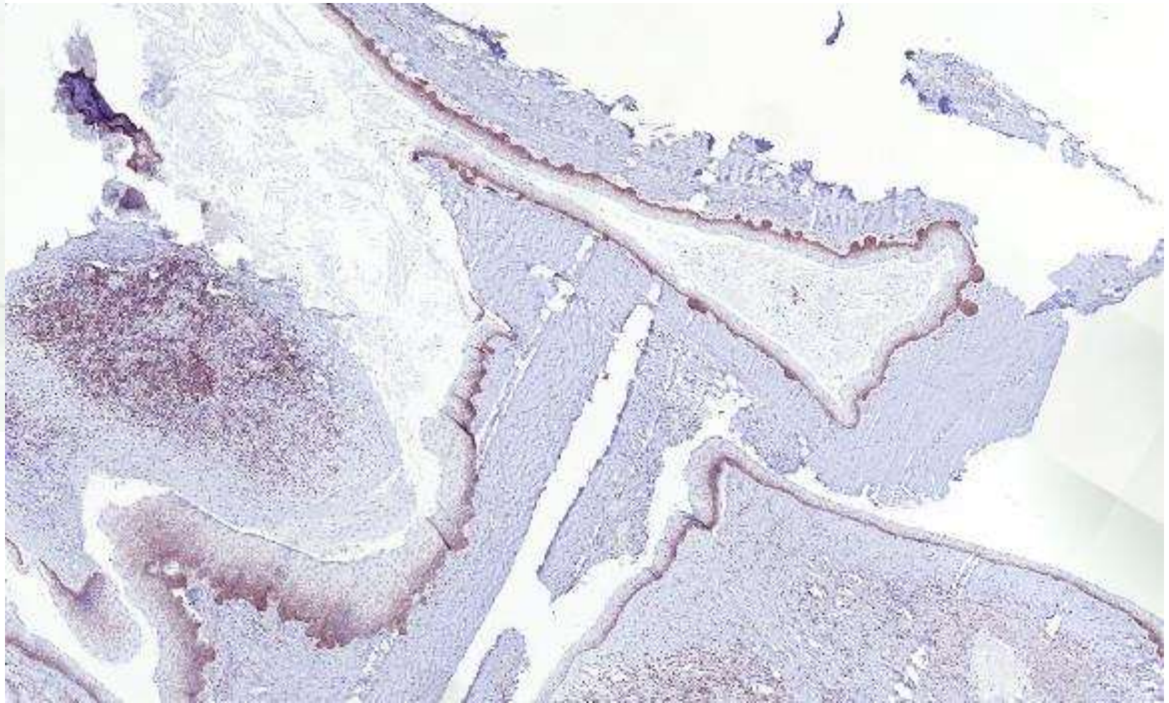

KR15 – CK10, CK19 (x5)

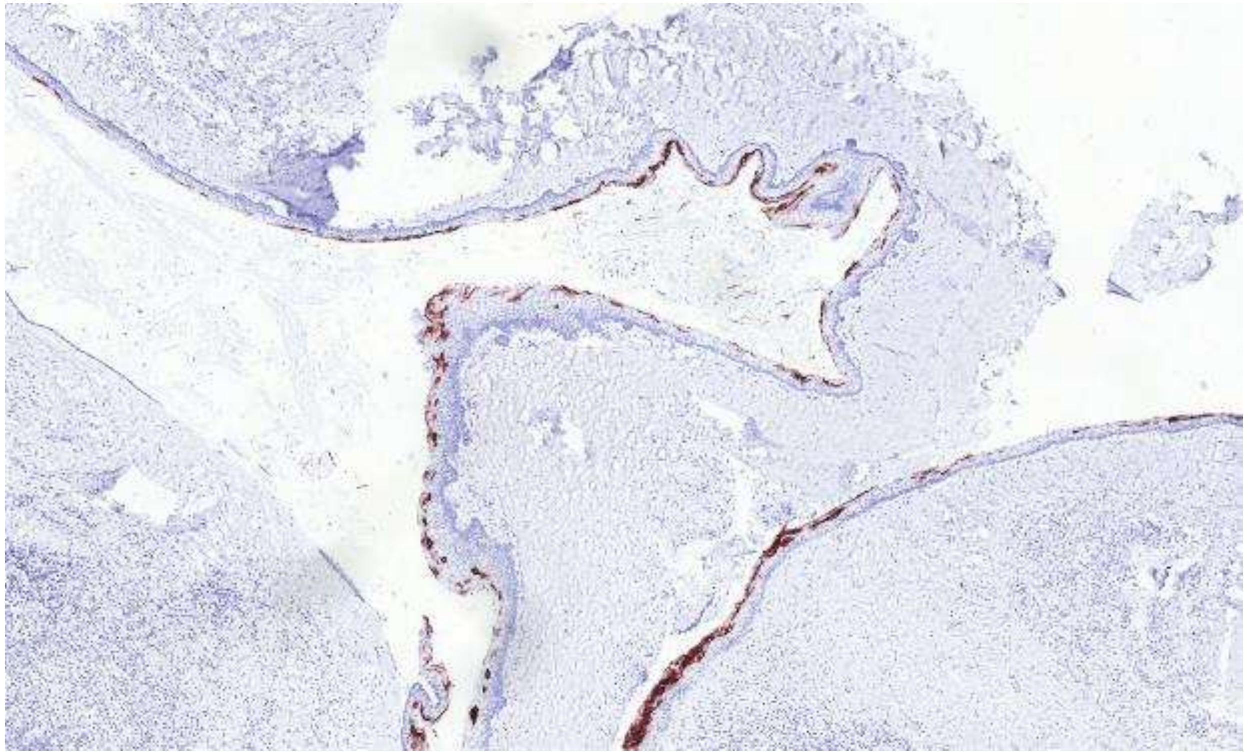

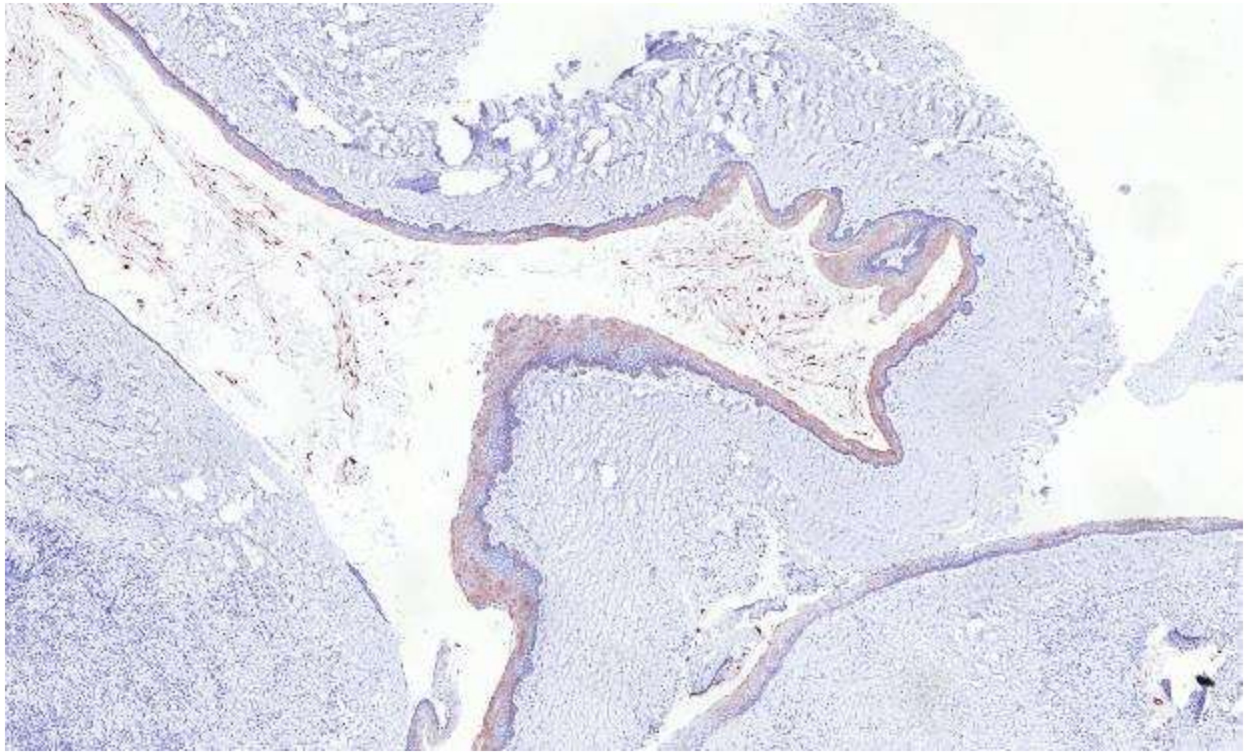

KR15 –
HE, CK17, bcl2 (x10)

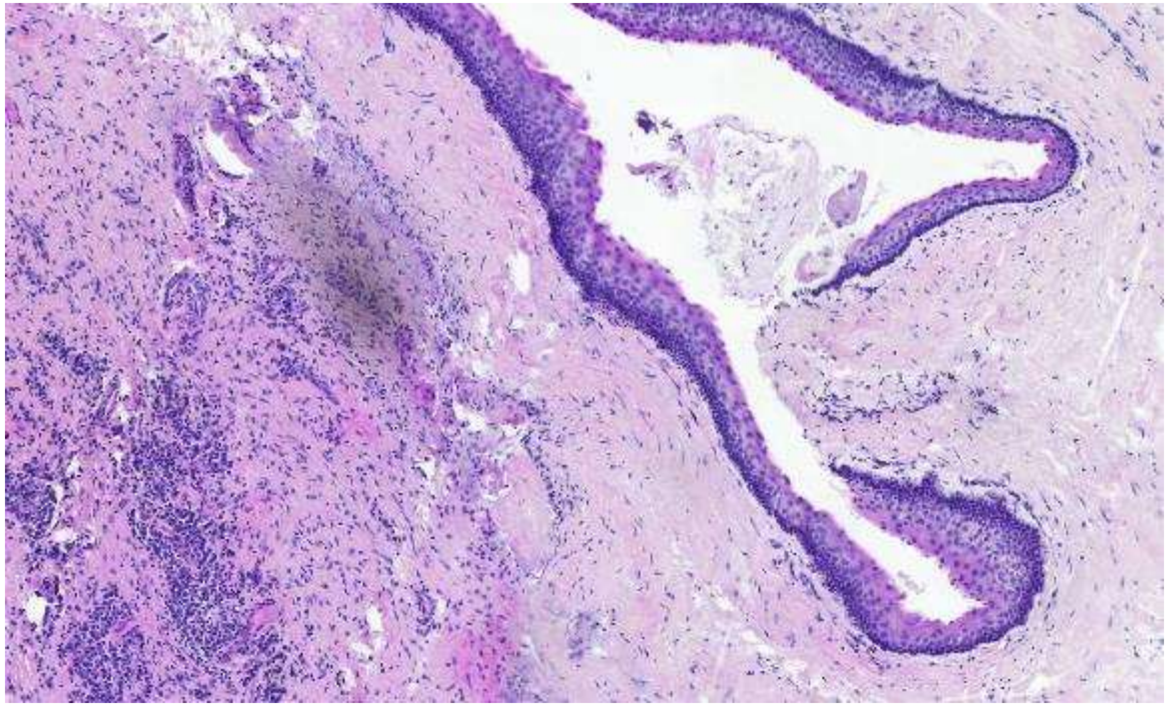

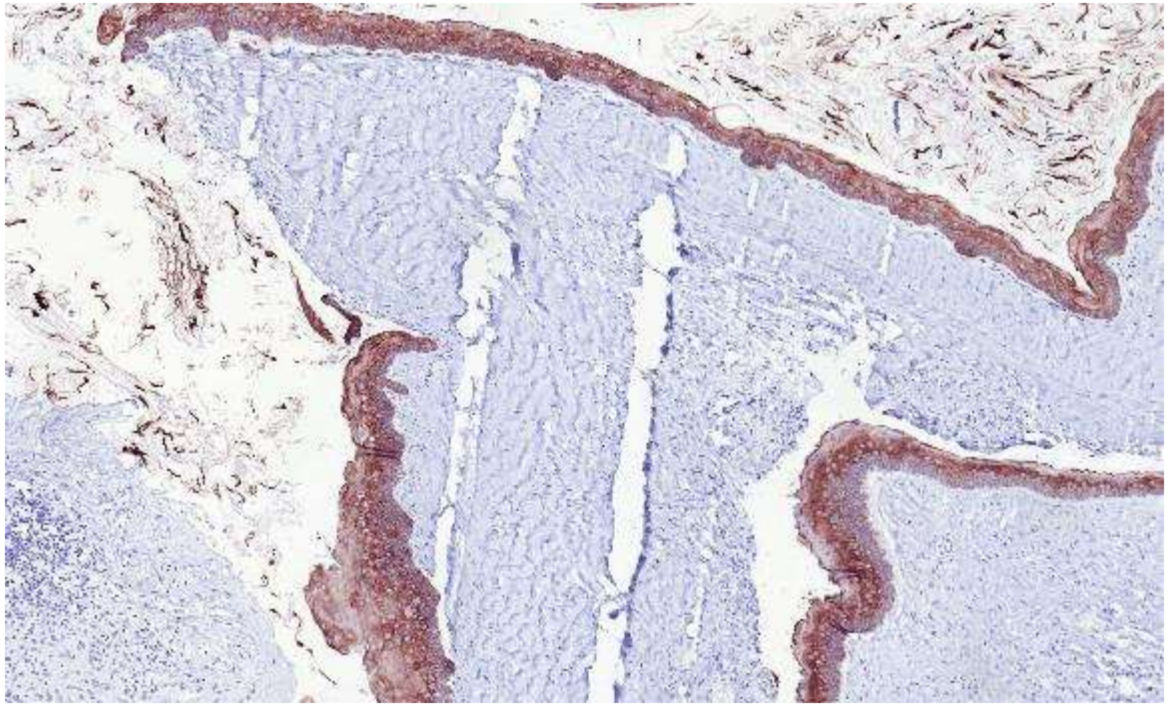

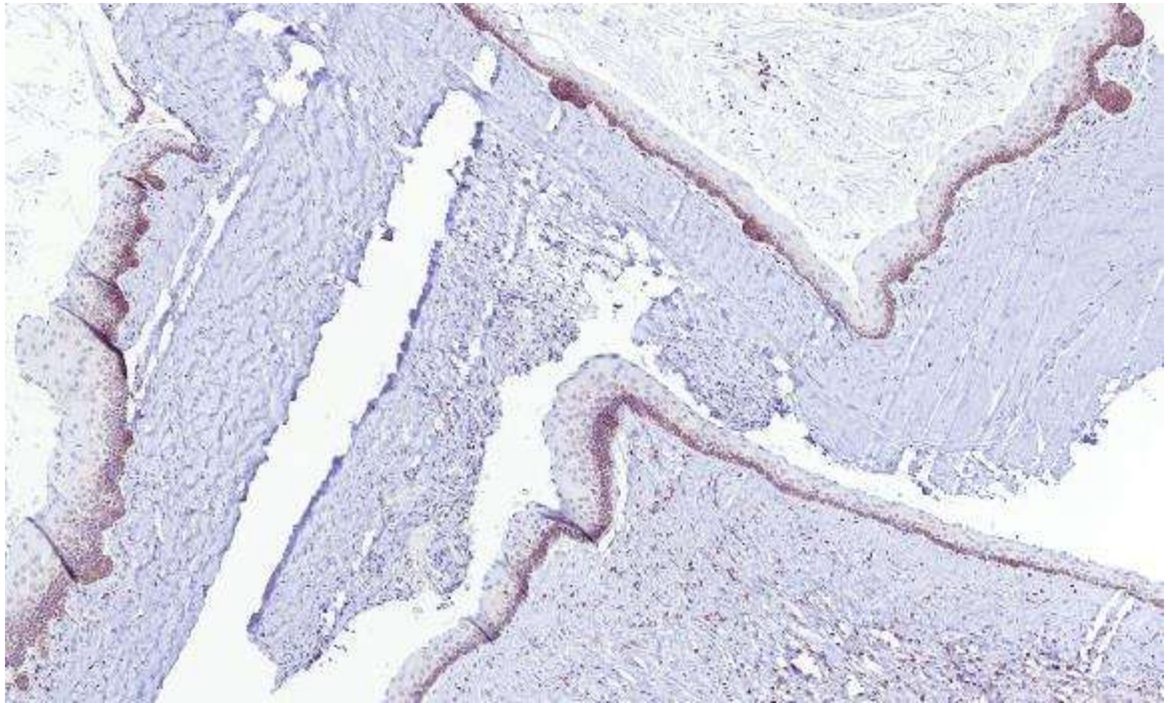

KR15 – CK10, CK19 (x10)

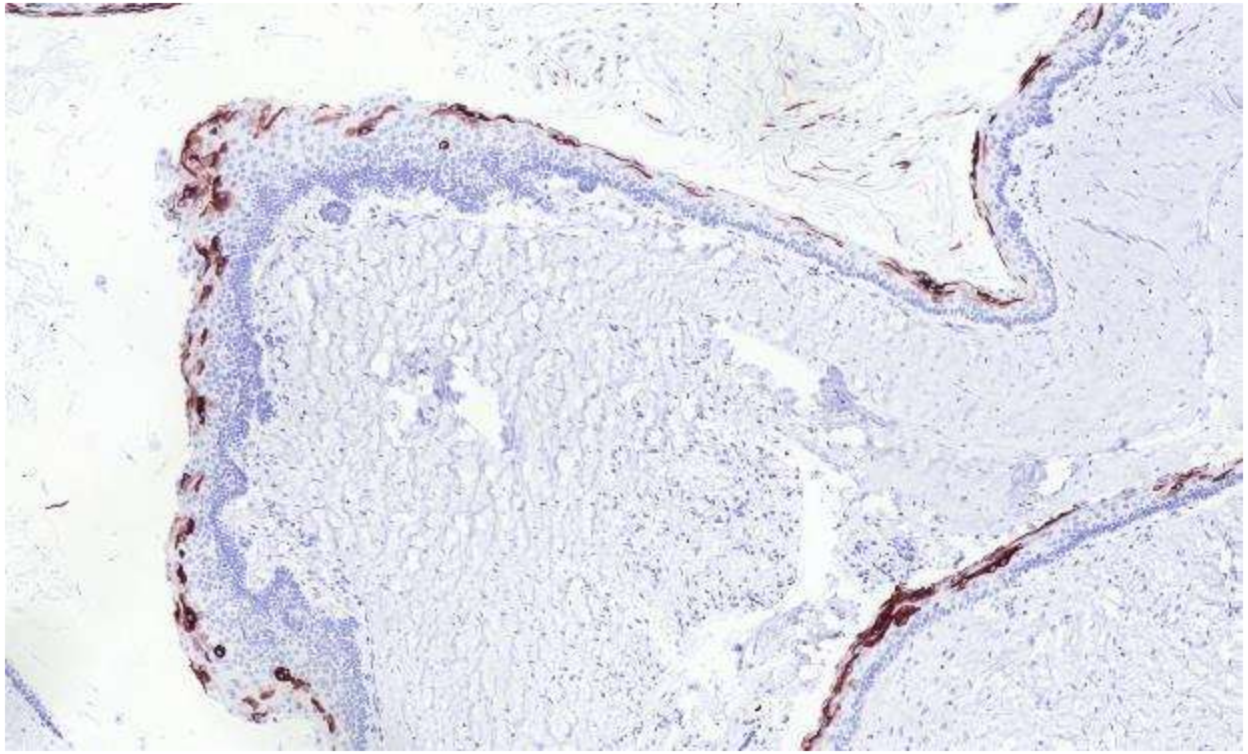

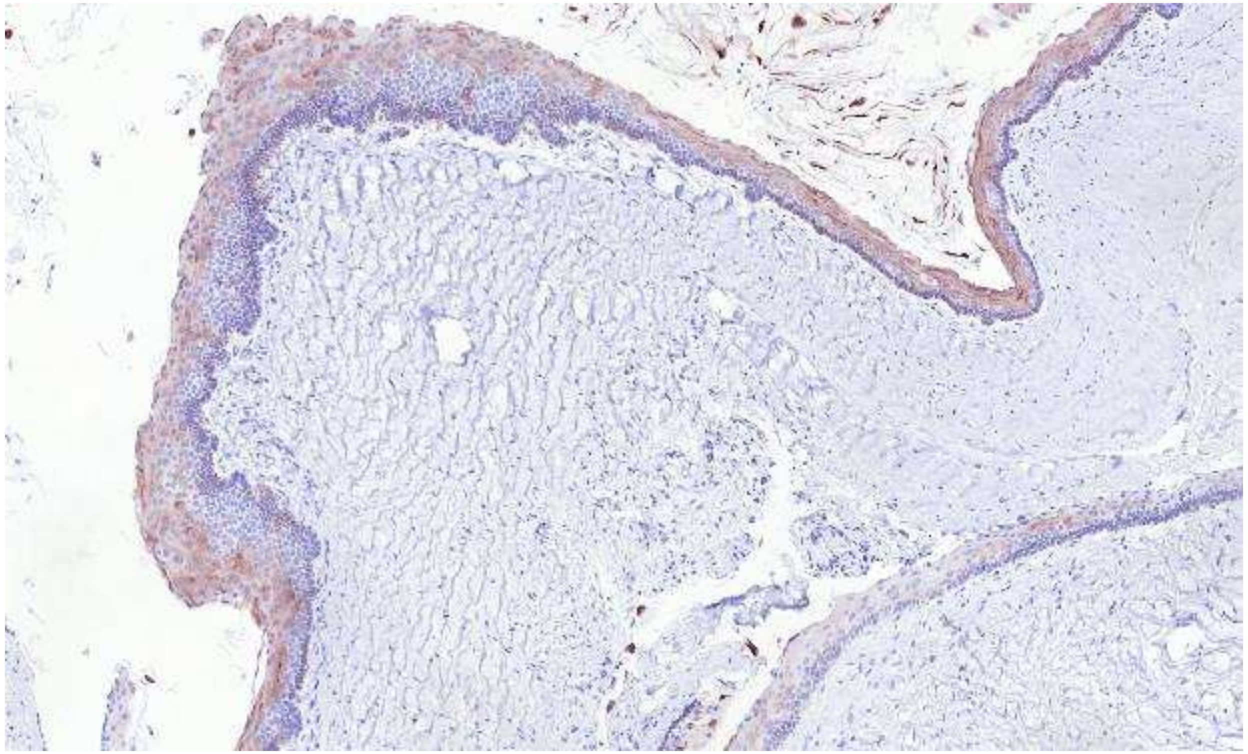

KR15 – CK10 (x10) – focal negativity

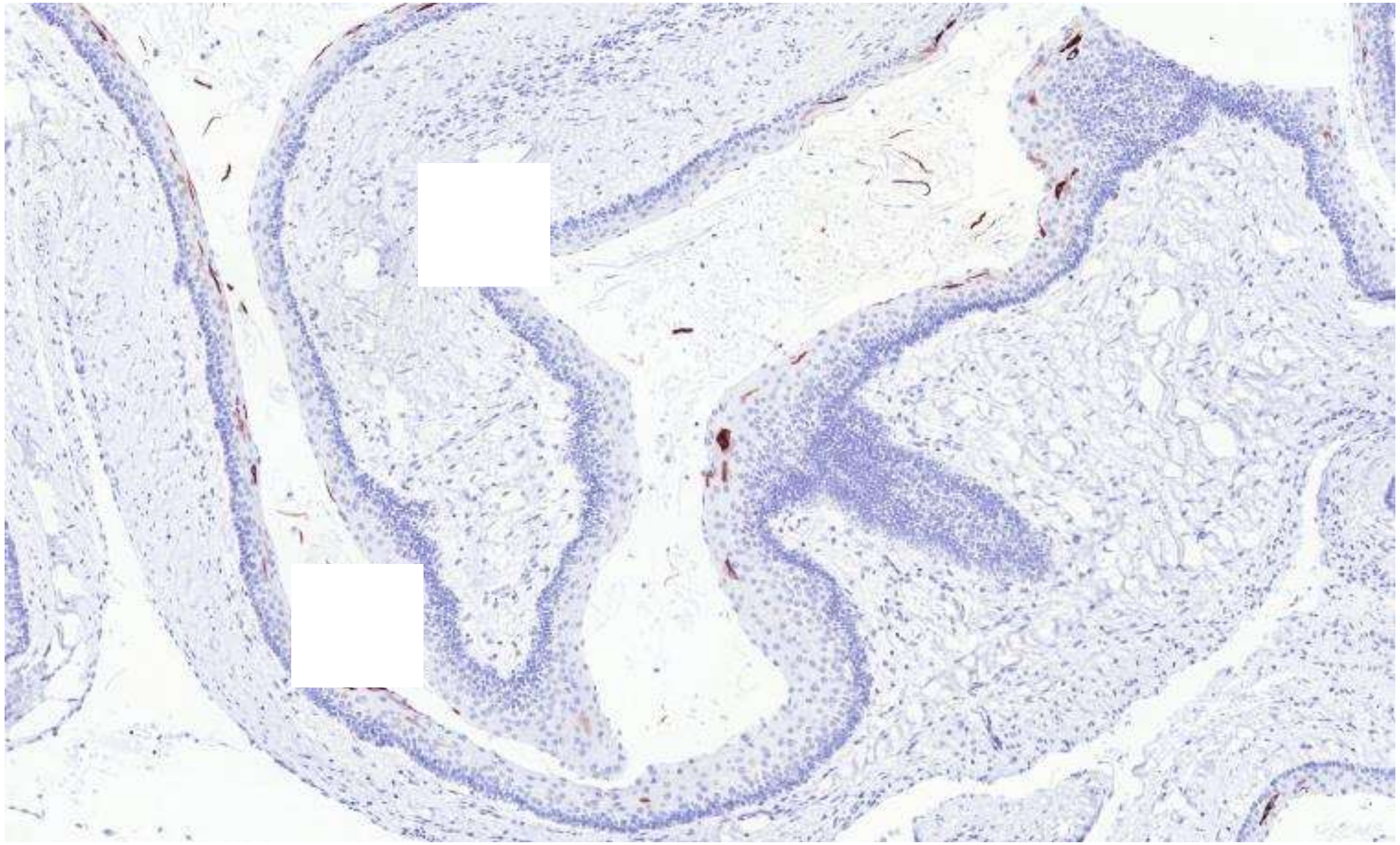

KR15 – Conclusion

- H&E: relatively thin squamous epithelium (5-7 cell layers) with slightly ticker areas, parakeratosis, wavy surface, obvious basal palisading, even epithelial-subepithelial contours – typical OKC morphology
- Focal chronic inflammation in the cyst wall
- Bcl2: diffuse, strong basal / suprabasal staining visible at low power
- CK17: diffuse, strong transepithelial staining
- CK10: dominant superficial staining with focal negative areas
- CK19: dominant transepithelial staining
- Histopathology: odontogenic keratocyst (OKC)
- Combined clinical and pathological opinion: OKC (of the maxillary sinus)

Case KR16

- 47-year-old female
- Dental hygiene/status: partly conserved, partly missing, treated teeth
- Radiology: incomplete root filling of tooth 12, periapical radiolucency, buccal fistula
- Operation: removal of the cyst and apex of root 12.
- Clinical diagnosis: periapical cyst

KR16 – OPT

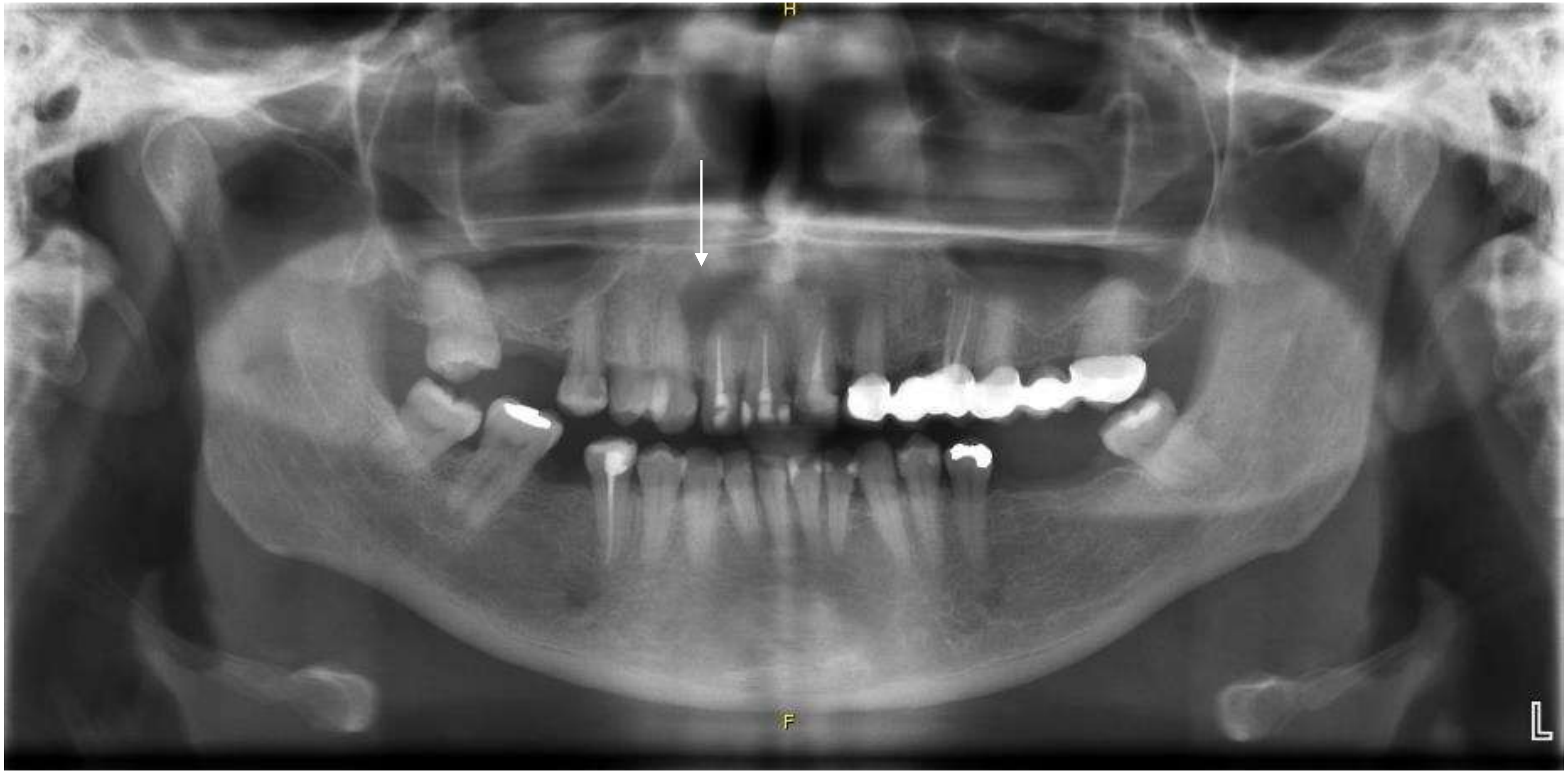

KR16 – HE, CK17, bcl2 (x1.3)

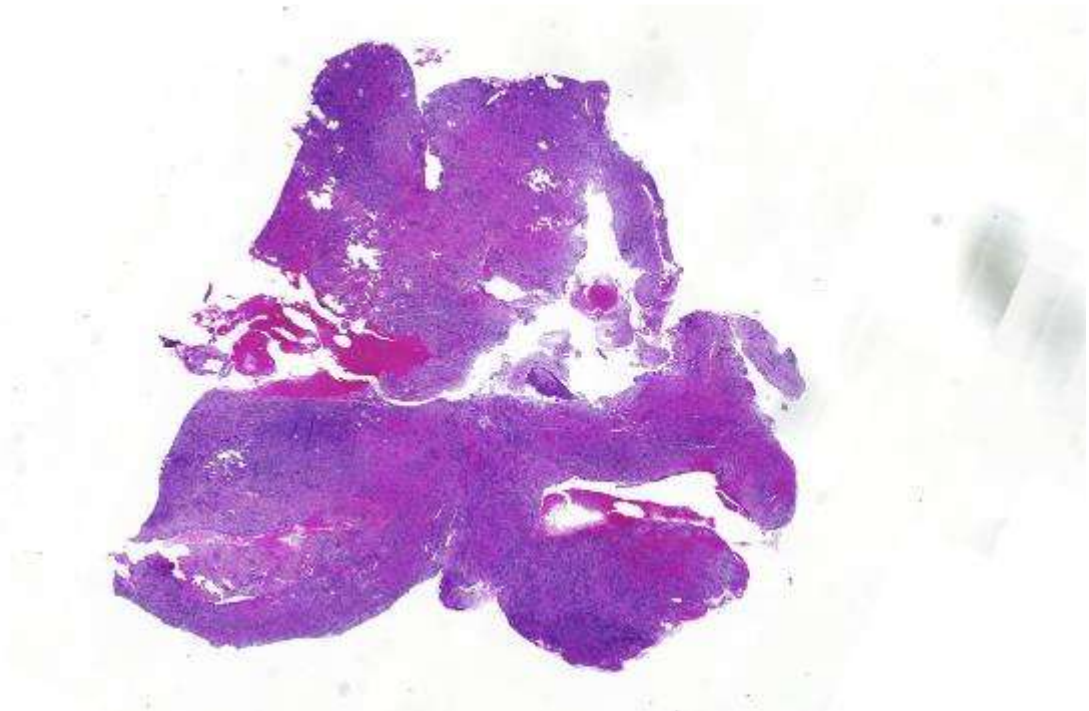

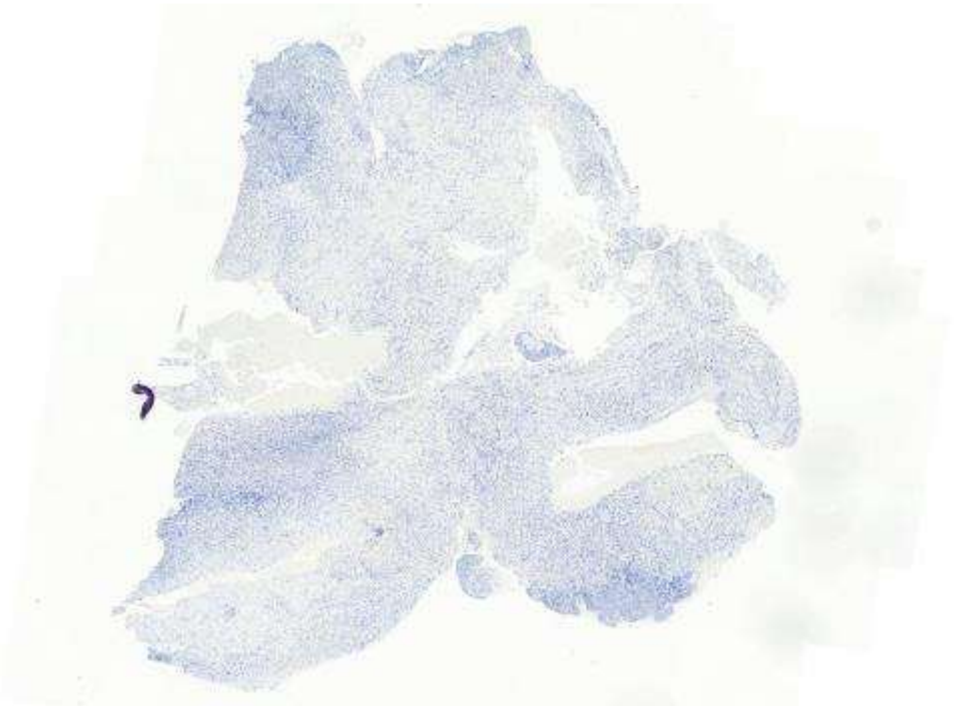

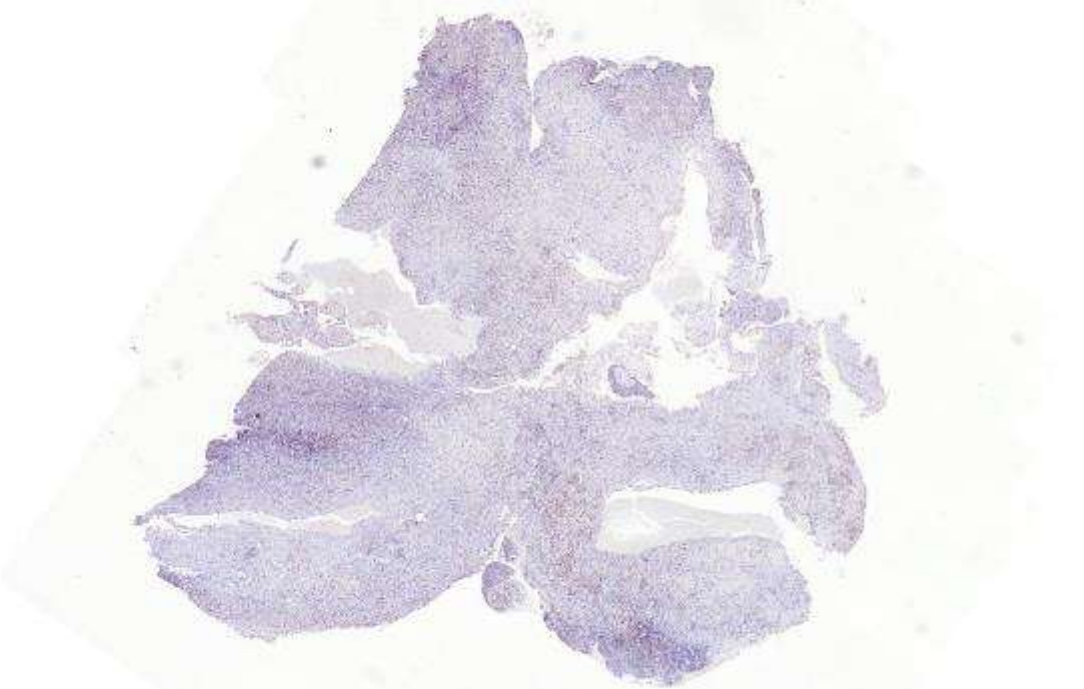

KR16 – HE, CK17, bcl2 (x5)

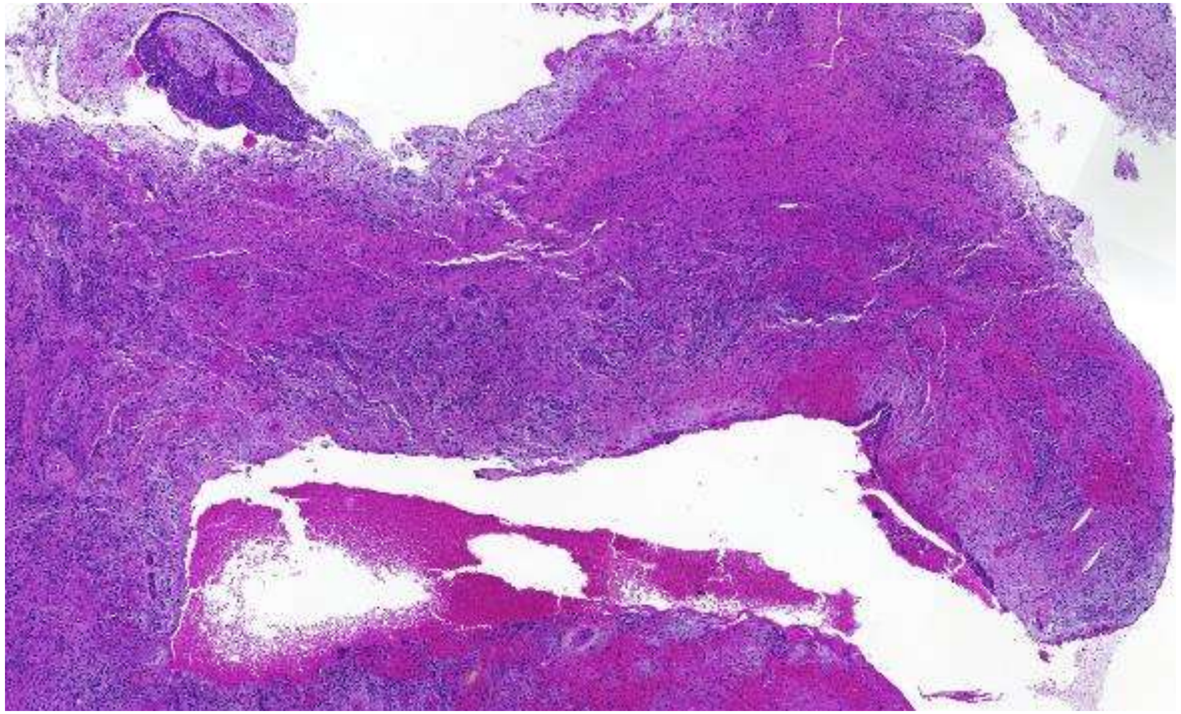

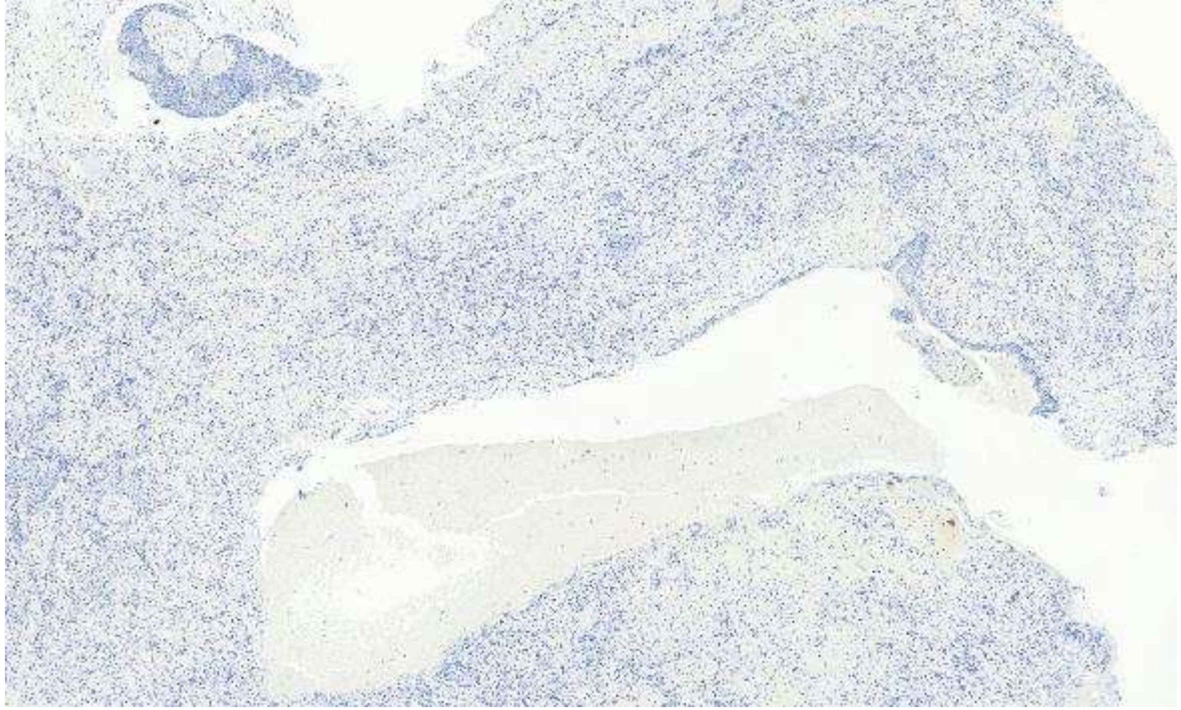

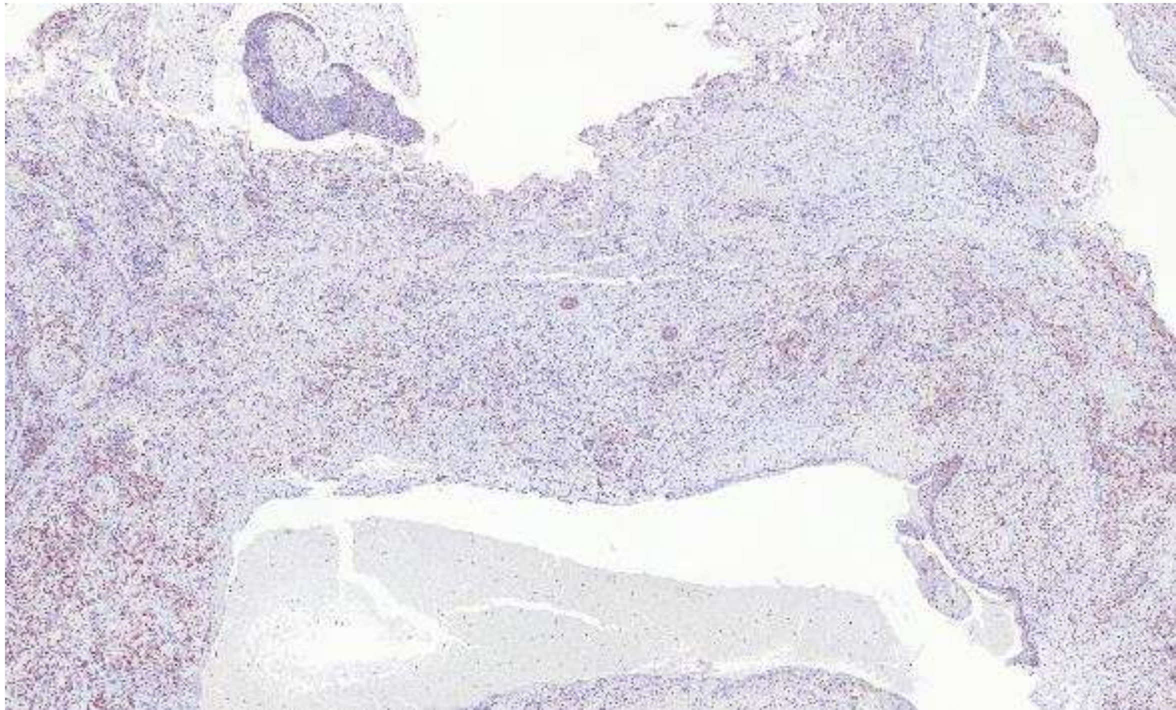

KR16 – Conclusion

- H&E: vastly denuded areas with fibrinoid exsudate on the inflamed connective tissue base; relatively thin squamous epithelium with focal thickening, no parakeratosis, no wavy surface, no basal palisading, even epithelial-subepithelial contours
- Intense mixed inflammatory infiltration of the cyst wall
- Bcl2: negative
- CK17: negative
- Histopathology: radicular cyst
- Combined clinical and pathological opinion: radicular cyst

Case KR17

- 32-year-old male
- Dental hygiene: moderate, less than perfect; partly missing teeth.
- History: Had complaints related to tooth 38 which was removed 6 years ago and the symptoms ceased. 1.5-2 years later a swelling of the area and face occurred and he noted some discharge from the area, but the problem self-solved. 1-2 years ago similar symptoms were noted, 9 days ago he noted paraesthesia of the left face, lip, mentum, and tongue.
- Radiology: circumscribed radiolucent lesion of 40x20 mm in size in the left angle/ramus region of the mandible. On CT, the destruction of the cortical bone can be appreciated.
- Operation: removal of cyst (in two pieces) with its separation from the bony cavum
- Clinical diagnosis: residual cyst

KR17 – OPT

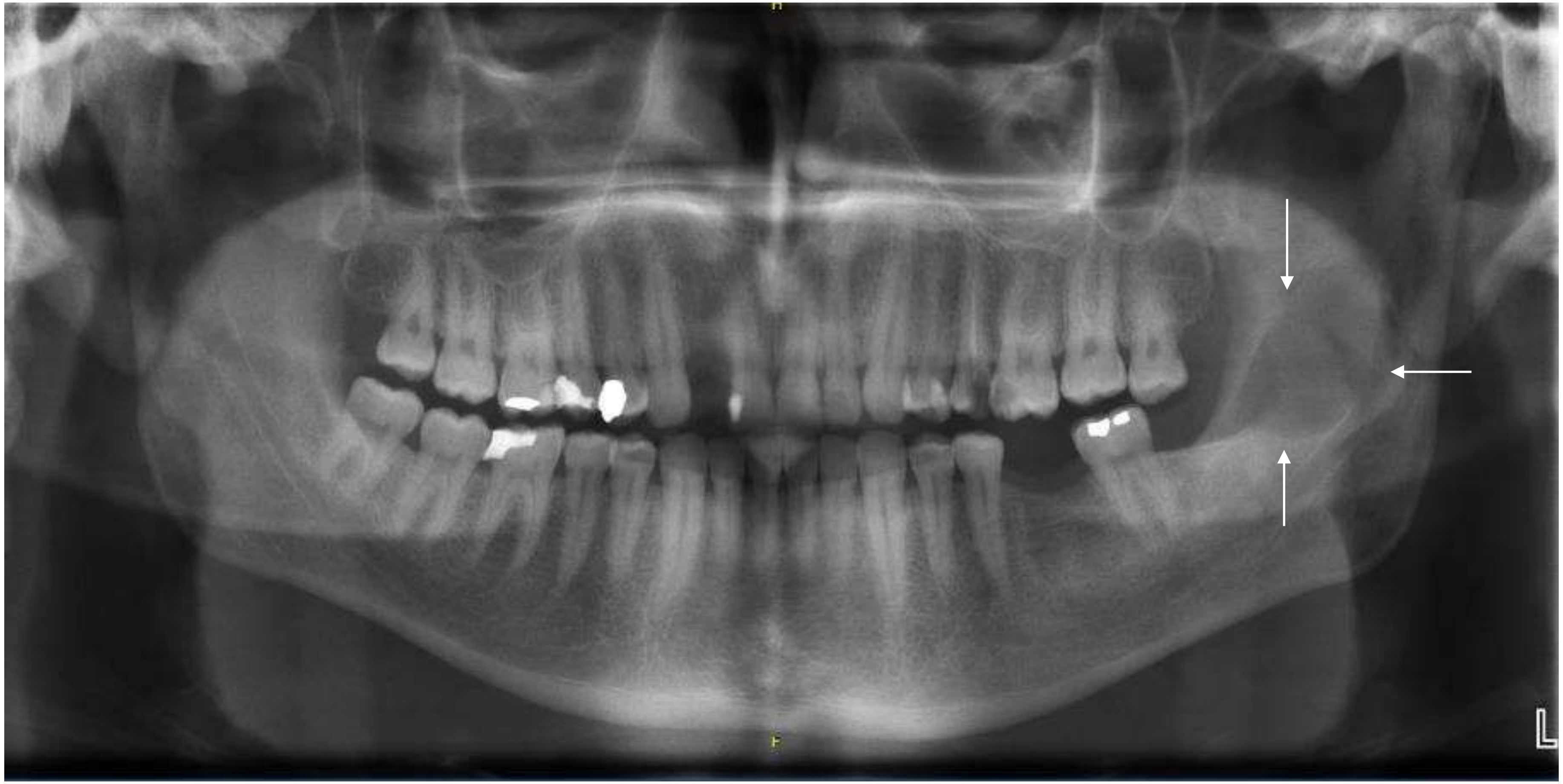

KR17 – CT coronal

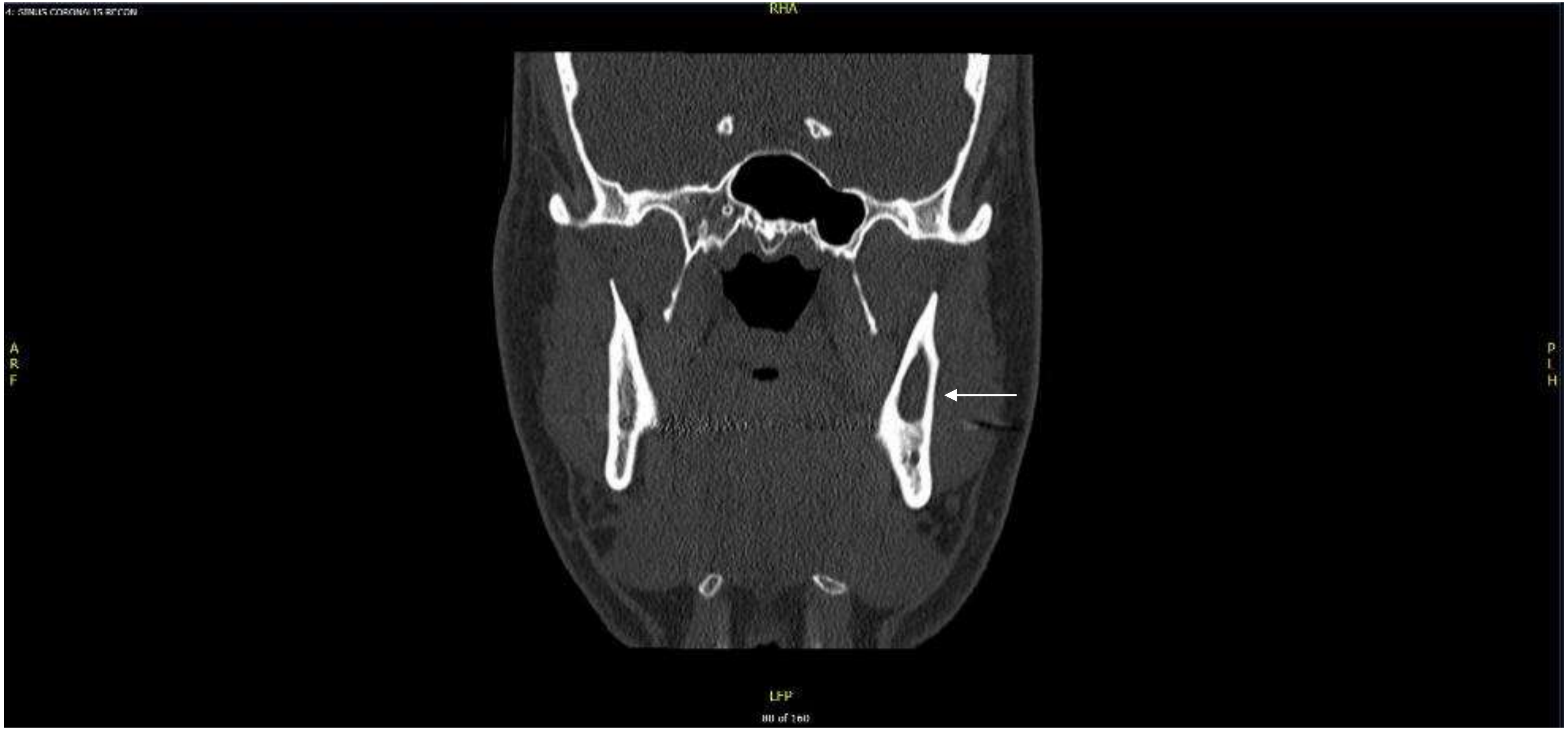

KR17 –
HE, CK17, bcl2 (x0.4)

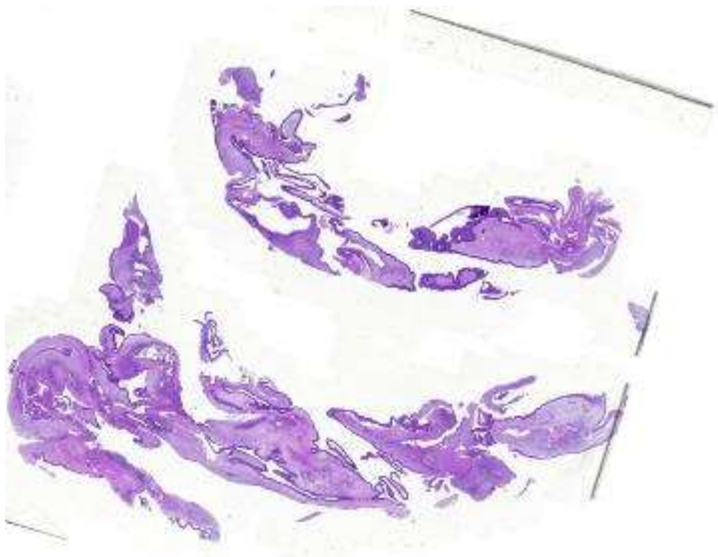

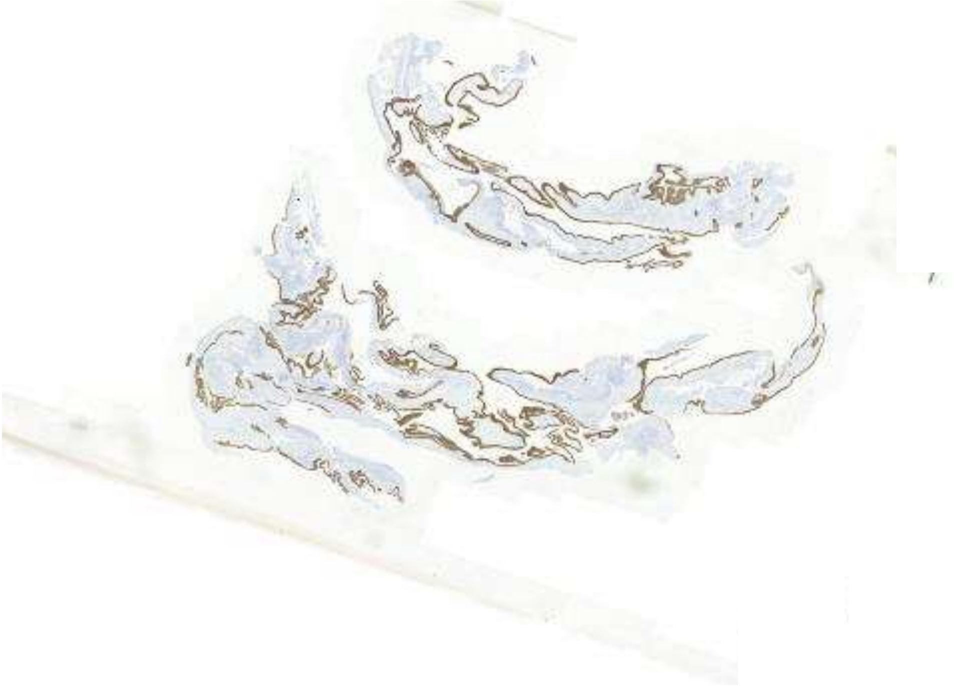

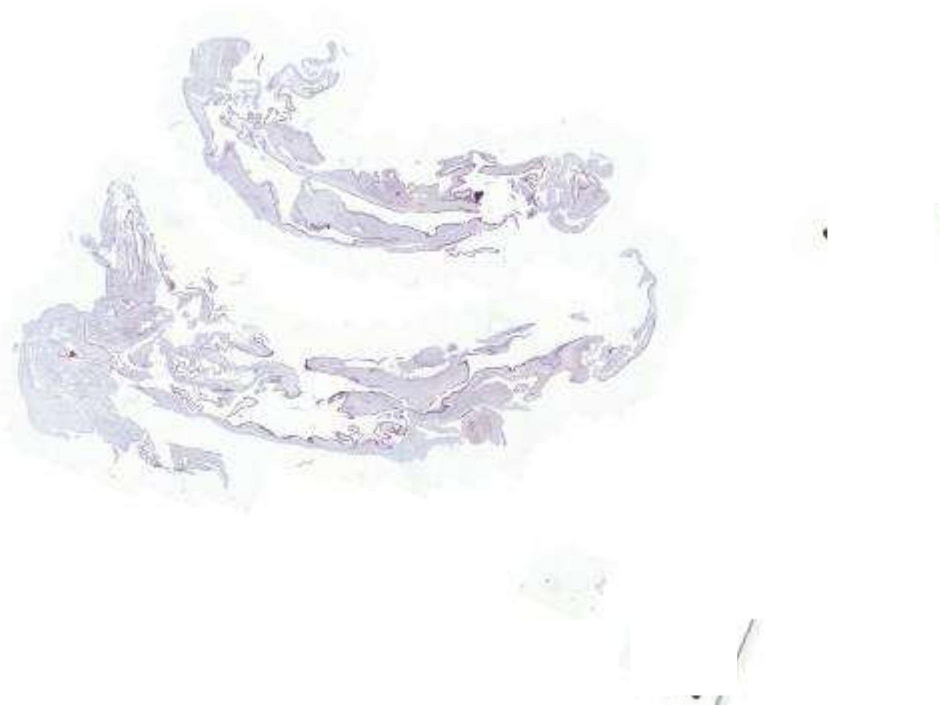

KR17 – CK10, CK19 (x0.4)

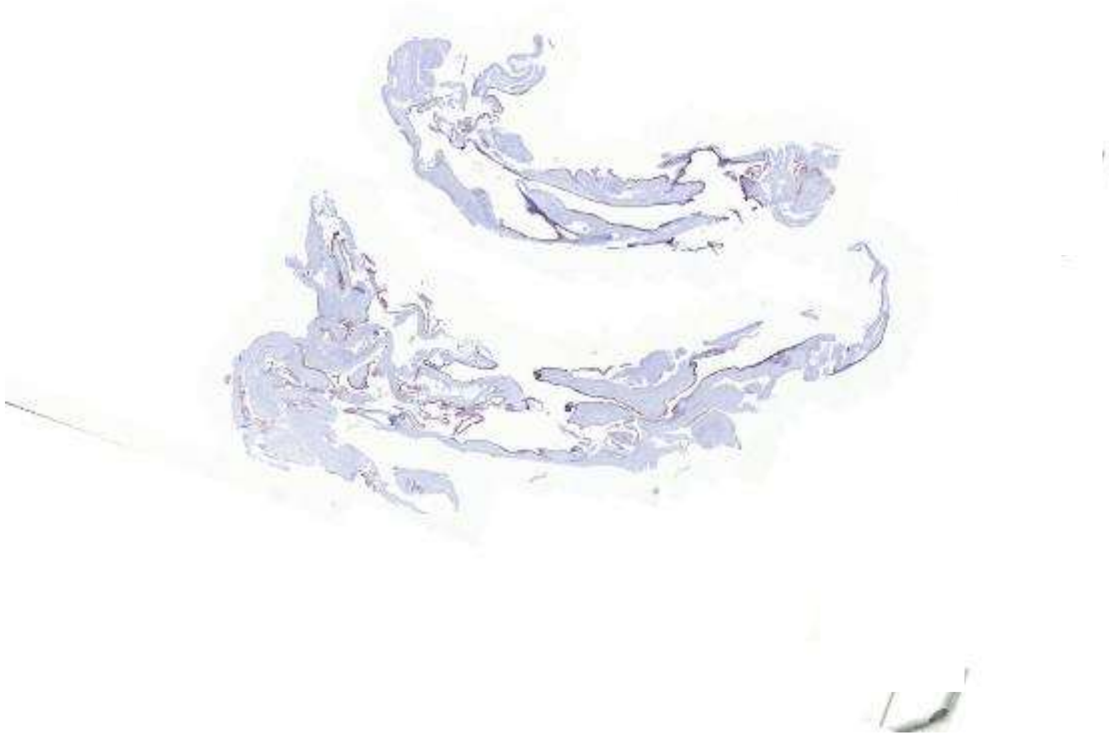

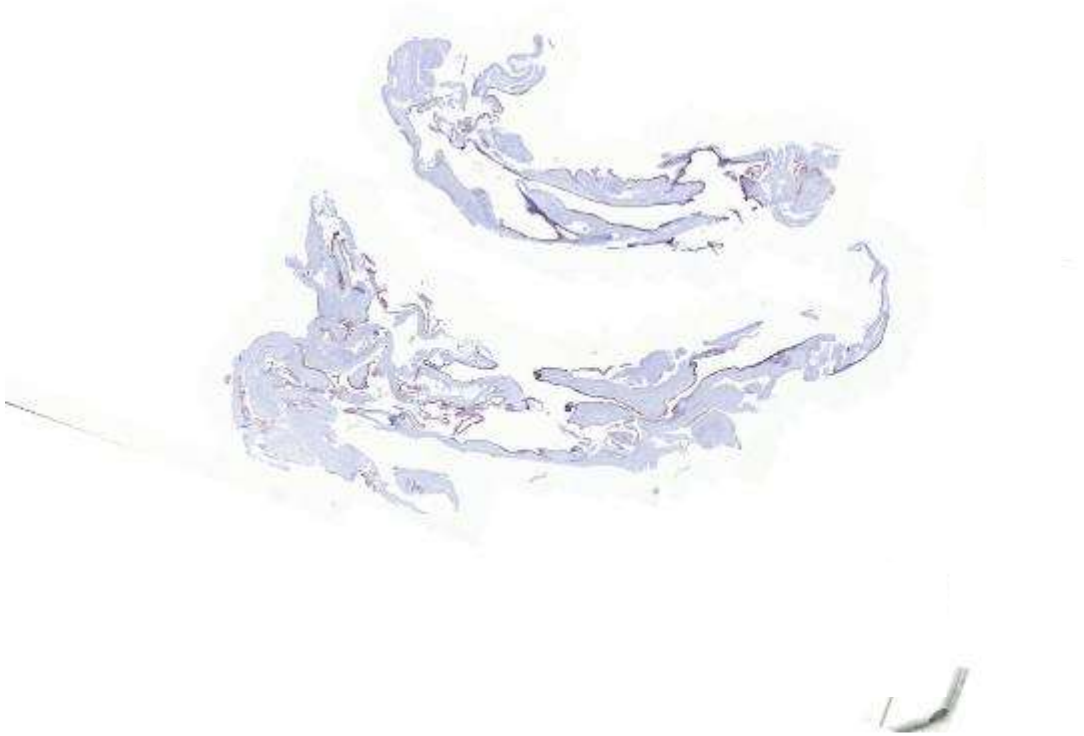

KR17 –
HE, CK17, bcl2 (x5)

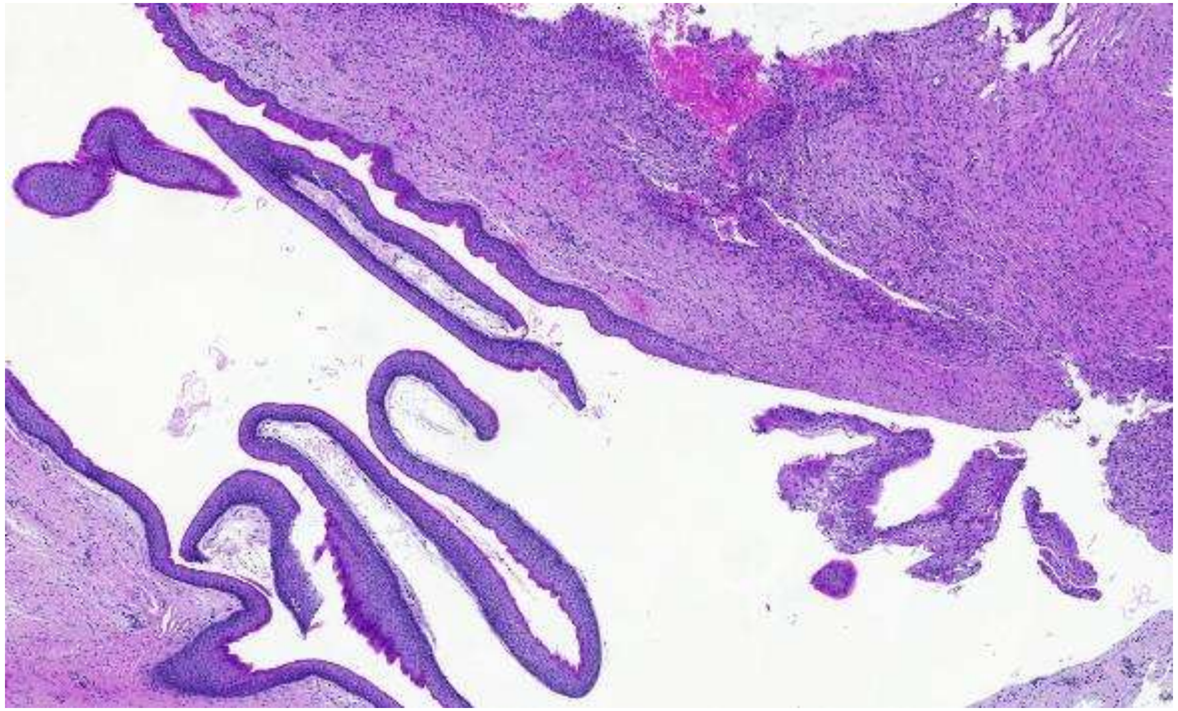

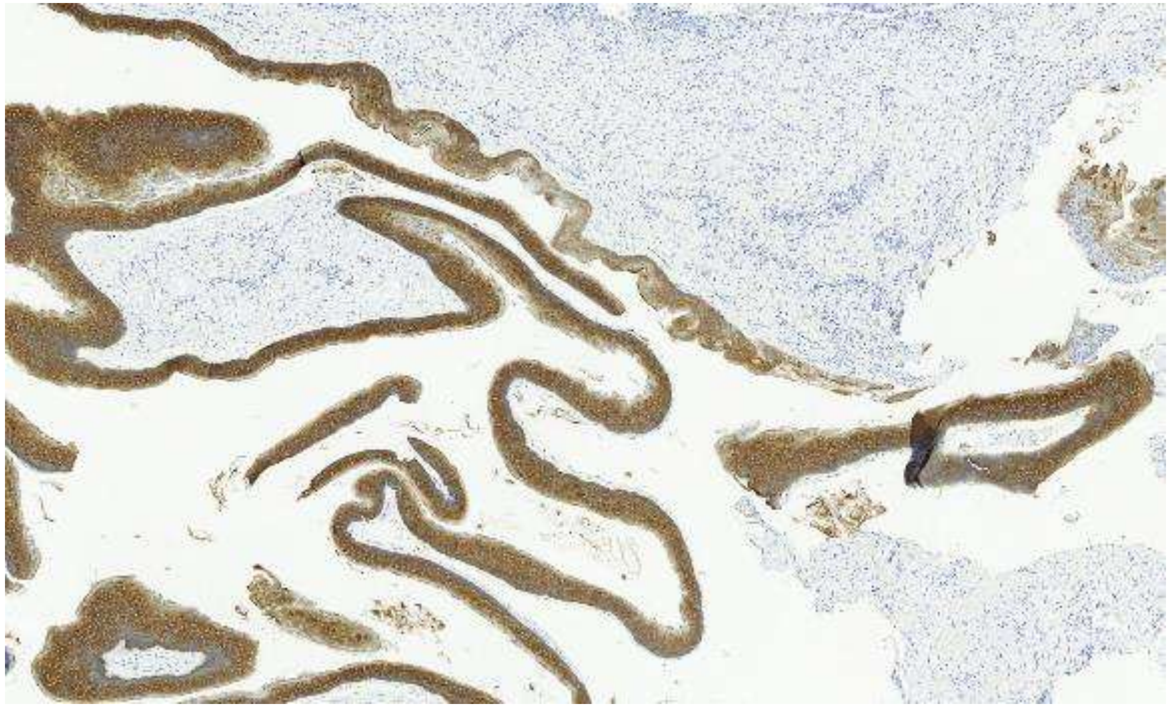

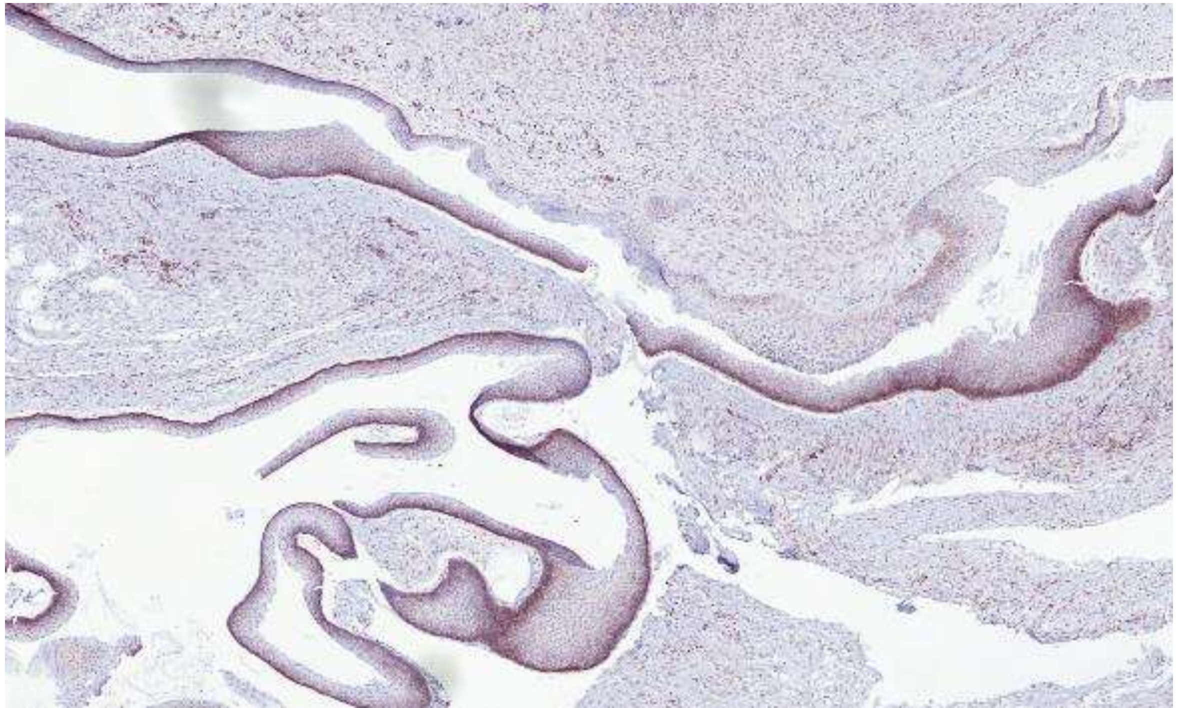

KR17 – CK10, CK19 (x5)

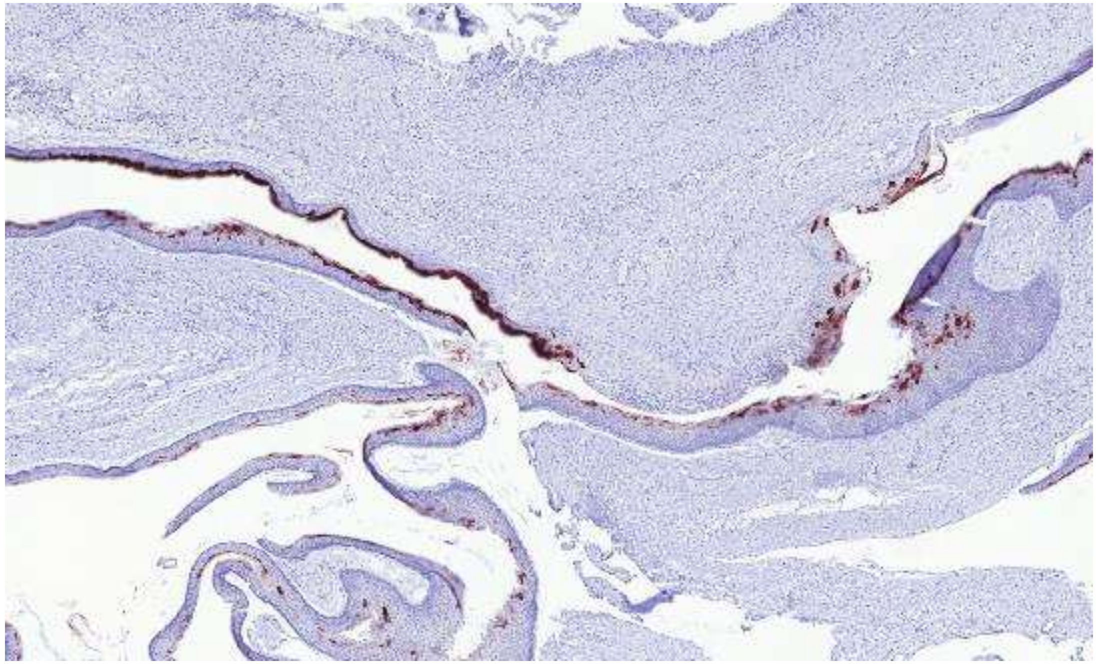

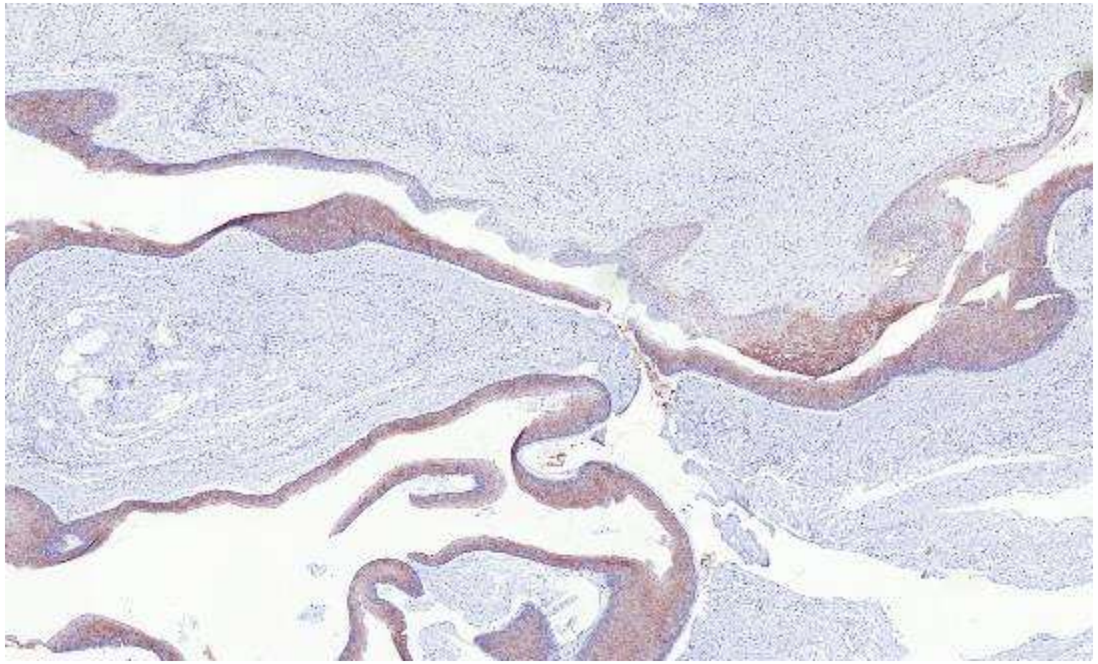

KR17 –
HE, CK17, bcl2 (x20)

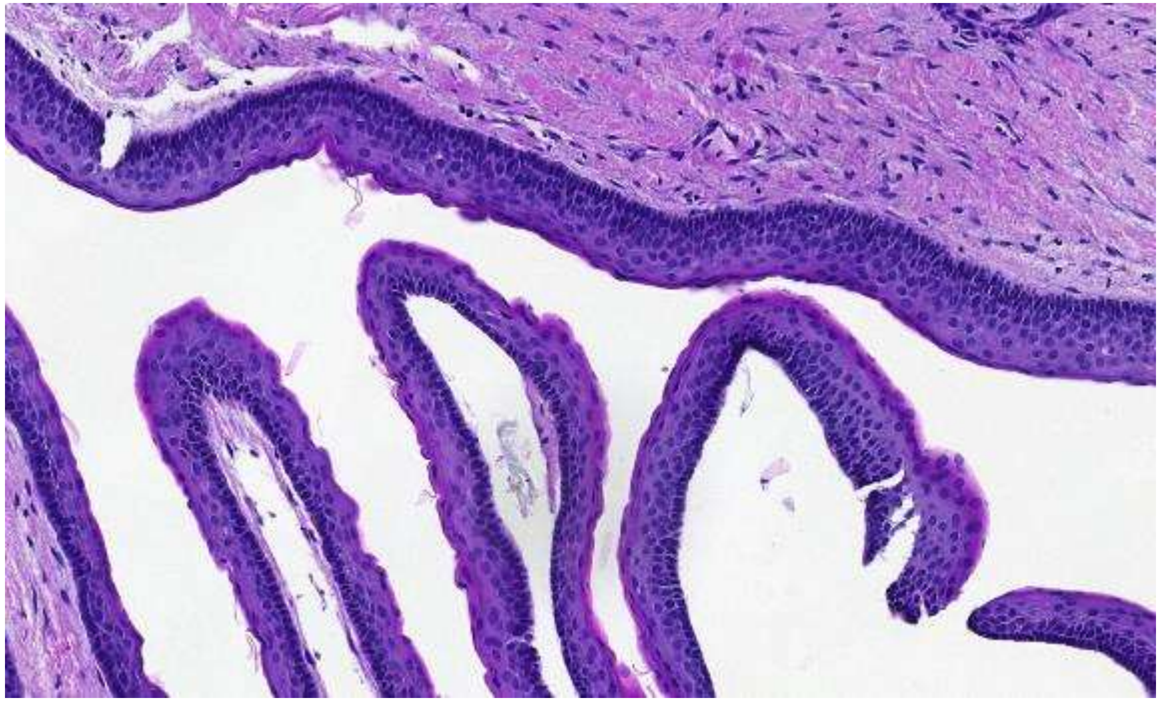

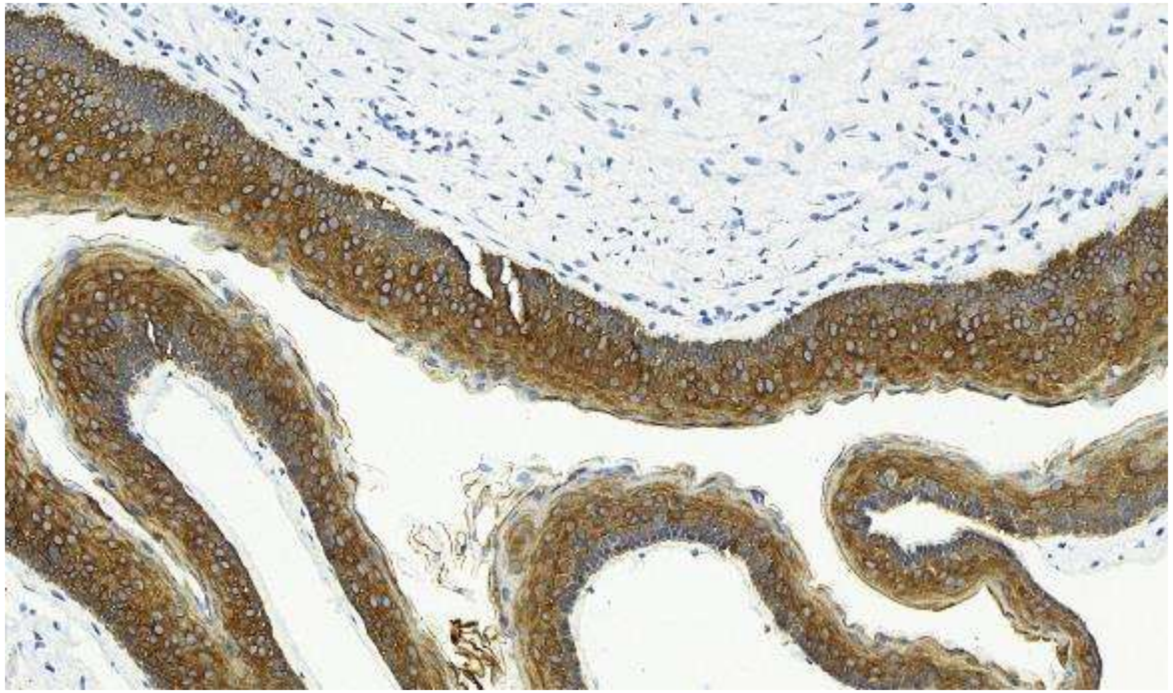

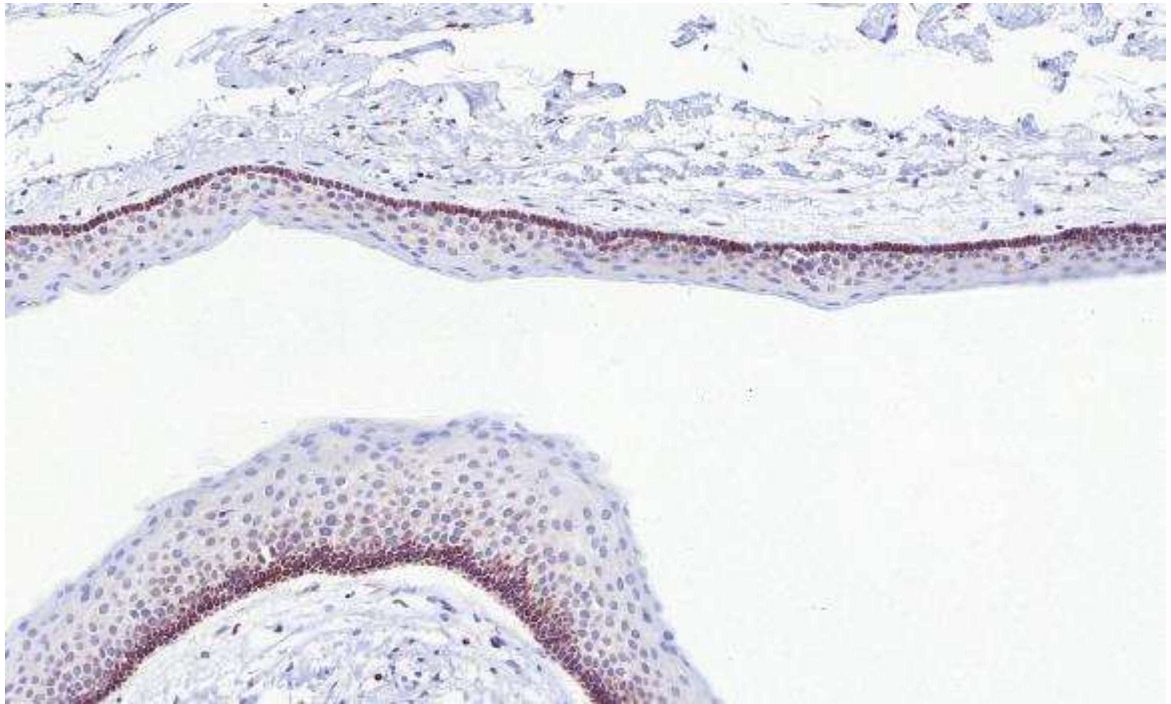

KR17 – CK10, CK19 (x20)

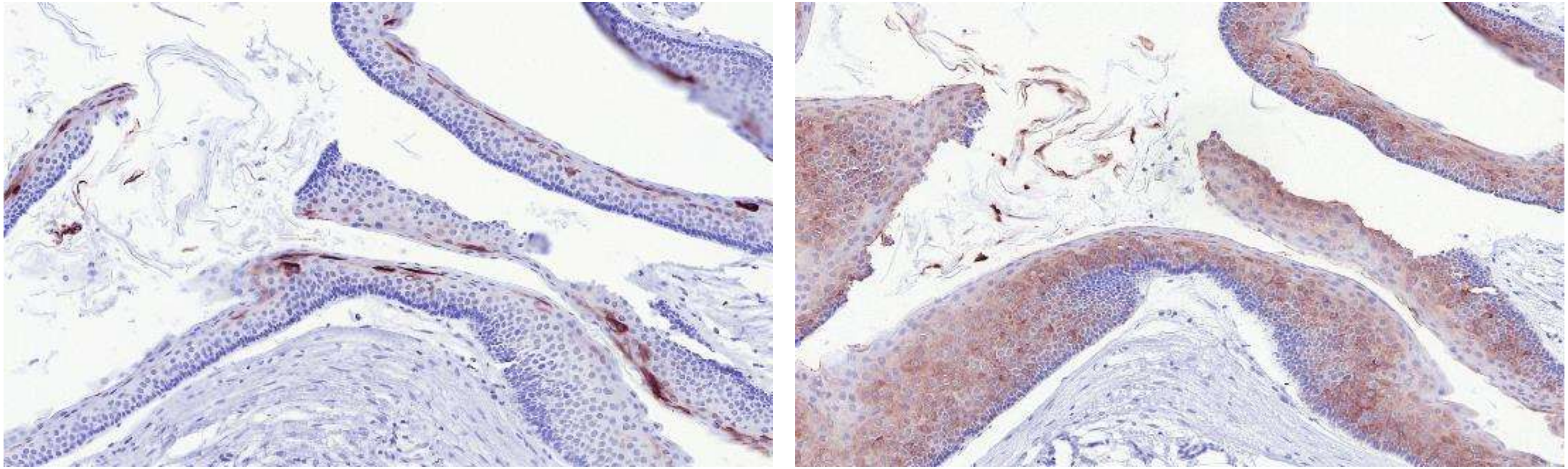

KR17 – CK10 (x7), CK19 (x10)
Areas with basal palisading without staining (←)

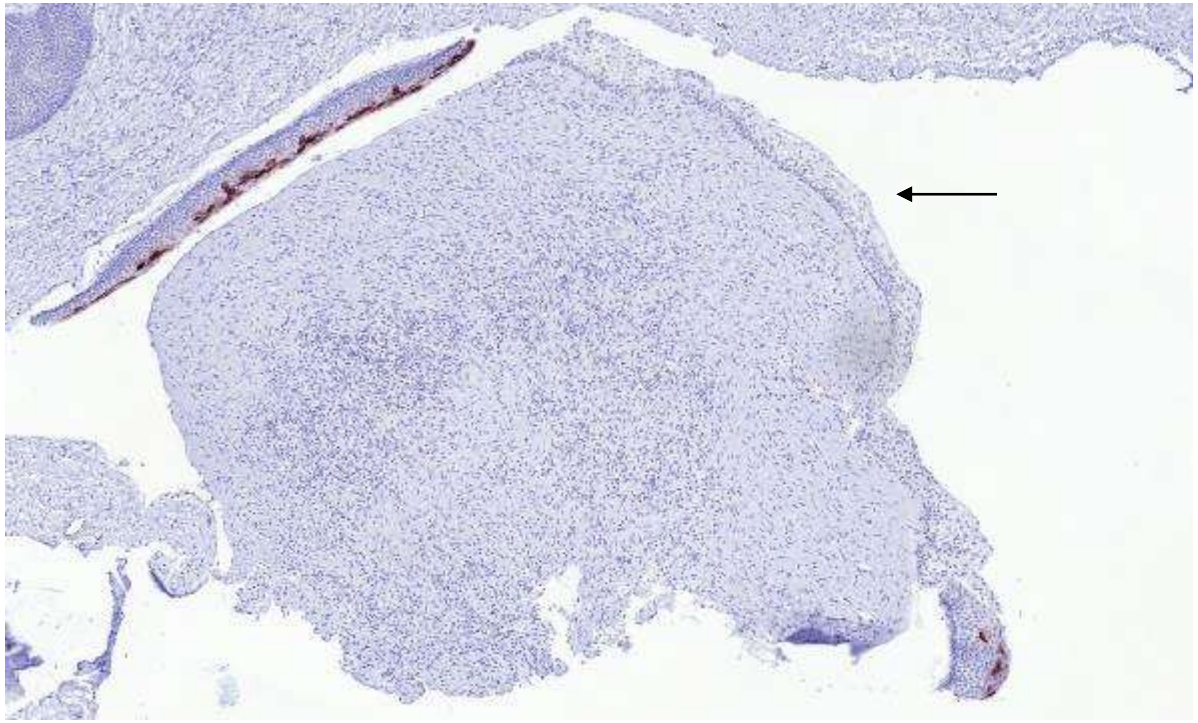

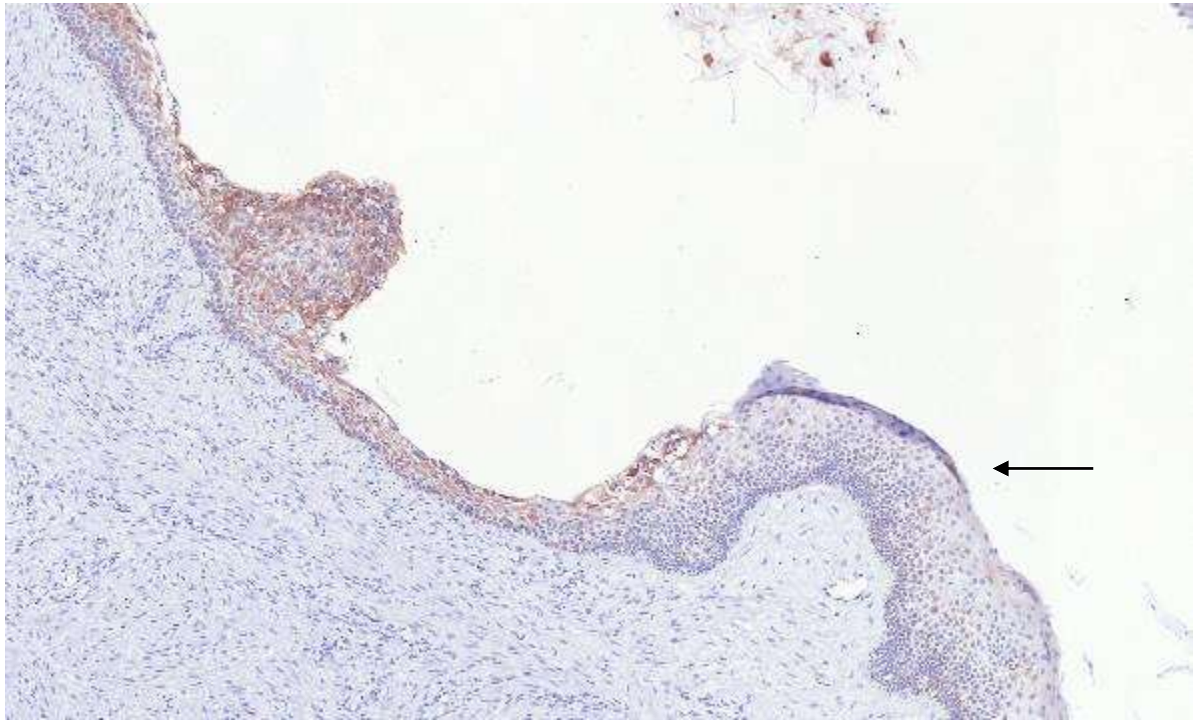

KR17 – Conclusion

- H&E: relatively thin squamous epithelium (5-7 cell layers) with slightly ticker areas, parakeratosis, wavy surface, obvious basal palisading, even epithelial-subepithelial contours – typical OKC morphology
- Focal inflammation in the cyst wall
- Bcl2: diffuse, strong basal / suprabasal staining visible at low power
- CK17: diffuse, strong transepithelial staining
- CK10: dominant superficial staining with focal negative areas
- CK19: dominant transepithelial staining with focal negative areas
- Histopathology: odontogenic keratocyst (OKC)
- Combined clinical and pathological opinion: OKC

Case KR18

- 66-year-old male
- Dental hygiene/status: total lack of teeth in the upper quadrants and subtotal lack in the lower ones. Teeth 35 and 34 united with a metal bridge, II. degree mobility, vitality could not be assessed; tooth 38 impacted in horizontal position
- Radiology: „well circumscribed radiolucent area involving the crown of tooth 38 and partly resorbed root of tooth 35" – Note: resorption of the root of tooth 35, buccal growth, double contour on OPT
- Operation: removal of the cyst together with tooth 38, and removal of teeth 35 and 34.
- Clinical diagnosis: radicular cyst

KR18 – OPT

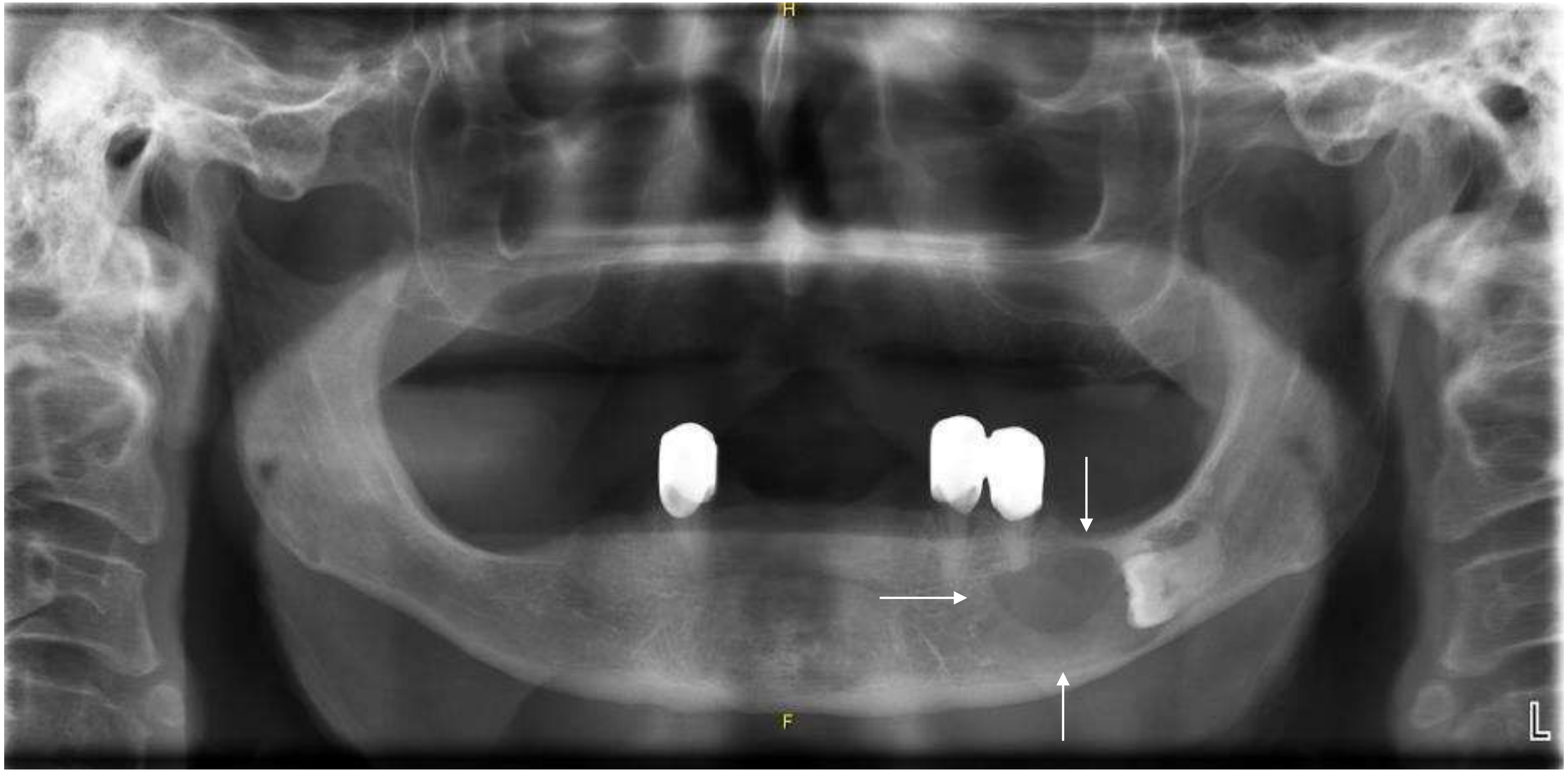

KR18 – OPT magnified - Note: resorption of the root of tooth 35 & the double contour

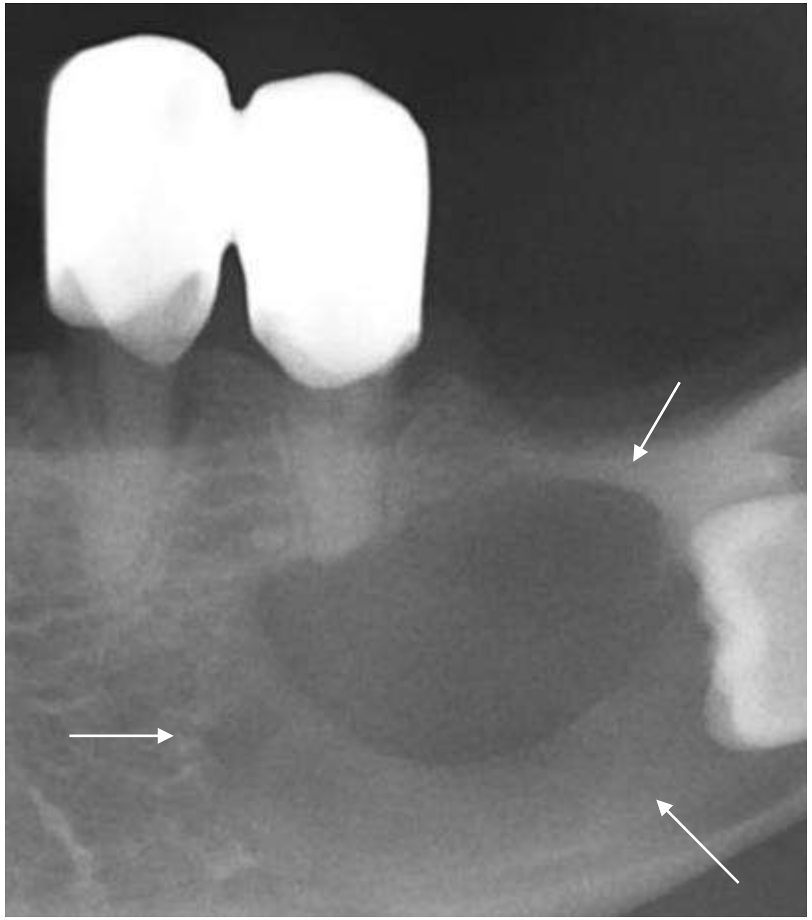

KR18 – CT axial (114-112-110-106/130 slices)

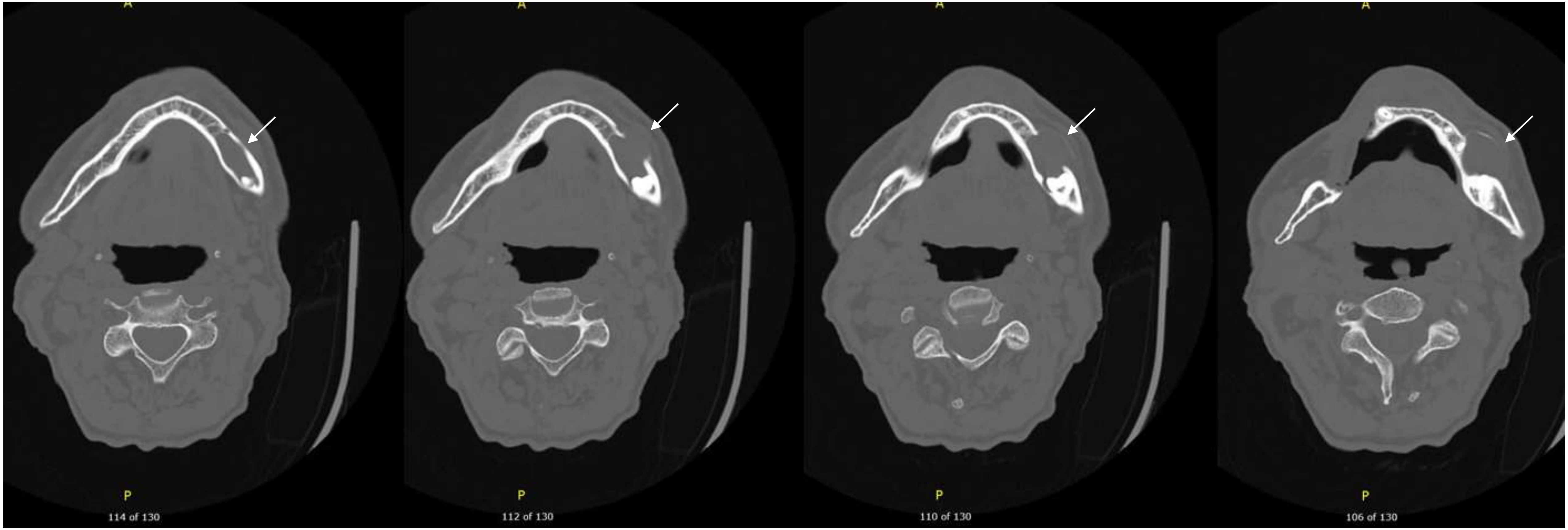

KR18 – CT coronal (18-19-20-22/67 slices)

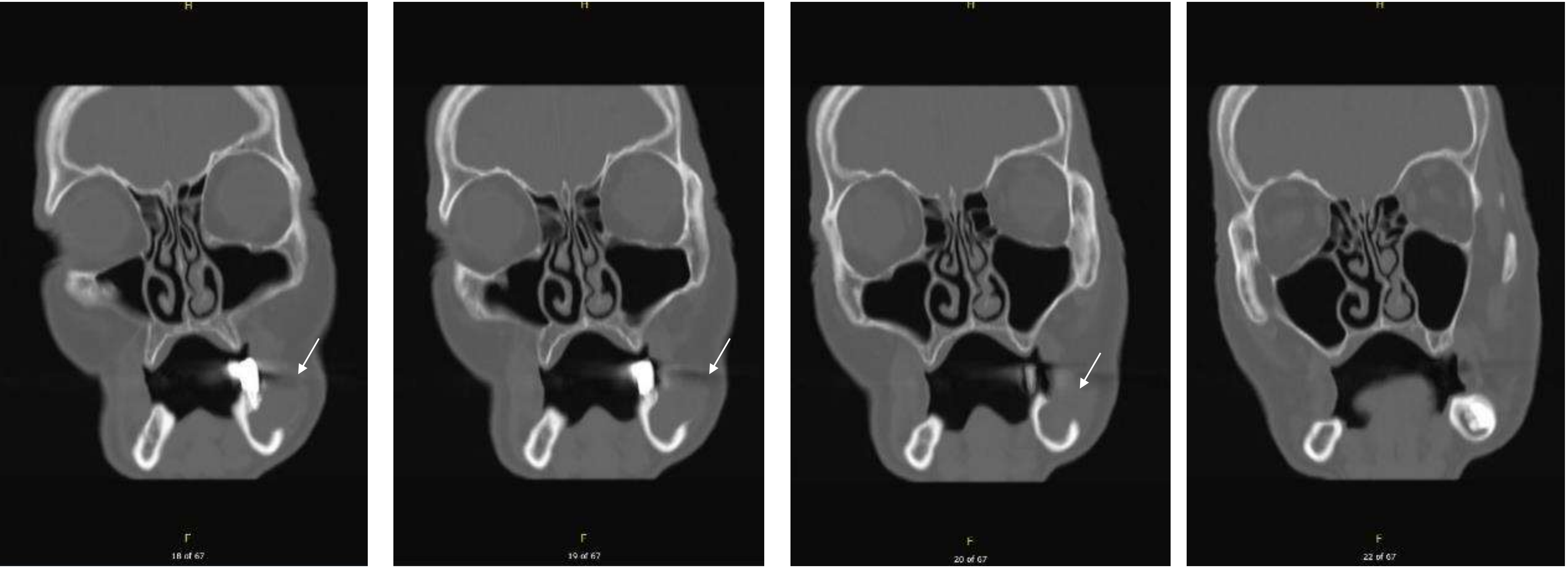

KR18 – CT sagittal (17-19-20/57 slices)

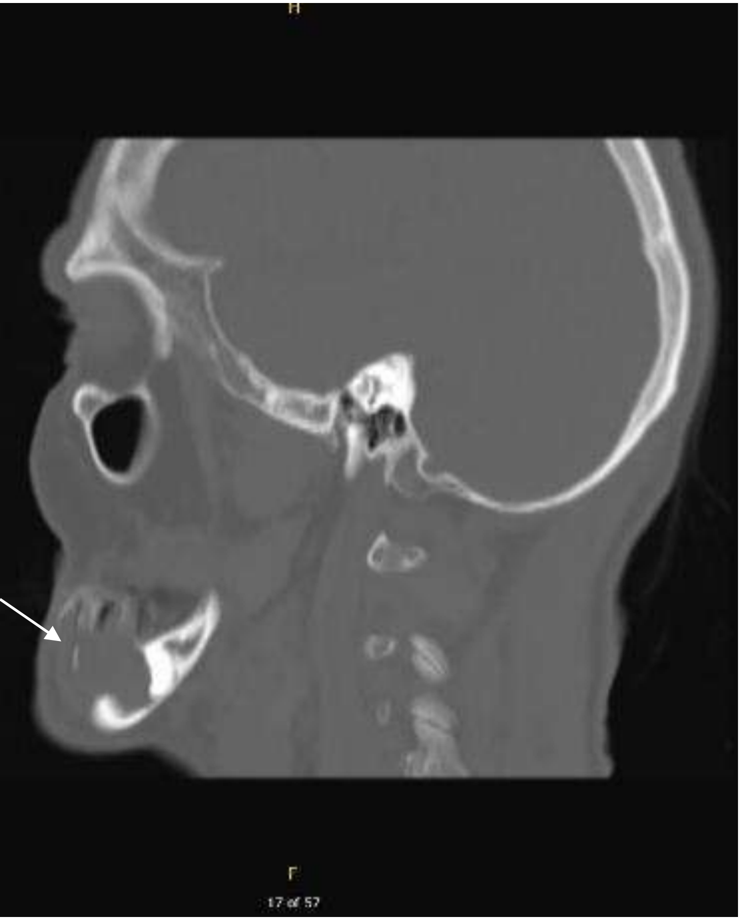

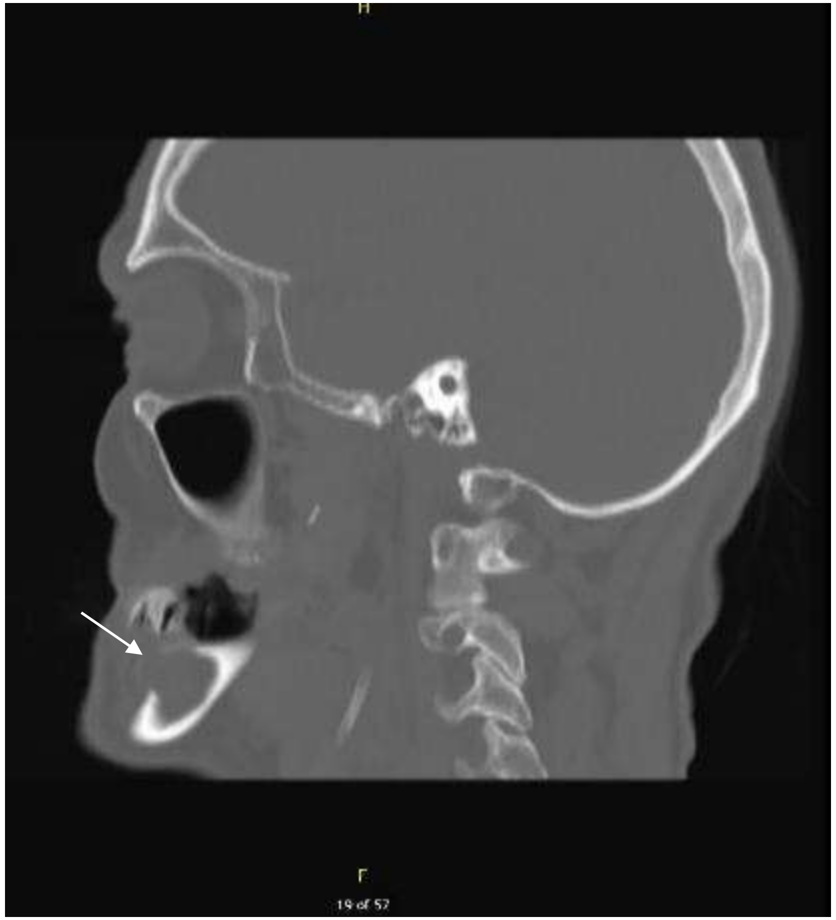

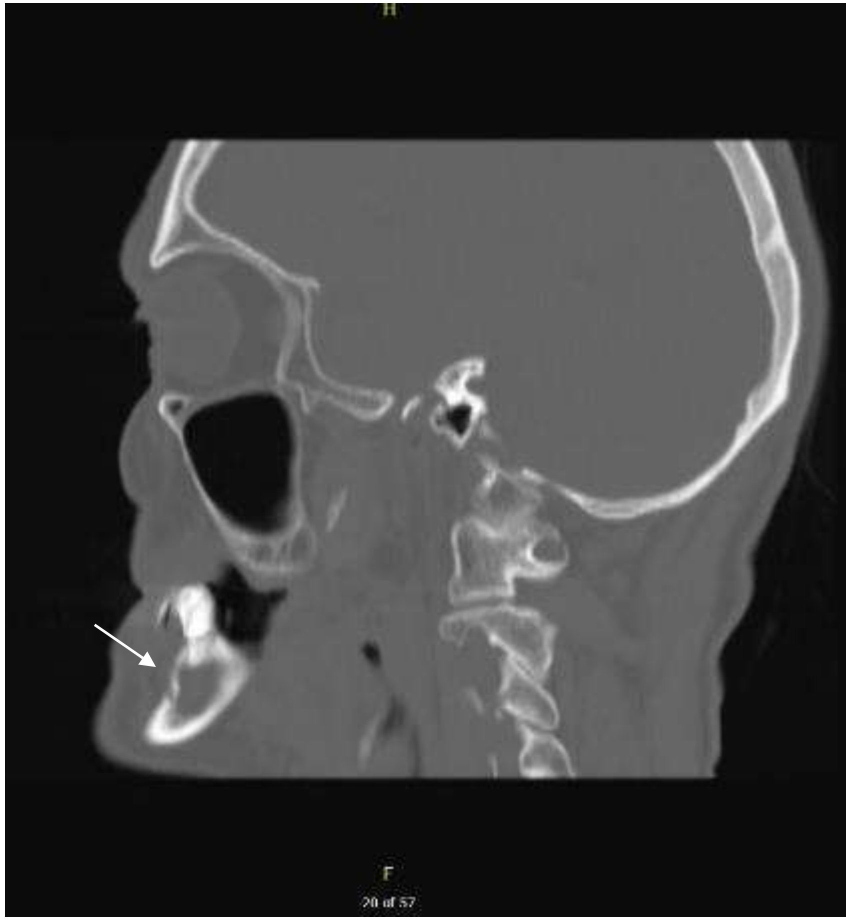

KR18 – HE, CK17, bcl2 (x1)

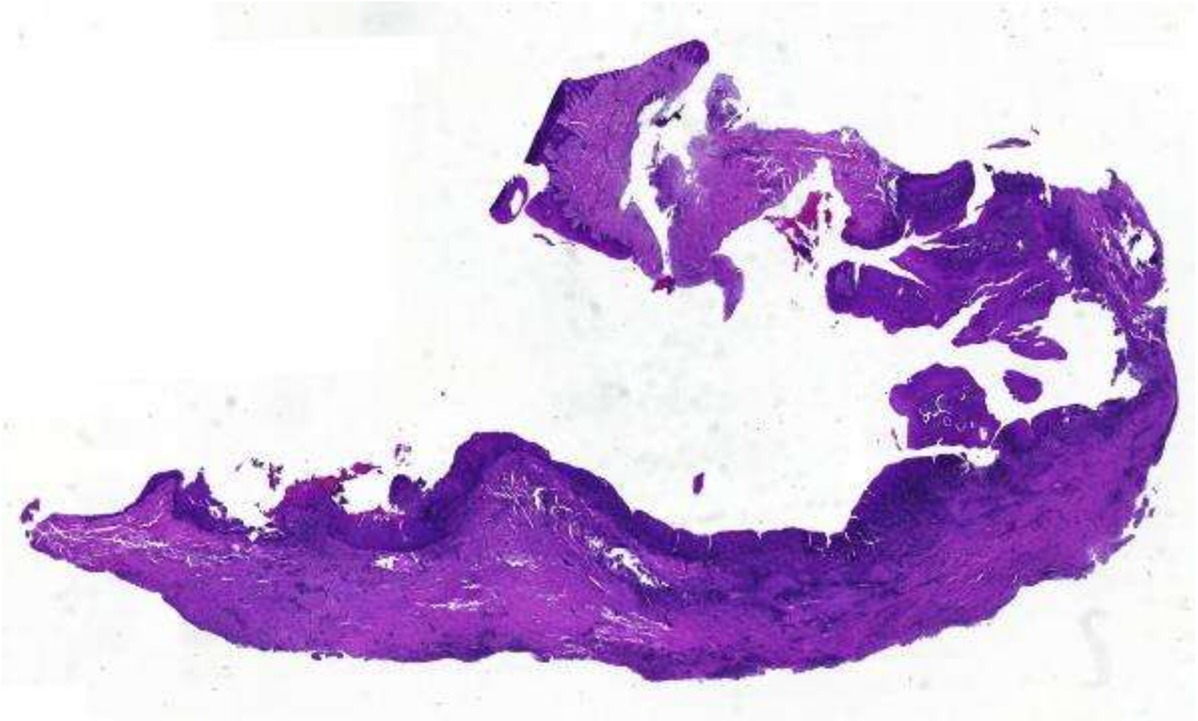

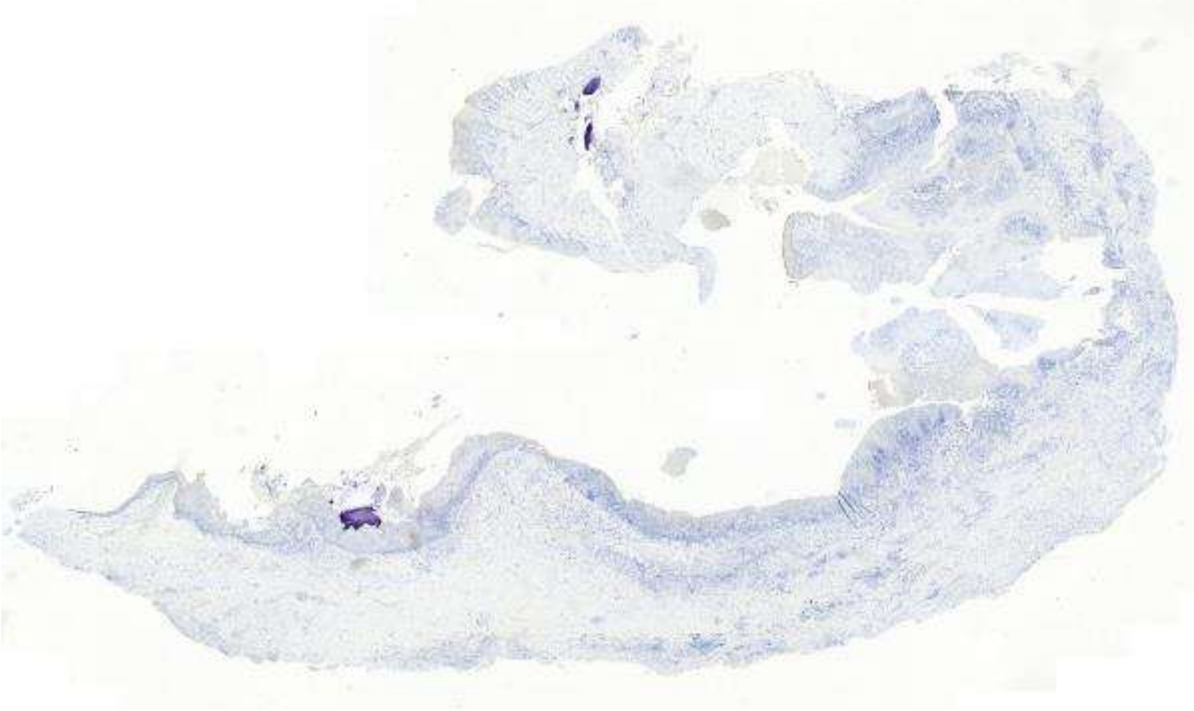

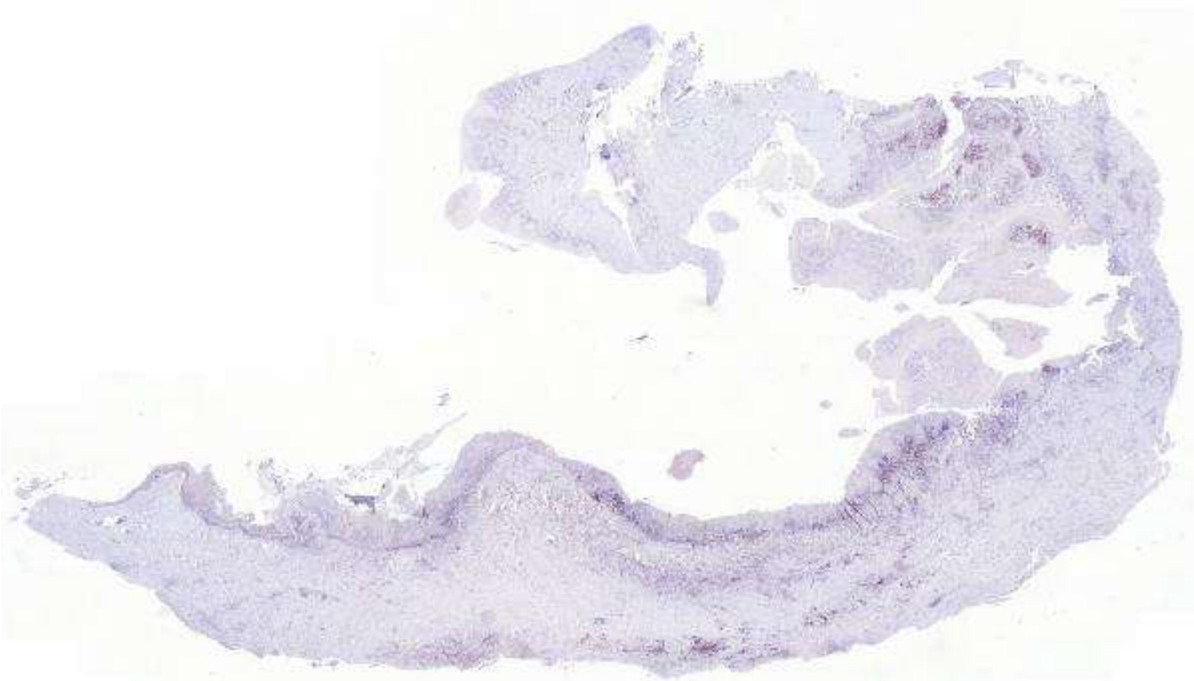

KR18 – HE, CK17, bcl2 (x5)

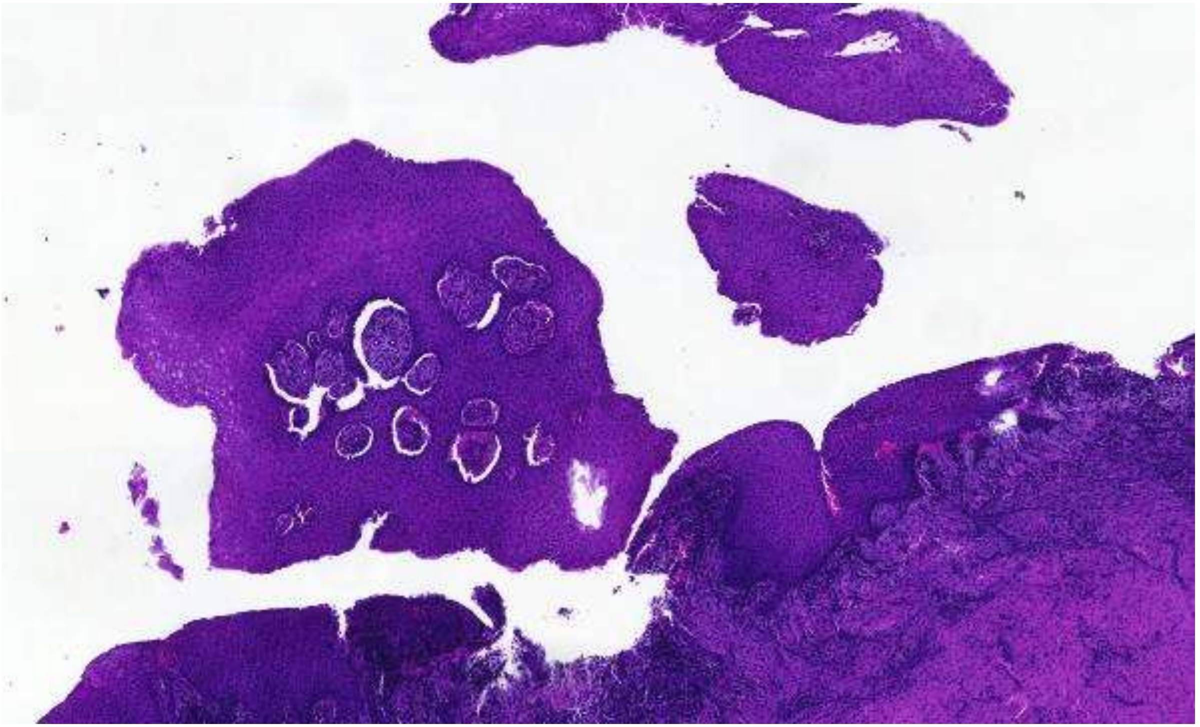

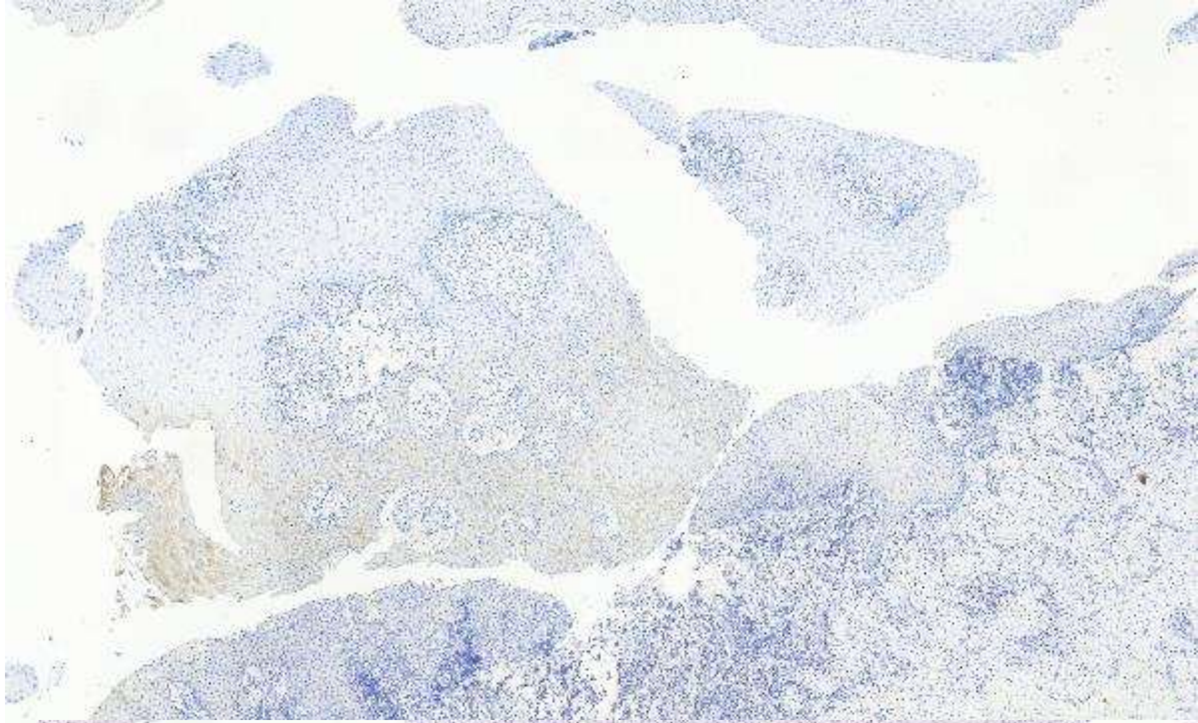

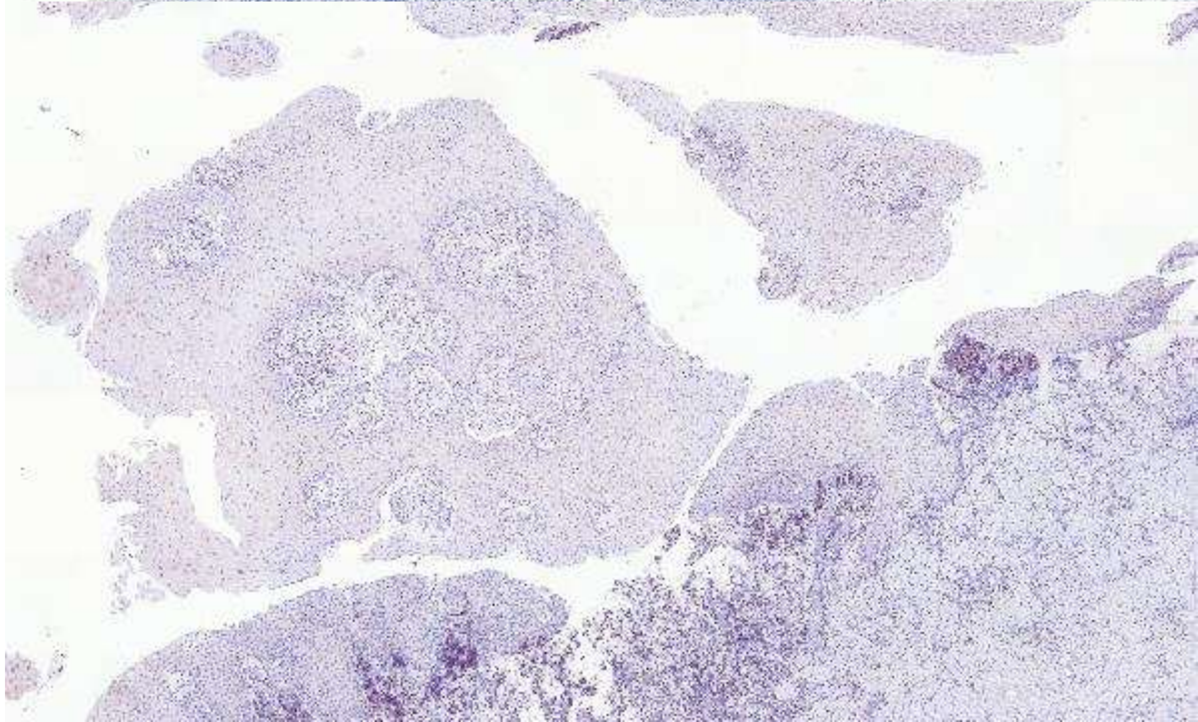

KR18 – HE, CK17, bcl2 (x5)

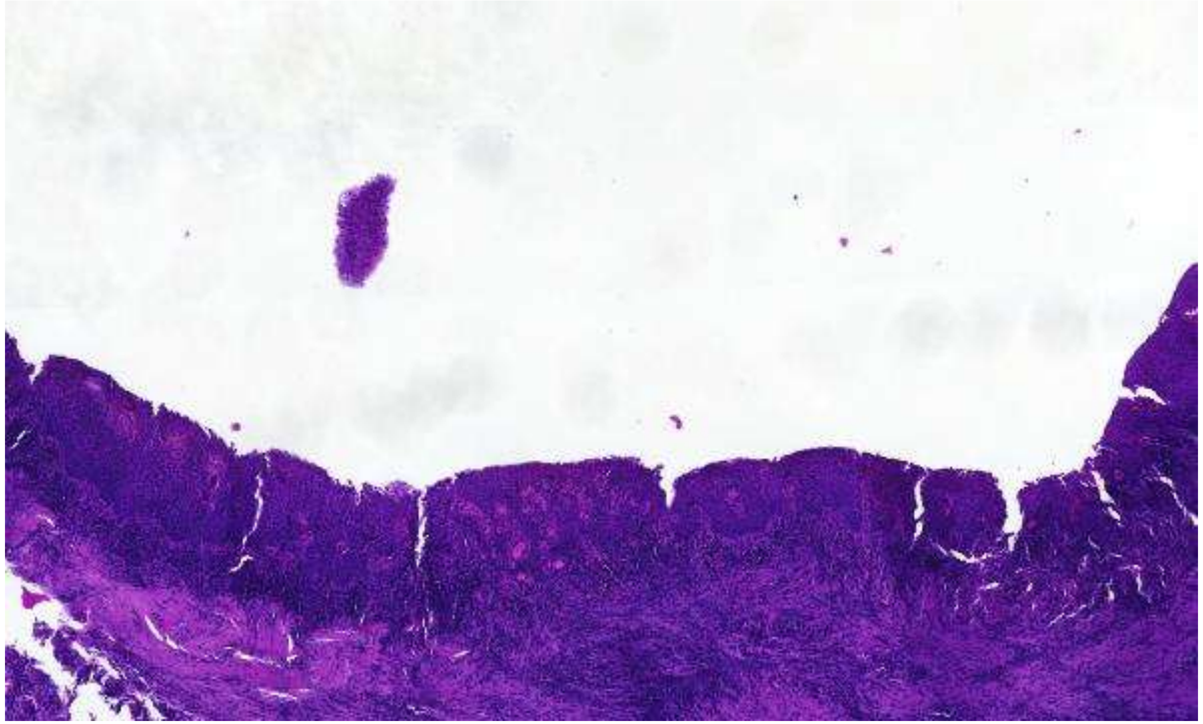

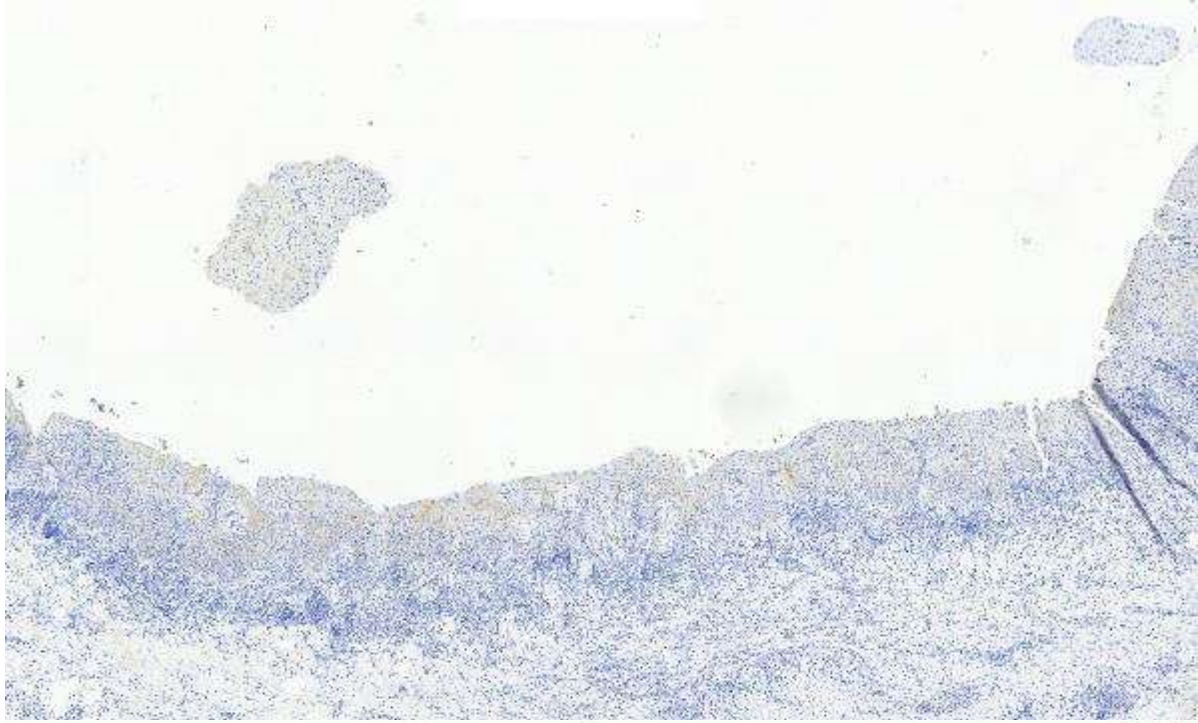

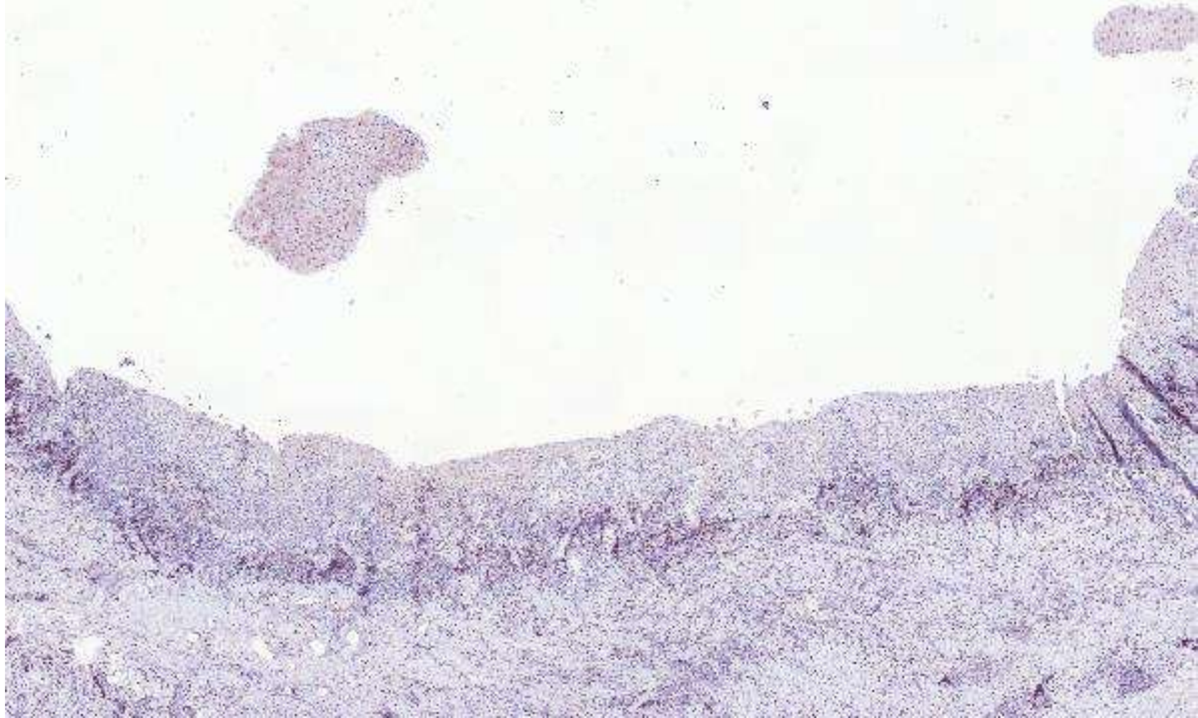

KR18 – HE, CK17, bcl2 (x15)

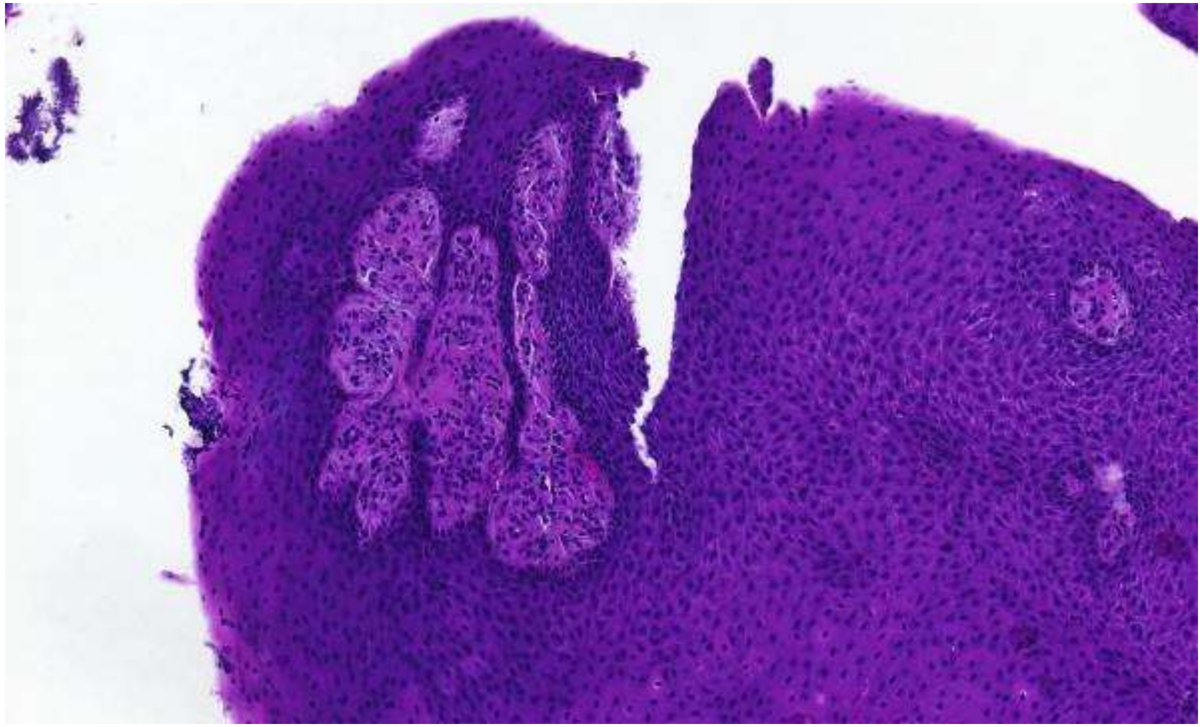

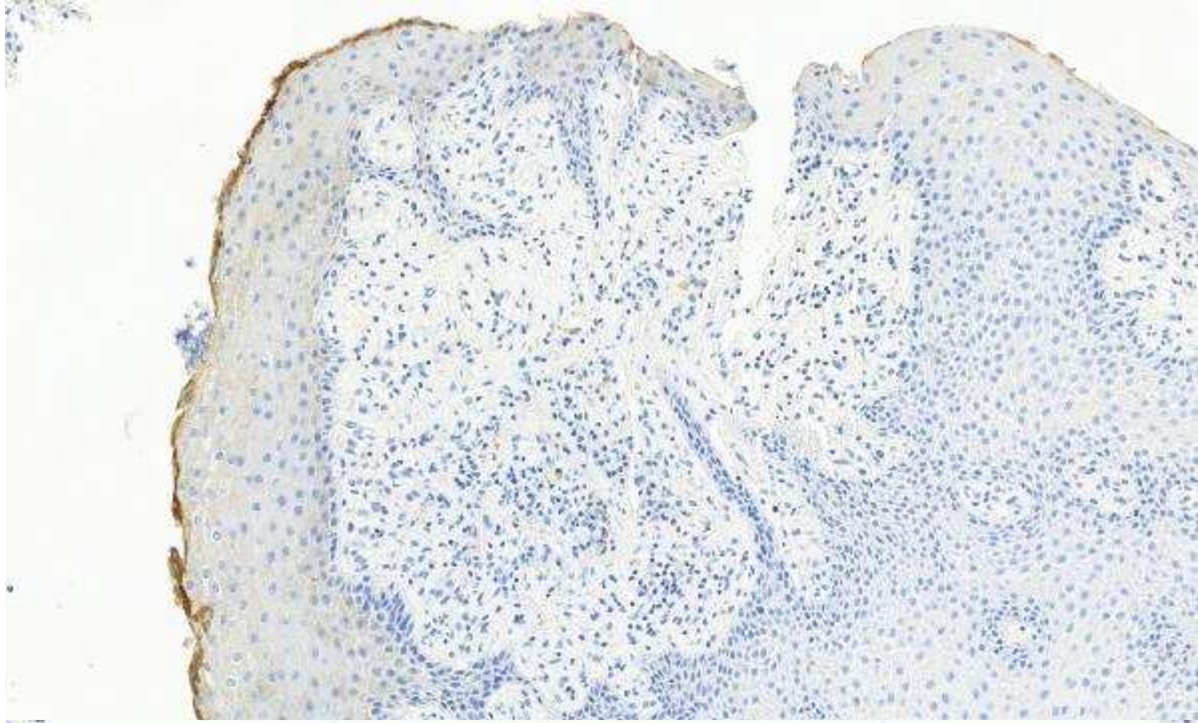

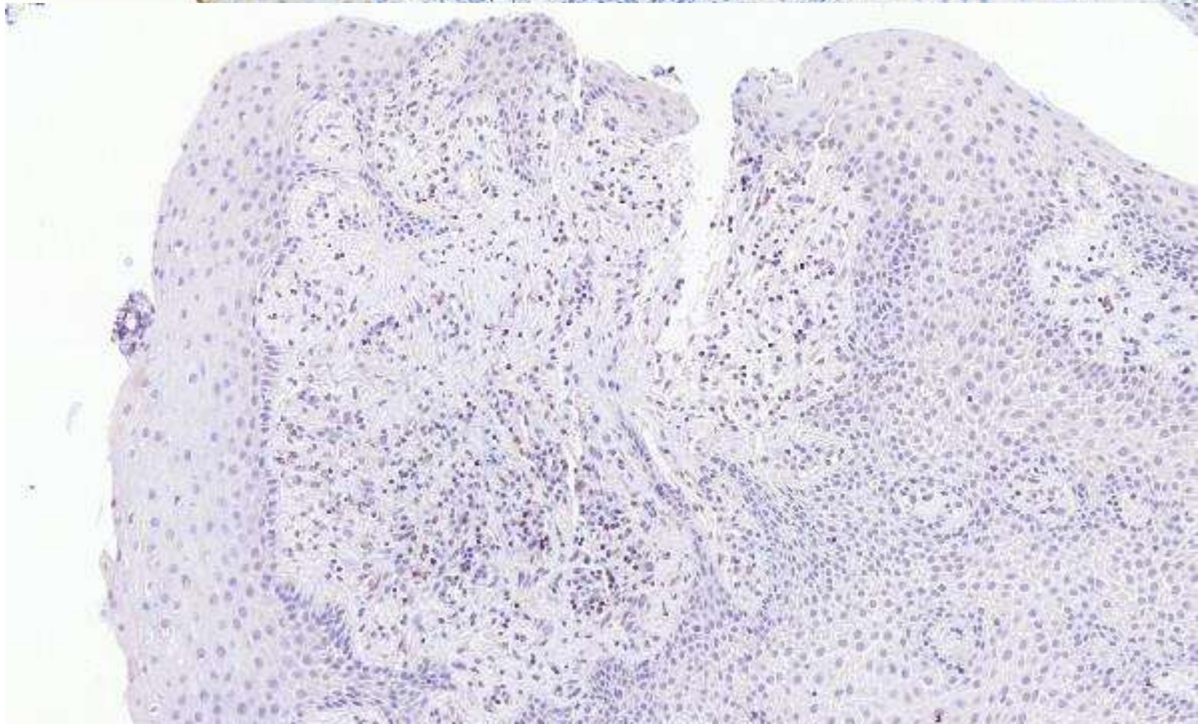

KR18 – Conclusion

- H&E: relatively thick squamous epithelium, no parakeratosis, no wavy surface, no basal palisading, uneven epithelial-subepithelial contours
- Mixed inflammatory infiltrate of the cyst wall
- Bcl2: negative
- CK17: dominantly negative, focal superficial and spinous
- Histopathology: inflammatory odontogenic cyst (N.O.S.)
- Combined clinical and pathological opinion: inflamed dentigerous cyst

Case KR19 (Same patient as KR13, but different cyst)

- 48-year-old male
- Dental hygiene/status: partly conserved, partly missing, untreated teeth, with symptomatic residual roots for teeth 11, 12, 13, 21; caries in multiple teeth
- Radiology: periapical (non-vital teeth 13-21) radiolucent lesion of 35 mm
- Operation: removal of roots 13-21, cystectomy
- Clinical diagnosis: periapical cyst

KR19 – OPT

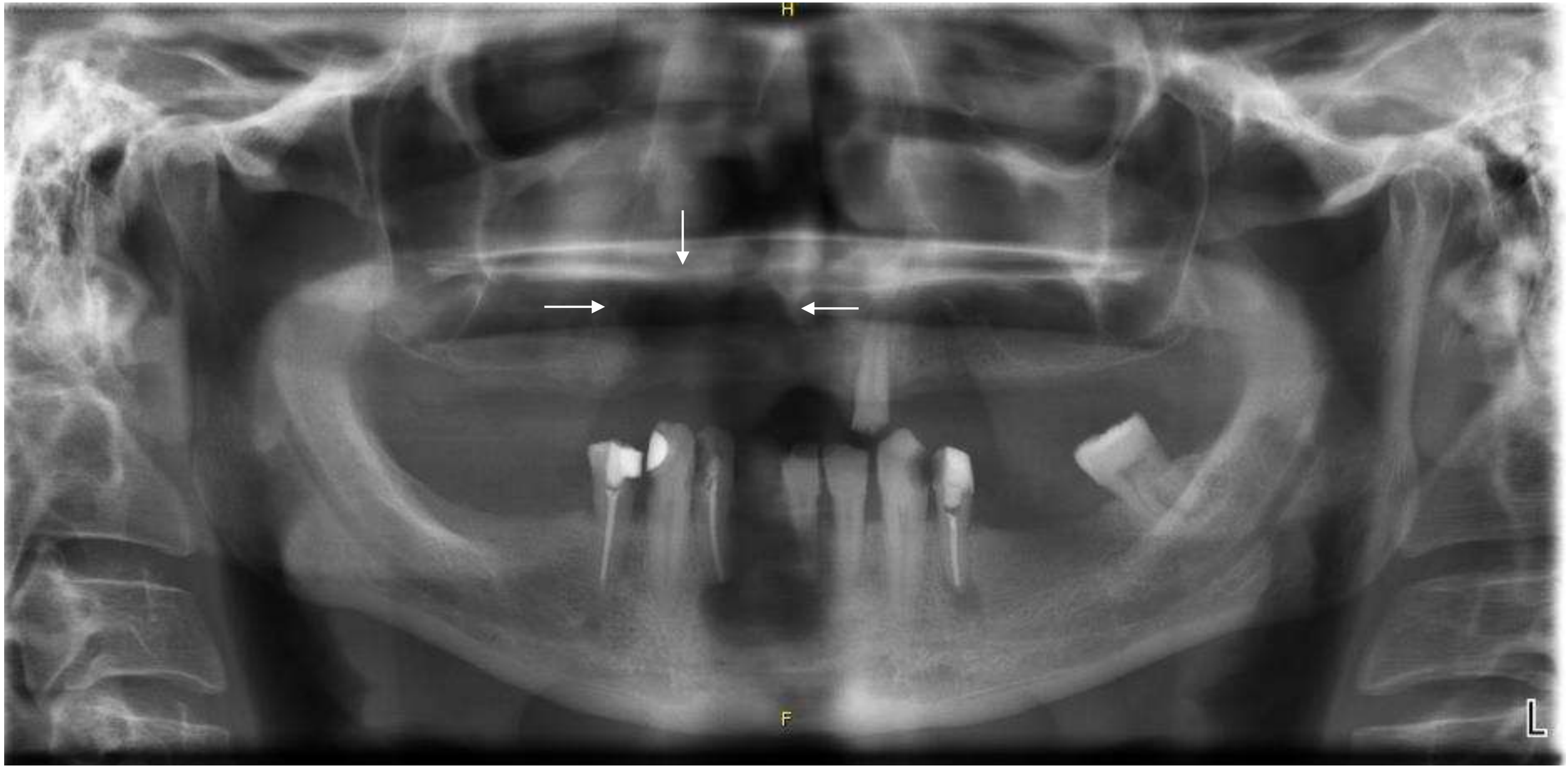

KR19 – HE, CK17, bcl2 (x1.5)

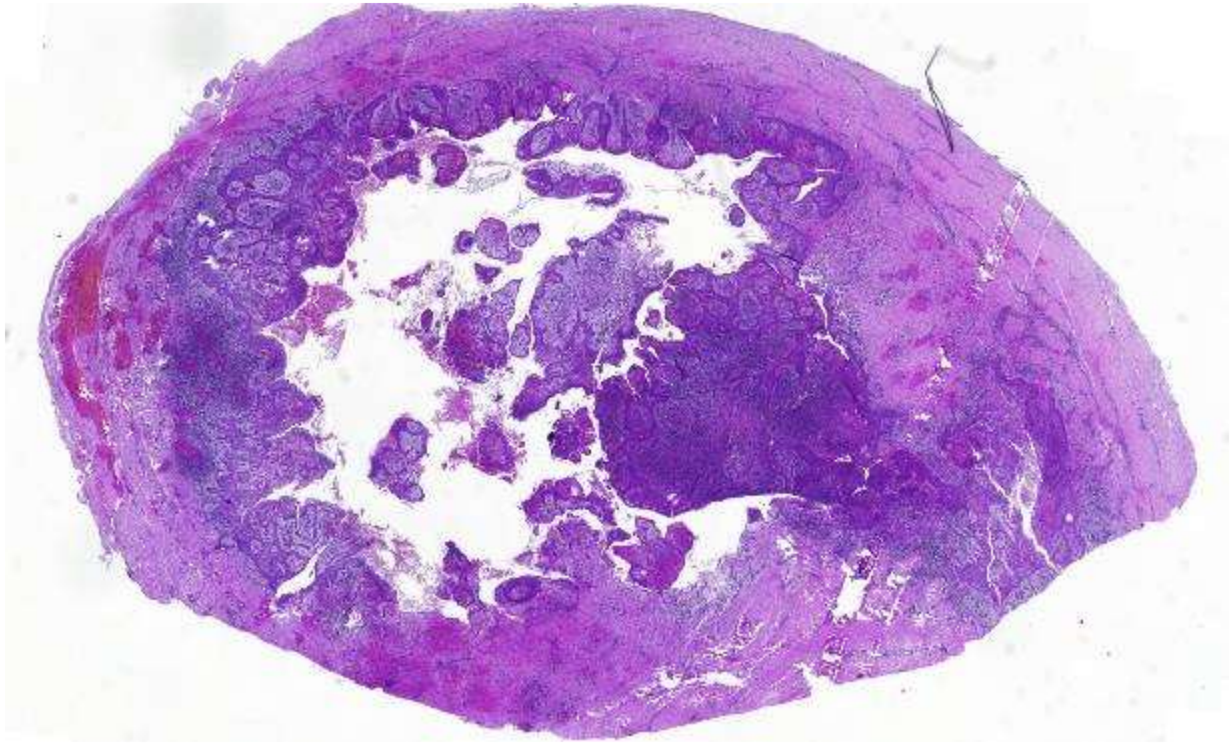

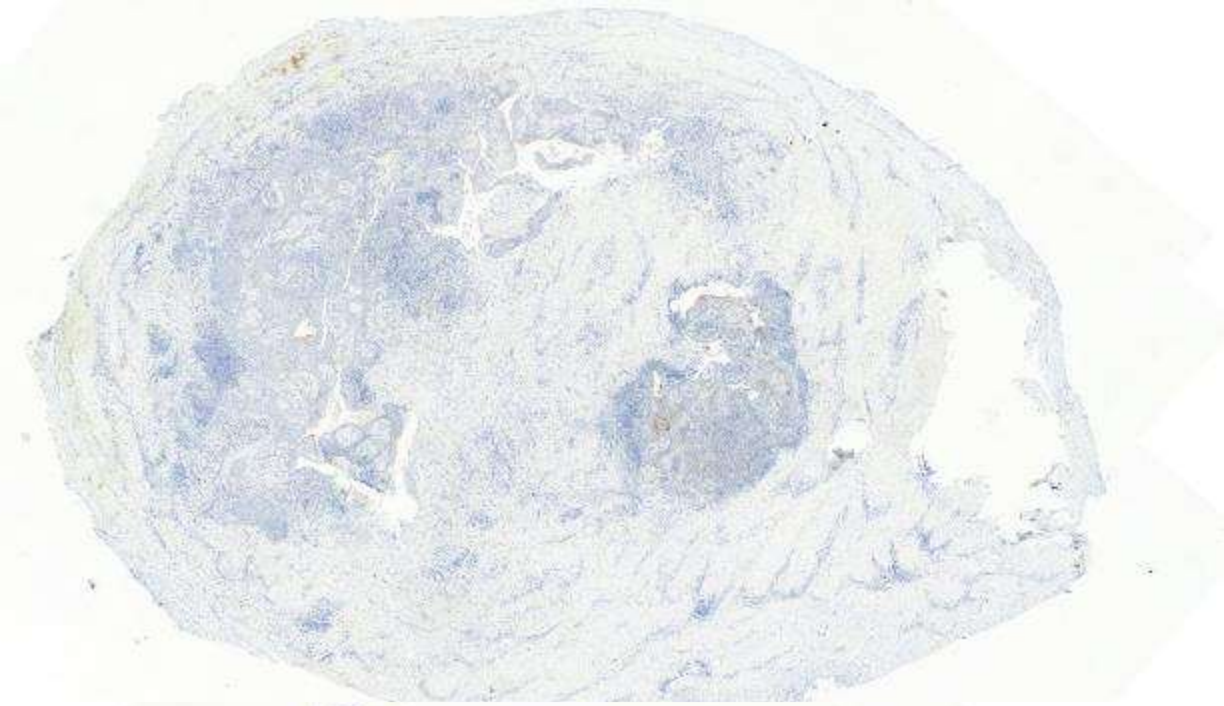

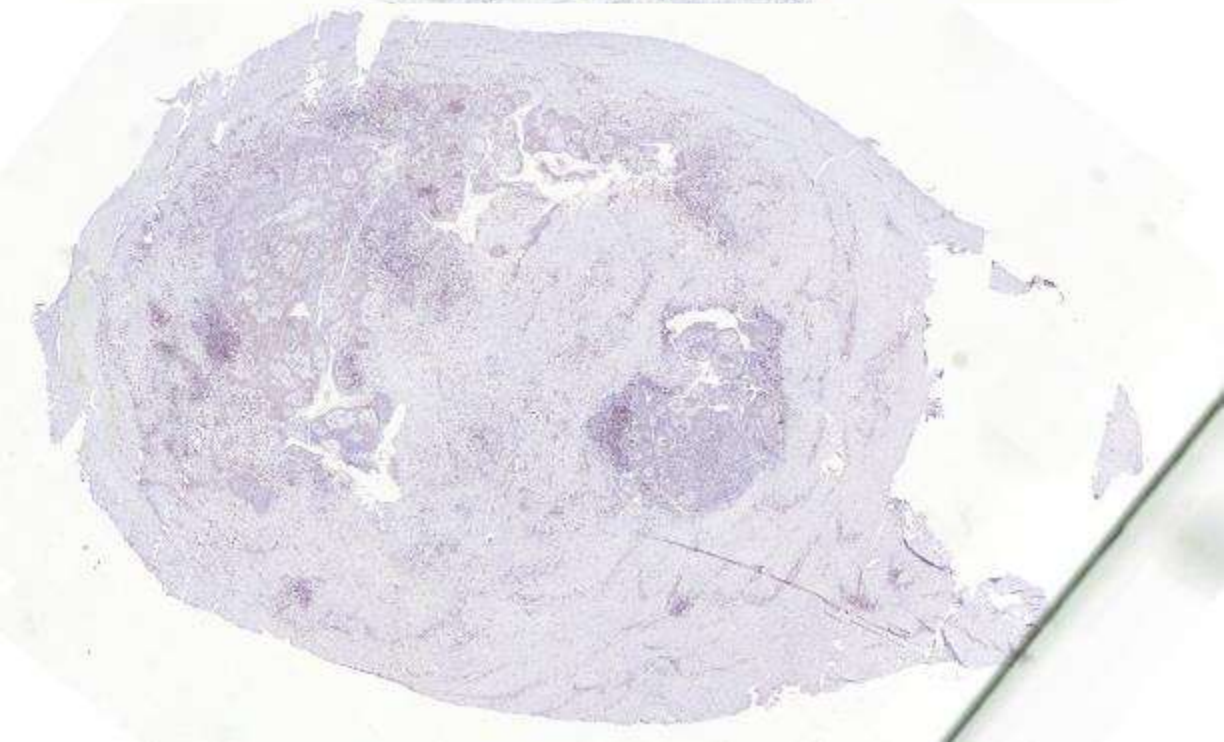

KR19 – HE, CK17, bcl2 (x5)

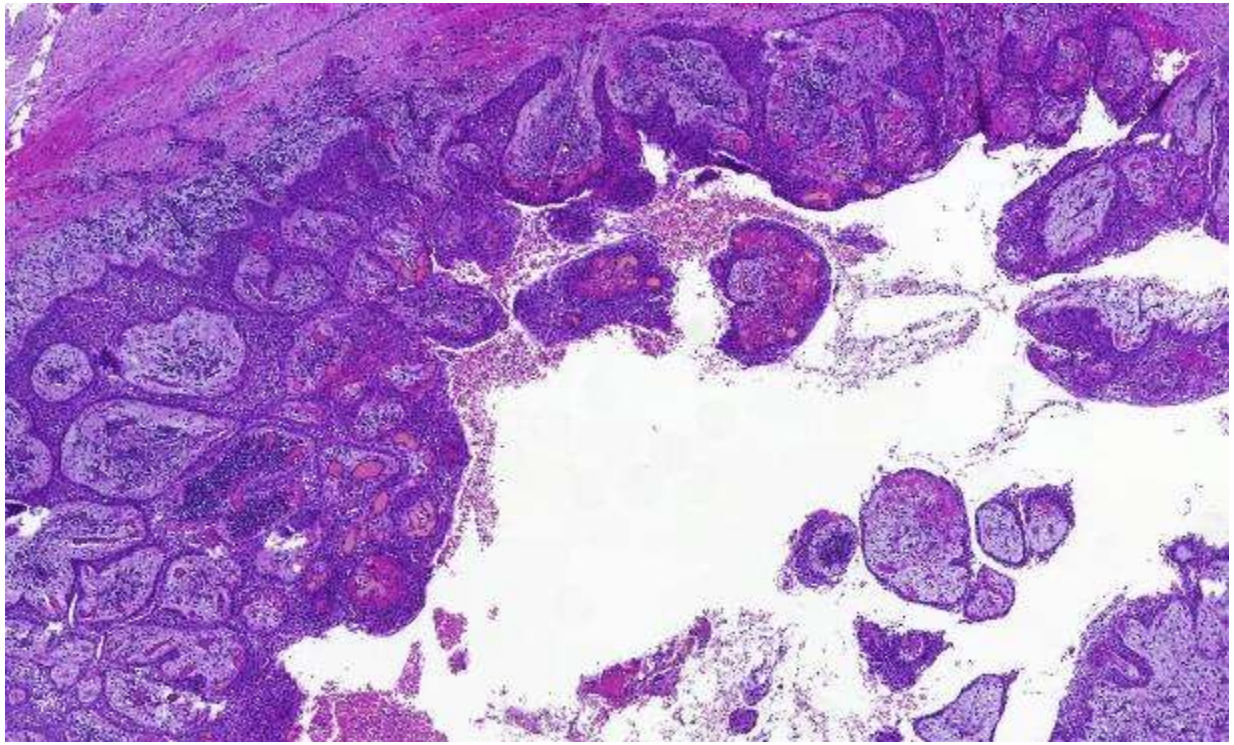

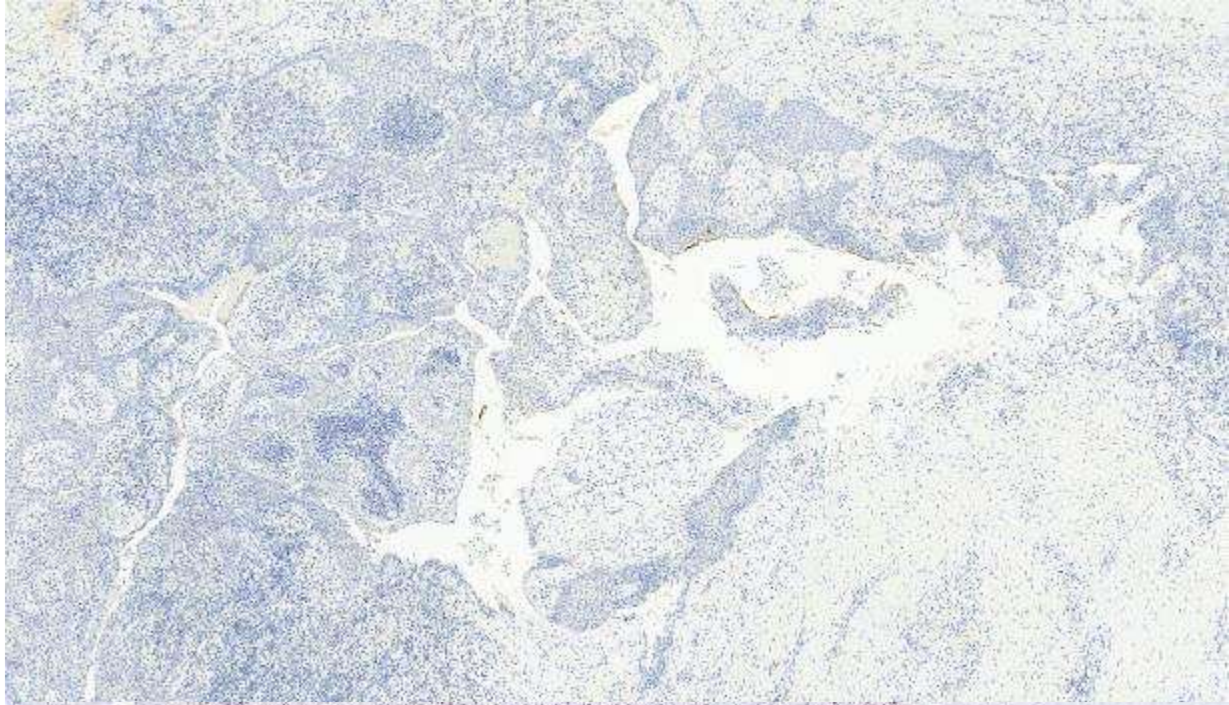

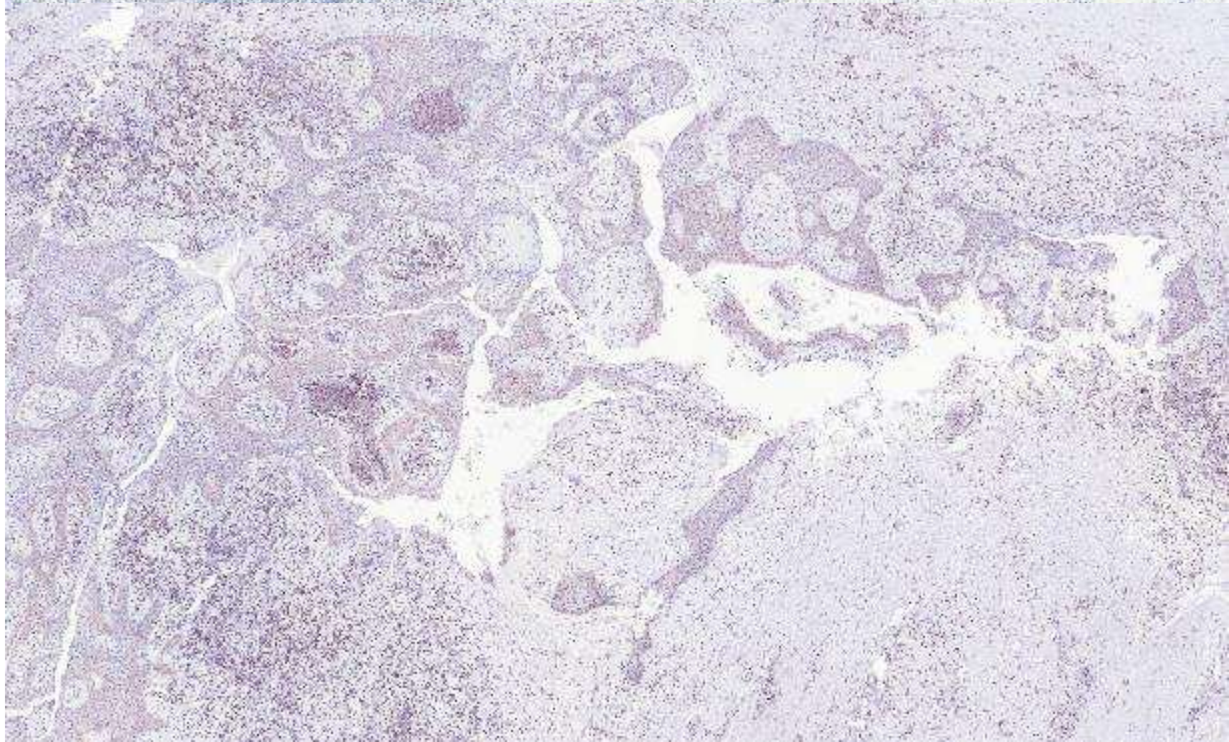

KR19 – HE, CK17, bcl2 (x6)

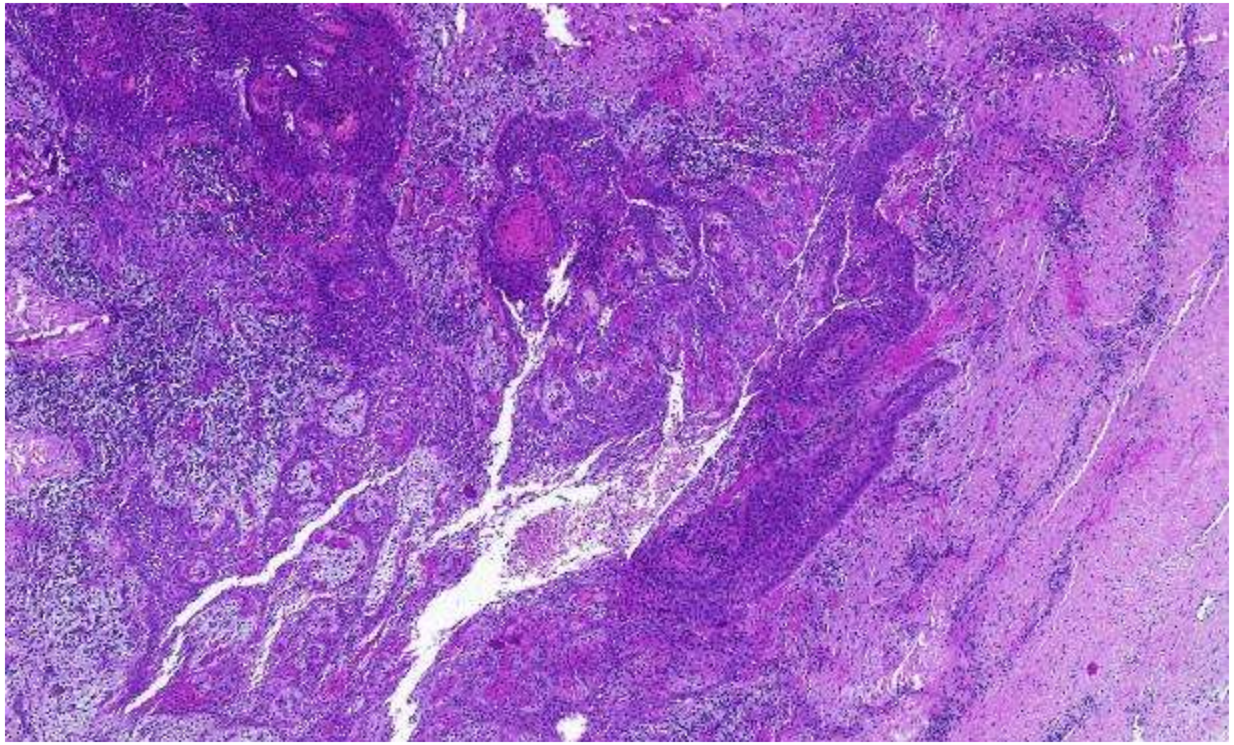

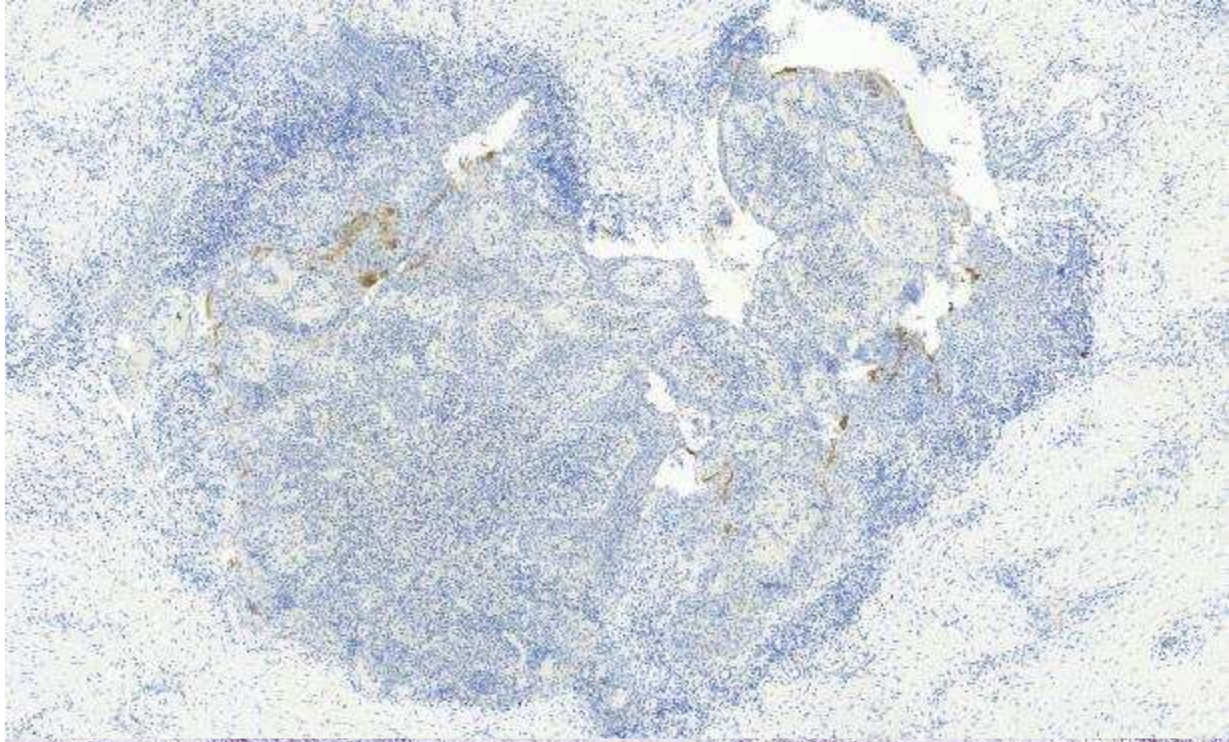

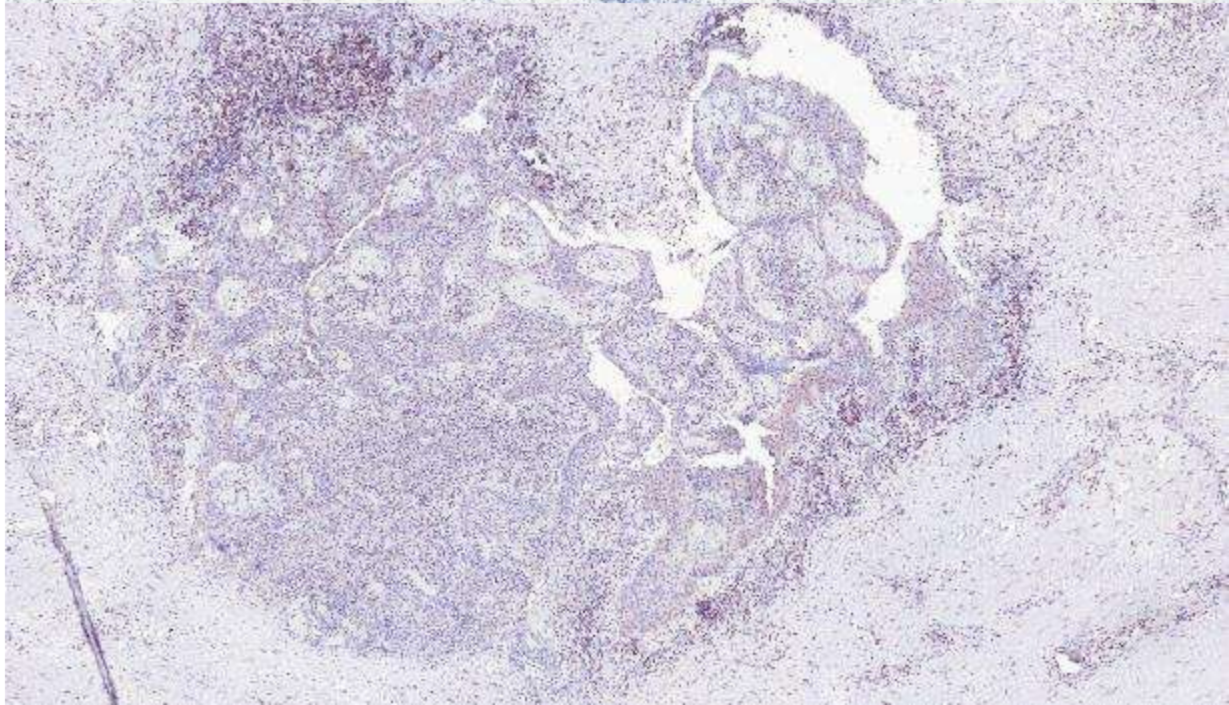

KR19 – CK17, bcl2 (x20) Single superficial cell CK17+ (*)

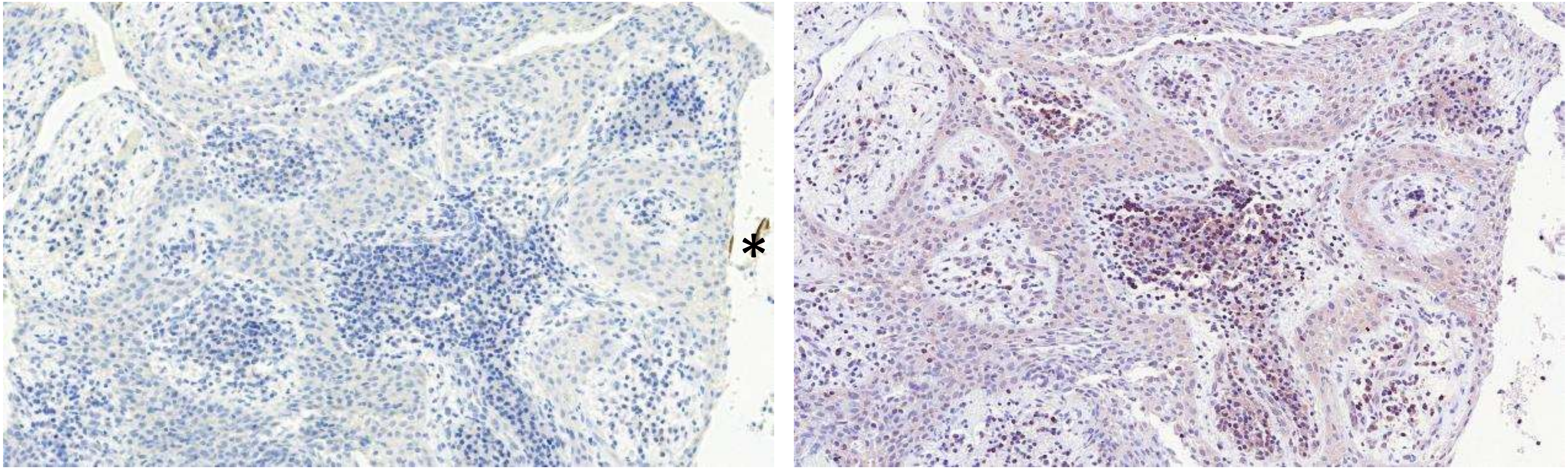

KR19 – Conclusion

- H&E: relatively thick squamous epithelium, no parakeratosis, no wavy surface, no basal palisading, uneven epithelial-subepithelial contours with prominent rete pegs; partly denuded, granulation tissue
- Inflammation of the cyst wall, mixed infiltrate with lymphocytes, plasma cells predominating
- Bcl2: negative
- CK17: dominantly negative, focal superficial staining
- Histopathology: apical cyst
- Combined clinical and pathological opinion: radicular cyst

Cases

S1-S41

S5, S23, S33, S36, S40 missing
(excluded due to lack of evaluable
epithelium from odontogenic cysts)

Case S1

- 35-year-old male
- History: intraoral incision, drainage and removal of tooth 37 were performed due to a left sided, infected mandible cyst one year ago.
- Dental hygiene/status: Pulsating pain occurs at the site of operation.
- Operation: cystectomy
- Clinical diagnosis: keratocyst? (uncommunicated history includes 2 previous cystectomies of the same region; i.e. this is a recurrent cyst – previous histology not mentioned)

S1 - OPT

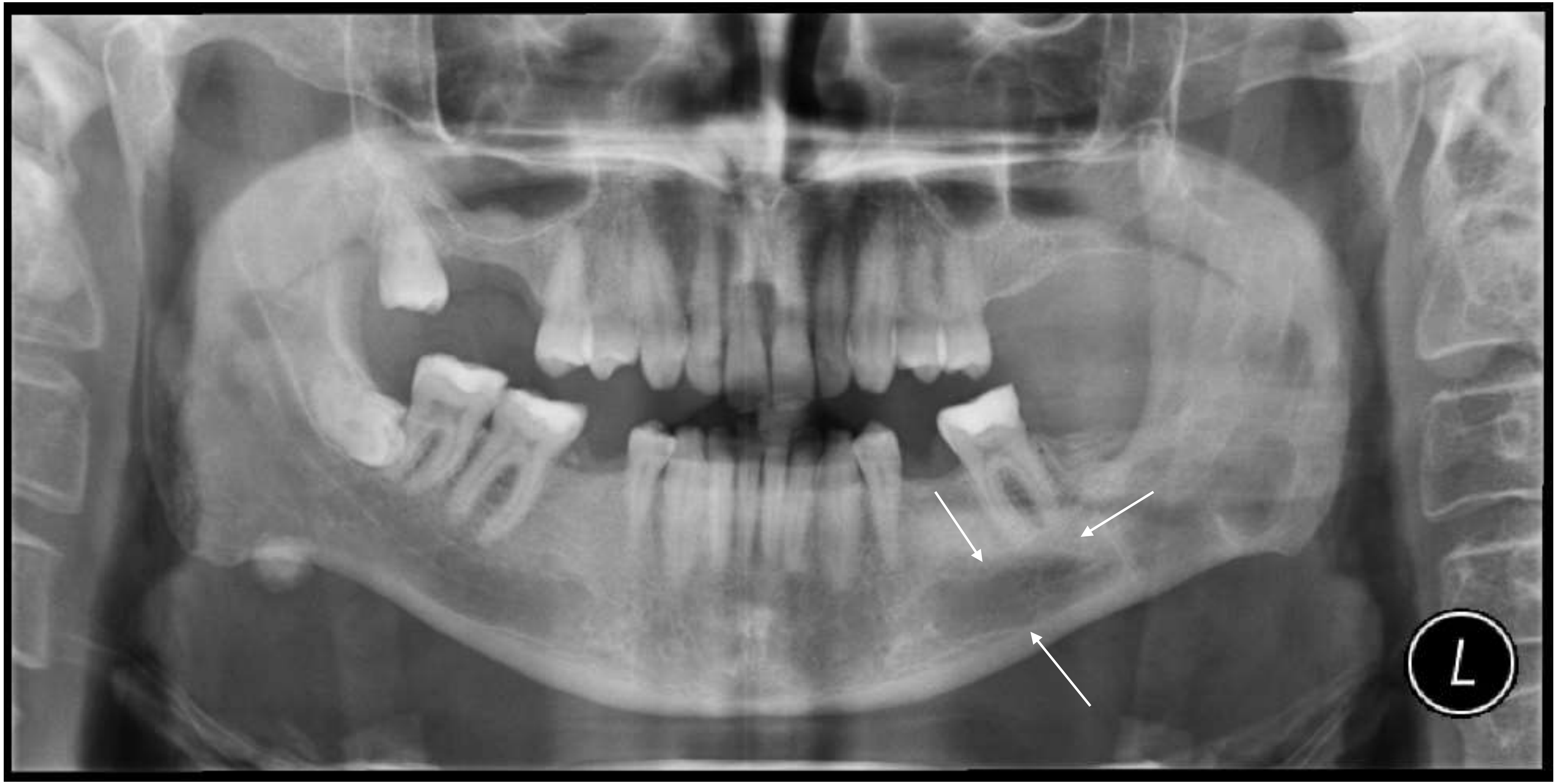

S1 CT - axial

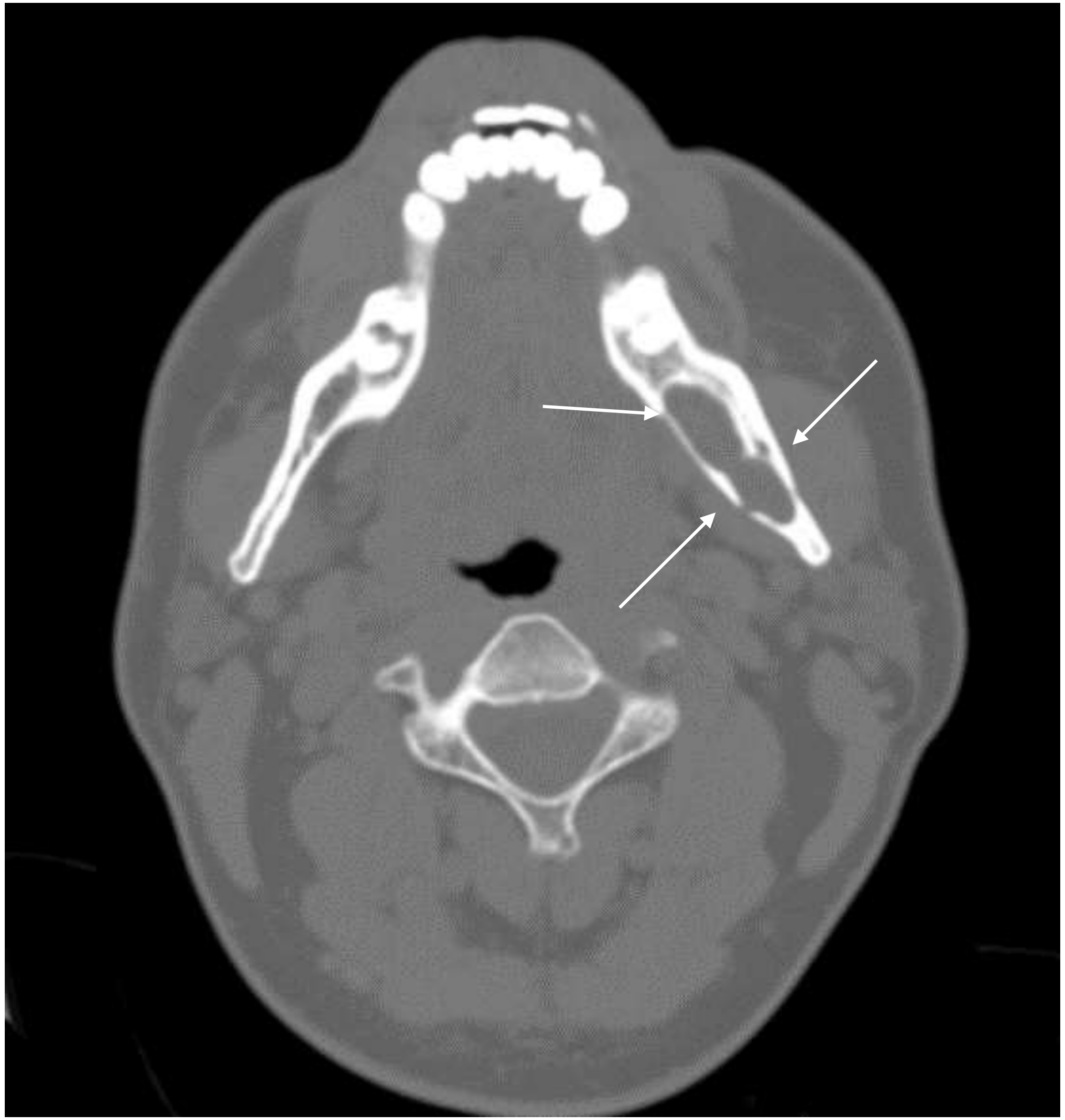

S1 – HE, CK17, bcl2 (x20)

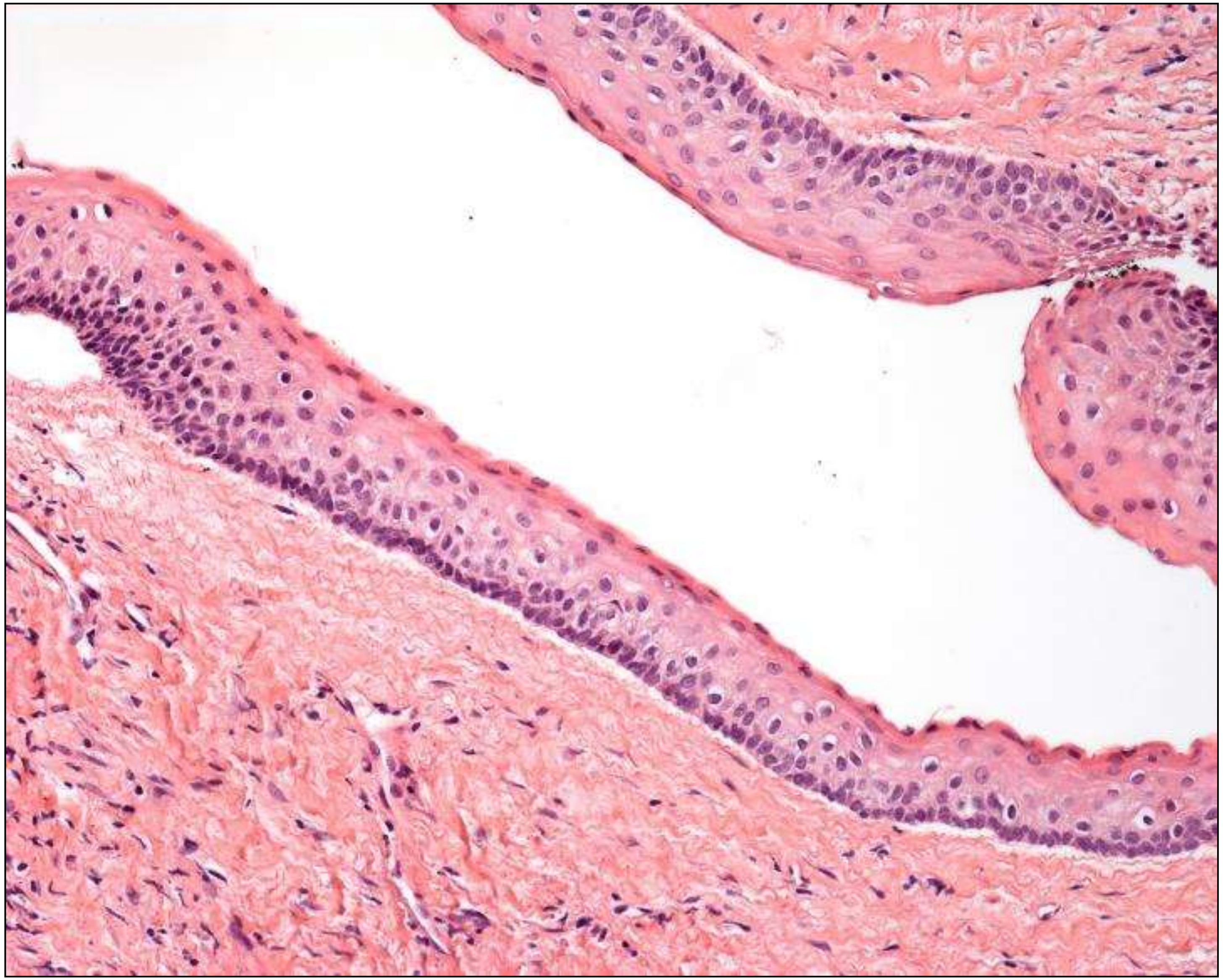

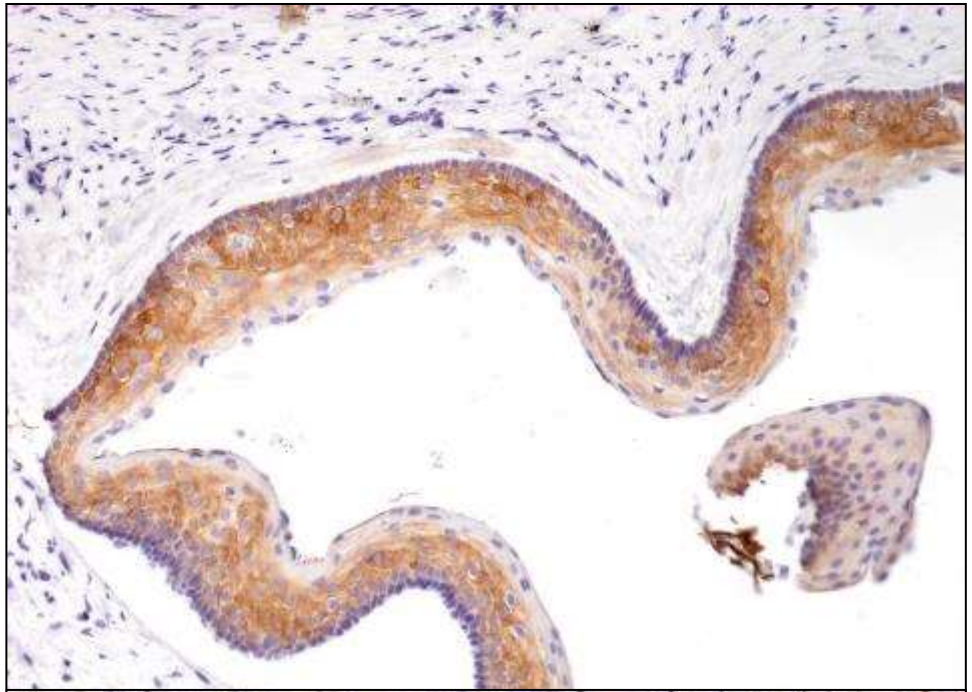

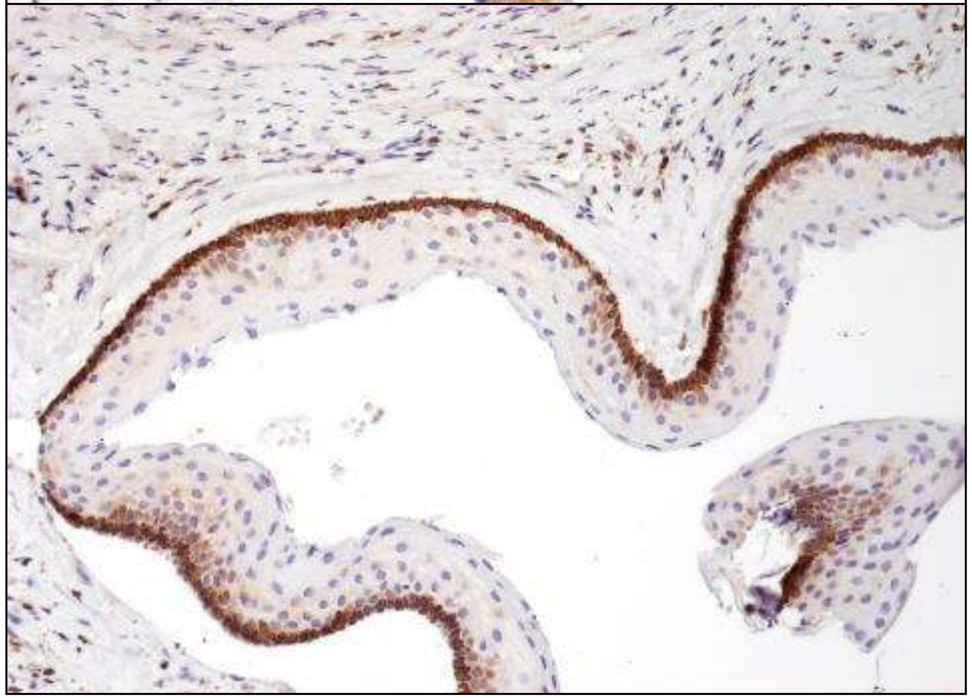

Case S1 – Conclusion

- H&E: thin parakeratotic epithelium with wavy surface and basal palisading, even epithelial stromal interface
- Inflammation dominantly absent; focal lymphocytic infiltrate in the cyst wall, beneath the epithelium
- Bcl2: diffuse, strong basal, suprabasal positivity
- CK17: transepithelial positivity (focal superficial sparing)
- Histopathological diagnosis: OKC
- Combined clinical and pathological opinion: OKC

Case S2

- 31-year-old male
- Dental hygiene/status: temporary inlay in tooth 25 (root canal treatment in-process), vitality test of tooth 26 is negative. Left maxillary sinus is inflamed.
- Radiology: periapical radiolucent area at tooth 26 (non-vital) and thickening of right sinus mucosa.
- Operation: Caldwell-Luc surgery with removal of tooth 26
- Clinical diagnosis: soft tissue from left maxillary sinus, radicular cyst

S2 – OPT (postoperative)

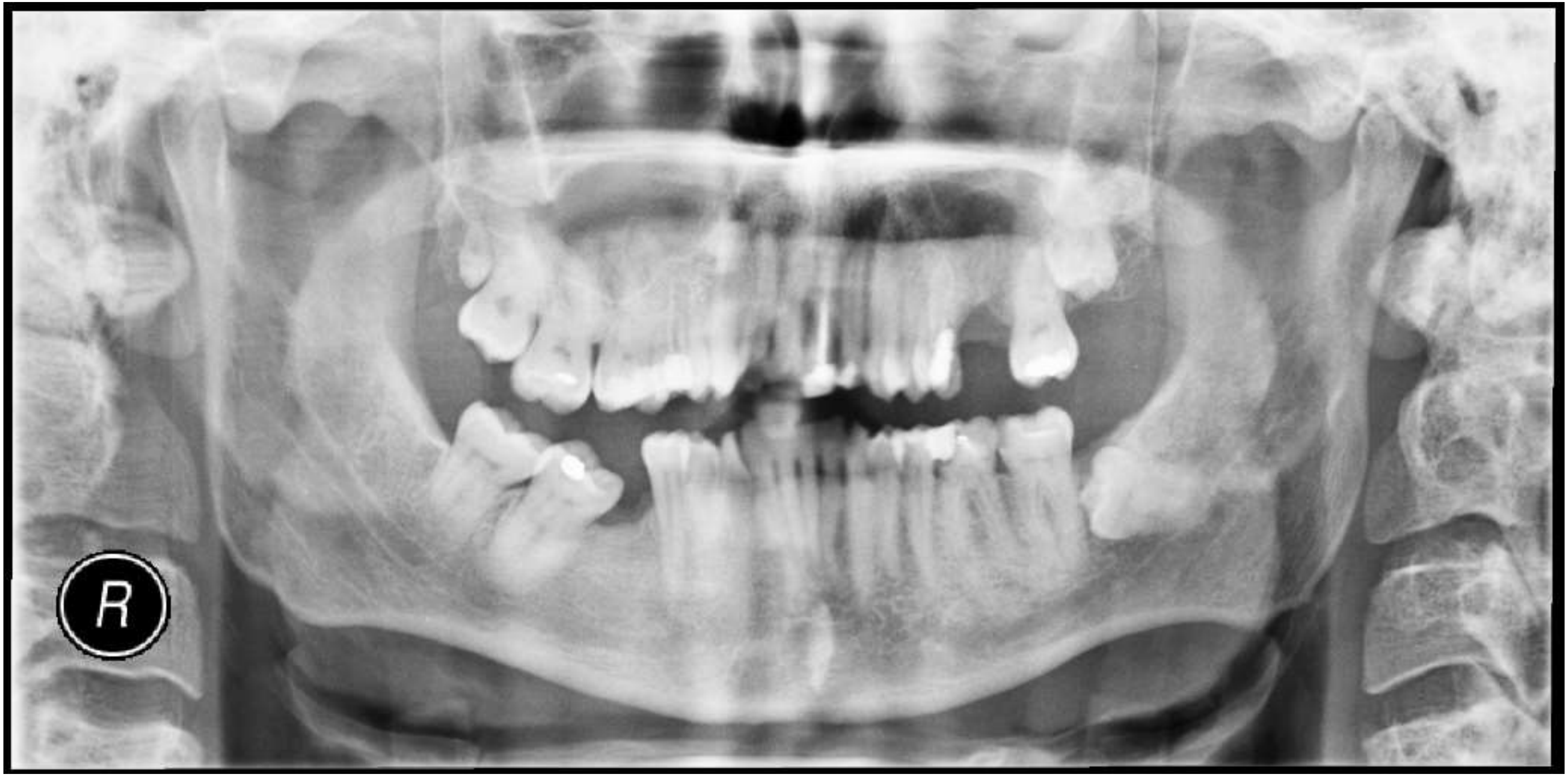

S2 CT coronal

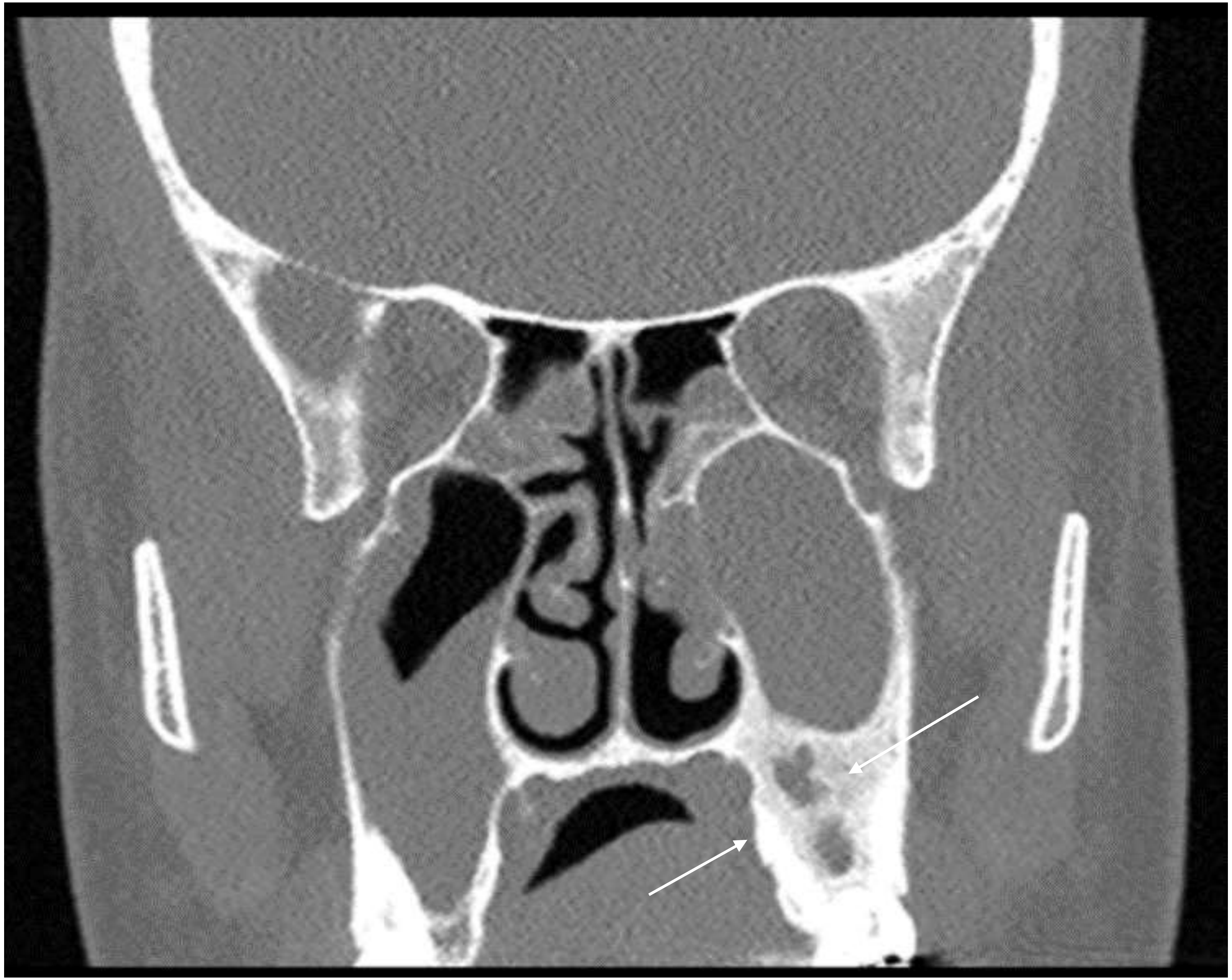

S2 – HE, CK17, bcl2 (x20)

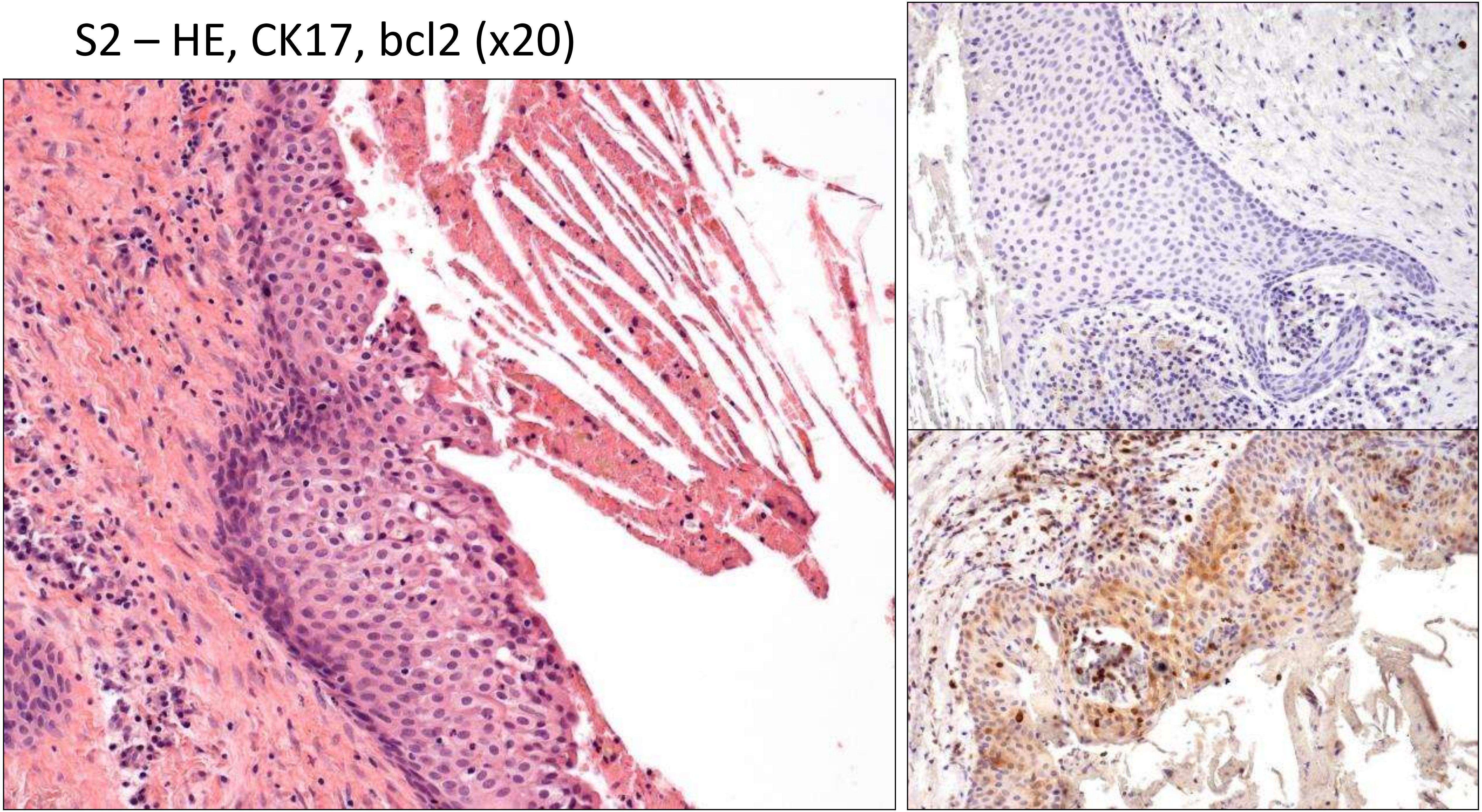

Case S2 – Conclusion

- H&E: multilayered, focally thick squamous epithelium without parakeratosis, basal palisading or wavy surface, uneven epithelial stromal interface
- Focally marked chronic inflammation without acute component
- Bcl2: dominantly negative, focal spinous and transepithelial positivity
- CK17: negative
- Histopathological diagnosis: radicular cyst
- Combined clinical and pathological opinion: radicular cyst

Case S3

- 23-year-old male
- Dental hygiene/status: impaction of teeth 18, 28, 38, 48, palpable fluctuation behind tooth 37 (tooth 37 is vital)
- OPT: radiolucent area around tooth 38 affecting the root of tooth 37 and basis of the mandible
- Operation: removal of teeth 18, 28, 38, 48, cystectomy
- Clinical diagnosis: none; keratocyst?

S3 - OPT

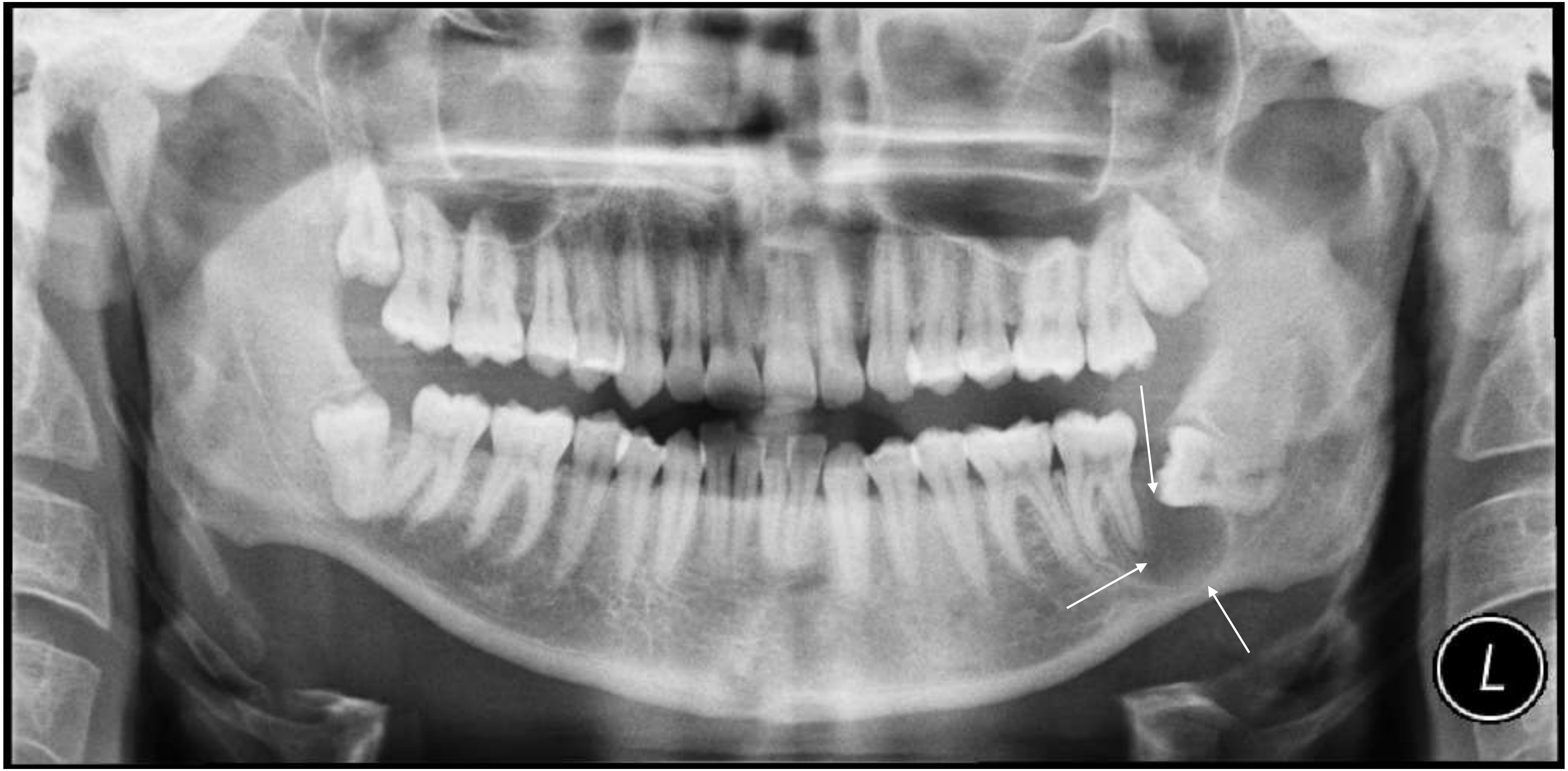

S3 – HE, CK17, bcl2 (x20)

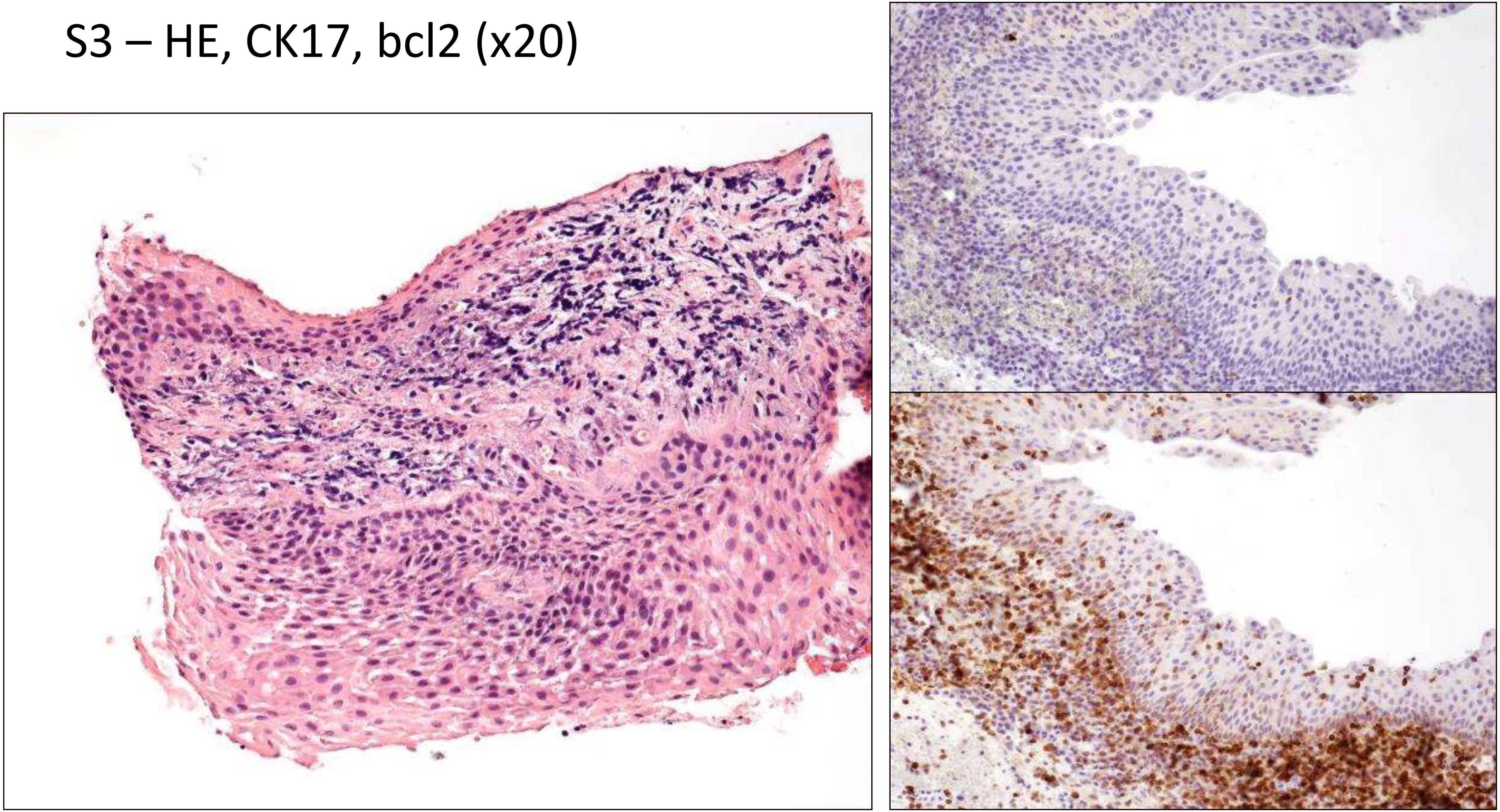

S3 – bcl2 (x20)

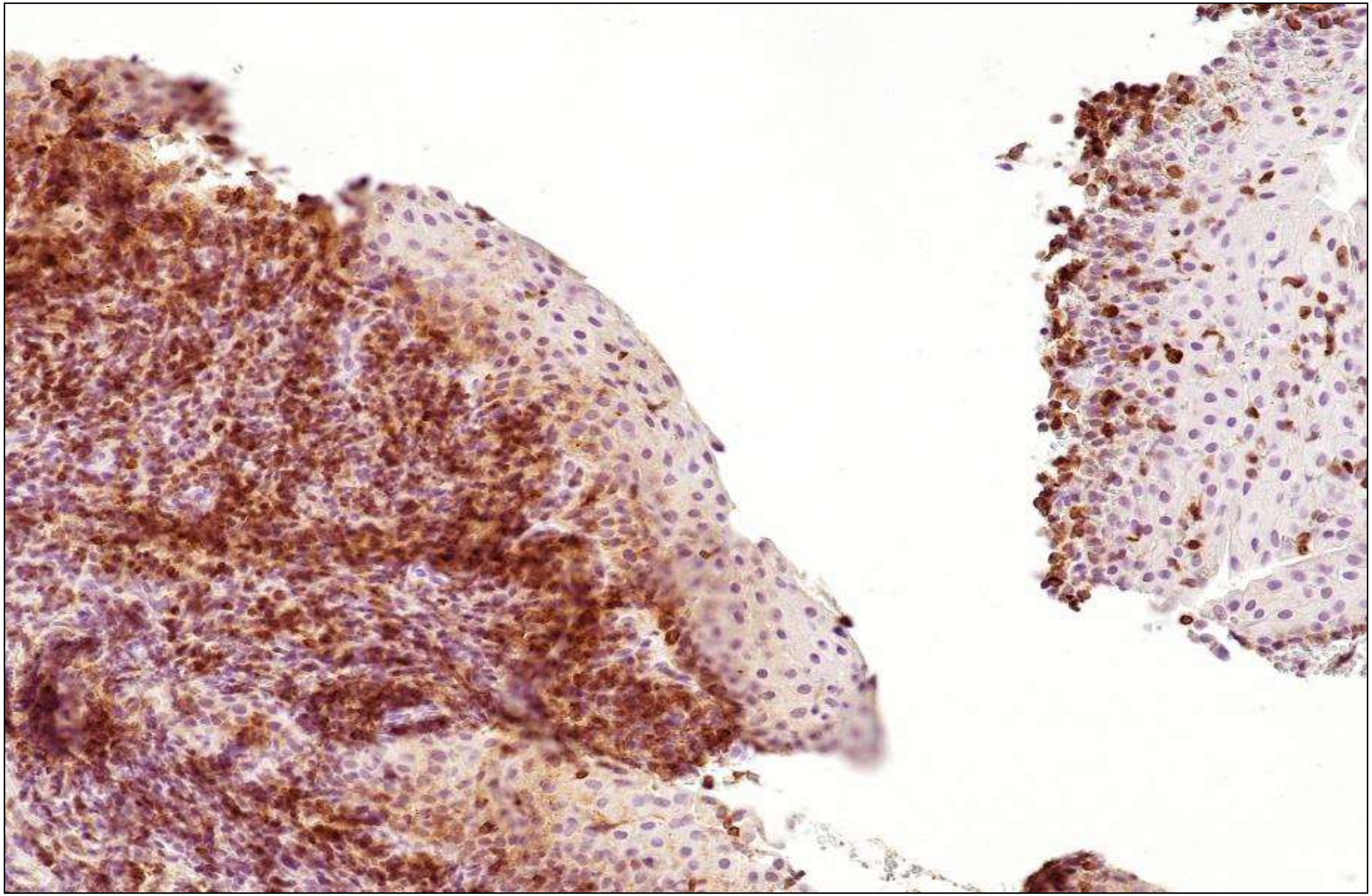

Case S3 - Conclusion

- H&E: relatively thick, squamous epithelium without keratinization, parakeratosis, basal palisading or wavy surface, uneven epithelial stromal interface
- Focally marked chronic inflammation without significant acute component
- Bcl2: negative, minimal focal faint basal staining
- CK17: negative
- Histopathological diagnosis: radicular cyst
- Combined clinical and pathological opinion: inflamed dentigerous cyst

Case S4

- 26-year-old male
- Dental hygiene/status: neglected teeth, root of tooth 46, the vestibule of right lower quadrant shows an elevated fornix.
- OPT: radiolucent area around the apexes of teeth 36 and 46.
- Operation: removal of tooth 46, cystectomy
- Clinical diagnosis: radicular cyst

S4 - OPT

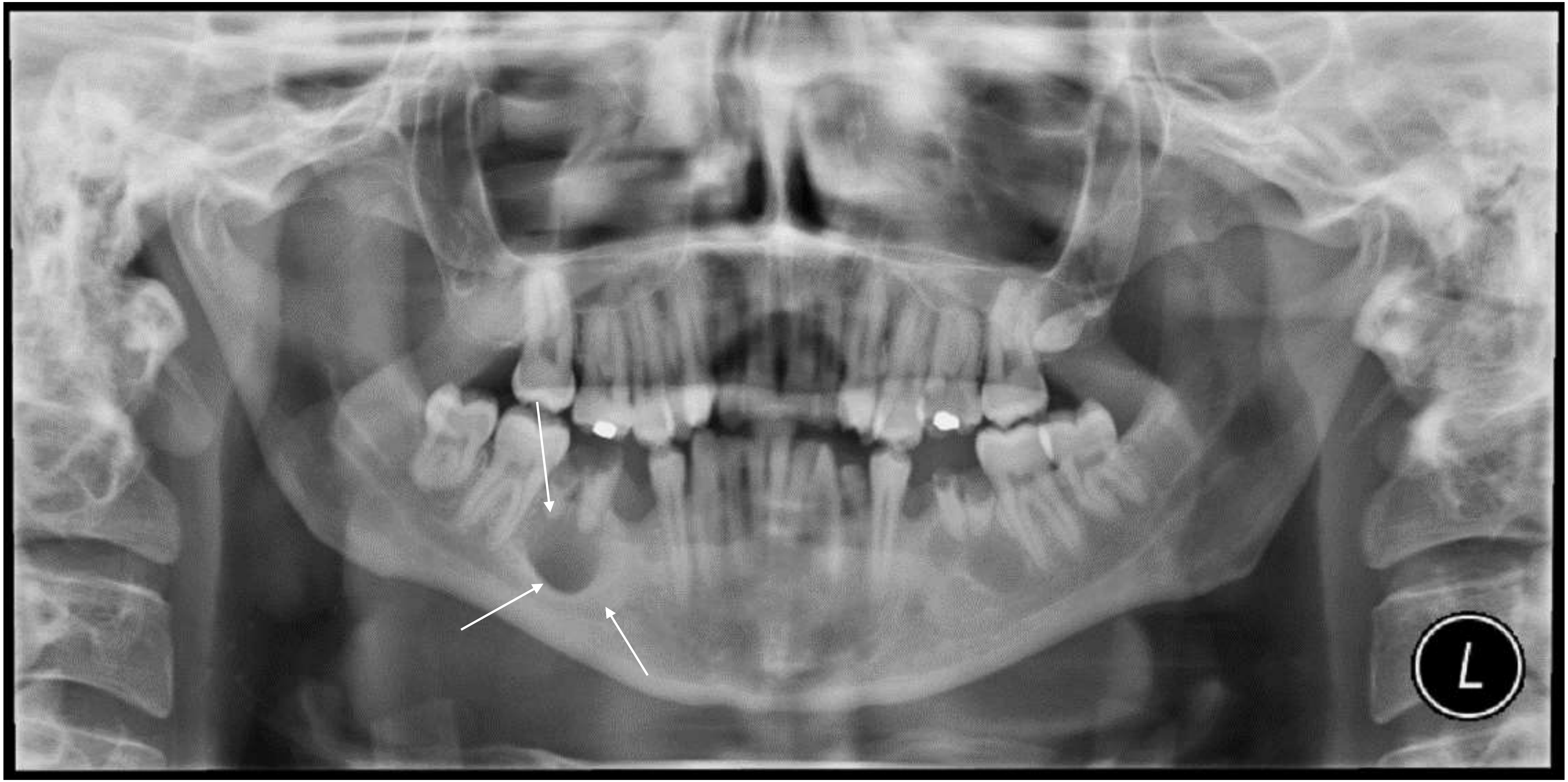

S4 – HE, CK17, bcl2 (x20)

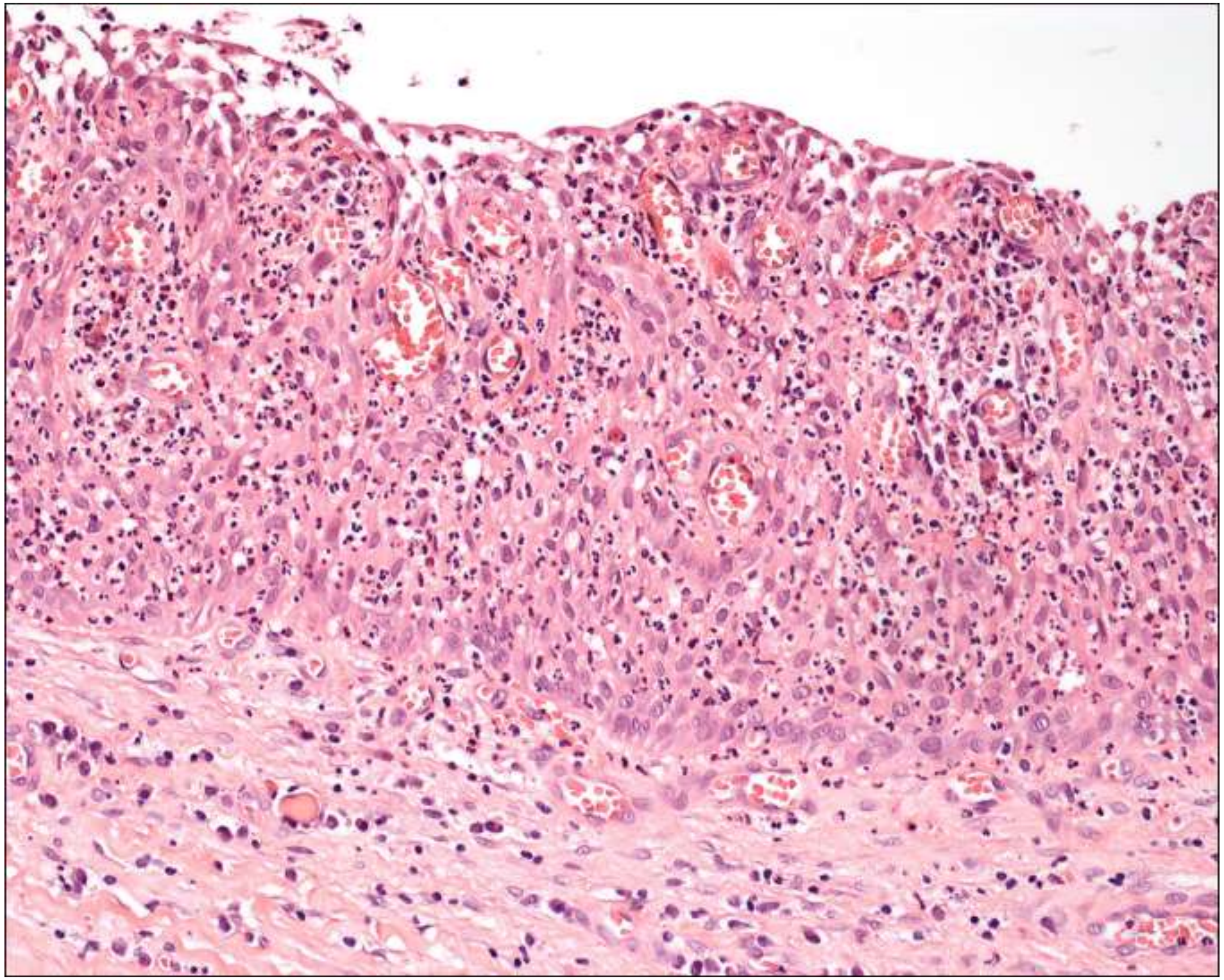

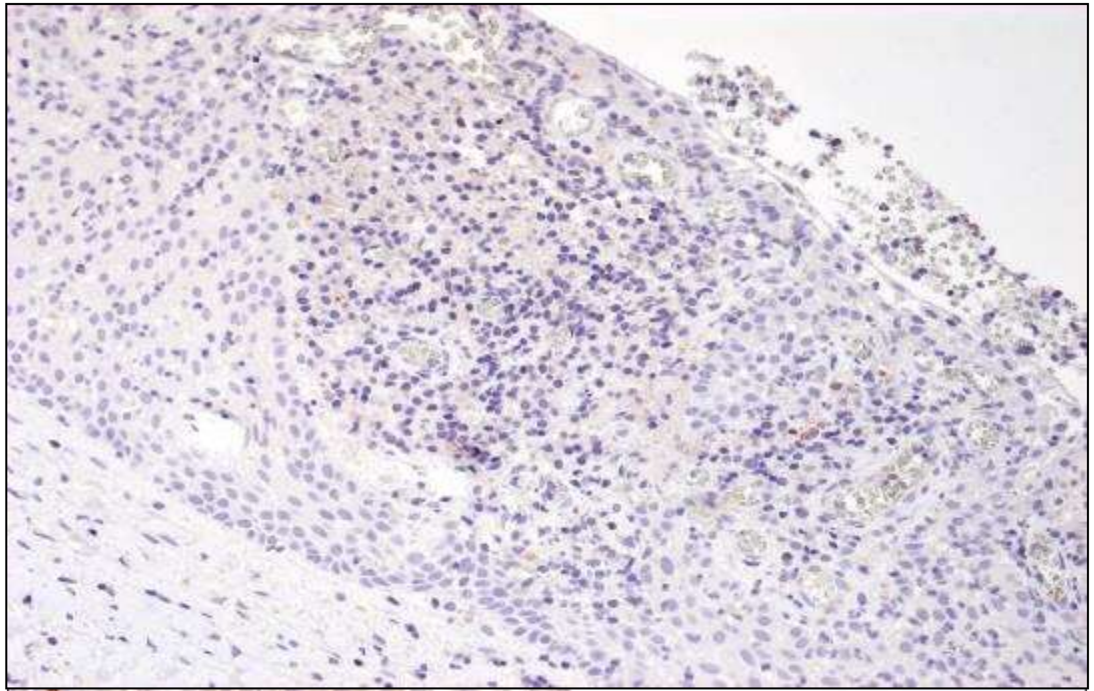

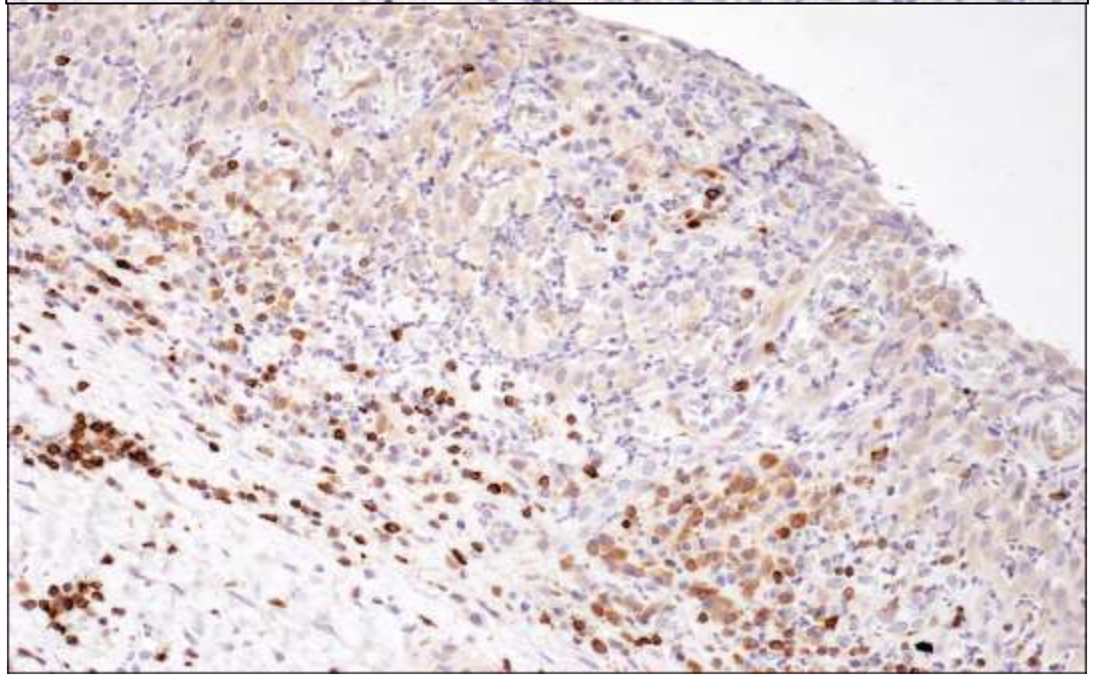

Case S4 – Conclusion

- H&E: relatively thick squamous epithelium without keratinization, parakeratosis, basal palisading or wavy surface; focally uneven epithelial stromal interface
- Predominately acute inflammation with focal chronic component
- Bcl2: negative, ignored focal weak (background like) transepithelial cytoplasmic staining
- CK17: negative
- Histopathological diagnosis: radicular cyst
- Combined clinical and pathological opinion: radicular cyst

Case S6

- 49-year-old female
- Dental hygiene/status: conserved, treated teeth
- OPT: radiolucent area in the region of teeth 12-23
- Operation: cystectomy, root resection of teeth 22, 21, 11, 12.
- Clinical diagnosis: keratocyst?

S6 – OPT (nothing obvious, postoperative radiograph)

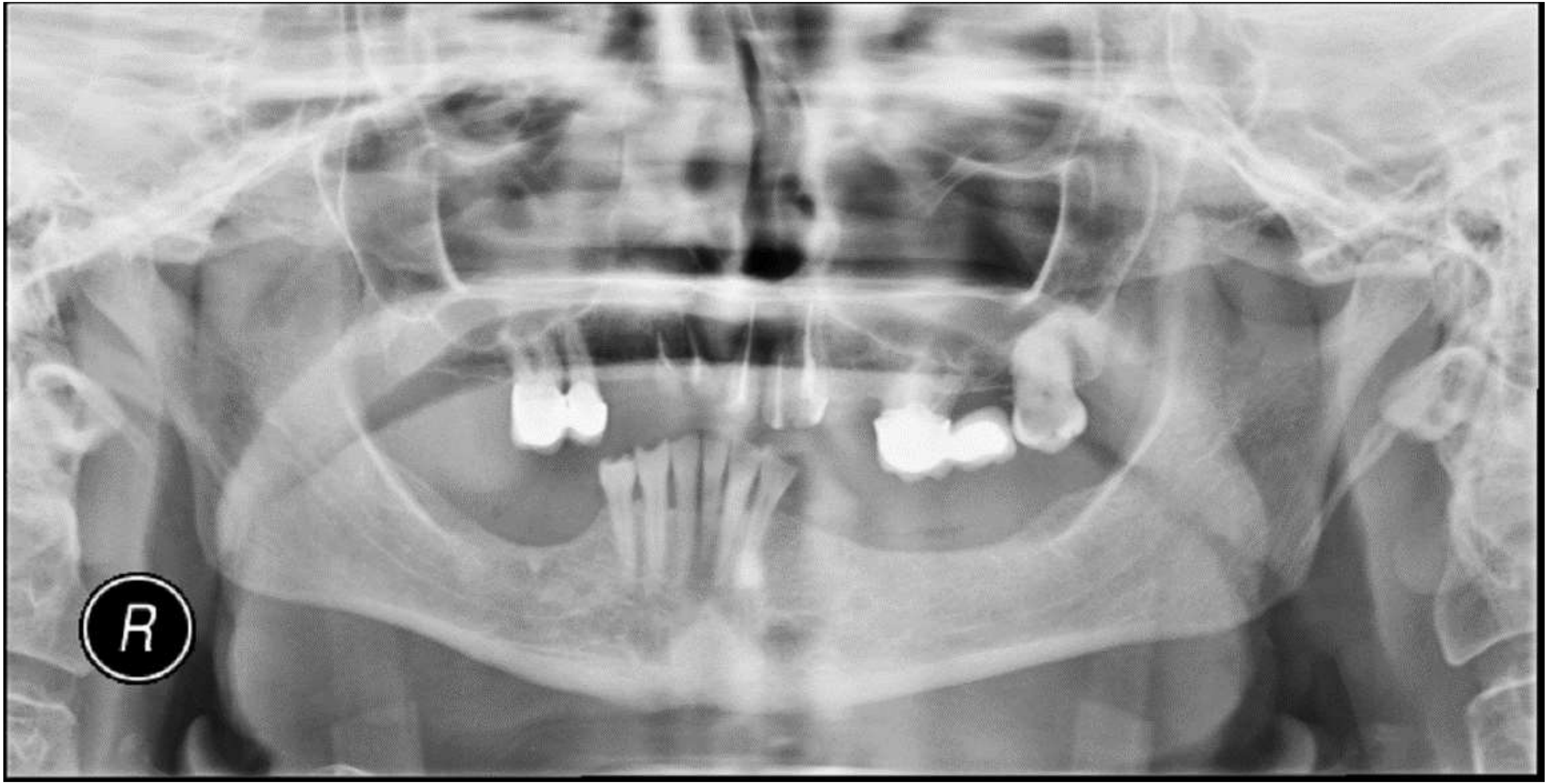

S6 – HE, CK17, bcl2 (x20)

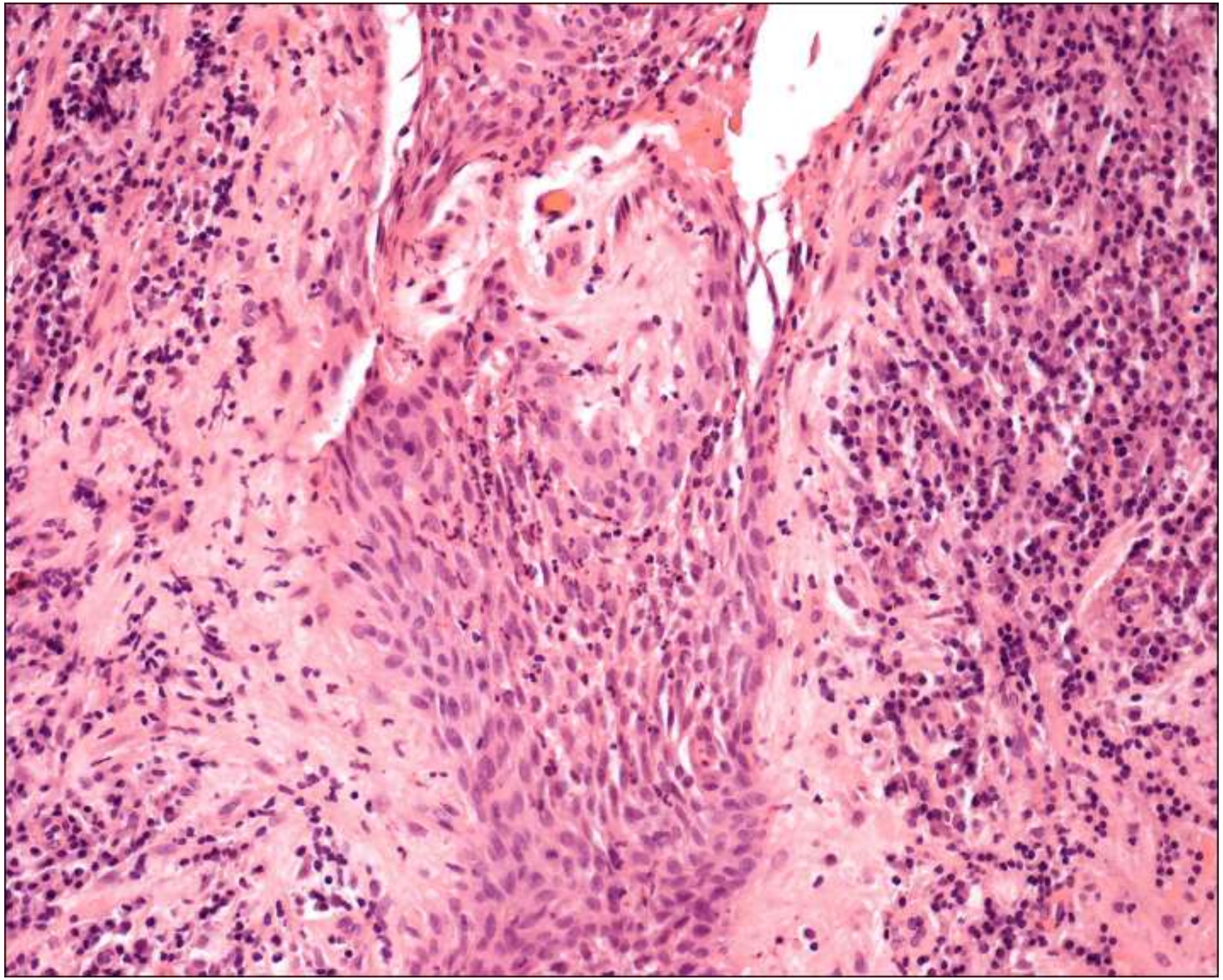

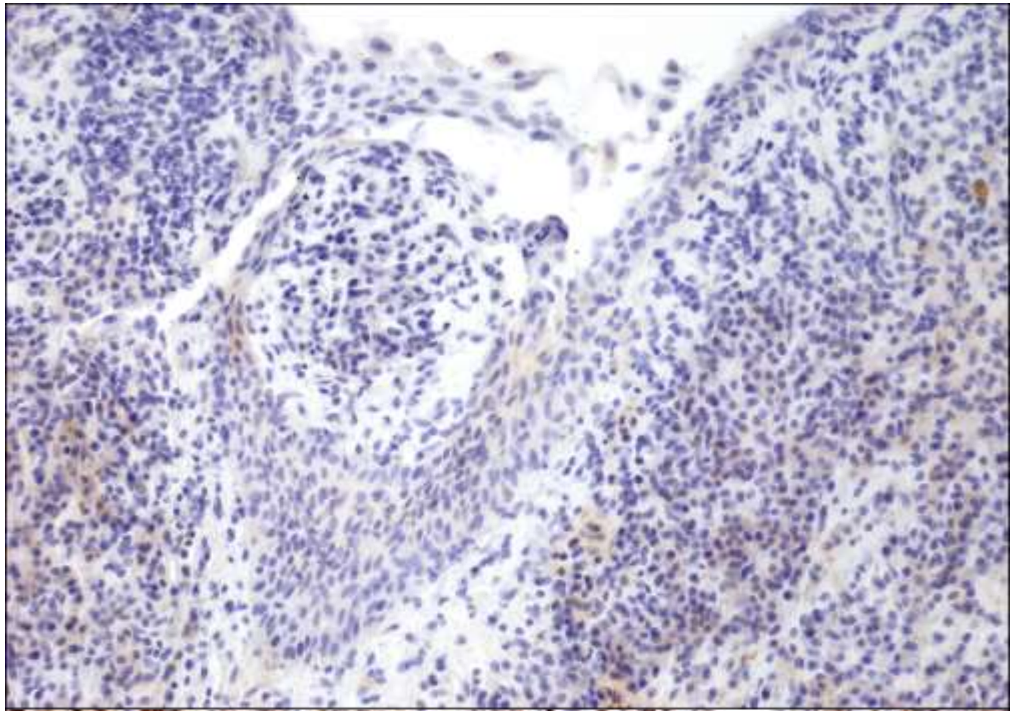

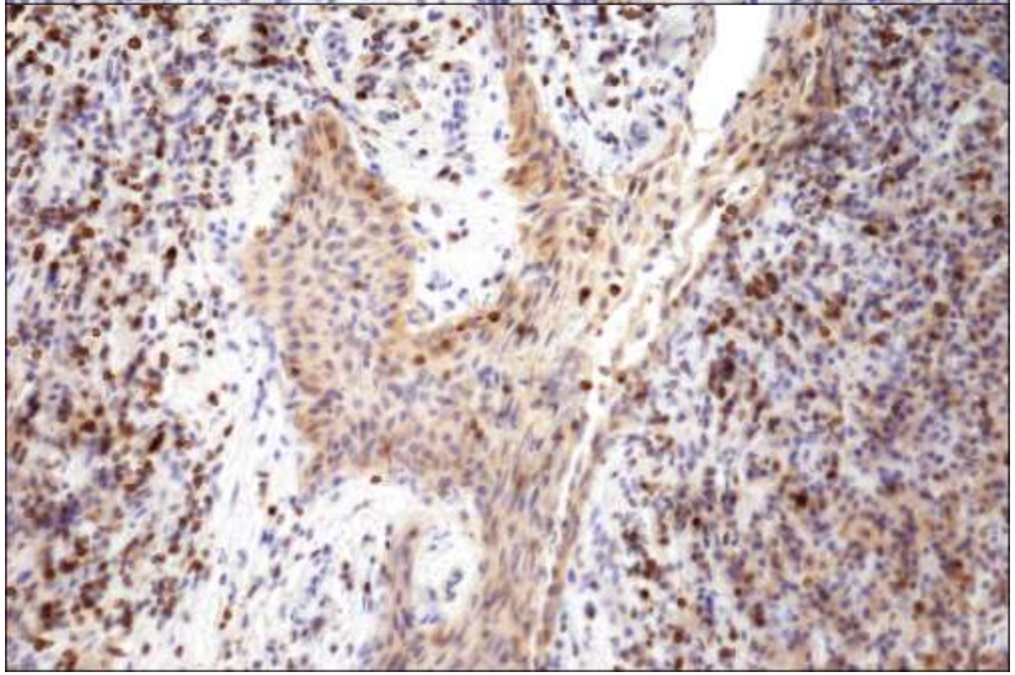

Case S6 – Conclusion

- H&E: relatively thick multi-layered squamous epithelium with focal keratinization without parakeratosis, basal palisading and wavy surface; uneven epithelial stromal interface; largely denuded surface
- Significant chronic inflammation is present with acute component; cholesterol clefts
- Bcl2: weak (ignorable) transepithelial cytoplasmic expression
- CK17: negative
- Histopathological diagnosis: radicular cyst
- Combined clinical and pathological opinion: cannot be specified; inflammatory/inflamed odontogenic cyst N.O.S.

Case S7

- 33-year-old male
- Dental hygiene/status: neglected, missing, partly treated teeth
- OPT: left inferior quadrant radiolucent area
- Operation: cystectomy
- Clinical diagnosis: keratocyst?

S7 - OPT

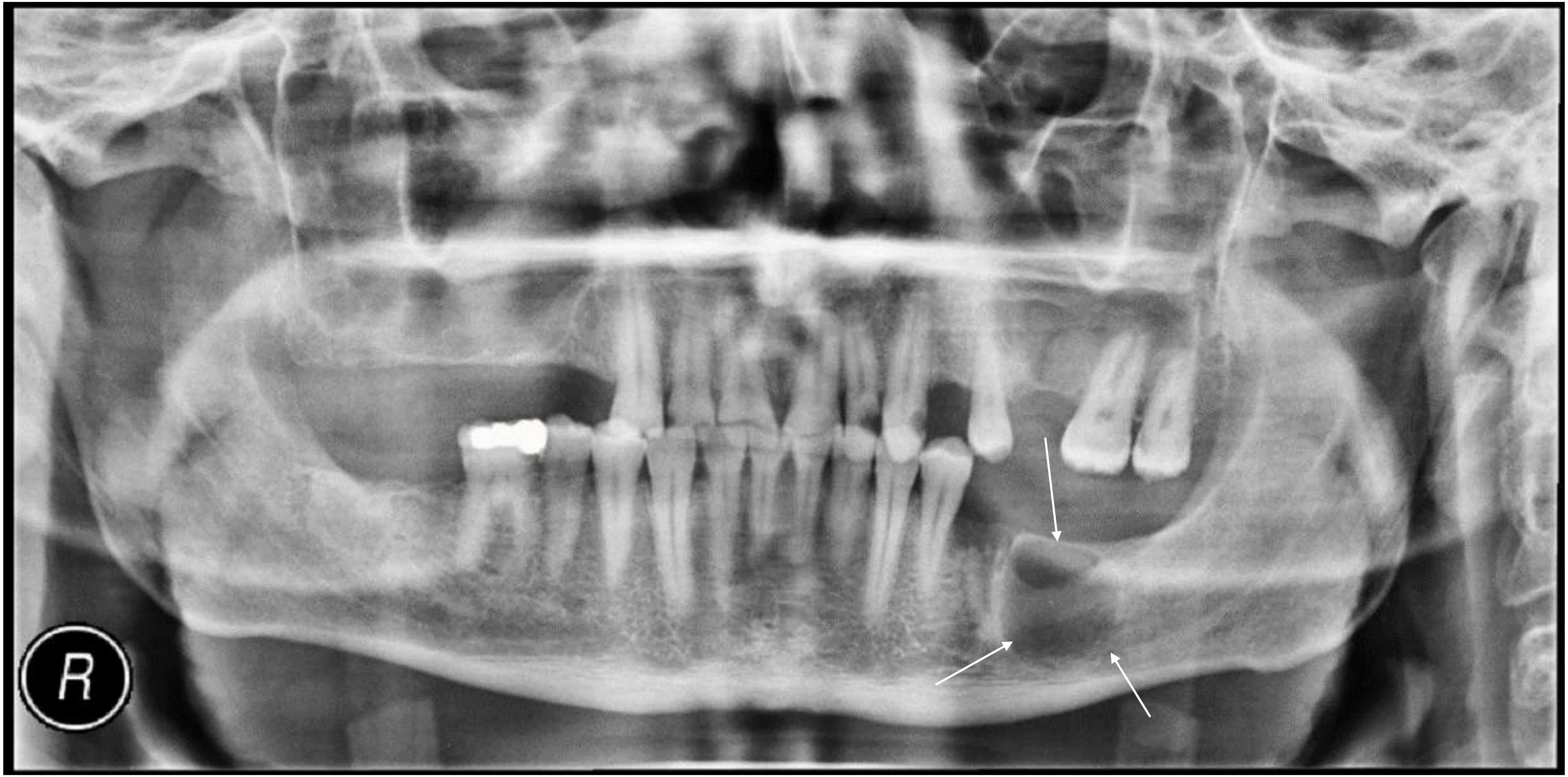

S7 – HE, CK17, bcl2 (x20)

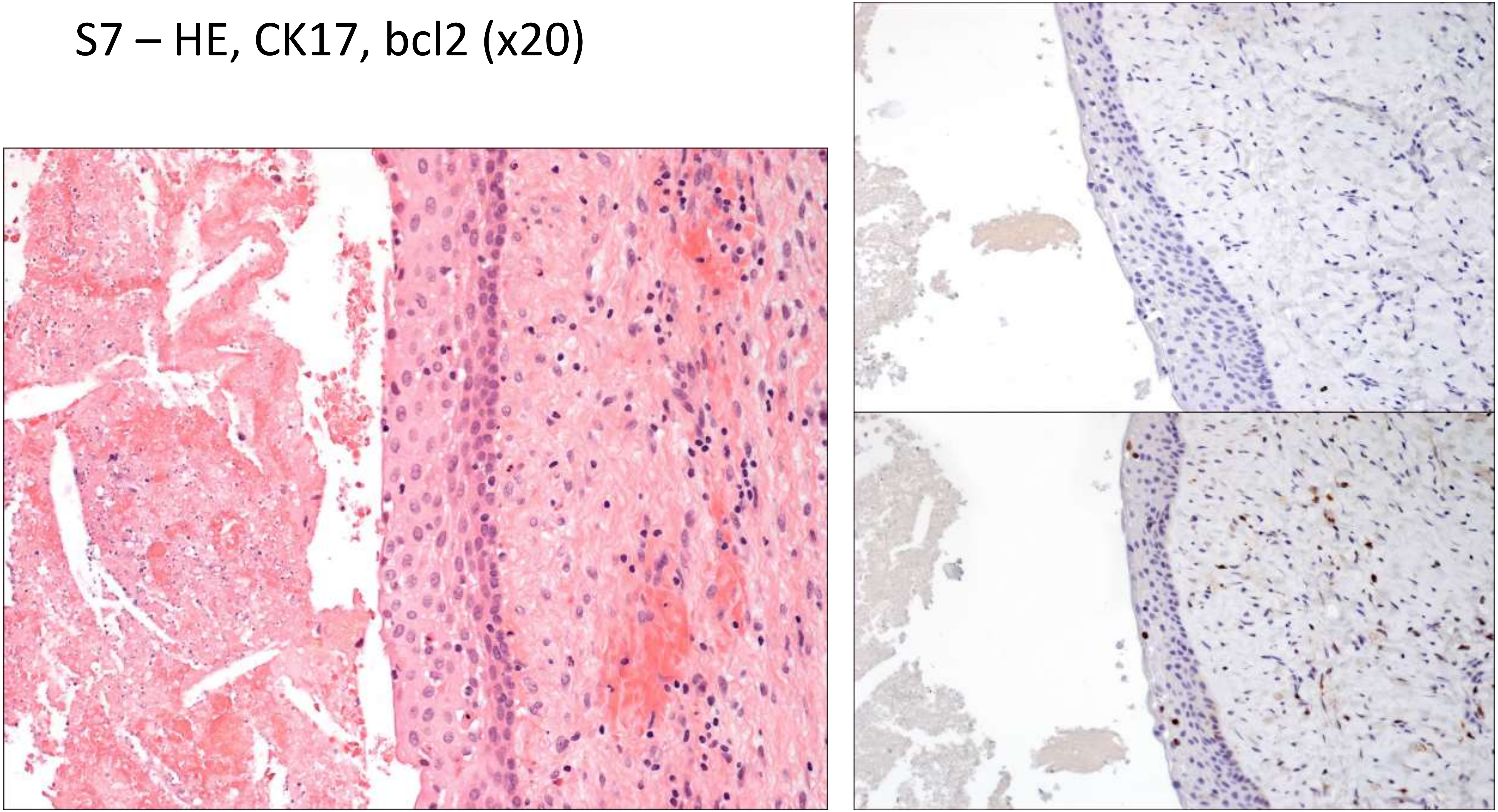

S7 – HE, mucous metaplasia (x20)

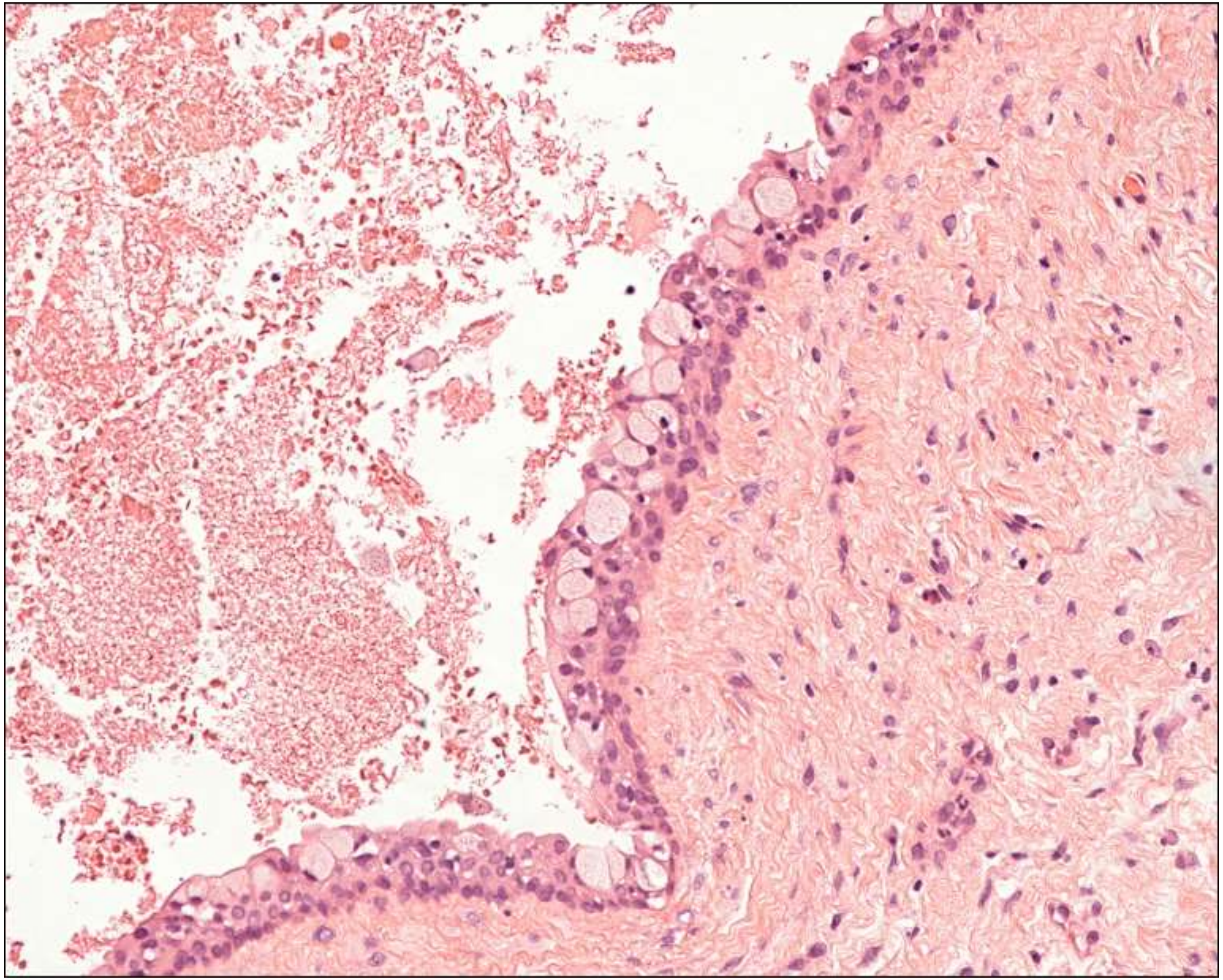

Case S7 Conclusion

- H&E: thin squamous epithelium without keratinization, parakeratosis, basal palisading or wavy surface; even epithelial stromal interface; mucous metaplasia
- No inflammation in the cyst wall, but cholesterol granuloma present
- Bcl2: negative
- CK17: negative
- Histopathological diagnosis: follicular cyst
- Combined clinical and pathological opinion: most consistent with residual cyst

Case S8

- 47-year-old male
- Dental hygiene/status: mandible prognathism, partly maintained, treated teeth
- OPT: radiolucent, cystic area affecting teeth 12, 11, 21, 22, 23
- Operation: cystectomy, root resection of teeth 12, 11, 21, augmentation with own iliac bone, sinus lifting
- Clinical diagnosis: keratocyst?

S8 – OPT (no evidence of the cyst, postoperative radiograph)

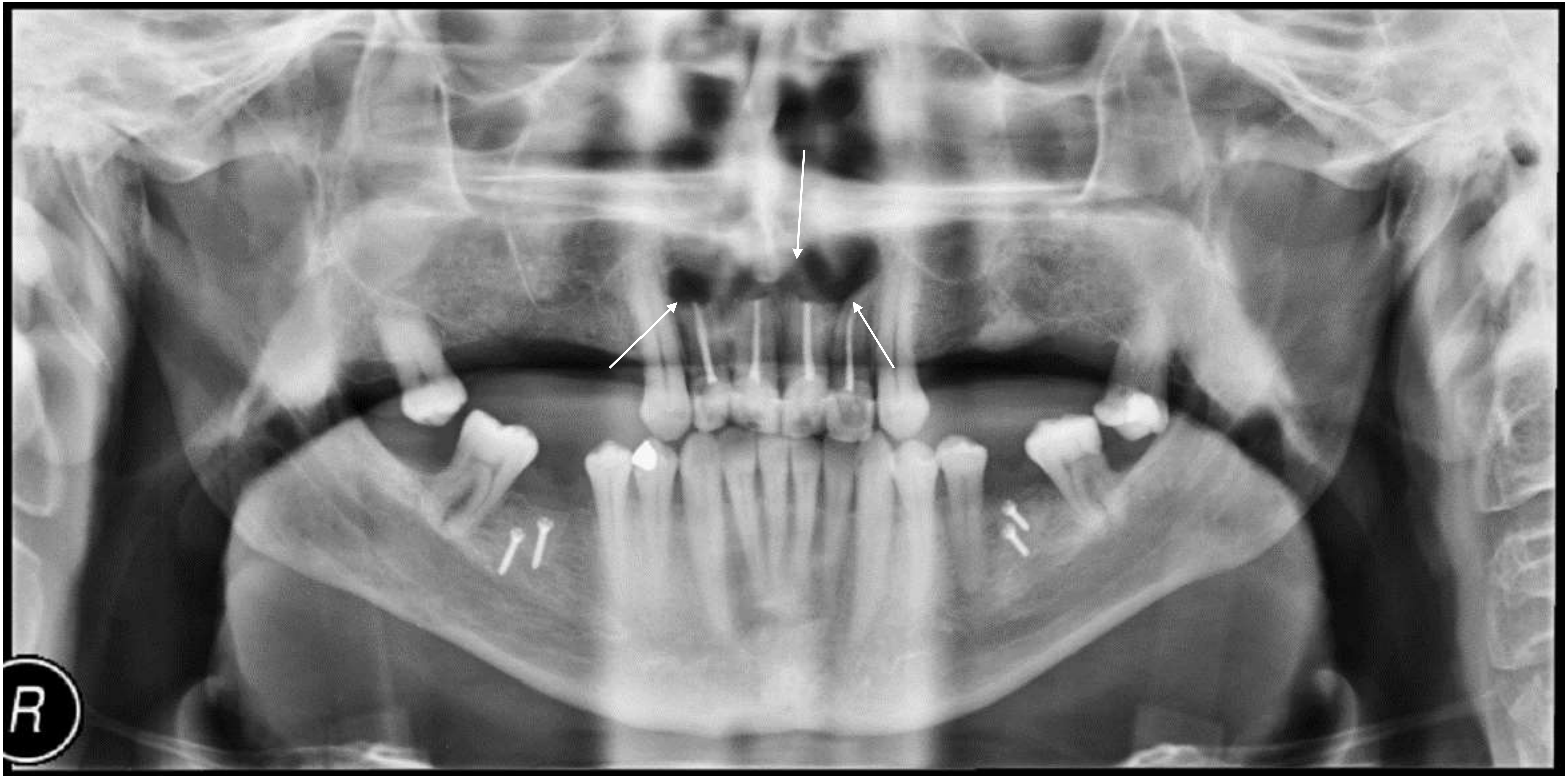

S8 – HE, CK17, bcl2 (x20)

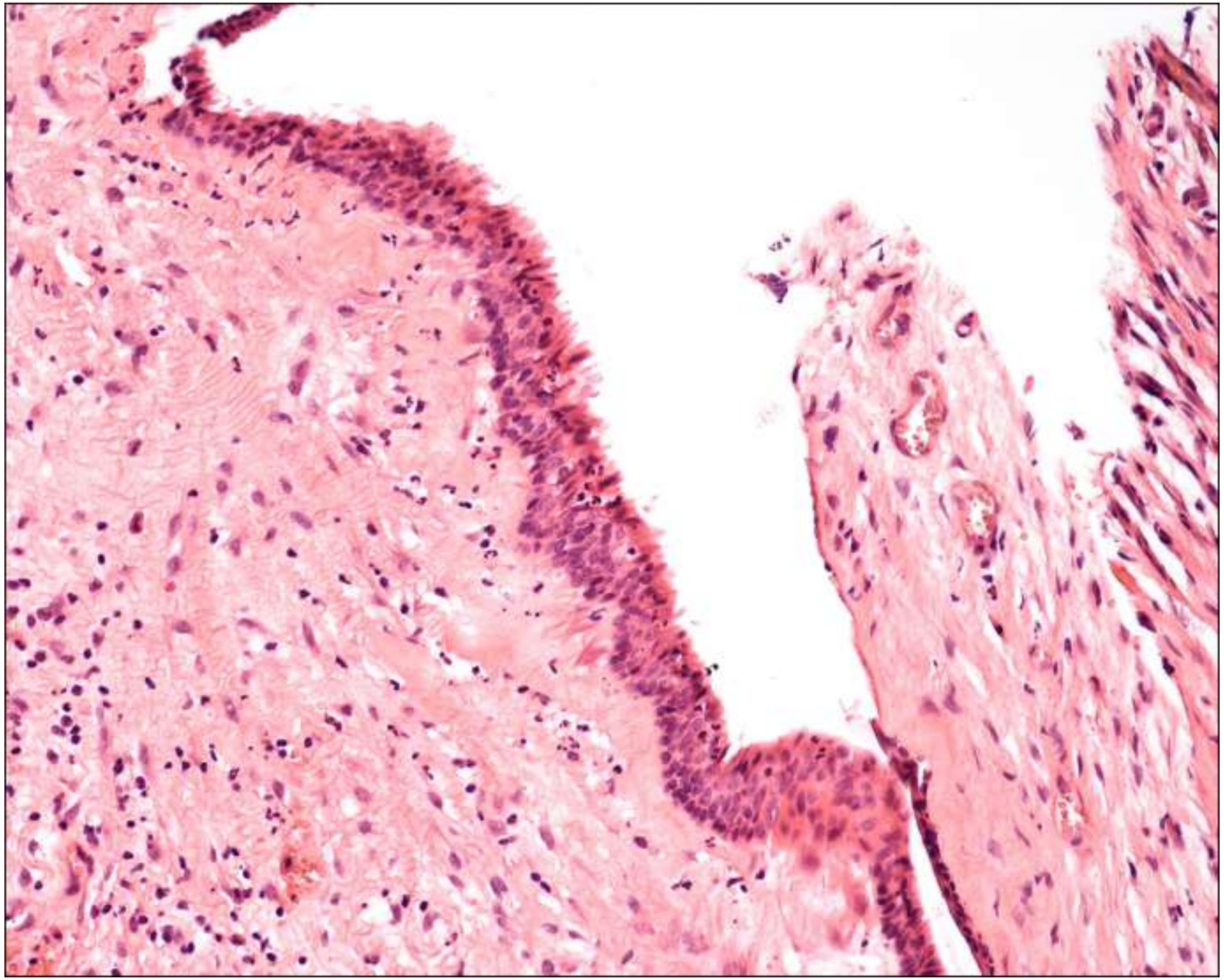

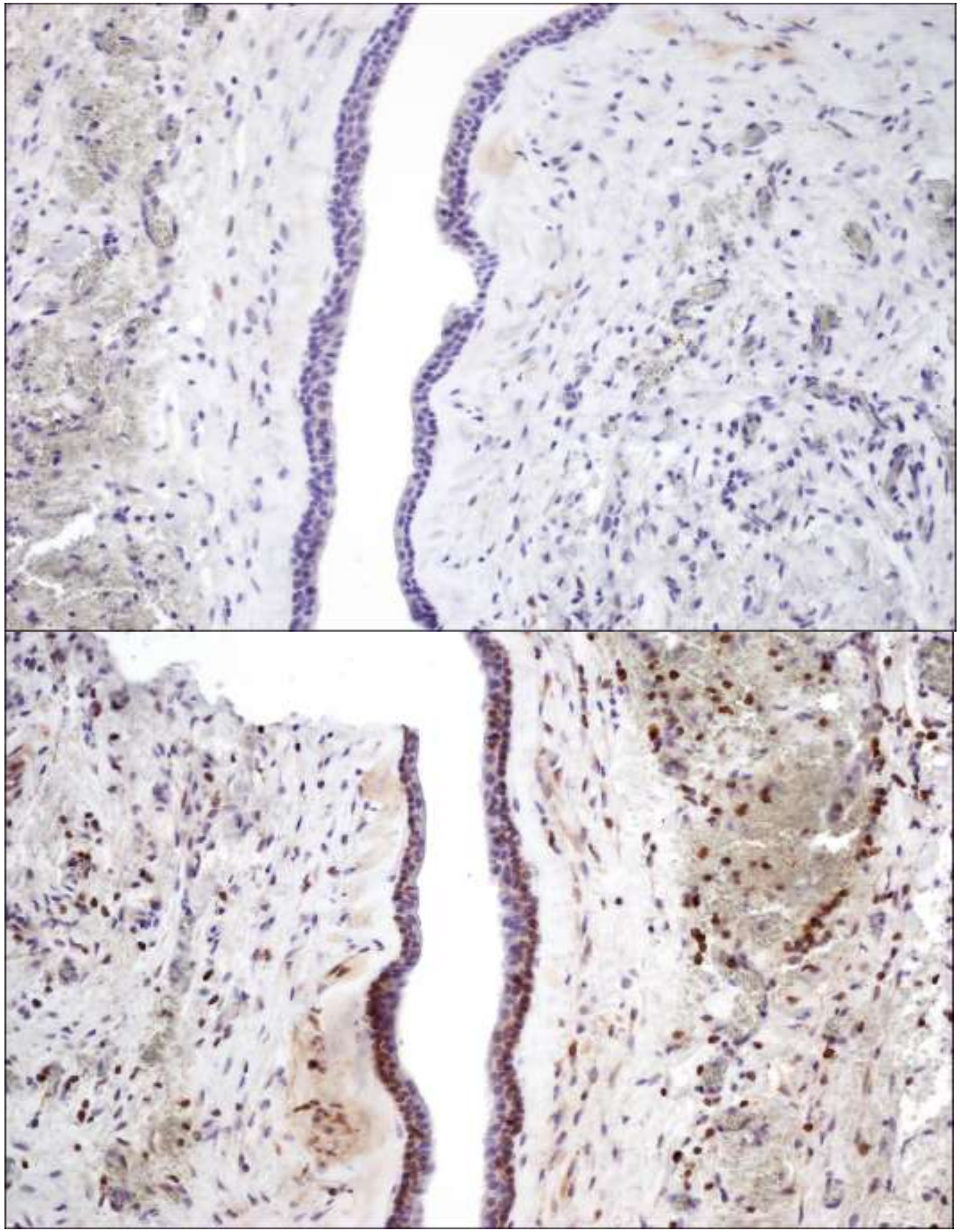

S8 – CK17 (x40)

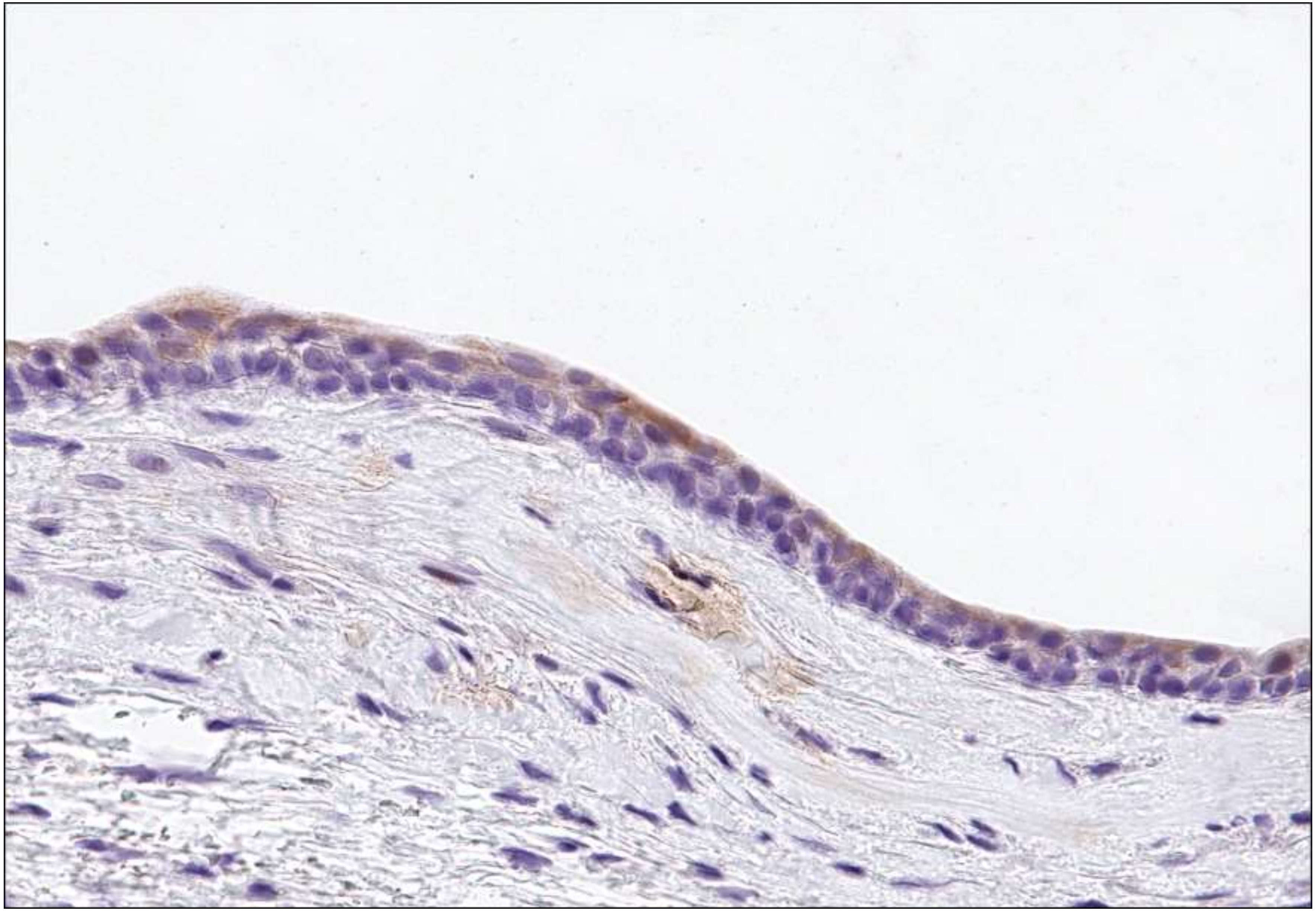

Case S8 – Conclusion

- H&E: thin squamous epithelium without keratinization, parakeratosis, basal palisading or wavy surface; even epithelial stromal interface
- Mild chronic inflammation
- Bcl2: moderate to marked basal epithelial expression
- CK17: dominantly negative, focal superficial staining
- Histopathological diagnosis: follicular cyst
- Combined clinical and pathological opinion: cannot be specified; developmental or mature inflammatory cyst N.O.S.

Case S9

- 49-year-old female
- Dental hygiene/status: partly missing, partly maintained, treated teeth
- OPT: radiolucent area around tooth 44
- Operation: removal of tooth 44, cystectomy
- Clinical diagnosis: odontogenic cyst

S9 - OPT

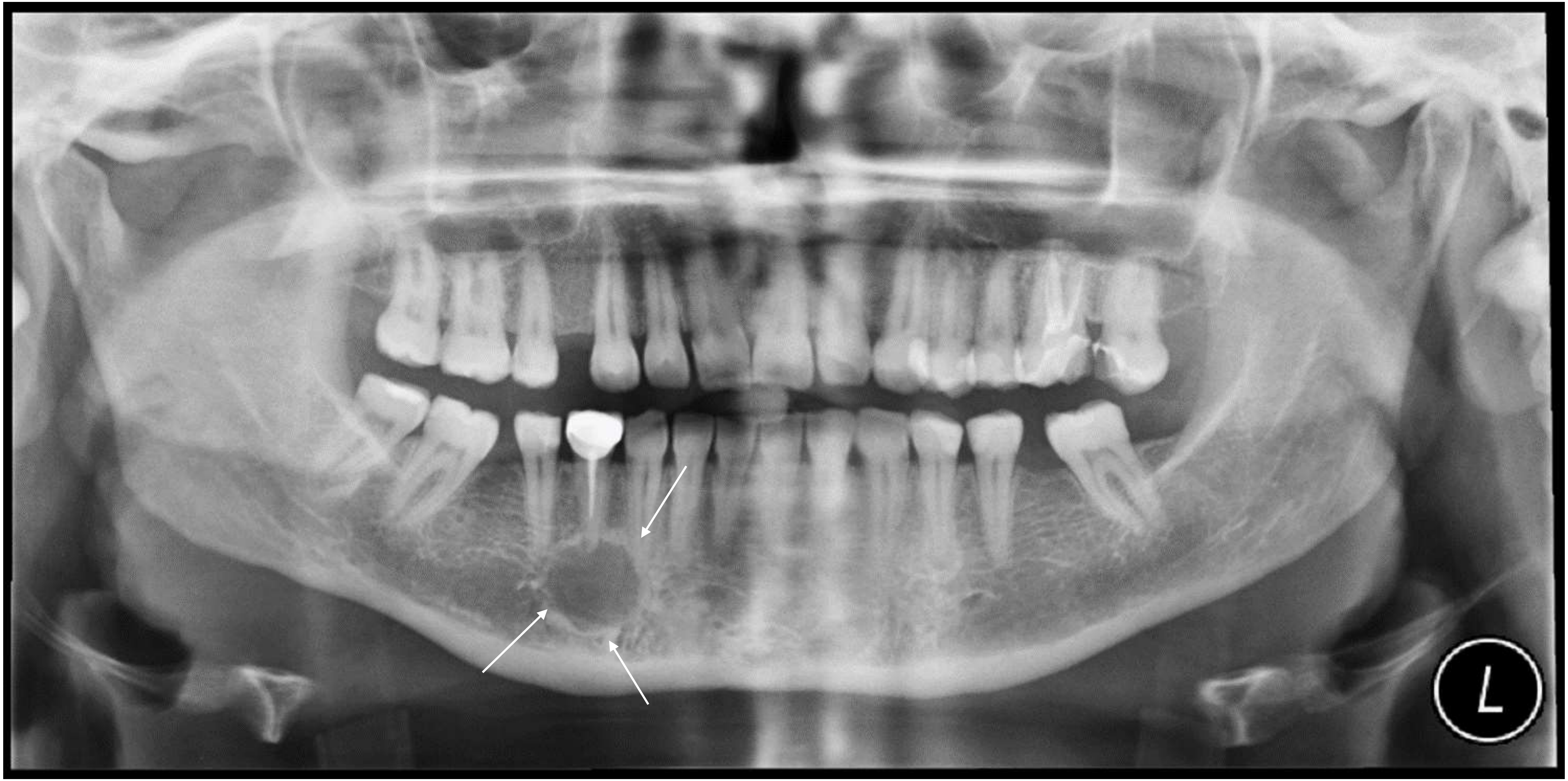

S9 – HE, CK17, bcl2 (x20)

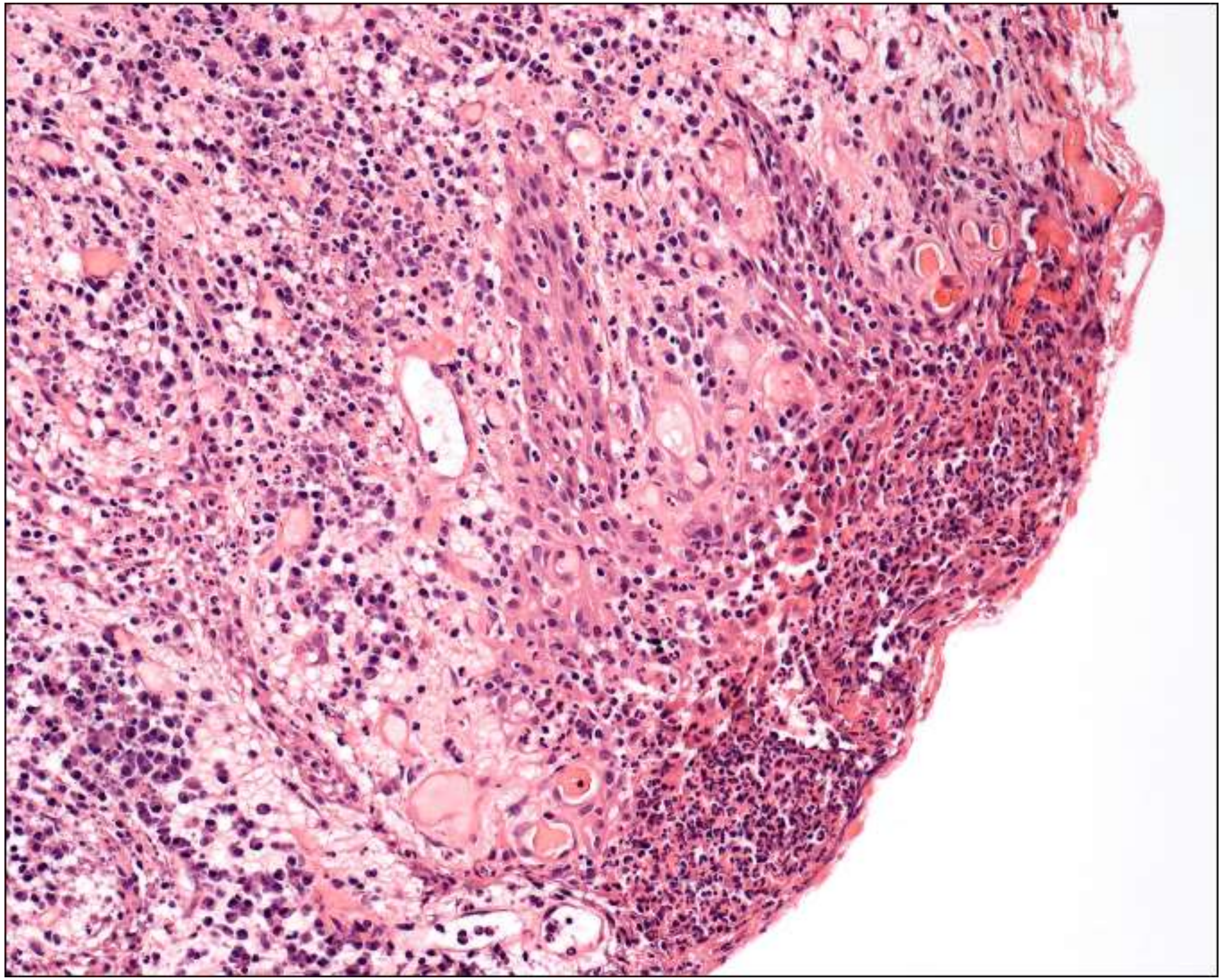

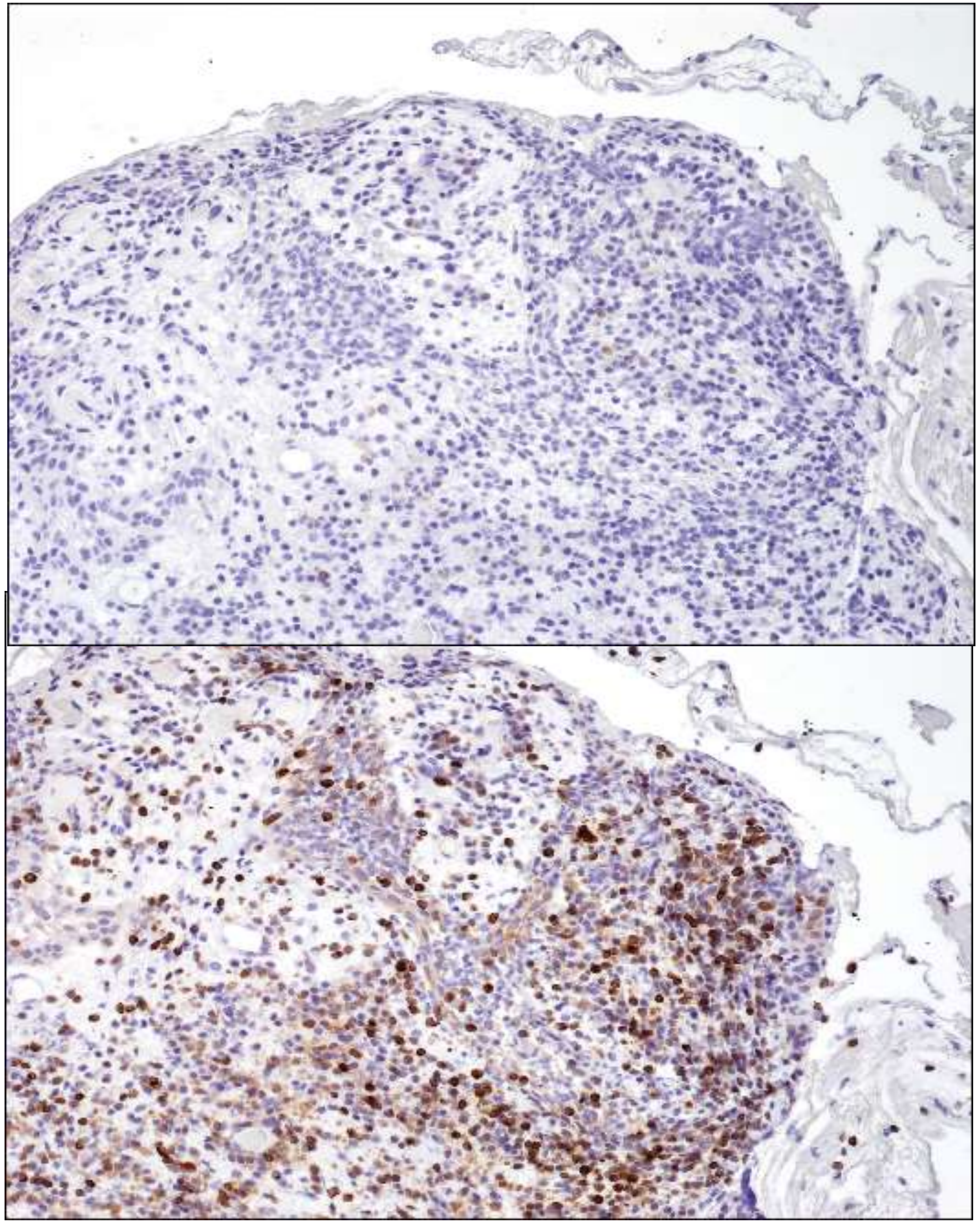

Case S9 – Conclusion

- H&E: thick squamous epithelium with spongiosis and without keratinization, parakeratosis, basal palisading or wavy surface; uneven epithelial stromal interface
- Significant acute and chronic inflammation
- BCL2: negative
- CK17: negative
- Histopathological diagnosis: radicular cyst
- Combined clinical and pathological opinion: radicular cyst

Case S10-14

- 11-year-old boy
- History: He was operated on due to cleft palate and cleft lip in 2009 and 2010. Testing for Gorlin-Goltz syndrome is in-progress.
- Dental hygiene/status: Neglected teeth.
- OPT: large multifocal radiolucent areas in the mandible.
- Operation: removal of cysts in area of teeth 13, 27, 37, 43 and 47 and removal of teeth 13, 27, 28, 37, 43 and 47
- Clinical diagnosis: keratocyst

S10 – HE (x15), CK17, bcl2 (x20)

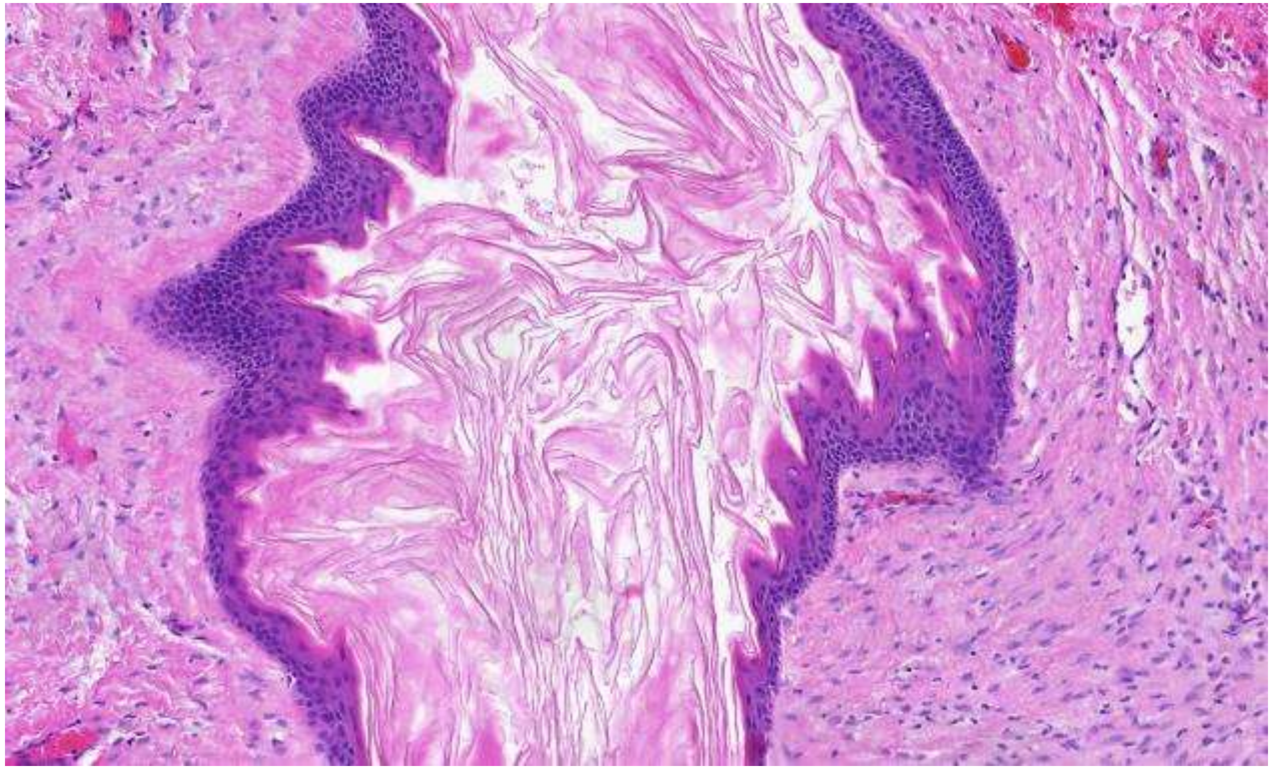

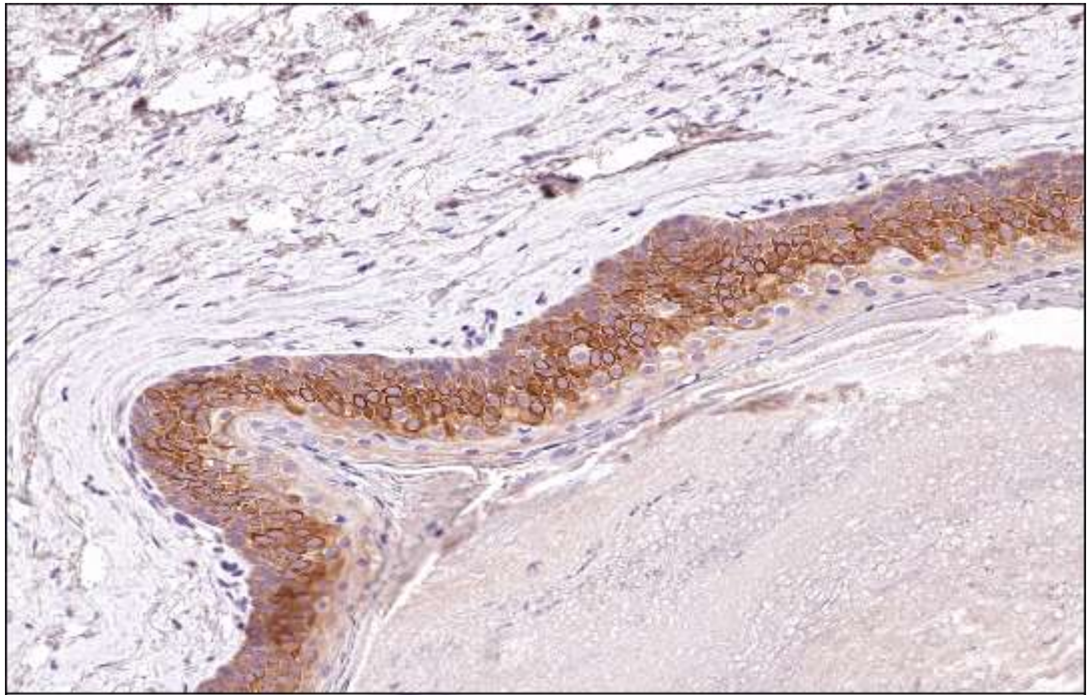

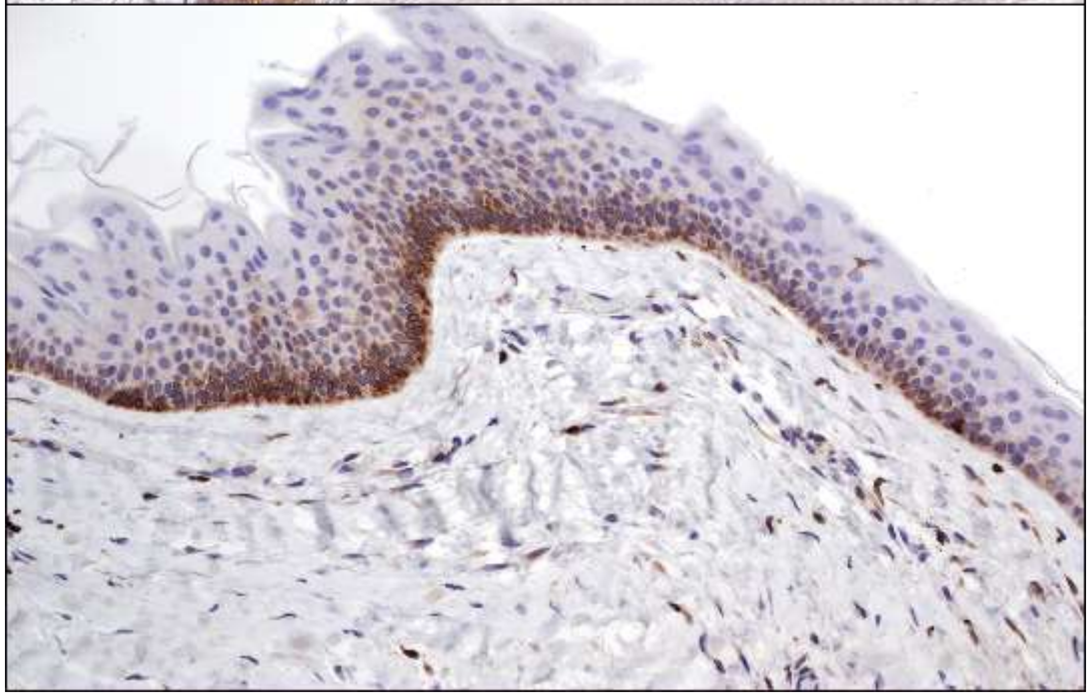

S10 – CK17 (x15) variability of staining pattern: transepithelial and tarnsepithelial with parakeratotic squames partly spared

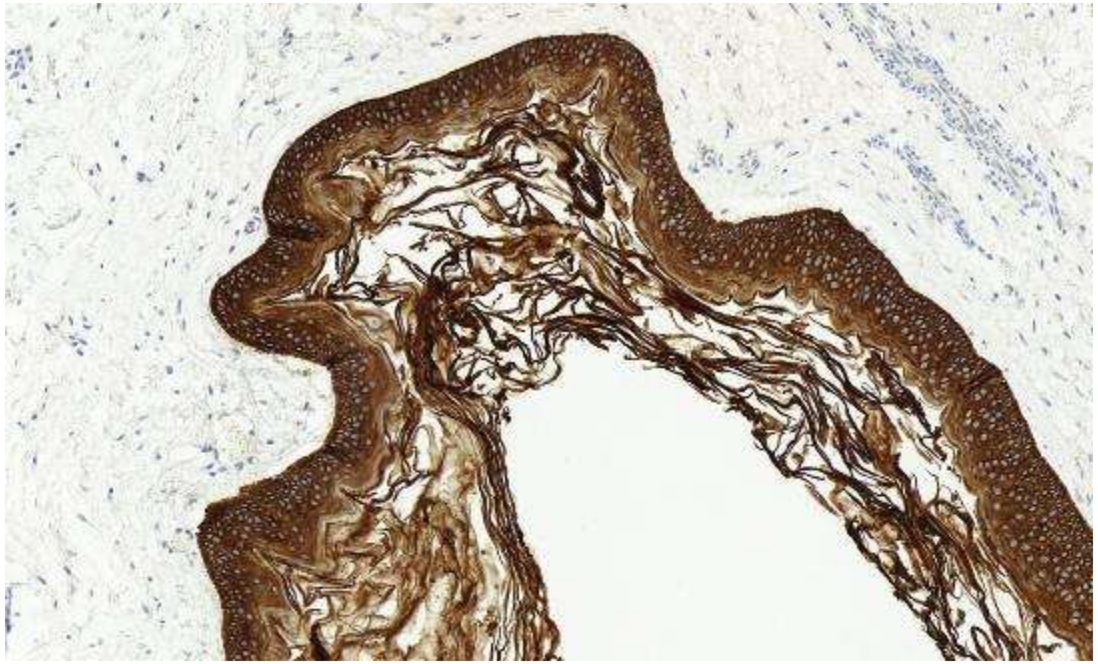

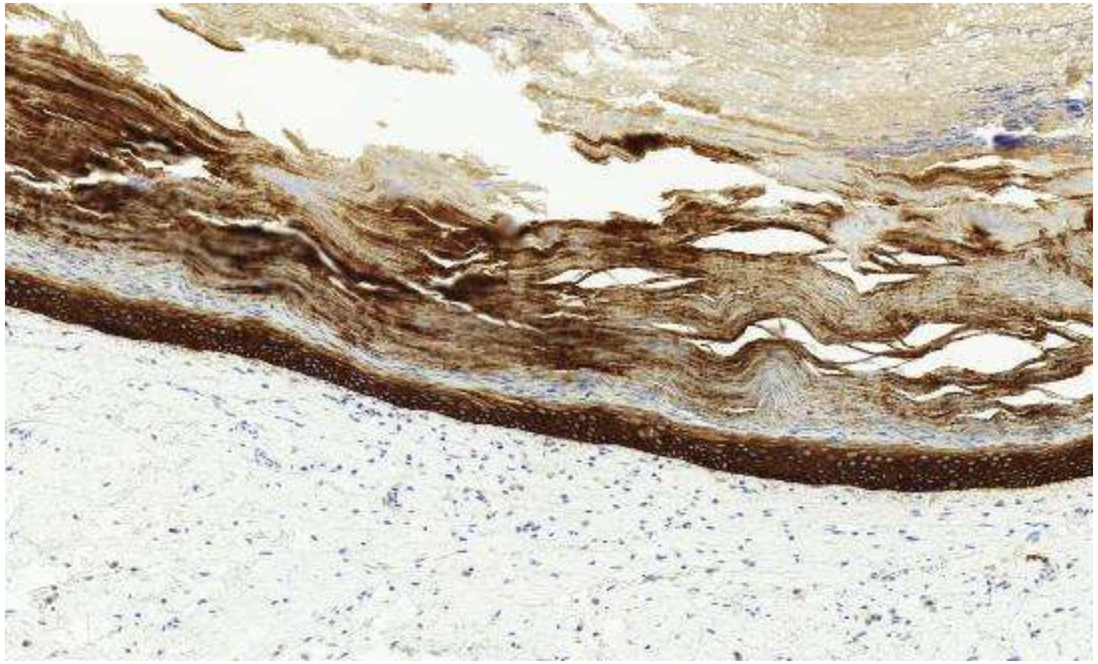

Case S10 – Conclusion

- H&E: thin parakeratotic epithelium with wavy surface and basal palisading; even epithelial stromal interface
- Inflammation absent
- BCL2: basal, suprabasal positivity
- CK17: transepithelial positivity, focal superficial sparing
- Histopathological diagnosis: OKC
- Combined clinical and pathological opinion: OKC (associated with the Gorlin-Goltz syndrome)

S11 - HE, CK17, bcl2 (x10)

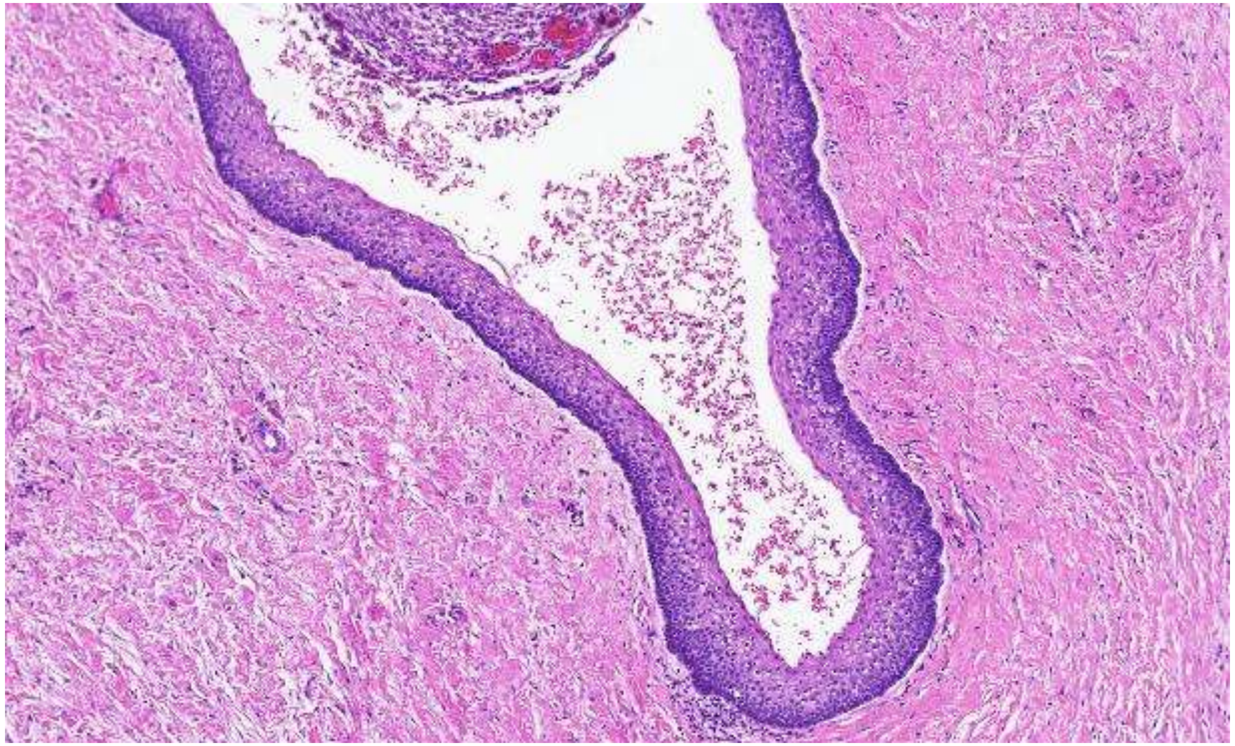

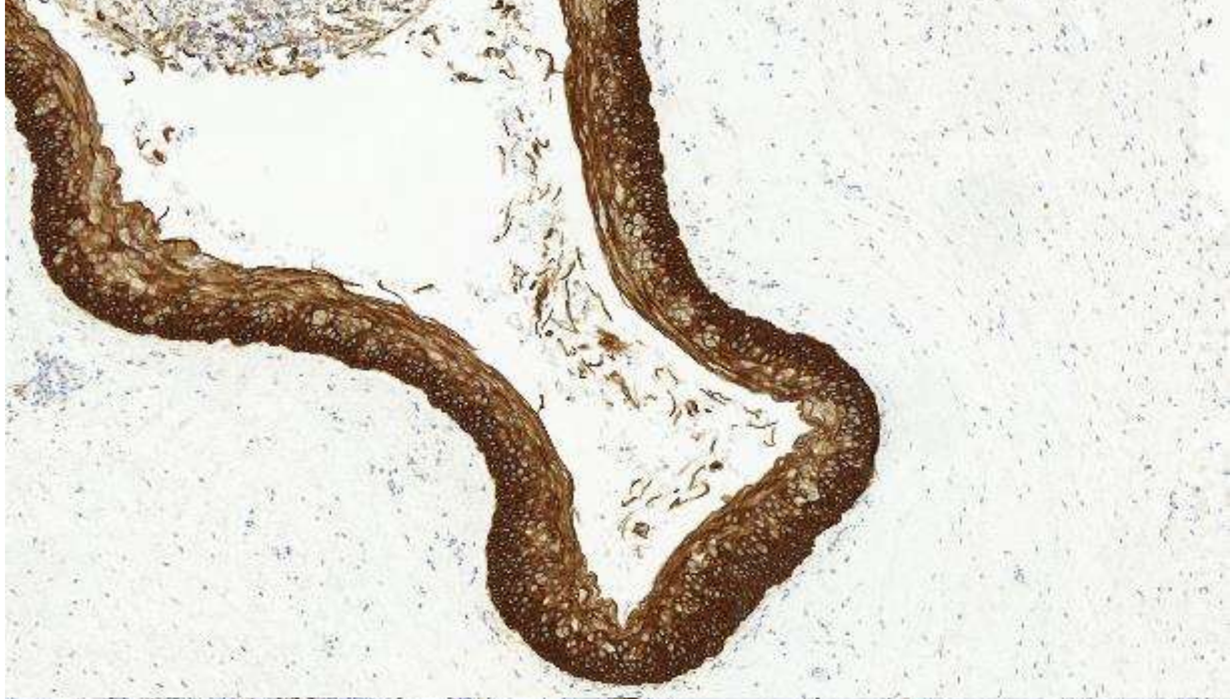

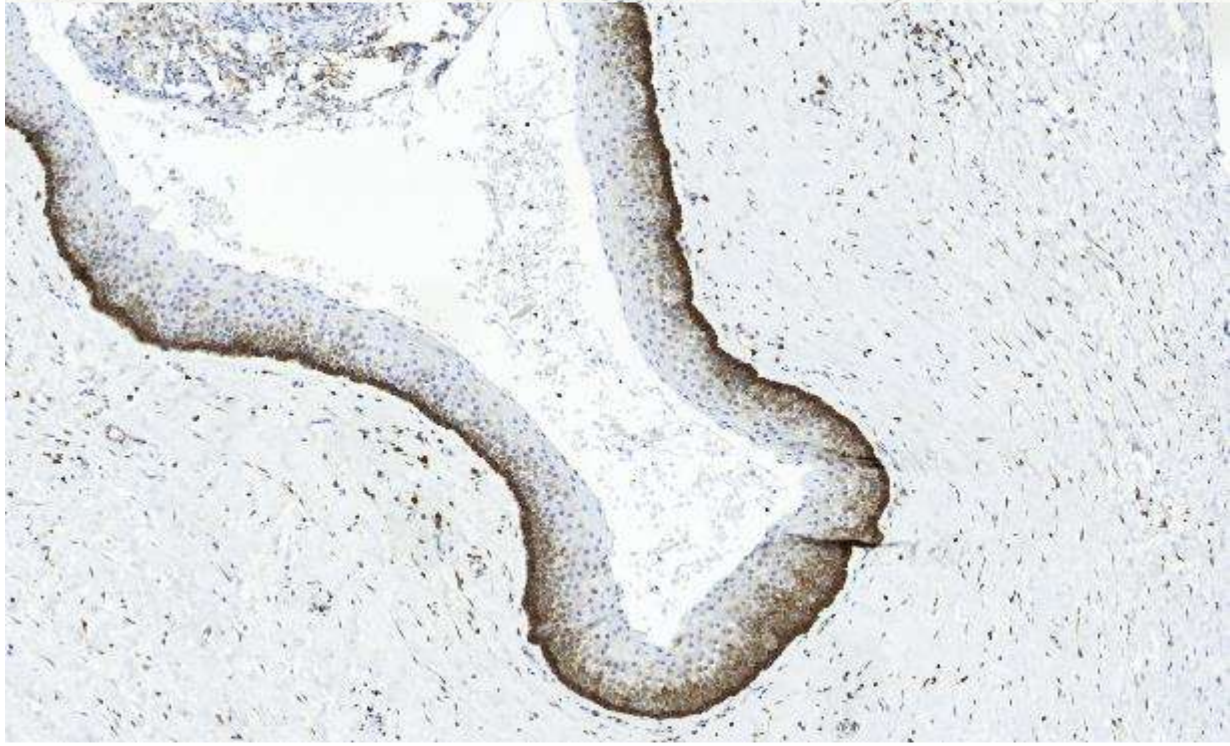

S11 - HE, CK17, bcl2 (x6)

Loss of transepithelial CK17 and basal bcl2 staining at site of inflammation associated altered squamous epithelium (*)

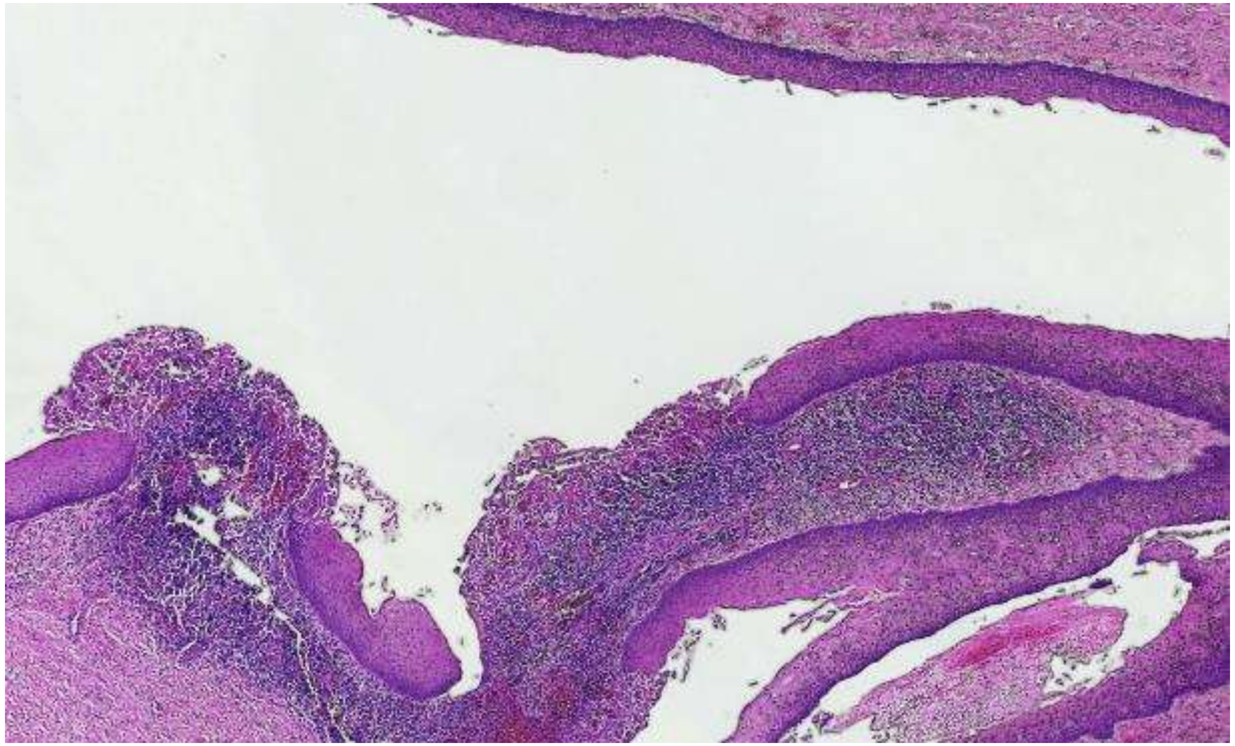

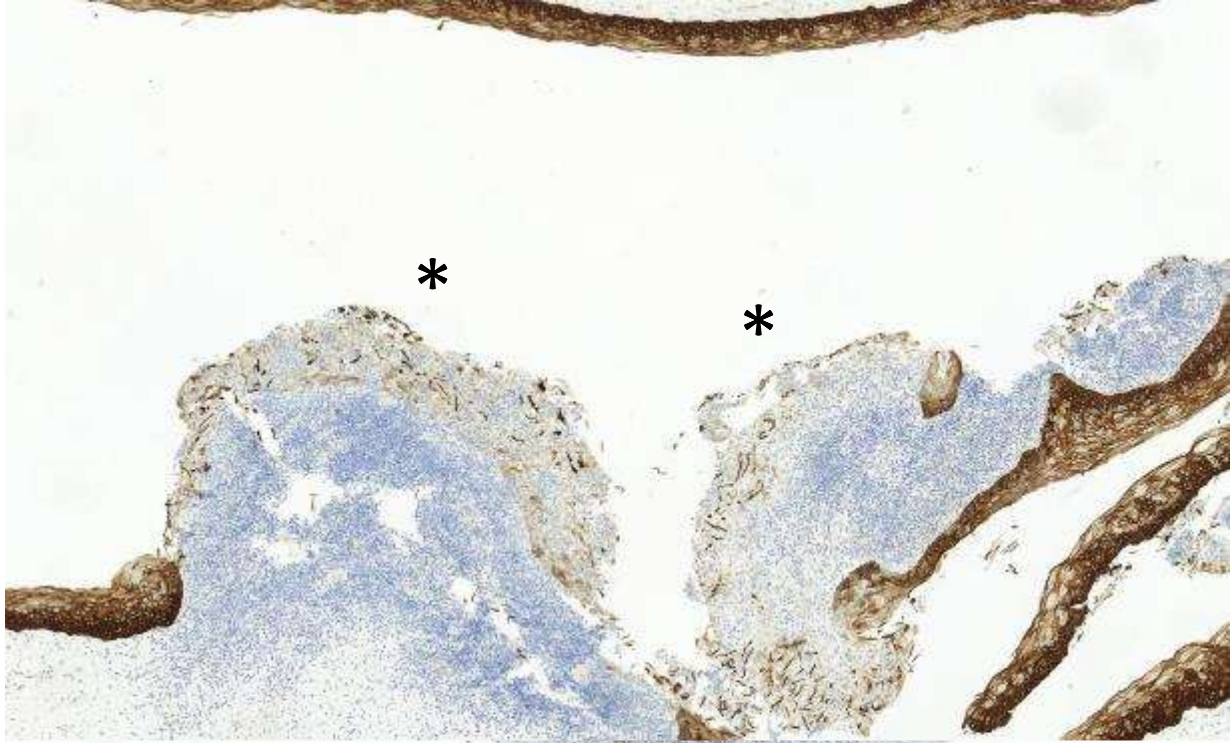

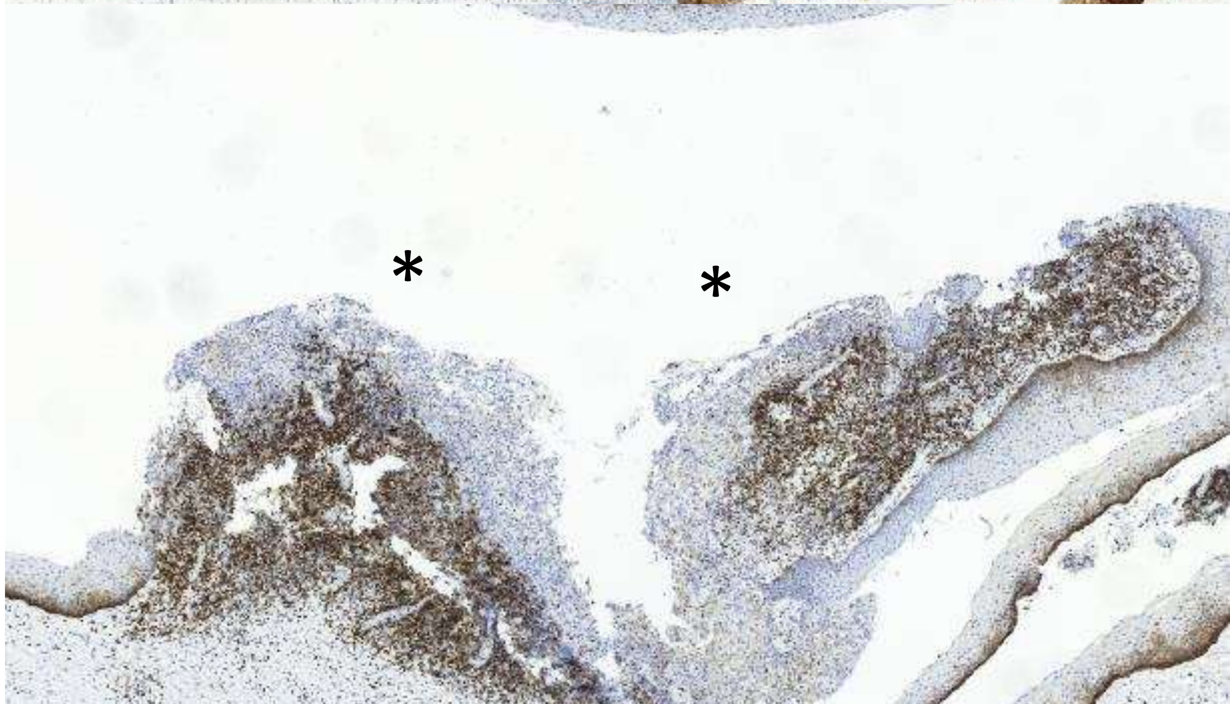

Case S11 – Conclusion

- H&E: thin parakeratotic epithelium with wavy surface and basal palisading; even epithelial stromal interface
- Inflammation absent, minimal focus of moderate chronic inflammation
- Bcl2: basal, suprabasal positivity, focal loss associated with inflammation associated altered epithelium
- CK17: transepithelial positivity, focal patchy positivity associated with inflammation associated altered epithelium
- Histopathological diagnosis: OKC
- Combined clinical and pathological opinion: OKC (associated with the Gorlin-Goltz syndrome)

S12 – HE (air bubble), CK17, bcl2 (x1)

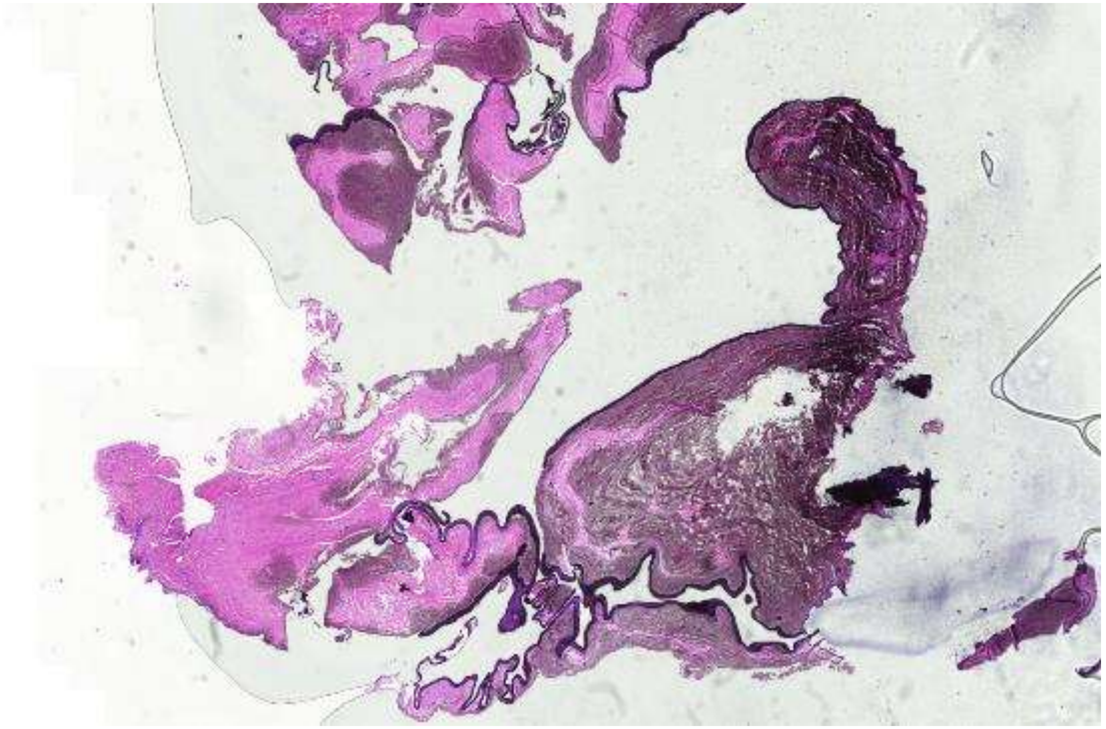

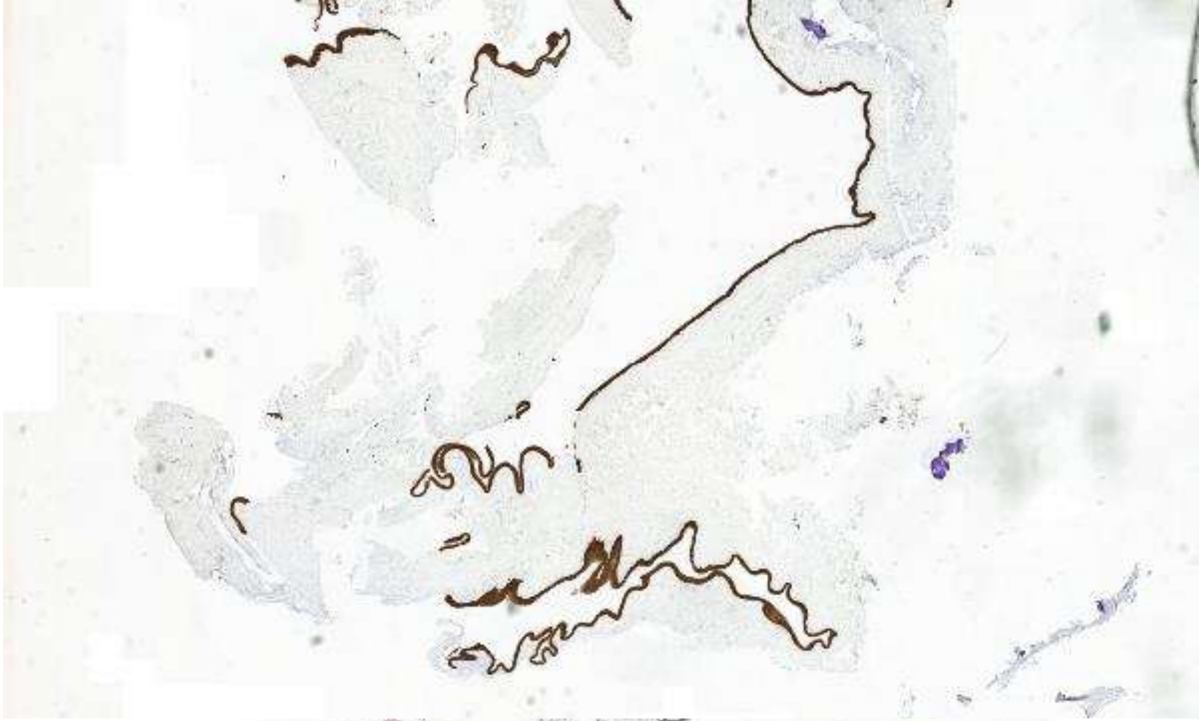

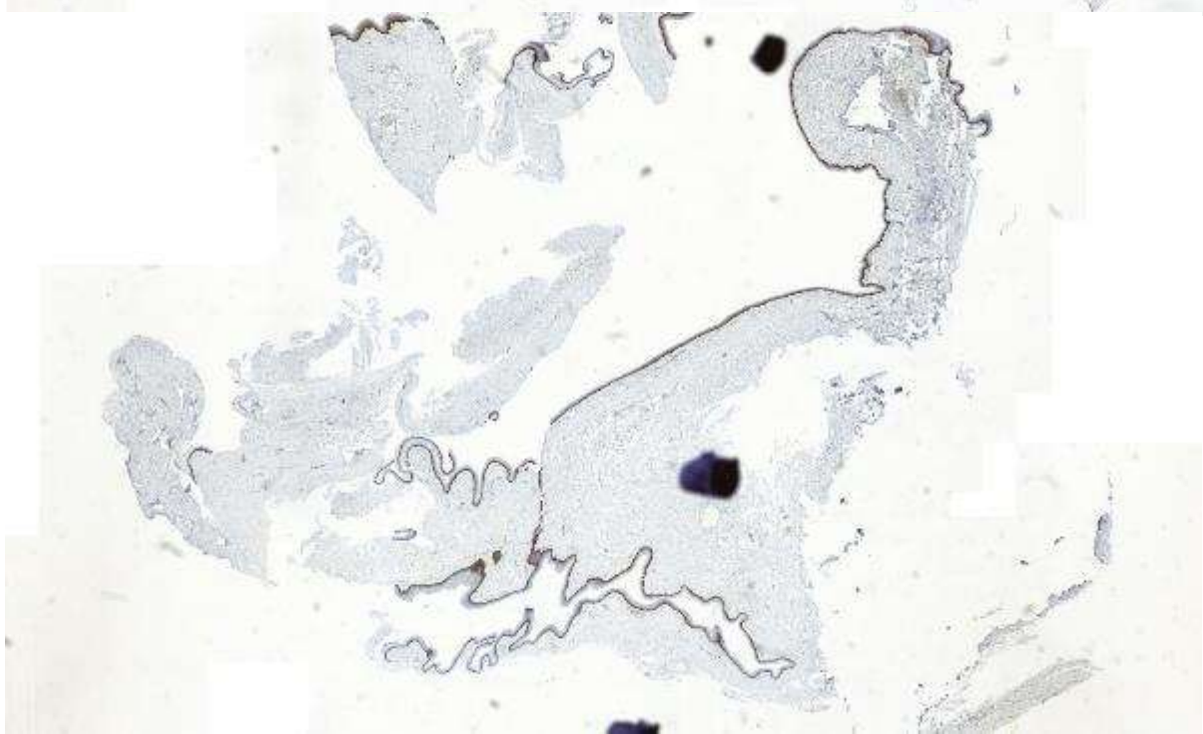

S12 – HE, CK17, bcl2 (x10)

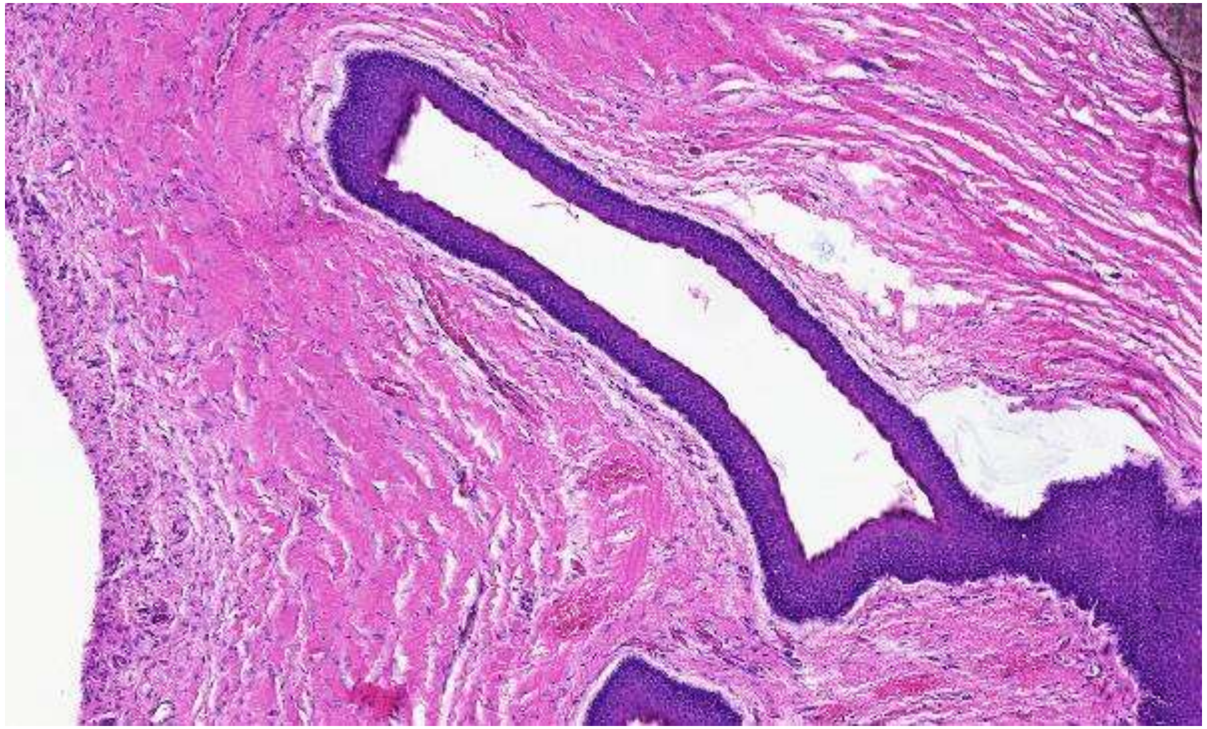

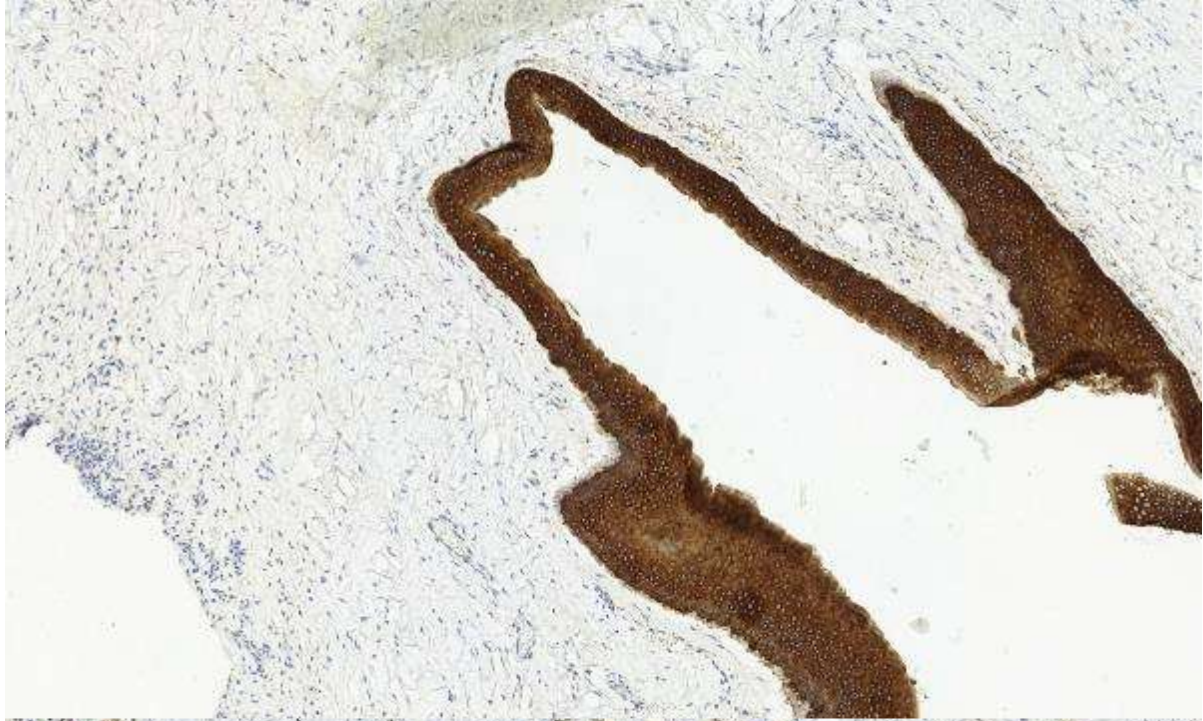

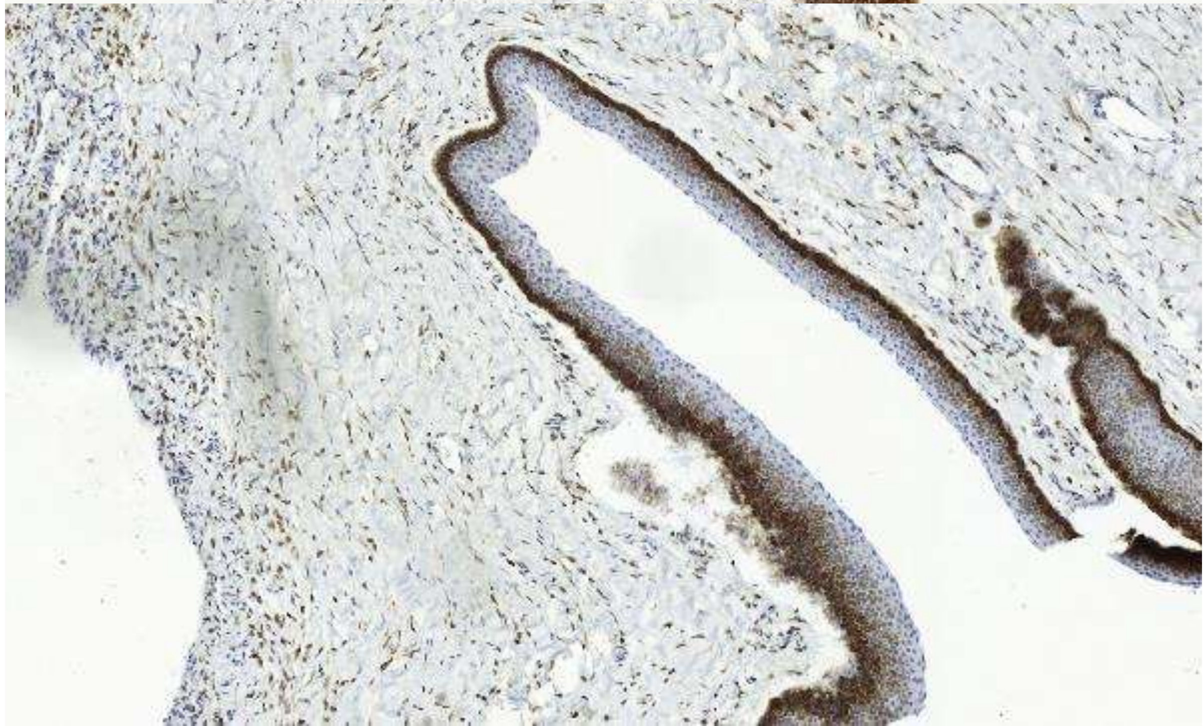

Case S12 – Conclusion

- H&E: thin parakeratotic epithelium with wavy surface and basal palisading; even epithelial stromal interface
- Inflammation absent
- Bcl2: basal (suprabasal) positivity
- CK17: transepithelial
- Histopathological diagnosis: OKC
- Combined clinical and pathological opinion: OKC (associated with the Gorlin-Goltz syndrome)

S13 – HE, CK17, bcl2 (x20)

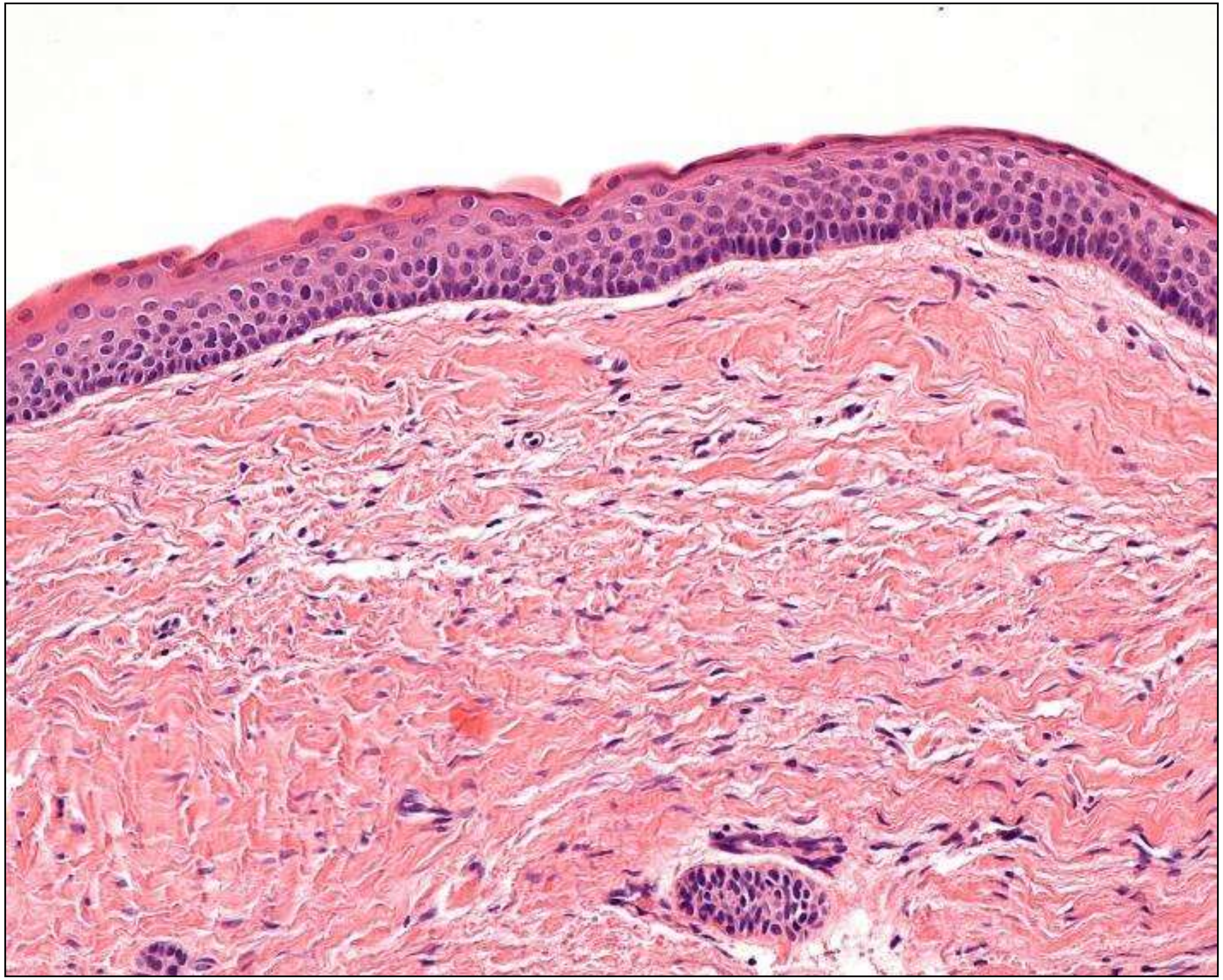

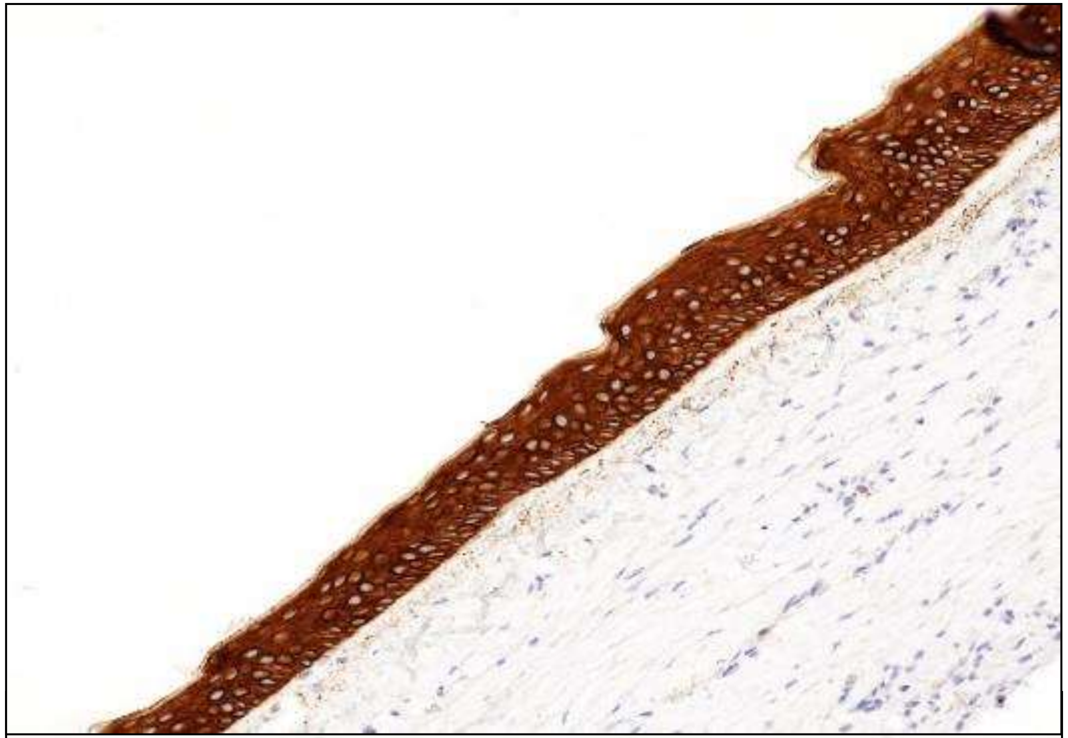

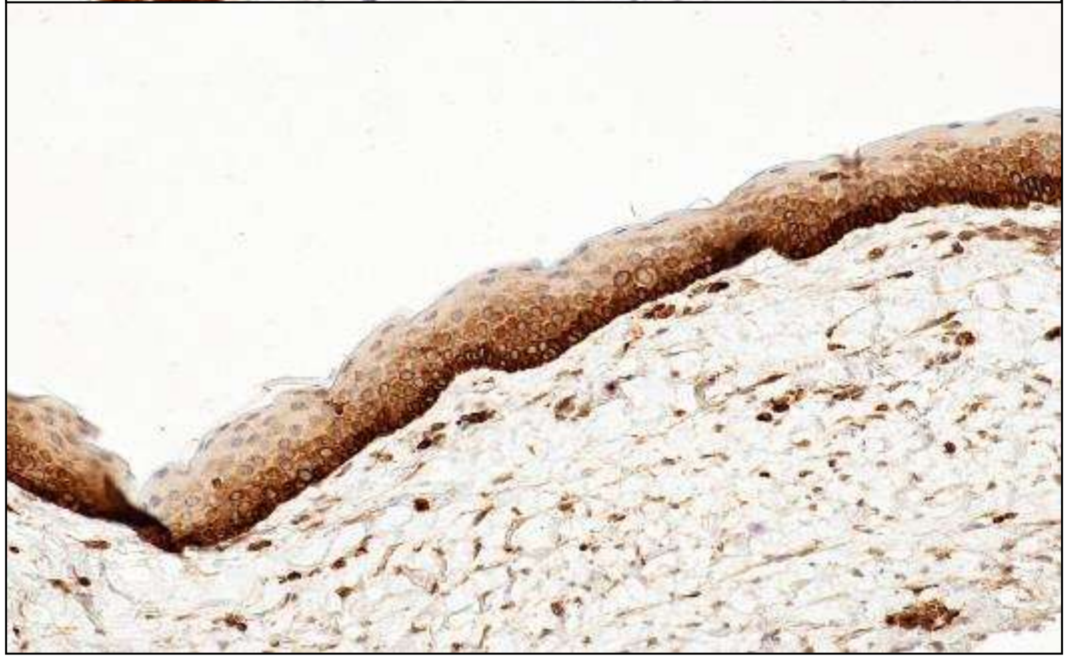

S13 – HE, CK17, bcl2 (x8)

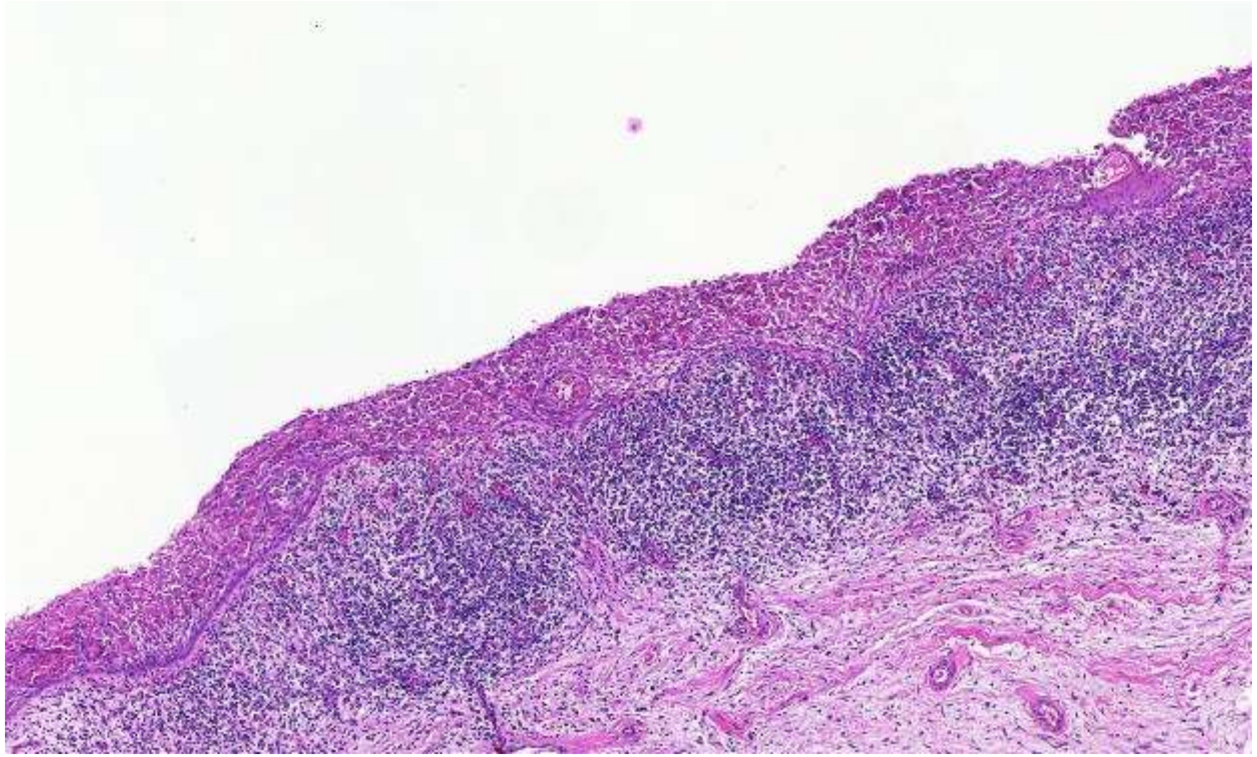

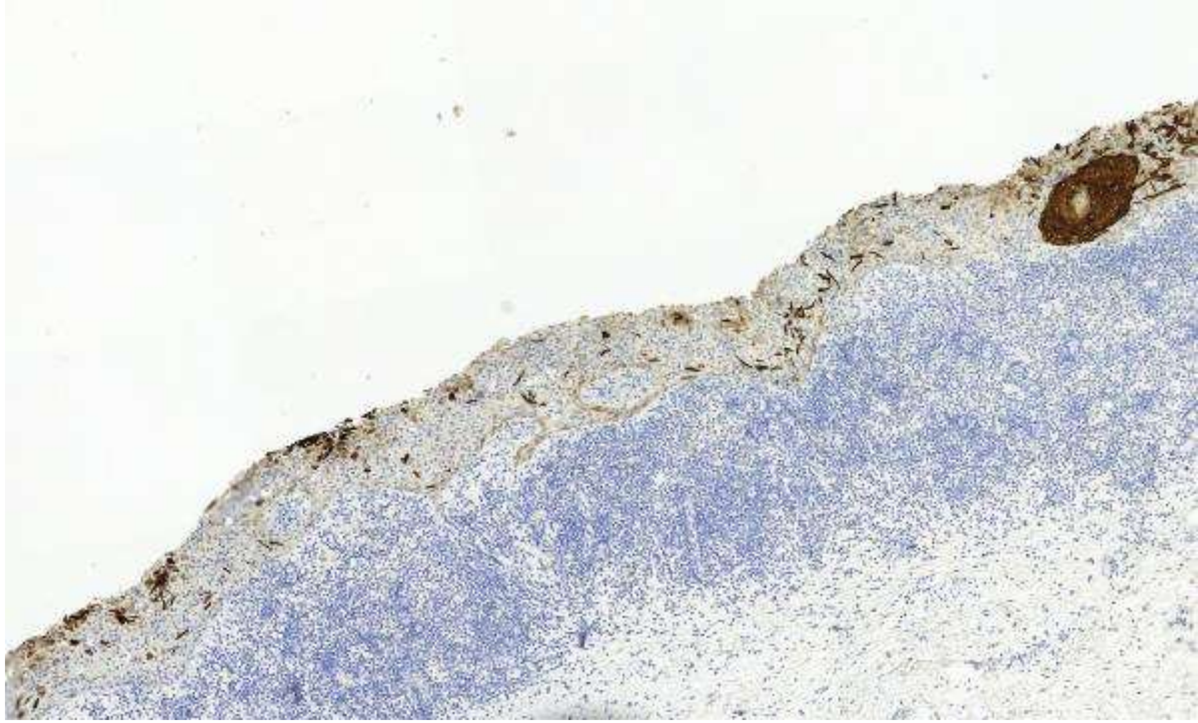

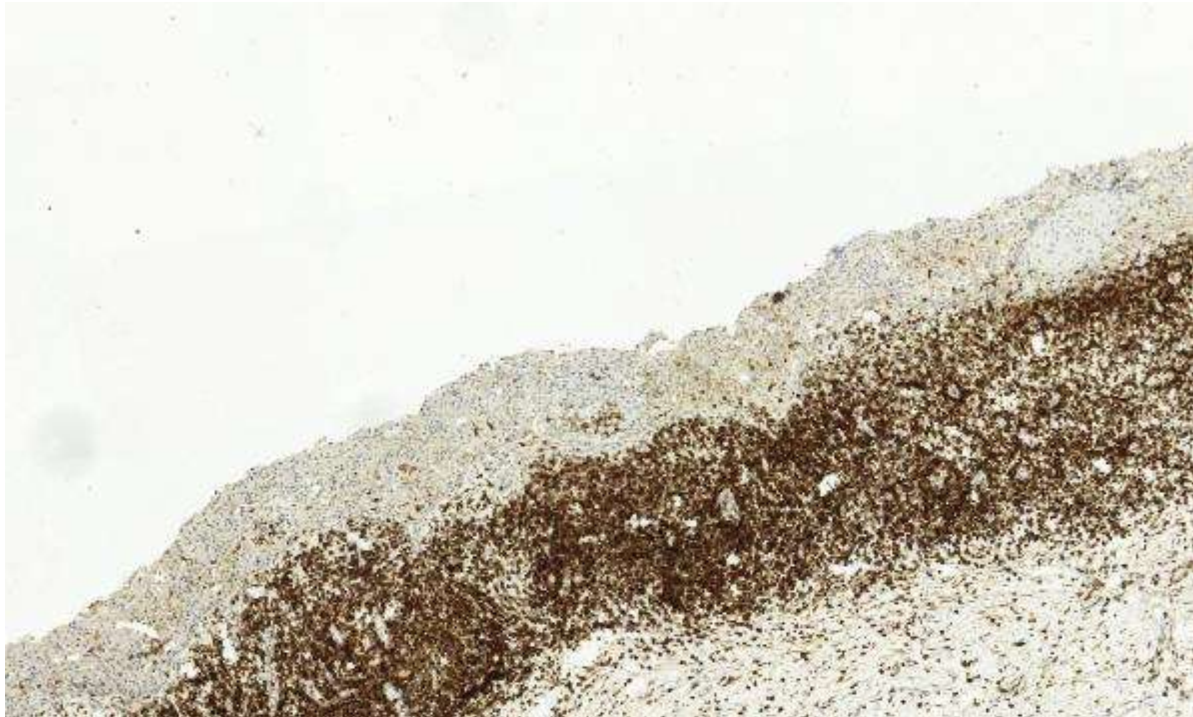

S13 – bcl2 negativity at the site of inflammation associated epithelial change (x20)

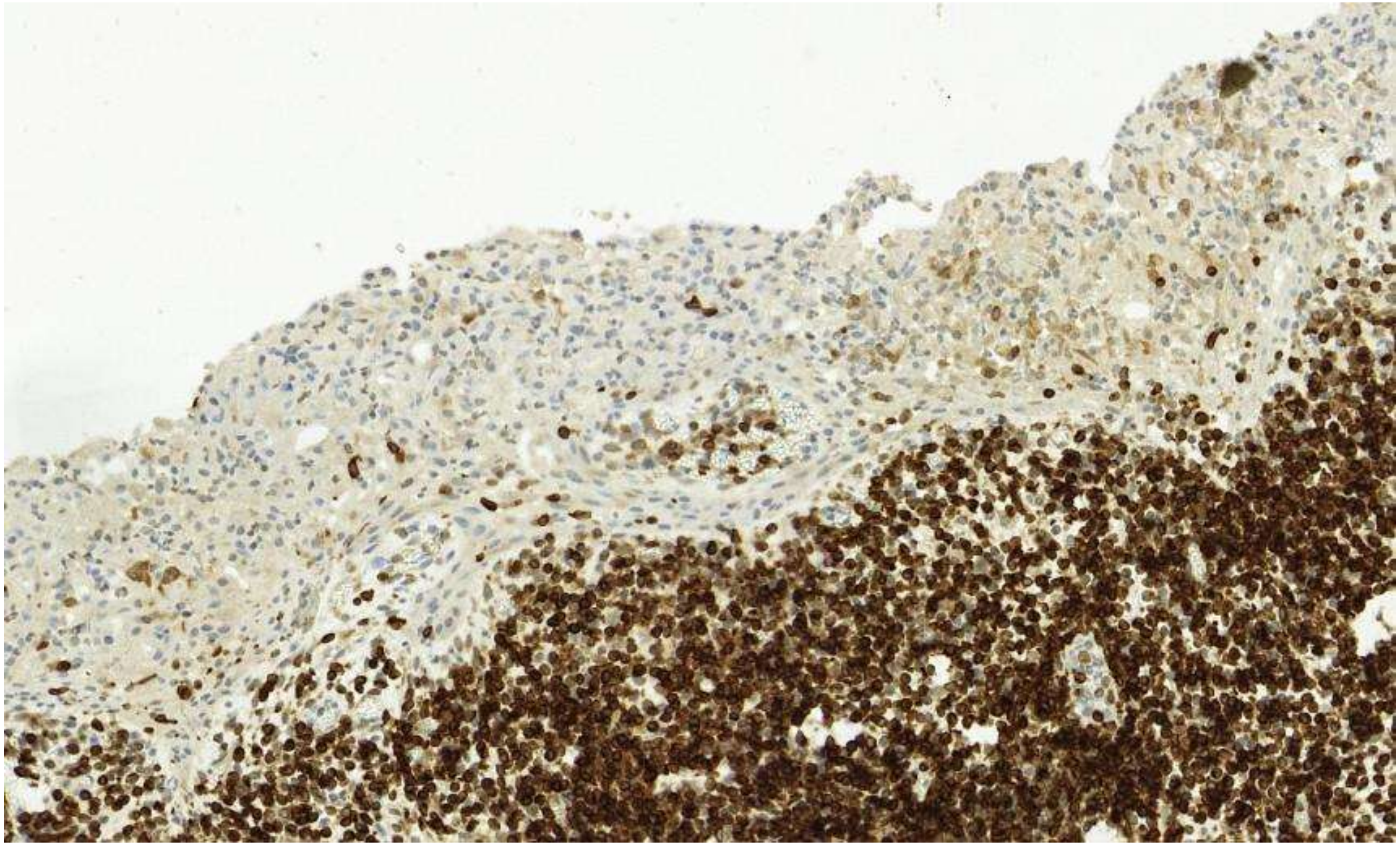

Case S13 – Conclusion

- H&E: thin parakeratotic epithelium with wavy surface and basal palisading, even epithelial stromal interface; focal inflammation associated thicker epithelium without parakeratosis, basal palisading or wavy surface, irregular stromal epithelial interface
- Inflammation absent, focally present (altered epithelium)
- Bcl2: basal positivity; focally negative at site of inflammation and altered epithelium
- CK17: dominantly transepithelial; focally weak to ignorable spinous and/or basal staining at site of inflammation and altered epithelium
- Histopathological diagnosis: OKC
- Combined clinical and pathological opinion: OKC (associated with the Gorlin-Goltz syndrome)

S14 – HE, CK17, Bcl2 (x20)

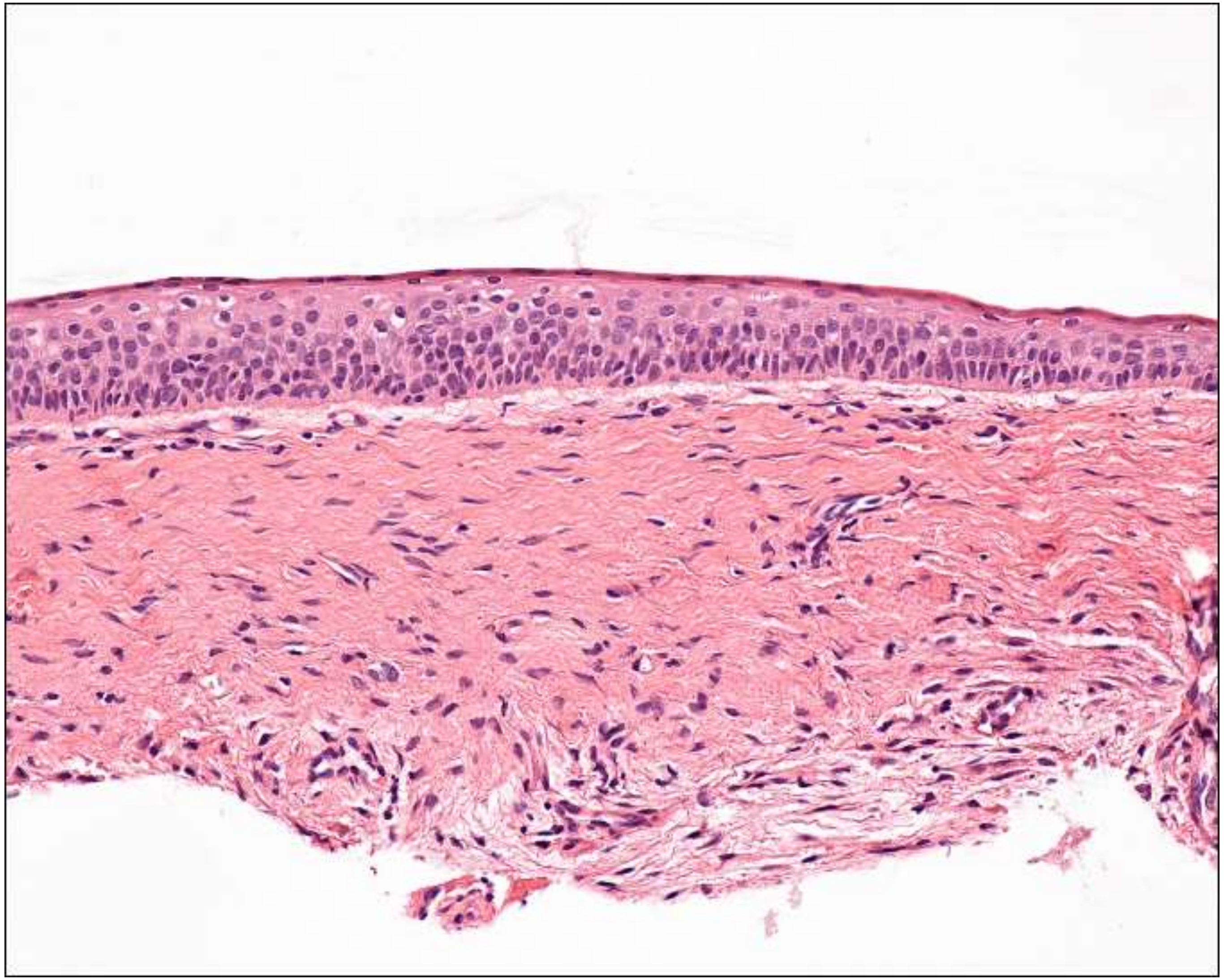

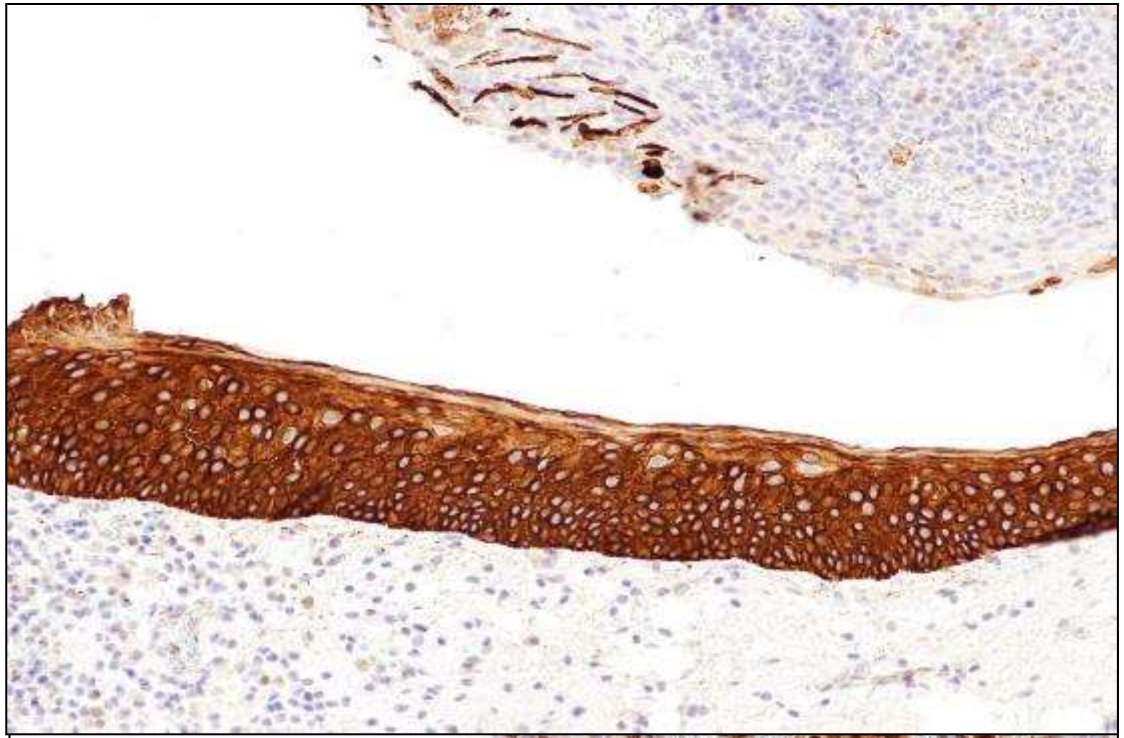

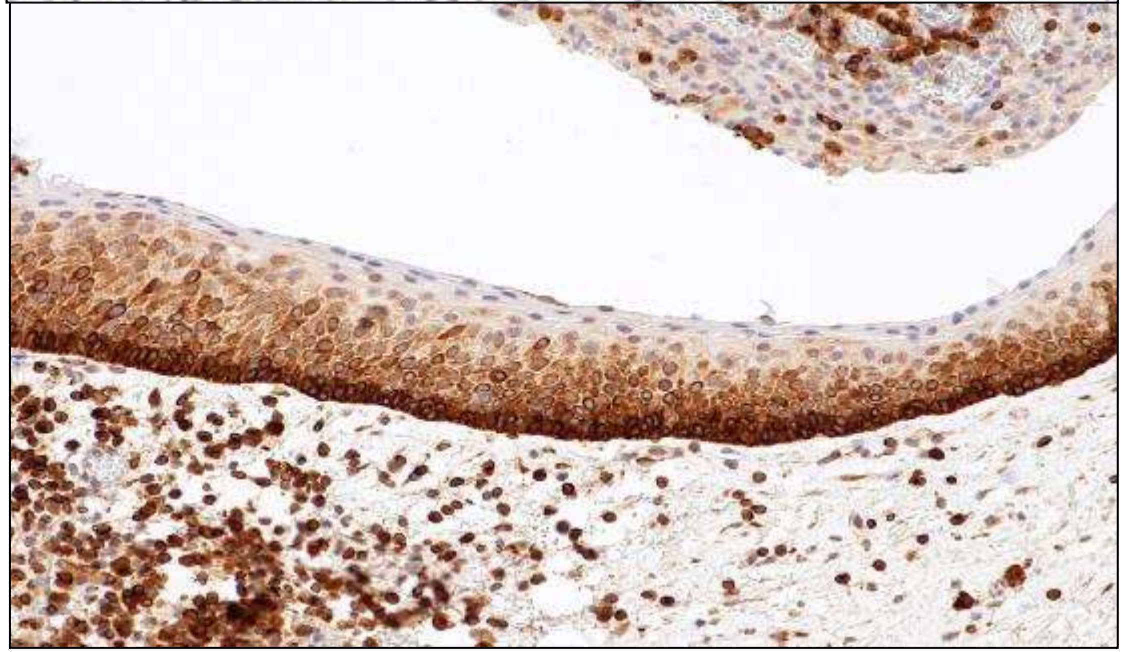

S14 – HE, CK17, Bcl2 (x20)

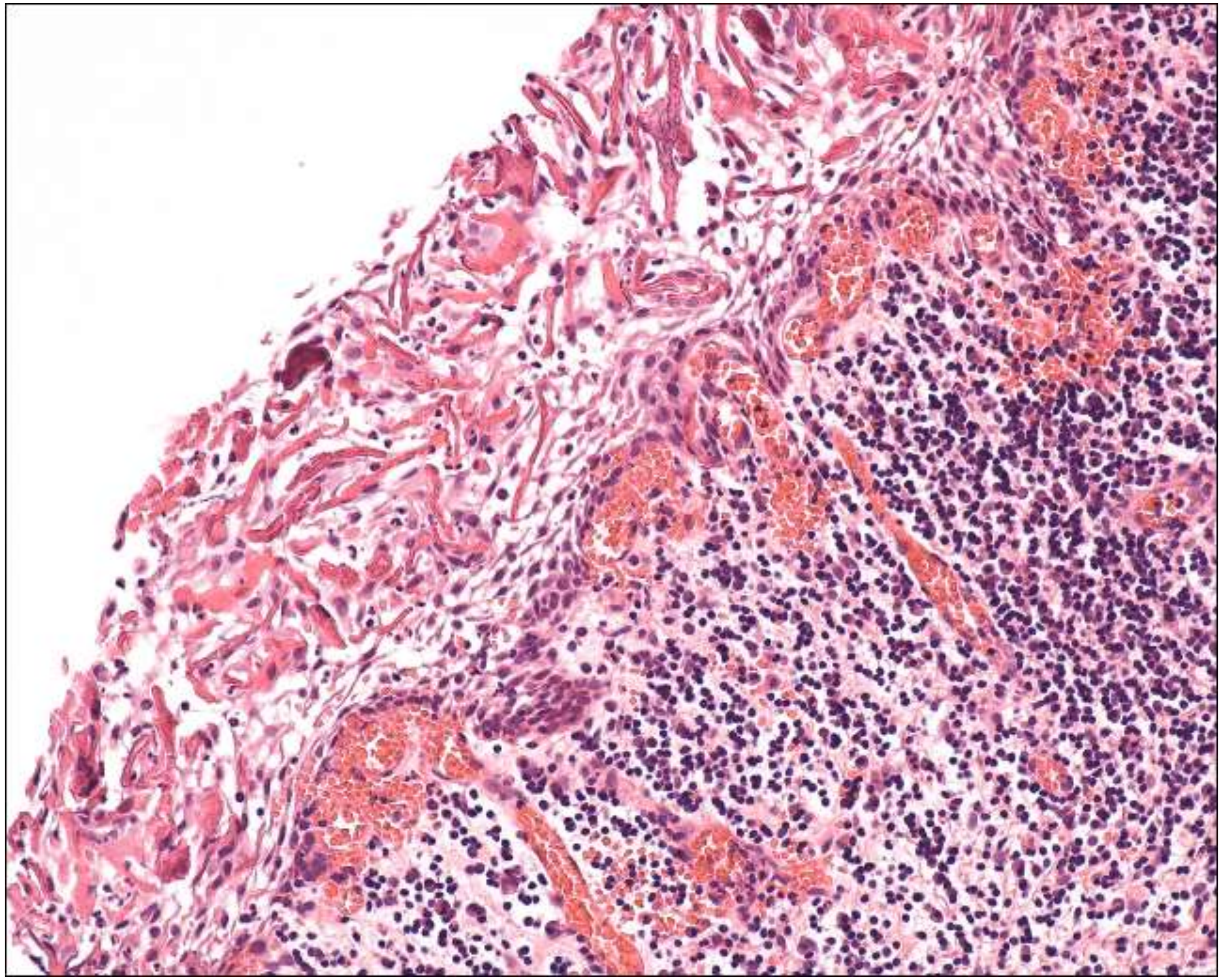

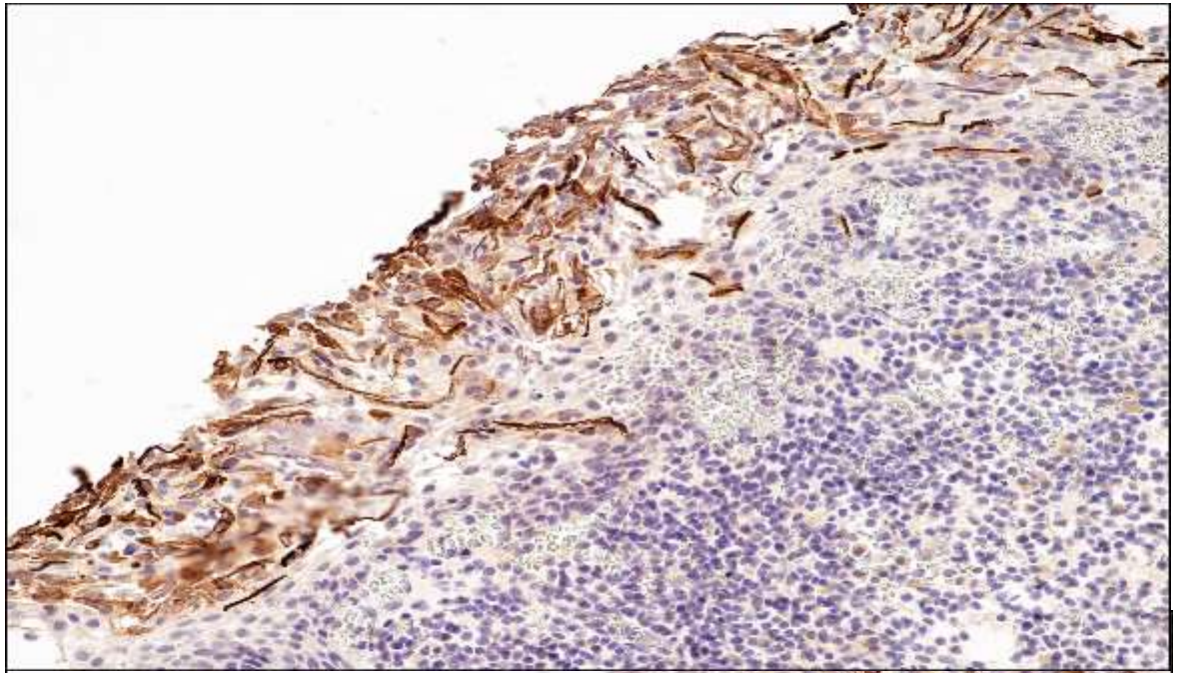

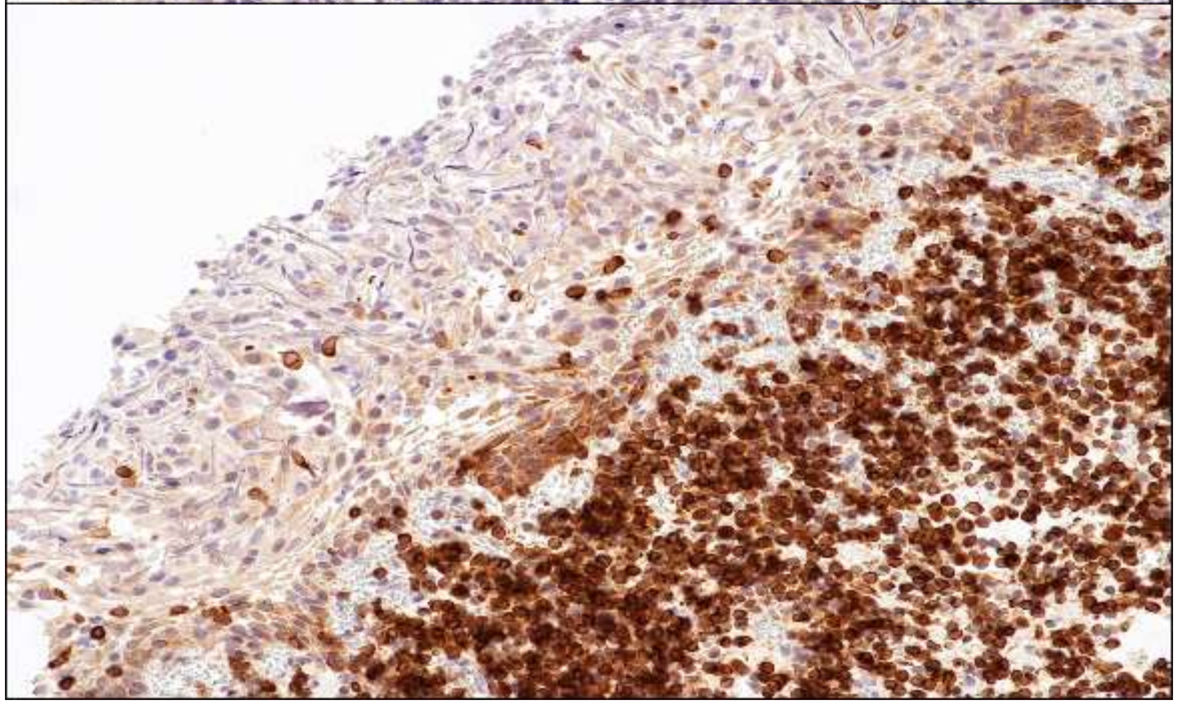

Case S14 – Conclusion

- H&E: thin parakeratotic epithelium with wavy surface and basal palisading, even epithelial stromal interface
- Focal chronic inflammation
- Bcl2: basal positivity, focally negative / weak spinous to moderate basal at the site of inflammation and altered epithelium
- CK17: dominantly transepithelial; focal weak spinous staining and positivity of squames at the site of inflammation and altered epithelium
- Histopathological diagnosis: OKC
- Combined clinical and pathological opinion: OKC (associated with the Gorlin-Goltz syndrome)

Case S15-16

- 39-year-old female
- History: She was operated on with right mandible and bilateral maxillary OKCs in 2016. Genetic testing for Gorlin-Goltz syndrome is in progress.
- Dental hygiene/status:
- CBCT: radiolucent areas from tooth 21 to 23 and distal to tooth 17.
- Operation: removal of the cysts, root resection of teeth 17, 21, 22, 23
- Clinical diagnosis: keratocyst

S15 – OPT

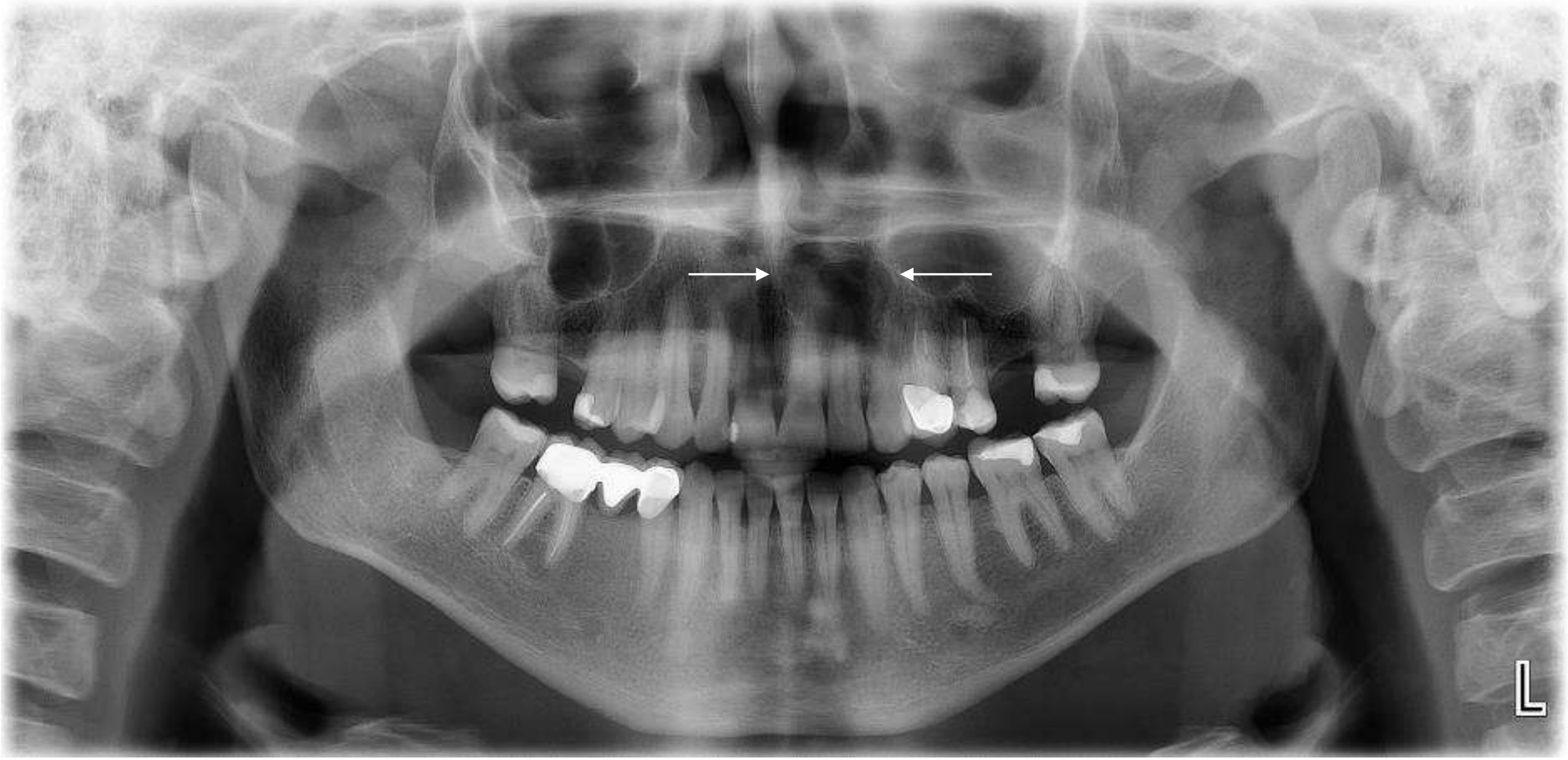

S15 – HE, CK17, bcl2 (x0.5)

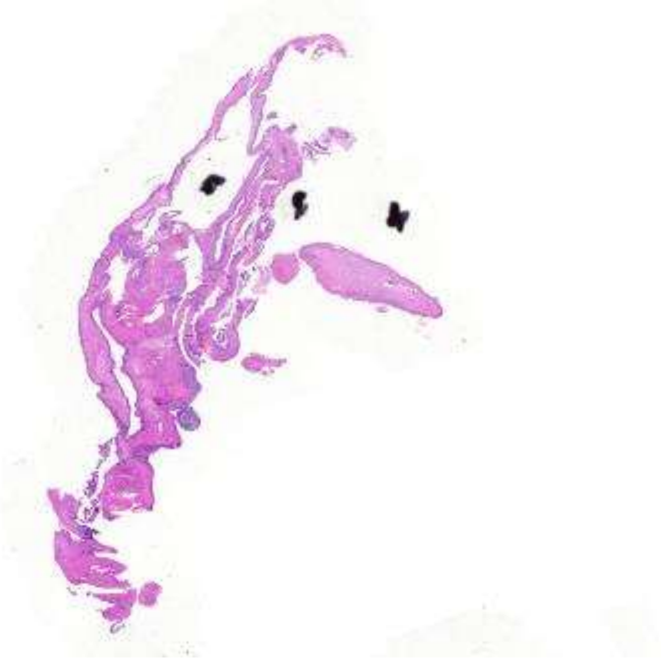

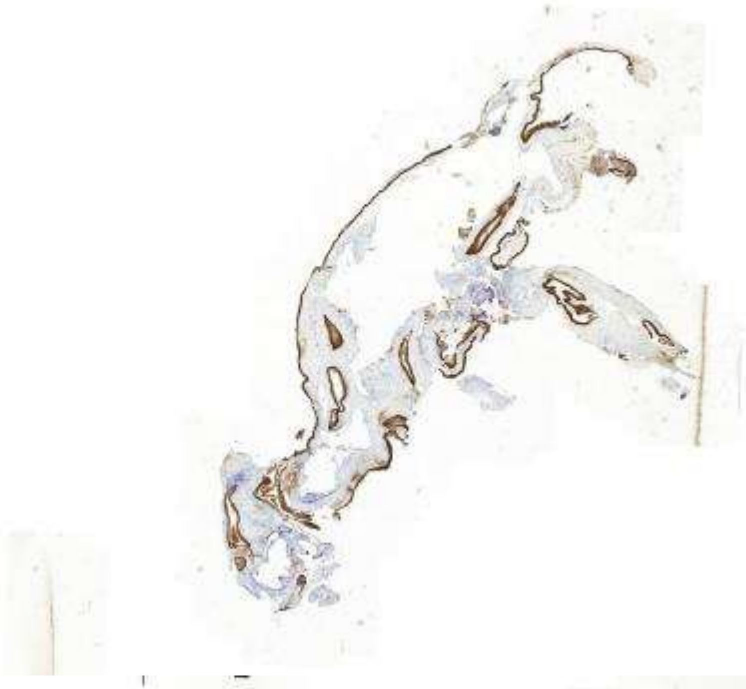

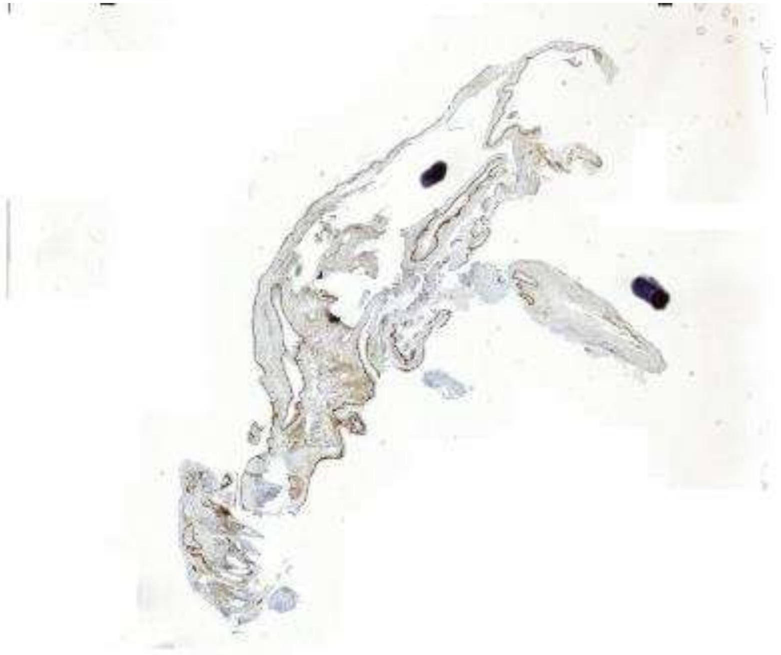

S15 – HE, CK17, bcl2 (x6)

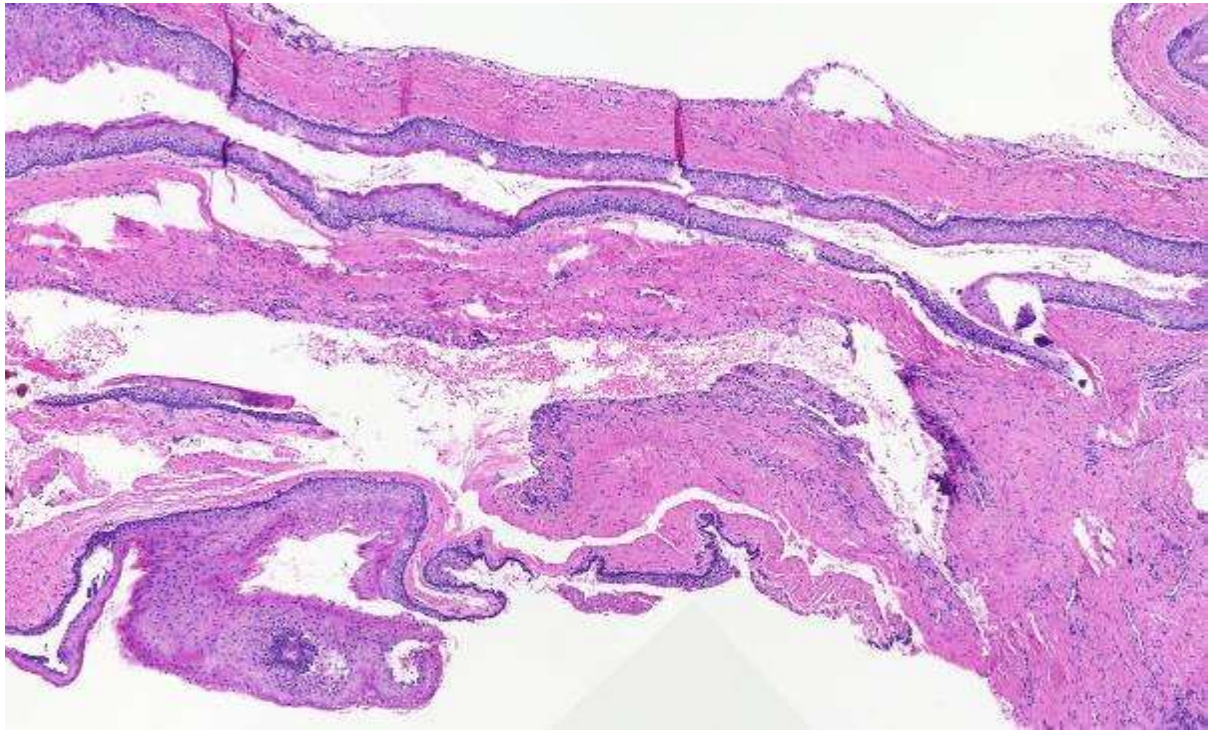

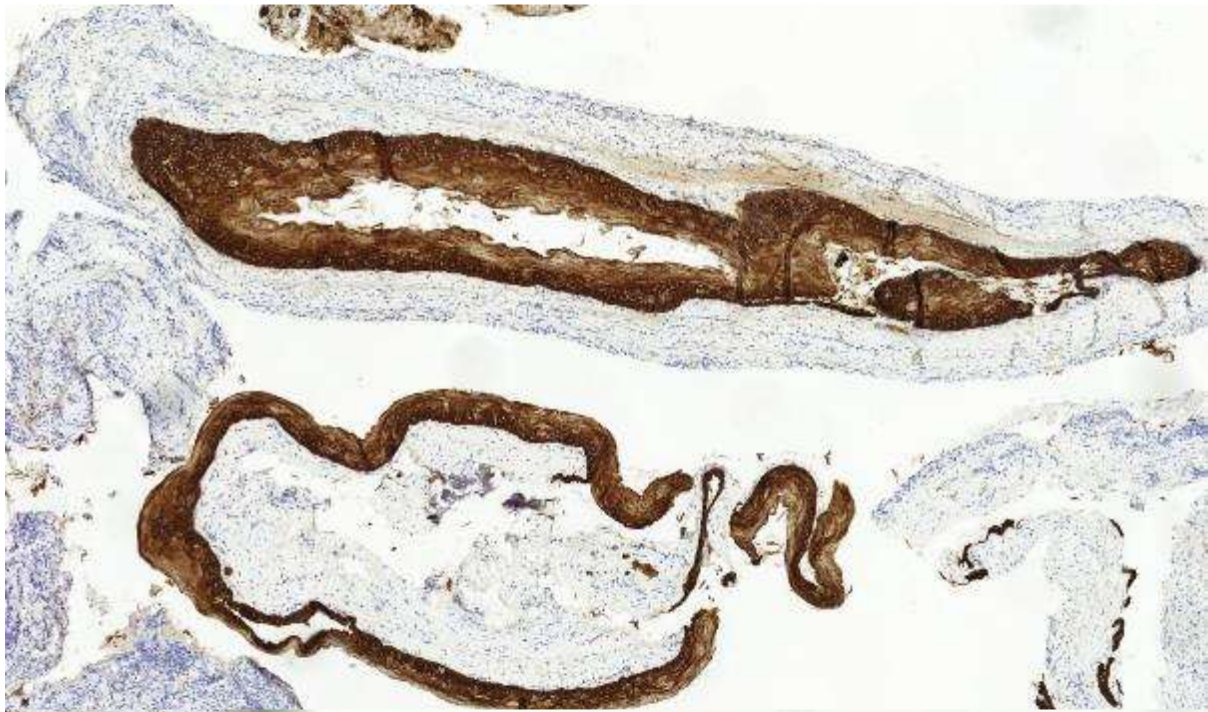

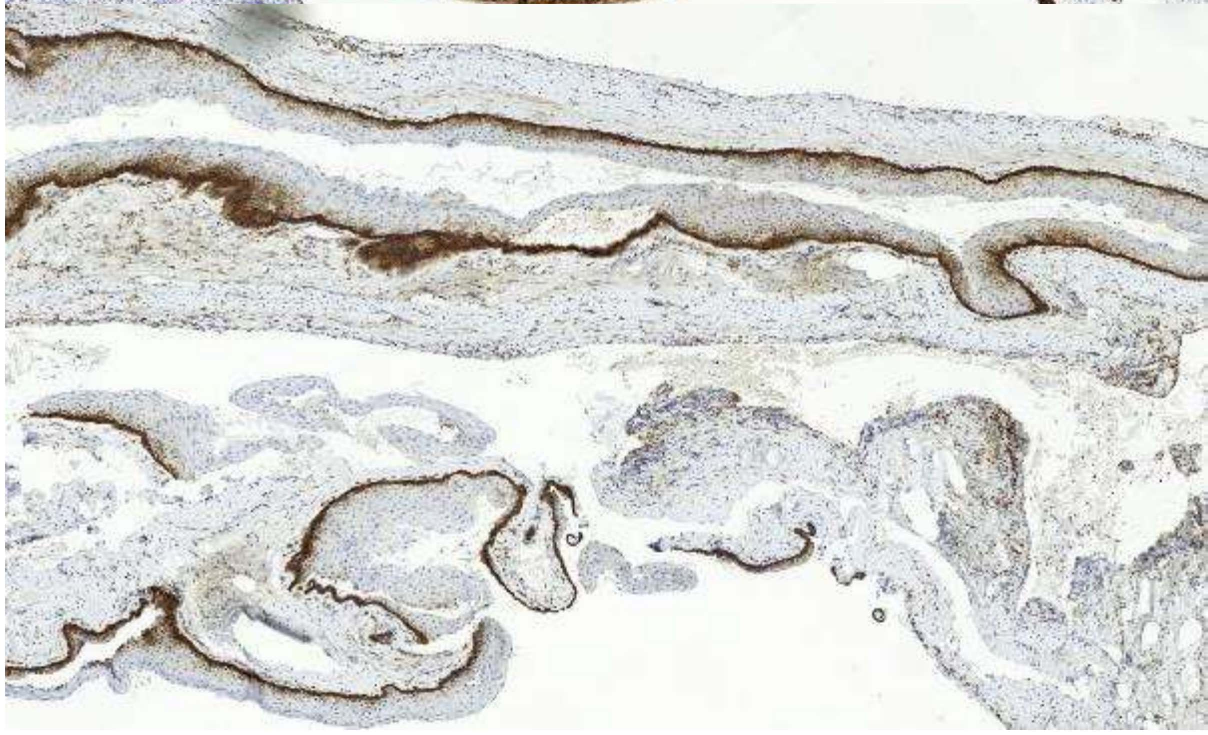

Case S15 – Conclusion

- H&E: thin parakeratotic epithelium with wavy surface and basal palisading, even epithelial stromal interface
- No inflammation present
- Bcl2: diffuse basal, suprabasal positivity
- CK17: diffuse transepithelial
- Histopathological diagnosis: OKC
- Combined clinical and pathological opinion: OKC (likely to be associated with the Gorlin-Goltz syndrome)

S16 – OPT

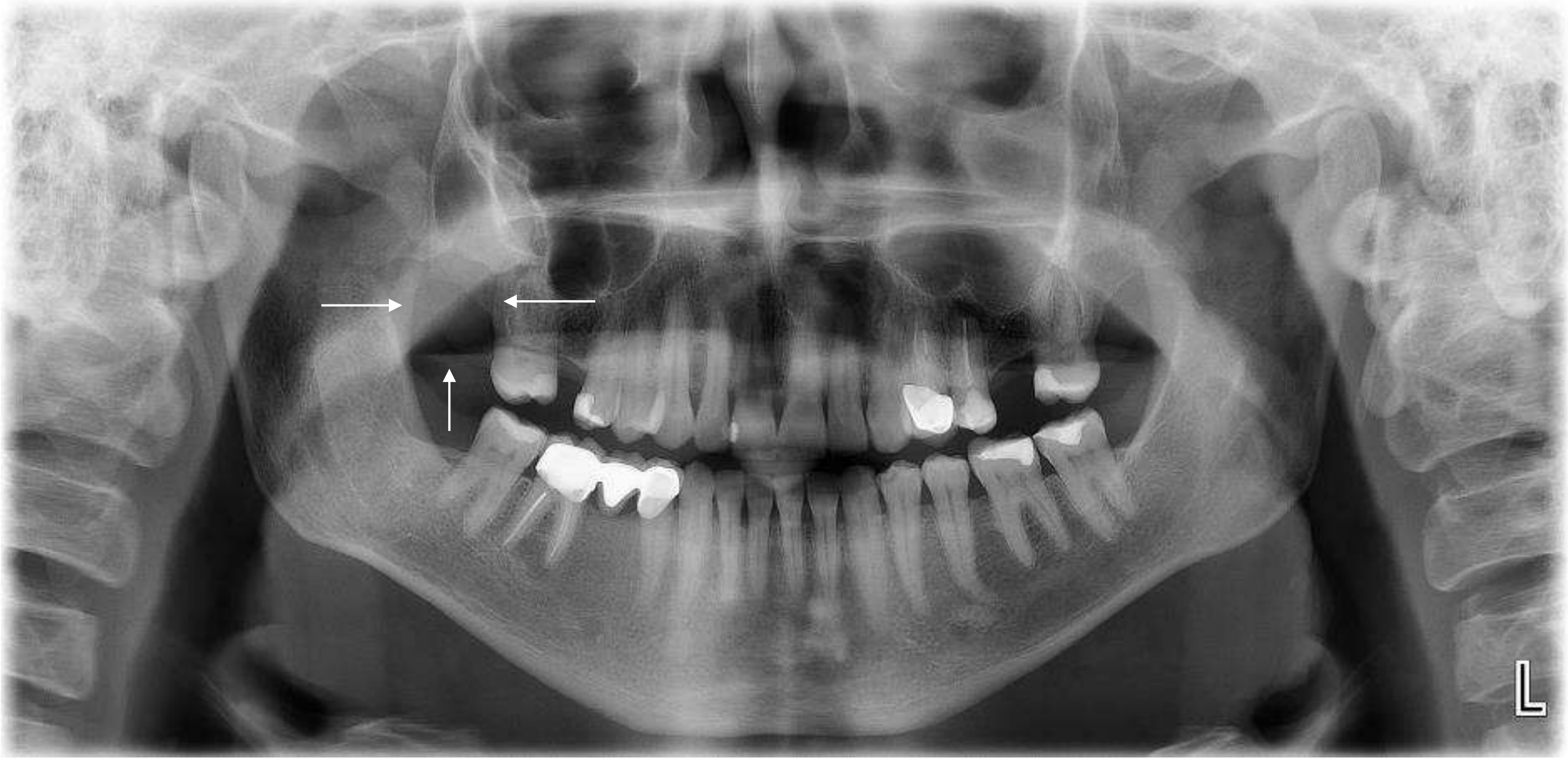

S16 – HE, CK17, bcl2 (x14)

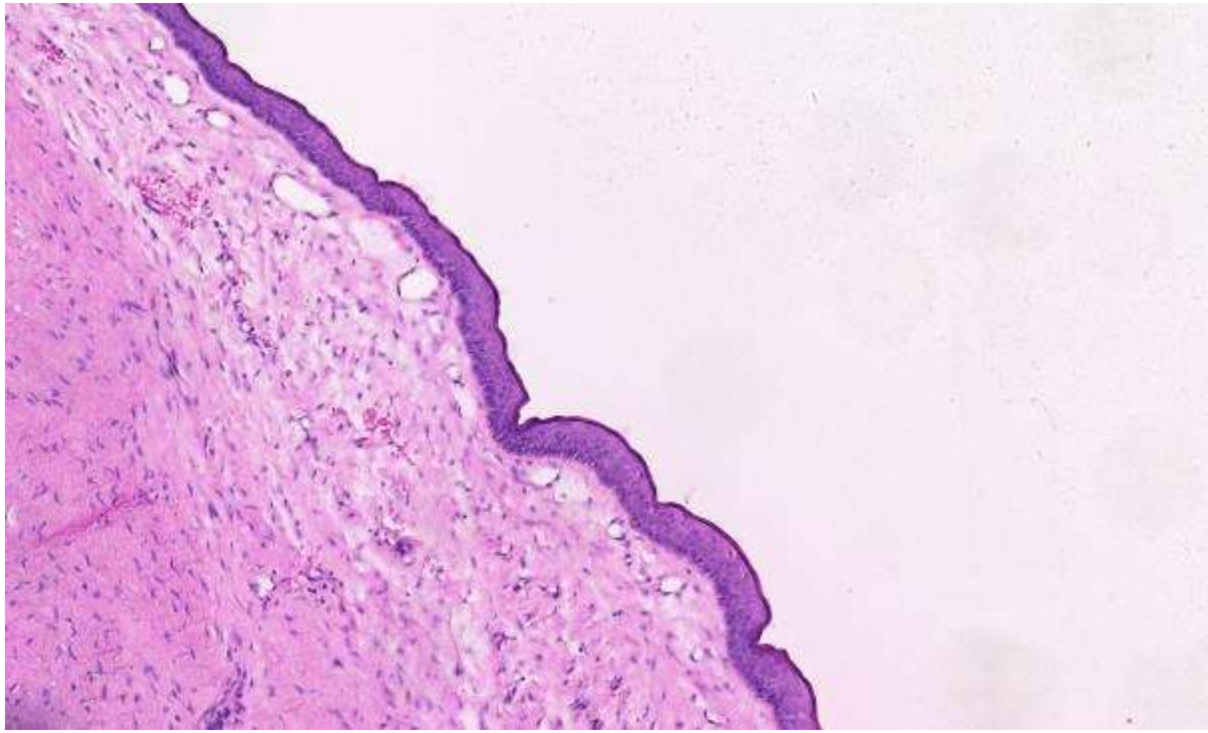

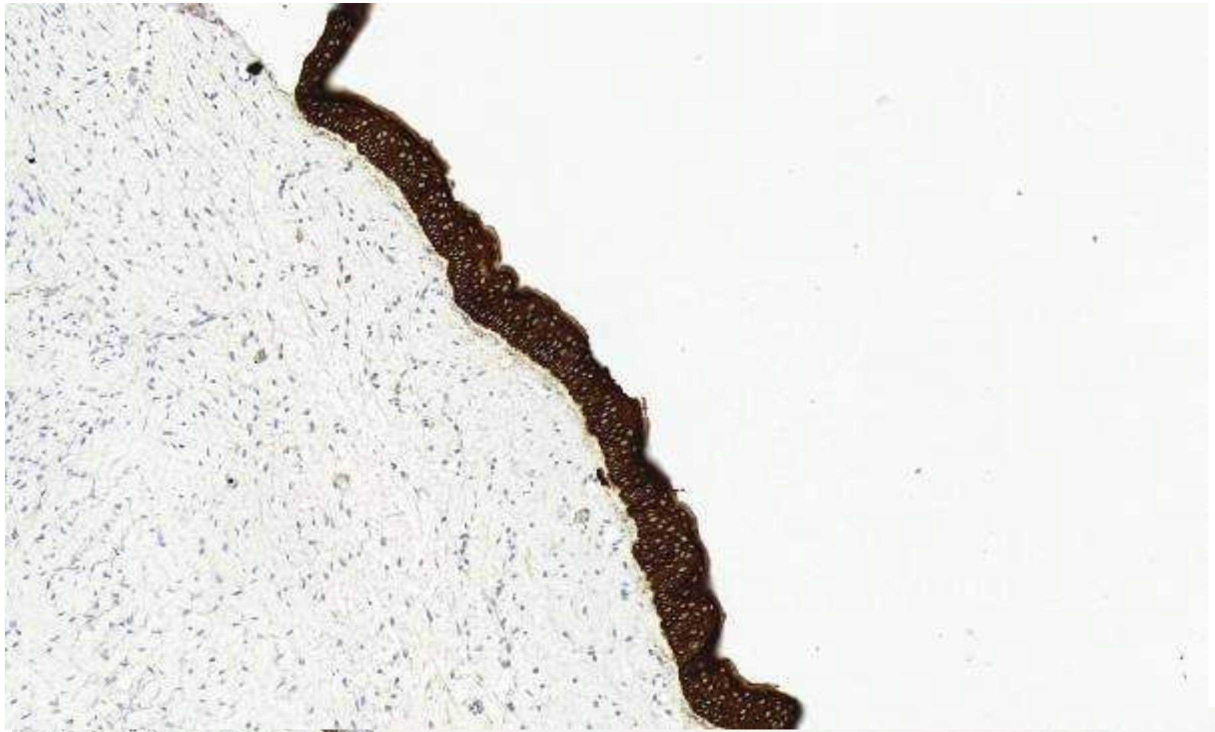

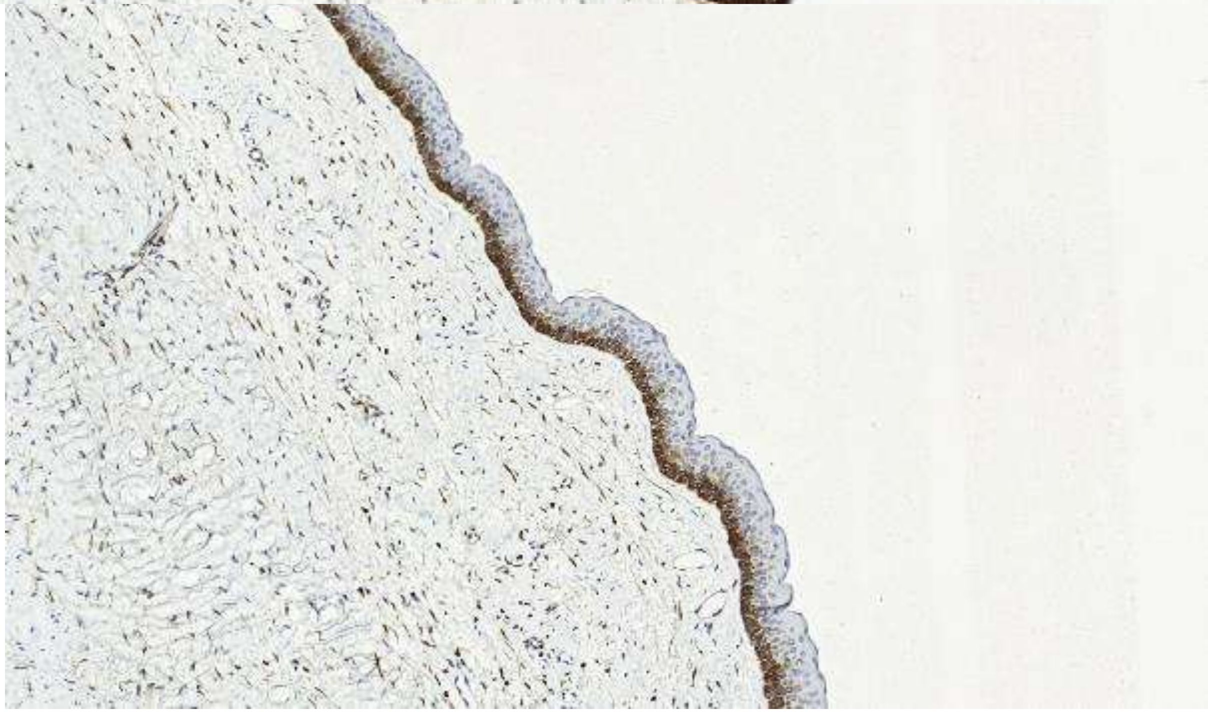

S16 – CK17, bcl2 (x5) Epithelium without basal palisading: bcl2 basal staining lost

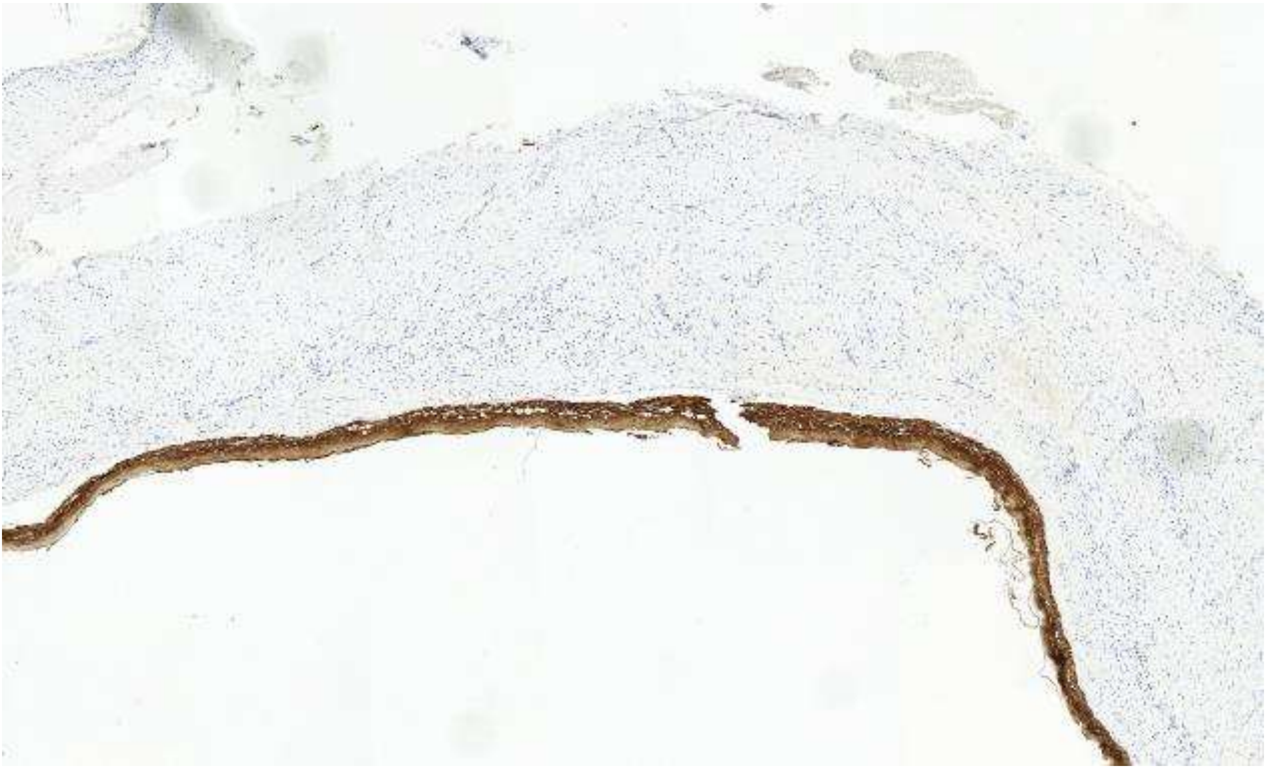

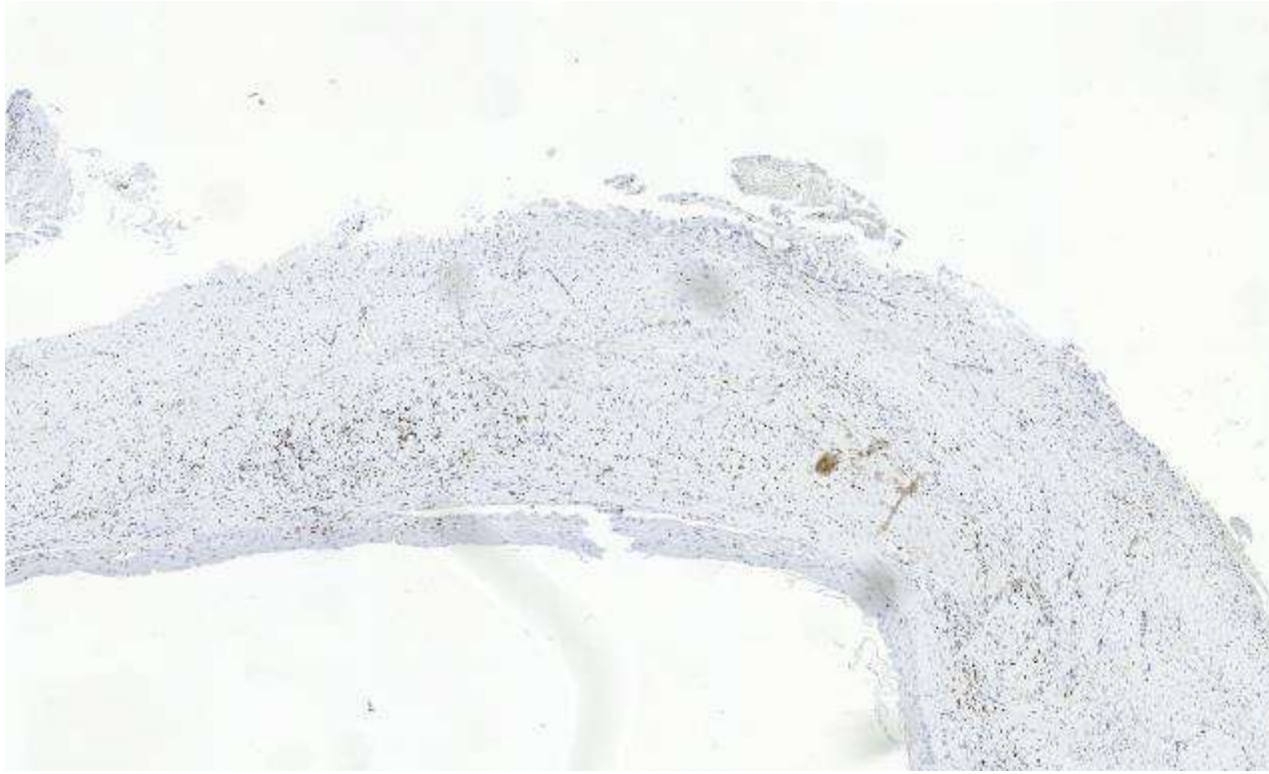

Case 16 – Conclusion

- H&E: thin parakeratotic epithelium with wavy surface and basal palisading, even epithelial stromal interface
- Inflammation absent
- Bcl2: basal, suprabasal positivity; small area without basal palisading and lost basal staining
- CK17: transepithelial positivity
- Histopathological diagnosis: OKC
- Combined clinical and pathological opinion: OKC (likely to be associated with the Gorlin-Goltz syndrome)

Case S17

- 30-year-old male
- Dental hygiene/status: maintained, treated teeth. Impaction of teeth 18, 28, 38, 48.
- CBCT: cystic radiolucent area around tooth 48
- Operation: removal of the cyst and teeth 28, 48
- Clinical diagnosis: odontogenic cyst, keratocyst?

S17 – HE, CK17, bcl2 (x20)

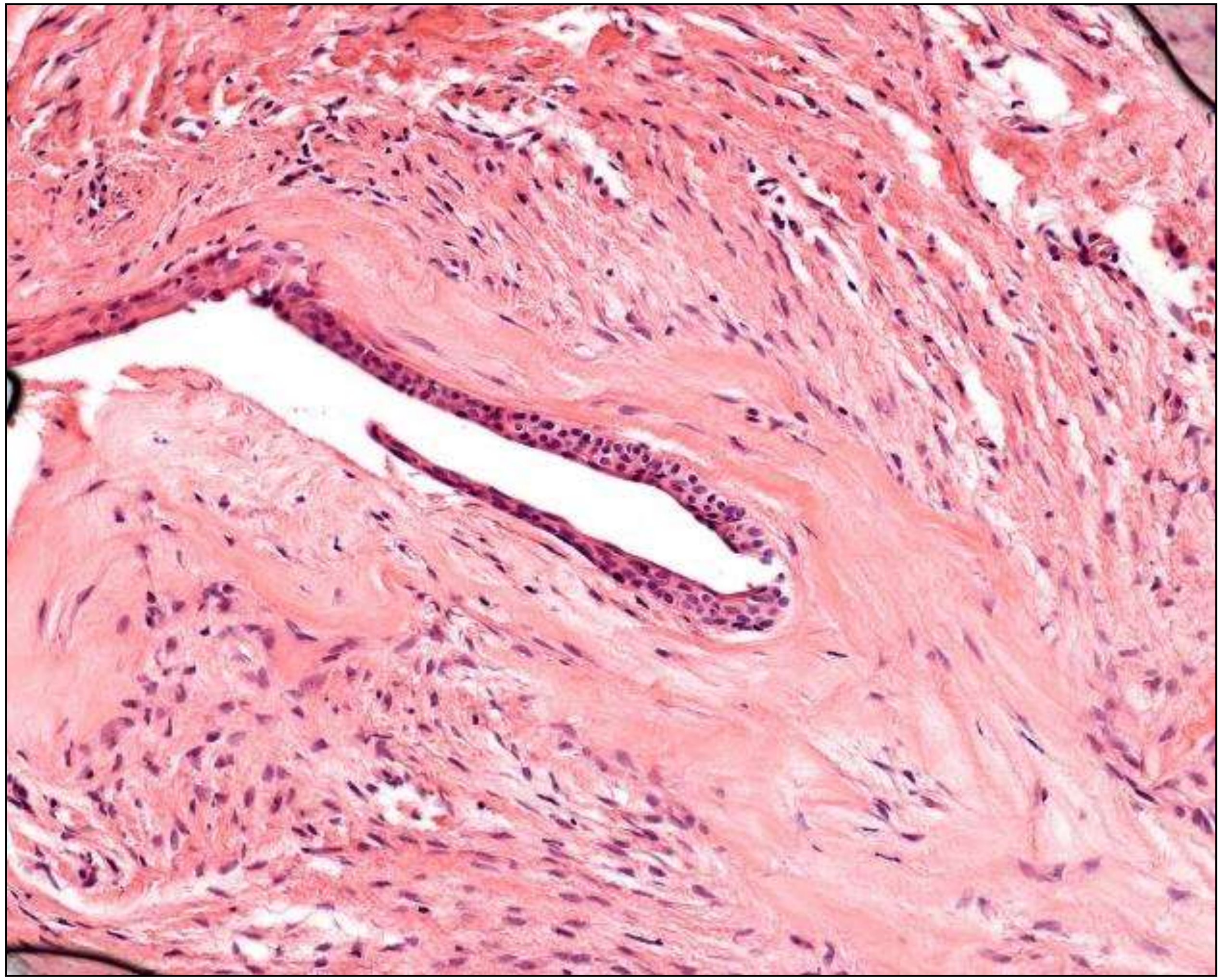

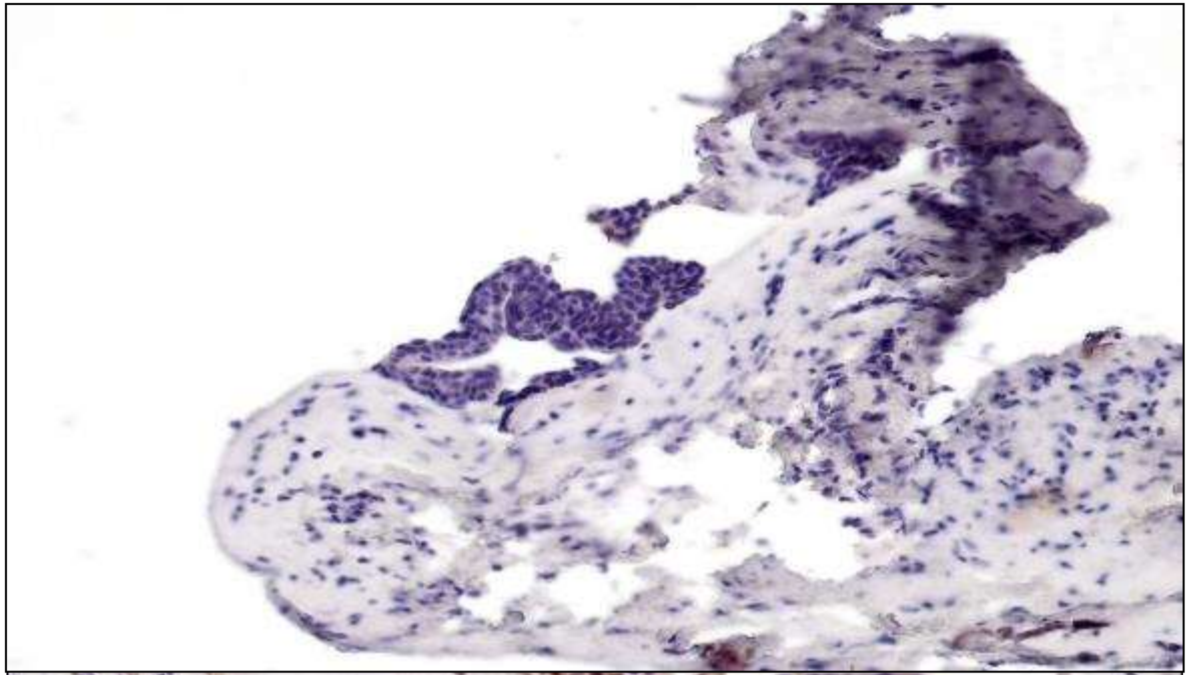

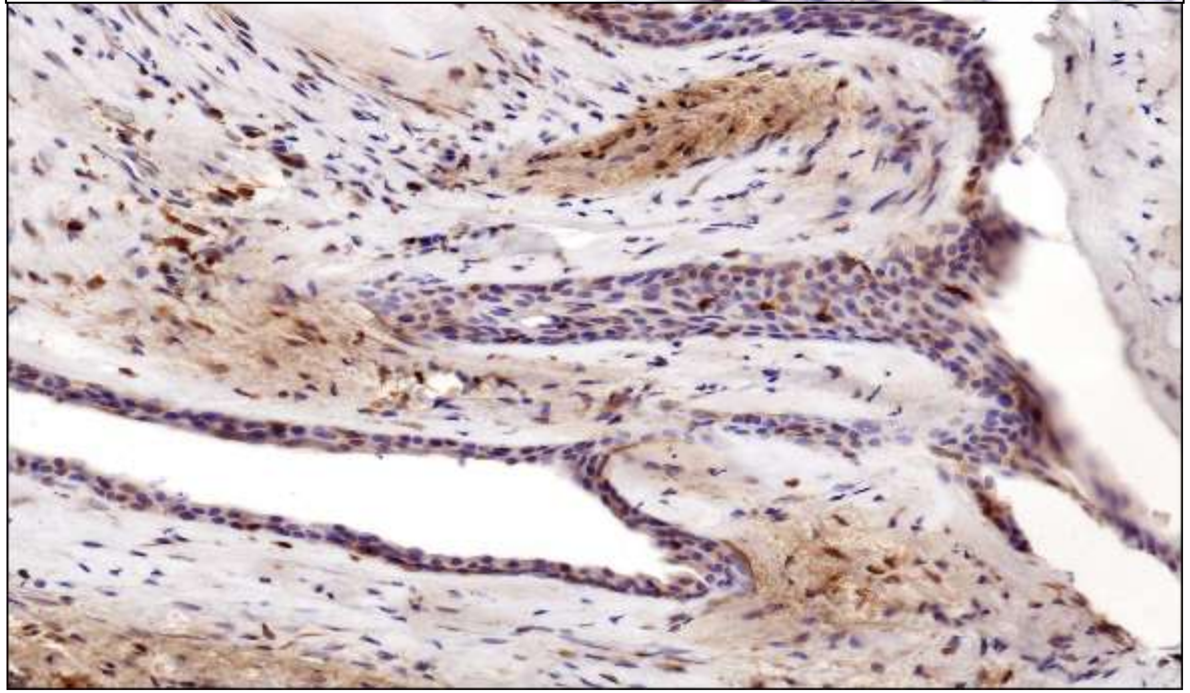

S17 – CK17, CK17, bcl2 (x20)

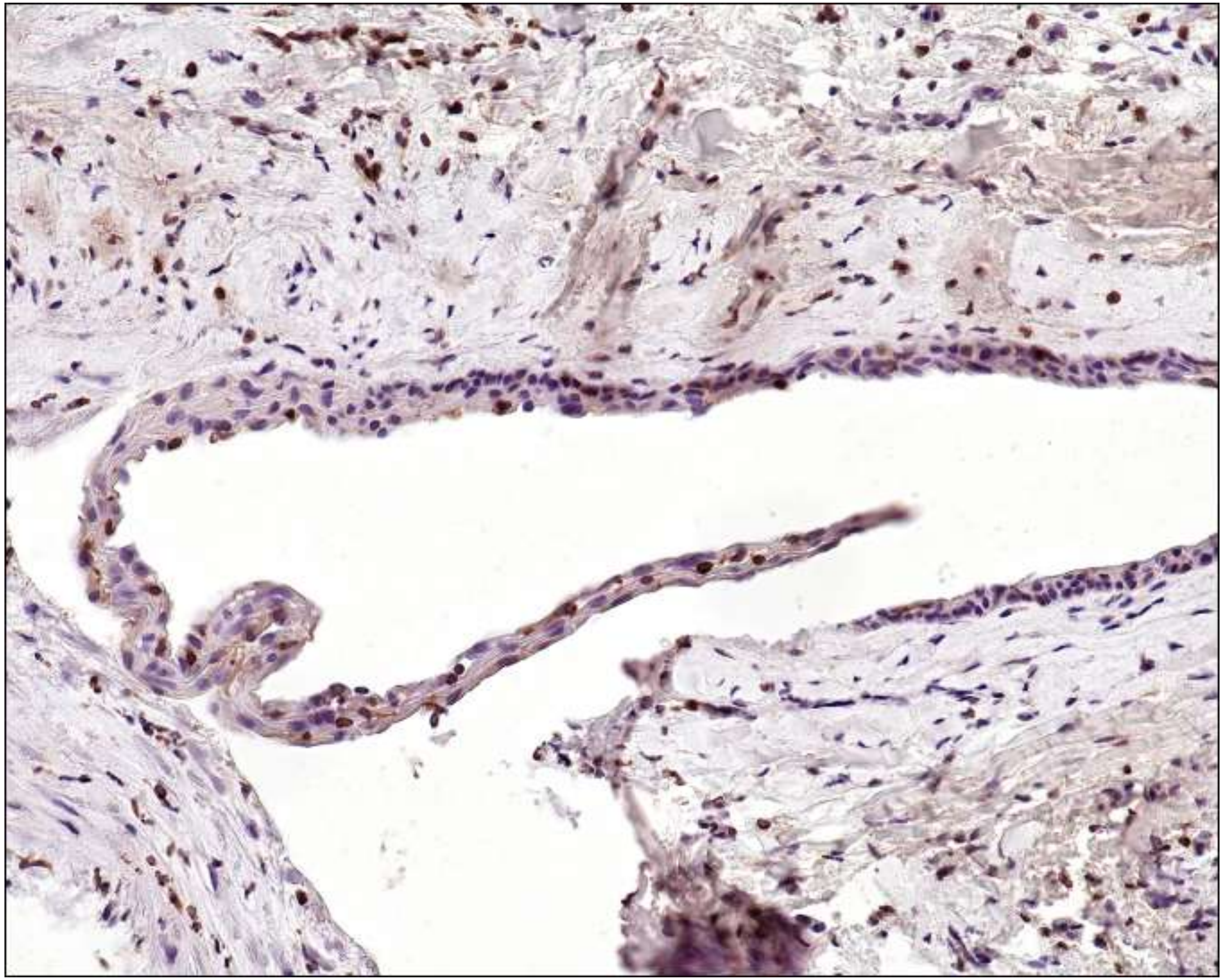

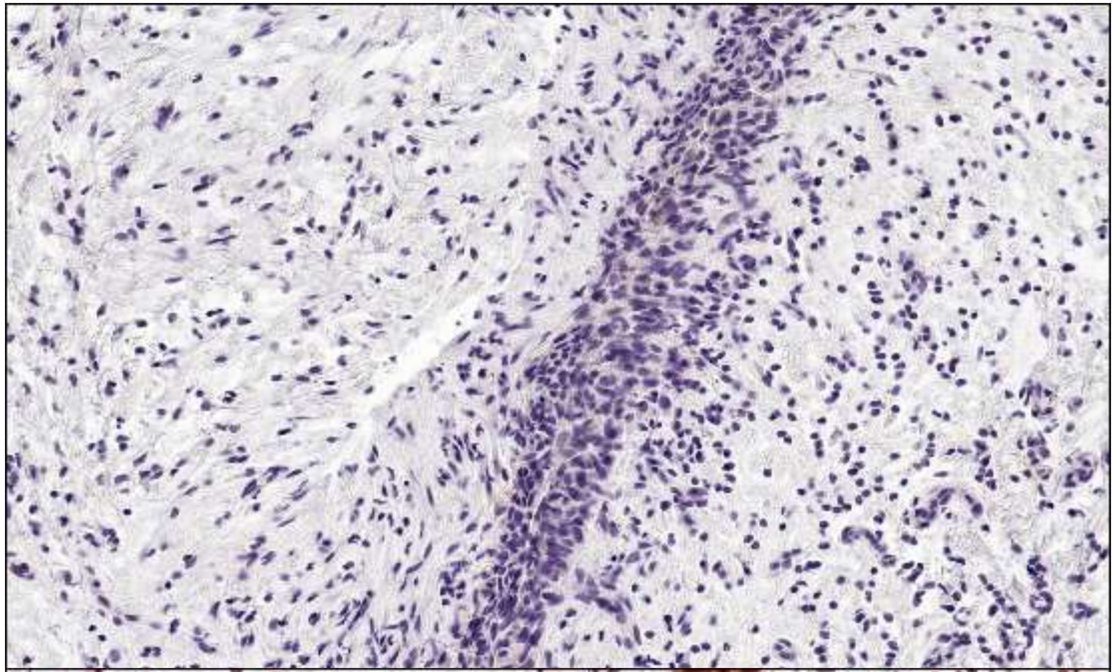

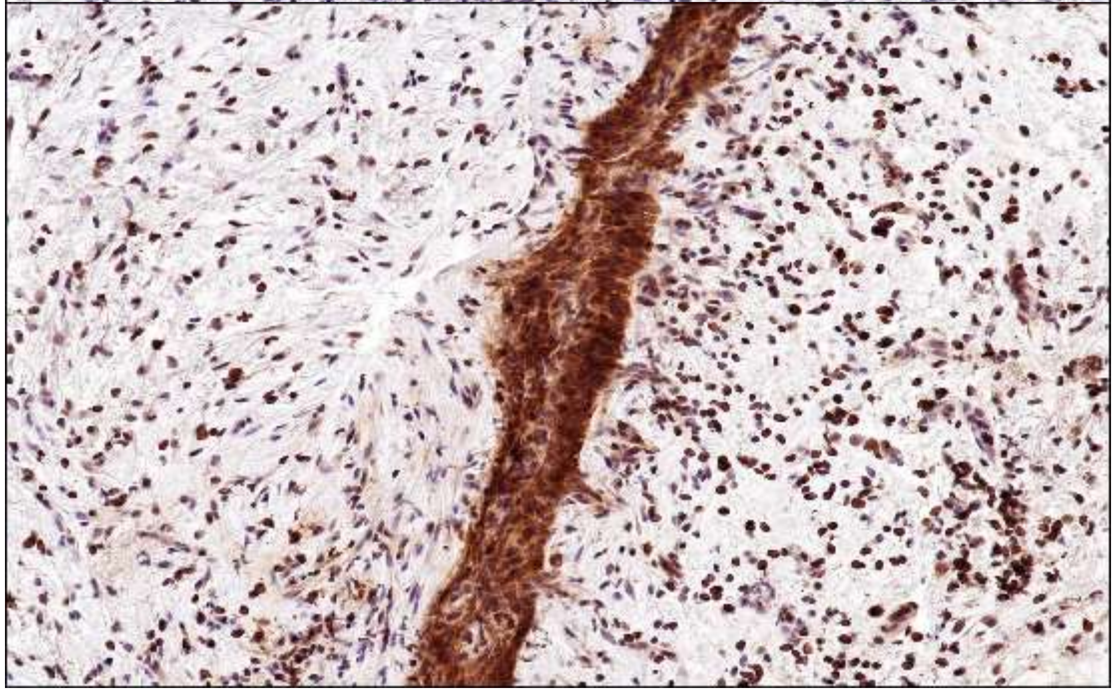

Case S17 Conclusion

- H&E: damaged and fragmented cyst lined by thin squamous epithelium without keratinization, undulant surface and basal palisading
- Inflammation is scant
- Bcl2: dominantly negative, focally transepithelial positivity
- CK17: dominantly negative, focal superficial
- Histopathological diagnosis: may be consistent with follicular cyst
- Combined clinical and pathological opinion: cannot be specified; developmental or mature inflammatory cyst N.O.S. (could be a dentigerous cyst)

Case S18

- 63-year-old male
- History: Left upper tooth 25 and right upper tooth 12 were removed with cysts in 2019.
- Dental hygiene/status: maintained, treated teeth. Teeth 15, 14, 11, 21, 22 are not vital. Maxillary bilateral radiolucent cysts around teeth 12, 11, 21, 22.
- Operation: Caldwell-Luc surgery - cystectomy, resection of root of teeth 11, 21, 22
- Clinical diagnosis: keratocyst?

S18 - OPT

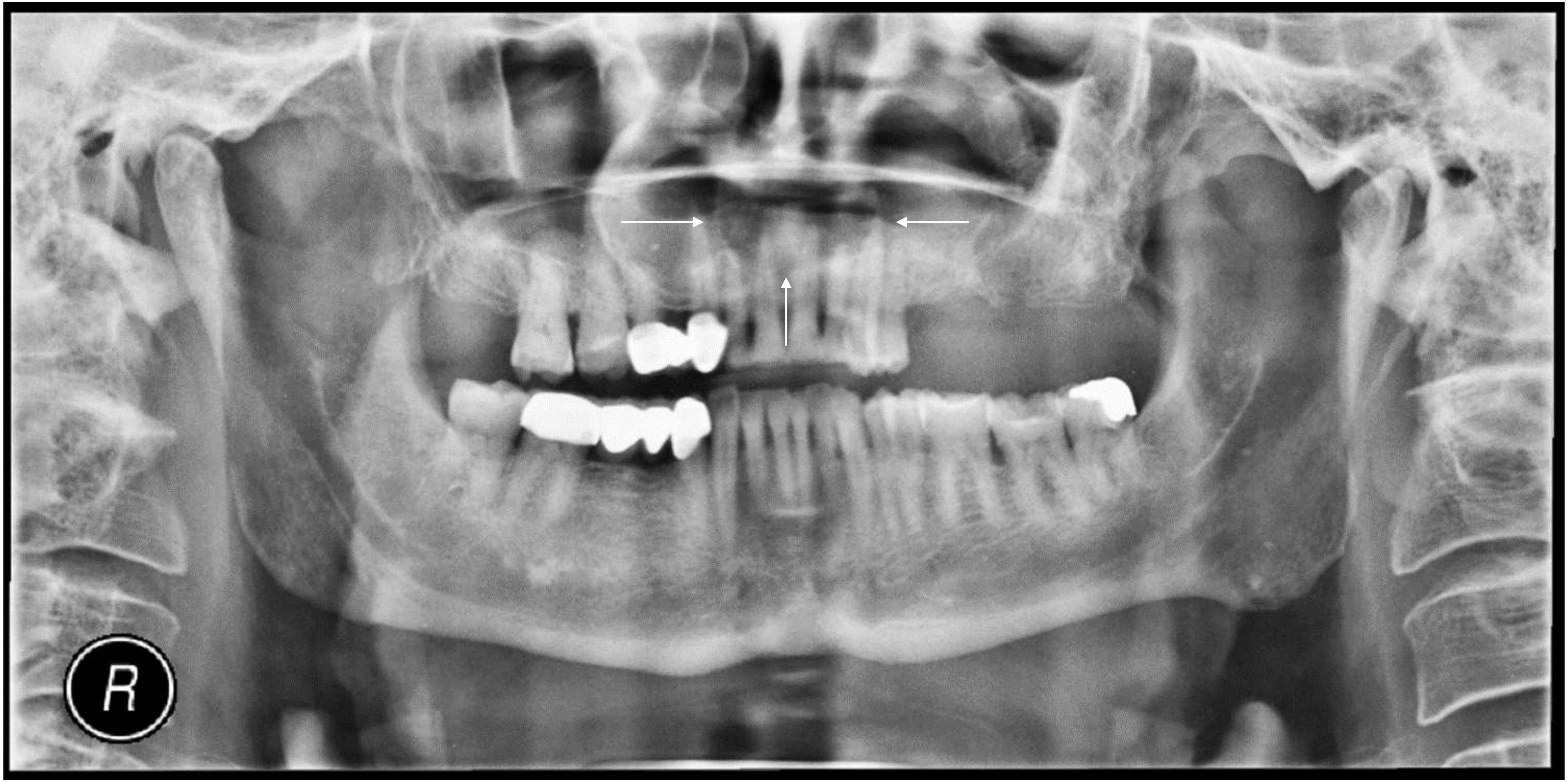

S18 – HE, CK17, bcl2 (x20)

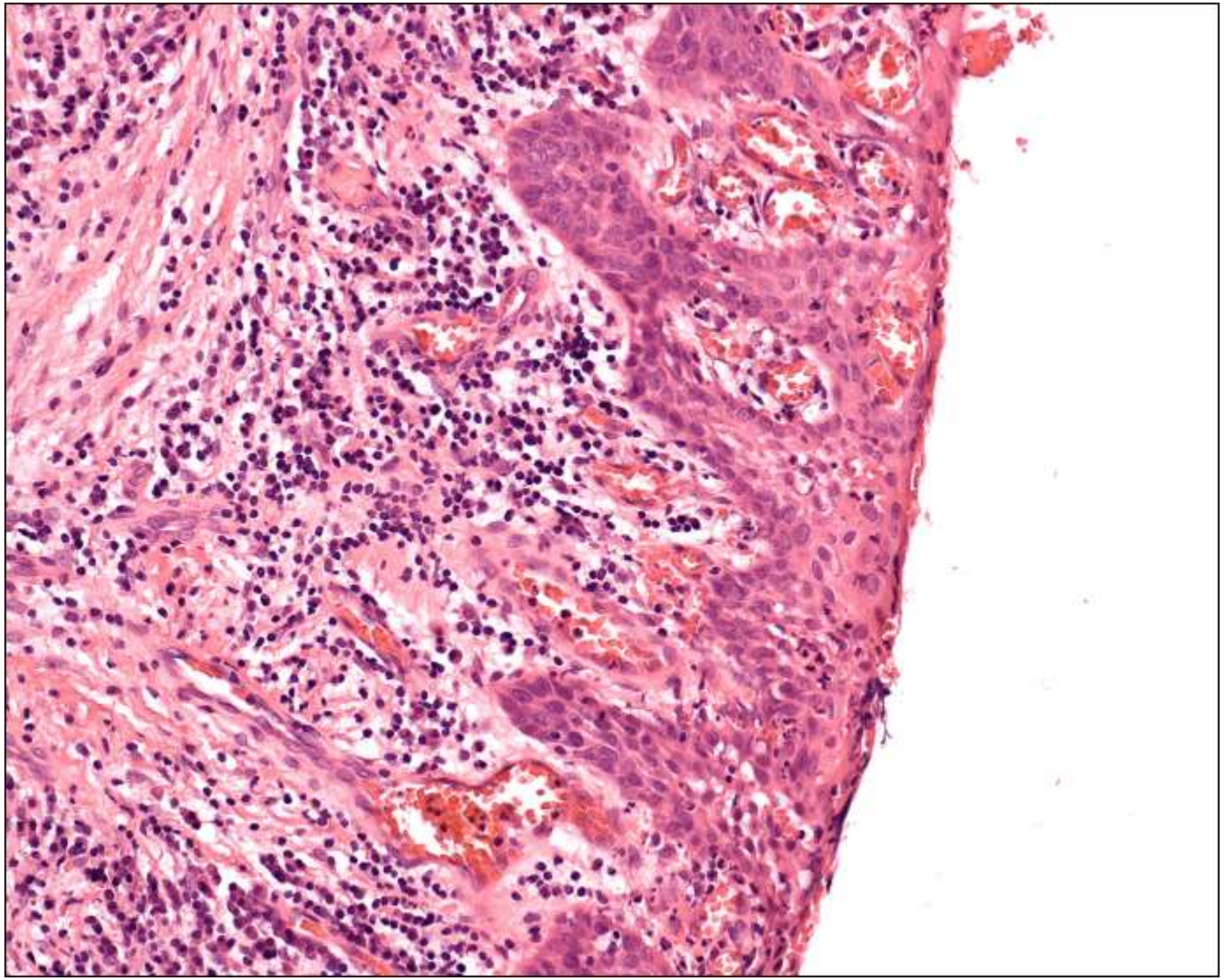

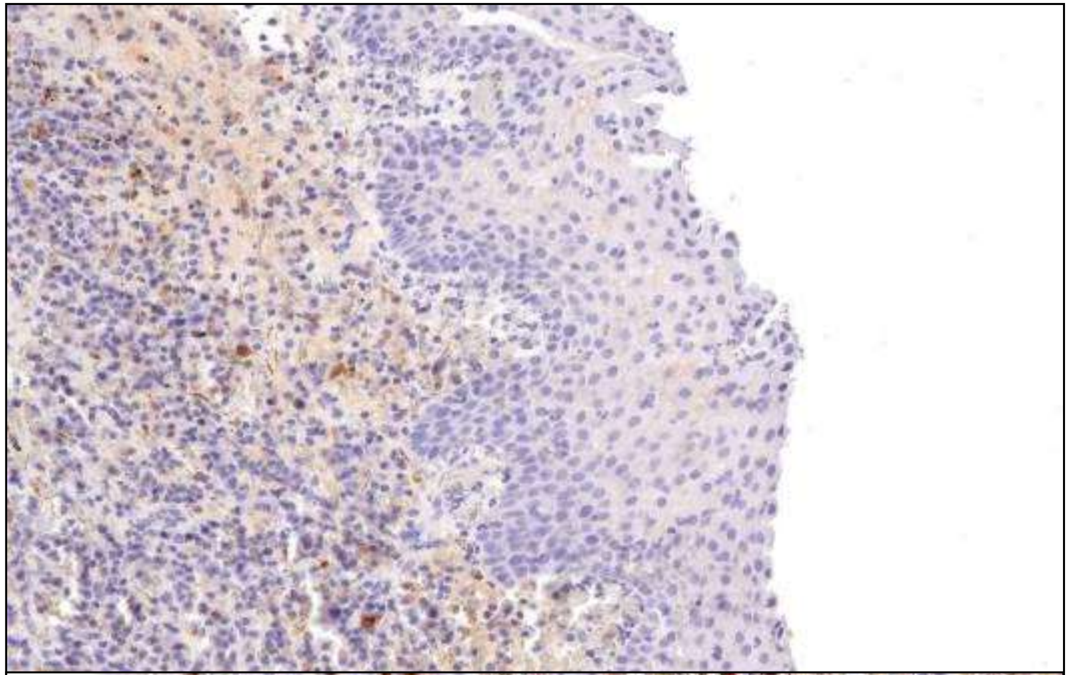

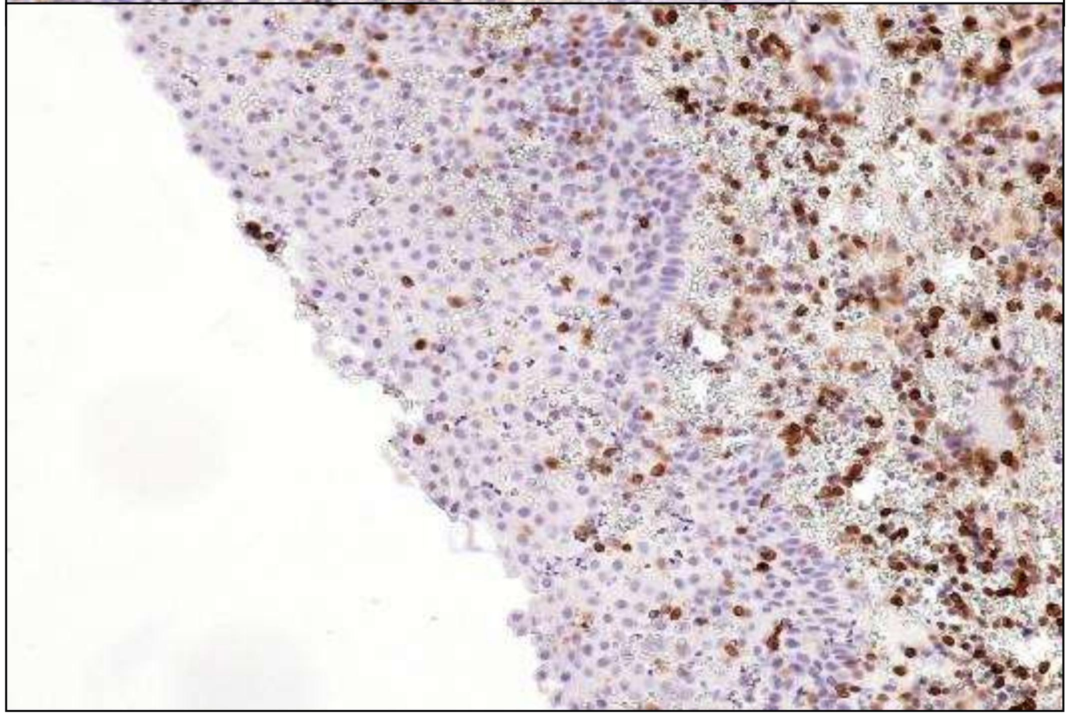

Case S18 – Conclusion

- H&E: relatively thick squamous epithelium without basal palisading, parakeratosis or wavy surface, uneven epithelial stromal interface
- Marked chronic inflammation with an acute component
- Bcl2: negative
- CK17: negative
- Histopathological diagnosis: radicular cyst
- Combined clinical and pathological opinion: cannot be specified; inflammatory or inflamed developmental cyst N.O.S. (compatible with radicular cyst)

Case S19

- 30-year-old male
- Dental hygiene/status: mostly maintained, treated teeth
- OPT: radiolucent lesion around teeth 32-42.
- Operation: removal of cyst and teeth 32, 31, 41, 42.
- Clinical diagnosis: odontogenic cyst, keratocyst?

S19 - OPT

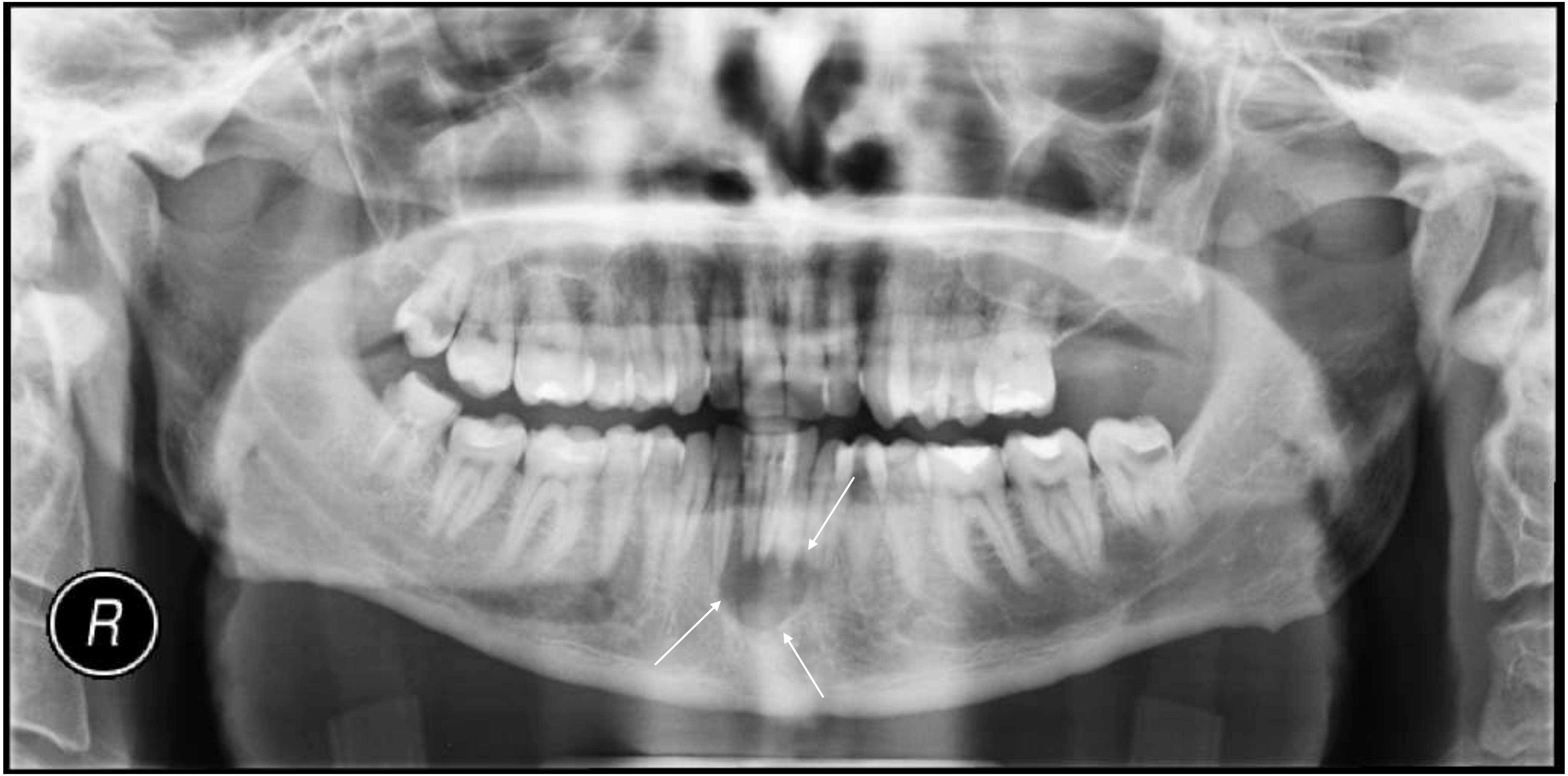

S19 – HE, CK17, bcl2 (x20)

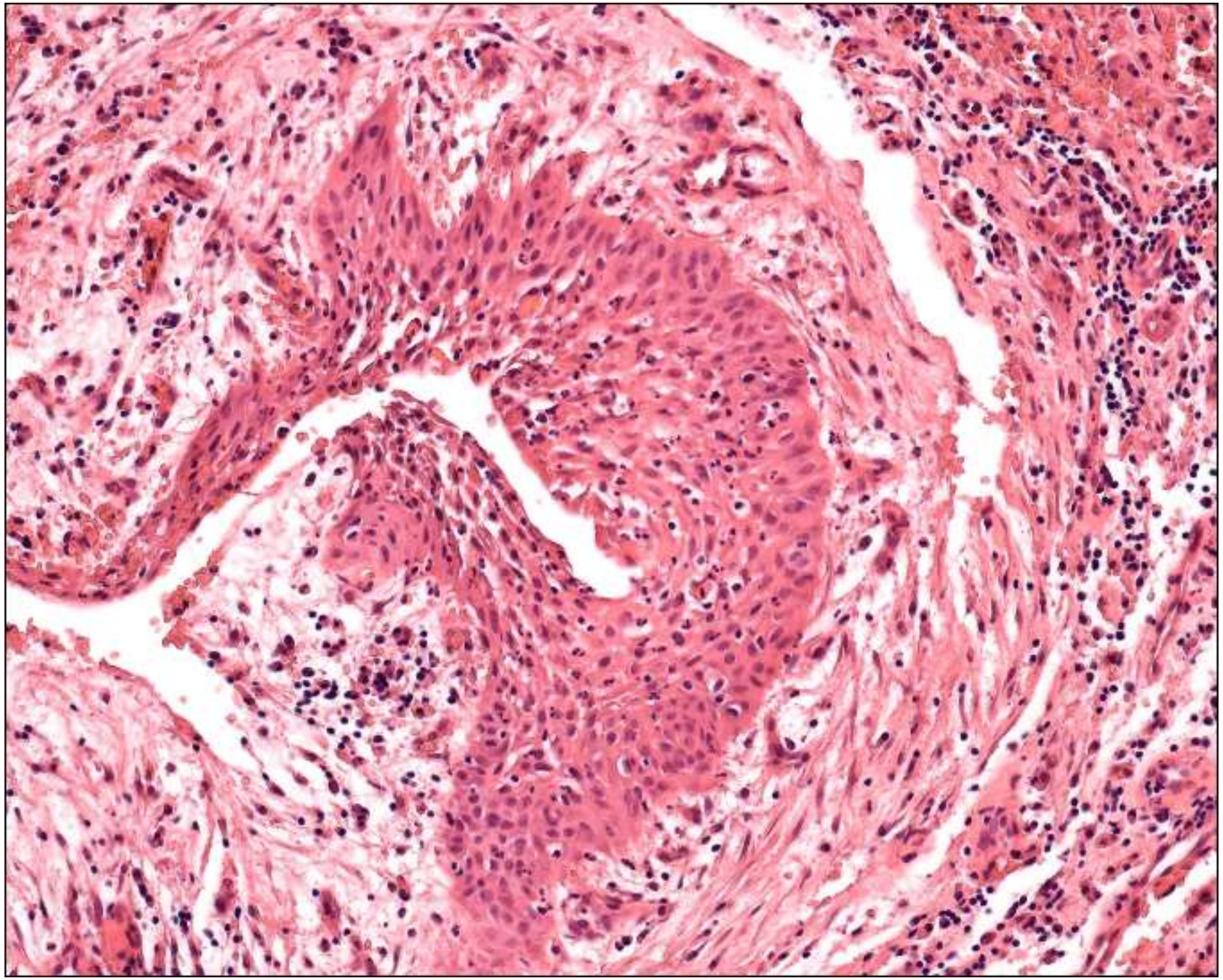

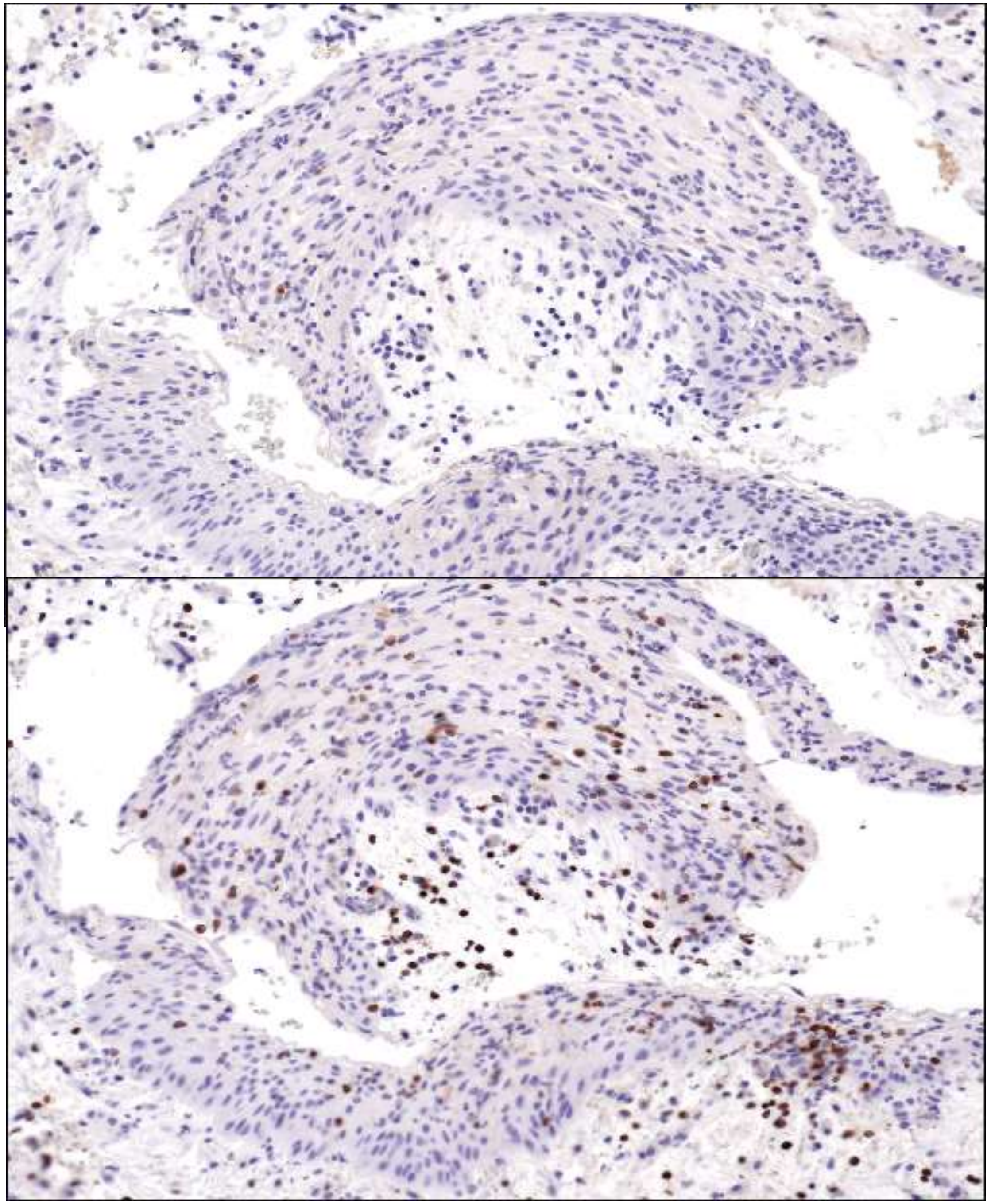

Case S19 – Conclusion

- H&E: thick squamous epithelium without keratinization, basal palisading, parakeratosis or wavy surface, uneven epithelial stromal interface
- Significant chronic inflammation with an acute component
- Bcl2: negative
- CK17: negative
- Histopathological diagnosis: radicular cyst
- Combined clinical and pathological opinion: radicular cyst

Case S20

- 54-year-old male
- Dental hygiene/status: Neglected teeth.
- OPT: radiolucent area around root of tooth 44.
- Operation: removal of cyst and tooth 44.
- Clinical diagnosis: radicular cyst

S20 - OPT

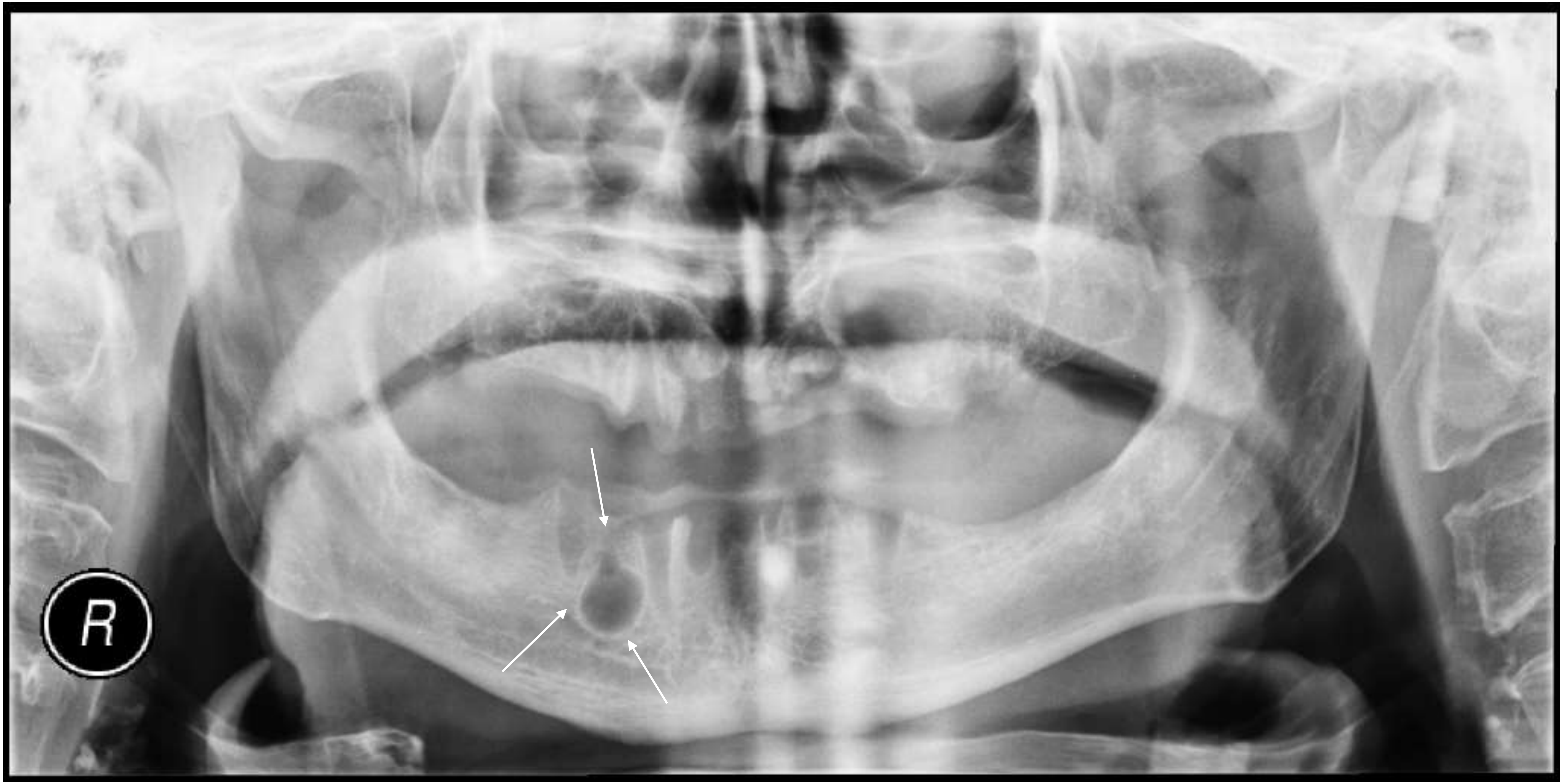

S20 – HE, CK17, bcl2 (x20)

Focal basal bcl2 staining (↑)

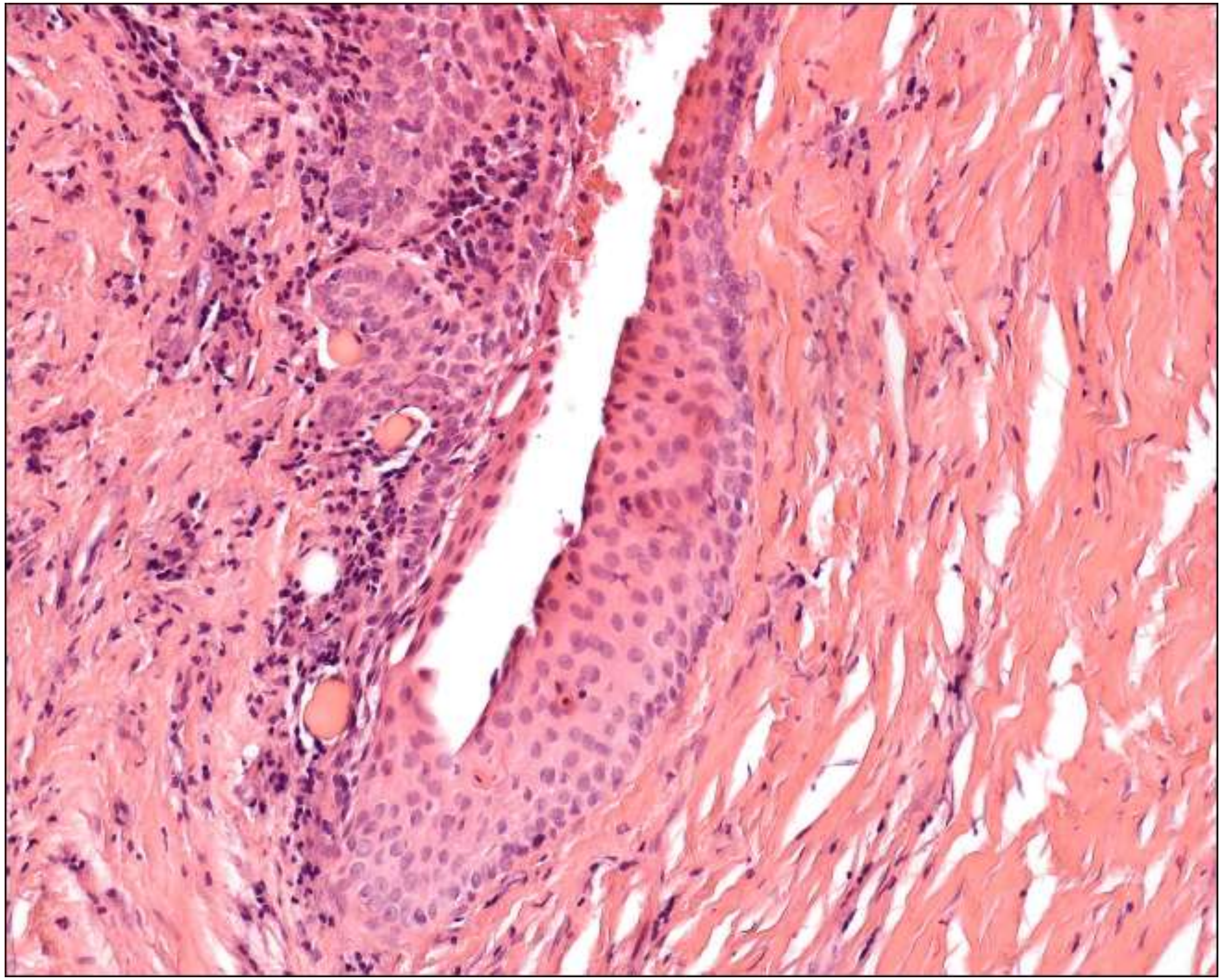

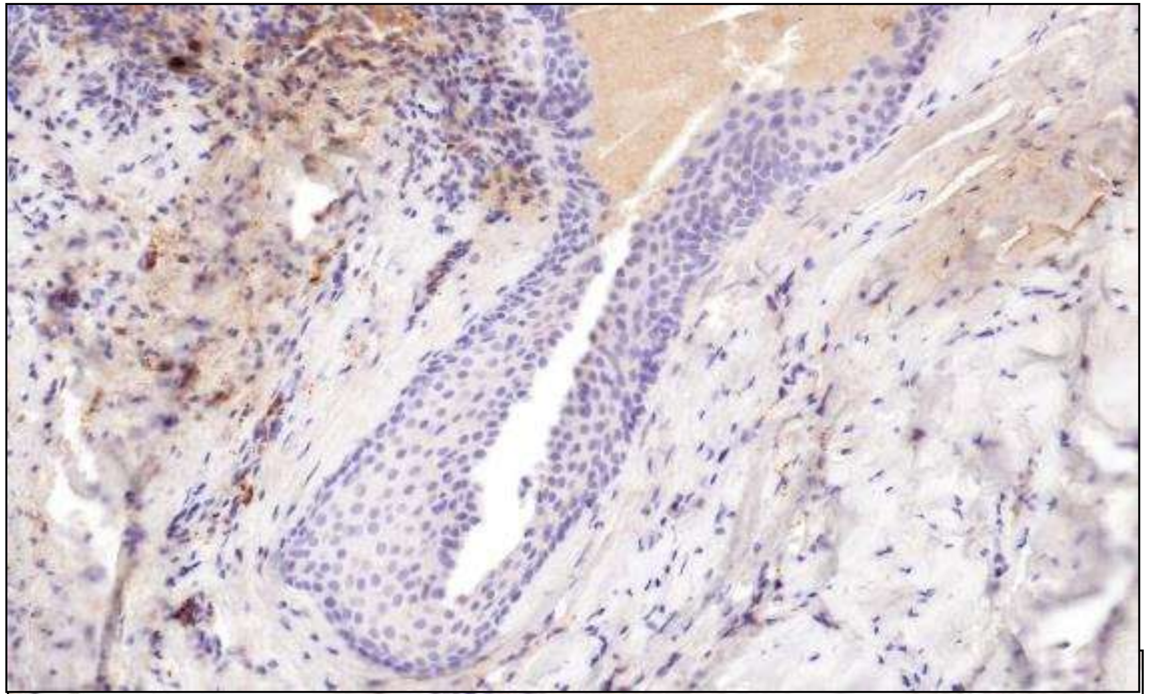

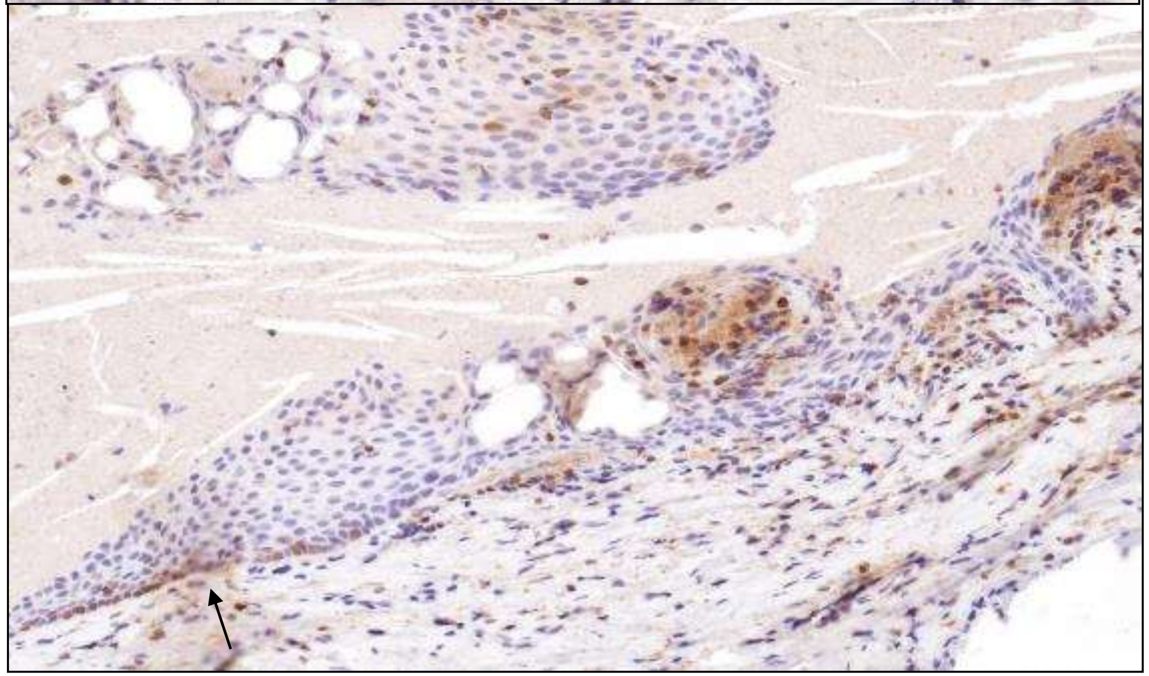

Case S20 – Conclusion

- H&E: thin squamous epithelium without keratinization, basal palisading, parakeratosis or wavy surface, even epithelial stromal interface
- Chronic inflammation is scant.
- Bcl2: negative; focal basal positivity
- CK17: negative
- Histopathological diagnosis: radicular cyst, residual cyst?
- Combined clinical and pathological opinion: radicular cyst

Case S21

- 46-year-old male
- Dental hygiene/status: conserved, treated teeth. Tooth 38 is not visible from oral cavity.
- OPT: radiolucent area is present around tooth 38.
- Operation: removal of tooth 38, 48, cystectomy
- Clinical diagnosis: keratocyst?

S21 – OPT

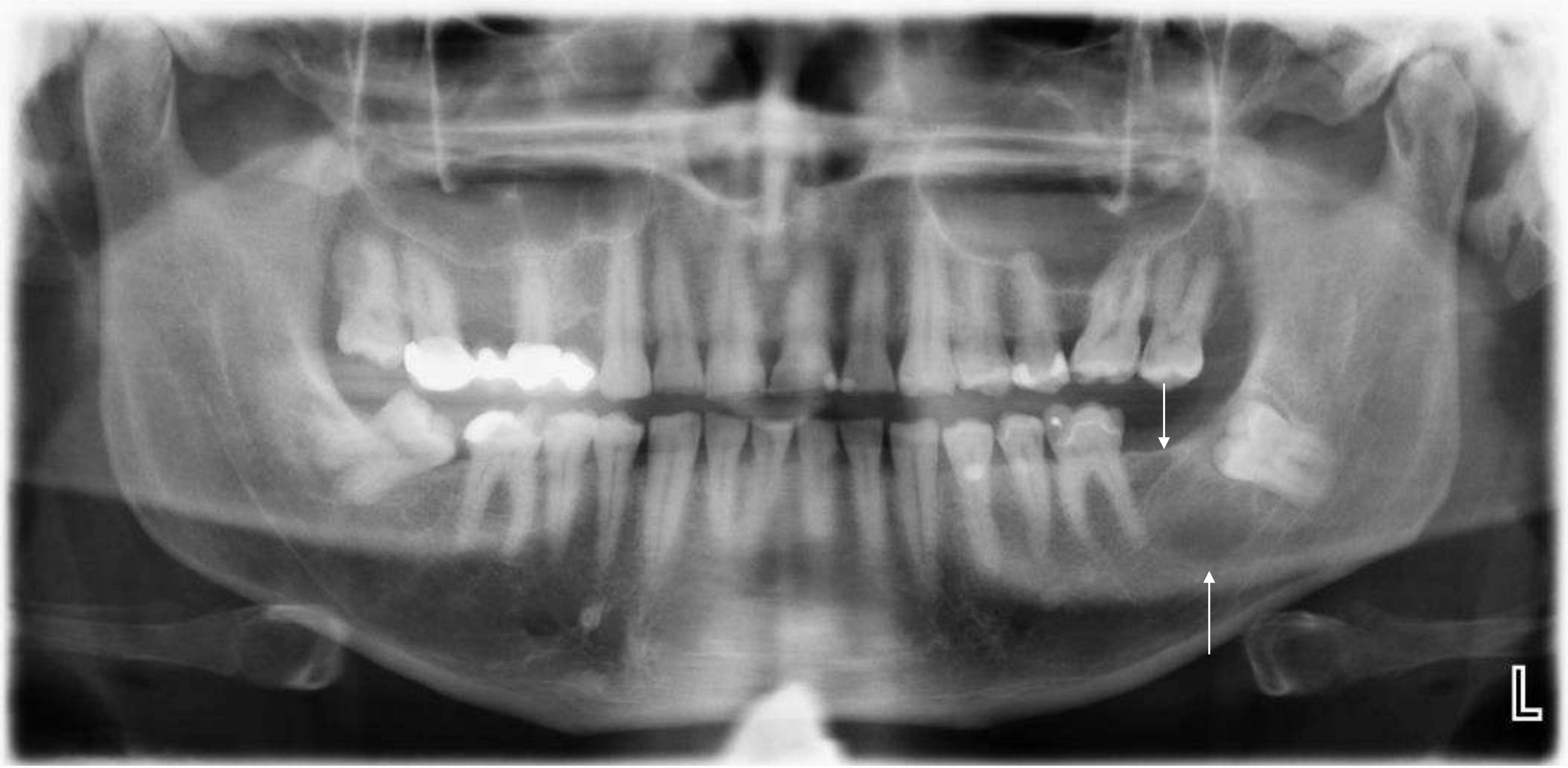

S21 – HE, CK17, bcl2 (x20)

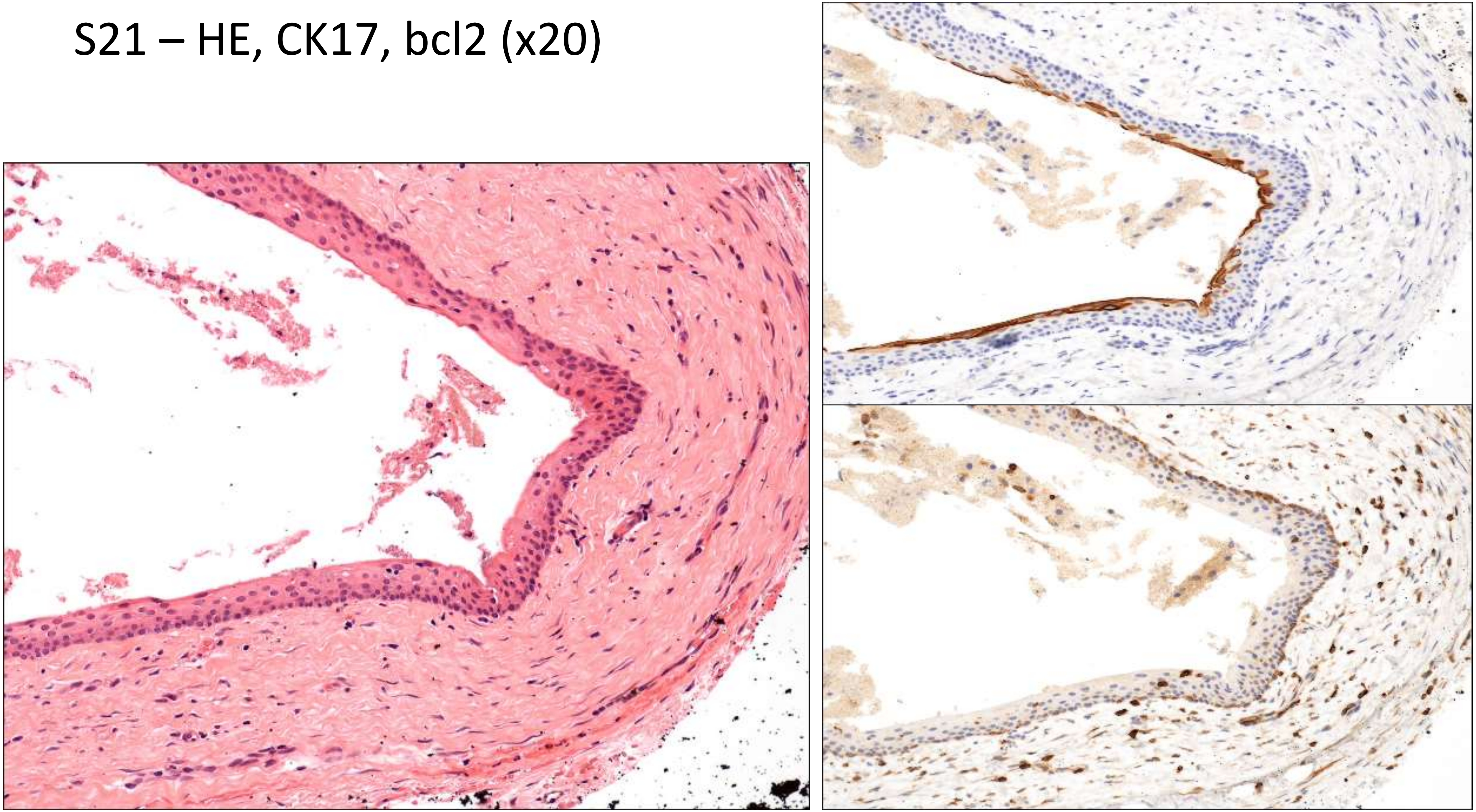

S21 – CK17, bcl2 (x20)

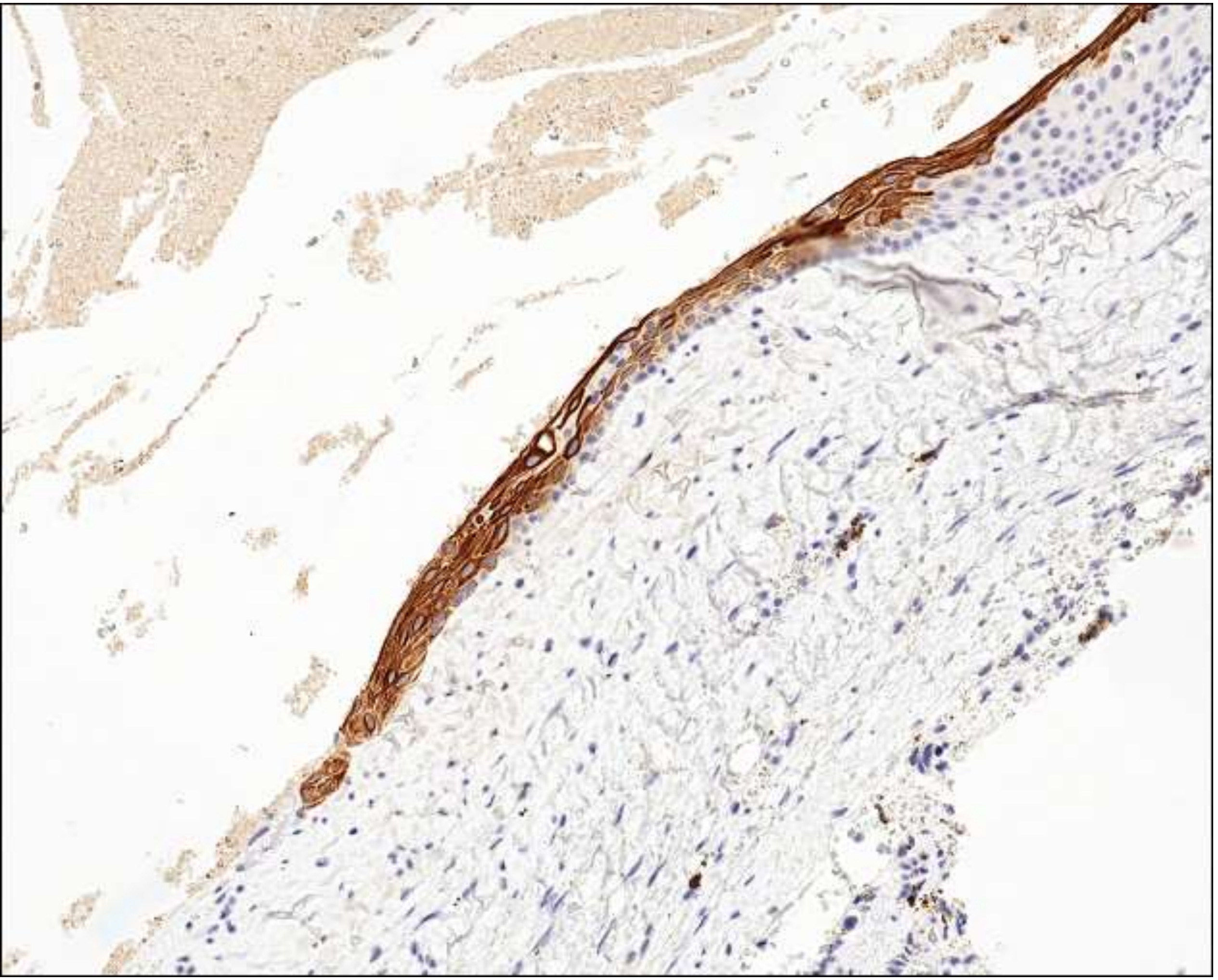

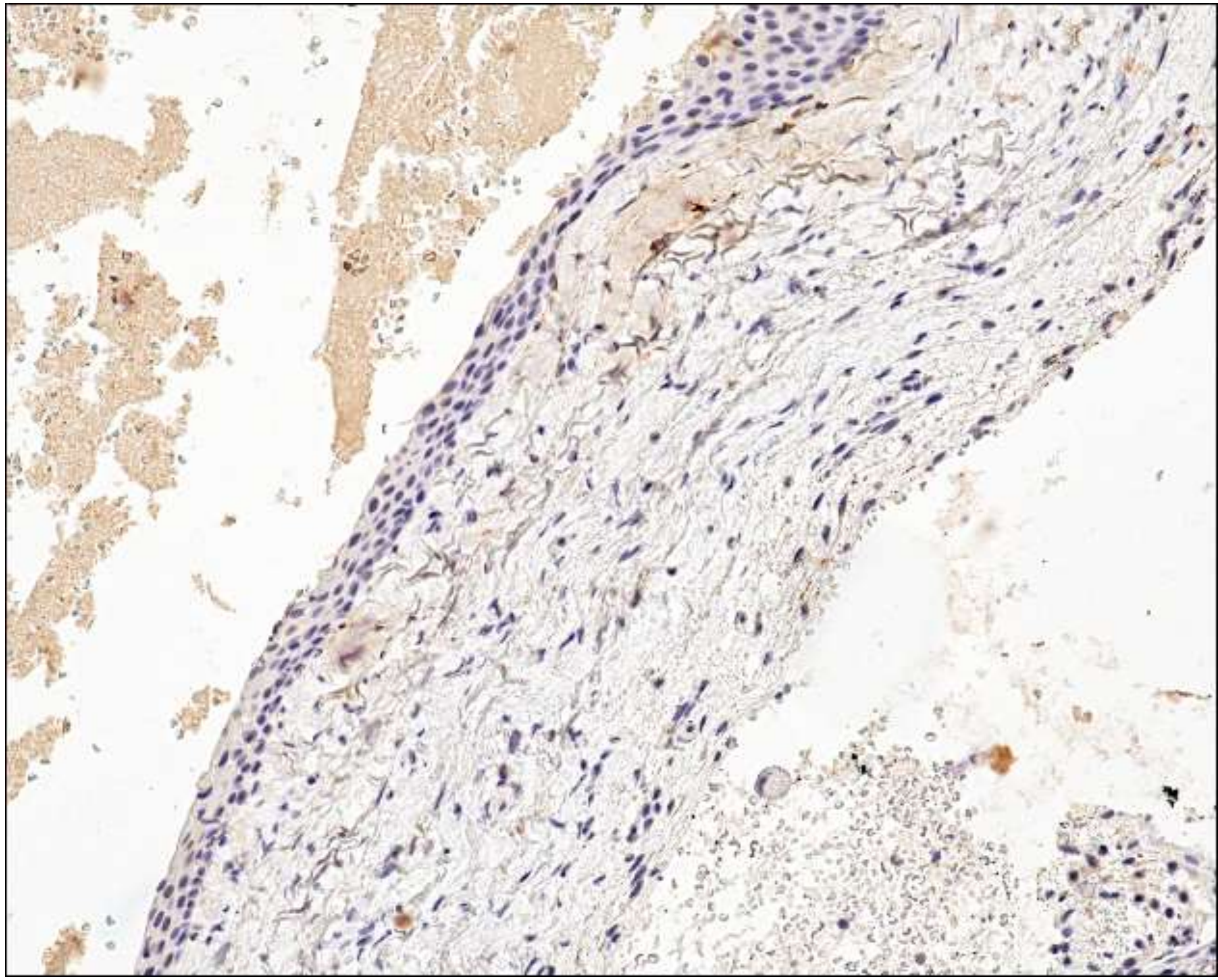

Case S21 – Conclusion

- H&E: thin squamous epithelium without parakeratosis, surface undulation and basal palisading, with even epithelil stromal interface
- Inflammation is absent
- Bcl2: moderate basal expression
- CK17: apical (surface) positivity, minimal focal transepithelial staining
- Histopathological diagnosis: follicular cyst
- Combined clinical and pathological opinion: dentigerous (follicular) cyst

Case S22

- 72-year-old male
- Dental hygiene/status: neglected teeth, removed tooth 26
- CBCT: large intraosseal cystic lesion around tooth 48
- Operation: cystectomy
- Clinical diagnosis: keratocyst?

S22 – OPT

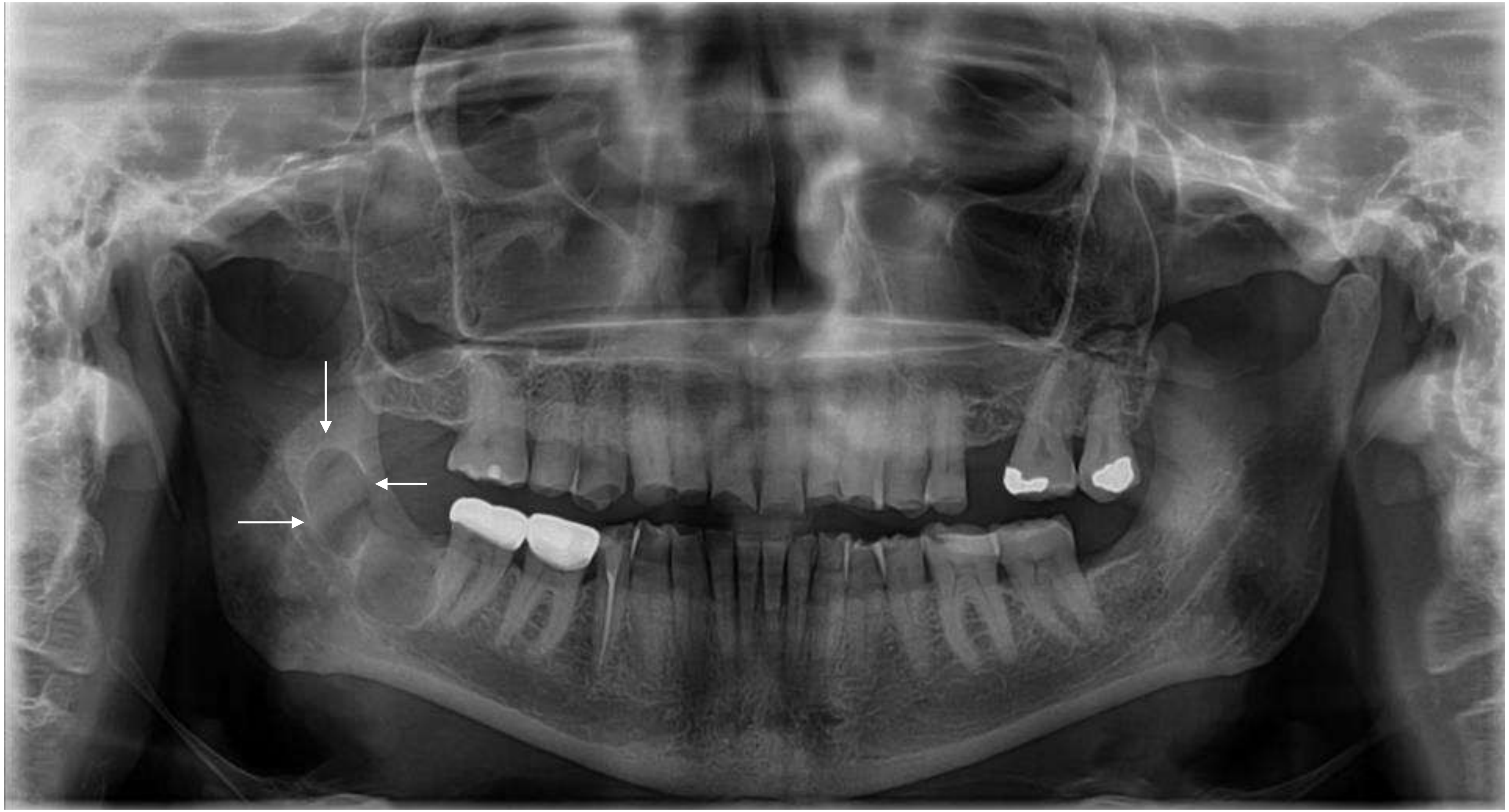

S22 – HE, CK17, bcl2 (x20)

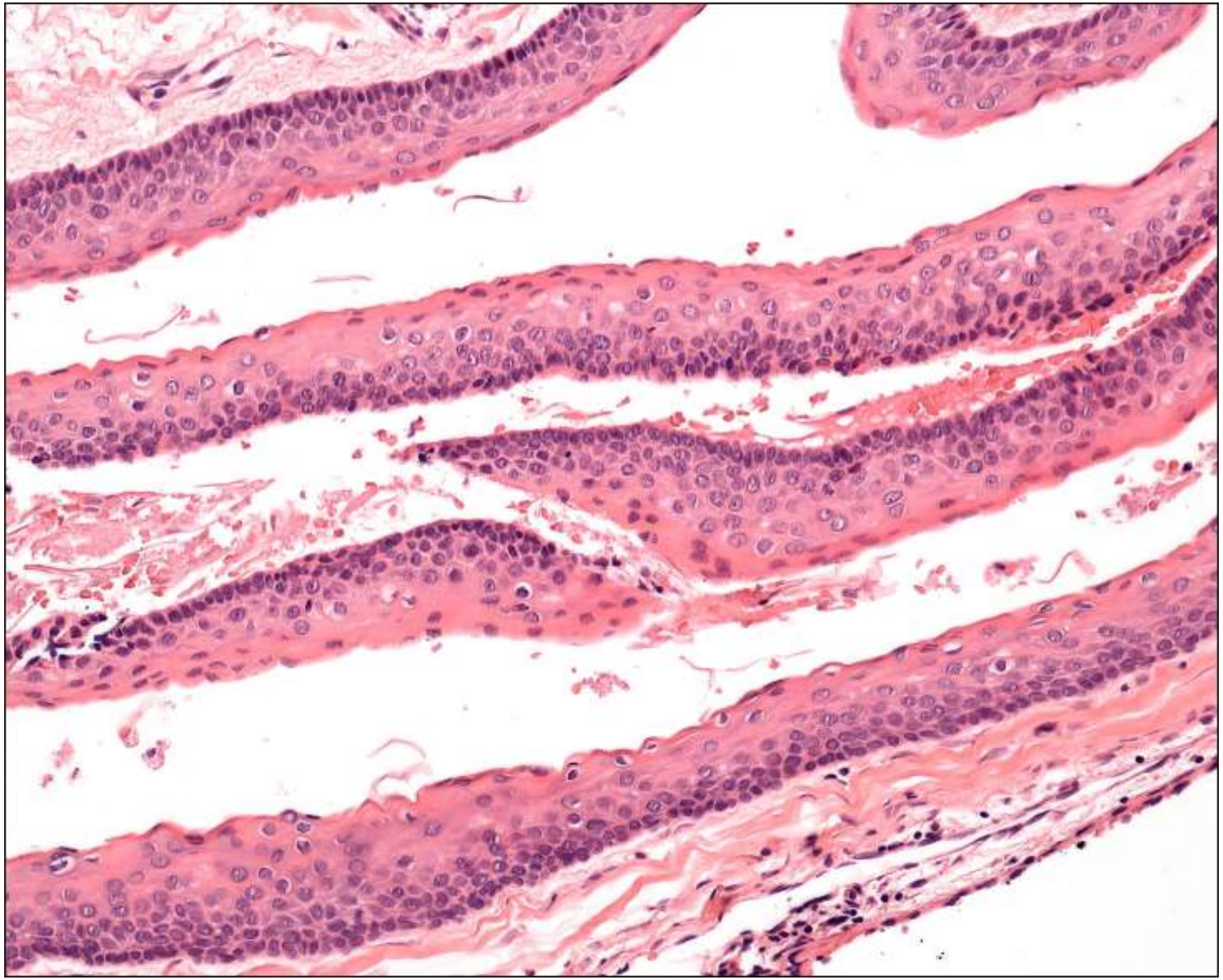

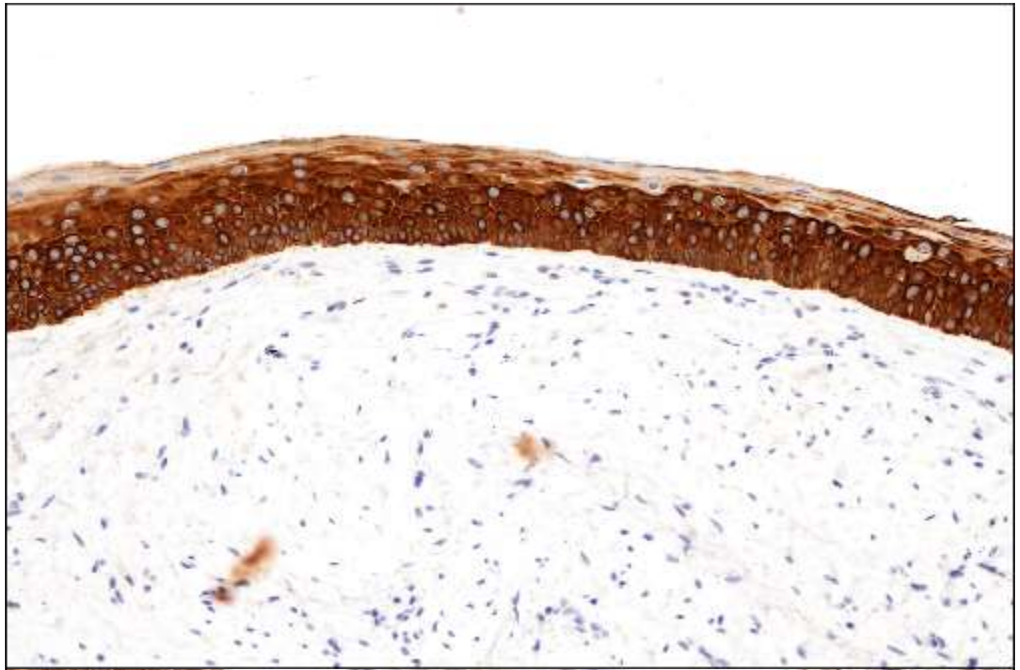

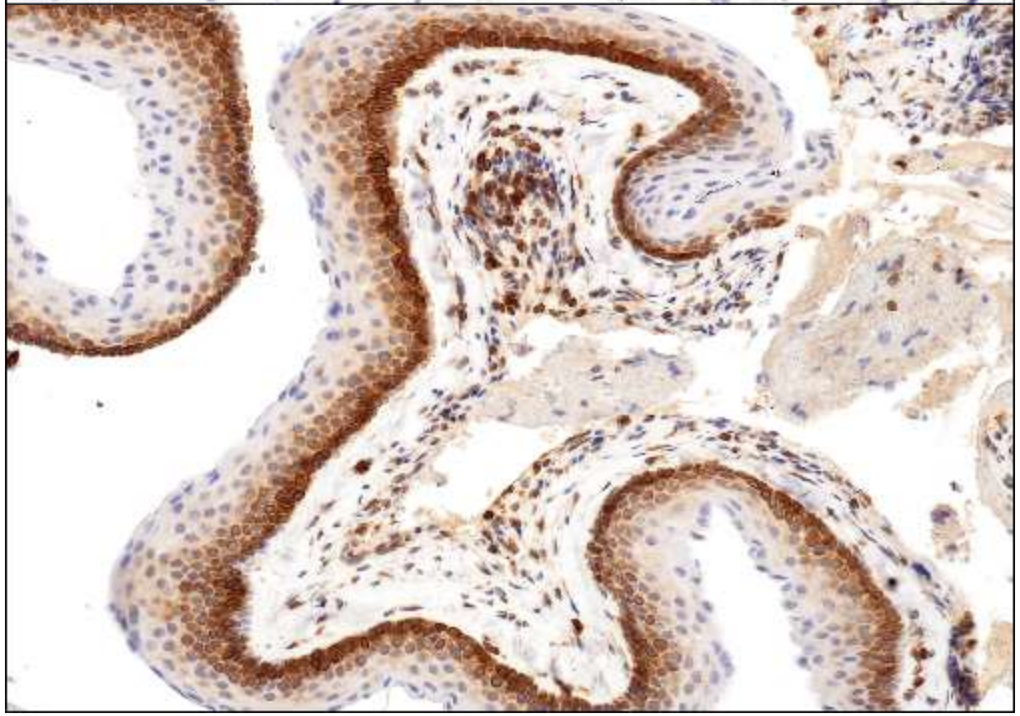

Case S22 – Conclusion

- H&E: thin parakeratotic epithelium with wavy surface and basal palisading, smooth, even epithelial stromal interface
- Inflammation absent
- Bcl2: basal, suprabasal positivity
- CK17: transepithelial positivity
- Histopathological diagnosis: OKC
- Combined clinical and pathological opinion: OKC

Case S24

- 33-year-old male with verified Gorlin-Goltz syndrome
- Dental hygiene/status: Neglected teeth. Removal of several teeth is described.
- OPT and CT: radiolucent areas in the left molar region of the mandible and around tooth 33.
- Operation: cystectomy.
- Clinical diagnosis: keratocyst?

S24 - OPT

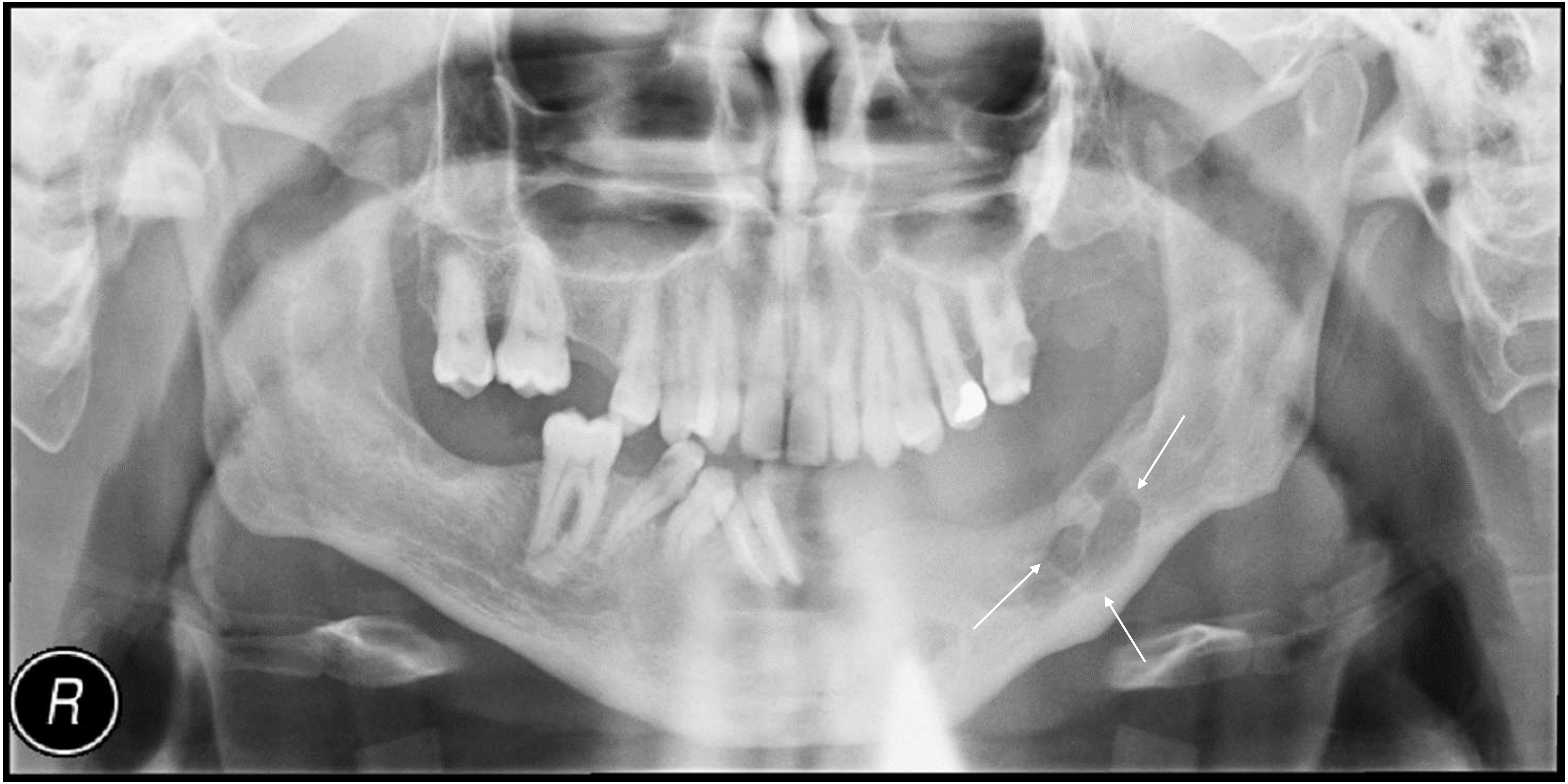

S24 – HE, CK17, bcl2 (x20)

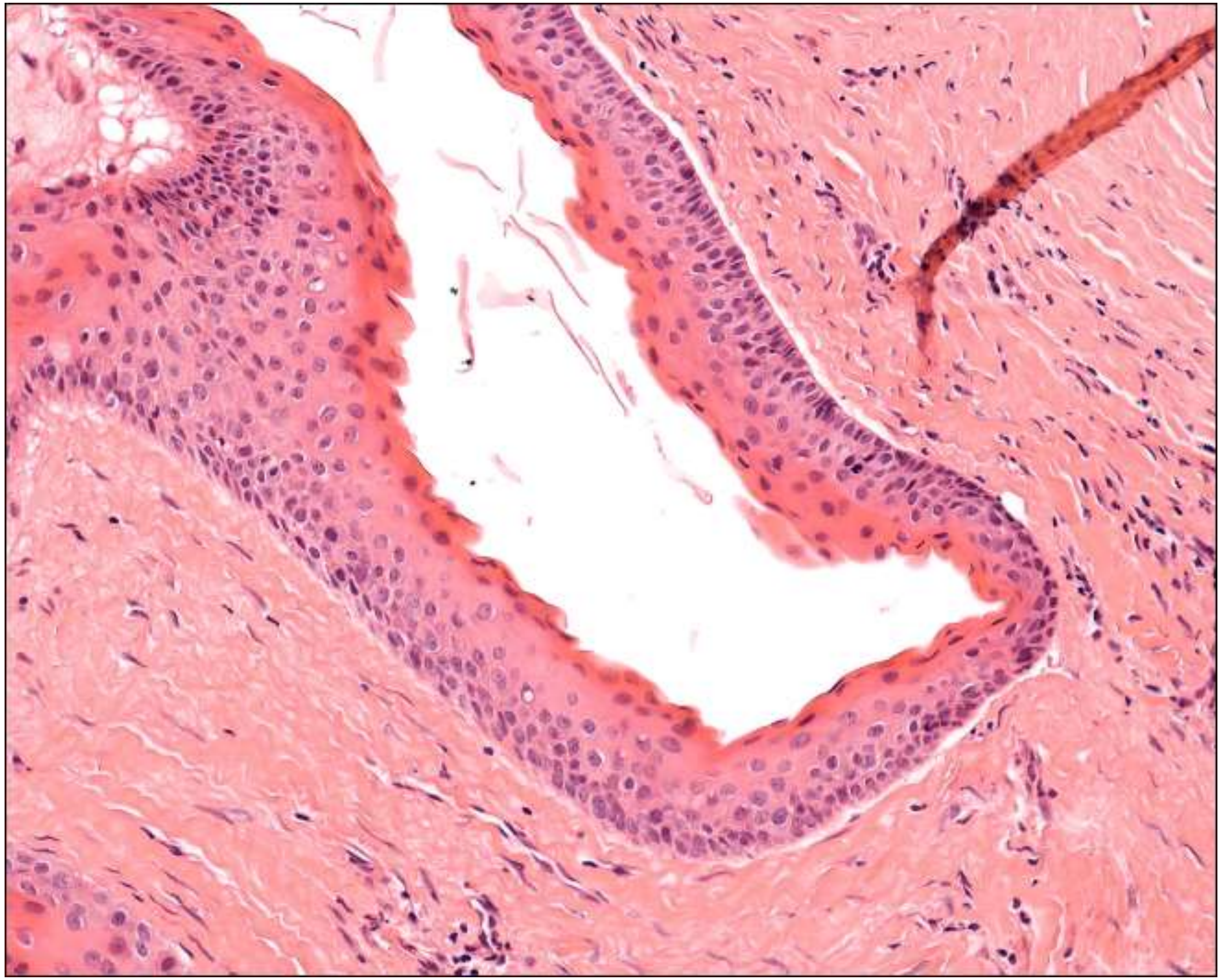

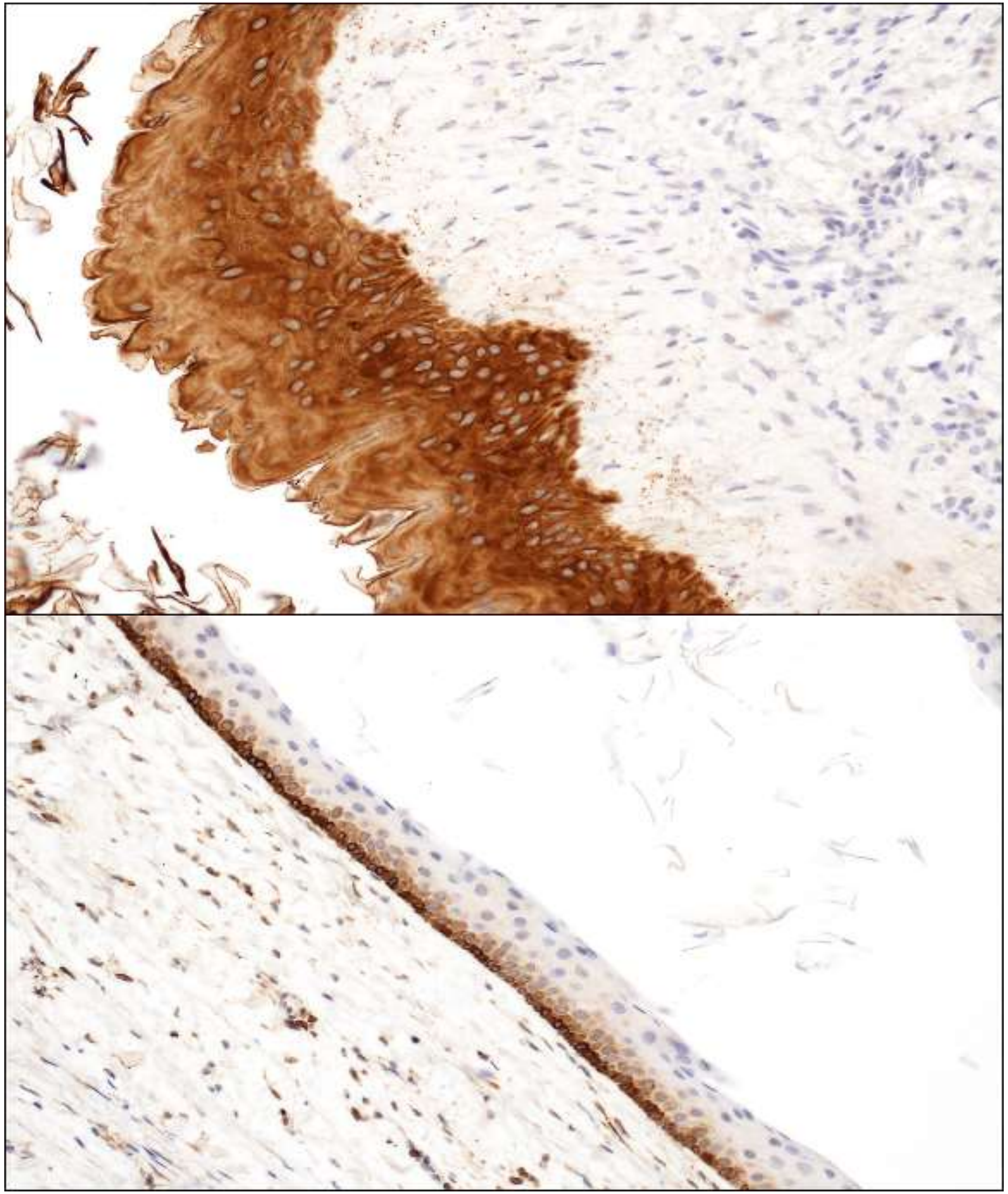

S24 – HE, CK17, bcl2 (x2.5)

Area without basal palisading and bcl2 staining (↑)

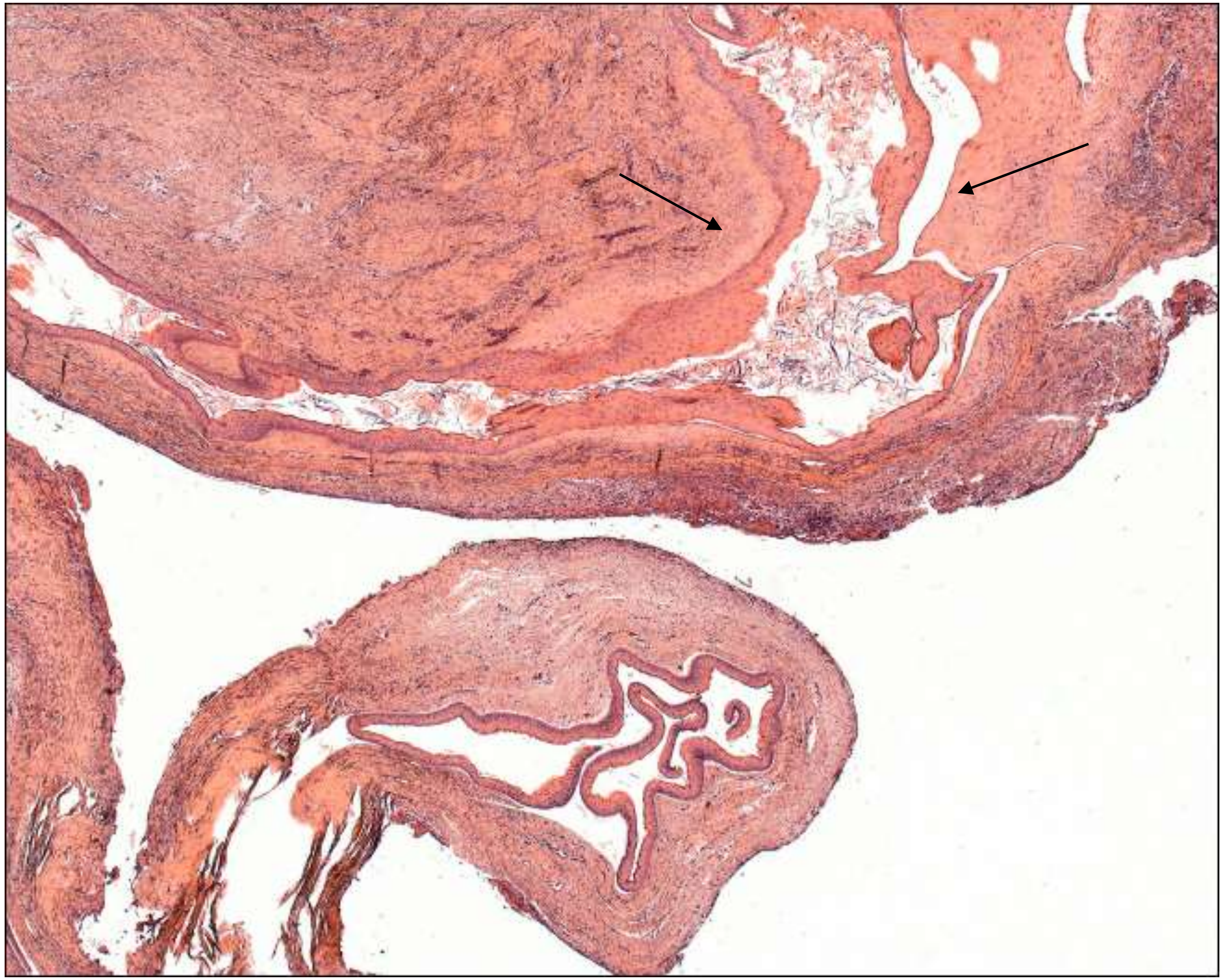

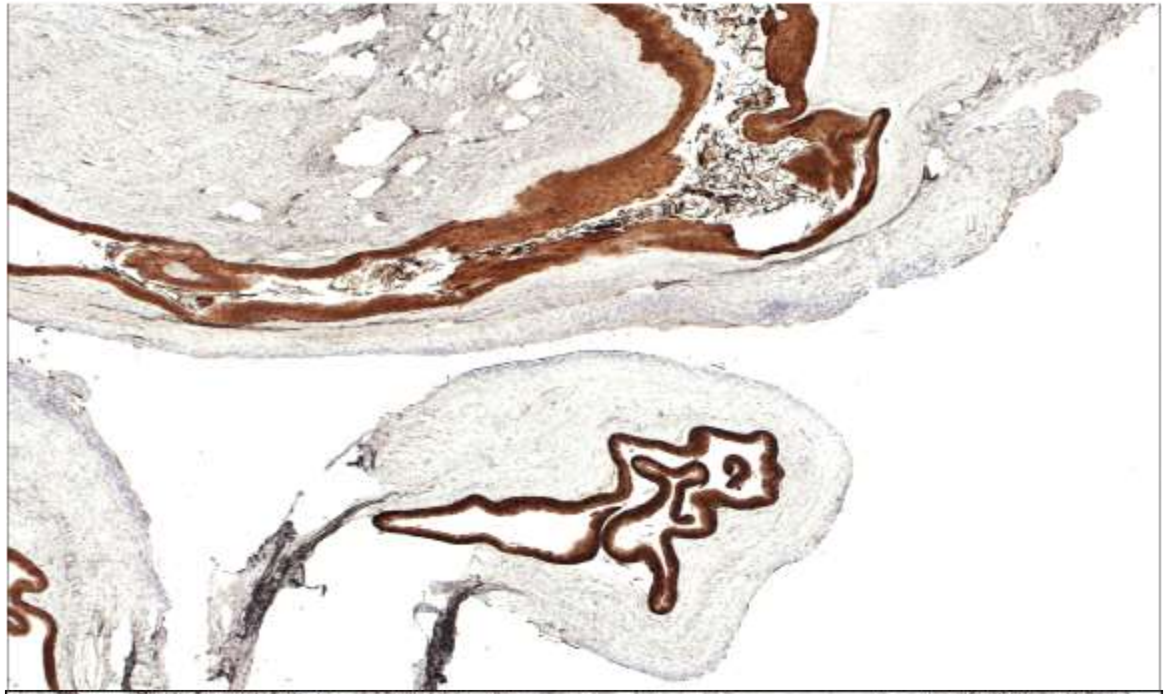

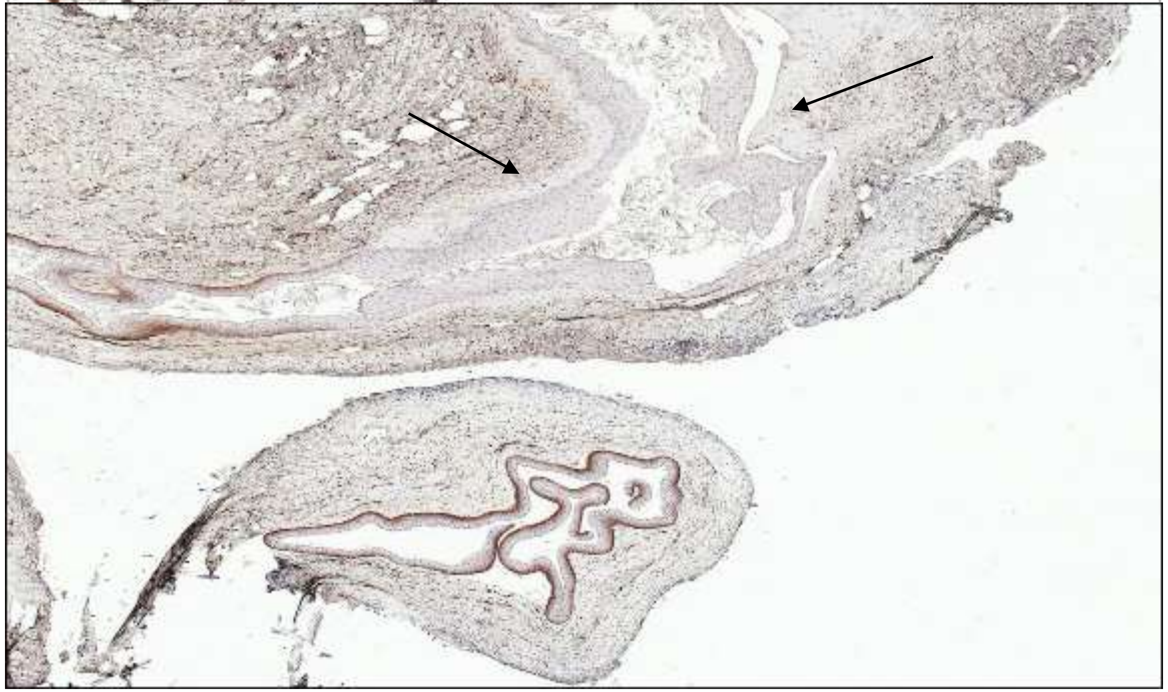

Case S24 – Conclusion

- H&E: thin parakeratotic epithelium with wavy surface and basal palisading, even epithelial stromal interface; area of thicker parakeratotic epithelium without basal palisading
- Inflammation absent
- Bcl2: basal, suprabasal positivity; missing from altered epithelium without basal palisading
- CK17: transepithelial positivity
- Histopathological diagnosis: OKC
- Combined clinical and pathological opinion: OKC

Case S25(-26)

- 38-year-old male
- Dental hygiene/status: partly maintained, treated teeth. Palpable, but non-fluctuating swelling in the region of the vital teeth 37, 36. Horizontal impaction of tooth 48.
- OPT: multiple cystic radiolucent lesions in the left mandible.
- Operation: Removal of cysts and teeth 36, 37
- Clinical diagnosis: ameloblastoma? mandibular cyst?
- Note: the larger cyst was biopsied first, then the two cysts on the OPT removed and sent in as a single histology specimen; because of the impossibility to separate the cysts, this is considered as a simple cyst for all purposes.

S25(-26) - OPT

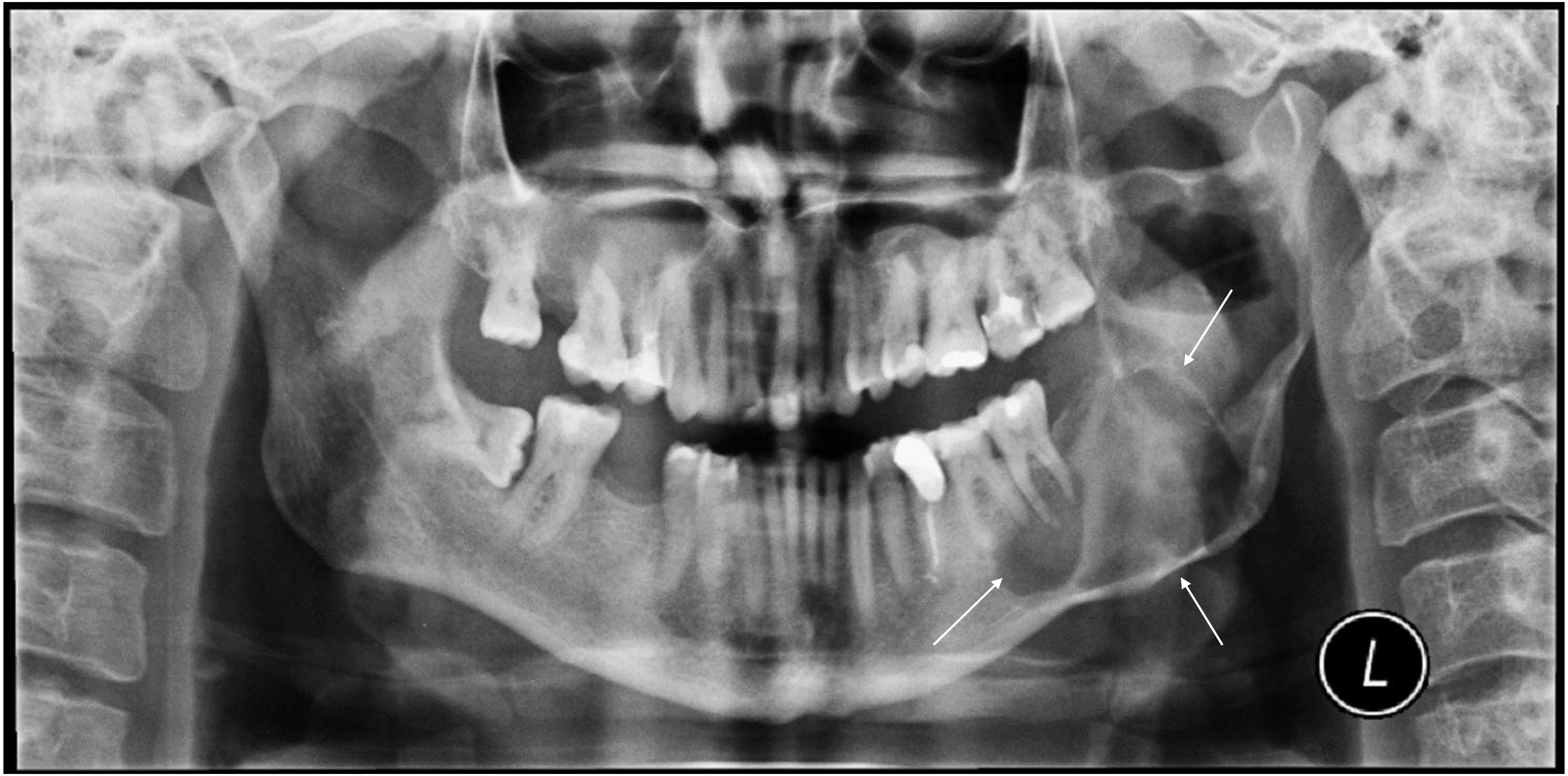

S25(-26) – HE, CK17, bcl2 (x20) biopsy

Inflamed area lacking bcl2 staining (↑) (and CK17 not shown); note staining of lymphocytes

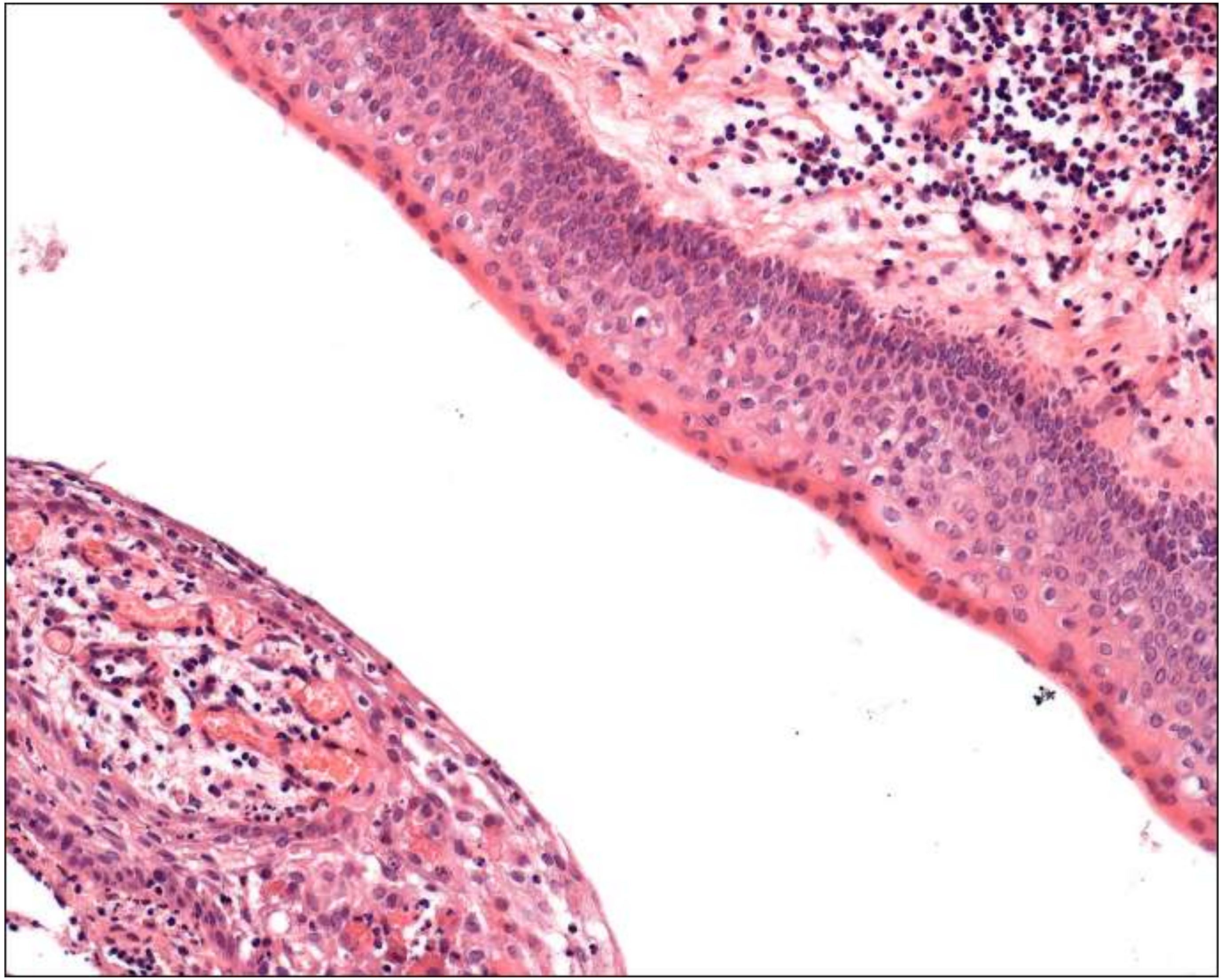

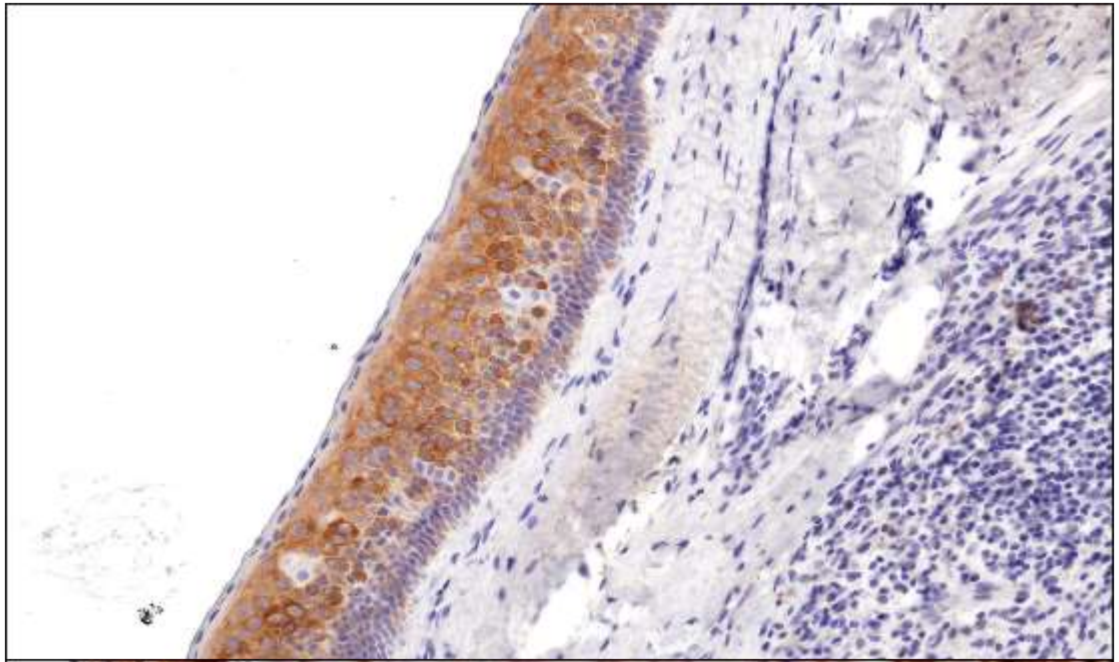

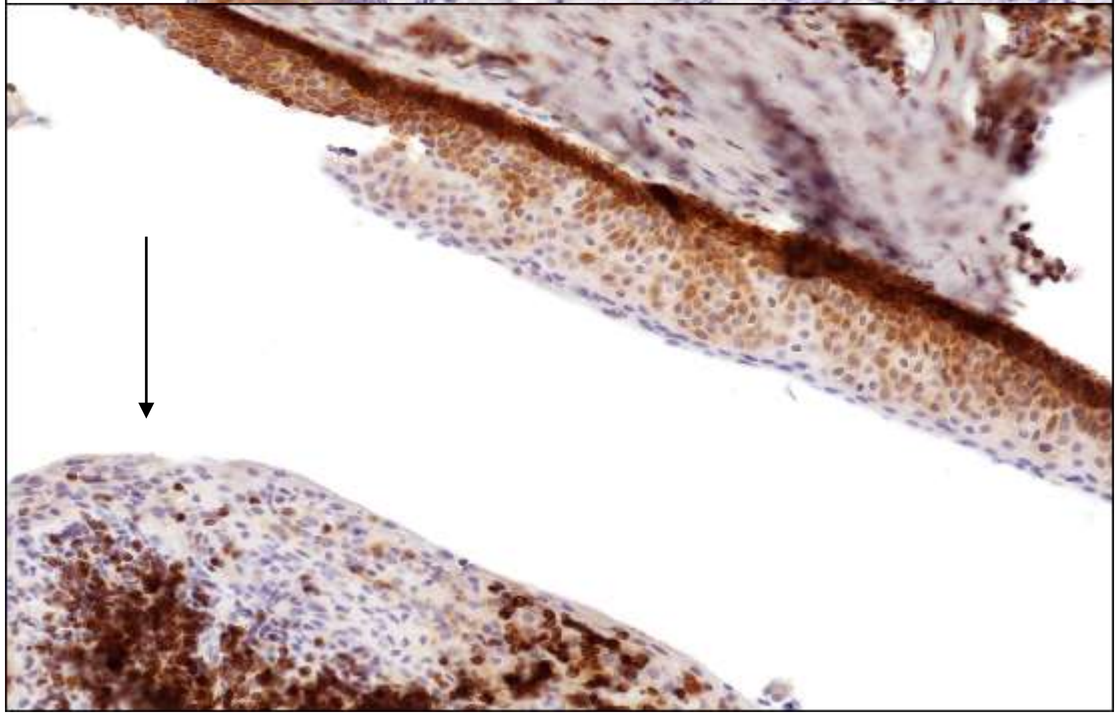

S25(-26) HE, CK17, bcl2 (scan failure) (x10)

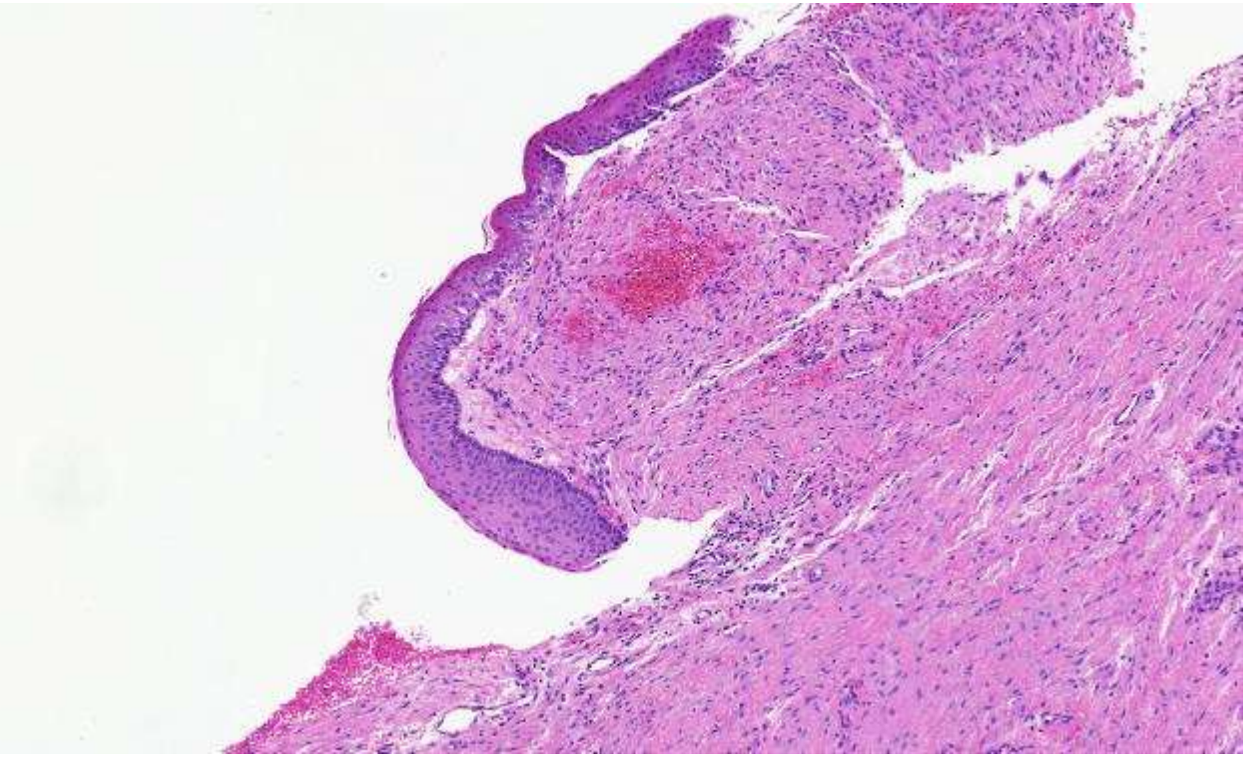

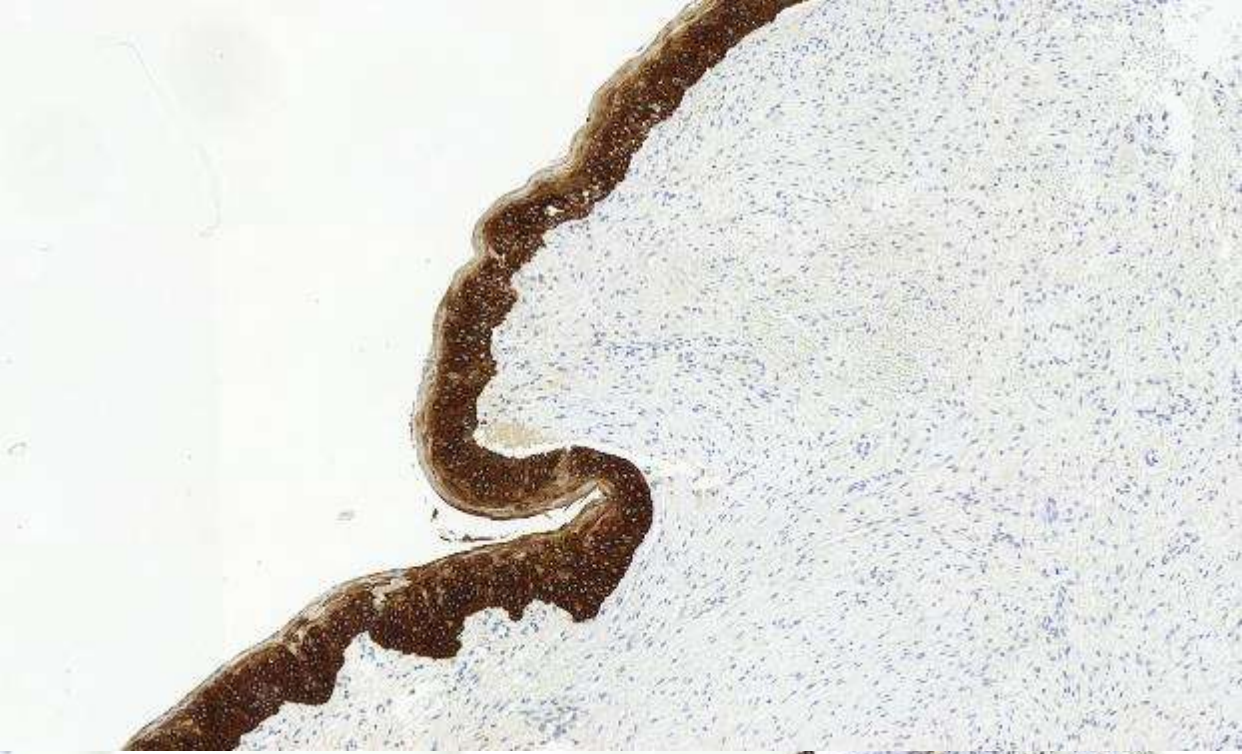

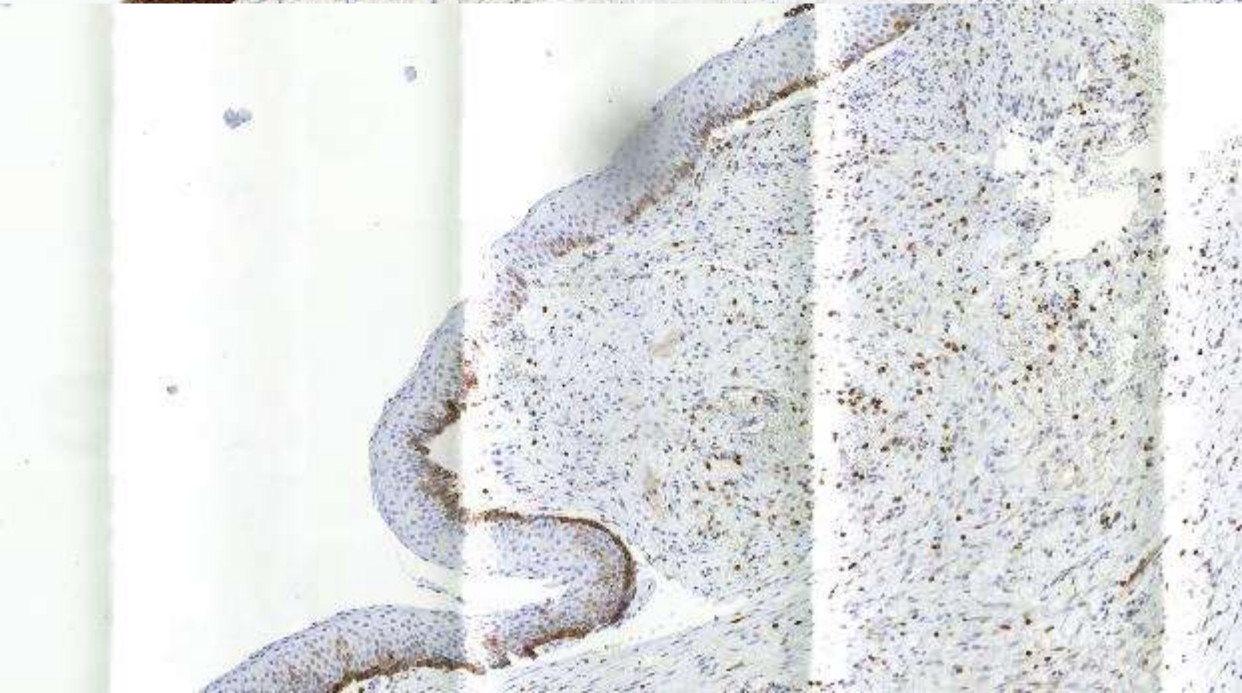

S25(-26) CK17, bcl2 (scan failure) (x8)

Superficial CK17 staining and loss of basal bcl2 staining in the inflamed area

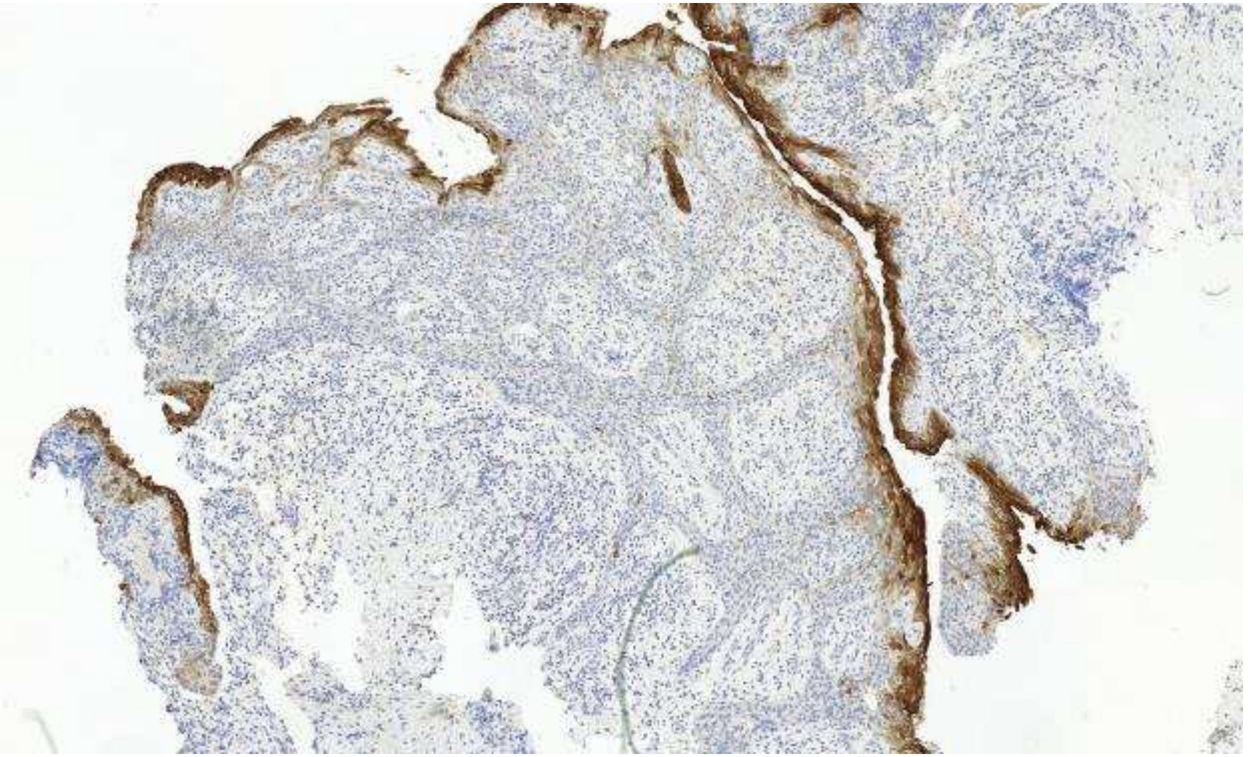

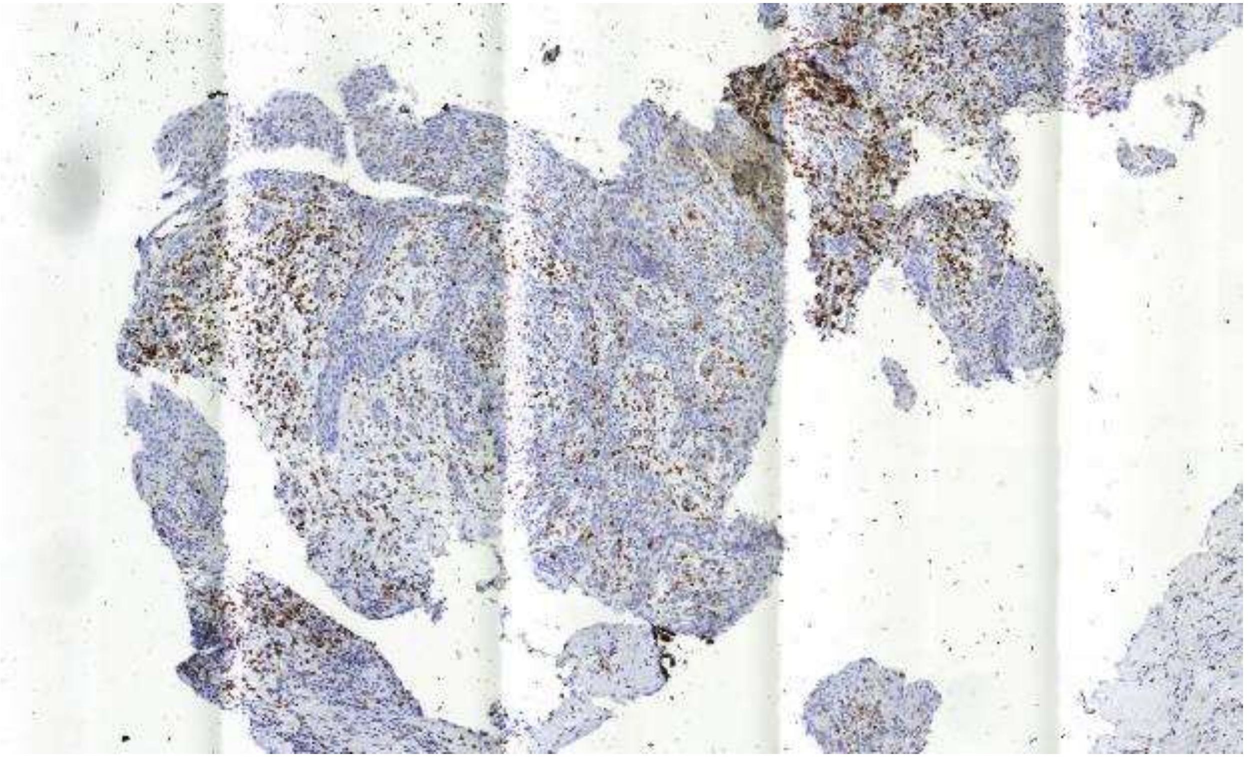

Case S25(-26) – Conclusion

- H&E: relatively thick, focally parakeratotic epithelium with basal palisading and focal wavy surface & areas of inflammation covered by non keratinizing thick epithelium
- Chronic inflammation present focally; cholesterol granuloma.
- Bcl2: basal, suprabasal positivity, missing from the non-keratinizing epithelium
- CK17: transepithelial positivity, , missing from the non-keratinizing epithelium
- Histopathological diagnosis: radicular cyst
- Combined clinical and pathological opinion: OKC

Case S27

- 72-year-old male
- Dental hygiene/status: partly maintained, partly missing, treated teeth. Tooth 38 is not visible.
- OPT: inverse impaction of tooth 38. Radiolucent cyst around teeth 38-36.
- Operation: removal of cyst and teeth 15, 38, 37, 47, 48
- Clinical diagnosis: keratocyst?

S27 – HE, CK17, bcl2 (x4)

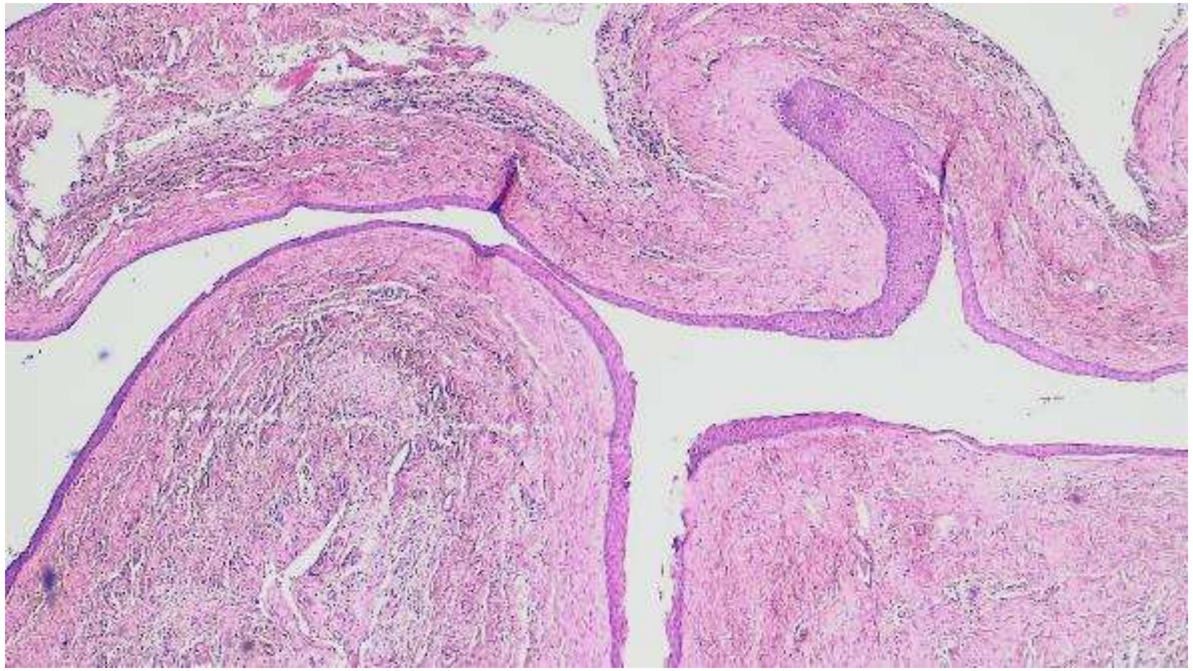

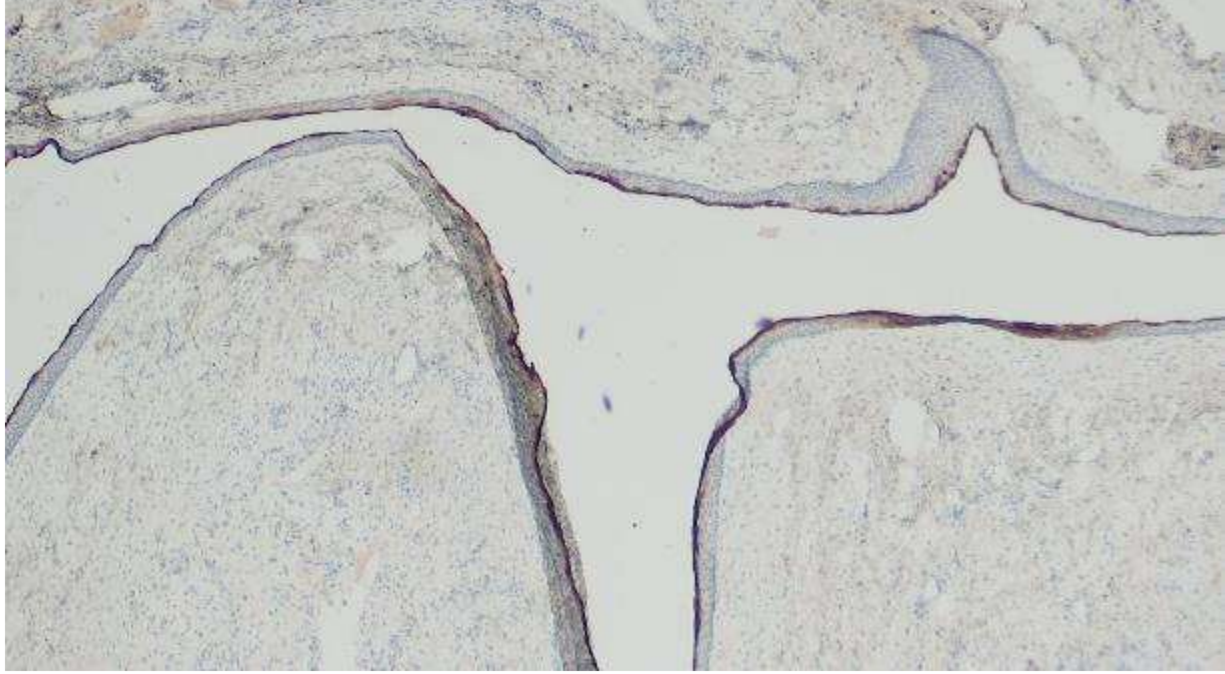

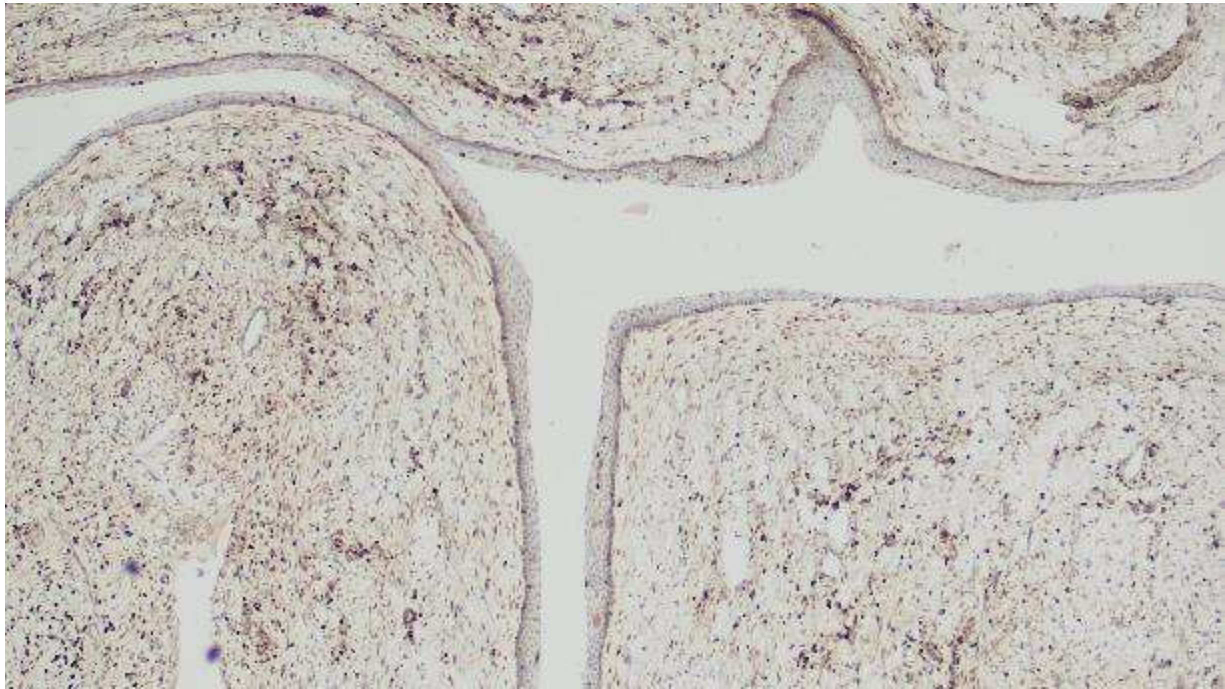

S27 – HE, CK17, bcl2 (x40)

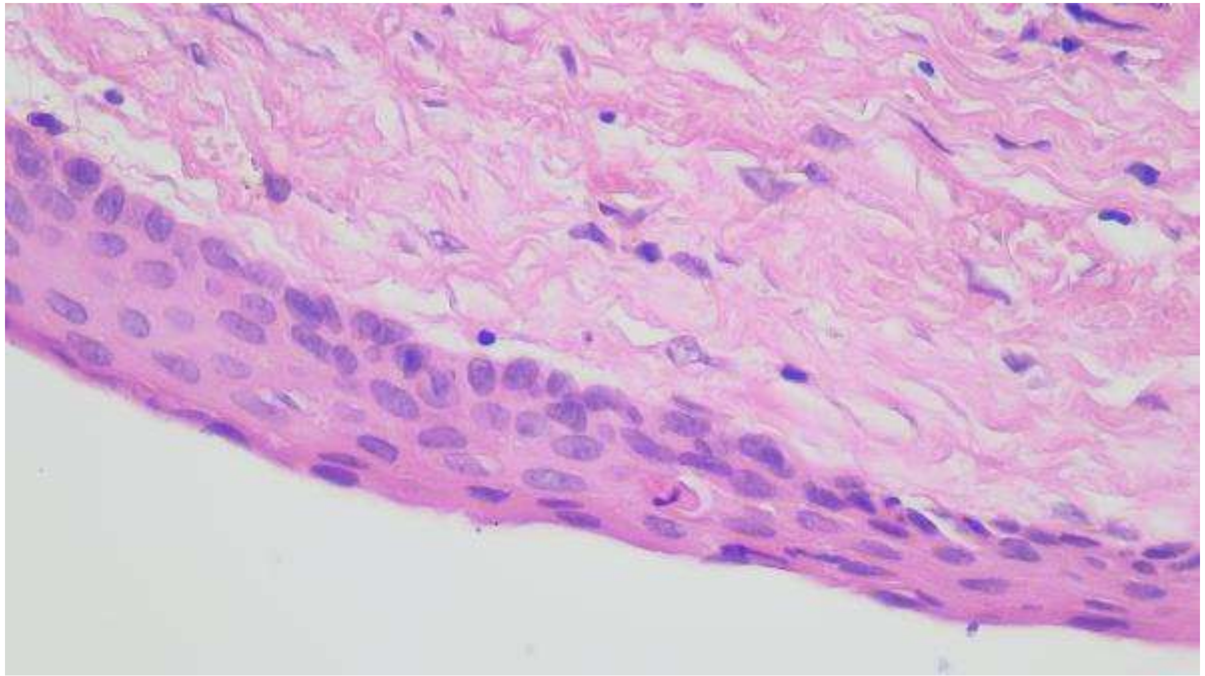

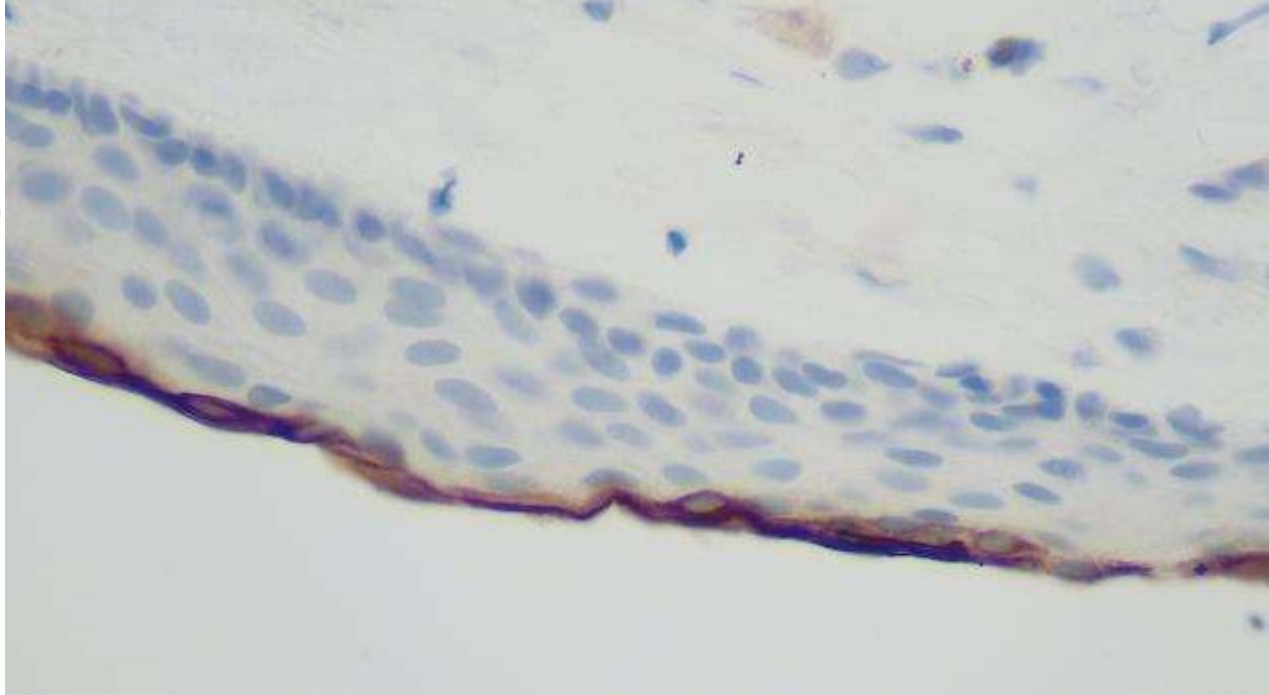

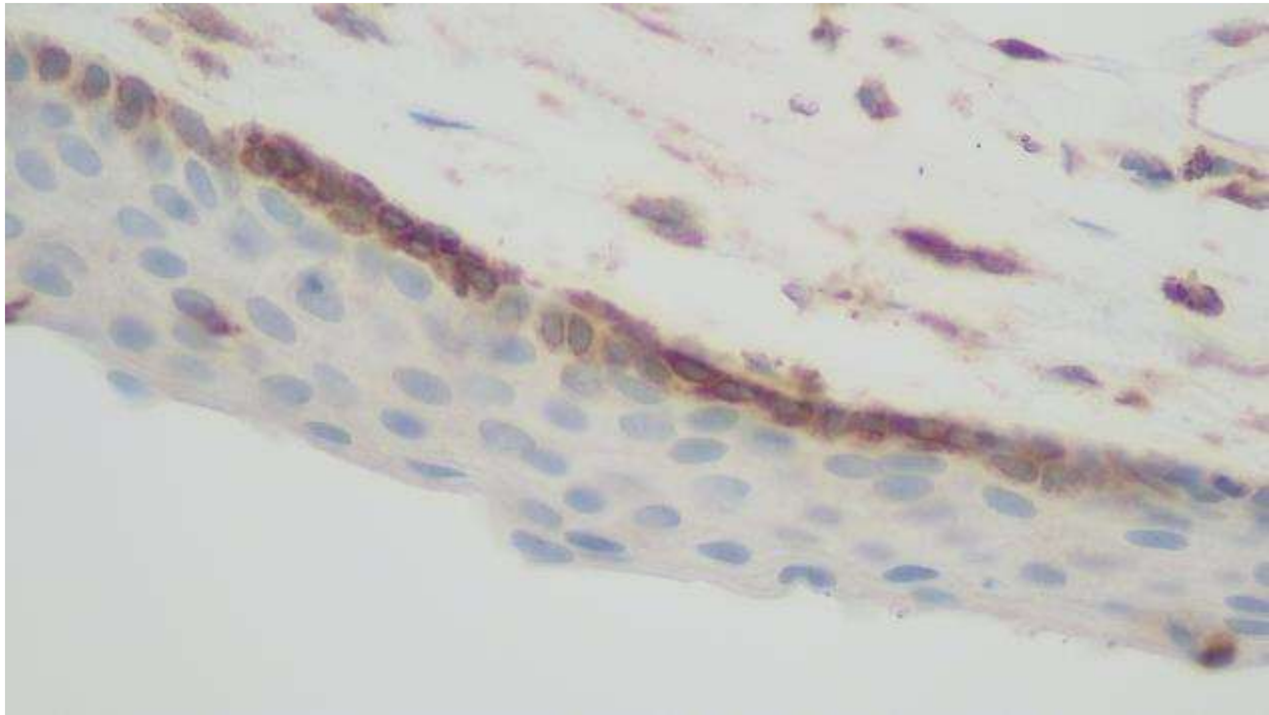

S27 – HE, CK17, bcl2 (x20)

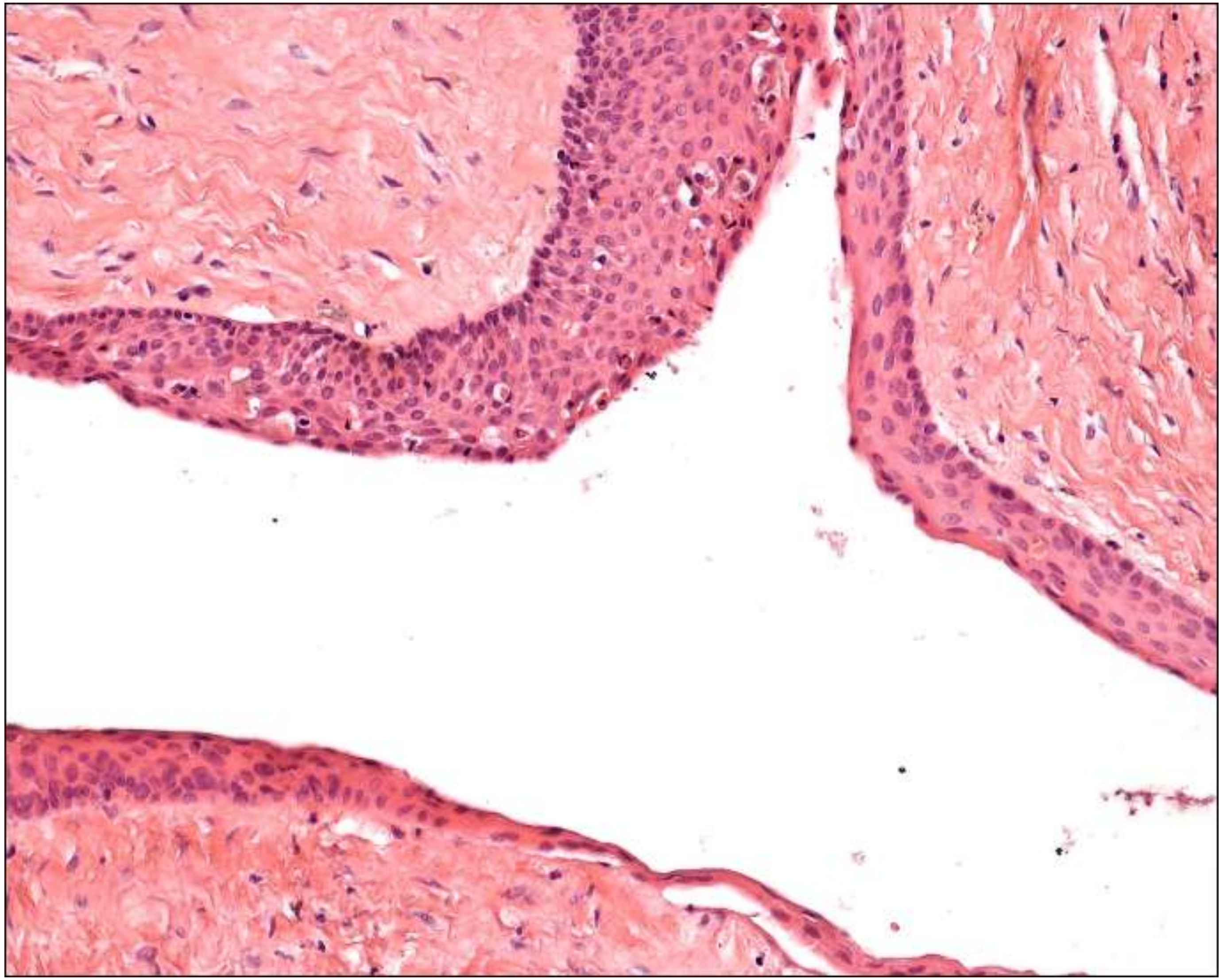

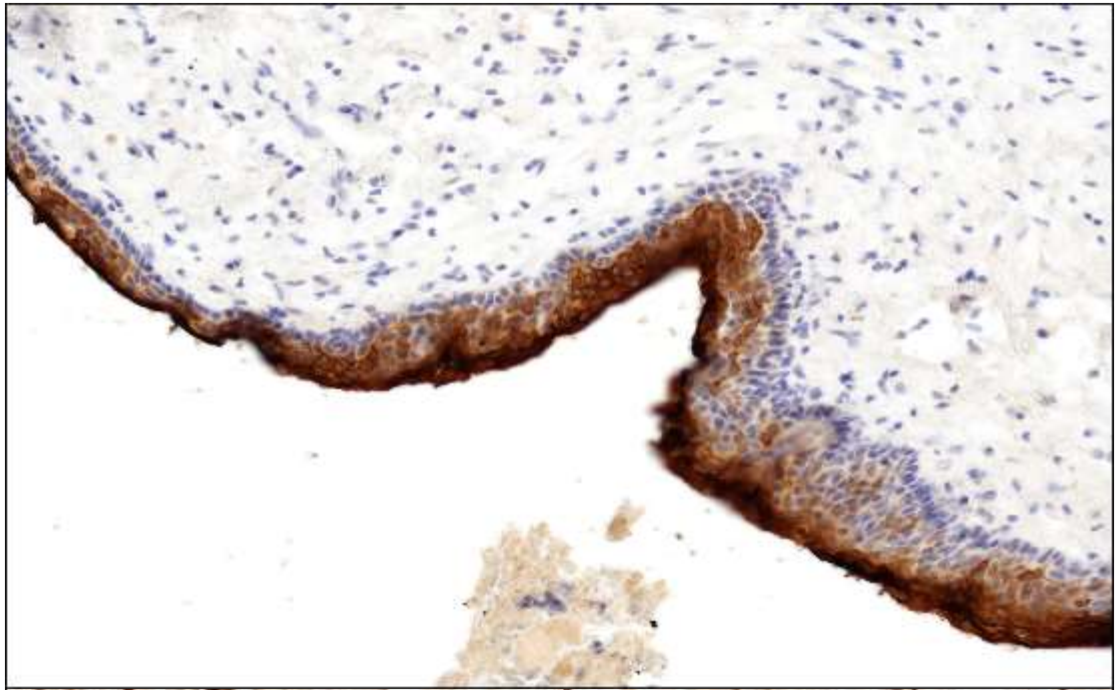

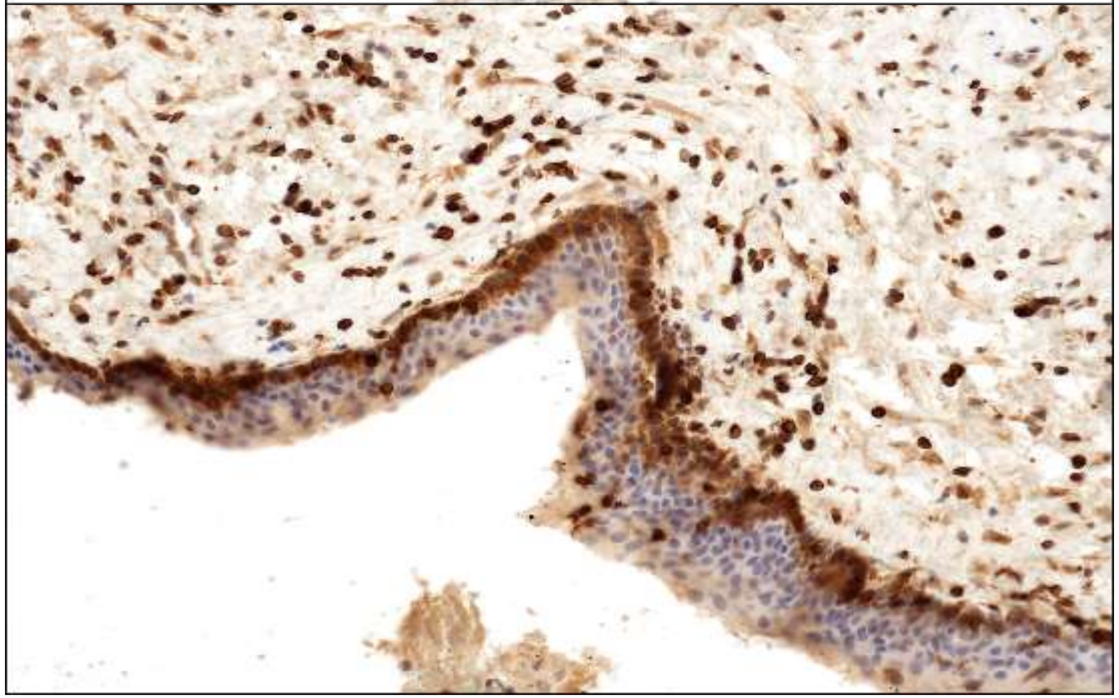

Case S27 – Conclusion

- H&E: thin (focally thicker) non-keratinizing squamous epithelium with focal wavy surface and basal palisading, even epithelial stromal interface
- Inflammation absent
- Bcl2: basal positivity
- CK17: dominantly superficial (+/- spinous), focal transepithelial positivity
- Histopathological diagnosis: apical or lateral radicular cyst
- Combined clinical and pathological opinion: cannot be given because of lacking clinical details; developmental or mature inflammatory cyst N.O.S.

Case S28-31

- 11-year-old girl with verified Gorlin-Goltz syndrome and mental retardation
- Dental hygiene/status: Status not observable due to lack of cooperation.
- CT: cystic lesion in all quadrants (namely around teeth 44, 45, 37, 38, 23, 24, 13, 18).
- Operation: removal of cysts and teeth 44, 45, 37, 38, 23, 24, 13, 18
- Clinical diagnosis: odontogenic cyst? radicular cyst?

S28 – CT coronal section (right mandibular cyst)

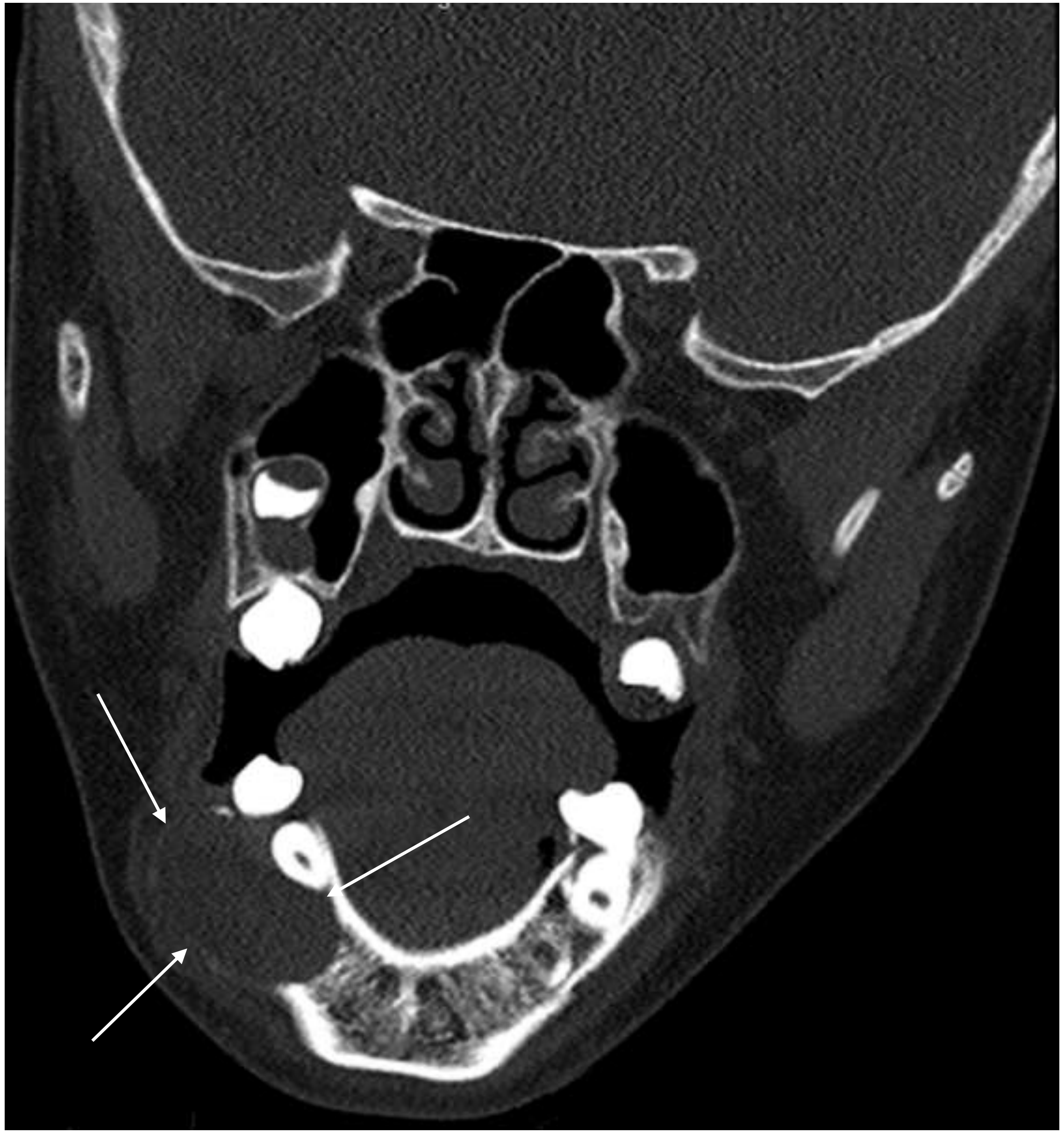

S28 – HE, CK17, bcl2 (x20)

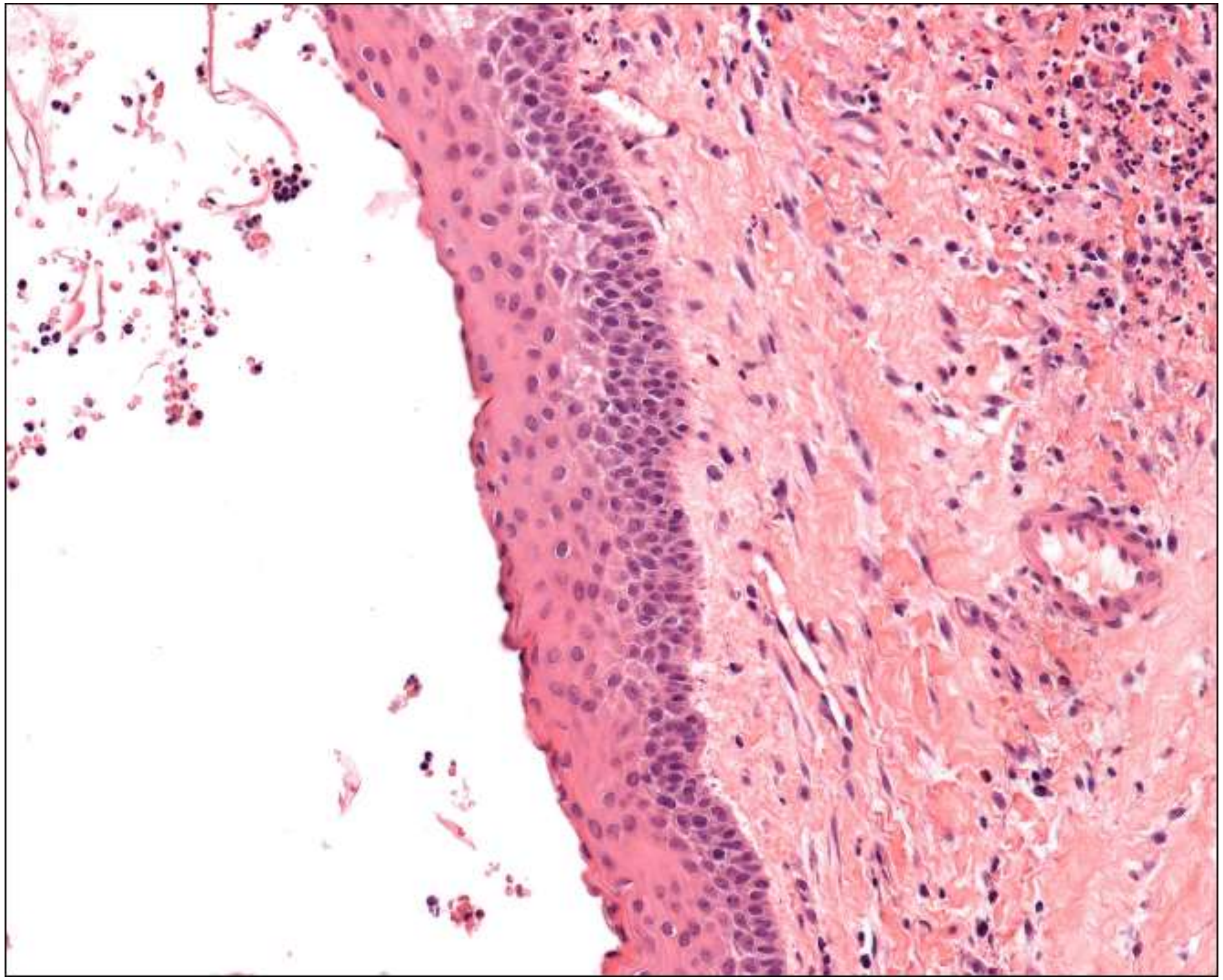
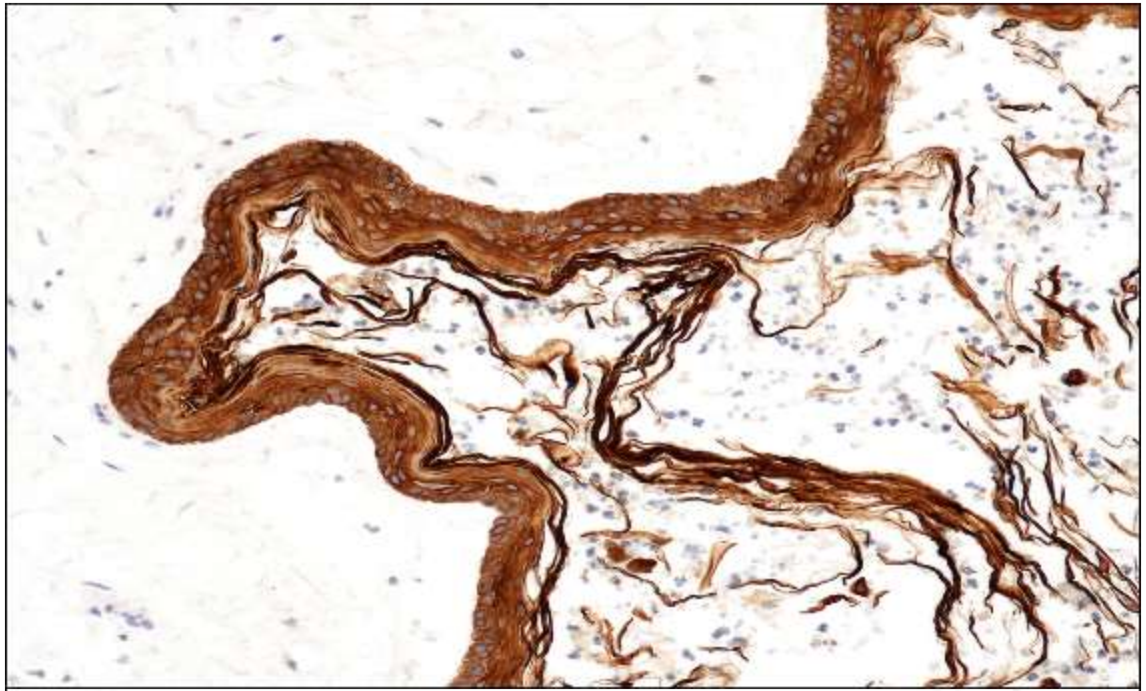
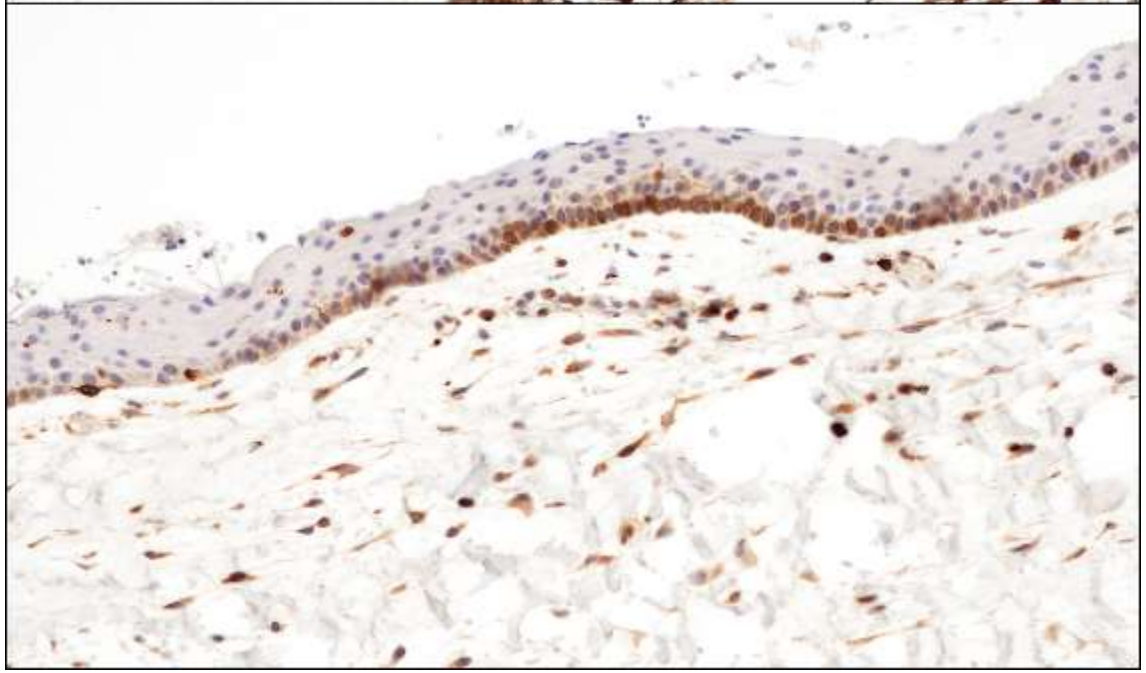

S28 – HE, CK17, bcl2 (x20)

Inflamed area lacking both CK17 and bcl2 stainings

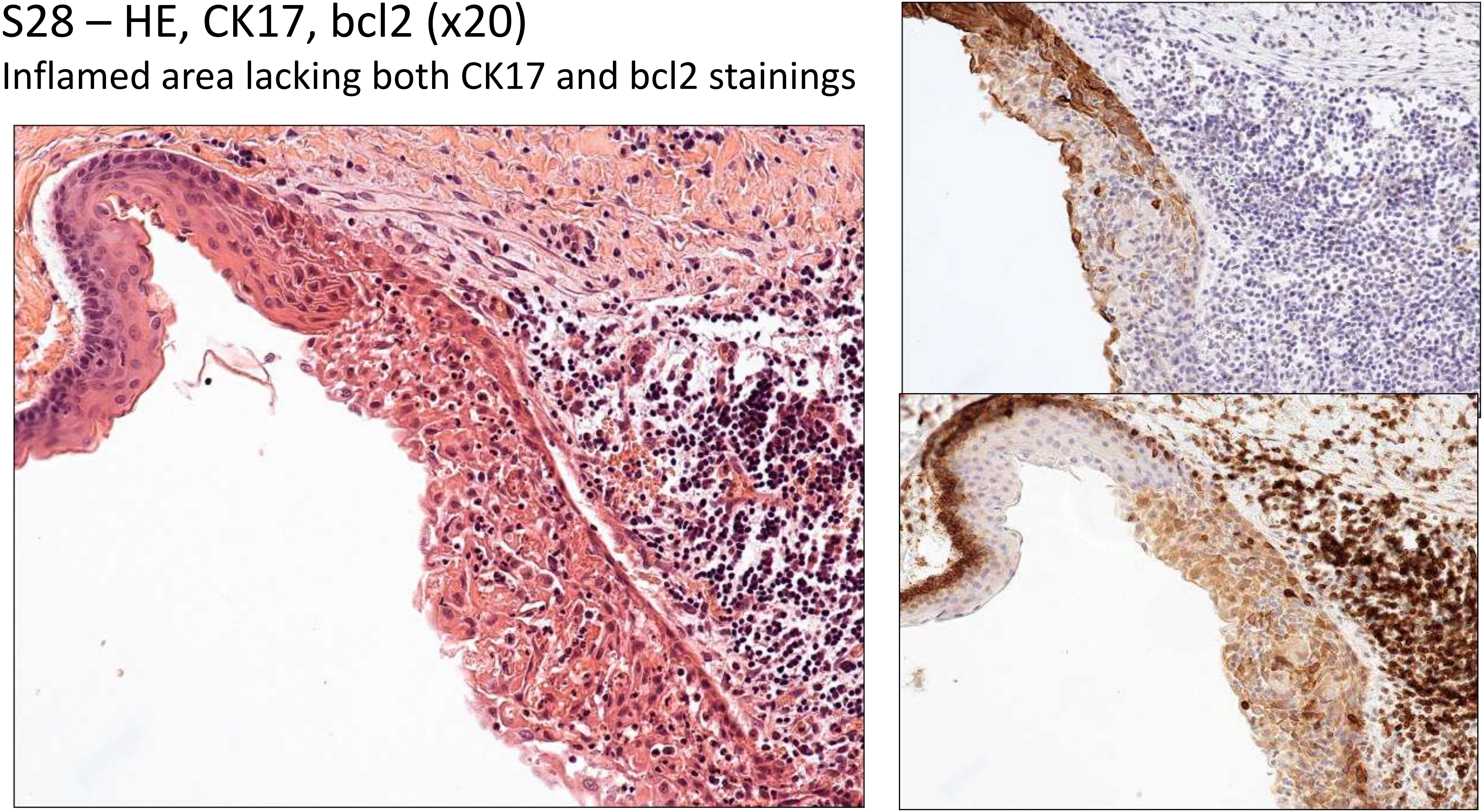

Case S28 – Conclusion

- H&E: thin parakeratotic epithelium with wavy surface and basal palisading, even epithelial stromal interface; non-keratinizing squamous epithelium associated with inflammation
- Marked mixed inflammatory infiltrate present in some areas
- Bcl2: basal, suprabasal positivity; lack of staining in the non-specific epithelium
- CK17: transepithelial positivity; mosaic like weaker transepithelial or superficial staining in the altered epithelium
- Histopathological diagnosis: OKC
- Combined clinical and pathological opinion: OKC (associated with the Gorlin-Goltz syndrome)

S29 – CT coronal section (left mandibular cyst)

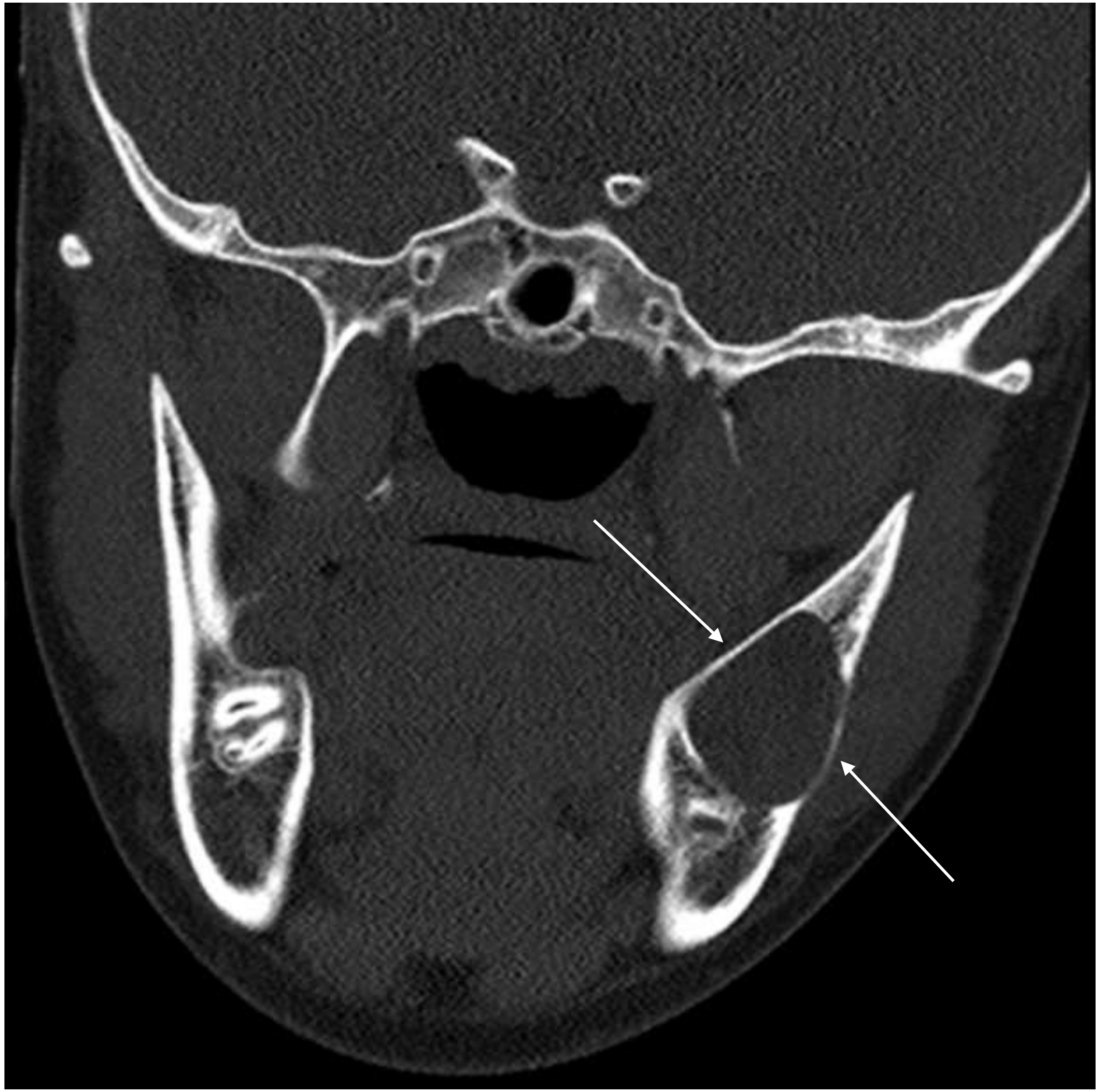

S29 – HE, CK17, bcl2 (x20)

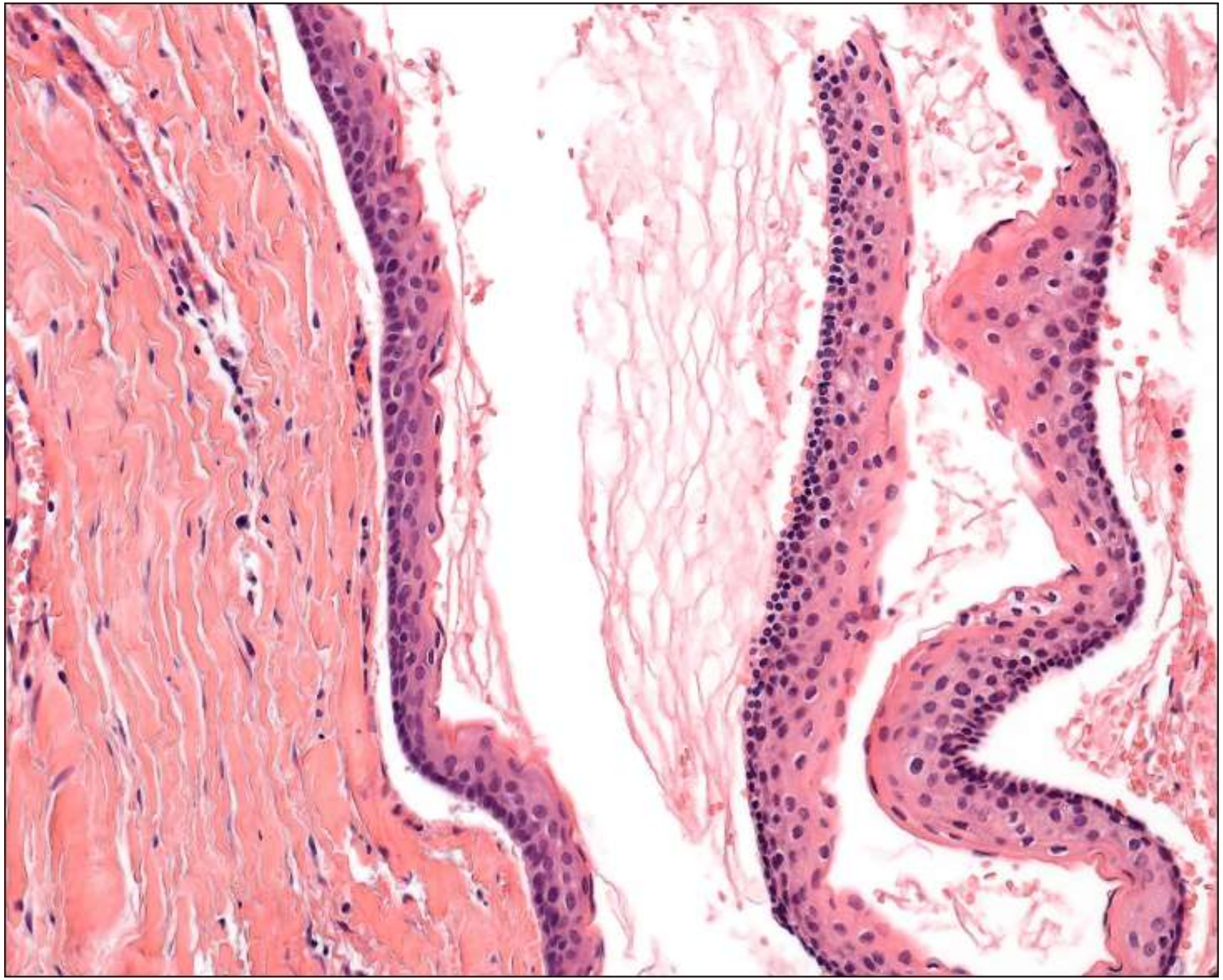

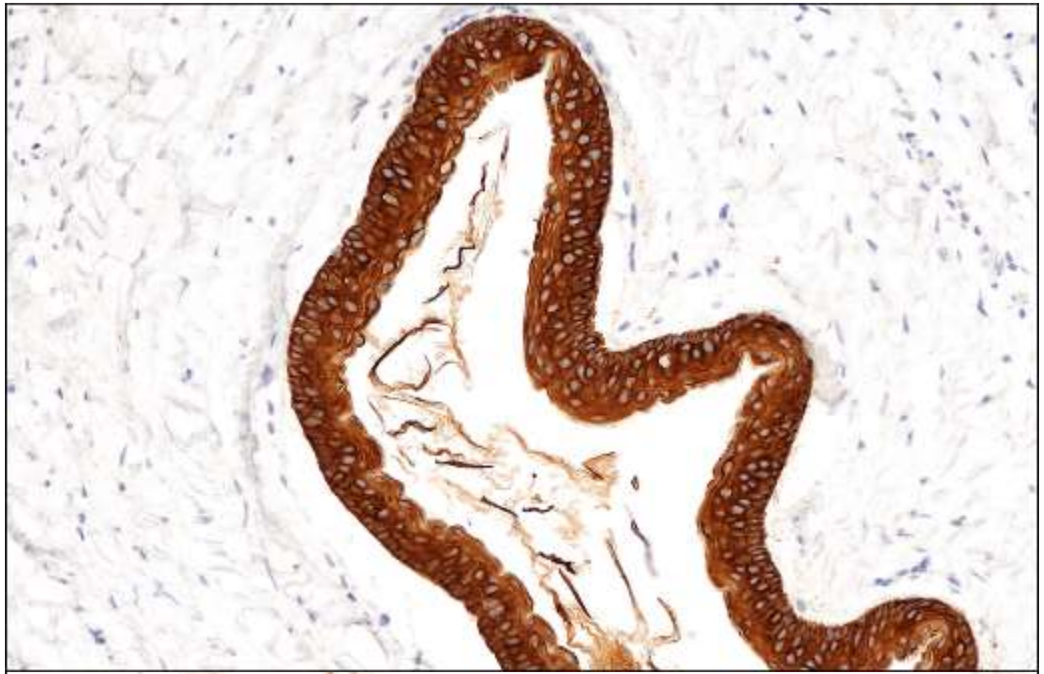

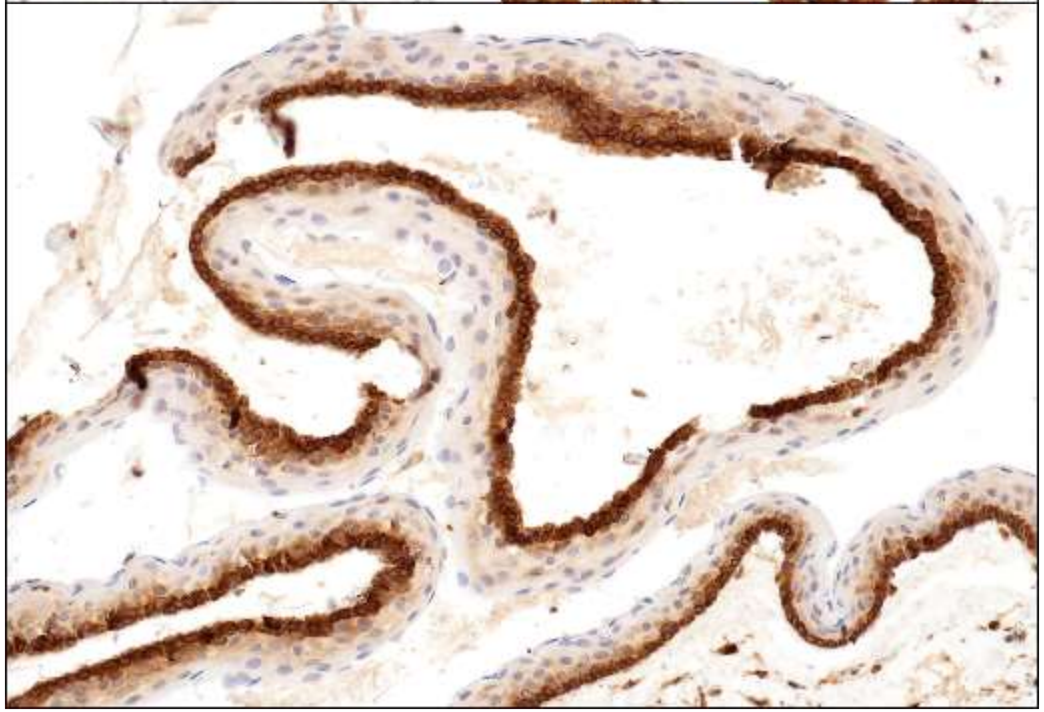

Case S29 – Conclusion

- H&E: thin parakeratotic epithelium with wavy surface and basal palisading, even epithelial stromal interface
- Inflammation absent
- Bcl2: basal, suprabasal positivity
- CK17: dominantly transepithelial, focal surface positivity
- Histopathological diagnosis: OKC
- Combined clinical and pathological opinion: OKC (associated with the Gorlin-Goltz syndrome)

S30 – CT coronal section (left maxillary cyst)

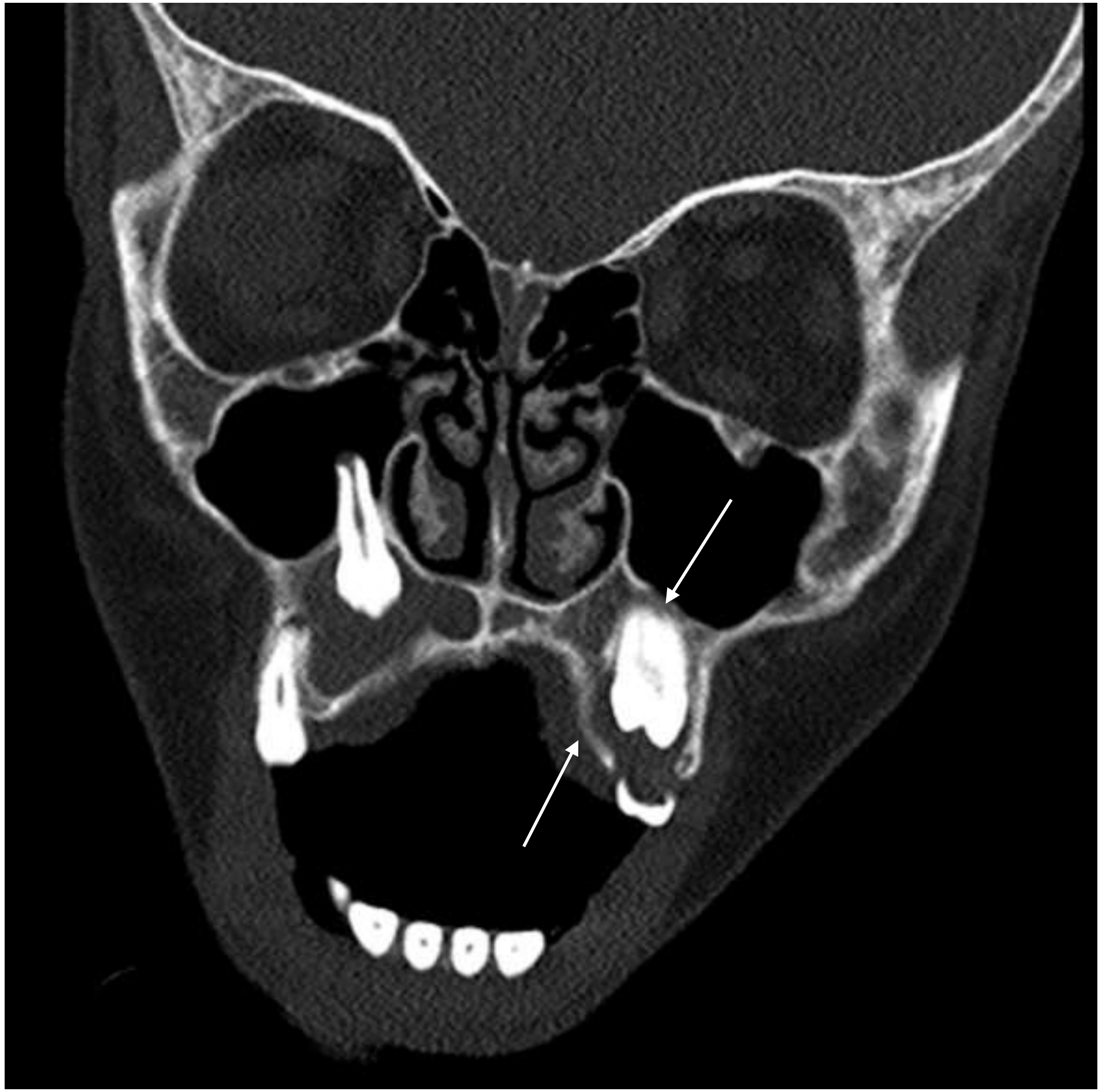

S30 – HE, CK17, bcl2 (x20)

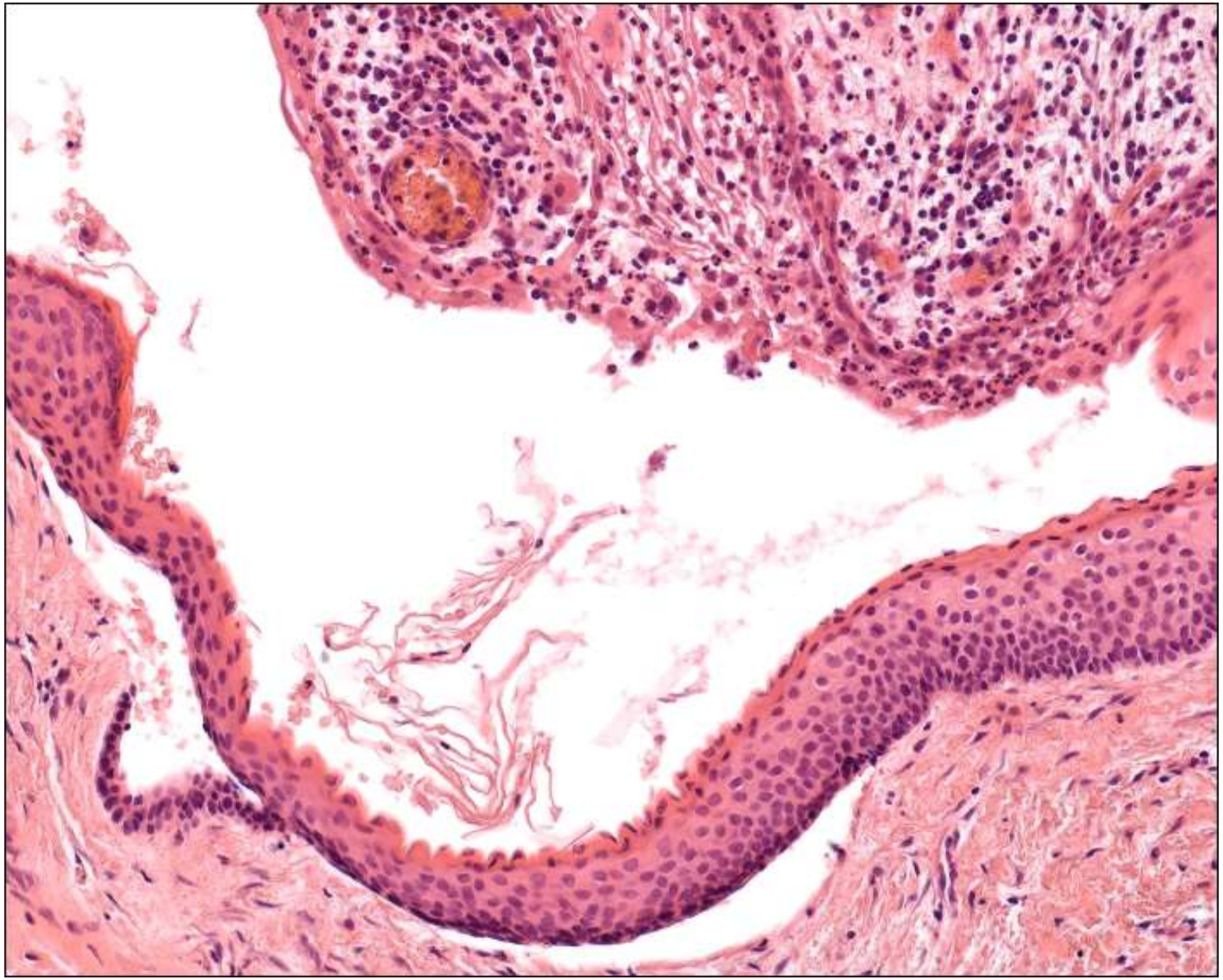

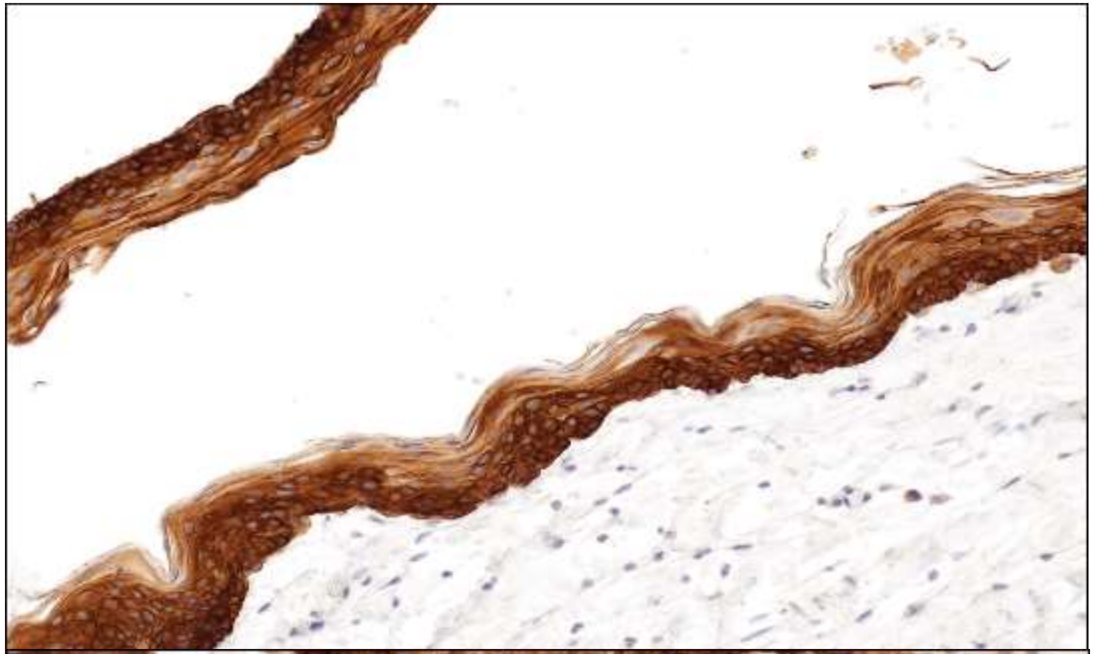

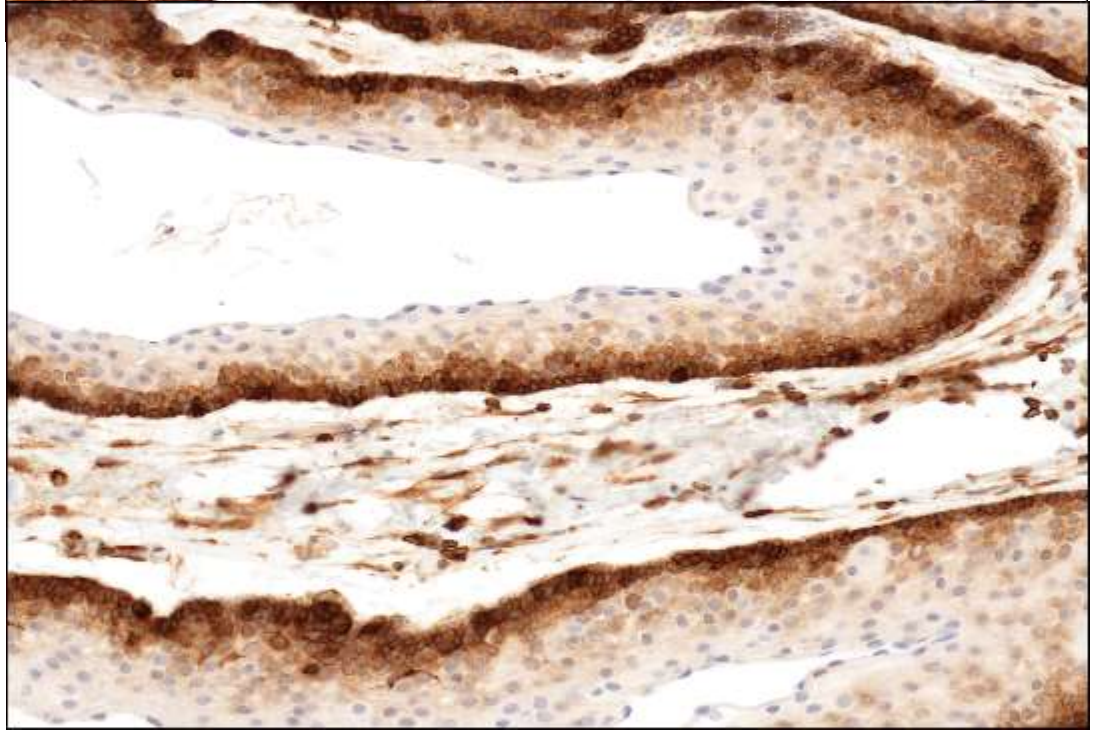

S30 – HE, CK17, bcl2 (x20)

Inflamed area lacking both CK17 & basal bcl2 stainings; moderate spinous bcl2 positivity

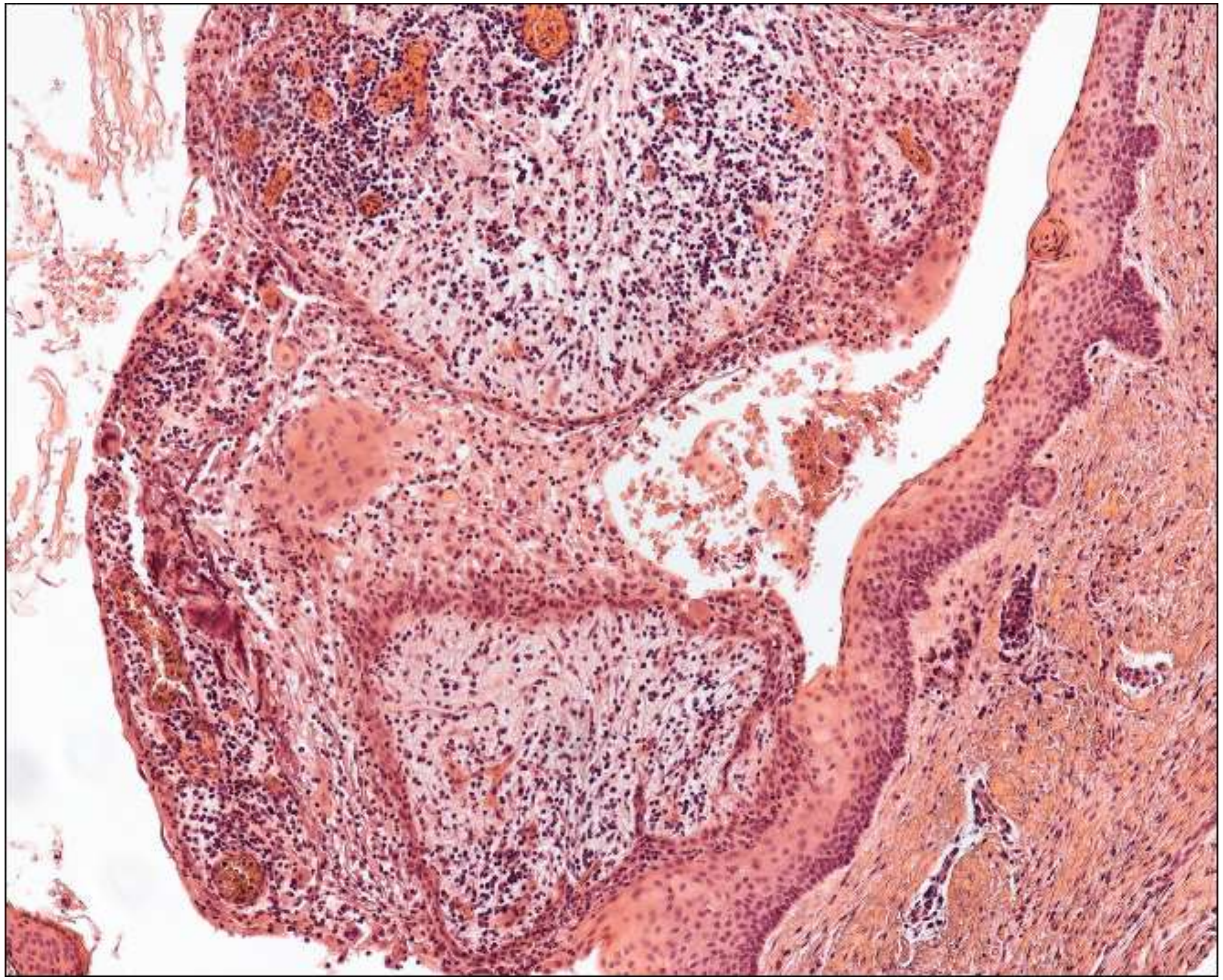

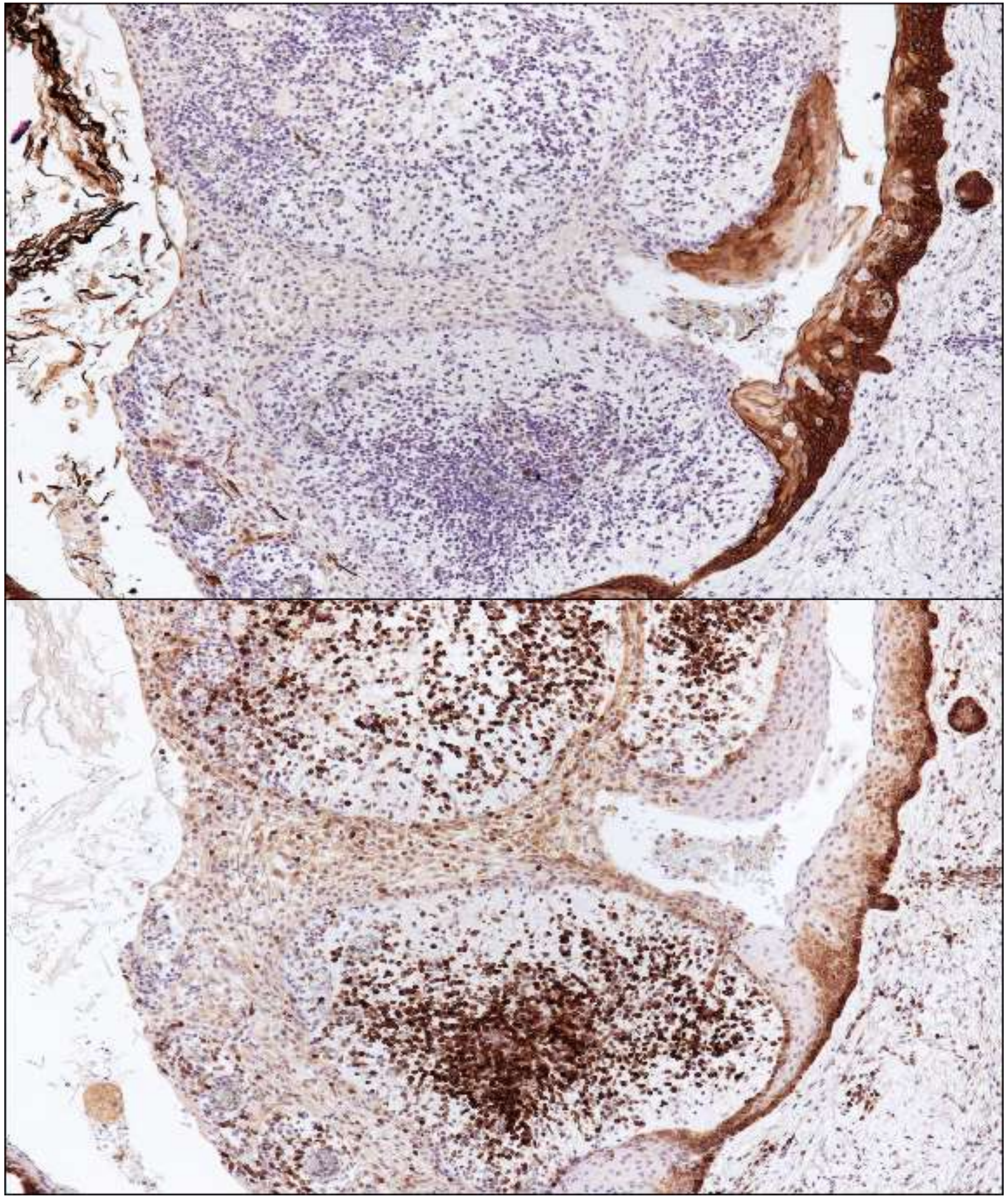

Case S30 – Conclusion

- H&E: thin parakeratotic epithelium with wavy surface and basal palisading; non-keratinizing squamous epithelium without basal palisading at site of inflammation
- Inflammation focally present, dominantly chronic with neutrophils
- Bcl2: basal, suprabasal positivity (lost in altered epithelium associated with inflammation)
- CK17: transepithelial positivity (focal negativity or weak spinous staining in the altered epithelium associated with inflammation)
- Histopathological diagnosis: OKC
- Combined clinical and pathological opinion: OKC (associated with the Gorlin-Goltz syndrome)

S31 – CT coronal section (right maxillary cyst)

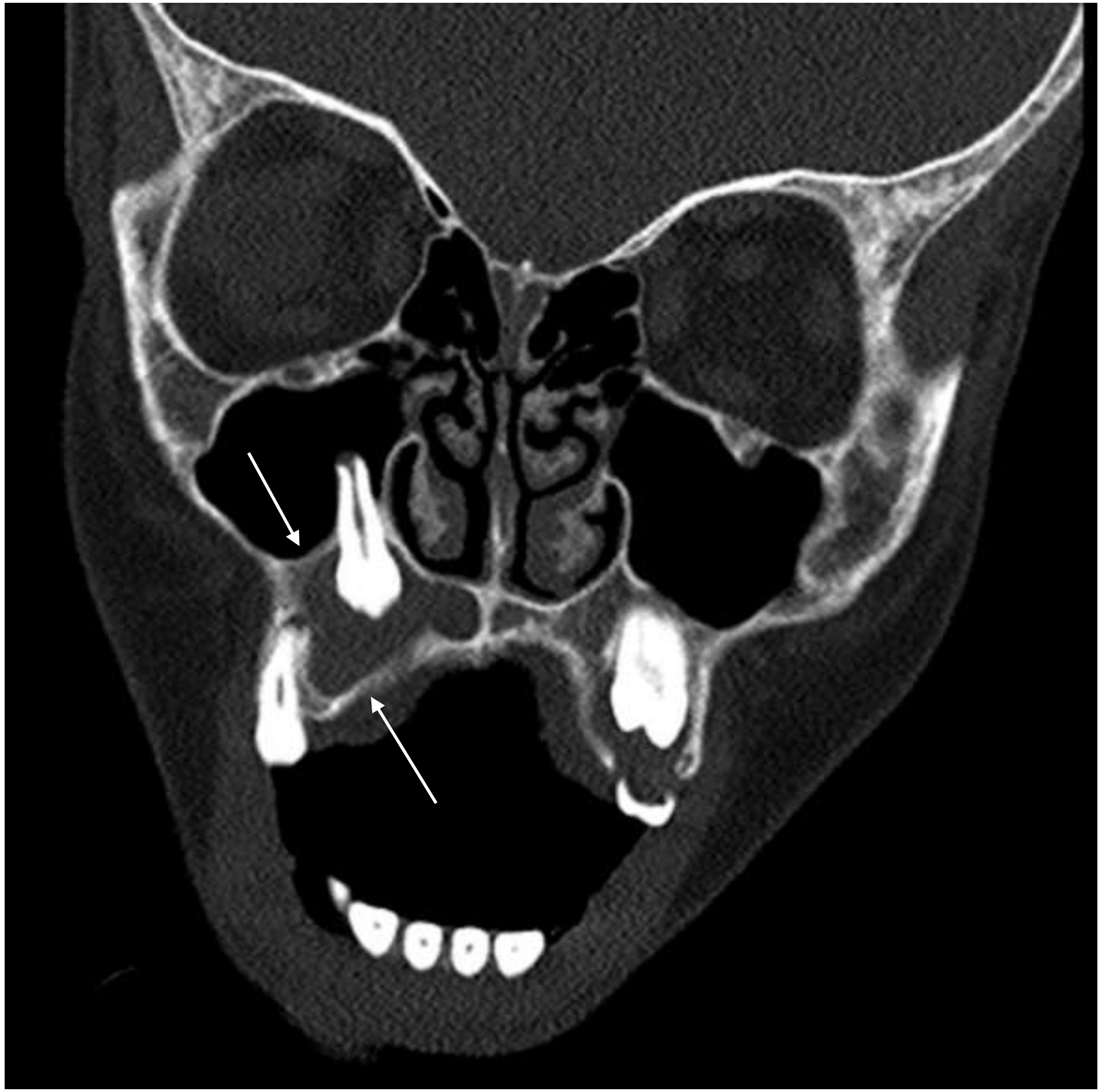

S31 HE, CK17, bcl2 (x22)

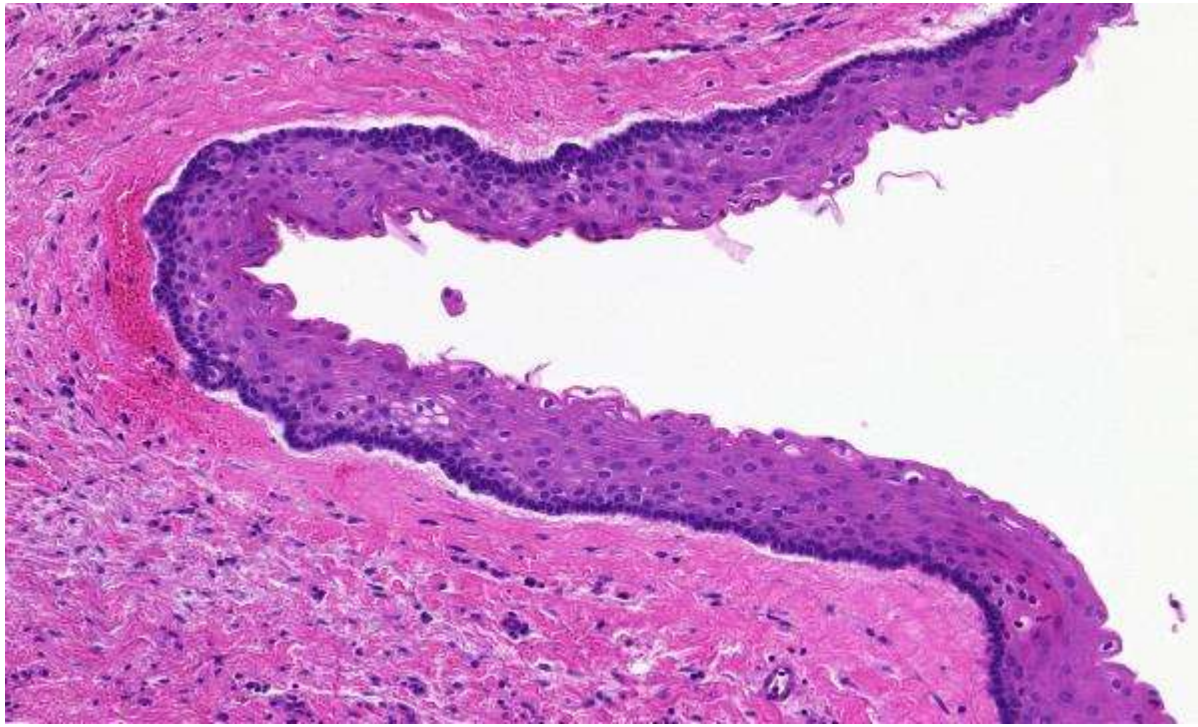

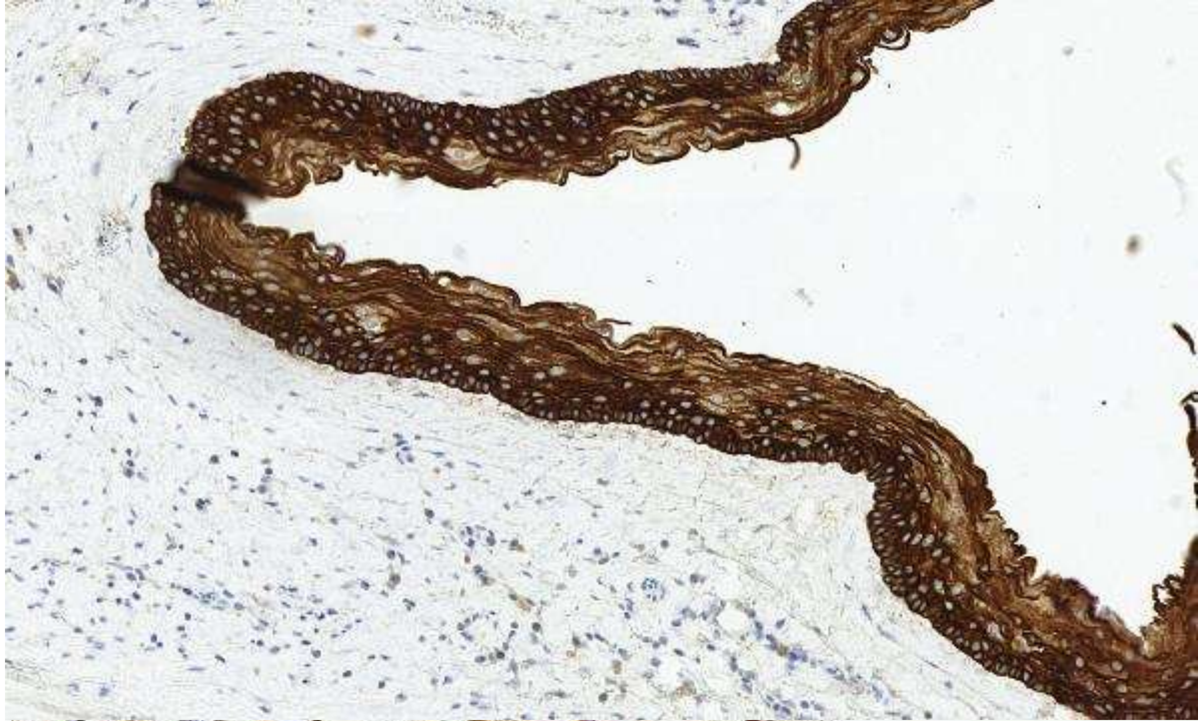

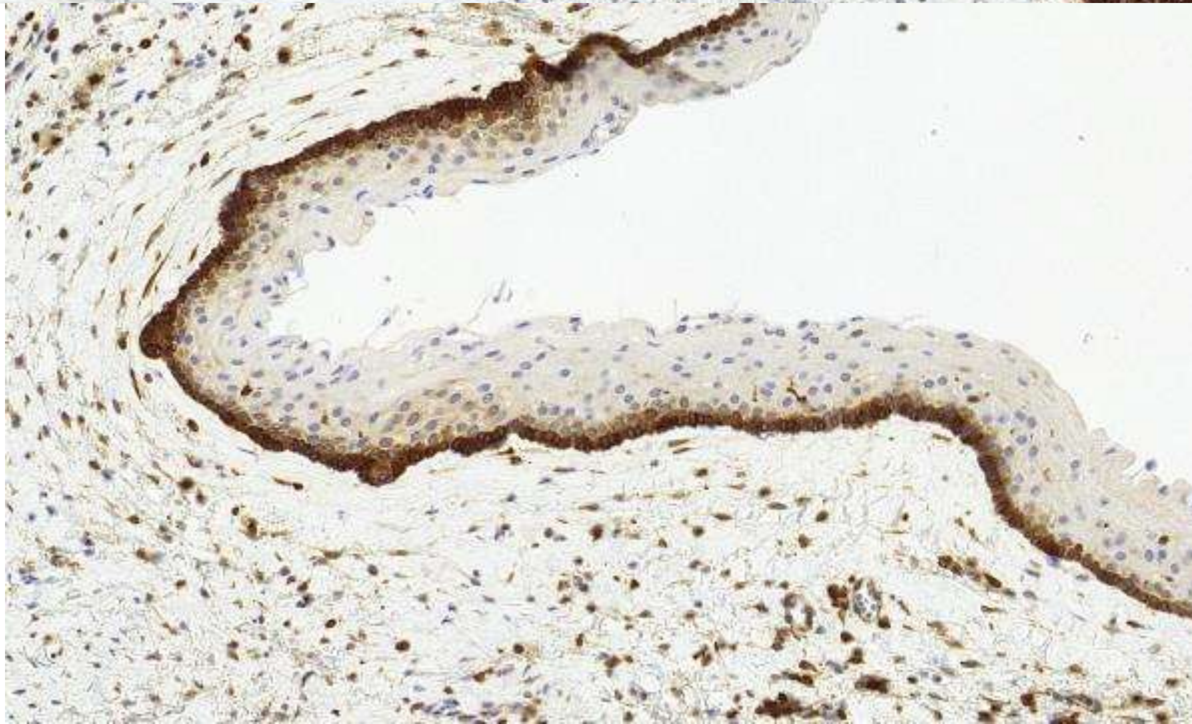

S31 - CK17, bcl2 (x30)

Different patterns of CK17 staining: transepithelial, basal, patchy (transepithelial) (with maintained basal (suprabasal) bcl2 positivity

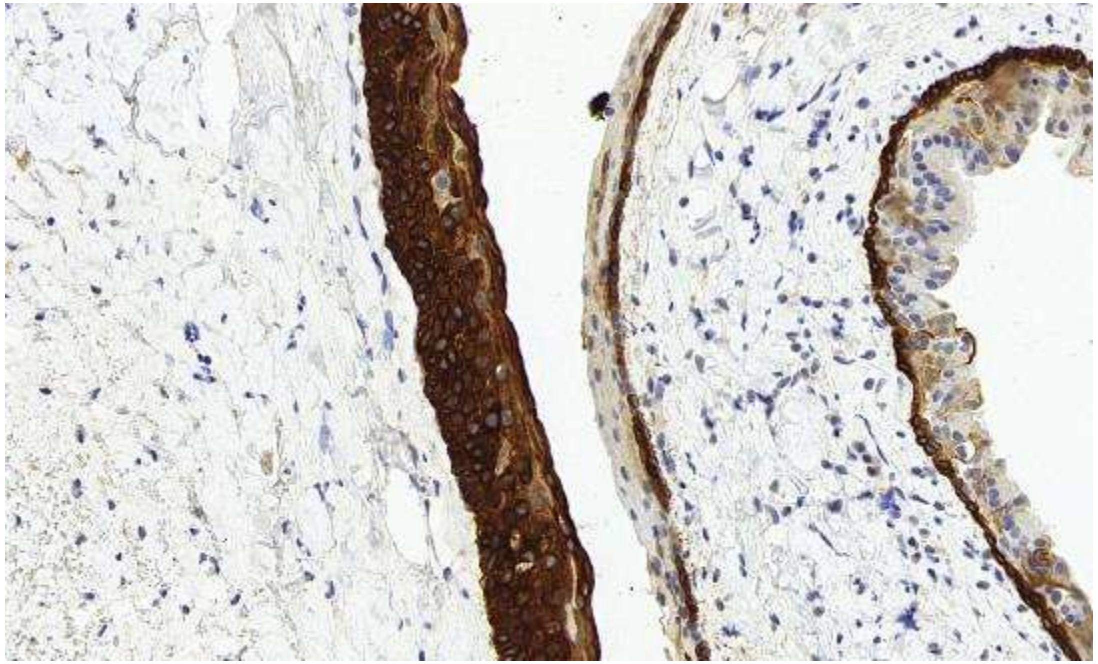

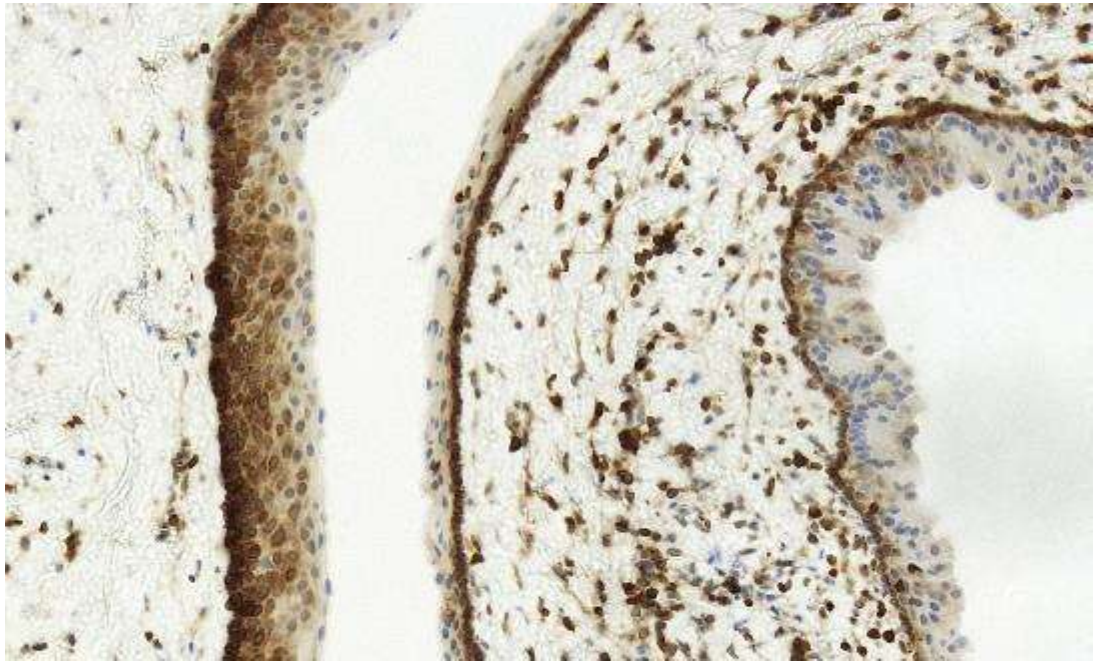

S31 – HE, CK17, bcl2 (x20)

Inflamed area with loss of transepithelial CK17 staining

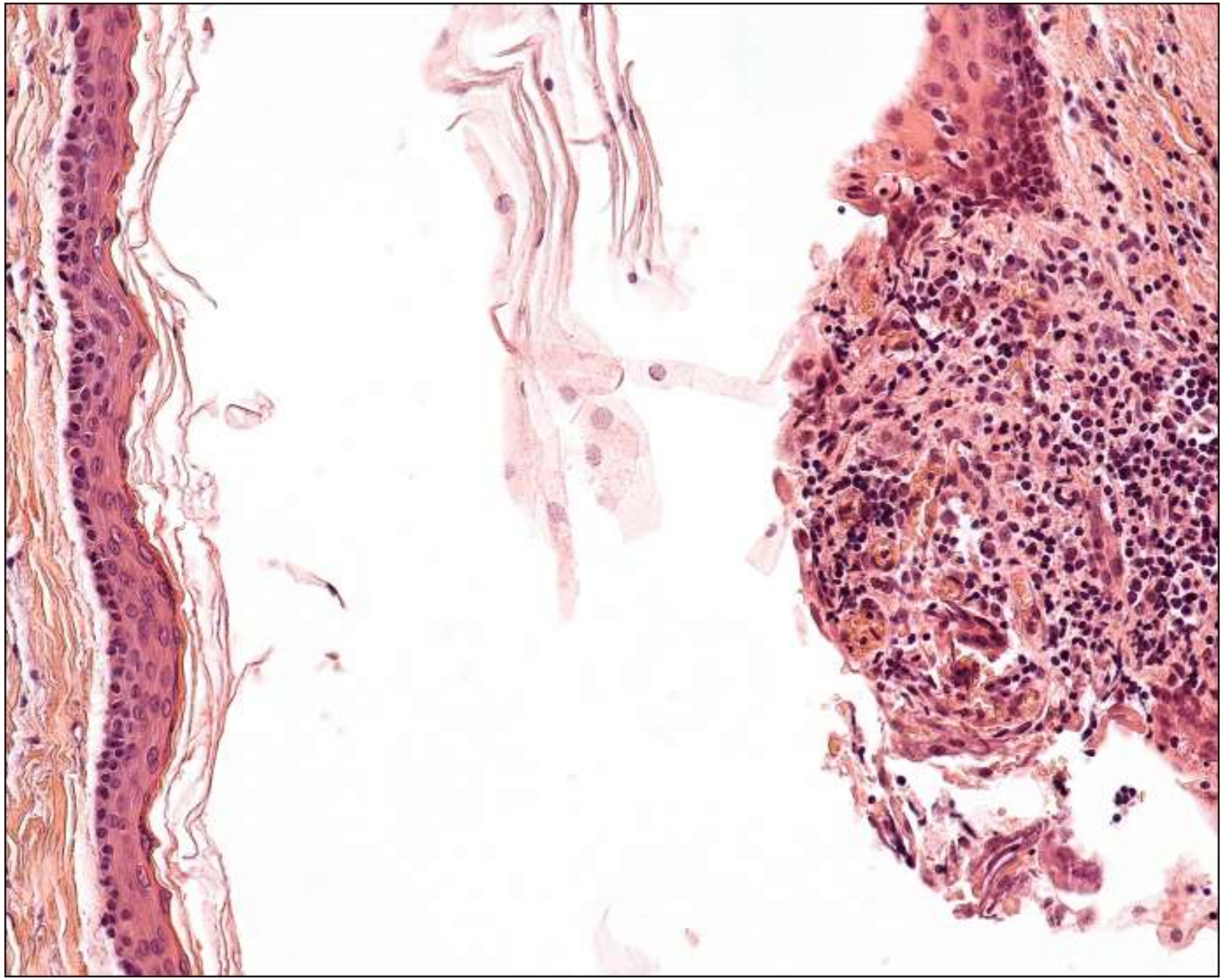

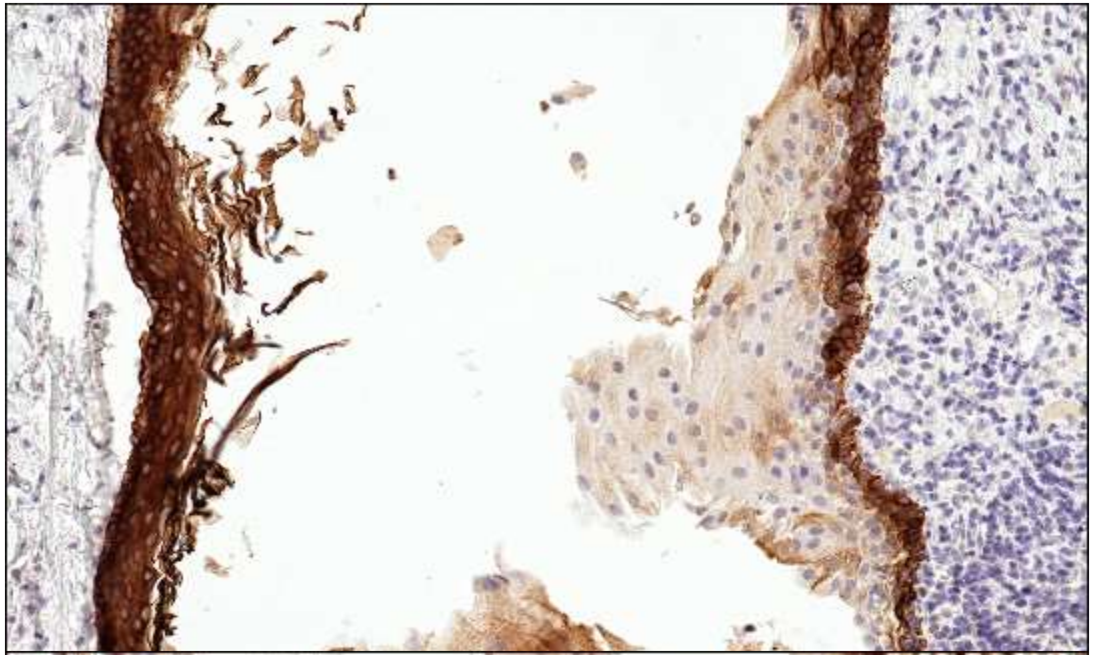

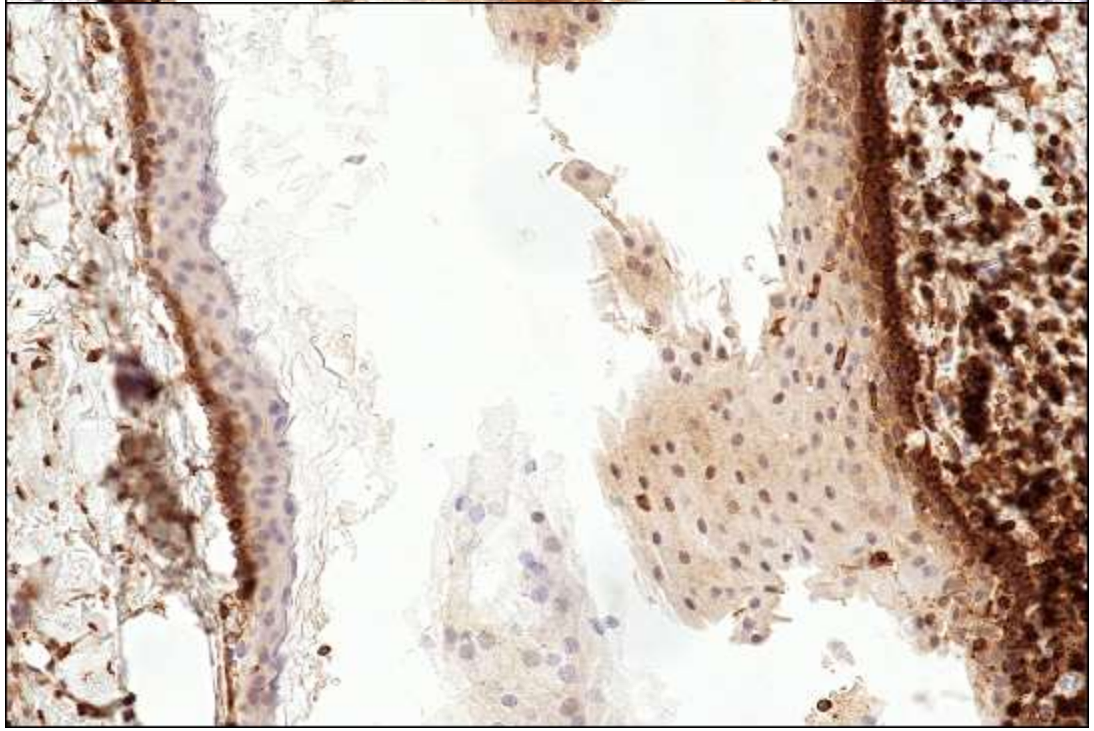

Case S31 – Conclusion

- H&E: thin parakeratotic epithelium with wavy surface and basal palisading, even epithelial stromal interface; thicker non parakeratinizing epithelium at the focus of inflammation
- Inflammation dominantly absent, small focus of mixed inflammatory infiltrate
- Bcl2: basal, suprabasal positivity (also maintained at the site of inflammation associated altered epithelium)
- CK17: transepithelial; areas of more patchy staining (focal basal positivity at the site of inflammation associated altered epithelium)
- Histopathological diagnosis: OKC
- Combined clinical and pathological opinion: OKC (associated with the Gorlin-Goltz syndrome)

Case S32

- 53-year-old male
- Dental hygiene/status: partly maintained, partly missing, treated teeth. Teeth 21, 22 were treated with root filling.
- CBCT: radiolucent, cystic lesion around teeth 21 and 22 (23x17 mm).
- Operation: removal of cyst and teeth 21, 22
- Clinical diagnosis: odontogenic cyst, keratocyst?

S32 – OPT

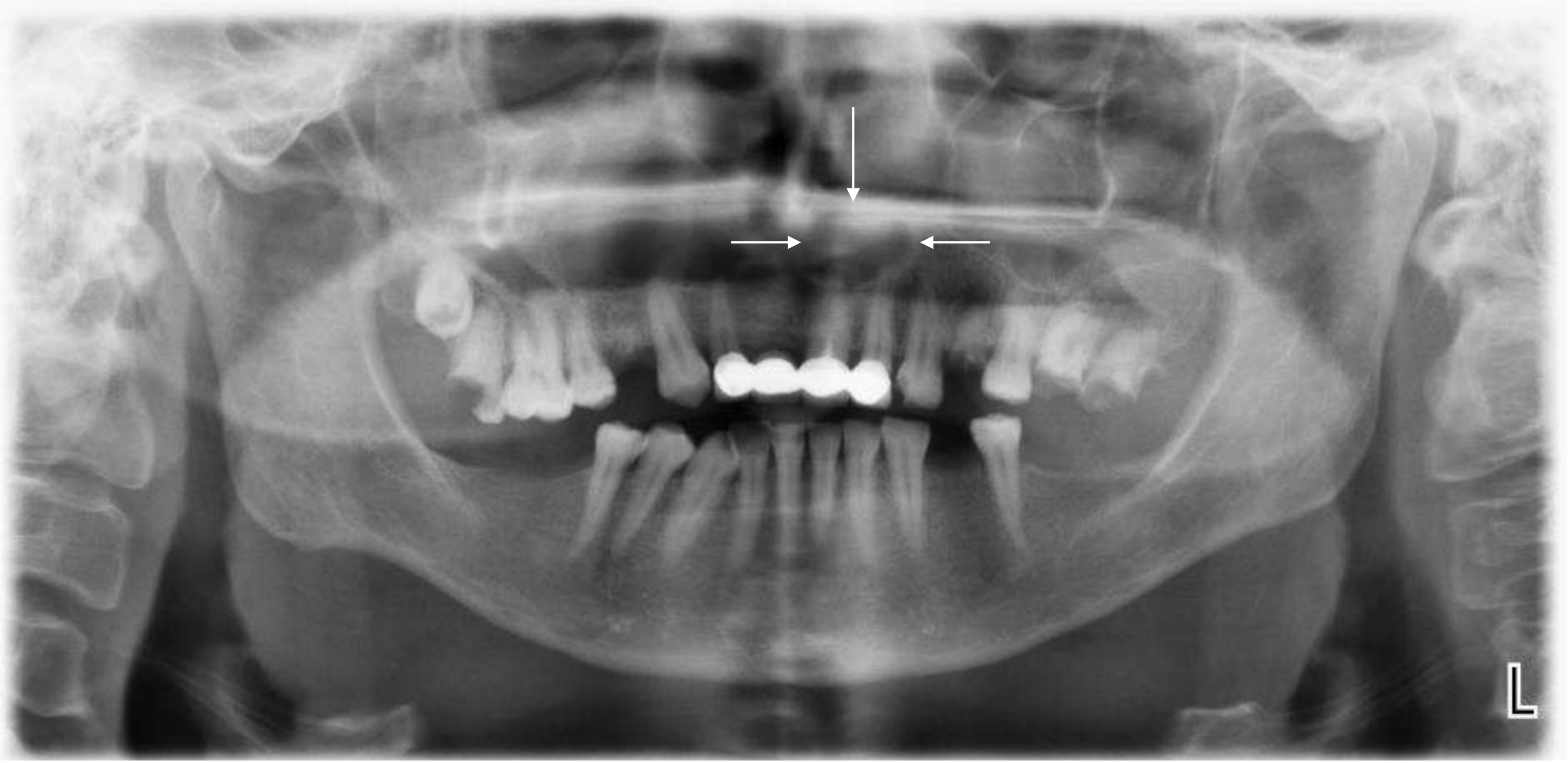

S32 – HE, CK17, bcl2 (x20)

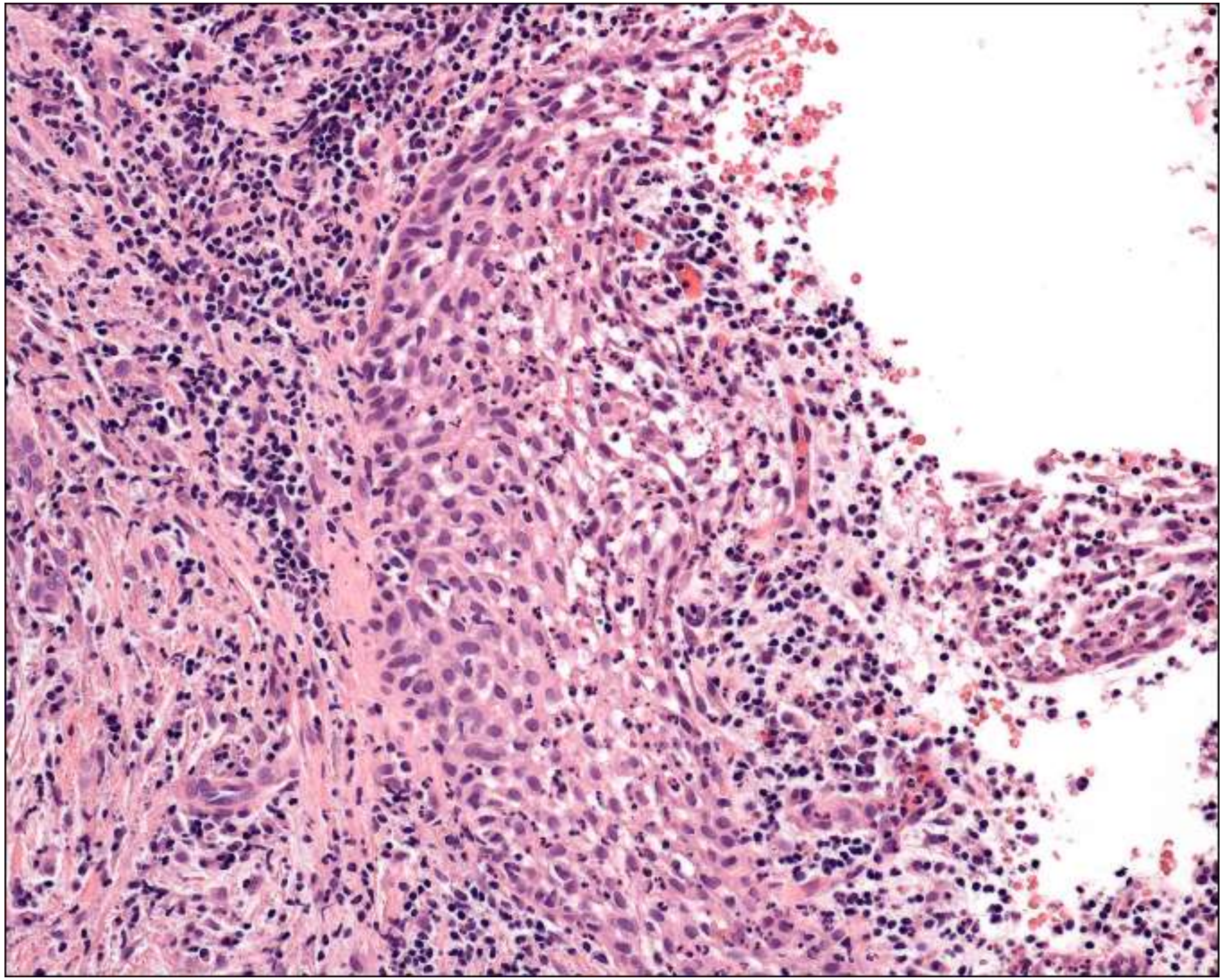

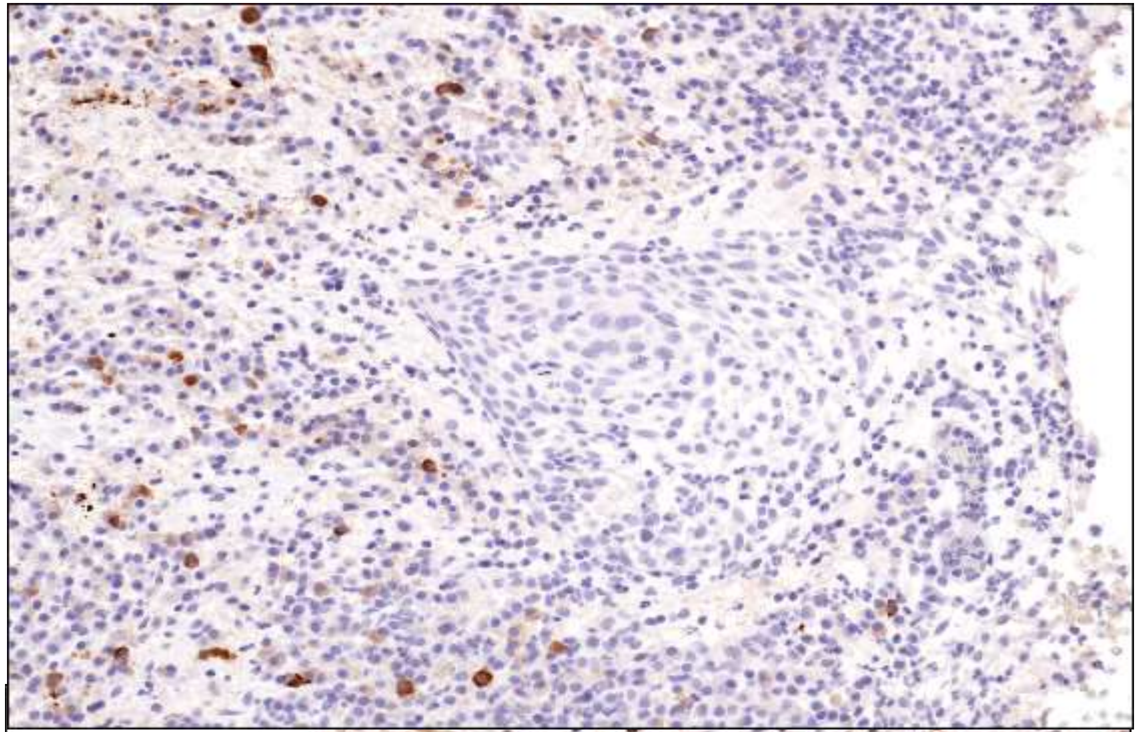

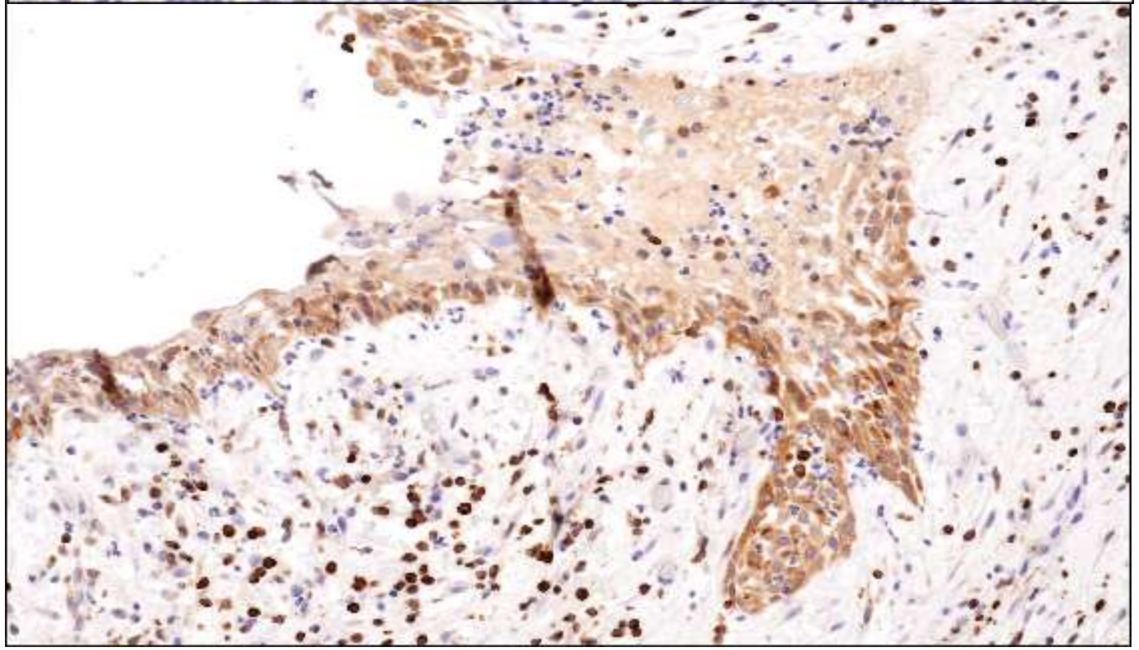

S32 – CK17 (x20)

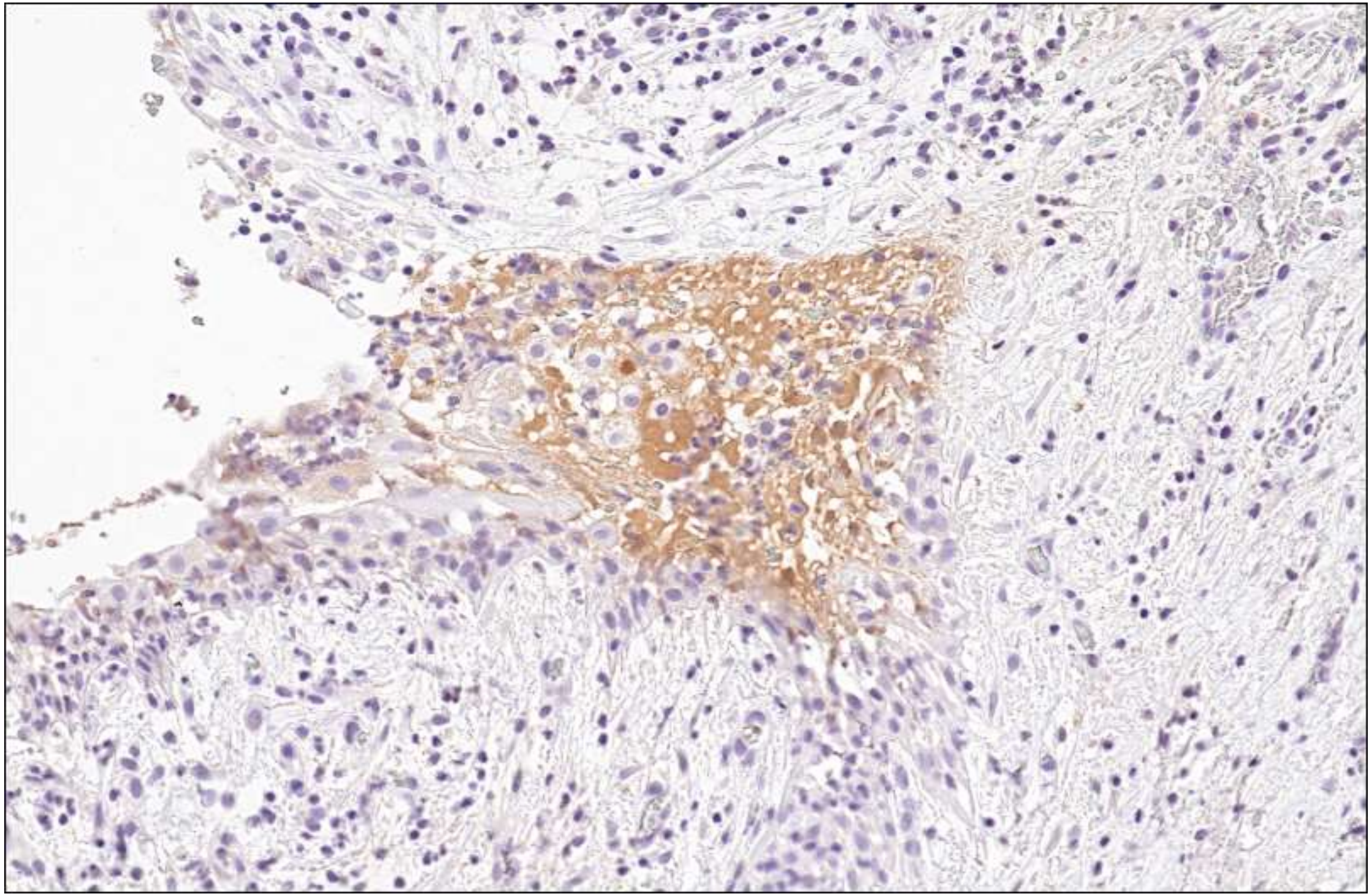

Case S32 – Conclusion

- H&E: relatively thick squamous epithelium with spongiosis without parakeratosis, basal palisading or wavy surface; even to uneven stromal epithelial interface.
- Prominent chronic inflammation is present with acute component.
- Bcl2: transepithelial positivity
- CK17: dominantly negative, focal transepithelial positivity
- Histopathological diagnosis: radicular cyst
- Combined clinical and pathological opinion: radicular cyst

Case S34

- 47-year-old female
- Dental hygiene/status: conserved, treated teeth. Teeth 21, 22, 23 are not vital.
- CBCT: radiolucent cystic area around teeth 21-23
- Operation: removal of the cyst and root of tooth 21 plus tooth 22
- Clinical diagnosis: odontogenic cyst, keratocyst?

S34 – OPT

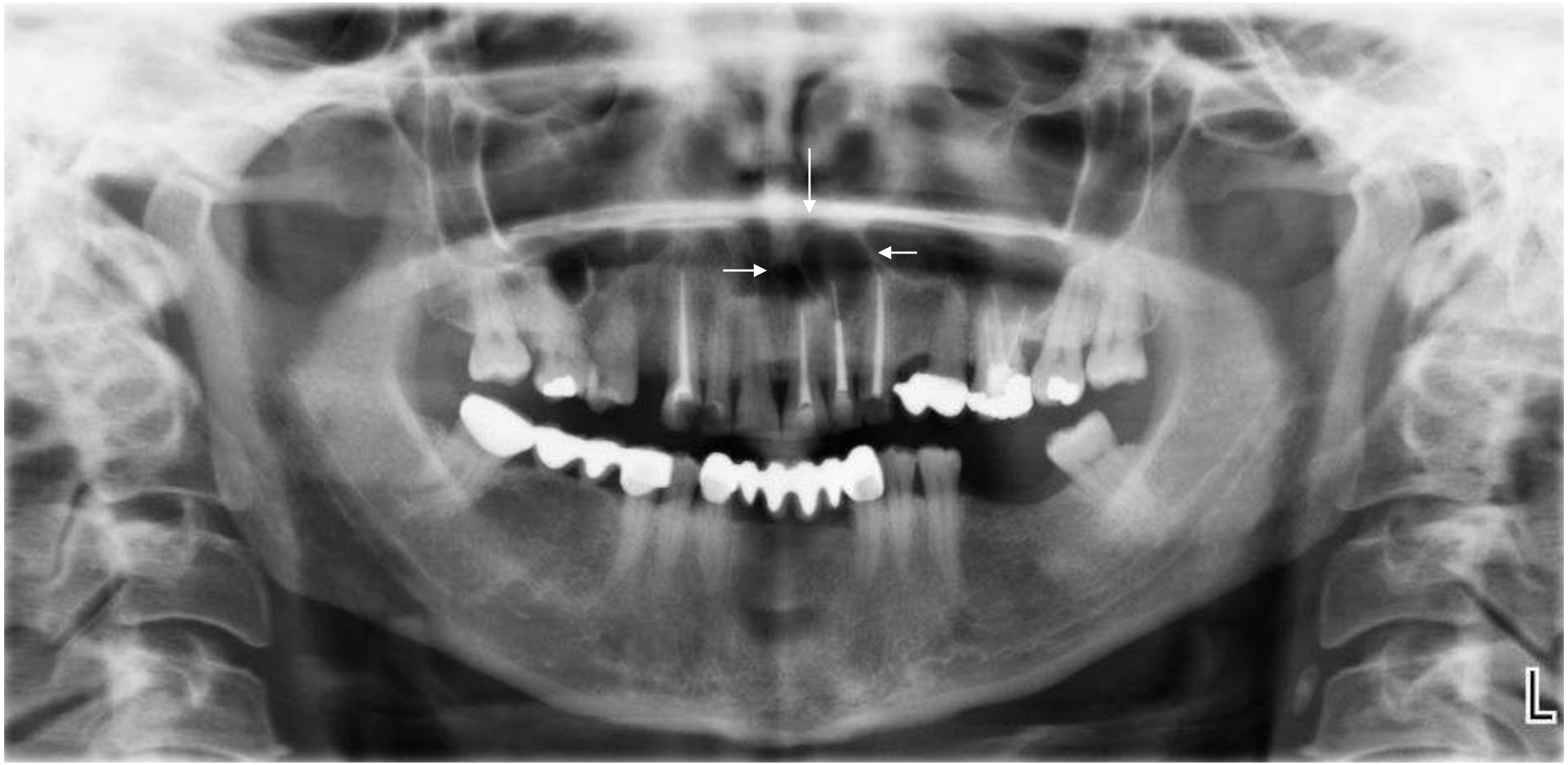

S34 - CBCT

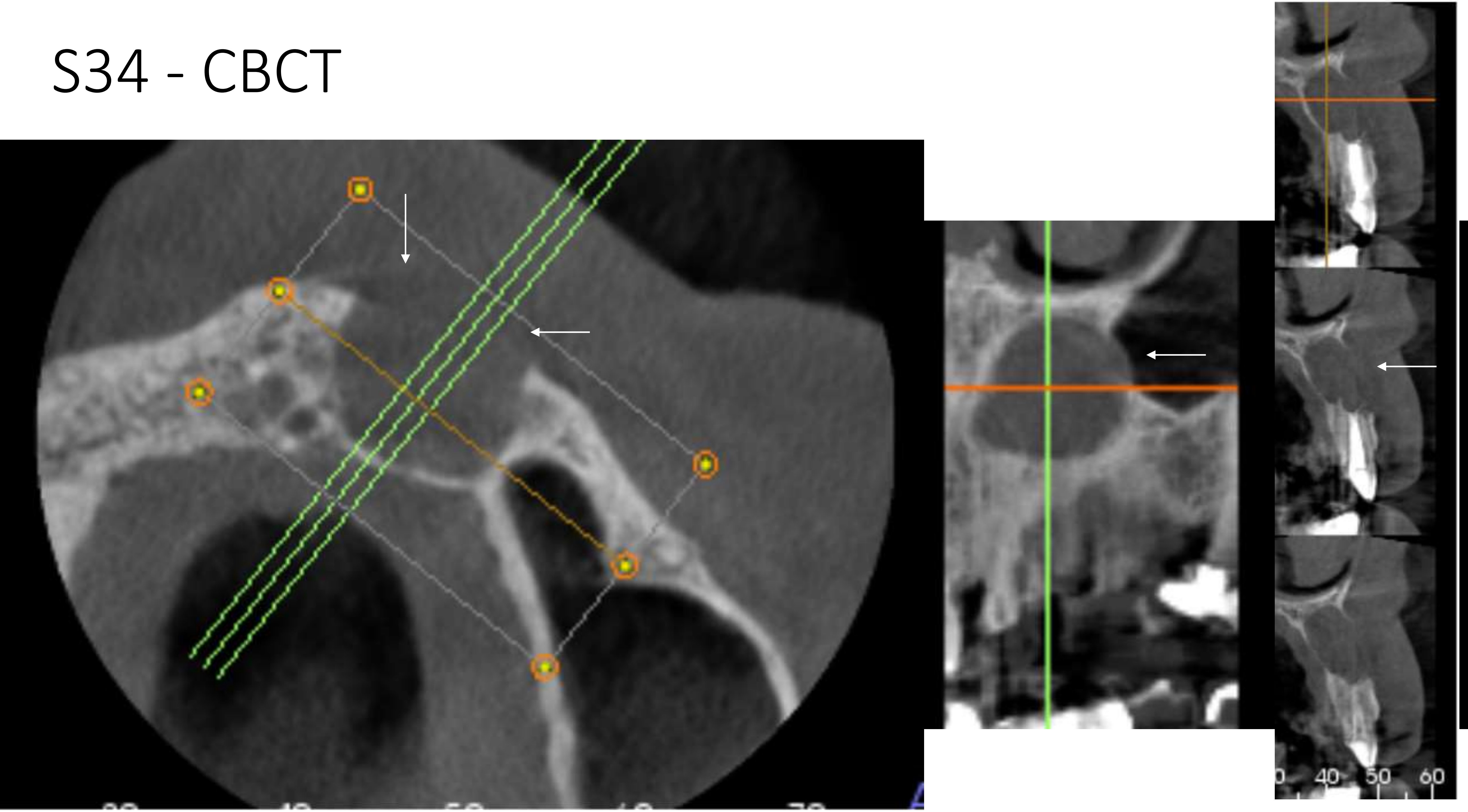

S34 – HE, CK17, bcl2 (x20)

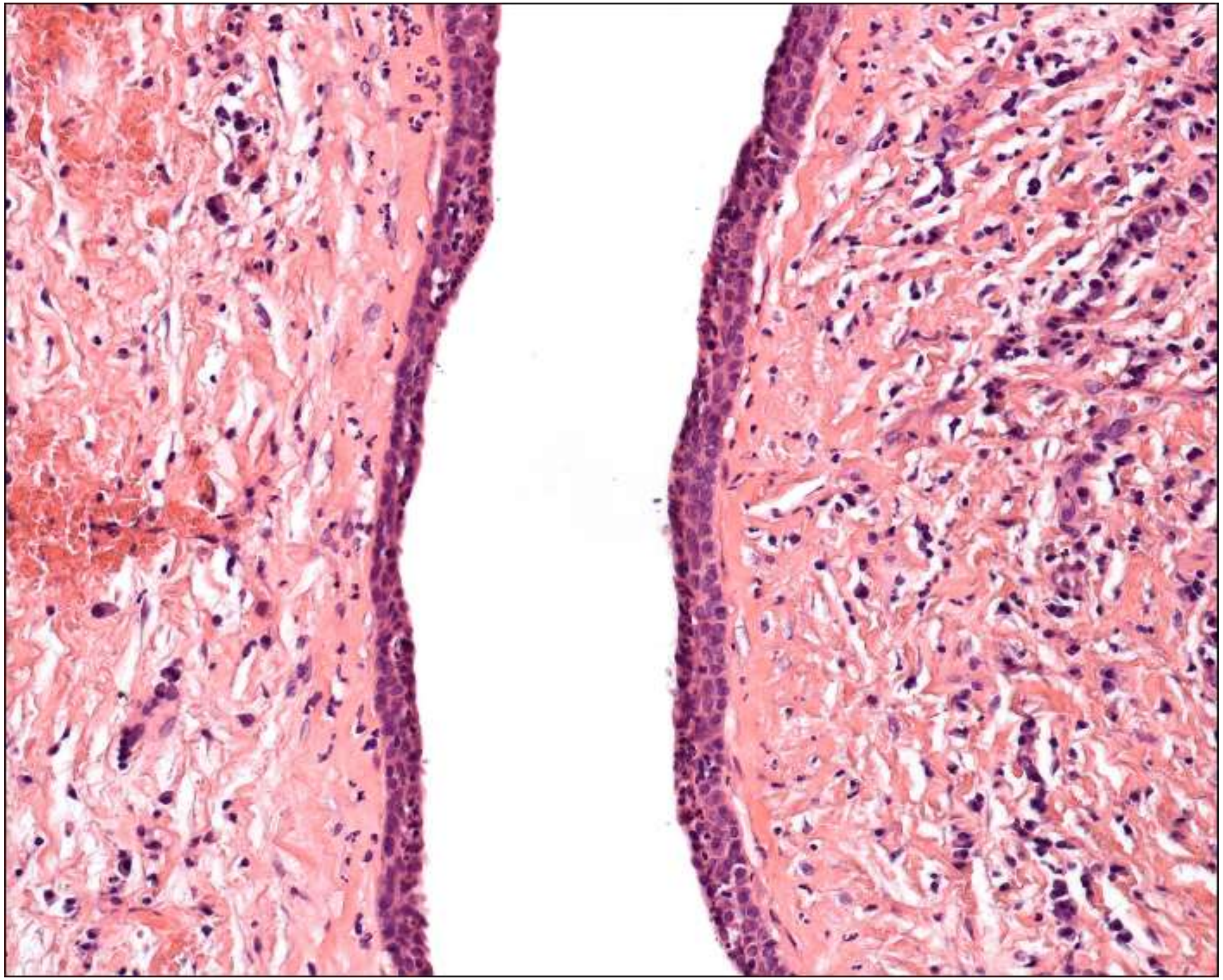

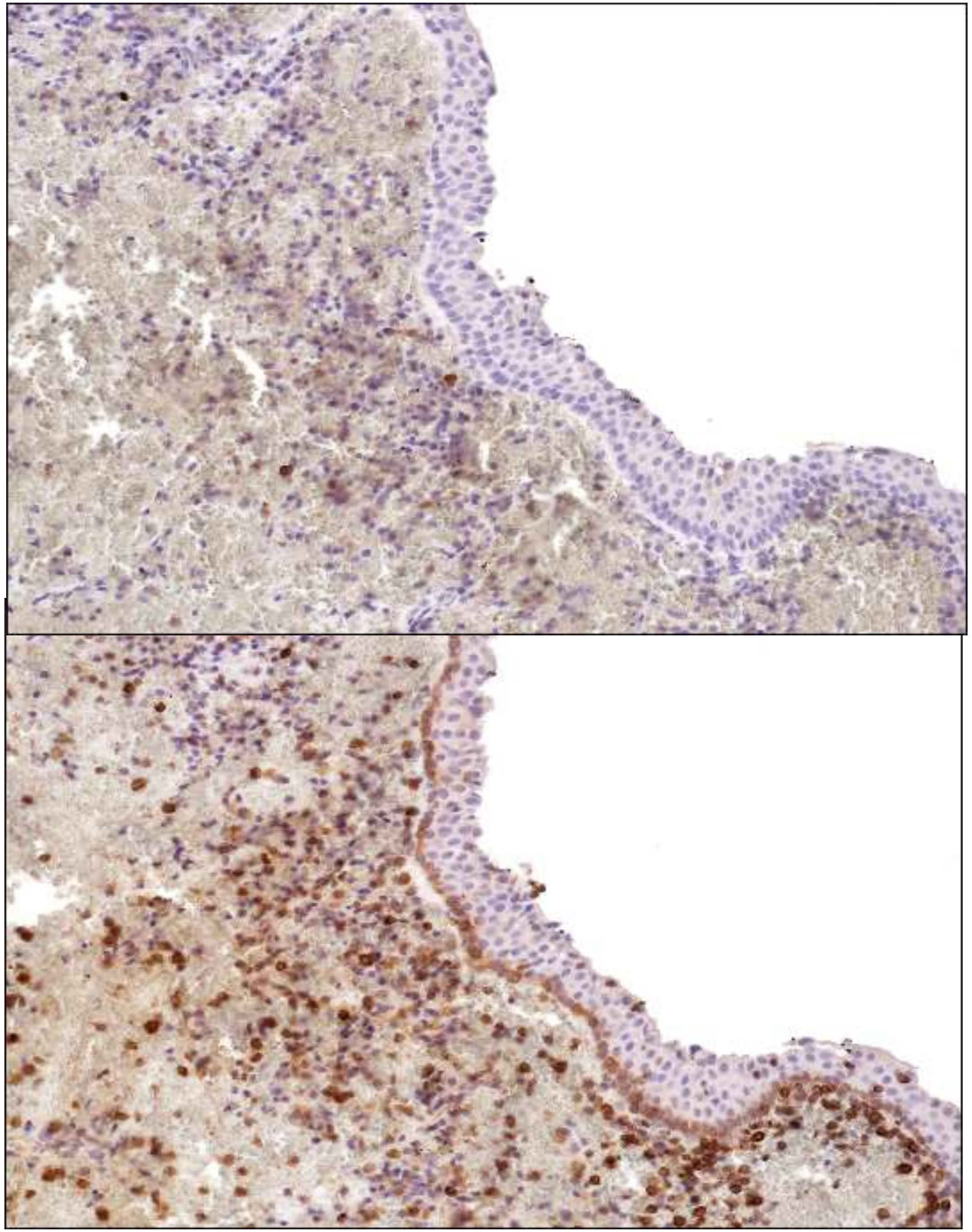

S34 – CK17 (x20)

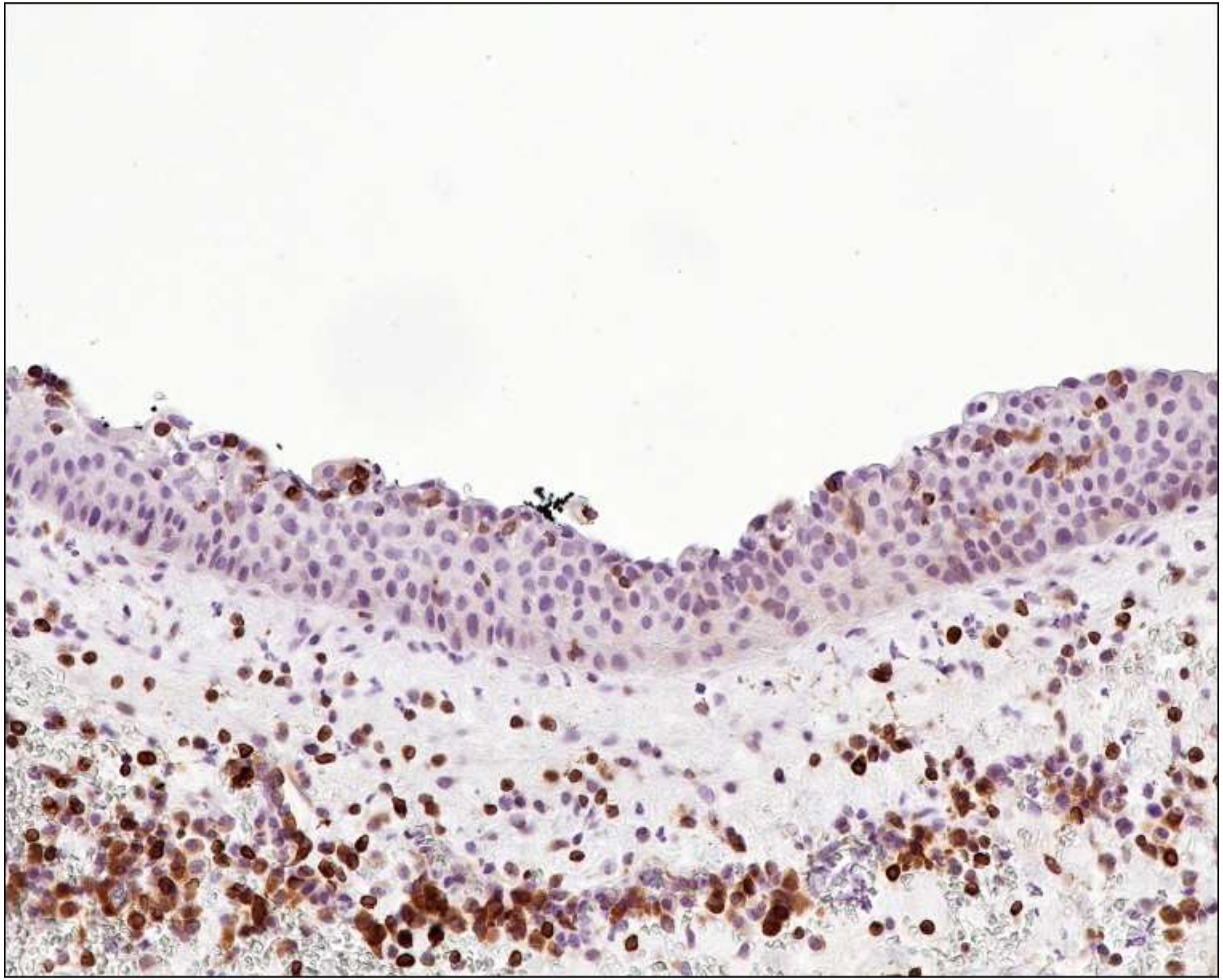

Case S34 – Conclusion

- H&E: thin parakeratotic epithelium with wavy surface and basal palisading
- Inflammation is mild to moderate chronic
- Bcl2: negative, focally basally positive
- CK17: negative (focally spinous, few cells staining)
- Histopathological diagnosis: radicular cyst
- Combined clinical and pathological opinion: radicular cyst

Case S35

- 59-year-old male
- Dental hygiene/status: Neglected teeth. Teeth 45, 43, 42, 41 are vital.
- OPT: radiolucent area around teeth 45, 44.
- Operation: removal of cyst and teeth 45, 44, 43
- Clinical diagnosis: mandibular cyst

S35 – HE, CK17, bcl2 (x20)

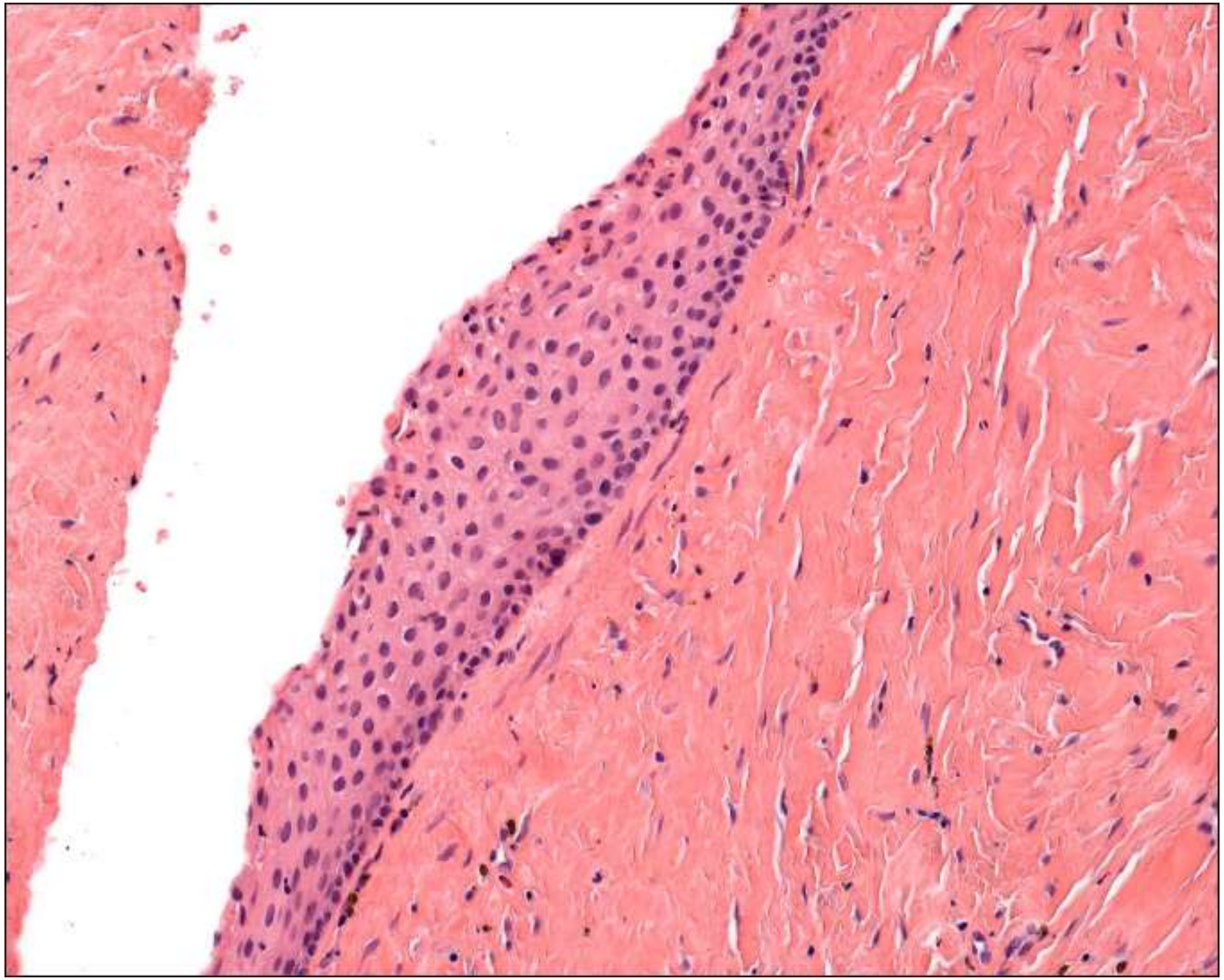

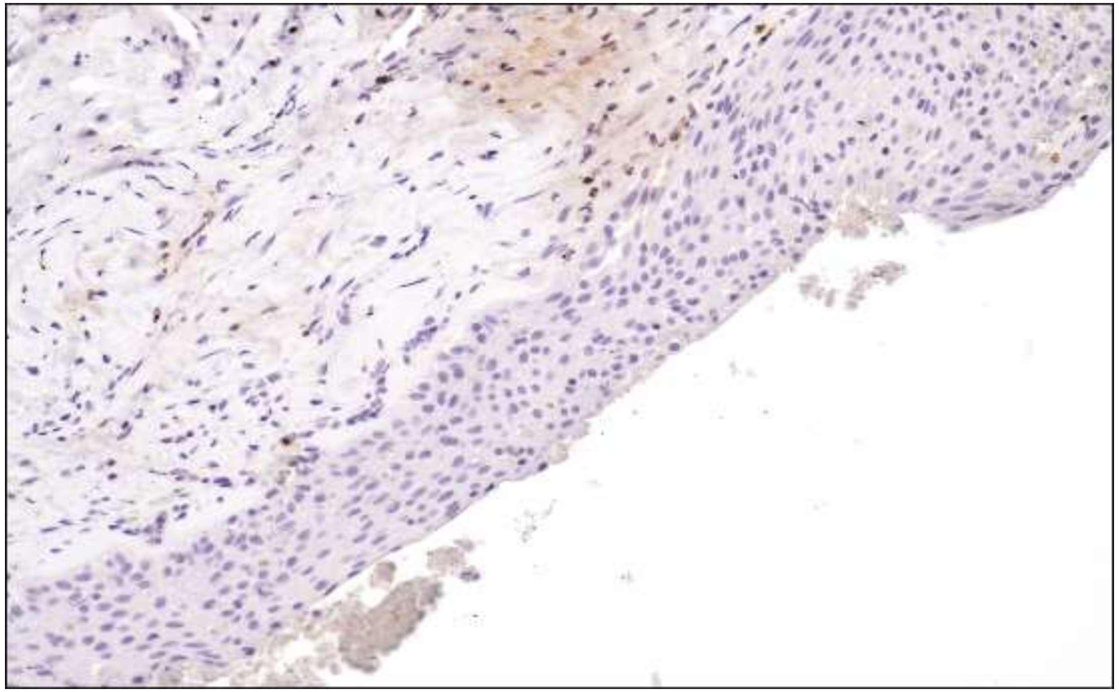

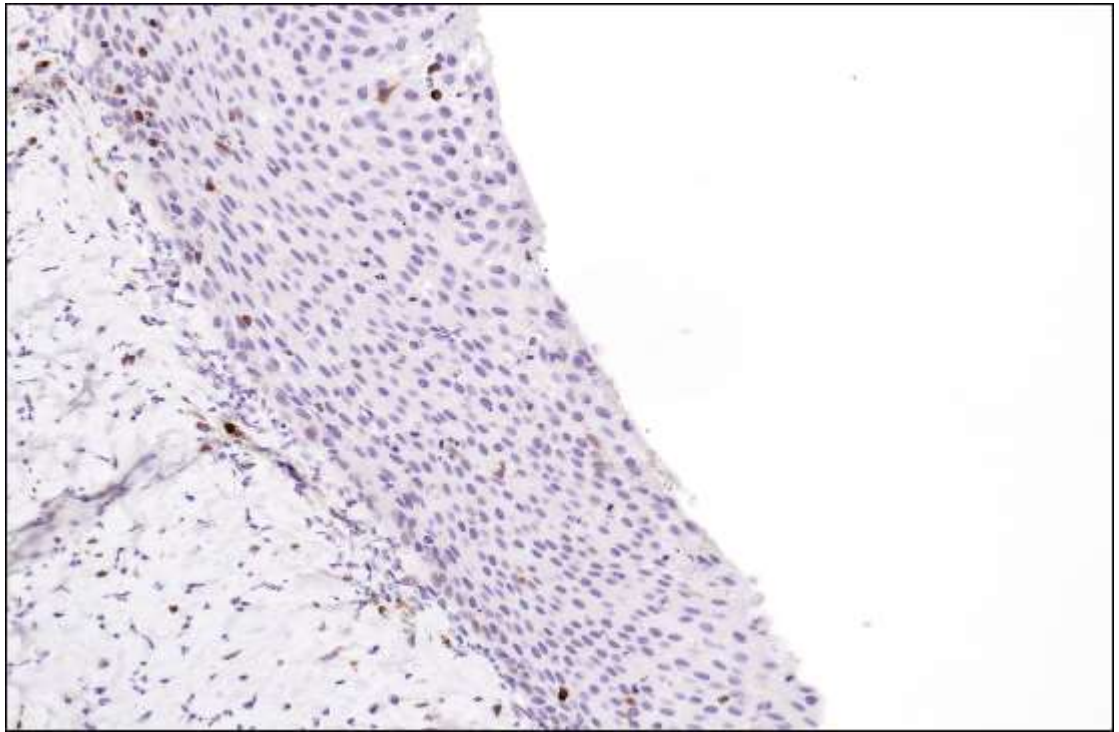

Case S35 – Conclusion

- H&E: thick stratified squamous epithelium without parakeratosis, wavy surface or basal palisading, even epithelial stromal interface
- Inflammation is absent to focally intensive mixed; cholesterol granuloma.
- Bcl2: negative.
- CK17: negative.
- Histopathological diagnosis: radicular cyst.
- Combined clinical and pathological opinion: radicular cyst (mature)

Case S37

- 41-year-old male
- Dental hygiene/status: Neglected, partly missing, partly treated teeth. Palpable swelling is present in the vestibule.
- OPT& CBCT: radiolucent lesion destroying the basis of the nose, the wall of right maxillary sinus is present around teeth 13-11.
- Operation: removal of cyst and teeth 13, 11.
- Clinical diagnosis: keratocyst?

S37 - OPT

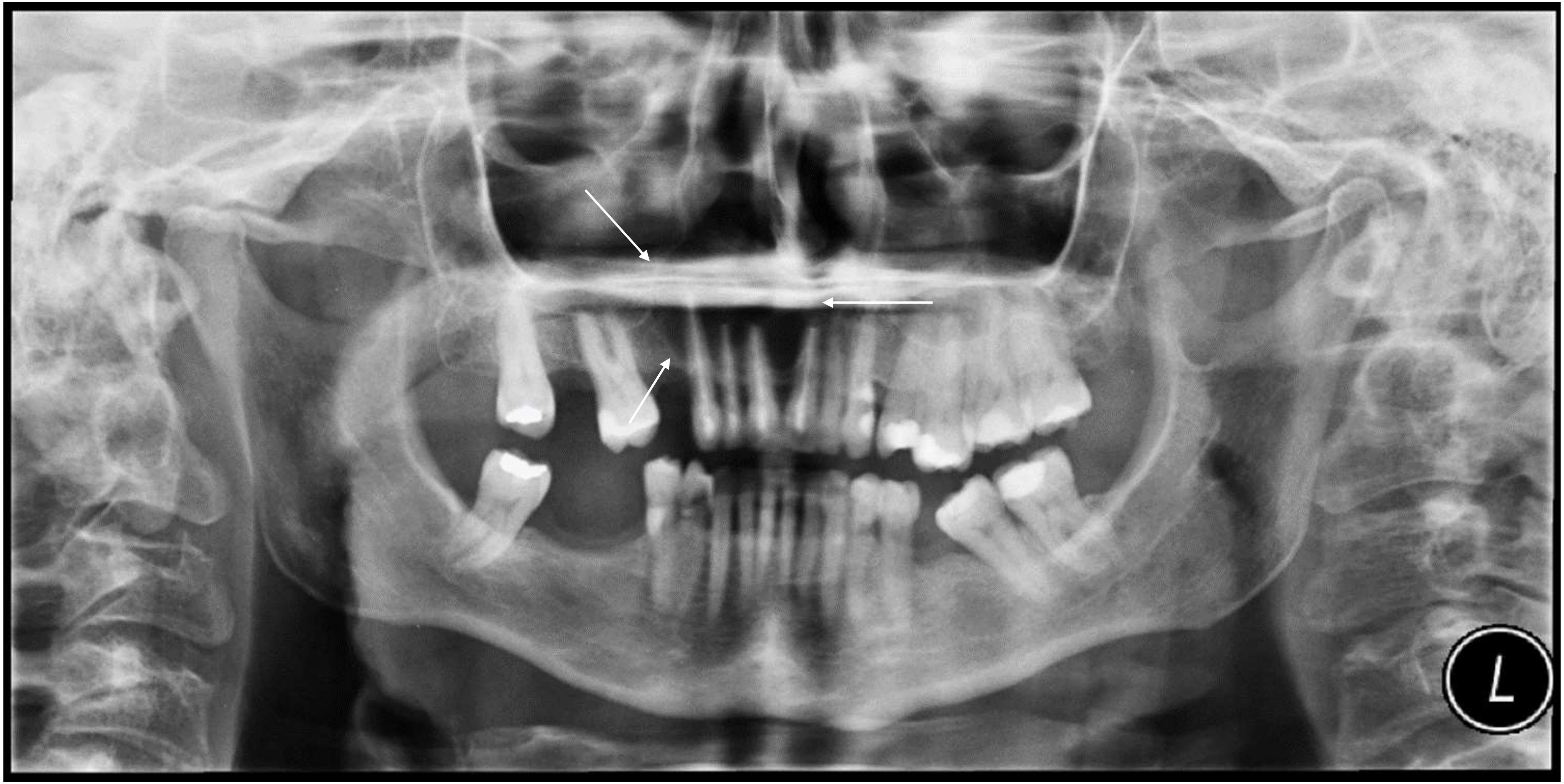

S37 – HE, CK17, bcl2 (x20)

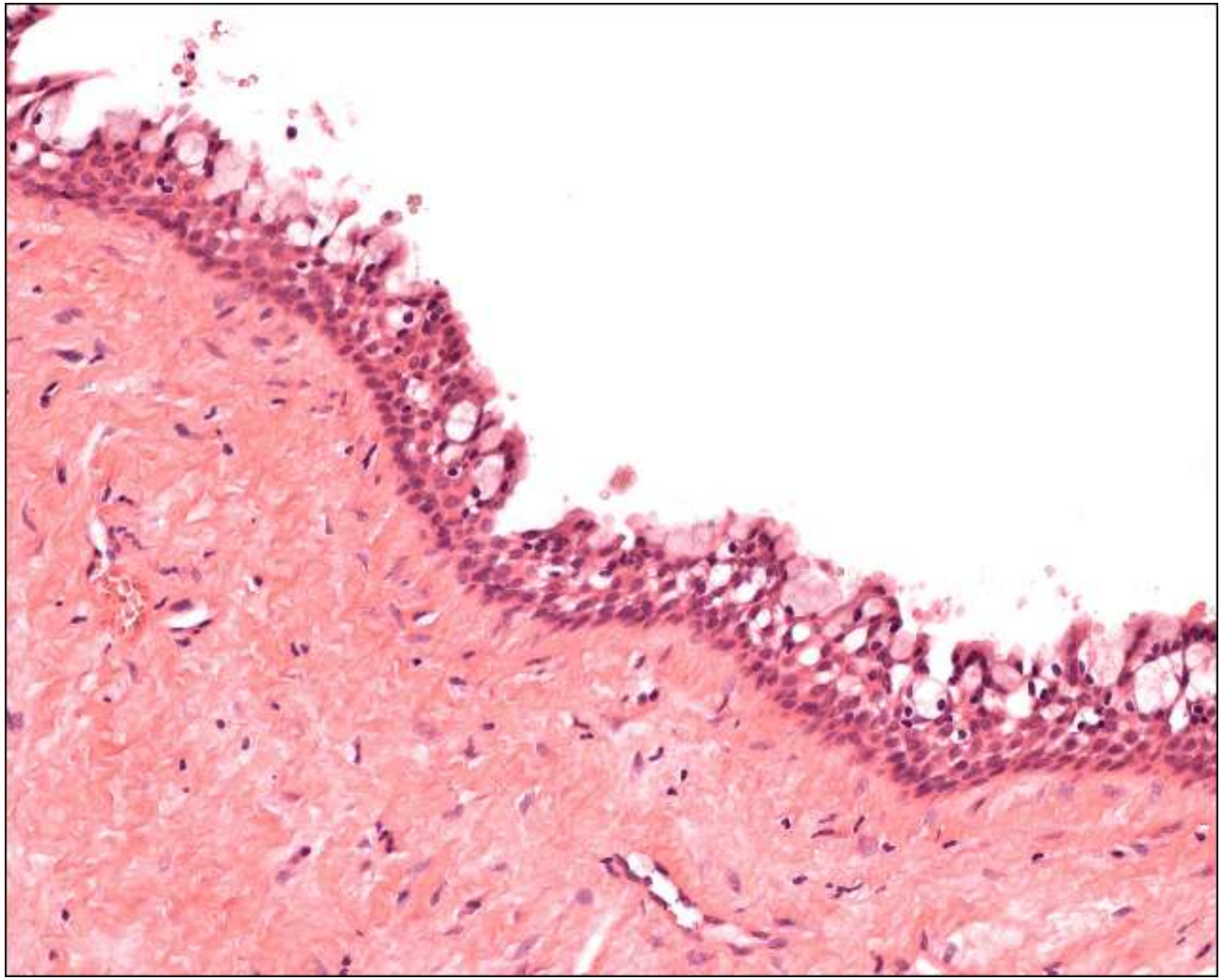

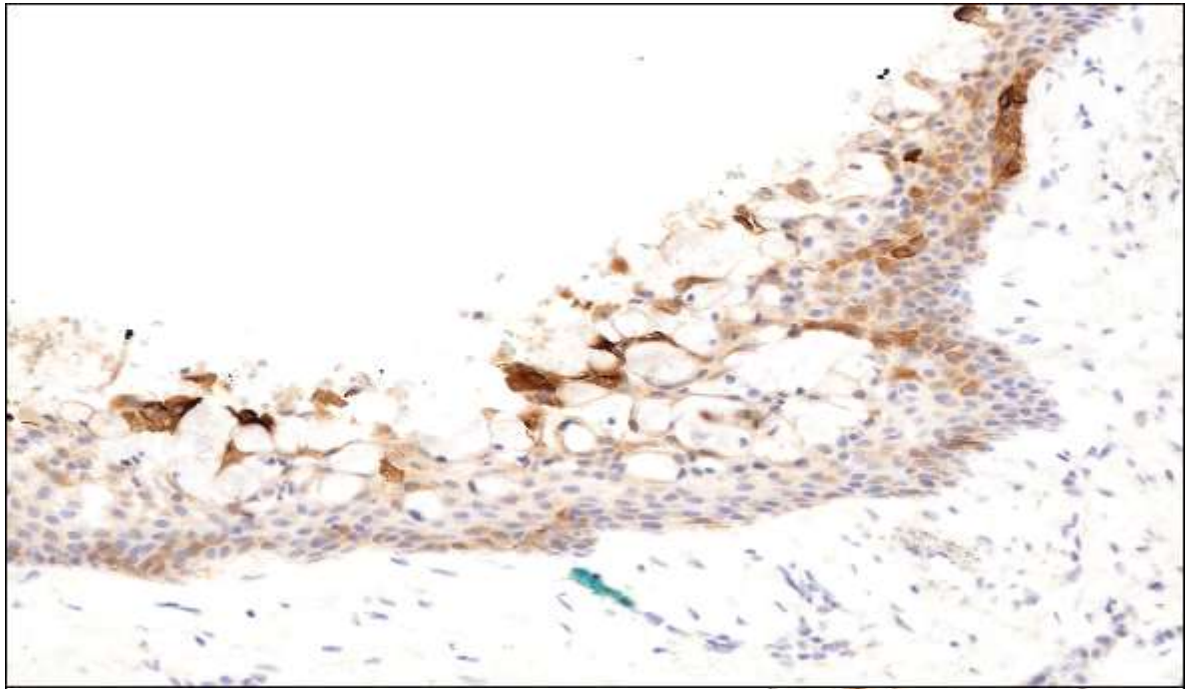

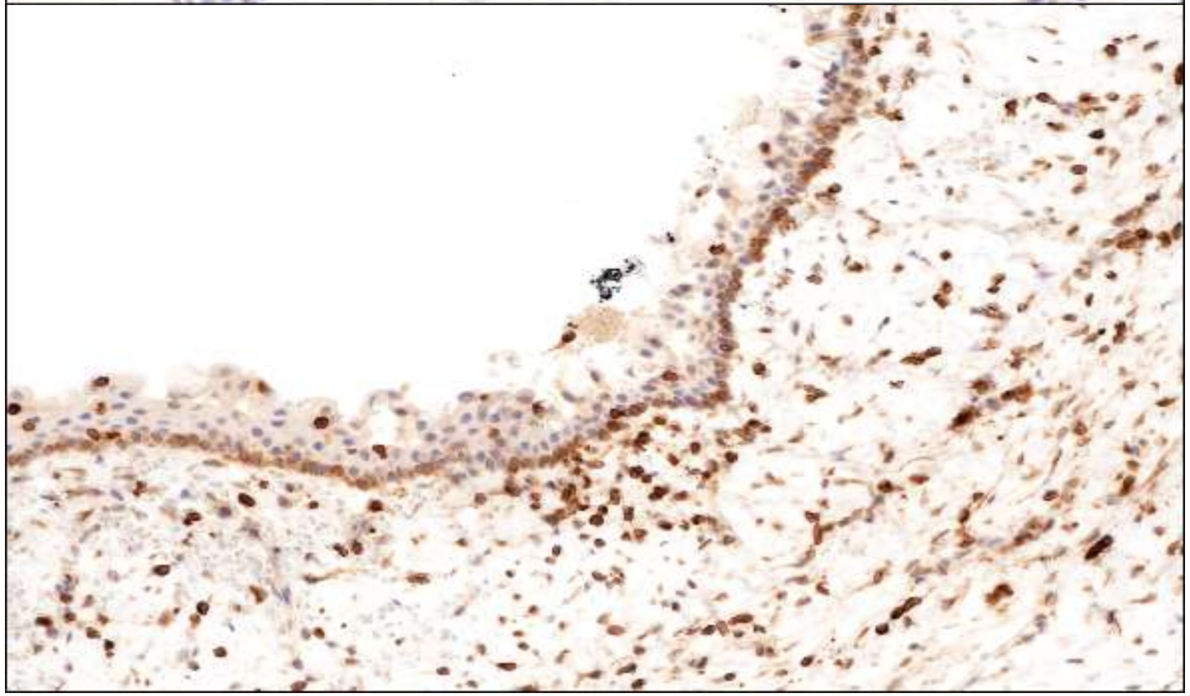

Case S37 - Conclusion

- H&E: thin epithelium with numerous mucous cells
- Inflammation is absent, cholesterol clefts in the lumen
- Bcl2: basal positivity (focally negative)
- CK17: dominantly superficial, focally transepithelial positivity, with focal negativity
- Histopathological diagnosis: „mucoepidermoid” cyst
- Combined clinical and pathological opinion: mature radicular cyst

Case S38

- 59-year-old male
- Dental hygiene/status: Partly missing, treated teeth
- CBCT: Radiolucent cystic lesion around the root of teeth 11, 12, 13, 21.
- Operation: removal of cyst and root of teeth 13, 12, 11, 21.
- Clinical diagnosis: odontogenic cyst

S38 – OPT

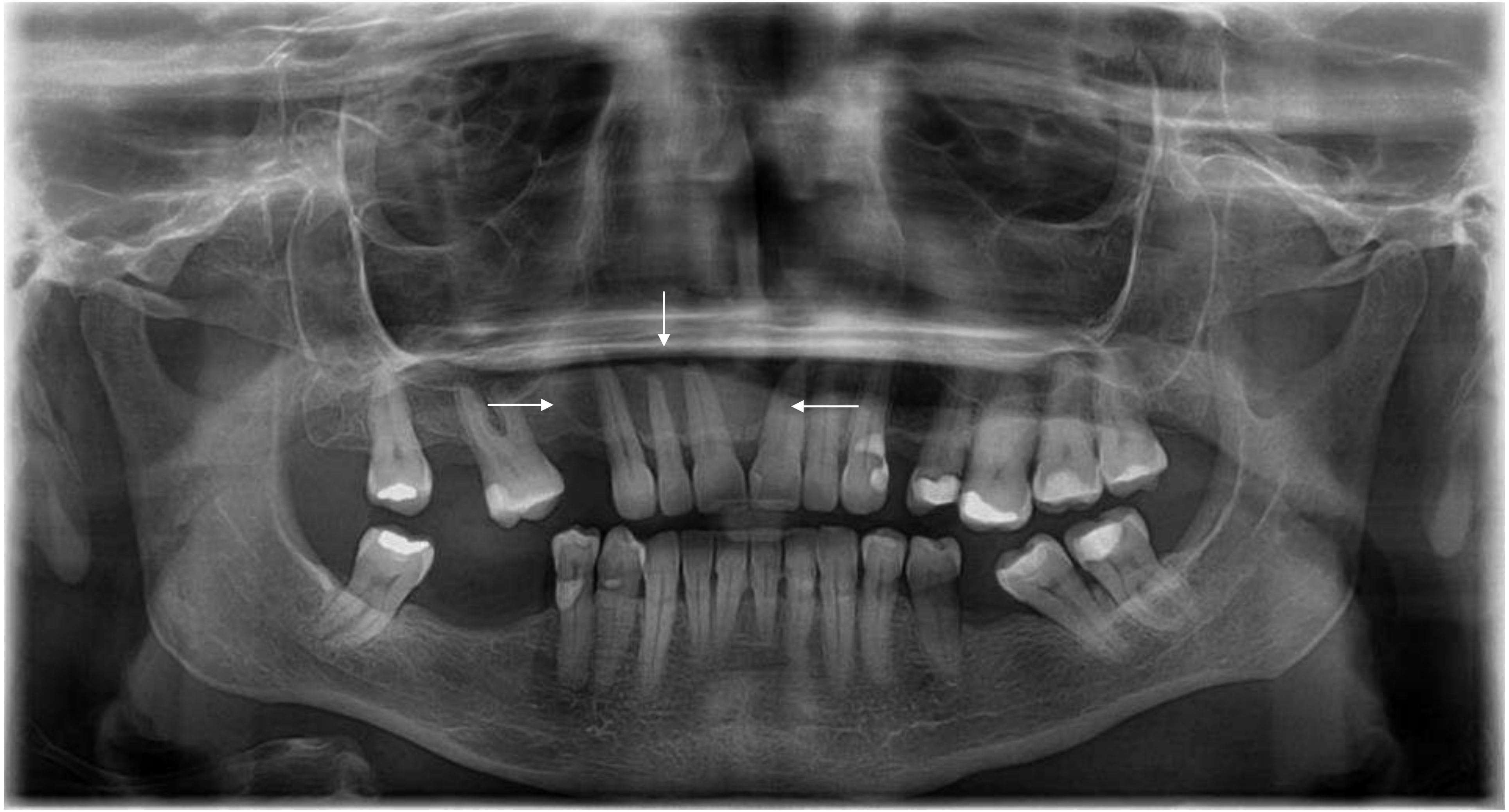

S38 - CBCT

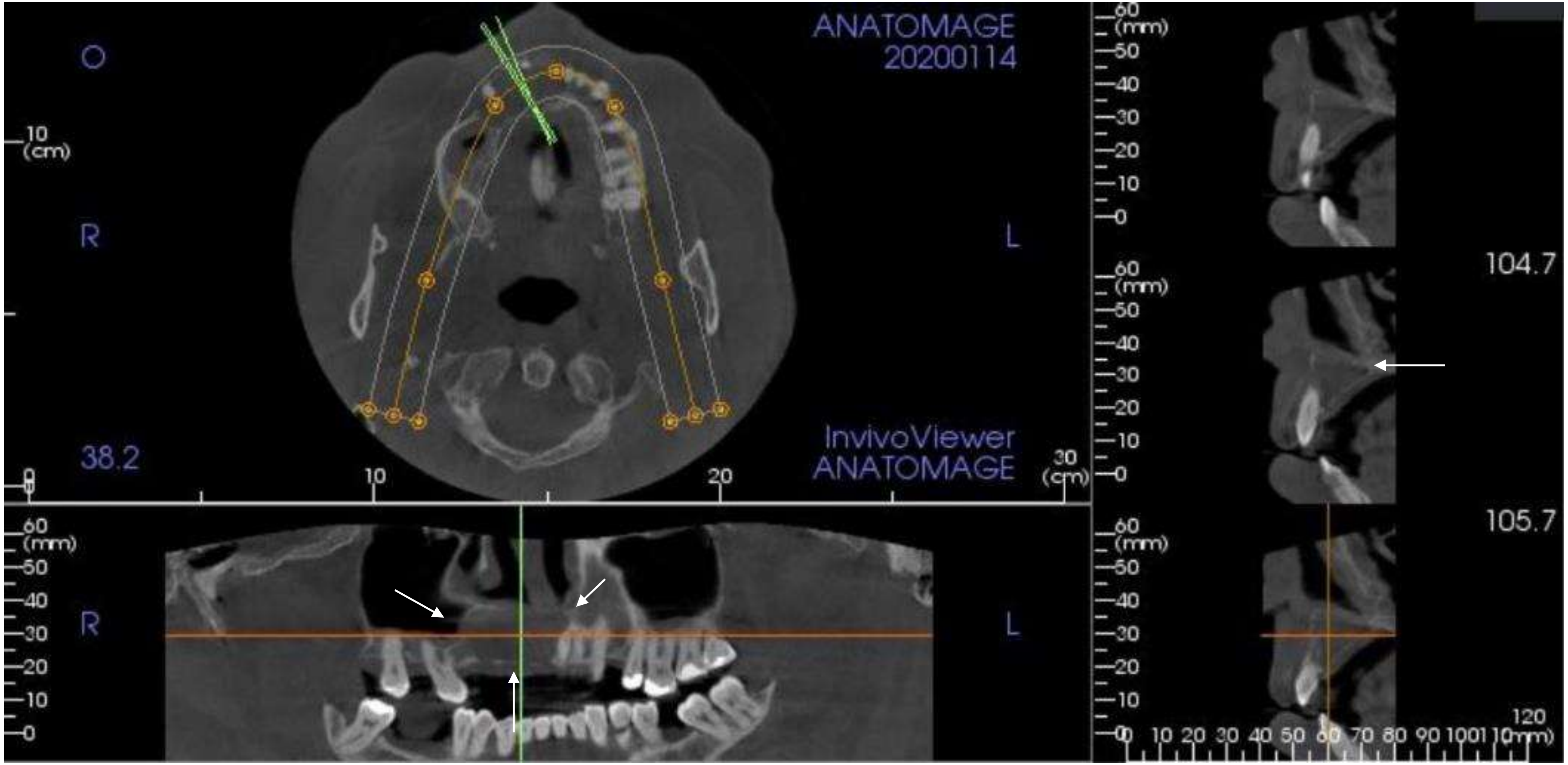
ANATOMAGE
20200114
O
R
L
38.2
InvivoViewer
ANATOMAGE
104.7
105.7

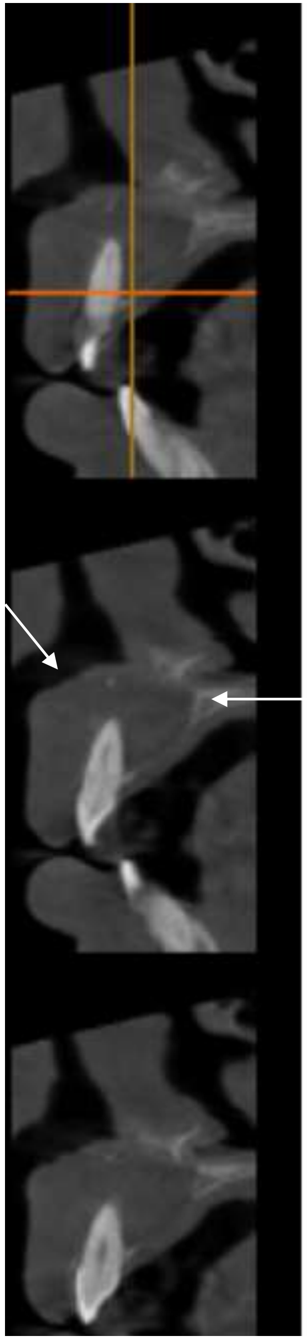

S38 – HE (x2)

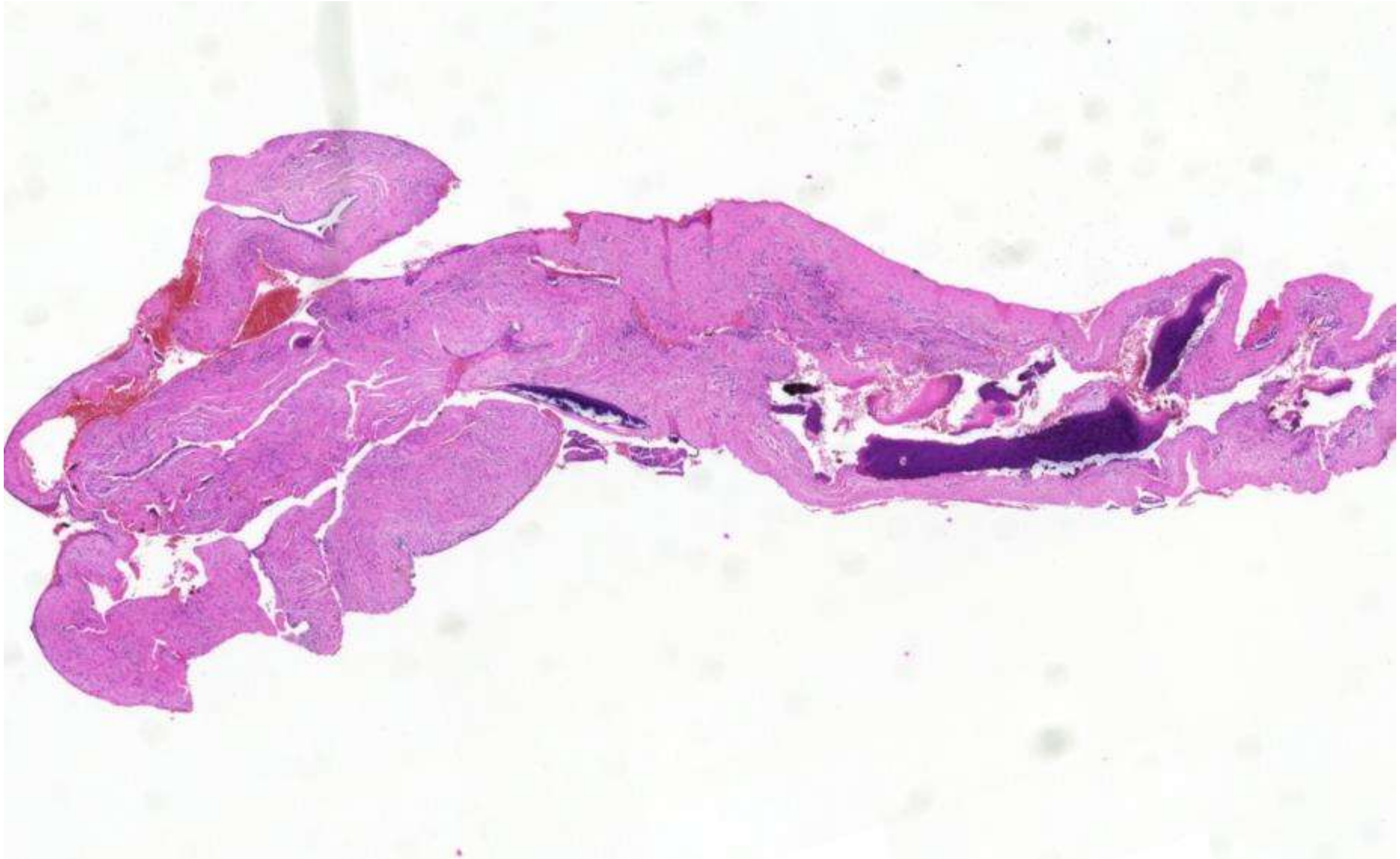

S38 – HE (x20)

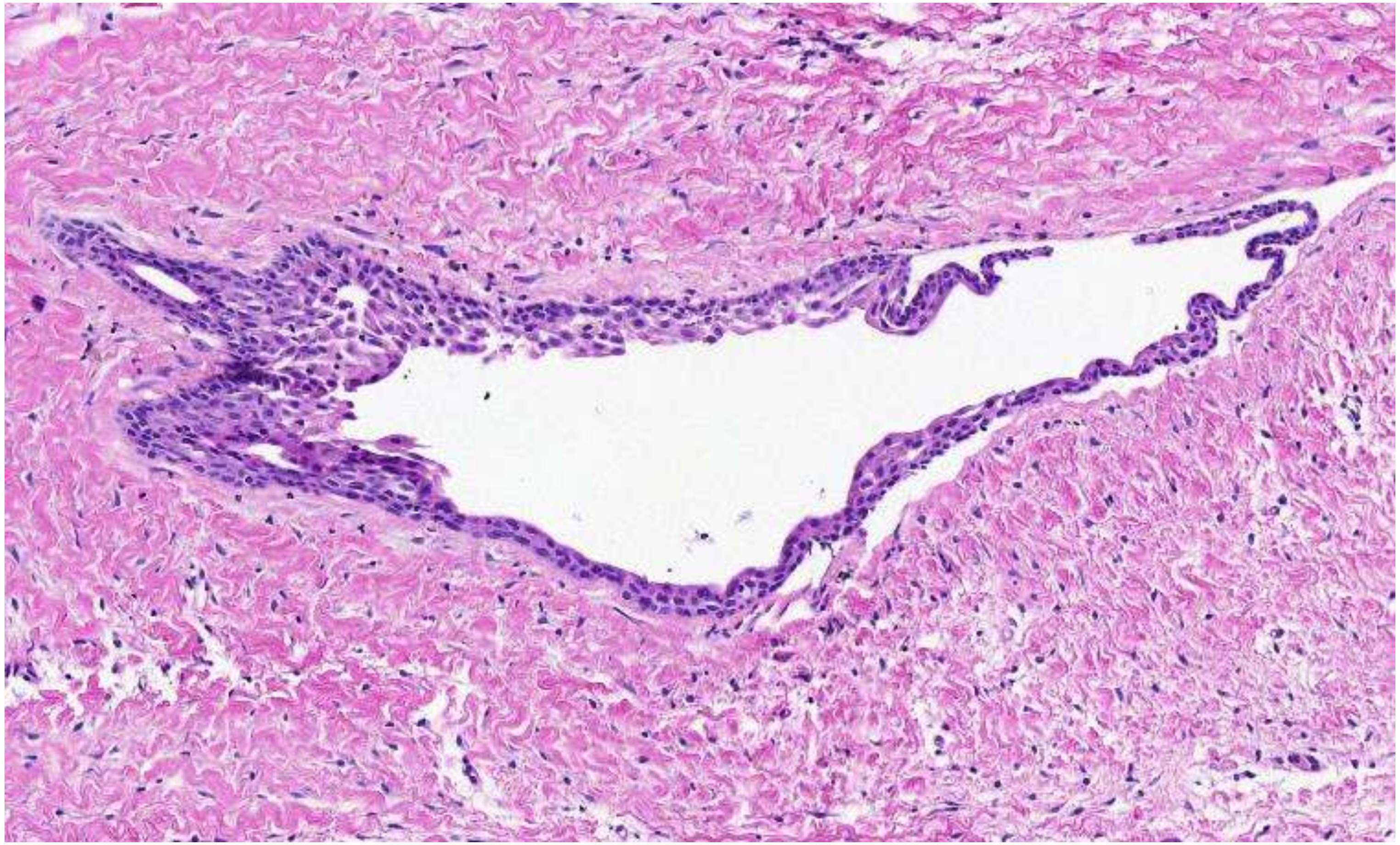

S38 - Corresponding area with CK17, bcl2 (x17, x20)

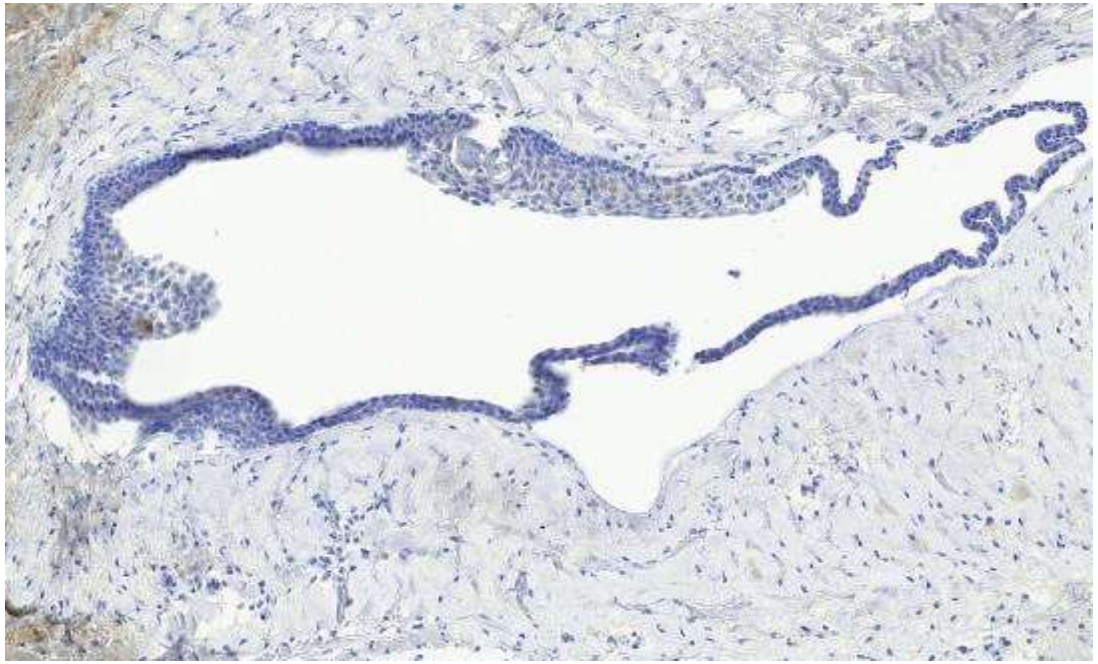

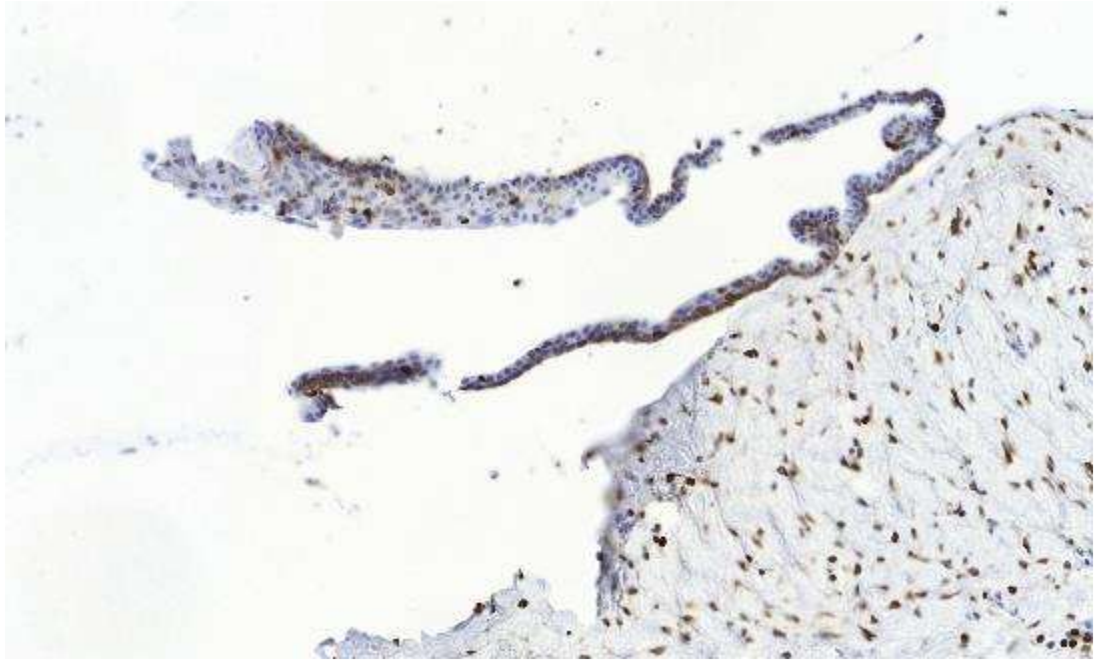

S38 – HE, CK17, bcl2 (x20)

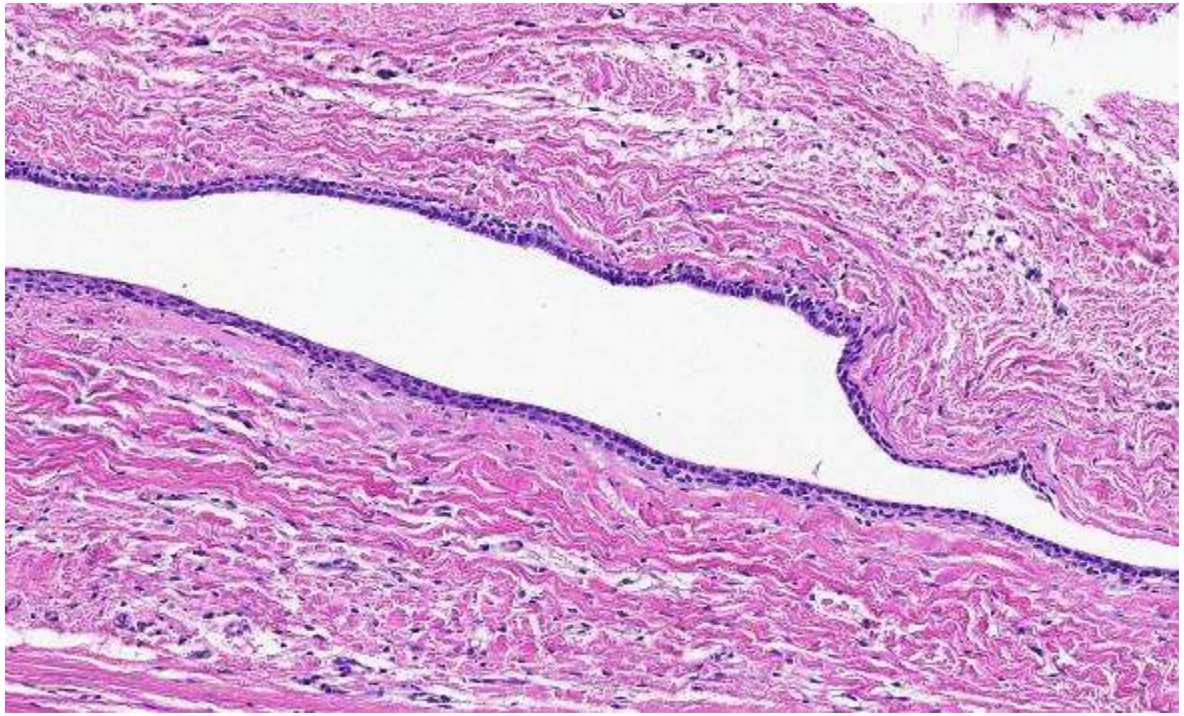

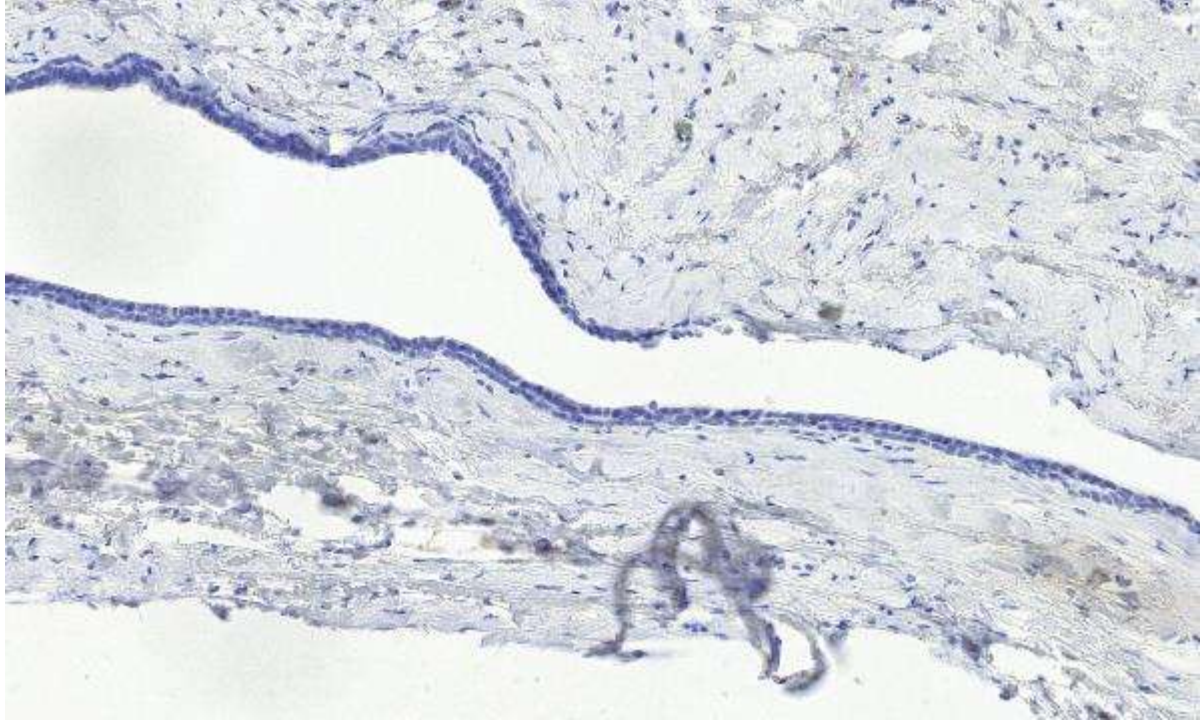

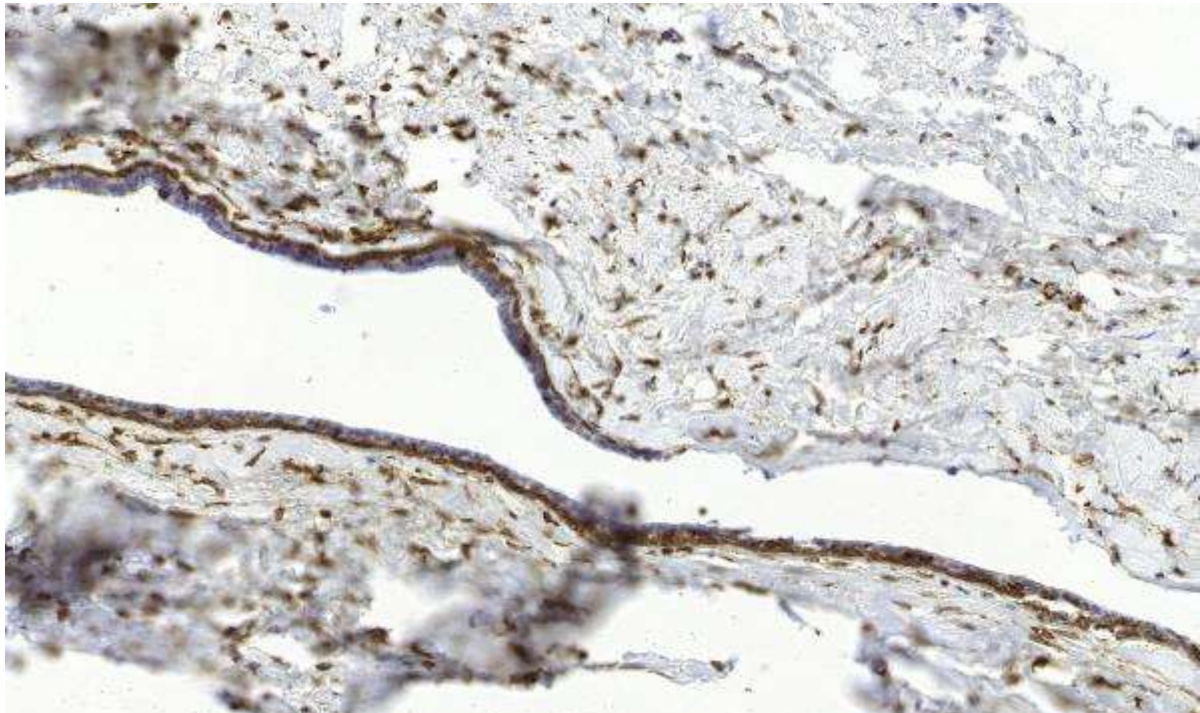

S38 – weaker and focal bcl2 positivity (basal)

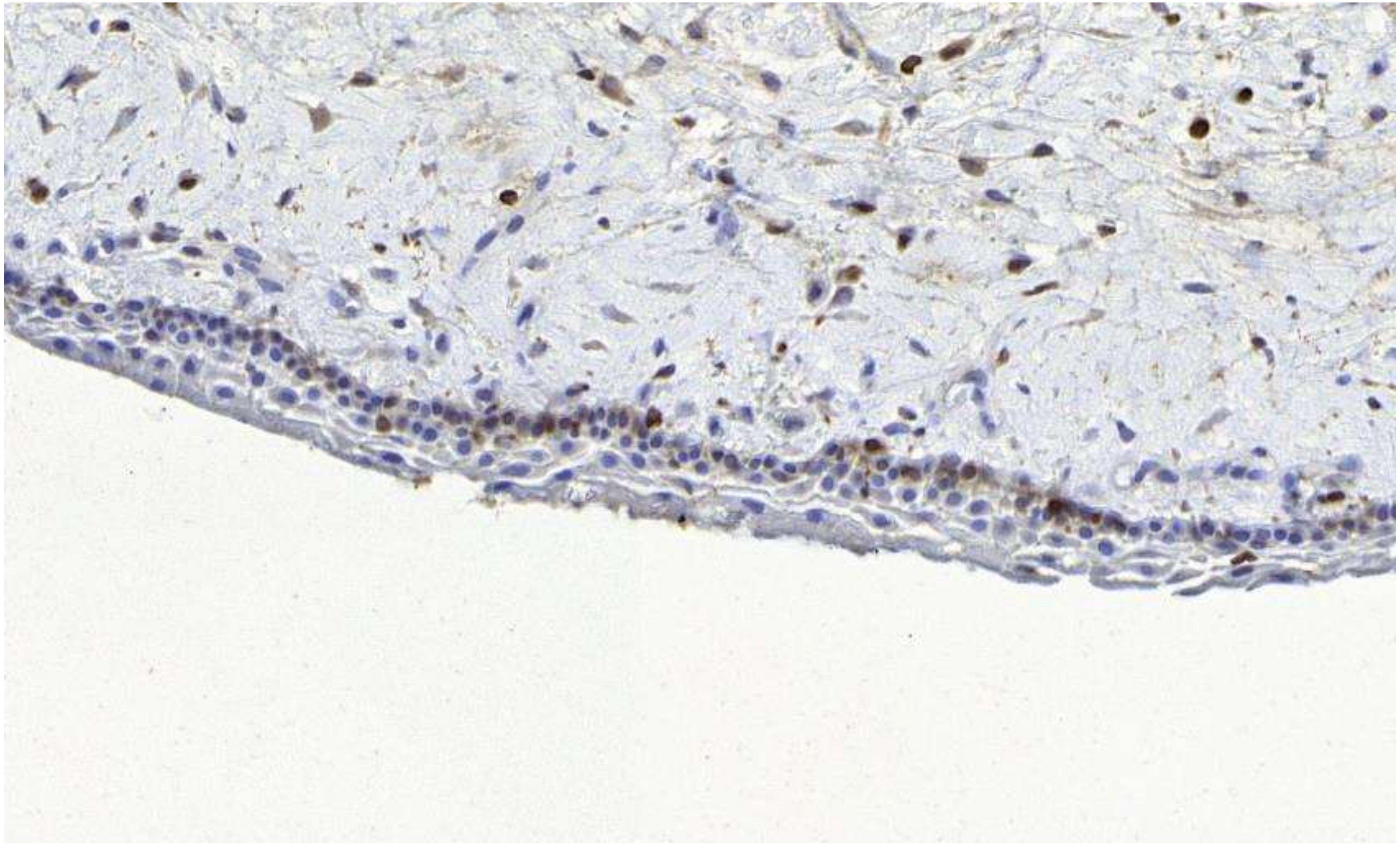

Case S38 – Conclusion

- H&E: thin, dominantly bilayered epithelium without parakeratosis, basal palisading or wavy surface and an even epithelial stromal interface, very few multi-layered thickenings; few ciliated(?) cells
- Inflammation: absent
- Bcl2: moderate basal positivity, focally weaker (background also conspicuous in some areas)
- CK17: negative
- Histopathological diagnosis: odontogenic cyst N.O.S. (no signs of OKC)
- Combined clinical and pathological opinion on revision of the slides: nasopalatine duct cyst considered (midline location and palatal extension) more likely (favoured), but botryoid odontogenic cyst also considered in the differential diagnosis.

Case S39

- 34-year-old male
- Dental hygiene/status: neglected, partly missing, untreated teeth.
- OPT: radiolucent cystic lesion around teeth 35-36. Tooth 35 is vital.
- Operation: removal of cyst and teeth 18, 15, 25, 28, 38, 36, 48
- Clinical diagnosis: odontogenic cyst, keratocyst?

S39 - OPT

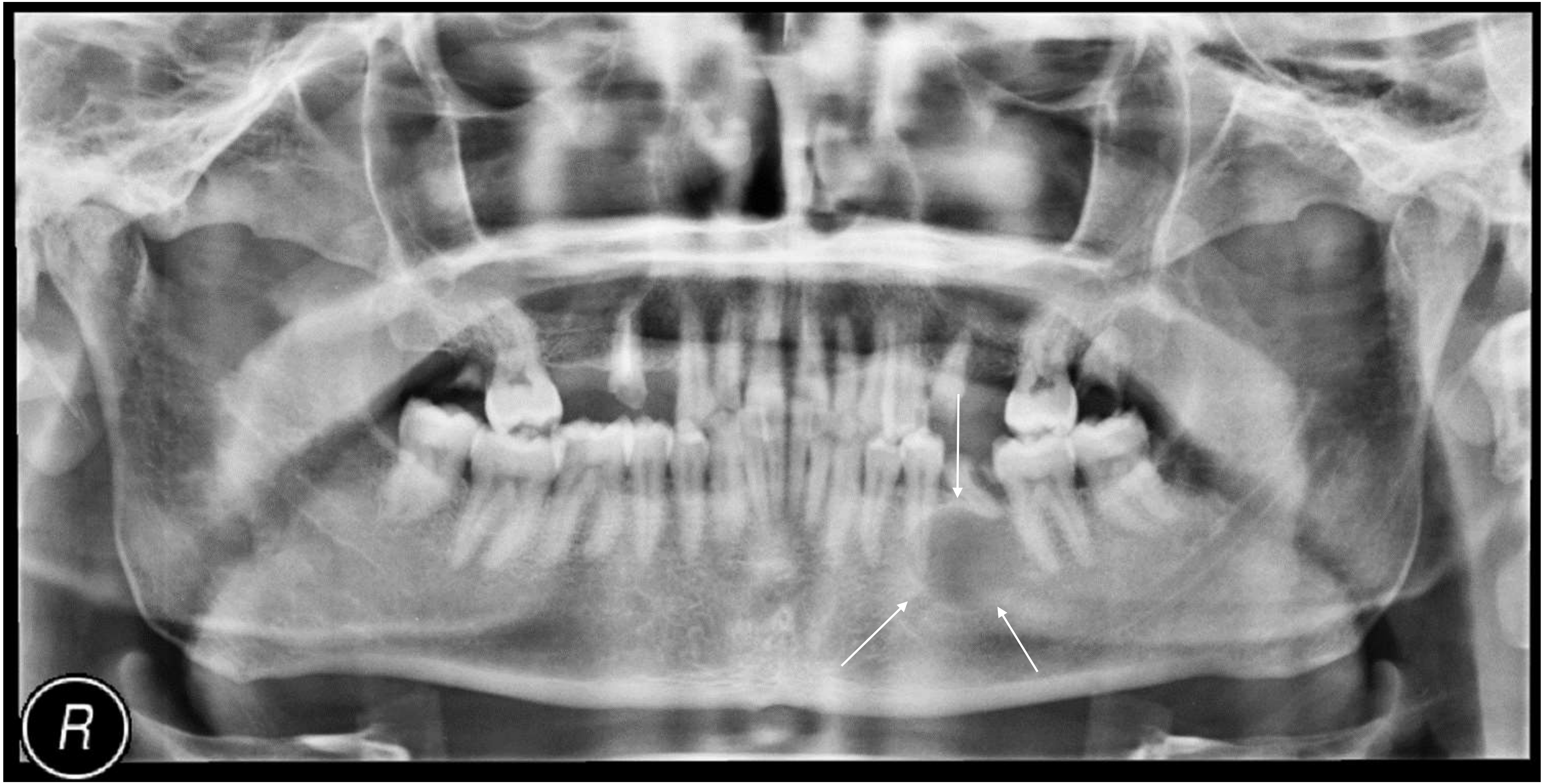

S39 – HE, CK17, bcl2 (x20)

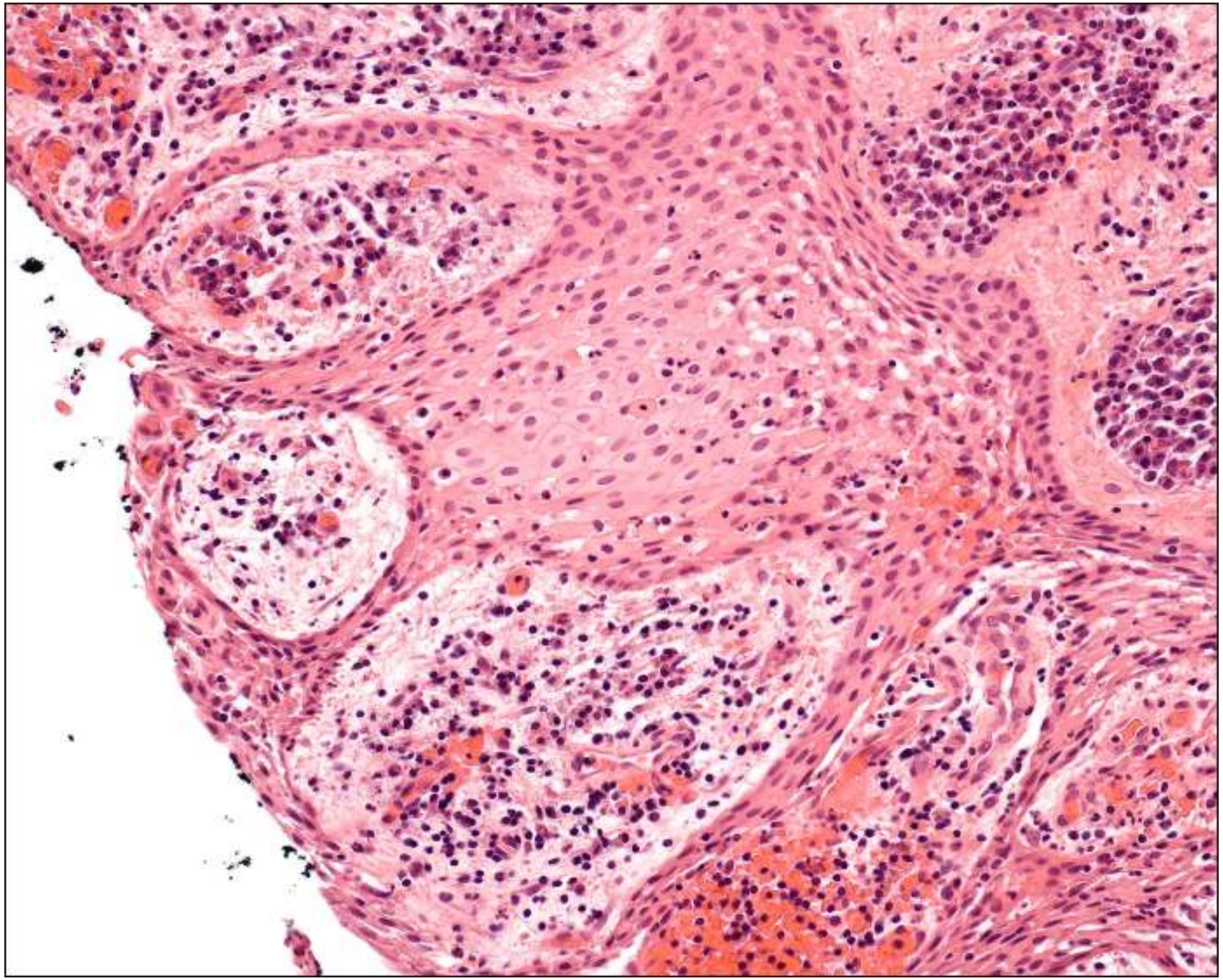

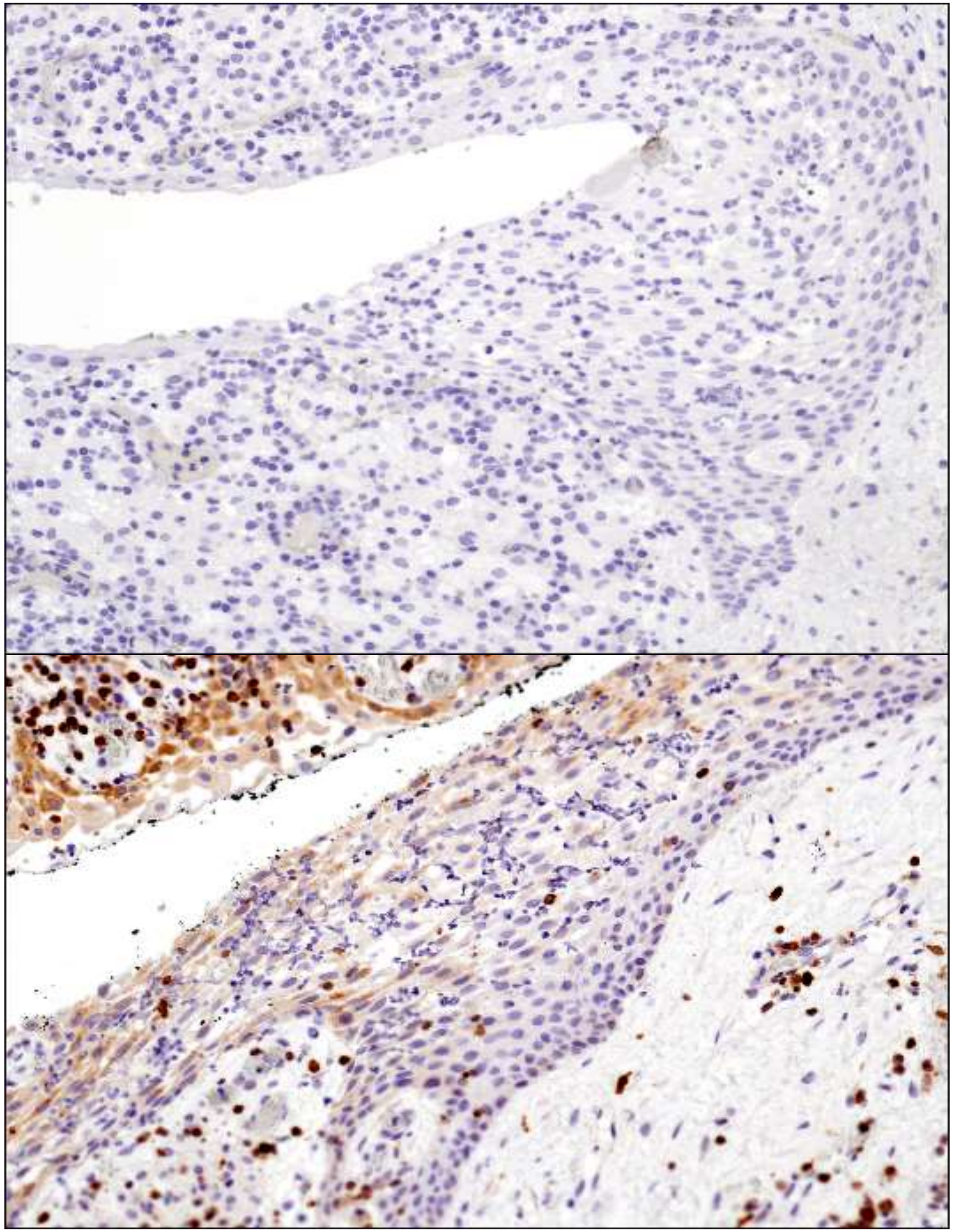

Case S39 – Conclusion

- H&E: relatively thick squamous epithelium with spongiosis. Surface undulation, parakeratosis, basal palisading are absent. The stromal epithelial interface is uneven.
- Prominent chronic inflammation with a minor acute component.
- Bcl2: negative or weak/moderate spinous – suferficial - transepithelial.
- CK17: dominantly negative, focal superficial
- Histopathological diagnosis: radicular cyst
- Combined clinical and pathological opinion: radicular cyst

Case S41

- 44-year-old female
- Dental hygiene/status: Neglected teeth, multiple roots in the alveoli.
- OPT: roots of teeth 12, 14, 18, 21, 23, 25, 26; cystic lesion around tooth 44
- Operation: removal of cyst and teeth 12, 14, 18, 21, 23, 25, 26
- Clinical diagnosis: keratocyst?

S41 – HE, CK17, bcl2 (x20)

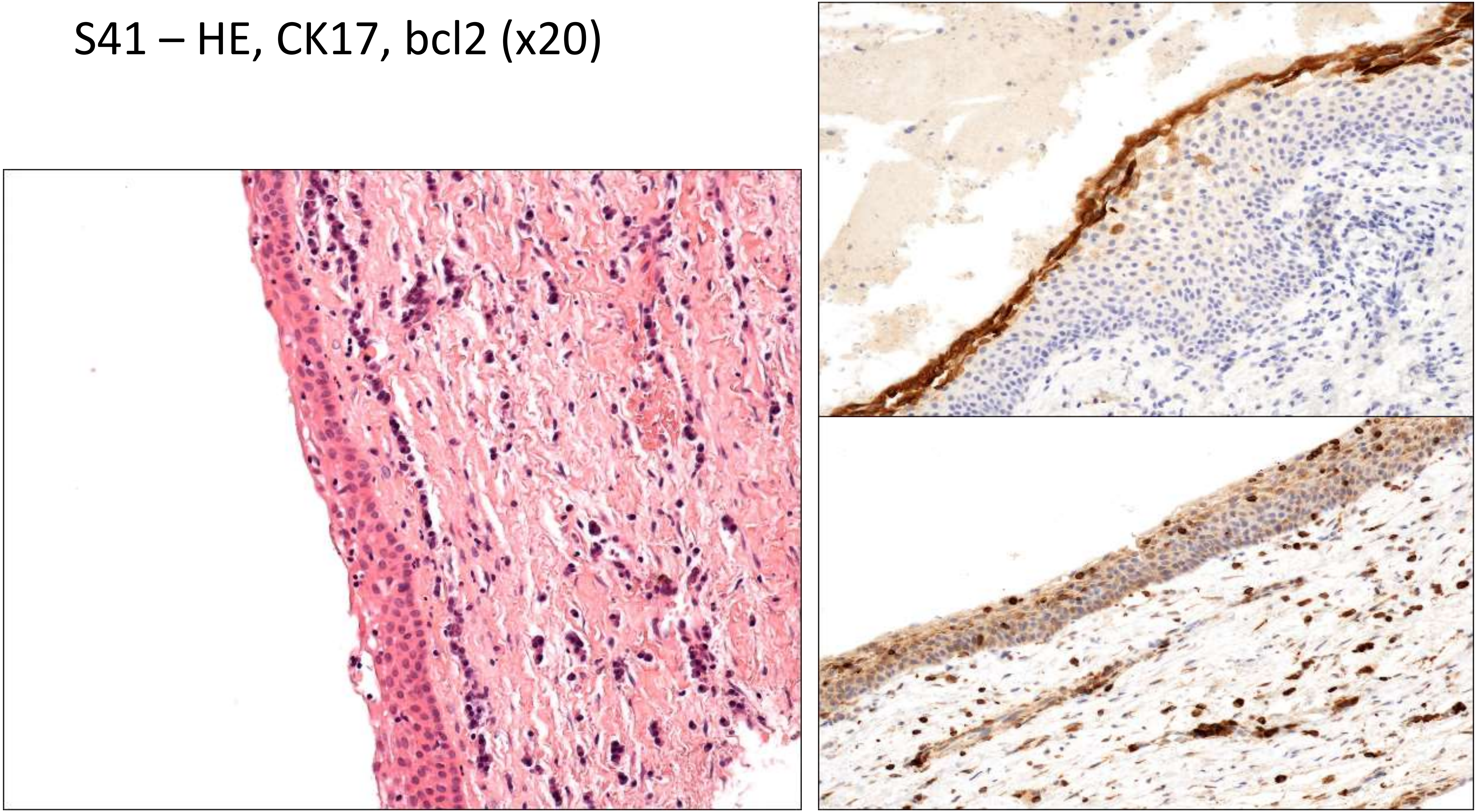

Case S41 – Conclusion

- H&E: thin squamous epithelium without keratinization. Surface undulation, parakeratosis, basal palisading are absent. The epithelial stroma interface is even.
- No conspicuous inflammatory infiltrate
- Bcl2: transepithelial positivity of moderate intensity to negligibly weak background like staining
- CK17: superficially positive, focally negative
- Histopathological diagnosis: apical radicular cyst
- Combined clinical and pathological opinion: cannot be specified; developmental or mature inflammatory odontogenic cyst N.O.S.

Acknowledgment*

The authors thank the following colleagues for the data presented in this supplementry material

- Radiology: Drs. Andrea Herédi, Edina Klement, Orsolya Serfőző, Andrea Krisztina Pócs, Márta Lóránd; Katalin Gion
- Surgery: Drs. Péter Kozma, László Zsubori, Marica Heiner, Tamás Sass, Gergely Kővágó, Flóra Tóth, Dóra Cseh, Tamás Tőkés, Balázs Szakály; Éva Vetró, Sofiane Menzou, Anita Raul, László Seres, Tamás Vereb, Marietta Mucsi, Máté Vlocskó, Csilla Prágai, Attila Rácz and Prof. József Piffkó
- Informatics: Béla Balogh
- Pathology: Drs. István Sejben, Rita Bori, Lajos Kocsis, Rozália Hajnal-Papp(†), Tibor Vágó, Erika Csörgő; Sándor Hamar, Béla Vasas, Levente Kuthi
- Photography of the S series: Mihály Dezső
- Consultation: Prof. Emeritus Paul Speight, School of Clinical Dentistry University of Sheffield

*All at Bács-Kiskun County Teaching Hospital or University of Szeged; except where otherwise stated
